# A synergistic Rh(I)/organoboron-catalysed site-selective carbohydrate functionalization that involves multiple stereocontrol

**DOI:** 10.1038/s41557-022-01110-z

**Published:** 2022-12-30

**Authors:** V. U. Bhaskara Rao, Caiming Wang, Daniel P. Demarque, Corentin Grassin, Felix Otte, Christian Merten, Carsten Strohmann, Charles C. J. Loh

**Affiliations:** 1 Abteilung Chemische Biologie, Max Planck Institut für Molekulare Physiologie, Dortmund, Germany; 2 Fakültät für Chemie und Chemische Biologie, Technische Universität Dortmund, Dortmund, Germany; 3 Ruhr-University Bochum, Organic Chemistry II, Bochum, Germany; 4 Department of Inorganic Chemistry, Technische Universität Dortmund, Dortmund, Germany

**Keywords:** Asymmetric catalysis, Carbohydrate chemistry, Synthetic chemistry methodology, Synthetic chemistry methodology

## Abstract

Site-selective functionalization is a core synthetic strategy that has broad implications in organic synthesis. Particularly, exploiting chiral catalysis to control site selectivity in complex carbohydrate functionalizations has emerged as a leading method to unravel unprecedented routes into biologically relevant glycosides. However, robust catalytic systems available to overcome multiple facets of stereoselectivity challenges to this end still remain scarce. Here we report a synergistic chiral Rh(I)- and organoboron-catalysed protocol, which enables access into synthetically challenging but biologically relevant arylnaphthalene glycosides. Our method depicts the employment of chiral Rh(I) catalysis in site-selective carbohydrate functionalization and showcases the utility of boronic acid as a compatible co-catalyst. Crucial to the success of our method is the judicious choice of a suitable organoboron catalyst. We also determine that exquisite multiple aspects of stereocontrol, including enantio-, diastereo-, regio- and anomeric control and dynamic kinetic resolution, are concomitantly operative.

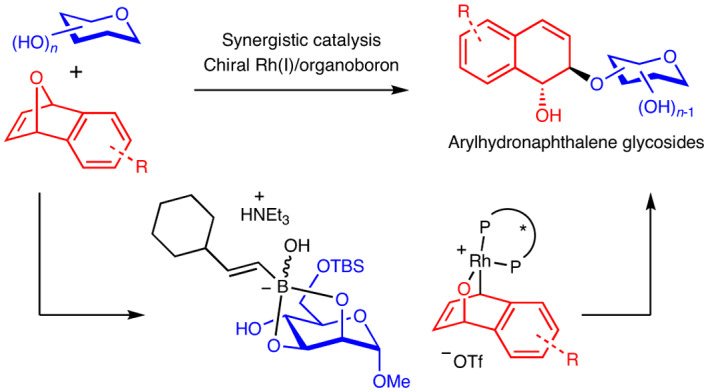

## Abstract (web summary)

Asymmetric systems for catalytic carbohydrate functionalization are mostly limited to chiral copper complexes and organocatalysts. Now, a synergistic chiral Rh(I)- and organoboron-catalysed site-selective functionalization of carbohydrate polyols has been developed, giving stereocontrolled access to biologically relevant arylhydronaphthalene glycosides. Enantio-, diastereo-, regio- and anomeric control and dynamic kinetic resolution were found to be concomitantly operative.

## Main

Site-selective functionalization has emerged as a powerful strategy at the forefront of stereoselective methods^1,2^, largely due to its capability in enabling discrimination of chemically equivalent functionalities in different stereochemical environments. Compared to the more commonly addressed challenge of enantioselectivity, which uses chiral information on catalysts to generate new stereocentres in prochiral substrates, site- or regio-selective methods that enforce regiocontrol have broader relevance to biological reactions. These reactions often involve substrates endowed with dense stereogenic information. However, this endeavour poses a formidable degree of challenge as the overpowering substrate control arising from the inherent stereogenic information must be surmounted by the catalyst.

**Fig. 1:**
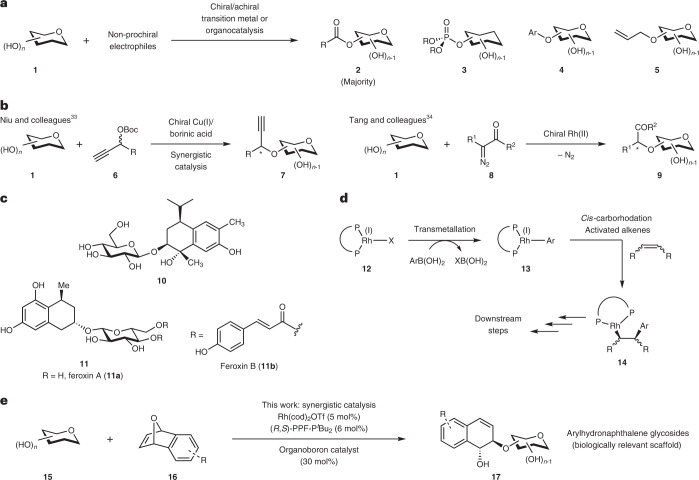
Prior reports of site-selective functionalizations, the conventional role of organoboron reagents in Rh(I) catalysis and current work. **a**, Site-selective functionalizations with non-prochiral electrophiles. **b**, Rare examples of site-selective glycofunctionalizations with concomitant external chirality generation. Asterisks denote new stereogenic centres formed with simultaneous regio- and enantio-control by the participating catalysts. **c**, Bioactive arylnaphthalene glycosides. Compound **10** inhibits Eppstein-Barr Virus Early Antigen (EBV-EA) activation and possesses potent anti-tumour promoting activity. Feroxins A and B possess cathartic properties. **d**, Established transmetallating role of boronic acids in Rh(I) catalysis. **e**, Current work of synergistically employing Rh(I) and organoboron catalysis to achieve multiple stereocontrol in synthesizing arylhydronaphthalene glycosides. Boc, *tert*-butyloxycarbonyl.

Carbohydrates are a key natural class of biomolecules implicated in this challenge. Despite the importance of carbohydrates in a multitude of physiological processes^3^, universal stereoselective access to carbohydrates has largely remained elusive due to the stereochemical complexity of sugars^4^. Hence, chiral catalytic strategies for site-selective functionalizations of carbohydrates provide a highly promising avenue to access hitherto unachievable glycosidic scaffolds^5–8^. An unresolved gap in this field pertains to the majority of currently developed chiral catalytic systems for this purpose being still limited to chiral copper complexes and organocatalysis (Fig. 1a)^9–28^, despite the overwhelming number of examples of asymmetric transition-metal complexes available for enantioselective catalysis. This severely limits selective bond-forming opportunities for carbohydrate functionalizations. The known usage of achiral transition-metal complexes for site-selectivity control, while proved to be useful to a certain extent^29–32^, does not allow the simultaneous enantiocontrol of newly generated chiral centres. A fundamental drawback of such protocols lies in the high reliance on conventional substrate control by the carbohydrate, and the lack of chiral information on these catalysts precludes the exploitation of versatile catalyst control in numerous facets of stereoselection. Thus, this significantly constricts the scope to functionalizations that do not involve chirality generation from prochiral electrophiles.

To the best of our understanding, there are only two rare instances in the literature that have sought to tackle the multiple stereoselectivity issues of chirality generation of the external prochiral electrophile concurrently with site-selective carbohydrate functionalization. These included a copper-catalysed site-selective propargylation reported by Niu and colleagues using synergistic copper and borinic acid catalysis^33^ and a Rh(II)-catalysed site-selective alkylation by carbenoid insertion by Tang and colleagues (Fig. 1b)^34^. These examples are currently limited to the exogenous formation of only one single chirality centre, and additional formation of further chiral centres would unavoidably require the surmounting of an additional diastereoselectivity issue by the catalytic system. To the best of our understanding, the generation of more than one chirality centre in site-selective carbohydrate functionalization is not yet known. The current lack of quality catalytic strategies beyond these mentioned cases for concomitant stereocontrol constitutes a major impediment for the applicability of chiral catalysis in constructing the vast array of complex biologically relevant glycosides with stereoprecision.

As part of our ongoing research efforts in expediting access to synthetic glycosides with biological relevance^35–37^, we were intrigued by a class of carbohydrate scaffolds known as arylhydronaphthalene glycosides^38^. These constitute an important class of natural products in plants such as cotton or Aloe species (Fig. 1c). In particular, analogue **10** displays important anti-tumour activity^39^ and feroxins A and B **11** show cathartic properties^40^. However, any conceivable stereoselective catalytic strategy towards related scaffolds would involve a challenging blend of enantio-, diastereo- and regio-control, as well as chirality generation from an external electrophile. This has hampered a direct stereoselective entry into the arylhydronaphthalene glycoside scaffold to date.

We then targeted chiral Rh(I) catalysis^41–45^. This is not yet explored as a synthetic tool in site-selective carbohydrate functionalizations, despite enjoying significant success in enantioselective catalysis^41,44–46^, such as the Rh(I)-catalysed asymmetric ring-opening reaction (ARO)^47–51^. However, the central question of whether the chiral information endowed in such chiral Rh(I) complexes could also be harnessed to achieve the challenging site-selective control, as well as multiple stereocontrol in complex carbohydrate polyols, still remains unanswered.

Moreover, while organoboron compounds such as boronic acids are widely exploited in Rh(I) catalysis based on excellent pioneering work by Miyaura, Hayashi and colleagues^52–54^, they are currently only known to function as transmetallating agents involving *cis*-carborhodation (Fig. 1d)^50^. Lately, the alternate utility of organoboron reagents as catalysts in glycofunctionalization is gaining traction^55–63^. Hence, assimilating organoboron catalysis synergistically with chiral Rh(I) catalysis would pave a direct route into hydronaphthalene glycoside motifs. However, this non-trivial endeavour requires orchestrating a challenging mechanistic manoeuvre that bypasses the boron to Rh(I) transmetallation pathway.

In this Article, we report a multi-stereocontrolled access to biologically interesting arylhydronaphthalene glycosides by harnessing the power of synergistic chiral Rh(I) and organoboron catalysis^64,65^, with the contemporaneous formation of two external stereogenic centres on a prochiral *meso*-oxanorbornadiene with diastereo-, enantio-, regio- and anomeric control (Fig. 1e). This method employs chiral Rh(I) catalysis in site-selective carbohydrate functionalization. The cooperative effect of rhodium and boronic acid catalysis collectively enforces fourfold stereocontrol in (Supplementary Note 13): (1) site-selective functionalization on a broad range of carbohydrate polyols, (2) enantiocontrol on the Rh(I) oxidative addition step of a bridgehead C–O bond in *meso*-oxanorbornadienes, (3) a dynamic kinetic resolution-type process in anomeric oxygen functionalization and (4) *trans*-diastereocontrol on the hydronaphthalene scaffold. Our protocol introduces a co-catalytic role that boronic acids can serve in the domain of Rh(I) catalysis, taking precedence over the conventional transmetallation pathway. Moreover, the *trans*-diastereoselectivity observed in our methodology is in marked contrast to the *cis*-selectivity when boronic acid nucleophiles were employed in ARO reactions^50^. Fine tuning of the boronic acid catalyst enabled us to identify an unexplored cyclohexylvinylboronic acid as the optimal catalyst. Our studies also revealed that the ligand choice had a pivotal influence in determining both the stereoselectivity and the reaction pathway. Further kinetic studies and computations provide further evidence that the resting states of both the Rh and the boronic acid catalysts are actively involved in the rate-limiting step of the mechanism.

## Results and discussion

### Establishment of the synergistic catalysis methodology

We initiated our investigation by exploring a series of rhodium complexes/chiral ligand combinations for the site-selective functionalization of a mannosyl triol derivative **15a** with oxanorbornadiene **16a**, in the absence of boronic acid. It became apparent in our early experiments that unlike simple alcohols^48,51^, the carbohydrate polyol substrate performed poorly as a naked nucleophile. When neutral dimeric rhodium complexes such as [Rh(cod)Cl]_2_ (cod is 1,5-cyclooctadiene) were employed under conventional ARO conditions, negligible yields of hydronaphthalene product were observed (Supplementary Table 1). When 5 mol% of cationic rhodium complex Rh(cod)_2_OTf was used, an encouraging combined 15% yield (regiomeric ratio (r.r.) 8.3:1, **17a**:**18a**) was obtained, although the overall reaction outcome was still unsatisfactory.

Promising results were only obtained when a panel of boronic acids was evaluated as potential co-catalysts (Table 1). While aryl-based boronic acids were more commonly employed as catalysts in cases involving carbohydrate diol complexations^62,63,66^, we observed that in our instance vinylboronic acid performed comparatively much better as a catalyst, while aliphatic boronic acids **22** and **23** were deleterious in the reaction. Density functional theory (DFT) studies (Supplementary Section ‘Computational Details’) suggested that fine-tuning electron-donating and conjugation effects on the boronate substituent is pivotal to increase nucleophilicity and also to provide stability to the negatively charged boronate resting-state species. In particular, we have identified the cyclohexylvinylboronic acid **26** (see below) possessing the suitable balance of the above mentioned effects as the optimal co-catalyst candidate. This generated the C3-functionalized hydronaphthalene mannoside **17a** with 92% yield and excellent regio-, diastereo- and enantio-control. Changing the cyclohexyl moiety in **26** to an *n*-octyl moiety also gave reasonably good NMR yields of **17a** at 84% with >20:1 r.r. (**17a**:**18a**). We also used instead the borinic acid **27** under the same conditions. This also gave reasonably good NMR yields of **17a** at 85% with slightly diminished regioselectivity (15:1 r.r., **17a**:**18a**). No by-products arising from the transmetallation pathway were detected by meticulous analysis of the NMR of our crude reaction mixtures.

**Table 1:**
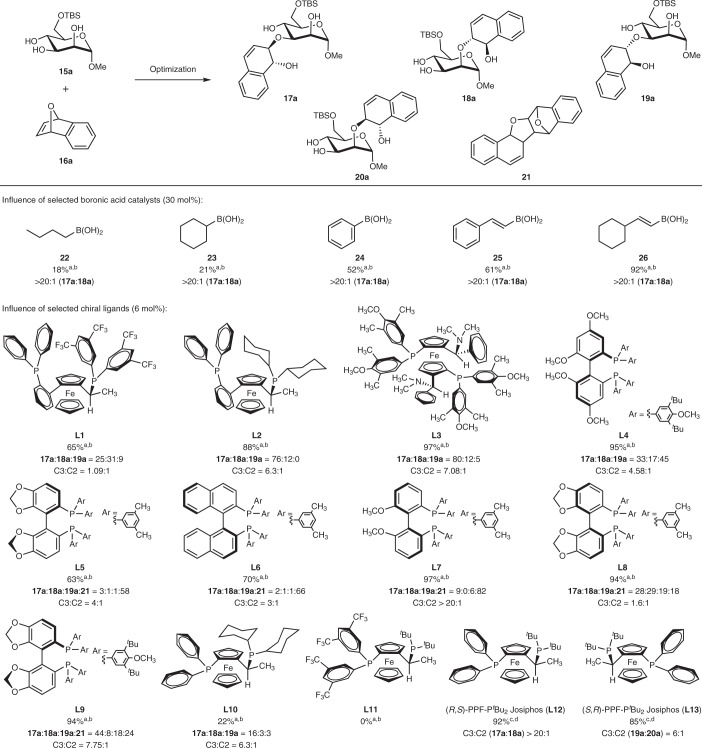
Selected optimization by screening influences of boronic acid catalysts and chiral ligands

Conditions: [a] Polyol **15a** (0.1 mmol), **16a** (0.2 mmol), Rh(cod)_2_OTf (5 mol%), ligand (6 mol%), organoboron catalyst (30 mol%), in THF (1 ml), argon, 50 °C, 24 h. Screening of boronic acid catalyst achieved with **L12** and screening of ligand achieved with boronic acid **26**. [b] Yields and r.r. (C3:C2) ratio were determined by analysis of the crude ^1^H NMR spectra obtained using 1,3,5-trimethoxybenzene as an internal standard. [c] Polyol **15a** (0.2 mmol), **16a** (0.4 mmol), Rh(cod)_2_OTf (5 mol%), ligand (6 mol%), organoboron catalyst (30 mol%), in THF (2 ml), argon, 50 °C, 24 h. [d] Isolated yields. cod, 1,5-cyclooctadiene; TBS, *tert*-butyldimethylsilyl.

Subsequently, we studied a range of chiral bisphosphine ligands across various ligand families and discovered that the chiral ligand choice and its corresponding stereogenic information had a clear influence on stereoselectivity (r.r. and diastereomeric ratio (d.r.)) and in steering the mechanistic pathway of the reaction. When planar chiral ligands based on the ferroceno backbone were screened (Table 1, **L1**–**L3** and **L10**–**L13**), such as those belonging to the Walphos, Mandyphos and Josiphos families, we observed generally good to excellent combined yields of both C3 and C2 regio and diastereoisomers with a range of regioselectivities. A fine balance between the steric hindrance and electronics of these ligands appeared to be pivotal for optimal yields and selectivity.

Bisphosphine ligands with axial chirality such as those based on the Garphos (**L4**), Segphos (**L5**, **L8**, **L9**), BINAP (**L6**) and BIPHEP (**L7**) families were also tested and found to have a diverse influence on the Rh(I) catalysis outcomes. While **L4** gave 95% yield of a mixture of C3 and C2 regioisomers (5:1 r.r.), **L8** and **L9** gave a more complicated mixture containing the C3,C2 regioisomers **17**–**19a** and a cyclodimerized product **21** that was previously observed by Hayashi and colleagues^67^. Notably, when chiral ligands containing the xylyl substituent such as in **L5**–**L7** were used, the synergistic catalysis pathway was significantly suppressed and the cyclodimerization pathway towards **21** was instead favoured.

Overall, the (*R*,*S*)-PPF-P^*t*^Bu_2_ (**L12**) ligand of the Josiphos family in the presence of Rh(cod)_2_OTf provided the most optimal conditions for synergistic catalysis, yielding the C3 regioisomer with excellent yields and selectivity (92%, >20:1 r.r.), while other variants of the same family such as **L10** and **L11** showed inferior performance. When other cationic rhodium complexes bearing norbornadiene (nbd) ligands such as Rh(nbd)_2_OTf and Rh(ndb)_2_BF_4_ were employed under similar conditions, the regioselectivities obtained were also excellent (>20:1 r.r.), although yields were slightly decreased to 81–83% (Supplementary Table 4). Further, when we employed the enantiomeric (*S*,*R*)-PPF-P^*t*^Bu_2_ Josiphos ligand (**L13**), significantly diminished C3,C2 selectivity (6:1 r.r.) was obtained, albeit with similar yields. Our optimization data suggest that a matching ligand–polyol pairing was pivotal in the regioselective control.

### Substrate scope

With these optimal conditions in hand, we proceeded to evaluate the substrate scope of the reaction, and tested a wide range of carbohydrate polyols including mannose, galactose, rhamnose, arabinose, galactal, fucose, lyxose, 1,6-anhydromannose, sedoheptulose and also, significantly, anomeric unprotected saccharides (Table 2).

**Table 2:**
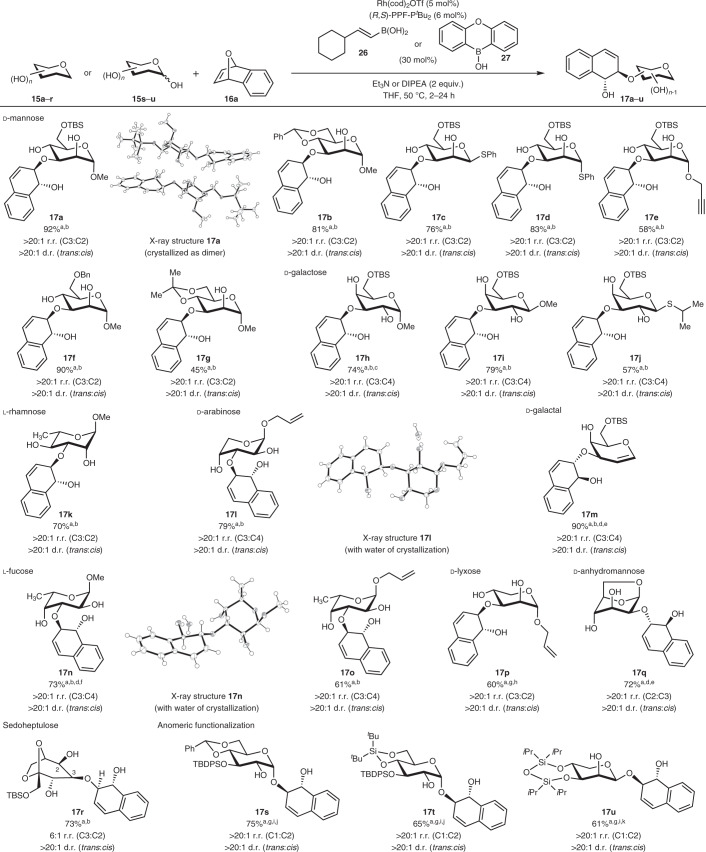
Substrate scope with respect to the carbohydrate polyol

Conditions: [a] Polyol **15a–p** (0.2 mmol), **16a** (0.4 mmol), Rh(cod)_2_OTf (5 mol%), (*R*,*S*)-PPF-P^*t*^Bu_2_ (6 mol%), organoboron catalyst (30 mol%), in THF (2 ml), argon, 50 °C, 24 h. r.r. and d.r. were determined by analysis of the crude ^1^H NMR spectra. [b] **26** was used as the catalyst. [c] 48 h reaction time. [d] 0.5 ml THF was used. [e] (*S*,*R*)-PPF-P^*t*^Bu_2_ was used instead. [f] 12 h reaction time. [g] **27** was used. [h] 6% of C3,C2-disubstituted product was isolated (Supplementary Note 14), 1.5 equiv. **16a** (0.3 mmol) was used. [i] DIPEA was used as the base instead. [j] 2 h reaction time. [k] 13 h reaction time. Bn, benzyl; cod,1,5-cyclooctadiene; TBS, *tert*-butyldimethylsilyl; TBDPS, *tert*-butyldiphenylsilyl. For X-ray structures, thermal ellipsoids shown at 50% probability.

These were very well tolerated in this synergistic catalysis protocol to yield generally *trans*-hydronaphthalene glycosides with excellent regio- and diastereo-selectivity. Both the α- and β-anomers were accommodated in multiple cases, for instance the thiomannosides **17c** and **17d** and the methoxygalactosides **17h**–**i**. When lyxose **15p** was employed, an organoboron reagent switch from the vinylboronic acid **26** to borinic acid catalyst **27** was essential to tolerate this substrate. Interestingly, when galactal and 1,6-anhydromannose **15m** and **15q** were employed, the (*S*,*R*)-PPF-P^*t*^Bu_2_ Josiphos ligand provided instead the matching conditions for excellent regiocontrol to generate **17m** and **17q**, respectively.

We further embarked on the immense challenge of functionalizing the anomeric oxygen of anomeric unprotected saccharides **15s**–**u**, more commonly found in bioactive arylnaphthalene glycosides. Such substrates uniquely contain a non-static hemiacetal moiety that enables dynamic equilibration between the α- and β-anomers, thus elevating the stereoselectivity challenge. Delightfully, by applying catalyst **27** and *N*,*N*-diisopropylethylamine (DIPEA) as a base^68,69^, we were able to functionalize **15s**–**u** cleanly with concomitant enantio-, diastereo-, site- and anomeric selectivity to yield 1,2-*cis* **17s**–**u**. Hence, this unravelled a deeper level of catalytic intricacy: a dynamic kinetic resolution-type control by the organoboron catalyst can also be activated by our strategy to control anomeric selectivity.

In all, our scope suggested that the chirality and identity of the ligand, the three-dimensional structure of the carbohydrate polyol and the electronic effect of the organoboron reagent were collectively contributing to the resulting stereoselectivity and yields. By selecting representative polyol substrates from each of our monosaccharide family of sugars and subjecting them to the opposite enantiomeric chiral ligand under exact conditions, we noted varying carbohydrate substrate influences on yields and regioselectivity (Supplementary Table 30). While deleterious effects were generally observed with mismatching combinations, in cases when mannose, galactal, arabinose, lyxose and 1,6-anhydromannose substrates were employed, the mismatching influence on regioselectivities in these cases was substantial. On the other hand, when galactose, fucose and sedoheptulose substrates were employed, the mismatching combinations resulted instead in significantly diminished yields with marginal influence on regioselectivity. The only exception was l-rhamnose **15k**, where employment of the (*S*,*R*)-PPF-P^*t*^Bu_2_ ligand generated the other diastereomer **19k** with good stereoselectivity and yields.

Taking into consideration structural elucidation ambiguities that could arise from subtle diastereomeric or regioisomeric variations of our products, further confidence in our stereogenic assignments beyond NMR analysis was paramount. Hence, single crystal X-ray crystallography was conducted on derivatives **17a**, **17l** and **17n** (Table 2). Furthermore, we also subjected isolated **17a**, **17s**, **17u**, **19a** and **20a** (**19a** and **20a** obtained using (*S*,*R*)-PPF-P^*t*^Bu_2_ ligand, Table 1) to vibrational circular dichroism (VCD) analysis^70^ and compared the measured spectroscopic data with the DFT-calculated spectra (Supplementary Figs. 1, 2, 4 and 5). Both X-ray crystallography and VCD analysis unambiguously confirmed the absolute and regioisomeric configurations of our hydronaphthalene glycosides that were obtained using both enantiomers of the PPF-P^*t*^Bu_2_ Josiphos ligands. The regiomeric identities of other derivatives were accordingly assigned by analogy and counterchecked with 2-dimensional hetereonuclear multiple bond correlation (HMBC) NMR spectroscopy (Supplementary Section ‘Structural elucidation with the help of HMBC’). The anomeric configurations of **17s**, **17t** and **17u** were further confirmed with the diagnostic ^1^*J*_C–H_ coupling constant and 2D-nuclear Overhauser effect spectroscopy (NOESY) (Supplementary Note 15 and Supplementary Figs. 409–414).

To assess the scope of our method with respect to the electrophile, we tested a range of *meso*-oxanorbornadienes **16b**–**i** bearing a variety of electron-donating and electron-withdrawing moieties (Table 3). Methyl, methoxyl and dioxolane groups in different aromatic positions on the oxanorbornadienes were accommodated to generate the hydronaphthalene glycosides with excellent stereocontrol and yields. Bridgehead oxanorbornadienes can also be employed to yield **17ae** efficiently. In addition, electron-withdrawing halogens such as fluorines and bromines can be incorporated to yield **17af** and **17ag** with very good yields and stereoselectivities. The additional fusion of a benzene ring in oxabicycle **16i** was also well tolerated to yield **17ah** effectively.

**Table 3:**
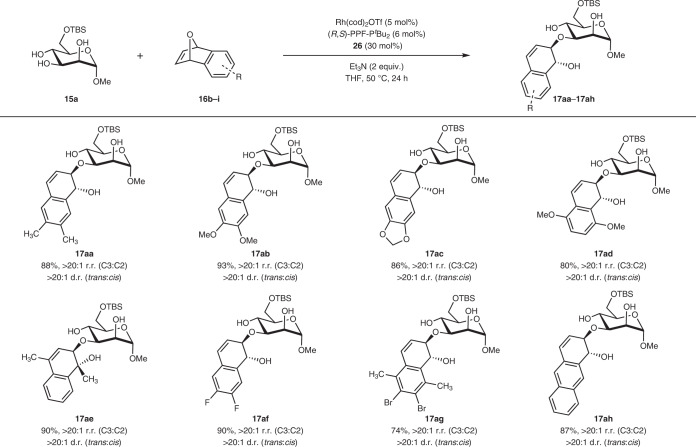
Expanded substrate scope with respect to the *meso*-oxanorbornadiene

Conditions: Polyol **15a** (0.2 mmol), **16aa**–**ah** (0.4 mmol), Rh(cod)_2_OTf (5 mol%), (*R*,*S*)-PPF-P^*t*^Bu_2_ (6 mol%), organoboron catalyst **26** (30 mol%), in THF (2 ml), argon, 50 °C, 24 h. r.r. and d.r. were determined by analysis of the crude ^1^H NMR spectra. TBS, *tert*-butyldimethylsilyl.

Taking our methodology a step further, we sought to evaluate if this synergistic concept could also be extended to the structurally related allylic carbonate substrates **16j**–**m** (Table 4). While ligand and solvent modifications were found to be essential^71^, we noted that the reaction still proceeded smoothly to yield the corresponding functionalized products **17v**–**za** with concomitant regio and diastereoselectivity. We further observed that the allylic substitution can be extended to allylic carbonates with sterically and electronically differing substituents ranging from aliphatic and cyclic to aromatic moieties. Significantly, since a racemic mixture of the allylic carbonate **16j**–**m** was employed, an additional facet of dynamic kinetic resolution by the Rh(I) catalyst on the electrophile is operative in such systems, further confirming the rich multifaceted stereocontrol through our strategy. The absolute configuration of **17v** and its mismatched congener **20v** was further ascertained using VCD analysis and the other derivatives were assigned by analogy (Supplementary Fig. 3). Further, the regioselectivity of all allylic substitution products was double checked with 2-dimensional HMBC NMR spectroscopy (Supplementary Section ‘Structural elucidation with the help of HMBC’).

**Table 4:**
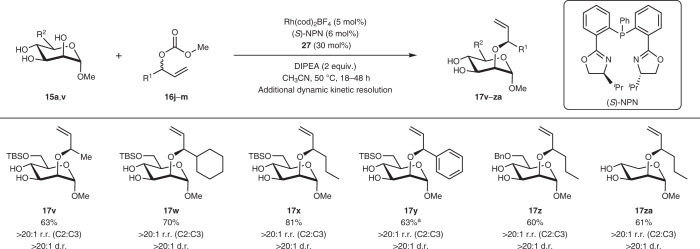
Expanded substrate scope using allylic carbonates as electrophiles

Conditions: Polyol **15a** (0.2 mmol), **16i** (0.24 mmol), Rh(cod)_2_BF_4_ (5 mol%), (*S*)-NPN (6 mol%), organoboron catalyst **27** (30 mol%), in CH_3_CN (1 ml), argon, 50 °C, 18-48 h. r.r. and d.r. were determined by analysis of the crude ^1^H NMR spectra. [a] 10 mol% Rh(cod)_2_BF_4_, 12 mol% (*S*)-NPN, 70 °C. cod,1,5-cyclooctadiene; TBS, *tert*-butyldimethylsilyl.

### Mechanistic study

**Fig. 2:**
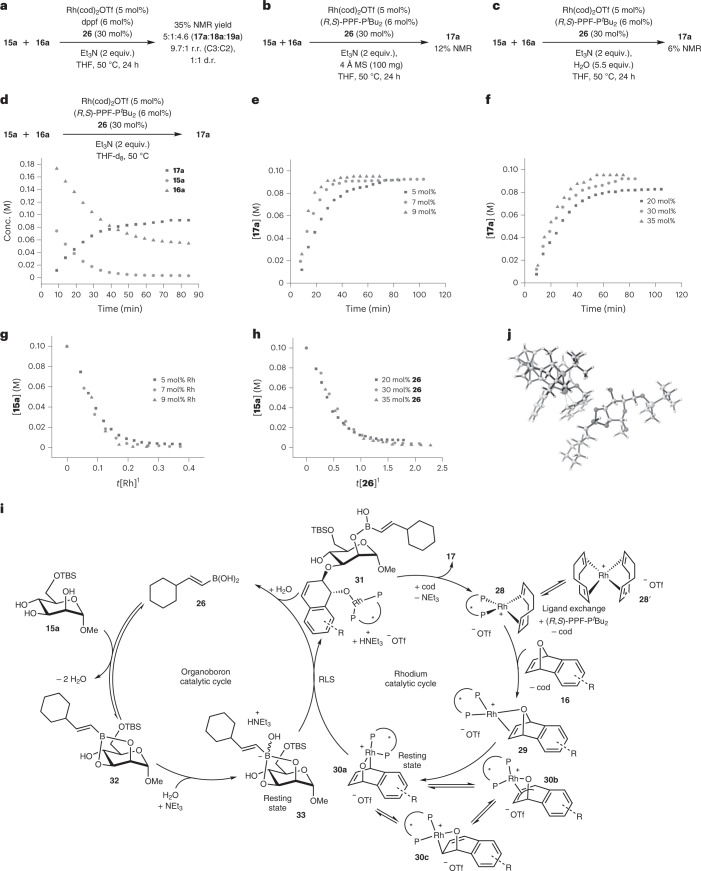
Mechanistic insights into the synergistic catalysis through control experiments, kinetic investigation and DFT computations. **a**, Control experiment using achiral dppf as the ligand revealed depletion of yields and stereoselectivity. **b**, Control experiment adding 4 Å molecular sieves (MS) to sequester H_2_O was deleterious to product yield. **c**, Control experiment adding 5.5 equiv. H_2_O under standard conditions gave trace product yield. **d**, Temporal kinetic profile by in situ NMR monitoring. **e**, Influence of rhodium catalyst concentration on the kinetic profile. **f**, Influence of boronic acid catalyst **26** on the kinetic profile. **g**, Burés overlaid plot with respect to rhodium catalyst concentration. **h**, Burés overlaid plot with respect to boronic acid **26** concentration. **i**, Proposed mechanism of the site-selective functionalization by Rh/organoboron synergistic catalysis. The two interlocking catalytic cycles are comprised of the organoboron catalytic cycle (left) and the rhodium catalytic cycle (right). The reversible covalent complexation of the boronic acid **26** and the polyol **15a** forms eventually the resting-state organoboronate **33**. Similarly, the Rh(I)/Rh(III) redox couple forms the resting-state bridged Rh(III) intermediate **30a**. Compounds **33** and **30a** then react in the rate-limiting step (RLS) through multiple stereocontrol to generate stereoselectively **17**. **j**, Computed transition state with Gibbs free energy barrier of 18.3 kcal mol^–1^ of the rate-limiting step at the *ω*B97M-V/def2-QZVPP/CPCM(THF)//r^2^SCAN-3c/CPCM(THF) level of theory.

To shed further light on the mechanism, we conducted a series of control experiments. First, we employed the 1,1′-bis(diphenylphosphino)ferrocene (dppf) achiral ligand instead of (*R*,*S*)-PPF-P^*t*^Bu_2_ with our optimized standard conditions (Fig. 2a). This ligand permutation resulted in a concurrent depletion of yields, regio- and diastereo-selectivity. Furthermore, the depletion of yields and stereocontrol in a separate control experiment in the absence of boronic acid (15% yield, 8.3:1 r.r. (C3:C2), >20:1 d.r., see Supplementary Section ‘Control Experiment 1’) under standard conditions corroborate the pivotal role both catalysts play in the exquisite stereocontrol of the reaction mechanism.

The crucial role of water in the mechanism is worthy of special mention. We accordingly performed two control experiments to understand the role of water in the catalytic cycle (Fig. 2b,c). Our data suggest that fine-balancing of the catalytic amount of water liberated from the condensation of the boronic acid **26** on a *cis*-diol motif was pivotal. Addition of 4 Å molecular sieves to sequester the liberated water was deleterious and suppressed the reaction. Conversely, addition of 5.5 equiv. H_2_O into the reaction only gave trace amounts of product, as detected by ^1^H NMR. These observations suggest that the turnover of liberated catalytic water at equilibrium during the in situ formation of the boronic ester from the polyol is absolutely essential in the overall mechanism.

We then conducted in situ NMR monitoring of the model reaction between **15a** and **16a** under standard conditions to obtain the temporal kinetic profile of this synergistic protocol (Fig. 2d). To evaluate the influence of the catalysts on the kinetic profile, in situ NMR monitoring was then performed at different catalyst concentrations. The overlay of these profiles revealed a positive kinetic correlation with respect to both Rh and boronic acid catalyst concentrations (Fig. 2e,f). This supports the hypothesis that the synergistic actions of these two catalysts are vital in the rate-limiting step of the reaction. By employing the graphical method reported by Burés^72^, we further determined a first-order kinetic dependence with respect to Rh, as well as a first-order kinetic dependence with respect to boronic acid (Fig. 2g,h).

Additionally, we noted that the kinetic profiles overlapped well and were rather invariant to changes in concentrations of both the NEt_3_ base and oxabicycle (Supplementary Figs. 22 and 26), indicating that they were not actively involved in the rate-limiting step. Interestingly, we noticed a negative kinetic correlation with respect to the increase of concentration of carbohydrate polyol **15a** (Supplementary Fig. 18), which suggested the presence of off-cycle secondary effects in the reaction.

Based on observations from our previous work on non-covalent catalysed glycosylations^37,73^, as well as literature reports documenting the formation of hydrogen-bonded aggregates between carbohydrates^74^, we surmised that this observed negative order could be accounted for by the formation of intermolecular hydrogen-bonded carbohydrate clusters at higher concentrations, which may lower the reactivity of the carbohydrate polyols, hence retarding the formation of the boronic ester. We propose a synergistic mechanism consisting of two interlocking catalytic cycles (Fig. 2i). In the rhodium catalytic cycle, the cationic Rh(cod)_2_OTf complex **28′** will first undergo ligand exchange with the (*R*,*S*)-PPF-P^*t*^Bu_2_ ligand to yield complex **28**, followed by the departure of the first cod ligand. Compound **28** will further undergo ligand exchange with oxanorbornadiene **16** with the concomitant expulsion of the second cod ligand to form the *exo*-coordinated intermediate **29**. Subsequently, a Rh(I) oxidative addition will yield the Rh(III) intermediate **30a**, which we propose is the resting state of the rhodium catalyst. Based on previous reports^75^, **30a** can also exist in equilibrium with other resonance structures such as the rhodium-π-allyl complex **30b** and the rhodaoxetane **30c**. Simultaneously, the organoboron catalytic cycle (Fig. 2i, left) is also operative. We postulate that cyclohexylvinylboronic acid **26** will first undergo a reversible condensation with the vicinal *cis*-diol motif on the carbohydrate polyol **15** to generate the boronic ester **32**. Two molecules of water would be liberated in this conversion, which will be consumed in subsequent elementary steps. Compound **32** will then react with a molecule of liberated water in the presence of the NEt_3_ base to yield the more nucleophilic boronate complex **33**, which we propose as the resting state of the organoboron catalyst.

In the rate-limiting step, the reaction between the resting states **30a** and **33** of the rhodium and organoboron catalysts enabled a highly stereocontrolled site-selective outer-sphere attack of the equatorial O3 of **33** on the *endo* face of **30a** in an S_N_2′ fashion. A subsequent reductive elimination led to the generation of boronic acid hemiester **31** and the ammonium hydrotriflate by-product. To gain further insights into the proposed boronate resting-state intermediate **33** and the feasibility of the pivotal rate-limiting step connecting both catalytic cycles, we conducted DFT calculations using ORCA ^76^ at the *ω*B97M-V/def2-QZVPP/CPCM(THF)//r^2^SCAN-3c/CPCM(THF) level of theory^77,78^ to model the reaction path of the rate-limiting step. Delightfully, we successfully located the modelled transition state connecting **33**, **30a** and **31** (Fig. 2j and Supplementary Section ‘Computational Details’). Furthermore, we computed a kinetically feasible 18.3 kcal mol^–1^ Gibbs free energy barrier for this elementary step. Finally, hydrolysis with a second molecule of liberated water, a proton transfer from the ammonium hydrotriflate salt and a ligand exchange with a molecule of cod will regenerate both catalysts **28** and **26**, thereafter restarting both synergistic catalytic cycles.

**Fig. 3:**
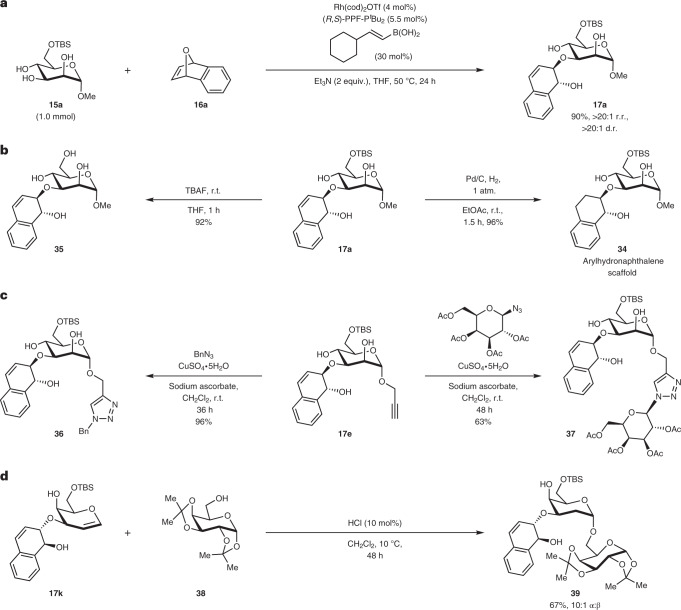
Upscaling example, further derivatizations and transformations. **a**, Larger-scale reaction using 1 mmol of **15a** at reduced catalyst loadings. **b**, Functional group interconversion of the methodology product through TBS deprotection (left) or alkene hydrogenation using palladium on a charcoal catalyst (right). **c**, Copper-catalysed click functionalizations on propargyl derivative **17e** that enables facile attachment of Bn (left) or saccharide (right) moieties. **d**, Acid-catalysed 2-deoxyglycosylation of galactal-containing products to access disaccharide **39**. Bn, benzyl; r.t., room temperature.

To evaluate the upscaling potential of our protocol, we conducted a 1-mmol-scale reaction at reduced catalyst loading (4 mol% Rh) and obtained **17a** with an almost identical result (90% yield, >20:1 r.r.) as for the 0.2 mmol reaction (Fig. 3a). Further derivatizations also proceeded smoothly (Fig. 3b). Hydrogenation of **17a** catalysed by palladium on charcoal under ambient conditions allowed entry into arylhydronaphthalene glycoside **34** with quantitative yield. The *tert*-butyldimethylsilyl (TBS) group on the O6 of **17a** can also be deprotected using tetra-*n*-butylammonium fluoride (TBAF) in a facile fashion to generate **35** with 92% yield. Employing glycosyl substrates with a propargyl moiety tethered to the anomeric carbon enabled the incorporation of ‘click’ chemistry^79^, allowing facile access into click derivatives such as **36** or a synthetic disaccharide **37** that expands the synthetic utility of these hydronaphthalene glycosides (Fig. 3c). Lastly, the galactal derivative **17k** can be subjected to an acid-catalysed 2-deoxyglycosylation with **38** as the glycosyl acceptor to generate disaccharide **39** in a highly α-selective fashion (Fig. 3d), enabling compatibility of our products with the biologically relevant glycosidic linkage formation^80^.

## Conclusion

In conclusion, we have demonstrated the employment of chiral Rh(I) catalysis synergistically with boronic acid catalysis in site-selective carbohydrate functionalizations,^81^ surmounting the challenge of multiple regio-, diastereo-, enantio- and anomeric control within a single bond-forming step. Thus, this method paves the way for stereoselective access into biologically relevant arylnaphthalene glycosides. An additional dimension of dynamic kinetic resolution by the catalytic system can also be observed for substrates such as anomeric unprotected saccharides or racemic allylic carbonates. Furthermore, the successful execution of this strategy required a challenging evasion of the decades-long established boron to Rh(I) transmetallation pathway. Hence, our strategy defines a compatible co-catalytic role that organoboron reagents can play in the realm of Rh(I) catalysis, thereby opening up future site-selective opportunities that could assimilate the repertoire of chiral Rh(I)-catalysed functionalizations in complex carbohydrate synthesis. Furthermore, kinetic and DFT studies provide further evidence that the resting states of the Rh and the organoboron catalysts are actively participating in the rate-limiting step. We anticipate that this protocol would inspire further work in the development of chiral transition catalytic systems for challenging site-selective functionalizations of carbohydrates with prochiral electrophiles—an endeavour that will potentially unravel novel synthetic solutions towards hitherto inaccessible but biologically important glycosides.

## Methods

### General procedure for the synergistic Rh(I)/organoboron-catalysed site-selective functionalization

An oven-dried dram vial purged with an argon balloon was charged with (*R*,*S*)-PPF-P^*t*^Bu_2_ Josiphos ligand (6.51 mg, 0.012 mmol, 6 mol%), organoboron **26** or **27** (0.06 mmol, 30 mol%), oxabicycle **16** (0.4 mmol, 2 equiv.), polyol **15** (0.2 mmol, 1 equiv.) and Rh(cod)_2_OTf (4.68 mg, 0.01 mmol, 5 mol%) and then dry THF (2 ml) and NEt_3_ or DIPEA (0.4 mmol, 2 equiv.) were added. The dram vial was sealed and the mixture was immersed in a 50 °C oil bath and stirred for 24–48 h. Upon completion of the reaction, the reaction mixture was filtered over a short silica plug and flushed with 250–300 ml ethyl acetate. The filtrate was then evaporated and the determination of the regiomeric ratio (r.r.) was then performed using ^1^H NMR analysis of this concentrated crude mixture with 1,3,5-trimethoxybenzene as the internal standard. The crude mixture was subsequently dry-loaded onto silica gel and subjected to flash column chromatography for purification.

## Online content

Any methods, additional references, Nature Portfolio reporting summaries, source data, extended data, supplementary information, acknowledgements, peer review information; details of author contributions and competing interests; and statements of data and code availability are available at 10.1038/s41557-022-01110-z.

## Supplementary information

Supplementary Information Supplementary Figs. 1–414, Tables 1–52 and Notes 1–16.

Supplementary Data 1 Cartesian coordinates of structures from VCD calculations.

Supplementary Data 2 Crystallographic data for compound **17a**; CCDC reference 2097738.

Supplementary Data 3 Crystallographic data for compound **17l**; CCDC reference 2097739.

Supplementary Data 4 Crystallographic data for compound **17n**; CCDC reference 2097740.

Supplementary Data 5 Cartesian coordinates (.xyz) of all DFT-computed structures for the mechanistic understanding.

## Data Availability

The data supporting the findings of this study are available within the Article and its Supplementary Information files. Crystallographic data for the structures reported in this Article have been deposited at the Cambridge Crystallographic Data Centre, under deposition numbers CCDC 2097738 (**17a**), CCDC 2097739 (**17l**) and CCDC 2097740 (**17n**). Copies of the data can be obtained free of charge via https://www.ccdc.cam.ac.uk/structures/.
