# Supplementary material for: A synergistic Rh(I)/organoboron-catalysed site-selective carbohydrate functionalization that involves multiple stereocontrol
Source: Nat Chem. 2022 Dec 30;15(3):424–35. doi: 10.1038/s41557-022-01110-z (PMC9986112; doi:10.1038/s41557-022-01110-z)
Supplement: Supplementary file 1 — Supplementary Figs. 1–414, Tables 1–52 and Notes 1–16. [file 41557_2022_1110_MOESM1_ESM.pdf]

nature chemistry

Article

https://doi.org/10.1038/s41557-022-01110-z

A synergistic Rh(I)/organoboron-catalysed site-selective carbohydrate functionalization that involves multiple stereocontrol

In the format provided by the authors and unedited

Table of Contents

Table of contents…………………………………………………………………………………………1
Supplementary methods……………………………………………………………………………………2
General information……………………………………………………………………………………2
General procedures…………………………………………………………………………………2-4
General experimental procedure for reduction………………………………………………………4
General experimental procedure for the deprotection of TBS ether………………………………4
General experimental procedure for CLICK reaction…………………………………………………4
General experimental procedure for synthesis of disaccharide……………………………………4
General method for *in-situ* NMR monitoring……………………………………………………………4
NMR monitoring under standard condition……………………………………………………………4
General method for concentration dependence studies…………………………………………5-7
Rh(cod)$_2$OTf catalyst concentration dependence…………………………………………………5
Boronic acid **26** catalyst concentration dependence………………………………………………5
Carbohydrate polyol **15a** concentration dependence…………………………………………5-6
Oxabicycle **16a** concentration dependence…………………………………………………………6
Triethylamine concentration dependence…………………………………………………………6-7
Procedure for initial optimization…………………………………………………………………………7
Methods for control experiment……………………………………………………………………7-8
Synthesis and characterization of starting materials……………………………………………8-20
Optimization studies…………………………………………………………………………………………21
Supplementary table S1: Initial screening search for suitable catalyst……………………………21
Supplementary table S2: Screening of chiral ligands…………………………………………………22
Supplementary table S3: Screening of chiral ligands…………………………………………………23
Supplementary table S4: Screening of cationic rhodium catalysts…………………………………24
Supplementary table S5: Conditions screening for allylic carbonate reaction……………………24
Control experiments……………………………………………………………………………………24-26
Optimization table for lyxose **17p** ……………………………………………………………………26
Absolute configurations of **17a-20a, 17s,u,v and 20v** by VCD spectroscopy…………………27-32
X-Ray crystallography data……………………………………………………………………………33-58
Structural elucidation with the help of HMBC………………………………………………………58-59
Supplementary Notes…………………………………………………………………………………59-60
Mismatching combinations using the opposite enantiomeric ligand ……………………………61-62
NMR data………………………………………………………………………………………………63-92
NMR monitoring data for kinetics studies………………………………………………………………93
NMR monitoring under standard conditions……………………………………………………93-94
Concentration dependence studies……………………………………………………………………95
Rh(cod)$_2$OTf -Catalyst concentration dependence……………………………………………96-99
Boronic acid **26** Catalyst concentration dependence…………………………………………100-102
Carbohydrate polyol **15a** concentration dependence…………………………………………103-106
Oxabicycle **16a** concentration dependence……………………………………………………107-110
Base Et_3N concentration dependence…………………………………………………………111-114
Kinetic Analysis………………………………………………………………………………………………115
Bures Method to determine the order with respect to Rh(cod)$_2$OTf…………………………115-119
Bures Method to determine the order with respect to **26**……………………………………119-123
Computational Details……………………………………………………………………………124-132
References……………………………………………………………………………………………132-136
NMR Spectra…………………………………………………………………………………………137-323
Proton coupled ^{13}C spectra and NOESY for determining anomeric configuration…………323-326

Supplementary Methods

General Information: Unless otherwise stated, all reactions were set up under inert atmosphere (argon) utilizing glassware that were oven dried and cooled under argon purging. Silica Gel Flash Column Chromatography was performed on *Silica gel Merck 60* (*particle size 40-63 µm.* Starting materials were purchased directly from commercial suppliers (Sigma Aldrich, Acros, Alfa Aesar, VWR, TCI) and used without further purifications unless otherwise stated. All solvents were dried according to standard procedures or brought from commercial suppliers. Reactions were monitored using thin-layer chromatography (TLC) on *Merck silica gel aluminium plates with F254 indicator*. Visualization of the developed plates was performed under UV light (254 nm) or $KMnO_4$ stain or H_2SO_4-EtOH (10% H_2SO_4 v/v).

NMR characterization data (^{1}H NMR, ^{13}C NMR and 2D spectra) were collected at 300 K on a *Bruker DRX400 (400 MHz)*, *Bruker DRX500 (500 MHz)*, *INOVA500 (500 MHz), Bruker DRX600 (600 MHz)* and *Bruker DRX700 (700 MHz)* using $CDCl_3$, THF-d_8 and CD_3OD as solvent. Data for ^{1}H NMR are reported as follows: chemical shift (δ ppm), multiplicity (s = singlet, d = doublet, t = triplet, q = quartet, m = multiplet, br = broad), coupling constant (Hz), integration with the solvent resonance as internal standard ($CDCl_3$: δ = 7.26 ppm for ^{1}H, δ = 77.16 ppm for ^{13}C; CD_3OD: δ = 4.87, 3.31 ppm for ^{1}H, δ = 49 ppm for ^{13}C of CD_3, C_4D_8O (THF-d_8) : δ = 3.58, 1.73 ppm for ^{1}H, δ = 67.57, 25.37 ppm for ^{13}C).

High resolution mass spectra were recorded on an *LTQ Orbitrap* mass spectrometer coupled to an *Accela HPLC*-System (HPLC column: *Hypersyl GOLD*, 50 mm x 1 mm, particle size 1.9 µm, ionization method: electron spray ionization) and Bruker ultrafleXtreme MALDI-TOF-TOF (3 decimal accuracy). Optical rotations were measured in a *Schmidt + Haensch Polartronic HH8* polarimeter equipped with a sodium lamp source (589 nm), and are reported as follows: $[\alpha]_D^{T\,°C}$ (c = g/100 mL, solvent).

The ratio of regiomers was determined by ^{1}H-NMR of the crude reaction mixture *via* integration of characteristic signals in the ^{1}H NMR spectra, Chemical yields refer to isolated substances after flash column chromatography. NMR yields were determined using 1,3,5-trimethoxybenzene or mesitylene as internal standard.

General procedure for optimization of site selective functionalization:(see Supplementary table S1) To an oven dried dram vial purged with an argon balloon was charged with ligand (5 mol%), boronic acid (30 mol%), oxabicycle (0.1 mmol, 1-3 equiv.), carbohydrate polyol (1-2 eq), Rh metal-catalyst (5 mol%) and then dry THF (1 mL), Base were added. The dram vial was sealed and the mixture was stirred at a preheated temperature. Upon completion of the reaction, the reaction mixture was filtered over a short silica plug and flushed with 150-200 mL of ethyl acetate or methanol. The filtrate was then evaporated and the determination of the regiomeric ratio (r.r.) is by ^{1}H NMR analysis of this concentrated crude mixture with 1,3,5-trimethoxybenzene as the internal standard. The crude mixture is subsequently dry loaded onto silica gel and subjected to flash column chromatography for purification.

General procedure for site selective functionalization:

Procedure A: To an oven dried dram vial purged with an argon balloon was charged with (*R,S*)-PPF-P^{t}Bu$_2$ Josiphos ligand (6.51 mg, 0.012 mmol, 6 mol%), cyclohexyl vinyl boronic acid **26** (9.24 mg, 0.06 mmol, 30 mol%), oxabicycle **16** (0.4 mmol, 2 equiv.), carbohydrate polyol **15** (0.2 mmol, 1 equiv.), Rh(cod)$_2$OTf (4.68 mg, 0.01 mmol, 5 mol%) and then dry THF (2 mL), triethyl amine base (0.4 mmol, 2 equiv.) were added. The dram vial was sealed and the mixture was immersed in a 50 °C oil bath stirred for 24-48 h. Upon completion of the reaction, the reaction mixture was filtered over a short silica plug and flushed with 250-300 mL of ethyl acetate. The filtrate was then evaporated and the determination of the regiomeric ratio (r.r.) is by ^{1}H-NMR analysis of this concentrated crude mixture with 1,3,5-trimethoxybenzene as the internal standard. The crude mixture is subsequently dry loaded onto silica gel and subjected to flash column chromatography for purification.

Procedure B: To an oven dried dram vial purged with an argon balloon was charged with (*S,R*)-PPF-P ^{t}Bu$_2$ Josiphos ligand (6.51 mg, 0.012 mmol, 6 mol%), cyclohexyl vinyl boronic acid **26** (9.24 mg, 0.06 mmol, 30 mol%), oxabicycle **16** (0.4 mmol, 2 equiv.), carbohydrate polyol **15** (0.2 mmol, 1 equiv.), Rh(cod)$_2$OTf (4.68 mg, 0.01 mmol, 5 mol%) and then dry THF (2 mL), triethyl amine base (0.4 mmol, 2

equiv.) were added. The dram vial was sealed and the mixture was immersed in a 50 °C oil bath stirred for 24-48 h. Upon completion of the reaction, the reaction mixture was filtered over a short silica plug and flushed with 250-300 mL of ethyl acetate. The filtrate was then evaporated and the determination of the regiomeric ratio (r.r.) is by ^{1}H-NMR analysis of this concentrated crude mixture with 1,3,5-trimethoxybenzene as the internal standard. The crude mixture is subsequently dry loaded onto silica gel and subjected to flash column chromatography for purification.

Procedure C: To an oven dried dram vial purged with an argon balloon was charged with (*R,S*)-PPF-P^{t}Bu$_2$ Josiphos ligand (6.51 mg, 0.012 mmol, 6 mol%), borinic acid **27** (11.7 mg, 0.06 mmol, 30 mol%), oxabicycle **16** (0.3 mmol, 1.5 equiv.), carbohydrate polyol **15** (0.2 mmol, 1 eq), Rh(cod)$_2$OTf (4.68 mg, 0.01 mmol, 5 mol%) and then dry THF (2 mL), triethyl amine base (0.4 mmol, 2 equiv.) were added. The dram vial was sealed and the mixture was immersed in a 50 °C oil bath stirred for 24-48 h. Upon completion of the reaction, the reaction mixture was filtered over a short silica plug and flushed with 250-300 mL of ethyl acetate. The filtrate was then evaporated and the determination of the regiomeric ratio (r.r.) is by ^{1}H-NMR analysis of this concentrated crude mixture with 1,3,5-trimethoxybenzene as the internal standard. The crude mixture is subsequently dry loaded onto silica gel and subjected to flash column chromatography for purification.

Procedure D: To an oven dried dram vial purged with an argon balloon was charged with (*S,R*)-PPF-P ^{t}Bu$_2$ Josiphos ligand (6.51 mg, 0.012 mmol, 6 mol%), borinic acid **27** (11.7 mg, 0.06 mmol, 30 mol%), oxabicycle **16** (0.4 mmol, 2 equiv.), carbohydrate polyol **15** (0.2 mmol, 1 eq), Rh(cod)$_2$OTf (4.68 mg, 0.01 mmol, 5 mol%) and then dry THF (2 mL), triethyl amine base (0.4 mmol, 2 equiv.) were added. The dram vial was sealed and the mixture was immersed in a 50 °C oil bath stirred for 24-48 h. Upon completion of the reaction, the reaction mixture was filtered over a short silica plug and flushed with 250-300 mL of ethyl acetate. The filtrate was then evaporated and the determination of the regiomeric ratio (r.r.) is by ^{1}H-NMR analysis of this concentrated crude mixture with 1,3,5-trimethoxybenzene as the internal standard. The crude mixture is subsequently dry loaded onto silica gel and subjected to flash column chromatography for purification.

General procedure for anomeric functionalization:

Procedure A1: To an oven dried dram vial purged with an argon balloon was charged with (*R,S*)-PPF-P ^{t}Bu$_2$ Josiphos ligand (6.51 mg, 0.012 mmol, 6 mol%), Taylor's boronic acid **27** (11.76 mg, 0.06 mmol, 30 mol%), oxabicycle **16** (0.4 mmol, 2 equiv.), carbohydrate polyol **15** (0.2 mmol, 1 equiv.), Rh(cod)$_2$OTf (4.68 mg, 0.01 mmol, 5 mol%) and then dry THF (2 mL), DIPEA base (0.4 mmol, 2 equiv.) were added. The dram vial was sealed and the mixture was immersed in a 50 °C oil bath stirred for 2-6 h. Upon completion of the reaction, the reaction mixture was filtered over a short silica plug and flushed with 30-50 mL of ethyl acetate. The filtrate was then evaporated and the determination of the regiomeric ratio (r.r.) is by ^{1}H-NMR analysis of this concentrated crude mixture with 1,3,5-trimethoxybenzene as the internal standard. The crude mixture is subsequently dry loaded onto silica gel and subjected to flash column chromatography for purification.

Procedure B1: To an oven dried dram vial purged with an argon balloon was charged with (*S,R*)-PPF-P ^{t}Bu$_2$ Josiphos ligand (6.51 mg, 0.012 mmol, 6 mol%), Taylor's boronic acid **27** (11.76 mg, 0.06 mmol, 30 mol%), oxabicycle **16** (0.4 mmol, 2 equiv.), carbohydrate polyol **15** (0.2 mmol, 1 equiv.), Rh(cod)$_2$OTf (4.68 mg, 0.01 mmol, 5 mol%) and then dry THF (2 mL), DIPEA base (0.4 mmol, 2 equiv.) were added. The dram vial was sealed and the mixture was immersed in a 50 °C oil bath stirred for 16 h. Upon completion of the reaction, the reaction mixture was filtered over a short silica plug and flushed with 30-50 mL of ethyl acetate. The filtrate was then evaporated and the determination of the regiomeric ratio (r.r.) is by ^{1}H-NMR analysis of this concentrated crude mixture with 1,3,5-trimethoxybenzene as the internal standard. The crude mixture is subsequently dry loaded onto silica gel and subjected to flash column chromatography for purification.

General procedure for allylic carbonate functionalization:

Procedure C1: To an oven dried dram vial purged with an argon balloon was charged with (*S*)-NPN ligand (5.82 mg, 0.012 mmol, 6 mol%), Taylor's boronic acid **27** (11.76 mg, 0.06 mmol, 30 mol%), allylic carbonate **16** (0.24 mmol, 1.2 equiv.), carbohydrate polyol **15** (0.2 mmol, 1 equiv.), Rh(cod)$_2$BF$_4$ (4.06

mg, 0.01 mmol, 5 mol%) and then dry acetonitrile (1 mL), DIPEA base (0.4 mmol, 2 equiv.) were added. The dram vial was sealed and the mixture was immersed in a 50 °C oil bath stirred for 18-41 h. Upon completion of the reaction, the reaction mixture was filtered over a short silica plug and flushed with 30-50 mL of ethyl acetate. The filtrate was then evaporated and the determination of the regiomeric ratio (r.r.) is by ^{1}H-NMR analysis of this concentrated crude mixture with 1,3,5-trimethoxybenzene as the internal standard. The crude mixture is subsequently dry loaded onto silica gel and subjected to flash column chromatography for purification.

Procedure D1: To an oven dried dram vial purged with an argon balloon was charged with (*R*)-NPN ligand (5.82 mg, 0.012 mmol, 6 mol%), Taylor's boronic acid **27** (11.76 mg, 0.06 mmol, 30 mol%), allylic carbonate **16** (0.24 mmol, 1.2 equiv.), carbohydrate polyol **15** (0.2 mmol, 1 equiv.), $Rh(cod)_2BF_4$ (4.06 mg, 0.01 mmol, 5 mol%) and then dry acetonitrile (1 mL), DIPEA base (0.4 mmol, 2 equiv.) were added. The dram vial was sealed and the mixture was immersed in a 50 °C oil bath stirred for 18-24 h. Upon completion of the reaction, the reaction mixture was filtered over a short silica plug and flushed with 30-50 mL of ethyl acetate. The filtrate was then evaporated and the determination of the regiomeric ratio (r.r.) is by ^{1}H-NMR analysis of this concentrated crude mixture with 1,3,5-trimethoxybenzene as the internal standard. The crude mixture is subsequently dry loaded onto silica gel and subjected to flash column chromatography for purification.

General experimental procedure for reduction:[35] **Procedure E:** Compound **17a** (90.4mg, 1 equiv., 0.2mmol) was dissolved in EtOAc (2 mL) in a 5 mL flask with a magnetic stirring bar. Palladium on charcoal (10%, 24 mg, 0.1 equiv.) was added to the solution and the flask was covered with a septa. The atmosphere in the flask was replaced by hydrogen by evacuating the gas phase thrice till the solvent started to boil and refilling it with a hydrogen balloon. The balloon was remained on the flask and the suspension was stirred vigorously for 1.5 h at rt. The reaction mixture was filtered through a short plug of silica, concentrated under reduced pressure and purified by flash column chromatography (dry loading, 2:1-1:1 Pentane: Ethyl acetate).

General experimental procedure for the deprotection of TBS ether:[36] **Procedure F:** A mixture of TBS ether **17a** (90.4 mg, 0.2 mmol) and TBAF (0.4mL, 0.4 mmol) in THF (2 mL) was stirred at room temperature for 1h. After completion of the reaction, as indicated by TLC, was concentrated under reduced pressure and the crude compound was purified by column chromatography over silica gel to afford the pure alcohol.

General experimental procedure for CLICK reaction:[37] **Procedure G:** Compound **17e** (90.4 mg, 0.2 mmol), $CuSO_4•5H_2O$ (10 mol%), sodium ascorbate (10 mol%), corresponding azide CLICK partner (0.6 mmol, 3 equiv.) and a mixed solution of CH_2Cl_2 (1 mL) and water (1 mL) was added to the residue under air. The tube was then sealed and stirred at room temperature for 36 h-48 h. The mixture was diluted with 5 mL of dichloromethane and 5 mL of water. The organic phases were separated, dried with sodium sulfate, and concentrated at reduced pressure and purified by column chromatography (dry loading).

General experimental procedure for synthesis of disaccharide:[38] **Procedure H:** General procedure for 2-deoxy glycosylation: A mixture of glycosyl donor **17k** (3 equiv.), acceptor (0.05 mmol, 1 equiv.) in dry dichloromethane (0.5 mL) was stirred under argon, the mixture was cooled to 10 ^{o}C, 1M HCl in diethyl ether (5 µL, 10 mol%) was added, the resulting mixture was stirred at the same temperature for 48h, upon completion Et_3N (4 µL) was added and concentrated, anomeric ratio were determined by comparison of the integral intensities of relevant signals in ^{1}H NMR spectra.

General method for *in-situ* NMR monitoring: NMR monitoring under standard condition: (See Supplementary table S33, Supplementary figure S6-7): To a dry NMR tube charged with trimethoxy benzene (8.4 mg 1 equiv.), (*R, S*)-PPF-P^{t}Bu$_2$ Josiphos ligand (1.63 mg, 6 mol%), cyclohexyl vinyl boronic acid **26** (2.3 mg, 30 mol%), oxabicycle **16** (14.42mg, 2 equiv.), carbohydrate polyol **15a** (15.42mg, 0.05 mmol, 1 equiv.), $Rh(cod)_2OTf$ (1.17mg, 5 mol%) and then THF-d_8 (0.5 mL), triethyl amine base (14 µL, 2 equiv.) were added. Afterwards, the tube was sealed with cap and measured on the NMR spectrometer at 50 ˚C by recording ^{1}H spectra at different time.

General method for concentration dependence studies:

$Rh(cod)_2OTf$ catalyst concentration dependence: (See Supplementary Figure 8-11, Supplementary Table 35) **experiment 1 (5 mol**%): To a dry NMR tube charged with trimethoxy benzene (8.4 mg 1 equiv.), (*R, S*)-PPF-P^tBu_2 Josiphos ligand (1.63 mg, 6 mol%), cyclohexyl vinyl boronic acid **26** (2.3 mg, 30 mol%), oxabicycle **16** (14.42mg, 2 equiv.), carbohydrate polyol **15a** (15.42mg, 0.05 mmol, 1 equiv.), $Rh(cod)_2OTf$ (1.17mg, 5 mol%) and then THF-d_8 (0.5 mL), triethyl amine base (14 μL, 2 equiv.) were added. Afterwards, the tube was sealed with cap and measured on the NMR spectrometer at 50 ˚C by recording ^{1}H spectra at different time.

$Rh(cod)_2OTf$ catalyst concentration dependence: **experiment 2 (7 mol%):** To a dry NMR tube charged with trimethoxy benzene (8.4 mg 1 eq), (*R, S*)-PPF-P^tBu_2 Josiphos ligand (2.17 mg, 8 mol%), cyclohexyl vinyl boronic acid **26** (2.3 mg, 30 mol%), oxabicycle **16** (14.42mg, 2 equiv.), carbohydrate polyol **15a** (15.42mg, 0.05 mmol, 1 equiv.), $Rh(cod)_2OTf$ (1.64 mg, 5 mol%) and then THF-d_8 (0.5 mL), triethyl amine base (14 μL, 2 equiv.) were added. Afterwards, the tube was sealed with cap and measured on the NMR spectrometer at 50 ˚C by recording ^{1}H spectra at different time.

$Rh(cod)_2OTf$ catalyst concentration dependence: **experiment 3 (9 mol%):** To a dry NMR tube charged with trimethoxy benzene (8.4 mg 1 eq), (*R,S*)-PPF-P^tBu_2 Josiphos ligand (2.7 mg, 10.6 mol%), cyclohexyl vinyl boronic acid **26** (2.3 mg, 30 mol%), oxabicycle **16** (14.42mg, 2 equiv.), carbohydrate polyol **15a** (15.42mg, 0.05 mmol, 1 equiv.), $Rh(cod)_2OTf$ (2.1 mg, 5 mol%) and then THF-d_8 (0.5 mL), triethyl amine base (14 μL, 2 equiv.) were added. Afterwards, the tube was sealed with cap and measured on the NMR spectrometer at 50 ˚C by recording ^{1}H spectra at different time.

Boronic acid 26 catalyst concentration dependence: (See Supplementary Figures 12-14, Supplementary Table 36): **experiment 4 (20 mol%):** To a dry NMR tube charged with trimethoxy benzene (8.4 mg 1 equiv.), (*R,S*)-PPF-P^tBu_2 Josiphos ligand (1.63 mg, 6 mol%), cyclohexyl vinyl boronic acid **26** (1.54 mg, 30 mol%), oxabicycle **16** (14.42mg, 2 equiv.), carbohydrate polyol **15a** (15.42mg, 0.05 mmol, 1 equiv.), $Rh(cod)_2OTf$ (1.17mg, 5 mol%) and then THF-d_8 (0.5 mL), triethyl amine base (14 μL, 2 equiv.) were added. Afterwards, the tube was sealed with cap and measured on the NMR spectrometer at 50 ˚C by recording ^{1}H spectra at different times.

Boronic acid 26 catalyst concentration dependence: **experiment 5 (30 mol%):** To a dry NMR tube charged with trimethoxy benzene (8.4 mg 1 eq), (*R,S*)-PPF-P^tBu_2 Josiphos ligand (1.63 mg, 6 mol%), cyclohexyl vinyl boronic acid **26** (2.3 mg, 30 mol%), oxabicycle **16** (14.42mg, 2 equiv.), carbohydrate polyol **15a** (15.42mg, 0.05 mmol, 1 equiv.), $Rh(cod)_2OTf$ (1.17mg, 5 mol%) and then THF-d_8 (0.5 mL), triethyl amine base (14 μL, 2 equiv.) were added. Afterwards, the tube was sealed with cap and measured on the NMR spectrometer at 50 ˚C by recording ^{1}H spectra at different time.

Boronic acid 26 catalyst concentration dependence: **experiment 6 (35 mol%):** To a dry NMR tube charged with trimethoxy benzene (8.4 mg 1 equiv.), (*R,S*)-PPF-P^tBu_2 Josiphos ligand (1.63 mg, 6 mol%), cyclohexyl vinyl boronic acid **26** (2.7 mg, 30 mol%), oxabicycle **16** (14.42mg, 2 equiv.), carbohydrate polyol **15a** (15.42mg, 0.05 mmol, 1 equiv.), $Rh(cod)_2OTf$ (1.17mg, 5 mol%) and then THF-d_8 (0.5 mL), triethyl amine base (14 μL, 2 equiv.) were added. Afterwards, the tube was sealed with cap and measured on the NMR spectrometer at 50 ˚C by recording ^{1}H spectra at different time.

Carbohydrate polyol 15a concentration dependence (See Supplementary Figures 15-18, Supplementary Table 37): **experiment 7 (0.05M):** To a dry NMR tube charged with trimethoxy benzene (8.4 mg 1 equiv.), (*R,S*)-PPF-P^tBu_2 Josiphos ligand (1.63 mg, 6 mol%), cyclohexyl vinyl boronic acid **26** (2.3 mg, 30 mol%), oxabicycle **16** (14.42mg, 2 equiv.), carbohydrate polyol **15a** (7.71 mg, 0.05 mmol, 1 eq), $Rh(cod)_2OTf$ (1.17mg, 5 mol%) and then THF-d_8 (0.5 mL), triethyl amine base (14 μL, 2 equiv.) were added. Afterwards, the tube was sealed with cap and measured on the NMR spectrometer at 50 ˚C by recording ^{1}H spectra at different time.

Carbohydrate polyol 15a concentration dependence: **experiment 8 (0.1M):** To a dry NMR tube charged with trimethoxy benzene (8.4 mg 1 eq), (*R,S*)-PPF-P^tBu_2 Josiphos ligand (1.63 mg, 6 mol%), cyclohexyl vinyl boronic acid **26** (2.3 mg, 30 mol%), oxabicycle **16** (14.42mg, 2 equiv.), carbohydrate polyol **15a** (15.42 mg, 0.05 mmol, 1 equiv.), $Rh(cod)_2OTf$ (1.17mg, 5 mol%) and then THF-d_8 (0.5 mL),

triethyl amine base (14 μL, 2 equiv.) were added. Afterwards, the tube was sealed with cap and measured on the NMR spectrometer at 50 ˚C by recording ^{1}H spectra at different time.

Carbohydrate polyol 15a concentration dependence: **experiment 9 (0.15M):** To a dry NMR tube charged with trimethoxy benzene (8.4 mg 1 equiv.), (*R,S*)-PPF-P^{t}Bu$_2$ Josiphos ligand (1.63 mg, 6 mol%), cyclohexyl vinyl boronic acid **26** (2.3 mg, 30 mol%), oxabicycle **16** (14.42mg, 2 equiv.), carbohydrate polyol **15a** (23.13 mg, 0.05 mmol, 1 equiv.), Rh(cod)$_2$OTf (1.17mg, 5 mol%) and then THF-*d$_8$* (0.5 mL), triethyl amine base (14 μL, 2 equiv.) were added. Afterwards, the tube was sealed with cap and measured on the NMR spectrometer at 50 ˚C by recording ^{1}H spectra at different time.

Carbohydrate polyol 15a concentration dependence: **experiment 10 (0.2M):** To a dry NMR tube charged with trimethoxy benzene (8.4 mg 1 equiv.), (*R,S*)-PPF-P^{t}Bu$_2$ Josiphos ligand (1.63 mg, 6 mol%), cyclohexyl vinyl boronic acid **26** (2.3 mg, 30 mol%), oxabicycle **16** (14.42mg, 2 equiv.), carbohydrate polyol **15a** (30.84 mg, 0.05 mmol, 1 eq), Rh(cod)$_2$OTf (1.17mg, 5 mol%) and then THF-*d$_8$* (0.5 mL), triethyl amine base (14 μL, 2 equiv.) were added. Afterwards, the tube was sealed with cap and measured on the NMR spectrometer at 50 ˚C by recording ^{1}H spectra at different time.

Oxabicycle 16a concentration dependence (See Supplementary Figures 19-22, Supplementary Table 38): **experiment 11 (0.15M):** To a dry NMR tube charged with trimethoxy benzene (8.4 mg 1 equiv.), (*R, S*)-PPF-P ^{t}Bu$_2$ Josiphos ligand (1.63 mg, 6 mol%), cyclohexyl vinyl boronic acid **26** (2.3 mg, 30 mol%), oxabicycle **16** (10.81 mg, 2 equiv.), carbohydrate polyol **15a** (15.42mg, 0.05 mmol, 1 eq), Rh(cod)$_2$OTf (1.17mg, 5 mol%) and then THF-*d$_8$* (0.5 mL), triethyl amine base (14 μL, 2 equiv.) were added. Afterwards, the tube was sealed with cap and measured on the NMR spectrometer at 50 ˚C by recording ^{1}H spectra at different time.

Oxabicycle 16a concentration dependence: **experiment 12 (0.2M):** To a dry NMR tube charged with trimethoxy benzene (8.4 mg 1 equiv.), (*R,S*)-PPF-P ^{t}Bu$_2$ Josiphos ligand (1.63 mg, 6 mol%), cyclohexyl vinyl boronic acid **26** (2.3 mg, 30 mol%), oxabicycle **16** (14.41 mg, 2 equiv.), carbohydrate polyol **15a** (15.42mg, 0.05 mmol, 1 equiv.), Rh(cod)$_2$OTf (1.17mg, 5 mol%) and then THF-*d$_8$* (0.5 mL), triethyl amine base (14 μL, 2 equiv.) were added. Afterwards, the tube was sealed with cap and measured on the NMR spectrometer at 50 ˚C by recording ^{1}H spectra at different time.

Oxabicycle 16a concentration dependence: **experiment 13 (0.25M):** To a dry NMR tube charged with trimethoxy benzene (8.4 mg 1 equiv.), (*R,S*)-PPF-P^{t}Bu$_2$ Josiphos ligand (1.63 mg, 6 mol%), cyclohexyl vinyl boronic acid **26** (2.3 mg, 30 mol%), oxabicycle **16** (18.02 mg, 2 equiv.), carbohydrate polyol **15a** (15.42mg, 0.05 mmol, 1 equiv.), Rh(cod)$_2$OTf (1.17mg, 5 mol%) and then THF-*d$_8$* (0.5 mL), triethyl amine base (14 μL, 2 equiv.) were added. Afterwards, the tube was sealed with cap and measured on the NMR spectrometer at 50 ˚C by recording ^{1}H spectra at different time.

Oxabicycle 16a concentration dependence: **experiment 14 (0.3M):** To a dry NMR tube charged with trimethoxy benzene (8.4 mg 1 equiv.), (*R, S*)-PPF-P^{t}Bu$_2$ Josiphos ligand (1.63 mg, 6 mol%), cyclohexyl vinyl boronic acid **26** (2.3 mg, 30 mol%), oxabicycle **16** (21.63 mg, 2 equiv.), carbohydrate polyol **15a** (15.42mg, 0.05 mmol, 1 equiv.), Rh(cod)$_2$OTf (1.17mg, 5 mol%) and then THF-*d$_8$* (0.5 mL), triethyl amine base (14 μL, 2 equiv.) were added. Afterwards, the tube was sealed with cap and measured on the NMR spectrometer at 50 ˚C by recording ^{1}H spectra at different time.

Triethylamine concentration dependence (See Supplementary Figures 23-26, Supplementary Table 39): **experiment 15 (0.15M):** To a dry NMR tube charged with trimethoxy benzene (8.4 mg 1 eq), (*R,S*)-PPF-P^{t}Bu$_2$ Josiphos ligand (1.63 mg, 6 mol%), cyclohexyl vinyl boronic acid **26** (2.3 mg, 30 mol%), oxabicycle **16** (14.42mg, 2 equiv.), carbohydrate polyol **15a** (15.42mg, 0.05 mmol, 1 eq), Rh(cod)$_2$OTf (1.17mg, 5 mol%) and then THF-*d$_8$* (0.5 mL), triethyl amine base (10.4 μL, 1.5 equiv.) were added. Afterwards, the tube was sealed with cap and measured on the NMR spectrometer at 50 ˚C by recording ^{1}H spectra at different time.

Triethylamine concentration dependence: **experiment 16 (0.2M):** To a dry NMR tube charged with trimethoxy benzene (8.4 mg 1 equiv.), (*R,S*)-PPF-P^{t}Bu$_2$ Josiphos ligand (1.63 mg, 6 mol%), cyclohexyl vinyl boronic acid **26** (2.3 mg, 30 mol%), oxabicycle **16** (14.42mg, 2 equiv.), carbohydrate polyol **15a** (15.42mg, 0.05 mmol, 1 equiv.), Rh(cod)$_2$OTf (1.17mg, 5 mol%) and then THF-*d$_8$* (0.5 mL), triethyl

amine base (14 μL, 2 equiv.) were added. Afterwards, the tube was sealed with cap and measured on the NMR spectrometer at 50 ˚C by recording ^{1}H spectra at different time.

Triethylamine concentration dependence: **experiment 17 (0.25M):** To a dry NMR tube charged with trimethoxy benzene (8.4 mg 1 equiv.), (*R,S*)-PPF-P^{t}Bu$_2$ Josiphos ligand (1.63 mg, 6 mol%), cyclohexyl vinyl boronic acid **26** (2.3 mg, 30 mol%), oxabicycle **16** (14.42mg, 2 equiv.), carbohydrate polyol **15a** (15.42mg, 0.05 mmol, 1 equiv.), Rh(cod)$_2$OTf (1.17mg, 5 mol%) and then THF-d_8 (0.5 mL), triethyl amine base (17.4 μL, 2.5 equiv.) were added. Afterwards, the tube was sealed with cap and measured on the NMR spectrometer at 50 ˚C by recording ^{1}H spectra at different time.

Triethylamine concentration dependence: **experiment 18 (0.3M):** To a dry NMR tube charged with trimethoxy benzene (8.4 mg 1 equiv.), (*R,S*)-PPF-P^{t}Bu$_2$ Josiphos ligand (1.63 mg, 6 mol%), cyclohexyl vinyl boronic acid **26** (2.3 mg, 30 mol%), oxabicycle **16** (14.42mg, 2 equiv.), carbohydrate polyol **15a** (15.42mg, 0.05 mmol, 1 equiv.), Rh(cod)$_2$OTf (1.17mg, 5 mol%) and then THF-d_8 (0.5 mL), triethyl amine base (21 μL, 1.5 equiv.) were added. Afterwards, the tube was sealed with cap and measured on the NMR spectrometer at 50 ˚C by recording ^{1}H spectra at different time.

Procedure for initial optimization: To an oven dried dram vial purged with an argon balloon was charged with ligand (6 mol%), boronic acid (30 mol%), oxabicycle **16** (2 equiv.), carbohydrate polyol **15** (0.1 mmol, 1 equiv.), metal catalyst (5 mol%) and then dry THF (1 mL), triethyl amine base (2 equiv.) were added. The dram vial was sealed and the mixture was immersed in a 50 °C oil bath stirred for 24-48 h. Upon completion of the reaction, the reaction mixture was filtered over a short silica plug and flushed with 250-300 mL of ethyl acetate. The filtrate was then evaporated and the determination of the regiomeric ratio (r.r.) is by ^{1}H-NMR analysis of this concentrated crude mixture with 1,3,5-trimethoxybenzene as the internal standard.

Methods for control experiments

General method for control experiment 1: To an oven dried dram vial purged with an argon balloon was charged with (*R,S*)-PPF-P^{t}Bu$_2$ Josiphos ligand (6.51 mg, 0.012 mmol, 6 mol%), oxabicycle **16a** (0.4 mmol, 2 equiv.), carbohydrate polyol **15a** (0.2 mmol, 1 equiv.), Rh(cod)$_2$OTf (4.68 mg, 0.01 mmol, 5 mol%) and then dry THF (2 mL), triethyl amine base (0.4 mmol, 2 equiv.) were added. The dram vial was sealed and the mixture was immersed in a 50 °C oil bath stirred for 24 h. Upon completion of the reaction, the reaction mixture was filtered over a short silica plug and flushed with 250-300 mL of ethyl acetate. The filtrate was then evaporated and the determination of the regiomeric ratio (r.r.) is by ^{1}H-NMR analysis of this concentrated crude mixture with 1,3,5-trimethoxybenzene as the internal standard.

General method for control experiment 2: To an oven dried dram vial purged with an argon balloon was charged with cyclohexyl vinyl boronic acid **26** (9.24 mg, 0.06 mmol, 30 mol%), oxabicycle **16a** (0.4 mmol, 2 equiv.), carbohydrate polyol **15a** (0.2 mmol, 1 equiv.), and then dry THF (2 mL), triethyl amine base (0.4 mmol, 2 equiv.) were added. The dram vial was sealed and the mixture was immersed in a 50 °C oil bath stirred for 24 h. Upon completion of the reaction, the reaction mixture was filtered over a short silica plug and flushed with 250-300 mL of ethyl acetate. The filtrate was then evaporated and the determination of the regiomeric ratio (r.r.) is by ^{1}H-NMR analysis of this concentrated crude mixture with 1,3,5-trimethoxybenzene as the internal standard.

General method for control experiment 3: To an oven dried dram vial purged with an argon balloon was charged with (*R,S*)-PPF-P^{t}Bu$_2$ Josiphos ligand (6.51 mg, 0.012 mmol, 6 mol%), cyclohexyl vinyl boronic acid **26** (9.24 mg, 0.06 mmol, 30 mol%), oxabicycle **16a** (0.4 mmol, 2 equiv.), 100mg 4Å molecular sieves, carbohydrate polyol **15a** (0.2 mmol, 1 equiv.), Rh(cod)$_2$OTf (4.68 mg, 0.01 mmol, 5 mol%) and then dry THF (2 mL), triethyl amine base (0.4 mmol, 2 equiv.) were added. The dram vial was sealed and the mixture was immersed in a 50 °C oil bath stirred for 24 h. Upon completion of the reaction, the reaction mixture was filtered over a short silica plug and flushed with 250-300 mL of ethyl acetate. The filtrate was then evaporated and the determination of the regiomeric ratio (r.r.) is by ^{1}H-NMR analysis of this concentrated crude mixture with 1,3,5-trimethoxybenzene as the internal standard.

General method for control experiment 4: To an oven dried dram vial purged with an argon balloon was charged with (*R,S*)-PPF-P^{t}Bu$_2$ Josiphos ligand (6.51 mg, 0.012 mmol, 6 mol%), cyclohexyl vinyl boronic acid **26** (9.24 mg, 0.06 mmol, 30 mol%), oxabicycle **16a** (0.4 mmol, 2 equiv.), carbohydrate

polyol **15a** (0.2 mmol, 1 equiv.), Rh(cod)$_2$OTf (4.68 mg, 0.01 mmol, 5 mol%) and then dry THF (2 mL), H_2O (5.5 eq,10 µL), triethyl amine base (0.4 mmol, 2 equiv.) were added. The dram vial was sealed and the mixture was immersed in a 50 °C oil bath stirred for 24 h. Upon completion of the reaction, the reaction mixture was filtered over a short silica plug and flushed with 250-300 mL of ethyl acetate. The filtrate was then evaporated and the determination of the regiomeric ratio (r.r.) is by ^{1}H-NMR analysis of this concentrated crude mixture with 1,3,5-trimethoxybenzene as the internal standard.

General method for control experiment 5: To an oven dried dram vial purged with an argon balloon was charged with dppf ligand (6.6 mg, 0.012 mmol, 6 mol%), cyclohexyl vinyl boronic acid **26** (9.24 mg, 0.06 mmol, 30 mol%), oxabicycle **16a** (0.4 mmol, 2 equiv.), carbohydrate polyol **15a** (0.2 mmol, 1 equiv.), Rh(cod)$_2$OTf (4.68 mg, 0.01 mmol, 5 mol%) and then dry THF (2 mL), triethyl amine base (0.4 mmol, 2 equiv.) were added. The dram vial was sealed and the mixture was immersed in a 50 °C oil bath stirred for 24 h. Upon completion of the reaction, the reaction mixture was filtered over a short silica plug and flushed with 250-300 mL of ethyl acetate. The filtrate was then evaporated and the determination of the regiomeric ratio (r.r.) is by ^{1}H-NMR analysis of this concentrated crude mixture with 1,3,5-trimethoxybenzene as the internal standard.

General method for control experiment 6: To an oven dried dram vial purged with an argon balloon was charged with (*R,S*)-PPF-P^{t}Bu$_2$ Josiphos ligand (4.4 mg, 0.0078 mmol, 6 mol%), borinic acid **27** (7.9 mg, 0.039 mmol, 30 mol%), oxabicycle **16a** (38.9 mg, 0.26 mmol, 2 equiv.), carbohydrate polyol **17n** (0.13 mmol, 1 equiv.), Rh(cod)$_2$OTf (3.1 mg, 0.0065 mmol, 5 mol%) and then dry THF (1.3 mL), triethyl amine base (13 μL, 0.26 mmol, 2 equiv.) were added. The dram vial was sealed and the mixture was immersed in a 50 °C oil bath stirred for 24 h. Upon completion of the reaction, the reaction mixture was filtered over a short silica plug and flushed with 250-300 mL of ethyl acetate. The filtrate was then evaporated and the determination of the regiomeric ratio (r.r.) is by ^{1}H-NMR analysis of this concentrated crude mixture with 1,3,5-trimethoxybenzene as the internal standard.

Synthesis of starting materials: The carbohydrate polyols (**15a-15p**) and oxabicyclic alkenes (**16a-16i)**[1-7] were prepared according to the previous published protocols, **15q** was purchased from Sigma-Aldrich (Product number A7429) and used directly. The allylic carbonate (**16j-16m**),[8-11] diols (**15s and 15t)** ,[12] methyl α-D-lyxopyranoside(**15v**)[13] were prepared according to the previous published protocols.

TBS protection: Procedure I: To a stirred solution of carbohydrate polyol (1 equiv.) in 8ml of dry pyridine was slowly added *tert*-butyldimethylsilyl chloride (1 equiv. or 1.2 equiv.) and the mixture was kept for overnight at room temperature. After removal of the solvent under reduced pressure, the residue was taken up in EtOAc and extracted with 0.5 M HCl, saturated aqueous $NaHCO_3$ and brine. The aqueous layers were re-extracted twice with EtOAc and the combined organic layers were dried over Na_2SO_4. After evaporation of the solvent and drying of the product in vacuo, the residue was purified by flash column chromatography (cyclohexane/EtOAc) yielding the silylether as a white solid. Substrates **15a**, **15c-15j**, **15m** (liquid) and **15r** were synthesized according to the previous published protocols.[14-25]

Bn protection: Procedure II: TFA (0.743 ml, 10 mmol), was added dropwise to a stirred solution of the benzylidene **15b** (564 mg, 2 mmol) and triethylsilane (1.593 ml, 10 mmol) in DCM (10 ml), at 0°C under an atmosphere of argon. After 45 min, TLC (hexane:ethyl acetate, 1:5) indicated complete conversion of the starting material (Rf =0.5) to a major product (Rf =0.1). The reaction mixture was diluted with ethyl acetate (50 ml), washed with saturated aqueous sodium bicarbonate solution (50 ml) and brine (50 ml). The organic phase was dried (Na_2SO_4), filtered and concentrated in vacuo. The residue was purified by flash column chromatography (hexane:ethyl acetate, 1:5-1:7) to give the triol **15f** (240 mg, 42%) as a colorless oil.

Propyl ketone protection: Procedure III: Add *p*-TsOH·H_2O (100 mg, 0.53 mmol) to a solution of the methyl α-D-mannopyranoside(1.0 g, 5.15 mmol) in anhydrous acetonitrile (100 mL) and 2,2-dimethoxypropane (0.59 g, 5.66 mmol) at room temperature, continue stirring for 5 h, quench the reaction with TEA, then concentrate the mixture and purify the mixture by silica gen column chromatography (petroleum ether/ethyl acetate, 1:1) to to obtain the substrate **15g** (400 mg, 33%) as a grey solid.

Disilicon protection: Procedure IV: A mixture of **15p** (380.388 mg, 2 mmol) and imidazole (299.54 mg, 4.4 mmol) were dissolved in anhydrous DMF (15 mL) under an argon atmosphere and the solution was cooled to 0 °C. and 1,3-dichloro-1,1,3,3-tetraisopropyldisiloxane (946.28 mg, 3 mmol) was added dropwise at this temperature. Afterwards, the solution was quenched with water, and extracted with DCM (20 mL) twice, the organic layer was washed with brine (20 mL), dried over Na_2SO_4, filtered and concentrated in vacuo. Following by column chromatography (PE/EA=20/1), the title compound was obtained **15u'** (677 mg, 78%) as a colorless oil.

Allyl deprotection: Procedure V: A mixture of **15u'** (216.35 mg, 0.5 mmol), $PdCl_2$ (177.325 mg, 1 mmol), and NaOAc (328.135 mg, 4 mmol) in AcOH-H_2O (9:1, v/v, 5 mL) was stirred 2 h at 30 °C. Then, the insoluble material was filtered off. The filtrate was concentrated in vacuo to give a residue which was extracted with EtOAc (20 mL × 2). The combined organic extracts were washed with water (10 mL × 2), saturated aqueous $NaHCO_3$ (15 mL × 2), and brine (15 mL), dried over anhydrous Na_2SO_4, and concentrated. The resulting residue was purified by column chromatography (5:1, petroleum ether-EtOAc) to afford the desired lactol **15u** (98 mg, 50%) as a colorless oil.

Polyol substrates for site selective functionalization

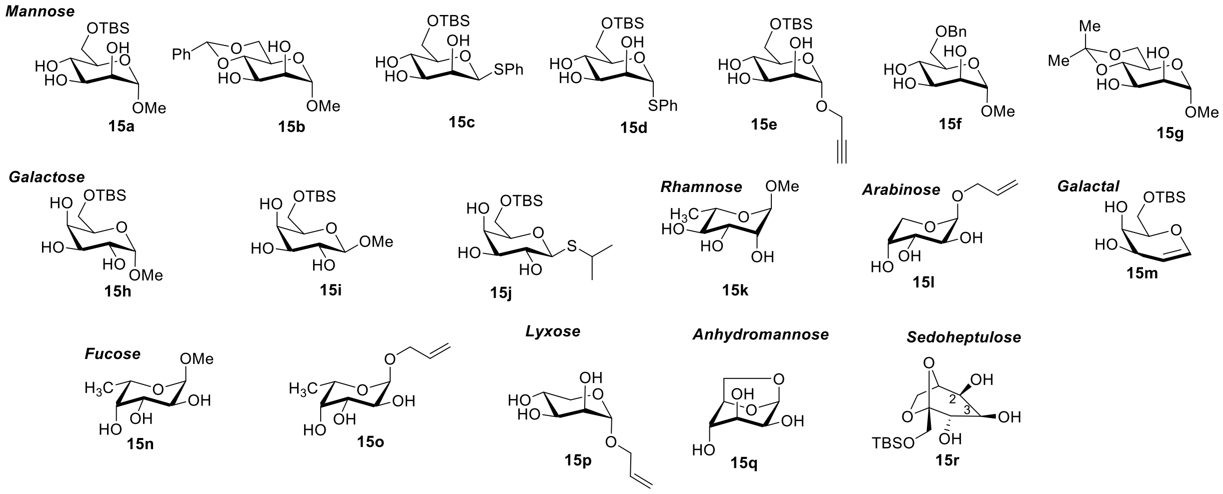

Substrates for anomeric functionalization

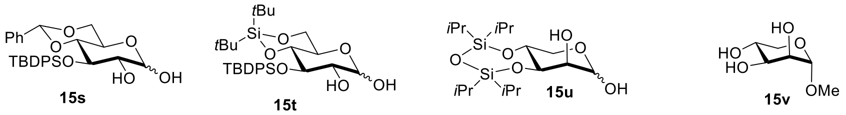

Electrophilic substrates for Rh(I)/organoboron catalyzed synergistic reaction:

Meso-oxanorbornadienes

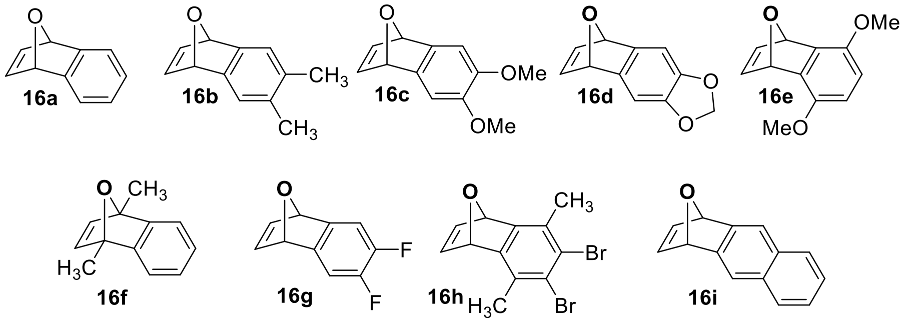

Racemic allylic carbonates

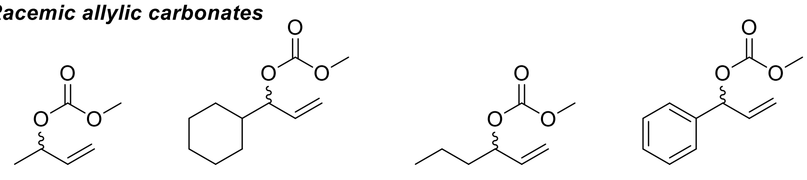

16j **16k** **16l** **16m**

(2*R*,3*S*,4*S*,5*S*,6*S*)-2-(((*tert*-butyldimethylsilyl)oxy)methyl)-6-methoxytetrahydro-2H-pyran-3,4,5-triol (15a)

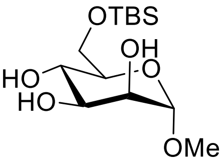

15a

The title product compound was prepared according to the general procedure **I**. **^{1}H NMR** (400 MHz, $CDCl_3$) δ 4.69 (d, *J* = 1.5 Hz, 1H), 3.89 (dd, *J* = 3.3, 1.6 Hz, 1H), 3.87 (d, *J* = 5.5 Hz, 2H), 3.81 – 3.72 (m, 2H), 3.59-3.53 (m, 1H), 3.38 (bs, 2H), 3.36 (s, 3H), 0.90 (s, 9H), 0.10 (d, *J* = 1.3 Hz, 5H).
The analytical data are in accordance with the reported literature.[14-16]

(2*R,*4a*R*,6*S*,7*S*,8*R*,8a*S*)-6-methoxy-2-phenylhexahydropyrano[3,2-d][1,3]dioxine-7,8-diol (15b)

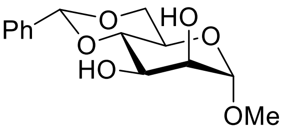

15b

The title product compound was prepared according to the general procedure **I**. **^{1}H NMR** (400 MHz, $CDCl_3$) δ 7.50 – 7.47 (m, 2H), 7.40 – 7.35 (m, 3H), 5.55 (s, 1H), 4.72 (d, *J* = 1.3 Hz, 1H), 4.29 – 4.26 (m, 1H), 4.05 – 3.98 (m, 2H), 3.92 – 3.87 (m, 1H), 3.85 – 3.76 (m, 2H), 3.38 (s, 3H), 2.82 (dd, *J* = 15.0, 2.4 Hz, 2H).
The analytical data are in accordance with the reported literature.[23-25]

(2*R*,3*S*,4*S*,5*S*,6*S*)-2-(((*tert*-butyldimethylsilyl)oxy)methyl)-6-(phenylthio)tetrahydro-2H-pyran-3,4,5-triol (15c)

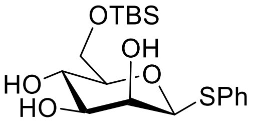

15c

The title product compound was prepared according to the general procedure **I.** **^{1}H NMR** (400 MHz, $CDCl_3$) δ 7.52 – 7.49 (m, 2H), 7.33 – 7.27 (m, 3H), 4.88 (s, 1H), 4.18 (t, *J* = 4.1 Hz, 1H), 3.93 (qd, *J* = 10.4, 5.8 Hz, 2H), 3.82 (td, *J* = 9.2, 1.4 Hz, 1H), 3.63-3.58 (m, 1H), 3.39-3.34 (m, 1H), 3.30 (d, *J* = 1.6 Hz, 1H), 2.65 (d, *J* = 6.0 Hz, 1H), 2.47 (d, *J* = 4.7 Hz, 1H), 0.91 (s, 9H), 0.11 (d, *J* = 7.2 Hz, 6H).
The analytical data are in accordance with the reported literature.[23-25]

(2*R*,3*S*,4*S*,5*S*,6*R*)-2-(((*tert*-butyldimethylsilyl)oxy)methyl)-6-(phenylthio)tetrahydro-2H-pyran-3,4,5-triol (15d)

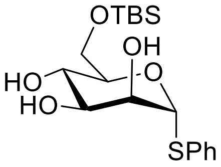

15d

The title product compound was prepared according to the general procedure **I.** **^{1}H NMR** (400 MHz, $CDCl_3$) δ 7.48 – 7.45 (m, 2H), 7.32 – 7.23 (m, 3H), 5.53 (d, *J* = 1.3 Hz, 1H), 4.21 – 4.19 (m, 1H), 4.15-4.10 (m, 1H), 3.89– 3.83 (m, 4H), 3.52 (d, *J* = 1.4 Hz, 1H), 3.14 (d, *J* = 4.4 Hz, 1H), 2.91 (d, *J* = 4.1 Hz, 1H), 0.90 (s, 9H), 0.09 (d, *J* = 1.3 Hz, 6H).

The analytical data are in accordance with the reported literature.[23-25]

(2*R*,3*S*,4*S*,5*S*,6*S*)-2-(((*tert*-butyldimethylsilyl)oxy)methyl)-6-(prop-2-yn-1-yloxy)tetrahydro-2H-pyran-3,4,5-triol

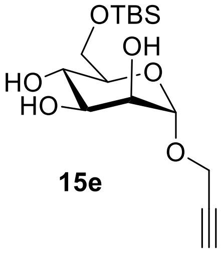

15e

The title product compound was prepared according to the general procedure **I.** **^{1}H NMR** (400 MHz, CD_3OD) δ 4.99 (d, *J* = 1.6 Hz, 1H), 4.30 – 4.29 (m, 2H), 4.02 (dd, *J* = 11.1, 2.0 Hz, 1H), 3.84 – 3.79 (m, 2H), 3.70 (dd, *J* = 9.1, 3.4 Hz, 1H), 3.60 (t, *J* = 9.5 Hz, 1H), 3.55 – 3.51 (m, 1H), 2.88 (t, *J* = 2.3 Hz, 1H), 0.96 (s, 9H), 0.14 (s, 6H).

The analytical data are in accordance with the reported literature.[20-22]

(*2R,3S,4S,5S,6S*)-2-((benzyloxy)methyl)-6-methoxytetrahydro-2H-pyran-3,4,5-triol(15f)

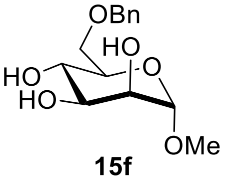

15f

The title product compound is prepared according to the general procedure **II**. **^{1}H NMR** (600 MHz, $CDCl_3$) δ 7.38 – 7.33 (m, 4H), 7.32 – 7.27 (m, 1H), 4.73 (s, 1H), 4.66 – 4.54 (m, 2H), 3.90 (s, 1H), 3.83 – 3.76 (m, 3H), 3.76 – 3.66 (m, 2H), 3.37 (s, 3H), 2.89 (s, 1H), 2.73 (s, 1H), 2.50 (s, 1H). **^{13}C NMR** (151 MHz, $CDCl_3$) δ 137.82, 128.67, 128.06, 127.94, 100.89, 73.92, 71.81, 70.67, 70.50, 70.05, 69.79, 55.24. **ESI-HRMS**: Calculated for $C_{14}H_{20}O_6Na$ $(M+Na)^+$: 307.11521, Found: 307.11510. $[\alpha]_D^{20}$ =+53.4 (c =0.45, $CHCl_3$).

(*4aR,6S,7S,8R,8aS*)-6-methoxy-2,2-dimethylhexahydropyrano[3,2-d][1,3]dioxine-7,8-diol(15g)

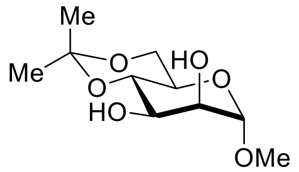

15g

The title product compound is prepared according to the general procedure **III**. **^{1}H NMR** (500 MHz, $CDCl_3$) δ 4.91 (s, 1H), 4.18 – 4.11 (m, 2H), 3.91 – 3.83 (m, 2H), 3.77 – 3.71 (m, 1H), 3.66 – 3.58 (m, 1H), 3.40 (s, 3H), 2.67 (d, J = 4.5 Hz, 1H), 2.21 – 2.09 (m, 1H), 1.53 (s, 3H), 1.36 (s, 3H). **^{13}C NMR** (126 MHz, $CDCl_3$) δ 109.82, 98.57, 78.30, 75.55, 69.83, 69.70, 62.71, 55.29, 28.05, 26.23. **ESI-HRMS**: Calculated for $C_{11}H_{21}O_5Na$ $(M+Na)^+$: 257.09956, Found: 257.09939. $[a]_D^{20}$ =+37.3 (c =0.40, $CHCl_3$).

(2*R*,3*R*,4*S*,5*R*,6*S*)-2-(((*tert*-butyldimethylsilyl)oxy)methyl)-6-methoxytetrahydro-2H-pyran-3,4,5-triol (15h)

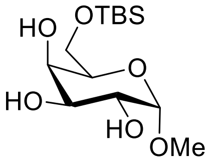

15h

The title product compound was prepared according to the general procedure **I**. **^{1}H NMR** (500 MHz, $CDCl_3$) δ 4.82 (d, J = 3.9 Hz, 1H), 4.10 (dd, J = 2.4, 0.8 Hz, 1H), 3.93 – 3.83 (m, 3H), 3.75 – 3.71 (m, 2H), 3.42 (s, 3H), 0.90 (s, 9H), 0.10 (d, J = 0.6 Hz, 6H).

The analytical data are in accordance with the reported literature.[14-19]

(2*R*,3*R*,4*S*,5*R*,6*R*)-2-(((*tert*-butyldimethylsilyl)oxy)methyl)-6-methoxytetrahydro-2H-pyran-3,4,5-triol (15i)

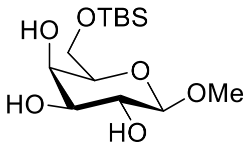

15i

The title product compound was prepared according to the general procedure **I**. **^{1}H NMR** (500 MHz, $CDCl_3$) δ 4.16 (d, J = 7.6 Hz, 1H), 4.04 (dd, J = 3.4, 1.1 Hz, 1H), 3.95 – 3.86 (m, 2H), 3.65 (dd, J = 9.5, 7.6 Hz, 1H), 3.59-3.56 (m, 1H), 3.54 (s, 3H), 3.49 (ddd, J = 6.1, 5.0, 1.2 Hz, 1H), 2.96 (s, OH, 0.36H), 2.88 (s, OH, 0.37H), 0.90 (s, 9H), 0.09 (d, J = 2.0 Hz, 6H).

The analytical data are in accordance with the reported literature.[14-16,18]

(*2R*,*3R*,*4S*,*5R*,*6S*)-2-(((*tert*-butyldimethylsilyl)oxy)methyl)-6-(isopropylthio)tetrahydro-2H-pyran-3,4,5-triol (15j)

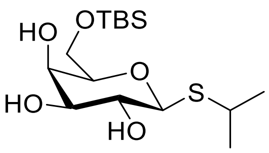

15j

The title product compound is prepared according to the general procedure **I**. **^{1}H NMR** (500 MHz, $CDCl_3$) δ 4.38 (d, *J* = 9.7 Hz, 1H), 4.04 (t, *J* = 3.7 Hz, 1H), 4.02 – 4.00 (m, 1H), 3.82 (m, 2H), 3.68 (td, *J* = 9.4, 2.9 Hz, 1H), 3.58 (m, 1H), 3.54 (bs, 1H), 3.49 (t, *J* = 5.7 Hz, 1H), 3.43 (d, *J* = 4.2 Hz, 1H), 3.21 (hept, *J* = 6.8 Hz, 1H), 1.31 (dd, *J* = 6.8, 3.6 Hz, 6H), 0.87 (s, 9H), 0.06 (d, *J* = 3.7 Hz, 6H). **^{13}C NMR** (126 MHz, Chloroform-*d*) δ 85.77, 78.66, 75.13, 70.61, 69.25, 62.79, 35.49, 25.95, 24.30, 24.11, 18.35, -5.28, -5.31. **ESI-HRMS**: Calculated for $C_{15}H_{33}O_5SSi$ $(M+H)^+$: 353.18125, Found: 353.18070. $[\alpha]_D^{20}$ =-30.5 (c =0.65 , $CHCl_3$).

(2*R*,3*R*,4*R*,5*R*,6*S*)-2-methoxy-6-methyltetrahydro-2H-pyran-3,4,5-triol (15k)

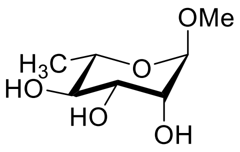

15k

The title product compound was prepared according to the general procedure **I**. **^{1}H NMR** (400 MHz, CD_3OD) δ 4.59 (d, *J* = 1.5 Hz, 1H), 3.82 (dd, *J* = 3.4, 1.7 Hz, 1H), 3.64 (dd, *J* = 9.5, 3.4 Hz, 1H), 3.61-3.54 (m, 1H), 3.42-3.39 (m, 1H), 3.38 (s, 3H), 1.31 (d, *J* = 6.2 Hz, 3H).
The analytical data are in accordance with the reported literature.[16]

(2*R*,3*S*,4*R*,5*R*)-2-(allyloxy)tetrahydro-2H-pyran-3,4,5-triol (15l)

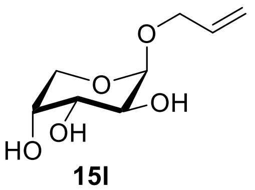

15l

The title product compound was prepared according to the general procedure **I**. **^{1}H NMR** (400 MHz, CD_3OD) δ 6.06-5.96 (m, 1H), 5.37 (dq, *J* = 17.3, 1.7 Hz, 1H), 5.22 (dq, *J* = 10.4, 1.3 Hz, 1H), 4.89 – 4.88 (m, 1H), 4.23 (ddt, *J* = 13.0, 5.2, 1.5 Hz, 1H), 4.07 (ddt, *J* = 13.0, 6.0, 1.4 Hz, 1H), 3.91-3.90 (m, 1H), 3.87 (dd, *J* = 12.3, 1.6 Hz, 1H), 3.83 – 3.82 (m, 2H), 3.62 (dd, *J* = 12.2, 2.3 Hz, 1H), 3.40 (bs, (OH) 0.15H).

The analytical data are in accordance with the reported literature.[14-16, 19]

(2*R*,3*R*)-2-(((*tert*-butyldimethylsilyl)oxy)methyl)-3,4-dihydro-2H-pyran-3,4-diol (15m)

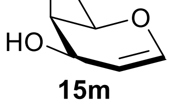

15m

The title product compound was prepared according to the general procedure **I**. **^{1}H NMR** (400 MHz, $CDCl_3$) δ 6.38 (dd, *J* = 6.2, 1.3 Hz, 1H), 4.73 – 4.70 (m, 1H), 4.32– 4.31 (m, 1H), 4.10 (d, *J* = 4.5 Hz, 1H), 3.99– 3.90 (m, 2H), 3.89 – 3.86 (m, 1H), 0.91 (d, *J* = 0.4 Hz, 9H), 0.11(d, *J* = 0.27 Hz, 6H).

The analytical data are in accordance with the reported literature.[21]

(2*R*,3*S*,4*R*,5*S*,6*S*)-2-methoxy-6-methyltetrahydro-2H-pyran-3,4,5-triol (15n)

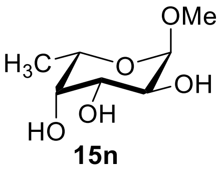

15n

The title product compound was prepared according to the general procedure **I.** **^{1}H NMR** (400 MHz, CD_3OD) δ 4.69 (d, *J* = 3.2 Hz, 1H), 3.98 – 3.93 (m, 1H), 3.80 – 3.74 (m, 2H), 3.70 (dd, *J* = 2.5, 1.2 Hz, 1H), 3.54 (s, OH, 0.16 H), 3.42 (s, 3H), 1.27 (d, *J* = 6.6 Hz, 3H).

The analytical data are in accordance with the reported literature.[22]

(2*R*,3*S*,4*R*,5*S*,6*S*)-2-(allyloxy)-6-methyltetrahydro-2H-pyran-3,4,5-triol (15o)

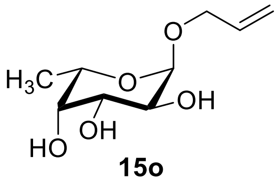

15o

The title product compound was prepared according to the general procedure **I.** **^{1}H NMR** (500 MHz, CD_3OD) δ 5.98-5.90 (m, 1H), 5.30 (dq, *J* = 17.3, 1.7 Hz, 1H), 5.15 (dq, *J* = 10.4, 1.3 Hz, 1H), 4.78 (d, *J* = 3.0 Hz, 1H), 4.15 (ddt, *J* = 13.1, 5.3, 1.5 Hz, 1H), 4.00 (ddt, *J* = 13.1, 6.0, 1.4 Hz, 1H), 3.94 (q, *J* = 6.8 Hz, 1H), 3.75-3.70 (m, 2H), 3.64– 3.64 (m, 1H), 1.19 (d, *J* = 6.6 Hz, 3H).

The analytical data are in accordance with the reported literature.[24]

(2*S*,3*S*,4*S*,5*R*)-2-(allyloxy)tetrahydro-2H-pyran-3,4,5-triol (15p)

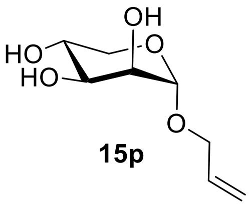

15p

The title product compound was prepared according to the general procedure **I.** **^{1}H NMR** (400 MHz, CD_3OD) δ 6.04 – 5.93 (m, 1H), 5.34 (dt, *J* = 17.3, 1.7 Hz, 1H), 5.22 (dt, *J* = 10.5, 1.6 Hz, 1H), 4.76 (d, *J* = 2.2 Hz, 1H), 4.26-4.21 (m, 1H), 4.05 (ddd, *J* = 13.1, 5.9, 1.5 Hz, 1H), 3.88 – 3.82 (m, 2H), 3.75 – 3.68 (m, 2H), 3.53-3.48 (m, 1H).

The analytical data are in accordance with the reported literature.[25]

(*1R*,*2S*,*3R*,*4S*,*5R*)-5-(((*tert*-butyldimethylsilyl)oxy)methyl)-6,8-dioxabicyclo[3.2.1]octane-2,3,4-triol (15p)

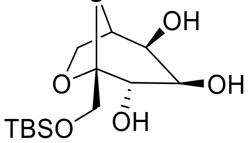

15r

The title product compound is prepared according to the general procedure **I.** **^{1}H NMR** (500 MHz, CD_3OD) δ 4.54 – 4.53 (m, 1H), 3.96 (d, *J* = 11.2 Hz, 1H), 3.81 (dd, *J* = 4.4, 2.4 Hz, 1H), 3.77 – 3.73 (m, 2H), 3.72 – 3.67 (m, 2H), 3.63 (dd, *J* = 8.6, 4.6 Hz, 1H), 0.91 (s, 9H), 0.09 (d, *J* = 1.4 Hz, 6H). **^{13}C NMR** (126 MHz, Methanol-*d_4*) δ 109.20, 79.30, 73.45, 72.42, 72.02, 67.26, 63.66, 26.42, 19.34, -5.21. **ESI-**

HRMS: Calculated for $C_{13}H_{27}O_6Si$ $(M+Na)^+$: 307.15714, Found: 307.15730. $[\alpha]_D^{20}$ =-87.0 (c =1.25 , $CHCl_3$).

(2*R*,4a*R*,7*R*,8*R*,8a*R*)-8-((*tert*-butyldiphenylsilyl)oxy)-2-phenylhexahydropyrano[3,2-d][1,3]dioxine-6,7-diol(15s)

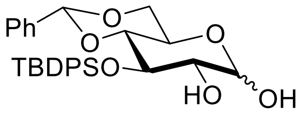

15s

^{1}H NMR (400 MHz, $CDCl_3$, 50:50 mixture of anomers) 7.65 – 7.61 (m, 4.0H), 7.41 – 7.35 (m, 2.0H), 7.33 – 7.21 (m, 7.0H), 7.12 – 7.05 (m, 2.0H), 5.25 (s, 0.5H), 5.24 – 5.22 (m, 1.0H), 4.60 (dd, J = 8.0, 6.4 Hz, 0.5H), 4.24 (dd, J = 10.4, 4.8 Hz, 0.5H), 4.19 (dd, J = 10.4, 5.2 Hz, 0.5H), 4.04 (t, J = 8.8 Hz, 0.5H), 3.89 (td, J = 10.0, 4.8 Hz, 0.5H), 3.84 (t, J = 8.4 Hz, 0.5H), 3.77 – 3.72 (m, 0.5H), 3.67 – 3.51 (m, 2.5H),3.31 (td, J = 9.6, 4.8 Hz, 0.5H), 3.18 (d, J = 5.2 Hz, 0.5H), 2.81 – 2.77 (m, 0.5H), 2.23 (d, J = 2.8 Hz, 0.5H), 2.01 (d, J = 6.4 Hz, 0.5H), 1.02 (s, 4.5H), 1.01 (s, 4.5H); The analytical data are in accordance with the reported literature.[12]

(4a*R*,7*R*,8*R*,8a*R*)-2,2-di-*tert*-butyl-8-((*tert*-butyldiphenylsilyl)oxy)hexahydropyrano[3,2-d][1,3,2]dioxasiline-6,7-diol(15t)

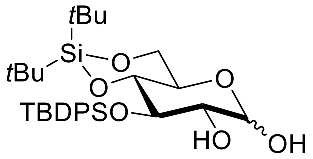

15t

^{1}H NMR (400 MHz, $CDCl_3$, 50:50 mixture of anomers) δ 7.83 – 7.80 (m, 2H), 7.77 – 7.70 (m, 2H),7.46 – 7.34 (m, 6H), 5.11 – 5.04 (m, 0.5H), 4.48 – 4.41 (m, 0.5H), 4.17 – 4.06 (m, 1H), 3.98 – 3.83 (m, 3H), 3.67 – 3.61 (m, 0.5H), 3.61 – 3.55 (m, 0.5H), 3.39 (d, J = 8.4 Hz, 0.5H), 3.35 – 3.26 (m, 0.5H),2.69 (s, 0.5H), 1.85 (s, 0.5H), 1.59 (d, J = 6.4 Hz, 0.5H), 1.10 (s, 4.5H), 1.09 (s, 4.5H), 1.08 (s, 4.5H), 1.07 (s, 4.5H), 0.97 (s, 4.5H), 0.95 (s, 4.5H).
The analytical data are in accordance with the reported literature.[12]

(*5aR,8S,9S,9aR*)-8-(allyloxy)-2,2,4,4-tetraisopropyltetrahydro-6H-pyrano[3,4-f][1,3,5,2,4]trioxadisilepin-9-ol(15u')

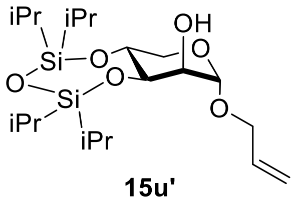

15u'

The title product compound is prepared according to the general procedure **IV**. **^{1}H NMR** (600 MHz, $CDCl_3$) δ 5.97 – 5.86 (m, 1H), 5.30 (dd, J = 17.4, 3.6 Hz, 1H), 5.21 (dd, J = 10.8, 3.0 Hz, 1H), 4.88 (s, 1H), 4.26 – 4.16 (m, 1H), 4.08 – 3.96 (m, 2H), 3.92 – 3.86 (m, 2H), 3.67 (dd, J = 10.8, 5. Hz, 1H), 3.49 (t, J = 10.8 Hz, 1H), 2.68 (s, 1H), 1.09 – 1.00 (m, 28H). **^{13}C NMR** (151 MHz, $CDCl_3$) δ 134.00, 117.55, 98.16, 74.86, 71.31, 69.87, 68.05, 62.15, 17.66, 17.64, 17.52, 17.42, 17.35, 17.34, 17.32, 13.08, 13.06, 12.36, 12.20. **ESI-HRMS**: Calculated for $C_{20}H_{40}O_6NaSi_2$ $(M+Na)^+$: 455.22556, Found: 455.22514, $[\alpha]_D^{20}$ =+40.2 (c =0.73, $CHCl_3$).

(*5aR,9S,9aR*)-2,2,4,4-tetraisopropyltetrahydro-6H-pyrano[3,4-f][1,3,5,2,4]trioxadisilepine-8,9-diol(15u)

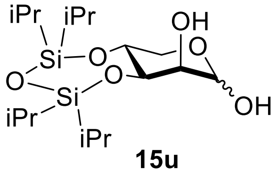

15u

The title product compound is prepared according to the general procedure **V**. **^{1}H NMR** (600 MHz, $CDCl_3$) δ 5.28 (d, J = 3.0 Hz, 1H), 4.71 (d, J = 12.0 Hz, 1H), 4.08 – 4.01 (m, 1H), 4.01 – 3.95 (m, 1H), 3.97 – 3.90 (m, 3.5H), 3.86 (d, J = 11.4 Hz, 1H), 3.74 – 3.67 (m, 3H), 3.21 – 3.14 (m, 1H), 2.69 (d, J = 12.6 Hz, 2H), 2.48 (d, J = 3.0z Hz, 1H), 2.06 (d, J = 3.0 Hz, 0.5H), 1.12 – 0.98 (m, 55H). **^{13}C NMR** (151 MHz, $CDCl_3$) δ 94.67, 93.98, 77.08, 74.41, 71.40, 71.33, 69.82, 69.58, 65.84, 62.19, 17.64, 17.61, 17.53, 17.49, 17.40, 17.33, 17.31, 17.29, 13.07, 13.03, 12.31, 12.20. **ESI-HRMS**: Calculated for $C_{17}H_{36}O_6NaSi_2$ $(M+Na)^+$: 415.19426, Found: 415.19413. $[\alpha]_D^{20}$ =+19.1 (c =0.59, $CHCl_3$).

(2*S*,3*S*,4*S*,5*R*)-2-methoxytetrahydro-2H-pyran-3,4,5-triol(15v)

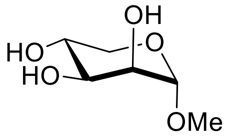

15v

^{1}H NMR (400 MHz, CD_3OD) δ 4.59 (d, J = 2.8 Hz, 1H), 3.86 – 3.76 (m, 2H), 3.73 – 3.65 (m, 2H), 3.52 – 3.44 (m, 1H), 3.41 (s, 3H). The analytical data are in accordance with the reported literature.[13]

1,4-dihydro-1,4-epoxynaphthalene (16a)

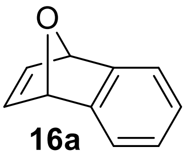

16a

The title product compound was prepared according to the previous published protocols. **^{1}H NMR** (400 MHz, $CDCl_3$) δ 7.18 – 7.16 (m, 2H), 6.95 – 6.95 (m, 2H), 6.89 (dd, *J* = 5.1, 3.0 Hz, 2H), 5.64 – 5.63 (m, 2H).

The analytical data are in accordance with the reported literature.[1-3,34]

6,7-dimethyl-1,4-dihydro-1,4-epoxynaphthalene (16b)

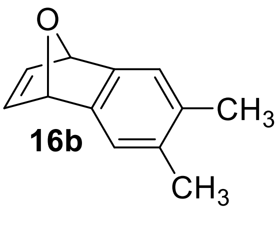

16b

The title product compound was prepared according to the previous published protocols. **^{1}H NMR** (400 MHz, $CDCl_3$) δ 7.06 (s, 2H), 7.00 (t, *J* = 1.0 Hz, 2H), 5.66 (t, *J* = 0.8 Hz, 2H), 2.20 (s, 6H).

The analytical data are in accordance with the reported literature.[1-5,34]

6,7-dimethoxy-1,4-dihydro-1,4-epoxynaphthalene(16c)

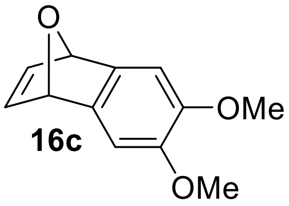

16c

The title product compound was prepared according to the previous published protocols. **^{1}H NMR** (400 MHz, $CDCl_3$) δ 7.04 (t, *J* = 1.0 Hz, 2H), 6.97 (s, 2H), 5.68 (t, *J* = 0.9 Hz, 2H), 3.85 (s, 6H).

The analytical data are in accordance with the reported literature.[1-2,4-5]

5,8-dihydro-5,8-epoxynaphtho[2,3-d][1,3]dioxole (16d)

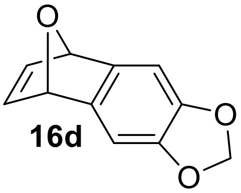

16d

The title product compound was prepared according to the previous published protocols. **^{1}H NMR** (400 MHz, $CDCl_3$) δ 7.04 (t, *J* = 1.0 Hz, 2H), 6.83 (d, *J* = 0.3 Hz, 2H), 5.94 (d, *J* = 1.5 Hz, 1H), 5.89 (d, *J* = 1.5 Hz, 1H), 5.64 (t, *J* = 0.8 Hz, 2H).
The analytical data are in accordance with the reported literature.[1-3,5]

5,8-dimethoxy-1,4-dihydro-1,4-epoxynaphthalene (16e)

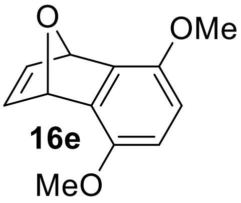

16e

The title product compound was prepared according to the previous published protocols. **^{1}H NMR** (400 MHz, $CDCl_3$) δ 7.07 (s, 2H), 6.54 (s, 2H), 5.93 (s, 2H), 3.79 (s, 6H).

The analytical data are in accordance with the reported literature.[2,4-5]

1,4-dimethyl-1,4-dihydro-1,4-epoxynaphthalene (16f)

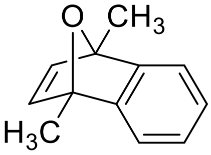

16f

The title product compound was prepared according to the previous published protocols. **^{1}H NMR** (400 MHz, $CDCl_3$) δ 7.13 (dd, *J* = 5.1, 3.0 Hz, 2H), 6.98 (dd, *J* = 5.1, 3.0 Hz, 2H), 6.78 (s, 2H), 1.90 (s, 6H).

The analytical data are in accordance with the reported literature.[3,4-5]

6,7-difluoro-1,4-dihydro-1,4-epoxynaphthalene (16g)

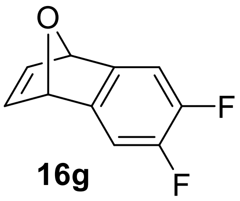

16g

The title product compound was prepared according to the previous published protocols. **^{1}H NMR** (400 MHz, $CDCl_3$) δ 7.07 (td, *J* = 7.7, 0.4 Hz, 2H), 7.03 (t, *J* = 1.0 Hz, 2H), 5.69 – 5.68 (m, 2H).

The analytical data are in accordance with the reported literature.[6-7,34]

6,7-dibromo-5,8-dimethyl-1,4-dihydro-1,4-epoxynaphthalene (16h)

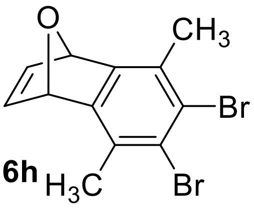

16h

The title product compound was prepared according to the previous published protocols. **^{1}H NMR** (400 MHz, $CDCl_3$) δ 7.03 (s, 2H), 5.78 (s, 2H), 2.41 (s, 6H).

The analytical data are in accordance with the reported literature.[6-7,34]

1,4-dihydro-1,4-epoxyanthracene (16i)

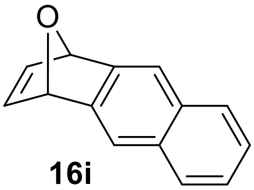

16i

The title product compound was prepared according to the previous published protocols. **^{1}H NMR** (400 MHz, $CDCl_3$) δ 7.72 (dd, *J* = 6.1, 3.3 Hz, 2H), 7.59 (s, 2H), 7.43 (dd, *J* = 6.2, 3.3 Hz, 2H), 6.97 (t, *J* = 1.0 Hz, 2H), 5.81 (s, 2H).

The analytical data are in accordance with the reported literature.[4-5,7]

But-3-en-2-yl methyl carbonate(16j)

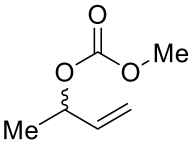

16j

^{1}H NMR (400 MHz, $CDCl_3$) δ 5.90 – 5.73 (m, 1H), 5.33 – 5.22 (m, 1H), 5.19 – 5.06 (m, 2H), 3.79 – 3.67 (m, 3H), 1.39 – 1.29 (m, 3H). The analytical data are in accordance with the reported literature.[8-9]

1-cyclohexylallyl methyl carbonate(16k)

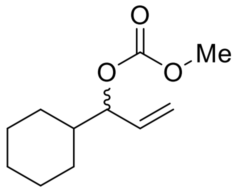

16k

^{1}H NMR (400 MHz, $CDCl_3$) δ 5.82 – 5.69 (m, 1H), 5.31 – 5.15 (m, 2H), 4.83 (t, J = 6.8 Hz, 1H), 3.75 (d, J = 0.8 Hz, 3H), 1.86 – 1.49 (m, 6H), 1.29 – 1.09 (m, 3H), 1.08 – 0.89 (m, 2H).

The analytical data are in accordance with the reported literature.[8,10]

Hex-1-en-3-yl methyl carbonate(16l)

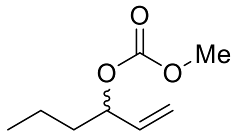

16l

^{1}H NMR (400 MHz, $CDCl_3$) δ 5.84 – 5.66 (m, 1H), 5.31 – 5.22 (m, 1H), 5.21 – 5.13 (m, 1H), 5.09 – 4.99 (m, 1H), 3.91 – 3.63 (m, 3H), 1.75 – 1.62 (m, 1H), 1.61 – 1.51 (m, 1H), 1.45 – 1.28 (m, 2H), 0.9 (t, J = 7.2 Hz, 3H).The analytical data are in accordance with the reported literature.[8,9]

Methyl (1-phenylallyl) carbonate(16m)

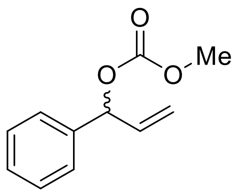

16m

^{1}H NMR (400 MHz, $CDCl_3$) δ 7.43 – 7.28 (m, 5H), 6.10 – 6.07 (m, 1H), 6.06 – 5.99 (m, 1H), 5.39 – 5.31 (m, 1H), 5.31 – 5.25 (m, 1H), 3.78 (s, 3H).The analytical data are in accordance with the reported literature.[8,10]

(4*S*,4'*S*)-2,2'-((phenylphosphanediyl)bis(2,1-phenylene))bis(4-isopropyl-4,5-dihydrooxazole)((*S*)-NPN)

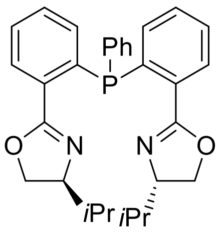

(*S*)-NPN

^{1}H NMR (400 MHz, $CDCl_3$) δ 7.91 – 7.81 (m, 2H), 7.37 – 7.21 (m, 10H), 7.00 – 6.86 (m, 2H), 4.23 – 4.07 (m, 2H), 3.98 – 3.80 (m, 4H), 1.69 – 1.49 (m, 2H), 0.78 (dd, J = 14.0, 6.8 Hz, 6H), 0.72 (t, J = 6.4 Hz, 6H). The analytical data are in accordance with the reported literature.[11]

(4*R*,4'*R*)-2,2'-((phenylphosphanediyl)bis(2,1-phenylene))bis(4-isopropyl-4,5-dihydrooxazole)((*R*)-NPN)

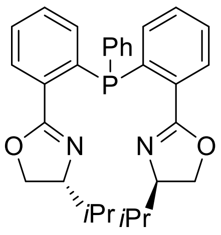

(*R*)-NPN

^{1}H NMR (400 MHz, $CDCl_3$) δ 7.89 – 7.77 (m, 2H), 7.37 – 7.16 (m, 10H), 7.00 – 6.82 (m, 2H), 4.23 – 4.04 (m, 2H), 3.94 – 3.76 (m, 4H), 1.64 – 1.44 (m, 2H), 0.75 (dd, J = 14.0, 6.8 Hz, 6H), 0.69 (t, J = 6.4 Hz, 6H).The analytical data are in accordance with the reported literature.[11]

Optimization studies:

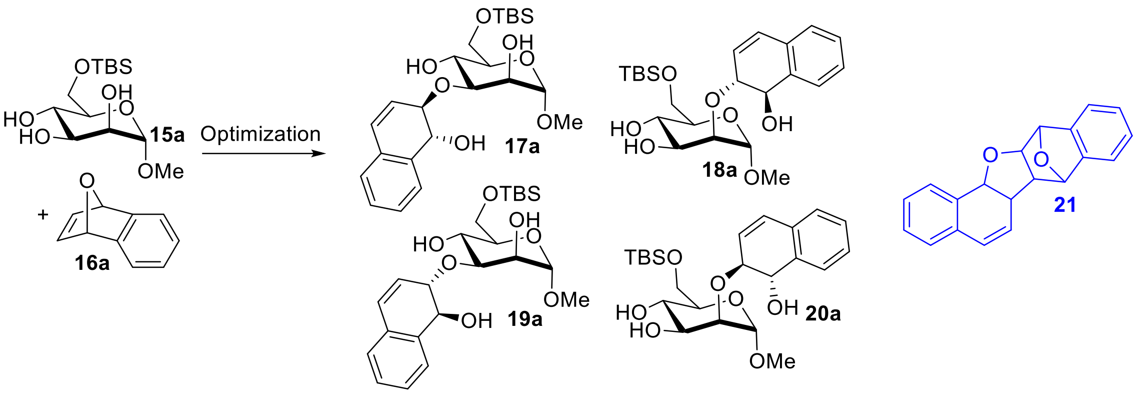

Supplementary Table 1: Initial screening search for suitable catalyst

S.No	Metal catalyst	ligand	boronic acid	base 2 equiv.	Solvent	Temp °C	time	yield (%)	**ratio 17a/18a/19a/20a/21**
1	$[Rh(cod)Cl]_2$ (5 mol%)	(*R*,*S*)-PPF-P*t*Bu_2	--	--	THF 1mL	rt	72 h	0	--
2	$[Rh(cod)Cl]_2$ (5 mol%)	(*R*,*S*)-PPF-P*t*Bu_2	--	Et_3N	THF 1mL	50	48 h	<5	nd
3	$Rh(cod)_2OTf$ (5 mol%)	(*R*,*S*)-PPF-P*t*Bu_2	--	Et_3N	THF 1mL	50	24 h	15%	8.3/1/0/0/0

Initial screening of boronic acid with $[Rh(cod)Cl]_2$ at 50 °C in THF

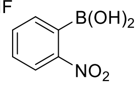

B1, (23%)[a,b]
17a/18a/19a= 23/0/0/0
r.r. >20:1 (C3:C2); d.r. >20:1

Solvent Screening with **B1** and $[Rh(cod)Cl]_2$

ACN	<5% yield
1,4-Dioxane	<5% yield
DMF	**17a/18a/19a/20a**=11/0/0/0
Toluene	<5% yield

base screening with **B1** and $[Rh(cod)Cl]_2$

Pyridine	< 5%
DBU	< 5%
$^i Pr_2NEt$	< 5%
Cs_2CO_3	< 5%
NaO^{t}Bu	< 5%
KO^{t}Bu	< 5%

Initial screening of boronic acids with $[Rh(cod)Cl]_2$ in THF solvent and Et_3N as a base at 50 °C

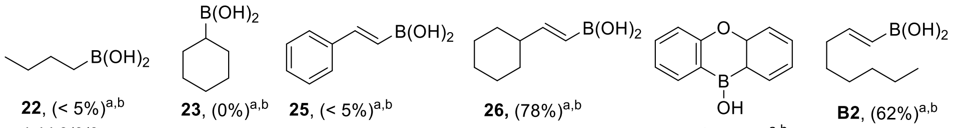

22, (< 5%)[a,b] yield 3/0/0

23, (0%)[a,b]

25, (< 5%)[a,b]

26, (78%)[a,b]
17a/18a/19a=16/1/2.5
r.r. 18:1 (C3:C2); d.r. 6.4:1

27, (0%)[a,b]

B2, (62%)[a,b]
17a/18a/19a= 12.5/1/2.5
r.r. 15:1 C3:C2;d.r. 5:1

screening of boronic acids with $Rh(cod)_2OTf$

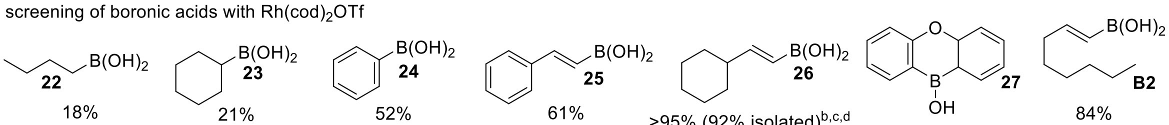

22: 18% >20:1 (**17a**:**18a**)[b,c]

23: 21% >20:1 (**17a**:**18a**)[b,c]

24: 52% >20:1 (**17a**:**18a**)[b,c]

25: 61% >20:1 (**17a**:**18a**)[b,c]

26: >95% (92% isolated)[b,c,d] >20:1 (**17a**:**18a**)

27: 85% 15:1 (**17a**:**18a**)[b,c]

B2: 84% >20:1 (**17a**:**18a**)[b,c]

Conditions: [a]Polyol **15a** (0.1 mmol), **16a** (0.2 mmol), $[Rh(cod)Cl]_2$ (5 mol%), (*R*,*S*)-PPF-P*t*Bu_2 (6 mol%), organoboron catalyst (30 mol%), in THF (1 mL), argon, 50 °C, 24 h.[b] Yields and r.r. (C3:C2) ratio were determined by crude ^1H NMR spectra analysis using 1,3,5-trimethoxybenzene as an internal standard.[c]Polyol **15a** (0.2 mmol), **16a** (0.4 mmol), $Rh(cod)_2OTf$ (5 mol%), (*R*,*S*)-PPF-P*t*Bu_2 (6 mol%), organoboron catalyst (30 mol%), in THF (2 mL), argon, 50 °C, 24 h. [d]isolated yields. nd = not determined

Supplementary Table 2: Screening of chiral ligands

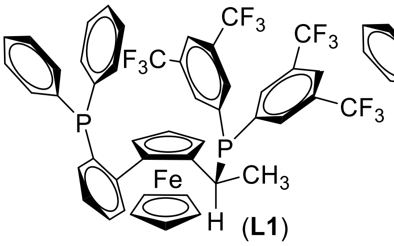
(**L1**)

65%,[a,b]
17a:**18a**:**19a**=25:31:9
C3:C2=1.09:1

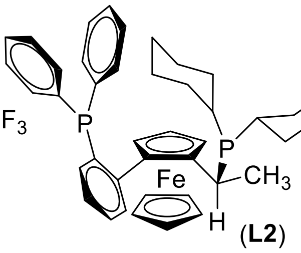
(**L2**)

88%,[a,b]
17a:**18a**:**19a**=76:12:0
C3:C2=6.3:1

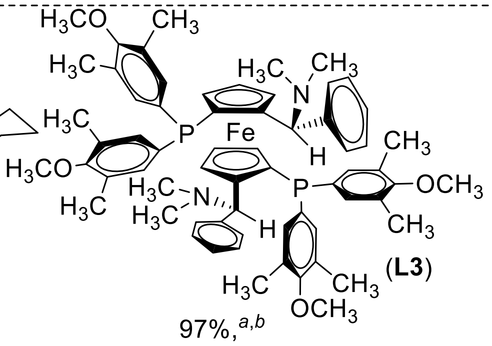
(**L3**)

97%,[a,b]
17a:**18a**:**19a**=80:12:5
C3:C2=7.08:1

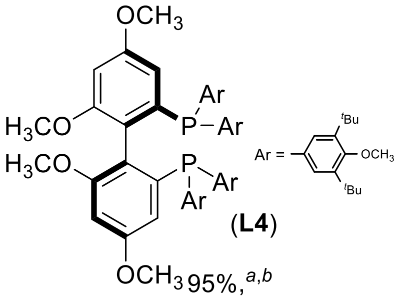
(**L4**)

95%,[a,b]
17a:**18a**:**19a**=33:17:45
C3:C2= 4.58:1

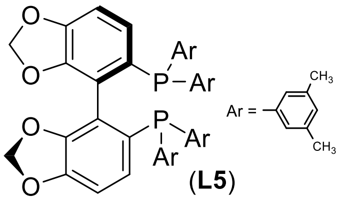
(**L5**)

63%,[a,b]
17a:**18a**:**19a**:**21**= 3:1:1:58
C3:C2= 4:1

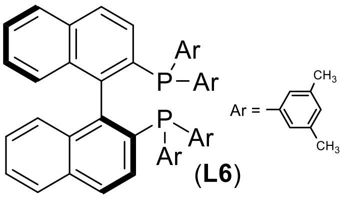
(**L6**)

70%,[a,b]
17a:**18a**:**19a**:**21**= 2:1:1:66
C3:C2= 3:1

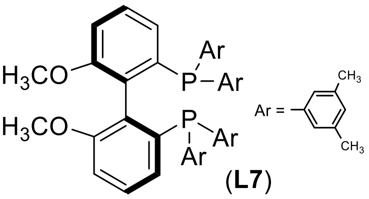
(**L7**)

97%,[a,b]
17a:**18a**:**19a**:**21**= 9:0:6:82
C3:C2 >20:1

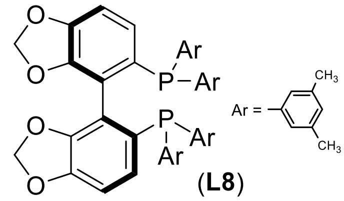
(**L8**)

94%,[a,b]
17a:**18a**:**19a**:**21**= 28:29:19:18
C3:C2= 1.6:1

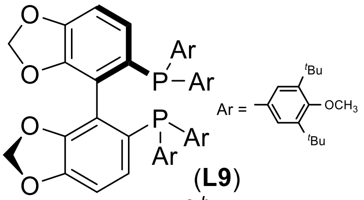
(**L9**)

94%,[a,b]
17a:**18a**:**19a**:**21**= 44:8:18:24
C3:C2= 7.75:1

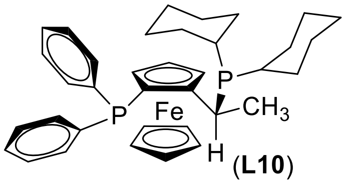
(**L10**)

22%,[a,b]
17a:**18a**:**19a**= 16:3:3
C3:C2= 6.3:1

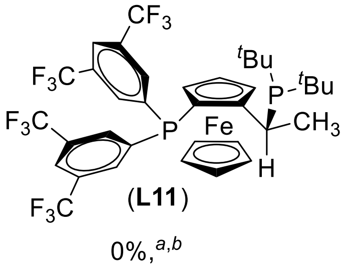
(**L11**)

0%,[a,b]

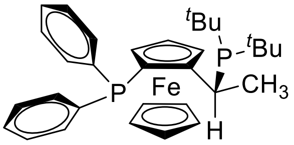
(*R*,*S*)-PPF-P^{t}Bu$_2$ Josiphos (**L12**)
92%,[c,d] C3:C2 (**17a**:**18a**) >20:1

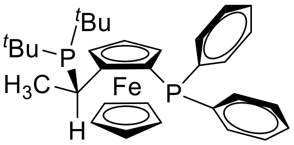
(*S*,*R*)-PPF-P^{t}Bu$_2$ Josiphos (**L13**)
85%,[c,d] C3:C2 (**19a:20a**)= 6:1

---

[a]Polyol **15a** (0.1 mmol), **16a** (0.2 mmol), Rh(cod)$_2$OTf (5 mol%), Ligand (6 mol%), organoboron catalyst (30 mol%), in THF (1 mL), argon, 50 °C, 24 h. [b]Yields and r.r. (C3:C2) ratio were determined by crude ^{1}H NMR spectra analysis using 1,3,5-trimethoxybenzene as an internal standard. [c]Polyol **15a** (0.2 mmol), **16a** (0.4 mmol), Rh(cod)$_2$OTf (5 mol%), Ligand (6 mol%), organoboron catalyst (30 mol%), in THF (2 mL), argon, 50 °C, 24 h. [d]isolated yields.

Supplementary Table 3: Screening of chiral ligands

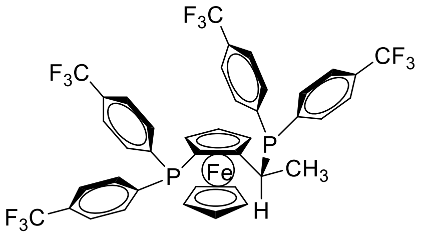

L14
0%[a,b]

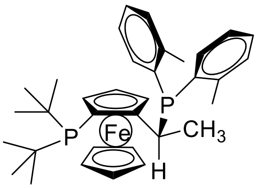

L15
0%[a,b]

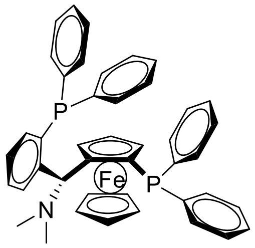

L16
10%[a,b]
17a:**18a**:**19a** = 5:0:5
C3:C2 >20:1

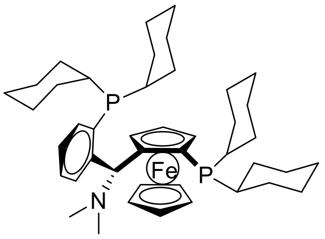

L17
25%[a,b]
17a:**18a**:**19a** = 11:2:12
C3:C2 = 11.5:1

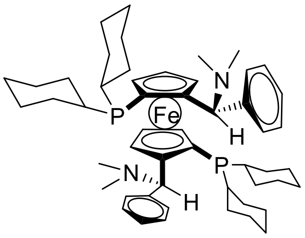

L18
20%[a,b]
17a:**18a**:**19a** = 16:1:3
C3:C2 = 19:1

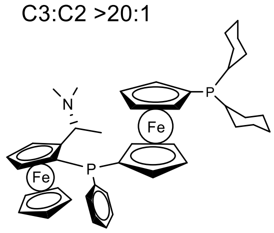

L19
3%[a,b]
17a:**18a**:**19a** = 3:0:0
C3:C>20:1

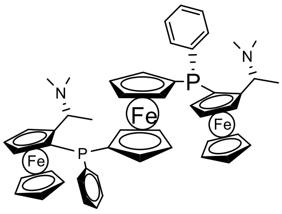

L20
26%[a,b]
17a:**18a**:**19a** = 24:1:1
C3:C2 >20:1

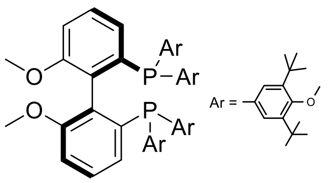

L21
90%[a,b]
17a:**18a**:**19a** = 51:14:25
C3:C2 >5.2:1

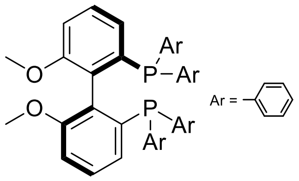

L22
88%[a,b]
17a:**18a**:**19a:21** = 11:4:0:73
C3:C2 >2.75:1

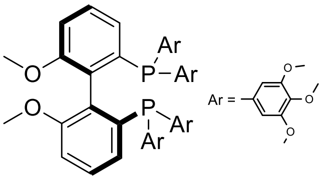

L23
17%[a,b]
17a:**18a**:**19a:21** = 6:2:5:4
C3:C2 >5.5:1

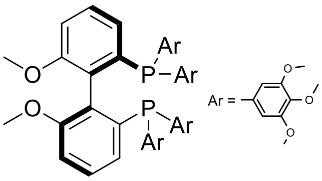

L24
5%[a,b]
17a:**18a**:**19a:21** = 1:0:1:3
C3:C2 >20:1

[a]Polyol **15a** (0.1 mmol), **16a** (0.2 mmol), Rh(cod)$_2$OTf (5 mol%), Ligand (6 mol%), organoboron catalyst (30 mol%), in THF (1 mL), argon, 50 °C, 24 h.
[b] Yields and r.r. (C3:C2) ratio were determined by crude ^{1}H NMR spectra analysis using 1,3,5-trimethoxybenzene as an internal standard.

Supplementary Table 4: Screening of cationic rhodium catalysts

Comparision with other cationic rhodium catalysts

Entry	Metal catalyst	ligand	boronic acid 30 mol%	yield[a,b,c] %	**17a/18a**
1	$Rh(cod)_2OTf$	(*R*,*S*)-PPF-P*t*Bu_2	26	92%	>20:1
2	$Rh(nbd)_2BF_4$	(*R*,*S*)-PPF-P*t*Bu_2	26	81%	>20:1
3	$Rh(nbd)_2OTf$	(*R*,*S*)-PPF-P*t*Bu_2	26	83%	>20:1

Conditions: [a]Polyol **15a** (0.2 mmol), **16a** (0.4 mmol), $Rh(cod)_2OTf$ (5 mol%), Ligand (6 mol%), organoboron catalyst (30 mol%), in THF (2 mL), argon, 50 °C, 24 h. [b] Yields and r.r. (C3:C2) ratio were determined by crude ^{1}H NMR spectra analysis using 1,3,5-trimethoxybenzene as an internal standard. [c]Isolated yields

Supplementary Table 5: Conditions screening of allylic carbonate reaction

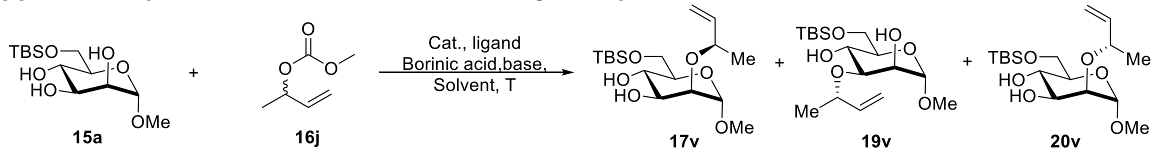

Entry	Metal catalyst	Ligand	Boronic acid (30 mol%)	Base 2 equiv.	Solvent	Temp °C	Time	Yield (%)	**Ratio 17v/19v/20v**
1	$Rh(cod)_2OTf$ (5 mol%)	(*R*,*S*)-PPF-P^tBu_2	**26**	Et_3N	THF 1mL	50	24 h	n.d.	-
2	$Rh(cod)_2OTf$ (5 mol%)	(*S*,*R*)-PPF-P^tBu_2	**26**	Et_3N	THF 1mL	50	24 h	n.d.	-
3	$Rh(cod)_2BF_4$ (5 mol%)	(*S*)-NPN	**27**	DIPEA	THF 1mL	50	24 h	n.d.	-
4	$Rh(cod)_2BF_4$ (5 mol%)	(*S*)-NPN	**27**	DIPEA	ACN 1mL	50	18 h	65%(63%[b])	20/1/0
5	$Rh(NBD)_2OTf$ (5 mol%)	(*S*)-NPN	**27**	DIPEA	ACN 1mL	50	18 h	n.d.	-
6	$Rh(NBD)_2BF_4$ (5 mol%)	(*S*)-NPN	**27**	DIPEA	ACN 1mL	50	18 h	n.d.	-
7	$Rh(NBD)_2BF_4$ (5 mol%)	(*R*)-NPN	**27**	DIPEA	ACN 1mL	50	18 h	55%	0/2/1

Conditions: [a]Polyol **15a** (0.1 mmol), **16j** (0.15 mmol), cat.(5 mol%), ligand(6 mol%), organoboron catalyst(30 mol%), argon, 50 °C, 18-24 h.[b]Isolated yield, n.d.: not detected.

Control experiments:

Experiment 1: Reaction conducted according to the general method for control experiment 1, with 5 mol% $Rh(cod)_2OTf$ catalyst, oxabicycle **16a** (0.4 mmol, 2 equiv.), and 0.2 mmol carbohydrate polyol **15a** at 50 ˚C for 24 h.

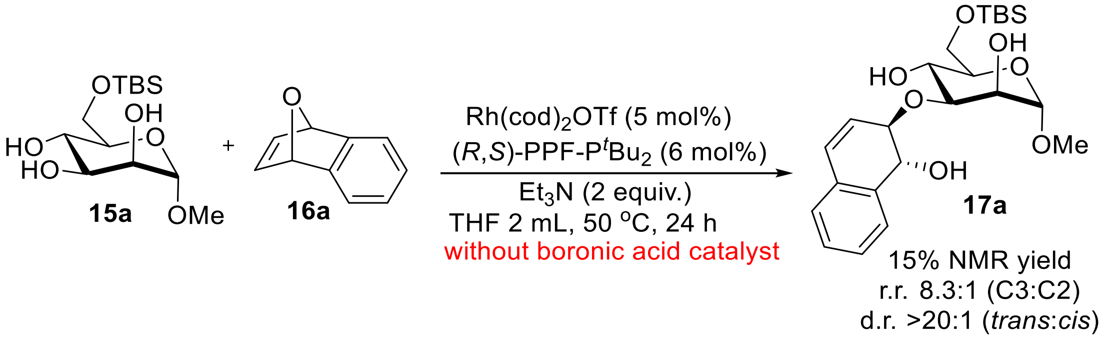

Experiment 2: Reaction conducted according to the general method for control experiment 2, with 30 mol% cyclohexyl vinyl boronic acid **26**, oxabicycle **16a** (0.4 mmol, 2 equiv.), and 0.2 mmol carbohydrate polyol **15a** at 50 ˚C for 24 h.

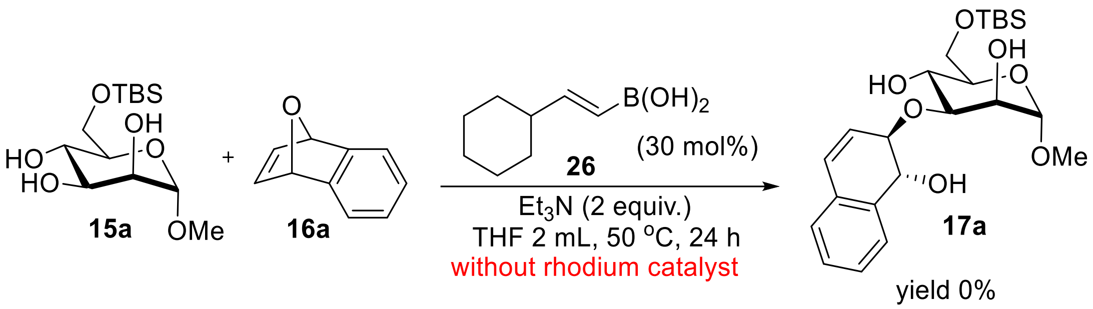

Experiment 3: without water: Reaction conducted according to the general method for control experiment 3, with 5 mol% Rh(cod)₂OTf catalyst, 30 mol% cyclohexyl vinyl boronic acid **26**, oxabicycle **16a** (0.4 mmol, 2 equiv.), 100mg 4Å MS and 0.2 mmol carbohydrate polyol **15a** at 50 ˚C for 24 h.

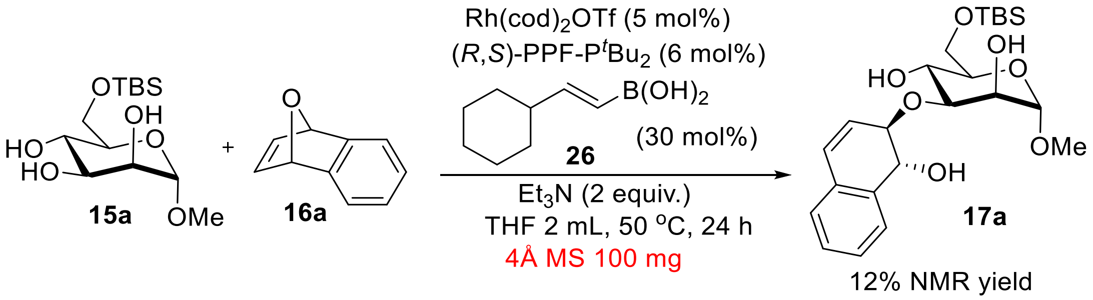

Experiment 4: with water**:** Reaction conducted according to the general method for control experiment 4, with 5 mol% Rh(cod)₂OTf catalyst, 30 mol% cyclohexyl vinyl boronic acid **26**, oxabicycle **16a** (0.4 mmol, 2 equiv.), and 0.2 mmol carbohydrate polyol **15a**, and then dry THF (2 mL), H_2O (5.5 eq,10 μL), triethyl amine base (0.4 mmol, 2 equiv.) were added, stirred at 50 ˚C for 24 h.

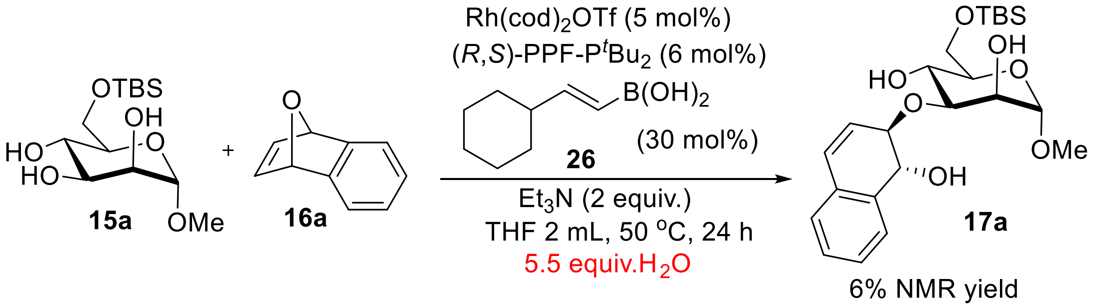

Experiment 5: with achiral ligand**:** Reaction conducted according to the general method for control experiment **5**, with dppf ligand (6.6 mg, 0.012 mmol, 6 mol%), 5 mol% Rh(cod)₂OTf catalyst, 30 mol% cyclohexyl vinyl boronic acid **26**, oxabicycle **16a** (0.4 mmol, 2 equiv.), and 0.2 mmol carbohydrate polyol **15a** at 50 ˚C for 24 h.

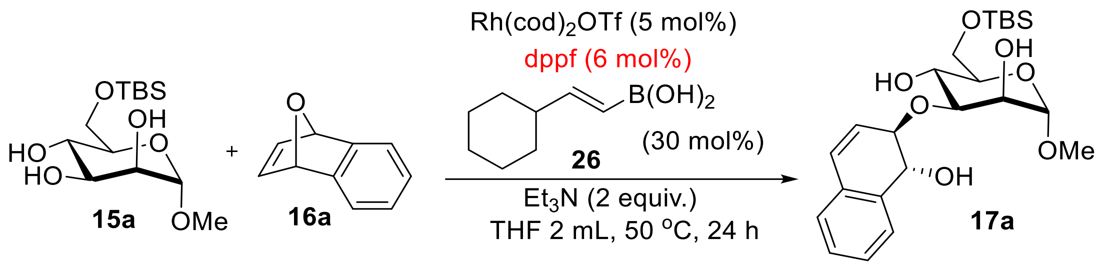

35% NMR yield
mixture of 5:1:4.6 (**17a**:**18a**:**19a**)
r.r. 9.7:1 (C3:C2), d.r. 1:1

Experiment 6: Reaction conducted according to the general method for control experiment **6**, with 5 mol% Rh(cod)₂OTf catalyst, 30 mol% borinic acid **27**, oxabicycle **16a** (0.26 mmol, 2 equiv.), and 0.13 mmol carbohydrate polyol **17p**, and then dry THF (1.3 mL), triethyl amine base (0.26 mmol, 2 equiv.) were added, stirred at 50 ˚C for 24 h.

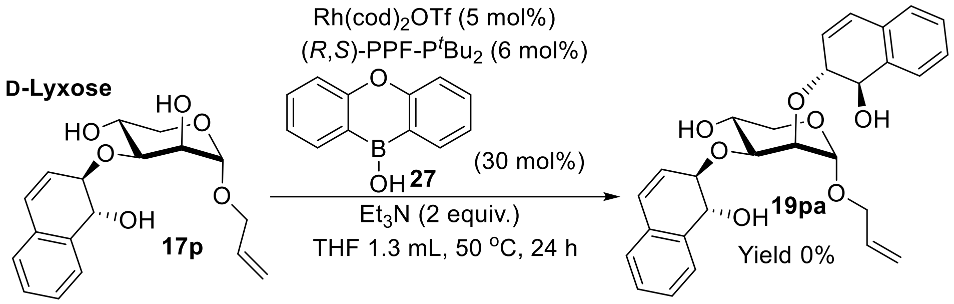

Supplementary Table 6: Optimization table for lyxose 17p

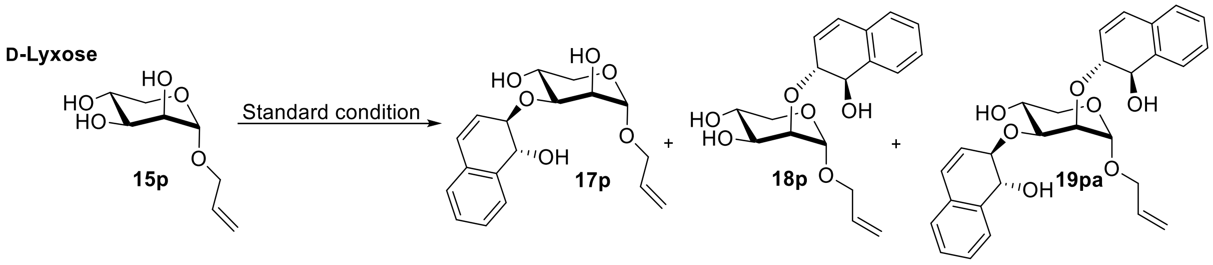

Entry	**16a**	Organoboron	Yield (ratio of **17p:18p**)^{a,b,c}	Yield of **19pa**^{a,b,c}
1	2 equiv.	**26**	90% (**17p**:**18p** = 4:1) inseparable mixture	--
2	2 equiv.	**27**	69% (**17p**:**18p** >20:1)	19%
3	1.2 equiv.	**27**	51% (**17p**:**18p** >20:1)	5%
4	1.5 equiv.	**27**	60% (**17p**:**18p** >20:1)	6%

Conditions: [a]Polyol **15p** (0.2 mmol), **16a** (0.4 mmol), $Rh(cod)_2OTf$ (5 mol%), (*R*,*S*)-PPF-P*t*Bu_2 (6 mol%), organoboron catalyst (30 mol%) in THF (2 mL), argon, 50 °C, 24 h. [b]Yields and r.r. (C3:C2) ratio were determined by crude ^1H NMR spectra analysis using 1,3,5-trimethoxybenzene as an internal standard. [c]Isolated yields

Absolute configurations of 17a-20a, 17s/u/v and 20v by VCD spectroscopy

The absolute configurations of the eight compounds shown in the scheme below were determined based on a comparison of experimental and computed VCD spectra. As the configurations of the stereocenters at the carbohydrate polyol moiety are known, only the relative configurations of the ethers/diols generated on the external electrophile had to be determined.

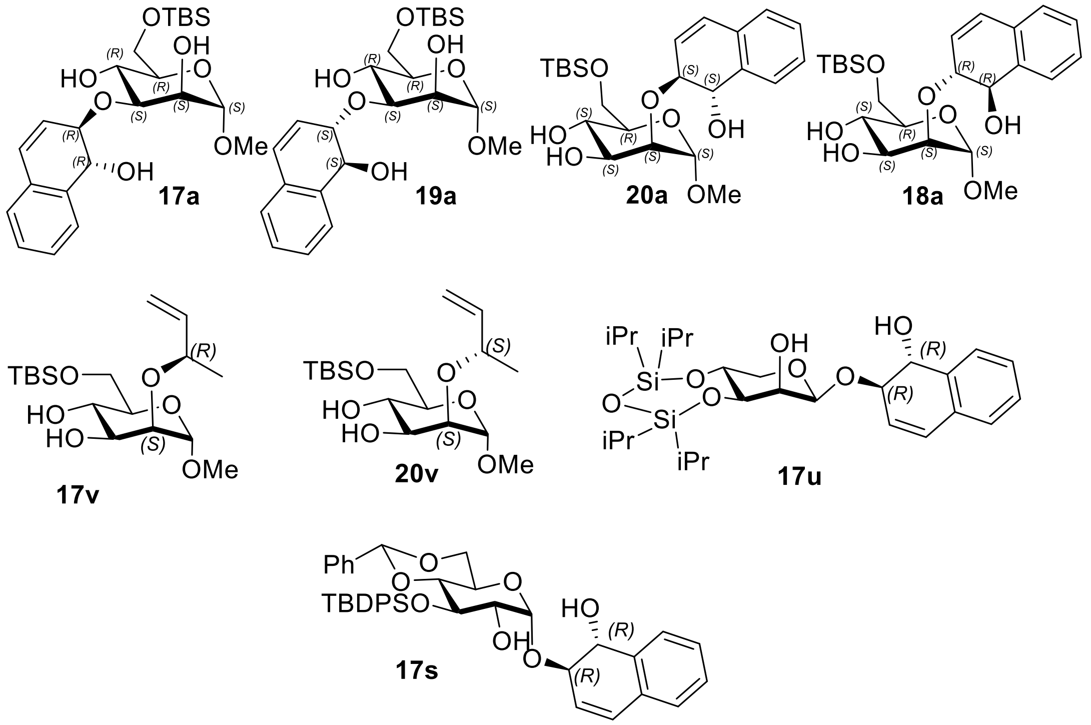

Experimental details. The IR and VCD spectra were recorded on a Bruker Vertex 70/PMA 50 VCD spectrometer at 4 cm^{-1} spectral resolution by accumulating 32 scans for the IR and ~24000 scans (6 h accumulation time) for VCD. Samples were dissolved in $CDCl_3$ and measured using a BaF_2 IR cell with 100 μm optical path length. Baseline correction of the VCD spectra was done by subtraction of the spectra of the solvent recorded under identical conditions. Concentrations are given in the captions of the figures showing the spectra.

Computational details. Deriving the absolute configuration from the experimental spectra requires the computation of IR and VCD spectra. Therefore, a conformational sampling was carried out based on a Monte-Carlo (MC) algorithm on force-field level (MMFF).[26,27] At least the first 100 lowest energy conformers of each compound were subjected to further geometry optimizations at B3LYP/6-31G(2d,p)/IEFPCM($CHCl_3$) level of theory using Gaussian 09 Rev E.01.[28] In order to evaluate whether a higher level of theory could further improve the match between experiment and theory, the lowest energy conformers of **17a**, **19a** and **20a**, that is, those that together accounted for more than 95% of the population, were further optimized at B3LYP/6-311+g(2d,p)/IEFPCM($CHCl_3$). As the different was negligible, the spectra of compounds **17s/u/v** and **20v** were computed with the smaller basis set only. For the final comparison with the experiment, the IR and VCD spectra were simulated from the single-conformer spectra using the ΔE_{ZPC}-based Boltzmann weights and by assigning a Lorentzian band shape with half-width at half-height of 6 cm^{-1} to the computed dipole and rotational strength.

Absolute configuration of the diastereomeric pair 17a/19a. The experimental and computed spectra of **17a** and **19a** are compared in **Supplementary Figure 1**. Visual comparison confirms a qualitatively good match between the experimental spectrum of **17a** and the computed structure of the structure with (*R*,*R*)-diol moiety and of **19a** with that featuring an (*S*,*S*)-diol configuration, respectively. As the spectral region below 1200cm^{-1} does not match as well as the above when using the frequency scaling factor of 0.98, we provide also an overlap without applying any scaling. Thereby also the lower-frequency range matches very nicely with the experiment. In addition to the plain spectra comparison, we computed a difference spectrum [**17a**-**19a**] to more clearly highlight the differences between the experimental VCD spectra. Computing the difference spectrum [(*S*,*S*)-(*R*,*R*)] with the computed spectra, we obtain a mirror-imaged spectrum. This further confirms our assignment of (*R*,*R*)-diol being present in **17a** and (*S*,*S*)-diol being in **19a.**

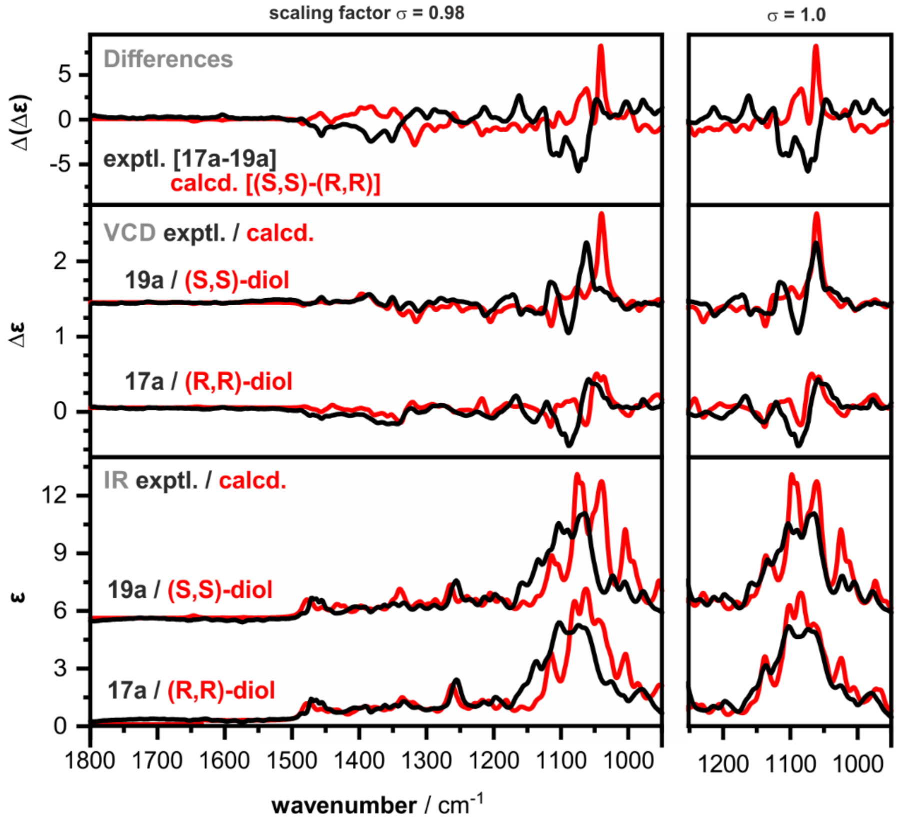

Supplementary Figure 1. Comparison of experimental and computed IR and VCD spectra of **17a** and **19a** (c=0.17 M). The molar absorptivity Ɛ is given in units of 10^{2} M^{-1} cm^{-1}, differential absorptivity ΔƐ in 10^{-1} M^{-1} cm^{-1} and the difference spectra in Δ(ΔƐ) of 10^{-2} M^{-1} cm^{-1}.

Absolute configuration of the diastereomeric pair 18a/20a. Due to a lack of sample material for **18a**, we could only record the experimental IR and VCD spectra of **20a**. We compare them with those computed for the corresponding (*S*,*S*)- and (*R*,*R*)-diols in **Supplementary Figure 2**. While the visual match is not as striking as for **17a**/**19a**, we nonetheless confidently assign the (*S*,*S*)-configuration to **20a**. Consequently, **18a** must possess (*R*,*R*) relative stereochemistry on the diol moiety.

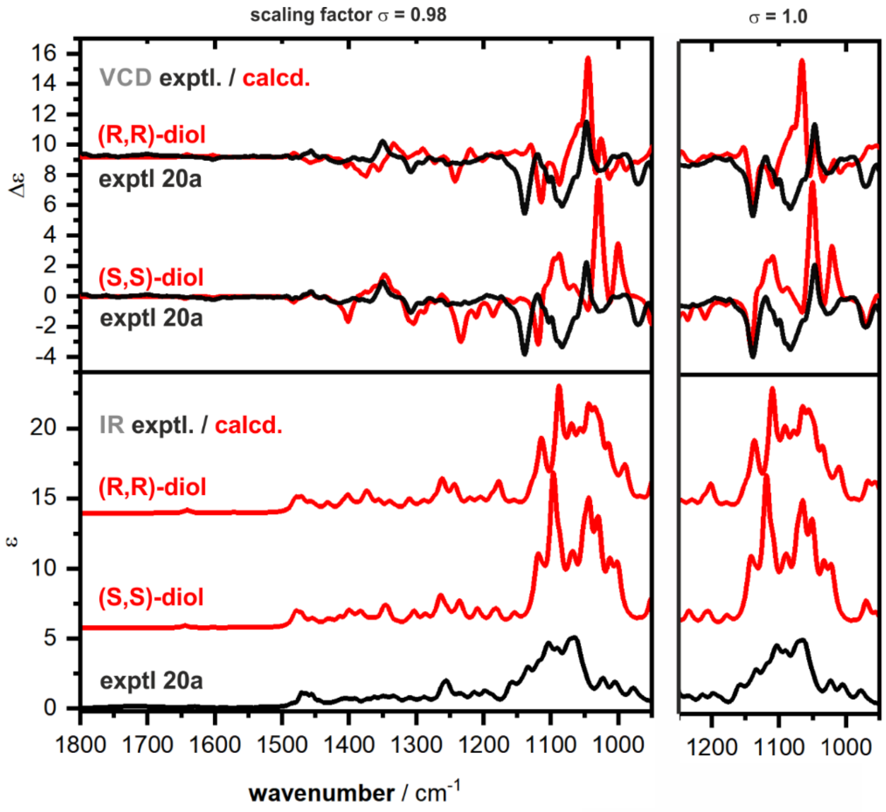

Supplementary Figure 2: Comparison of experimental IR and VCD spectra of **20a** (c=0.17 M) with those computed for the (*S*,*S*)-diol (**20a**) and (*R*,*R*)-diol (**18a**). The molar absorptivity Ɛ is given in units of 10^2 M^{-1} cm^{-1}, differential absorptivity ΔƐ in 10^{-1} M^{-1} cm^{-1} and the difference spectra in Δ(ΔƐ) of 10^{-2} M^{-1} cm^{-1}.

Absolute configuration of the diastereomeric pair 17v/20v. Both diastereomers were available in large enough quantity to record their IR and VCD spectra. They are compared with the computed spectra of the (*R*)- and (*S*)-ether isomers in **Supplementary Figure 3**. As can be seen from the direct comparison in the middle panel of the figure, difference between the experimental VCD spectra of the diastereomers are small. Likewise, also the differences in the computed spectra are not particularly strong. Hence, we again use the difference spectrum approach and compare the spectra **[17v-20v]** and **[(*S*)-(*R*)]**. With this methodology, the assignment of the unknown stereocenters as **(*R*)-17v** and **(*S*)-20v** can unambiguously be made.

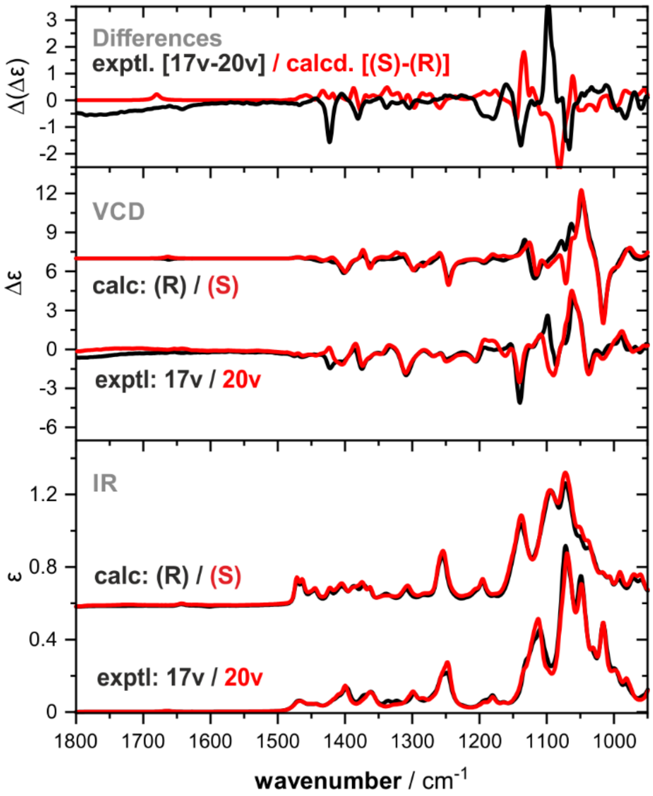

Supplementary Figure 3: Comparison of experimental IR and VCD spectra of **17v** and **20v** (c=0.22 M) with those computed for the (*R*)-ether isomer (**17v**) and (*S*)-ether isomer (**20v**). The molar absorptivity Ɛ is given in units of 10^3 M^{-1} cm^{-1}, differential absorptivity ΔƐ in 10^{-2} M^{-1} cm^{-1} and the difference spectra in Δ(ΔƐ) of 10^{-2} M^{-1} cm^{-1}.

Absolute configuration of 17u. The experimental IR and VCD spectra of **17u** are compared with the computed spectra of the (*R*,*R*)- and (*S*,*S*)-diol isomers in **Supplementary Figure 4**. Note that the experimental spectra were recorded at different concentrations for the regions above and below 1150 cm^{-1}. Over the full spectral range, the computed spectrum of the (*R*,*R*)-isomer provides a very good match with the experimental spectrum, while the (*S*,*S*)-isomer actually shows a mirror-image relation for a wide spectral range. Hence, the assignment of (*R*,*R*)-chirality to **17u** can be made with high confidence.

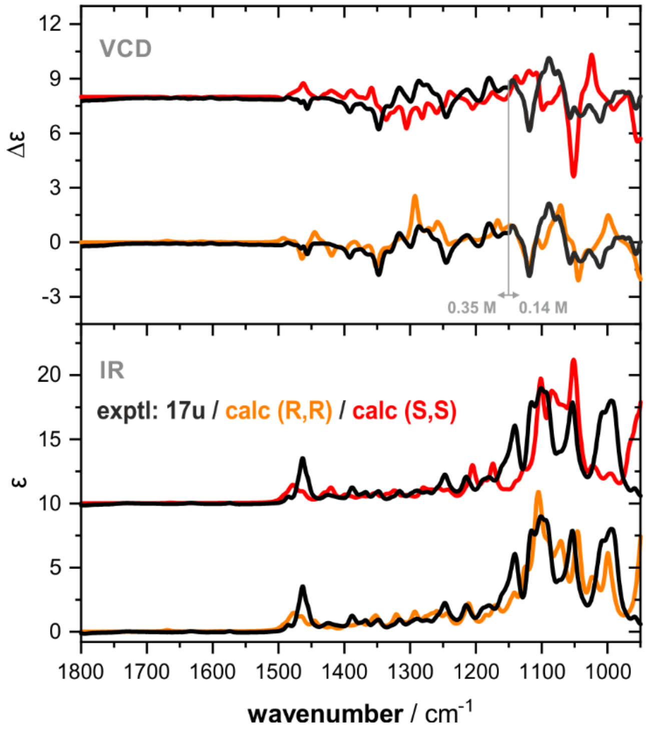

Supplementary Figure 4: Comparison of experimental IR and VCD spectra of **17u** with those computed for the (*S*,*S*)- and (*R*,*R*)-diol isomers. The molar absorptivity Ɛ is given in units of 10^2 M^{-1} cm^{-1} and the differential absorptivity ΔƐ in 10^{-2} M^{-1} cm^{-1}.

Absolute configuration of 17s. The experimental IR and VCD spectra of **17s** are compared to those computed for the (*R,R*)- and (*S,S*)-diol derivatives in **Supplementary Figure 5**. The computed spectra of the (*R*,*R*)-diol isomer provide a generally better match with the experimental VCD signatures. This can be seen, for instance, in the VCD pattern in the range 1450-1350 cm^{-1}. Also in the range 1250-950 cm^{-1}, especially when viewed in the σ=1.0 part of **Supplementary Figure 5**, a better agreement of relative intensities can be noted. Hence, we assign the (*R,R*)-configuration to the diol moiety of **17s**.

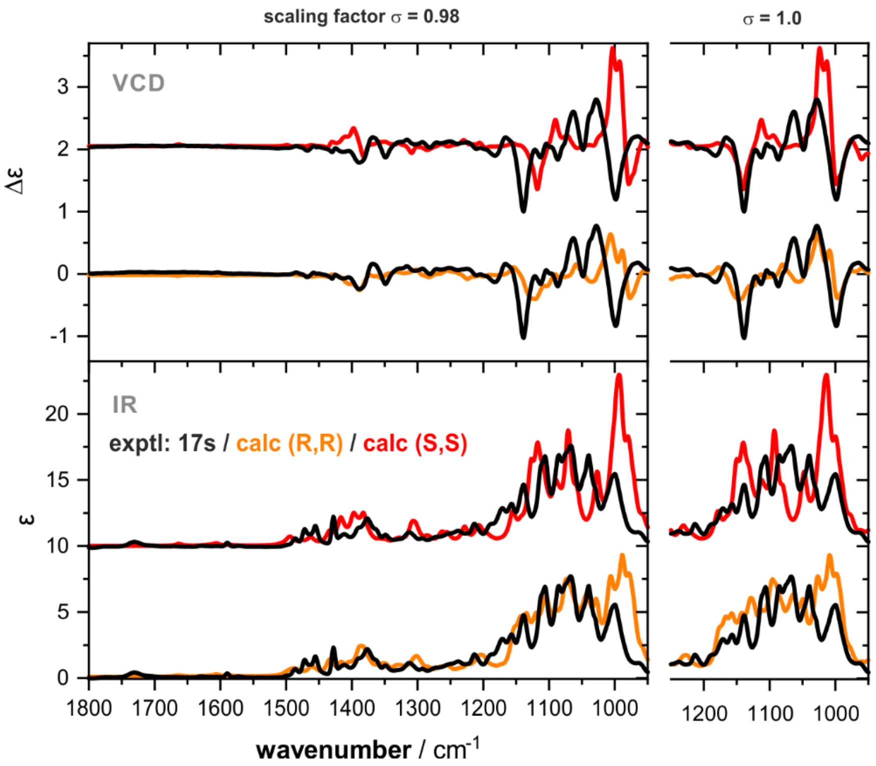

Supplementary Figure 5: Comparison of experimental IR and VCD spectra of **17s** (c=0.16 M) with those computed for the (*S,S*)- and (*R,R*)-diol isomer. The molar absorptivity Ɛ is given in units of 10^2 M^{-1} cm^{-1} and the differential absorptivity ΔƐ in 10^{-1} M^{-1} cm^{-1}.

X-Ray Crystallography Data:

(1) Crystal data for 17a (Crystal identifier B1794_0m): CCDC Deposition Number is 2097738, Unit Cell Parameters: a 6.7298(14) b 11.408(2) c 16.469(4) P1.

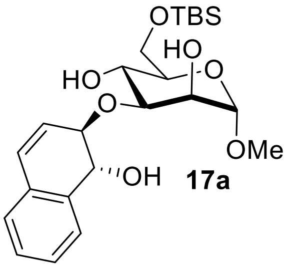

17a

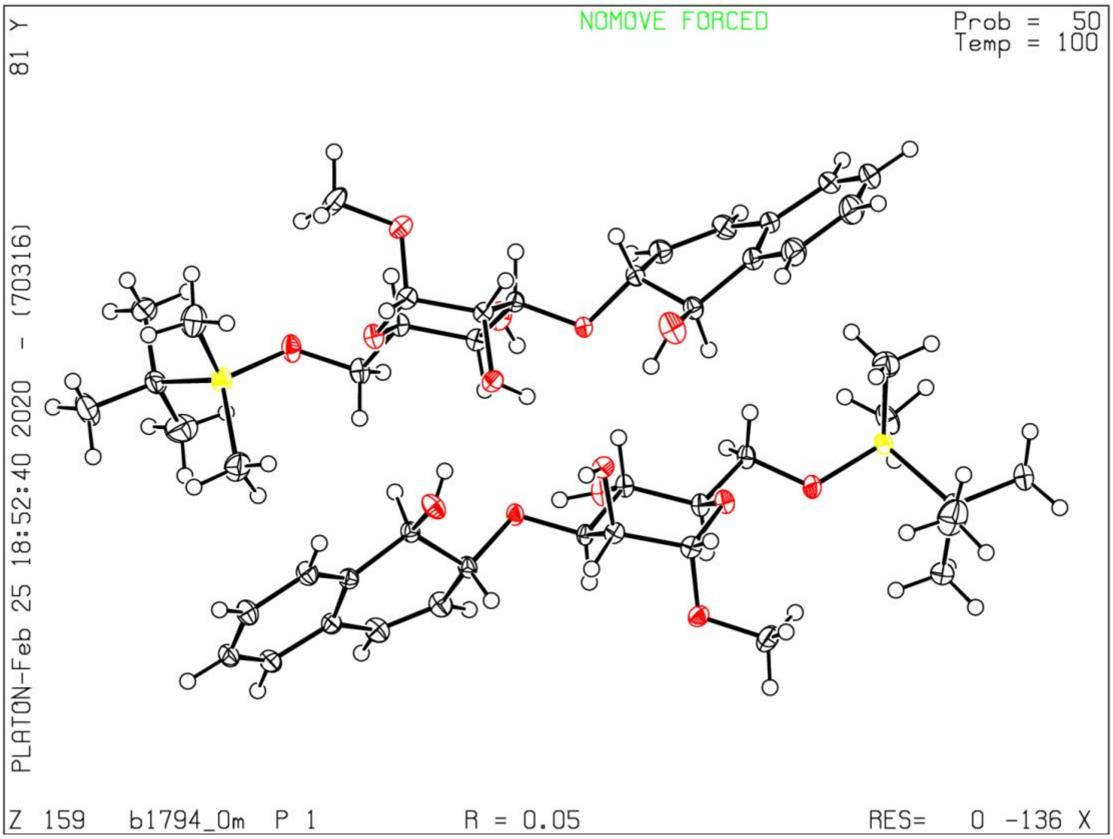

Supplementary Table 7: Crystal data and structure refinement for **B1794_0m.**

Identification code	B1794_0m
Empirical formula	$C_{23}H3_{6}O_{7}Si$
Formula weight	452.61
Temperature/K	100.0
Crystal system	triclinic
Space group	P1
a/Å	6.7298(14)
b/Å	11.408(2)
c/Å	16.469(4)

α/°	81.756(10)
β/°	86.847(10)
γ/°	75.985(9)
Volume/Å^3	1213.8(4)
Z	2
ρ_{calc}g/cm^3	1.238
μ/mm^{-1}	0.136
F(000)	488.0
Crystal size/mm^3	0.39 × 0.266 × 0.188
Radiation	MoKα (λ = 0.71073)
2Θ range for data collection/°	4.188 to 61.292
Index ranges	-9 ≤ h ≤ 9, -16 ≤ k ≤ 16, -23 ≤ l ≤ 23
Reflections collected	60460
Independent reflections	14864 [R_{int} = 0.0327, R_{sigma} = 0.0290]
Data/restraints/parameters	14864/3/595
Goodness-of-fit on F^2	1.109
Final R indexes [I>=2σ (I)]	R_1 = 0.0469, wR_2 = 0.1245
Final R indexes [all data]	R_1 = 0.0505, wR_2 = 0.1273
Largest diff. peak/hole / e Å^{-3}	1.00/-0.35
Flack parameter	0.02(3)

Supplementary Table 8: Fractional Atomic Coordinates (×10^4) and Equivalent Isotropic Displacement Parameters (Å^2×10^3) for B1794_0m. U_{eq} is defined as 1/3 of of the trace of the orthogonalised U_{IJ} tensor.

Atom	x	y	z	U(eq)
Si1	5204.3(12)	2292.4(7)	8896.2(5)	19.57(16)
O1	7026(3)	7562(2)	3953.5(16)	26.9(5)
O2	4380(3)	6515.3(18)	5088.9(12)	15.9(4)
O3	6290(3)	4523.6(19)	7316.1(13)	16.4(4)
O4	8003(3)	5082.2(19)	5697.0(13)	16.5(4)
O5	6368(3)	6529(2)	7482.3(13)	20.1(4)
O6	1458(3)	5656(2)	6231.4(15)	22.4(4)
O7	3472(3)	3316(2)	8331.4(14)	21.9(4)
C1	2046(5)	9767(3)	3565.5(19)	21.4(6)
C2	1252(7)	10904(3)	3106(2)	30.7(7)

C3	2550(8)	11494(3)	2616(2)	38.0(9)
C4	4619(8)	10962(4)	2573(2)	38.0(9)
C5	5410(6)	9800(3)	3000(2)	29.0(7)
C6	4140(5)	9201(3)	3492.0(18)	20.2(5)
C7	4861(4)	7903(3)	3913.9(18)	17.9(5)
C8	3838(4)	7783(3)	4756.6(17)	16.7(5)
C9	1553(5)	8221(3)	4693.1(19)	21.2(5)
C10	729(5)	9150(3)	4122(2)	23.8(6)
C11	4595(4)	6284(2)	5961.6(16)	14.5(5)
C12	6858(4)	5998(2)	6158.8(16)	13.8(4)
C13	7190(4)	5520(3)	7063.8(17)	15.4(5)
C14	4124(4)	4776(3)	7185.6(17)	15.9(5)
C15	3628(4)	5229(3)	6278.5(17)	14.9(5)
C16	7018(7)	6348(4)	8309(2)	34.6(8)
C17	3450(4)	3603(3)	7465.6(18)	18.3(5)
C18	6840(6)	3097(4)	9383(2)	31.8(7)
C19	6821(6)	1187(4)	8260(2)	32.5(8)
C20	3691(5)	1500(3)	9703.0(19)	22.5(6)
C21	2363(5)	2436(3)	10214(2)	25.0(6)
C22	5135(7)	538(4)	10281(3)	39.9(10)
C23	2291(7)	895(4)	9286(3)	37.6(9)
Si2	274.5(11)	7184.5(7)	1415.7(5)	15.51(14)
O8	10159(3)	2697(2)	5709.6(15)	20.6(4)
O9	6224(3)	3026.2(18)	4955.2(12)	16.1(4)
O10	5475(3)	5165.6(18)	2688.7(12)	15.5(4)
O11	8170(3)	4774.3(19)	4072.3(12)	15.4(4)
O12	7343(3)	3248.8(19)	2392.9(13)	18.2(4)
O13	2271(3)	3892(2)	4278.2(14)	19.8(4)
O14	1543(3)	5915(2)	1968.0(13)	19.7(4)
C24	8829(5)	-248(3)	6419.2(17)	18.0(5)
C25	9680(5)	-1360(3)	6899.8(19)	21.7(6)
C26	11508(5)	-1518(3)	7301.4(19)	23.2(6)
C27	12511(5)	-575(3)	7229(2)	24.1(6)
C28	11650(5)	551(3)	6768.3(19)	19.9(5)

C29	9825(4)	713(2)	6370.1(17)	15.7(5)
C30	8732(4)	1954(2)	5933.6(17)	15.3(5)
C31	7552(4)	1842(2)	5195.7(17)	15.9(5)
C32	6314(5)	892(3)	5399(2)	20.9(5)
C33	6927(5)	-73(3)	5972(2)	21.4(5)
C34	5981(4)	3351(2)	4091.7(16)	13.2(4)
C35	7805(4)	3809(3)	3679.0(17)	14.4(5)
C36	7341(4)	4275(3)	2776.2(17)	15.0(5)
C37	3730(4)	4740(2)	3035.5(17)	14.0(4)
C38	4020(4)	4350(3)	3957.1(16)	14.3(5)
C39	7148(6)	3533(3)	1524.1(19)	26.7(6)
C40	1901(4)	5800(3)	2820.9(17)	16.3(5)
C41	1094(6)	8532(3)	1689(2)	27.3(6)
C42	-2544(5)	7381(3)	1615(2)	25.0(6)
C43	993(5)	6925(3)	322.4(19)	21.7(6)
C44	3268(6)	6908(5)	168(3)	40.8(10)
C45	-303(6)	7942(3)	-280(2)	27.0(6)
C46	613(8)	5699(3)	172(2)	38.1(9)

Supplementary Table 9: Anisotropic Displacement Parameters ($Å^2×10^3$) for B1794_0m. The Anisotropic displacement factor exponent takes the form: $-2π^2[h^2a^{*2}U_{11}+2hka^*b^*U_{12}+…]$.

Atom	U11	U22	U33	U23	U13	U12
Si1	16.7(4)	22.6(4)	16.4(4)	0.7(3)	1.8(3)	-1.4(3)
O1	13.0(10)	29.9(12)	34.5(13)	4.9(10)	2.0(9)	-4.7(8)
O2	18.5(9)	14.6(8)	13.3(8)	-0.1(7)	-2.4(7)	-1.6(7)
O3	10.3(8)	19.9(9)	18.4(9)	1.5(7)	-1.6(7)	-4.4(7)
O4	10.8(8)	21.5(10)	16.2(9)	-4.6(7)	-0.9(7)	-0.3(7)
O5	20.7(10)	24.1(10)	16.8(9)	-5.9(8)	1.2(7)	-6.7(8)
O6	11.5(9)	33.5(12)	22.1(10)	-1.5(9)	-0.5(8)	-6.5(8)
O7	18.5(10)	26.1(11)	17.2(10)	4.6(8)	2.1(8)	-2.4(8)
C1	29.1(15)	16.0(12)	16.8(12)	-1.7(10)	-4.8(11)	-0.3(11)
C2	48(2)	17.0(14)	21.7(14)	-2.6(11)	-8.1(14)	4.3(13)
C3	71(3)	17.4(14)	22.1(15)	2.6(12)	-5.8(16)	-5.2(15)
C4	64(3)	27.3(17)	25.0(17)	3.2(14)	3.2(17)	-20.3(18)
C5	35.6(18)	27.3(16)	25.1(15)	0.5(12)	1.7(13)	-12.3(13)

C6	25.6(14)	18.0(12)	17.2(12)	-0.9(10)	-1.5(10)	-6.1(11)
C7	17.8(12)	17.7(12)	17.3(12)	0.7(10)	0.6(10)	-4.3(10)
C8	17.7(12)	15.0(12)	15.9(12)	-0.1(9)	-1.4(9)	-2.2(9)
C9	17.3(13)	21.7(13)	21.8(13)	-1.9(10)	2.6(10)	-0.7(10)
C10	17.8(13)	22.7(14)	26.4(15)	-2.5(11)	-2.8(11)	3.7(10)
C11	13.2(11)	16.4(11)	12.9(11)	-1.1(9)	-0.1(8)	-2.2(9)
C12	9.8(10)	17.7(11)	14.5(11)	-3.2(9)	0.5(8)	-3.8(8)
C13	10.7(11)	20.2(12)	16.1(12)	-2.9(9)	0.1(9)	-5.0(9)
C14	12.2(11)	19.0(12)	16.4(11)	0.0(9)	-1.0(9)	-4.4(9)
C15	8.6(10)	19.5(12)	16.7(11)	-1.9(9)	-0.4(9)	-3.8(9)
C16	47(2)	44(2)	15.3(14)	-9.7(14)	-0.3(14)	-11.3(17)
C17	15.2(12)	21.7(13)	18.0(13)	2.9(10)	-1.5(10)	-7.6(10)
C18	28.1(17)	39.9(19)	27.7(16)	0.5(14)	-3.6(13)	-10.8(14)
C19	27.7(17)	38.8(19)	23.9(15)	-5.1(14)	3.4(13)	5.8(14)
C20	27.5(15)	17.5(12)	19.1(13)	0.7(10)	4.3(11)	-1.7(11)
C21	25.2(15)	24.8(14)	23.0(14)	-3.6(11)	6.6(12)	-3.3(11)
C22	45(2)	28.5(18)	31.4(18)	10.7(14)	8.3(16)	8.7(15)
C23	50(2)	32.5(18)	36.7(19)	-7.8(15)	10.9(17)	-22.2(17)
Si2	13.5(3)	16.1(3)	15.7(3)	0.6(3)	-1.8(3)	-2.2(2)
O8	15.6(10)	16.4(10)	28.5(11)	2.1(8)	-3.4(8)	-3.4(8)
O9	14.4(9)	17.5(9)	12.9(8)	0.4(7)	-1.3(7)	1.8(7)
O10	9.0(8)	18.5(9)	17.4(9)	1.6(7)	-0.2(7)	-2.3(7)
O11	8.6(8)	20.3(9)	17.7(9)	-4.1(7)	-0.4(7)	-3.4(7)
O12	18.8(9)	20.2(9)	14.8(9)	-3.7(7)	1.0(7)	-2.6(7)
O13	11.3(9)	26.2(11)	19.7(10)	4.3(8)	-0.2(7)	-4.6(8)
O14	20.5(10)	19.9(10)	15.8(9)	0.2(7)	-5.5(8)	0.2(8)
C24	20.1(13)	16.8(12)	15.6(12)	-1.6(9)	2.1(10)	-2.4(9)
C25	25.6(15)	16.8(12)	20.8(13)	-0.5(10)	1.1(11)	-2.6(10)
C26	26.4(15)	18.3(13)	19.1(13)	0.0(10)	-0.5(11)	4.3(11)
C27	21.9(14)	22.4(14)	23.8(14)	-0.4(11)	-7.0(11)	2.8(11)
C28	15.9(12)	20.7(13)	21.9(13)	-0.9(10)	-3.9(10)	-2.2(10)
C29	13.6(11)	16.3(12)	15.7(11)	-1.6(9)	-0.1(9)	-1.1(9)
C30	13.1(11)	15.5(11)	15.7(11)	-0.6(9)	-0.5(9)	-1.4(9)
C31	13.1(11)	16.4(12)	16.0(11)	0.2(9)	-2.2(9)	-0.3(9)

C32	16.9(13)	22.1(13)	24.5(14)	-1.2(11)	-4.8(10)	-6.1(10)
C33	18.6(13)	20.3(13)	26.2(14)	-1.0(11)	-0.7(11)	-7.5(10)
C34	10.2(10)	16.3(11)	12.0(10)	0.2(8)	-1.3(8)	-1.7(8)
C35	8.6(10)	19.2(12)	15.3(11)	-2.8(9)	0.3(8)	-2.7(9)
C36	8.4(10)	20.0(12)	16.6(11)	-2.3(9)	1.0(9)	-3.6(9)
C37	8.2(10)	18.4(12)	14.8(11)	0.7(9)	-1.3(8)	-3.3(9)
C38	9.4(10)	18.1(11)	14.4(11)	1.4(9)	-0.5(8)	-3.2(9)
C39	33.9(17)	31.5(16)	15.5(13)	-4.6(11)	0.9(12)	-8.7(13)
C40	10.5(11)	21.0(12)	15.3(12)	1.2(10)	-2.7(9)	-1.6(9)
C41	30.1(16)	24.0(15)	30.0(16)	-3.5(12)	-3.1(13)	-10.4(12)
C42	15.2(13)	23.9(14)	31.0(16)	7.8(12)	0.4(11)	-1.8(10)
C43	25.1(14)	20.5(13)	17.2(12)	-0.8(10)	-2.0(11)	-1.5(11)
C44	25.8(18)	60(3)	30.8(19)	-6.3(18)	7.7(14)	-1.1(17)
C45	34.5(17)	23.6(15)	19.2(13)	3.2(11)	-5.2(12)	-2.2(12)
C46	70(3)	19.1(15)	22.3(15)	-3.7(12)	-8.1(16)	-2.7(16)

Supplementary Table 10: Bond Lengths for B1749_0m.

Atom	Atom	Length/Å	Atom	Atom	Length/Å
Si1	O7	1.654(2)	Si2	O14	1.662(2)
Si1	C18	1.869(4)	Si2	C41	1.872(3)
Si1	C19	1.861(4)	Si2	C42	1.871(3)
Si1	C20	1.890(3)	Si2	C43	1.885(3)
O1	C7	1.417(4)	O8	C30	1.430(3)
O2	C8	1.437(3)	O9	C31	1.442(3)
O2	C11	1.432(3)	O9	C34	1.425(3)
O3	C13	1.415(3)	O10	C36	1.411(3)
O3	C14	1.437(3)	O10	C37	1.436(3)
O4	C12	1.427(3)	O11	C35	1.430(3)
O5	C13	1.408(3)	O12	C36	1.407(3)
O5	C16	1.425(4)	O12	C39	1.427(4)
O6	C15	1.426(3)	O13	C38	1.445(3)
O7	C17	1.417(4)	O14	C40	1.420(3)
C1	C2	1.402(4)	C24	C25	1.405(4)
C1	C6	1.407(4)	C24	C29	1.408(4)

C1	C10	1.467(5)	C24	C33	1.469(4)
C2	C3	1.386(6)	C25	C26	1.390(5)
C3	C4	1.380(7)	C26	C27	1.390(5)
C4	C5	1.403(5)	C27	C28	1.403(4)
C5	C6	1.380(4)	C28	C29	1.384(4)
C6	C7	1.518(4)	C29	C30	1.524(4)
C7	C8	1.518(4)	C30	C31	1.524(4)
C8	C9	1.502(4)	C31	C32	1.512(4)
C9	C10	1.342(4)	C32	C33	1.340(4)
C11	C12	1.522(4)	C34	C35	1.536(4)
C11	C15	1.519(4)	C34	C38	1.524(4)
C12	C13	1.521(4)	C35	C36	1.528(4)
C14	C15	1.536(4)	C37	C38	1.529(4)
C14	C17	1.517(4)	C37	C40	1.519(4)
C20	C21	1.537(4)	C43	C44	1.534(5)
C20	C22	1.530(5)	C43	C45	1.538(4)
C20	C23	1.535(6)	C43	C46	1.537(5)

Supplementary Table 11: Bond Angles for B1794_0m.

Atom	Atom	Atom	Angle/˚	Atom	Atom	Atom	Angle/˚
O7	Si1	C18	108.90(16)	O14	Si2	C41	110.01(14)
O7	Si1	C19	110.90(15)	O14	Si2	C42	109.76(13)
O7	Si1	C20	105.34(13)	O14	Si2	C43	103.90(13)
C18	Si1	C20	110.66(16)	C41	Si2	C43	111.93(15)
C19	Si1	C18	110.05(19)	C42	Si2	C41	109.65(16)
C19	Si1	C20	110.89(17)	C42	Si2	C43	111.45(16)
C11	O2	C8	114.7(2)	C34	O9	C31	114.5(2)
C13	O3	C14	114.7(2)	C36	O10	C37	113.9(2)
C13	O5	C16	113.1(3)	C36	O12	C39	112.8(2)
C17	O7	Si1	127.9(2)	C40	O14	Si2	124.36(19)
C2	C1	C6	119.8(3)	C25	C24	C29	119.0(3)
C2	C1	C10	121.1(3)	C25	C24	C33	121.2(3)
C6	C1	C10	119.1(3)	C29	C24	C33	119.8(3)
C3	C2	C1	119.9(4)	C26	C25	C24	120.4(3)

C4	C3	C2	120.2(3)	C25	C26	C27	120.1(3)
C3	C4	C5	120.3(4)	C26	C27	C28	120.0(3)
C6	C5	C4	120.3(4)	C29	C28	C27	120.0(3)
C1	C6	C7	117.5(3)	C24	C29	C30	117.7(2)
C5	C6	C1	119.5(3)	C28	C29	C24	120.4(3)
C5	C6	C7	122.8(3)	C28	C29	C30	121.7(3)
O1	C7	C6	110.8(2)	O8	C30	C29	110.1(2)
O1	C7	C8	112.2(2)	O8	C30	C31	111.6(2)
C6	C7	C8	109.4(2)	C31	C30	C29	112.2(2)
O2	C8	C7	107.2(2)	O9	C31	C30	106.8(2)
O2	C8	C9	110.2(2)	O9	C31	C32	110.5(2)
C9	C8	C7	110.6(2)	C32	C31	C30	111.6(2)
C10	C9	C8	120.4(3)	C33	C32	C31	121.0(3)
C9	C10	C1	120.4(3)	C32	C33	C24	121.2(3)
O2	C11	C12	109.0(2)	O9	C34	C35	111.9(2)
O2	C11	C15	107.6(2)	O9	C34	C38	107.4(2)
C15	C11	C12	112.1(2)	C38	C34	C35	109.9(2)
O4	C12	C11	110.4(2)	O11	C35	C34	110.6(2)
O4	C12	C13	107.7(2)	O11	C35	C36	109.3(2)
C13	C12	C11	110.7(2)	C36	C35	C34	109.2(2)
O3	C13	C12	111.9(2)	O10	C36	C35	111.4(2)
O5	C13	O3	113.1(2)	O12	C36	O10	112.8(2)
O5	C13	C12	105.3(2)	O12	C36	C35	106.7(2)
O3	C14	C15	111.2(2)	O10	C37	C38	109.2(2)
O3	C14	C17	107.2(2)	O10	C37	C40	105.1(2)
C17	C14	C15	110.8(2)	C40	C37	C38	114.0(2)
O6	C15	C11	108.4(2)	O13	C38	C34	110.2(2)
O6	C15	C14	107.1(2)	O13	C38	C37	107.6(2)
C11	C15	C14	110.8(2)	C34	C38	C37	108.9(2)
O7	C17	C14	111.6(2)	O14	C40	C37	108.7(2)
C21	C20	Si1	109.6(2)	C44	C43	Si2	109.2(2)
C22	C20	Si1	110.5(2)	C44	C43	C45	109.4(3)
C22	C20	C21	108.3(3)	C44	C43	C46	109.5(3)
C22	C20	C23	109.8(3)	C45	C43	Si2	110.6(2)

C23	C20	Si1	109.6(2)	C46	C43	Si2	109.8(2)
C23	C20	C21	108.9(3)	C46	C43	C45	108.4(3)

Supplementary Table 12 Hydrogen Bonds for B1794_0m.

D	H	A	d(D-H)/Å	d(H-A)/Å	d(D-A)/Å	D-H-A/°
O4	H4	O11	0.85(6)	2.10(6)	2.741(3)	132(5)
O6	H6	O41	0.81(5)	2.14(5)	2.782(3)	136(5)
O8	H8A	O4	0.74(6)	2.02(6)	2.754(3)	169(6)
O11	H11A	O132	0.87(4)	1.86(4)	2.721(3)	167(4)

[1]-1+X,+Y,+Z; [2]1+X,+Y,+Z

Supplementary Table 13: Hydrogen Atom Coordinates (Å×10^4) and Isotropic Displacement Parameters (Å^2×10^3) for B1794_0m.

Atom	x	y	z	U(eq)
H1	7400(70)	6780(50)	4010(30)	31(12)
H4	7310(90)	5110(50)	5280(40)	52(16)
H6	980(80)	5310(50)	5930(30)	33(12)
H2	-173.96	11268.05	3130.9	37
H3	2013.93	12267.39	2308.33	46
H4A	5510.48	11384.93	2253.19	46
H5	6826.4	9424.39	2949.96	35
H7	4417.41	7348.21	3580.75	21
H8	4356.35	8271.65	5118.72	20
H9	687.01	7838.28	5058.76	25
H10	-718	9409.26	4079.1	29
H11	3854.91	7023.94	6209.28	17
H12	7375.07	6755.64	6020.53	17
H13	8695.6	5248.73	7160.99	18
H14	3396.69	5413.67	7527.09	19
H15	4119.31	4547.56	5941.52	18
H16A	8518.48	6134.55	8319.01	52
H16B	6492.35	5686.04	8623.01	52
H16C	6491.35	7099.75	8554.8	52
H17A	2048.39	3689.42	7272.42	22
H17B	4375.71	2927.49	7217.03	22

H18A	7695.47	3444.74	8960.47	48
H18B	7718.51	2516.22	9788.26	48
H18C	5964.58	3751.45	9655.7	48
H19A	5939.55	855.91	7945.76	49
H19B	7690.28	520.95	8617.19	49
H19C	7685.54	1602.03	7880.89	49
H21A	3246.7	2819.77	10487.64	38
H21B	1567.93	2024.25	10627.38	38
H21C	1427.96	3061.21	9853.07	38
H22A	5921.7	-101.39	9973.17	60
H22B	4331.83	179.28	10719.67	60
H22C	6075.66	921.77	10521.87	60
H23A	1427.43	1505.73	8892.3	56
H23B	1422.3	546.02	9702.4	56
H23C	3130.79	246.31	8998.06	56
H8A	9550(90)	3320(60)	5770(40)	48(16)
H11A	9480(60)	4600(30)	4160(20)	12(8)
H13A	2590(80)	3390(50)	4700(30)	38(13)
H25	9002.03	-2007.95	6950.4	26
H26	12073.42	-2272.25	7626.05	28
H27	13779.55	-693.17	7491.49	29
H28	12318.83	1200.95	6729.83	24
H30	7716.13	2362.98	6332.27	18
H31	8538.67	1617.32	4735.16	19
H32	5082.02	979.52	5117.15	25
H33	6116.91	-654.65	6090.65	26
H34	5840.49	2623.79	3845.14	16
H35	9055.22	3120.59	3717.32	17
H36	8470.08	4648.02	2526.67	18
H37	3599.55	4028.12	2769.93	17
H38	4106.2	5063.91	4230.73	17
H39A	7224.4	2783.64	1285.27	40
H39B	8258.51	3905.79	1293.94	40
H39C	5825.88	4103.83	1396.43	40

H40A	676.37	5650.92	3140.9	20
H40B	2177.95	6561.67	2957.59	20
H41A	586.96	8680.78	2243.53	41
H41B	530.64	9251.59	1296.56	41
H41C	2592.79	8367.97	1671.23	41
H42A	-3012.13	6753.35	1383.04	38
H42B	-3270.73	8189.72	1359.37	38
H42C	-2823.72	7303.9	2208.5	38
H44A	3489.41	7713.39	215.78	61
H44B	3678.73	6703.45	-384.08	61
H44C	4089.96	6296.56	574.7	61
H45A	-1743.59	7904.45	-218.12	41
H45B	175.47	7834.22	-843.09	41
H45C	-166.67	8736.94	-161.68	41
H46A	1320.42	5050.37	586.63	57
H46B	1136.68	5523.47	-375.45	57
H46C	-860.55	5741.7	209.55	57

Experimental

Single crystals of $C_{23}H_{36}O_7Si$ [B1794_0m]. A suitable crystal was selected and measured on a Bruker D8 Venture diffractometer. The crystal was kept at 100.0 K during data collection. Using Olex2,[29] the structure was solved with the ShelXT[30] structure solution program using Intrinsic Phasing and refined with the XL[31] refinement package using Least Squares minimisation.

Crystal structure determination of [B1794_0m]

Crystal Data for $C_{23}H_{36}O_7Si$ (M =452.61 g/mol): triclinic, space group P1 (no. 1), a = 6.7298(14) Å, b = 11.408(2) Å, c = 16.469(4) Å, α = 81.756(10)°, β = 86.847(10)°, γ = 75.985(9)°, V = 1213.8(4) Å3, Z = 2, T = 100.0 K, μ(MoKα) = 0.136 mm-1, $Dcalc$ = 1.238 g/cm^3, 60460 reflections measured (4.188° ≤ 2Θ ≤ 61.292°), 14864 unique (R_{int} = 0.0327, R_{sigma} = 0.0290) which were used in all calculations. The final R_1 was 0.0469 (I > 2σ(I)) and wR_2 was 0.1273 (all data).

Refinement model description

Number of restraints - 3, number of constraints - unknown.

Details:

1. Fixed Uiso

At 1.2 times of:

All C(H) groups, All C(H,H) groups

At 1.5 times of:

All C(H,H,H) groups

2.a Ternary CH refined with riding coordinates:

C7(H7), C8(H8), C11(H11), C12(H12), C13(H13), C14(H14), C15(H15), C30(H30), C31(H31), C34(H34), C35(H35), C36(H36), C37(H37), C38(H38)

2.b Secondary CH2 refined with riding coordinates:

C17(H17A,H17B), C40(H40A,H40B)

2.c Aromatic/amide H refined with riding coordinates:

C2(H2), C3(H3), C4(H4A), C5(H5), C9(H9), C10(H10), C25(H25), C26(H26), C27(H27), C28(H28), C32(H32), C33(H33)

2.d Idealised Me refined as rotating group:

C16(H16A,H16B,H16C), C18(H18A,H18B,H18C), C19(H19A,H19B,H19C), C21(H21A,H21B, H21C), C22(H22A,H22B,H22C), C23(H23A,H23B,H23C), C39(H39A,H39B,H39C), C41(H41A, H41B,H41C), C42(H42A,H42B,H42C), C44(H44A,H44B,H44C), C45(H45A,H45B,H45C), C46(H46A,H46B,H46C)

(2) Crystal data for 17l (Crystal identifier cu_B1969_0m): CCDC Deposition Number is 2097739, Unit Cell Parameters: a 12.9698(3) b 4.94080(10) c 13.0387(3) P21.

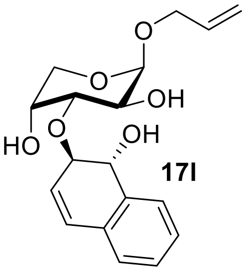

17l

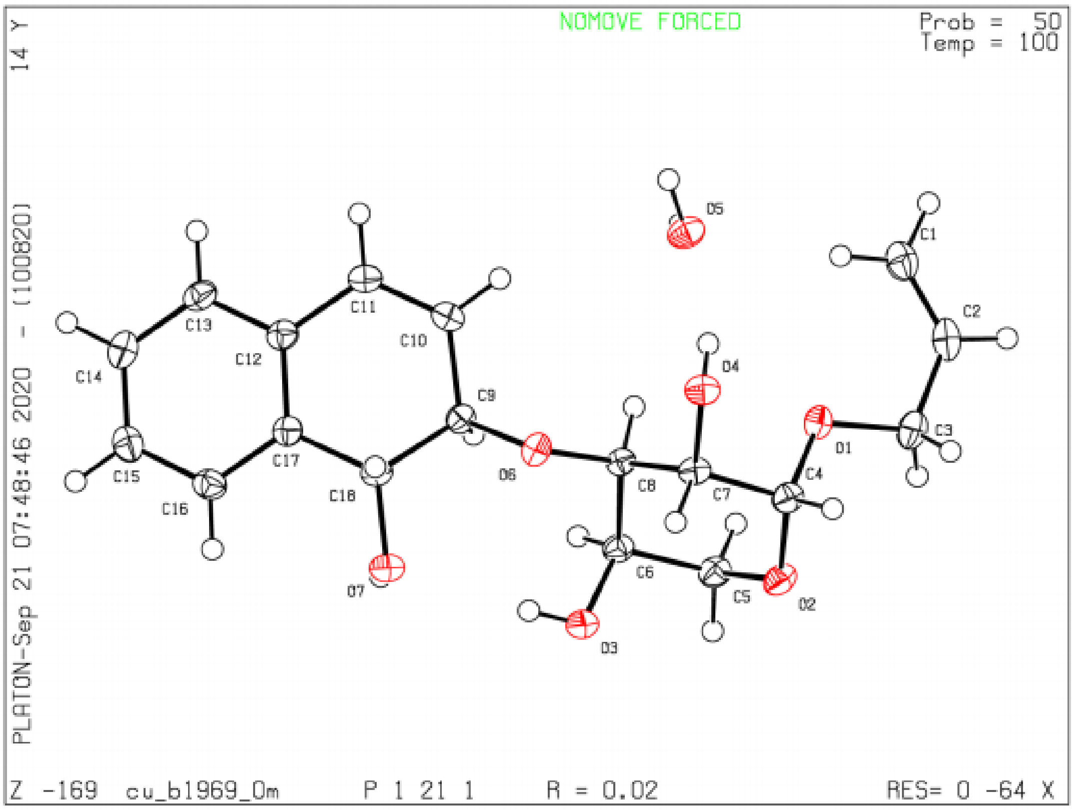

Supplementary Table 14: Crystal data and structure refinement for cu_B1969_0m.

Identification code	cu_B1969_0m
Empirical formula	C18H24O7
Formula weight	352.37
Temperature/K	100.0
Crystal system	monoclinic
Space group	P21
a/Å	12.9698(3)
b/Å	4.94080(10)
c/Å	13.0387(3)

α/°	90
β/°	90.5860(10)
γ/°	90
Volume/Å3	835.49(3)
Z	2
ρ_{calc}g/cm3	1.401
μ/mm^{-1}	0.901
F(000)	376.0
Crystal size/mm^3	0.546 × 0.148 × 0.061
Radiation	CuKα (λ = 1.54178)
2Θ range for data collection/°	6.78 to 134.856
Index ranges	-15 ≤ h ≤ 14, -5 ≤ k ≤ 5, -15 ≤ l ≤ 15
Reflections collected	16135
Independent reflections	2966 [R_{int} = 0.0190, R_{sigma} = 0.0129]
Data/restraints/parameters	2966/1/251
Goodness-of-fit on F^2	1.054
Final R indexes [I>=2σ (I)]	R_1 = 0.0228, wR_2 = 0.0575
Final R indexes [all data]	R_1 = 0.0231, wR_2 = 0.0580
Largest diff. peak/hole / e Å^{-3}	0.14/-0.14
Flack parameter	0.05(4)

Supplementary Table 15: Fractional Atomic Coordinates (×10^4) and Equivalent Isotropic Displacement Parameters (Å^2×10^3) for cu_B1969_0m. U_{eq} is defined as 1/3 of of the trace of the orthogonalised U_{IJ} tensor.

Atom	x	y	z	U(eq)
O6	5282.4(8)	7043(2)	2491.9(9)	18.8(3)
O1	2164.5(8)	5956(3)	3309.9(9)	21.3(3)
O7	6482.1(9)	6989(3)	685.4(9)	22.4(3)
O3	4248.5(10)	8133(3)	685.6(9)	24.2(3)
O4	3868.2(9)	8915(3)	3956.4(9)	20.7(3)
O2	2219.2(9)	7966(3)	1678.6(9)	22.5(3)
O5	4109.5(10)	3981(3)	4925.7(10)	23.8(3)
C7	3621.1(12)	8585(3)	2899.2(12)	17.7(3)
C8	4225.3(12)	6306(3)	2413.2(12)	16.5(3)
C12	8041.1(12)	2698(4)	2468.9(13)	19.4(3)

C4	2460.7(13)	8225(4)	2735.3(13)	20.2(4)
C13	8924.0(13)	1093(4)	2451.8(14)	22.8(4)
C18	6860.9(12)	6162(4)	1664.8(13)	18.4(3)
C14	9578.5(13)	1136(4)	1614.6(14)	24.4(4)
C17	7805.2(12)	4354(3)	1620.6(13)	18.0(4)
C9	6003.1(12)	4895(4)	2289.4(13)	17.9(3)
C5	2727.3(13)	5715(4)	1204.0(13)	21.2(4)
C6	3893.9(12)	5909(4)	1289.5(12)	19.1(3)
C11	7332.8(13)	2636(4)	3342.2(13)	22.2(4)
C16	8471.1(13)	4413(4)	792.2(14)	22.3(4)
C10	6392.3(13)	3670(4)	3270.6(13)	21.1(4)
C1	1483.0(14)	2546(4)	4846.8(15)	27.5(4)
C15	9349.7(13)	2799(4)	790.5(14)	25.6(4)
C2	820.2(13)	3702(4)	4217.8(15)	26.0(4)
C3	1072.4(13)	5670(4)	3395.3(15)	26.3(4)

Supplementary Table 16: Anisotropic Displacement Parameters ($Å^2×10^3$) for cu_B1969_0m. The Anisotropic displacement factor exponent takes the form: $-2\pi^2[h^2a^{*2}U_{11}+2hka^*b^*U_{12}+…]$.

Atom	U11	U22	U33	U23	U13	U12
O6	17.0(5)	15.3(6)	24.2(6)	-3.0(5)	-1.8(4)	-0.3(5)
O1	16.8(5)	22.8(6)	24.2(6)	-0.8(5)	-0.2(4)	0.0(5)
O7	26.8(6)	21.8(6)	18.6(6)	2.5(5)	-2.3(5)	1.1(5)
O3	22.5(6)	29.5(7)	20.6(6)	5.8(5)	-1.2(5)	1.4(5)
O4	26.5(6)	18.1(6)	17.3(6)	-3.4(6)	-2.9(5)	-1.1(5)
O2	22.6(6)	23.9(6)	20.9(6)	-3.1(5)	-5.0(5)	5.3(5)
O5	27.7(6)	20.2(7)	23.3(7)	-1.6(6)	-5.7(5)	1.6(5)
C7	22.5(8)	14.6(9)	15.8(8)	-1.9(7)	-2.8(6)	-0.4(7)
C8	17.0(7)	15.1(9)	17.4(8)	0.1(7)	-2.1(6)	-1.2(6)
C12	18.6(7)	19.8(9)	19.7(8)	-1.1(7)	-3.8(6)	-3.4(7)
C4	23.1(8)	18.9(9)	18.7(8)	-3.0(7)	-1.2(6)	4.0(7)
C13	20.9(8)	23.8(9)	23.4(9)	2.2(8)	-5.6(6)	0.0(7)
C18	20.9(8)	16.7(8)	17.6(8)	0.8(7)	-2.9(6)	-1.1(7)
C14	18.7(8)	24.8(10)	29.7(10)	-4.3(8)	-1.5(7)	1.5(7)
C17	17.3(7)	17.4(9)	19.2(8)	-1.4(7)	-2.1(6)	-3.5(6)
C9	18.5(7)	16.0(8)	19.2(8)	-1.2(7)	-0.9(6)	1.2(6)

C5	23.1(8)	22.0(9)	18.3(8)	-3.7(8)	-2.8(6)	1.8(7)
C6	21.4(8)	19.0(8)	16.9(8)	-0.9(7)	-2.0(6)	1.9(7)
C11	25.2(8)	23.7(9)	17.5(8)	2.1(8)	-2.6(6)	0.4(7)
C16	23.8(8)	22.1(9)	21.0(9)	2.6(7)	-0.2(7)	-3.1(7)
C10	24.1(8)	21.6(9)	17.7(8)	1.5(7)	1.7(6)	-0.8(7)
C1	23.5(9)	30.2(11)	28.9(10)	-3.0(9)	3.5(7)	-4.6(8)
C15	22.0(8)	28.9(10)	26.1(9)	-1.5(9)	3.5(7)	-2.5(8)
C2	18.3(8)	27.9(10)	32.0(10)	-8.6(8)	5.7(7)	-2.5(7)
C3	17.4(8)	29.5(10)	32.1(10)	-2.8(8)	-2.5(7)	-0.6(7)

Supplementary Table 17: Bond Lengths for cu_B1969_0m.

Atom	Atom	Length/Å	Atom	Atom	Length/Å
O6	C8	1.4213(19)	C12	C17	1.407(2)
O6	C9	1.441(2)	C12	C11	1.471(2)
O1	C4	1.404(2)	C13	C14	1.390(2)
O1	C3	1.429(2)	C18	C17	1.518(2)
O7	C18	1.423(2)	C18	C9	1.520(2)
O3	C6	1.430(2)	C14	C15	1.382(3)
O4	C7	1.421(2)	C17	C16	1.390(2)
O2	C4	1.416(2)	C9	C10	1.498(2)
O2	C5	1.436(2)	C5	C6	1.519(2)
C7	C8	1.515(2)	C11	C10	1.325(2)
C7	C4	1.528(2)	C16	C15	1.391(3)
C8	C6	1.535(2)	C1	C2	1.313(3)
C12	C13	1.393(2)	C2	C3	1.486(3)

Supplementary Table 18: Bond Angles for cu_B1969_0m.

Atom	Atom	Atom	Angle/˚	Atom	Atom	Atom	Angle/˚
C8	O6	C9	115.15(13)	C17	C18	C9	111.91(14)
C4	O1	C3	113.42(13)	C15	C14	C13	119.46(16)
C4	O2	C5	113.04(13)	C12	C17	C18	118.78(14)
O4	C7	C8	112.24(13)	C16	C17	C12	119.43(15)
O4	C7	C4	111.16(13)	C16	C17	C18	121.68(15)
C8	C7	C4	111.59(13)	O6	C9	C18	105.93(14)

O6	C8	C7	106.38(13)	O6	C9	C10	110.78(13)
O6	C8	C6	111.23(13)	C10	C9	C18	112.45(13)
C7	C8	C6	110.67(13)	O2	C5	C6	112.37(14)
C13	C12	C17	119.38(15)	O3	C6	C8	109.81(14)
C13	C12	C11	121.36(16)	O3	C6	C5	109.50(14)
C17	C12	C11	119.23(15)	C5	C6	C8	110.30(13)
O1	C4	O2	112.84(14)	C10	C11	C12	121.29(16)
O1	C4	C7	107.03(13)	C17	C16	C15	120.31(16)
O2	C4	C7	110.76(13)	C11	C10	C9	121.18(15)
C14	C13	C12	120.82(17)	C14	C15	C16	120.59(16)
O7	C18	C17	113.96(13)	C1	C2	C3	125.99(16)
O7	C18	C9	110.55(13)	O1	C3	C2	110.29(14)

Supplementary Table 19: Hydrogen Bonds for cu_B1969_0m.

D	H	A	d(D-H)/Å		d(H-A)/Å		d(D-A)/Å		D-H-A/°
O7	H7	O31	0.84	1.96	2.7728(18)		161.3		
O4	H4	O5	0.83(3)	1.95(3)	2.762(2)		165(2)		
O3	H3	O7	0.82(2)	2.15(3)	2.9516(17)		166(3)		
O5	H5C	O42	0.88(3)	2.26(3)	2.9878(18)		140(2)		
O5	H5D	O43	0.91(4)	1.92(4)	2.8197(19)		169(3)		

^{1}1-X,-1/2+Y,-Z; ^{2}1-X,-1/2+Y,1-Z; ^{3}+X,-1+Y,+Z

Supplementary Table 20: Torsion Angles for cu_B1969_0m.

A	B	C	D	Angle/˚	A	B	C	D	Angle/˚	
O6	C8	C6	O3	-47.25(18)		C4	C7	C8	C6	51.31(18)
O6	C8	C6	C5	-168.02(14)		C13	C12	C17	C18	-177.96(15)
O6	C9	C10	C11	-149.07(17)		C13	C12	C17	C16	-1.7(3)
O7	C18	C17	C12	-158.85(15)		C13	C12	C11	C10	-164.83(18)
O7	C18	C17	C16	25.0(2)	C13	C14	C15	C16	-0.2(3)	
O7	C18	C9	O6	-66.29(17)		C18	C17	C16	C15	177.74(16)
O7	C18	C9	C10	172.57(14)		C18	C9	C10	C11	-30.7(2)
O4	C7	C8	O6	-62.22(16)		C17	C12	C13	C14	0.9(3)
O4	C7	C8	C6	176.84(13)		C17	C12	C11	C10	13.3(3)
O4	C7	C4	O1	-58.09(17)		C17	C18	C9	O6	165.52(13)

O4	C7	C4	O2	178.53(14)	C17	C18	C9	C10	44.38(19)
O2	C5	C6	O3	-67.54(18)	C17	C16	C15	C14	-0.6(3)
O2	C5	C6	C8	53.42(19)	C9	O6	C8	C7	166.43(13)
C7	C8	C6	O3	70.78(16)	C9	O6	C8	C6	-72.98(17)
C7	C8	C6	C5	-49.99(19)	C9	C18	C17	C12	-32.5(2)
C8	O6	C9	C18	139.39(13)	C9	C18	C17	C16	151.33(15)
C8	O6	C9	C10	-98.40(16)	C5	O2	C4	O1	-61.01(16)
C8	C7	C4	O1	68.03(17)	C5	O2	C4	C7	58.95(18)
C8	C7	C4	O2	-55.35(18)	C11	C12	C13	C14	178.94(16)
C12	C13	C14	C15	0.1(3)	C11	C12	C17	C18	3.9(2)
C12	C17	C16	C15	1.6(3)	C11	C12	C17	C16	-179.82(15)
C12	C11	C10	C9	1.3(3)	C1	C2	C3	O1	5.6(3)
C4	O1	C3	C2	-165.36(15)	C3	O1	C4	O2	-69.44(17)
C4	O2	C5	C6	-59.15(18)	C3	O1	C4	C7	168.48(14)
C4	C7	C8	O6	172.25(13)					

Supplementary Table 21: Hydrogen Atom Coordinates (Å×10[4]) and Isotropic Displacement Parameters (Å[2]×10[3]) for cu_B1969_0m.

Atom	x	y	z	U(eq)
H7	6378.42	5618.75	317.11	34
H7A	3822.75	10295.4	2543.49	21
H8	4106.54	4592.35	2802.83	20
H4A	2099.34	9855.81	3009.41	24
H13	9080.52	-46.21	3019.5	27
H18	7076.42	7845.3	2034.92	22
H14	10178.24	32.93	1608.79	29
H9	5652.73	3464.66	1869.86	21
H5A	2524.64	5638.34	470.47	25
H5B	2496.03	4017	1532.4	25
H6	4205.2	4197.55	1025.16	23
H11	7554.03	1839.09	3969.94	27
H16	8325.31	5561.11	224.55	27
H10	5956.78	3633.07	3851.9	25
H15	9796.49	2839.53	217.91	31
H2	114.38	3239.1	4295.56	31

H3A	779.51	5033.09	2733.97	32
H3B	760.98	7447.83	3554.85	32
H4	3822(18)	7440(60)	4264(19)	39(7)
H3	4880(19)	8090(50)	713(17)	37(6)
H5C	4480(20)	3790(70)	5490(20)	60(9)
H5D	4030(20)	2260(70)	4700(20)	57(8)
H1A	2206(17)	2830(50)	4813(16)	35(6)
H1B	1258(19)	1180(60)	5367(19)	44(7)

Experimental

Single crystals of $C_{18}H_{24}O_7$ [cu_B1969_0m]. A suitable crystal was selected and measured on a Bruker D8 Venture diffractometer. The crystal was kept at 100.0 K during data collection. Using Olex2,[29] the structure was solved with the XT[30] structure solution program using Intrinsic Phasing and refined with the XL[31] refinement package using Least Squares minimisation.

Crystal structure determination of [cu_B1969_0m]

Crystal Data for $C_{18}H_{24}O_7$ (M =352.37 g/mol): monoclinic, space group P21 (no. 4), a = 12.9698(3) Å, b = 4.94080(10) Å, c = 13.0387(3) Å, β = 90.5860(10)°, V = 835.49(3) Å3, Z = 2, T = 100.0 K, μ(CuKα) = 0.901 mm-1, $Dcalc$ = 1.401 g/cm^3, 16135 reflections measured (6.78° ≤ 2Θ ≤ 134.856°), 2966 unique (R_{int} = 0.0190, R_{sigma} = 0.0129) which were used in all calculations. The final R_1 was 0.0228 (I > 2σ(I)) and wR_2 was 0.0580 (all data).

Refinement model description

Number of restraints - 1, number of constraints - unknown.

Details:

1. Fixed Uiso

At 1.2 times of:

All C(H) groups, All C(H,H) groups

At 1.5 times of:

All O(H) groups

2.a Ternary CH refined with riding coordinates:

C7(H7A), C8(H8), C4(H4A), C18(H18), C9(H9), C6(H6)

2.b Secondary CH2 refined with riding coordinates:

C5(H5A,H5B), C3(H3A,H3B)

2.c Aromatic/amide H refined with riding coordinates:

C13(H13), C14(H14), C11(H11), C16(H16), C10(H10), C15(H15), C2(H2)

2.d Idealised tetrahedral OH refined as rotating group:

O7(H7)

(3) Crystal data for 17n (Crystal identifier cu_B1997_0m): CCDC Deposition Number is 2097740**,** Unit Cell Parameters: a 28.4791(12) b 4.6584(2) c 12.9341(5) C2.

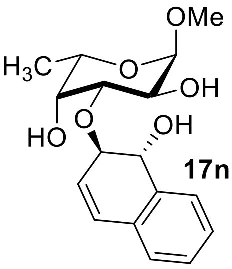

17n

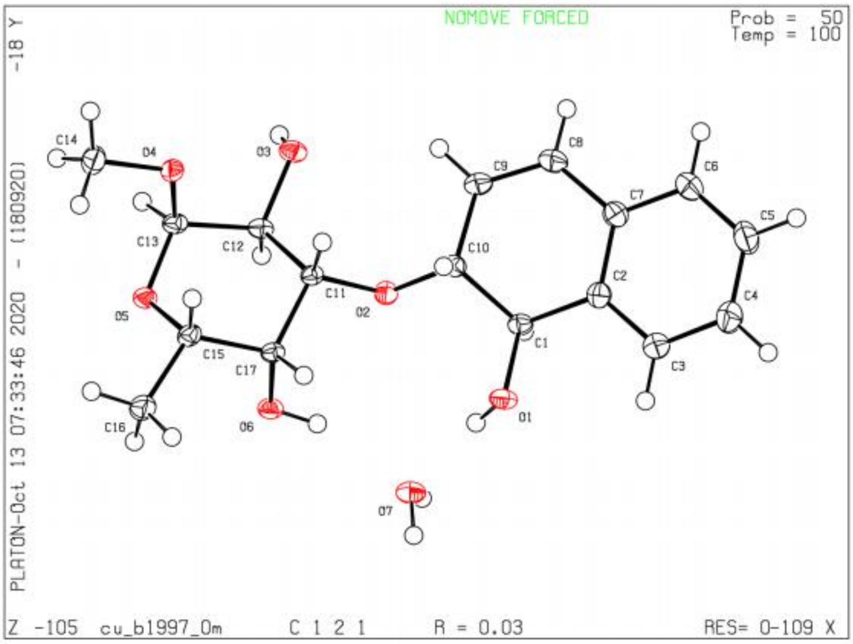

Supplementary Table 22: Crystal data and structure refinement for cu_B1997_0m.

Identification code	cu_B1997_0m
Empirical formula	$C_{17}H_{24}O_7$
Formula weight	340.36
Temperature/K	100.0
Crystal system	monoclinic
Space group	C2
a/Å	28.4791(12)
b/Å	4.6584(2)
c/Å	12.9341(5)
α/°	90
β/°	99.769(2)
γ/°	90

Volume/Å^3	1691.05(12)
Z	4
ρ_{calc}g/cm^3	1.337
μ/mm^{-1}	0.869
F(000)	728.0
Crystal size/mm^3	0.788 × 0.149 × 0.116
Radiation	CuKα (λ = 1.54178)
2Θ range for data collection/°	6.298 to 158.848
Index ranges	-36 ≤ h ≤ 35, -5 ≤ k ≤ 5, -16 ≤ l ≤ 16
Reflections collected	29300
Independent reflections	3596 [R_{int} = 0.0275, R_{sigma} = 0.0138]
Data/restraints/parameters	3596/1/239
Goodness-of-fit on F^2	1.049
Final R indexes [I>=2σ (I)]	R_1 = 0.0259, wR_2 = 0.0685
Final R indexes [all data]	R_1 = 0.0260, wR_2 = 0.0685
Largest diff. peak/hole / e Å^{-3}	0.15/-0.24
Flack parameter	-0.02(2)

Supplementary Table 23: Fractional Atomic Coordinates (×10^4) and Equivalent Isotropic Displacement Parameters (Å^2×10^3) for cu_B1997_0m. U_{eq} is defined as 1/3 of of the trace of the orthogonalised U_{IJ} tensor.

Atom	x	y	z	U(eq)
O1	5543.5(4)	4637(3)	7199.8(8)	18.3(2)
O2	6327.2(3)	3171(2)	6184.7(8)	15.9(2)
O3	7233.1(3)	3347(3)	5428.0(8)	18.0(2)
O4	6971.4(4)	5394(2)	3469.7(8)	17.1(2)
O5	6364.1(3)	2012(2)	3047.4(8)	16.6(2)
O6	5739.4(3)	637(2)	4505.6(8)	16.8(2)
C1	6029.0(5)	4650(3)	7706.1(11)	15.8(3)
C2	6086.9(5)	6693(3)	8625.6(11)	17.9(3)
C3	5708.3(6)	7443(4)	9120.0(13)	24.3(3)
C4	5779.6(6)	9221(5)	9997.7(13)	31.0(4)
C5	6231.2(6)	10245(4)	10393.8(13)	29.5(4)
C6	6610.9(6)	9499(4)	9903.8(12)	26.3(3)
C7	6543.8(5)	7742(3)	9019.0(11)	20.3(3)

C8	6936.2(5)	7027(5)	8460.3(13)	27.5(4)
C9	6854.6(5)	5961(4)	7492.5(13)	24.7(3)
C10	6353.6(5)	5467(3)	6930.9(11)	15.7(3)
C11	6386.3(5)	3997(3)	5148.2(10)	13.7(3)
C12	6799.7(5)	2320(3)	4832.0(11)	15.0(3)
C13	6810.6(5)	2605(3)	3656.4(11)	15.9(3)
C14	7007.9(6)	5810(4)	2391.4(12)	23.6(3)
C15	5995.6(5)	3928(3)	3273.0(11)	16.2(3)
C16	5562.5(5)	3353(4)	2443.8(12)	24.2(3)
C17	5917.0(5)	3479(3)	4394.8(11)	14.0(3)
O7	5262.1(4)	-343(3)	6215.6(8)	19.2(2)

Supplementary Table 24: Anisotropic Displacement Parameters (Å^2×10^3) for cu_B1997_0m. The Anisotropic displacement factor exponent takes the form: -2π^2[h^{2}a*^{2}U$_{11}$+2hka*b*U$_{12}$+…].

Atom	U11	U22	U33	U23	U13	U12	
O1	12.2(5)	17.3(5)	24.6(5)	-2.9(4)	1.1(4)	-1.3(4)	
O2	17.6(5)	13.4(5)	16.7(5)	0.1(4)	3.4(4)	-1.1(4)	
O3	11.8(5)	18.0(5)	23.0(5)	-1.1(4)	-0.3(4)	1.7(4)	
O4	18.1(5)	17.1(5)	17.2(5)	-2.0(4)	5.7(4)	-4.4(4)	
O5	12.3(4)	17.3(5)	20.3(5)	-5.5(4)	3.4(4)	-0.6(4)	
O6	15.0(4)	15.4(5)	20.0(5)	-0.9(4)	3.1(4)	-3.8(4)	
C1	12.3(6)	16.9(7)	17.7(6)	1.7(6)	1.2(5)	0.4(5)	
C2	19.6(7)	17.6(7)	16.1(6)	2.9(6)	2.3(5)	0.9(6)	
C3	19.4(7)	30.4(9)	23.3(7)	-3.6(7)	4.3(6)	-1.2(6)	
C4	27.5(8)	41.3(11)		25.5(8)	-8.5(8)	8.1(6)	1.6(8)
C5	32.0(8)	35.5(10)		20.3(7)	-7.9(7)	2.1(6)	0.5(7)
C6	22.9(7)	34.1(9)	20.4(7)	-3.2(7)	-1.0(6)	-1.9(7)	
C7	19.2(7)	23.9(8)	16.9(6)	1.3(6)	0.5(5)	0.2(6)	
C8	14.5(7)	41.3(10)		25.6(8)	-6.5(8)	-0.2(6)	-3.2(7)
C9	13.1(6)	35.0(9)	26.4(8)	-5.4(7)	4.1(6)	-2.5(6)	
C10	15.0(6)	15.6(7)	16.2(6)	-2.0(5)	2.0(5)	-1.3(5)	
C11	12.5(6)	12.3(7)	16.5(6)	-0.5(5)	3.3(5)	-0.7(5)	
C12	11.3(6)	13.1(7)	20.1(7)	-0.6(5)	1.5(5)	-0.2(5)	
C13	11.9(6)	14.1(7)	22.0(7)	-3.7(5)	3.7(5)	0.3(5)	
C14	27.7(7)	25.4(8)	19.8(7)	-2.3(6)	9.5(6)	-4.2(7)	

C15	12.6(6)	18.2(7)	18.3(6)	0.0(5)	4.2(5)	2.4(5)
C16	14.9(6)	38.2(9)	19.2(7)	0.8(7)	2.0(5)	1.0(7)
C17	11.3(6)	12.6(6)	18.2(6)	-0.4(5)	3.0(5)	0.1(5)
O7	13.4(5)	18.7(6)	24.6(5)	2.5(5)	0.5(4)	-0.3(4)

Supplementary Table 25: Bond Lengths for cu_B1997_0m.

Atom	Atom	Length/Å	Atom	Atom	Length/Å
O1	C1	1.4260(16)	C3	C4	1.392(2)
O2	C10	1.4338(17)	C4	C5	1.386(2)
O2	C11	1.4317(16)	C5	C6	1.387(2)
O3	C12	1.4225(16)	C6	C7	1.393(2)
O4	C13	1.4123(18)	C7	C8	1.468(2)
O4	C14	1.4291(17)	C8	C9	1.330(2)
O5	C13	1.4048(17)	C9	C10	1.5050(19)
O5	C15	1.4447(17)	C11	C12	1.5257(18)
O6	C17	1.4333(18)	C11	C17	1.5334(18)
C1	C2	1.510(2)	C12	C13	1.5319(19)
C1	C10	1.5229(18)	C15	C16	1.5148(19)
C2	C3	1.388(2)	C15	C17	1.5196(19)
C2	C7	1.402(2)			

Supplementary Table 26: Bond Angles for cu_B1997_0m.

Atom	Atom	Atom	Angle/˚	Atom	Atom	Atom	Angle/˚	
C11	O2	C10	115.40(11)		O2	C10	C1	106.77(11)
C13	O4	C14	111.77(11)		O2	C10	C9	112.11(12)
C13	O5	C15	112.63(11)		C9	C10	C1	110.63(11)
O1	C1	C2	109.40(11)		O2	C11	C12	109.40(11)
O1	C1	C10	110.39(11)		O2	C11	C17	108.94(10)
C2	C1	C10	111.32(12)		C12	C11	C17	112.22(11)
C3	C2	C1	122.12(13)		O3	C12	C11	108.70(11)
C3	C2	C7	119.37(14)		O3	C12	C13	110.30(11)
C7	C2	C1	118.43(13)		C11	C12	C13	111.61(11)
C2	C3	C4	120.54(15)		O4	C13	C12	108.14(11)
C5	C4	C3	120.28(16)		O5	C13	O4	111.95(12)

C4	C5	C6	119.42(16)	O5	C13	C12	111.76(11)
C5	C6	C7	120.89(15)	O5	C15	C16	106.26(12)
C2	C7	C8	118.63(14)	O5	C15	C17	109.73(11)
C6	C7	C2	119.50(14)	C16	C15	C17	114.69(12)
C6	C7	C8	121.84(14)	O6	C17	C11	111.47(11)
C9	C8	C7	121.46(14)	O6	C17	C15	109.52(11)
C8	C9	C10	120.75(13)	C15	C17	C11	109.36(10)

Supplementary Table 27: Hydrogen Bonds for cu_B1997_0m.

D	H	A	d(D-H)/Å	d(H-A)/Å	d(D-A)/Å	D-H-A/°
O7	H7A	O11	0.84(3)	1.88(3)	2.7174(17)	174(2)
O7	H7B	O62	0.81(3)	2.09(3)	2.8828(15)	169(3)

[1]+X,-1+Y,+Z; [2]1-X,+Y,1-Z

Supplementary Table 28: Torsion Angles for cu_B1997_0m.

A	B	C	D	Angle/˚	A	B	C	D	Angle/˚
O1	C1	C2	C3	23.5(2)	C6	C7	C8	C9	-163.44(19)
O1	C1	C2	C7	-159.65(13)	C7	C2	C3	C4	0.0(3)
O1	C1	C10	O2	-67.13(14)	C7	C8	C9	C10	0.4(3)
O1	C1	C10	C9	170.64(13)	C8	C9	C10	O2	-151.59(18)
O2	C11	C12	O3	-70.75(14)	C8	C9	C10	C1	-32.5(2)
O2	C11	C12	C13	167.38(11)	C10	O2	C11	C12	123.08(12)
O2	C11	C17	O6	-50.49(14)	C10	O2	C11	C17	-113.91(12)
O2	C11	C17	C15	-171.74(11)	C10	C1	C2	C3	145.81(15)
O3	C12	C13	O4	-47.55(15)	C10	C1	C2	C7	-37.37(18)
O3	C12	C13	O5	-171.21(12)	C11	O2	C10	C1	148.91(11)
O5	C15	C17	O6	-64.45(13)	C11	O2	C10	C9	-89.79(14)
O5	C15	C17	C11	57.97(15)	C11	C12	C13	O4	73.39(13)
C1	C2	C3	C4	176.82(16)	C11	C12	C13	O5	-50.27(15)
C1	C2	C7	C6	-176.36(15)	C12	C11	C17	O6	70.81(14)
C1	C2	C7	C8	5.7(2)	C12	C11	C17	C15	-50.44(15)
C2	C1	C10	O2	171.17(11)	C13	O5	C15	C16	170.85(12)
C2	C1	C10	C9	48.94(17)	C13	O5	C15	C17	-64.63(14)
C2	C3	C4	C5	-0.5(3)	C14	O4	C13	O5	-58.46(14)

C2	C7	C8	C9	14.5(3)	C14	O4	C13	C12	177.99(11)
C3	C2	C7	C6	0.5(2)	C15	O5	C13	O4	-61.39(14)
C3	C2	C7	C8	-177.44(16)	C15	O5	C13	C12	60.10(15)
C3	C4	C5	C6	0.4(3)	C16	C15	C17	O6	55.03(16)
C4	C5	C6	C7	0.1(3)	C16	C15	C17	C11	177.45(13)
C5	C6	C7	C2	-0.6(3)	C17	C11	C12	O3	168.21(11)
C5	C6	C7	C8	177.28(18)	C17	C11	C12	C13	46.34(15)

Supplementary Table 29: Hydrogen Atom Coordinates (Å×10^4) and Isotropic Displacement Parameters (Å^2×10^3) for cu_B1997_0m.

Atom	x	y	z	U(eq)
H1A	6115.38	2674.03	7975.27	19
H3A	5398.02	6737.83	8857.08	29
H4	5517.63	9734.35	10326.51	37
H5	6280.33	11447.5	10995.73	35
H6A	6920.93	10194.92	10175.04	32
H8	7255.41	7331.78	8797.69	33
H9	7115.6	5507.55	7151.79	30
H10	6231.77	7268.05	6558.38	19
H11	6463.14	6091.48	5149.3	16
H12	6762.52	245.86	5000.73	18
H13	7046.09	1190.3	3466.68	19
H14A	6691.5	6216.84	1988.03	35
H14B	7136.18	4068.76	2117.99	35
H14C	7220.92	7429.32	2328.9	35
H15	6101.81	5951.66	3196.56	19
H16A	5635.32	3840.22	1750.97	36
H16B	5295.34	4529.99	2585.69	36
H16C	5476.65	1318.39	2458.12	36
H17	5675.17	4896.64	4552.9	17
H7A	5329(9)	-1900(60)	6540(20)	38(6)
H7B	4975(10)	-210(60)	6080(20)	44(7)
H3	7429(9)	2010(60)	5468(18)	37(6)
H6	5657(9)	420(60)	5110(20)	41(6)

H1	5490(9)	3110(70)	6930(20)	44(7)

Experimental

Single crystals of $C_{17}H_{24}O_7$ [cu_B1997_0m]. A suitable crystal was selected and measured on a Bruker D8 Venture diffractometer. The crystal was kept at 100.0 K during data collection. Using Olex2,[29] the structure was solved with the XT[30] structure solution program using Intrinsic Phasing and refined with the XL[31] refinement package using Least Squares minimisation.

Crystal structure determination of [cu_B1997_0m]

Crystal Data for $C_{17}H_{24}O_7$ (M =340.36 g/mol): monoclinic, space group C2 (no. 5), *a* = 28.4791(12) Å, *b* = 4.6584(2) Å, *c* = 12.9341(5) Å, β = 99.769(2)°, *V* = 1691.05(12) Å3, *Z* = 4, T = 100.0 K, μ(CuKα) = 0.869 mm-1, *Dcalc* = 1.337 g/cm^3, 29300 reflections measured (6.298° ≤ 2Θ ≤ 158.848°), 3596 unique (R_{int} = 0.0275, R_{sigma} = 0.0138) which were used in all calculations. The final R_1 was 0.0259 (I > 2σ(I)) and wR_2 was 0.0685 (all data).

Refinement model description

Number of restraints - 1, number of constraints - unknown.

Details:

1. Fixed Uiso

At 1.2 times of:

All C(H) groups

At 1.5 times of:

All C(H,H,H) groups

2.a Ternary CH refined with riding coordinates:

C1(H1A), C10(H10), C11(H11), C12(H12), C13(H13), C15(H15), C17(H17)

2.b Aromatic/amide H refined with riding coordinates:

C3(H3A), C4(H4), C5(H5), C6(H6A), C8(H8), C9(H9)

2.c Idealised Me refined as rotating group:

C14(H14A,H14B,H14C), C16(H16A,H16B,H16C)

Structural elucidation with the help of HMBC:

The HMBC (Heteronuclear Multiple Bond Correlation) is one of the most useful 2D-NMR techniques to understand correlations between carbons and protons that are separated by two to three bonds. HMBC provided us a facile NMR method to elucidate the identity of our major/minor regioisomers, which corresponded well with crystal data and VCD analysis. For instance in **17a**, which was also crossed

checked with X-ray crystallography and VCD, we observed bidirectional three bond HMBC cross-peaks between H_3 on the carbohydrate polyol and the C8 carbon of dihydronaphthalene moiety as well as between the C3-carbon of the carbohydrate polyol with H_8 of dihydronaphthalene moiety. This provide a further line of support for the C3 regioisomeric elucidation. The elucidation for the products (**17v-za, 19v, 19za, 20v**) of the allylic substitution from allylic carbonates were also performed analogously. For ease of reading, we have circled the correlation peaks in HMBC used in our structural elucidation.

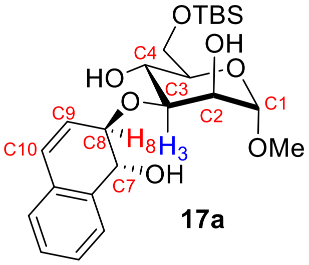

17a

Supplementary Notes

Supplementary Note 1: Compound **17h** is prepared according to the general procedure **A** with 5 mol% $Rh(cod)_2OTf$ catalyst, 30 mol% cyclohexyl vinyl boronic acid **26**, oxabicycle **16a** (0.4 mmol, 2 equiv.), and 0.2 mmol carbohydrate polyol **15h** at 50 ˚C for **48 h.**

Supplementary Note 2: Compound **19h** is prepared according to the general procedure **B** with 5 mol% $Rh(cod)_2OTf$ catalyst, 30 mol% cyclohexyl vinyl boronic acid **26**, oxabicycle **16a** (0.4 mmol, 2 equiv.), and 0.2 mmol carbohydrate polyol **15h** at 50 ˚C for **48 h.**

Supplementary Note 3: Compound **17m** is prepared according to the general procedure **B** with 5 mol% $Rh(cod)_2OTf$ catalyst, 30 mol% cyclohexyl vinyl boronic acid **26**, oxabicycle **16a** (0.4 mmol, 2 equiv.), and 0.2 mmol carbohydrate polyol **15m,** in **0.5 ml** THF at 50 ˚C for 24 h.

Supplementary Note 4: Compound **19m** is prepared according to the general procedure **A** with 5 mol% $Rh(cod)_2OTf$ catalyst, 30 mol% cyclohexyl vinyl boronic acid **26**, oxabicycle **16a** (0.4 mmol, 2 equiv.), and 0.2 mmol carbohydrate polyol **19m,** in **0.5 ml** THF at 50 ˚C for 24 h.

Supplementary Note 5: Compound **17n** is prepared according to the general procedure **A** with 5 mol% $Rh(cod)_2OTf$ catalyst, 30 mol% cyclohexyl vinyl boronic acid **26**, oxabicycle **16a** (0.4 mmol, 2 equiv.), and 0.2 mmol carbohydrate polyol **15n,** in **0.5 ml** THF at 50 ˚C for **12 h.**

Supplementary Note 6: Compound **17p** is prepared according to the general procedure **C** with 5 mol% $Rh(cod)_2OTf$ catalyst, 30 mol% borinic acid **27**, oxabicycle **16a** (0.3 mmol, 1.5 equiv.), and 0.2 mmol carbohydrate polyol **15p,** at 50 ˚C for 24 h.

Supplementary Note 7: Compound **19pa** is prepared according to the general procedure **C** with 5 mol% $Rh(cod)_2OTf$ catalyst, 30 mol% borinic acid **27**, oxabicycle **16a** (0.3 mmol, 1.5 equiv.), and 0.2 mmol carbohydrate polyol **15p,** at 50 ˚C for 24 h.

Supplementary Note 8: Compound **19p, 20p** are prepared according to the general procedure **D** with 5 mol% $Rh(cod)_2OTf$ catalyst, 30 mol% borinic acid **27**, oxabicycle **16a** (0.3 mmol, 1.5 equiv.), and 0.2 mmol carbohydrate polyol **15p,** at 50 ˚C for 24 h.

Supplementary Note 9: Compound **20pa** is prepared according to the general procedure **D** with 5 mol% $Rh(cod)_2OTf$ catalyst, 30 mol% borinic acid **27**, oxabicycle **16a** (0.3 mmol, 1.5 equiv.), and 0.2 mmol carbohydrate polyol **15p,** at 50 ˚C for 24 h.

Supplementary Note 10: Compound **17q** is prepared according to the general procedure **B** with 5 mol% $Rh(cod)_2OTf$ catalyst, 30 mol% cyclohexyl vinyl boronic acid **26**, oxabicycle **16a** (0.4 mmol, 2 equiv.), and 0.2 mmol carbohydrate polyol **15q,** in **0.5 ml** THF at 50 ˚C for 24 h.

Supplementary Note 11: Compound **18q** is prepared according to the general procedure **A** with 5 mol% Rh(cod)$_2$OTf catalyst, 30 mol% cyclohexyl vinyl boronic acid **26**, oxabicycle **16a** (0.4 mmol, 2 equiv.), and 0.2 mmol carbohydrate polyol **15q,** in 0.5 ml THF at 50 ˚C for 24 h.

Supplementary Note 12: Compound **19q** is prepared according to the general procedure **B** with 5 mol% Rh(cod)$_2$OTf catalyst, 30 mol% cyclohexyl vinyl boronic acid **26**, oxabicycle **16a** (0.4 mmol, 2 equiv.), and 0.2 mmol carbohydrate polyol **15q,** in 0.5 ml THF at 50 ˚C for 24 h.

Supplementary Note 13: In this manuscript, we define enantiocontrol as the selective oxidative addition of the chiral Rh(I) complex into specifically one of the prochiral *meso*-oxanorbornadiene bridgehead C-O bonds, hence desymmetrizing the meso substrate into only one of the two possible enantiomeric Rh(III) intermediates, leading up to two possible enantiomeric hydronapthalenes if an achiral nucleophile were to be employed. We distinguish this from the term diastereocontrol, where the reaction selectively proceeded through the outer sphere nucleophilic attack involving the Rh(I)/Rh(III) redox couple to yield selectively the *trans*-hydronapthalene, rather than the inner sphere isohypsic Rh(I) pathway which would generate instead the *cis*-hydronapthalene typical of the classical reactivity of boronic acids in Rh(I) catalysis. However it is pertinent to point out that in the former case, since the polyol boronate nucleophile arising from the boronic acid catalysis is chiral, the resulting stereoisomeric products should be broadly classified as diastereomeric with respect to each other, although the nature of diastereomers generated in this way is based on an initial enantioselective oxidative addition elementary step, resulting in chirality generation from a prochiral *meso*-substrate. Furthermore, the terms site-selectivity and regioselectivity are to be understood interchangably in this manuscript, as they are used synonymously in the literature.

Supplementary Note 14: We propose that the C3, C2 di-substituted side product arose from the nucleophilic attack of the boronic acid hemiester intermediate generated after the initial C3 monofunctionalization of the lyxose derivative with excess oxanorbornadiene **16a**. When 2 equiv. of the oxanorbornadiene was used instead, while we observed a marginal increase of **17p** yield to 69%, the di-substituted product yield rose substantially to 19%. Subjecting isolated **17p** to the exact synergistic catalysis conditions did not lead to any observable di-substituted product formed, suggesting that while the *cis*-diol motif on the polyol can react with the boronic acid catalyst to form the boronic ester, the absence of the *cis*-diol in the monofunctionalized product **17p** did not result in direct formation of the boronic acid hemiester. Furthermore, we did not detect any mono C2-functionalized product *via* NMR analysis of our crude reaction mixture, suggesting that formation of **17p** from a putative mono C2 functionalized product is highly unlikely.

Supplementary Note 15: For the α or β anomer confirmation of compound **17s**, **17t** and **17u**. Apart from the VCD analysis which confirmed the two newly generated stereogenic centers on the hydronapthalene scaffold, we also measured the $^{1}J_{C\text{-}H}$ coupling constant of the anomeric carbon, which is a reliable indicator of anomeric configuration of hexopyranoses previously reported in the literature.[32,33] For **17s** and **17t**, the $^{1}J_{C\text{-}H}$ coupling constants are 169.1 Hz and 167.6 Hz (~170 Hz) respectively, which indicate an equatorial proton (and substituent is hence α-configured) at C-1. For **17u**, the $^{1}J_{C\text{-}H}$ coupling constant is 157.5 Hz (~160 Hz), indicates an axial proton (and substituent is hence β-configured) at C-1 (See Supplementary Figures 409-411). 2D-NOESY cross-peaks (See Supplementary Figures 412-414) are further consistent with the anomeric assignment from $^{1}J_{C\text{-}H}$ coupling.

Supplementary Note 16: For the allylic carbonate reaction, we tried the conditions of the model reaction condition (**15a** + **16a** → **17a**) of the site selective functionalization (both Josiphos enantiomers tried). However, there were no product formed and only the substrates remained (See Supplementary table S5).

Supplementary Table 30: Mismatching combinations using the opposite enantiomeric ligand (Selected representative polyol substrates from every class of sugars from the substrate scope)

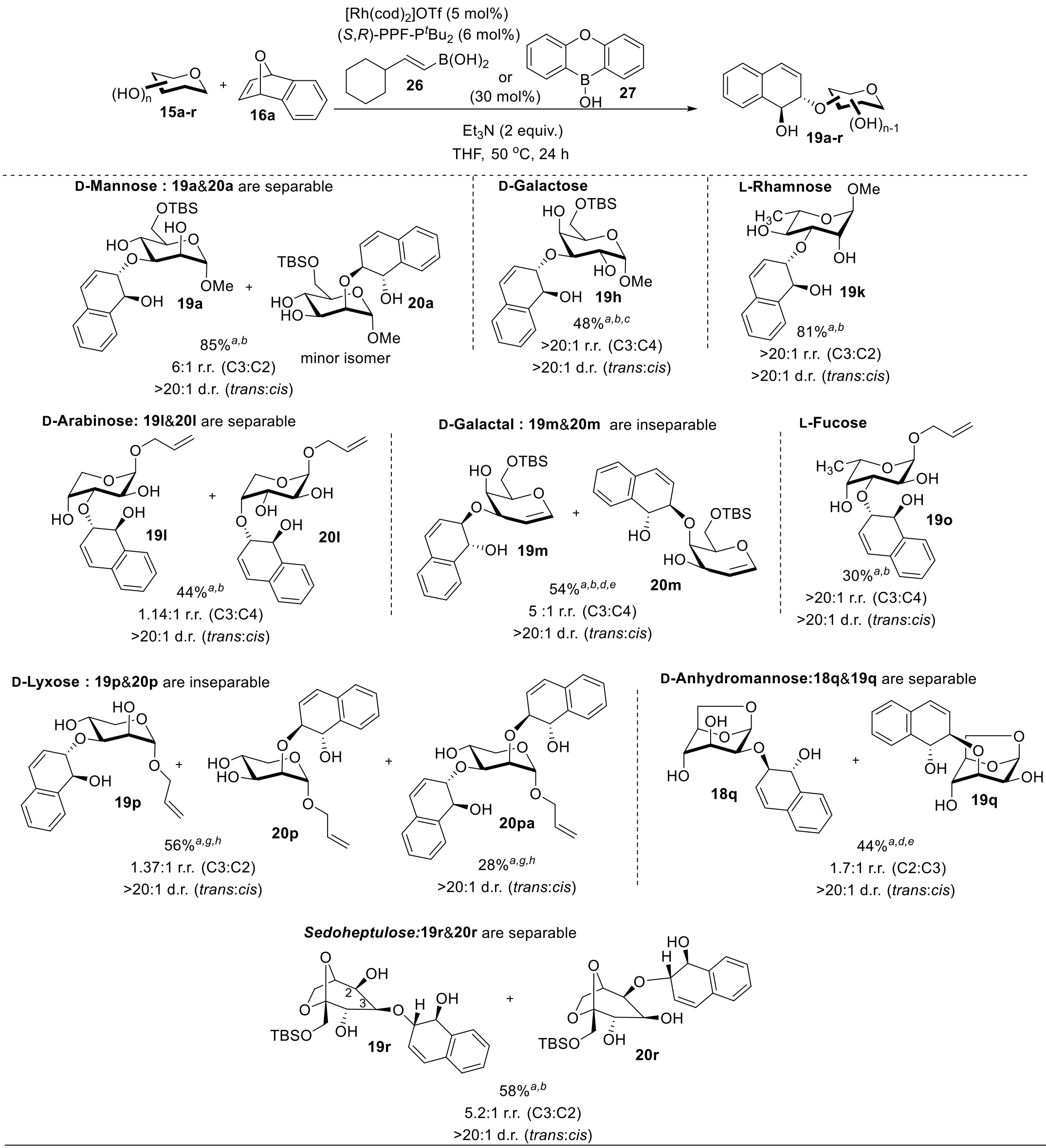

[a]Polyol **15a-r** (0.2 mmol), **16a** (0.4 mmol), $Rh(cod)_2OTf$ (5 mol%), (*S*,*R*)-PPF-P*t*Bu_2 (6 mol%), organoboron catalyst (30 mol%), in THF (2 mL), argon, 50 °C, 24 h. r.r. and d.r. were determined by crude ^{1}H NMR spectra analysis. [b]**26** was used as catalyst. [c]48h reaction time. [d]0.5 mL THF was used. [e](*R*,*S*)-PPF-P*t*Bu_2 was used instead. [f]12h reaction time. [g]**27** was used. [h]28% of C3,C2 disubstituted product was isolated, 1.5 equiv **16a** (0.3 mmol) used.

Supplementary Table 31: Mismatching combinations using the opposite enantiomeric ligand for anomeric functionalization (Selected representative polyol substrates from every class of sugars from the substrate scope)

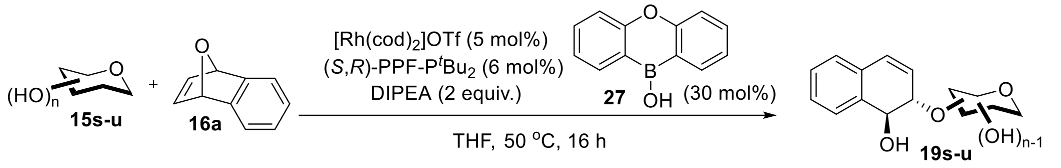

19s&**20s** are separable

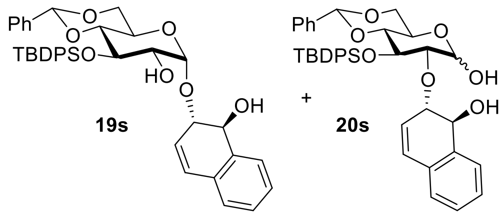

51%[a]
1:1 r.r. (C1:C2)
>20:1 d.r. (*trans*:*cis*)

19u&**20u** are inseparable

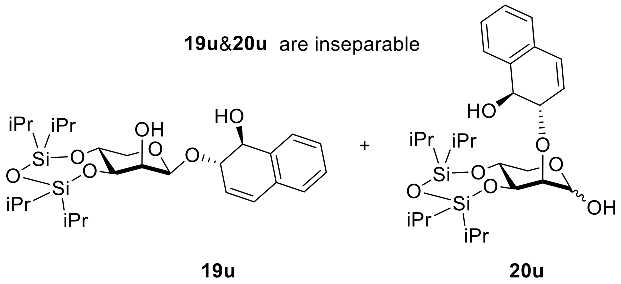

55%[a]
2:1 r.r. (C1:C2)
>20:1 d.r. (*trans*:*cis*)

[a]Polyol **15s-u** (0.2 mmol), **16a** (0.4 mmol), $Rh(cod)_2OTf$ (5 mol%), (*S*,*R*)-PPF-P*t*Bu_2 (6 mol%), organoboron catalyst **27** (30 mol%), in THF (2 mL), argon, 50 °C, 16 h. r.r. and d.r. were determined by crude ^{1}H NMR spectra analysis.

Supplementary Table 32: Mismatching combinations using the opposite enantiomeric ligand for allylic carbonate reaction (Selected representative polyol substrates from every class of sugars from the substrate scope)

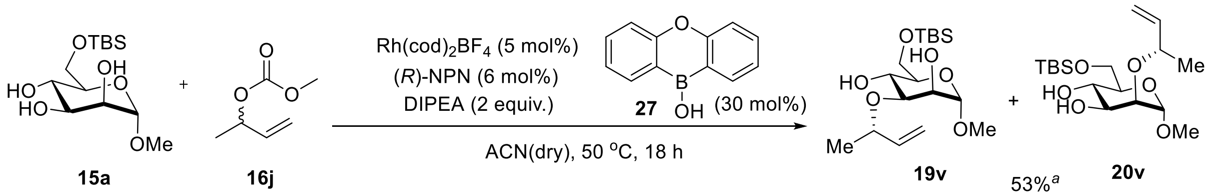

53%[a]
2:1 r.r. (C3:C2)
>20:1 d.r.
19v&**20v** are separable

[a]Polyol **15a** (0.2 mmol), **16j** (0.24 mmol), $Rh(cod)_2BF_4$ (5 mol%),(*R*)-NPN (6 mol%), organoboron catalyst **27** (30 mol%), in dry ACN (1 mL), argon, 50 °C, 18 h. rr and dr were determined by crude ^{1}H NMR spectra analysis.

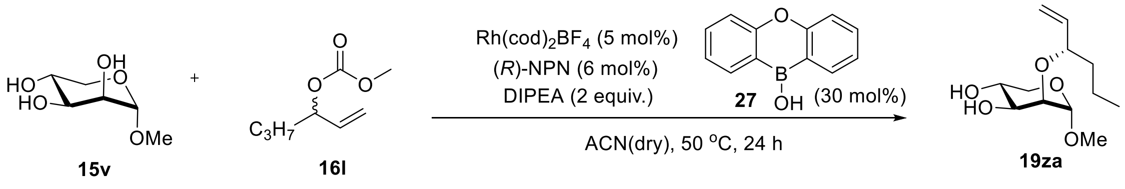

71%[a]
>20:1 r.r. (C2:C3)
>20:1 d.r.
only isomer

[a]Polyol **15v** (0.2 mmol), **16l** (0.24 mmol), $Rh(cod)_2BF_4$ (5 mol%),(*R*)-NPN (6 mol%), organoboron catalyst **27** (30 mol%), in dry ACN (1 mL), argon, 50 °C, 24 h. r.r. and d.r. were determined by crude ^{1}H NMR spectra analysis.

NMR data:

(*2R,3R,4S,5S,6S*)-2-(((*tert*-butyldimethylsilyl)oxy)methyl)-4-(((*1R,2R*)-1-hydroxy-1,2-dihydronaphthalen-2-yl)oxy)-6-methoxytetrahydro-2H-pyran-3,5-diol (17a)

The title product compound is prepared according to the general procedure **A** with 5 mol% Rh(cod)$_2$OTf catalyst, 30 mol% cyclohexyl vinyl boronic acid **26**, oxabicycle **16a** (0.4 mmol, 2 equiv.), and 0.2 mmol carbohydrate polyol **15a** at 50 ˚C for 24 h and isolated by flash column chromatography (9:1-2:1 Pentane: Ethyl Acetate) giving **17a** as a white solid (83.7 mg, 92% yield, r.r. >20:1(C3:C2), d.r. > 20:1 (*trans*:*cis*)).

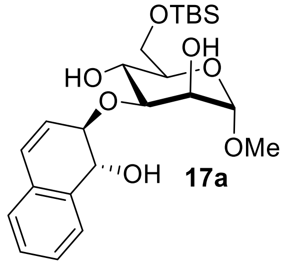

17a

^{1}H NMR (600 MHz, CDCl$_3$) δ 7.58 (d, *J* = 7.3 Hz, 1H), 7.26 – 7.19 (m, 2H), 7.04 (d, *J* = 7.2 Hz, 1H), 6.39 (dd, *J* = 10.1, 2.1 Hz, 1H), 6.00 (dd, *J* = 9.9, 1.9 Hz, 1H), 5.06 (d, *J* = 11.7 Hz, 1H), 4.74 (s, 1H), 4.47 (dd, J = 11.9, 2.3 Hz, 1H), 4.33 – 4.28 (bm, 1H), 4.04 (s, 1H), 3.93 – 3.90 (m, 3H), 3.83 – 3.80 (m, 1H), 3.66-3.63 (m, 1H), 3.53 – 3.46 (m, 2H), 3.35 (s, 3H), 0.91 (s, 9H), 0.11 (d, *J* = 2.3 Hz, 6H).**13C NMR** (151 MHz, CDCl$_3$) δ 136.38, 132.31, 128.89, 128.59, 128.01, 127.71, 126.24, 124.49, 100.71, 79.89, 76.89, 72.52, 70.70, 69.06, 67.81, 65.18, 55.00, 26.01, 18.40, -5.32, -5.34. **ESI-HRMS**: Calculated for $C_{23}H_{37}O_7Si$ $(M+H)^+$: 453.23031, Found: 453.23068. $[\alpha]_D^{20}$ = -32.09 (c = 0.43, CHCl$_3$).

(*2R,3R,4S,5S,6S*)-2-(((*tert*-butyldimethylsilyl)oxy)methyl)-4-(((*1S,2S*)-1-hydroxy-1,2-dihydronaphthalen-2-yl)oxy)-6-methoxytetrahydro-2H-pyran-3,5-diol (19a)

(*2R,3S,4S,5S,6S*)-2-(((*tert*-butyldimethylsilyl)oxy)methyl)-5-(((*1S,2S*)-1-hydroxy-1,2-dihydronaphthalen-2-yl)oxy)-6-methoxytetrahydro-2H-pyran-3,4-diol (20a)

The title products are prepared according to the general procedure **B** with 5 mol% Rh(cod)$_2$OTf catalyst, 30 mol% cyclohexyl vinyl boronic acid **26**, oxabicycle **16a** (0.4 mmol, 2 equiv.), and 0.2 mmol carbohydrate polyol **15a** at 50 ˚C for 24 h and isolated by flash column chromatography (9:1-2:1 Pentane: Ethyl Acetate) giving major C3 regioisomer **19a** (66 mg, d.r. > 20:1(*trans*:*cis*)) as a white solid and minor C2 regioisomer **20a** (11 mg, d.r. > 20:1(*trans*:*cis*)) as a white solid (77 mg combined total mass of **19a** and **20a**, 85% combined yield of **19a** and **20a**, r.r. 6:1(C3:C2)).

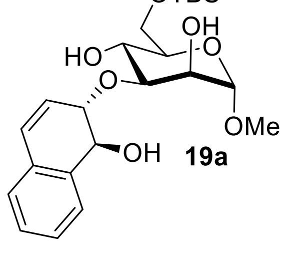

19a

^{1}H NMR (600 MHz, CDCl$_3$) δ 7.63 (d, *J* = 7.5 Hz, 1H), 7.26 – 7.19 (m, 2H), 7.03 (d, *J* = 7.2 Hz, 1H), 6.35 (dd, *J* = 9.9, 2.5 Hz, 1H), 5.85 (dd, *J* = 9.8, 1.8 Hz, 1H), 5.29 (bs, 1H), 5.08 (d, *J* = 11.5 Hz, 1H), 4.72 (s, 1H), 4.46 (dt, *J* = 11.5, 2.1 Hz, 1H), 4.39 (bs, 1H), 4.06 (t, *J* = 9.2 Hz, 1H), 3.96 – 3.93 (m, 3H), 3.85 (dd, *J* = 9.9, 8.0 Hz, 1H), 3.67-3.63 (m, 1H), 3.35 (s, 3H), 2.78 (s, 1H), 0.92 (s, 9H), 0.12 (d, *J* = 3.2 Hz, 6H). **^{13}C NMR** (151 MHz, CDCl$_3$) δ 136.68, 132.11, 130.00, 128.07, 128.00, 127.50, 126.11,

124.47, 100.40, 85.33, 81.57, 74.87, 71.50, 69.75, 69.58, 66.14, 55.08, 25.96, 18.32, -5.43, -5.48. **ESI-HRMS**: Calculated for $C_{23}H_{36}O_7NaSi$ $(M+Na)^+$: 475.21225, Found: 475.21159. $[\alpha]_D^{20}$ = +77.8 (c = 0.71, $CHCl_3$).

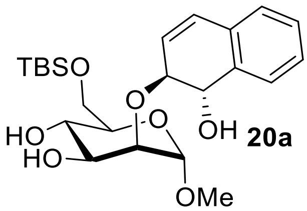

20a

¹H NMR (500 MHz, $CDCl_3$) δ 7.58 (d, *J* = 7.2 Hz, 1H), 7.24 – 7.18 (m, 2H), 7.03 (dd, *J* = 7.2, 1.4 Hz, 1H), 6.33 (dd, *J* = 9.9, 2.5 Hz, 1H), 5.86 (dd, *J* = 9.9, 1.8 Hz, 1H), 5.05 (d, *J* = 11.6 Hz, 1H), 4.73 (d, *J* = 1.4 Hz, 1H), 4.39 (dt, *J* = 11.6, 2.1 Hz, 1H), 3.94– 3.93 (m, 2H), 3.92 – 3.89 (m, 2H), 3.88 – 3.87 (m, 1H), 3.62 (s, 1H), 3.59-3.55 (m, 1H), 3.37 (s, 3H), 0.91 (s, 9H), 0.11 (d, *J* = 4.5 Hz, 6H). **¹³C NMR** (126 MHz, $CDCl_3$) δ 136.71, 132.12, 130.09, 127.99, 127.76, 127.58, 126.16, 124.49, 100.70, 86.31, 80.38, 74.57, 72.08, 71.51, 70.53, 65.32, 54.95, 25.98, 18.34, -5.33. **ESI-HRMS**: Calculated for $C_{23}H_{36}O_7NaSi$ $(M+Na)^+$: 475.21225, Found: 475.21170. $[\alpha]_D^{20}$ = +59.7 (c =0.38, $CHCl_3$).

6a,6b,7,12,12a,13a-hexahydro-7,12-epoxydinaphtho[1,2-b:2',3'-d]furan (21)

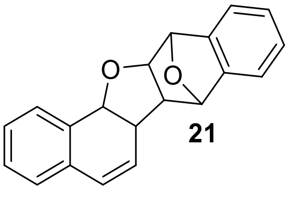

21

¹H NMR (600 MHz, $CDCl_3$) δ 7.36 (d, *J* = 7.3 Hz, 1H), 7.31 – 7.28 (m, 1H), 7.26 – 7.23 (m, 3H), 7.18-7.13 (m, 3H), 6.61 (dd, *J* = 9.9, 2.2 Hz, 1H), 6.02 (dd, *J* = 9.9, 3.2 Hz, 1H), 5.42 (s, 1H), 5.20 (s, 1H), 5.01 (d, *J* = 7.2 Hz, 1H), 4.36 (d, *J* = 6.4 Hz, 1H), 3.27 (ddt, *J* = 9.8, 7.1, 2.8 Hz, 1H), 2.93 (dd, *J* = 9.6, 6.4 Hz, 1H). **¹³C NMR** (151 MHz, $CDCl_3$) δ 146.23, 143.19, 132.56, 131.73, 128.95, 127.60, 127.36, 127.27, 126.96, 126.75, 126.19, 126.18, 120.71, 119.55, 83.27, 83.18, 81.81, 79.29, 52.61, 39.38. **ESI-HRMS**: Calculated for $C_{20}H_{16}O_2Na$ $(M+Na)^+$: 311.10425, Found: 311.10436.
The analytical data is in accordance to those reported in the literature.[18]

(*2R,4aR,6S,7S,8aR*)-8-(((*1R,2R*)-1-hydroxy-1,2-dihydronaphthalen-2-yl)oxy)-6-methoxy-2-phenylhexahydropyrano[3,2-d][1,3]dioxin-7-ol (17b)

The title product compound is prepared according to the general procedure **A** with 5 mol% $Rh(cod)_2OTf$ catalyst, 30 mol% cyclohexyl vinyl boronic acid **26**, oxabicycle **16a** (0.4 mmol, 2 equiv.), and 0.2 mmol carbohydrate polyol **15b** at 50 ˚C for 24 h and isolated by flash column chromatography (9:1-2:1 Pentane: Ethyl Acetate) giving **17b** as a white solid (69.2 mg, 81% yield, r.r. >20:1(C3:C2), d.r. > 20:1(*trans*:*cis*)).

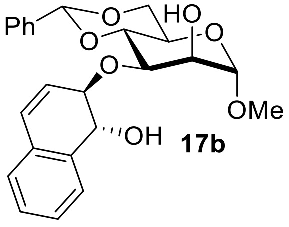

17b

¹H NMR (500 MHz, $CDCl_3$) δ 7.51 – 7.49 (m, 3H), 7.40 – 7.36 (m, 3H), 7.24 – 7.20 (m, 2H), 7.05 – 7.03 (m, 1H), 6.38 (dd, *J* = 9.9, 2.4 Hz, 1H), 6.00 (dd, *J* = 9.9, 2.0 Hz, 1H), 5.60 (s, 1H), 5.04 (dd, *J* = 11.7, 4.3 Hz, 1H), 4.81 (d, *J* = 1.4 Hz, 1H), 4.49 (dt, J = 11.6, 2.3 Hz, 1H), 4.32 – 4.25 (m, 1H), 4.17 (s, 1H), 4.10 – 4.03 (m, 2H), 3.90 – 3.81 (m, 2H), 3.61 (s, 1H), 3.44 (d, *J* = 5.4 Hz, 1H), 3.37 (s, 3H). **¹³C NMR** (126 MHz, $CDCl_3$) δ 137.39, 136.23, 132.34, 129.19, 129.08, 128.54, 128.40, 128.03, 127.92, 126.36,

126.24, 124.53, 102.00, 101.44, 81.15, 77.61, 74.63, 72.71, 69.57, 68.98, 55.13. **ESI-HRMS**: Calculated for $C_{24}H_{26}O_7Na$ (M+Na)$^+$: 449.15707, Found: 449.15756. $[\alpha]_D^{20}$ = -20.9 (c = 0.33, $CHCl_3$).

(*2R,3R,4S,5S,6S*)-2-(((*tert*-butyldimethylsilyl)oxy)methyl)-4-(((*1R,2R*)-1-hydroxy-1,2-dihydronaphthalen-2-yl)oxy)-6-(phenylthio)tetrahydro-2H-pyran-3,5-diol (17c)

The title product compound is prepared according to the general procedure **A** with 5 mol% Rh(cod)$_2$OTf catalyst, 30 mol% cyclohexyl vinyl boronic acid **26**, oxabicycle **16a** (0.4 mmol, 2 equiv.), and 0.2 mmol Carbohydrate polyol **15c** at 50 ˚C for 24 h and isolated by flash column chromatography (9:1-2:1 Pentane: Ethyl Acetate) giving **17c** as a white solid (80.2 mg, 76% yield, r.r. >20:1(C3:C2), d.r. > 20:1(*trans*:*cis*)).

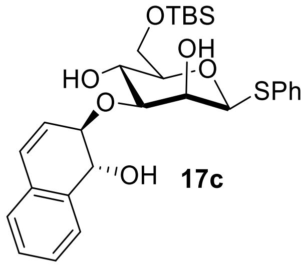

17c

^{1}H NMR (500 MHz, $CDCl_3$) δ 7.61 (d, *J* = 7.3 Hz, 1H), 7.49 – 7.47 (m, 2H), 7.30-7.20 (3, 5H), 7.05 (d, *J* = 7.2 Hz, 1H), 6.41 (dd, *J* = 9.9, 2.1 Hz, 1H), 5.99 (dd, *J* = 9.9, 1.6 Hz, 1H), 5.09 (d, *J* = 11.7 Hz, 1H), 4.85 (s, 1H), 4.51 (dt, *J* = 11.8, 2.2 Hz, 1H), 4.35 (s, 1H), 4.18 (bs, 1H), 3.99 – 3.92 (m, 3H), 3.61 (dd, *J* = 9.1, 3.2 Hz, 1H), 3.56 (s, 1H), 3.51 (bs, 1H), 3.44-3.40 (m, 1H), 0.91 (s, 9H), 0.10 (d, *J* = 8.9 Hz, 6H). ^{13}C NMR (126 MHz, $CDCl_3$) δ 136.22, 134.84, 132.16, 131.04, 129.10, 128.83, 128.54, 128.15, 127.79, 127.44, 126.31, 124.53, 87.08, 80.26 (2-Carbons), 78.73, 72.59, 69.63, 68.82, 65.04, 26.00, 18.37, -5.31, -5.38. **ESI-HRMS**: Calculated for $C_{28}H_{38}O_6NaSSi$ (M+Na)$^+$: 553.20506, Found: 553.20631. $[\alpha]_D^{20}$ = -47.9 (c =0.29, $CHCl_3$).

(*2R,3R,4S,5S,6R*)-2-(((*tert*-butyldimethylsilyl)oxy)methyl)-4-(((*1R,2R*)-1-hydroxy-1,2-dihydronaphthalen-2-yl)oxy)-6-(phenylthio)tetrahydro-2H-pyran-3,5-diol (17d)

The title product compound is prepared according to the general procedure **A** with 5 mol% Rh(cod)$_2$OTf catalyst, 30 mol% cyclohexyl vinyl boronic acid **26**, oxabicycle **16a** (0.4 mmol, 2 equiv.), and 0.2 mmol carbohydrate polyol **15d** at 50 ˚C for 24 h and isolated by flash column chromatography (9:1-2:1 Pentane: Ethyl Acetate) giving **17d** as a pale yellow sticky solid (87.6 mg, 83% yield, r.r. >20:1 (C3:C2), d.r. > 20:1(*trans*:*cis*)).

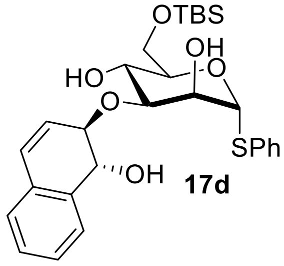

17d

^{1}H NMR (500 MHz, $CDCl_3$) δ 7.58 (d, *J* = 7.1 Hz, 1H), 7.46 – 7.43 (m, 2H), 7.30 – 7.21 (m, 5H), 7.06 (d, *J* = 6.8 Hz, 1H), 6.44 (d, *J* = 9.9 Hz, 1H), 6.02 (dd, *J* = 9.7, 1.4 Hz, 1H), 5.57 (s, 1H), 5.09 (dd, *J* = 11.8, 3.8 Hz, 1H), 4.50 (d, *J* = 11.8 Hz, 1H), 4.33 (s, 1H), 4.21 – 4.10 (m, 2H), 4.03 (td, *J* = 9.4, 1.3 Hz, 1H), 3.90 (d, *J* = 5.5 Hz, 2H), 3.85 – 3.83 (m, 1H), 3.68 (bs, 1H), 3.57 – 3.56 (m, 1H), 0.90 (s, 9H), 0.09 (d, *J* = 2.1 Hz, 6H). **^{13}C NMR** (126 MHz, $CDCl_3$) δ 136.22, 133.99, 132.26, 131.54, 129.19, 128.85, 128.61, 128.12, 127.82, 127.60, 126.34, 124.44, 87.74, 80.26, 77.37, 72.58, 71.75, 69.52, 69.27, 64.99, 26.04, 18.44, -5.33, -5.35. **ESI-HRMS**: Calculated for $C_{28}H_{38}O_6NaSSi$ (M+Na)$^+$: 553.20506, Found: 553.20622. $[\alpha]_D^{20}$ = +75 (c = 0.46, $CHCl_3$).

(*2R,3R,4S,5S,6S*)-2-(((*tert*-butyldimethylsilyl)oxy)methyl)-4-(((*1R,2R*)-1-hydroxy-1,2-dihydronaphthalen-2-yl)oxy)-6-(prop-2-yn-1-yloxy)tetrahydro-2H-pyran-3,5-diol (17e)

The title product compound is prepared according to the general procedure **A** with 5 mol% $Rh(cod)_2OTf$ catalyst, 30 mol% cyclohexyl vinyl boronic acid **26**, oxabicycle **16a** (0.4 mmol, 2 equiv.), and 0.2 mmol carbohydrate polyol **15e** at 50 ˚C for 24 h and isolated by flash column chromatography (9:1-2:1 Pentane: Ethyl Acetate) giving **17e** as a pale yellow sticky solid (55.7 mg, 58% yield, r.r. >20:1 (C3:C2), d.r. > 20:1(*trans*:*cis*)).

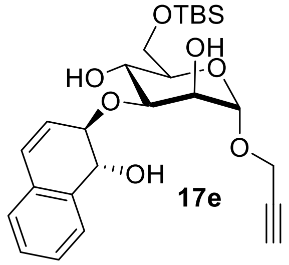

17e

^{1}H NMR (500 MHz, $CDCl_3$) δ 7.58 (d, *J* = 7.2 Hz, 1H), 7.26-7.20 (m, 2H), 7.05 (d, *J* = 7.0 Hz, 1H), 6.41 (dd, *J* = 9.9, 2.3 Hz, 1H), 5.99 (dd, *J* = 9.9, 1.8 Hz, 1H), 5.09-5.05 (m, 2H), 4.49 (d, *J* = 11.9 Hz, 1H), 4.23 (t, *J* = 2.4 Hz, 2H), 4.08 – 4.07 (m, 1H), 4.01 – 3.93 (m, 2H), 3.92 – 3.89 (m, 2H), 3.88-3.85 (m, , 1H), 3.70-3.66 (m, 1H), 3.50 (s, 1H), 3.36-3.33 (m, 1H), 2.43 (t, *J* = 2.4 Hz, 1H), 0.91 (s, 9H), 0.11 (d, *J* = 2.1 Hz, 6H). ^{13}C NMR (126 MHz, $CDCl_3$) δ 136.31, 132.28, 128.79, 128.75, 128.07, 127.79, 126.31, 124.42, 98.21, 79.97, 78.78, 76.54, 75.03, 72.49, 71.03, 69.12, 67.81, 65.09, 54.44, 26.01, 18.40, -5.32, -5.35. **ESI-HRMS**: Calculated for $C_{25}H_{36}O_7NaSi$ $(M+Na)^+$: 499.21225, Found: 499.21302. $[\alpha]_D^{20}$ = -13.0 (c =0.20, $CHCl_3$).

(*2R,3R,4S,5S,6S*)-2-((benzyloxy)methyl)-4-(((*1R,2R*)-1-hydroxy-1,2-dihydronaphthalen-2-yl)oxy)-6-methoxytetrahydro-2H-pyran-3,5-diol (17f)

The title product compound is prepared according to the general procedure **A** with 5 mol% $Rh(cod)_2OTf$ catalyst, 30 mol% cyclohexyl vinyl boronic acid **26**, oxabicycle **16a** (0.4 mmol, 2 equiv.), and 0.2 mmol carbohydrate polyol **15f** at 50 ˚C for 14 h and isolated by flash column chromatography (5:1-1:1 Pentane: Ethyl Acetate) giving **17f** as a grey solid (77.0 mg, 90% yield, r.r. >20:1 (C3:C2), d.r. > 20:1(*trans*:*cis*)).

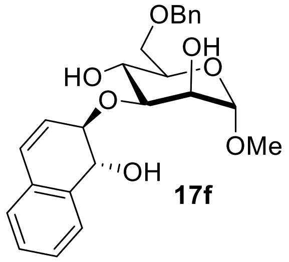

17f

^{1}H NMR (600 MHz, $CDCl_3$) δ 7.58 (d, J = 7.2 Hz, 1H), 7.34 (d, J = 4.2 Hz, 4H), 7.31 – 7.27 (m, 1H), 7.25 – 7.20 (m, 2H), 7.06 – 7.03 (m, 1H), 6.41 (dd, J = 10.2, 2.4 Hz, 1H), 5.97 (dd, J = 9.6, 1.8 Hz, 1H), 5.02 (dd, J = 11.4, 3.6 Hz, 1H), 4.77 (d, J = 1.8 Hz, 1H), 4.66 – 4.57 (m, 2H), 4.45 (dt, J = 11.4, 2.4 Hz, 1H), 4.03 (d, J = 4.2 Hz, 2H), 3.97 (td, J = 9.6, 1.8 Hz, 1H), 3.85 – 3.72 (m, 4H), 3.36 (s, 3H), 3.33 (d, J = 4.2 Hz, 1H), 3.13 (d, J = 2.4 Hz, 1H). **^{13}C NMR** (151 MHz, $CDCl_3$) δ 137.85, 136.31, 132.25, 128.71, 128.68, 128.63, 128.11, 127.98, 127.92, 127.79, 126.30, 124.52, 100.94, 79.85, 77.08, 73.93, 72.62, 70.78, 70.10, 67.90, 55.18. **ESI-HRMS**: Calculated for $C_{24}H_{28}O_7Na$ $(M+Na)^+$: 451.17272, Found: 451.17198. $[\alpha]_D^{20}$ = -37.6(c =0.42, $CHCl_3$).

(*4aR,6S,7S,8R,8aR*)-8-(((*1R,2R*)-1-hydroxy-1,2-dihydronaphthalen-2-yl)oxy)-6-methoxy-2,2-dimethylhexahydropyrano[3,2-d][1,3]dioxin-7-ol(17g)

The title product compound is prepared according to the general procedure **A** with 5 mol% $Rh(cod)_2OTf$ catalyst, 30 mol% cyclohexyl vinyl boronic acid 26, oxabicycle 16a (0.4 mmol, 2 equiv.), and 0.2 mmol carbohydrate polyol **15g** at 50 ˚C for 14 h and isolated by flash column chromatography (5:1-1:1

Pentane: Ethyl Acetate) giving **17g** as a grey solid (34.0 mg, 45% yield, r.r. >20:1 (C3:C2), d.r. > 20:1(*trans*:*cis*)).

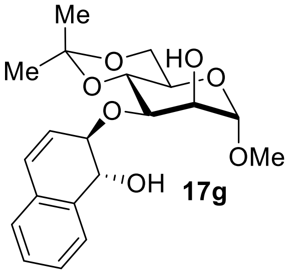

17g

^{1}H NMR (500 MHz, $CDCl_3$) δ 7.57 (d, J = 7.2 Hz, 1H), 7.26 – 7.18 (m, 2H), 7.08 – 7.02 (m, 1H), 6.40 (dd, J = 10.0, 2.5 Hz, 1H), 6.01 (dd, J = 10.0, 2.0 Hz, 1H), 5.00 (d, J = 11.0 Hz, 1H), 4.95 (s, 1H), 4.32 (dt, J = 11.0, 2.5 Hz, 1H), 4.18 – 4.13 (m, 2H), 4.10 (dd, J = 11.0, 3.0 Hz, 1H), 3.88 (dd, J = 11.0, 5.0 Hz, 1H), 3.86 – 3.81 (m, 1H), 3.77 – 3.71 (m, 2H), 3.41 (s, 3H), 3.31 (s, 1H), 1.52 (s, 3H), 1.35 (s, 3H). **^{13}C NMR** (126 MHz, $CDCl_3$) δ 136.28, 132.23, 128.74, 128.12, 127.99, 127.79, 126.29, 124.65, 109.82, 98.72, 83.41, 78.31, 75.66, 73.75, 70.07, 69.87, 69.31, 55.32, 28.07, 26.18. **ESI-HRMS**: Calculated for $C_{20}H_{26}O_7Na$ $(M+Na)^+$: 401.15707, Found: 401.15680. $[\alpha]_D^{20}$ = -76.2 (c =0.50, $CHCl_3$).

(*2R,3S,4S,5R,6S*)-2-(((*tert*-butyldimethylsilyl)oxy)methyl)-4-(((*1R,2R*)-1-hydroxy-1,2-dihydronaphthalen-2-yl)oxy)-6-methoxytetrahydro-2H-pyran-3,5-diol (17h)

The title product compound is prepared according to the general procedure **A** with 5 mol% $Rh(cod)_2OTf$ catalyst, 30 mol% cyclohexyl vinyl boronic acid **26**, oxabicycle **16a** (0.4 mmol, 2 equiv.), and 0.2 mmol carbohydrate polyol **15h** at 50 ˚C for 48 h and isolated by flash column chromatography (9:1-1:2 Pentane: Ethyl Acetate) giving **17h** as a white solid (67.2 mg, 74% yield, r.r. >20:1(C3:C4), d.r. > 20:1(*trans*:*cis*)).

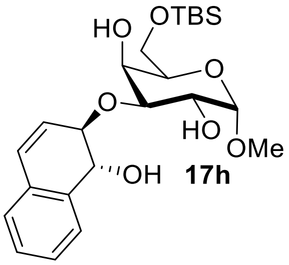

17h

1H NMR (700 MHz, $CDCl_3$) δ 7.59 (d, *J* = 7.5 Hz, 1H), 7.24 – 7.19 (m, 2H), 7.03 (d, *J* = 7.3 Hz, 1H), 6.35 (d, *J* = 9.8 Hz, 1H), 5.89 (dd, *J* = 9.9, 1.1 Hz, 1H), 5.06 (d, *J* = 11.4 Hz, 1H), 4.82 (d, *J* = 3.2 Hz, 1H), 4.46 (d, *J* = 11.4 Hz, 1H), 4.11 – 4.08 (m, 2H), 3.89 (dd, *J* = 10.4, 5.6 Hz, 1H), 3.83 (dd, *J* = 10.4, 5.4 Hz, 1H), 3.75 (dd, *J* = 9.8, 2.6 Hz, 1H), 3.73 (t, *J* = 5.4 Hz, 1H), 3.39 (s, 3H), 0.90 (s, 9H), 0.09 (d, *J* = 2.7 Hz, 6H). **13C NMR** (176 MHz, $CDCl_3$) δ 136.52, 132.02, 129.91, 128.07, 128.02, 127.61, 126.19, 124.58, 99.49, 85.28, 81.80, 74.30, 70.11, 69.34, 69.07, 63.00, 55.33, 25.96, 18.40, -5.27, -5.34. **ESI-HRMS**: Calculated for $C_{23}H_{36}O_7NaSi$ $(M+Na)^+$: 475.21225, Found: 475.21174. $[\alpha]_D^{20}$ = + 56.2 (c =0.32, $CHCl_3$).

(*2R,3S,4S,5R,6S*)-2-(((*tert*-butyldimethylsilyl)oxy)methyl)-4-(((*1S,2S*)-1-hydroxy-1,2-dihydronaphthalen-2-yl)oxy)-6-methoxytetrahydro-2H-pyran-3,5-diol (19h)

The title product compound is prepared according to the general procedure **B** with 5 mol% $Rh(cod)_2OTf$ catalyst, 30 mol% cyclohexyl vinyl boronic acid **26**, oxabicycle **16a** (0.4 mmol, 2 equiv.), and 0.2 mmol carbohydrate polyol **15h** at 50 ˚C for 48 h and isolated by flash column chromatography (9:1-1:2 Pentane: Ethyl Acetate) giving **19h** as a white solid (43.6 mg, 48% yield, r.r. >20:1(C3:C4), d.r. > 20:1(*trans*:*cis*)).

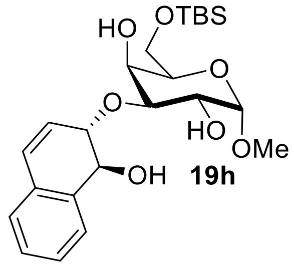

19h

^{1}H NMR (500 MHz, $CDCl_3$) δ 7.60 (d, *J* = 7.3 Hz, 1H), 7.26 – 7.19 (m, 2H), 7.03 (dd, *J* = 7.2, 1.1 Hz, 1H), 6.38 (dd, *J* = 9.9, 2.4 Hz, 1H), 5.97 (dd, *J* = 9.9, 1.6 Hz, 1H), 5.02 (d, *J* = 11.7 Hz, 1H), 4.89 (d, *J* = 3.9 Hz, 1H), 4.64 (d, *J* = 2.6 Hz, 1H), 4.43 (dt, *J* = 11.8, 2.0 Hz, 1H), 4.26 (s, 1H), 4.23 (s, 1H), 4.15-4.11 (m, 1H), 3.94 – 3.84 (m, 2H), 3.72 (dd, *J* = 9.7, 3.1 Hz, 1H), 3.69 (t, *J* = 4.4 Hz, 1H), 3.42 (s, 3H), 2.72 (d, *J* = 6.6 Hz, 1H), 0.88 (s, 9H), 0.08 (d, *J* = 1.6 Hz, 6H). **^{13}C NMR** (126 MHz, $CDCl_3$) δ 136.31, 132.25, 129.01, 128.32, 128.00, 127.64, 126.18, 124.46, 99.55, 79.66, 77.14 (please see DEPT), 72.79, 69.22, 67.74, 67.67, 64.44, 55.36, 25.92, 18.36, -5.36. **ESI-HRMS**: Calculated for $C_{23}H_{36}O_7NaSi$ $(M+Na)^+$: 475.21225, Found: 475.21179. $[\alpha]_D^{20}$ = +132.7 (c =1.15, $CHCl_3$).

(*2R,3S,4S,5R,6R*)-2-(((*tert*-butyldimethylsilyl)oxy)methyl)-4-(((*1R,2R*)-1-hydroxy-1,2-dihydronaphthalen-2-yl)oxy)-6-methoxytetrahydro-2H-pyran-3,5-diol (17i)

The title product compound is prepared according to the general procedure **A** with 5 mol% $Rh(cod)_2OTf$ catalyst, 30 mol% cyclohexyl vinyl boronic acid **26**, oxabicycle **16a** (0.4 mmol, 2 equiv.), and 0.2 mmol carbohydrate polyol **15i** at 50 ˚C for 24 h and isolated by flash column chromatography (9:1-1:2 Pentane: Ethyl Acetate) giving **17i** as a white solid (71.4 mg, 79% yield, r.r. >20:1(C3:C4), d.r. > 20:1(*trans*:*cis*)).

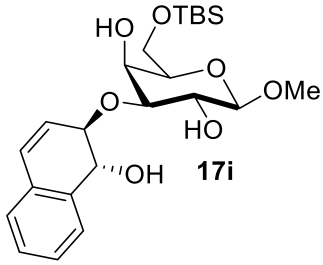

17i

^{1}H NMR (500 MHz, $CDCl_3$) δ 7.61 (d, *J* = 7.3 Hz, 1H), 7.24 – 7.17 (m, 2H), 7.01 (d, *J* = 6.4 Hz, 1H), 6.34 (d, *J* = 8.5 Hz, 1H), 5.89 (dd, *J* = 9.8, 1.5 Hz, 1H), 5.08 (d, *J* = 11.5 Hz, 1H), 4.45 (d, *J* = 11.5 Hz, 1H), 4.19 (d, *J* = 7.7 Hz, 1H), 4.00 (s, 1H), 3.93 – 3.89 (m, 2H), 3.84 (dd, *J* = 10.3, 5.4 Hz, 1H), 3.68 (dd, *J* = 9.7, 2.5 Hz, 1H), 3.52 (s, 3H), 3.44 (t, *J* = 5.8 Hz, 1H), 0.89 (s, 9H), 0.08 (d, *J* = 2.6 Hz, 6H). **^{13}C NMR** (126 MHz, $CDCl_3$) δ 136.50, 132.14, 129.83, 128.07, 127.97, 127.52, 126.10, 124.49, 104.10, 85.08, 83.37, 74.69 (2-carbons), 71.29, 67.95, 62.34, 57.06, 25.95, 18.41, -5.26, -5.30. **ESI-HRMS**: Calculated for $C_{23}H_{36}O_7NaSi$ $(M+Na)^+$: 475.21225, Found: 475.21262. $[\alpha]_D^{20}$ = -33.4 (c = 0.38, $CHCl_3$).

(*2R,3S,4S,5R,6S*)-2-(((*tert*-butyldimethylsilyl)oxy)methyl)-4-(((*1R,2R*)-1-hydroxy-1,2-dihydronaphthalen-2-yl)oxy)-6-(isopropylthio)tetrahydro-2H-pyran-3,5-diol (17j)

The title product compound is prepared according to the general procedure **A** with 5 mol% $Rh(cod)_2OTf$ catalyst, 30 mol% cyclohexyl vinyl boronic acid **26**, oxabicycle **16a** (0.4 mmol, 2 equiv.), and 0.2 mmol carbohydrate polyol **15j** at 50 ˚C for 24 h and isolated by flash column chromatography (9:1-1:1 Pentane: Ethyl Acetate) giving **17j** as a colorless sticky solid (56.8 mg, 57% yield, r.r. >20:1(C3:C4), d.r. > 20:1(*trans*:*cis*)).

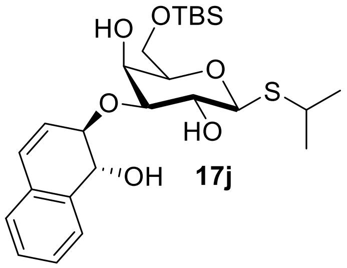

17j

^{1}H NMR (700 MHz, $CDCl_3$) δ 7.62 (d, *J* = 7.4 Hz, 1H), 7.22 (dt, *J* = 29.5, 7.3 Hz, 2H), 7.03 (d, *J* = 7.1 Hz, 1H), 6.36 (d, *J* = 9.7 Hz, 1H), 5.89 (d, *J* = 9.8 Hz, 1H), 5.08 (d, *J* = 7.3 Hz, 1H), 4.96 (bs, 1H), 4.47 (d, *J* = 11.5 Hz, 1H), 4.39 (d, *J* = 9.8 Hz, 1H), 4.06 (s, 1H), 3.92 – 3.88 (m, 2H), 3.83 (dd, *J* = 10.4, 5.4 Hz, 1H), 3.69 (d, *J* = 9.1 Hz, 1H), 3.47 (t, *J* = 5.5 Hz, 1H), 3.27 (bs, 1H), 3.22 (hept, *J* = 6.7 Hz, 1H), 2.87 (bs, 1H), 1.33 (dd, *J* = 6.5, 4.2 Hz, 6H), 0.89 (s, 9H), 0.08 (d, *J* = 6.2 Hz, 6H). **^{13}C NMR** (176 MHz, $CDCl_3$) δ 136.51, 132.13, 129.83, 128.17, 128.02, 127.57, 126.14, 124.52, 86.24, 85.17, 84.63, 78.62, 74.74, 69.83, 68.33, 62.65, 35.78, 25.96, 24.40, 24.31, 18.39, -5.27, -5.34. **ESI-HRMS**: Calculated for $C_{25}H_{40}O_6NaSSi$ $(M+Na)^+$: 519.22071, Found: 519.22004. $[\alpha]_D^{20}$ = -45.5 (c =1.21, $CHCl_3$).

(*2R,3R,4R,5S,6S*)-4-(((*1R,2R*)-1-hydroxy-1,2-dihydronaphthalen-2-yl)oxy)-2-methoxy-6-methyltetrahydro-2H-pyran-3,5-diol (17k)

The title product compound is prepared according to the general procedure **A** with 5 mol% $Rh(cod)_2OTf$ catalyst, 30 mol% cyclohexyl vinyl boronic acid **26**, oxabicycle **16a** (0.4 mmol, 2 equiv.), and 0.2 mmol carbohydrate polyol **15k** at 50 ˚C for 24 h and isolated by flash column chromatography (9:1-1:4 Pentane: Ethyl Acetate) giving **17k** as a white solid (45.3 mg, 70% yield, r.r. >20:1(C3:C2), d.r. > 20:1(*trans*:*cis*)).

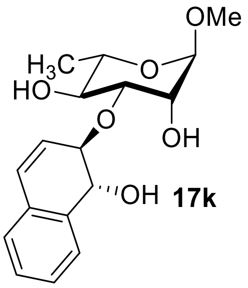

17k

^{1}H NMR (500 MHz, $CDCl_3$) δ 7.55 (d, *J* = 6.9 Hz, 1H), 7.22 (p, *J* = 7.4 Hz, 2H), 7.03 (d, *J* = 6.7 Hz, 1H), 6.35 (d, *J* = 9.8 Hz, 1H), 5.87 (dd, *J* = 9.8, 1.9 Hz, 1H), 5.05 (d, *J* = 11.4 Hz, 1H), 4.84 (bs, 1H), 4.69 (s, 1H), 4.50 (d, *J* = 11.4 Hz, 1H), 4.16 (bs, 1H), 3.97 (s, 1H), 3.81 (dd, *J* = 9.2, 3.1 Hz, 1H), 3.71 (t, *J* = 9.3 Hz, 1H), 3.65 (m, 1H), 3.33 (s, 3H), 3.01 (bs, 1H), 1.32 (d, *J* = 6.0 Hz, 3H). **^{13}C NMR** (126 MHz, $CDCl_3$) δ 136.30, 132.11, 129.70, 128.19, 128.09, 127.84, 126.35, 124.55, 100.40, 84.47, 81.74, 74.64, 72.61, 70.56, 68.10, 55.00, 17.73. **ESI-HRMS**: Calculated for $C_{17}H_{22}O_6Na$ $(M+Na)^+$: 345.13086, Found: 345.13098. $[\alpha]_D^{20}$ = -83.7 (c = 1.26, $CHCl_3$).

(*2R,3R,4R,5S,6S*)-4-(((*1S,2S*)-1-hydroxy-1,2-dihydronaphthalen-2-yl)oxy)-2-methoxy-6-methyltetrahydro-2H-pyran-3,5-diol (19k)

The title product compound is prepared according to the general procedure **B** with 5 mol% $Rh(cod)_2OTf$ catalyst, 30 mol% cyclohexyl vinyl boronic acid **26**, oxabicycle **16a** (0.4 mmol, 2 equiv.), and 0.2 mmol carbohydrate polyol **15k** at 50 ˚C for 24 h and isolated by flash column chromatography (9:1-1:4 Pentane: Ethyl Acetate) giving **19k** as a white solid (52.3 mg, 81% yield, r.r. >20:1(C3:C2), d.r. > 20:1(*trans*:*cis*)).

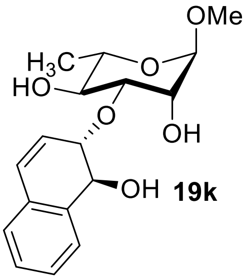

19k

^{1}H NMR (500 MHz, CD_3OD) δ 7.53 (d, *J* = 7.1 Hz, 1H), 7.22 – 7.16 (m, 2H), 7.04 (dd, *J* = 6.7,1.4 Hz, 1H), 6.39 (dd, *J* = 10.0, 2.2 Hz, 1H), 6.11 (dd, *J* = 9.9, 1.9 Hz, 1H), 4.85 (d, *J* = 11.3 Hz, 1H), 4.62 (d, *J* = 1.5 Hz, 1H), 4.31 (dt, *J* = 11.2, 2.1 Hz, 1H), 4.02 (dd, *J* = 3.0, 1.8 Hz, 1H), 3.62 – 3.56 (m, 2H), 3.48 (t, *J* = 9.4 Hz, 1H), 3.33 (s, 3H), 1.29 (d, *J* = 6.2 Hz, 3H). **^{13}C NMR** (126 MHz, CD_3OD) δ 137.96, 133.80, 130.75, 128.65, 128.63, 128.46, 127.14, 125.71, 102.25, 81.53, 79.92, 74.07, 72.52, 69.57, 69.31, 55.12, 18.17. **ESI-HRMS**: Calculated for $C_{17}H_{22}O_6Na$ $(M+Na)^+$: 345.13086, Found: 345.13100. $[\alpha]_D^{20}$ = +27.4 (c =1.49, $CHCl_3$).

(*2R,3S,4R,5R*)-2-(allyloxy)-4-(((*1R,2R*)-1-hydroxy-1,2-dihydronaphthalen-2-yl)oxy)tetrahydro-2H-pyran-3,5-diol (17l)

The title product compound is prepared according to the general procedure **A** with 5 mol% $Rh(cod)_2OTf$ catalyst, 30 mol% cyclohexyl vinyl boronic acid **26**, oxabicycle **16a** (0.4 mmol, 2 equiv.), and 0.2 mmol carbohydrate polyol **15l** at 50 ˚C for 24 h and isolated by flash column chromatography (9:1-1:4 Pentane: Ethyl Acetate) giving **17l** as a white solid (52.6 mg, 79% yield, r.r. >20:1(C3:C4), d.r. > 20:1(*trans*:*cis*)).

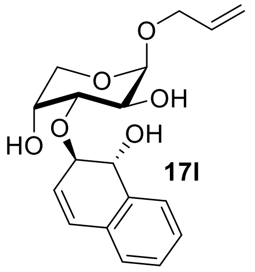

17l

^{1}H NMR (500 MHz, $CDCl_3$) δ 7.59 (d, *J* = 7.0 Hz, 1H), 7.28 – 7.21 (m, 2H), 7.06 – 7.05 (m, 1H), 6.39 (dd, *J* = 9.9, 2.3 Hz, 1H), 6.02 (dd, *J* = 9.8, 1.6 Hz, 1H), 5.99-5.91 (m, 1H), 5.33 (dd, *J* = 17.2, 1.5 Hz, 1H), 5.24 – 5.22 (m, 1H), 5.06 (d, *J* = 11.7 Hz, 1H), 5.01 (d, *J* = 3.7 Hz, 1H), 4.46 (d, *J* = 11.7 Hz, 1H), 4.23 (dd, *J* = 12.7, 5.4 Hz, 1H), 4.14 (s, 1H), 4.10 (dd, *J* = 9.6, 3.8 Hz, 1H), 4.09 – 4.04 (m, 1H), 3.88 – 3.80 (m, 3H). **^{13}C NMR** (126 MHz, $CDCl_3$) δ 136.39, 133.75, 132.30, 128.93, 128.34, 128.03, 127.77, 126.26, 124.47, 118.19, 97.94, 79.71, 76.43, 72.83, 68.76, 67.97, 66.42, 62.37. **ESI-HRMS**: Calculated for $C_{18}H_{22}O_6Na$ $(M+Na)^+$: 357.13086, Found: 357.13104. $[\alpha]_D^{20}$ = -166.6 (c = 0.15, $CHCl_3$).

(*2R,3S,4R,5R*)-2-(allyloxy)-4-(((*1S,2S*)-1-hydroxy-1,2-dihydronaphthalen-2-yl)oxy)tetrahydro-2H-pyran-3,5-diol (19l)

(*2R,3S,4S,5R*)-2-(allyloxy)-5-(((*1S,2S*)-1-hydroxy-1,2-dihydronaphthalen-2-yl)oxy)tetrahydro-2H-pyran-3,4-diol (20l)

The title products are prepared according to the general procedure **B** with 5 mol% $Rh(cod)_2OTf$ catalyst, 30 mol% cyclohexyl vinyl boronic acid **26**, oxabicycle **16a** (0.4 mmol, 2 equiv.), and 0.2 mmol carbohydrate polyol **15l** at 50 ˚C for 24 h and isolated by flash column chromatography (9:1-1:4 Pentane: Ethyl Acetate) giving the C3 major product **19l** as a white solid (15.8 mg, d.r. > 20:1(*trans*:*cis*)) and the C4 minor product **20l** (13.9 mg, d.r. > 20:1(*trans*:*cis*)) as a white solid (29.7 mg total mass combined for **19l** and **20l**, 44% combined yield of **19l** and **20l**, r.r. 1.14:1(C3:C4)).

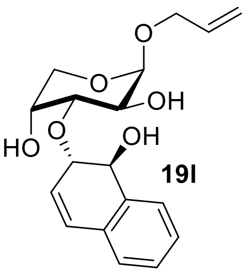

19l

^{1}H NMR (500 MHz, CD_3OD) δ 7.51 (d, *J* = 6.5 Hz, 1H), 7.22 – 7.17 (m, 2H), 7.05 – 7.04 (m, 1H), 6.40 (dd, *J* = 9.9, 2.2 Hz, 1H), 6.06 (dd, *J* = 9.9, 2.3 Hz, 1H), 5.99-5.91 (m, 1H), 5.32 (dq, *J* = 17.3, 1.6 Hz, 1H), 5.16 (dd, *J* = 10.4, 1.5 Hz, 1H), 4.93 (d, *J* = 10.5 Hz, 1H), 4.86 (d, 1H, merged with CD_3OD), 4.43 (dt, *J* = 10.5, 2.3 Hz, 1H), 4.17 (ddt, *J* = 13.0, 5.2, 1.4 Hz, 1H), 4.04 – 3.98 (m, 3H), 3.92 (dd, *J* = 9.8, 3.2 Hz, 1H), 3.81 (dd, *J* = 12.3, 1.1 Hz, 1H), 3.58 (dd, *J* = 12.3, 2.3 Hz, 1H). **^{13}C NMR** (126 MHz, CD_3OD) δ 137.98, 135.59, 133.65, 131.07, 128.67 (two carbon), 128.62, 127.19, 126.08, 117.51, 99.93, 83.75, 79.90, 74.91, 69.59 (two carbon), 69.53, 64.21. **ESI-HRMS**: Calculated for $C_{18}H_{22}O_6Na$ $(M+Na)^+$: 357.13086, Found: 357.13097. $[\alpha]_D^{20}$ = -86.3 (c = 0.47, $CHCl_3$).

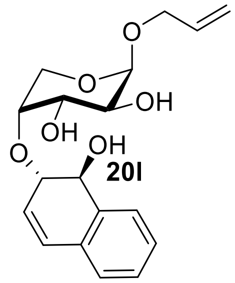

20l

^{1}H NMR (500 MHz, CD_3OD) δ 7.52 (s, 1H), 7.22 – 7.18 (m, 2H), 7.05 – 7.03 (m, 1H), 6.38 (dd, *J* = 9.9, 2.2 Hz, 1H), 5.99 – 5.91 (m, 2H), 5.31 (dq, *J* = 17.3, 1.5 Hz, 1H), 5.16 (dq, *J* = 10.4, 1.3 Hz, 1H), 4.90 (d, *J* = 11.2 Hz, 1H), 4.86 (d, *J* = 2.7 Hz, 1H), 4.33 (dt, *J* = 11.1, 2.2 Hz, 1H), 4.18 (ddt, *J* = 13.0, 5.2, 1.4 Hz, 1H), 4.02 (ddt, *J* = 13.0, 6.0, 1.1 Hz, 1H), 3.91 (s, 1H), 3.87 – 3.86 (m, 2H), 3.80 (d, *J* = 12.6 Hz, 1H), 3.69 (dd, *J* = 12.6, 2.3 Hz, 1H). **^{13}C NMR** (126 MHz, CD_3OD) δ 138.21, 135.61, 133.63, 131.38, 128.65, 128.61, 128.52, 127.17, 125.70, 117.52, 99.92, 84.59, 80.57, 74.88, 71.11, 70.83, 69.61, 63.15. **ESI-HRMS:** Calculated for $C_{18}H_{22}O_6Na$ $(M+Na)^+$: 357.13086, Found: 357.13103. $[\alpha]_D^{20}$ = -30.1 (c = 0.69, $CHCl_3$).

(*2R,3R*)-2-(((*tert*-butyldimethylsilyl)oxy)methyl)-4-(((*1S,2S*)-1-hydroxy-1,2-dihydronaphthalen-2-yl)oxy)-3,4-dihydro-2H-pyran-3-ol (17m)

The title product compound is prepared according to the general procedure **B** with 5 mol% $Rh(cod)_2OTf$ catalyst, 30 mol% cyclohexyl vinyl boronic acid **26**, oxabicycle **16a** (0.4 mmol, 2 equiv.), and 0.2 mmol carbohydrate polyol **15m,** in 0.5 ml THF at 50 ˚C for 24 h and isolated by flash column chromatography (15:1-2:1 Pentane: Ethyl Acetate) giving **17m** as a color less liquid (72.9 mg, 90% yield, r.r. >20:1(C3:C4), d.r. > 20:1(*trans*:*cis*)).

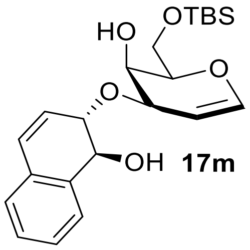

17m

^{1}H NMR (500 MHz, $CDCl_3$) δ 7.63 (d, *J* = 7.4 Hz, 1H), 7.27 – 7.20 (m, 2H), 7.06 – 7.04 (m, 1H), 6.47 (dd, *J* = 6.3, 1.6 Hz, 1H), 6.39 (dd, *J* = 9.9, 2.4 Hz, 1H), 5.92 (dd, *J* = 9.9, 1.7 Hz, 1H), 5.01 (d, *J* = 11.5 Hz, 1H), 4.73 (dt, *J* = 6.2, 1.7 Hz, 1H), 4.68 (bs, 1H), 4.37 (dt, *J* = 11.5, 2.1 Hz, 1H), 4.31 – 4.30 (m,

1H), 4.28 (s, 1H), 4.04 (dd, J = 10.9, 4.8 Hz, 1H), 3.98 (s, 1H), 3.92 (dd, J = 10.9, 4.2 Hz, 1H), 3.81 (t, J = 4.4 Hz, 1H), 0.90 (s, 9H), 0.11 (d, J = 0.6 Hz, 6H). ^{13}C NMR (126 MHz, $CDCl_3$) δ 145.46, 136.38, 132.08, 129.27, 128.05, 128.00, 127.59, 126.15, 124.61, 100.81, 80.55, 75.52, 73.13, 71.39, 64.33, 63.41, 18.45, -5.32, -5.35. **ESI-HRMS**: Calculated for $C_{22}H_{32}O_5NaSi$ $(M+Na)^+$: 427.19112, Found: 427.19132. $[\alpha]_D^{20}$ = +186.3 (c = 0.11, $CHCl_3$).

(*2R,3R*)-2-(((*tert*-butyldimethylsilyl)oxy)methyl)-4-(((*1R,2R*)-1-hydroxy-1,2-dihydronaphthalen-2-yl)oxy)-3,4-dihydro-2H-pyran-3-ol (19m)

The title product compound is prepared according to the general procedure **A** with 5 mol% $Rh(cod)_2OTf$ catalyst, 30 mol% cyclohexyl vinyl boronic acid **26**, oxabicycle **16a** (0.4 mmol, 2 equiv.), and 0.2 mmol carbohydrate polyol **15m,** in 0.5 ml THF at 50 ˚C for 24 h and isolated by flash column chromatography (15:1-2:1 Pentane: Ethyl Acetate) giving **19m** as a color less liquid, inseparable mixture. (43.6 mg, 54% yield, r.r. 5:1 (C3:C4), d.r. > 20:1(*trans*:*cis*)).

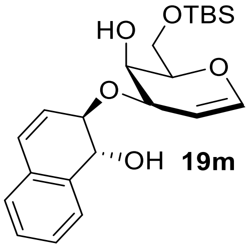

19m

^{1}H NMR (500 MHz, $CDCl_3$) δ 7.59 (d, J = 7.3 Hz, 1H), 7.27 – 7.19 (m, 2H), 7.07 (d, J = 6.9, 1.3 Hz 1H), 6.46 – 6.35 (m, 2H), 6.03 (dd, J = 9.9, 2.3 Hz, 1H), 4.96 (d, J = 10.4 Hz, 1H), 4.77 (dt, J = 6.3, 1.9 Hz, 1H), 4.45 – 4.42 (m, 2H), 4.15 (d, J = 2.8 Hz, 1H), 4.00 – 3.96 (m, 1H), 3.90– 3.85 (m, 2H), 0.91 (s, 9H), 0.10 (d, J = 1.6 Hz, 6H**). ^{13}C NMR** (126 MHz, $CDCl_3$) δ 145.37, 136.02, 132.02, 128.66, 128.32, 128.15, 127.96, 126.41, 125.24, 99.69, 79.89, 76.45, 72.85, 71.43, 64.58, 62.73, 26.01, 18.48, -5.27, -5.29. **ESI-HRMS**: Calculated for $C_{22}H_{32}O_5NaSi$ $(M+Na)^+$: 427.19112, Found: 427.19090. $[\alpha]_D^{20}$ = -84.1 (c = 1.52, $CHCl_3$).

(*2R,3S,4R,5R,6S*)-4-(((*1R,2R*)-1-hydroxy-1,2-dihydronaphthalen-2-yl)oxy)-2-methoxy-6-methyltetrahydro-2H-pyran-3,5-diol (17n)

The title product compound is prepared according to the general procedure **A** with 5 mol% $Rh(cod)_2OTf$ catalyst, 30 mol% cyclohexyl vinyl boronic acid **26**, oxabicycle **16a** (0.4 mmol, 2 equiv.), and 0.2 mmol carbohydrate polyol **15n,** in 0.5 ml THF at 50 ˚C for 12 h and isolated by flash column chromatography (9:1-1:15 Pentane: Ethyl Acetate) giving **17n** as a white solid. (47 mg, 73% yield, r.r. >20:1(C3:C4), d.r. > 20:1(*trans*:*cis*)).

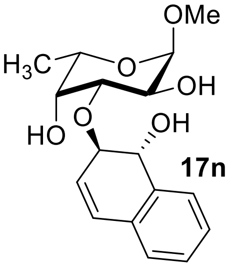

17n

^{1}H NMR (400 MHz, CD_3OD) δ 7.57 (d, J = 7.0 Hz, 1H), 7.26 – 7.19 (m, 2H), 7.08 – 7.06 (m, 1H), 6.41 (dd, J = 10.0, 2.2 Hz, 1H), 6.15 (dd, J = 9.9, 1.9 Hz, 1H), 4.90 (d, J = 11.8 Hz, 1H), 4.71 (d, J = 3.9 Hz, 1H), 4.39 (dt, J = 11.4, 2.1 Hz, 1H), 3.96-3.92 (m, 2H), 3.91-3.88 (m, 1H), 3.74 (dd, J = 10.0, 3.1 Hz, 1H), 3.41 (s, 3H), 1.28 (d, J = 6.6 Hz, 3H). **^{13}C NMR** (101 MHz, CD_3OD) δ 138.08, 133.86, 130.97, 128.61, 128.59, 128.33, 127.10, 125.62, 101.52, 81.28, 79.03, 74.13, 70.46, 68.65, 66.97, 55.58, 16.68. **ESI-HRMS**: Calculated for $C_{17}H_{22}O_6Na$ $(M+Na)^+$: 345.13086, Found: 345.13112 . $[\alpha]_D^{20}$ = -184.2 (c = 0.26, $CHCl_3$).

(*2R,3S,4R,5R,6S*)-2-(allyloxy)-4-(((*1R,2R*)-1-hydroxy-1,2-dihydronaphthalen-2-yl)oxy)-6-methyltetrahydro-2H-pyran-3,5-diol (17o)

The title product compound is prepared according to the general procedure **A** with 5 mol% Rh(cod)$_2$OTf catalyst, 30 mol% Cyclohexyl vinyl boronic acid **26**, oxabicycle **16a** (0.4 mmol, 2 equiv.), and 0.2 mmol Carbohydrate polyol **15o** at 50 ˚C for 24 h and isolated by flash column chromatography (9:1-1:15 Pentane: Ethyl Acetate) giving **17o** as a white solid. (42.8 mg, 61% yield, r.r. >20:1 (C3:C4), d.r. > 20:1(*trans*:*cis*)).

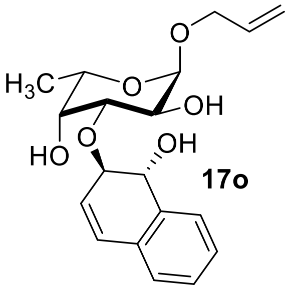
17o

^{1}H NMR (500 MHz, $CDCl_3$) δ 7.58 (d, *J* = 7.1 Hz, 1H), 7.26 – 7.20 (m, 2H), 7.06 – 7.04 (m, 1H), 6.39 (dd, *J* = 9.9, 2.3 Hz, 1H), 6.00 (dd, *J* = 9.9, 1.6 Hz, 1H), 5.97 – 5.89 (m, 1H), 5.30 (dq, *J* = 17.2, 1.4 Hz, 1H), 5.21 (dd, *J* = 10.3, 1.2 Hz, 1H), 5.04 (d, *J* = 11.8 Hz, 1H), 4.97 (d, *J* = 4.0 Hz, 1H), 4.46 (dt, *J* = 11.8, 2.0 Hz, 1H), 4.22-4.18 (m, 1H), 4.07– 4.03 (m, 2H), 3.97 – 3.93 (m, 2H), 3.79 (dd, *J* = 9.7, 3.2 Hz, 1H), 1.30 (d, *J* = 6.6 Hz, 3H). **^{13}C NMR** (126 MHz, $CDCl_3$) δ 136.33, 133.81, 132.31, 128.94, 128.49, 128.07, 127.80, 126.28, 124.44, 118.19, 97.70, 79.55, 77.10, 72.72, 69.30, 68.79, 67.69, 65.81, 16.41. **ESI-HRMS**: Calculated for $C_{19}H_{24}O_6Na$ $(M+Na)^+$: 371.14651, Found: 371.14680. $[\alpha]_D^{20}$ = -160.3 (c = 0.31, $CHCl_3$).

(*2R,3S,4R,5R,6S*)-2-(allyloxy)-4-(((*1S,2S*)-1-hydroxy-1,2-dihydronaphthalen-2-yl)oxy)-6-methyltetrahydro-2H-pyran-3,5-diol (19o)

The title product compound is prepared according to the general procedure **B** with 5 mol% Rh(cod)$_2$OTf catalyst, 30 mol% cyclohexyl vinyl boronic acid **26**, oxabicycle **16a** (0.4 mmol, 2 equiv.), and 0.2 mmol carbohydrate polyol **15o** at 50 ˚C for 24 h and isolated by flash column chromatography (9:1-1:15 Pentane: Ethyl Acetate) giving **19o** as a white solid. (20.9 mg, 30% yield, r.r. >20:1 (C3:C4), d.r. > 20:1(*trans*:*cis*)).

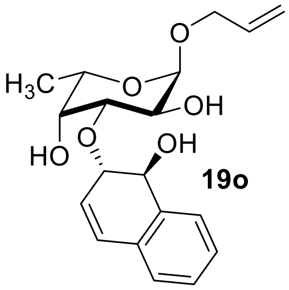
19o

^{1}H NMR (700 MHz, CD_3OD) δ 7.56 (d, *J* = 7.0 Hz, 1H), 7.25 – 7.22 (m, 2H), 7.09 (d, *J* = 6.8 Hz, 1H), 6.44 (dd, *J* = 9.9, 1.9 Hz, 1H), 6.09 (dd, *J* = 9.9, 2.1 Hz, 1H), 6.02-5.96 (m, 1H), 5.36 (dd, *J* = 17.2, 1.5 Hz, 1H), 5.20 (d, *J* = 10.4 Hz, 1H), 4.98 (d, *J* = 10.4 Hz, 1H), 4.87 (s, 1H, C1 carbon merged with CD_3OD) 4.47 (dt, *J* = 10.5, 2.0 Hz, 1H), 4.20 (dd, *J* = 13.0, 5.2 Hz, 1H), 4.07 (dd, *J* = 13.0, 6.0 Hz, 1H), 4.02-3.97 (m, 2H), 3.95 (dd, *J* = 10.2, 3.1 Hz, 1H), 3.86 (d, *J* = 2.5 Hz, 1H), 1.25 (d, *J* = 6.6 Hz, 3H). **^{13}C NMR** (176 MHz, CD_3OD) δ 137.98, 135.69, 133.64, 131.16, 128.66, 128.65, 128.61, 127.18, 126.10, 117.46,

99.53, 83.71, 80.94, 74.96, 72.33, 69.53, 69.29, 67.64, 16.57. **ESI-HRMS**: Calculated for $C_{19}H_{24}O_6Na$ $(M+Na)^+$: 371.14651, Found: 371.14656. $[\alpha]_D^{20}$ = -68.5 (c = 0.89, $CHCl_3$).

(*2S,3S,4S,5R*)-2-(allyloxy)-4-(((*1R,2R*)-1-hydroxy-1,2-dihydronaphthalen-2-yl)oxy)tetrahydro-2H-pyran-3,5-diol (17n)

(*1R,1'R,2R,2'R*)-2,2'-(((*2S,3S,4S,5R*)-2-(allyloxy)-5-hydroxytetrahydro-2H-pyran-3,4-diyl)bis(oxy))bis(1,2-dihydronaphthalen-1-ol) (19pa)

The title products are prepared according to the general procedure **C** with 5 mol% $Rh(cod)_2OTf$ catalyst, 30 mol% boronic acid **27**, oxabicycle **16a** (0.3 mmol, 1.5 equiv.), and 0.2 mmol carbohydrate polyol **15p,** at 50 ˚C for 24 h and isolated by flash column chromatography (9:1-1:15 Pentane: Ethyl Acetate) giving Major-**17p** as a white solid. (40.3 mg, 60% yield, r.r. >20:1 (C3:C2), d.r. > 20:1(*trans*:*cis*)) and difunctionalized **19pa** as a minor white solid. (5.9 mg, 6% yield, d.r. > 20:1(*trans*:*cis*)).

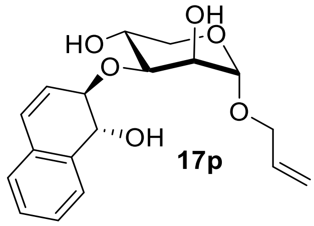

17p

^{1}H NMR (600 MHz, CD_3OD) δ 7.56 (d, *J* = 7.2 Hz, 1H), 7.25 – 7.20 (m, 2H), 7.08 – 7.07 (m, 1H), 6.43 (dd, *J* = 9.9, 2.2 Hz, 1H), 6.11 (dd, *J* = 9.9, 2.0 Hz, 1H), 5.98 – 5.91 (m, 1H), 5.31 (dq, *J* = 17.2, 1.7 Hz, 1H), 5.18 (dq, *J* = 10.5, 1.3 Hz, 1H), 4.89 (d, *J* = 11.2 Hz, 1H), 4.80 (d, *J* = 2.8 Hz, 1H), 4.37 (dt, *J* = 11.2, 2.2 Hz, 1H), 4.22-4.19 (m, 1H), 4.03 – 4.00 (m, 2H), 3.96-3.93 (m, 1H), 3.74 – 3.70 (m, 2H), 3.54 (dd, *J* = 11.0, 9.5 Hz, 1H). **^{13}C NMR** (151 MHz, CD_3OD) δ 137.96, 135.41, 133.76, 130.76, 128.65, 128.55, 127.17, 125.75, 117.49, 100.82, 82.29, 80.71, 74.29, 69.40, 69.27, 67.23, 64.24. **ESI-HRMS**: Calculated for $C_{18}H_{22}O_6Na$ $(M+Na)^+$: 357.13086, Found: 357.13104. $[\alpha]_D^{20}$ = -34.0 (c = 0.22, $CHCl_3$).

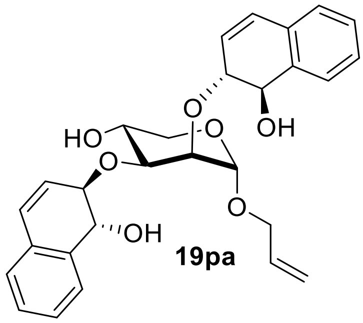

19pa

^{1}H NMR (600 MHz, CD_3OD) δ 7.54 (t, *J* = 6.2 Hz, 2H), 7.25 – 7.20 (m, 4H), 7.08 – 7.07 (m, 2H), 6.44 (ddd, *J* = 9.9, 4.5, 2.2 Hz, 2H), 6.12 (dd, *J* = 9.9, 2.2 Hz, 2H), 5.99-5.93 (m, 1H), 5.32 (dq, *J* = 17.2, 1.7 Hz, 1H), 5.19 (dq, *J* = 10.5, 1.4 Hz, 1H), 4.99 (d, *J* = 2.0 Hz, 1H), 4.86 (s, 1H merged with CD_3OD), 4.80 (d, *J* = 10.4 Hz, 1H), 4.41 (dt, *J* = 10.5, 2.3 Hz, 1H), 4.37 (dt, *J* = 10.4, 2.2 Hz, 1H), 4.23 (ddt, *J* = 13.0, 5.1, 1.5 Hz, 1H), 4.04 (ddt, *J* = 13.0, 5.8, 1.4 Hz, 1H), 3.93-3.91 (m, 4H), 3.61 – 3.57 (m, 1H), 3.35 (s, 1H). **^{13}C NMR** (151 MHz, CD_3OD) δ 138.25, 138.11, 135.44, 133.83, 133.80, 130.67, 130.40, 128.84, 128.74 (two carbon), 128.71, 128.68 (two carbon), 127.23, 127.22, 126.29, 126.19, 117.35, 99.77, 83.99, 82.99, 79.71, 76.73, 74.05 (two carbon), 71.58, 69.42, 62.50. **ESI-HRMS**: Calculated for $C_{28}H_{30}O_7Na$ $(M+Na)^+$: 501.18837, Found: 501.18777. $[\alpha]_D^{20}$ = -94.3 (c = 0.32, $CHCl_3$).

(*2S,3S,4S,5R*)-2-(allyloxy)-4-(((*1S,2S*)-1-hydroxy-1,2-dihydronaphthalen-2-yl)oxy)tetrahydro-2H-pyran-3,5-diol (19n) and **(*3R,4S,5S,6S*)-6-(allyloxy)-5-(((*1S,2S*)-1-hydroxy-1,2-dihydronaphthalen-2-yl)oxy)tetrahydro-2H-pyran-3,4-diol (20p)**

(*1S,1'S,2S,2'S*)-2,2'-(((*2S,3S,4S,5R*)-2-(allyloxy)-5-hydroxytetrahydro-2H-pyran-3,4-diyl)bis(oxy))bis(1,2-dihydronaphthalen-1-ol) (20pa)

The title products are prepared according to the general procedure **D** with 5 mol% Rh(cod)$_2$OTf catalyst, 30 mol% boronic acid **27**, oxabicycle **16a** (0.3 mmol, 1.5 equiv.), and 0.2 mmol carbohydrate polyol **15p,** at 50 ˚C for 24 h and isolated by flash column chromatography (9:1-1:15 Pentane: Ethyl Acetate) giving inseparable mixture of **19p** and **20p** as a white solid. (37.4 mg, 56% yield, r.r. 1.37:1 (C3:C2), d.r. > 20:1(*trans*:*cis*)) and minor **20pa** as a white solid. (26.9 mg, 28% yield, d.r. > 20:1(*trans*:*cis*)).

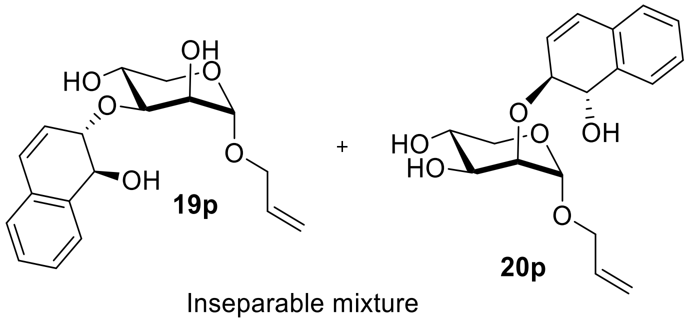

Inseparable mixture

^{1}H NMR (500 MHz, CD$_3$OD) δ 7.52 – 7.50 (m, 2H), 7.25 – 7.17 (m, 4H), 7.06 – 7.04 (m, 2H), 6.39 (td, *J* = 9.3, 8.5, 2.2 Hz, 2H), 6.04 (dd, *J* = 9.9, 2.2 Hz, 1H), 5.96 – 5.88 (m, 3H), 5.31-5.29 (m, 1H), 5.27-5.25 (m, 1H), 5.17-5.14 (m, 2H), 4.93 (d, *J* = 10.7 Hz, 1H), 4.89 (d, *J* = 11.3 Hz, 1H), 4.83 (d, *J* = 2.7 Hz, 1H), 4.73 (d, *J* = 2.7 Hz, 1H), 4.40-4.34 (m, 2H), 4.21-4.15 (m, 2H), 4.05 – 3.96 (m, 3H), 3.90 (t, *J* = 3.0 Hz, 1H), 3.88 – 3.77 (m, 4H), 3.72-3.66 (m, 2H), 3.51-3.43 (m, 2H). **^{13}C NMR** (126 MHz, CD$_3$OD) δ 138.03, 137.94, 135.43, 135.40, 133.65, 133.55, 131.03, 130.91, 128.69, 128.67, 128.66 (two carbon), 128.65, 128.64, 127.22, 127.19, 125.95, 125.80, 117.50, 117.25, 101.03, 100.11, 85.34, 84.13, 81.96, 81.33, 74.93 (two carbon), 72.73, 70.71, 69.30, 69.22, 68.95, 67.58, 64.32, 64.18. **ESI-HRMS**: Calculated for $C_{18}H_{22}O_6Na$ (M+Na)$^+$: 357.13086, Found: 357.13094. $[\alpha]_D^{20}$ = + 81.8 (c = 0.66, $CHCl_3$).

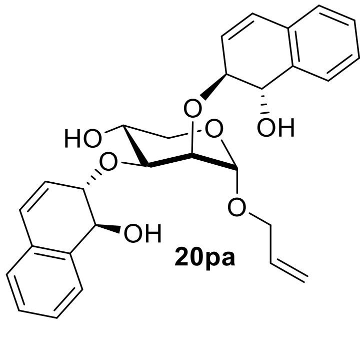

^{1}H NMR (500 MHz, CD$_3$OD) δ 7.52 – 7.50 (m, 2H), 7.23 – 7.17 (m, 4H), 7.06 – 7.03 (m, 2H), 6.40 (ddd, *J* = 14.1, 9.9, 2.1 Hz, 2H), 5.96 – 5.88 (m, 3H), 5.29 (dq, *J* = 17.2, 1.7 Hz, 1H), 5.16 (dq, *J* = 10.5, 1.3 Hz, 1H), 4.90 (d, *J* = 10.7 Hz, 1H), 4.85 (d, 1H, merged with CD$_3$OD , 4.82 (d, *J* = 2.9 Hz, 1H), 4.37 (dt, *J* = 10.4, 2.2 Hz, 2H), 4.20 (ddt, *J* = 13.0, 5.1, 1.5 Hz, 1H), 4.02 – 3.98 (m, 2H), 3.97-3.92 (m, 1H), 3.89 (t, *J* = 3.1 Hz, 1H), 3.79 (dd, *J* = 11.2, 4.8 Hz, 1H), 3.52 (dd, *J* = 11.1, 9.0 Hz, 1H). **^{13}C NMR** (126 MHz, CD$_3$OD) δ 137.95, 137.93, 135.42, 133.58, 133.56, 130.81, 130.53, 128.97, 128.77 (two carbon), 128.74, 128.72, 128.69, 127.30, 127.24, 126.36, 125.91, 117.32, 99.92, 85.23, 83.59, 81.23, 78.12, 74.99, 74.60, 72.00, 69.41, 62.95. **ESI-HRMS**: Calculated for $C_{28}H_{30}O_7Na$ (M+Na)$^+$: 501.18837, Found: 501.18780. $[\alpha]_D^{20}$ = +95.0 (c = 0.92, $CHCl_3$).

(*1R,2S,3S,4S,5R*)-4-(((*1S,2S)*-1-hydroxy-1,2-dihydronaphthalen-2-yl)oxy)-6,8-dioxabicyclo[3.2.1]octane-2,3-diol (17q)

The title product compound is prepared according to the general procedure **B** with 5 mol% Rh(cod)$_2$OTf catalyst, 30 mol% cyclohexyl vinyl boronic acid **26**, oxabicycle **16a** (0.4 mmol, 2 equiv.), and 0.2 mmol carbohydrate polyol **15q,** in 0.5 ml THF at 50 ˚C for 24 h and isolated by flash column chromatography (9:1-1:15 Pentane: Ethyl Acetate) giving **17q** as a sticky brown solid. (44.2 mg, 72% yield, r.r. >1:20 (C3:C2), d.r. > 20:1(*trans*:*cis*)).

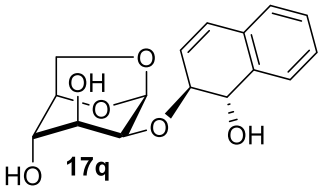

17q

^{1}H NMR (500 MHz, CD_3OD) δ 7.51 (d, *J* = 7.1 Hz, 1H), 7.24 – 7.18 (m, 2H), 7.06 (dd, *J* = 6.9, 1.9 Hz, 1H), 6.45 (dd, *J* = 9.9, 2.2 Hz, 1H), 6.00 (dd, *J* = 9.9, 2.1 Hz, 1H), 5.39 (s, 1H), 4.84 (d, 1H merged with CD_3OD), 4.46 (d, *J* = 5.1 Hz, 1H), 4.32 (dt, *J* = 11.0, 2.1 Hz, 1H), 4.22 (d, *J* = 7.1 Hz, 1H), 4.06 – 4.05 (m, 1H), 3.81 (s, 1H), 3.74 (dd, *J* = 4.9, 1.9 Hz, 1H), 3.67 (t, *J* = 6.5 Hz,1H). **^{13}C NMR** (126 MHz, CD_3OD) δ 138.00, 133.55, 129.36, 129.21, 128.83, 128.76, 127.31, 125.87, 102.30, 80.48, 77.79, 74.20, 73.88, 72.98, 70.21, 65.81. **ESI-HRMS**: Calculated for $C_{16}H_{18}O_6Na$ $(M+Na)^+$: 329.09956, Found: 329.09972. $[\alpha]_D^{20}$ = +26.3 (c = 0.46, CH_3OH).

(*1R,2S,3S,4S,5R*)-4-(((*1R,2R*)-1-hydroxy-1,2-dihydronaphthalen-2-yl)oxy)-6,8-dioxabicyclo[3.2.1]octane-2,3-diol (18q)

(*1R,2R,3S,4S,5R*)-3-(((*1R,2R*)-1-hydroxy-1,2-dihydronaphthalen-2-yl)oxy)-6,8-dioxabicyclo[3.2.1]octane-2,4-diol (19q)

The title products are prepared according to the general procedure **A** with 5 mol% Rh(cod)$_2$OTf catalyst, 30 mol% cyclohexyl vinyl boronic acid **26**, oxabicycle **16a** (0.4 mmol, 2 equiv.), and 0.2 mmol carbohydrate polyol **15q,** in 0.5 ml THF at 50 ˚C for 24 h and isolated by flash column chromatography (9:1-1:15 Pentane: Ethyl Acetate) giving the C2 major regioisomer **18q** (17 mg, dr > 20:1(*trans*:*cis*)) as a sticky brown solid and the C3 minor regioisomer **19q** (9.9 mg , d.r. > 20:1(*trans*:*cis*)) as a sticky brown solid (26.9 mg combined mass of **18q** and **19q**, 44% yield of **18q** and **19q**, r.r. 1:1.7 **(**C3:C2), d.r. > 20:1(*trans*:*cis*)).

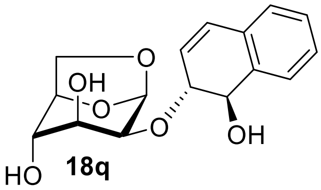

18q

^{1}H NMR (700 MHz, CD_3OD) δ 7.58 (d, *J* = 7.3 Hz, 1H), 7.28 – 7.23 (m, 2H), 7.11 (dd, *J* = 7.1, 1.6 Hz, 1H), 6.49 (dd, *J* = 9.9, 2.0 Hz, 1H), 6.05 (dd, *J* = 9.9, 2.4 Hz, 1H), 5.56 (s, 1H), 4.91 (d, *J* = 10.1 Hz, 1H), 4.49 (d, *J* = 5.2 Hz, 1H), 4.43 (dt, *J* = 10.2, 2.2 Hz, 1H), 4.28 (d, *J* = 7.2 Hz, 1H), 4.03 – 4.02 (m, 1H), 3.88 (dd, *J* = 5.1, 1.7 Hz, 1H), 3.83 (s, 1H), 3.71 (t, *J* = 6.7 Hz, 1H). **^{13}C NMR** (176 MHz, CD_3OD) δ 138.05, 133.59, 129.51, 129.36, 128.85, 128.82, 127.34, 126.35, 101.64, 81.75, 77.70, 75.04, 74.08, 73.41, 72.11, 65.93. **ESI-HRMS**: Calculated for $C_{16}H_{18}O_6Na$ $(M+Na)^+$: 329.09956, Found: 329.09968. $[\alpha]_D^{20}$ = -100.2 (c = 0.35, CH_3OH).

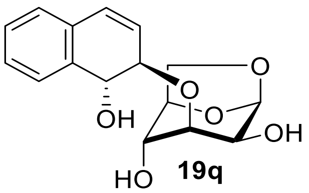

19q

^{1}H NMR (700 MHz, CD_3OD) δ 7.53 (d, *J* = 7.1 Hz, 1H), 7.27 – 7.22 (m, 2H), 7.11 – 7.10 (m, 1H), 6.48 (dd, *J* = 9.9, 2.1 Hz, 1H), 6.04 (dd, *J* = 9.9, 2.3 Hz, 1H), 5.27 (s, 1H), 4.94 (d, *J* = 10.6 Hz, 1H), 4.48 (d, *J* = 5.0 Hz, 1H), 4.35 (dt, *J* = 10.6, 2.2 Hz, 1H), 4.26 (d, *J* = 7.2 Hz, 1H), 3.96 (s, 1H), 3.89 – 3.88 (m, 1H), 3.75 (dd, *J* = 5.5, 1.9 Hz, 1H), 3.70 (t, *J* = 6.3 Hz,1H). **^{13}C NMR** (176 MHz, CD_3OD) δ 138.24, 133.48, 129.78, 129.21, 128.80, 128.73, 127.31, 126.11, 103.40, 85.58, 81.45, 77.29, 74.31, 72.78, 68.37, 65.77. **ESI-HRMS**: Calculated for $C_{16}H_{18}O_6Na$ $(M+Na)^+$: 329.09956, Found: 329.09972. $[\alpha]_D^{20}$ = -93.6 (c = 0.49, CH_3OH)

(*1R,2R,3R,4S,5R*)-5-(((*tert*-butyldimethylsilyl)oxy)methyl)-3-(((*1R,2R*)-1-hydroxy-1,2-dihydronaphthalen-2-yl)oxy)-6,8-dioxabicyclo[3.2.1]octane-2,4-diol (17r)

(*1R,2S,3S,4S,5R*)-5-(((*tert*-butyldimethylsilyl)oxy)methyl)-2-(((*1R,2R*)-1-hydroxy-1,2-dihydronaphthalen-2-yl)oxy)-6,8-dioxabicyclo[3.2.1]octane-3,4-diol (18r)

The title products are prepared according to the general procedure **A** with 5 mol% $Rh(cod)_2OTf$ catalyst, 30 mol% cyclohexyl vinyl boronic acid **26**, oxabicycle **16a** (0.4 mmol, 2 equiv.), and 0.2 mmol carbohydrate polyol **15r** at 50 ˚C for 24 h and isolated by flash column chromatography (9:1-1:15 Pentane: Ethyl Acetate) giving the C3 major regioisomer **17r** (58.6 mg, d.r. > 20:1(*trans*:*cis*)) as a white solid and the C2 minor regioisomer **18r** (7 mg, d.r. > 20:1(*trans*:*cis*)) as a white solid (65.6 mg combined mass of **17r** and **18r**, 73% combined yield of **17r** and **18r**, r.r. 6:1 (C3:C2)).

.

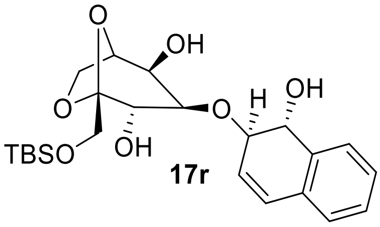

17r

^{1}H NMR (500 MHz, $CDCl_3$) δ 7.58 (d, *J* = 7.1 Hz, 1H), 7.26 – 7.20 (m, 2H), 7.05 (d, *J* = 6.8 Hz 1H), 6.40 (d, *J* = 9.9 Hz, 1H), 5.99 (dd, *J* = 9.9, 1.7 Hz, 1H), 5.05 (d, *J* = 11.8 Hz, 1H), 4.69 (dd, *J* = 5.1, 1.5 Hz, 1H), 4.39 (d, *J* = 11.8 Hz, 1H), 4.24 (bs, 1H), 4.06 (dd, *J* = 8.4, 3.7 Hz, 1H), 4.02 (s, 1H), 3.99 (d, *J* = 11.1 Hz, 1H), 3.86 – 3.82 (m, 2H), 3.74-3.70 (m, 2H), 3.55 (bs, 1H), 3.16 (bs, 1H), 0.91 (s, 9H), 0.11 (d, *J* = 1.3 Hz, 6H). **^{13}C NMR** (126 MHz, $CDCl_3$) δ 136.26, 132.21, 128.66, 128.59, 128.07, 127.78, 126.31, 124.43, 107.03, 80.70, 77.55, 77.49, 72.80, 72.03, 68.02, 66.34, 64.77, 26.01, 18.48, -5.31, -5.33. **ESI-HRMS**: Calculated for $C_{23}H_{34}O_7NaSi$ $(M+Na)^+$: 473.19660, Found: 473.19607. $[\alpha]_D^{20}$ = -98.8 (c = 0.36, $CHCl_3$).

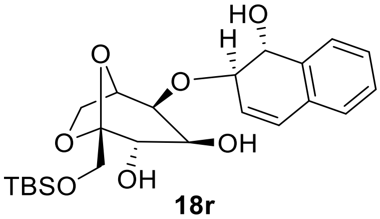

18r

^{1}H NMR (500 MHz, $CDCl_3$) δ 7.64 (d, *J* = 7.3 Hz, 1H), 7.30 – 7.28 (m, 1H), 7.24-7.21 (m, 1H), 7.07 (d, *J* = 7.2 Hz, 1H), 6.44 (dd, *J* = 9.8, 2.0 Hz, 1H), 5.95 (d, *J* = 9.8 Hz, 1H), 4.99 (d, *J* = 11.4 Hz, 1H), 4.81 (d, *J* = 4.5 Hz, 1H), 4.44 (d, *J* = 11.4 Hz, 1H), 4.03 (d, *J* = 11.2 Hz, 1H), 3.95 (d, *J* = 5.8 Hz, 1H), 3.88 – 3.85 (m, 2H), 3.82 – 3.74 (m, 3H), 3.52 (s, 1H), 2.74 (s, 2H), 0.92 (s, 9H), 0.12 (d, *J* = 6.8 Hz, 6H). **^{13}C NMR** (126 MHz, $CDCl_3$) δ 135.63, 131.96, 129.11, 128.27, 127.83, 127.78, 126.36, 124.70, 107.68, 81.26, 76.47, 74.53, 74.33, 72.80, 70.83, 66.02, 63.89, 25.94, 18.42, -5.22, -5.26. **ESI-HRMS**: Calculated for $C_{23}H_{34}O_7NaSi$ $(M+Na)^+$: 473.19660, Found: 473.19611. $[\alpha]_D^{20}$ = -25.2 (c = 0.31, $CHCl_3$).

(*1R,2R,3R,4S,5R*)-5-(((*tert*-butyldimethylsilyl)oxy)methyl)-3-(((*1S,2S*)-1-hydroxy-1,2-dihydronaphthalen-2-yl)oxy)-6,8-dioxabicyclo[3.2.1]octane-2,4-diol (19r)

(*1R,2S,3S,4S,5R*)-5-(((*tert*-butyldimethylsilyl)oxy)methyl)-2-(((*1S,2S*)-1-hydroxy-1,2-dihydronaphthalen-2-yl)oxy)-6,8-dioxabicyclo[3.2.1]octane-3,4-diol (20r)

The title products are prepared according to the general procedure **B** with 5 mol% Rh(cod)$_2$OTf catalyst, 30 mol% cyclohexyl vinyl boronic acid **26**, oxabicycle **16a** (0.4 mmol, 2 equiv.), and 0.2 mmol carbohydrate polyol **15r** at 50 ˚C for 24 h and isolated by flash column chromatography (9:1-1:15 Pentane: Ethyl Acetate) giving major C3 regioisomer **19r** (43.9 mg, d.r. > 20:1(*trans*:*cis*)) as a white solid and minor C2 regioisomer **20r** (8.4 mg, d.r. > 20:1(*trans*:*cis*)) as a white solid (52.3 mg combined mass of **19r** and **20r**, 58% combined yield of **19r** and **20r**, r.r. 5.2:1 (C3:C2)).

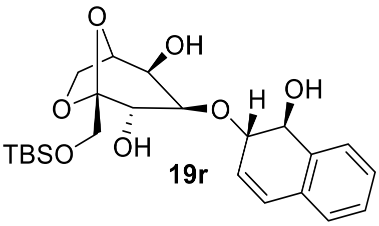

19r

^{1}H NMR (600 MHz, CD_3OD) δ 7.55 (d, *J* = 7.0 Hz, 1H), 7.25 – 7.21 (m, 2H), 7.09 – 7.08 (m, 1H), 6.44 (dd, *J* = 9.9, 2.2 Hz, 1H), 6.12 (dd, *J* = 9.9, 2.4 Hz, 1H), 4.97 (d, *J* = 10.4 Hz, 1H), 4.58 (dd, *J* = 5.1, 2.1 Hz, 1H), 4.47 (dt, *J* = 10.4, 2.3 Hz, 1H), 4.02 – 3.89 (m, 2H), 3.97 (d, *J* = 8.7 Hz, 1H), 3.83 (dd, *J* = 8.7, 4.5 Hz, 1H), 3.81 – 3.79 (m, 1H), 3.75 (dd, *J* = 7.9, 5.6 Hz, 1H), 3.71 (d, *J* = 11.2 Hz, 1H), 0.94 (s, 9H), 0.13 (d, *J* = 1.8 Hz, 6H). **^{13}C NMR** (151 MHz, CD_3OD) δ 137.96, 133.64, 130.87, 128.69, 128.67 (two carbon), 127.19, 126.12, 109.26, 83.72, 81.26, 79.34, 74.83, 72.55, 70.71, 67.20, 63.68, 26.42, 19.32, -5.18, -5.21. **ESI-HRMS**: Calculated for $C_{23}H_{34}O_7NaSi$ $(M+Na)^+$: 473.19660, Found: 473.19595. $[\alpha]_D^{20}$ = +2.6 (c = 1.15, $CHCl_3$**).**

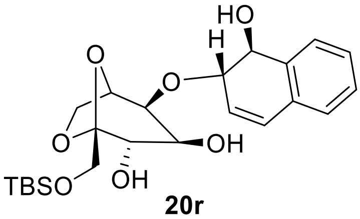

20r

^{1}H NMR (600 MHz, CD_3OD) δ 7.54 (d, *J* = 7.1 Hz, 1H), 7.25 – 7.20 (m, 2H), 7.09 – 7.07 (m, 1H), 6.42 (dd, *J* = 10.0, 2.1 Hz, 1H), 6.07 (dd, *J* = 9.9, 2.0 Hz, 1H), 4.97 (d, *J* = 11.1 Hz, 1H), 4.72-4.71 (m, 1H), 4.45 (dt, *J* = 11.2, 2.2 Hz, 1H), 4.01 (d, *J* = 11.2 Hz, 1H), 3.95 (dd, *J* = 4.5, 2.4 Hz, 1H), 3.81 (d, *J* = 8.8 Hz, 1H), 3.78 – 3.75 (m, 3H), 3.71 (d, *J* = 11.3 Hz, 1H), 0.94 (s, 9H), 0.13 (d, *J* = 6.4 Hz, 6H). **^{13}C NMR** (151 MHz, CD_3OD) δ 138.30, 133.62, 131.19, 128.67, 128.62, 128.56, 127.19, 125.72, 109.35, 84.84, 81.35, 78.43, 74.72, 74.14, 72.80, 67.25, 63.72, 26.44, 19.38, -5.12, -5.16. **ESI-HRMS**: Calculated for $C_{23}H_{34}O_7NaSi$ $(M+Na)^+$: 473.19660, Found: 473.19605. $[\alpha]_D^{20}$ = +7.9 (c = 0.53, $CHCl_3$).

(*2R,4aR,6S,7R,8R,8aR*)-8-((tert-butyldiphenylsilyl)oxy)-6-(((*1R,2R*)-1-hydroxy-1,2-dihydronaphthalen-2-yl)oxy)-2-phenylhexahydropyrano[3,2-d][1,3]dioxin-7-ol(17s)

The title product compound is prepared according to the general procedure **A1** with 5 mol% Rh(cod)$_2$OTf catalyst, 30 mol% Taylor's boronic acid **27**, oxabicycle **16a** (0.4 mmol, 2 equiv.), and 0.2 mmol carbohydrate polyol **15s** at 50 ˚C for 2 h and isolated by flash column chromatography (10:1-5:1 Pentane: Ethyl Acetate) giving **17s** as a yellow solid (98.0 mg, 75% yield, r.r. >20:1 (C1:C2), d.r. > 20:1(*trans*:*cis*)).

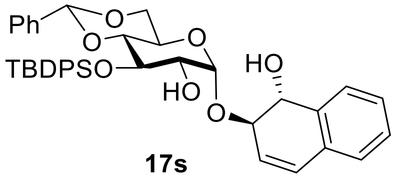

17s

^{1}H NMR (600 MHz, CDCl$_3$) δ 7.68 – 7.61 (m, 4H), 7.56 (d, J = 7.8 Hz, 1H), 7.45 – 7.34 (m, 2H), 7.34 – 7.29 (m, 3H), 7.29 – 7.26 (m, 3H), 7.26 – 7.20 (m, 3H), 7.15 – 7.12 (m, 2H), 7.05 (d, J = 7.2 Hz, 1H), 6.35 (dd, J = 10.2, 2.4 Hz, 1H), 5.85 (dd, J = 9.6, 1.8 Hz, 1H), 5.25 (s, 1H), 5.12 (d, J = 4.2 Hz, 1H), 4.86 (d, J = 11.4 Hz, 1H), 4.38 (d, J = 10.8 Hz, 1H), 4.23 (dd, J = 10.2, 4.8 Hz, 1H), 4.18 (t, J = 9.0 Hz, 1H), 4.00 – 3.91 (m, 1H), 3.86 – 3.78 (m, 2H), 3.67 (t, J = 10.2 Hz, 1H), 3.55 (t, J = 9.6 Hz, 1H), 2.31 (d, J = 5.4 Hz, 1H), 1.03 (s, 9H). **^{13}C NMR** (151 MHz, CDCl$_3$) δ 137.16, 136.09, 136.03, 136.03, 136.02, 133.79, 133.78, 132.14, 129.96, 129.79, 129.38, 128.92, 128.07, 128.05, 127.89, 127.75, 127.61, 126.40, 126.32, 124.48, 101.80, 101.72, 85.57, 81.45, 74.56, 74.05, 73.55, 69.04, 63.49, 27.12, 19.69. **ESI-HRMS**: Calculated for $C_{39}H_{42}ONaSi$ (M+Na)$^+$: 673.25920, Found: 673.25810. $[\alpha]_D^{20}$ = -13.9 (c =0.18, CHCl$_3$).

(*2R,4aR,6S,7R,8R,8aR*)-8-((tert-butyldiphenylsilyl)oxy)-6-(((*1S,2S*)-1-hydroxy-1,2-dihydronaphthalen-2-yl)oxy)-2-phenylhexahydropyrano[3,2-d][1,3]dioxin-7-ol(19s)

(*2R,4aR,7R,8S,8aR*)-8-((tert-butyldiphenylsilyl)oxy)-7-(((*1S,2S*)-1-hydroxy-1,2-dihydronaphthalen-2-yl)oxy)-2-phenylhexahydropyrano[3,2-d][1,3]dioxin-6-ol (20s)

The title product compound is prepared according to the general procedure **B1** with 5 mol% Rh(cod)$_2$OTf catalyst, 30 mol% Taylor's boronic acid **27**, oxabicycle **16a** (0.4 mmol, 2 equiv.), and 0.2 mmol carbohydrate polyol **15s** at 50 ˚C for 16 h and isolated by flash column chromatography (10:1-5:1 Pentane: Ethyl Acetate) giving C1 regioisomer **19s** (33.0 mg, d.r. > 20:1(*trans*:*cis*)) as a yellow solid and C2 regioisomer **20s** (34.0 mg, d.r. > 20:1(*trans*:*cis*)) as a yellow solid (67 mg combined total mass of **19s** and **20s**, 51% combined yield of **19s** and **20s**, r.r. 1:1(C1:C2)).

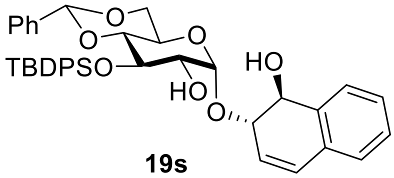

19s

^{1}H NMR (700 MHz, CDCl$_3$) δ 7.68 – 7.61 (m, 4H), 7.56 (d, J = 7.7 Hz, 1H), 7.42 – 7.37 (m, 1H), 7.37 – 7.34 (m, 1H), 7.33 – 7.28 (m, 3H), 7.28 – 7.26 (m, 2H), 7.25 – 7.21 (m, 4H), 7.16 – 7.12 (m, 2H), 7.05 (d, J = 7.0 Hz, 1H), 6.42 (dd, J = 10.5, 2.1 Hz, 1H), 5.78 (dd, J = 9.1, 2.1 Hz, 1H), 5.30 (s, 1H), 5.13 (d, J = 4.2 Hz, 1H), 4.97 – 4.84 (m, 1H), 4.47 (dt, J = 10.5, 2.1 Hz, 1H), 4.25 (dd, J = 9.8, 4.9 Hz, 1H), 4.03 (t, J = 9.1 Hz, 1H), 3.93 (td, J = 9.8, 4.9 Hz, 1H), 3.77 (td, J = 8.4, 4.2 Hz, 1H), 3.70 (t, J = 10.5 Hz, 1H), 3.59 (t, J = 9.1 Hz, 1H), 3.39 (d, J = 3.5 Hz, 1H), 1.76 (d, J = 8.4 Hz, 1H), 1.03 (s, 9H). **^{13}C NMR** (176 MHz, CDCl$_3$) δ 137.08, 136.28, 136.07, 135.51, 134.19, 133.73, 131.87, 129.79, 129.62, 129.21, 128.94, 128.37, 128.07, 128.04, 127.64, 127.48, 126.90, 126.47, 126.44, 125.34, 101.75, 98.68, 82.83, 81.38, 73.71, 73.57, 72.72, 68.81, 63.73, 27.13, 19.76. **ESI-HRMS**: Calculated for $C_{39}H_{42}ONaSi$ (M+Na)$^+$: 673.25920, Found: 673.25823. $[\alpha]_D^{20}$ = +175.0 (c =0.22, CHCl$_3$).

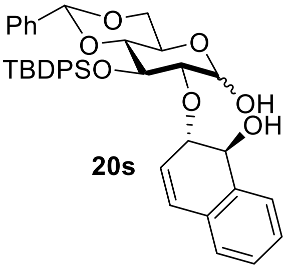

20s

^{1}H NMR (700 MHz, $CDCl_3$) δ 7.67 – 7.55 (m, 4H), 7.48 (d, J = 7.7 Hz, 2H), 7.41 – 7.30 (m, 2H), 7.28 – 7.26 (m, 3H), 7.25 – 7.20 (m, 3H), 7.20 – 7.15 (m, 2H), 7.15 – 7.10 (m, 2H), 7.08 – 7.00 (m, 2H), 6.65 (d, J = 7.7 Hz, 2H), 6.42 – 6.32 (m, 2H), 6.26 (d, J = 9.8 Hz, 1H), 5.97 (d, J = 10.5 Hz, 1H), 5.11 (d, J = 11.9 Hz, 1H), 4.99 (d, J = 11.9 Hz, 2H), 4.82 (d, J = 7.0 Hz, 1H), 4.72 (s, 1H), 4.57 (d, J = 10.5 Hz, 1H), 4.19 – 4.11 (m, 2H), 4.03 (t, J = 9.1 Hz, 1H), 3.90 (t, J = 7.7 Hz, 1H), 3.70 (s, 1H), 3.57 (t, J = 9.8 Hz, 1H), 3.51 (t, J = 9.1 Hz, 1H),), 3.22 – 3.13 (m, 1H), 0.93 (s, 7H). **^{13}C NMR** (176 MHz, $CDCl_3$) δ 136.60, 136.37, 136.21, 135.94, 135.40, 135.32, 132.63, 132.59, 132.26, 129.81, 129.56, 129.44, 129.18, 128.79, 128.26, 128.02, 127.88, 127.85, 127.83, 127.77, 127.40, 127.26, 126.60, 126.26, 126.16, 124.95, 124.49, 105.09, 101.76, 84.55, 82.70, 80.96, 80.81, 75.58, 74.52, 73.17, 68.59, 66.07, 26.98, 19.84. **ESI-HRMS**: Calculated for $C_{39}H_{42}ONaSi$ $(M+Na)^+$: 673.25920, Found: 673.25888. $[\alpha]_D^{20}$ = +84.2 (c =0.19, $CHCl_3$).

(*4aR,6S,7R,8R,8aR*)-2,2-di-tert-butyl-8-((tert-butyldiphenylsilyl)oxy)hexahydropyrano[3,2-d][1,3,2]dioxasiline-6,7-diol(17t)

The title product compound is prepared according to the general procedure **A1** with 5 mol% $Rh(cod)_2OTf$ catalyst, 30 mol% Taylor's boronic acid **27**, oxabicycle **16a** (0.4 mmol, 2 equiv.), and 0.2 mmol carbohydrate polyol **15t** at 50 ˚C for 2 h and isolated by flash column chromatography (10:1-5:1 Pentane: Ethyl Acetate) giving **17t** as a yellow solid (91.0 mg, 65% yield, r.r. >20:1 (C1:C2), d.r. > 20:1(*trans*:*cis*)).

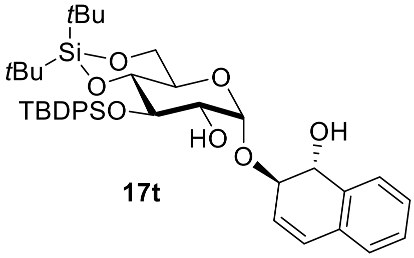

17t

^{1}H NMR (600 MHz, $CDCl_3$) δ 7.83 – 7.80 (m, 2H), 7.75 – 7.70 (m, 2H), 7.50 (d, J = 7.2 Hz, 1H), 7.46 – 7.34 (m, 6H), 7.25 – 7.18 (m, 2H), 7.07 – 6.97 (m, 1H), 6.32 (dd, J = 10.2, 2.4 Hz, 1H), 5.79 (dd, J = 9.6, 1.8 Hz, 1H), 4.95 (d, J = 3.6 Hz, 1H), 4.73 (d, J = 11.4 Hz, 1H), 4.28 (td, J = 11.4 Hz, J = 2.4 Hz 1H), 4.12 – 4.06 (m, 1H), 3.98 – 3.93 (m, 1H), 3.91 – 3.84 (m, 3H), 3.79 (d, J = 2.4 Hz, 1H), 3.68 – 3.62 (m, 1H), 1.90 (d, J = 5.4 Hz, 1H), 1.12 (s, 9H), 1.10 (s, 9H), 0.98 (s, 9H). **^{13}C NMR** (151 MHz, $CDCl_3$) δ 136.51, 136.12, 135.51, 135.20, 132.74, 132.08, 130.12, 130.01, 129.44, 128.14, 128.00, 127.95, 127.68, 126.26, 124.52, 101.51, 85.39, 77.82, 76.25, 73.98, 73.86, 67.41, 66.87, 27.74, 27.20, 27.15, 22.92, 20.08, 19.88. **ESI-HRMS**: Calculated for $C_{40}H_{54}O_7NaSi_2$ $(M+Na)^+$: 725.33003, Found: 725.32924. $[\alpha]_D^{20}$ = -7.7 (c =0.43, $CHCl_3$).

(*5aR,8S,9S,9aR*)-8-(((*1R,2R*)-1-hydroxy-1,2-dihydronaphthalen-2-yl)oxy)-2,2,4,4-tetraisopropyltetrahydro-6H-pyrano[3,4-f][1,3,5,2,4]trioxadisilepin-9-ol(17u)

The title product compound is prepared according to the general procedure **A1** with 5 mol% $Rh(cod)_2OTf$ catalyst, 30 mol% Taylor's boronic acid **27**, oxabicycle **16a** (0.4 mmol, 2 equiv.), and 0.2 mmol carbohydrate polyol **15u** at 50 ˚C for 6 h and isolated by flash column chromatography (15:1-8:1 Pentane: Ethyl Acetate) giving **17u** as a yellow solid (66.0 mg, 61% yield, r.r. >20:1 (C1:C2), d.r. > 20:1(*trans*:*cis*)).

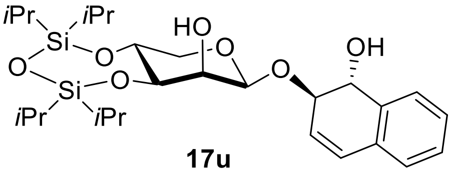

17u

^{1}H NMR (500 MHz, $CDCl_3$) δ 7.66 (d, J = 7.5 Hz, 1H), 7.29 – 7.24 (m, 1H), 7.21 (t, J = 7.5 Hz, 1H), 7.05 (d, J = 7.0 Hz, 1H), 6.39 (dd, J = 10.0, 2.5 Hz, 1H), 5.86 (dd, J = 10.0, 2.0 Hz, 1H), 5.07 (d, J = 11.0 Hz, 1H), 4.59 (s, 1H), 4.56 (t, J = 2.0 Hz, 1H), 4.46 (s, 1H), 4.16 – 4.12 (m, 1H), 4.11 (d, J = 2.5 Hz, 1H), 4.06 (dd, J = 11.5, 6.0 Hz, 1H), 3.70 (dd, J = 8.5, 3.5 Hz, 1H), 3.24 (dd, J = 11.5, 10.0 Hz, 1H), 2.66 (s, 1H), 1.12 – 0.98 (m, 28H). **^{13}C NMR** (126 MHz, $CDCl_3$) δ 135.88, 131.85, 128.50, 128.42, 128.24, 127.67, 126.20, 125.11, 101.75, 86.57, 77.05, 72.82, 71.66, 69.35, 66.22, 17.69, 17.61, 17.46, 17.45, 17.36, 17.32, 17.29, 13.09, 12.99, 12.30, 12.27. **ESI-HRMS**: Calculated for $C_{27}H_{44}O_7NaSi_2$ $(M+Na)^+$: 559.25178, Found: 559.25097. $[\alpha]_D^{20}$ = -33.2 (c =0.69, $CHCl_3$).

(*5aR,8S,9S,9aR*)-8-(((*1S,2S*)-1-hydroxy-1,2-dihydronaphthalen-2-yl)oxy)-2,2,4,4-tetraisopropyltetrahydro-6H-pyrano[3,4-f][1,3,5,2,4]trioxadisilepin-9-ol (19u)

(*5aR,9S,9aR*)-9-(((*1S,2S*)-1-hydroxy-1,2-dihydronaphthalen-2-yl)oxy)-2,2,4,4-tetraisopropyltetrahydro-6H-pyrano[3,4-f][1,3,5,2,4]trioxadisilepin-8-ol(20u)

The title product compound is prepared according to the general procedure **B1** with 5 mol% $Rh(cod)_2OTf$ catalyst, 30 mol% Taylor's boronic acid **27**, oxabicycle **16a** (0.4 mmol, 2 equiv.), and 0.2 mmol carbohydrate polyol **15u** at 50 ˚C for 16 h and isolated by flash column chromatography (15:1-8:1 Pentane: Ethyl Acetate) giving inseparable mixture of **19u** and **20u** as a white solid (59.0 mg, 55% yield, r.r. 2:1 (C1:C2), d.r. > 20:1(*trans*:*cis*)).

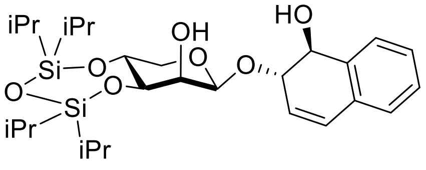

19u

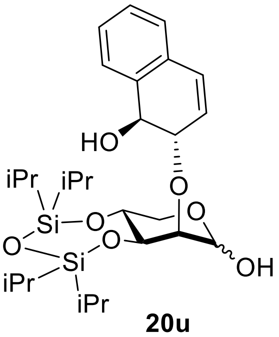

20u

^{1}H NMR (700 MHz, $CDCl_3$) δ 7.65 – 7.57 (m, 2H), 7.25 – 7.18 (m, 3H), 7.09 – 7.01 (m, 2H), 6.45 – 6.38 (m, 1.3H), 6.35 (dd, J = 9.8, 2.8 Hz, 0.6H), 6.10 (dd, J = 9.8, 2.1 Hz, 0.3H), 6.07 (dd, J = 9.8, 2.1 Hz, 1H), 5.90 (dd, J = 9.8, 2.1 Hz, 0.6H), 5.25 – 5.20 (m, 0.5H), 5.08 (d, J = 11.2 Hz, 1H), 5.02 (d, J = 11.9 Hz, 1H), 4.76 (s, 1H), 4.71 (d, J = 10.5 Hz, 0.3H), 4.64 (dt, J = 11.2, 2.1 Hz, 1H), 4.58 (dt, J = 11.2, 2.1 Hz, 0.3H), 4.50 (d, J = 2.1 Hz, 0.6H), 4.35 (dt, J = 11.9, 2.1 Hz, 0.6H), 4.27 – 4.21 (m, 0.6H), 4.19 – 4.14 (m, 0.3H), 4.12 – 4.06 (m, 2.5H), 4.02 – 3.94 (m, 2H), 3.92 (d, J = 2.8 Hz, 0.3H), 3.79 (dd, J = 11.2, 5.6 Hz, 0.6H), 3.77 – 3.73 (m, 0.3H), 3.70 – 3.66 (m, 1.5H), 3.49 (d, J = 10.5 Hz, 0.3H), 3.19 (dd, J = 11.9, 9.8 Hz, 0.3H), 3.14 (dd, J = 11.9, 10.5 Hz, 1H), 2.95 (s, 1H), 2.69 (s, 1H), 2.52 (d, J = 2.8 Hz, 0.6H), 1.14 – 0.98 (m, 51H). **^{13}C NMR** (176 MHz, $CDCl_3$) δ 136.73, 136.54, 135.98, 132.25, 132.11, 131.94, 129.99, 129.64, 128.62, 128.60, 128.37, 128.08, 128.03, 127.96, 127.93, 127.91, 127.91, 127.65, 127.42, 126.36, 126.36, 126.22, 126.04, 125.06, 124.77, 124.49, 100.96, 94.63, 94.18, 86.26, 85.58, 82.90, 81.44, 79.98, 78.13, 77.09, 74.82, 74.52, 73.74, 72.96, 71.27, 70.00, 69.77, 69.62, 66.49, 66.24, 62.92, 17.71, 17.70, 17.65, 17.62, 17.61, 17.46, 17.46, 17.46, 17.44, 17.37, 17.37, 17.35, 17.33, 17.31, 17.30, 17.28, 17.25, 17.22, 13.11, 13.08, 13.02, 12.94, 12.88, 12.58, 12.57, 12.35, 12.32, 12.31, 12.29. **ESI-HRMS**: Calculated for $C_{27}H_{44}O_7NaSi_2$ $(M+Na)^+$: 559.25178, Found: 559.25042. $[\alpha]_D^{20}$ = +55.3 (c =0.27, $CHCl_3$).

(*2R,3R,4S,5S,6S*)-2-(((*tert*-butyldimethylsilyl)oxy)methyl)-4-(((*1R,2R*)-1-hydroxy-6,7-dimethyl-1,2-dihydronaphthalen-2-yl)oxy)-6-methoxytetrahydro-2H-pyran-3,5-diol (17aa)

The title product compound is prepared according to the general procedure **A** with 5 mol% Rh(cod)$_2$OTf catalyst, 30 mol% cyclohexyl vinyl boronic acid **26**, oxabicycle **16b** (0.4 mmol, 2 equiv.), and 0.2 mmol carbohydrate polyol **15a** at 50 ˚C for 24 h and isolated by flash column chromatography (9:1-2:1 Pentane: Ethyl Acetate) giving **17aa** as a white solid (85 mg, 88% yield, r.r. >20:1 (C3:C2), d.r. > 20:1(*trans*:*cis*)).

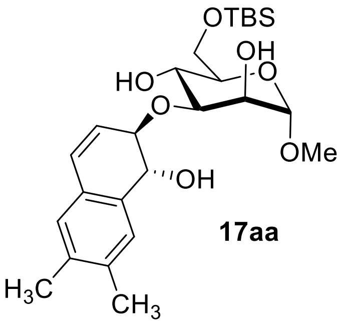

17aa

^{1}H NMR (400 MHz, CDCl$_3$) δ 7.34 (s, 1H), 6.84 (s, 1H), 6.36 (dd, *J* = 9.9, 2.4 Hz, 1H), 5.92 (dd, *J* = 9.8, 2.0 Hz, 1H), 5.01 (d, *J* = 11.6 Hz, 1H), 4.76 (d, *J* = 1.5 Hz, 1H), 4.44 (dt, *J* = 11.6, 2.2 Hz, 1H), 4.03 (dd, *J* = 3.3, 1.7 Hz, 1H), 3.94 – 3.88 (m, 3H), 3.87 – 3.83 (m, 1H), 3.67-3.62 (m, 1H), 3.37 (s, 3H), 2.25 (s, 3H), 2.22 (s, 3H), 0.92 (s, 9H), 0.11 (d, *J* = 1.2 Hz, 6H). **^{13}C NMR** (101 MHz, CDCl$_3$) δ 136.36, 135.64, 133.61, 129.92, 128.43, 127.76, 127.64, 125.97, 100.74, 79.88, 77.03, 72.57, 71.00, 68.67, 67.67, 64.97, 54.91, 26.00, 19.77, 19.38, 18.39, -5.31, -5.34. **ESI-HRMS**: Calculated for $C_{25}H_{40}O_7NaSi$ (M+Na)$^+$: 503.24355, Found: 503.24299. $[\alpha]_D^{20}$ = -48.1 (c = 0.38, CHCl$_3$).

(*2R,3R,4S,5S,6S*)-2-(((*tert*-butyldimethylsilyl)oxy)methyl)-4-(((*1R,2R*)-1-hydroxy-6,7-dimethoxy-1,2-dihydronaphthalen-2-yl)oxy)-6-methoxytetrahydro-2H-pyran-3,5-diol (17ab)

The title product compound is prepared according to the general procedure **A** with 5 mol% Rh(cod)$_2$OTf catalyst, 30 mol% cyclohexyl vinyl boronic acid **26**, oxabicycle **16c** (0.4 mmol, 2 equiv.), and 0.2 mmol carbohydrate polyol **15a** at 50 ˚C for 24 h and isolated by flash column chromatography (9:1-1:1 Pentane: Ethyl Acetate) giving **17ab** as a white solid (96 mg, 93% yield, r.r. >20:1 (C3:C2), d.r. > 20:1(*trans*:*cis*)).

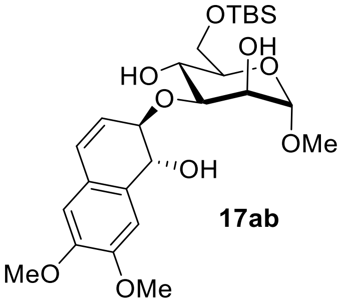

17ab

^{1}H NMR (700 MHz, CDCl$_3$) δ 7.13 (s, 1H), 6.56 (s, 1H), 6.28 (dd, *J* = 9.9, 2.5 Hz, 1H), 5.90 (dd, *J* = 9.8, 1.9 Hz, 1H), 4.97 (d, *J* = 12.0 Hz, 1H), 4.71 (s, 1H), 4.54 (bs, 1H), 4.41 (dt, *J* = 12.0, 2.3 Hz, 1H), 4.03 (s, 1H), 3.89-3.85 (m, 3H), 3.84 (s, 3H), 3.82 (s, 3H), 3.78 (dd, *J* = 9.2, 3.2 Hz, 1H), 3.68 (bs, 1H), 3.62-3.60 (m, *J* = 10.3, 1H), 3.47 (bs, 1H), 3.32 (s, 3H), 0.88 (s, 9H), 0.07 (s, 6H). **^{13}C NMR** (176 MHz, CDCl$_3$) δ 148.61, 148.08, 129.15, 128.10, 126.85, 125.02, 109.99, 108.42, 100.70, 80.01, 76.90, 72.55, 71.01, 68.59, 67.69, 64.91, 56.07, 56.05, 54.89, 25.95, 18.34, -5.36, -5.39. **ESI-HRMS**: Calculated for $C_{25}H_{40}O_9NaSi$ (M+Na)$^+$: 535.23338, Found: 535.23266. $[\alpha]_D^{20}$ = -50.3 (c = 0.55, CHCl$_3$).

(*2R,3R,4S,5S,6S*)-2-(((*tert*-butyldimethylsilyl)oxy)methyl)-4-(((*5R,6R*)-5-hydroxy-5,6-dihydronaphtho[2,3-d][1,3]dioxol-6-yl)oxy)-6-methoxytetrahydro-2H-pyran-3,5-diol (17ac)

The title product compound is prepared according to the general procedure **A** with 5 mol% Rh(cod)$_2$OTf catalyst, 30 mol% cyclohexyl vinyl boronic acid **26**, oxabicycle **16d** (0.4 mmol, 2 equiv.), and 0.2 mmol carbohydrate polyol **15a** at 50 ˚C for 24 h and isolated by flash column chromatography (9:1-2:1 Pentane: Ethyl Acetate) giving **17ac** as a white solid (85.6 mg, 86% yield, r.r. >20:1 (C3:C2), d.r. > 20:1(*trans*:*cis*)).

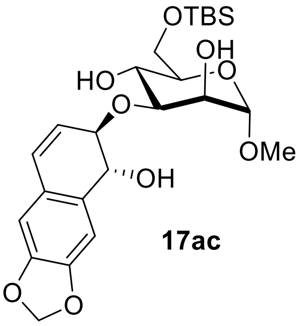

17ac

^{1}H NMR (500 MHz, CDCl$_3$) δ 7.09 (s, 1H), 6.52 (s, 1H), 6.25 (dd, *J* = 10.0, 2.3 Hz, 1H), 5.91-5.89 (m, 3H), 4.91 (dd, *J* = 12.0, 3.8 Hz, 1H), 4.73 (d, *J* = 1.1 Hz 1H), 4.48 (bs, 1H), 4.39 (dt, *J* = 12.0, 2.0 Hz, 1H), 4.03 (s, 1H), 3.92-3.86 (m, 3H), 3.78 (dd, *J* = 9.2, 3.2 Hz, 1H), 3.69 (bs, 1H), 3.64-3.60 (m, 1H), 3.54 (d, *J* = 1.3 Hz, 1H), 3.34 (s, 3H), 0.89 (s, 9H), 0.09 (d, *J* = 1.1 Hz, 6H). **^{13}C NMR** (126 MHz, CDCl$_3$) δ 147.16, 146.73, 130.87, 128.12, 126.90, 126.24, 106.91, 106.08, 101.04, 100.59, 79.76, 76.82 (merged with CDCl3), 72.50, 70.80, 68.65, 67.61, 64.92, 54.87, 25.90, 18.30, -5.41, -5.45. **ESI-HRMS**: Calculated for $C_{24}H_{36}O_9NaSi$ (M+Na)$^+$: 519.20208, Found: 519.20152. $[\alpha]_D^{20}$ = -71.8 (c = 0.16, CHCl$_3$).

(*2R,3R,4S,5S,6S*)-2-(((*tert*-butyldimethylsilyl)oxy)methyl)-4-(((*1R,2R*)-1-hydroxy-5,8-dimethoxy-1,2-dihydronaphthalen-2-yl)oxy)-6-methoxytetrahydro-2H-pyran-3,5-diol (17ad)

The title product compound is prepared according to the general procedure **A** with 5 mol% Rh(cod)$_2$OTf catalyst, 30 mol% cyclohexyl vinyl boronic acid **26**, oxabicycle **16e** (0.4 mmol, 2 equiv.), and 0.2 mmol carbohydrate polyol **15a** at 50 ˚C for 24 h and isolated by flash column chromatography (9:1-1:1 Pentane: Ethyl Acetate) giving **17ad** as a white solid (82.5 mg, 80% yield, r.r. >20:1 (C3:C2), d.r. > 20:1(*trans*:*cis*)).

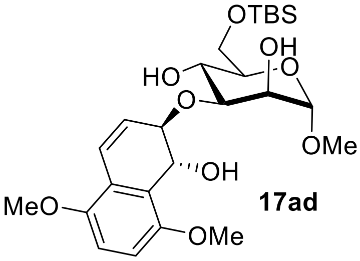

17ad

^{1}H NMR (700 MHz, CDCl$_3$) δ 6.78 – 6.73 (m, 3H), 6.01 (dd, *J* = 10.1, 2.6 Hz, 1H), 5.21 (dd, *J* = 9.4, 1.5 Hz, 1H), 4.90 (d, *J* = 1.6 Hz, 1H), 4.78 (d, *J* = 1.6 Hz, 1H), 4.47 (dt, *J* = 9.4, 2.4 Hz, 1H), 4.07 (s, 1H), 3.94 – 3.91 (m, 2H), 3.87 – 3.83 (m, 2H), 3.82 (s, 3H), 3.76 (s, 3H), 3.71 (dd, *J* = 9.0, 3.3 Hz, 1H), 3.62 (dt, *J* = 10.2, 5.4 Hz, 1H), 3.35 (s, 3H), 3.07 (d, *J* = 1.7 Hz, 1H), 0.89 (s, 9H), 0.08 (d, *J* = 1.1 Hz, 6H). **^{13}C NMR** (176 MHz, CDCl$_3$) δ 151.50, 150.09, 127.35, 123.61, 122.41, 121.93, 111.61, 111.21, 100.82, 78.73, 76.84, 72.28, 71.11, 68.51, 66.82, 64.86, 56.26, 56.18, 54.77, 25.98, 18.36, -5.33. **ESI-HRMS**: Calculated for $C_{25}H_{40}O_9NaSi$ (M+Na)$^+$: 535.19341, Found: 535.23289. $[\alpha]_D^{20}$ = -47.1 (c = 0.45, CHCl$_3$)

(*2R,3R,4S,5S,6S*)-2-(((*tert*-butyldimethylsilyl)oxy)methyl)-4-(((*1R,2R*)-1-hydroxy-1,4-dimethyl-1,2-dihydronaphthalen-2-yl)oxy)-6-methoxytetrahydro-2H-pyran-3,5-diol (17ae)

The title product compound is prepared according to the general procedure **A** with 5 mol% $Rh(cod)_2OTf$ catalyst, 30 mol% cyclohexyl vinyl boronic acid **26**, oxabicycle **16f** (0.4 mmol, 2 equiv.), and 0.2 mmol carbohydrate polyol **15a** at 50 ˚C for 24 h and isolated by flash column chromatography (9:1-2:1 Pentane: Ethyl Acetate) giving **17ae** as a white solid (86.4 mg, 90% yield, r.r. >20:1 (C3:C2), d.r. > 20:1(*trans*:*cis*)).

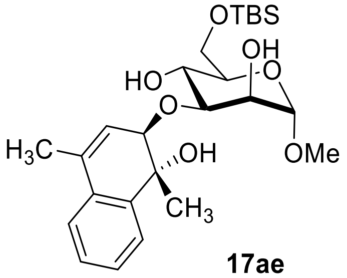

17ae

^{1}H NMR (500 MHz, $CDCl_3$) δ 7.60 – 7.58 (m, 1H), 7.29-7.23 (m, 2H), 7.20 – 7.18 (m, 1H), 5.82 (s, 1H), 4.72 (s, 1H), 4.44 (t, *J* = 1.8 Hz, 1H), 4.05 (s, 1H), 3.95 (dd, *J* = 10.6, 5.0 Hz, 1H), 3.89 – 3.84 (m, 2H), 3.72 (dd, *J* = 9.1, 3.3 Hz, 2H), 3.66-3.62 (m, 1H), 3.52 (bs, 1H), 3.33 (s, 3H), 3.13 (s, 1H), 2.05 (s, 3H), 1.41 (s, 3H), 0.93 (s, 9H), 0.12 (s, 6H). **^{13}C NMR** (126 MHz, $CDCl_3$) δ 142.24, 133.39, 132.60, 128.15, 127.57, 126.99, 123.48, 123.42, 100.76, 80.19, 78.09, 76.47, 71.18, 68.38, 66.59, 64.75, 54.83, 26.02, 22.79, 18.83, 18.42, -5.27. **ESI-HRMS**: Calculated for $C_{25}H_{40}O_7NaSi$ $(M+Na)^+$: 503.24355, Found: 503.24296. $[\alpha]_D^{20}$ = +38.8 (c = 0.42, $CHCl_3$).

(*2R,3R,5S,6S*)-2-(((*tert*-butyldimethylsilyl)oxy)methyl)-4-(((*1R,2R*)-6,7-difluoro-1-hydroxy-1,2-dihydronaphthalen-2-yl)oxy)-6-methoxytetrahydro-2H-pyran-3,5-diol (17af)

The title product compound is prepared according to the general procedure **A** with 5 mol% $Rh(cod)_2OTf$ catalyst, 30 mol% cyclohexyl vinyl boronic acid **26**, oxabicycle **16g** (0.4 mmol, 2 equiv.), and 0.2 mmol carbohydrate polyol **15a** at 50 ˚C for 24 h and isolated by flash column chromatography (9:1-2:1 Pentane: Ethyl Acetate) giving **17af** as a white solid (87.9 mg, 90% yield, r.r. >20:1 (C3:C2), d.r. > 20:1(*trans*:*cis*)).

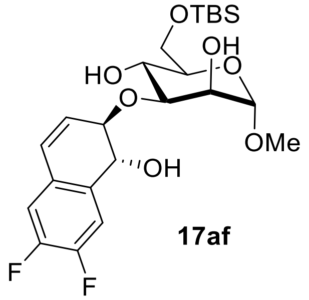

17af

1H NMR (700 MHz, $CDCl_3$) δ 7.39 (dd, J = 10.4, 8.2 Hz, 1H), 6.83 (dd, J = 10.4, 7.4 Hz, 1H), 6.27 (dd, J = 10.0, 2.5 Hz, 1H), 6.04 (d, J = 10.1 Hz, 1H), 4.95 (d, J = 12.1 Hz, 1H), 4.74 (d, J = 4.2 Hz, 1H), 4.73 (d, J = 1.7 Hz, 1H), 4.42 (dt, J = 12.1, 2.2 Hz, 1H), 4.02 (s, 1H), 3.93 – 3.87 (m, 3H), 3.80 (dd, J = 9.2, 3.3 Hz, 1H), 3.65 – 3.61 (m, 3H), 3.35 (s, 3H), 0.90 (s, 9H), 0.10 (d, J = 2.4 Hz, 6H). **^{13}C NMR** (176 MHz, $CDCl_3$) δ 150.38 (dd, *J* = 12.9, 10.7 Hz), 148.97 (dd, *J* = 12.9, 8.9 Hz), 133.68 (dd, *J* = 5.7, 3.4 Hz), 129.99 (d, *J* = 2.6 Hz), 129.08 (dd, *J* = 6.3, 3.8 Hz), 126.76, 114.78 (dd, *J* = 78.7, 18.7 Hz), 100.68, 79.63, 76.92, 71.70, 70.62, 69.12, 67.94, 65.23, 55.05, 25.97, 18.37, -5.39 (d, *J* = 6.0 Hz). **ESI-HRMS**: Calculated for $C_{23}H_{34}O_7F_2NaSi$ $(M+Na)^+$: 511.19341, Found: 511.19281. $[\alpha]_D^{20}$ = -27.9 (c = 0.48, $CHCl_3$).

(*2R,3R,4S,5S,6S*)-2-(((*tert*-butyldimethylsilyl)oxy)methyl)-4-(((*1R,2R*)-6,7-dibromo-1-hydroxy-5,8-dimethyl-1,2-dihydronaphthalen-2-yl)oxy)-6-methoxytetrahydro-2H-pyran-3,5-diol (17ag)

The title product compound is prepared according to the general procedure **A** with 5 mol% Rh(cod)$_2$OTf catalyst, 30 mol% cyclohexyl vinyl boronic acid **26**, oxabicycle **16h** (0.4 mmol, 2 equiv.), and 0.2 mmol carbohydrate polyol **15a** at 50 ˚C for 24 h and isolated by flash column chromatography (9:1-2:1 Pentane: Ethyl Acetate) giving **17ag** as a white solid (94.6 mg, 74% yield, r.r. >20:1 (C3:C2), d.r. > 20:1(*trans*:*cis*)).

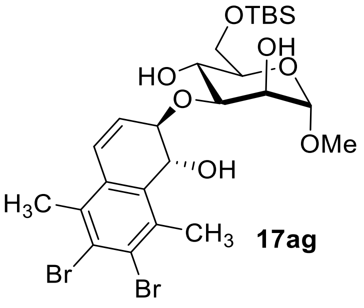

17ag

^{1}H NMR (500 MHz, $CDCl_3$) δ 6.95 (d, *J* = 9.9 Hz, 1H), 6.25 (dd, *J* = 9.9, 5.2 Hz, 1H), 4.93 (s, 1H), 4.64 (d, *J* = 1.6 Hz, 1H), 4.34 (dd, *J* = 5.5, 2.3 Hz, 1H), 3.87 (s, 1H), 3.81 (d, *J* = 5.2 Hz, 2H), 3.74 (dd, *J* = 9.2, 3.2 Hz, 1H), 3.67 (t, *J* = 9.3 Hz, 1H), 3.54-3.49 (m, *J* = 9.9, 5.1 Hz, 1H), 3.34 (s, 3H), 2.79 (s, 1H), 2.58 (s, 3H), 2.55 (s, 3H), 2.42 (s, 1H), 2.19 (s, 1H), 0.88 (s, 9H), 0.07 (s, 6H). **^{13}C NMR** (126 MHz, $CDCl_3$) δ 137.54, 134.42, 133.06, 129.70, 129.66, 129.13, 128.11, 125.38, 100.39, 77.91, 73.85, 71.06, 68.83, 68.64, 67.88, 64.45, 54.89, 25.91, 21.13, 20.84, 18.31, -5.40, -5.41. **ESI-HRMS**: Calculated for $C_{25}H_{38}O_7Br_2NaSi$ $(M+Na)^+$: 659.06458, Found: 659.06442. $[\alpha]_D^{20}$ = -136.2 (c = 0.45, $CHCl_3$)

(*2R,3R,4S,5S,6S*)-2-(((*tert*-butyldimethylsilyl)oxy)methyl)-4-(((*1R,2R*)-1-hydroxy-1,2-dihydroanthracen-2-yl)oxy)-6-methoxytetrahydro-2H-pyran-3,5-diol (17ah)

The title product compound is prepared according to the general procedure **A** with 5 mol% Rh(cod)$_2$OTf catalyst, 30 mol% cyclohexyl vinyl boronic acid **26**, oxabicycle **16i** (0.4 mmol, 2 equiv.), and 0.2 mmol carbohydrate polyol **15a** at 50 ˚C for 24 h and isolated by flash column chromatography (9:1-2:1 Pentane: Ethyl Acetate) giving **17ah** as a white solid (87.5mg, 87% yield, r.r. >20:1 (C3:C2), d.r. > 20:1(*trans*:*cis*)).

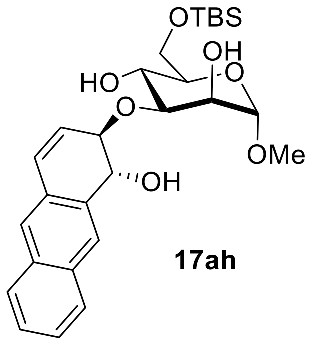

17ah

^{1}H NMR (600 MHz, $CDCl_3$) δ 7.98 (s, 1H), 7.72 (t, *J* = 8.5 Hz, 2H), 7.42 – 7.37 (m, 3H), 6.54 (dd, *J* = 10.0, 2.4 Hz, 1H), 6.06 (dd, *J* = 9.9, 1.8 Hz, 1H), 5.14 (d, *J* = 10.7 Hz, 1H), 4.77 (s, 1H), 4.50 (dt, *J* = 10.9, 2.1 Hz, 1H), 4.10 (bs, 1H), 3.96 (t, *J* = 9.4 Hz, 1H), 3.93-3.89 (m, 2H), 3.86 (dd, *J* = 9.2, 3.1 Hz, 1H), 3.69-3.65 (m, 1H), 3.59 (bs, 1H), 3.34 (s, 3H), 0.92 (s, 9H), 0.11 (s, 6H). **^{13}C NMR** (151 MHz, $CDCl_3$) δ 134.37, 133.09, 132.99, 130.81, 129.27, 128.68, 128.07, 127.76, 126.25, 126.20, 125.09, 123.62, 100.73, 79.55, 77.03, 72.55, 70.94, 68.82, 67.83, 65.06, 54.95, 26.00, 18.39, -5.32, -5.35. **ESI-**

HRMS: Calculated for $C_{27}H_{38}O_7NaSi$ $(M+Na)^+$: 525.22790, Found: 525.22736. $[\alpha]_D^{20}$ = -40.5 (c = 0.38, $CHCl_3$).

(*2R,3S,5S,6S*)-5-(((*R*)-but-3-en-2-yl)oxy)-2-(((tert-butyldimethylsilyl)oxy)methyl)-6-methoxytetrahydro-2*H*-pyran-3,4-diol(17v)

The title product compound is prepared according to the general procedure **C1** with 5 mol% $Rh(cod)_2BF_4$ catalyst, 6% (*S*)-NPN ligand, 30 mol% Taylor's boronic acid **27**, allylic carbonate **16j** (0.24 mmol, 1.2 equiv.), and 0.2 mmol carbohydrate polyol **15a** at 50 ˚C for 18 h and isolated by flash column chromatography (5:1-1:1 Pentane: Ethyl Acetate) giving **17v** as a colorless oil (45.6 mg, 63% yield, r.r. >20:1 (C2:C1), d.r. > 20:1).

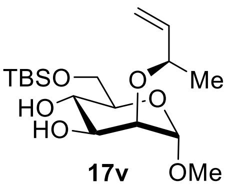

17v

^{1}H NMR (500 MHz, $CDCl_3$) δ 5.76 (ddd, J = 17.0, 10.0, 6.5 Hz, 1H), 5.22 (dt, J = 17.0, 1.5 Hz, 1H), 5.10 (dt, J = 10.5, 1.0 Hz, 1H), 4.65 (d, J = 1.5 Hz, 1H), 4.05 – 3.97 (m, 1H), 3.86 (d, J = 5.5 Hz, 2H), 3.80 – 3.73 (m, 1H), 3.70 (t, J = 9.5 Hz, 1H), 3.63 (dd, J = 4.0, 1.5 Hz, 1H), 3.54 – 3.47 (m, 1H), 3.32 (s, 3H), 3.09 (s, 1H), 2.31 (d, J = 9.0 Hz, 1H), 1.27 (d, J = 6.5 Hz, 3H), 0.90 (s, 9H), 0.09 (d, J = 3.0 Hz, 6H). **^{13}C NMR** (126 MHz, $CDCl_3$) δ 140.20, 116.09, 99.70, 78.86, 76.66, 71.50, 71.27, 70.75, 64.74, 54.83, 25.99, 21.38, 18.37, -5.30, -5.33. **ESI-HRMS**: Calculated for $C_{17}H_{34}O_6NaSi$ $(M+Na)^+$: 385.20169, Found: 385.20161. $[\alpha]_D^{20}$ = +9.6 (c =0.49, $CHCl_3$).

(*2R,3R,5S,6S*)-4-(((*S*)-but-3-en-2-yl)oxy)-2-(((tert-butyldimethylsilyl)oxy)methyl)-6-methoxytetrahydro-2*H*-pyran-3,5-diol (19v)

(*2R,3S,5S,6S*)-5-(((*S*)-but-3-en-2-yl)oxy)-2-(((tert-butyldimethylsilyl)oxy)methyl)-6-methoxytetrahydro-2*H*-pyran-3,4-diol(20v)

The title product compound is prepared according to the general procedure **D1** with 5 mol% $Rh(cod)_2BF_4$ catalyst, 6% (*R*)-NPN ligand, 30 mol% Taylor's boronic acid **27**, allylic carbonate **16j** (0.24 mmol, 1.2 equiv.), and 0.2 mmol carbohydrate polyol **15a** at 50 ˚C for 18 h and isolated by flash column chromatography (5:1-1:1 Pentane: Ethyl Acetate) giving C3 regioisomer **19v** (25.0 mg, d.r. > 20:1) as a colorless oil and C2 regioisomer **20v** (12.5 mg, d.r. > 20:1) as a colorless oil (37.5 mg combined total mass of **19v** and **20v**, 53% combined yield of **19v** and **20v**, r.r. 2:1(C3:C2)).

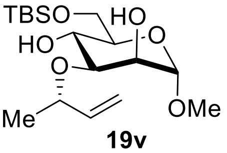

19v

^{1}H NMR (600 MHz, $CDCl_3$) δ 5.78 (ddd, J = 17.4, 10.2, 7.2 Hz, 1H), 5.25 (dt, J = 17.4, 1.2 Hz, 1H), 5.21 – 5.17 (m,1H), 4.75 (d, J = 1.8 Hz, 1H), 4.12 – 4.04 (m, 1H), 3.92 (dd, J = 3.6, 1.8 Hz, 1H), 3.86 (d, J = 5.4 Hz, 2H), 3.77 (td, J = 9.6, 1.2 Hz, 1H), 3.66 (dd, J = 9.0, 3.6 Hz, 1H), 3.61 – 3.55 (m, 1H), 3.36 (s, 3H), 2.79 (s, 1H), 2.40 (s, 1H), 1.31 (d, J = 6.0 Hz, 3H), 0.89 (s, 9H), 0.08 (s, 6H). **^{13}C NMR** (151 MHz, $CDCl_3$) δ 139.71, 117.16, 100.42, 76.45, 75.02, 71.07, 68.50, 67.65, 64.77, 54.96, 26.03, 22.09, 18.44, -5.31. **ESI-HRMS**: Calculated for $C_{17}H_{34}O_6NaSi$ $(M+Na)^+$: 385.20169, Found: 385.20149. $[\alpha]_D^{20}$ = +6.0 (c =0.30, $CHCl_3$).

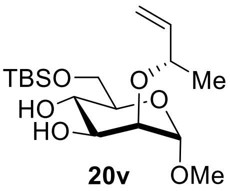

20v

¹H NMR (600 MHz, $CDCl_3$) δ 5.69 (ddd, J = 17.4, 10.2, 7.8 Hz, 1H), 5.22 – 5.18 (m, 1H), 5.18 – 5.16 (m, 1H), 4.71 (d, J = 1.2 Hz, 1H), 4.00 – 3.93 (m, 1H), 3.90 – 3.83 (m, 2H), 3.74 – 3.69 (m, 1H), 3.69 – 3.66 (m, 1H), 3.66 – 3.63 (m, 1H), 3.53 – 3.48 (m, 1H), 3.35 (s, 3H), 2.90 (s, 1H), 2.26 (d, J = 9.0 Hz, 1H), 1.25 (d, J = 6.6 Hz, 3H), 0.90 (s, 9H), 0.09 (d, J = 2.4 Hz, 6H). **¹³C NMR** (151 MHz, $CDCl_3$) δ 139.51, 117.45, 98.39, 76.64, 74.81, 71.29, 71.21, 70.55, 64.22, 54.83, 26.01, 21.80, 18.40, -5.22, -5.28. **ESI-HRMS**: Calculated for $C_{17}H_{34}O_6NaSi$ $(M+Na)^+$: 385.20169, Found: 385.20149. $[\alpha]_D^{20}$ = -4.1 (c =0.39, $CHCl_3$).

(*2R,3S,4S,5S,6S*)-2-(((tert-butyldimethylsilyl)oxy)methyl)-5-(((*S*)-1-cyclohexylallyl)oxy)-6-methoxytetrahydro-2*H*-pyran-3,4-diol (17w)

The title product compound is prepared according to the general procedure **C1** with 5 mol% $Rh(cod)_2BF_4$ catalyst, 6% (*S*)-NPN ligand, 30 mol% Taylor's boronic acid **27**, allylic carbonate **16k** (0.24 mmol, 1.2 equiv.), and 0.2 mmol carbohydrate polyol **15a** at 50 ˚C for 40 h and isolated by flash column chromatography (5:1-1:1 Pentane: Ethyl Acetate) giving **17w** as a colorless oil (60.2 mg, 70% yield, r.r. >20:1 (C2:C1), d.r. > 20:1).

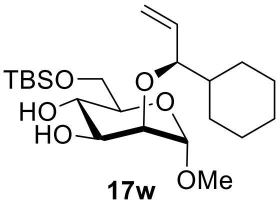

17w

¹H NMR (600 MHz, $CDCl_3$) δ 5.66 (ddd, J = 16.8, 10.8, 8.4 Hz, 1H), 5.22 – 5.19 (m, 1H), 5.19 – 5.16 (m,1H), 4.61 (d, J = 1.2 Hz, 1H), 3.91 – 3.82 (m, 2H), 3.77 (td, J = 9.0, 3.6 Hz, 1H), 3.73 (td, J = 9.0, 1.8 Hz, 1H), 3.59 (dd, J = 3.6, 1.2 Hz, 1H), 3.54 (t, J = 7.2 Hz, 1H), 3.50 (dt, J = 9.0, 5.4 Hz, 1H), 3.31 (s, 3H), 3.03 (d, J = 1.2 Hz, 1H), 2.17 (d, J = 8.4 Hz, 1H), 1.99– 1.91 (m,1H), 1.77 – 1.68 (m, 2H), 1.67 – 1.61 (m, 2H), 1.53 – 1.45 (m,1H), 1.27 – 1.08 (m, 3H), 1.06 – 0.96 (m, 1H), 0.90 (s, 9H), 0.09 (d, J = 3.6 Hz, 6H). **¹³C NMR** (151 MHz, $CDCl_3$) δ 137.93, 118.54, 100.24, 88.83, 76.82, 72.16, 71.43, 70.79, 64.69, 54.82, 42.45, 29.34, 29.08, 26.63, 26.16, 26.08, 25.99, 18.37, -5.29, -5.34. **ESI-HRMS**: Calculated for $C_{22}H_{36}O_6NaSi$ $(M+Na)^+$: 453.26429, Found: 453.26379. $[\alpha]_D^{20}$ = +10.0 (c =0.30, $CHCl_3$).

(*2R,3S,5S,6S*)-2-(((tert-butyldimethylsilyl)oxy)methyl)-5-(((*R*)-hex-1-en-3-yl)oxy)-6-methoxytetrahydro-2*H*-pyran-3,4-diol(17x)

The title product compound is prepared according to the general procedure **C1** with 5 mol% $Rh(cod)_2BF_4$ catalyst, 6% (*S*)-NPN ligand, 30 mol% Taylor's boronic acid **27**, allylic carbonate **16l** (0.24 mmol, 1.2 equiv.), and 0.2 mmol carbohydrate polyol **15a** at 50 ˚C for 40 h and isolated by flash column chromatography (5:1-3:1 Pentane: Ethyl Acetate) giving **17x** as a colorless oil (63.5 mg, 81% yield, r.r. >20:1 (C2:C1), d.r. > 20:1).

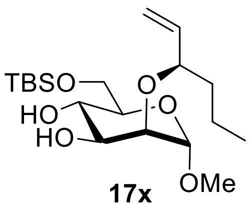

17x

¹H NMR (700 MHz, $CDCl_3$) δ 5.74 – 5.65 (m, 1H), 5.21 (dt, J = 17.5, 1.4 Hz, 1H), 5.16 – 5.12 (m, 1H), 4.64 (d, J = 1.4 Hz, 1H), 3.87 (dt, J = 10.5, 5.6 Hz, 2H), 3.85 – 3.81 (m, 1H), 3.77 (td, J = 9.1, 3.5 Hz,

1H), 3.71 (td, J = 9.1, 1.4 Hz, 1H), 3.62 (dd, J = 3.5, 1.4 Hz, 1H), 3.50 (dt, J = 9.8, 5.6 Hz, 1H), 3.31 (s, 3H), 3.07 (s, 1H), 2.25 (d, J = 9.1 Hz, 1H), 1.70 – 1.61 (m, 1H), 1.48 – 1.37 (m, 2H), 1.37 – 1.32 (m,1H), 0.91 (t, J = 9.1 Hz, 12H), 0.09 (d, J = 4.4 Hz, 6H). **^{13}C NMR** (176 MHz, $CDCl_3$) δ 139.33, 117.21, 100.04, 83.68, 76.87, 71.87, 71.42, 70.76, 64.79, 54.83, 37.92, 25.99, 18.84, 18.37, 14.15, -5.31, -5.34. **ESI-HRMS**: Calculated for $C_{19}H_{38}O_6NaSi$ $(M+Na)^+$: 413.23299, Found: 413.23281. $[\alpha]_D^{20}$ = +11.1 (c =0.45, $CHCl_3$).

(*2R,3S,4S,5S,6S*)-2-(((tert-butyldimethylsilyl)oxy)methyl)-6-methoxy-5-(((*S*)-1-phenylallyl)oxy)tetrahydro-2*H*-pyran-3,4-diol (17y)

The title product compound is prepared according to the general procedure **C1** with 10 mol% $Rh(cod)_2BF_4$ catalyst, 12% (*S*)-NPN ligand, 30 mol% Taylor's boronic acid **27**, allylic carbonate **16m** (0.24 mmol, 1.2 equiv.), and 0.2 mmol carbohydrate polyol **15a** at 70 ˚C for 41 h and isolated by flash column chromatography (5:1-3:1 Pentane: Ethyl Acetate) giving **17y** as a colorless oil (53.3 mg, 63% yield, r.r. >20:1 (C2:C1), d.r. > 20:1).

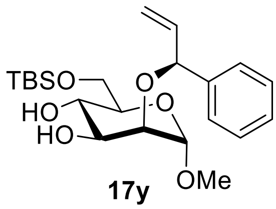

17y

^{1}H NMR (700 MHz, $CDCl_3$) δ 7.38 – 7.31 (m, 4H), 7.32 – 7.27 (m, 1H), 5.96 – 5.87 (m, 1H), 5.35 (dt, J = 16.8, 1.4 Hz, 1H), 5.21 (dt, J = 10.5, 1.4 Hz, 1H), 4.92 (d, J = 6.3 Hz, 1H), 4.80 (s, 1H), 3.93 – 3.86 (m, 2H), 3.81 – 3.77 (m, 1H), 3.76 – 3.74 (m, 1H), 3.74 – 3.73 (m, 1H), 3.53 (dt, J = 9.1, 4.9 Hz, 1H), 3.36 (s, 3H), 2.89 – 2.84 (m, 1H), 2.06 (d, J = 9.1 Hz, 1H), 0.92 (s, 9H), 0.11 (d, J = 3.5 Hz, 6H). **^{13}C NMR** (176 MHz, $CDCl_3$) δ 140.53, 138.68, 128.82, 128.19, 126.99, 116.79, 99.19, 83.83, 76.48, 71.60, 71.23, 70.86, 64.39, 54.90, 26.04, 18.43, -5.23, -5.30. **ESI-HRMS**: Calculated for $C_{22}H_{36}O_6NaSi$ $(M+Na)^+$: 447.21734, Found: 447.21688. $[\alpha]_D^{20}$ = +3.6 (c =0.28, $CHCl_3$).

(*2R,3S,5S,6S*)-2-((benzyloxy)methyl)-5-(((*R*)-hex-1-en-3-yl)oxy)-6-methoxytetrahydro-2*H*-pyran-3,4-diol (17z)

The title product compound is prepared according to the general procedure **C1** with 5 mol% $Rh(cod)_2BF_4$ catalyst, 6% (*S*)-NPN ligand, 30 mol% Taylor's boronic acid **27**, allylic carbonate **16l** (0.24 mmol, 1.2 equiv.), and 0.2 mmol carbohydrate polyol **15f** at 50 ˚C for 24 h and isolated by flash column chromatography (5:1-3:1 Pentane: Ethyl Acetate) giving **17z** as a colorless oil (44.0 mg, 60% yield, r.r. >20:1 (C2:C1), d.r. > 20:1).

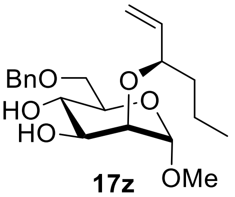

17z

^{1}H NMR (700 MHz, $CDCl_3$) δ 7.37 – 7.31 (m, 4H), 7.30 – 7.26 (m, 1H), 5.75 – 5.67 (m, 1H), 5.26 – 5.20 (m, 1H), 5.17 – 5.12 (m, 1H), 4.68 (d, J = 1.4 Hz, 1H), 4.63 (d, J = 11.9 Hz, 1H), 4.59 (d, J = 11.9 Hz, 1H), 3.87 – 3.81 (m, 1H), 3.79 – 3.73 (m, 3H), 3.72 (t, J = 9.1 Hz, 1H), 3.68 – 3.65 (m, 1H), 3.64 (dd, J = 3.5, 1.4 Hz, 1H), 3.33 (s, 3H), 2.75 (s, 1H), 2.29 (d, J = 9.1 Hz, 1H), 1.70 – 1.63 (m, 1H), 1.48 – 1.38 (m, 2H), 1.37 – 1.31 (m, 1H), 0.92 (t, J = 7.0 Hz, 3H). **^{13}C NMR** (176 MHz, $CDCl_3$) δ 139.25, 138.22, 128.51, 127.78, 127.77, 117.31, 100.07, 83.82, 76.98, 73.72, 71.92, 70.71, 70.51, 70.28, 54.94, 37.87, 18.85, 14.14. **ESI-HRMS**: Calculated for $C_{20}H_{30}O_6Na$ $(M+Na)^+$: 389.19346, Found: 389.19329. $[\alpha]_D^{20}$ = +15.6 (c =0.34, $CHCl_3$).

(*3R,5S,6S*)-5-(((*R*)-hex-1-en-3-yl)oxy)-6-methoxytetrahydro-2*H*-pyran-3,4-diol (17za)

The title product compound is prepared according to the general procedure **C1** with 5 mol% Rh(cod)$_2$BF$_4$ catalyst, 6% (*S*)-NPN ligand, 30 mol% Taylor's boronic acid **27**, allylic carbonate **16l** (0.24 mmol, 1.2 equiv.), and 0.2 mmol carbohydrate polyol **15v** at 50 °C for 24 h and isolated by flash column chromatography (5:1-1:1 Pentane: Ethyl Acetate) giving **17za** as a white solid (30.0 mg, 61% yield, r.r. >20:1 (C2:C1), d.r. > 20:1).

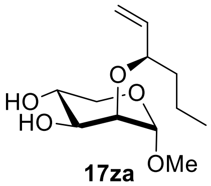

17za

^{1}H NMR (700 MHz, $CDCl_3$) δ 5.72 (ddd, J = 17.5, 10.5, 7.7 Hz, 1H), 5.22 (dt, J = 16.8, 1.4 Hz, 1H), 5.20 – 5.15 (m, 1H), 4.61 (d, J = 2.1 Hz, 1H), 3.85 – 3.81 (m, 1H), 3.83 – 3.79 (m, 1H), 3.78 – 3.73 (m, 1H), 3.75 – 3.70 (m, 1H), 3.63 – 3.60 (m, 1H), 3.47 – 3.42 (m, 1H), 3.35 (s, 3H), 2.24 – 2.16 (m, 2H), 1.70 – 1.63 (m, 1H), 1.49 – 1.43 (m, 1H), 1.43 – 1.38 (m, 1H), 1.37 – 1.33 (m, 1H), 0.93 (t, J = 7.0 Hz, 3H). **^{13}C NMR** (176 MHz, $CDCl_3$) δ 139.17, 117.44, 100.37, 83.68, 76.98, 71.90, 68.87, 62.11, 55.28, 37.85, 18.91, 14.12. **ESI-HRMS**: Calculated for $C_{12}H_{23}O_5Na$ $(M+Na)^+$: 269.13594, Found: 269.13603. $[\alpha]_D^{20}$ = +27.1 (c =0.17, $CHCl_3$).

(*3R,5S,6S*)-5-(((*S*)-hex-1-en-3-yl)oxy)-6-methoxytetrahydro-2*H*-pyran-3,4-diol (19za)

The title product compound is prepared according to the general procedure **D1** with 5 mol% Rh(cod)$_2$BF$_4$ catalyst, 6% (*R*)-NPN ligand, 30 mol% Taylor's boronic acid **27**, allylic carbonate **16l** (0.24 mmol, 1.2 equiv.), and 0.2 mmol carbohydrate polyol **15v** at 50 °C for 24 h and isolated by flash column chromatography (5:1-1:1 Pentane: Ethyl Acetate) giving **19za** as a colorless oil (35.0 mg, 73% yield, r.r. >20:1 (C2:C3), d.r. > 20:1).

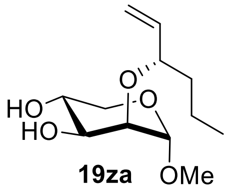

19za

^{1}H NMR (700 MHz, $CDCl_3$) δ 5.63 (ddd, J = 17.5, 10.5, 8.4 Hz, 1H), 5.23 (dd, J = 10.5, 1.4 Hz, 1H), 5.20 (ddd, J = 17.5, 1.4, 0.7 Hz, 1H), 4.62 (d, J = 2.8 Hz, 1H), 3.85 – 3.80 (m, 1H), 3.79 (dd, J = 8.4, 4.9 Hz, 1H), 3.77 – 3.74 (m, 1H), 3.71 – 3.66 (m, 1H), 3.64 (dd, J = 4.2, 2.8 Hz, 1H), 3.48 (dd, J = 10.5, 8.4 Hz, 1H), 3.39 (s, 3H), 2.26 (d, J = 8.4 Hz, 1H), 2.23 (d, J = 3.5 Hz, 1H), 1.64 – 1.58 (m, 1H), 1.48 – 1.39 (m, 2H), 1.37 – 1.30 (m, 1H), 0.92 (t, J = 7.7 Hz, 3H). **^{13}C NMR** (176 MHz, $CDCl_3$) δ 138.49, 118.53, 99.37, 81.11, 74.43, 71.68, 68.79, 62.42, 55.44, 37.86, 18.71, 14.09. **ESI-HRMS**: Calculated for $C_{12}H_{23}O_5Na$ $(M+Na)^+$: 269.13594, Found: 269.13593. $[\alpha]_D^{20}$ = -5.7 (c =0.44, $CHCl_3$).

(*2R,3R,4S,5S,6S*)-2-(((*tert*-butyldimethylsilyl)oxy)methyl)-4-(((*1R,2R*)-1-hydroxy-1,2,3,4-tetrahydronaphthalen-2-yl)oxy)-6-methoxytetrahydro-2H-pyran-3,5-diol (34)[35]

The title product compound is prepared according to the general procedure **E** and isolated by flash column chromatography (9:1-2:1 Pentane: Ethyl Acetate) giving **34** as a white solid (87.3 mg, 96% yield).

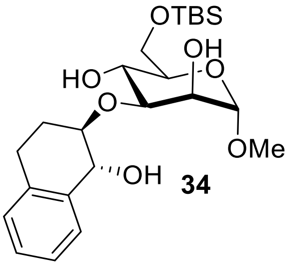

34

^{1}H NMR (600 MHz, $CDCl_3$) δ 7.55 (d, *J* = 7.7 Hz, 1H), 7.22 (t, *J* = 7.3 Hz, 1H), 7.18 (d, *J* = 7.4 Hz, 1H), 7.07 (d, *J* = 7.5 Hz, 1H), 4.74 (dd, *J* = 8.3, 2.8 Hz, 1H), 4.72 (d, *J* = 1.3 Hz, 1H), 4.02 (s, 1H), 3.91 – 3.89 (m, 2H), 3.86 (t, *J* = 9.0 Hz, 1H), 3.73 (dd, *J* = 9.1, 3.4 Hz, 1H), 3.65-3.61 (m, 3H), 3.42 (s, 1H), 3.36 (s, 3H), 3.20 (s, 1H), 2.93 – 2.84 (m, 2H), 2.21 – 2.17 (m, 1H), 1.89-1.82 (m, 1H), 0.91 (s, 9H), 0.11 (s, 6H). **^{13}C NMR** (151 MHz, $CDCl_3$) δ 137.36, 135.57, 128.28, 127.54, 127.08, 126.59, 100.81, 78.77, 76.94 (see DEPT), 72.85, 70.85, 68.68, 67.58, 64.99, 54.99, 28.38, 26.91, 26.04, 18.43, -5.29, -5.30. **ESI-HRMS**: Calculated for $C_{23}H_{38}O_7NaSi$ $(M+Na)^+$: 477.22790, Found: 477.22735. $[\alpha]_D^{20}$ = +49.5 (c = 0.4, $CHCl_3$).

(*2R,3R,4S,5S,6S*)-4-(((*1R,2R*)-1-hydroxy-1,2-dihydronaphthalen-2-yl)oxy)-2-(hydroxymethyl)-6-methoxytetrahydro-2H-pyran-3,5-diol (35) [36]

The title product compound is prepared according to the general procedure **F** and isolated by flash column chromatography (9:1-1:20 Pentane: Ethyl Acetate) giving **35** as a colorless liquid (62.3 mg, 92% yield).

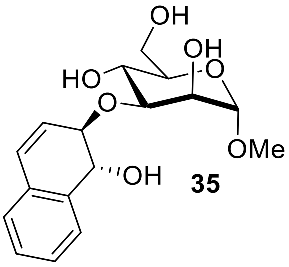

35

^{1}H NMR (500 MHz, CD_3OD) δ 7.53 (d, *J* = 7.0 Hz, 1H), 7.22 – 7.16 (m, 2H), 7.04 (d, *J* = 6.7 Hz, 1H), 6.39 (dd, *J* = 10.0, 2.0 Hz, 1H), 6.11 (dd, *J* = 9.9, 1.9 Hz, 1H), 4.85 (d, *J* = 11.7 Hz, 1H), 4.70 (d, *J* = 1.3 Hz, 1H), 4.32 (dt, *J* = 11.2, 2.0 Hz, 1H), 4.03 – 4.02 (m, 1H), 3.85 (dd, *J* = 11.8, 2.2 Hz, 1H), 3.75 – 3.71 (m, 2H), 3.65 (dd, *J* = 9.3, 3.1 Hz, 1H), 3.55-3.52 (m, 1H), 3.36 (s, 3H). **^{13}C NMR** (126 MHz, CD_3OD) δ 137.93, 133.79, 130.70, 128.66, 128.64, 128.48, 127.15, 125.73, 102.22, 81.61, 80.18, 74.46, 74.08, 69.20, 67.15, 62.94, 55.20. **ESI-HRMS**: Calculated for $C_{17}H_{22}O_7Na$ $(M+Na)^+$: 361.12577, Found: 361.12591. $[\alpha]_D^{20}$ = -27.8 (c = 0.33, CH_3OH).

(*2S,3S,4S,5R,6R*)-2-((1-benzyl-1H-1,2,3-triazol-4-yl)methoxy)-6-(((*tert*-butyldimethylsilyl)oxy)methyl)-4-(((*1R,2R*)-1-hydroxy-1,2-dihydronaphthalen-2-yl)oxy)tetrahydro-2H-pyran-3,5-diol (36) [37]

The title product compound is prepared according to the general procedure **G** and isolated by flash column chromatography (9:1-1:10 Pentane: Ethyl Acetate) giving **36** as a pale-yellow solid (0.1177 g, 96% yield).

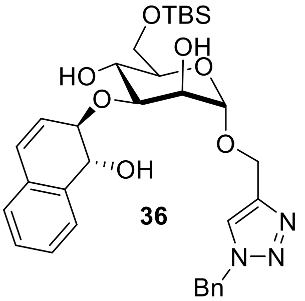

36

1H NMR (700 MHz, $CDCl_3$) δ 7.56 (d, *J* = 7.2 Hz, 1H), 7.41 (s, 1H), 7.37-7.35 (m, 3H), 7.26 – 7.25 (m, 2H), 7.22 – 7.17 (m, 2H), 7.01 (d, *J* = 7.2 Hz, 1H), 6.34 (d, *J* = 9.9 Hz, 1H), 5.94 (dd, *J* = 9.9, 1.9 Hz, 1H), 5.48 (s, 2H), 5.01 (d, *J* = 11.8 Hz, 1H), 4.93 (s, 1H), 4.76 (d, *J* = 12.5 Hz, 1H), 4.57 (d, *J* = 12.5 Hz, 1H), 4.41 (d, *J* = 11.8 Hz, 1H), 4.29 (bs, 1H), 4.05 (s, 1H), 3.92 (t, *J* = 9.4 Hz, 1H), 3.89 (d, J = 5.5 Hz, 2H), 3.79 – 3.77 (m, 1H), 3.71 – 3.68 (m, 1H), 3.56 (bs, 1H), 3.44 (s, 1H), 0.89 (s, 9H), 0.09 (d, *J* = 3.3 Hz, 6H). **^{13}C NMR** (176 MHz, $CDCl_3$) δ 144.41, 136.52, 134.49, 132.32, 129.29, 129.16, 128.95, 128.38, 128.28, 127.96, 127.69, 126.21, 124.51, 122.86, 98.92, 79.83, 77.24, 72.61, 71.26, 68.81, 67.57, 64.96, 60.21, 54.32, 26.02, 18.39, -5.28, -5.33. **ESI-HRMS**: Calculated for $C_{32}H_{44}O_7N_3Si$ $(M+H)^+$: 610.2943, Found: 610.29391. $[\alpha]_D^{20}$ = -5.7 (c = 0.56, $CHCl_3$).

(*2R,3S,4S,5R,6R*)-2-(acetoxymethyl)-6-(4-((((*2S,3S,4S,5R,6R*)-6-(((*tert*-butyldimethylsilyl)oxy)methyl)-3,5-dihydroxy-4-(((1R,2R)-1-hydroxy-1,2-dihydronaphthalen-2-yl)oxy)tetrahydro-2H-pyran-2-yl)oxy)methyl)-1H-1,2,3-triazol-1-yl)tetrahydro-2H-pyran-3,4,5-triyl triacetate (37)[37]

The title product compound is prepared according to the general procedure **G** and isolated by flash column chromatography (9:1-1:10 Pentane: Ethyl Acetate) giving **37** as a pale-yellow solid (0.1076 g, 63% yield).

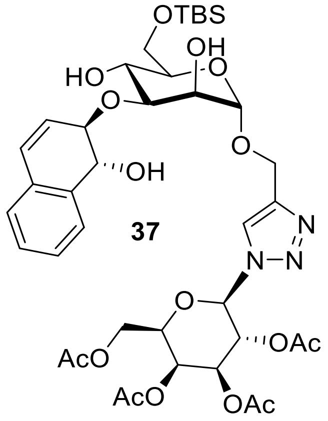

37

^{1}H NMR (500 MHz, $CDCl_3$) δ 7.78 (s, 1H), 7.57 (d, *J* = 7.2 Hz, 1H), 7.22 (p, *J* = 7.3 Hz, 2H), 7.03 (d, *J* = 7.0 Hz, 1H), 6.39 (d, *J* = 9.9 Hz, 1H), 5.98 (dd, *J* = 10.0, 1.8 Hz, 1H), 5.89 – 5.83 (m, 1H), 5.43 – 5.37 (m, 2H), 5.27 – 5.19 (m, 1H), 5.04 (dd, *J* = 12.1, 3.8 Hz, 1H), 4.87 (d, *J* = 1.6 Hz, 1H), 4.80 (d, *J* = 12.8 Hz, 1H), 4.66 (d, *J* = 12.8 Hz,1H), 4.47 (d, *J* = 11.8 Hz, 1H), 4.30 (dd, *J* = 12.7, 4.9 Hz, 1H), 4.13 (d, *J* = 11.6 Hz, 1H), 4.05 (s, 1H), 4.01 – 3.9 (m, 5H), 3.85 – 3.82 (m, 1H), 3.74 (dt, *J* = 10.3, 5.5 Hz, 1H), 3.45 (s, 1H), 3.27 (bs, 1H), 2.06 (s, 6H), 2.02 (s, 3H), 1.85 (s, 3H), 0.92 (s, 9H), 0.12 (s, 6H). **^{13}C NMR** (126 MHz, $CDCl_3$) δ 170.68, 170.04, 169.49, 169.45, 145.06, 136.43, 132.31, 129.11, 128.56, 128.00, 127.76, 126.28, 124.38, 121.36, 98.43, 85.84, 80.06, 76.97 (see dept), 75.31, 72.63, 72.60, 71.07, 70.50, 69.10, 67.79, 67.74, 65.15, 61.60, 59.97, 26.04, 20.84, 20.67, 20.65, 20.32, 18.42, -5.27, -5.33. **ESI-HRMS**: Calculated for $C_{39}H_{56}O_{16}N_3Si$ $(M+H)^+$: 850.34244, Found: 850.34229. $[\alpha]_D^{20}$ = -15.5 (c = 0.38, $CHCl_3$).

(*2R,3R,4R,6S*)-2-(((*tert*-butyldimethylsilyl)oxy)methyl)-4-(((*1S,2S*)-1-hydroxy-1,2-dihydronaphthalen-2-yl)oxy)-6-(((*3aR,5R,5aS,8aS,8bR*)-2,2,7,7-tetramethyltetrahydro-5H-bis([1,3]dioxolo)[4,5-b:4',5'-d]pyran-5-yl)methoxy)tetrahydro-2H-pyran-3-ol (39)[38]

The title product compound is prepared according to the general procedure **G** and isolated by flash column chromatography (9:1-1:10 Pentane: Ethyl Acetate) giving **39** as a white foam, inseparable mixture, *α/β* ratio was determined from isolated compound. (22.2 mg, 67% yield, *α/β* ratio 10:1)

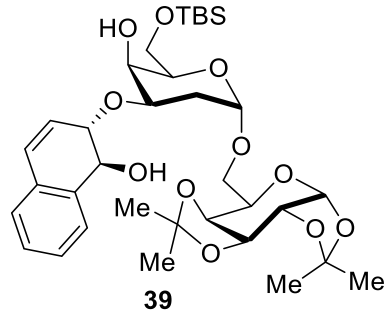

39

^{1}H NMR (500 MHz, $CDCl_3$) δ 7.63 (d, *J* = 7.4 Hz, 1H), 7.27 – 7.20 (m, 2H), 7.05 (d, *J* = 7.2 Hz, 1H), 6.37 (dd, *J* = 9.9, 2.4 Hz, 1H), 5.91 (dd, *J* = 9.9, 1.7 Hz, 1H), 5.52 (d, *J* = 5.0 Hz, 1H), 5.04 (d, *J* = 3.2 Hz, 1H), 4.95 (d, *J* = 11.6 Hz, 1H), 4.61 (dd, *J* = 7.9, 2.4 Hz, 1H), 4.49 (bs, 1H), 4.40 (dt, *J* = 11.6, 2.0 Hz, 1H), 4.32 (dd, *J* = 5.0, 2.4 Hz, 1H), 4.28 – 4.22 (m, 3H), 3.98 – 3.88 (m, 4H), 3.73 (dd, *J* = 10.7, 6.9 Hz, 1H), 3.67 – 3.64 (m, 2H), 2.16 (td, *J* = 12.8, 3.8 Hz, 1H), 1.94 (dd, *J* = 13.0, 5.1 Hz, 1H), 1.53 (s, 3H), 1.44 (s, 3H), 1.35 (s, 3H), 1.33 (s, 3H), 0.90 (s, 9H), 0.09 (d, *J* = 3.8 Hz, 6H). **^{13}C NMR** (126 MHz, $CDCl_3$) δ 136.39, 132.22, 129.89, 127.96, 127.90, 127.55, 126.13, 124.53, 109.51, 108.71, 97.65, 96.48, 79.10, 73.28, 72.47, 71.29, 70.79, 70.68, 68.25, 66.74, 66.31, 65.85, 65.77, 31.17, 26.26, 26.11, 25.99, 25.05, 24.68, 18.44, -5.32, -5.38. **ESI-HRMS**: Calculated for $C_{34}H_{52}O_{11}NaSi$ $(M+Na)^{+}$: 687.31711, Found: 687.31606. $[\alpha]_D^{20}$ = +40.2 (c = 1.41, $CHCl_3$).

NMR monitoring data for kinetics studies:

1. NMR monitoring under standard conditions

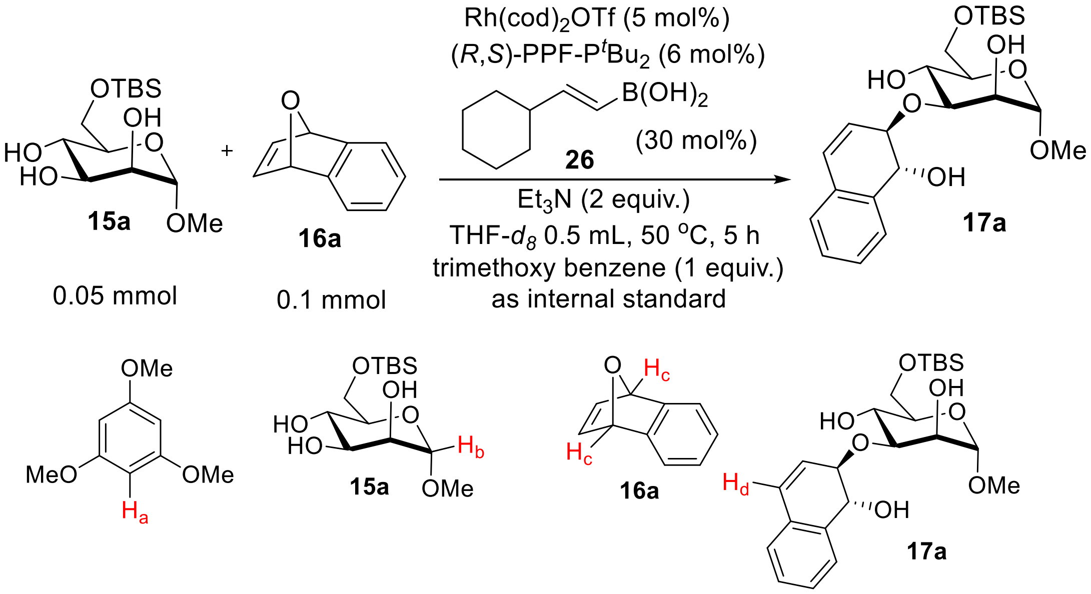

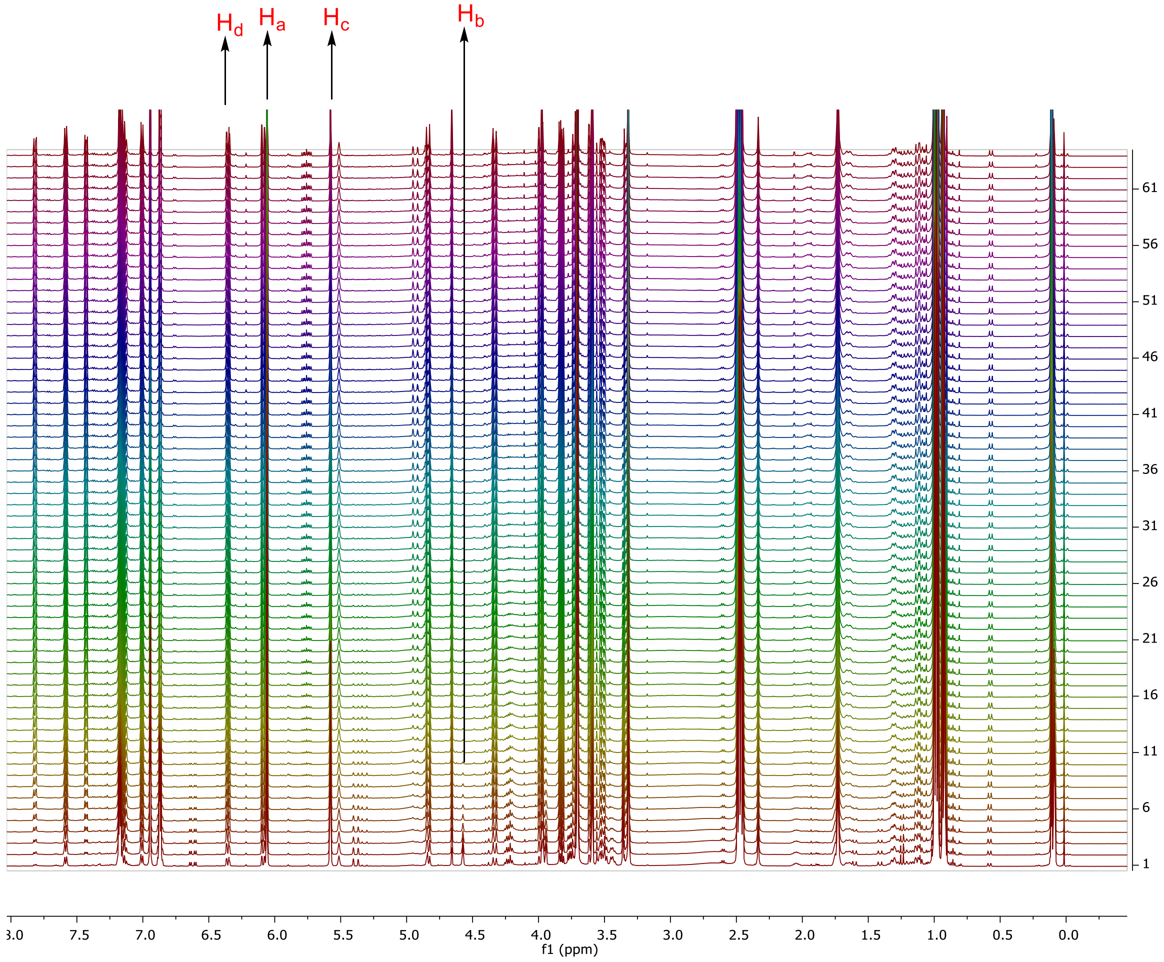

Supplementary Figure 6: Stacked ^{1}H NMR spectra for the monitoring under standard conditions

Supplementary Table 33: Concentration for **15a**, **16a**, **17a** calculated by ^{1}H NMR analysis for the monitoring under standard conditions.

Time/min	[**17a**]/M	[**15a**]/M	[**16a**]/M
9	0.012159463	0.074806469	0.173856056
14	0.032324157	0.053944349	0.153372971
19	0.045475177	0.038964861	0.136740888
24	0.057219216	0.026562816	0.11951751
29	0.06683156	0.018375647	0.10493222
34	0.074169689	0.012462691	0.092954694
39	0.077353588	0.009005886	0.083296867
44	0.081780724	0.007216838	0.076443903
49	0.083782032	0.00585231	0.070728046
54	0.085237529	0.00479101	0.065891551
59	0.086086569	0.004487782	0.062874428
64	0.0881182	0.004093585	0.060175694
69	0.089543374	0.004093585	0.058523099
74	0.091878233	0.004032939	0.057476961
79	0.091878233	0.004063262	0.056173078
84	0.091878233	0.003699388	0.05491468

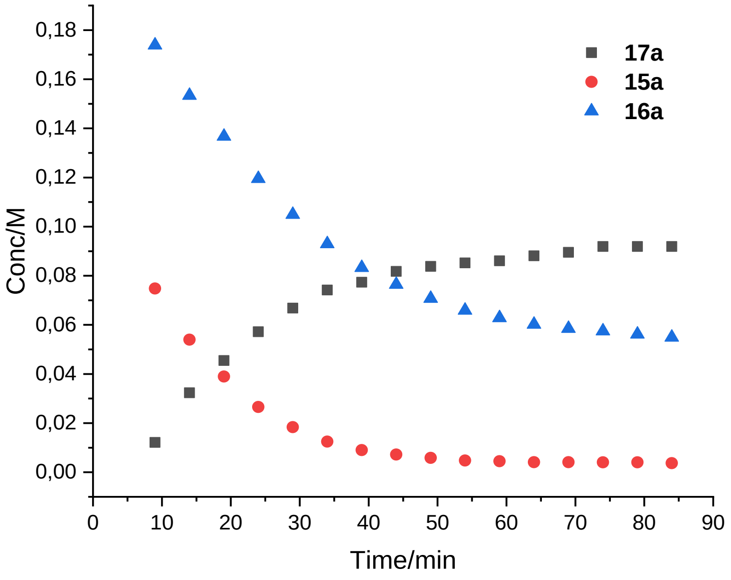

Supplementary Figure 7: Temporal kinetics profile for the monitoring under standard conditions

2. Concentration dependence studies

Supplementary Table 34. Overview for the concentration dependence experiments

Entry	% of $Rh(cod)_2OTf$	% of Boronic acid catalyst **26**	Concentration of **15a**	Concentration of **16a**	Concentration of $\mathbf{Et_3N}$
1	5 mol%	30 mol%	0.1 M	0.2 M	0.2 M
2	7 mol%	30 mol%	0.1 M	0.2 M	0.2 M
3	9 mol%	30 mol%	0.1 M	0.2 M	0.2 M
4	5 mol%	20 mol%	0.1 M	0.2 M	0.2 M
5	5 mol%	35 mol%	0.1 M	0.2 M	0.2 M
6	5 mol%	30 mol%	0.05 M	0.2 M	0.2 M
7	5 mol%	30 mol%	0.15 M	0.2 M	0.2 M
8	5 mol%	30 mol%	0.2 M	0.2 M	0.2 M
9	5 mol%	30 mol%	0.1 M	0.15 M	0.2 M
10	5 mol%	30 mol%	0.1 M	0.25 M	0.2 M
11	5 mol%	30 mol%	0.1 M	0.3 M	0.2 M
12	5 mol%	30 mol%	0.1 M	0.2 M	0.15 M
13	5 mol%	30 mol%	0.1 M	0.2 M	0.25 M
14	5 mol%	30 mol%	0.1 M	0.2 M	0.3 M

2.1 Rh(cod)$_2$OTf Catalyst concentration dependence

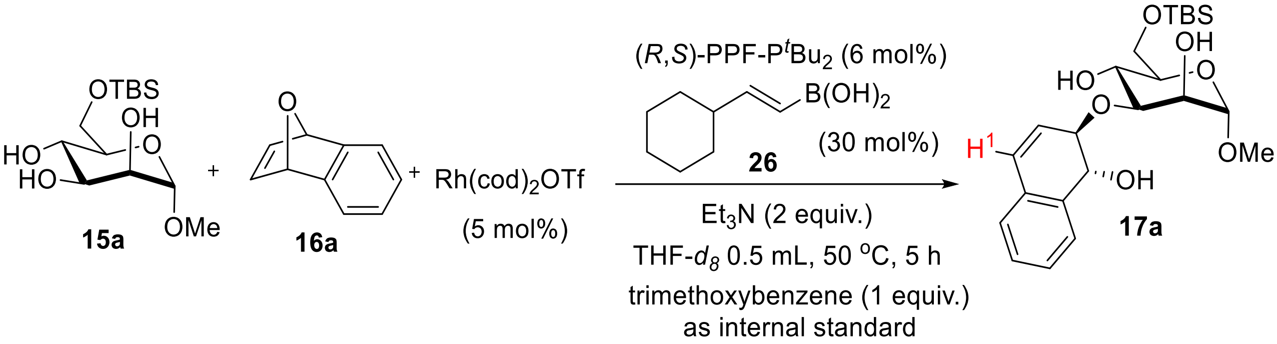

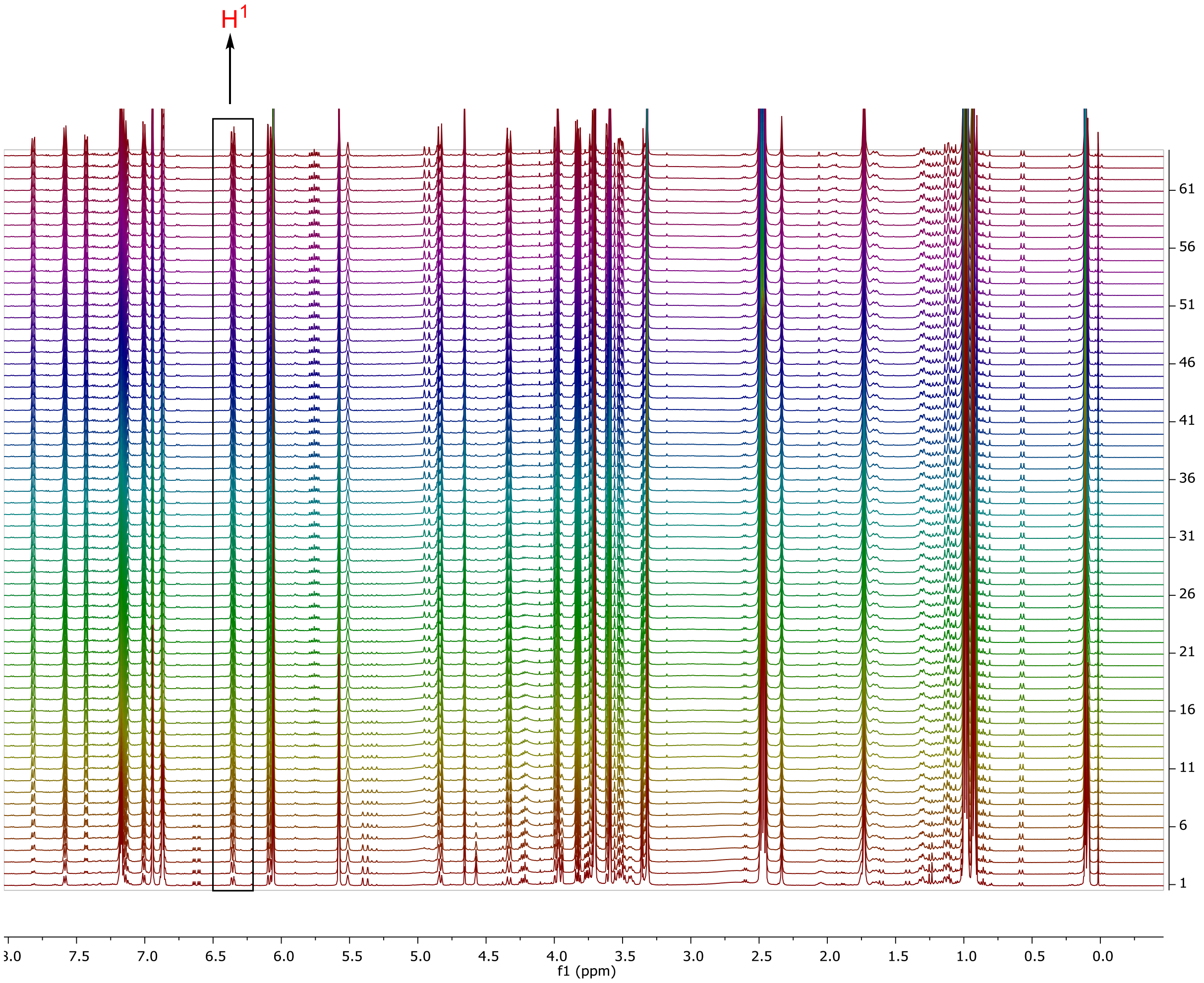

Supplementary Figure 8: Stacked ^{1}H spectra for the experiment of entry 1 in Supplementary Table 34

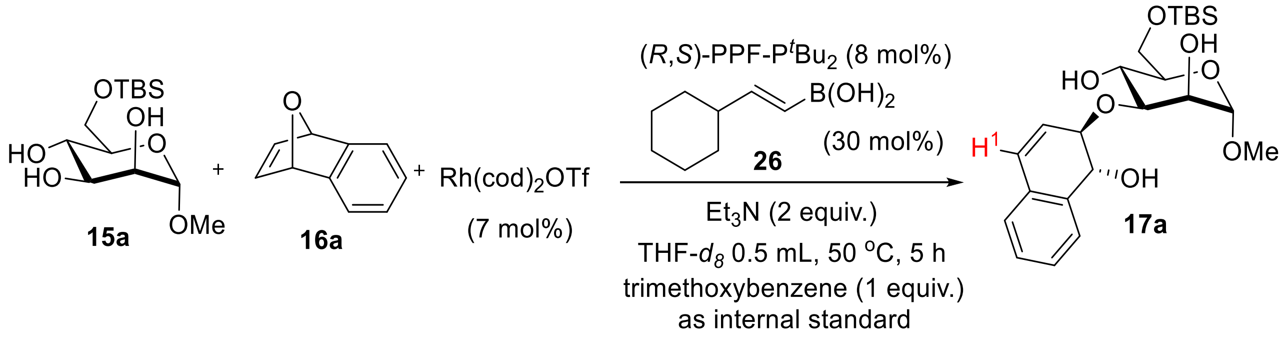

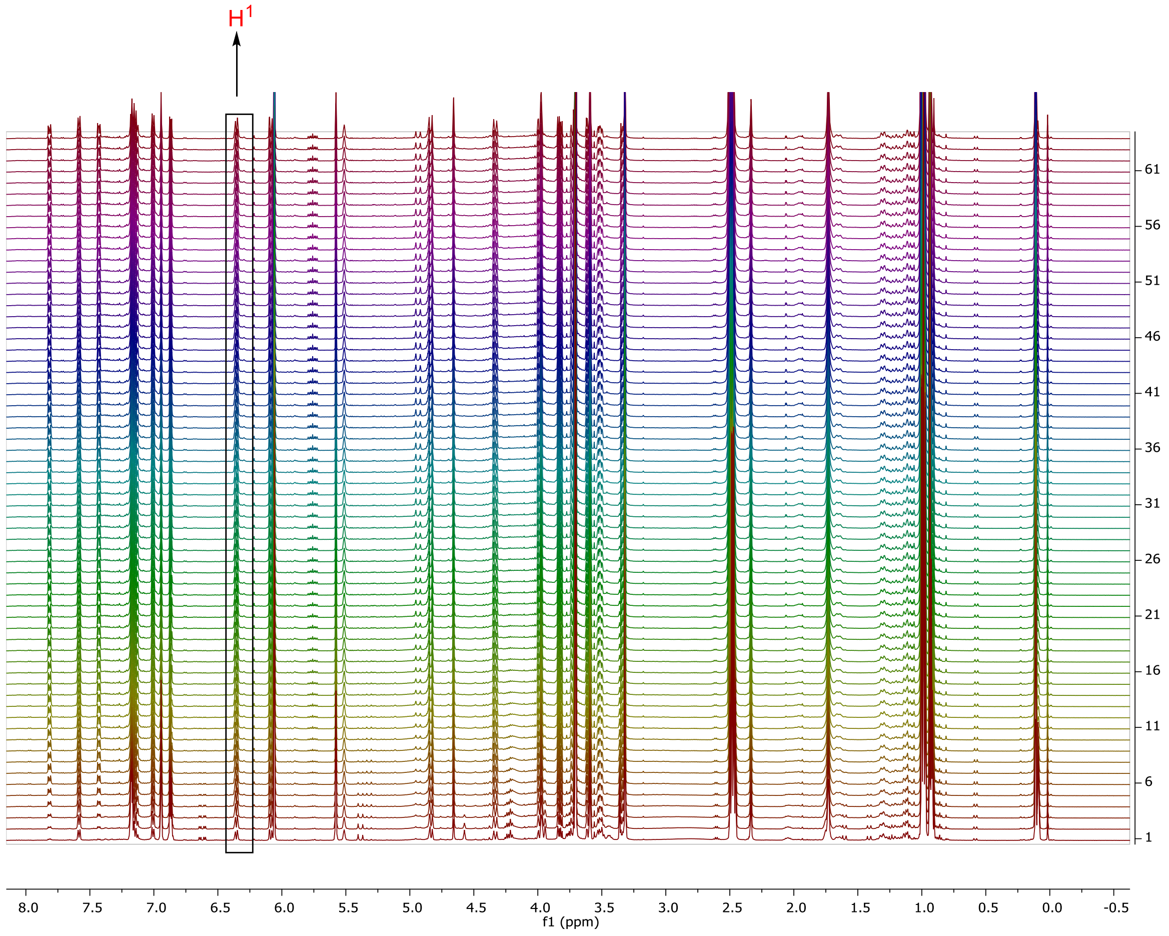

Supplementary Figure 9: Stacked ^{1}H spectra for the experiment of entry 2 in Supplementary Table 34

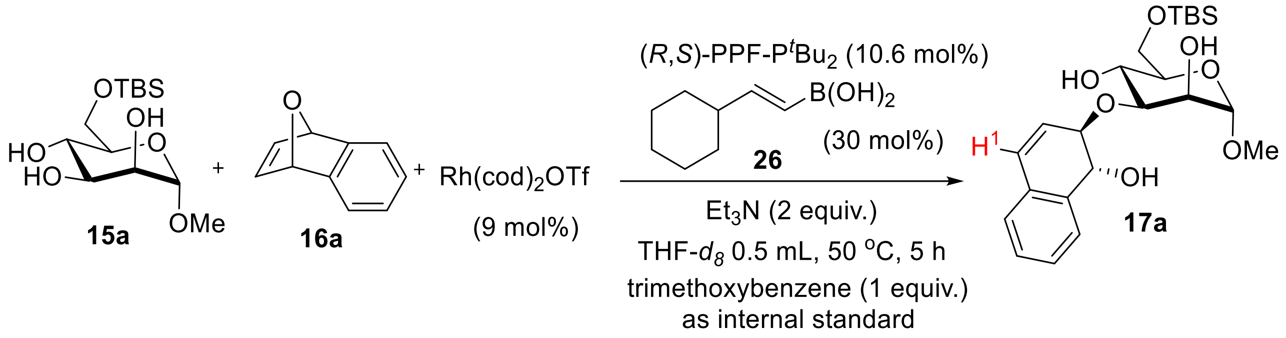

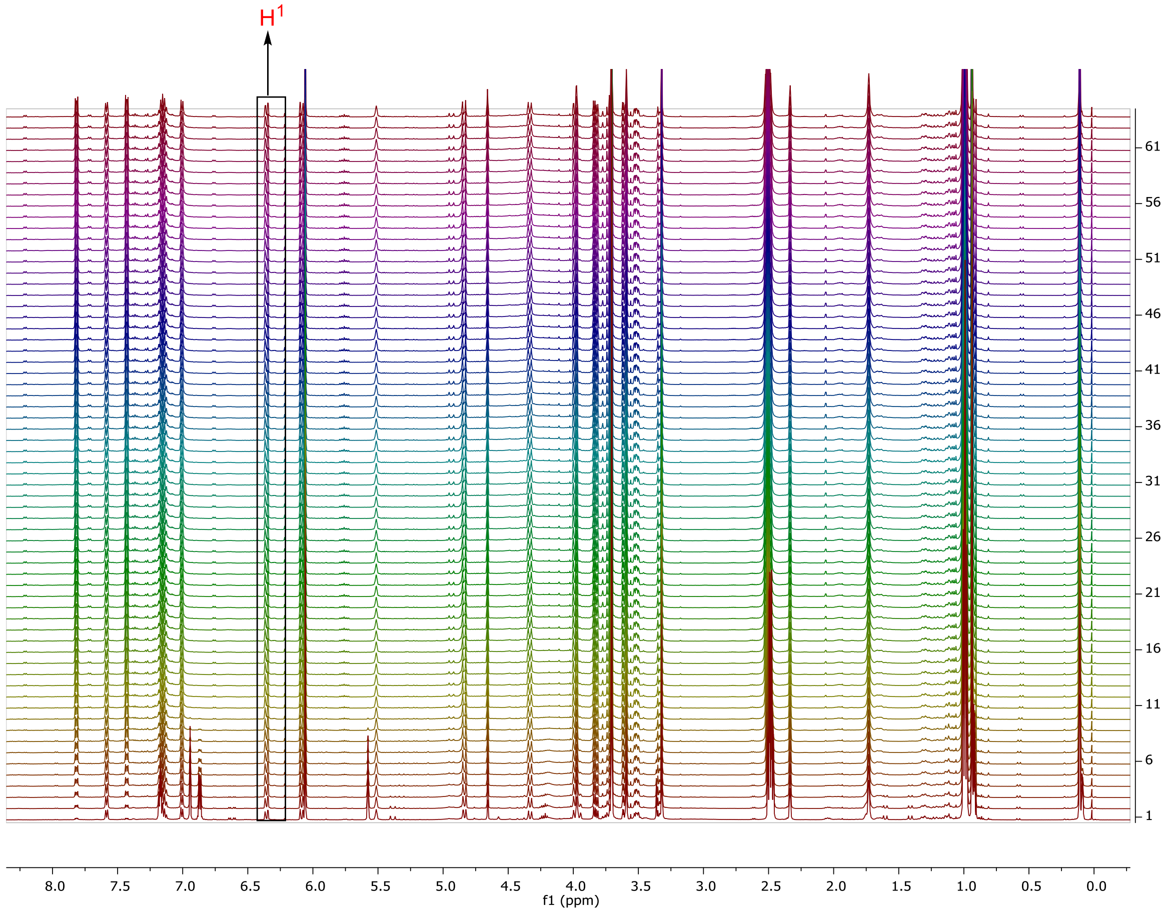

Supplementary Figure 10: Stacked ^{1}H spectra for the experiment of entry 3 in Supplementary Table 34

Supplementary Table 35. Concentration for **6** calculated by ^{1}H NMR analysis for the experiments of entry 1-3 in supplementary Table 34

Entry	5 mol% $Rh(cod)_2OTf$		7 mol% $Rh(cod)_2OTf$		9 mol% $Rh(cod)_2OTf$	
	Time/min	[**17a**]	Time/min	[**17a**]	Time/min	[**17a**]
1	9	0.012159463	8	0.019739105	9	0.026100482
2	14	0.032324157	13	0.046244129	14	0.064457102
3	19	0.045475177	18	0.062196801	19	0.080668767
4	24	0.057219216	23	0.074269933	24	0.085673108
5	29	0.06683156	28	0.083363577	29	0.091156906
6	34	0.074169689	33	0.088081099	34	0.091576431
7	39	0.077353588	38	0.089012189	39	0.093883822
8	44	0.081780724	43	0.090843332	44	0.094213449
9	49	0.083782032	48	0.090936441	49	0.095082466
10	54	0.085237529	53	0.090998514	54	0.095142398
11	59	0.086086569	58	0.091091623	59	0.095142398
12	64	0.0881182	63	0.091277841	64	0.095142398
13	69	0.089543374	68	0.091401986	69	0.095142398
14	74	0.091878233	73	0.091557167		
15	79	0.091878233	78	0.091774422		
16	84	0.091878233	83	0.092333076		
17			88	0.092581366		
18			93	0.092612403		
19			98	0.092612403		
20			103	0.092612403		

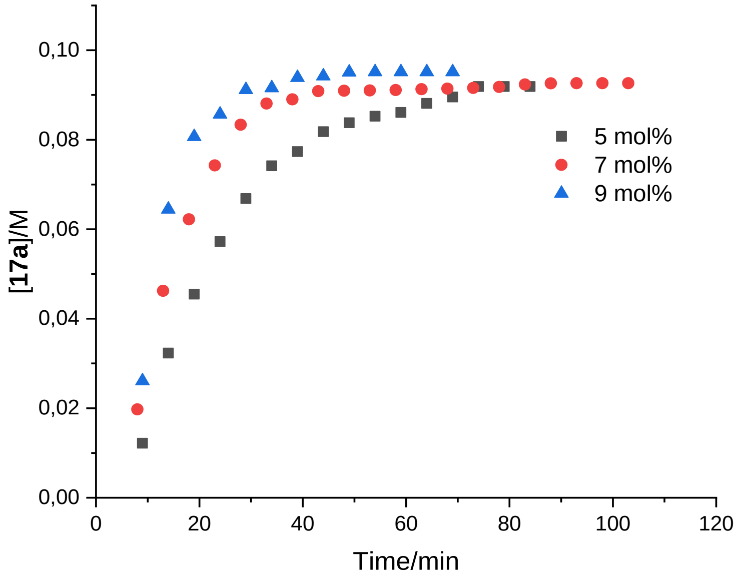

Supplementary Figure 11: Overlapped profile for the $Rh(cod)_2OTf$ concentration dependence experiments.

2.2 Boronic acid 26 Catalyst concentration dependence

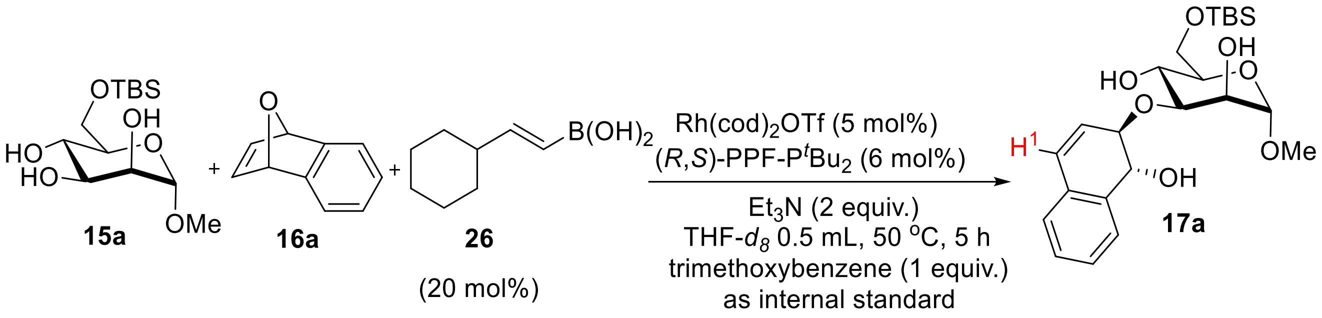

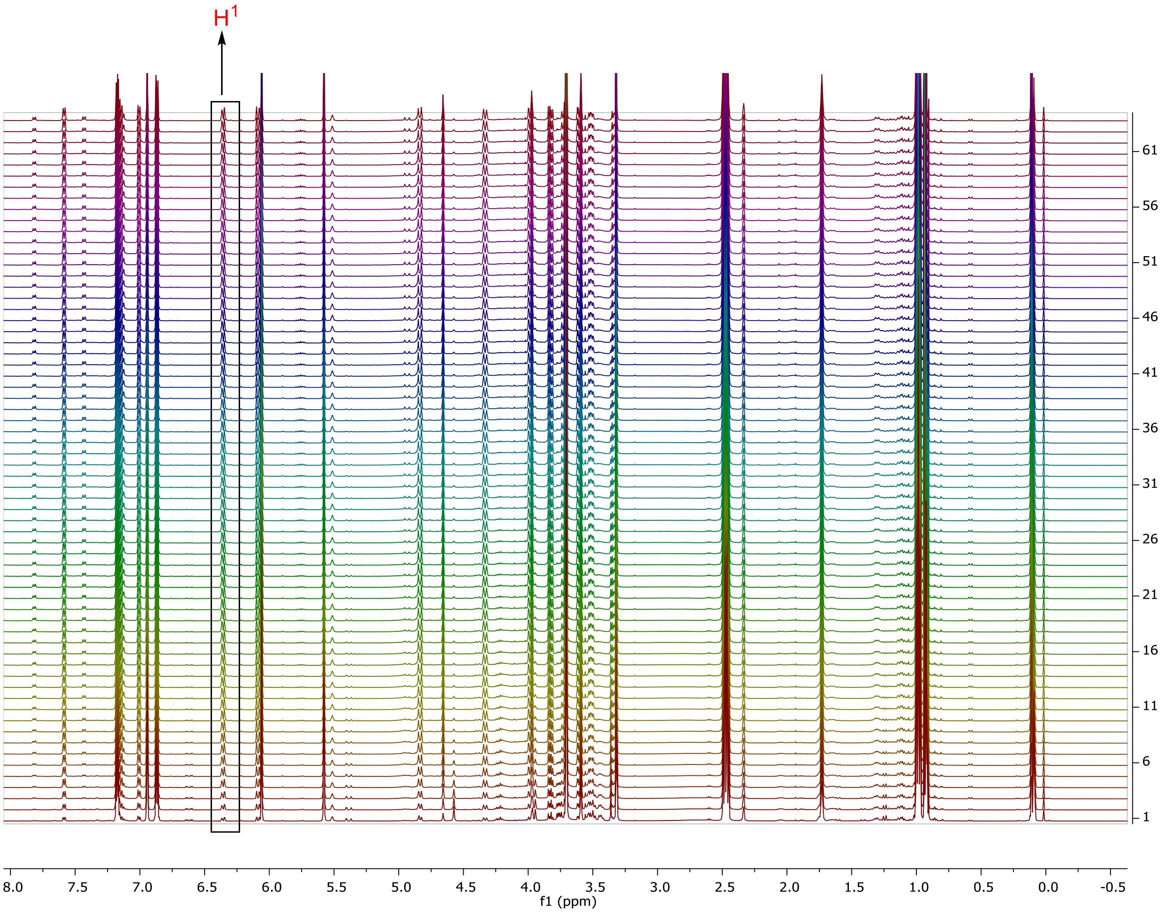

Supplementary Figure 12: Stacked ^{1}H spectra for the experiment of entry 4 in Supplementary Table 34

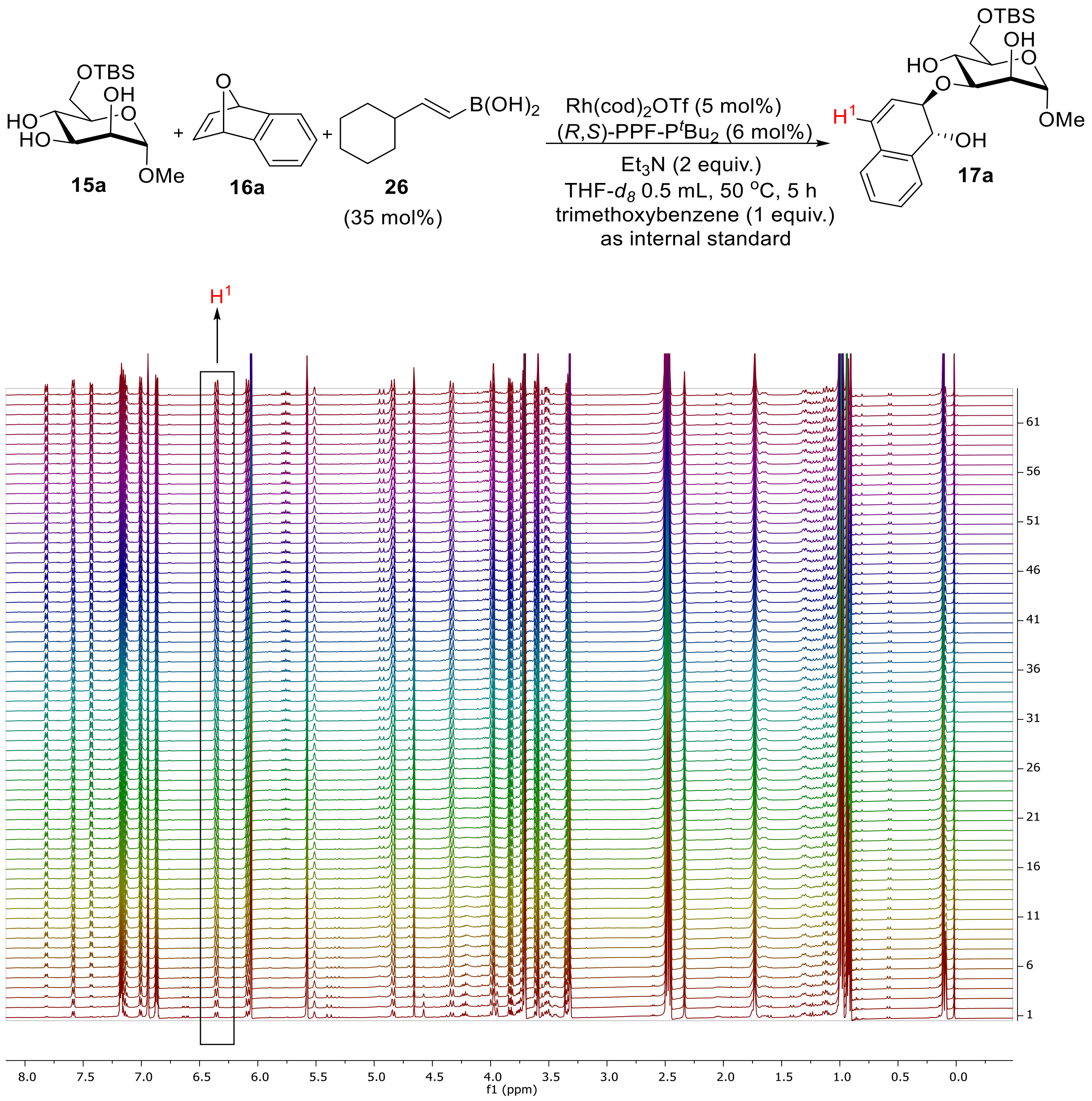

Supplementary Figure 13: Stacked ^{1}H spectra for the experiment of entry 5 in Supplementary Table 34

Supplementary Table 36. Concentration for **17a** calculated by ^{1}H NMR analysis for the experiments of entry 1, 4 & 5 in Supplementary Table 34

Entry	30 mol% catalyst **26**		20 mol% catalyst **26**		35 mol% catalyst **26**	
	Time/min	[**17a**]	Time/min	[**17a**]	Time/min	[**17a**]
1	9	0.012159463	9	0.007792615	10	0.015485701
2	14	0.032324157	14	0.025617456	15	0.040387181
3	19	0.045475177	19	0.035619002	20	0.053652179
4	24	0.057219216	24	0.043871812	25	0.067094833
5	29	0.06683156	29	0.055223259	30	0.075178191
6	34	0.074169689	34	0.062525001	35	0.082876628
7	39	0.077353588	39	0.068231405	40	0.08746608
8	44	0.081780724	44	0.07231179	45	0.088532017
9	49	0.083782032	49	0.07531839	50	0.091344908
10	54	0.085237529	54	0.077680718	55	0.095075688
11	59	0.086086569	59	0.078969261	60	0.095105298
12	64	0.0881182	64	0.080288483	65	0.095134907
13	69	0.089543374	69	0.080411202	70	0.095134907
14	74	0.091878233	74	0.081638385	75	0.095134907
15	79	0.091878233	79	0.081822463		
16	84	0.091878233	84	0.081914501		
17			89	0.082221297		
18			94	0.082374695		
19			99	0.082620132		
20			104	0.082681491		

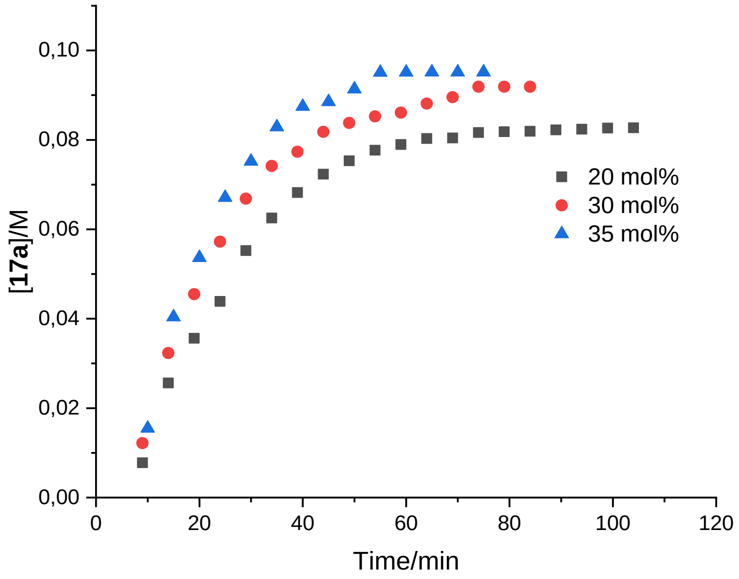

Supplementary Figure 14: Overlapped profile for the boronic acid catalyst concentration dependence experiments.

2.3 Carbohydrate polyol 15a concentration dependence

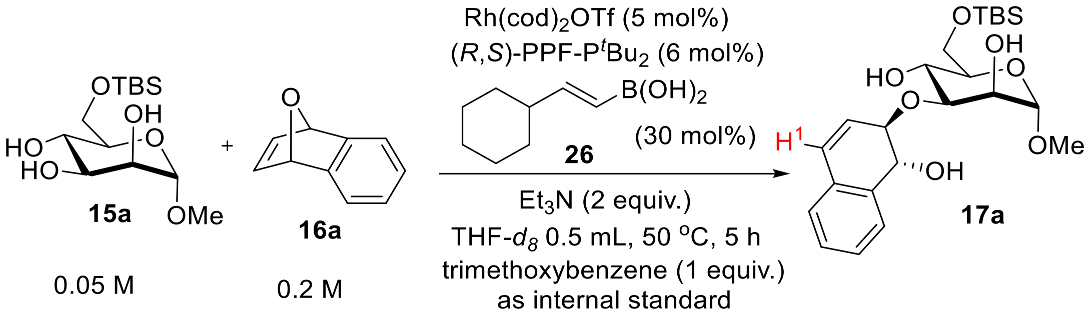

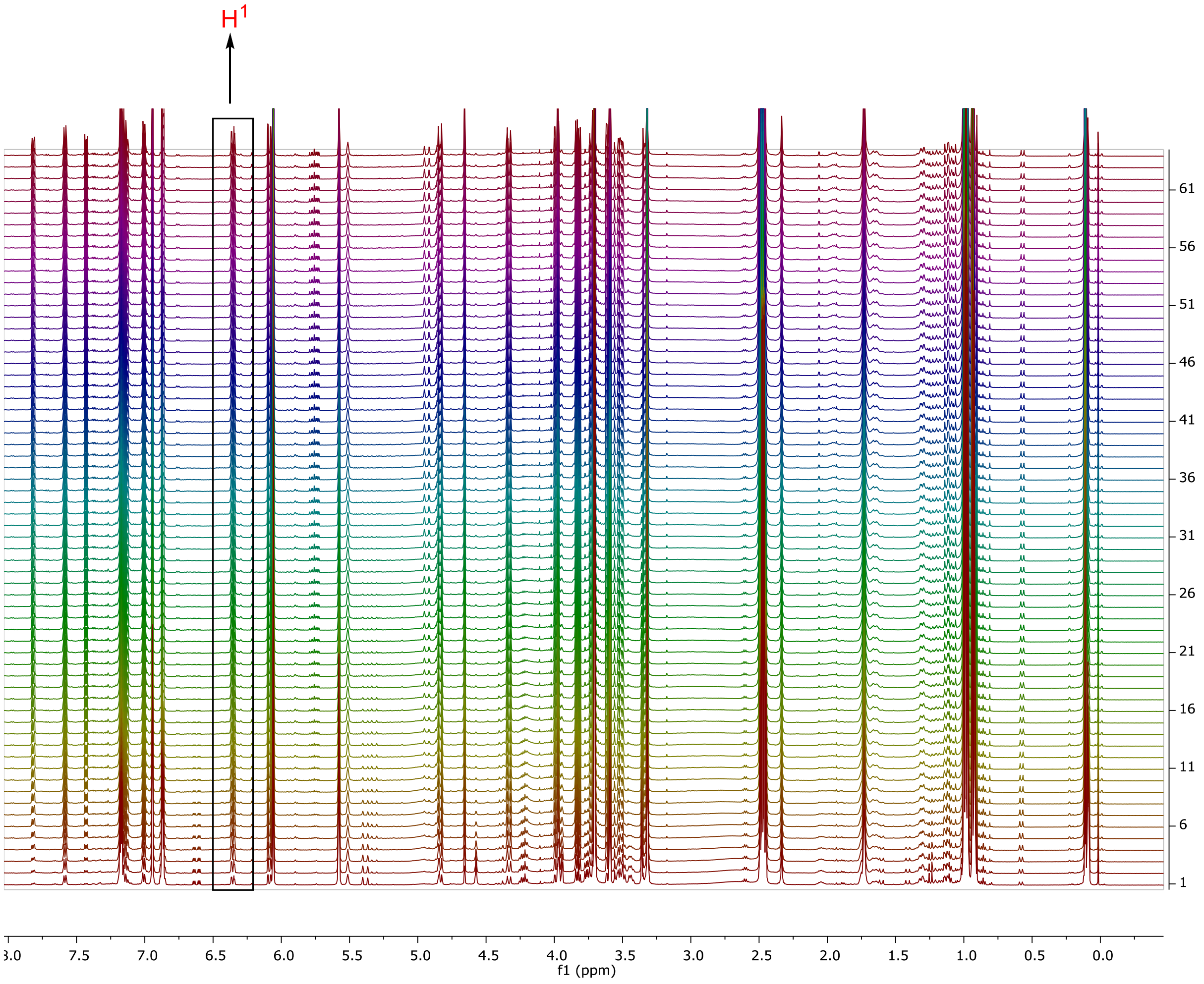

Supplementary Figure 15: Stacked ^1H spectra for the experiment of entry 6 in Supplementary Table 34

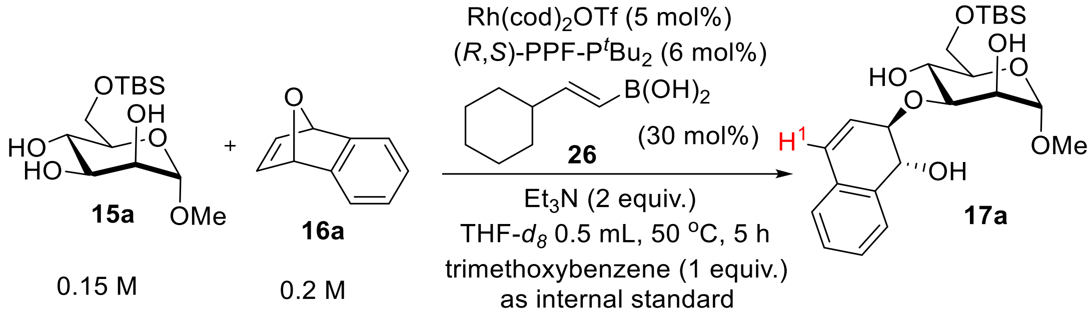

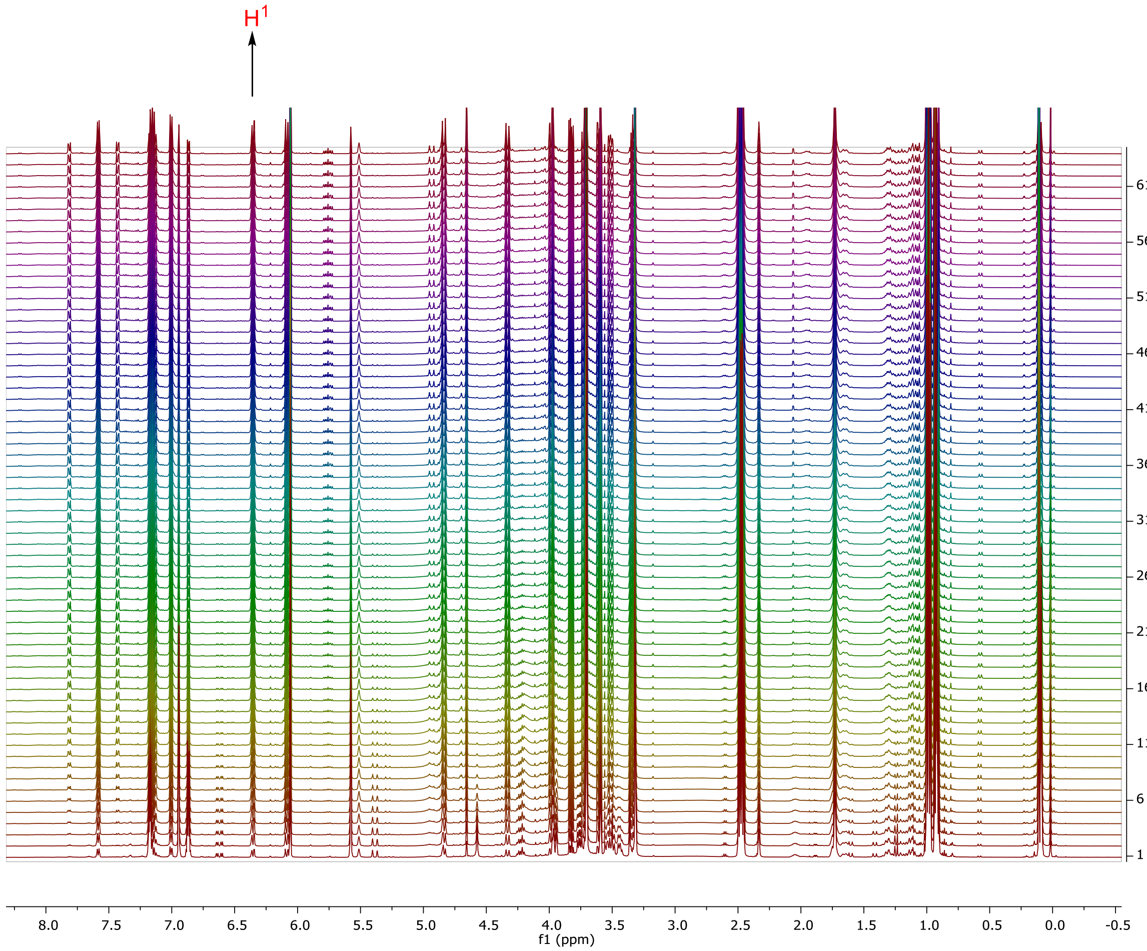

Supplementary Figure 16: Stacked ^{1}H spectra for the experiment of entry 7 in Supplementary Table 34

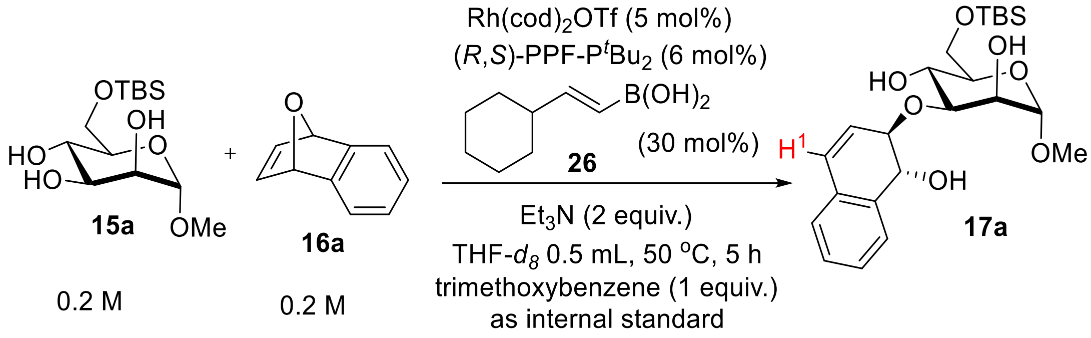

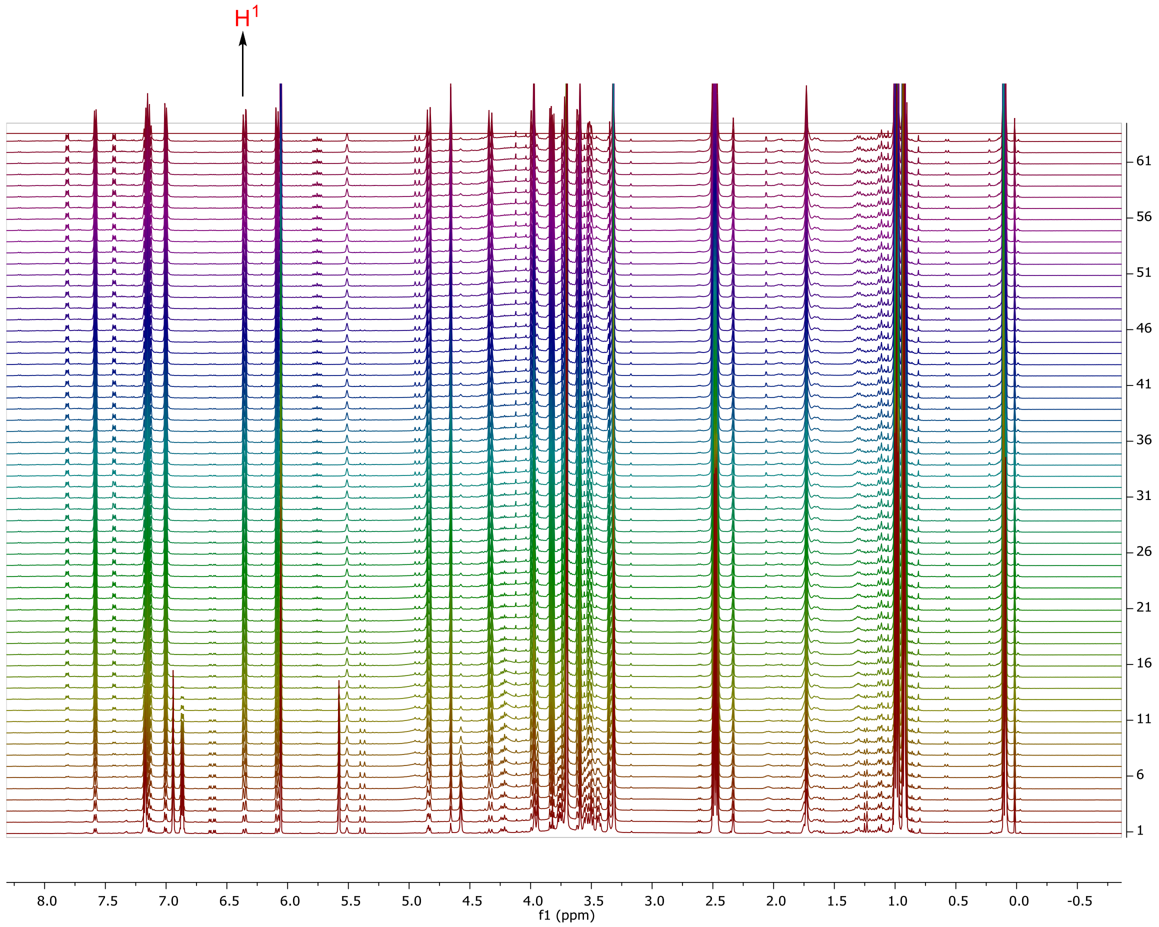

Supplementary Figure 17: Stacked ^{1}H spectra for the experiment of entry 8 in Supplementary Table 34

Supplementary Table 37. Concentration for **17a** calculated by ^{1}H NMR analysis for the experiments of entry 1, 6-8 in Supplementary Table 34

Entry	0.05 M-**15a**		0.1 M-**15a**		0.15 M-**15a**		0.2 M-**15a**	
	Time/min	[**17a**]	Time/min	[**17a**]	Time/min	[**17a**]	Time/min	[**17a**]
1	9	0.01212914	9	0.012159463	9	0.00823169	10	0.004146346
2	14	0.048516559	14	0.032324157	14	0.02381167	15	0.014566975
3	19	0.064587669	19	0.045475177	19	0.034392404	20	0.029697228
4	24	0.079627802	24	0.057219216	24	0.045274297	25	0.03813074
5	29	0.082114276	29	0.06683156	29	0.055272789	30	0.046282614
6	34	0.086298829	34	0.074169689	34	0.063845793	35	0.054121555
7	39	0.087511743	39	0.077353588	39	0.070913001	40	0.061616272
8	44	0.089149177	44	0.081780724	44	0.076574798	45	0.070613061
9	49	0.090665319	49	0.083782032	49	0.081433503	50	0.074024018
10	54	0.090968547	54	0.085237529	54	0.084244324	55	0.079328212
11	59	0.091271776	59	0.086086569	59	0.085790275	60	0.083537145
12	64	0.09163565	64	0.0881182	64	0.087858237	65	0.085352149
13	69	0.092120816	69	0.089543374	69	0.08946442	70	0.086838575
14	74	0.092424044	74	0.091878233	74	0.089805734	75	0.087417499
15	79	0.092424044	79	0.091878233	79	0.090046662	80	0.087996423
16	84	0.092424044	84	0.091878233	84	0.090247434	85	0.088966512
17					89	0.090347821	90	0.088982158
18					94	0.090367898	95	0.089654962
19					99	0.090408053	100	0.089905307
20					104	0.09042813	105	0.089905307

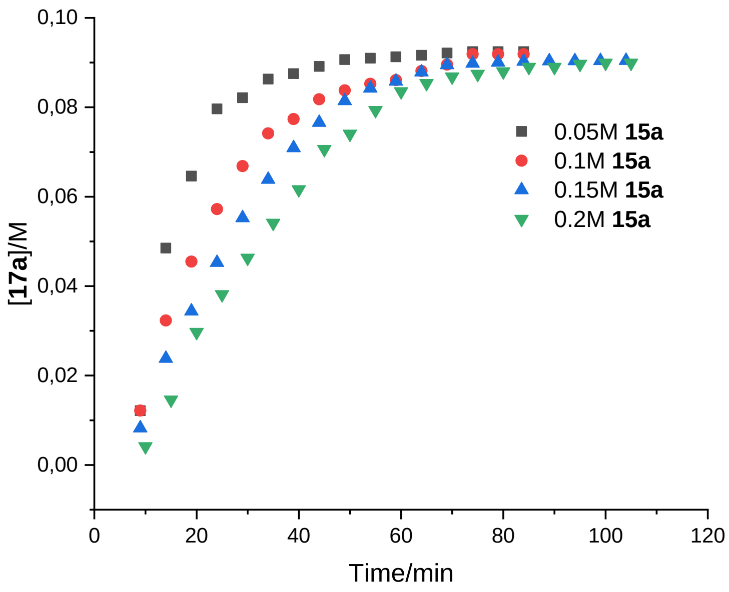

Supplementary Figure 18: Overlapped profile for the carbohydrate polyol **15a** concentration dependence experiments

2.4 Oxabicycle 16a concentration dependence

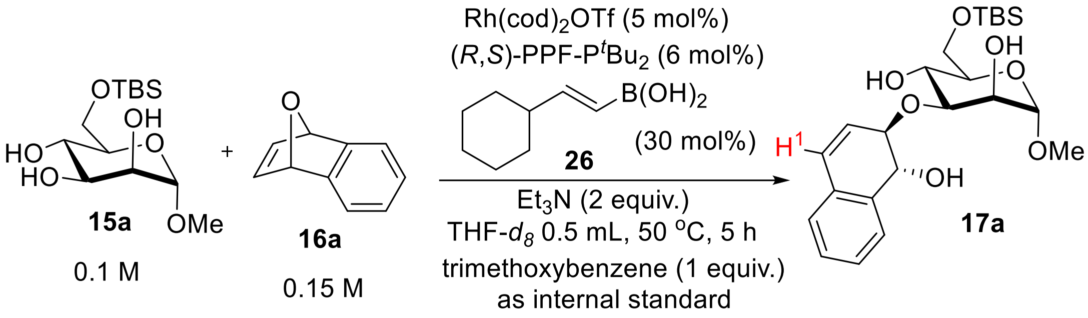

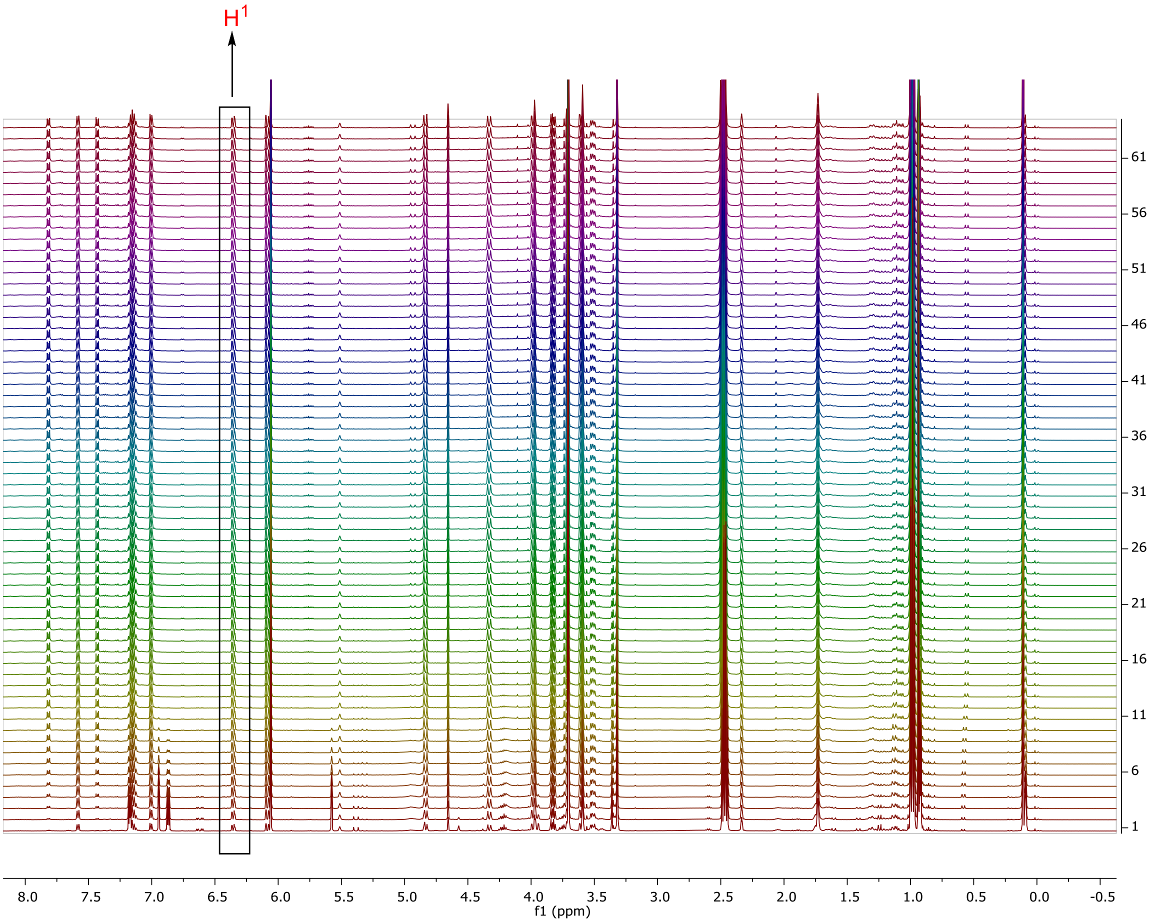

Supplementary Figure 19: Stacked ^{1}H spectra for the experiment of entry 9 in Supplementary Table 34

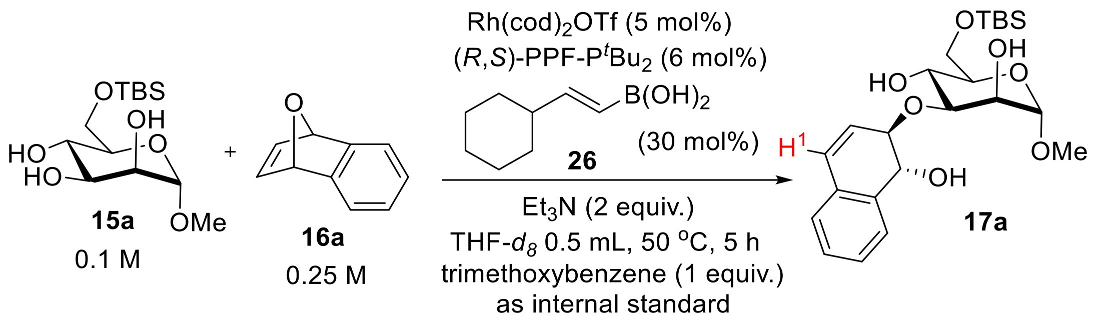

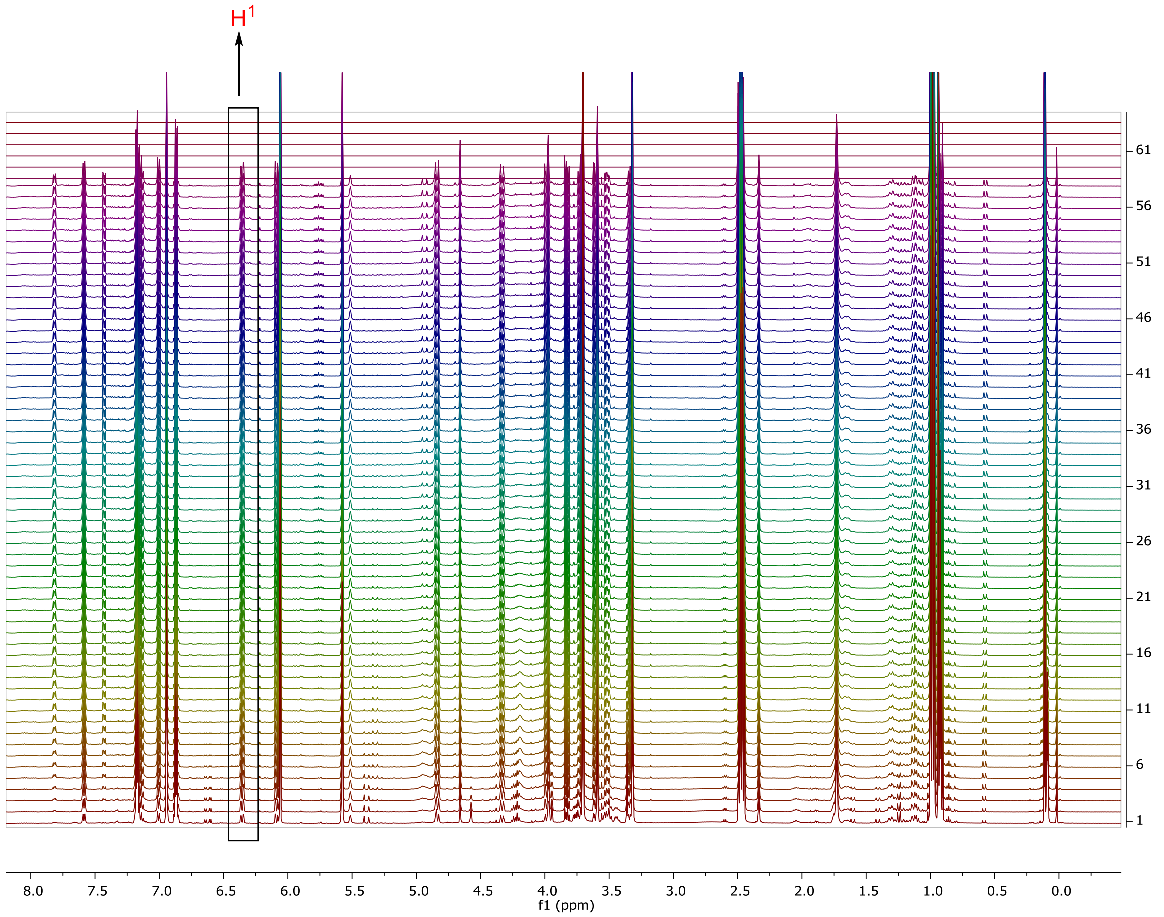

Supplementary Figure 20: Stacked ^{1}H spectra for the experiment of entry 10 in Supplementary Table 34

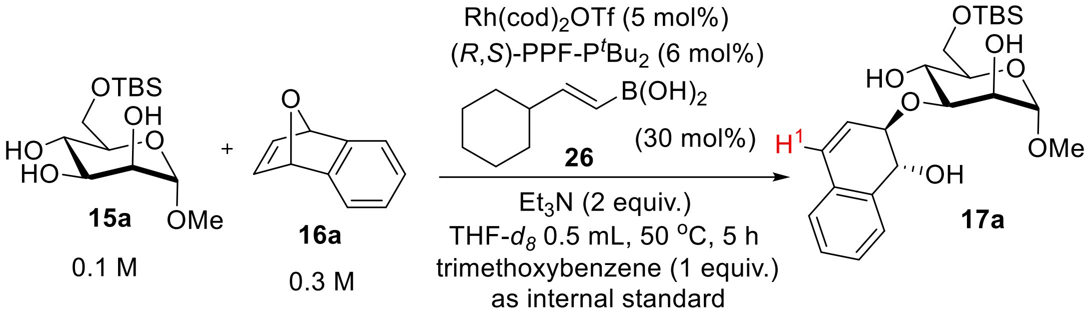

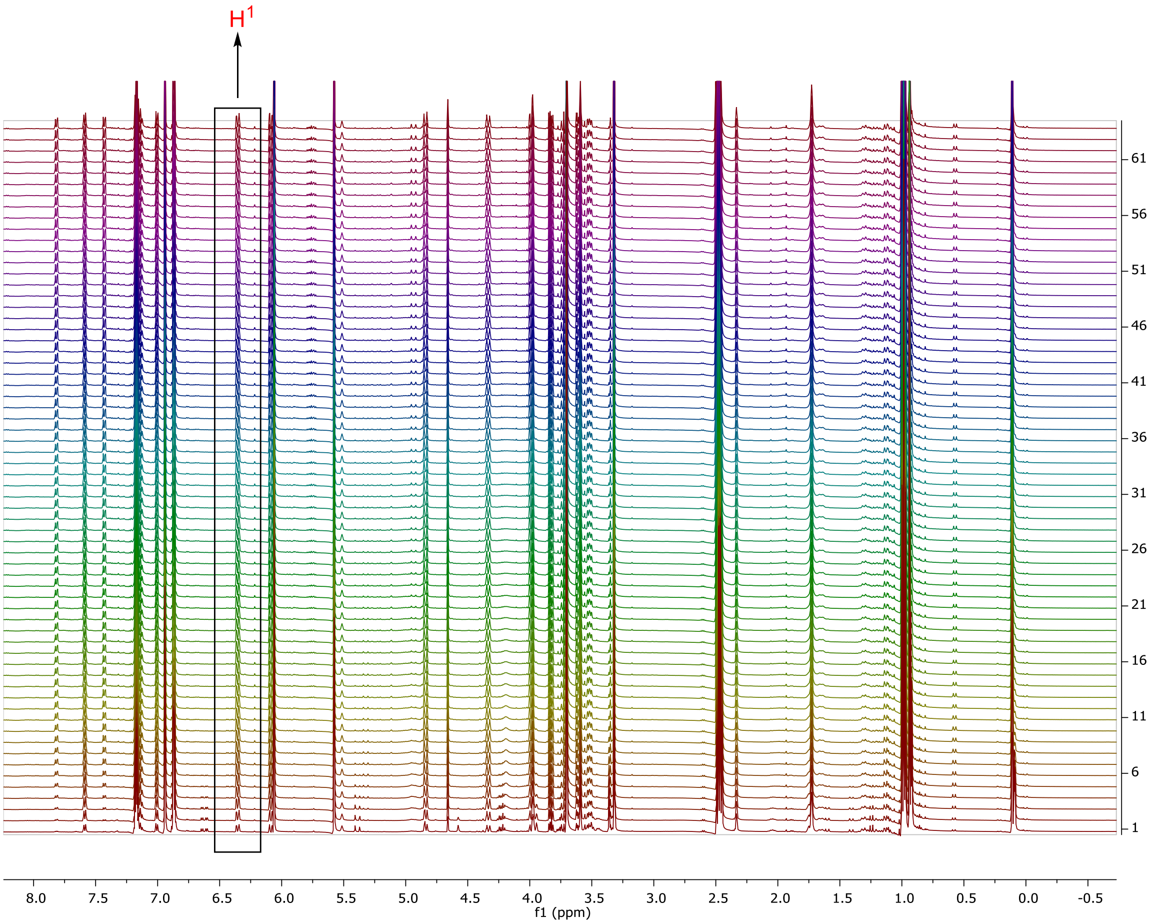

Supplementary Figure 21. Stacked ^{1}H spectra for the experiment of entry 11 in Supplementary Table 34

Supplementary Table 38. Concentration for **17a** calculated by ¹H NMR analysis for the experiments of entry 1, 9-11 in Supplementary Table 31

Entry	0.15 M-**16a**		0.2 M-**16a**		0.25 M-**16a**		0.3 M-**16a**	
	Time/min	[**17a**]	Time/min	[**17a**]	Time/min	[**17a**]	Time/min	[**17a**]
1	9	0.015192818	10	0.01741031	9	0.014683037	10	0.018809442
2	14	0.03835662	15	0.037187942	14	0.037369879	15	0.046511683
3	19	0.049084488	20	0.05576693	19	0.052024734	20	0.061869314
4	24	0.065745645	25	0.069101849	24	0.066454129	25	0.075062251
5	29	0.076623343	30	0.077432428	29	0.076402521	30	0.082638682
6	34	0.082796361	35	0.083155955	34	0.08398359	35	0.08594423
7	39	0.087680837	40	0.085553243	39	0.086576372	40	0.08816743
8	44	0.089418872	45	0.088130329	44	0.089760985	45	0.090332124
9	49	0.089988228	50	0.089478804	49	0.08970462	50	0.090683156
10	54	0.090317855	55	0.089568702	54	0.091423747	55	0.090917177
11	59	0.090557584	60	0.089688567	59	0.093762887	60	0.092379809
12	64	0.09058755	65	0.092025923	64	0.093706522	65	0.092379809
13	69	0.090947143	70	0.092385516	69	0.093762887	70	0.092379809
14	74	0.090827279	75	0.092145788	74	0.093621975		
15	79	0.090797313	80	0.092235686	79	0.093621975		

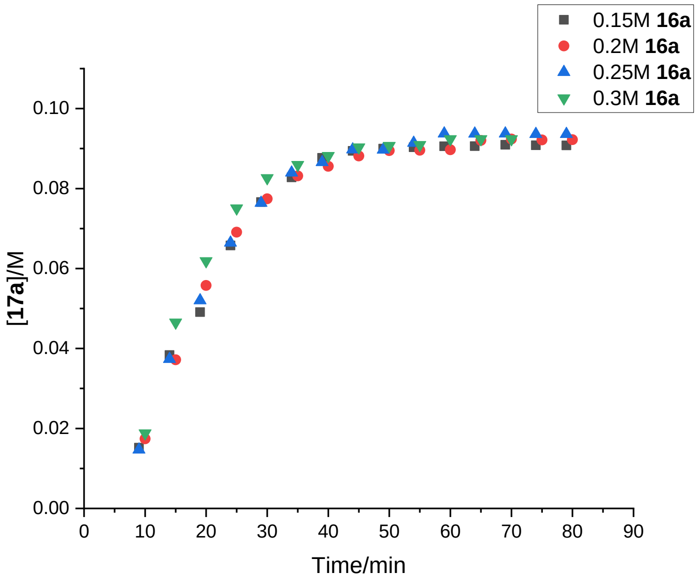

Supplementary Figure 22. Overlapped profile for the oxabicycle **16a** concentration dependence experiments

2.5 Base Et_3N concentration dependence

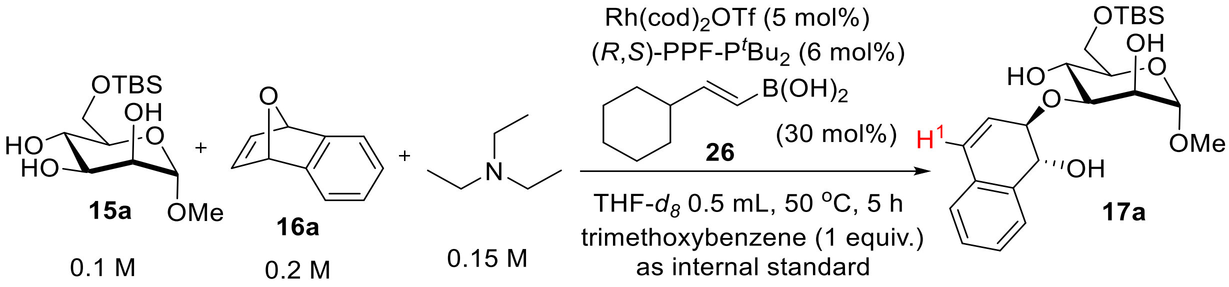

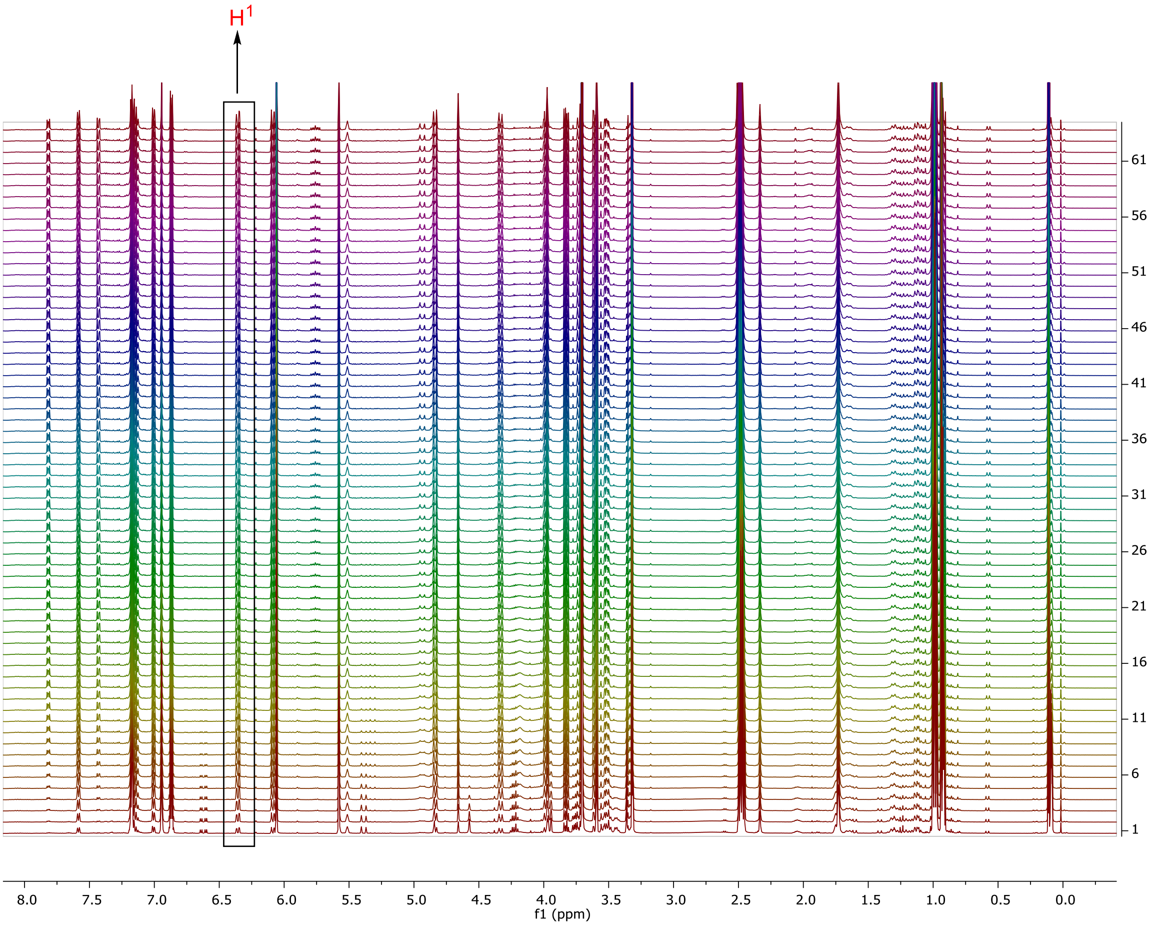

Supplementary Figure 23. Stacked ^{1}H spectra for the experiment of entry 12 in Supplementary Table 34

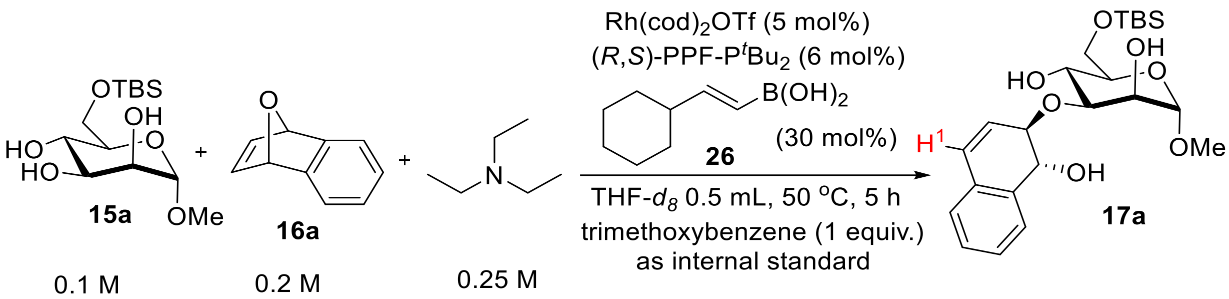

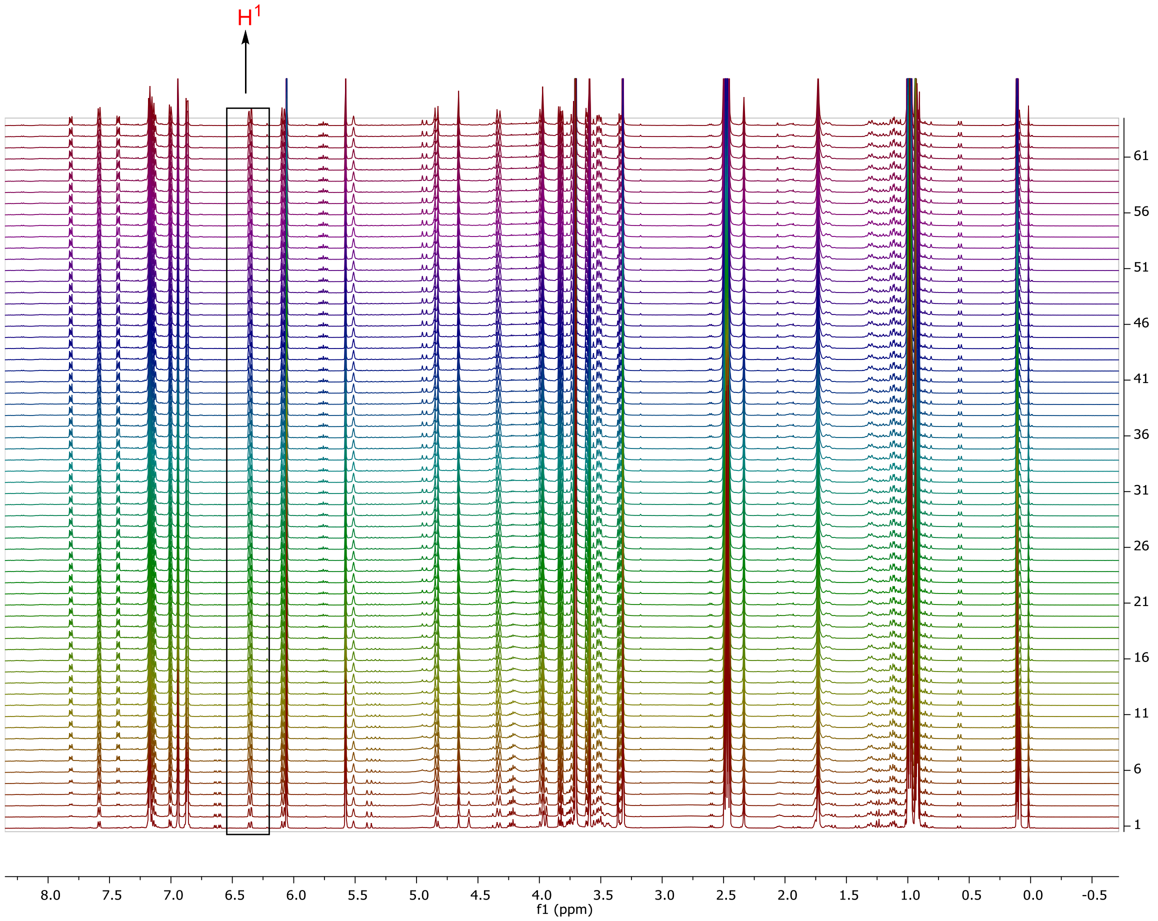

Supplementary Figure 24. Stacked ^{1}H spectra for the experiment of entry 13 in Supplementary Table 34

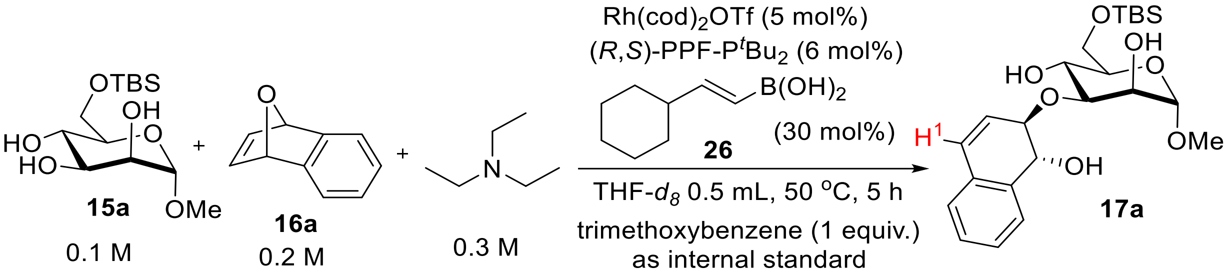

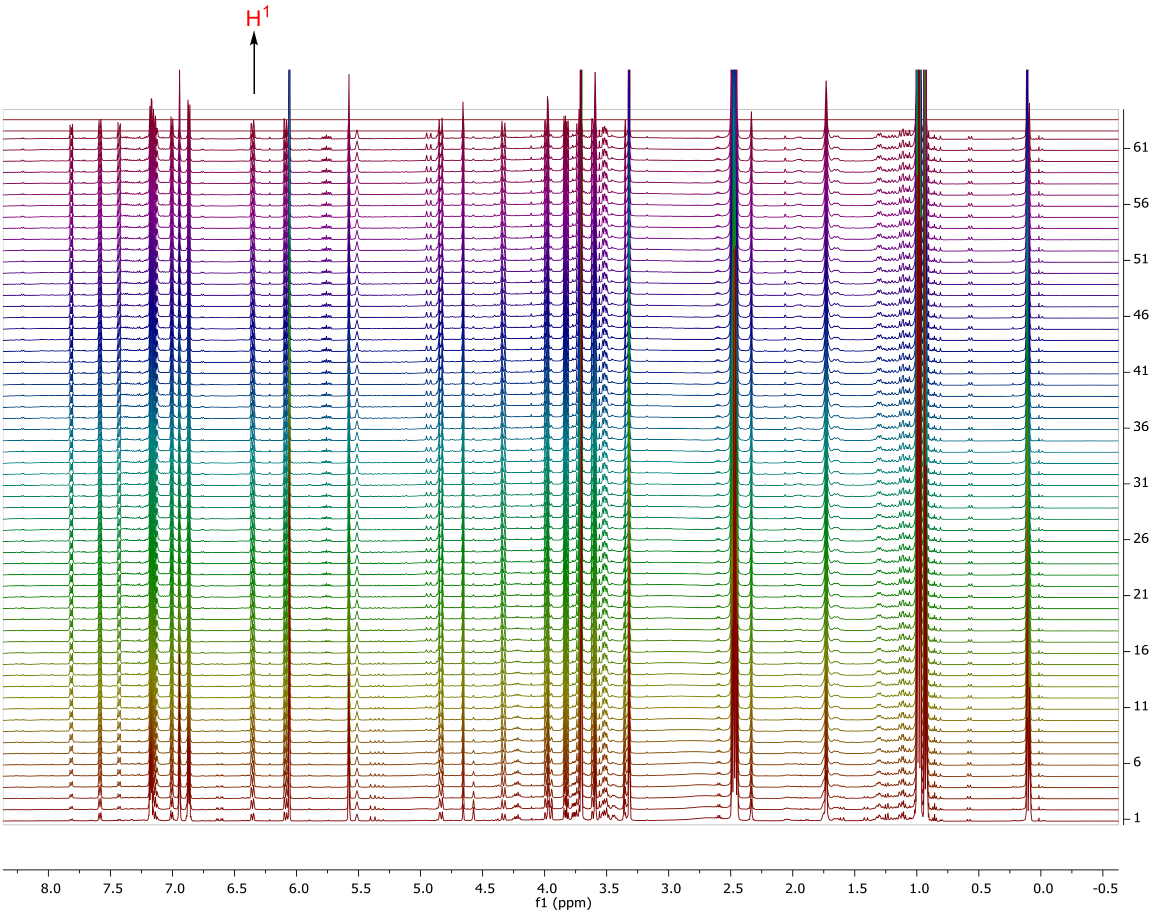

Supplementary Figure 25. Stacked ^{1}H spectra for the experiment of entry 14 in Supplementary Table 34

Supplementary Table 39. Concentration for **17a** calculated by ^{1}H NMR analysis for the experiments of entry 1, 12-14 in Supplementary Table 31

Entry	0.15 M-Et3N		0.2 M-Et3N		0.25 M-Et3N		0.3 M-Et3N	
	Time/min	[**17a**]	Time/min	[**17a**]	Time/min	[**17a**]	Time/min	[**17a**]
1	10	0.007341697	9	0.01216	10	0.012666389	9	0.015337654
2	15	0.027658719	14	0.03232	15	0.03624401	14	0.040683275
3	20	0.043810452	19	0.04548	20	0.049934241	19	0.052319757
4	25	0.056725846	24	0.05722	25	0.063507462	24	0.061942803
5	30	0.067393781	29	0.06683	30	0.073628872	29	0.067923896
6	35	0.075964088	34	0.07417	35	0.081029788	34	0.076747488
7	40	0.082167073	39	0.07735	40	0.084540103	39	0.078760925
8	45	0.087680837	44	0.08178	45	0.087085082	44	0.080093347
9	50	0.089538736	49	0.08378	50	0.090566145	49	0.083261549
10	55	0.090947143	54	0.08524	55	0.09094643	54	0.084268268
11	60	0.091007075	59	0.08609	60	0.091355966	59	0.08462358
12	65	0.092115821	64	0.08812	65	0.092847851	64	0.085393424
13	70	0.092115821	69	0.08954	70	0.093052619	69	0.086785065
14	75	0.092115821	74	0.09188	75	0.093052619	74	0.086814674
15			79	0.09188			79	0.086725846
16			84	0.09188				

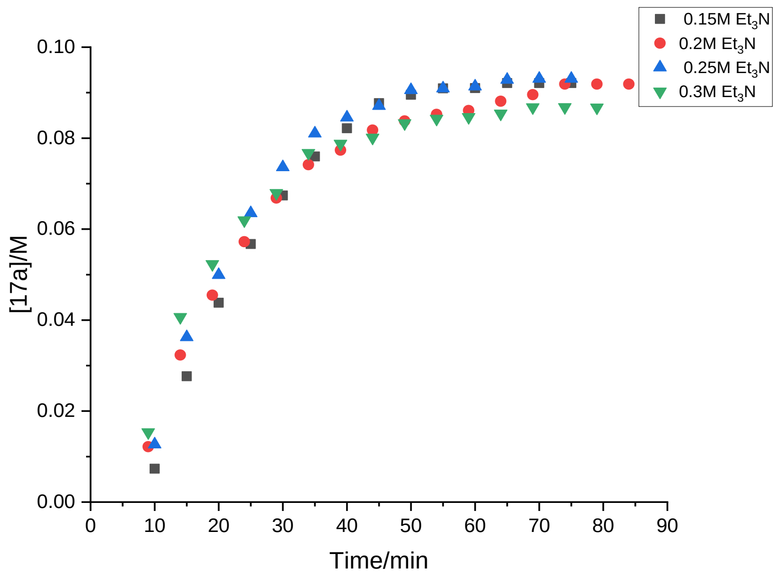

Supplementary Figure 26. Overlapped profile for the base Et_3N concentration dependence experiments

2.6 Kinetic Analysis[39-40]

2.6.1 Bures method to determine order with respect to $Rh(cod)_2OTf$

Supplementary Table 40. Concentration for carbohydrate polyol **15a** calculated by ^{1}H NMR analysis for the experiments of entry 1-3 in Supplementary Table 31

Entry	5 mol% $Rh(cod)_2OTf$		7 mol% $Rh(cod)_2OTf$		9 mol% $Rh(cod)_2OTf$	
	Time/min	[**15a**]	Time/min	[**15a**]	Time/min	[**15a**]
1	0	0.1	0	0.1	0	0.1
2	9	0.074806469	8	0.058875914	9	0.049803674
3	14	0.053944349	13	0.032929544	14	0.016391462
4	19	0.038964861	18	0.018776978	19	0.003056543
5	24	0.026562816	23	0.009528153	24	0.001168678
6	29	0.018375647	28	0.004283013	29	0.001168678
7	34	0.012462691	33	0.00338296		
8	39	0.009005886	38	0.002079434		
9	44	0.007216838	43	0.002017361		
10	49	0.00542779	48	0.001365598		
11	54	0.00479101	53	0.00117938		
12	59	0.004275522				
13	64	0.00394197				
14	69	0.003851002				
15	74	0.003669065				

Supplementary Table 41: Product formation over time multiplied by $[Rh(cod)_2OTf]$ to the 0 power.

Entry	5 mol% $Rh(cod)_2OTf$		7 mol% $Rh(cod)_2OTf$		9 mol% $Rh(cod)_2OTf$	
	Time/min	$t[Rh(cod)_2OTf]^0$	Time/min	$t[Rh(cod)_2OTf]^0$	Time/min	$t[Rh(cod)_2OTf]^0$
1	0	0	0	0	0	0
2	9	9	8	8	9	9
3	14	14	13	13	14	14
4	19	19	18	18	19	19
5	24	24	23	23	24	24
6	29	29	28	28	29	29
7	34	34	33	33	34	34
8	39	39	38	38	39	39
9	44	44	43	43	44	44
10	49	49	48	48	49	49
11	54	54	53	53	54	54
12	59	59	58	58	59	59
13	64	64	63	63	64	64
14	69	69	68	68	69	69
15	74	74	73	73	74	74

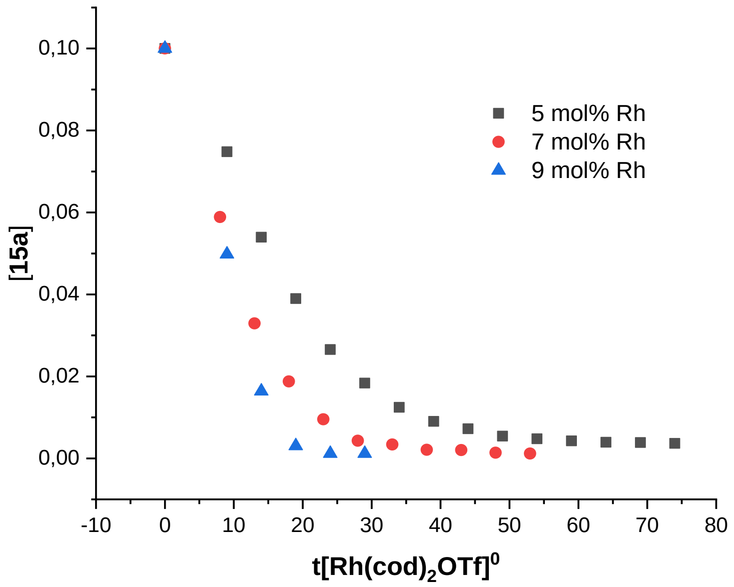

Supplementary Figure 27: Product formation over time multiplied by [Rh(cod)$_2$OTf] to the 0 power. The absence of overlay indicates the reaction is not zero order in catalyst.

Supplementary Table 42: Product formation over time multiplied by [Rh(cod)$_2$OTf] to the 0.5 power.

Entry	5 mol% catalyst		7 mol% catalyst		9 mol% catalyst	
	Time/min	t[Rh(cod)$_2$OTf]$^{0.5}$	Time/min	t[Rh(cod)$_2$OTf]$^{0.5}$	Time/min	t[Rh(cod)$_2$OTf]$^{0.5}$
1	0	0	0	0	0	0
2	9	0.636396103	8	0.669328021	9	0.853814968
3	14	0.989949494	13	1.087658034	14	1.328156617
4	19	1.343502884	18	1.505988048	19	1.802498266
5	24	1.697056275	23	1.924318061	24	2.276839915
6	29	2.050609665	28	2.342648074	29	2.751181564
7	34	2.404163056	33	2.760978088	34	3.225523213
8	39	2.757716447	38	3.179308101	39	3.699864862
9	44	3.111269837	43	3.597638114	44	4.174206511
10	49	3.464823228	48	4.015968127	49	4.64854816
11	54	3.818376618	53	4.434298141	54	5.122889809
12	59	4.171930009	58	4.852628154	59	5.597231458
13	64	4.5254834	63	5.270958167	64	6.071573108
14	69	4.87903679	68	5.68928818	69	6.545914757
15	74	5.232590181	73	6.107618194	74	7.020256406

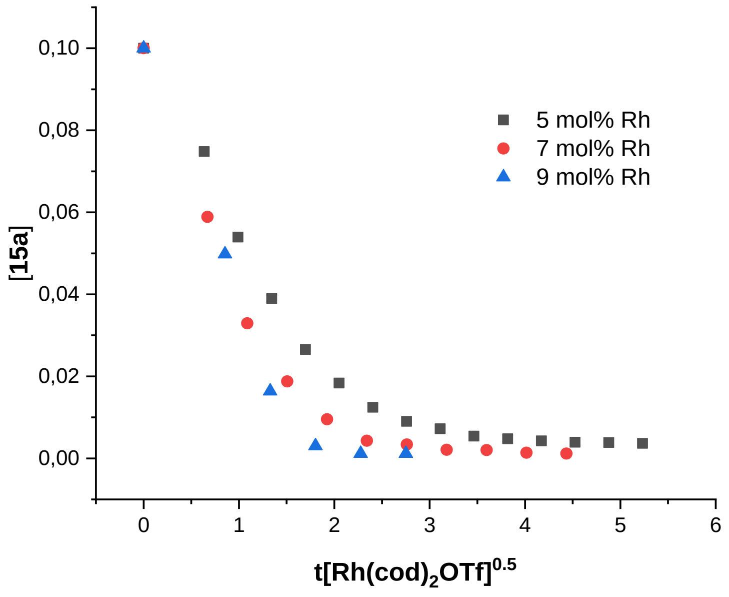

Supplementary Figure 28. Product formation over time multiplied by [Rh(cod)$_2$OTf] to the 0.5 power. The absence of overlay indicates the reaction is not 0.5 order in catalyst.

Supplementary Table 43. Product formation over time multiplied by [Rh(cod)$_2$OTf] to the first power.

Entry	5 mol% catalyst		7 mol% catalyst		9 mol% catalyst	
	Time/min	t[Rh(cod)$_2$OTf]^1	Time/min	t[Rh(cod)$_2$OTf]^1	Time/min	t[Rh(cod)$_2$OTf]^1
1	0	0	0	0	0	0
2	9	0.045	8	0.056	9	0.081
3	14	0.07	13	0.091	14	0.126
4	19	0.095	18	0.126	19	0.171
5	24	0.12	23	0.161	24	0.216
6	29	0.145	28	0.196	29	0.261
7	34	0.17	33	0.231	34	0.306
8	39	0.195	38	0.266	39	0.351
9	44	0.22	43	0.301	44	0.396
10	49	0.245	48	0.336	49	0.441
11	54	0.27	53	0.371	54	0.486
12	59	0.295	58	0.406	59	0.531
13	64	0.32	63	0.441	64	0.576
14	69	0.345	68	0.476	69	0.621
15	74	0.37	73	0.511	74	0.666

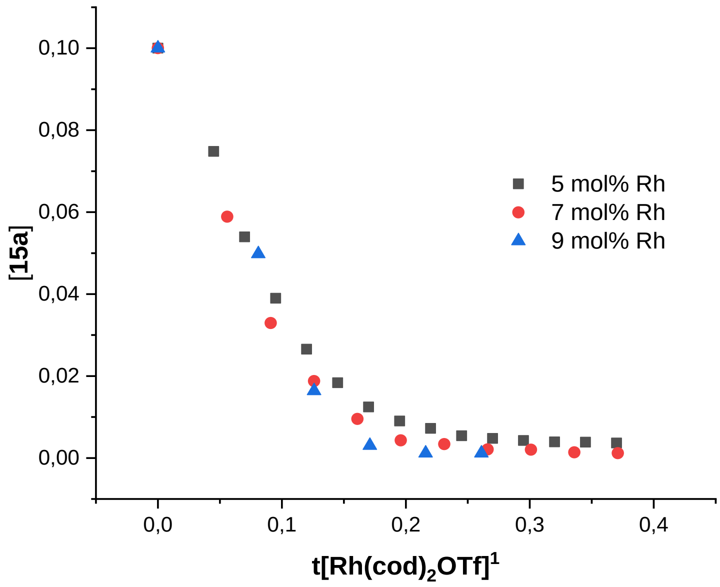

Supplementary Figure 29. Product formation over time multiplied by [Rh(cod)$_2$OTf] to the first power. Graphical overlay represents a first order dependence.

Supplementary Table 44. Product formation over time multiplied by [Rh(cod)$_2$OTf] to the second power

Entry	5 mol% Rh(cod)$_2$OTf		7 mol% Rh(cod)$_2$OTf		9 mol% Rh(cod)$_2$OTf	
	Time/min	t[cat.**3**]^2	Time/min	t[Rh(cod)$_2$OTf]^2	Time/min	t[Rh(cod)$_2$OTf]^2
1	0	0	0	0	0	0
2	9	0.000225	8	0.000392	9	0.000729
3	14	0.00035	13	0.000637	14	0.001134
4	19	0.000475	18	0.000882	19	0.001539
5	24	0.0006	23	0.001127	24	0.001944
6	29	0.000725	28	0.001372	29	0.002349
7	34	0.00085	33	0.001617	34	0.002754
8	39	0.000975	38	0.001862	39	0.003159
9	44	0.0011	43	0.002107	44	0.003564
10	49	0.001225	48	0.002352	49	0.003969
11	54	0.00135	53	0.002597	54	0.004374
12	59	0.001475	58	0.002842	59	0.004779
13	64	0.0016	63	0.003087	64	0.005184
14	69	0.001725	68	0.003332	69	0.005589
15	74	0.00185	73	0.003577	74	0.005994

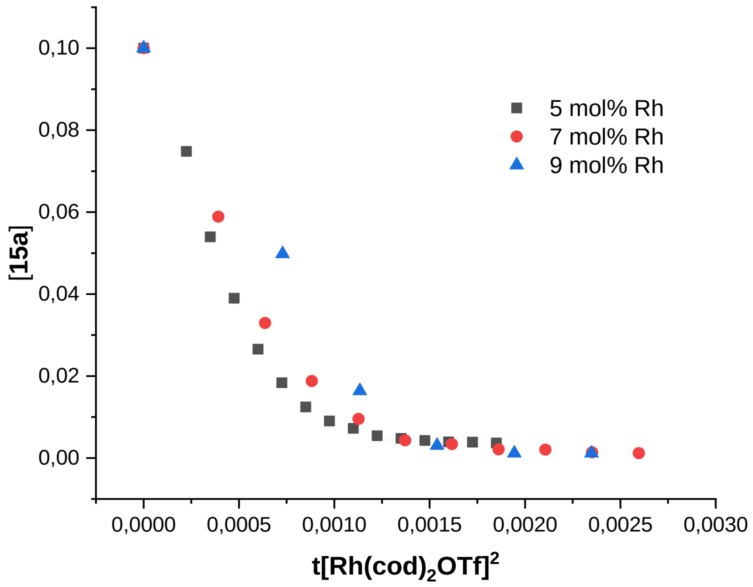

Supplementary Figure 30. Product formation over time multiplied by [Rh(cod)$_2$OTf] to the second power. The absence of overlay indicates the reaction is not second order in catalyst.

2.6.2 Bures method to determine order with respect to 26

Supplementary Table 45. Concentration for carbohydrate polyol **15a** calculated by ^{1}H NMR analysis for the experiments of entry 1, 4 & 5 in Supplementary Table 31

Entry	20 mol% catalyst **26**		30 mol% catalyst **26**		35 mol% catalyst **26**	
	Time/min	[**15a**]	Time/min	[**15a**]	Time/min	[**15a**]
1	0	0.1	0	0.1	0	0.1
2	9	0.079153338	9	0.074806469	10	0.058448897
3	14	0.065500922	14	0.053944349	15	0.036863666
4	19	0.050559962	19	0.038964861	20	0.023243356
5	24	0.036324633	24	0.026562816	25	0.016314763
6	29	0.030311433	29	0.018375647	30	0.00894203
7	34	0.024697069	34	0.012462691	35	0.00707664
8	39	0.019174743	39	0.009005886	40	0.005507343
9	44	0.015462513	44	0.007216838	45	0.003375468
10	49	0.012732029	49	0.00585231	50	0.002931328
11	54	0.011566205	54	0.00479101	55	0.002872109
12	59	0.010001546	59	0.004487782	60	0.002664843
13	64	0.009019799	64	0.004093585	65	0.002457578
14	69	0.008068732	69	0.004093585		
15	74	0.008007373				
16	79	0.007946013				
17	84	0.007853975				
18	89	0.007731256				

Supplementary Table 46. Product formation over time multiplied by [**26**] to the 0 power.

Entry	20 mol% catalyst **26**		30 mol% catalyst **26**		35 mol% catalyst **26**	
	Time/min	t[**26**]^0	Time/min	t[**26**]^0	Time/min	t[**26**]^0
1	0	0	0	0	0	0
2	9	9	9	9	10	10
3	14	14	14	14	15	15
4	19	19	19	19	20	20
5	24	24	24	24	25	25
6	29	29	29	29	30	30
7	34	34	34	34	35	35
8	39	39	39	39	40	40
9	44	44	44	44	45	45
10	49	49	49	49	50	50
11	54	54	54	54	55	55
12	59	59	59	59	60	60
13	64	64	64	64	65	65
14	69	69	69	69	70	70
15	74	74	74	74	75	75
16	79	79	79	79	80	80
17	84	84	84	84	85	85
18	89	89	89	89	90	90
19	94	94	94	94	95	95
20	99	99	99	99	100	100
21	104	104	104	104	105	105

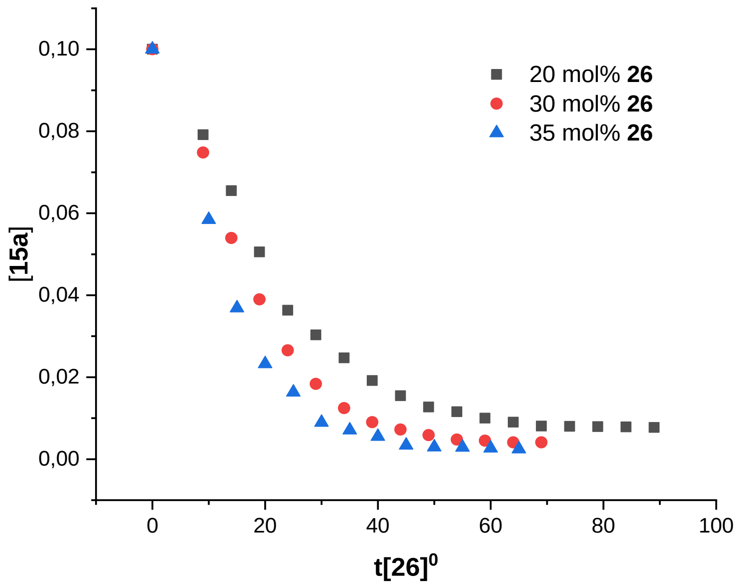

Supplementary Figure 31. Product formation over time multiplied by [**26**] to the zero power. The absence of overlay indicates the reaction is not zero order in catalyst.

Supplementary Table 47. Product formation over time multiplied by [boronic acid **26**] to the 0.5 power.

Entry	20 mol% catalyst **26**		30 mol% catalyst **26**		35 mol% catalyst **26**	
	Time/min	t[**26**]$^{0.5}$	Time/min	t[**26**]$^{0.5}$	Time/min	t[**26**]$^{0.5}$
1	0	0	0	0	0	0
2	9	1.272792206	9	1.558845727	10	1.870828693
3	14	1.979898987	14	2.424871131	15	2.80624304
4	19	2.687005769	19	3.290896534	20	3.741657387
5	24	3.39411255	24	4.156921938	25	4.677071733
6	29	4.101219331	29	5.022947342	30	5.61248608
7	34	4.808326112	34	5.888972746	35	6.547900427
8	39	5.515432893	39	6.75499815	40	7.483314774
9	44	6.222539674	44	7.621023553	45	8.41872912
10	49	6.929646456	49	8.487048957	50	9.354143467
11	54	7.636753237	54	9.353074361	55	10.28955781
12	59	8.343860018	59	10.21909976	60	11.22497216
13	64	9.050966799	64	11.08512517	65	12.16038651
14	69	9.75807358	69	11.95115057	70	13.09580085
15	74	10.46518036	74	12.81717598	75	14.0312152
16	79	11.17228714	79	13.68320138	80	14.96662955
17	84	11.87939392	84	14.54922678	85	15.90204389
18	89	12.58650071	89	15.41525219	90	16.83745824
19	94	13.29360749	94	16.28127759	95	17.77287259
20	99	14.00071427	99	17.14730299	100	18.70828693
21	104	14.70782105	104	18.0133284	105	19.64370128

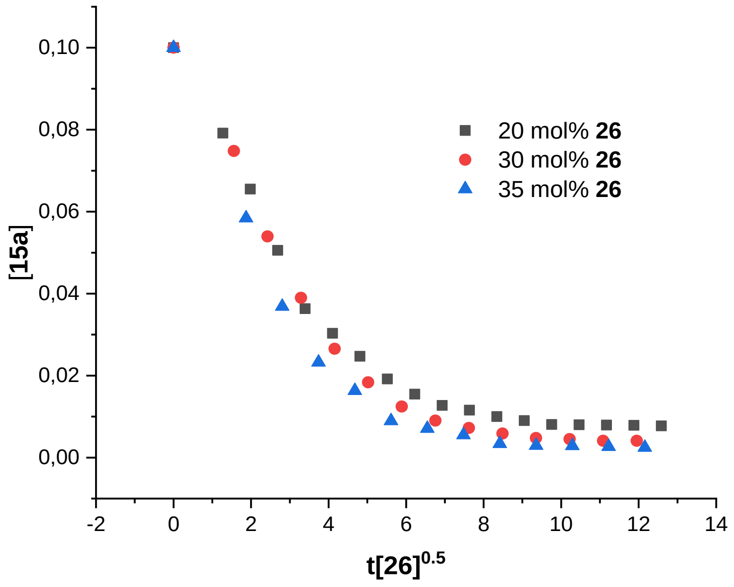

Supplementary Figure 32. Product formation over time multiplied by [**26**] to the 0.5 power. The absence of overlay indicates the reaction is not 0.5 order in catalyst.

Supplementary Table 48. Product formation over time multiplied by [**26**] to the first power.

Entry	20 mol% catalyst **26**		30 mol% catalyst **26**		35 mol% catalyst **26**	
	Time/min	t[**26**]^1	Time/min	t[**26**]^1	Time/min	t[**26**]^1
1	0	0	0	0	0	0
2	9	0.18	9	0.27	10	0.35
3	14	0.28	14	0.42	15	0.525
4	19	0.38	19	0.57	20	0.7
5	24	0.48	24	0.72	25	0.875
6	29	0.58	29	0.87	30	1.05
7	34	0.68	34	1.02	35	1.225
8	39	0.78	39	1.17	40	1.4
9	44	0.88	44	1.32	45	1.575
10	49	0.98	49	1.47	50	1.75
11	54	1.08	54	1.62	55	1.925
12	59	1.18	59	1.77	60	2.1
13	64	1.28	64	1.92	65	2.275
14	69	1.38	69	2.07	70	2.45
15	74	1.48	74	2.22	75	2.625
16	79	1.58	79	2.37	80	2.8
17	84	1.68	84	2.52	85	2.975
18	89	1.78	89	2.67	90	3.15
19	94	1.88	94	2.82	95	3.325
20	99	1.98	99	2.97	100	3.5
21	104	2.08	104	3.12	105	3.675

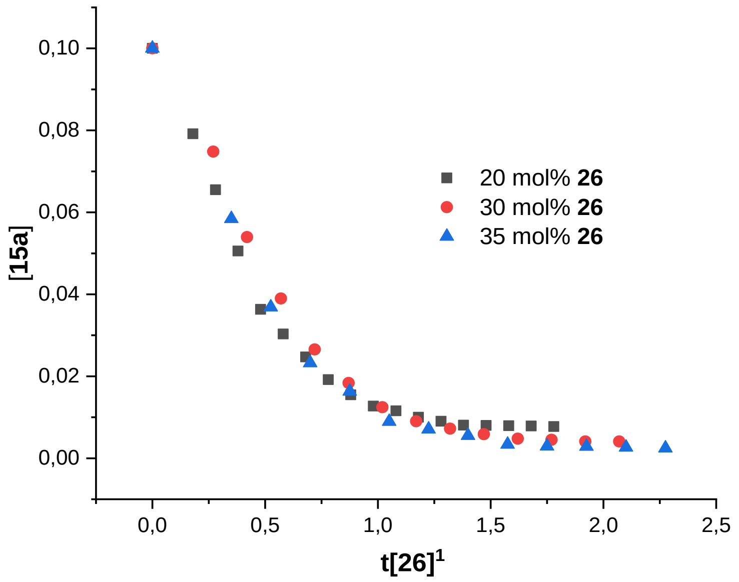

Supplementary Figure 33. Product formation over time multiplied by [**26**] to the first power. Graphical overlay represents a first order dependence.

Supplementary Table 49. Product formation over time multiplied by [**26**] to the second power.

Entry	20 mol% catalyst **26**		30 mol% catalyst **26**		35 mol% catalyst **26**	
	Time/min	t[**26**]^2	Time/min	t[**26**]^2	Time/min	t[**26**]^2
1	0	0	0	0	0	0
2	9	0.0036	9	0.0081	10	0.01225
3	14	0.0056	14	0.0126	15	0.018375
4	19	0.0076	19	0.0171	20	0.0245
5	24	0.0096	24	0.0216	25	0.030625
6	29	0.0116	29	0.0261	30	0.03675
7	34	0.0136	34	0.0306	35	0.042875
8	39	0.0156	39	0.0351	40	0.049
9	44	0.0176	44	0.0396	45	0.055125
10	49	0.0196	49	0.0441	50	0.06125
11	54	0.0216	54	0.0486	55	0.067375
12	59	0.0236	59	0.0531	60	0.0735
13	64	0.0256	64	0.0576	65	0.079625
14	69	0.0276	69	0.0621	70	0.08575
15	74	0.0296	74	0.0666	75	0.091875
16	79	0.0316	79	0.0711	80	0.098
17	84	0.0336	84	0.0756	85	0.104125
18	89	0.0356	89	0.0801	90	0.11025
19	94	0.0376	94	0.0846	95	0.116375
20	99	0.0396	99	0.0891	100	0.1225
21	104	0.0416	104	0.0936	105	0.128625

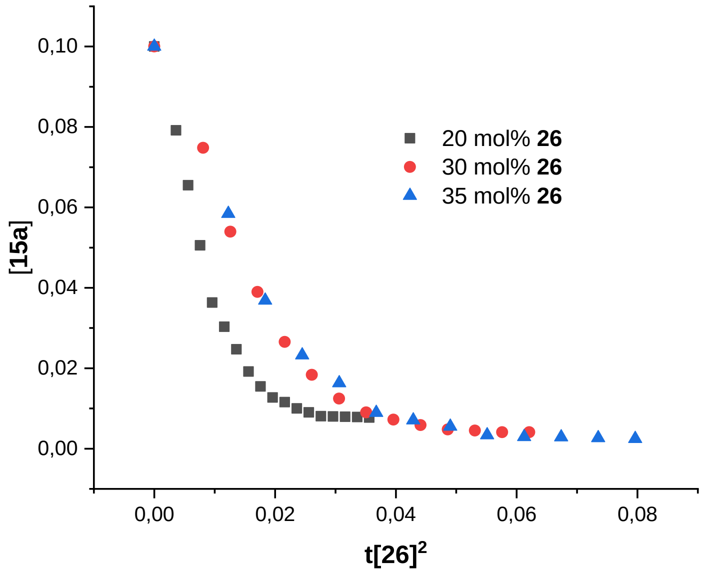

Supplementary Figure 34. Product formation over time multiplied by [**26**] to the second power. The absence of overlay indicates the reaction is not second order in catalyst.

Computational Details

All quantum chemical calculations were performed with the developmental version of the Orca 5.0.2 and 5.0.3 program package.[41-43] All molecular geometries were optimized (utilizing TIGHTSCF settings) using the robust meta GGA composite method r^{2}SCAN-3c developed by Grimme et al. consisting of a triple zeta basis set def2-mTZVPP,[44-45] corrected using the D4 dispersion model[46] and the geometric counterpoise correction scheme (gCP) for inter- and intra-molecular basis set superposition error (BSSE).[47] The default Gaussian charge scheme with a scaled vdW-type cavity of the conductor-like polarizable continuum (CPCM) solvation model for THF as implemented in Orca was applied.[48-49] The resolution-of-identity (RI) approximation[50-52] for Coulomb integrals was applied along with the appropriate corresponding auxiliary basis sets. Harmonic frequencies calculations at 298.15 K were computed at the same level of theory and used to verify the nature of stationary points as minima (no imaginary frequencies) or transition states (only 1 imaginary frequency) and to provide thermostatical corrections based on the modified ideal gas-rigid rotor-harmonic oscillator (RRHO) model.[53]

For Calculation of Nucleophilicity Indices and NBO Analysis

Natural population analysis,[54] Natural Bond Orbital (NBO) Analysis[55] and second order perturbation theory analysis in Natural Bond Orbital (NBO) basis were performed using the NBO 7.0.10 program package by Weinholdt and co-workers.[56] Nucleophilicity indices[57] based on the HOMO energy were calculated using Multiwfn version 3.8.[58] Population analyses and nucleophilicity indices were all calculated at the r^{2}SCAN-3c/CPCM(THF) level of theory. NBOs were rendered using VMD 1.9.4a51.

The nucleophilicity indices of the corresponding resting state boronate derivatives arising from different boronic acid catalysts were calculated using the following formula:

$$N_{\mathrm{Nu}} = E_{\mathrm{HOMO}}(\mathrm{Nu}) - E_{\mathrm{HOMO}}(\mathrm{TCE})$$

Nu denotes nucleophile, HOMO denotes highest occupied molecular orbital and TCE denotes tetracyanoethylene, which is the reference HOMO energy system (-8.43845 eV at r^{2}SCAN-3c/CPCM(THF) level of theory). The resting state boronates will be denoted with the "**rs**" suffix. Note that only the optimal boronic acid **26** is the exception, and the boronate resting state is denoted as **33**$_{\mathbf{trun}}$. For accuracy in definition, the subscript "trun" is used to distinguish the actual resting state, and the truncated modelled version without spectator countercation. Thus, for example, the computed model **33**$_{\mathbf{trun}}$ differs from resting state **33** through the absence of the ammonium countercation.

Structures of computed resting state intermediates (spectator ammonium cation omitted in modeling)

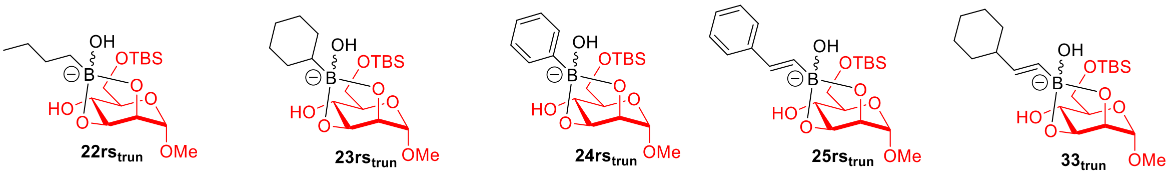

Substituent	Resting state	Derived from	E_{HOMO}(Nu) /eV	N_{Nu} /eV
n-butyl	**22rs$_{trun}$**	**22**	– 4.6511	3.787352
Cyclohexyl	**23rs$_{trun}$**	**23**	– 4.5869	3.851552
Cyclohexyl vinyl	**33$_{trun}$**	**26**	– 4.7042	3.734252
Phenyl vinyl	**25rs$_{trun}$**	**25**	– 4.7239	3.714552
Phenyl boronate	**24rs$_{trun}$**	**24**	– 4.7741	3.664352

Supplementary Table 50. Calculated E_{HOMO} and nucleophilicity indices.

The calculations showed that the presence of aliphatic groups in **22rs**$_{\mathbf{trun}}$ and **23rs**$_{\mathbf{trun}}$ generally resulted in HOMO elevation and correspondingly N_{Nu} elevation, suggesting higher nucleophilicity with electron donating boronate substituents. Substituents that promote conjugation and resonance effects such as alkenes or aromatic groups appeared to diminish the nucleophilicity indices.

Among the more stabilized boronates category (green shaded), we noticed that the presence of the aliphatic cyclohexyl scaffold adjacent to the vinyl group in **33_{trun}** has a net HOMO elevation compared to the absence of aliphatic groups in **$25rs_{trun}$, $24rs_{trun}$**, which also correspond to a higher nucleophilicity index of **33_{trun}**. We further noted that the increase in nucleophilicity index in this stabilized substituent category is consistent with the increase in yields in our boronic acid screening.

The above mentioned nucleophilicity indices provide a theoretical basis to the nucleophilicity of these boronates, however, this factor has to be balanced by the thermodynamic stabilization of these boronates by the substituent. The negatively charged boronate will be further destabilized by electron donating moieties and vice versa, stabilized by the presence of conjugation.

This is further computationally studied using NBO analysis and second order perturbation theory analysis in NBO basis.

By Natural Population Analysis, we observed that the negative charge is largely concentrated on the nucleophilic boronate oxygen (O7), rather than the boron (B41).

Supplementary Table 51. Summary of Natural Population Analysis of **33_{trun}** (spectator countercation excluded):

Atom No	Natural Charge	Natural Population Core	Valence	Rydberg	Total
O 1	-0.53967	1.99999	6.51829	0.02138	8.53967
C 2	0.29975	1.99999	3.67755	0.02271	5.70025
C 3	0.00163	1.99999	3.98132	0.01706	5.99837
C 4	-0.00216	1.99999	3.98537	0.01680	6.00216
C 5	-0.01872	1.99999	4.00048	0.01825	6.01872
C 6	0.01575	1.99999	3.96495	0.01931	5.98425
O 7	-0.73809	1.99999	6.71664	0.02145	8.73809
H 8	0.20993	0.00000	0.78807	0.00200	0.79007
C 9	-0.16348	1.99999	4.14654	0.01695	6.16348
H 10	0.24517	0.00000	0.75283	0.00200	0.75483
O 11	-0.89427	2.00000	6.86914	0.02513	8.89427
H 12	0.21306	0.00000	0.78492	0.00201	0.78694
H 13	0.23717	0.00000	0.76105	0.00178	0.76283
Si 14	1.98399	9.99998	1.98049	0.03553	12.01601
C 15	-0.48514	1.99999	4.46342	0.02173	6.48514
C 16	-0.72761	2.00000	4.71895	0.00867	6.72761
C 17	-0.72625	1.99999	4.71745	0.00880	6.72625
H 18	0.24895	0.00000	0.74968	0.00137	0.75105
H 19	0.23917	0.00000	0.75903	0.00180	0.76083
H 20	0.24434	0.00000	0.75433	0.00132	0.75566
H 21	0.24571	0.00000	0.75297	0.00132	0.75429
H 22	0.24534	0.00000	0.75334	0.00131	0.75466
H 23	0.24103	0.00000	0.75717	0.00180	0.75897
C 24	-0.72691	1.99999	4.71815	0.00877	6.72691
H 25	0.24460	0.00000	0.75408	0.00132	0.75540
H 26	0.23865	0.00000	0.75951	0.00184	0.76135
H 27	0.25032	0.00000	0.74833	0.00136	0.74968
C 28	-1.28455	1.99999	5.27267	0.01189	7.28455
H 29	0.27499	0.00000	0.72407	0.00094	0.72501
H 30	0.27901	0.00000	0.72016	0.00083	0.72099
H 31	0.28077	0.00000	0.71809	0.00114	0.71923
C 32	-1.27941	1.99999	5.26720	0.01221	7.27941
H 33	0.27333	0.00000	0.72574	0.00093	0.72667

H 34	0.28234	0.00000	0.71662	0.00104	0.71766
H 35	0.27192	0.00000	0.72725	0.00083	0.72808
O 36	-0.76658	1.99999	6.74771	0.01888	8.76658
H 37	0.20924	0.00000	0.78852	0.00224	0.79076
H 38	0.52182	0.00000	0.47511	0.00307	0.47818
O 39	-0.72403	1.99999	6.70239	0.02165	8.72403
H 40	0.20660	0.00000	0.79111	0.00229	0.79340
B 41	0.94671	1.99999	2.01807	0.03523	4.05329
C 42	-0.50921	1.99999	4.49189	0.01733	6.50921
C 43	-0.22894	1.99999	4.21337	0.01558	6.22894
C 44	-0.30167	1.99999	4.28702	0.01466	6.30167
C 45	-0.47184	2.00000	4.46290	0.00895	6.47184
H 46	0.20922	0.00000	0.78893	0.00185	0.79078
H 47	0.21464	0.00000	0.78334	0.00202	0.78536
C 48	-0.48894	1.99999	4.48064	0.00831	6.48894
H 49	0.24452	0.00000	0.75394	0.00153	0.75548
H 50	0.24105	0.00000	0.75727	0.00168	0.75895
C 51	-0.48591	1.99999	4.47785	0.00807	6.48591
H 52	0.24980	0.00000	0.74873	0.00147	0.75020
H 53	0.23552	0.00000	0.76280	0.00168	0.76448
C 54	-0.48412	2.00000	4.47582	0.00830	6.48412
H 55	0.23634	0.00000	0.76200	0.00166	0.76366
H 56	0.24842	0.00000	0.75013	0.00145	0.75158
C 57	-0.47737	1.99999	4.46867	0.00870	6.47737
H 58	0.24321	0.00000	0.75520	0.00159	0.75679
H 59	0.24225	0.00000	0.75616	0.00159	0.75775
H 60	0.25145	0.00000	0.74708	0.00147	0.74855
H 61	0.23415	0.00000	0.76417	0.00168	0.76585
H 62	0.23535	0.00000	0.76257	0.00208	0.76465
O 63	-0.98741	1.99999	6.96746	0.01996	8.98741
H 64	0.49994	0.00000	0.49666	0.00340	0.50006
O 65	-0.51400	1.99999	6.49197	0.02203	8.51400
H 66	0.21162	0.00000	0.78602	0.00235	0.78838
C 67	-0.40486	1.99999	4.39247	0.01240	6.40486
H 68	0.23985	0.00000	0.75881	0.00134	0.76015
H 69	0.22423	0.00000	0.77403	0.00174	0.77577
H 70	0.21828	0.00000	0.77995	0.00177	0.78172
* Total *	-1.00000	67.99980	173.42663	0.57356	242.00000

Further, by second order perturbation analysis in NBO basis, we calculated the $\Delta E_{ij}^{(2)}$ stabilization due to the delocalization of the lone pair of electron from the nucleophilic boronate oxygen (donor) into the σ* antiorbital (acceptor) of the B-**R** bond, where **R** is the substituent and B is boron.[28] The $\Delta E_{ij}^{(2)}$ stabilization value is indicative of the energy lowering stabilizing effect due to charge transfer of this NBO donor-acceptor interaction, and provides a theoretical indicator of thermodynamic stability enhancement due to electronic changes in the boronate substituents from aliphatic to alkene/aromatic based ones.

A VMD rendering of this NBO donor-acceptor interaction in resting state $\mathbf{33}_{\mathbf{trun}}$ is provided below:

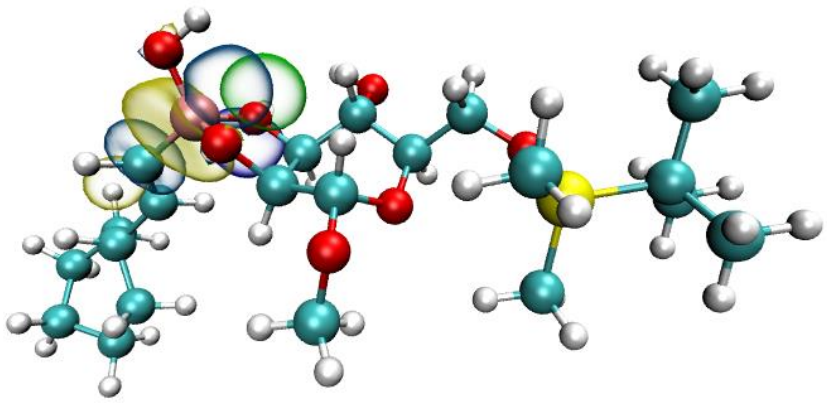

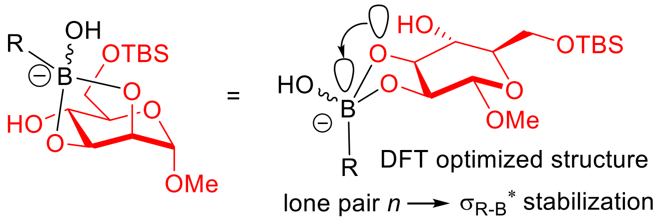

Key: Red atoms=Oxygen, Cyan atoms=Carbon, Pink atom= Boron, Yellow atom=Silicon, White atom: hydrogens.

**Substituent**	Resting state	$\Delta E_{ij}^{(2)}$ stabilization/kcalmol^{-1}
n-butyl	**22rs$_{trun}$**	4.33
Cyclohexyl	**23rs$_{trun}$**	4.27
Cyclohexyl vinyl	**33$_{trun}$**	6.1
Phenyl vinyl	**25rs$_{trun}$**	6.15
Phenyl boronate	**24rs$_{trun}$**	6.39

Supplementary Table 52. $\Delta E_{ij}^{(2)}$ stabilization of boronate resting states at r^{2}SCAN-3c/CPCM(THF) level of theory.

The above table shows a trend that the conjugated boronate resting states **24rs$_{trun}$, 25rs$_{trun}$ and 33$_{trun}$** were approximately 2 kcalmol^{-1} more stable in the $\Delta E_{ij}^{(2)}$ than the purely aliphatic boronates **22rs$_{trun}$** and **23rs$_{trun}$**. These suggest that the negatively charged aliphatic boronates (**22rs$_{trun}$** and **23rs$_{trun}$)** were less

stabilized in solution than those bearing conjugated/resonance stabilized moieties (**24rs$_{trun}$, 25rs$_{trun}$ and 33$_{trun}$**), despite the higher nucleophilicity of the former.

Hence, ***the above mentioned dual electronic effects arising from nucleophilicity elevation (through HOMO elevation) and oxygen lone pair stabilization by the R-B antiorbital offered the following plausible rationale for the observed reactivity trend of the various boronic acids***:

a) Aliphatic boronate intermediates **22rs$_{trun}$** and **23rs$_{trun}$** were likely to be highly destabilized due to the electron donating character of the **R** substituents, hence the lower thermodynamic stability overrides any potential nucleophilicity increase of the boronates.

b) For the resonance stabilized boronates **24rs$_{trun}$, 25rs$_{trun}$ and 33$_{trun}$**, the superior performance arising from the cyclohexyl vinyl scaffold could be rationalized by the concomitant presence of an electron donating cyclohexyl moiety which enhances nucleophilicity, delicately balanced by the vinyl group which provides anionic stabilization. Whereas in **24rs$_{trun}$, 25rs$_{trun}$**, although these moieties provide good stabilization of the boronate intermediates, the aryl substituent diminished the nucleophilicity of the boronates, hence resulting in lower yields compared to **33$_{trun}$** (employment of boronic acid **26**).

CYLView rendering of optimized boronate resting states

The cartesian coordinates (in Å) of all resting state boronate structures optimized at the r^{2}SCAN-3c/CPCM(THF) level of theory are provided alongside this manuscript within the “Cartesian Coordinates of Resting State Boronates” folder as part of a supplementary zip file. The .xyz file names of each individual structure are provided below in parenthesis.

Butyl boronate resting state 22rs$_{trun}$ (22rstrun_Butyl_Boronate.xyz)

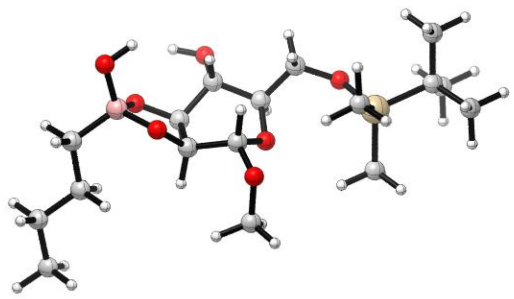

Cyclohexyl boronate resting state 23rs$_{trun}$ (23rstrun_Cyclohexyl_Boronate.xyz)

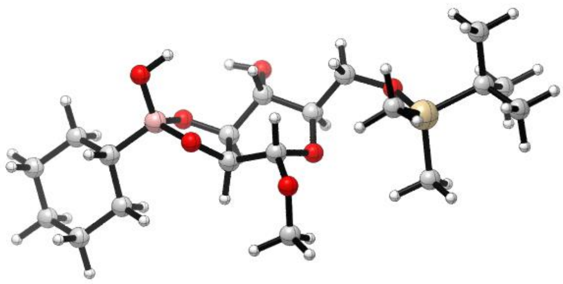

Cyclohexyl boronate resting state 33_{trun} (33trun_Cyclohexyl_Vinyl_Boronate.xyz)

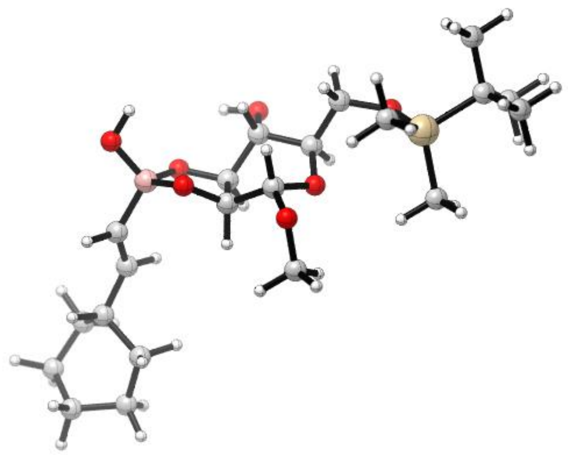

Phenyl vinyl boronate resting state $25rs_{trun}$ (25rstrun_Phenyl_Vinyl_Boronate.xyz)

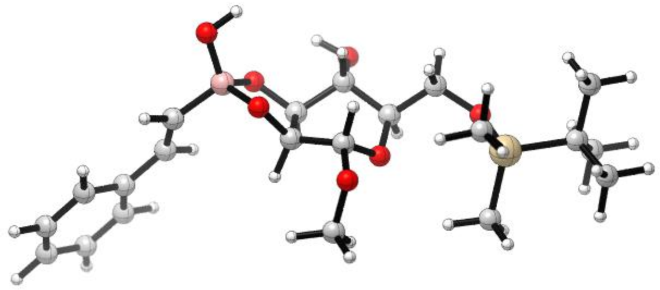

Phenyl boronate resting state $24rs_{trun}$ (24rstrun_Phenyl_Boronate.xyz)

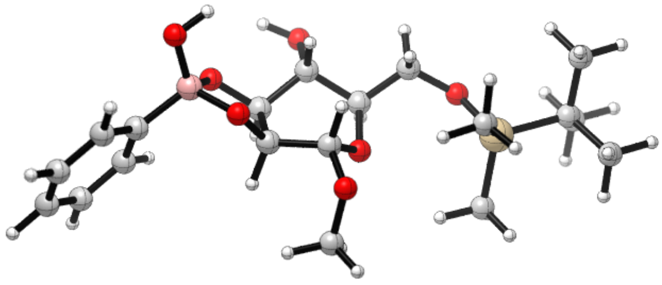

DFT modelling of the rate limiting elementary step

Geometry optimization was conducted in the at the r^{2}SCAN-3c/CPCM(THF) level of theory[44-45] employing tight integration grids (DEFGRID3 in Orca) and the default def2-ECP settings in Orca for Rh[59-60] and taking into account solvation using the conductor-like polarizable continuum (CPCM) solvation model for THF as implemented in Orca. Spectator counterions were excluded in the reaction path modelling. Reaction path optimization and transition state searches were preliminarily performed using the growing string method (GSM) implementation by Zimmermann et al.[61-62] at the GFN2-xTB/ALPB(THF) level of theory.[63-65] A higher level optimization constraining the relevant atoms involved in the reaction coordinate at the r^{2}SCAN-3c level of theory was performed to expedite the locating of the first order saddle point on the potential energy hypersurface. A subsequent transition state optimization and frequency calculation was performed to verify the single imaginary frequency mode. An intrinsic reaction coordinate (IRC) was finally performed using the EulerPC integrator of the open-source pysisyphus code[66-67] to confirm that the located transition state connects the resting state intermediate and the product of the rate limiting elementary step in mass-weighted cartesian coordinates. Harmonic frequencies calculations at 298.15 K were computed at the same level of theory and used to verify the nature of stationary points as minima (no imaginary frequencies) or transition states (only 1 imaginary frequency) and to provide thermostatical corrections based on the modified ideal gas-rigid rotor-harmonic oscillator (RRHO) model.[53]

Single point gas phase energies were calculated at the ωB97M-V/def2-QZVPP level of theory[68-71] employing the resolution-of-identity (RIJCOSX) approximation with tight integration grids (DEFGRID3), the VV10 nonlocal dispersion correction,[72] and taking into account of solvation using the conductor-like polarizable continuum (CPCM) solvation model for THF as implemented in Orca. The ionic species are assumed to remain in their solvated states for the multilevel calculation. Optimized intermediates and transition state geometries were rendered using the CYLView software.[73] The multilevel free energy profile is denoted as ωB97M-V/def2-QZVPP/CPCM(THF)//r^{2}SCAN-3c/CPCM(THF) in the manuscript.

DFT energetic profile calculated at the ωB97M-V/def2-QZVPP/CPCM(THF)//r^{2}SCAN-3c/CPCM(THF) level of theory

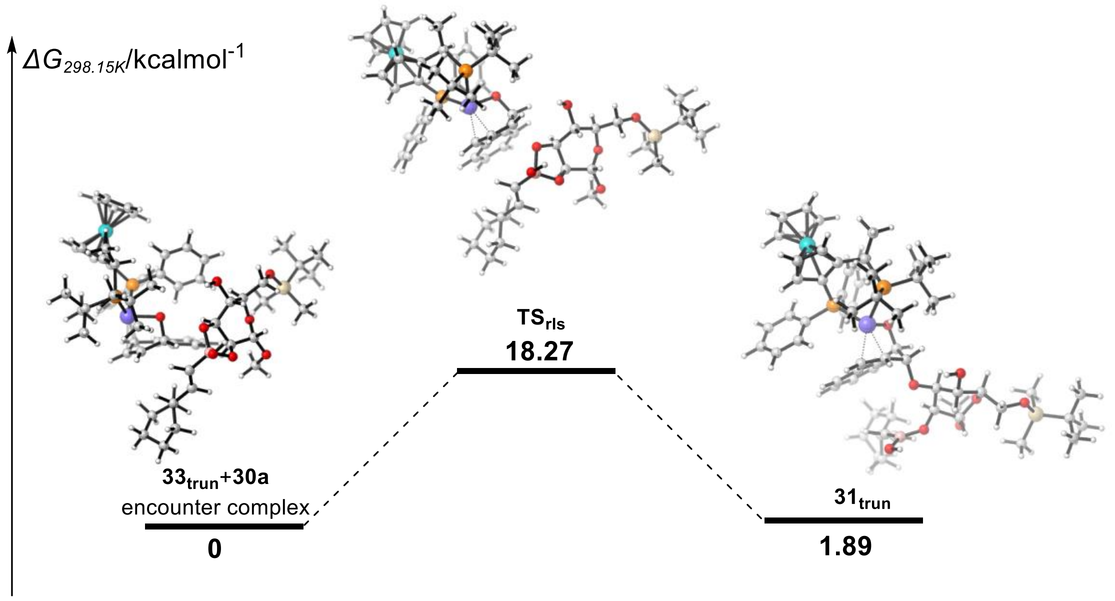

Supplementary Figure 35. Gibb’s free energy level diagram of the rate limiting elementary step computed at 298.15K.

Based on our DFT calculations, we computed the energetic profile of the rate limiting elementary step, which gave a 18.27 kcal/mol free energy barrier between the encounter complex and the transition state (TS_{rls}). IRC calculations also confirmed that TS_{rls} connects the encounter complex **33_{trun}+30a** and the

product complex **31_{trun}**. This barrier height is also indicative of the kinetic feasibility of the rate limiting elementary step. (The "trun" subscript denotes the exclusion of spectator counterions in the calculation)

CYLView rendering of stationary points involved in the rate limiting step

The cartesian coordinates (in Å) of all stationary points on the reaction path of the rate limiting step optimized at the r^{2}SCAN-3c/CPCM(THF) level of theory are provided alongside this manuscript within the "Cartesian Coordinates of Reaction Path" folder as part of a supplementary zip file. The .xyz file names of each individual structure are provided below in parenthesis.

Encounter Complex 33_{trun} + 30a (Encounter Complex 33trun + 30a.xyz)

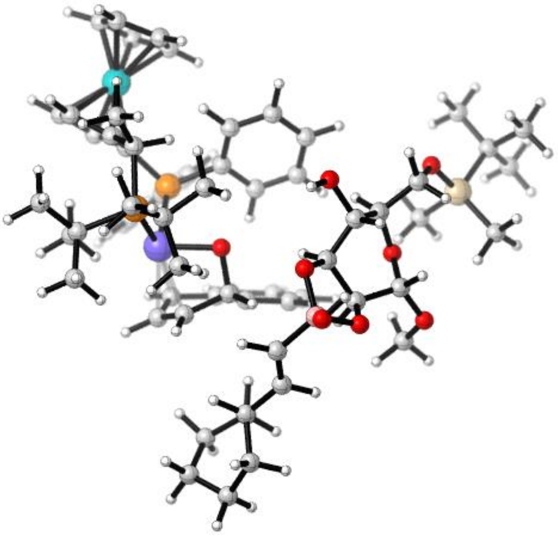

Transition state (TS_{rls}) linking encounter complex and 31_{trun} (Transition State.xyz)

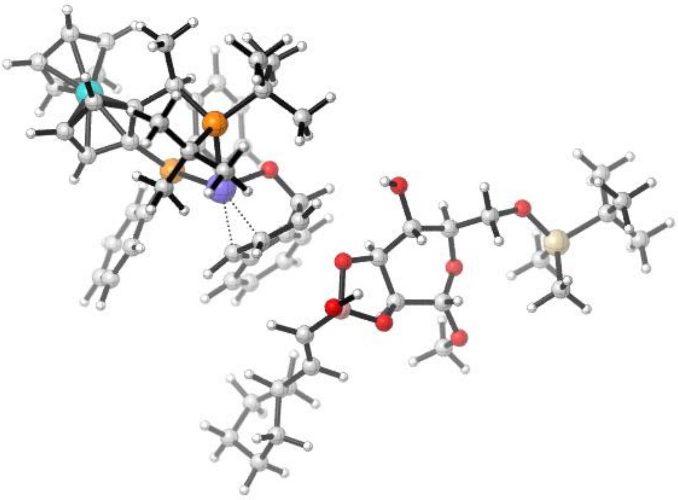

Imaginary frequency = – 244.10 cm^{-1}

Addition product 31_{trun} (31trun.xyz)

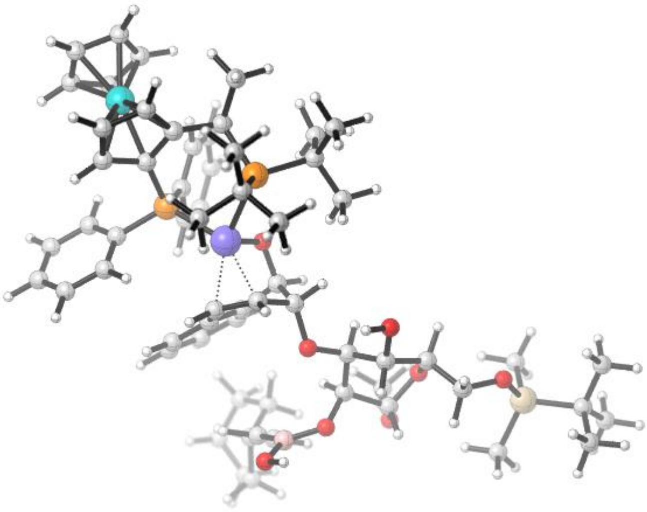

References:

1) Gandeepan, P.; Rajamalli, P.; Cheng, C.-H. Diastereoselective [3+2] Annulation of Aromatic/Vinylic Amides with Bicyclic Alkenes through Cobalt-Catalyzed C-H Activation and Intramolecular Nucleophilic Addition. *Angew. Chem. Int. Ed*. **2016**, *55*, 4308 –4311.

2) Lautens, M.; Fagnou, K.; Yang, D. Rhodium-Catalyzed Asymmetric Ring Opening Reactions of Oxabicyclic Alkenes: Application of Halide Effects in the Development of a General Process. J*. Am. Chem. Soc*. **2003**, *125*, 14884−14892.

3) Christi, M.; Groetsch, S. Cyclohexa-1,2,4-triene from 1-Bromocyclohexa-1,4-diene. *Eur. J. Org. Chem*. **2000**, 1871-1874.

4) Qiu, S.; Zhai, S.; Wang, H.; Chena, X.; Zhai, H. One-pot synthesis of benzo[b]fluorenones via a cobalt-catalyzed MHP-directed [3+2] annulation/ ring-opening/dehydration sequence. *Chem. Commun*., **2019**, *55*, 4206-4209.

5) Blanchot, M.; Candito, D. A. Formal Synthesis of Nitidine and NK109 via Palladium-Catalyzed Domino Direct Arylation/N-Arylation of Aryl Triflates. *Org. Lett*. **2011**, *13*, 1486−1489.

6) Davoust, M.; Kitching, J. A.; Fleming, M. J. Lautens. M. Diastereoselective Benzylic Arylation of Tetralins; *Chem. Eur. J.* **2010**, *16*, 50 –54.

7) Kitamura, T.; Fukatsu, N.; Fujiwara, Y. *J.* (Phenyl)[3-(Trimethylsilyl)-2-naphthyl]iodonium Triflate as a New Precursor of 2,3-Didehydronaphthalene**.** *Org. Chem.* **1998**, *63*, 8579−8581.

8) Xu, W-B.; Ghorai S.; Huang, W.; Li, C. Rh(I)/Bisoxazolinephosphine-Catalyzed Regio- and Enantioselective Allylic Substitutions. *ACS Catal.* **2020**, *10*, 4491−4496.

9) Bongini, A.; Cardillo, G.; Orena, M.; Porzi, G.; Sandri, S. Regio- and Stereocontrolled Synthesis of Epoxy Alcohols and Triols from Allylic and Homoallylic Alcohols via Iodo Carbonates *J. Org. Chem.* **1982**, *47*, 4626-4633.

10) Fischer, C.; Defieber, C.; Suzuki, T.; Carreira, E. M. Readily Available [2.2.2]-Bicyclooctadienes as New Chiral Ligands for Ir(I): Catalytic, Kinetic Resolution of Allyl Carbonates. *J. Am. Chem. Soc.* **2004**, *126*, 1628–1629.

11) Ghorai, S.; Chirke; S. S.; Xu, W-B.; Chen, J-F.; Li, C. Cobalt-Catalyzed Regio- and Enantioselective Allylic Amination. *J. Am. Chem. Soc*. **2019**, *141*, 11430−11434.

12) Izumi, S.; Kobayashi, Y.; Takemoto, Y. Regio- and Stereoselective Synthesis of 1,2-*cis*-Glycosides by Anomeric *O*-Alkylation with Organoboron Catalysis. *Org. Lett.* **2019**, *21*, 665−670.

13) Mohal, N.; Vasella, A. Synthesis of Fusion-Isomeric Imidazopyridines and Their Evaluation as Inhibitors of syn- and anti-Protonating Glycosidase; *Helv. Chim. Acta* **2005**, *88*, 100-119.

14) Lee, D.; Taylor, M. S. Borinic Acid-Catalyzed Regioselective Acylation of Carbohydrate Derivatives; *J. Am. Chem. Soc*. **2011**, *133*, 3724–3727.

15) Li, R.-Z.; Tang, H.; Wan, L.; Zhang, X.; Fu, Z.; Liu, J.; Yang, S.; Jia, Da; Niu, D. Site-Divergent Delivery of Terminal Propargyls to Carbohydrates by Synergistic Catalysis. *Chem.* **2017**, *3*, 834−845.

16) Milhomme, O.; Dhénin, S. G. Y.; Djedaïni-Pilard, F.; Moreau, V.; Grandjean, C. Synthetic studies toward the anthrax tetrasaccharide: alternative synthesis of this antigen. *Carbohydr. Res*. **2012**, *356*, 115–131.

17) Chambers, D. J.; Evans, G.R.; Fairbanks. A. J. An approach to the synthesis of α-(1-6)-C-disaccharides by tandem Tebbe methylenation and Claisen rearrangement. *Tetrahedron* **2005**, *61*, 7184–7192.

18) Lv, J.; Luo, T.; Zou, D.; Dong, H. Using DMF as Both a Catalyst and Cosolvent for the Regioselective Silylation of Polyols and Diols. *Eur. J. Org. Chem.* **2019**, *2019*, 6383− 6395.

19) Dey, K.; Jayaraman, N. Anomeric alkylations and acylations of unprotected mono- and disaccharides mediated by pyridoneimine in aqueous solutions. *Chem. Commun.*, **2022**,*58*, 2224-2227.

20) Hoogendoorn, S.; van Puijvelde, G. H. M.; Kuiper, J.; van der Marel, G. J.; Overkleeft. H. S.; A Multivalent Ligand for the Mannose-6-Phosphate Receptor for Endolysosomal Targeting of an Activity-Based Probe. *Angew. Chem. Int. Ed*. **2014**, *53*, 10975 –10978.

21) Leteux, C.; Veyrieres, A. Synthesis of α-C-Glycopyranosides of D-Galactosamine and D-Glucosamine via Iodocyclization of Corresponding Glycals and Silver Tetraf luoroboranuide-Promoted Alkynylation at the Anomeric Centre. *J. Chem. Soc. Perkin Trans. 1,* **1994**, 2647-2655.

22) Frihed, T. G.; Heuckendorff, M.; Pedersen, C. M.; Bols, M. Easy Access to L-Mannosides and L-Galactosides by Using C-H Activation of the Corresponding 6-Deoxysugars. *Angew. Chem., Int. Ed.* **2012**, *51*, 12285−12288.

23) Tennant-Eyles, R. J.; Davis, B. G.; Fairbanks. A. J. Solid phase peptide templated glycosidic bond formation. *Tetrahedron: Asymmetry* **2003**, *14*, 1201–1210.

24) Wiebe, C.; Schlemmer, C.; Weckab, S.; Opatz, T. Sweet (hetero)aromatics: glycosylated templates for the construction of saccharide mimetics. *Chem. Commun.,* **2011**, *47*, 9212–9214.

25) Mohal, N.; Bernet, B.; Vasella, A. Synthesis of a Fusion-Isomeric Cellobionoimidazole and Its Evaluation against the *syn*-Protonating Glycosidase Cel7A. *Helv. Chim. Acta* **2005**, *88*, 3232-3252.

26) Halgren, T. A. Merck Molecular Force Field. I. Basis, Form, Scope, Parameterization, and Performance of MMFF94. *J. Comput. Chem.*, **1996**, *17*, 490-519.

27) *Spartan 14, Wavefunction Inc., Irvine, CA, USA*, **2014**

28) *Gaussian 09, Rev E.01*, Frisch, M. J.; Trucks, G. W.; Schlegel, H. B.; Scuseria, G. E.; Robb, M. A.; Cheeseman, J. R.; Scalmani, G.; Barone, V.; Mennucci, B.; Petersson, G. A.; Nakatsuji, H.; Caricato, M.; Li, X.; Hratchian, H. P.; Izmaylov, A. F.; Bloino, J.; Zheng, G.; Sonnenberg, J. L.; Hada, M.; Ehara, M.; Toyota, K.; Fukuda, R.; Hasegawa, J.; Ishida, M.; Nakajima, T. ; Honda, Y.; Kitao, O.; Nakai, H.; Vreven, T.; Montgomery, J. J. A.; Peralta, J. E.; Ogliaro, F.; Bearpark, M.; Heyd, J. J.; Brothers, E.; Kudin, K. N.; Staroverov, V. N.; Keith, T.; Kobayashi, R.; Normand, J.; Raghavachari, K.; Rendell, A.; Burant, J. C.; Iyengar, S. S.; Tomasi, J.; Cossi, M.; Rega, N.; Millam, J. M.; Klene, M.; Knox, J. E.; Cross, J. B.; Bakken, V.; Adamo, C.; Jaramillo, J.; Gomperts, R.; Stratmann, R. E.; Yazyev, O.; Austin, A. J.; Cammi, R.; Pomelli, C.; Ochterski, J. W.; Martin, R. L.; Morokuma, K.; Zakrzewski, V. G.; Voth,

G. A.; Salvador, P.; Dannenberg, J. J.; Dapprich, S.; Daniels, A. D.; Farkas, O.; Foresman, J. B.; Ortiz, J. V.; Cioslowski, J.; Fox, D. J. *Wallingford CT*, USA, **2013.**

29) Dolomanov, O.V.; Bourhis, L.J.; Gildea, R.J.; Howard, J.A.K.; Puschmann, H. OLEX2: a complete structure solution, refinement and analysis program. *J. Appl. Cryst*. **2009**, *42*, 339-341.

30) Sheldrick, G.M. SHELXT – Integrated space-group and crystal structure determination. *Acta Cryst.*, **2015**, *A71*, 3-8.

31) Sheldrick, G.M. A short history of SHELX. *Acta Cryst*., **2008**, *A64*, 112-122.

32) Bock, K.; Lundt, I.; Pedersen, C. A Study of ^{13}CH Coupling Constants in Hexopyranoses *J. Chem. Soc. Perkin Trans. 2*, **1974**, 293.

33) Perlin, A. S.; Casu, B. Carbon-13 and proton magnetic resonance spectra of D-glucose-13C. *Tetrahedron Lett.*,**1969**, 10, 34.

34) Nishimura, T.; Kawamoto, T.; Sasaki, K.; Tsurumaki, E.; Hayashi, T. Rhodium-Catalzyed Asymmetric Cyclodimerization of Oxa- and Azabicyclic Alkenes, *J. Am. Chem. Soc.* **2007**, *129*, 1492–1493.

35) Reduction: Loh, C. C. J.; Schmid, M.; Peters, B.; Fang, X.; Lautens, M. Exploiting Distal Reactivity of Coumarins: A Rhodium-Catalyzed Vinylogous Asymmetric Ring-Opening Reaction. *Angew. Chem. Int. Ed.* **2016**, *55*, 4600-4604.

36) TBAF desilylation: St-Pierre, G.; Hanessian, S. Solution and Solid-Phase Stereocontrolled Synthesis of 1,2-cis Glycopyranosides with Minimally Protected Glycopyranosyl Donors Catalyzed by BF_3−*N,N*-Dimethylformamide Complex. *Org. Lett*. **2016**, *18*, 3106−3109.

37) Click reaction: Xu, C.; Loh, C. C. J. A Multistage Halogen Bond Catalyzed Strain-Release Glycosylation Unravels New Hedgehog Signaling Inhibitors. *J. Am. Chem. Soc.* **2019**, *141*, 5381–5391.

38) HCl-catalyzed 2-deoxyglycosylation: Xu, C.; Rao, V.U.B.; Weigen, J.; Loh, C. C. J. A robust and tunable halogen bond organocatalyzed 2-deoxyglycosylation involving quantum tunneling. *Nat. Commun.* **2020**, *11*, 4911.

39) Bures, J. A Simple Graphical Method to Determine the Order in Catalyst. *Angew. Chem. Int. Ed.* **2016**, *55*, 2028-2031.

40) Nielsen, C. D.-T.; Bures, J. Visual kinetic analysis, *Chem. Sci.*, **2019**, *10*, 348-353.

41) Neese, F. The ORCA program system. *Wiley Interdiscip. Rev. Comput. Mol. Sci.* **2012**, *2*, 73-78.

42) Neese, F. Software update: the ORCA program system, version 4.0. *Wiley Interdiscip. Rev. Comput. Mol. Sci.* **2018**, *8*, e1327.

43) Nesse, F. Software update: The ORCA program system—Version 5.0. *WIREs Comput. Mol. Sci.* **2022**, DOI: 10.1002/wcms.1606

44) Grimme, S.; Hansen, A.; Ehlert, S.; Mewes, J-M. r^{2}SCAN-3c: A “Swiss army knife” composite electronic-structure method. *J. Chem. Phys.* **2021**, *154*, 064103.

45) Furness, J. W.; Kaplan, A. D.; Ning, J.; Perdew, J. P.; J. Sun. J. Accurate and Numerically Efficient r^{2}SCAN Meta-Generalized Gradient Approximation. *J. Phys. Chem. Lett.* **2020**, *11*, 8208.

46) Caldeweyher, E.; Ehlert, S.; Hansen, A.; Neugebauer, H.; Spicher, S.; Bannwarth, C.; Grimme, S. A generally applicable atomic-charge dependent London dispersion correction. *J. Chem. Phys.* **2019**, *150*, 154122.

47) Kruse, H.; Grimme, S. A geometrical correction for the inter- and intra-molecular basis set superposition error in Hartree-Fock and density functional theory calculations for large systems. *J. Chem. Phys.* **2012**, *136*, 154101.

48) Garcia-Ratés, M.; Nesse, F. Effect of the Solute Cavity on the Solvation Energy and its Derivatives within the Framework of the Gaussian Charge Scheme. *J. Comput. Chem.* **2020**, *41*, 922-939.

49) Barone, V.; Cossi, M. Potential energy surfaces for the low-lying ^2A‘ and ^2A’ States of HO_2: Use of the diatomics in molecules model to fit *ab initio* data**.** *J. Phys. Chem. A* **1998**, *102*, 1995.

50) F. Neese, F. Wennmohs, A. Hansen, U. Becker. Efficient, approximate and parallel Hartree–Fock and hybrid DFT calculations. A ‘chain-of-spheres’ algorithm for the Hartree–Fock exchange. *Chem. Phys.* **2009**, *356*, 98-109.

51) K. Eichkorn, O. Treutler, H. Oehm, M. Häser, R. Ahlrichs. Auxiliary basis sets to approximate Coulomb potentials *Chem. Phys.* **1995**, *242*, 652-660.

52) K. Eichkorn, F. Weigend, O. Treutler, R. Ahlrichs. Auxiliary basis sets for main row atoms and transition metals and their use to approximate Coulomb potentials. *Theor. Chem. Acc.* **1997**, *97*, 119-124.

53) Grimme, S. Supramolecular Binding Thermodynamics by Dispersion-Corrected Density Functional Theory. *Chem. Eur. J.* **2012**, *18*, 9955-9964.

54) A. E. Reed, R. B. Weinstock, F. Weinhold. Natural population analysis. *J. Chem. Phys.* **1985**, *83*,735.

55) F. Weinhold, C. R. Landis, Valency and Bonding: A Natural Bond Orbital Donor-Acceptor Perspective; Cambridge UK Press: Cambridge, UK, **2005**.

56) NBO 7.0. E. D. Glendening, J. K. Badenhoop, A. E. Reed,J. E. Carpenter, J. A. Bohmann, C. M. Morales, P. Karafiloglou, C. R. Landis, and F. Weinhold, Theoretical Chemistry Institute, University of Wisconsin, Madison, WI (2018)

57) Domingo, L. R.; Chamorro, E.; Pérez, P. Understanding the Reactivity of Captodative Ethylenes in Polar Cycloaddition Reactions. A Theoretical Study. *J. Org. Chem.* **2008**, *73*, 4615-4624.

58) T. Lu, F.u Chen, Multiwfn: A Multifunctional Wavefunction Analyzer, *J. Comput. Chem.* **2012**, *33*, 580-592.

59) Y-Cd(ecp-28), Hf-Hg(ecp-46): Andrae, D.; Haeussermann, U.; Dolg, M.; Stoll, H.; Preuss, H. Energy-Adjusted *ab initio* Pseudopotentials for the Second and Third Row Transition Elements. *Theor. Chim. Acta* **1990**, *77*, 123-141.

60) ECP parameters for Rh [Def2-ECP] have been obtained from: TURBOMOLE (7.0.2)

61) Zimmerman, P. Reliable Transition State Searches Integrated with the Growing String Method. *J. Chem. Theory Comput.* **2013**, *9*, 3043-3050.

62) Zimmerman, P. M. Single-Ended Transition State Finding with the Growing String Method. *J. Comput.Chem.* **2015**, *36*, 601-611.

63) Grimme, S.; Bannwarth, C.; Shushkov, P. A Robust and Accurate Tight-Binding Quantum Chemical Method for Structures, Vibrational Frequencies, and Noncovalent Interactions of Large Molecular Systems Parametrized for All spd-Block Elements (Z = 1–86). *J. Chem. Theory Comput.* **2017**, *13*, 1989-2009.

64) Bannwarth, C.; Ehlert, S.; Grimme, S. GFN2-xTB—An Accurate and Broadly Parametrized Self-Consistent Tight-Binding Quantum Chemical Method with Multipole Electrostatics and Density-Dependent Dispersion Contributions. *J. Chem. Theory Comput.* **2019**, *15*, 1652-1671.

65) Ehlert, S.; Stahn, M., Spicher, S.; Grimme, S. Robust and Efficient Implicit Solvation Model for Fast Semiempirical Methods. *J. Chem. Theory Comput.* **2021**, *17*, 4250-4261.

66) Steinmetzer, J.; Kuper, S.; Gräfe, S. pysisyphus: Exploring potential energy surfaces in ground and excited states. *Int. J. Quantum Chem.* **2021**, *121*, e26390.

67) https://github.com/eljost/pysisyphus

68) Mardirossian. N.; Head-Gordon, M. *ω*B97M-V: A combinatorially optimized, range-separated hybrid, meta-GGA density functional with VV10 nonlocal correlation. *J. Chem. Phys.* **2016**, *144*, 214110.

For the superiority of the *ω*B97M-V functional in benchmarks, see Refs 69-71:

69) Mardirossian. N.; Head-Gordon, M. Thirty years of density functional theory in computational chemistry: an overview and extensive assessment of 200 density functionals. *Molecular Physics* **2017**, *115*, 2315-2372.

70) Iron, M. A.; Janes, T. Evaluating Transition Metal Barrier Heights with the Latest Density Functional Theory Exchange−Correlation Functionals: The MOBH35 Benchmark Database. *J. Chem. Phys.* **2019**, 123, 3761-3781.

71) Prasad, V. K.; Pei, Z.; Edelmann, S.; Otero-de-la-Roza, A.; DiLabio, G. A. New Comprehensive Benchmark Data Set for Barrier Heights and Reaction Energies: Assessment of Density Functional Approximations and Basis Set Incompleteness Potentials. *J. Chem. Theory Comput.* **2022**, *18*, 151-166.

72) Vydrov, O. A.; Voorhis, T. V. Nonlocal van der Waals density functional: The simpler the better. *J. Chem. Phys.* **2010**, 133, 244103.

73) C. Y. Legault, CYLview, 1.0b, Université de Sherbrooke, Sherbrooke, Quebec, Canada, 2009, http://www.cylview.org.

NMR Spectra

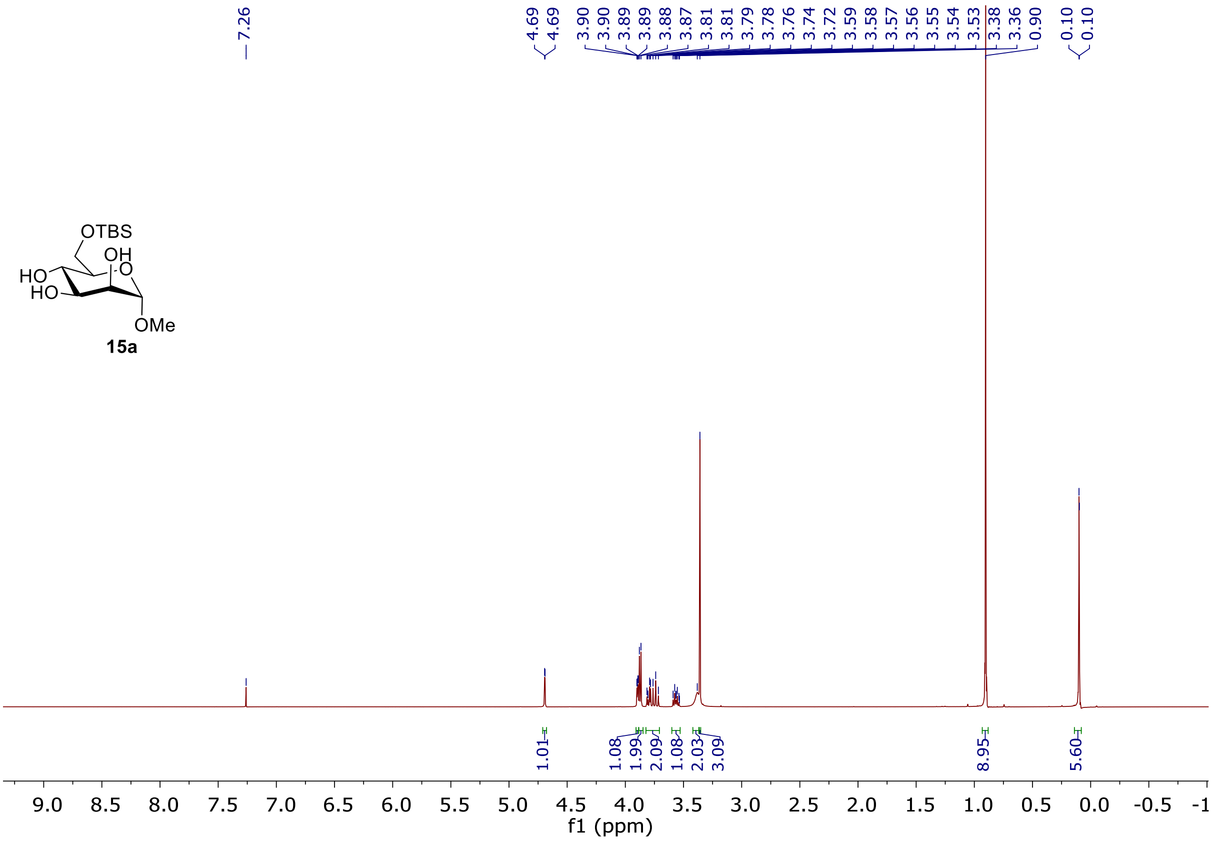

Supplementary Figure 36. ^{1}H spectra for **15a**

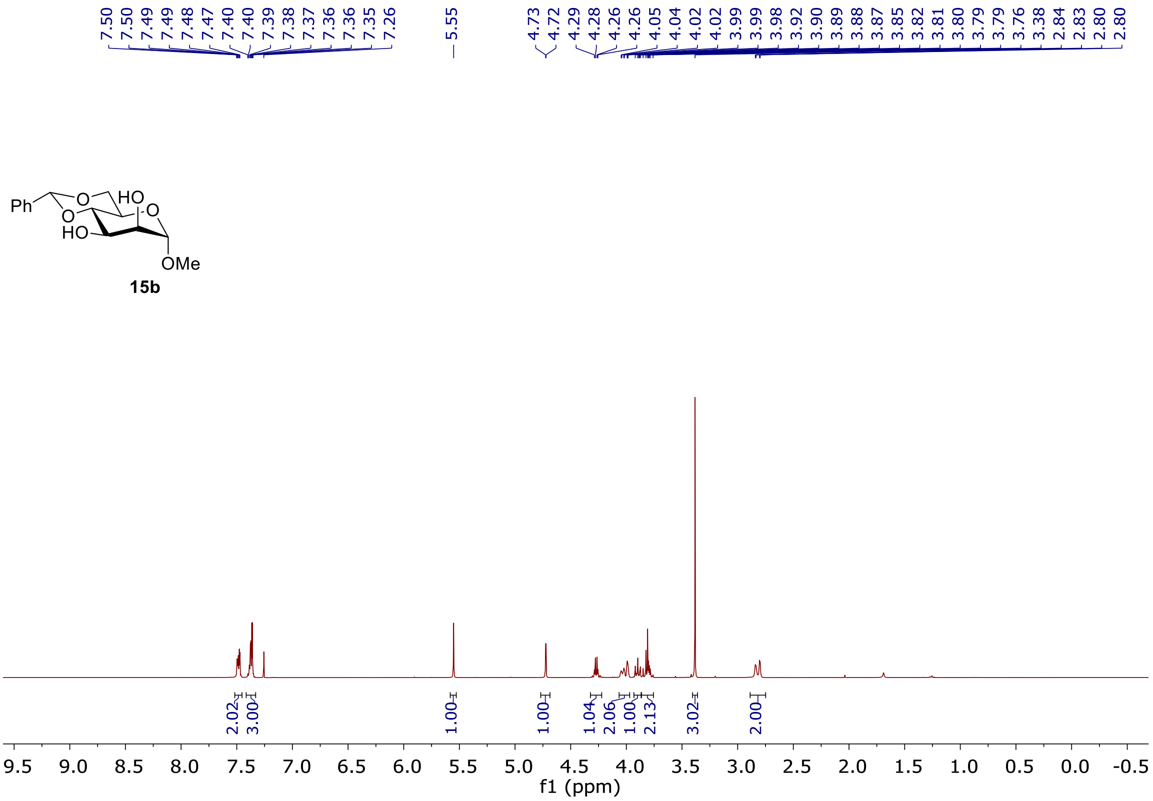

Supplementary Figure 37. ^{1}H spectra for **15b**

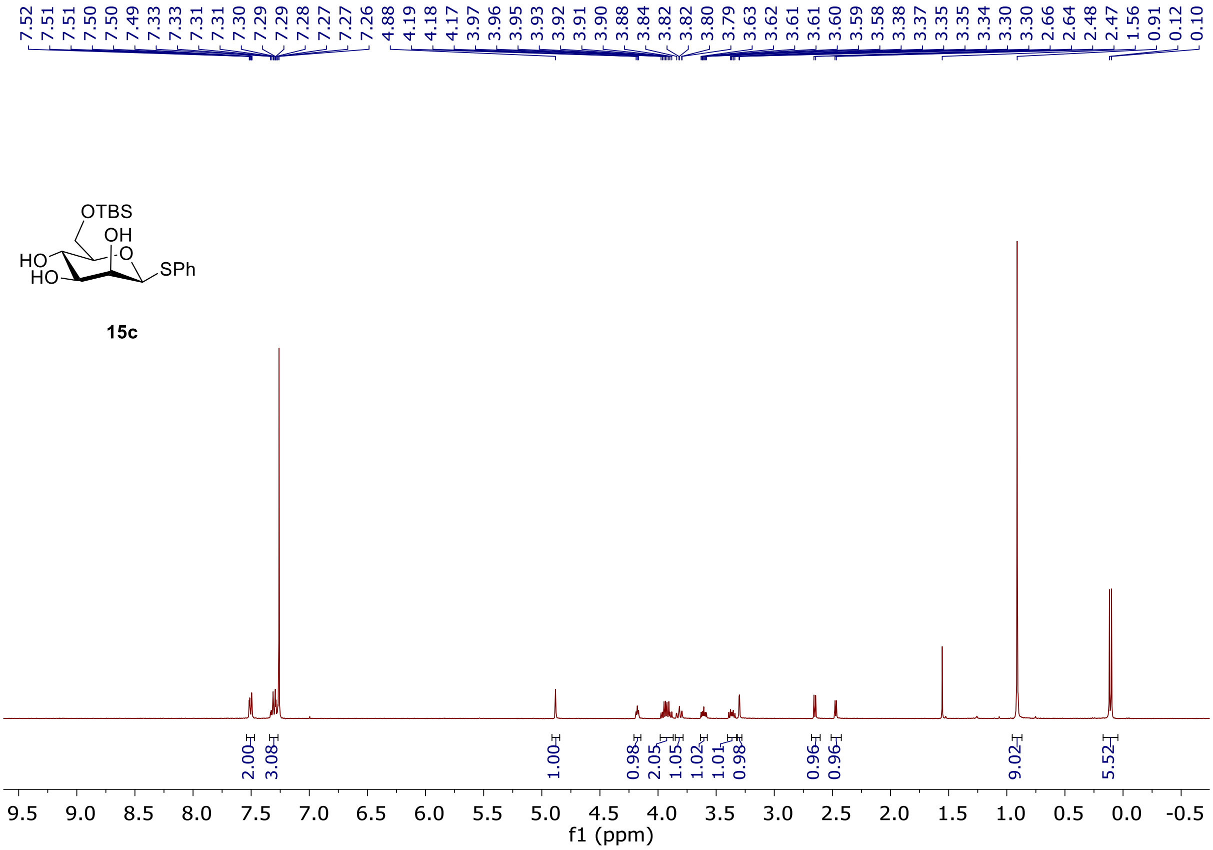

Supplementary Figure 38. ^{1}H spectra for **15c**

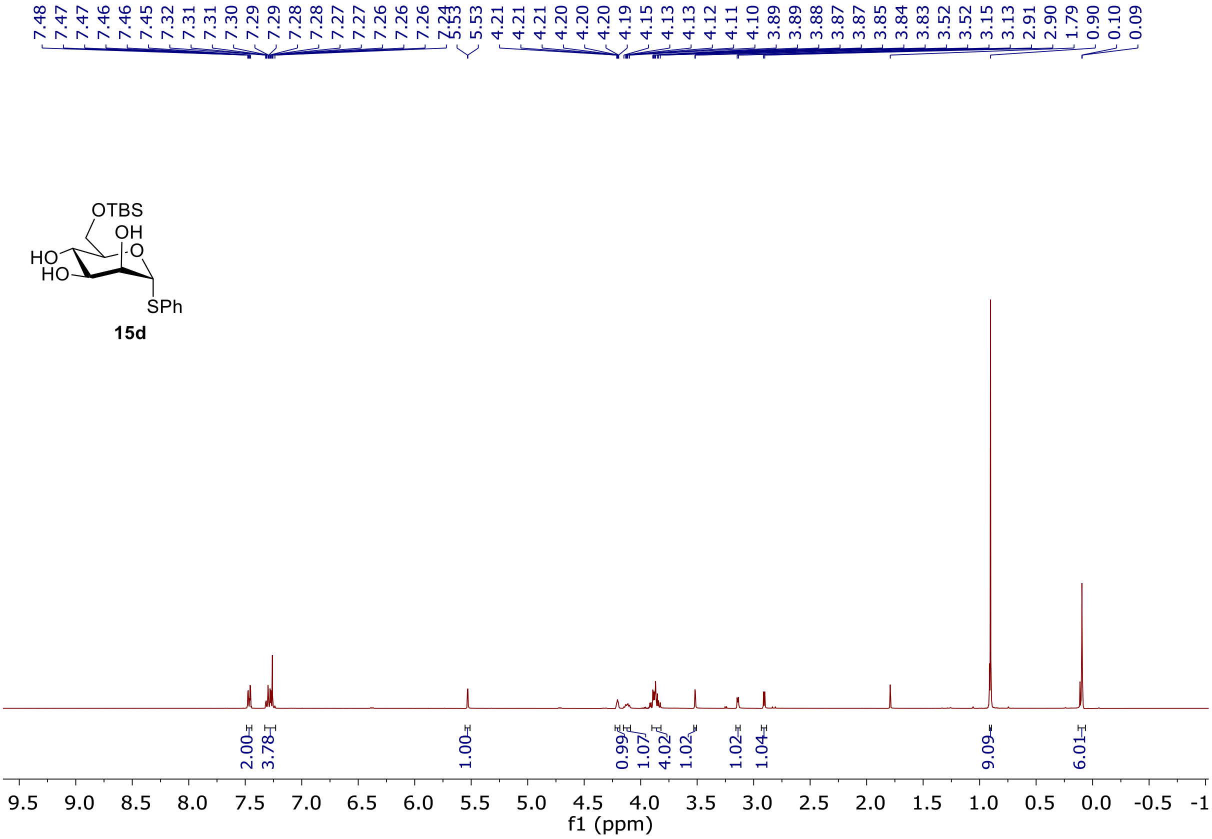

Supplementary Figure 39. ^{1}H spectra for **15d**

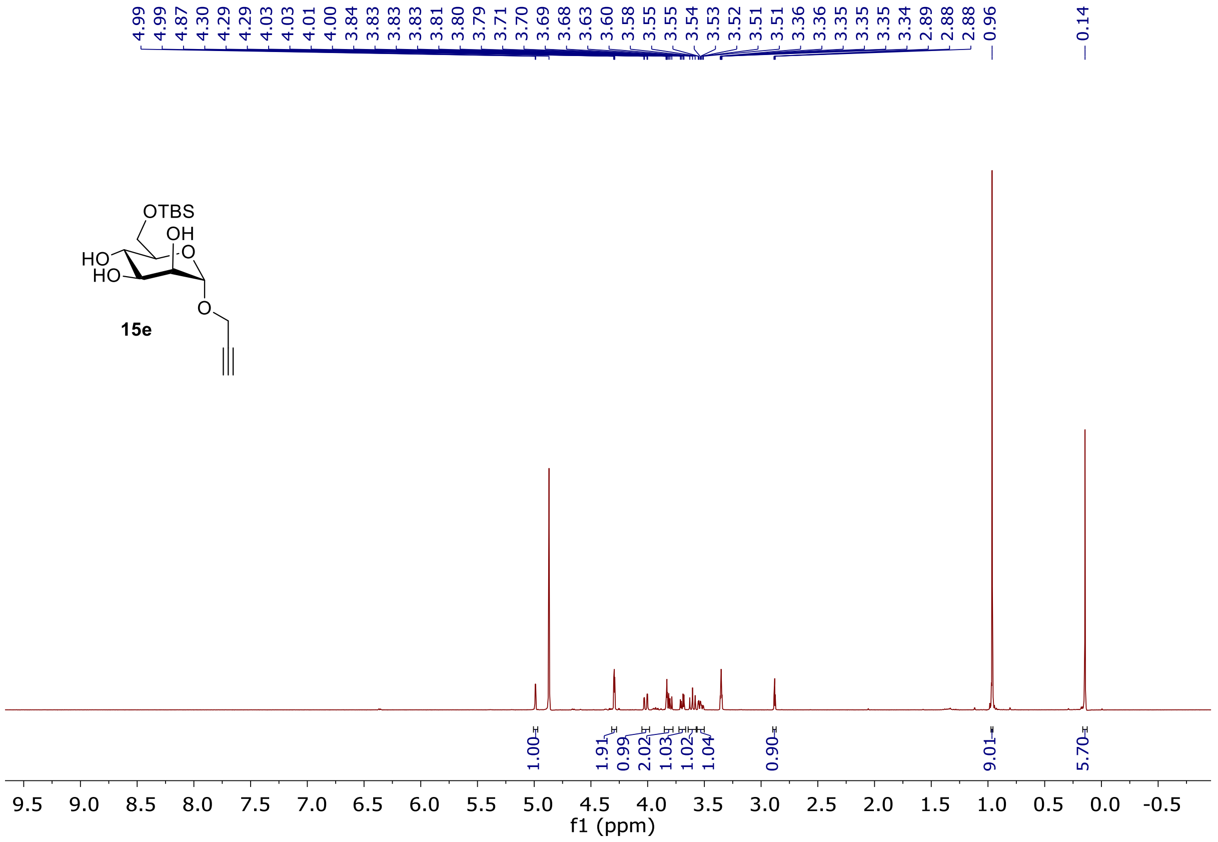

Supplementary Figure 40. ^{1}H spectra for **15e**

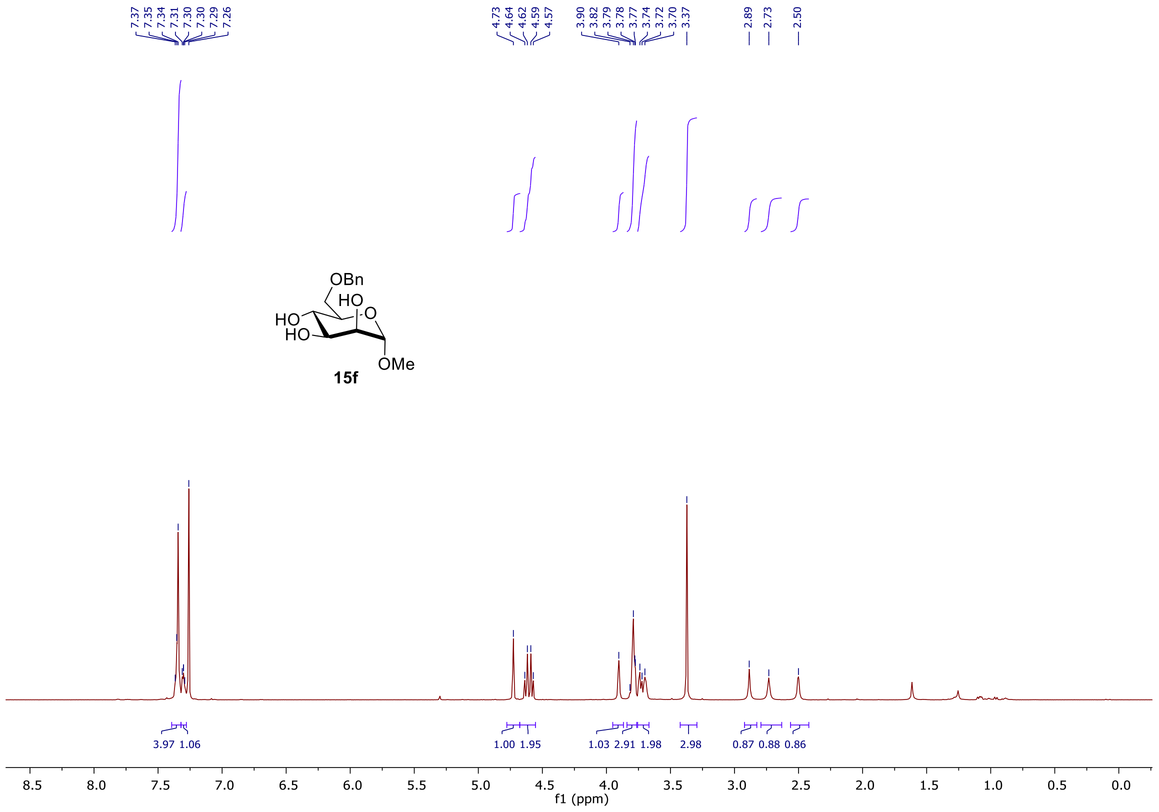

Supplementary Figure 41. ^{1}H spectra for **15f**

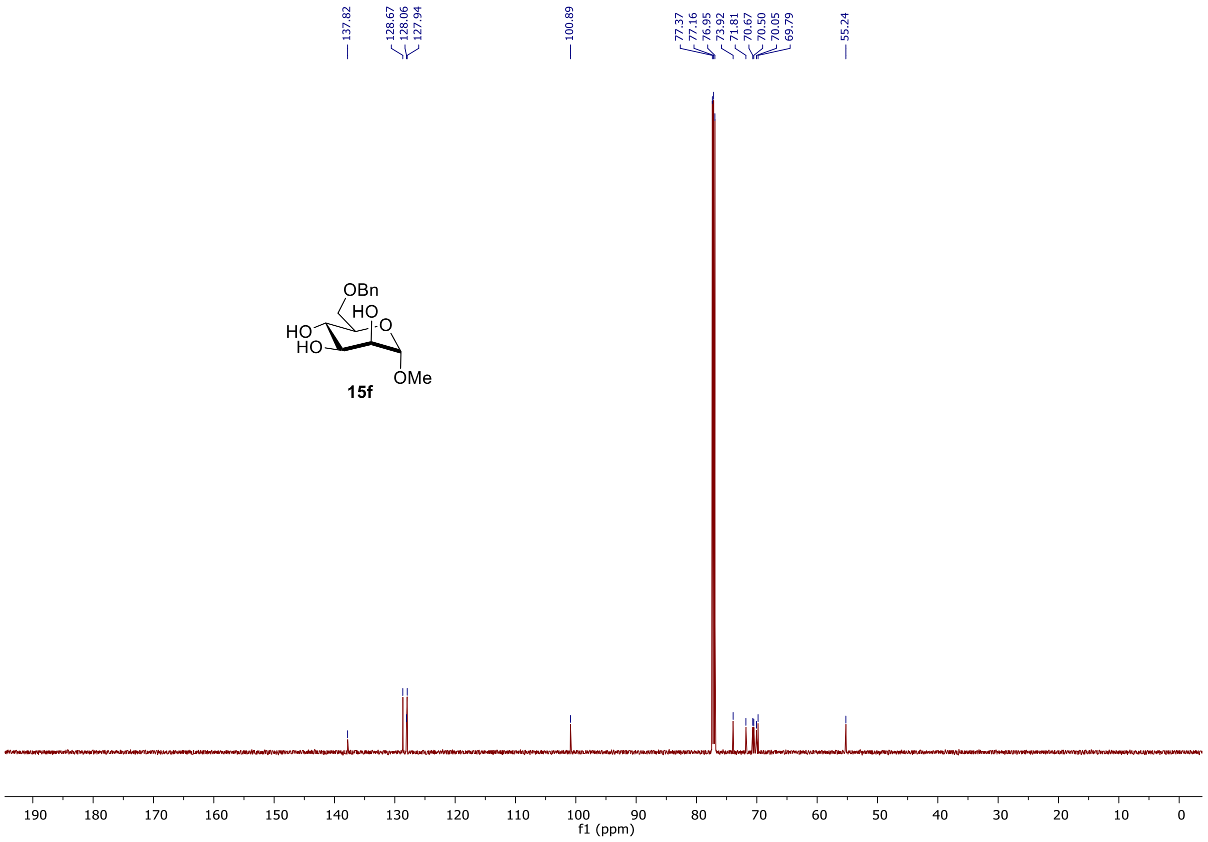

Supplementary Figure 42. ^{13}C spectra for **15f**

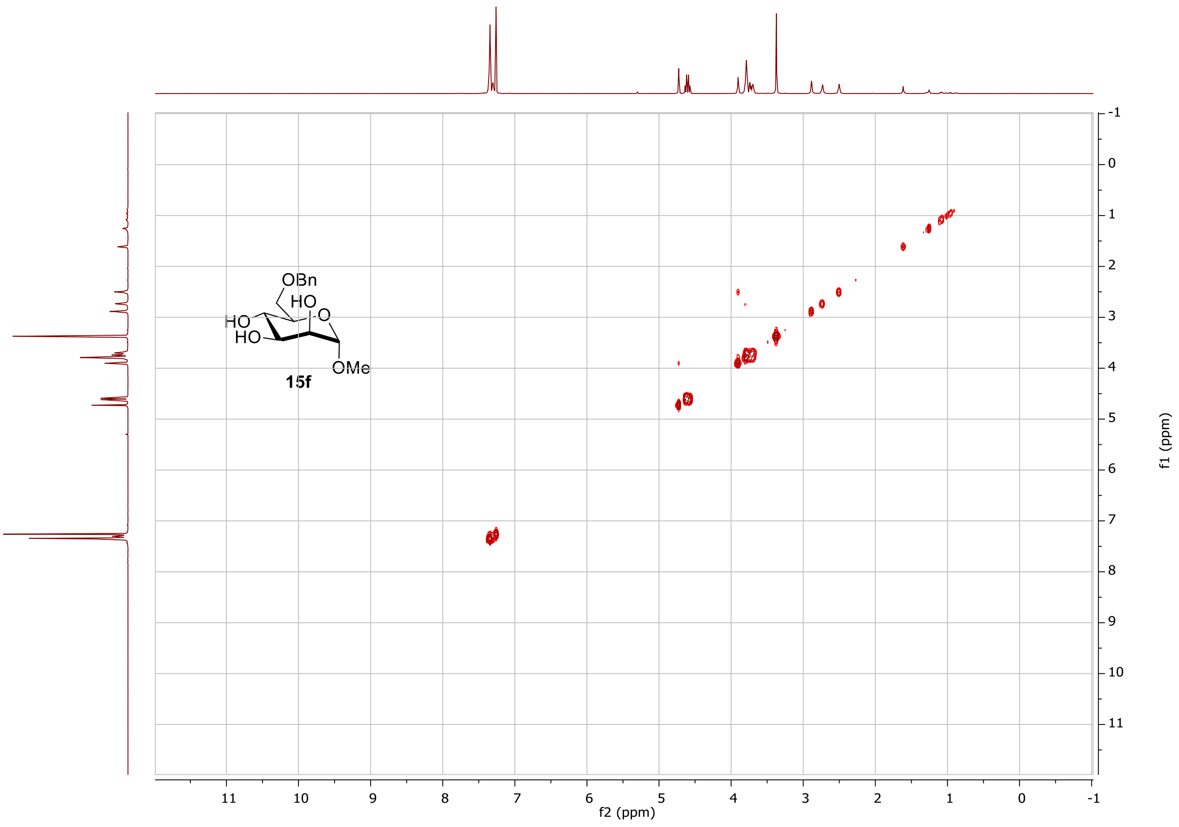

Supplementary Figure 43. COSY spectra for **15f**

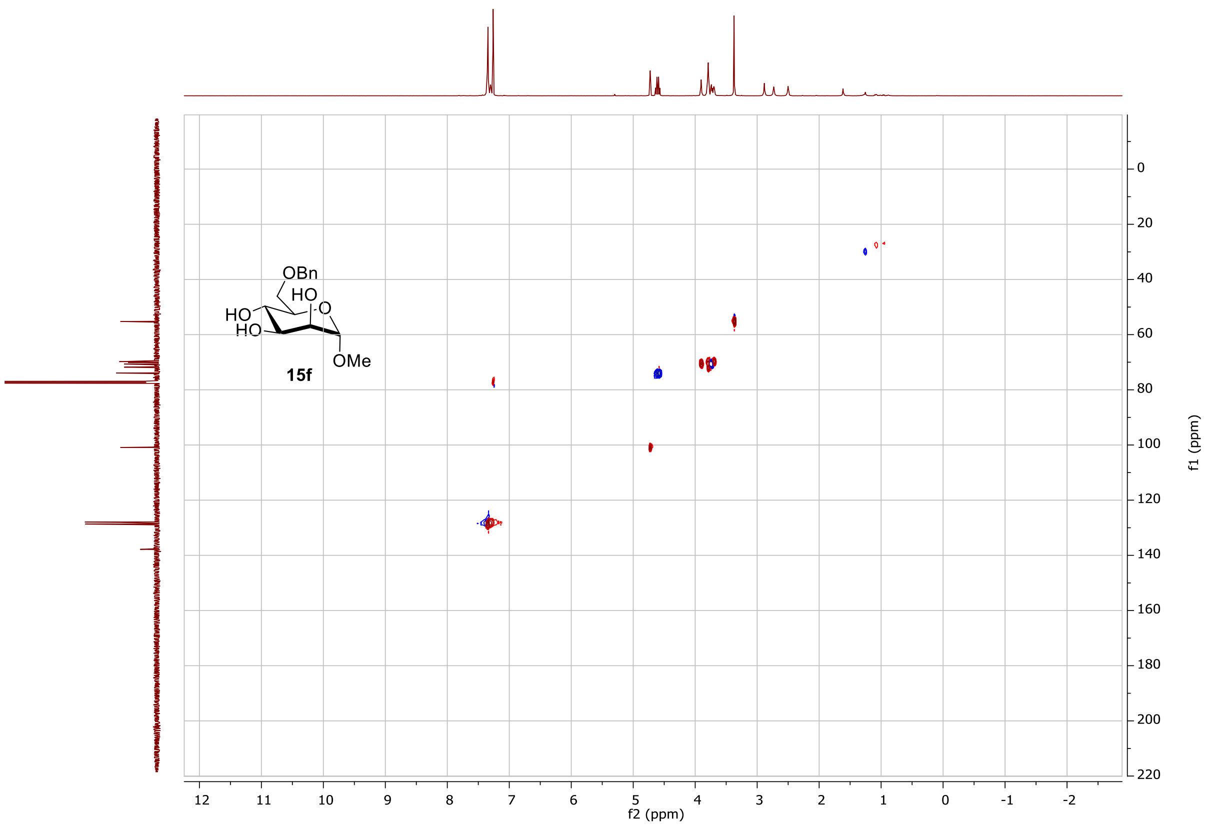

Supplementary Figure 44. HSQC spectra for **15f**

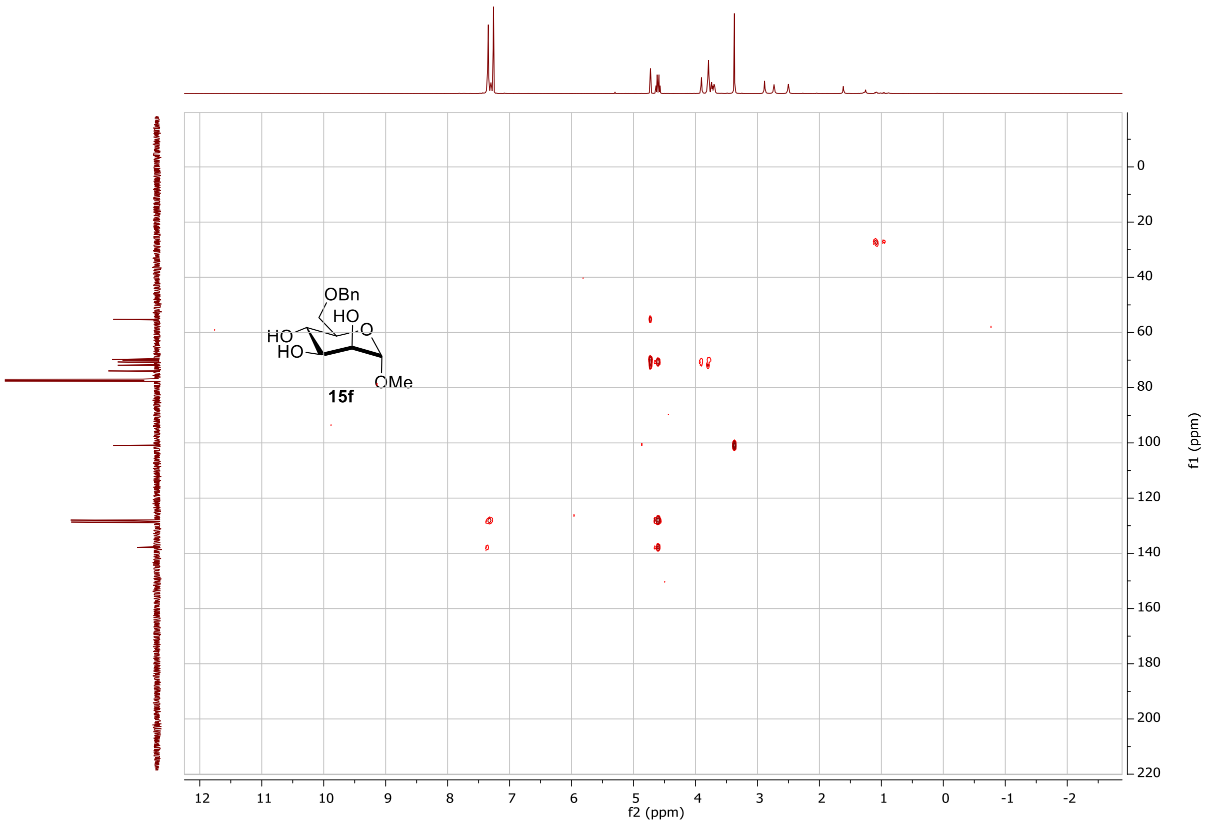

Supplementary Figure 45. HMBC spectra for **15f**

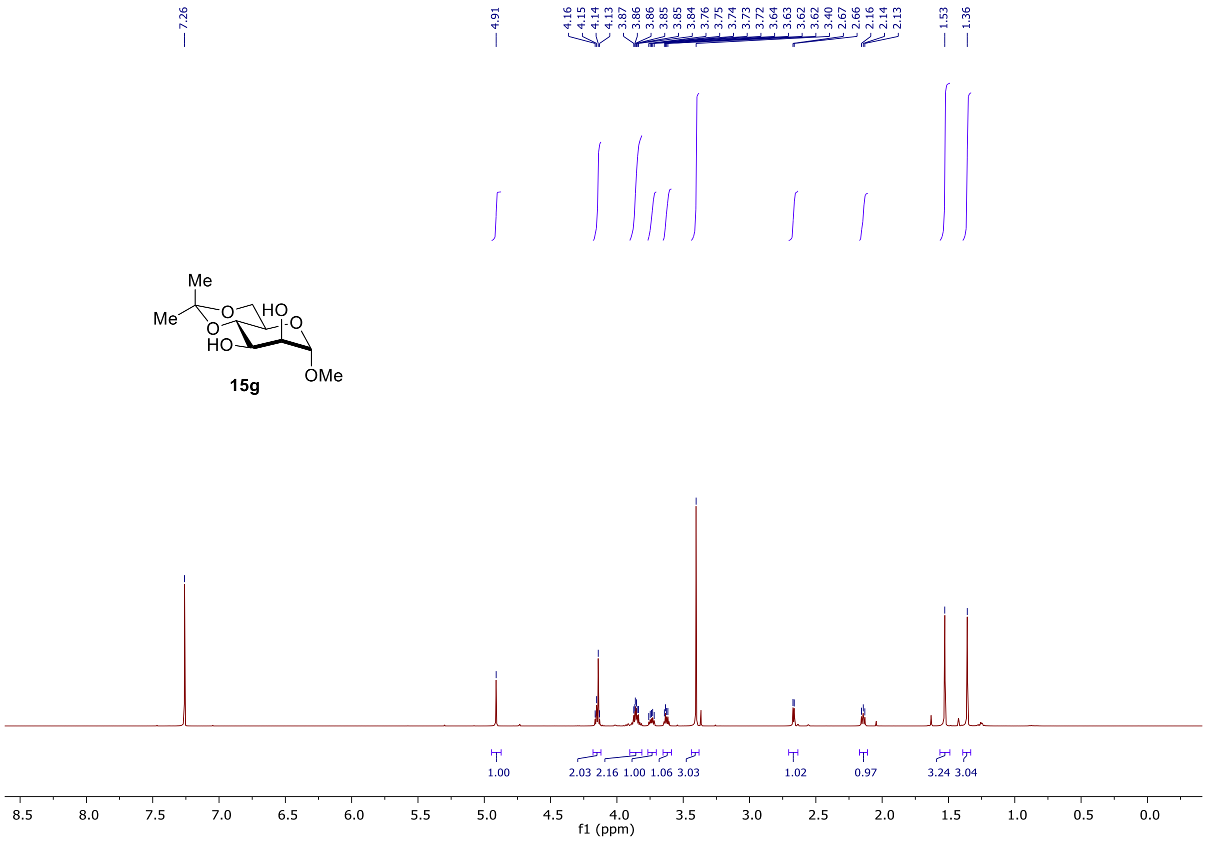

Supplementary Figure 46. ¹H spectra for **15g**

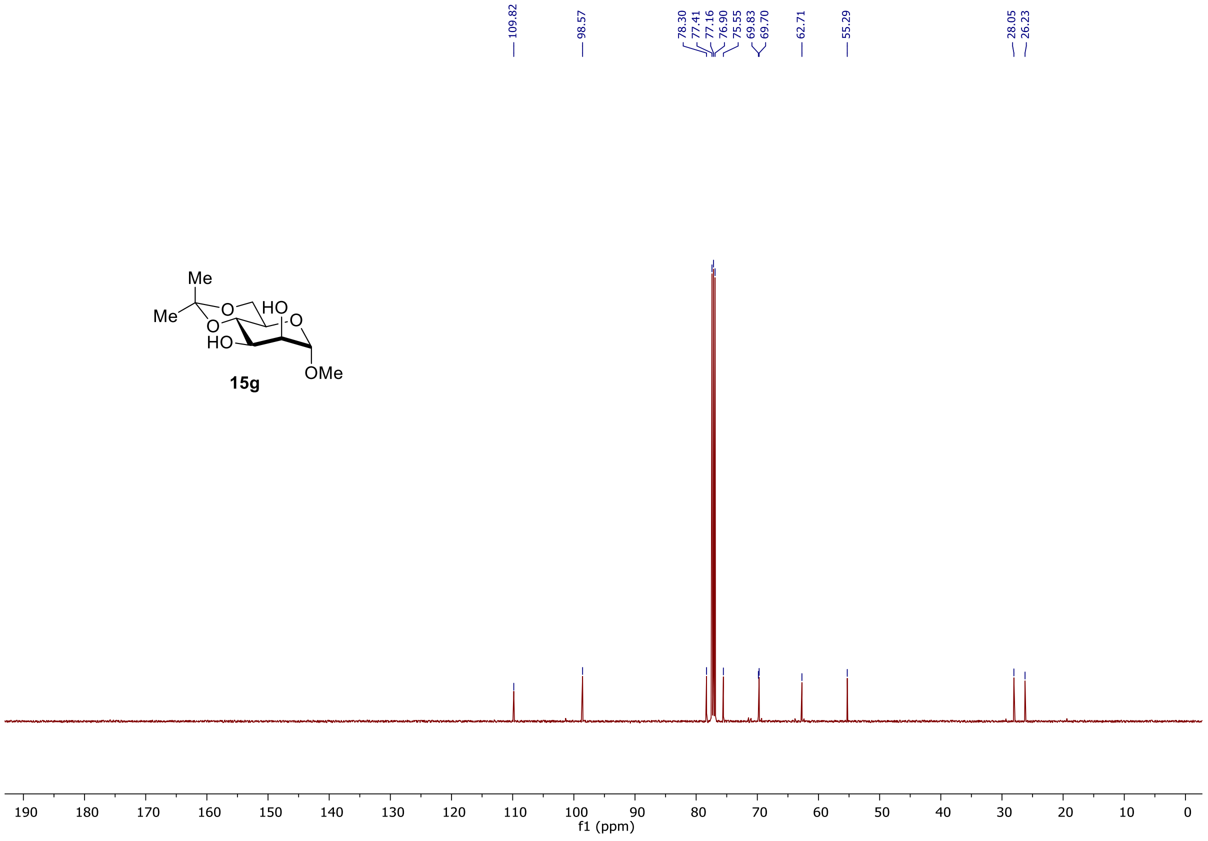

Supplementary Figure 47. ¹³C spectra for **15g**

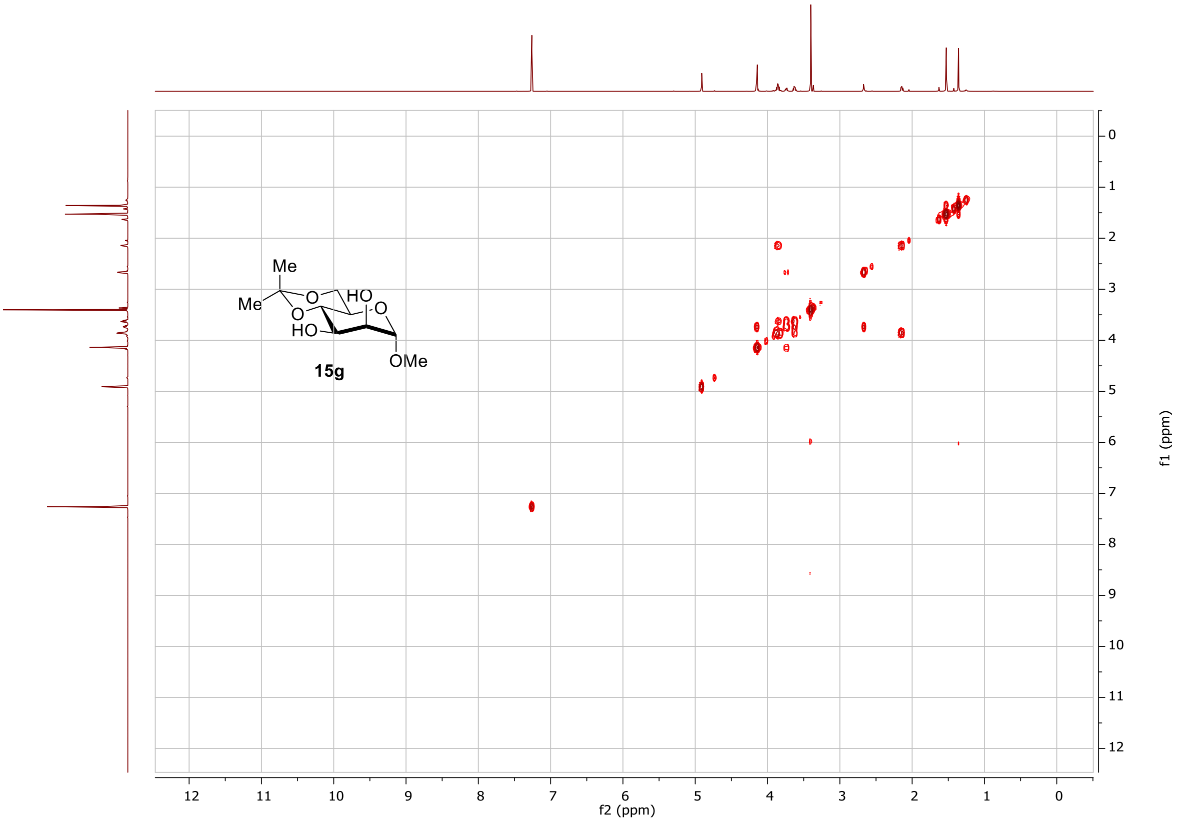

Supplementary Figure 48. COSY spectra for **15g**

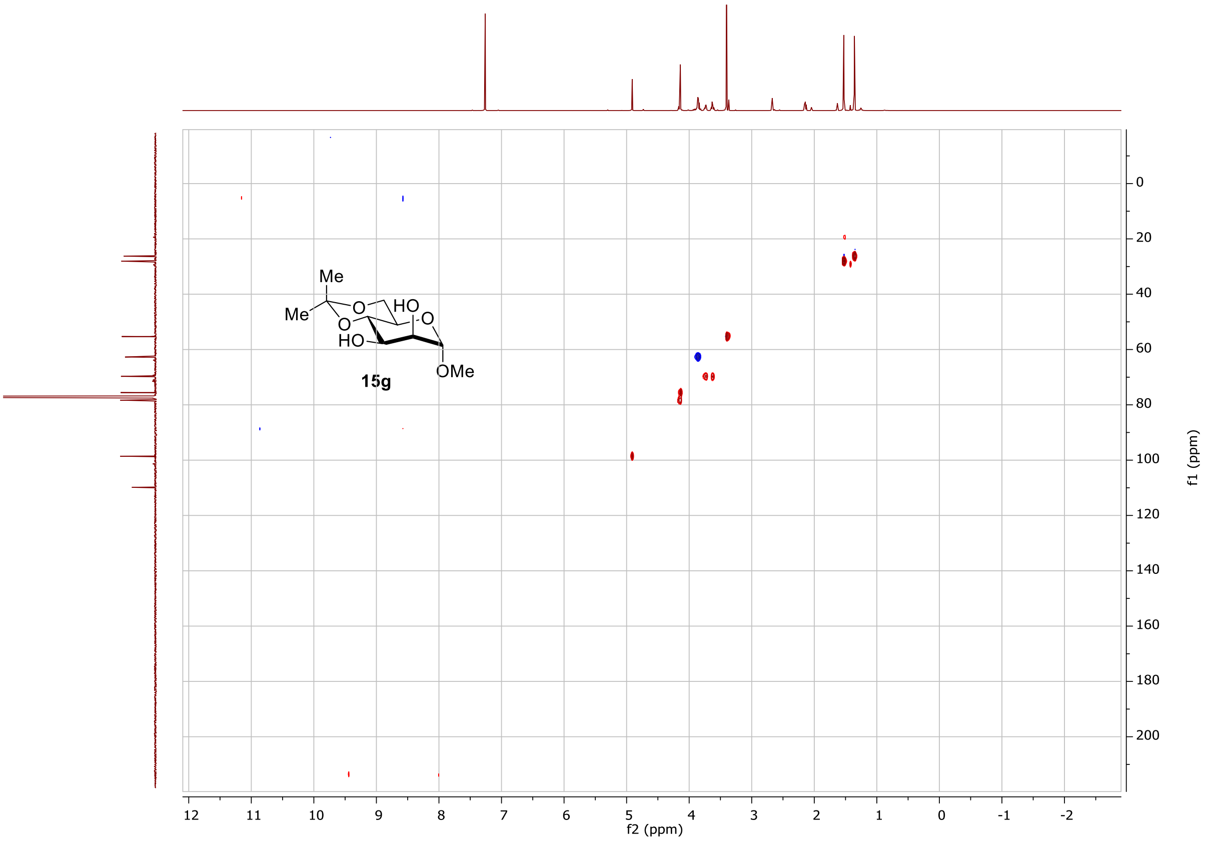

Supplementary Figure 49. HSQC spectra for **15g**

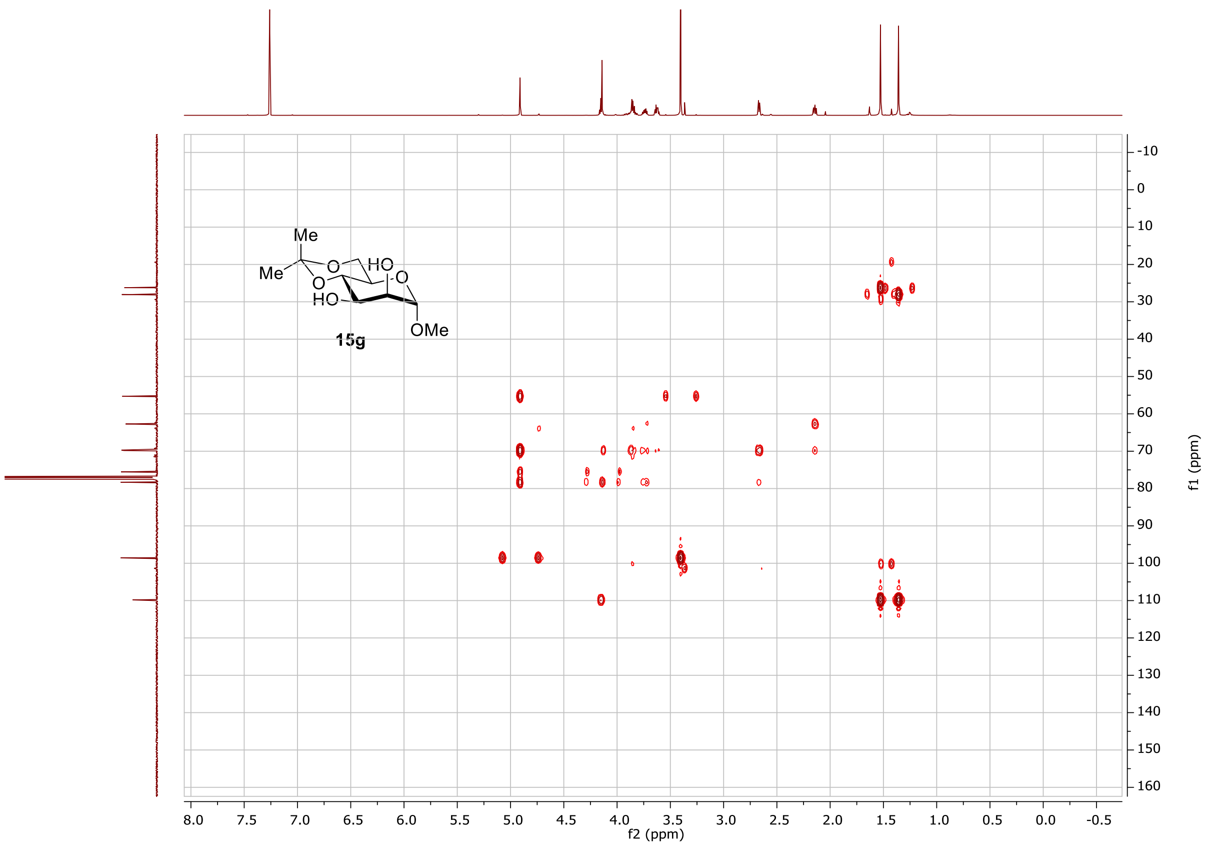

Supplementary Figure 50. HMBC spectra for **15g**

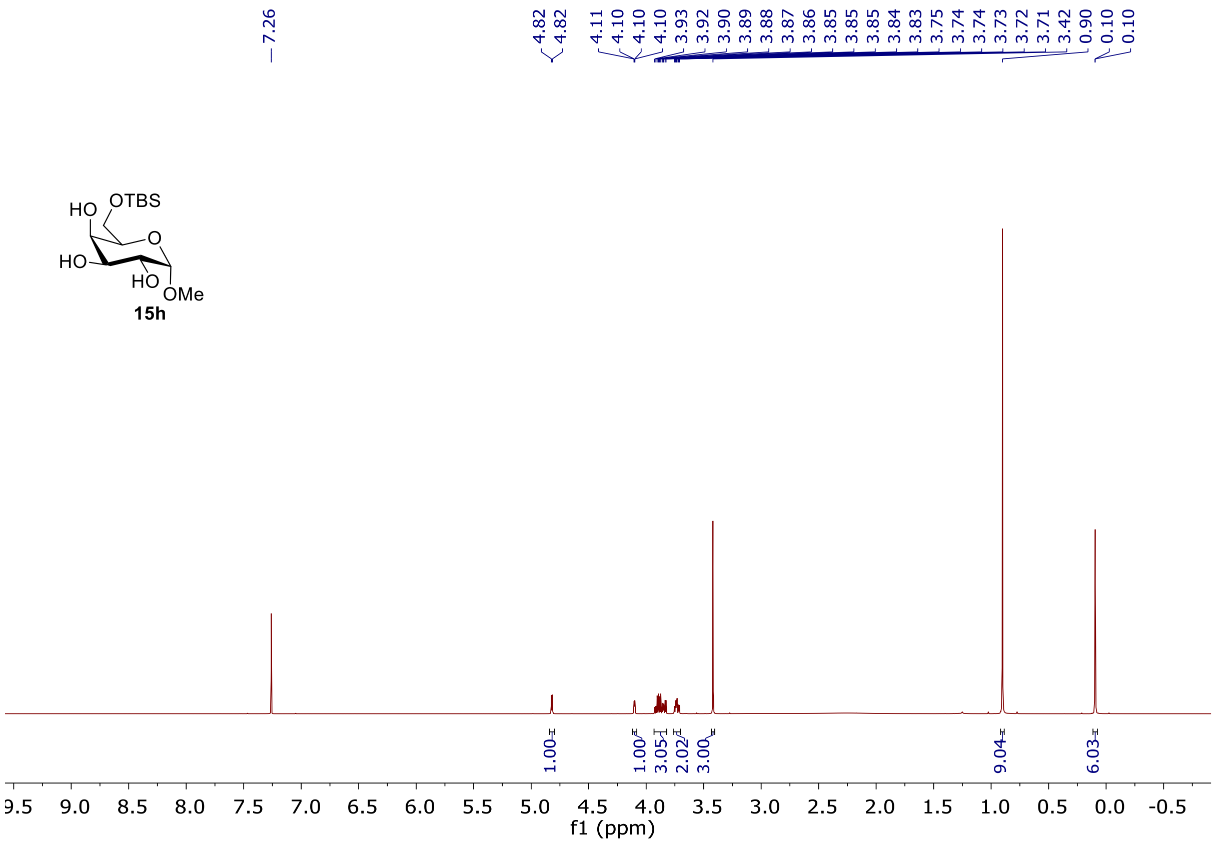

Supplementary Figure 51. ^{1}H spectra for **15h**

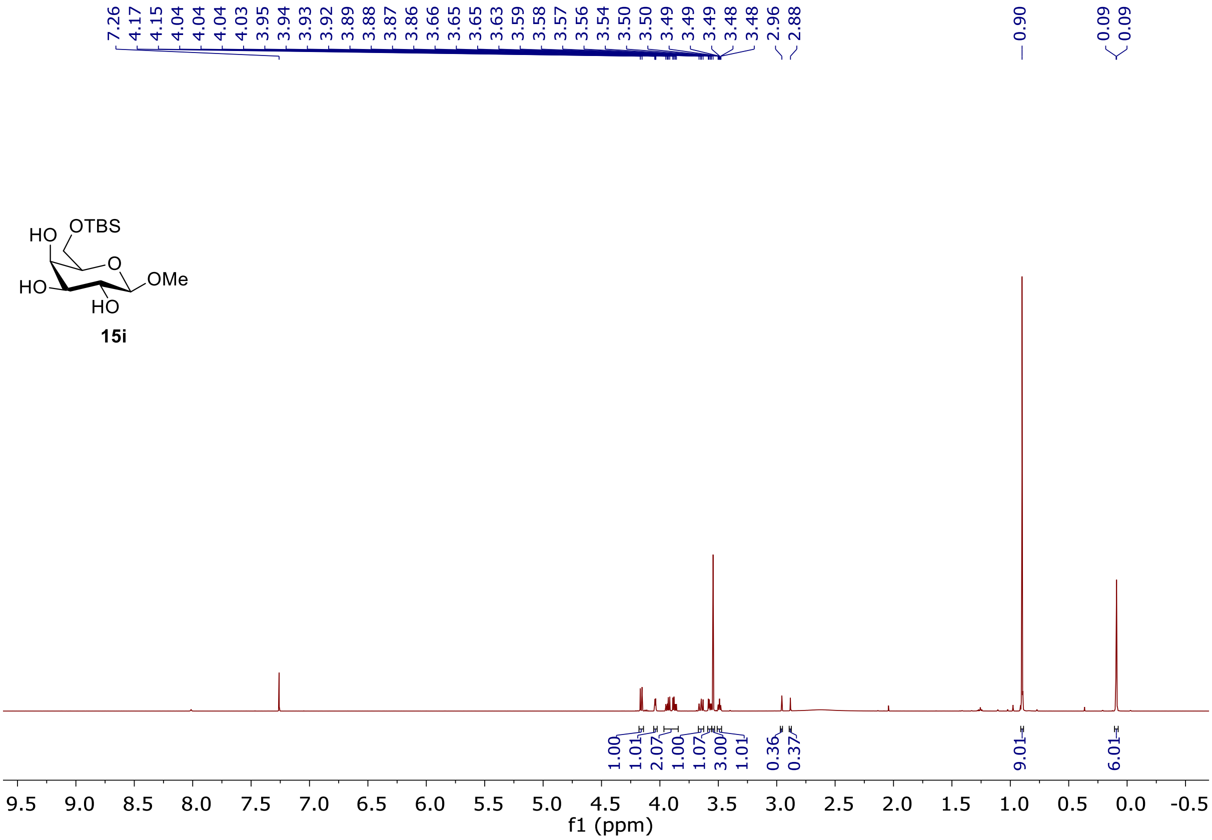

15i

Supplementary Figure 52. ^{1}H spectra for **15i**

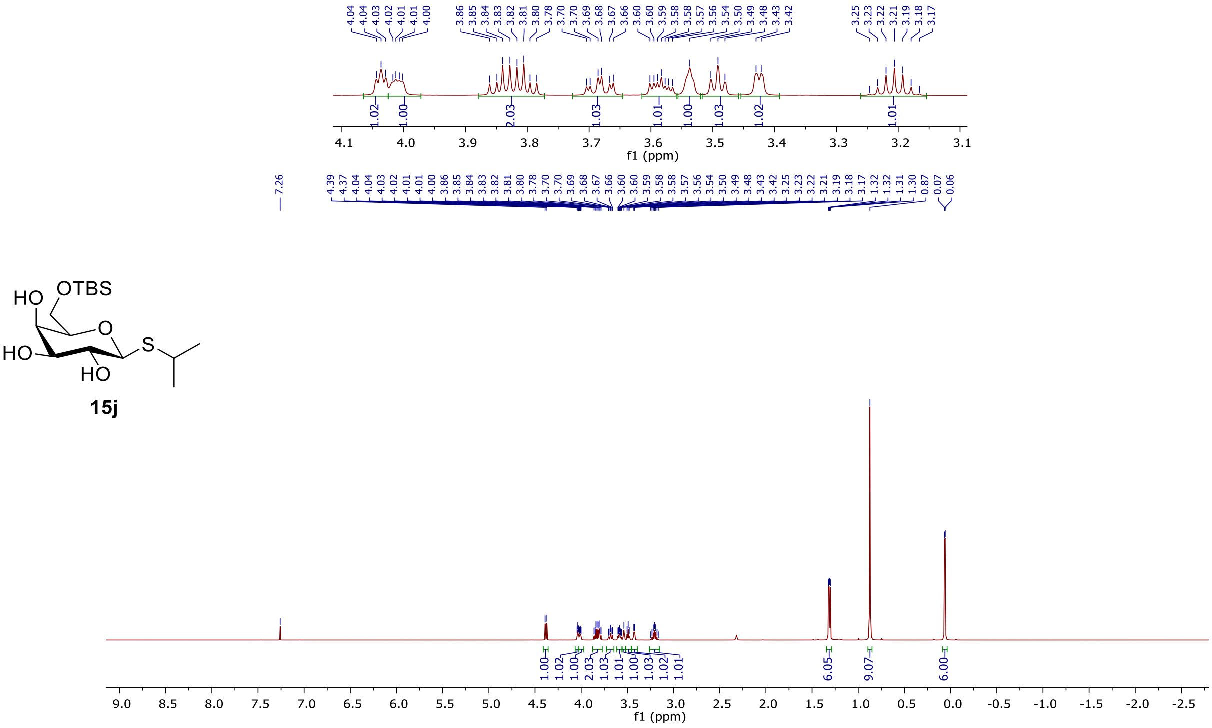

15j

Supplementary Figure 53. ^{1}H spectra for **15j**

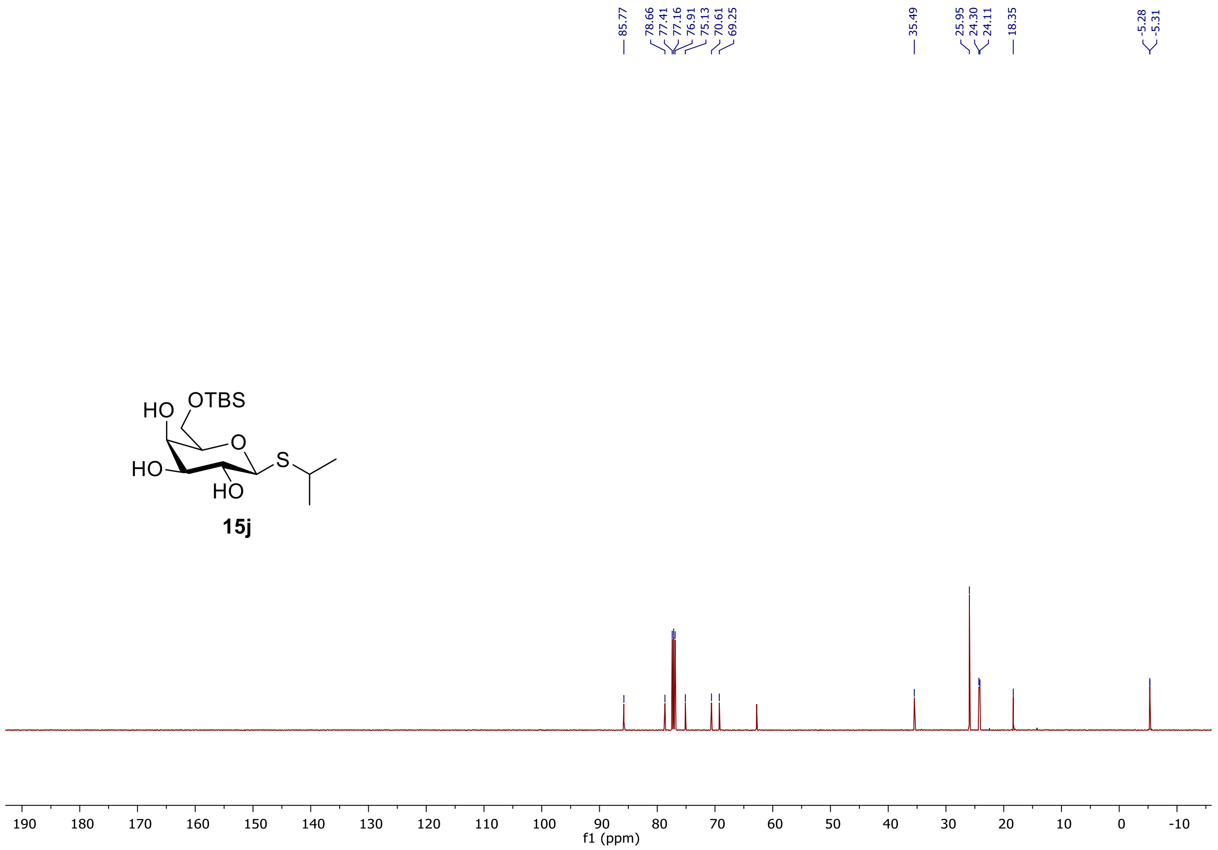

Supplementary Figure 54. ^{13}C spectra for **15j**

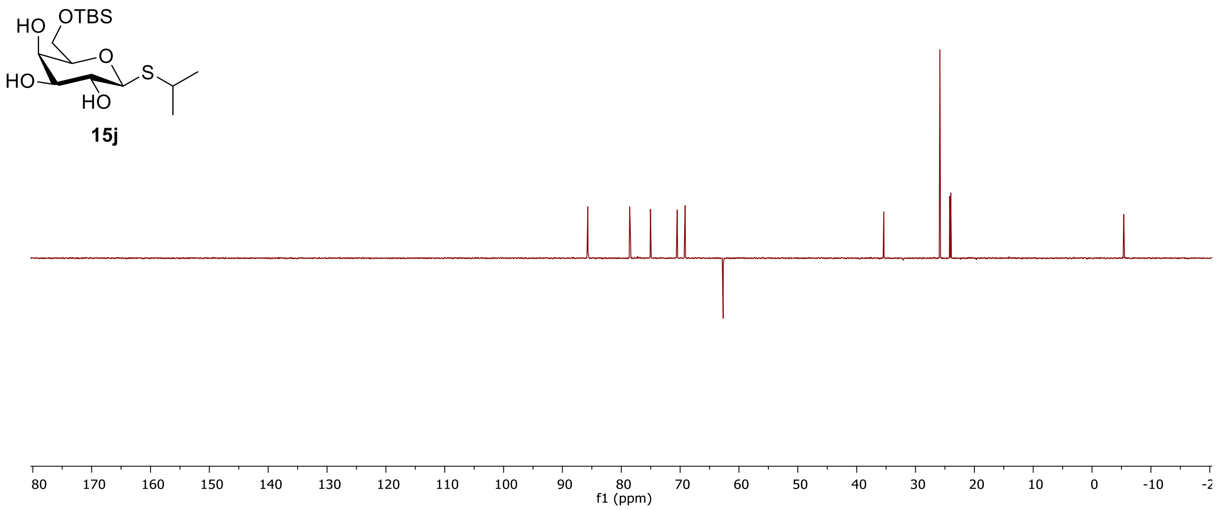

Supplementary Figure 55. DEPT spectra for **15j**

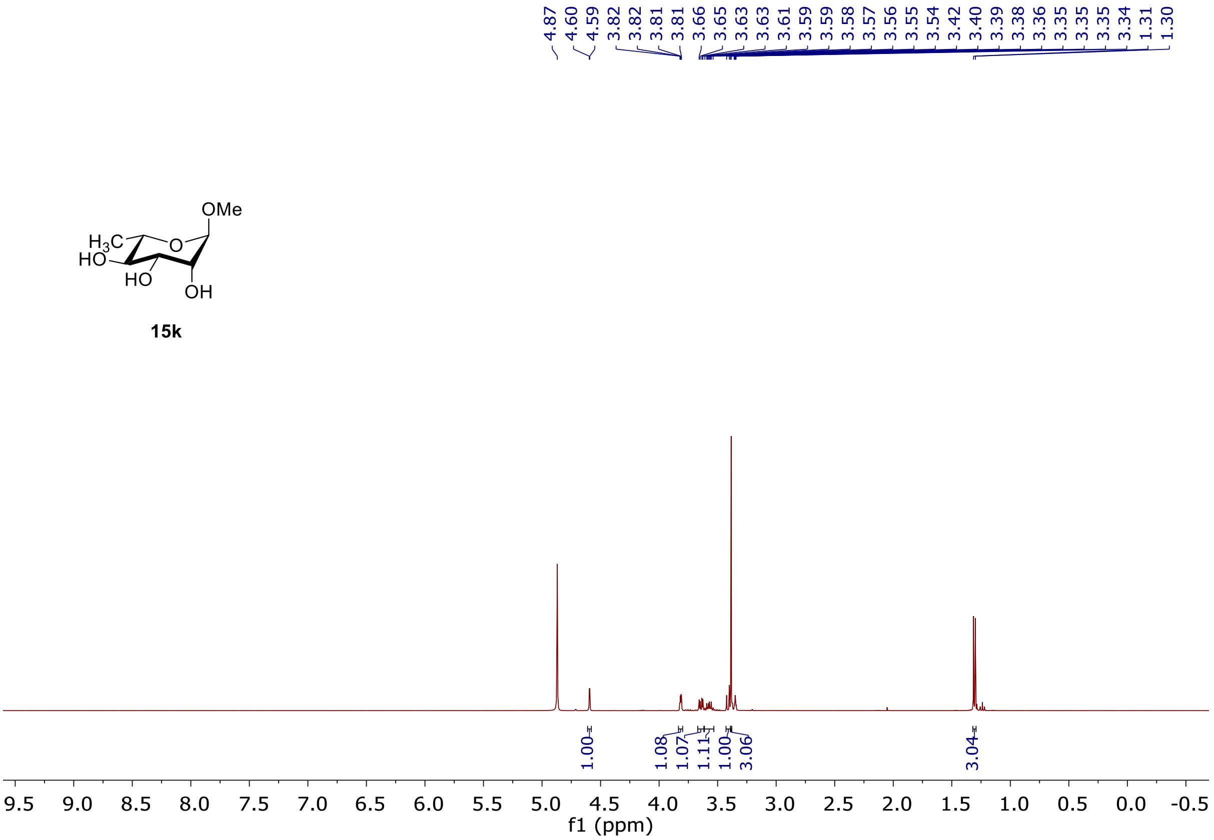

Supplementary Figure 56. ^{1}H spectra for **15k**

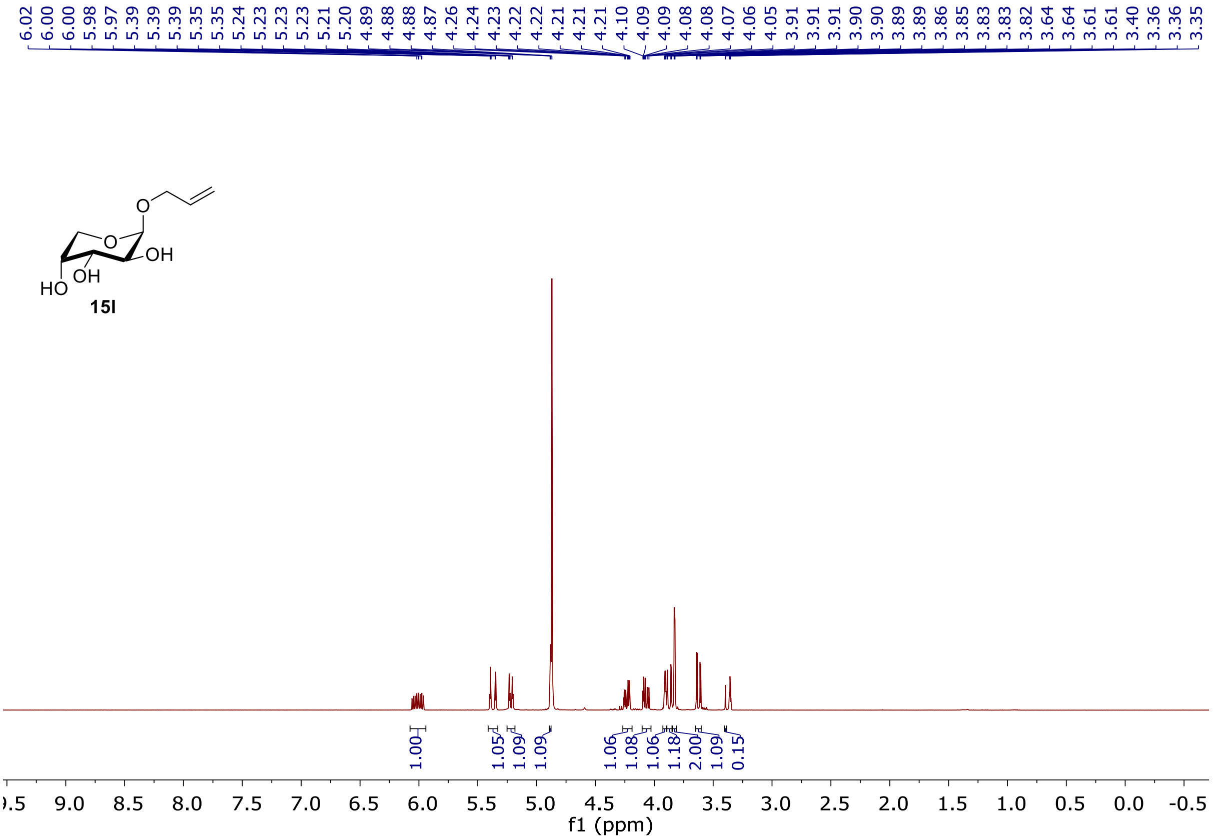

Supplementary Figure 57. ^{1}H spectra for **15l**

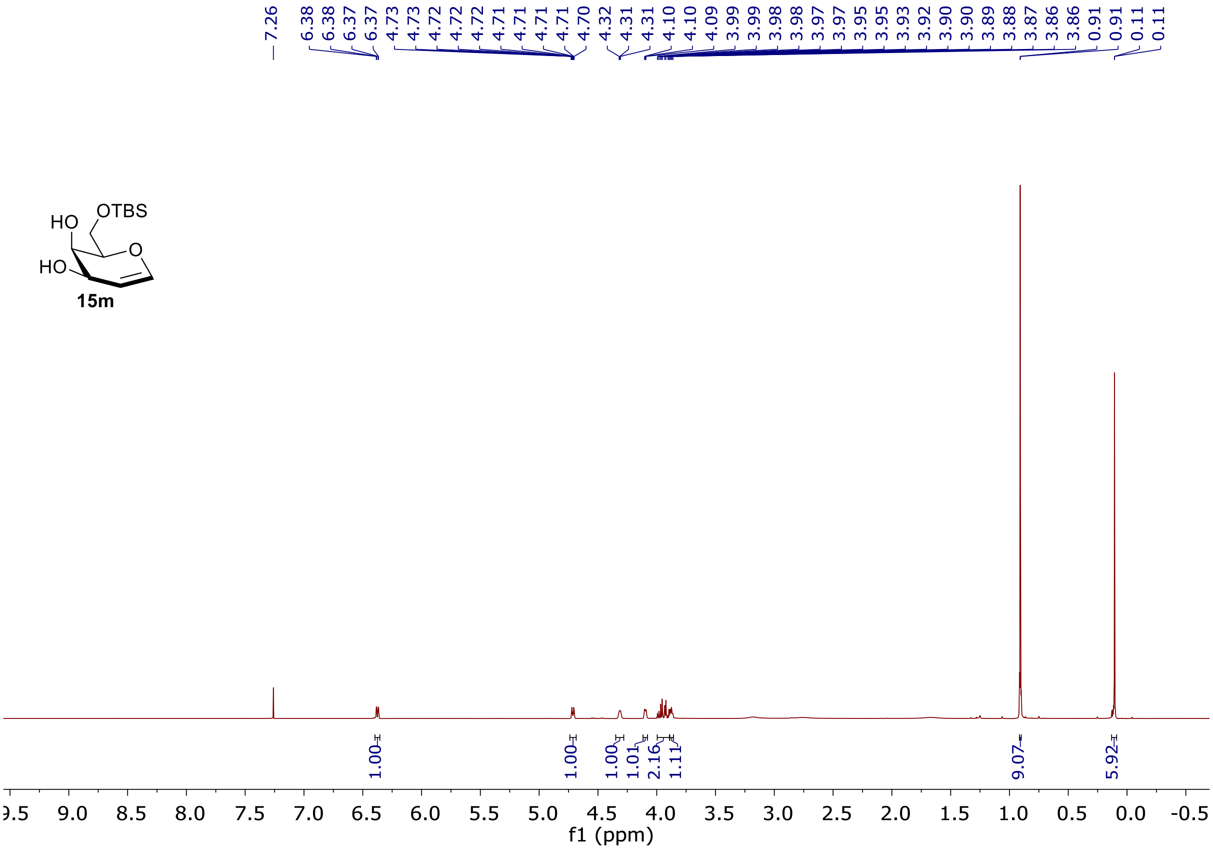

Supplementary Figure 58. ^{1}H spectra for **15m**

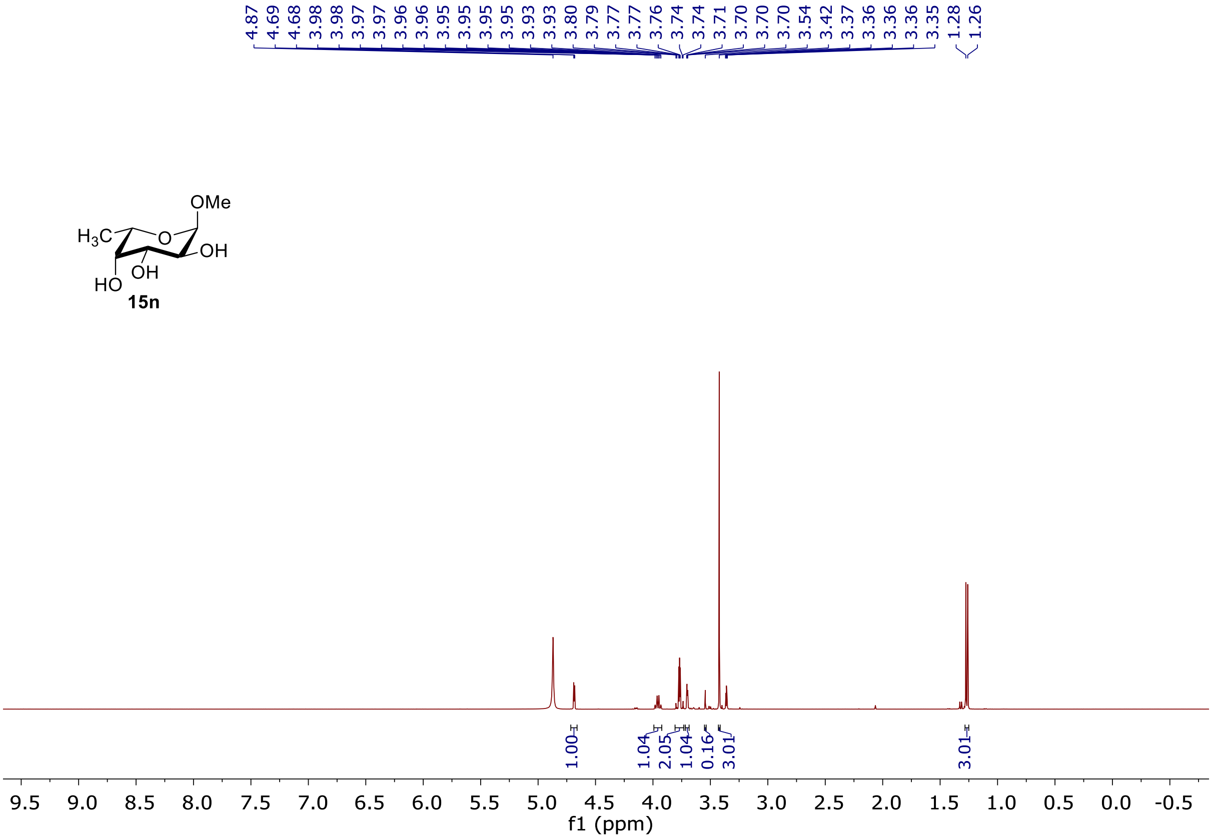

Supplementary Figure 59. ^{1}H spectra for **15n**

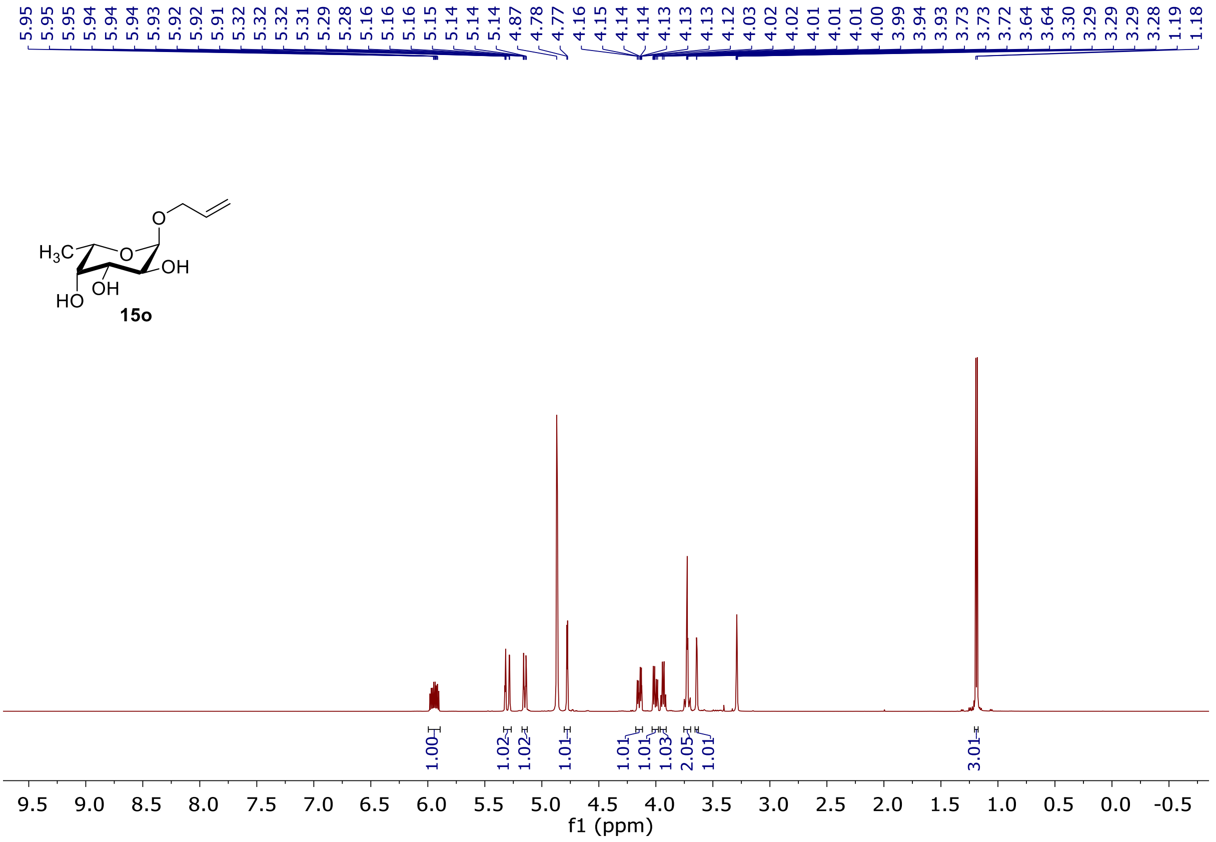

Supplementary Figure 60. ^{1}H spectra for **15o**

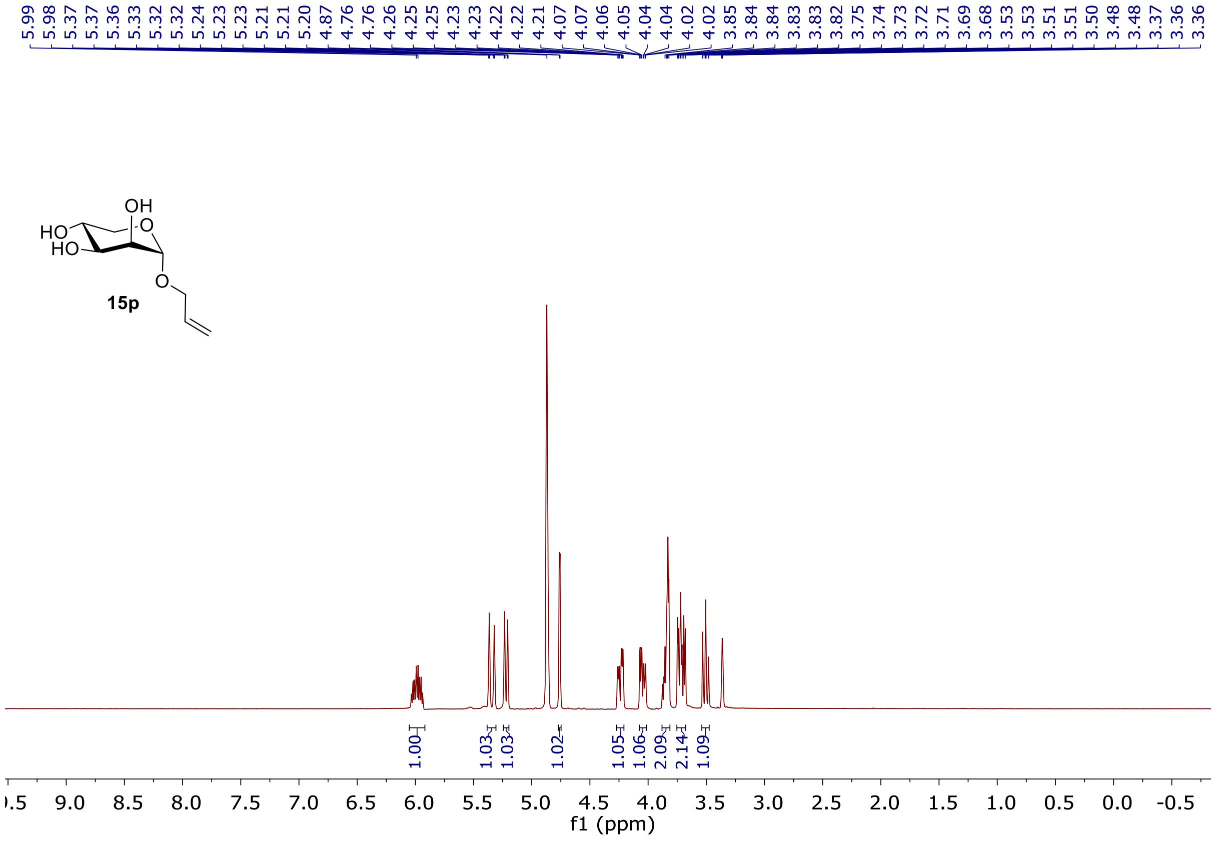

Supplementary Figure 61. ^{1}H spectra for **15p**

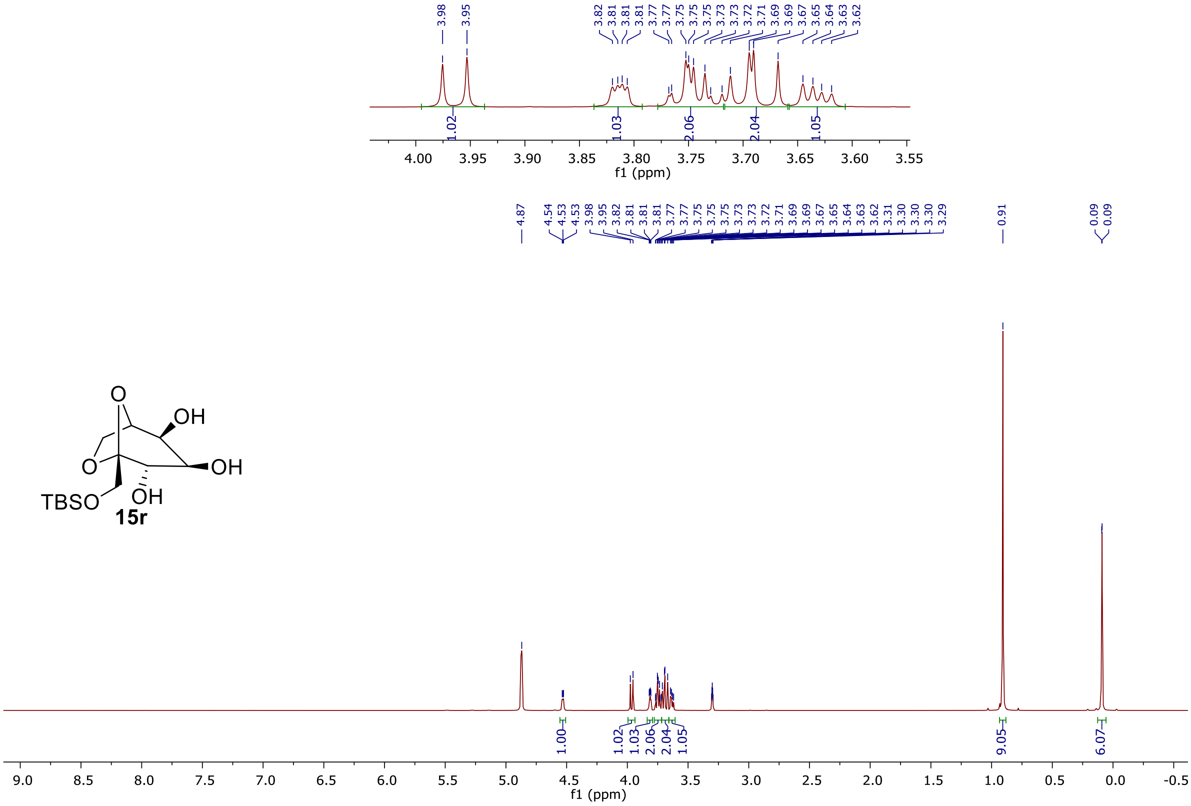

Supplementary Figure 62. ^{1}H spectra for **15r**

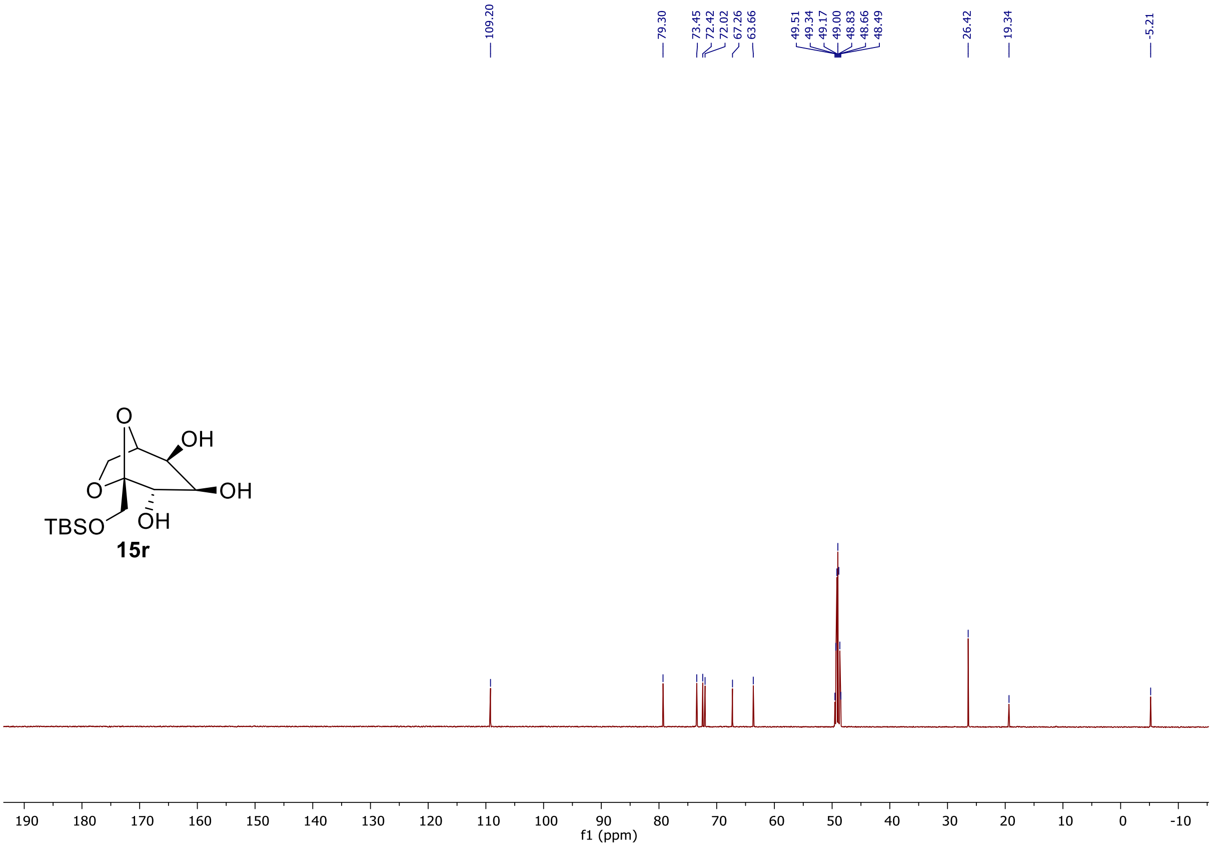

Supplementary Figure 63. ^{13}C spectra for **15r**

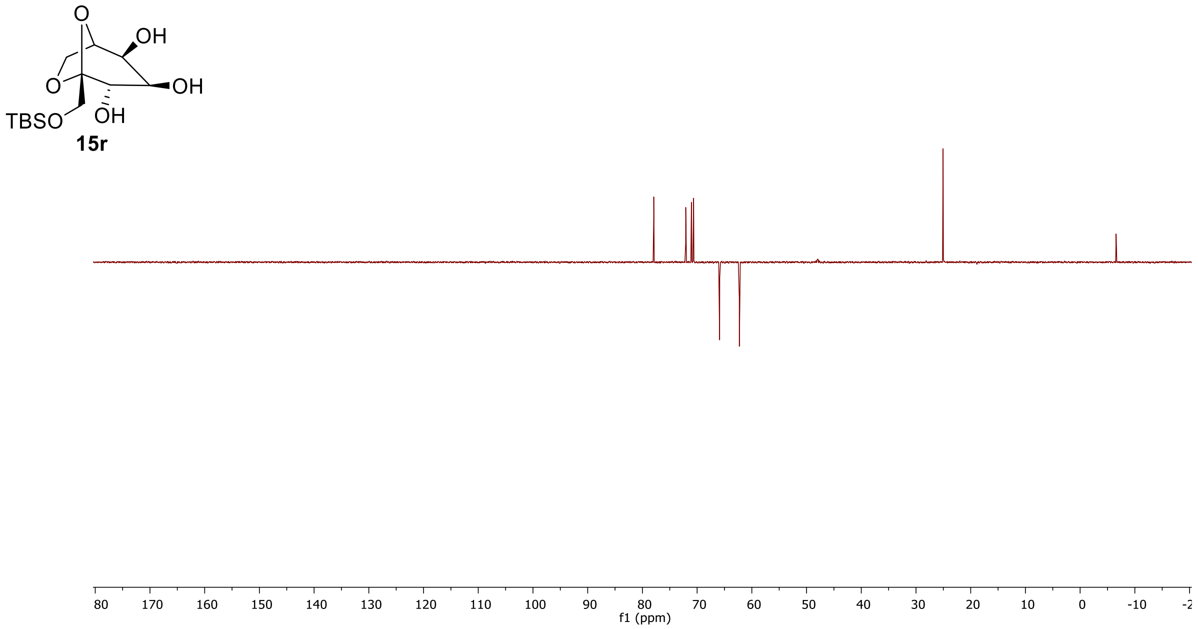

Supplementary Figure 64. DEPT spectra for **15r**

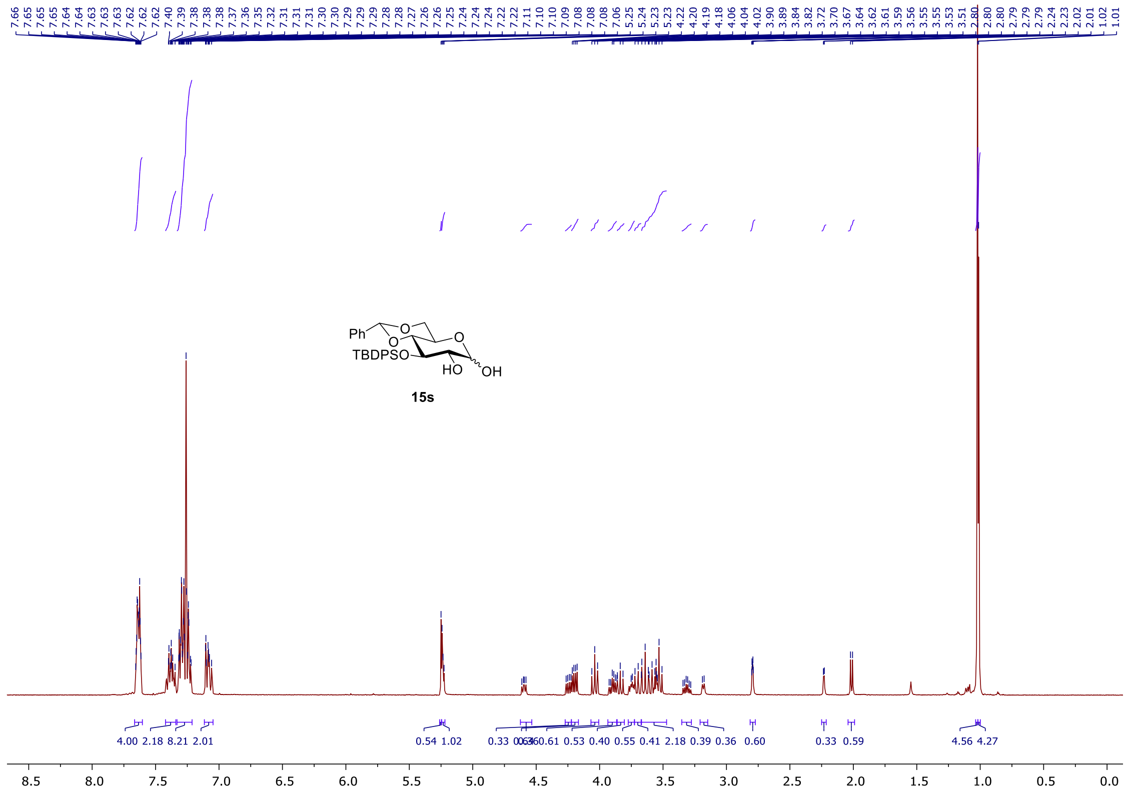

Supplementary Figure 65. ^{1}H spectra for **15s**

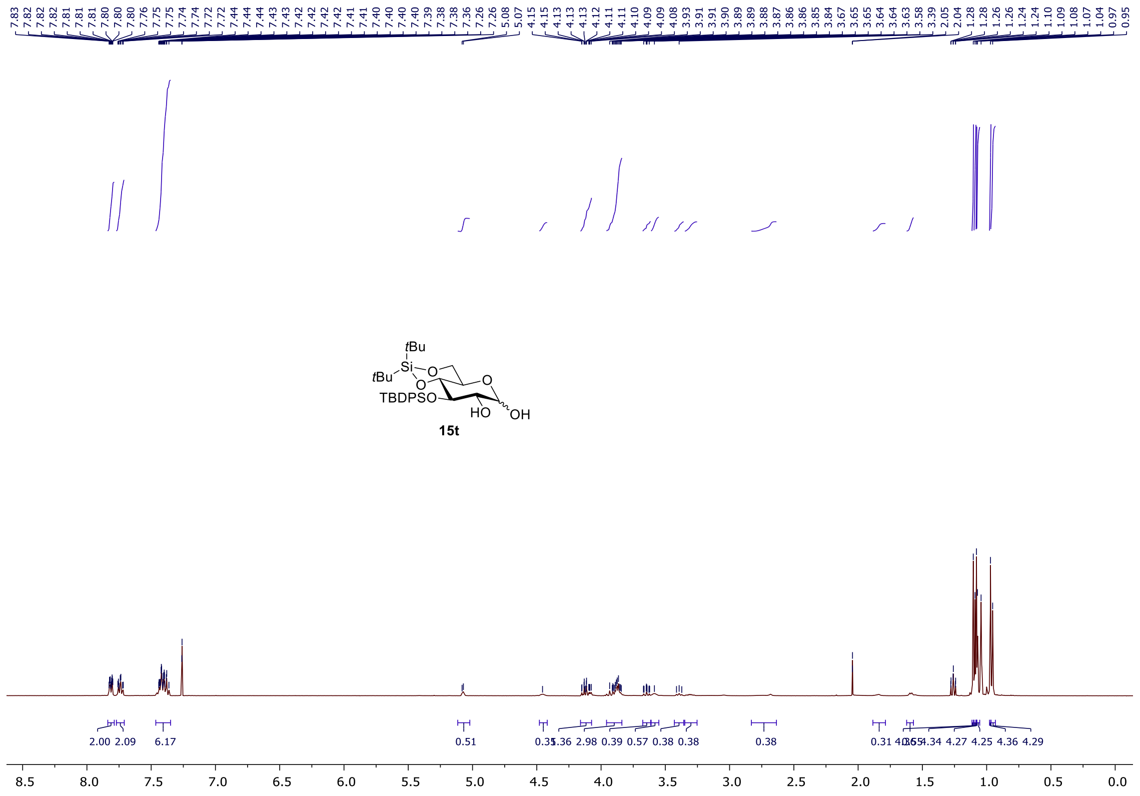

Supplementary Figure 66. ^{1}H spectra for **15t**

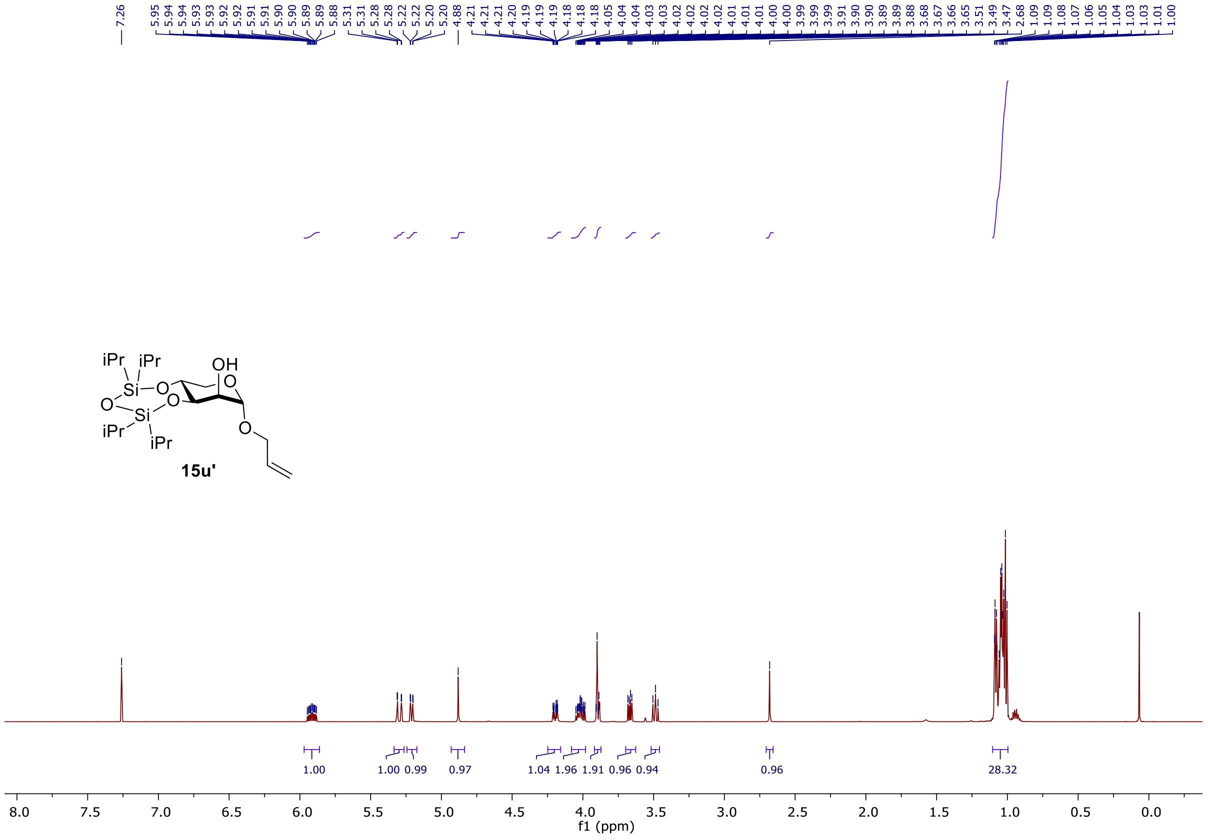

Supplementary Figure 67. ^{1}H spectra for **15u'**

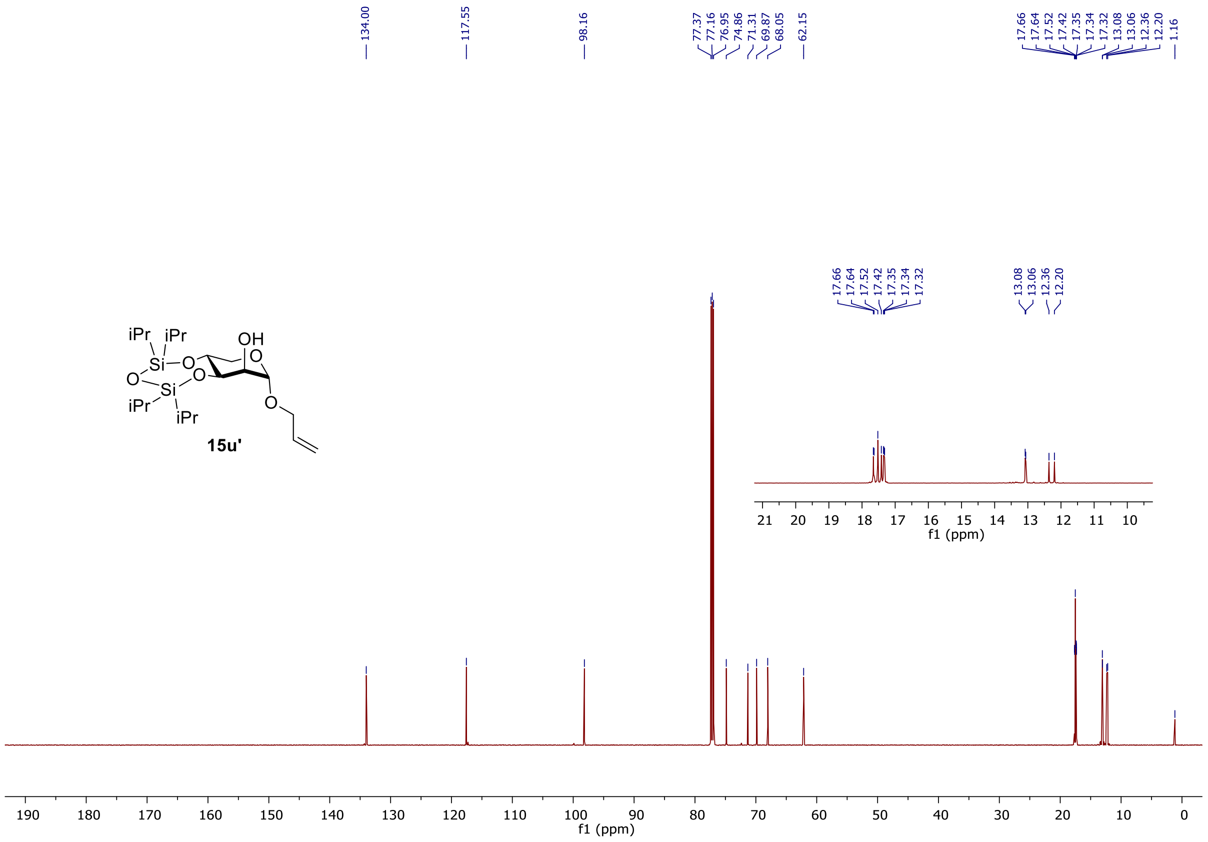

Supplementary Figure 68. ^{13}C spectra for **15u'**

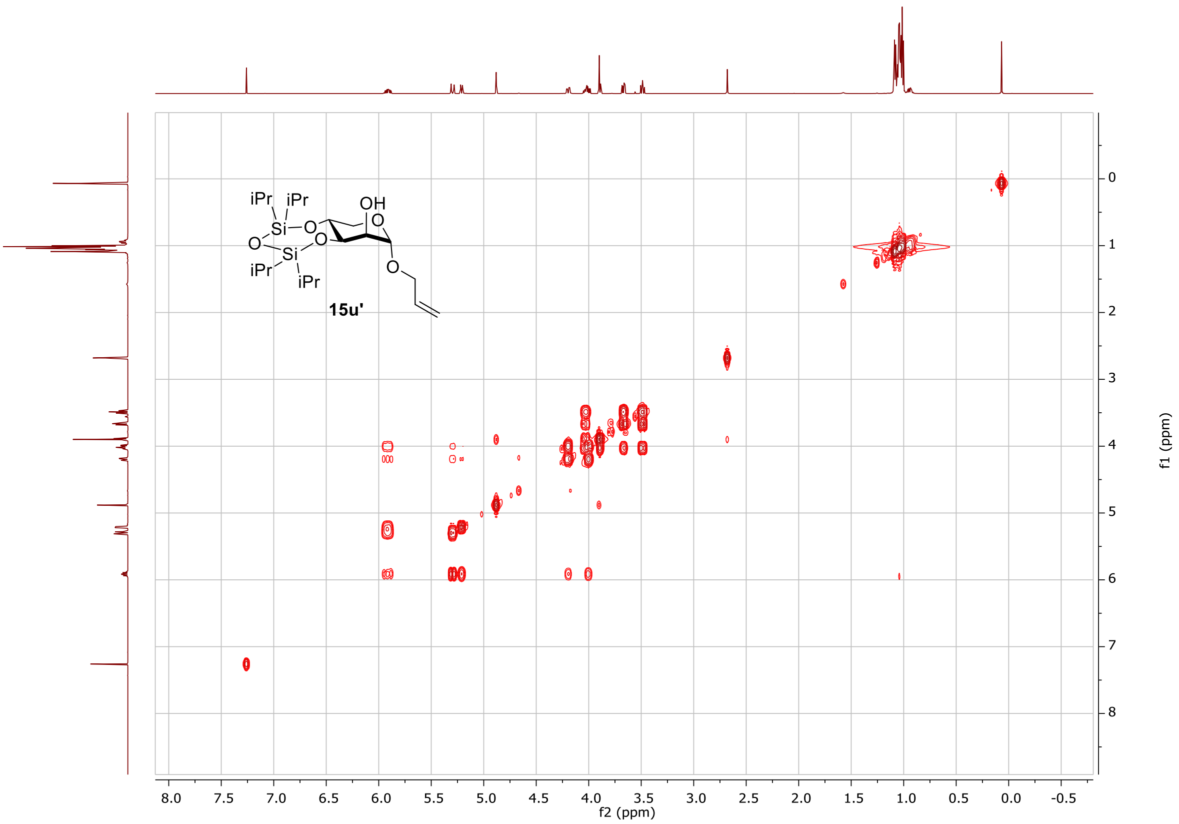

Supplementary Figure 69. COSY spectra for **15u'**

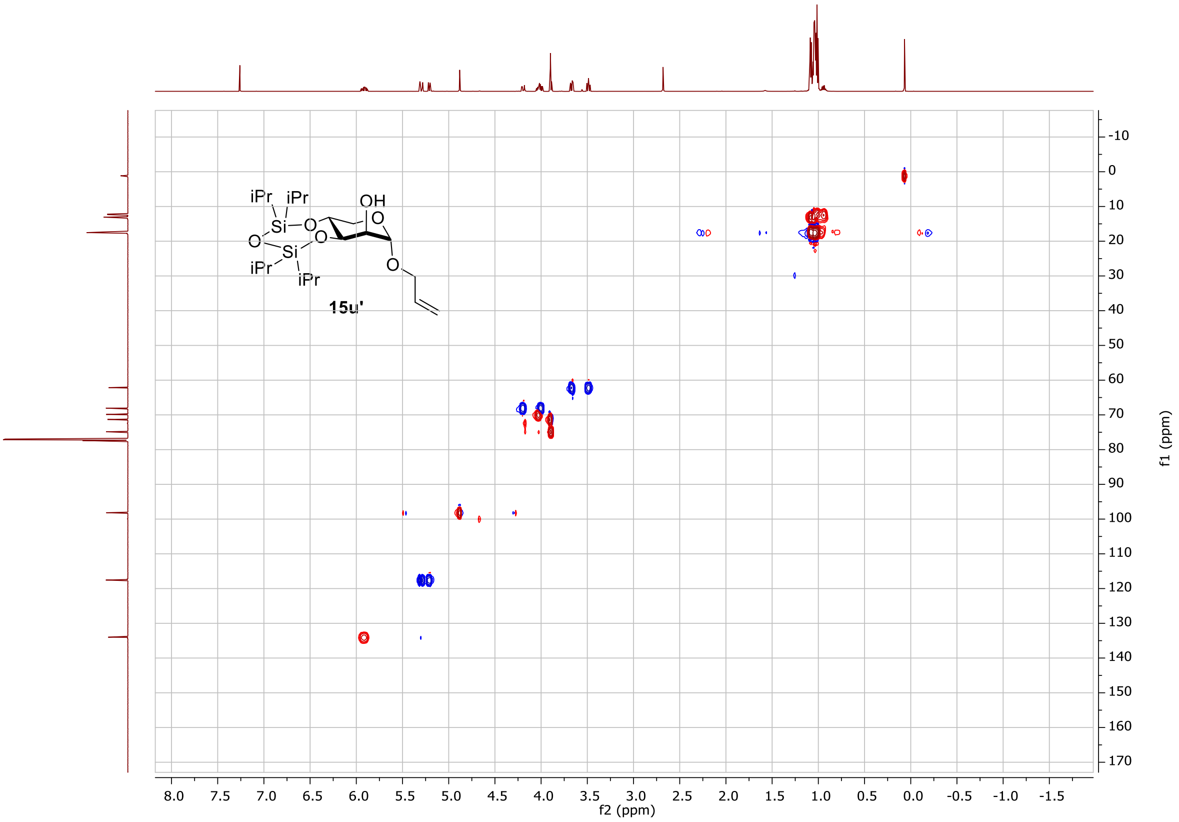

Supplementary Figure 70. HSQC spectra for **15u'**

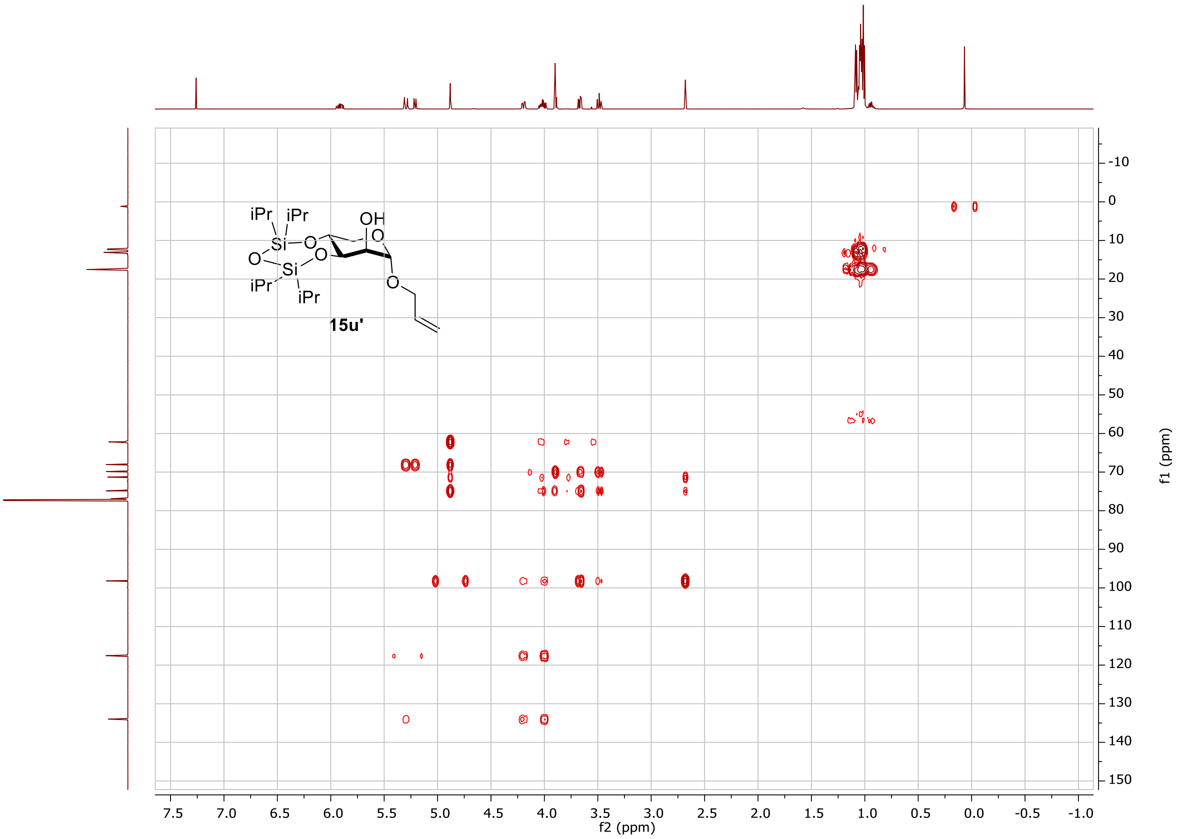

Supplementary Figure 71. HMBC spectra for **15u'**

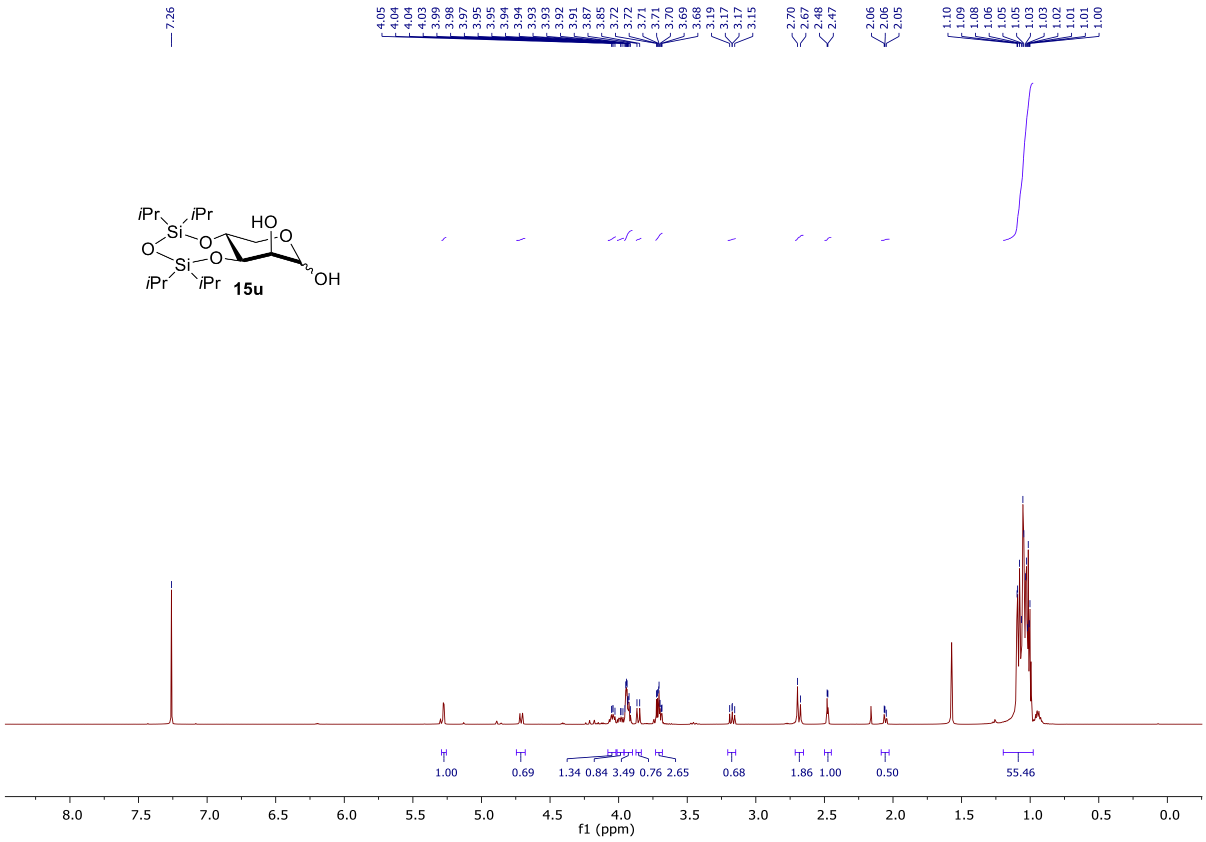

Supplementary Figure 72. ^{1}H spectra for **15u**

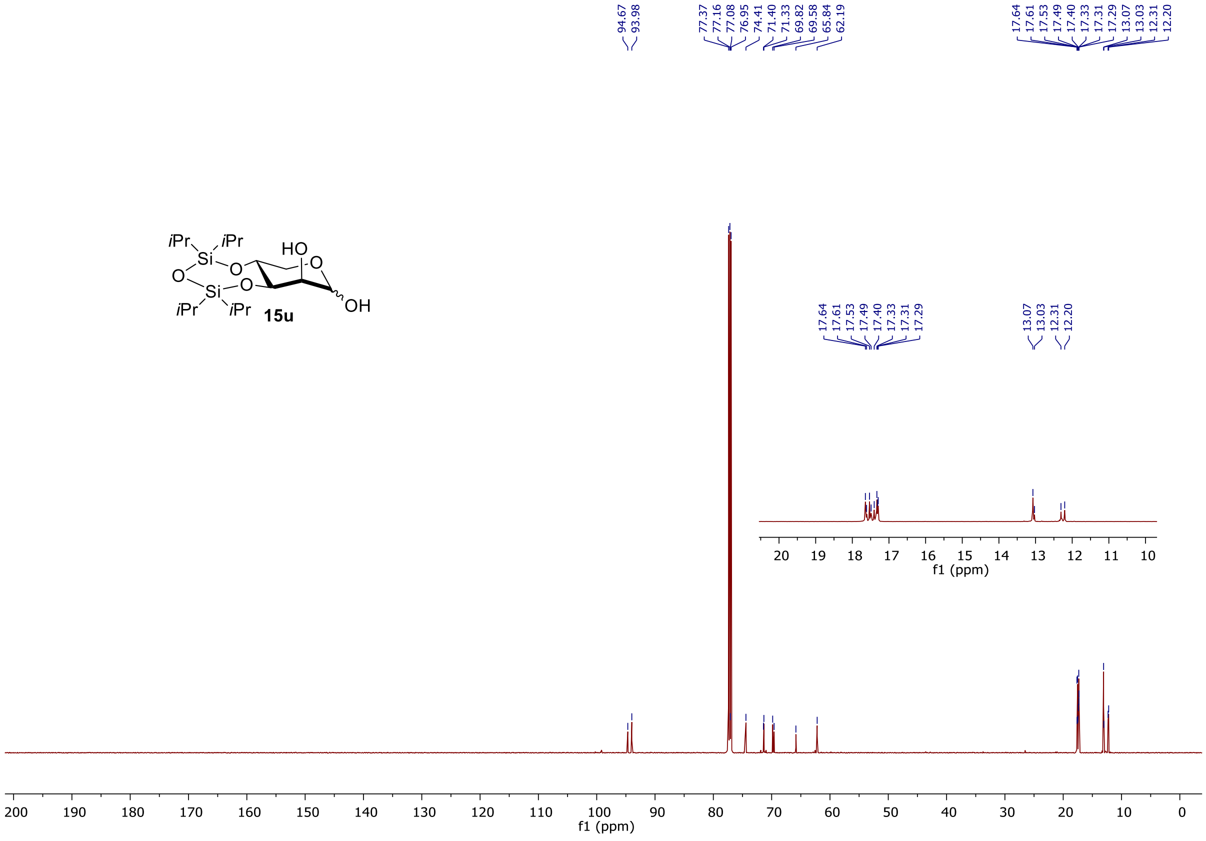

Supplementary Figure 73. ^{13}C spectra for **15u**

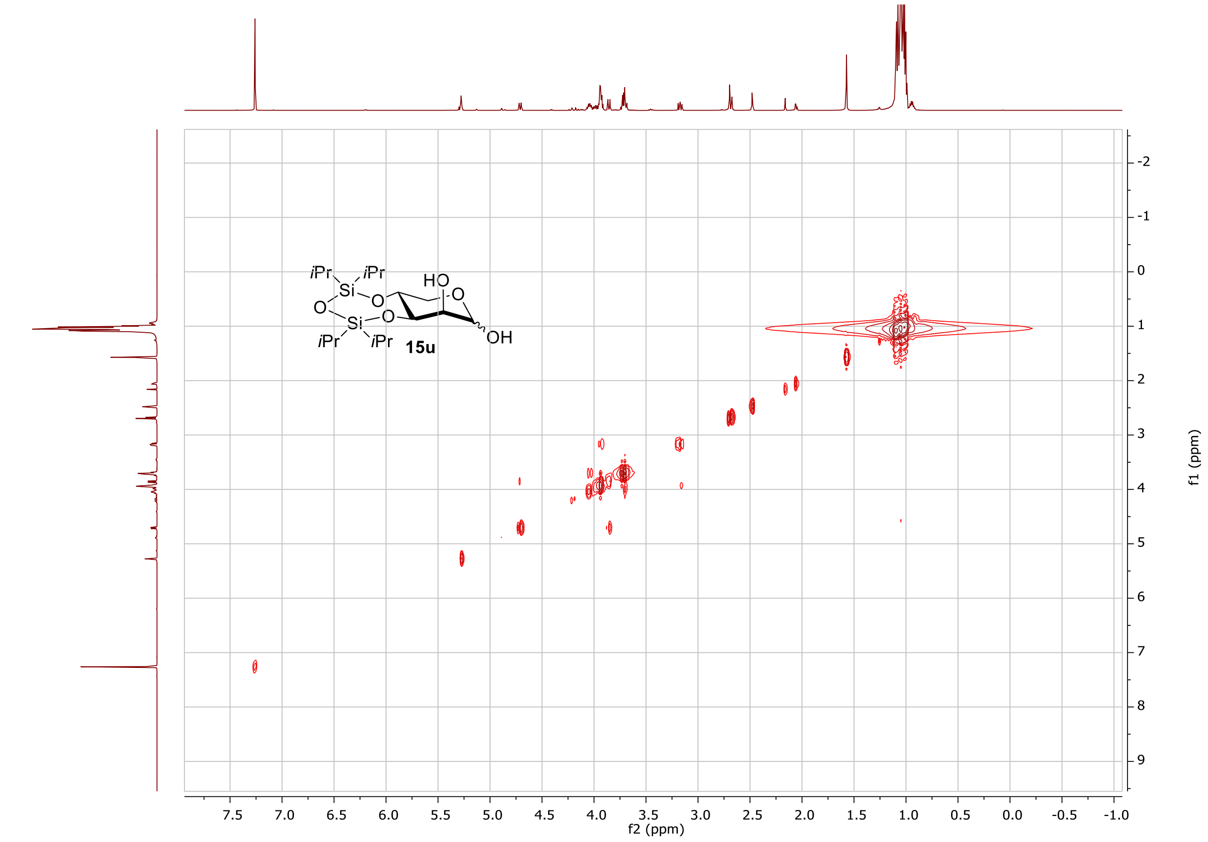

Supplementary Figure 74. COSY spectra for **15u**

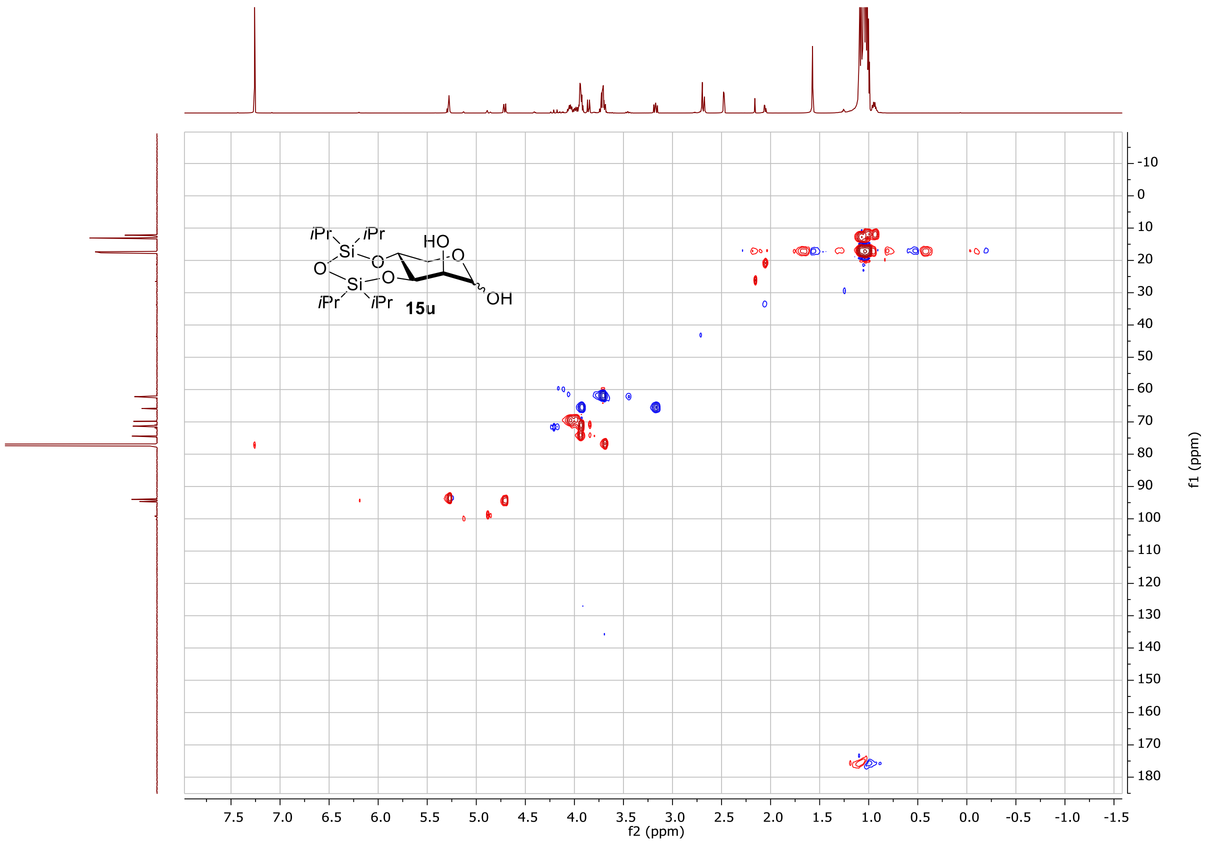

Supplementary Figure 75. HSQC spectra for **15u**

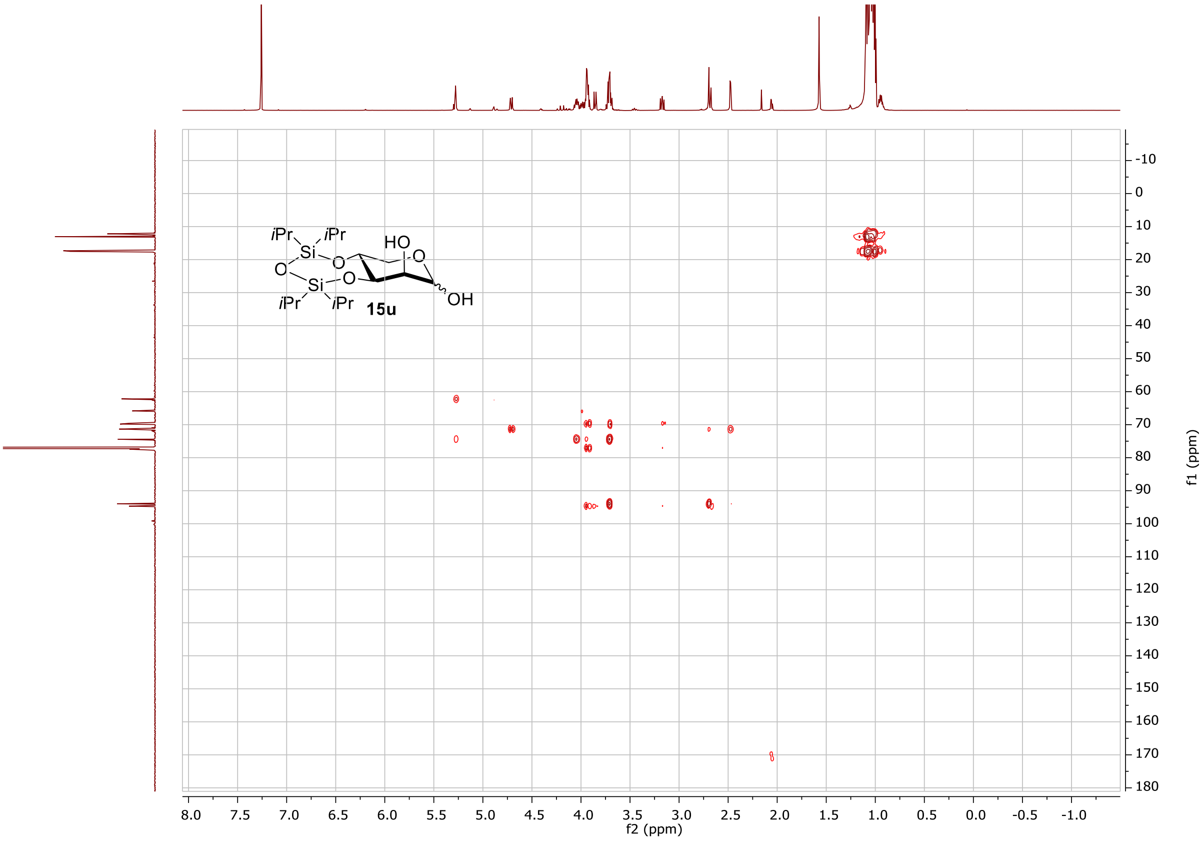

Supplementary Figure 76. HMBC spectra for **15u**

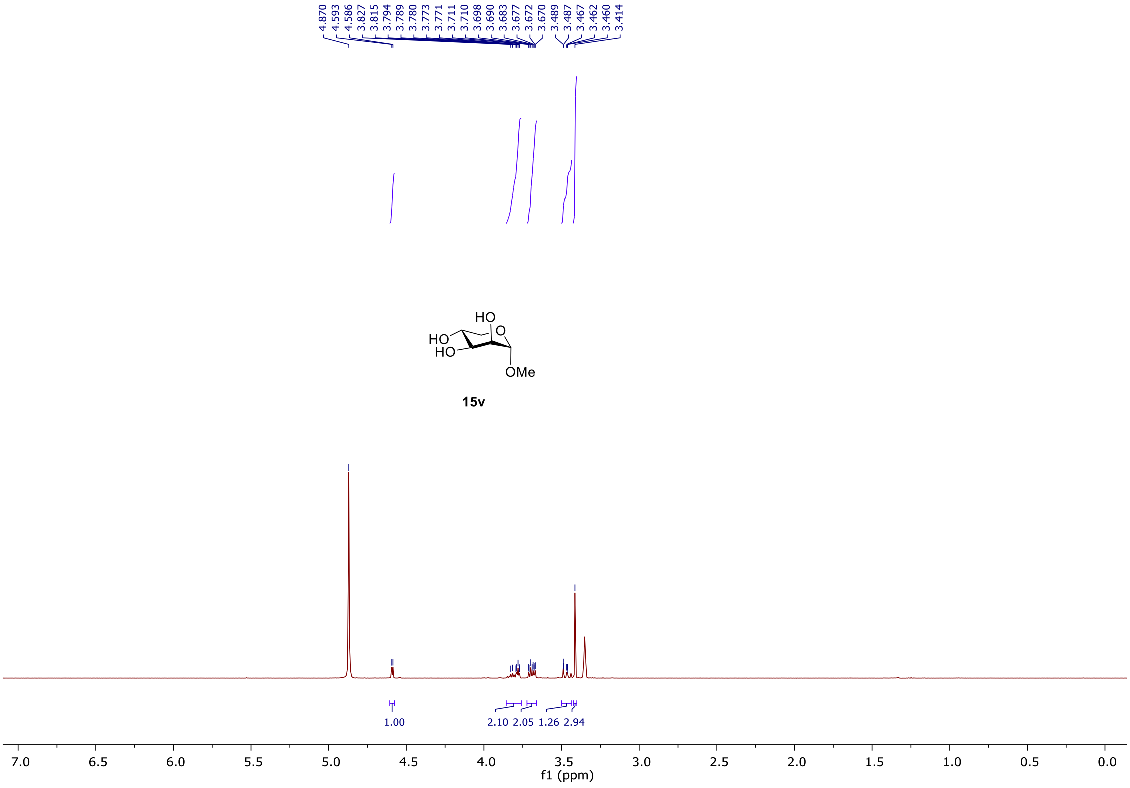

Supplementary Figure 77. ^{1}H spectra for **15v**

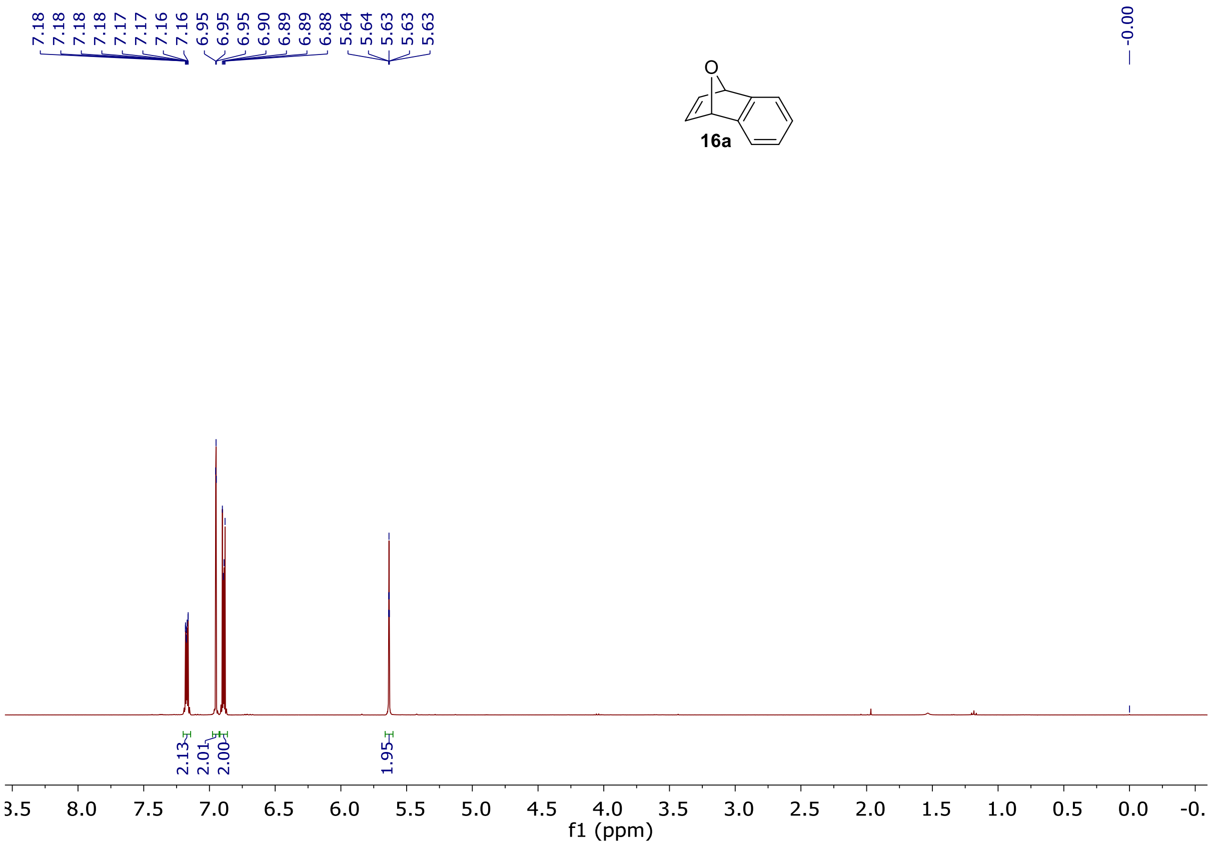

Supplementary Figure 78. ^{1}H spectra for **16a**

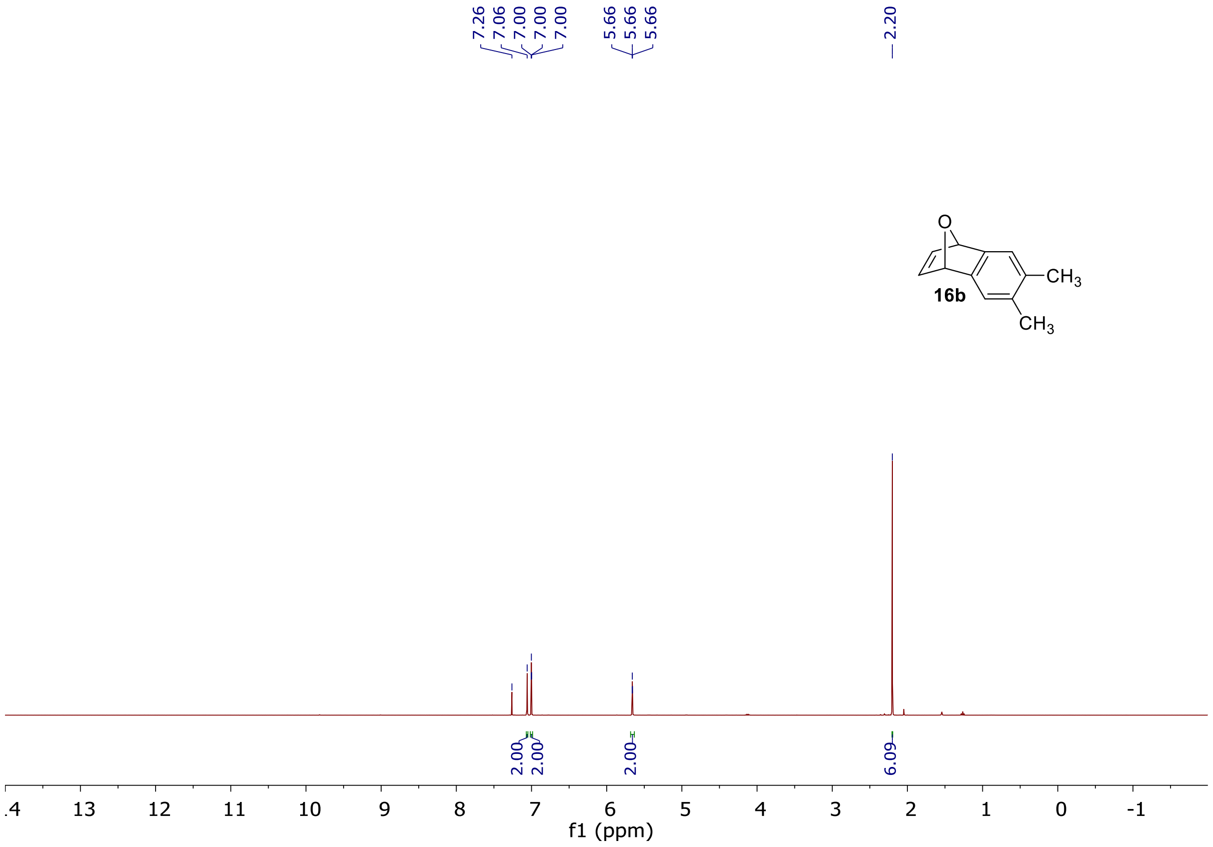

Supplementary Figure 79. ^{1}H spectra for **16b**

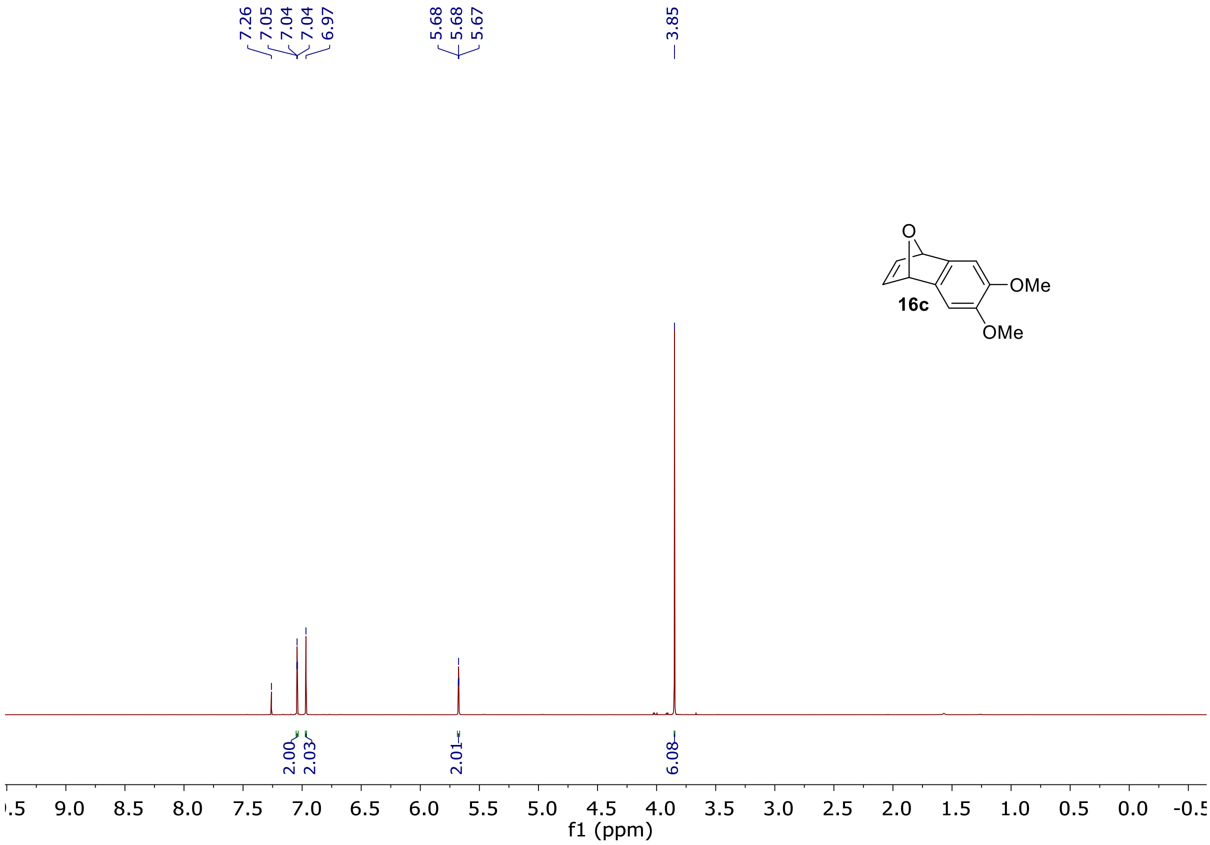

Supplementary Figure 80. ^{1}H spectra for **16c**

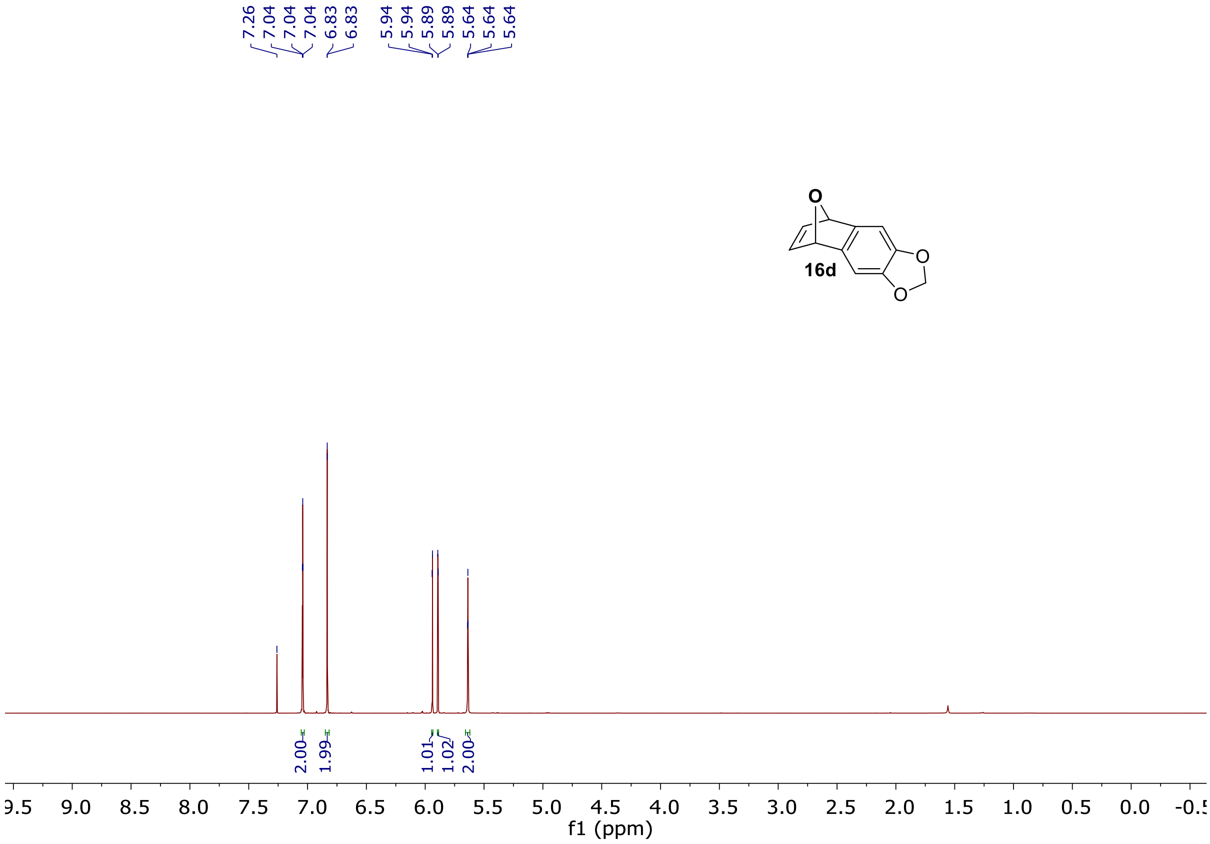

Supplementary Figure 81. ^{1}H spectra for **16d**

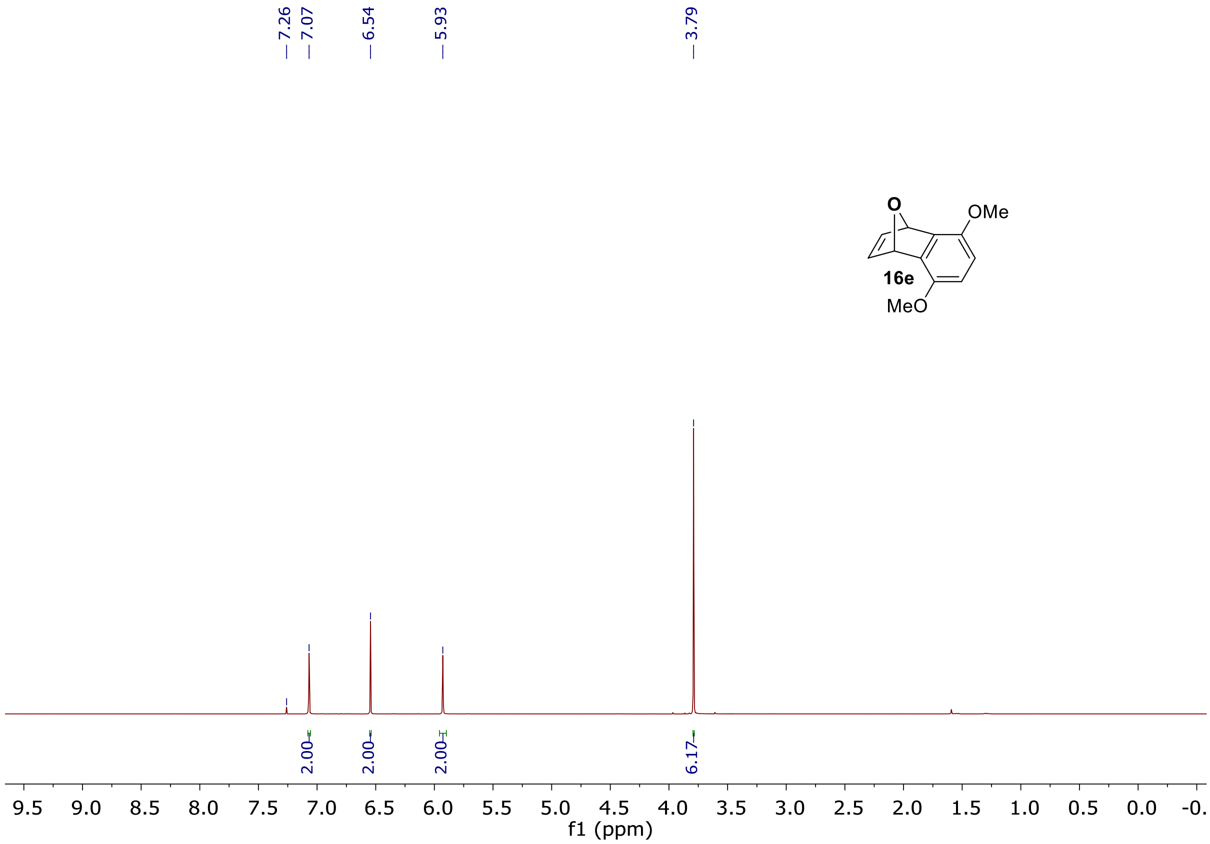

Supplementary Figure 82. ^{1}H spectra for **16e**

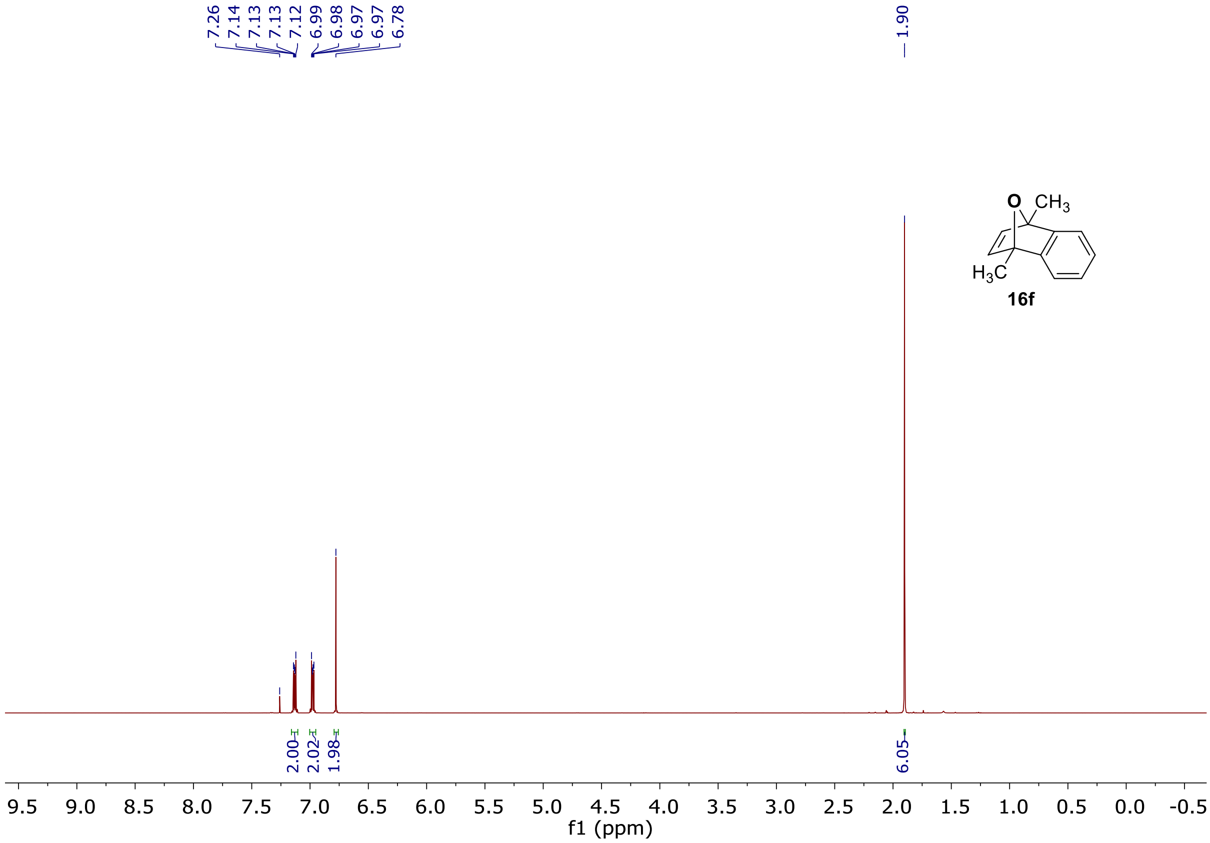

Supplementary Figure 83. ^{1}H spectra for **16f**

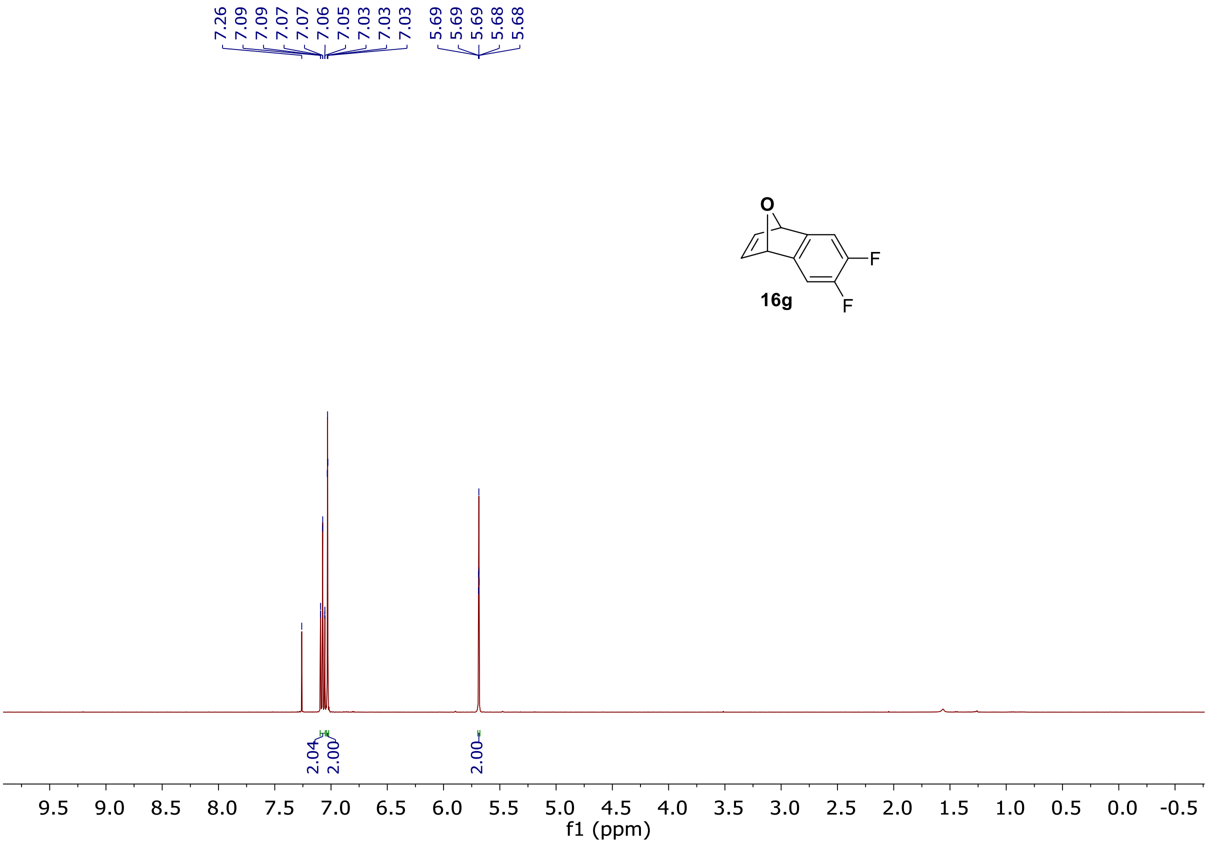

Supplementary Figure 84. ^{1}H spectra for **16g**

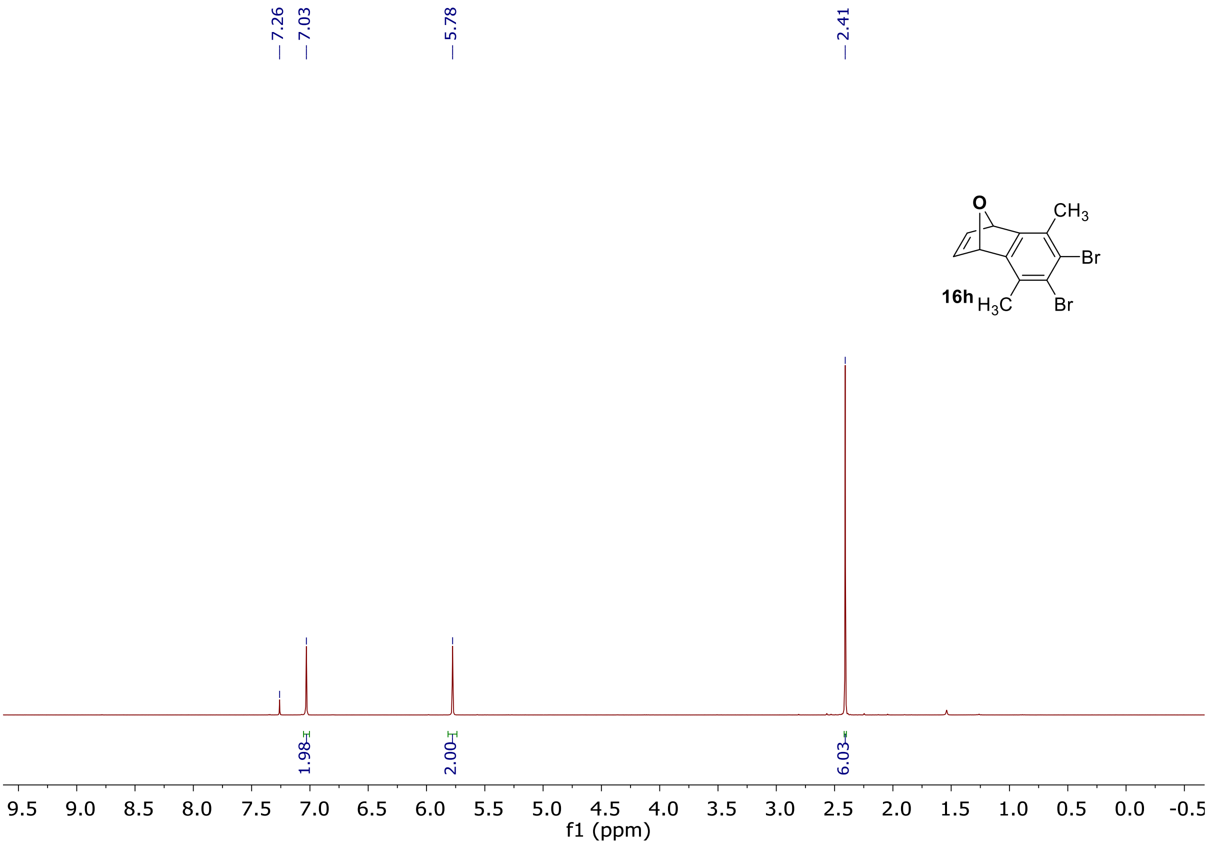

Supplementary Figure 85. ^{1}H spectra for **16h**

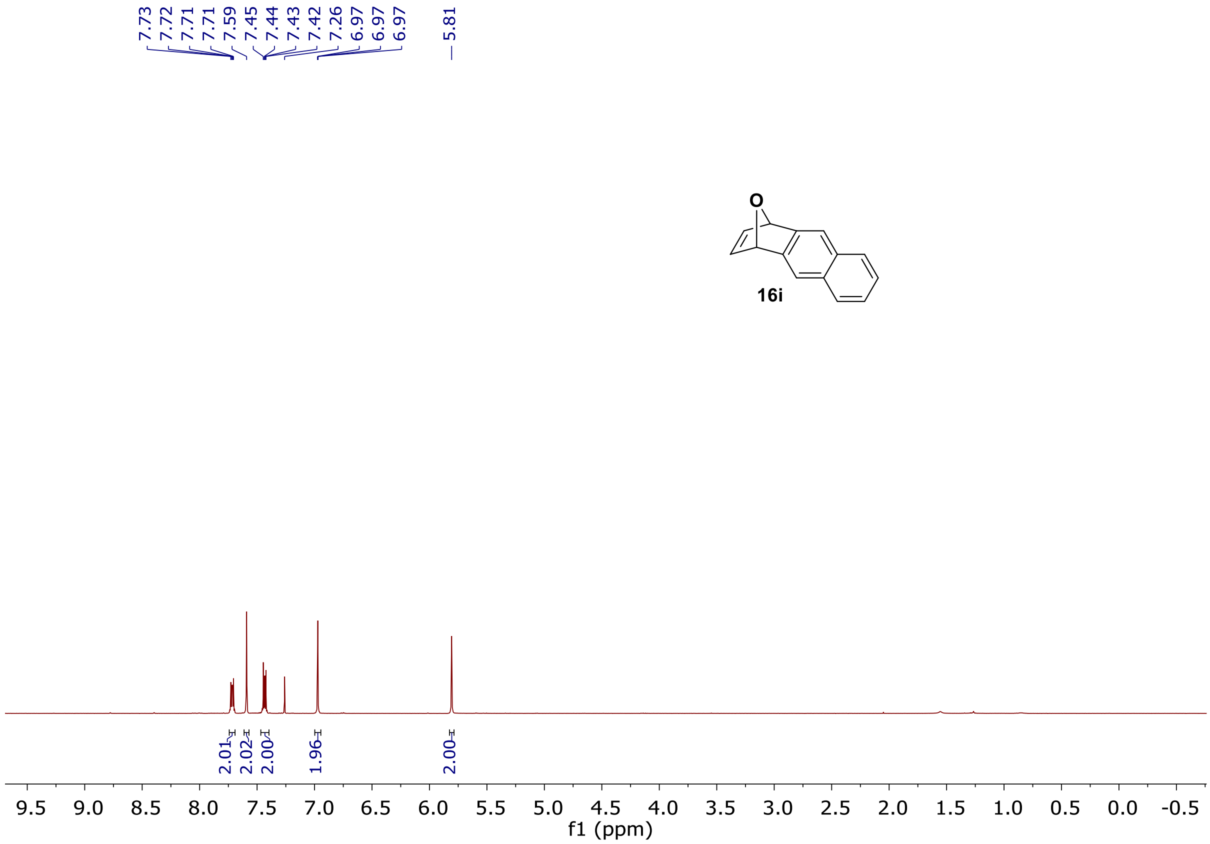

Supplementary Figure 86. ^{1}H spectra for **16i**

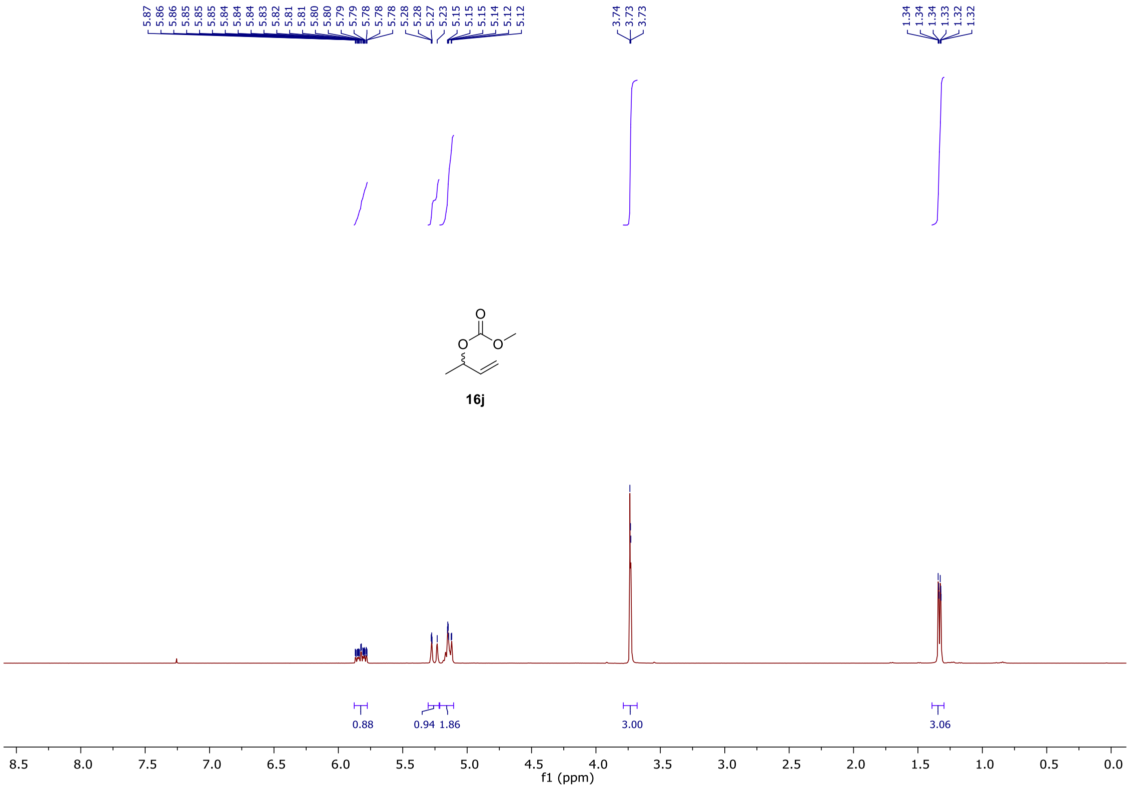

Supplementary Figure 87. ^{1}H spectra for **16j**

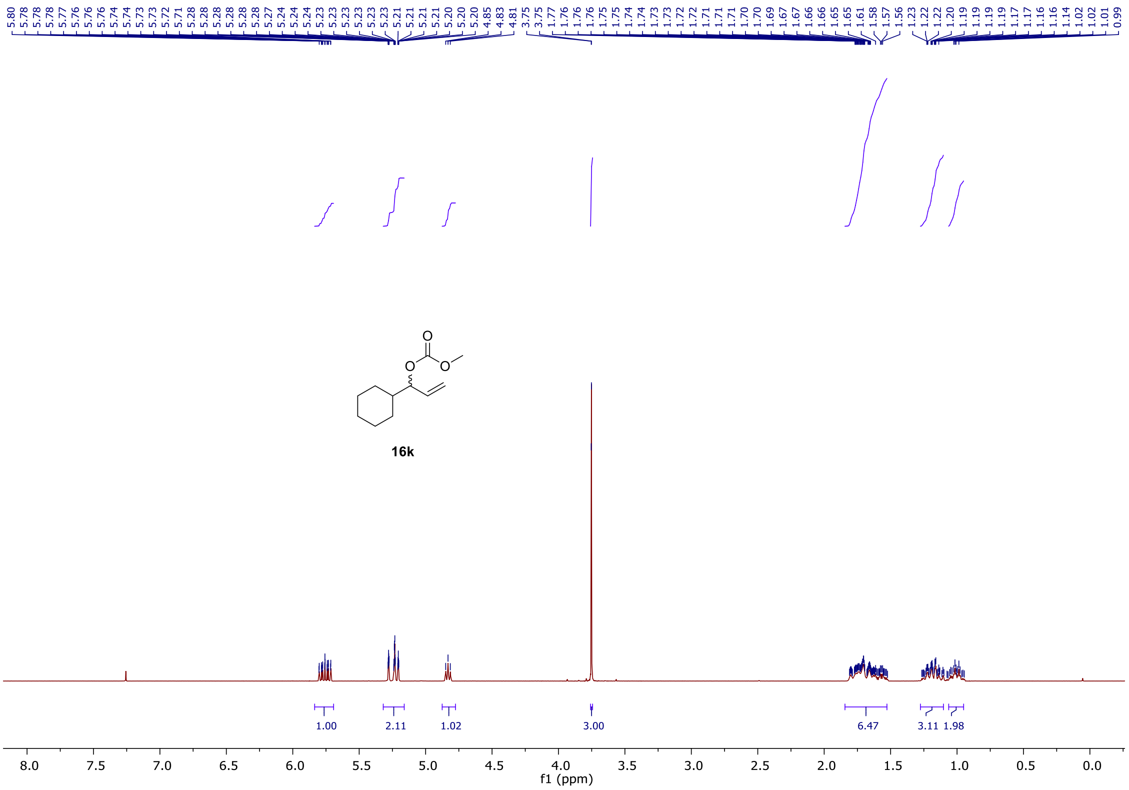

Supplementary Figure 88. ^{1}H spectra for **16k**

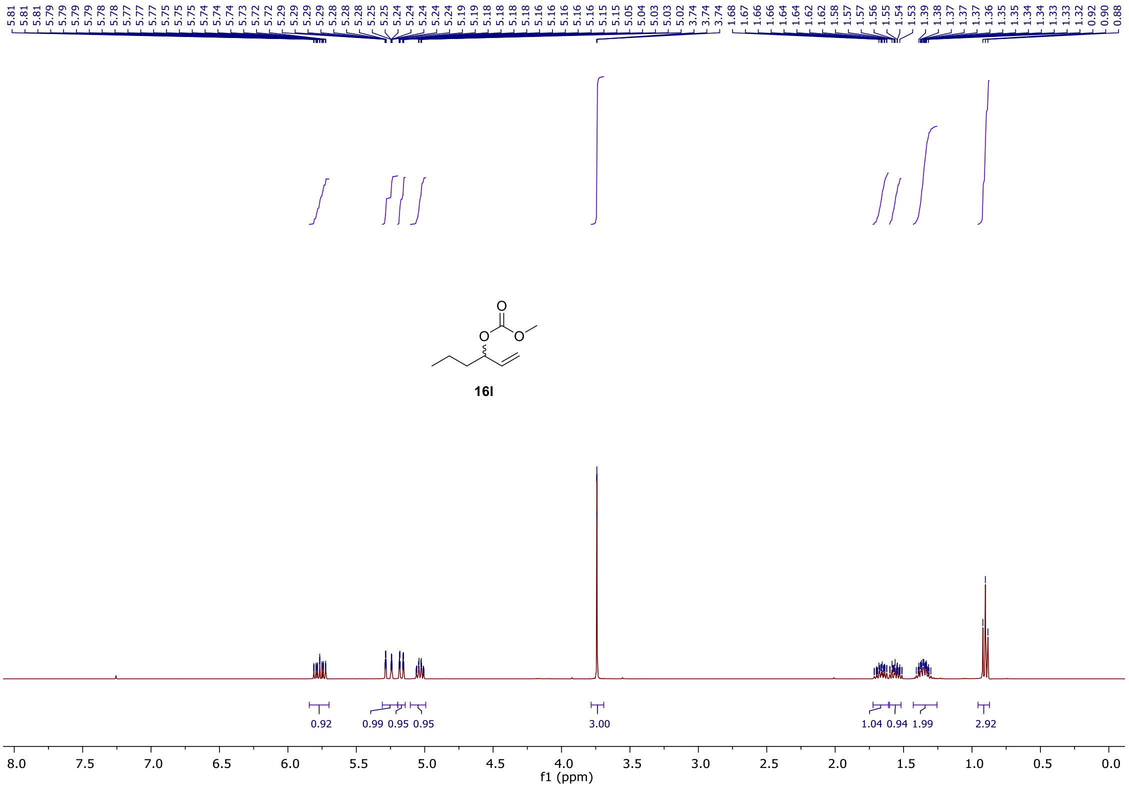

Supplementary Figure 89. ^{1}H spectra for **16l**

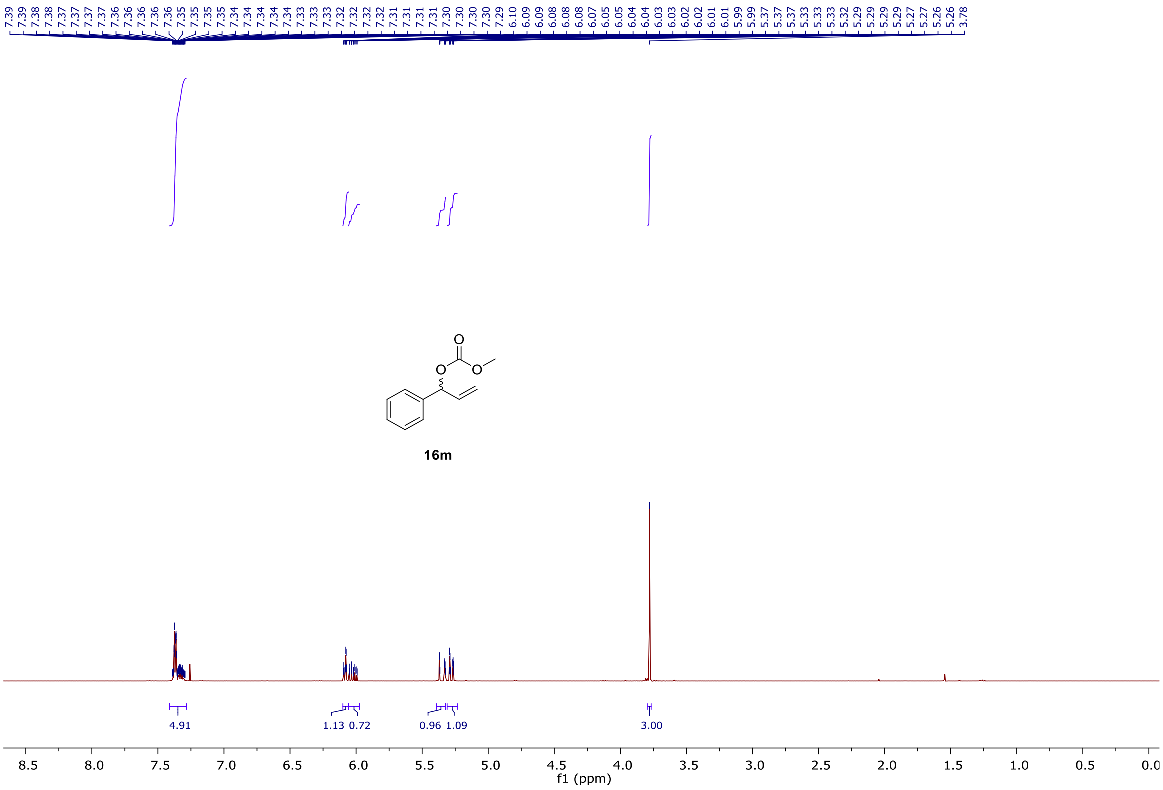

Supplementary Figure 90. ^{1}H spectra for **16m**

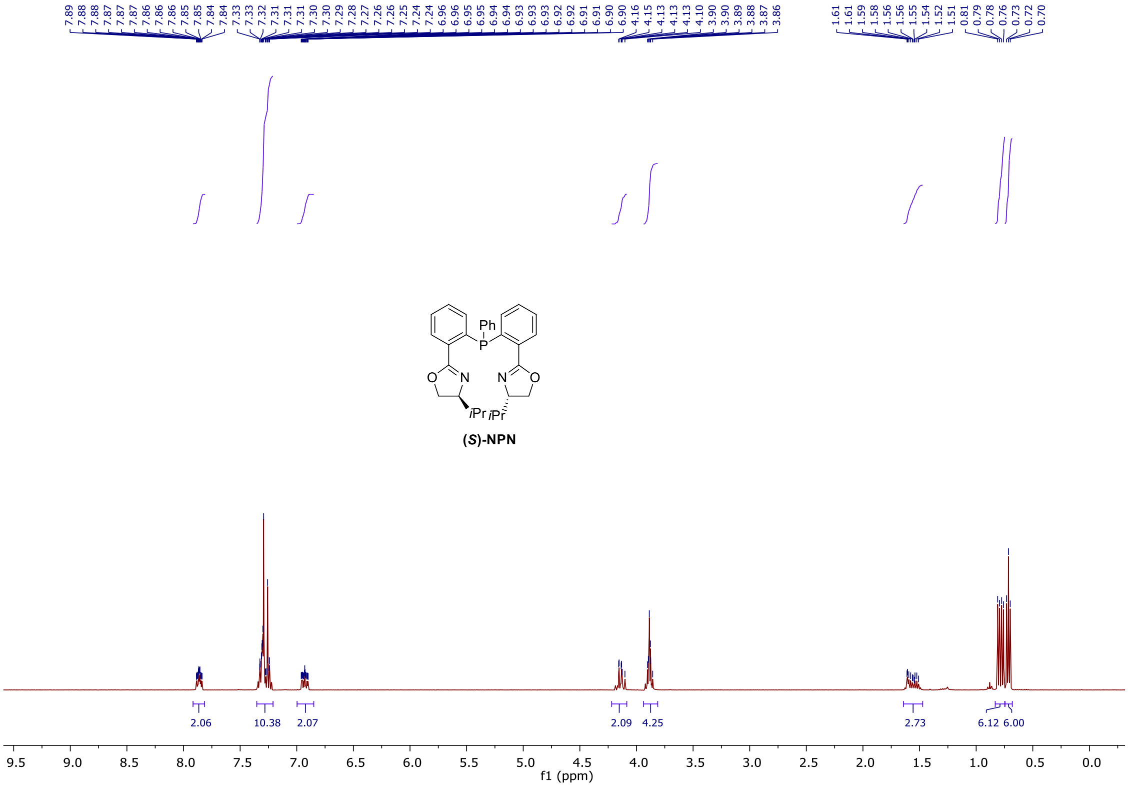

Supplementary Figure 91. ^{1}H spectra for **(*S*)-NPN**

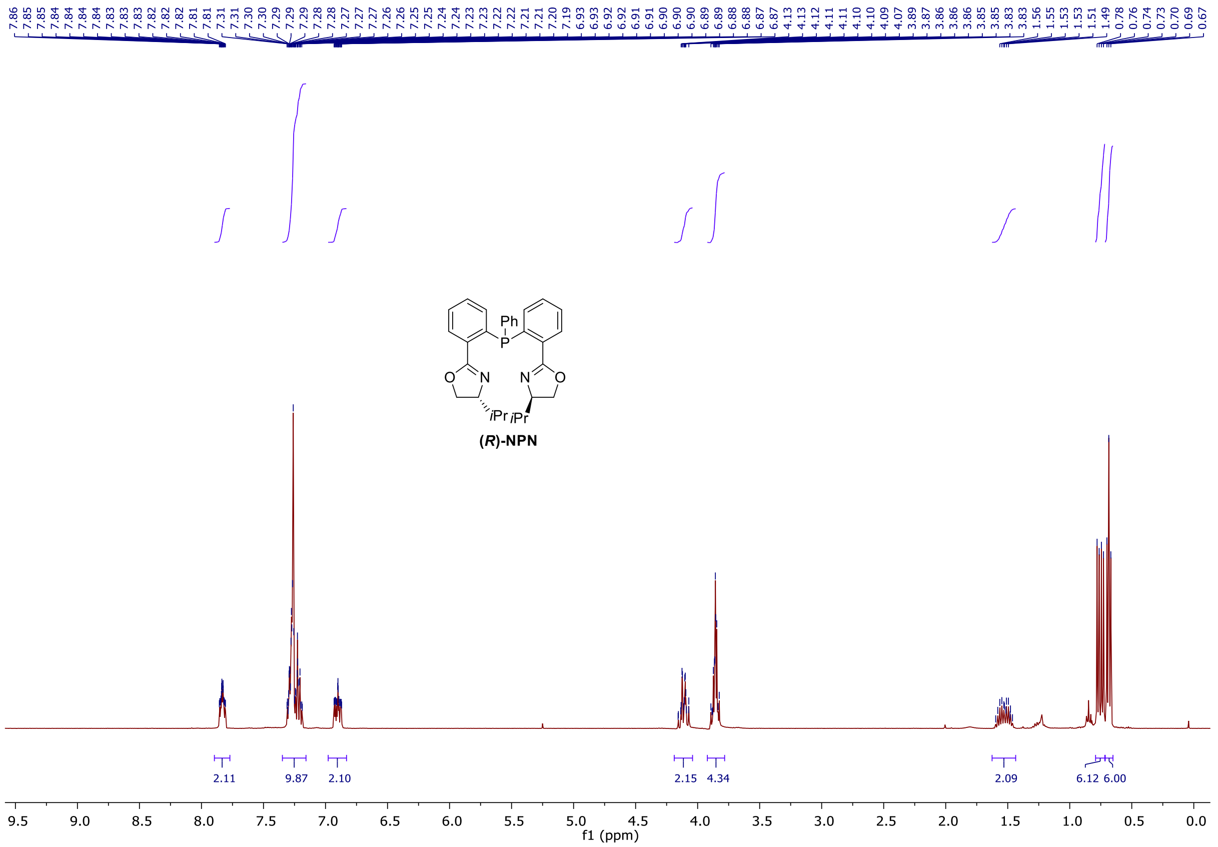

Supplementary Figure 92. ¹H spectra for **(*R*)-NPN**

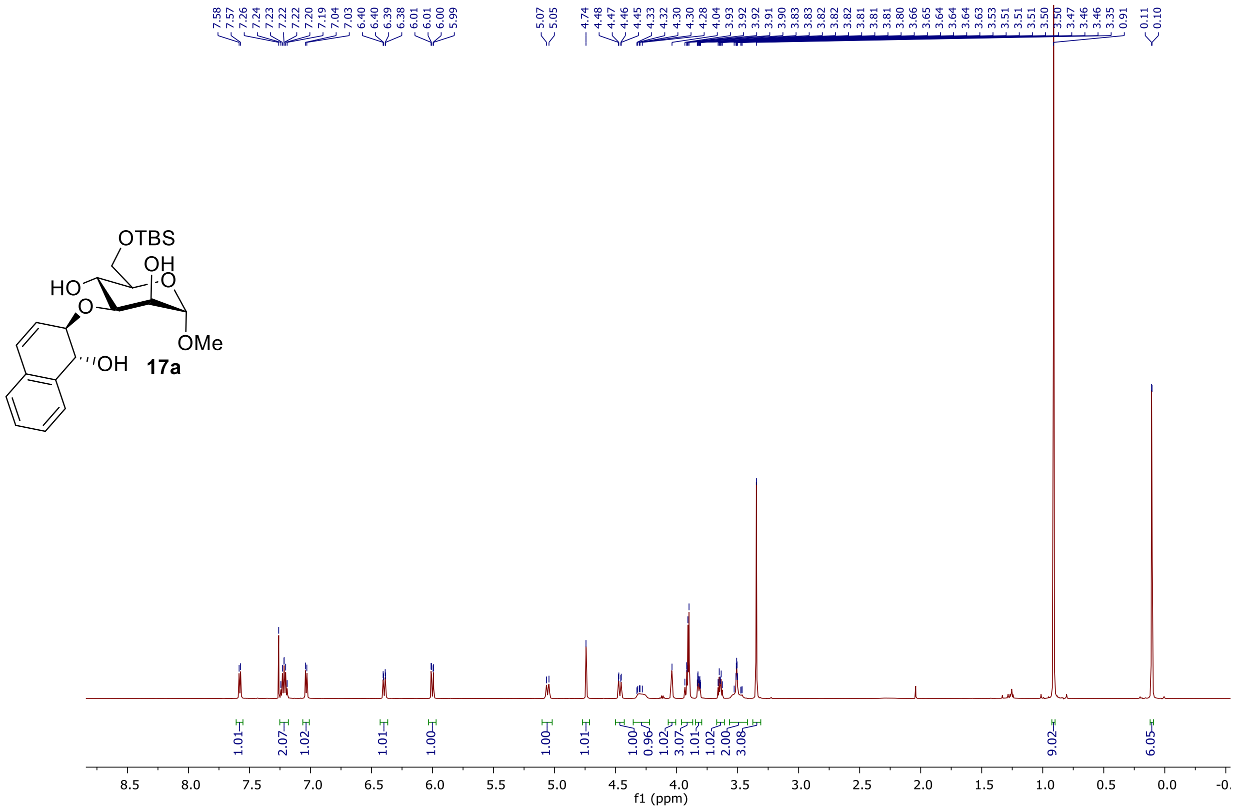

Supplementary Figure 93. ¹H spectra for **17a**

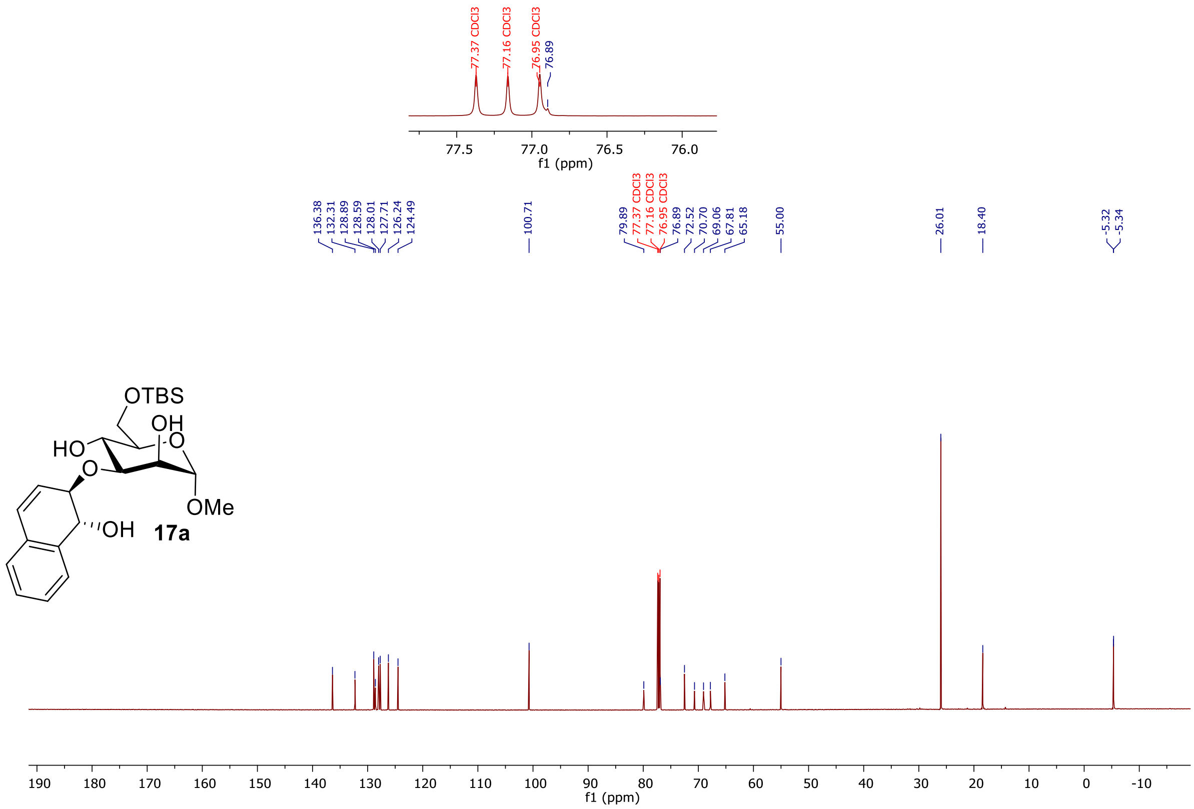

Supplementary Figure 94. ^{13}C spectra for **17a**

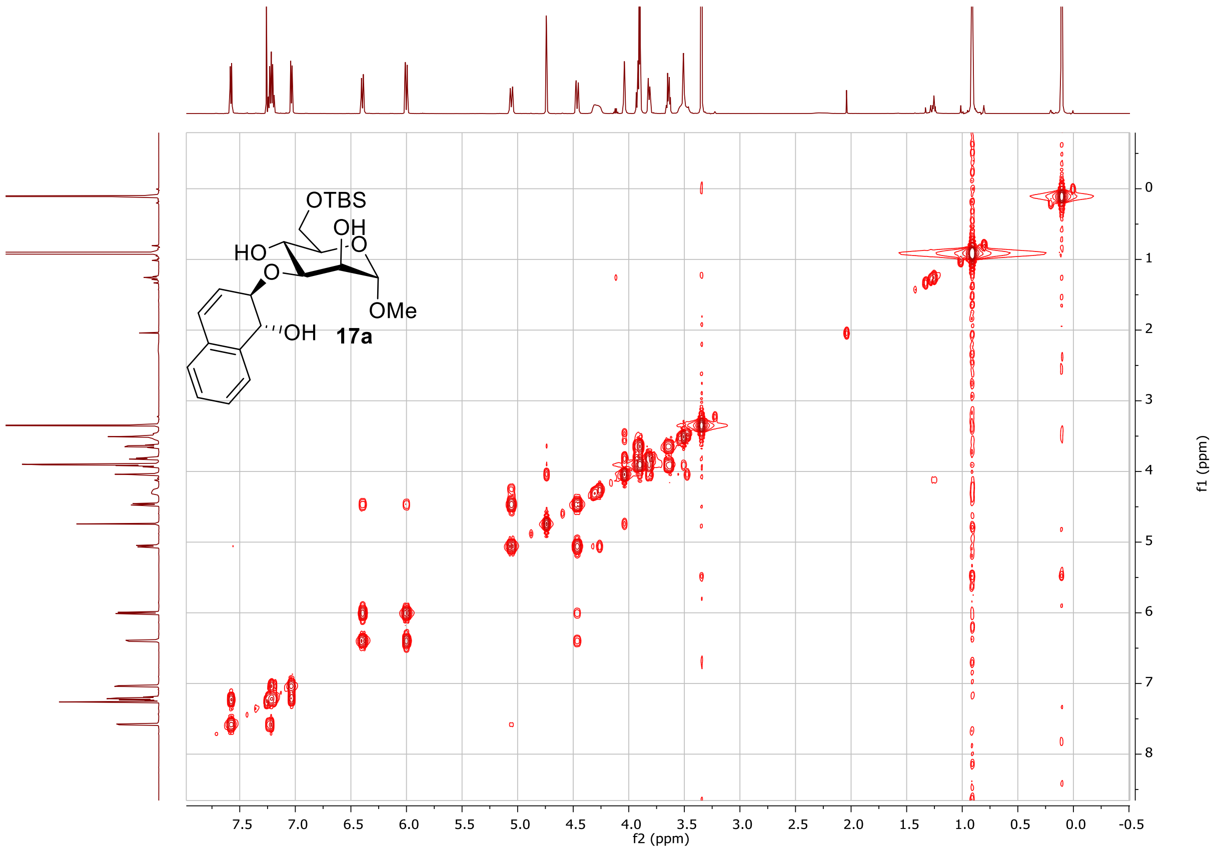

Supplementary Figure 95. COSY spectra for **17a**

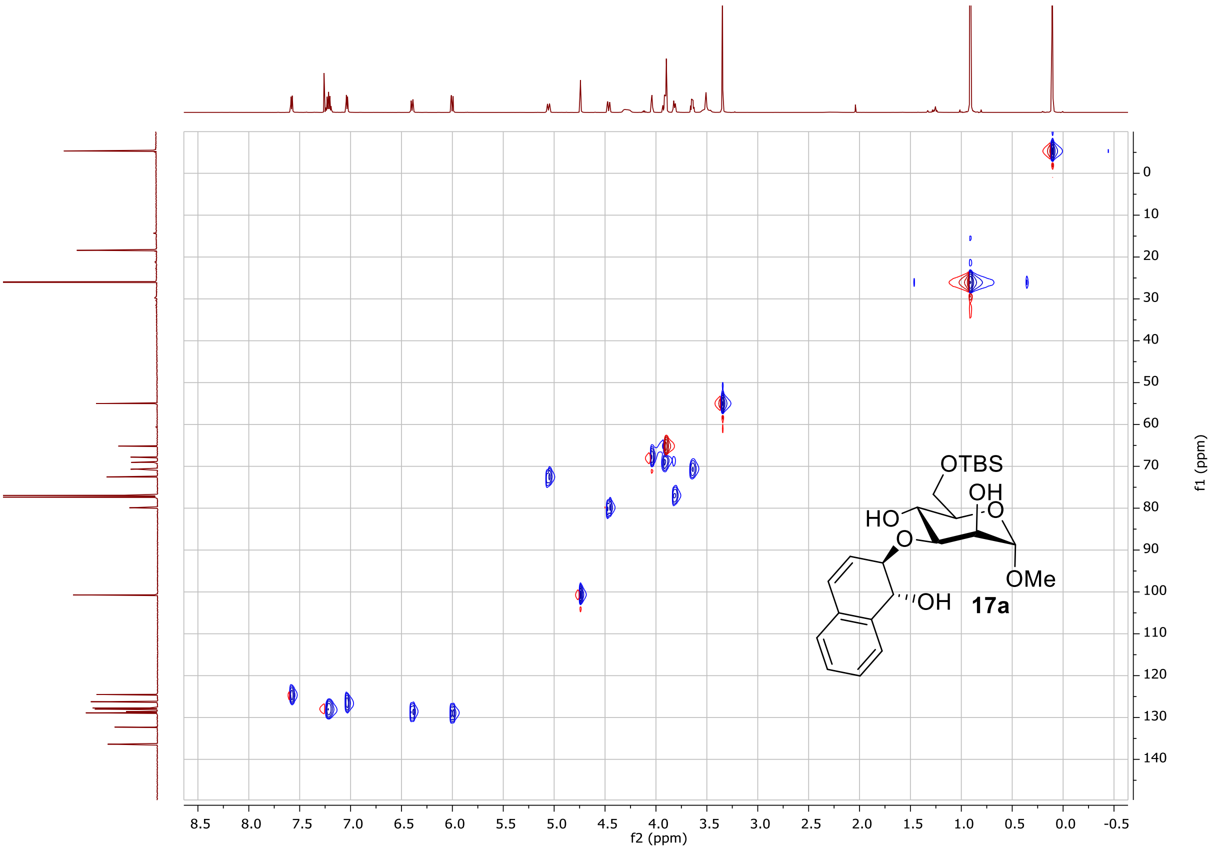

Supplementary Figure 96. HSQC spectra for **17a**

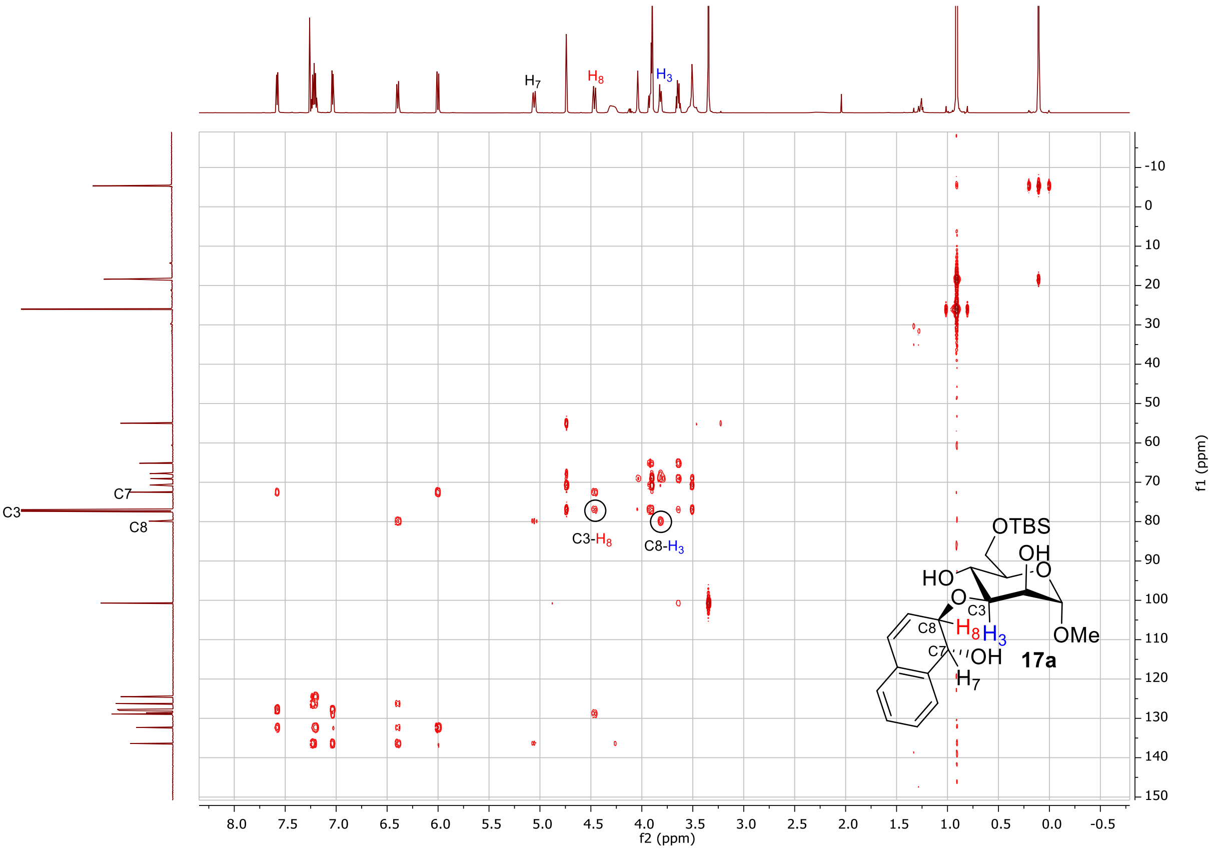

Supplementary Figure 97. HMBC spectra for **17a**

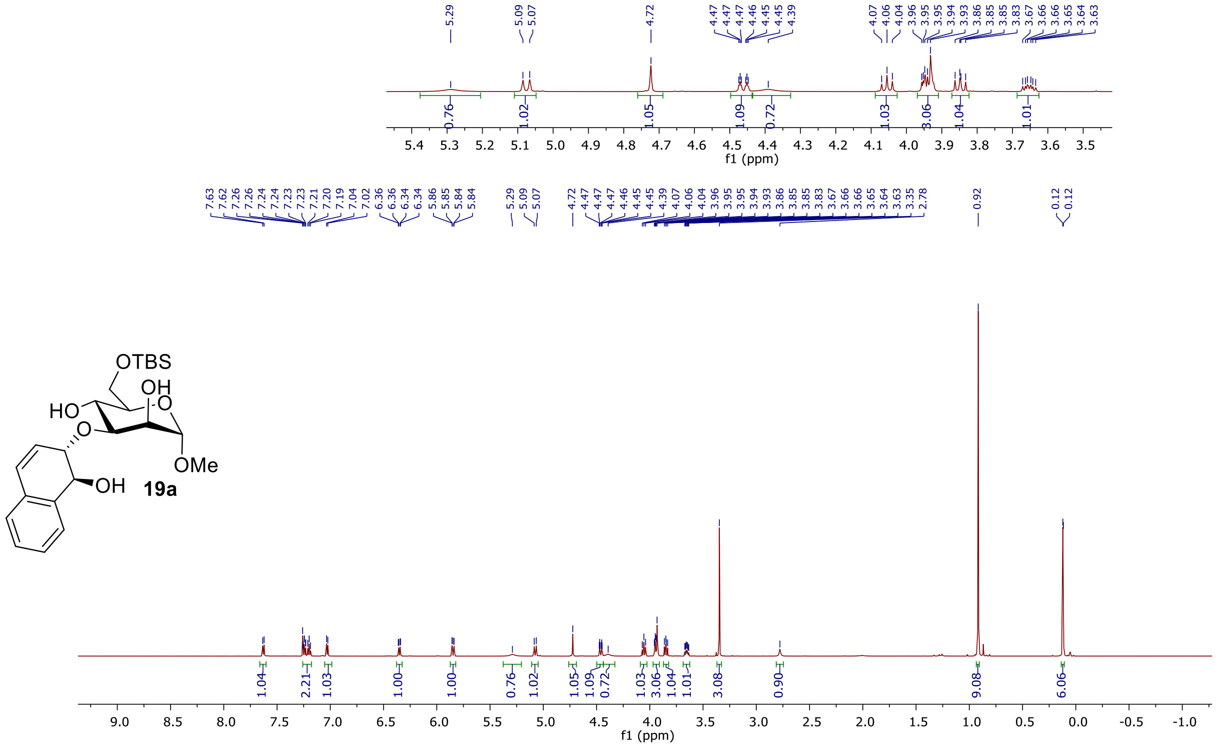

Supplementary Figure 98. ^{1}H spectra for **19a**

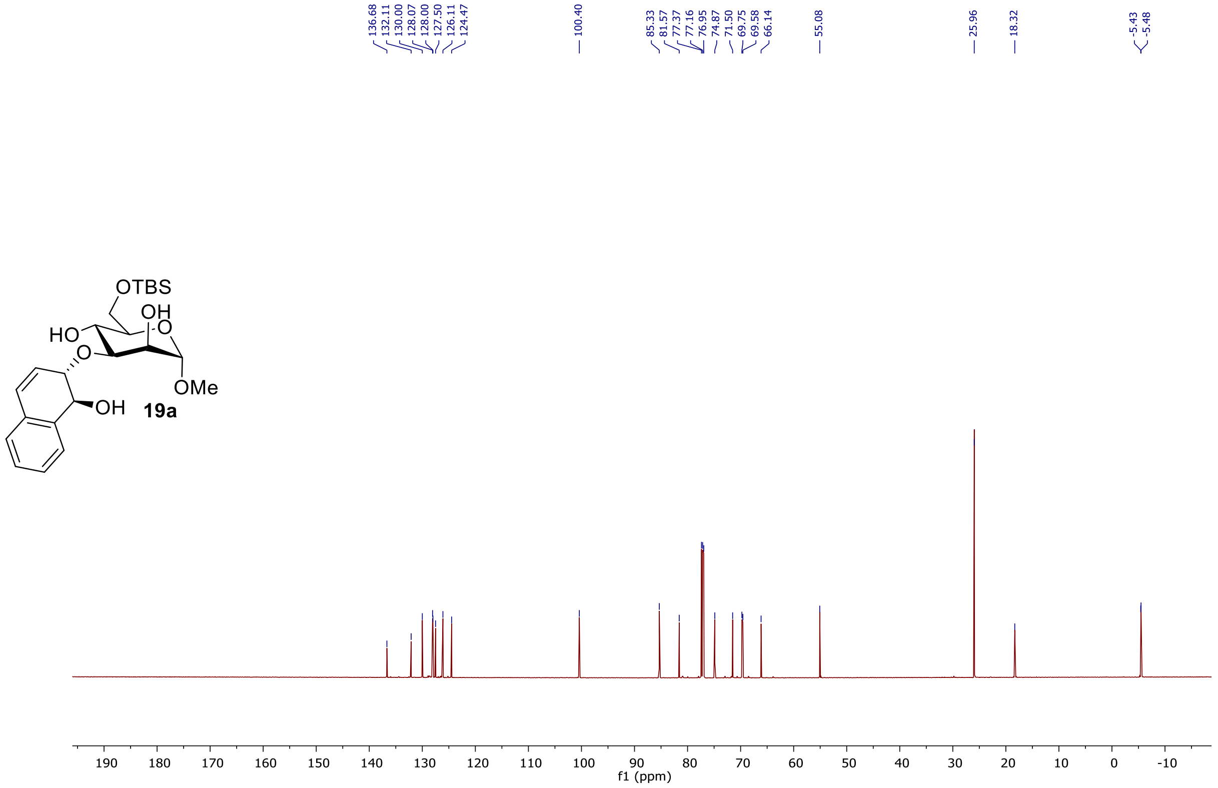

Supplementary Figure 99. ^{13}C spectra for **19a**

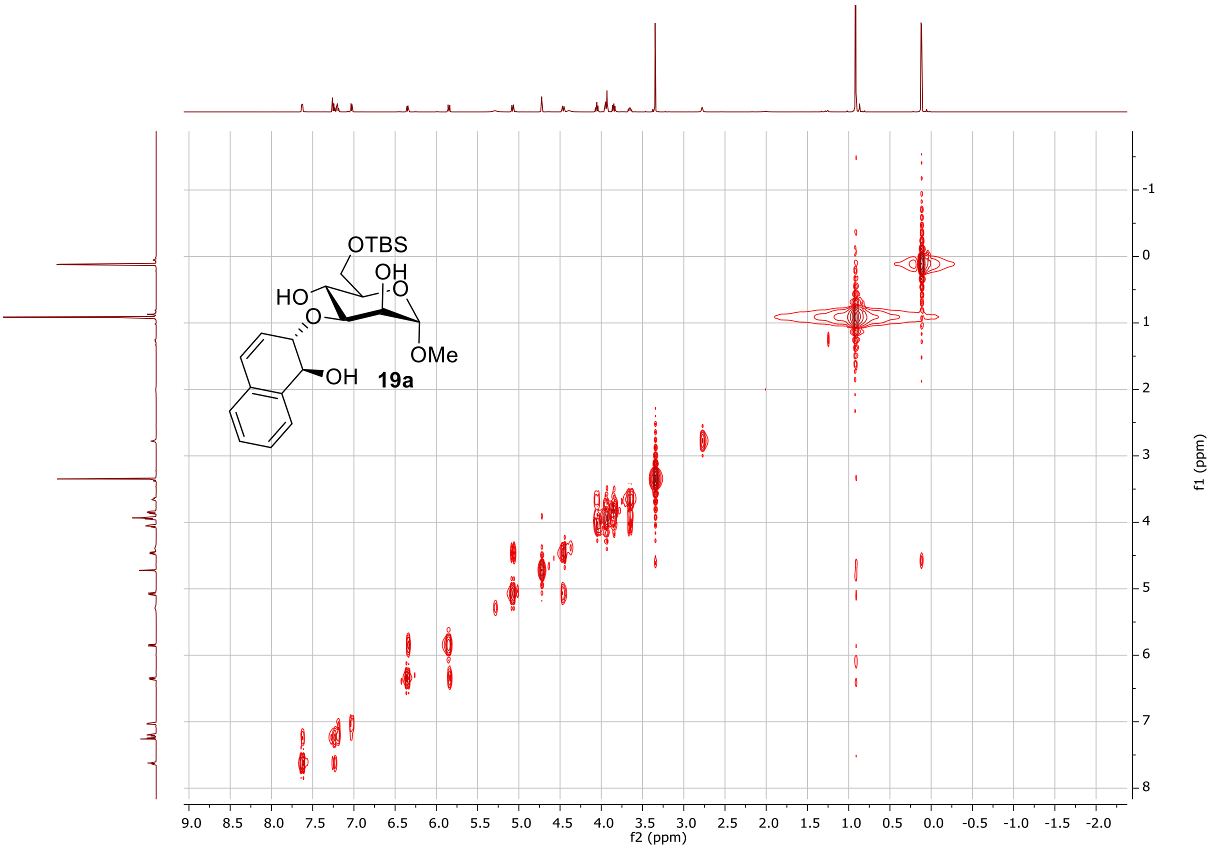

Supplementary Figure 100. COSY spectra for **19a**

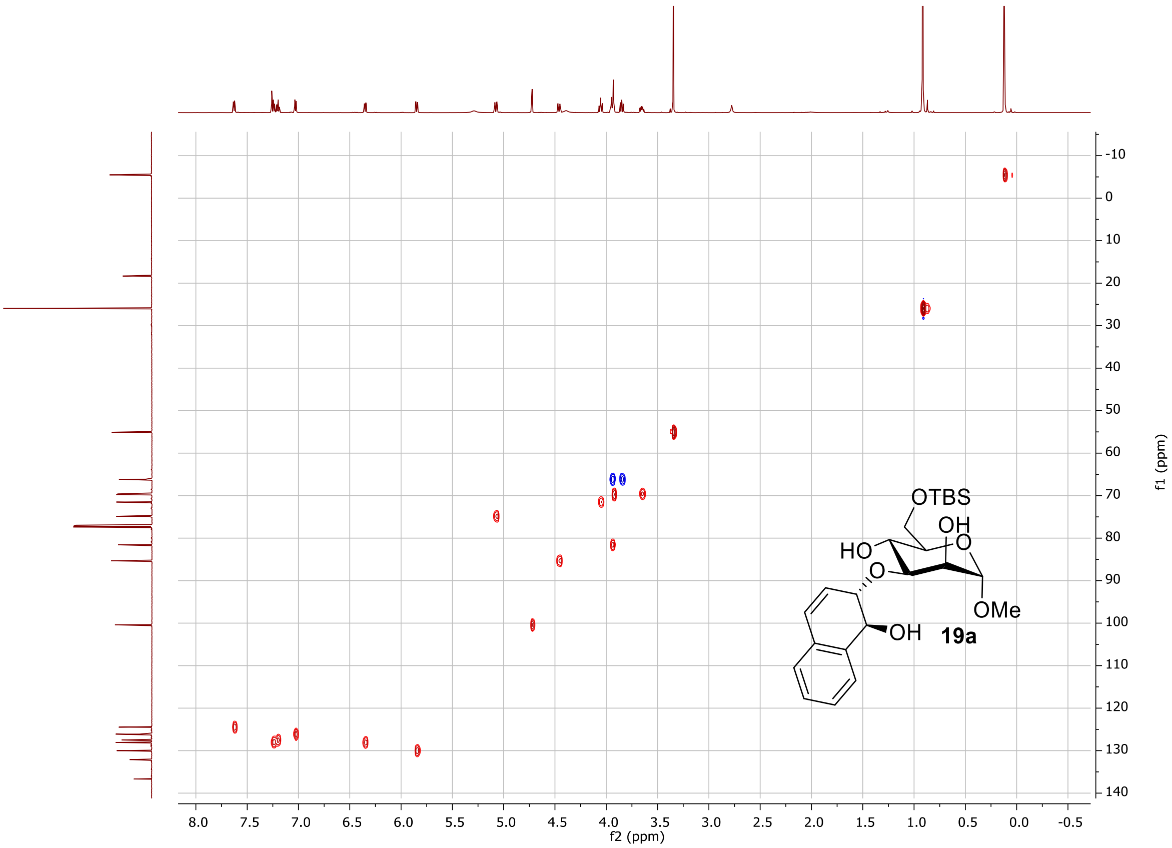

Supplementary Figure 101. HSQC spectra for **19a**

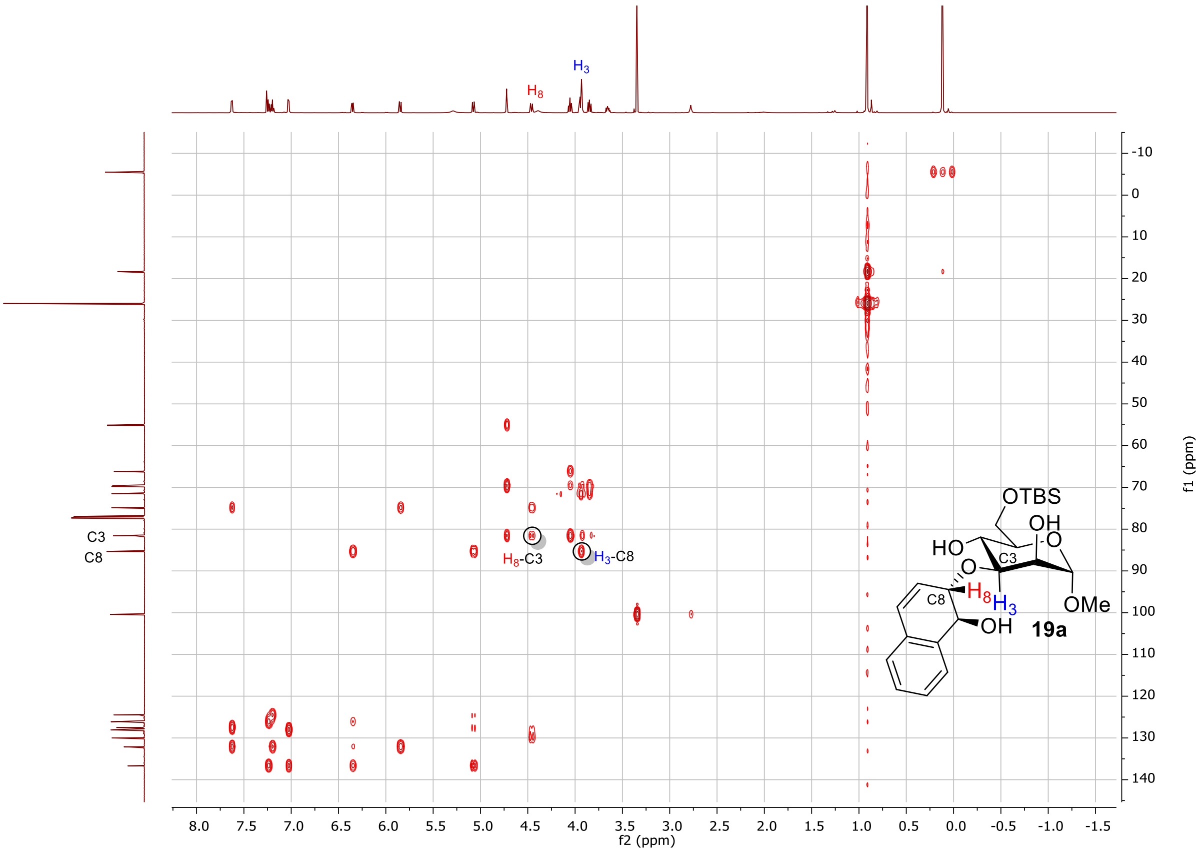

Supplementary Figure 102. HMBC spectra for **19a**

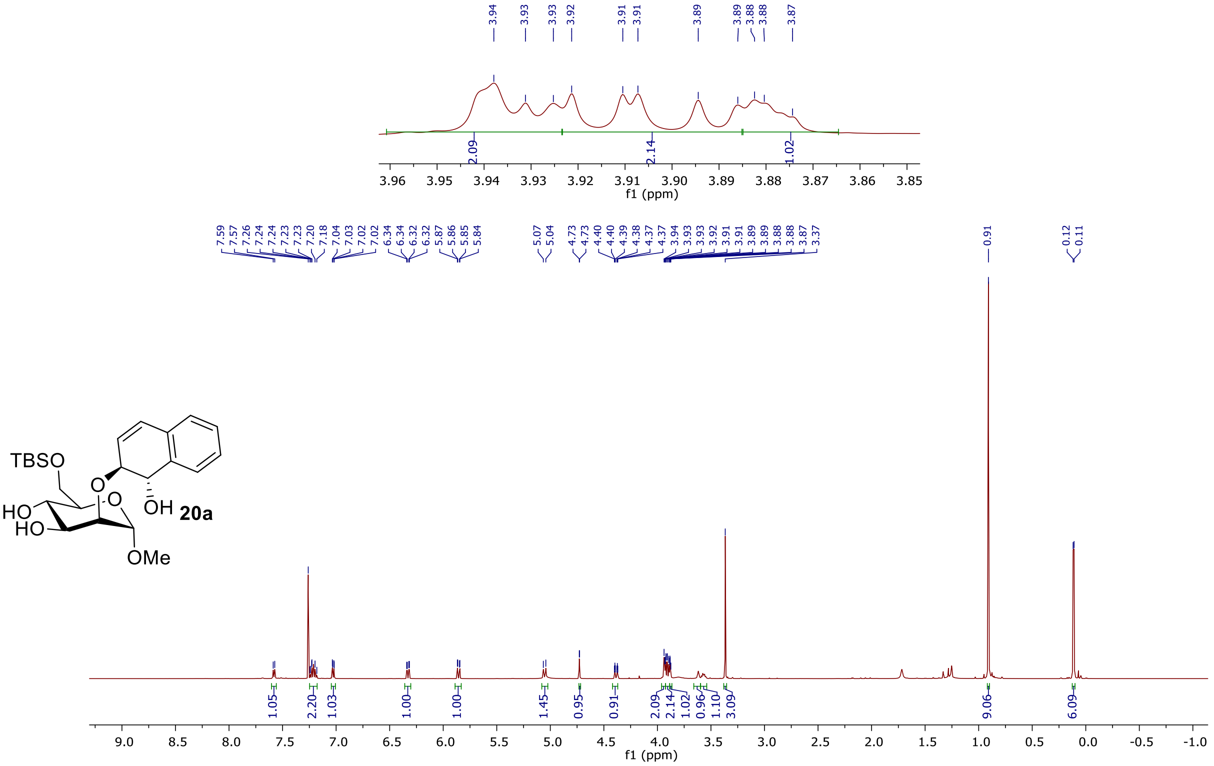

Supplementary Figure 103. ^{1}H spectra for **20a**

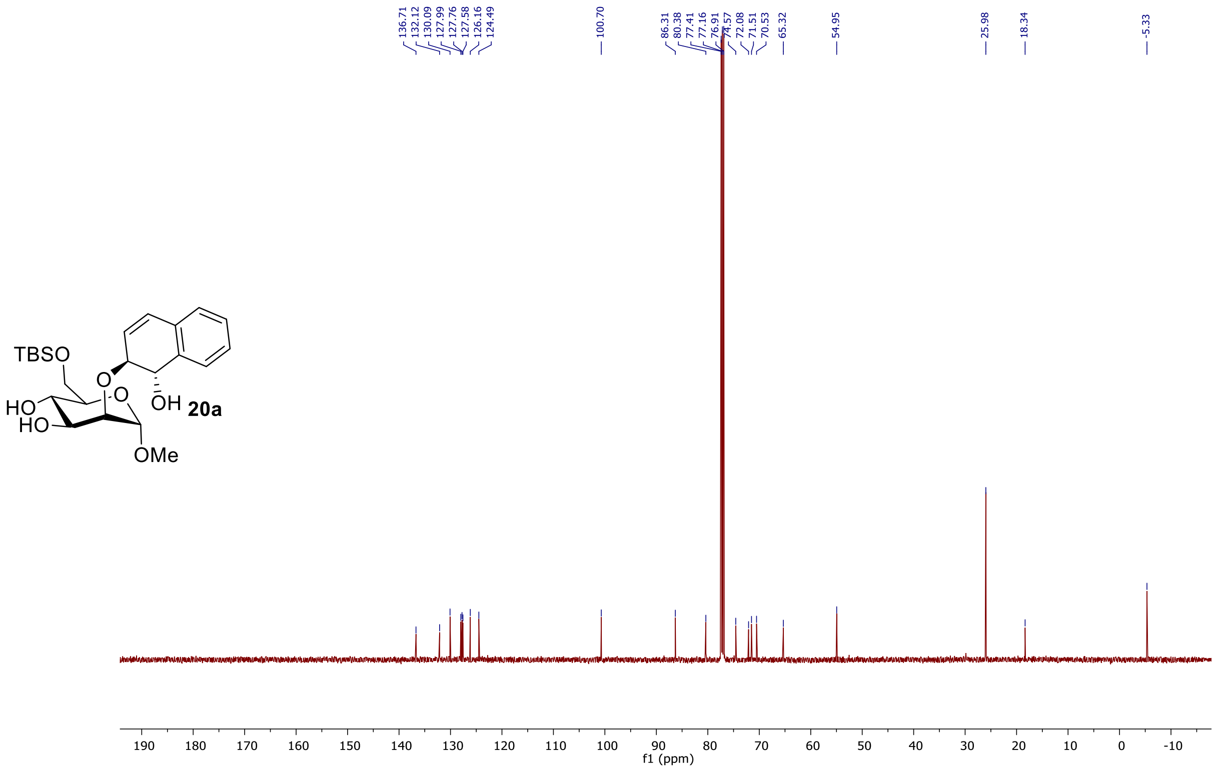

Supplementary Figure 104. ^{13}C spectra for **20a**

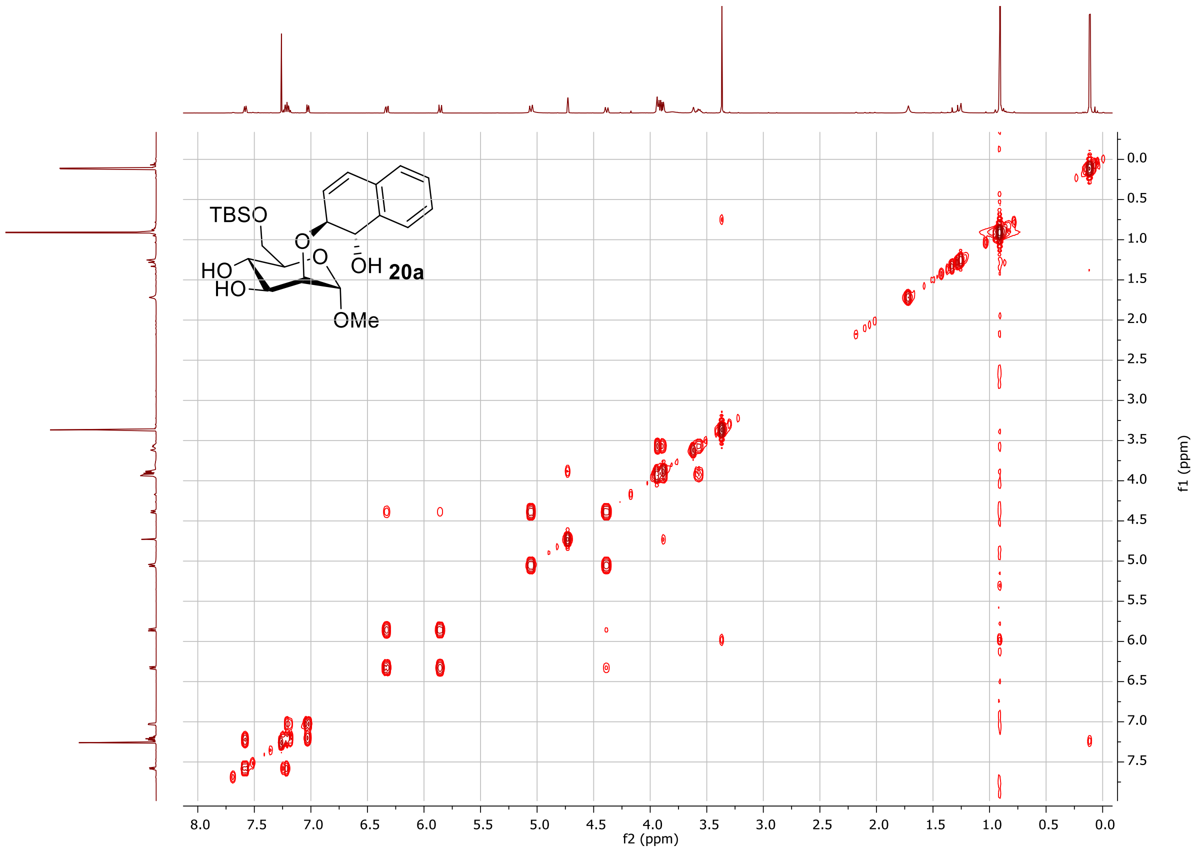

Supplementary Figure 105. COSY spectra for **20a**

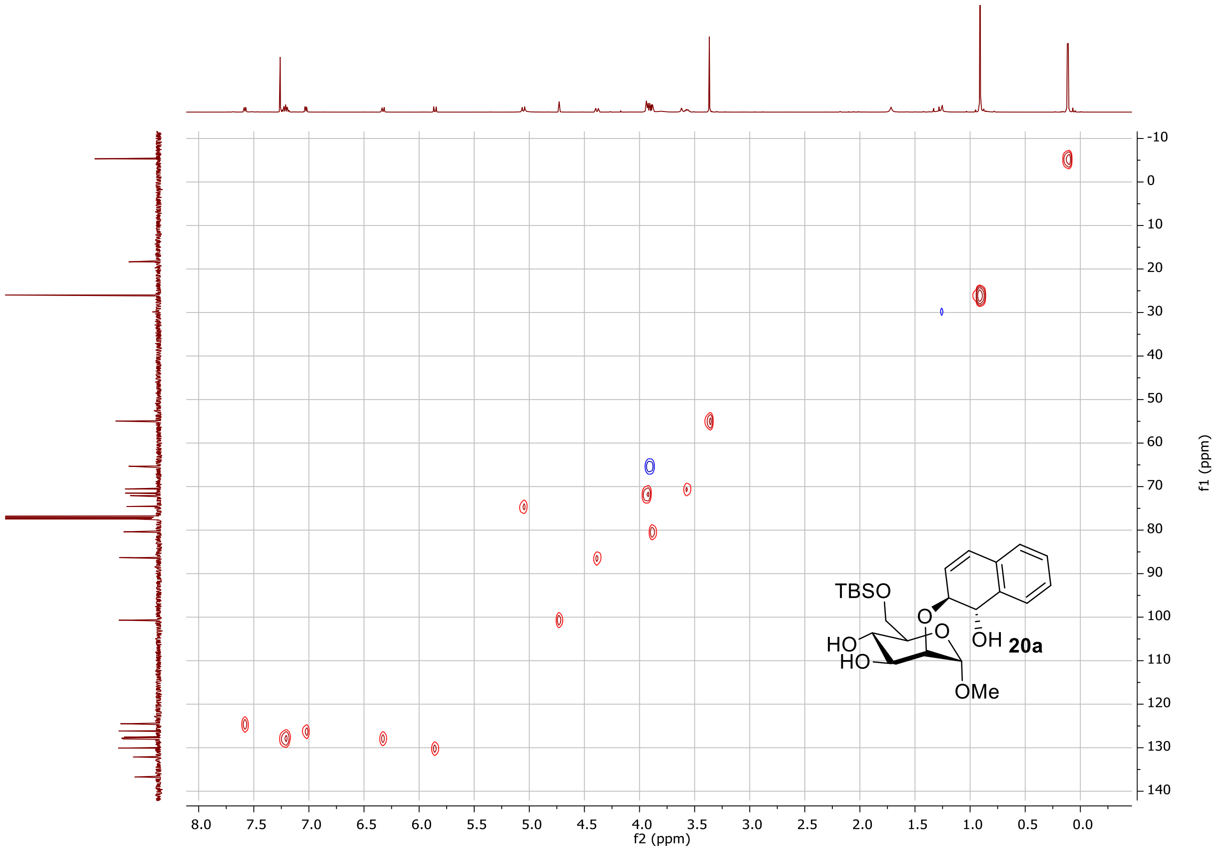

Supplementary Figure 106. HSQC spectra for **20a**

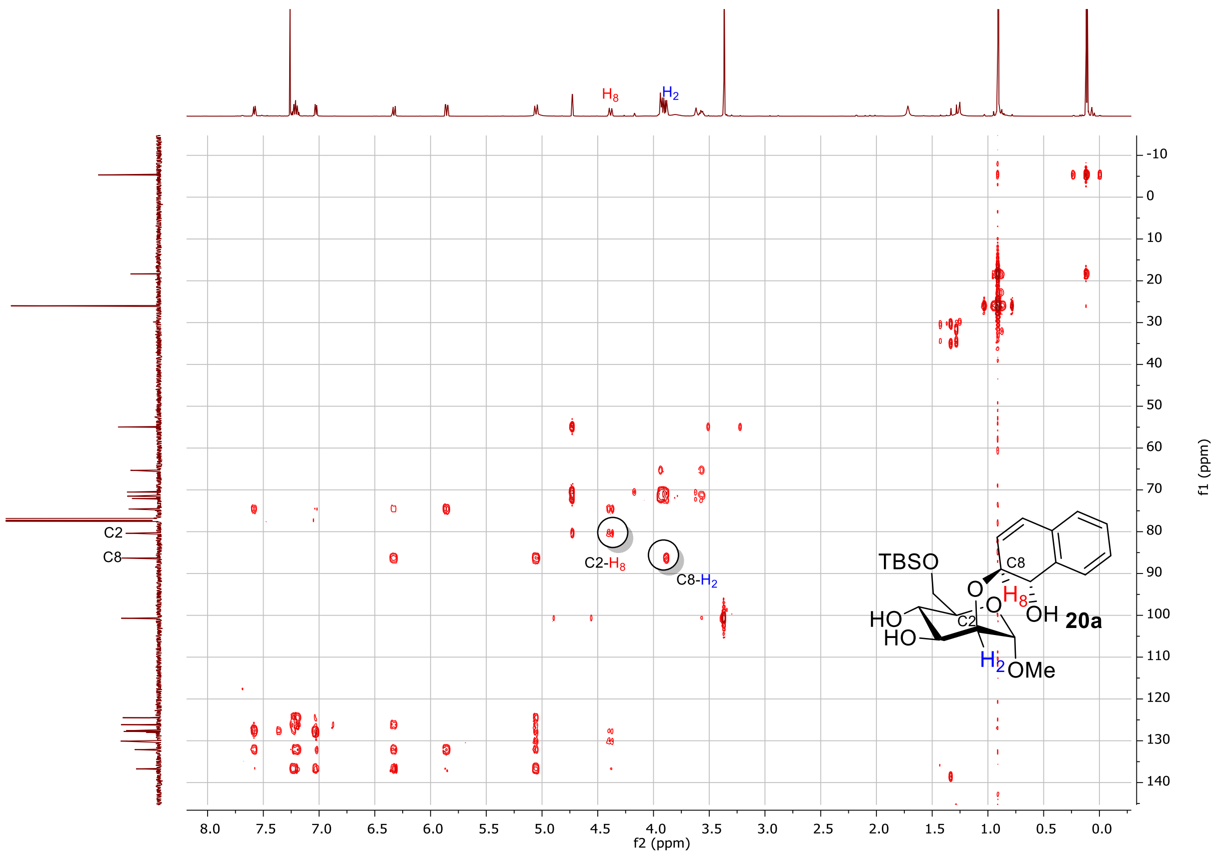

Supplementary Figure 107. HMBC spectra for **20a**

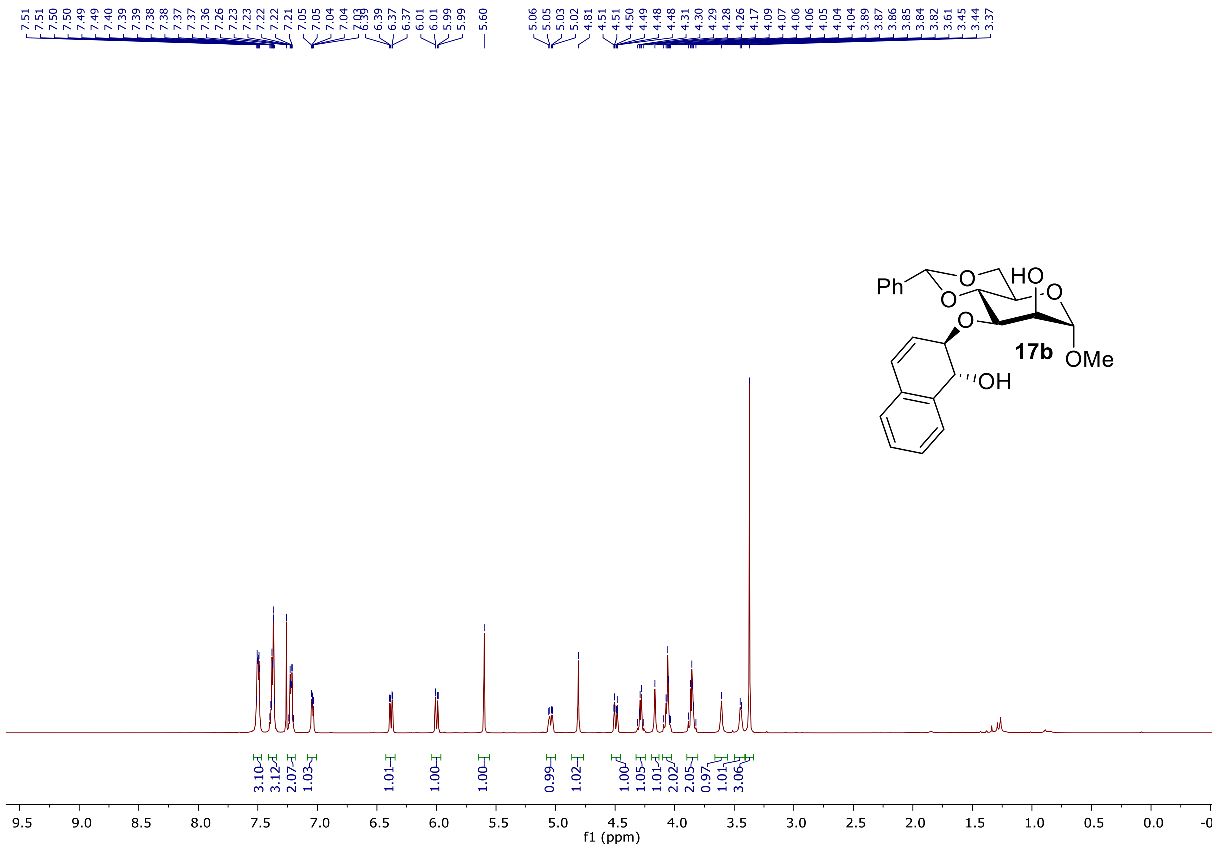

Supplementary Figure 108. ^{1}H spectra for **17b**

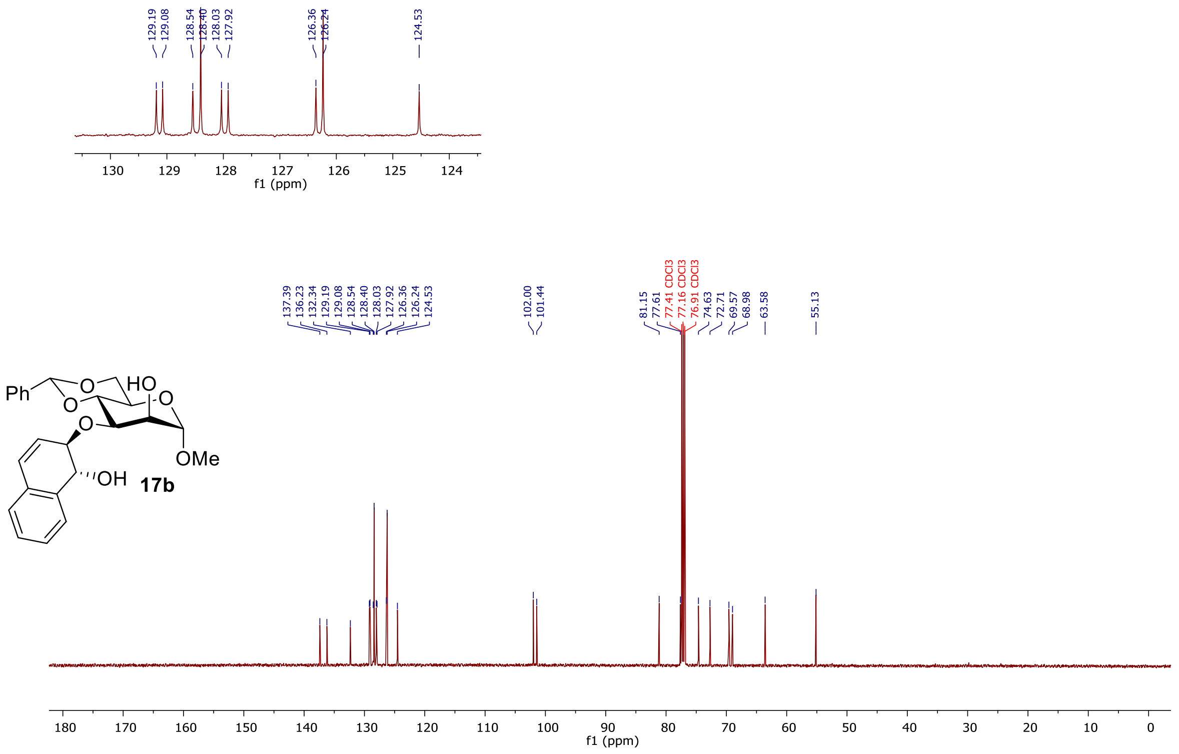

Supplementary Figure 109. ^{13}C spectra for **17b**

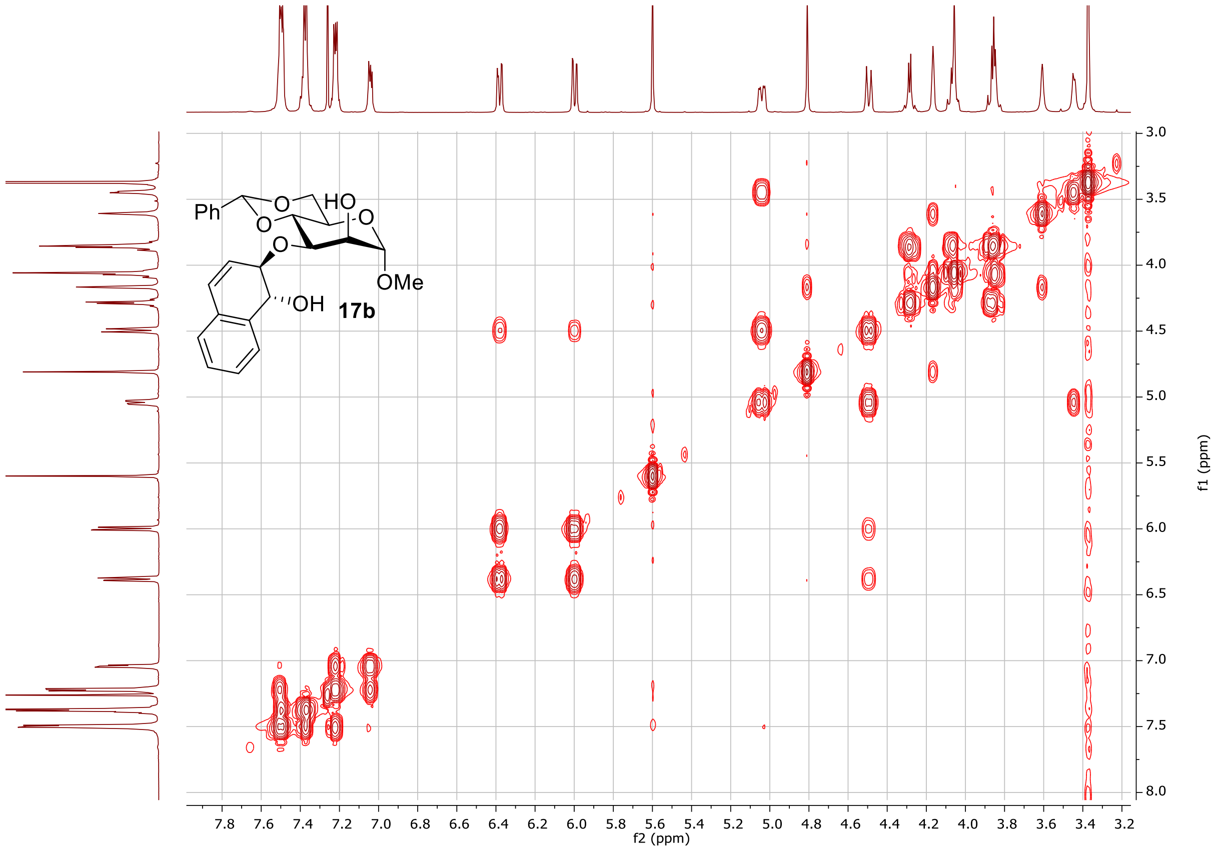

Supplementary Figure 110. COSY spectra for **17b**

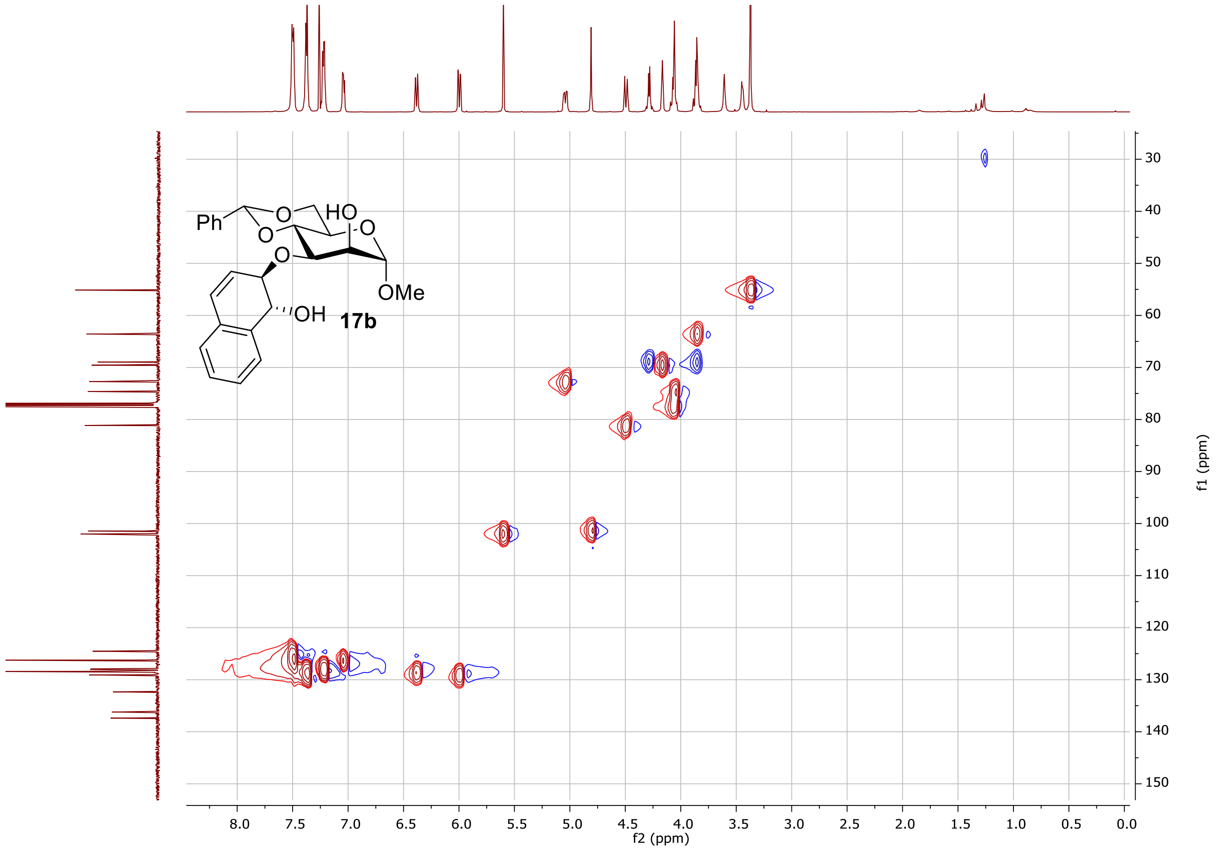

Supplementary Figure 111. HSQC spectra for **17b**

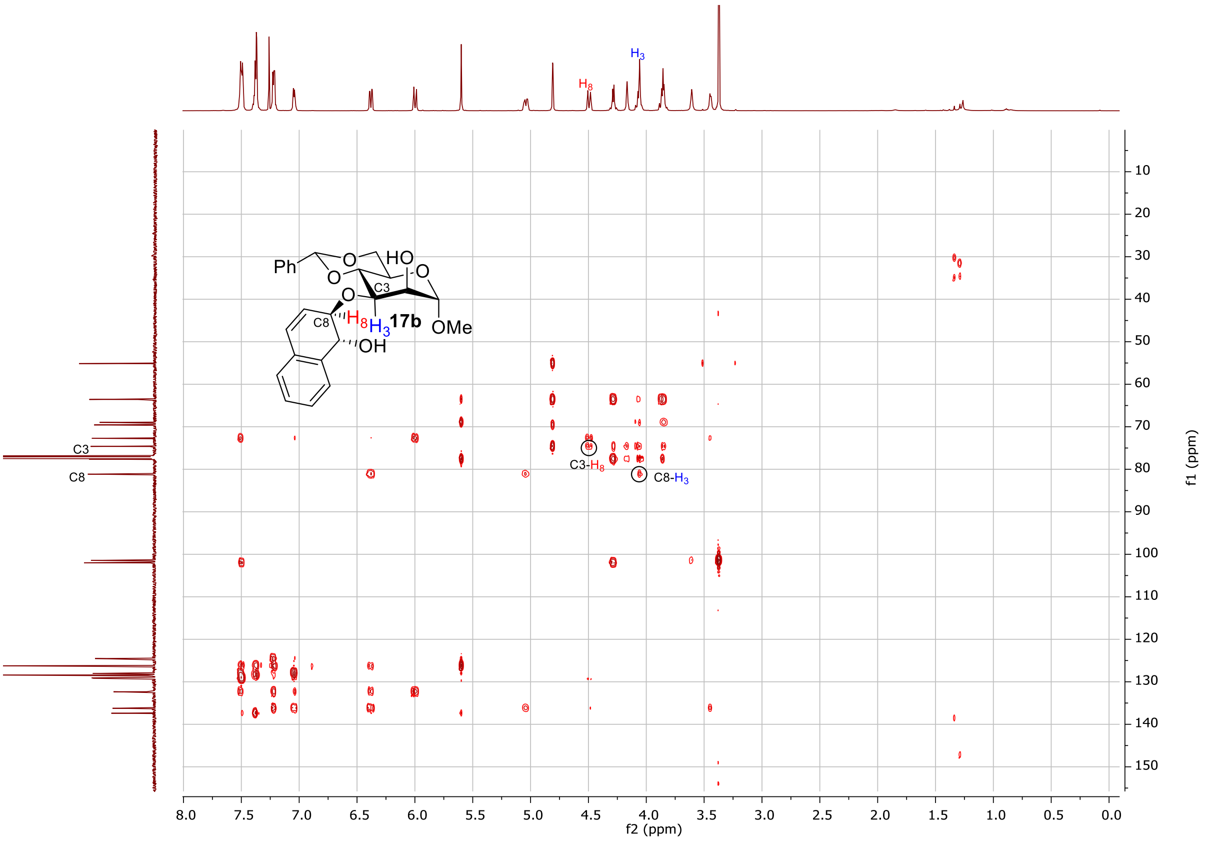

Supplementary Figure 112. HMBC spectra for **17b**

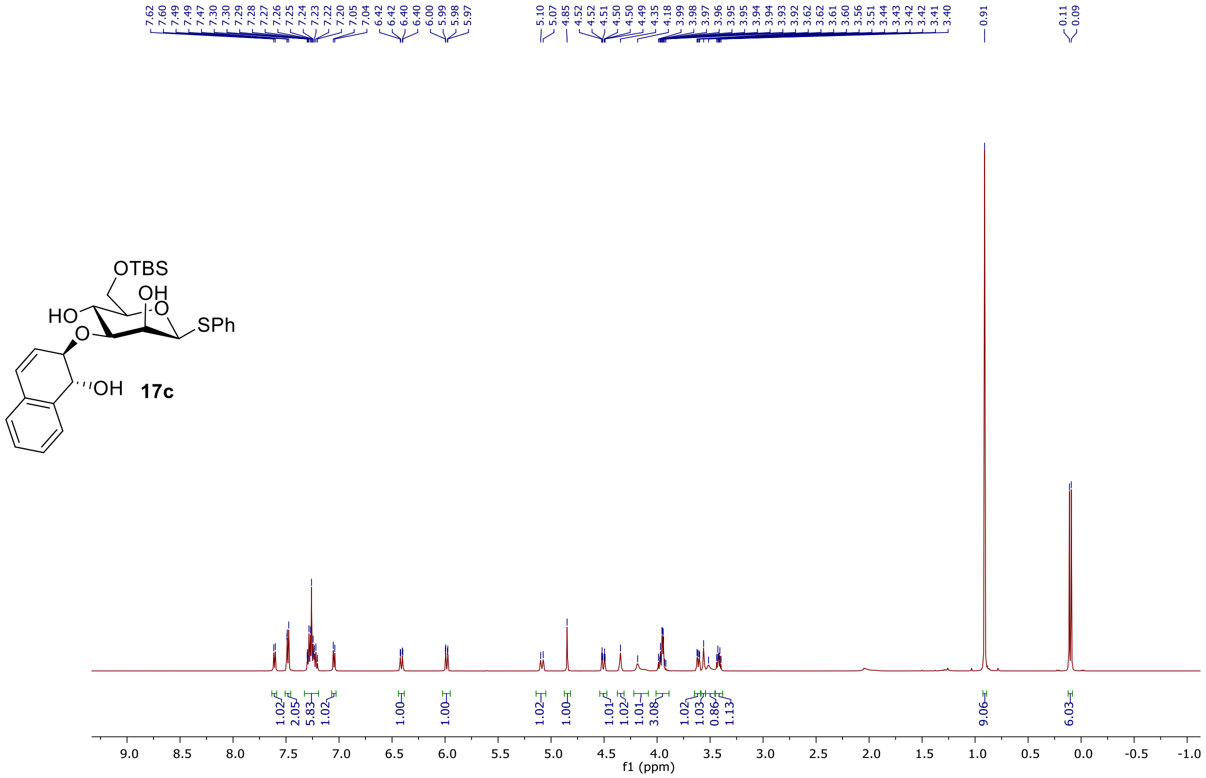

Supplementary Figure 113. ^{1}H spectra for **17c**

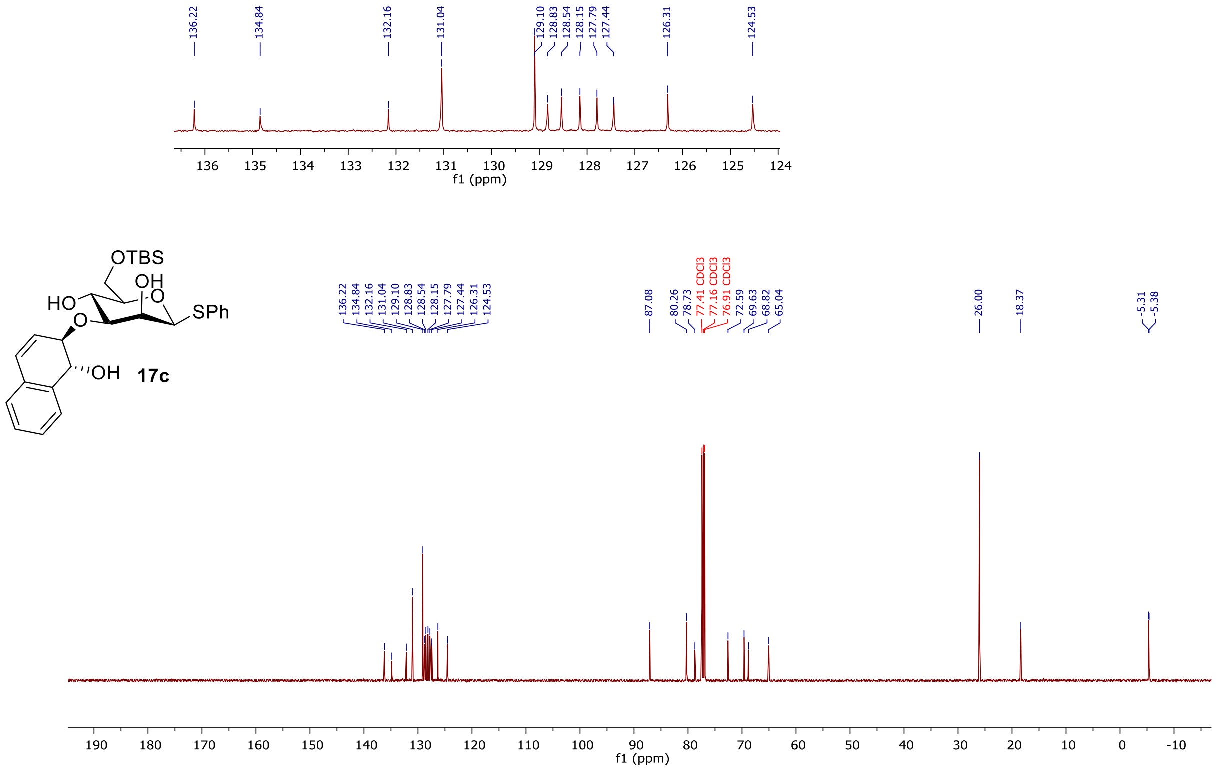

Supplementary Figure 114. ^{13}C spectra for **17c**

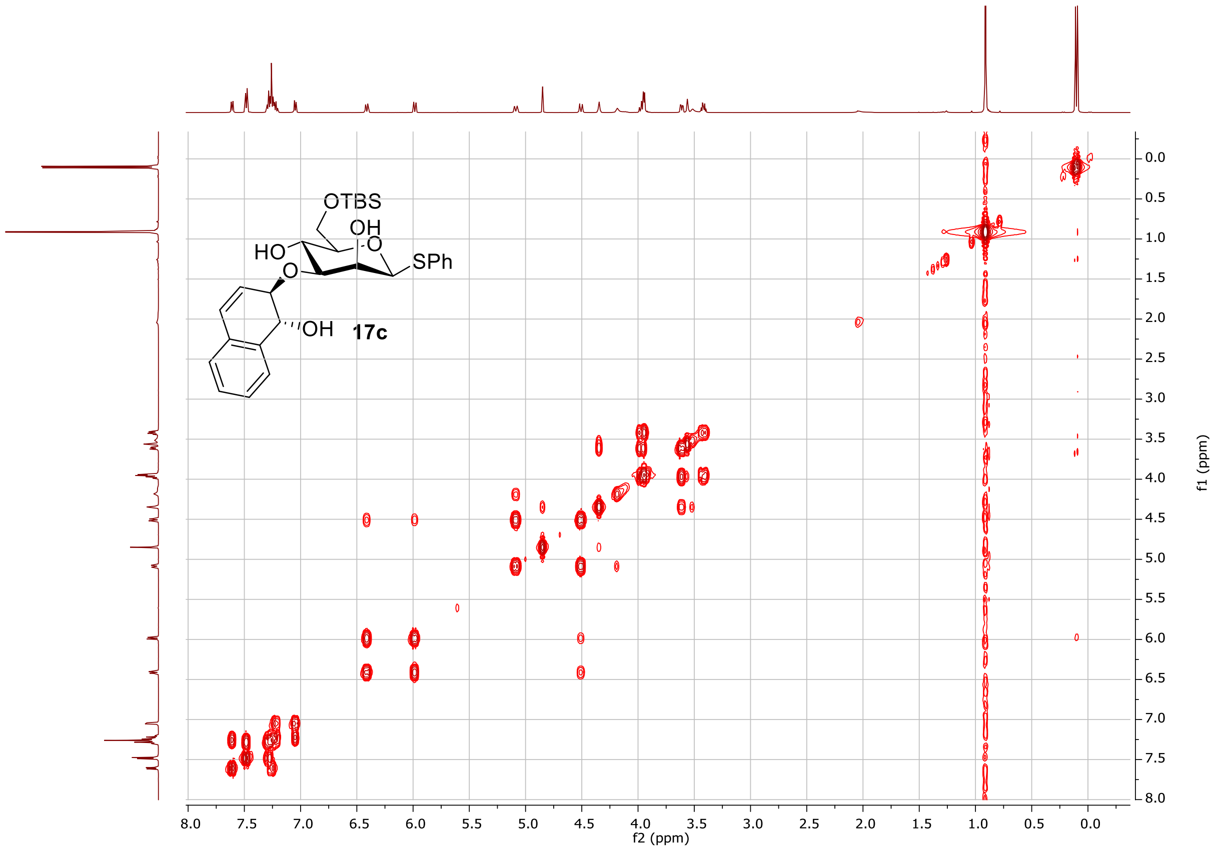

Supplementary Figure 115. COSY spectra for **17c**

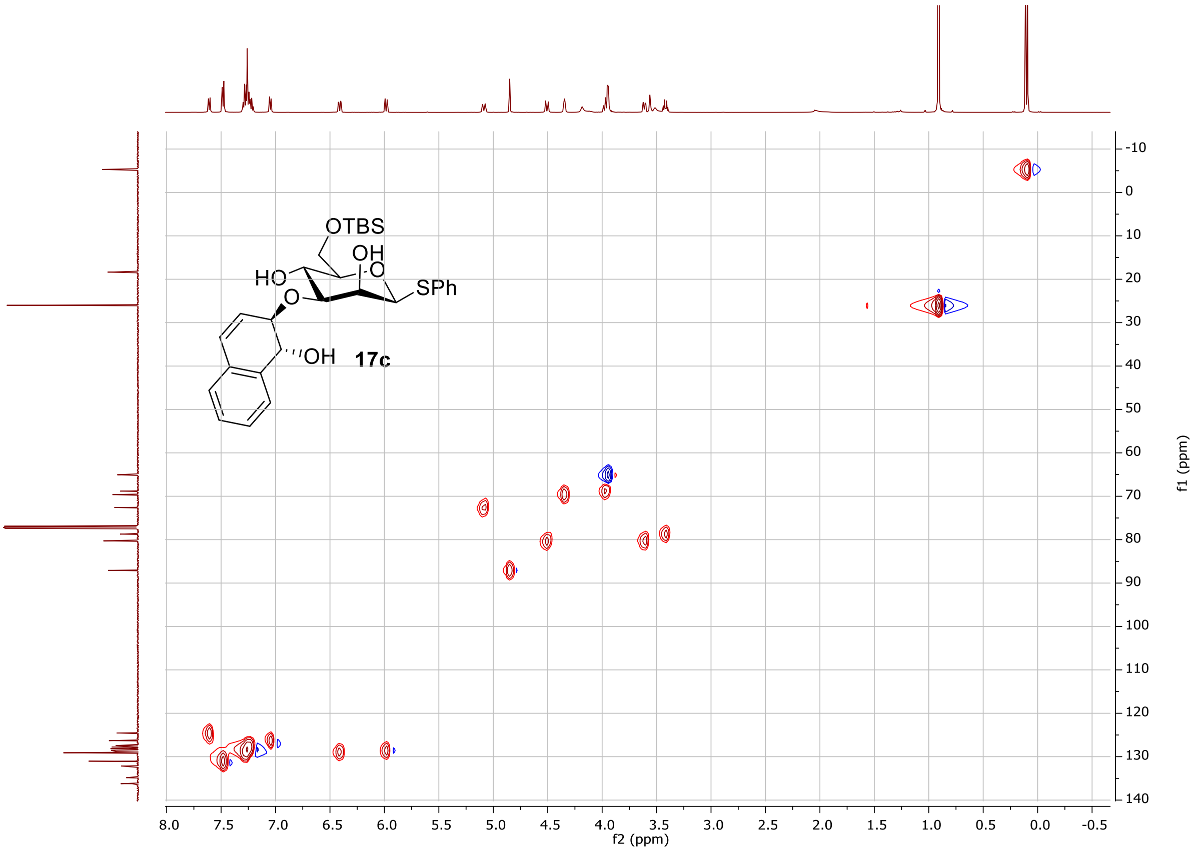

Supplementary Figure 116. HSQC spectra for **17c**

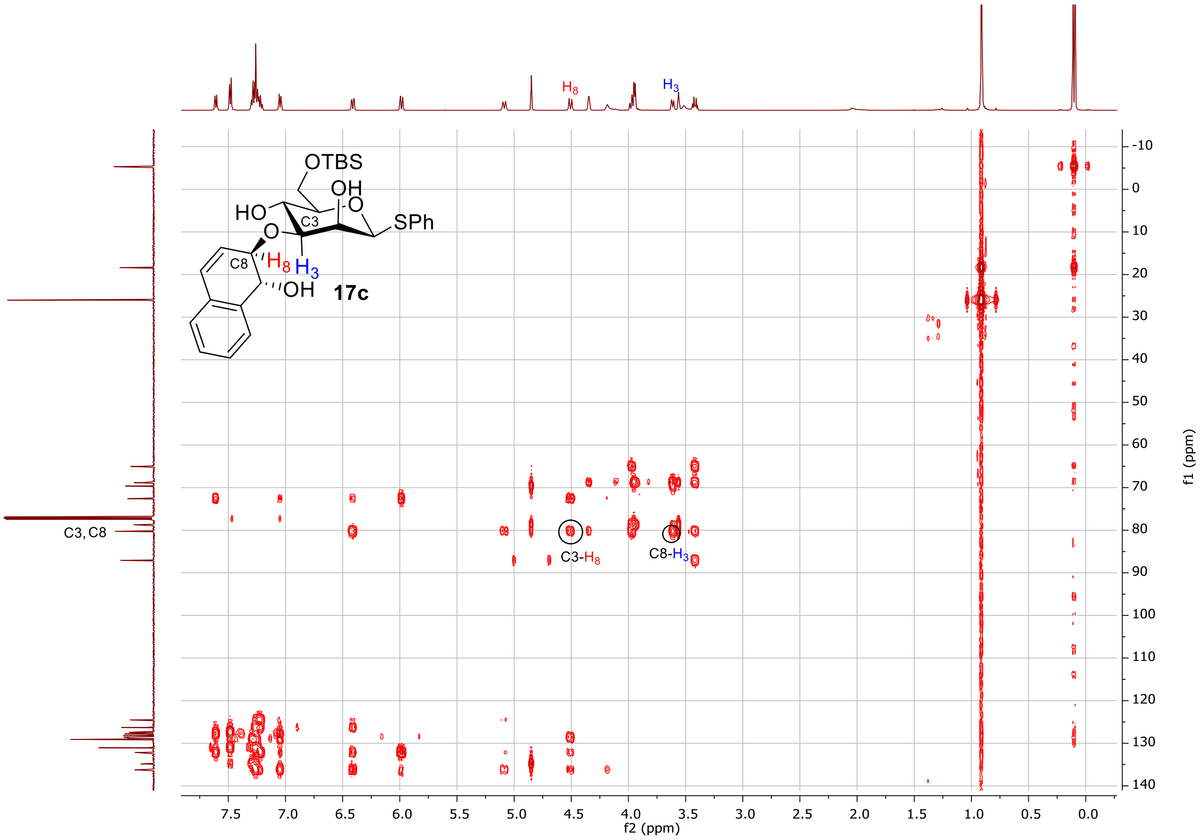

Supplementary Figure 117. HMBC spectra for **17c**

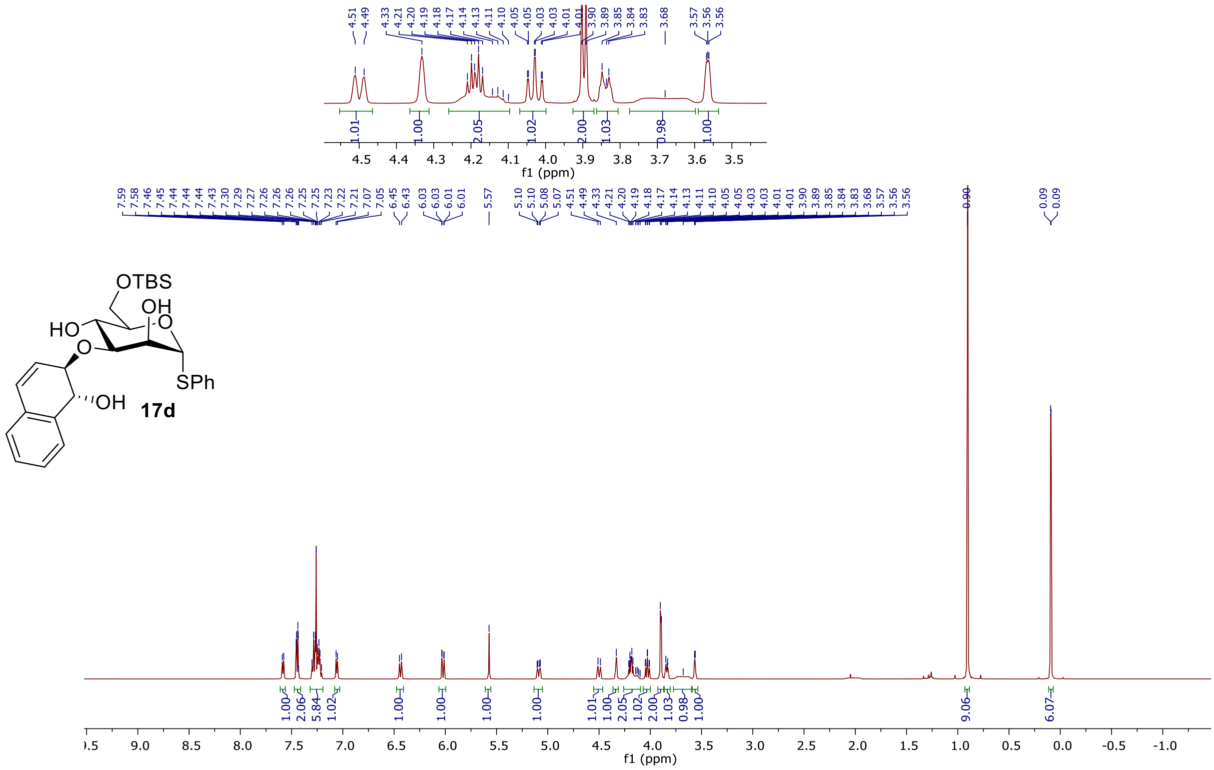

Supplementary Figure 118. ^{1}H spectra for **17d**

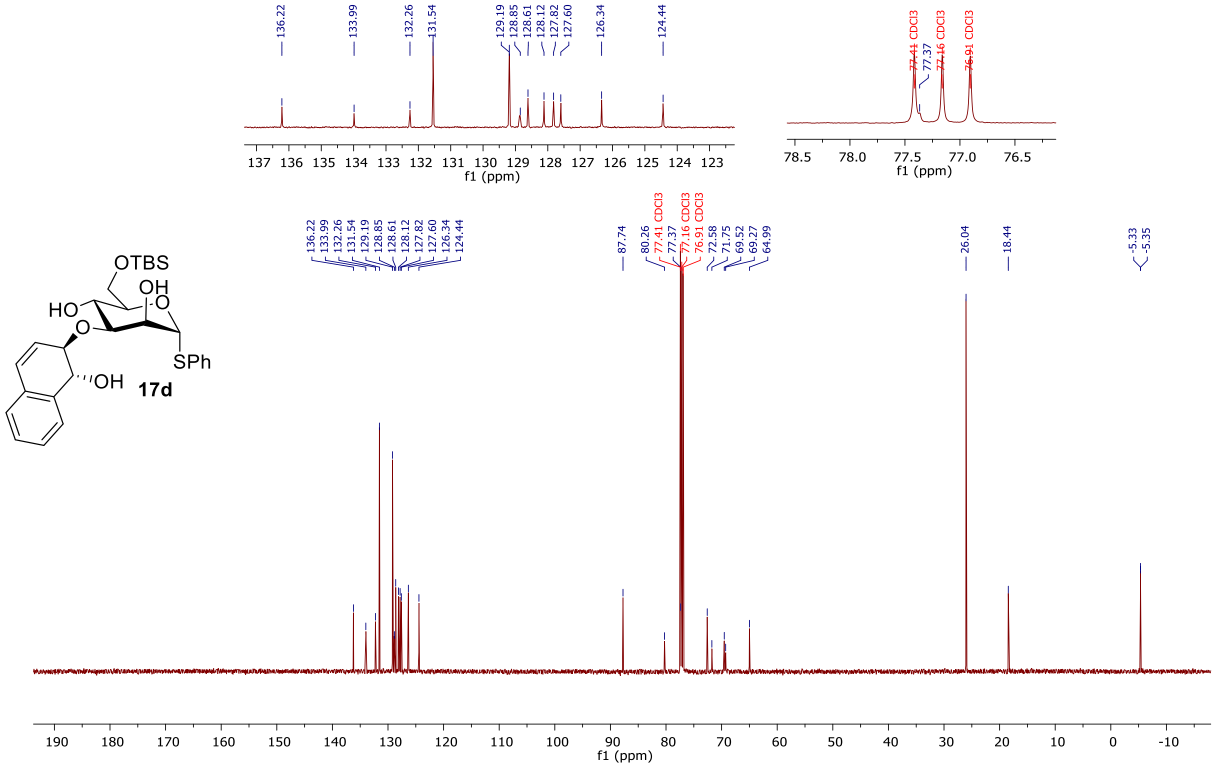

Supplementary Figure 119. ^{13}C spectra for **17d**

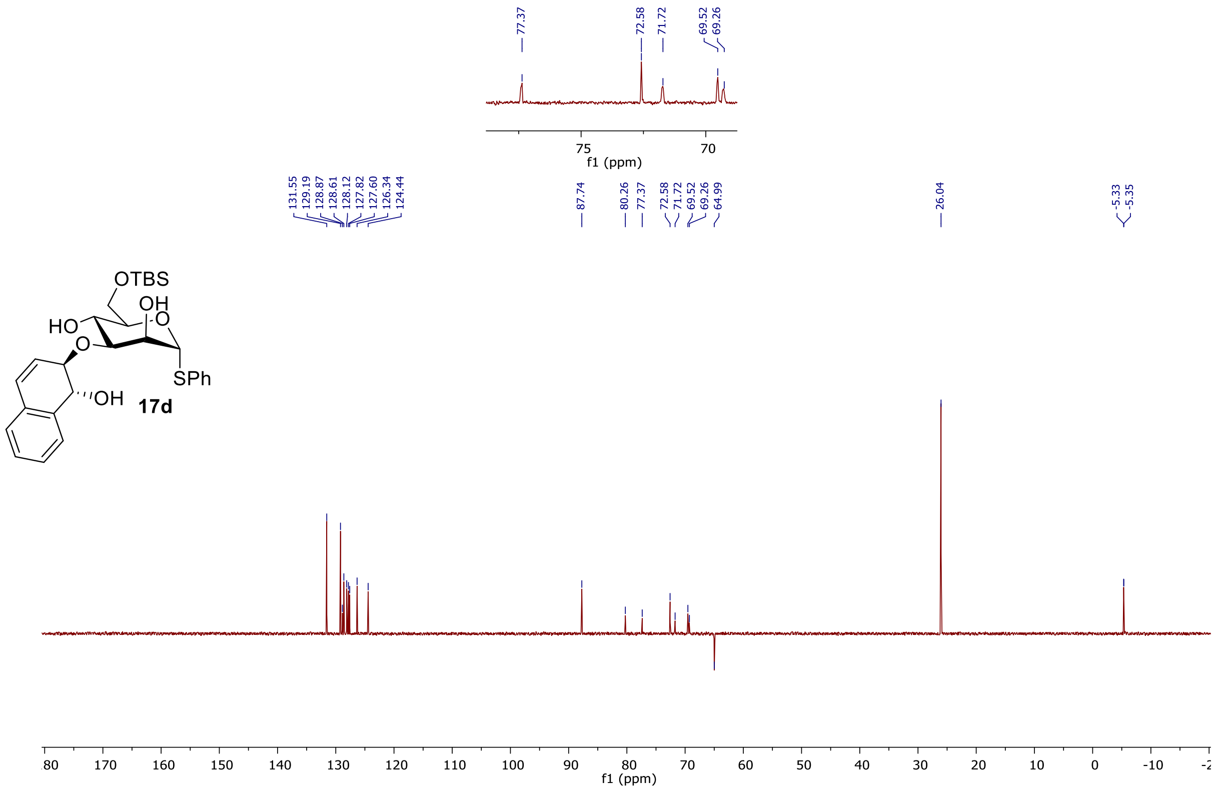

Supplementary Figure 120. DEPT spectra for **17d**

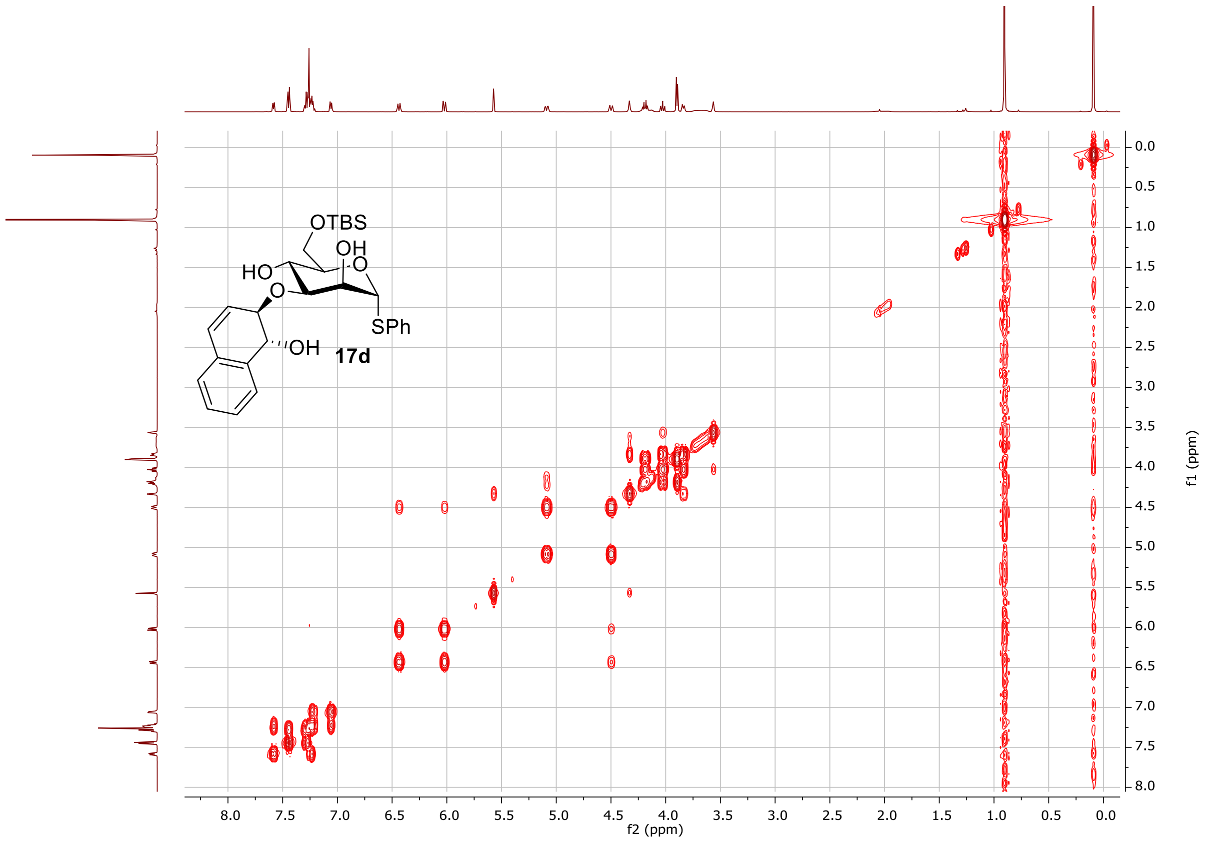

Supplementary Figure 121. COSY spectra for **17d**

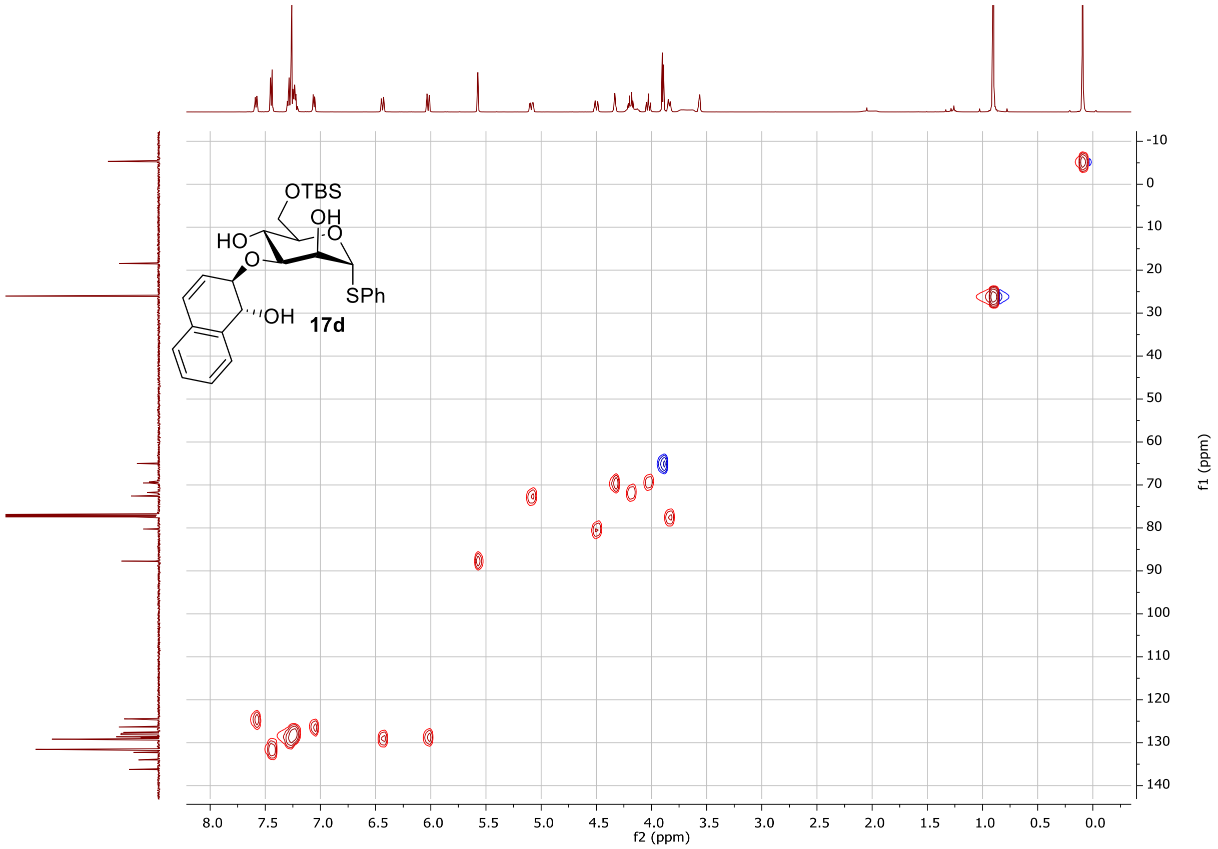

Supplementary Figure 122. HSQC spectra for **17d**

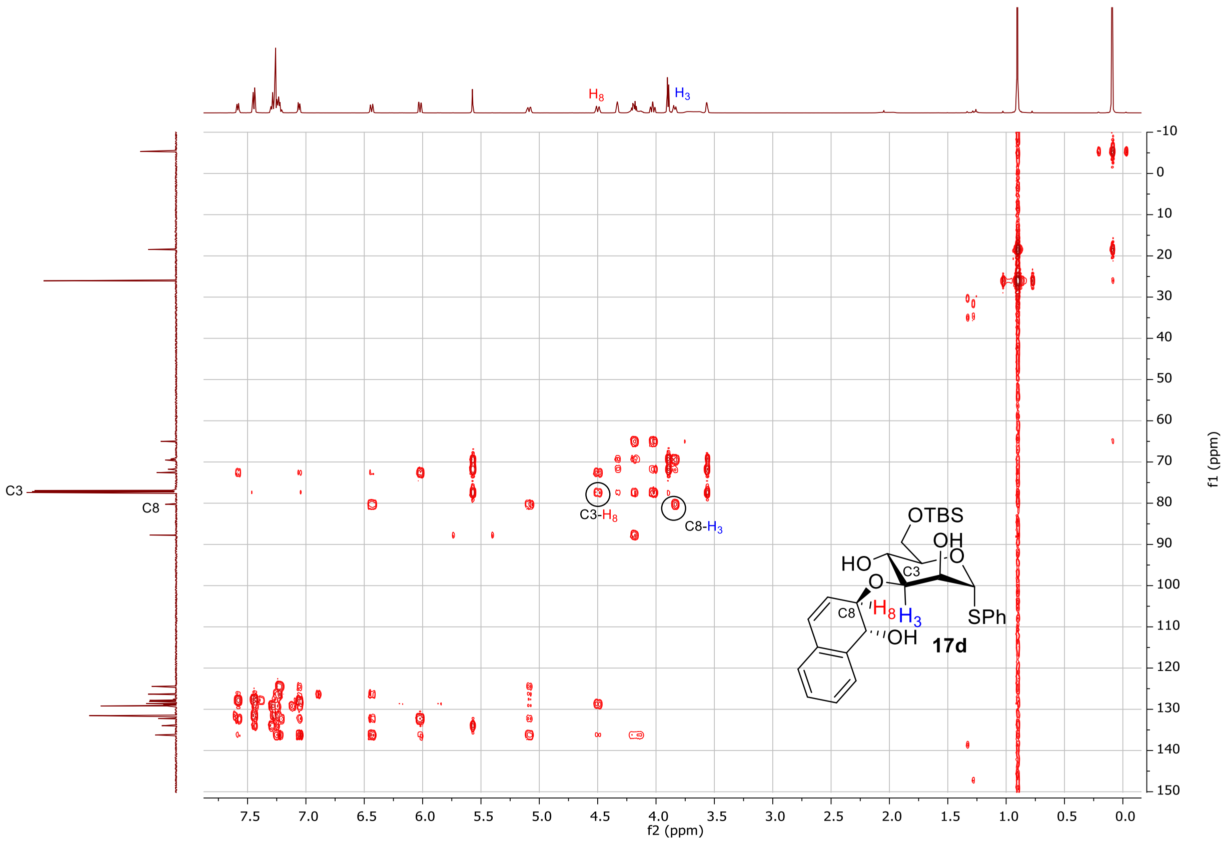

Supplementary Figure 123. **HMBC** spectra for **17d**

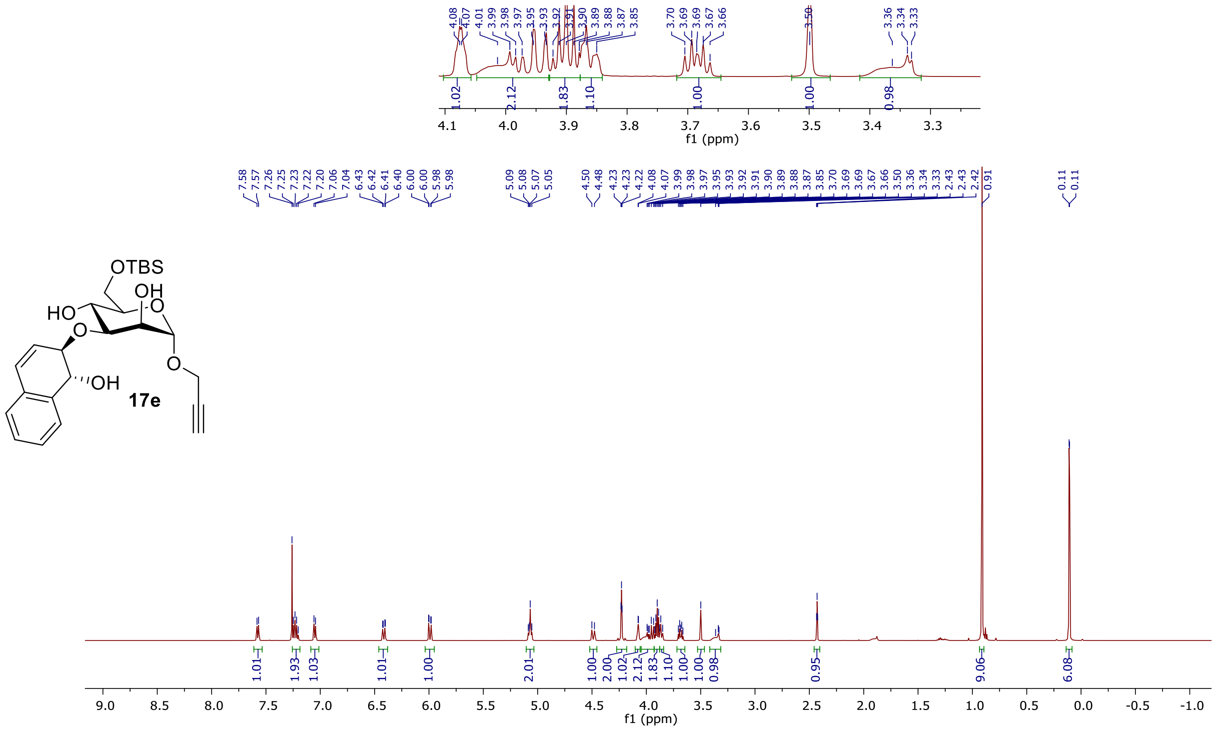

Supplementary Figure 124. ^{1}H spectra for **17e**

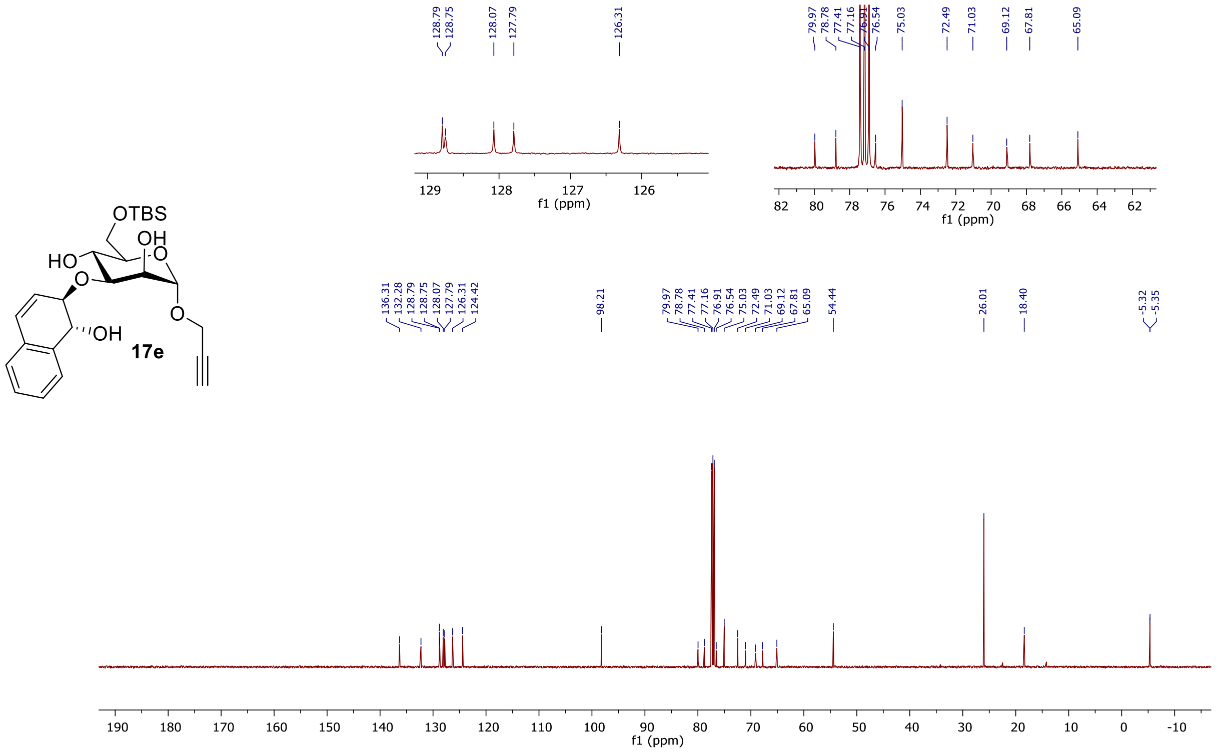

Supplementary Figure 125. ^{13}C spectra for **17e**

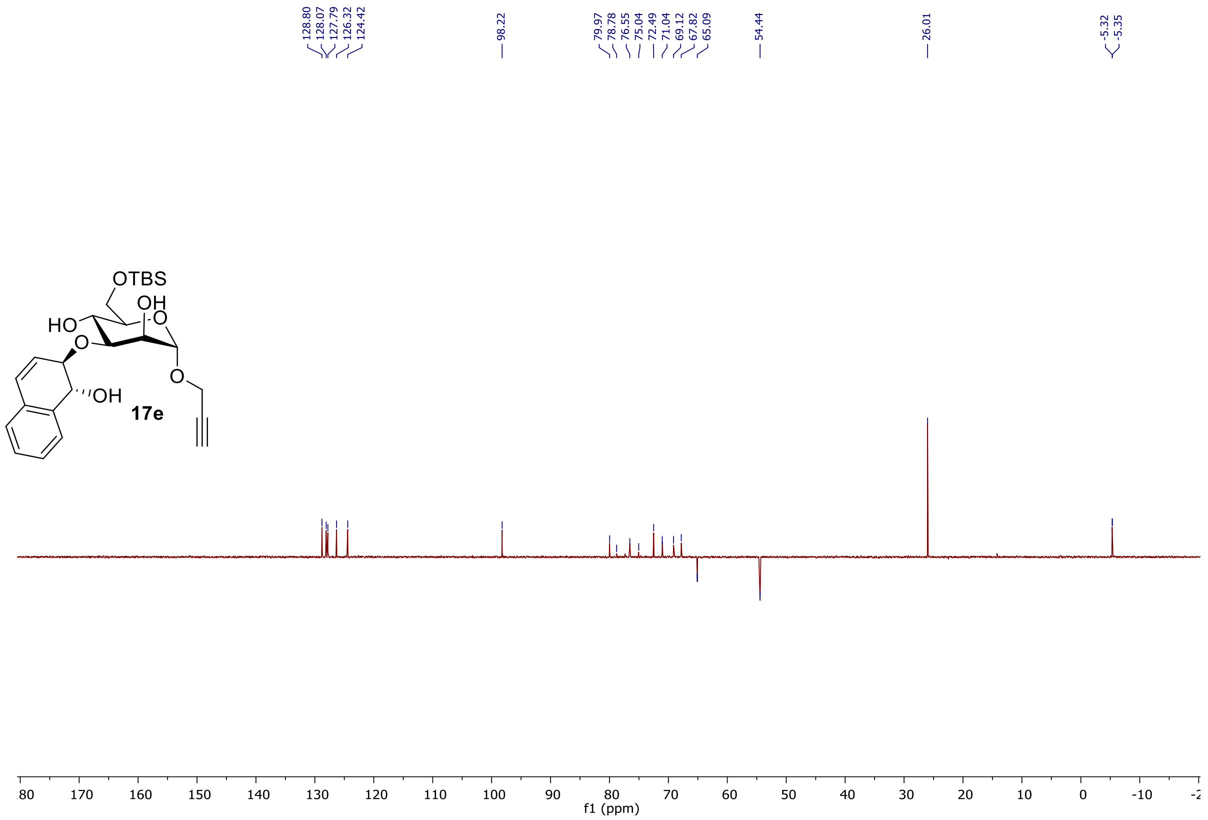

Supplementary Figure 126. DEPT spectra for **17e**

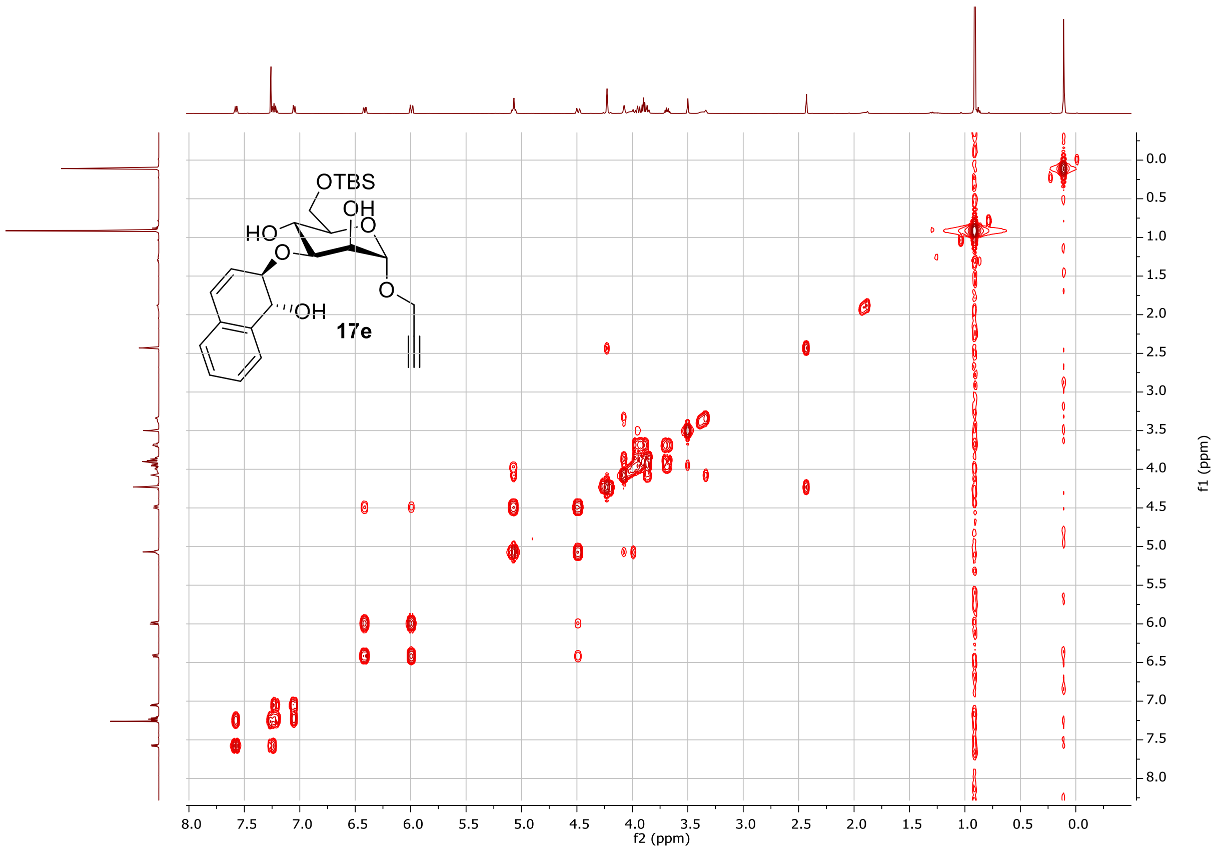

Supplementary Figure 127. COSY spectra for **17e**

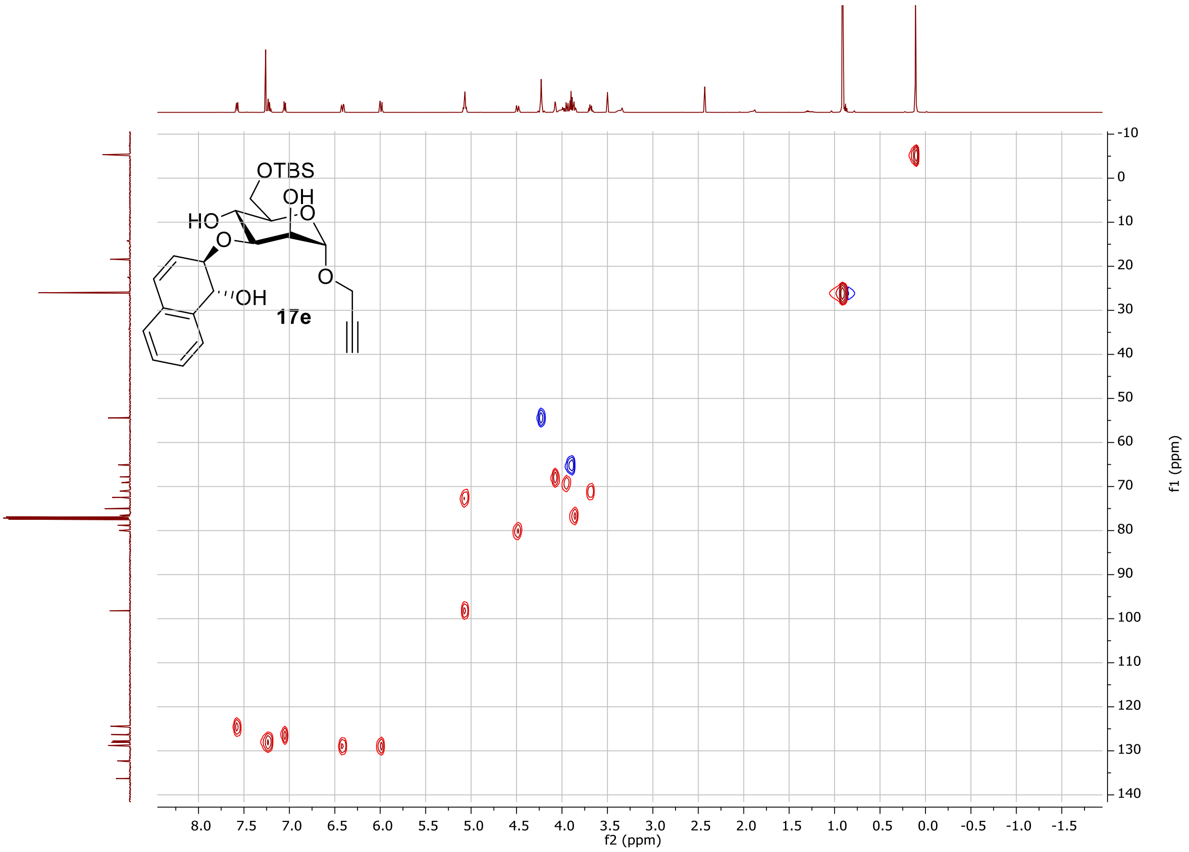

Supplementary Figure 128. HSQC spectra for **17e**

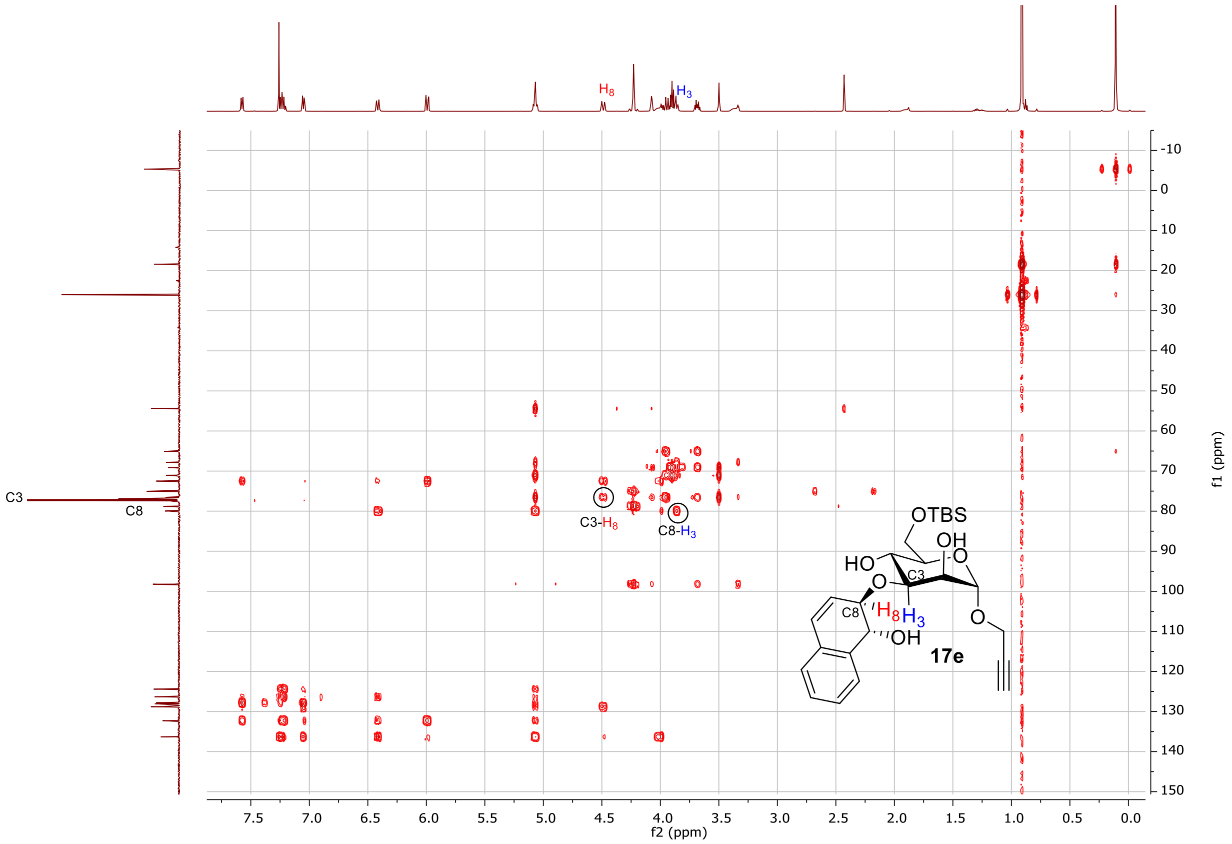

Supplementary Figure 129. HMBC spectra for **17e**

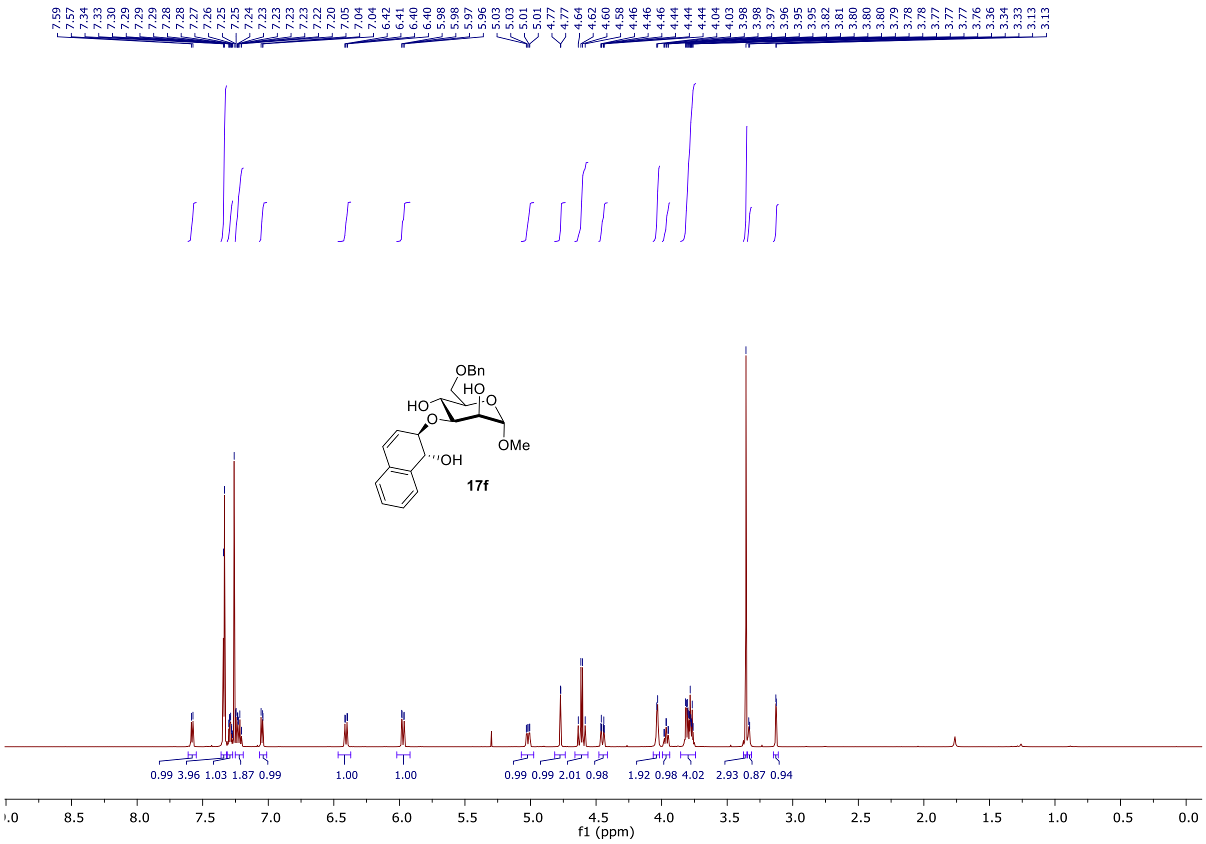

Supplementary Figure 130. ^{1}H spectra for **17f**

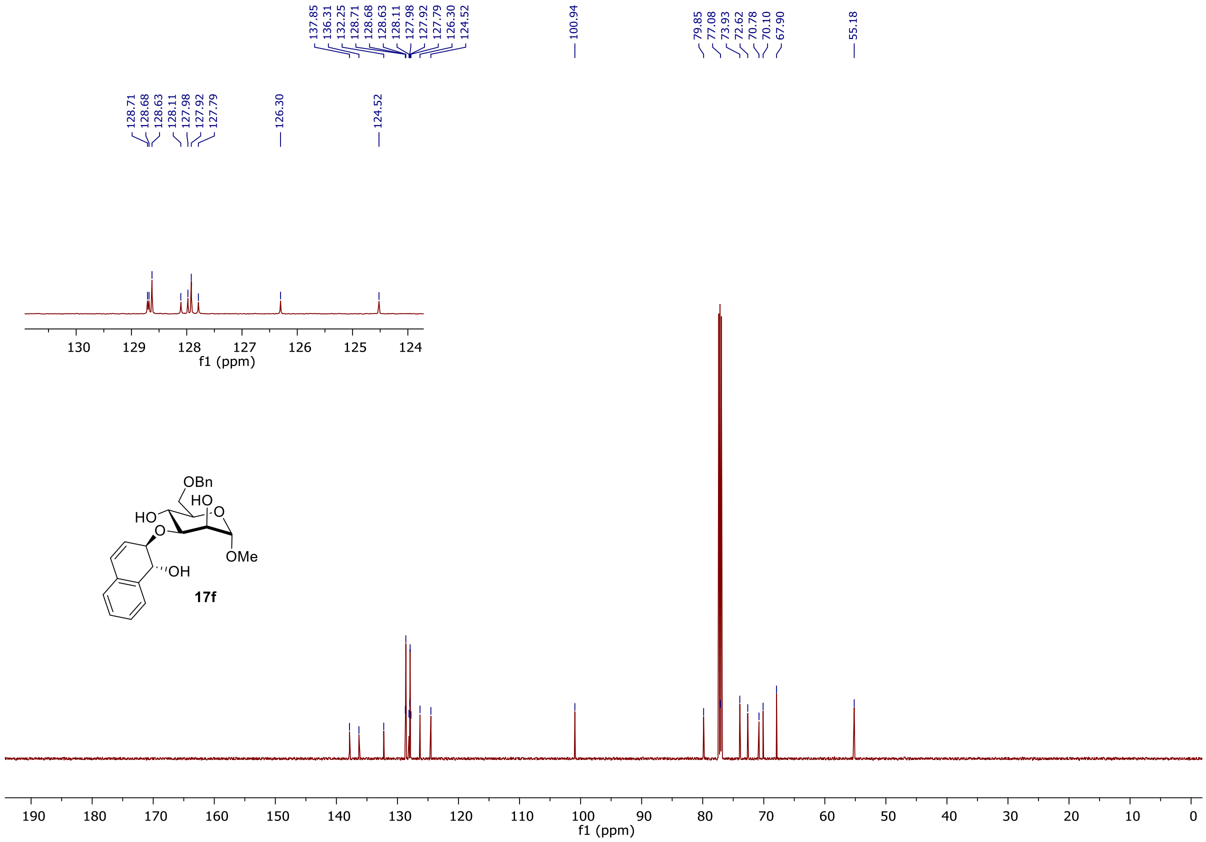

Supplementary Figure 131. ^{13}C spectra for **17f**

i

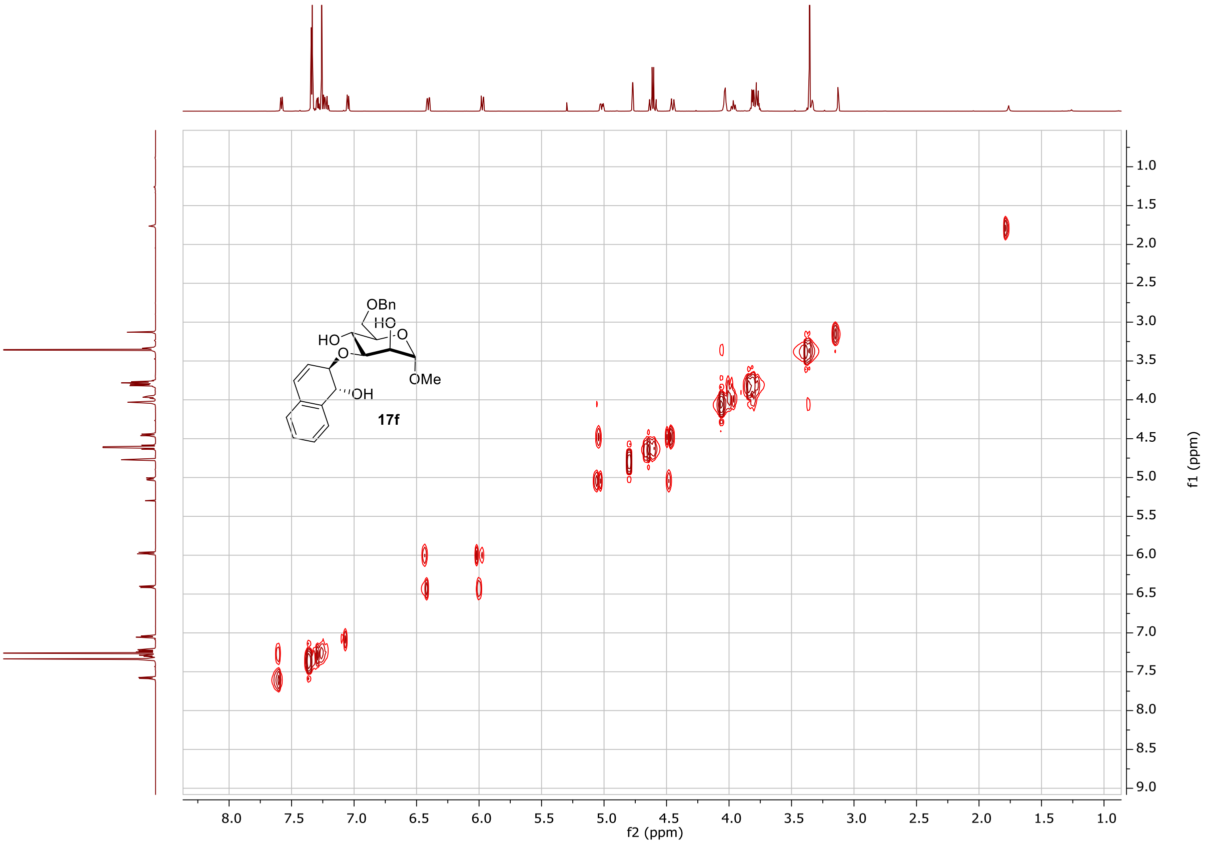

Supplementary Figure 132. COSY spectra for **17f**

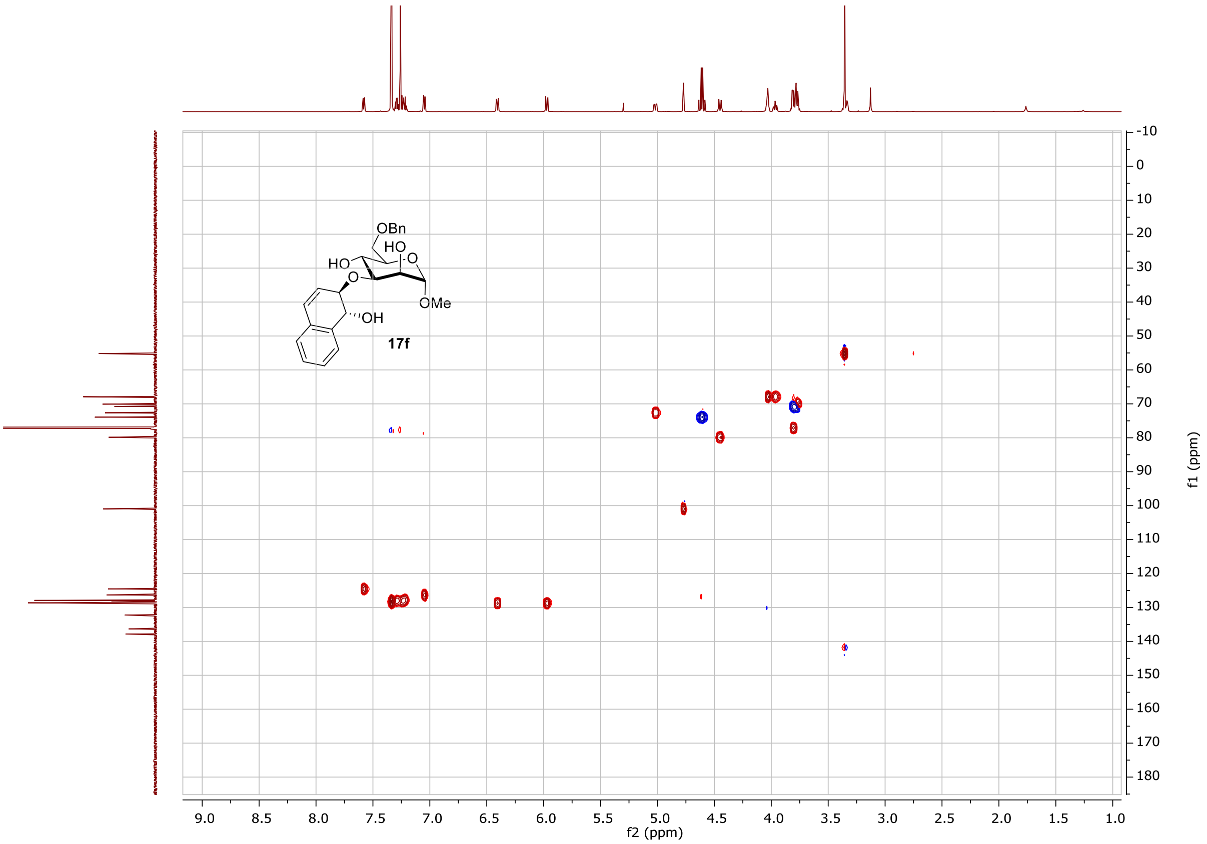

Supplementary Figure 133. HSQC spectra for **17f**

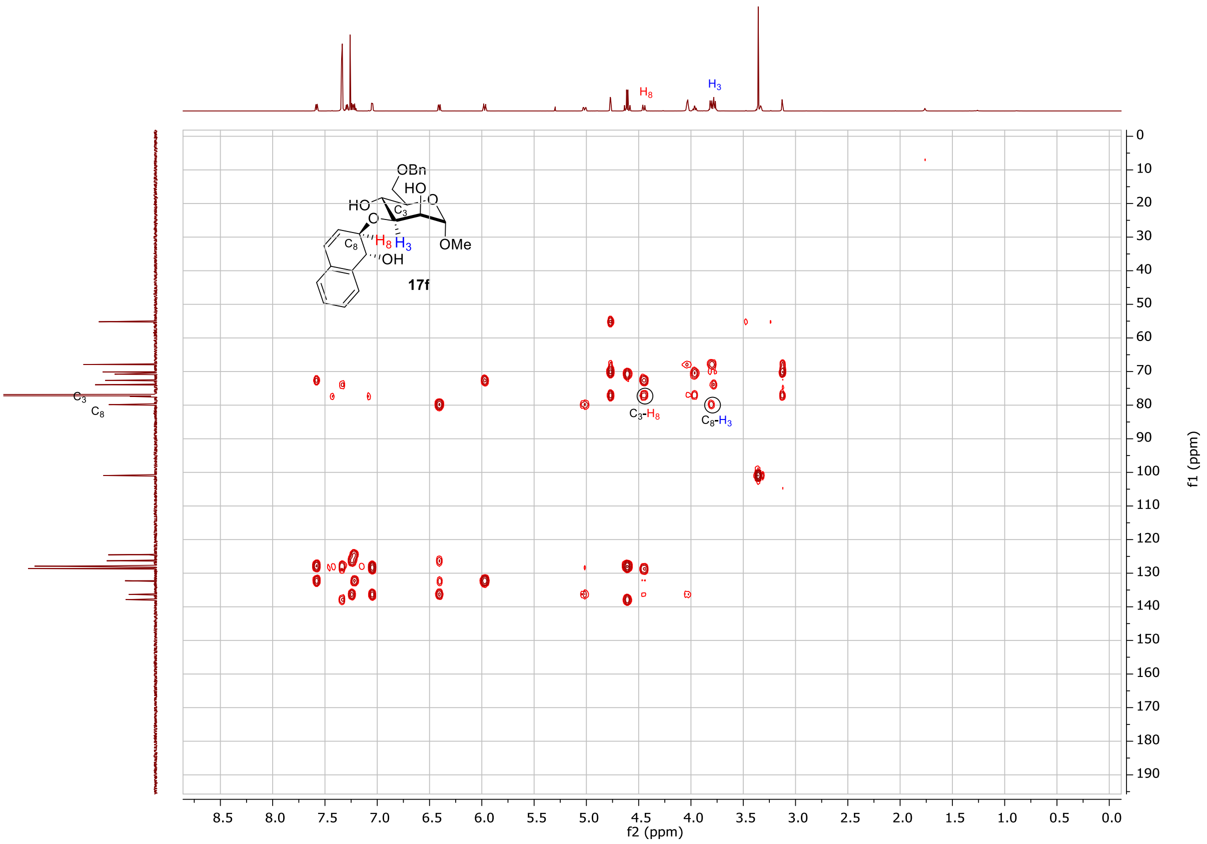

Supplementary Figure 134. HMBC spectra for **17f**

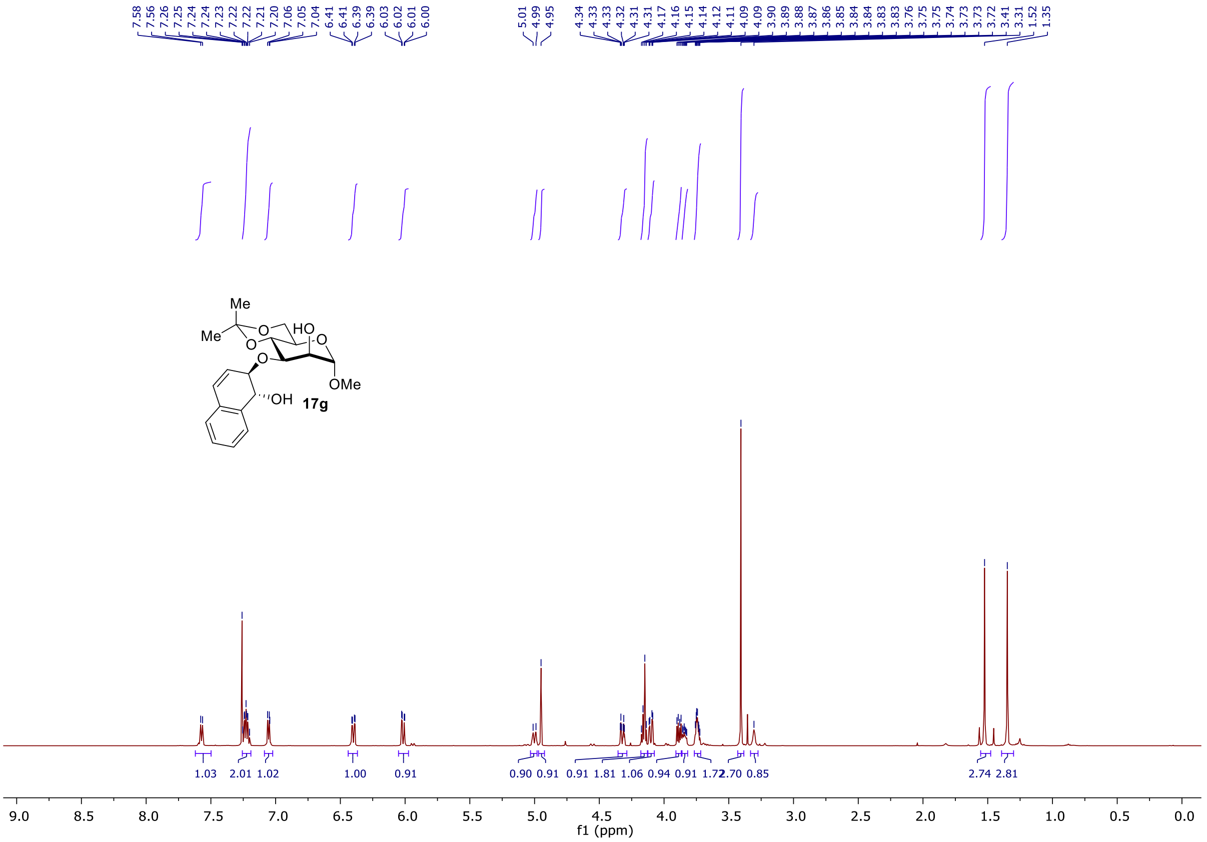

Supplementary Figure 135. ^{1}H spectra for **17g**

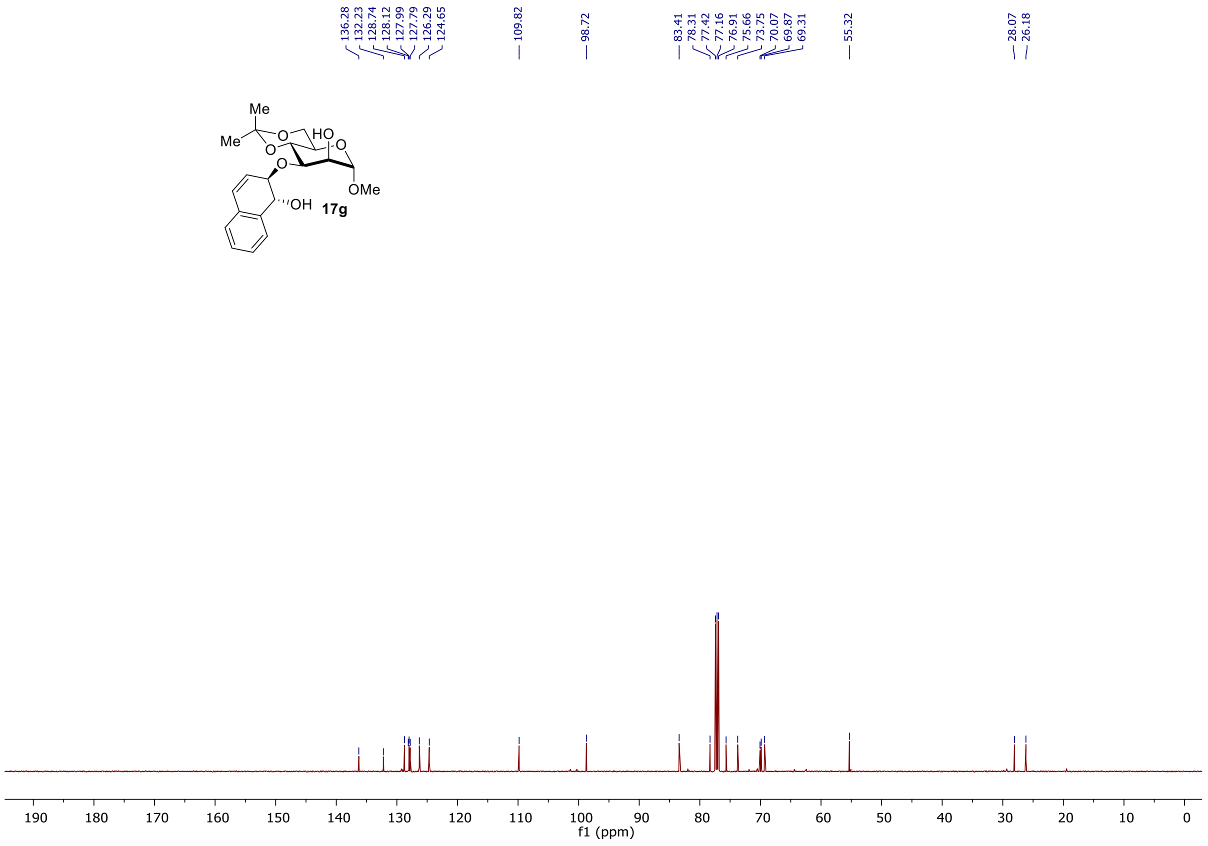

Supplementary Figure 136. ^{13}C spectra for **17g**

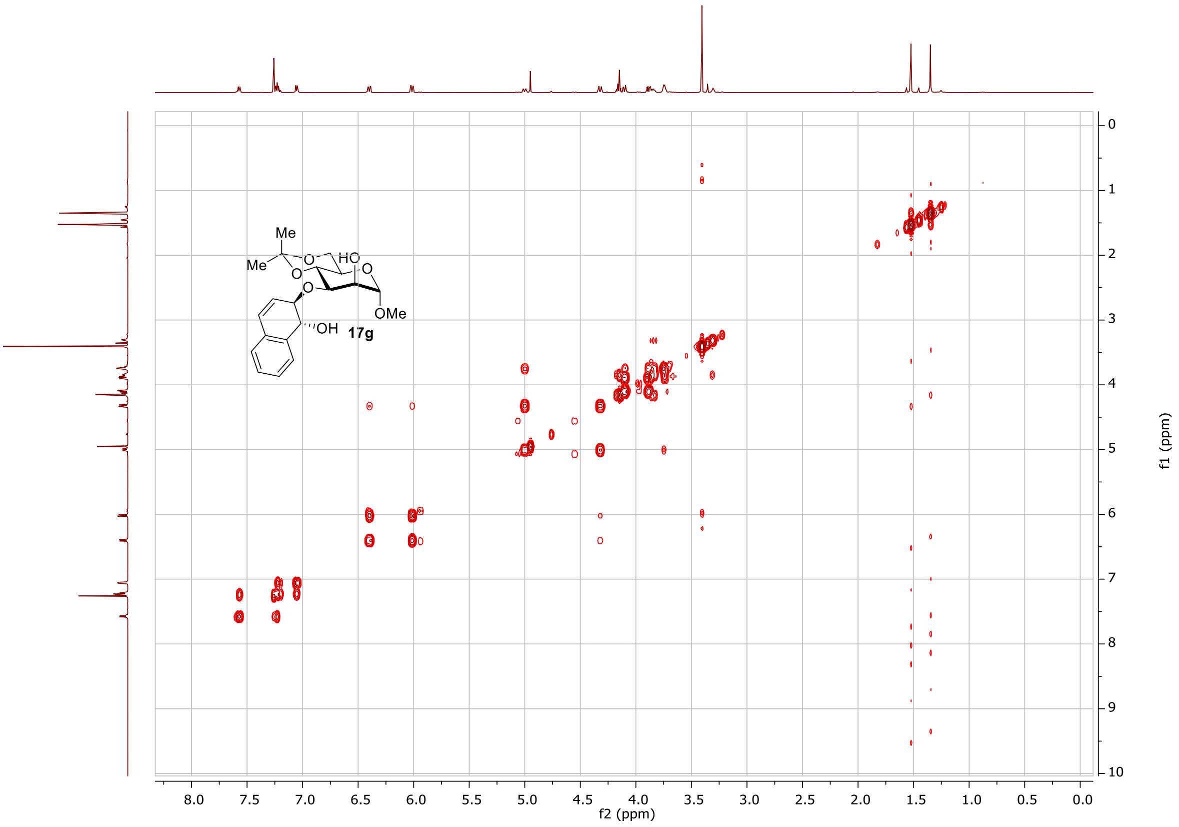

Supplementary Figure 137. COSY spectra for **17g**

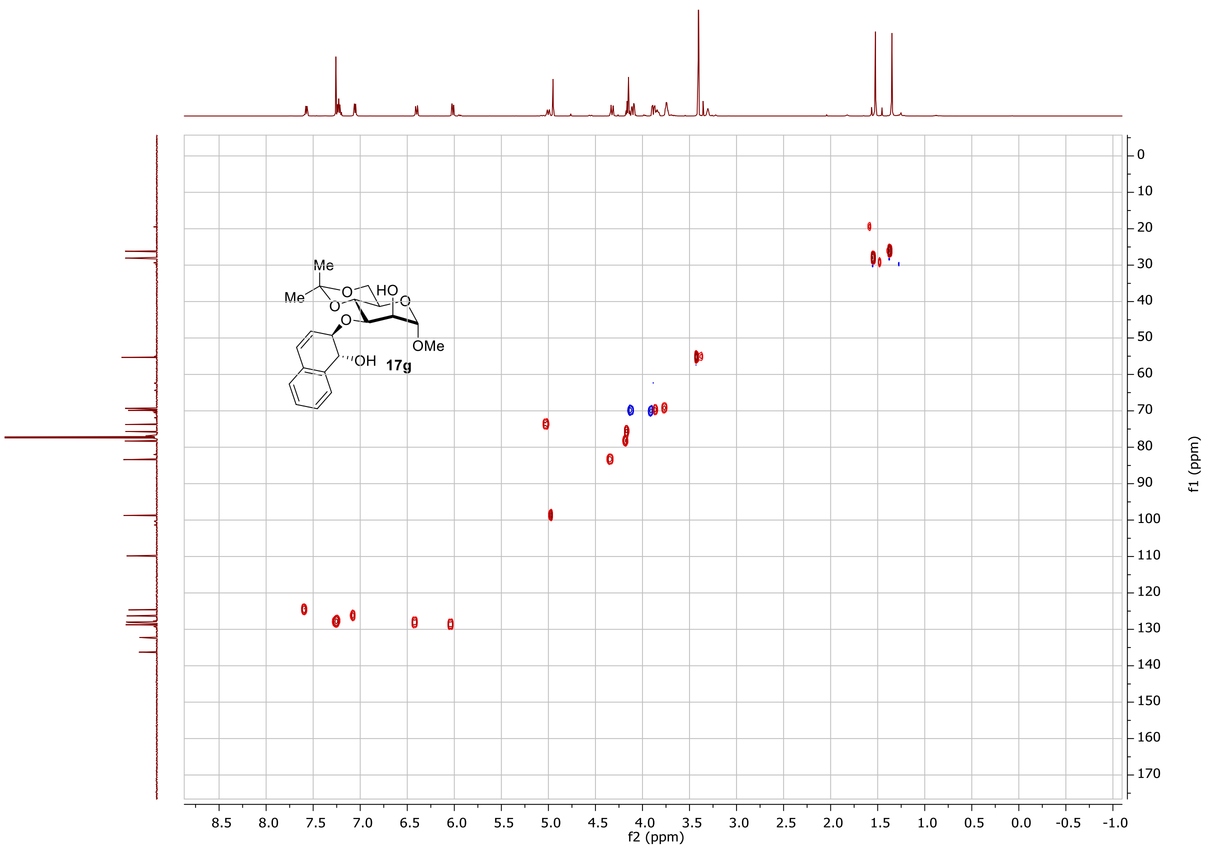

Supplementary Figure 138. HSQC spectra for **17g**

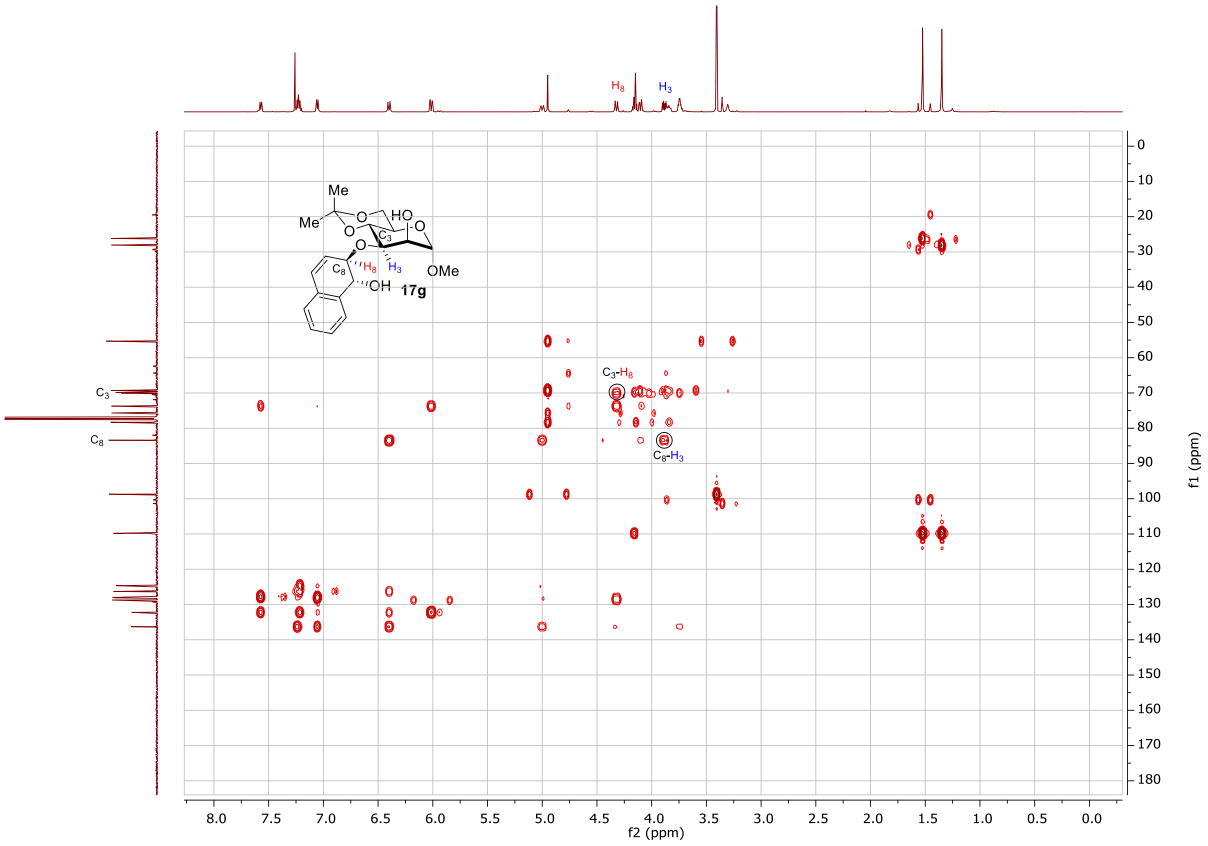

Supplementary Figure 139. HMBC spectra for **17g**

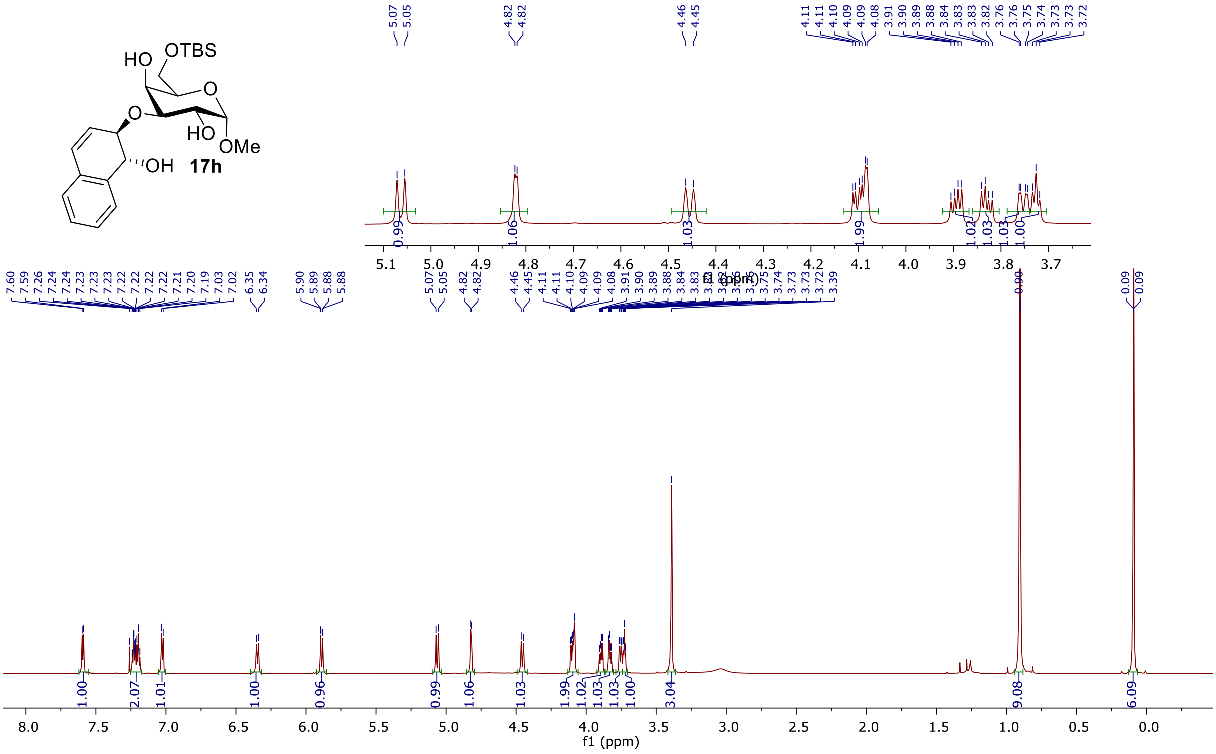

Supplementary Figure 140. ^{1}H spectra for **17h**

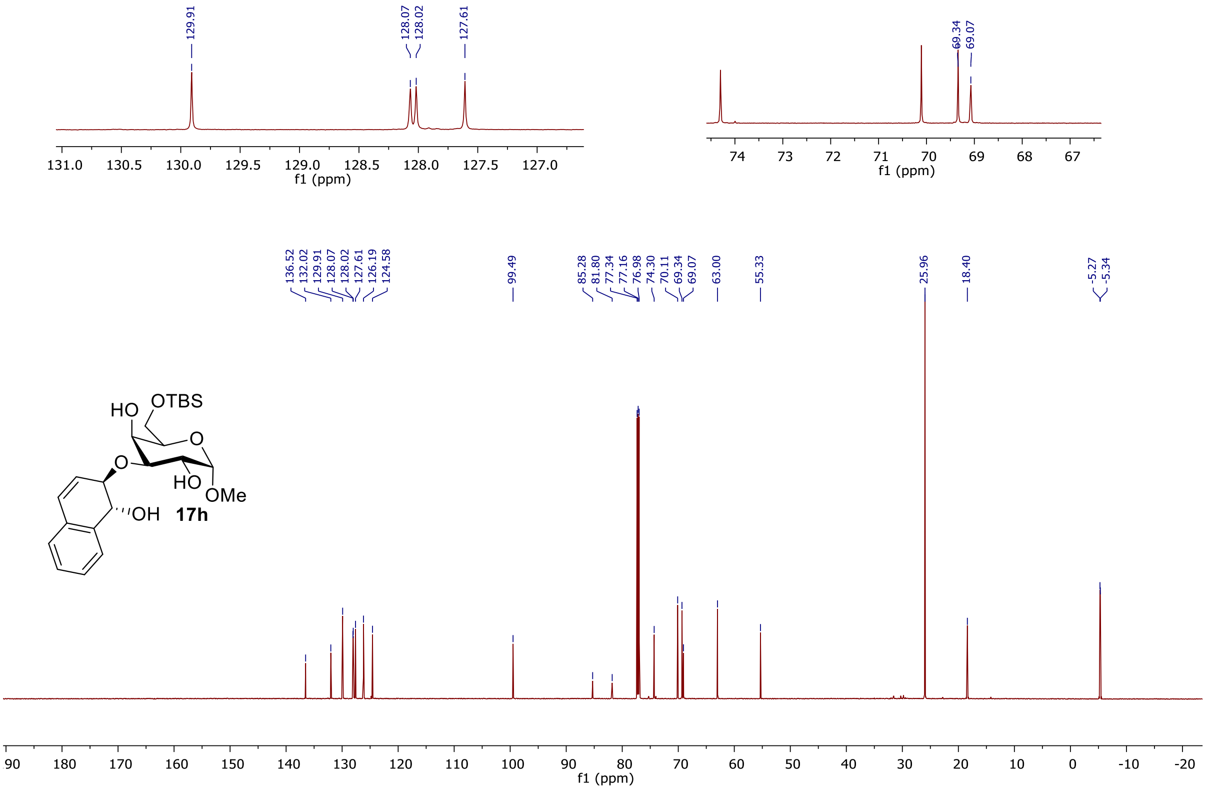

Supplementary Figure 141. ^{13}C spectra for **17h**

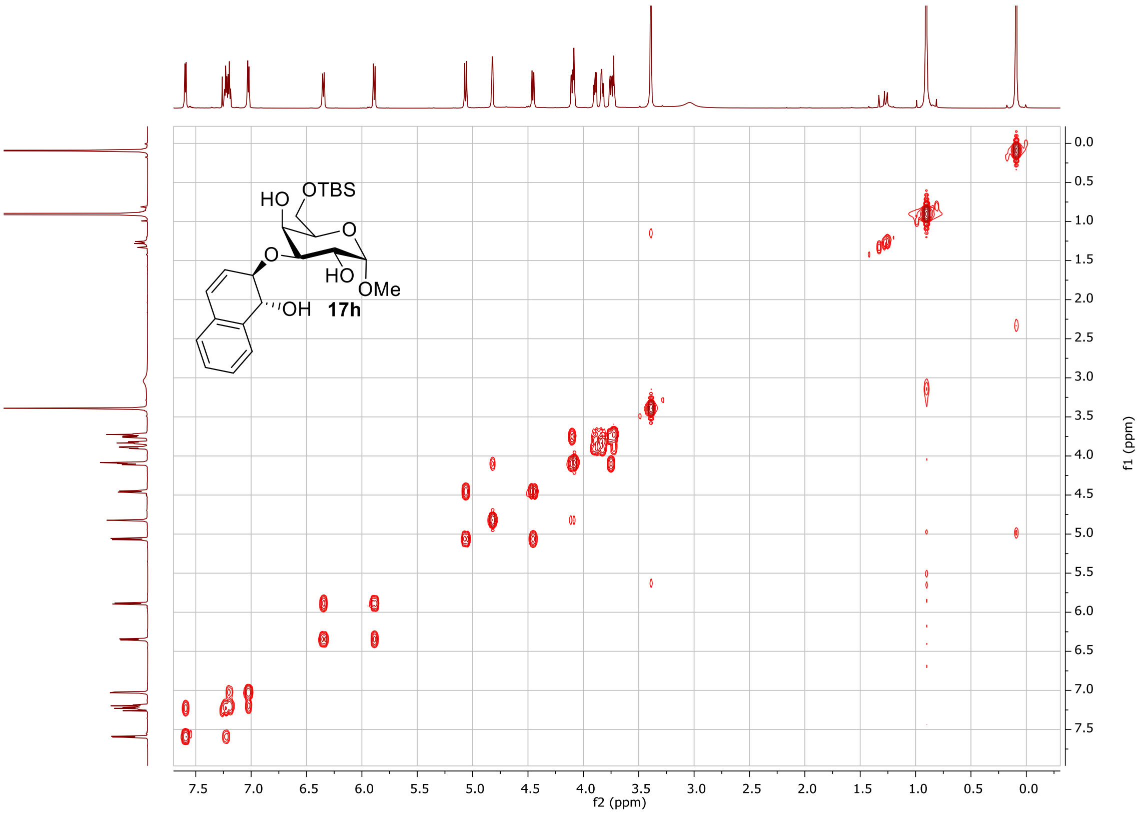

Supplementary Figure S142. COSY spectra for **17h**

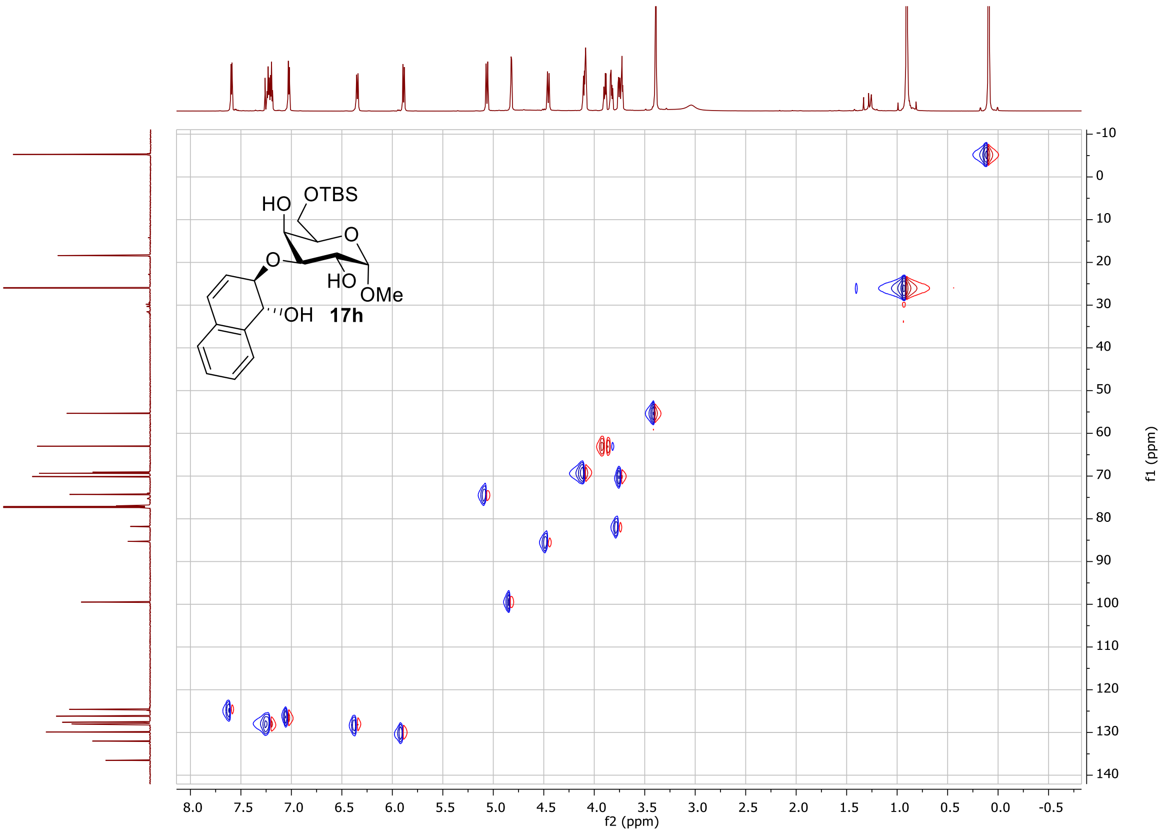

Supplementary Figure S143. HSQC spectra for **17h**

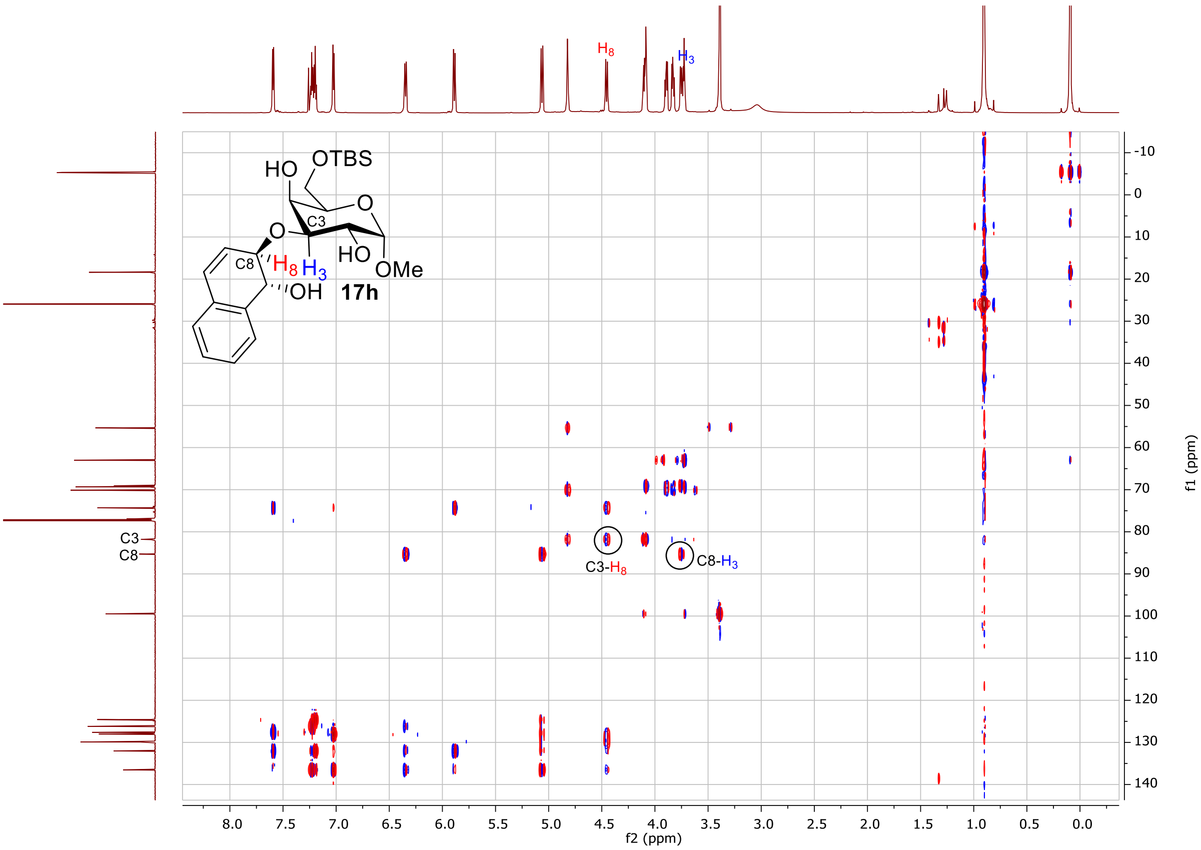

Supplementary Figure 144. HMBC spectra for **17h**

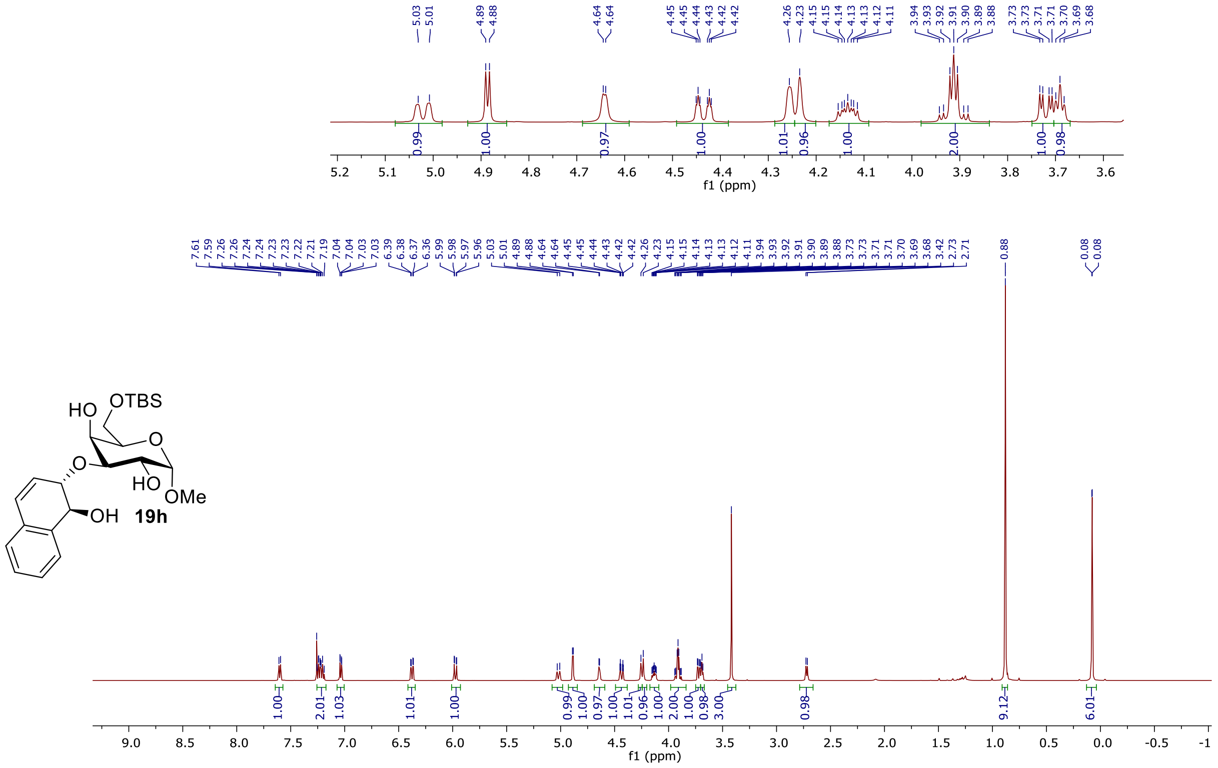

Supplementary Figure 145. ^{1}H spectra for **19h**

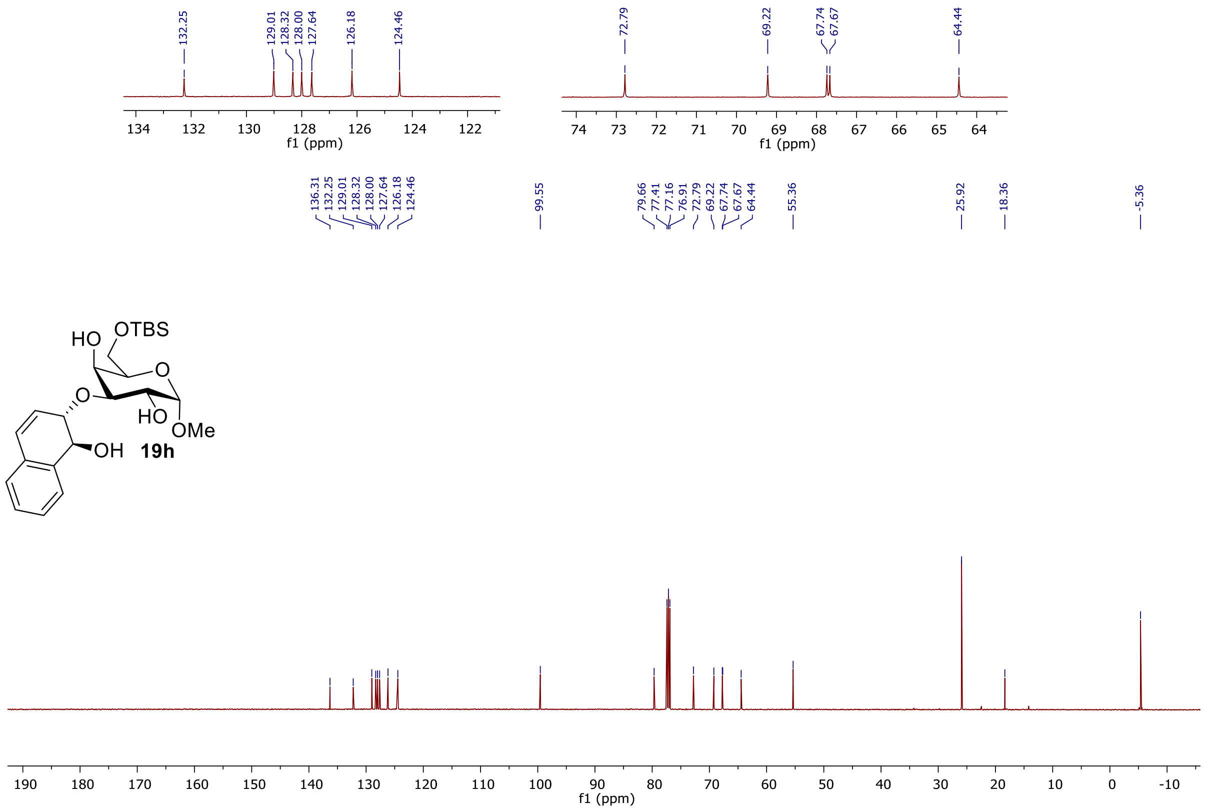

Supplementary Figure 146. ^{13}C spectra for **19h**

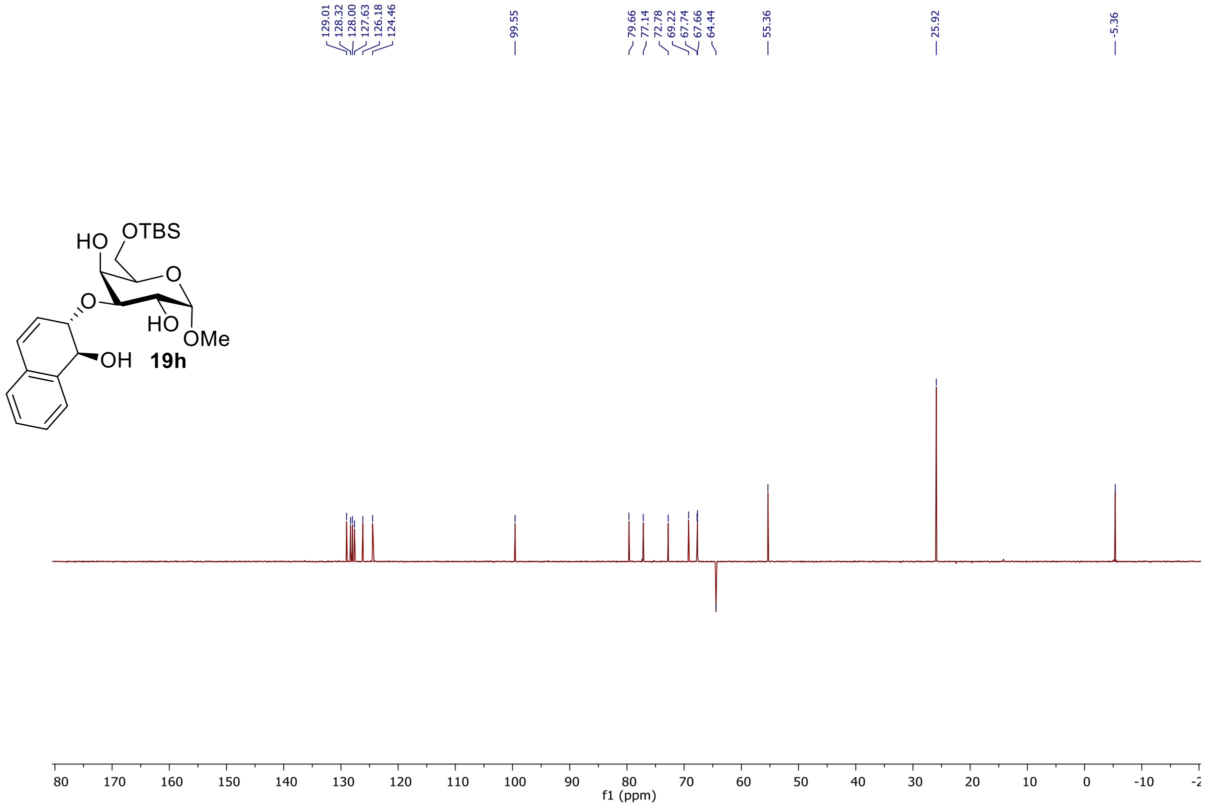

Supplementary Figure 147. DEPT spectra for **19h**

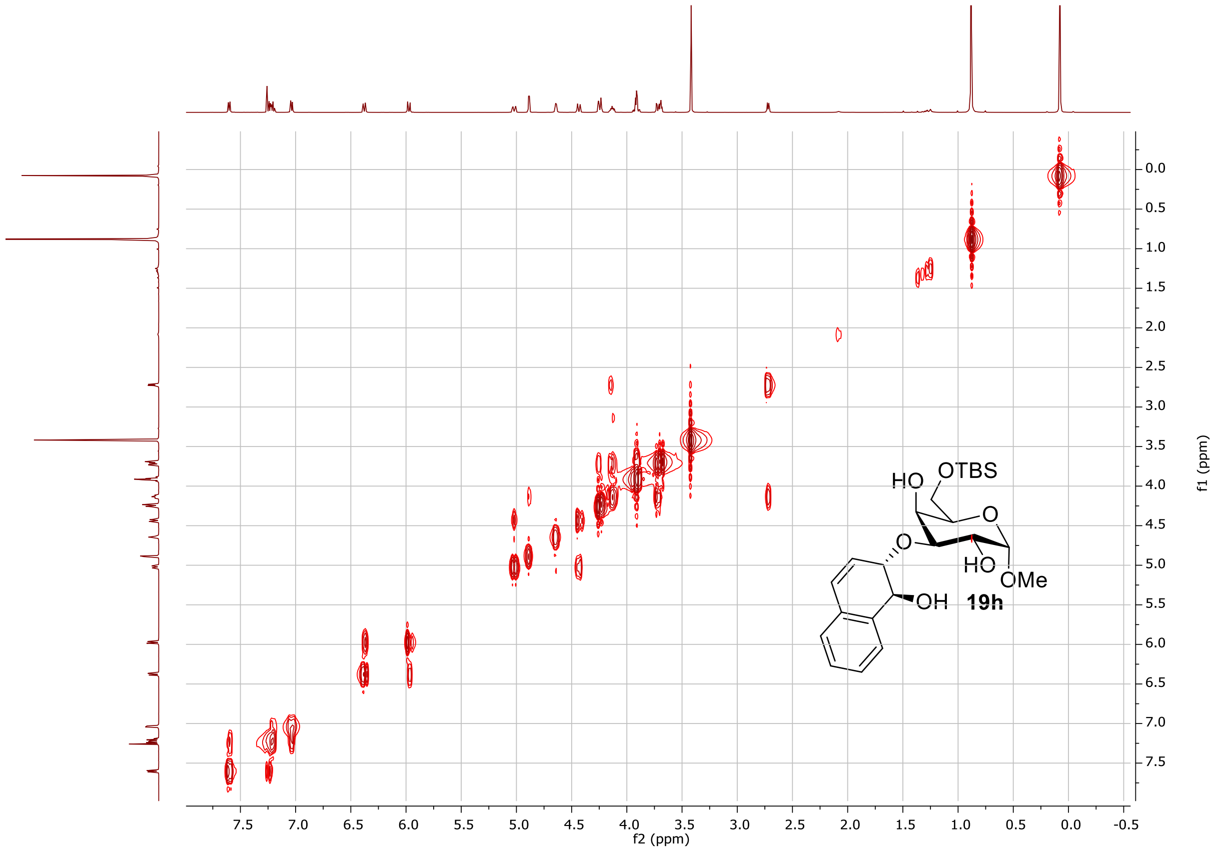

Supplementary Figure 148. COSY spectra for **19h**

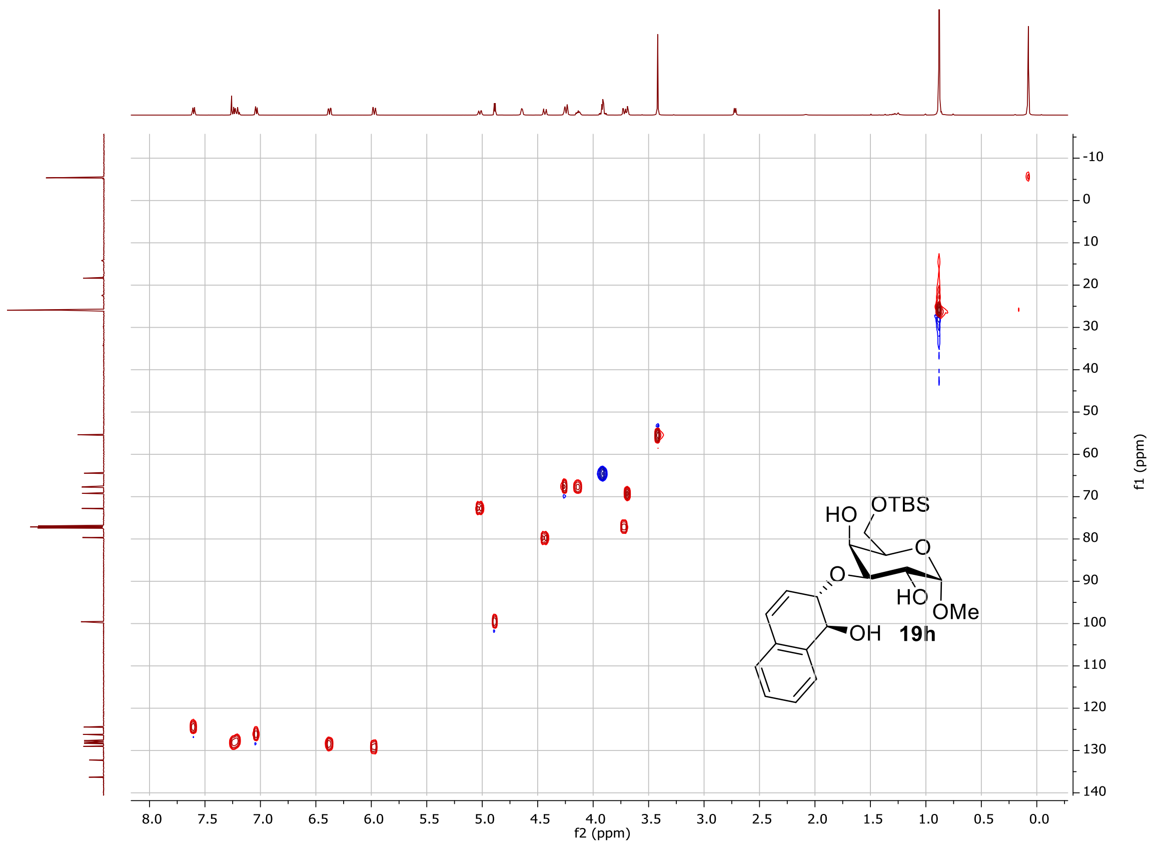

Supplementary Figure 149. HSQC spectra for **19h**

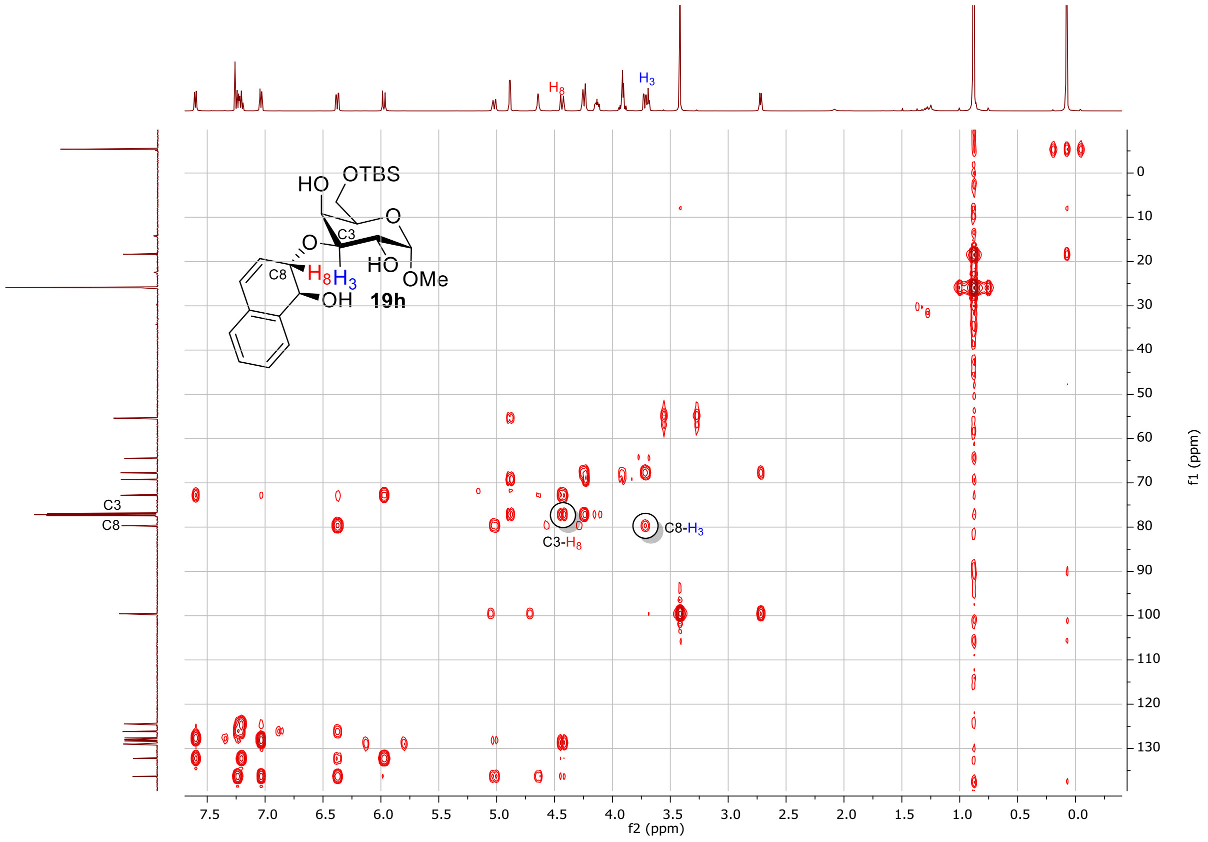

Supplementary Figure 150. HMBC spectra for **19h**

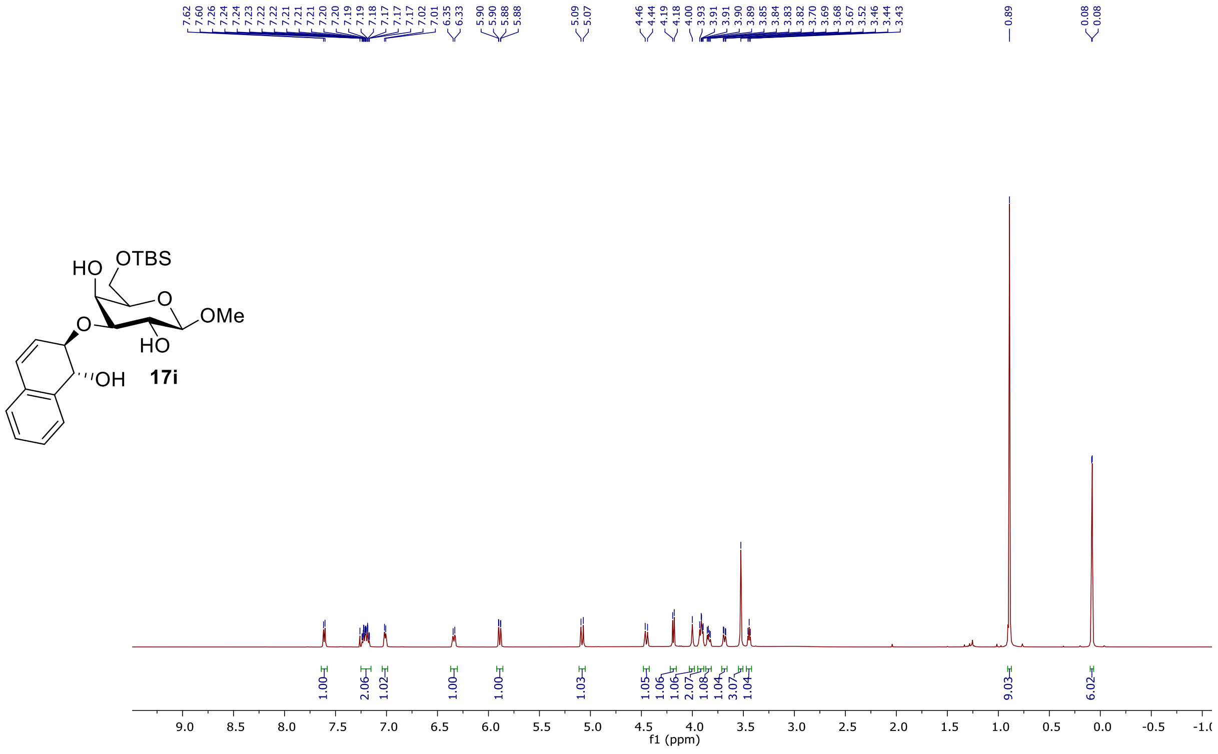

Supplementary Figure 151. ^{1}H spectra for **17i**

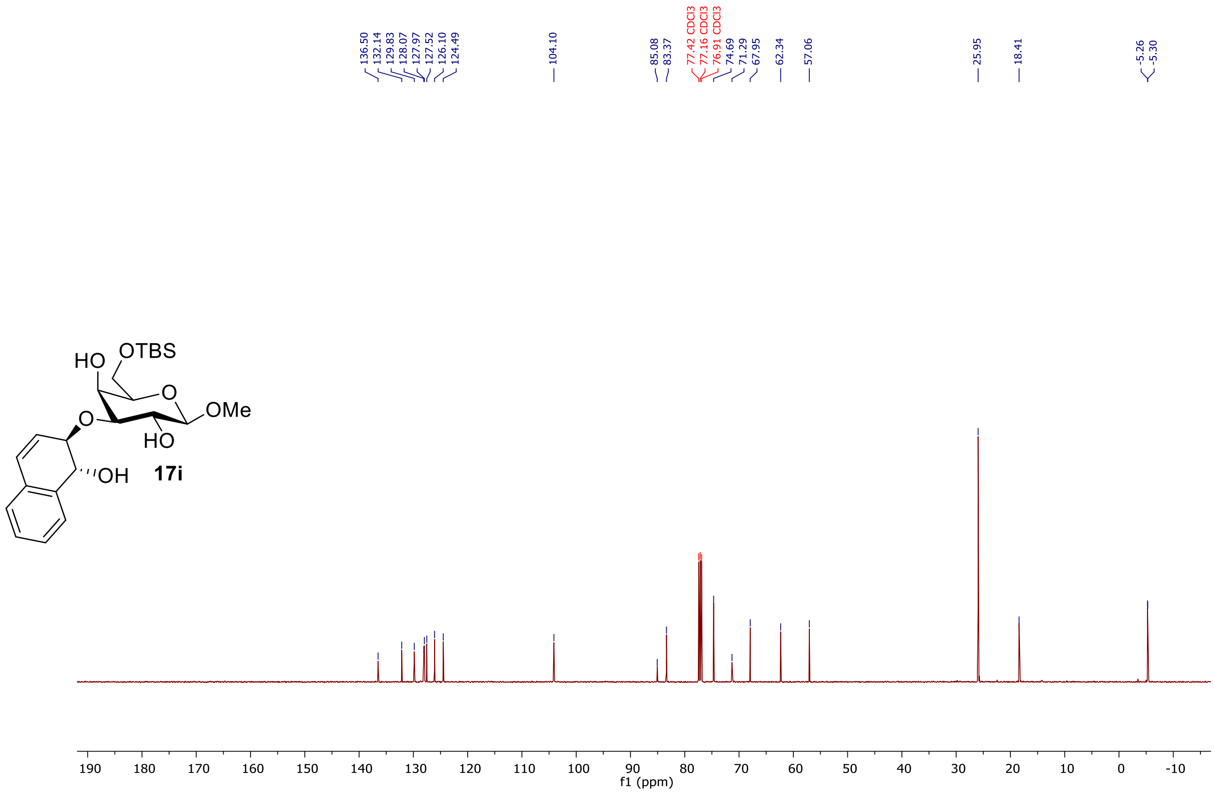

Supplementary Figure 152. ^{13}C spectra for **17i**

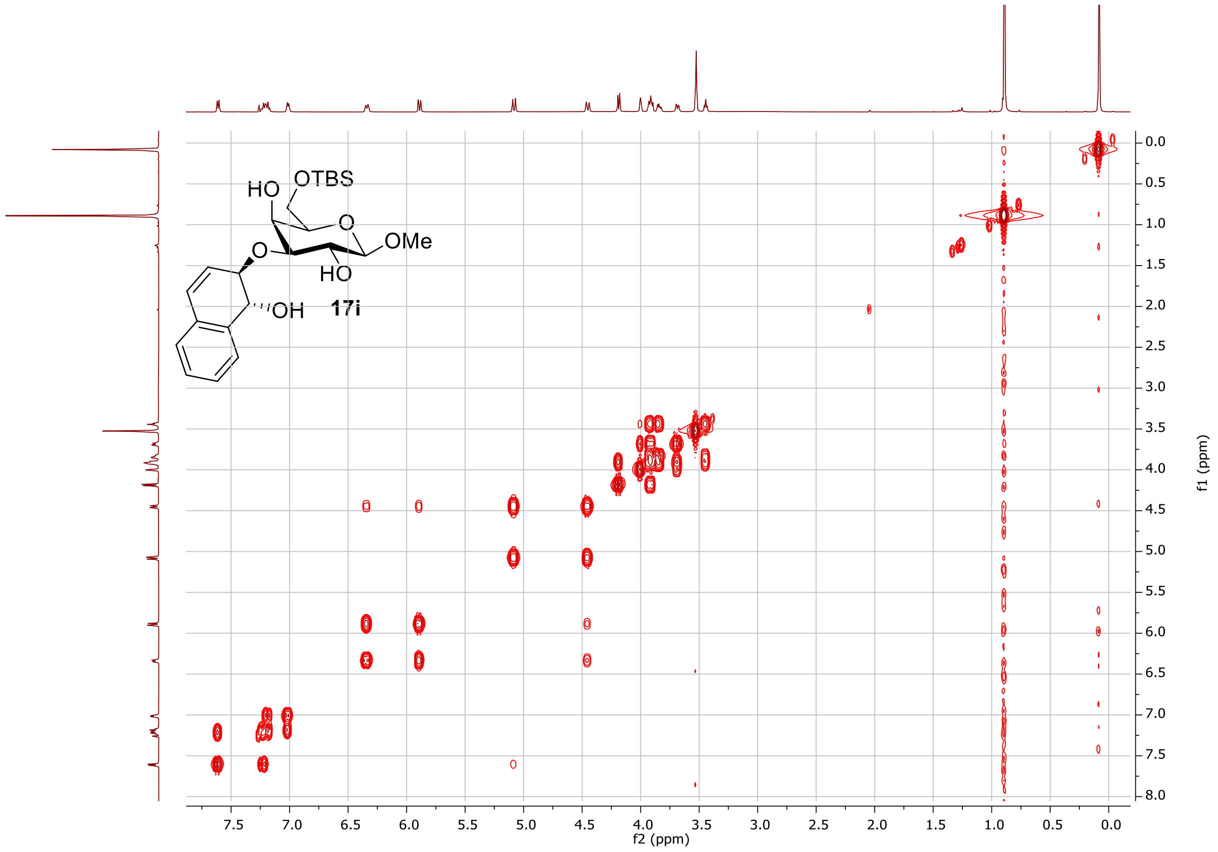

Supplementary Figure 153. COSY spectra for **17i**

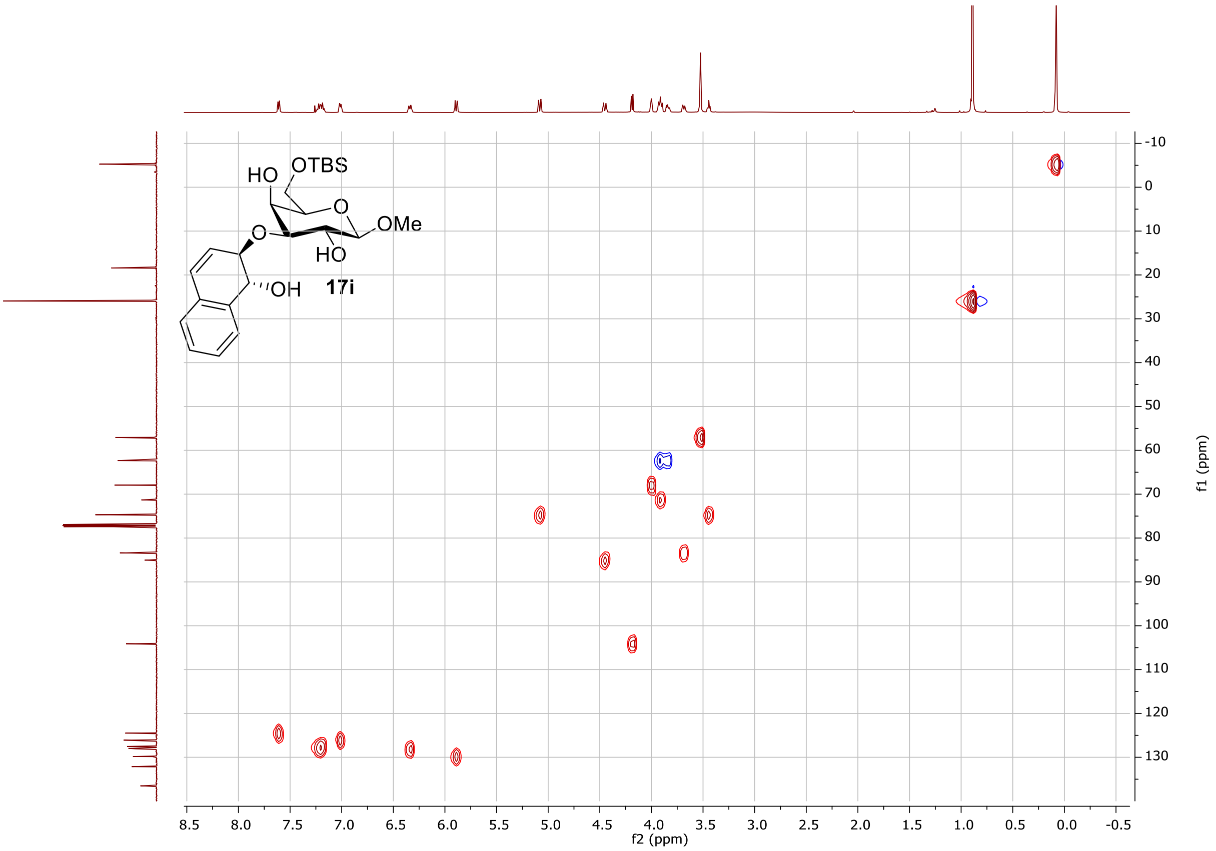

Supplementary Figure 154. HSQC spectra for **17i**

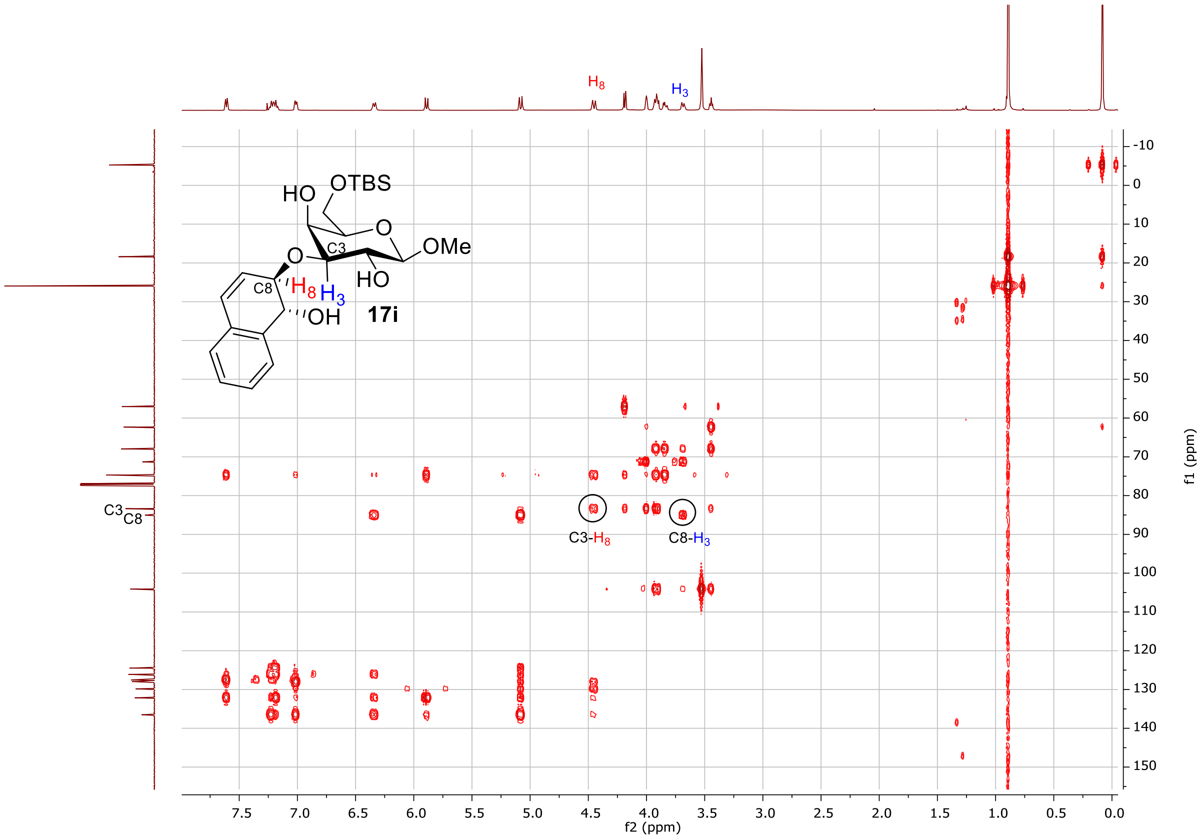

Supplementary Figure 155. HMBC spectra for **17i**

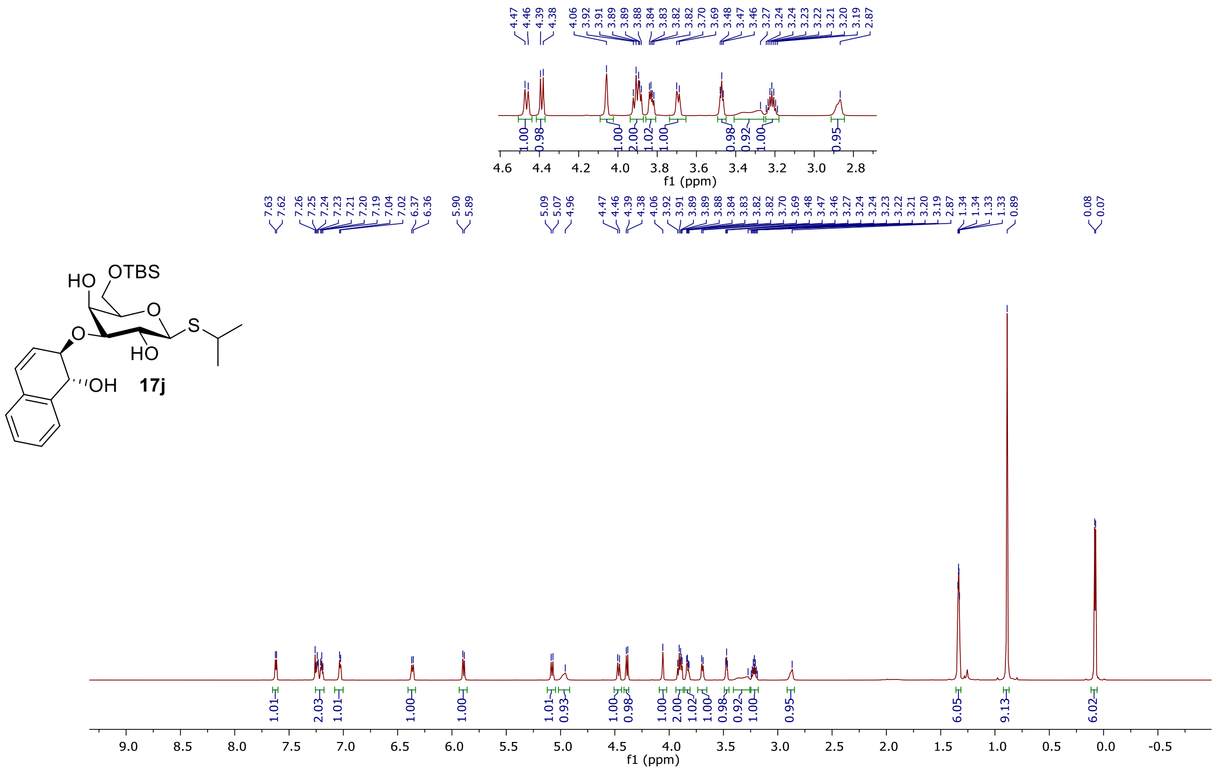

Supplementary Figure 156. ^{1}H spectra for **17j**

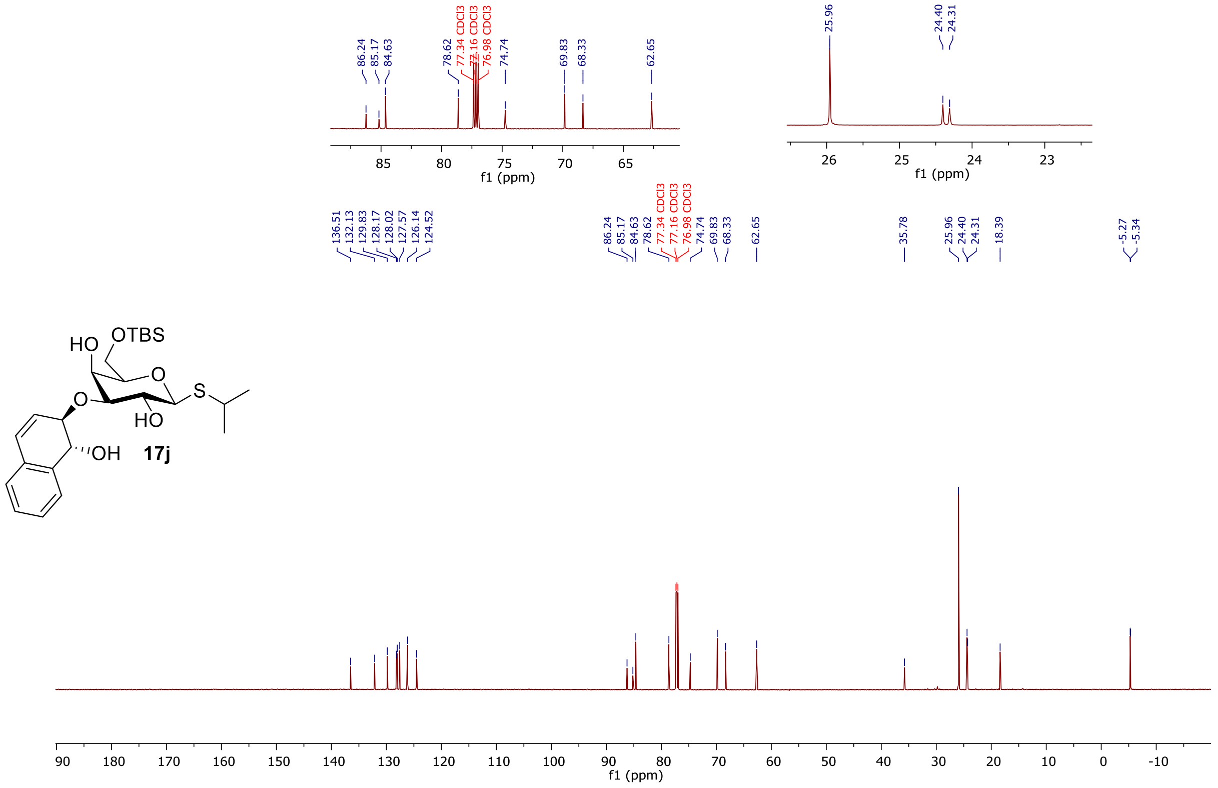

Supplementary Figure 157. ^{13}C spectra for **17j**

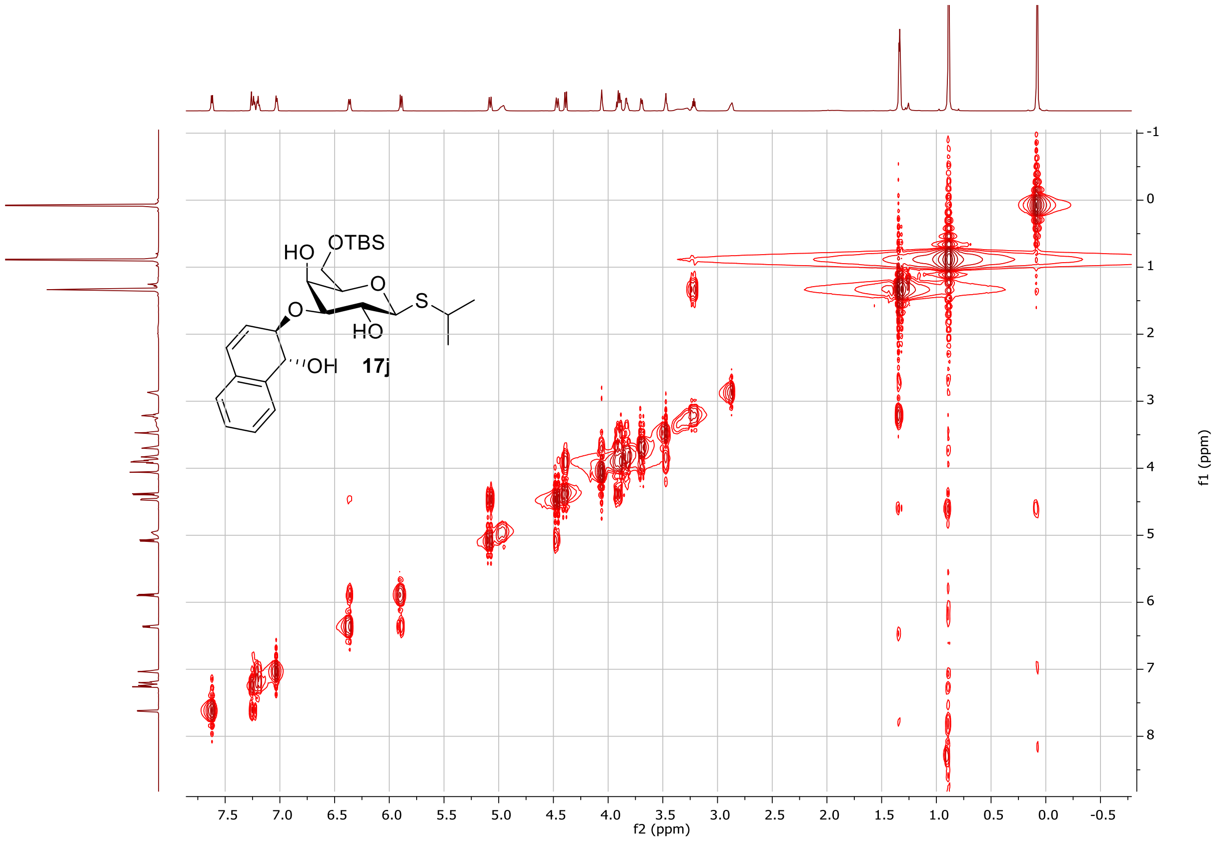

Supplementary Figure 158. COSY spectra for **17j**

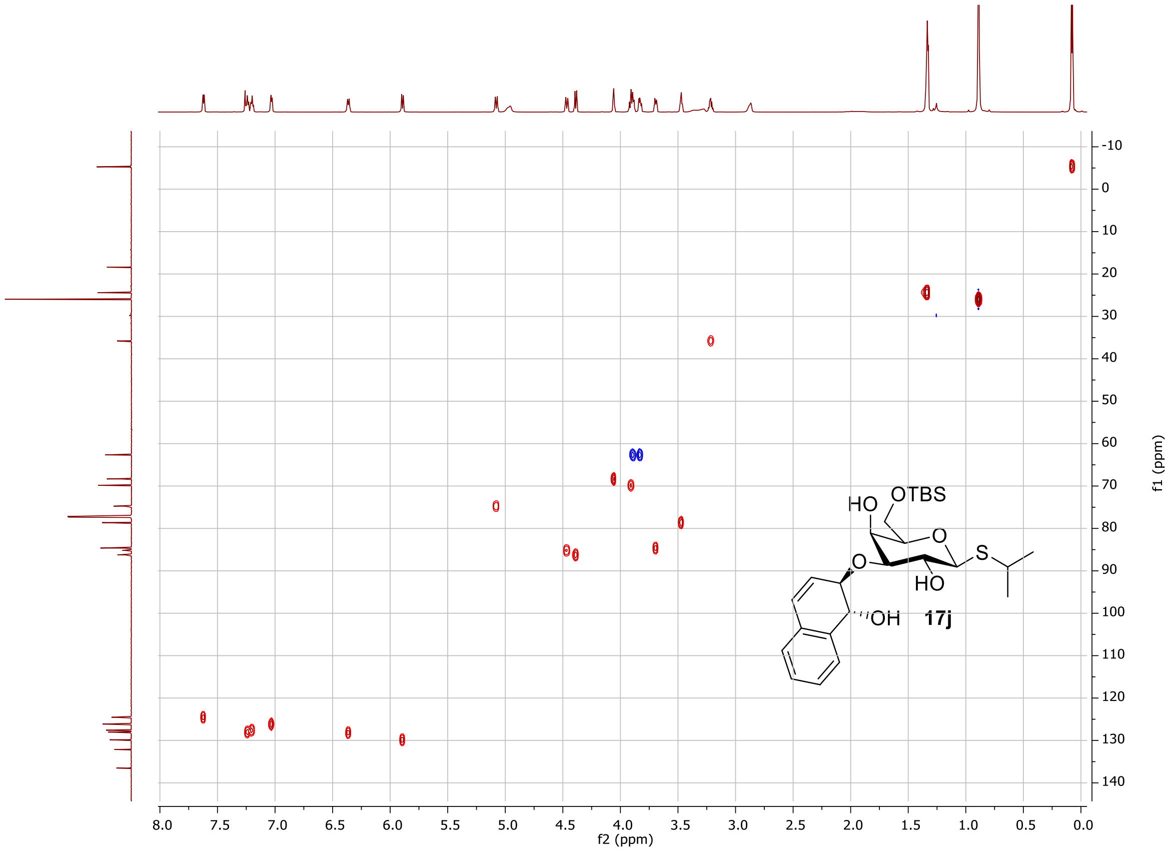

Supplementary Figure 159. HSQC spectra for **17j**

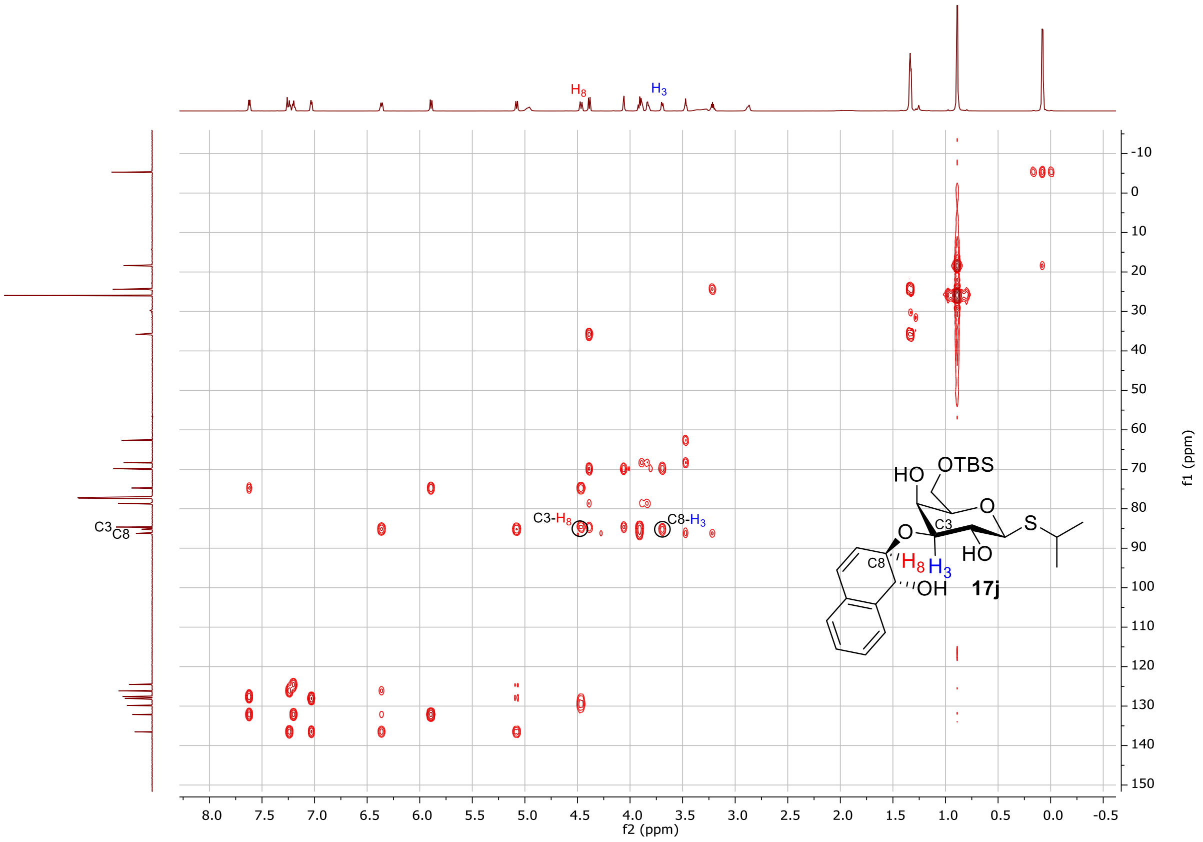

Supplementary Figure 160. HMBC spectra for **17j**

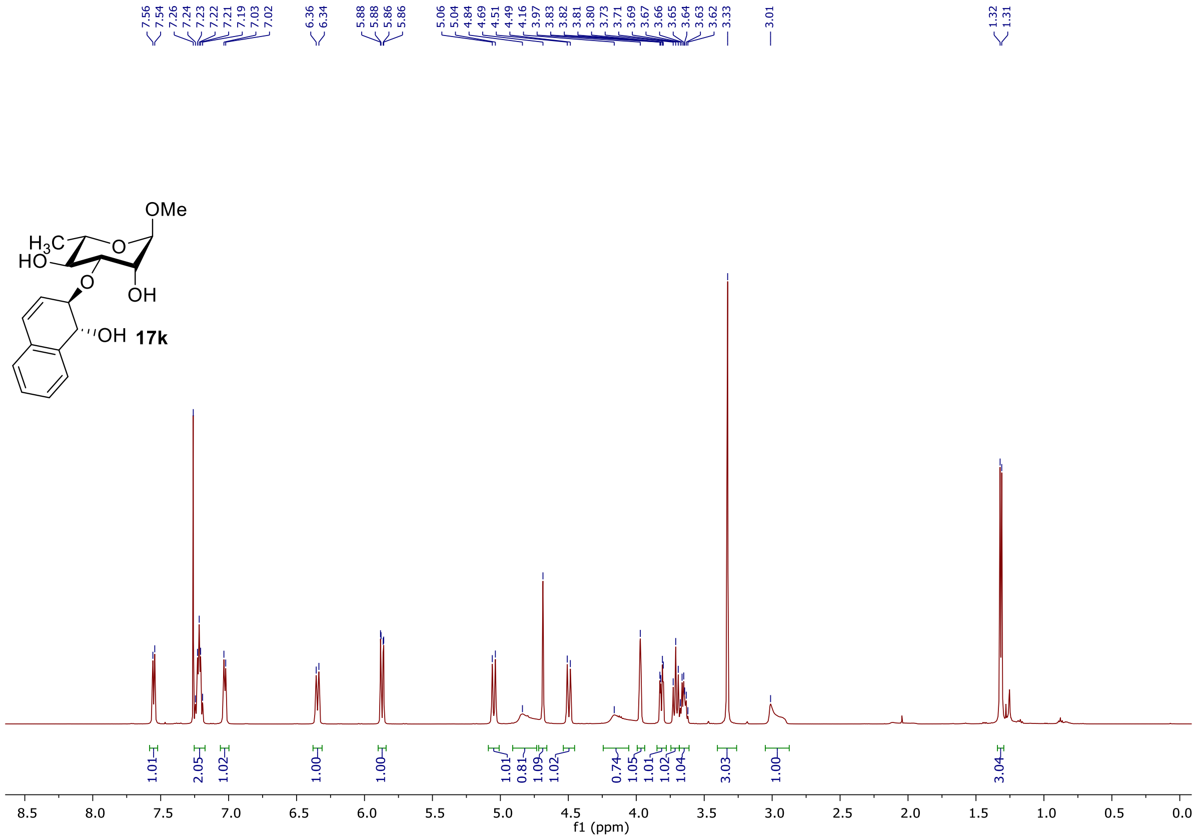

Supplementary Figure 161. ^{1}H spectra for **17k**

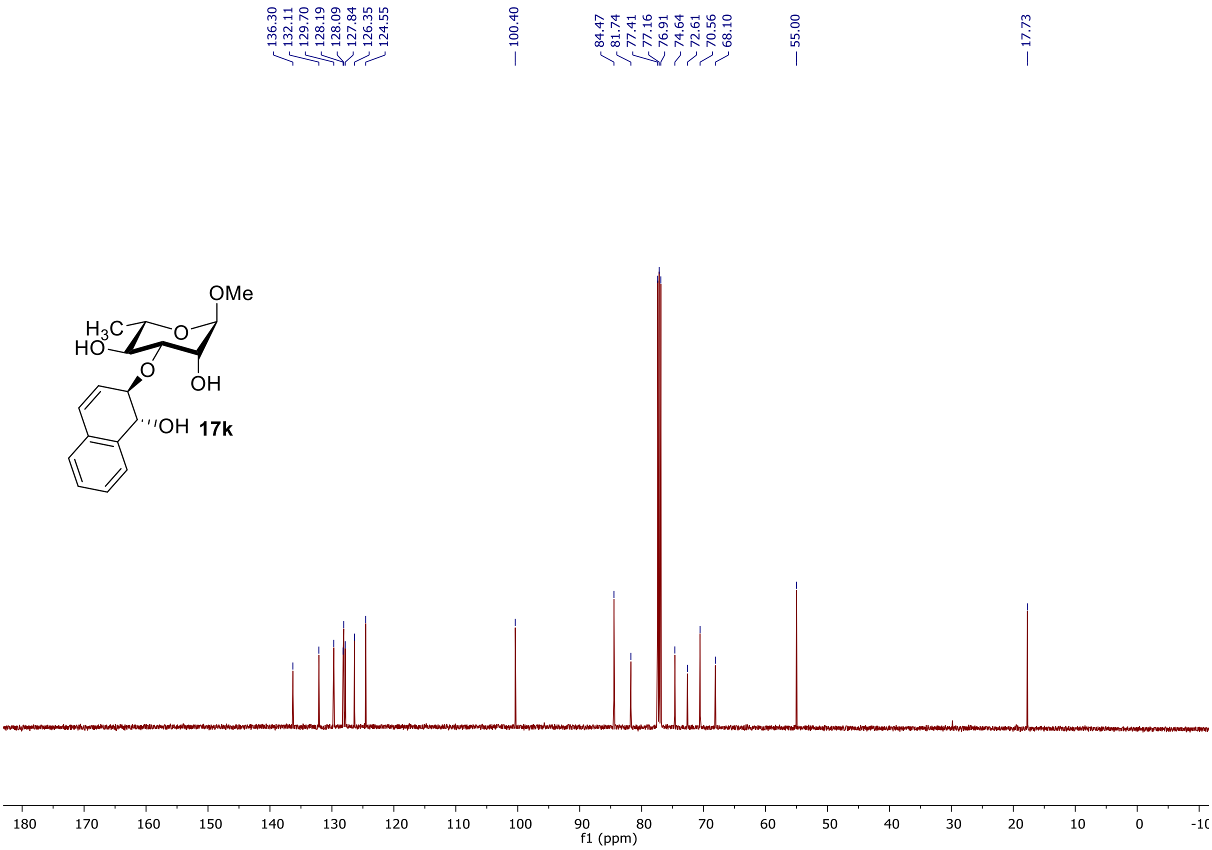

Supplementary Figure 162. ^{13}C spectra for **17k**

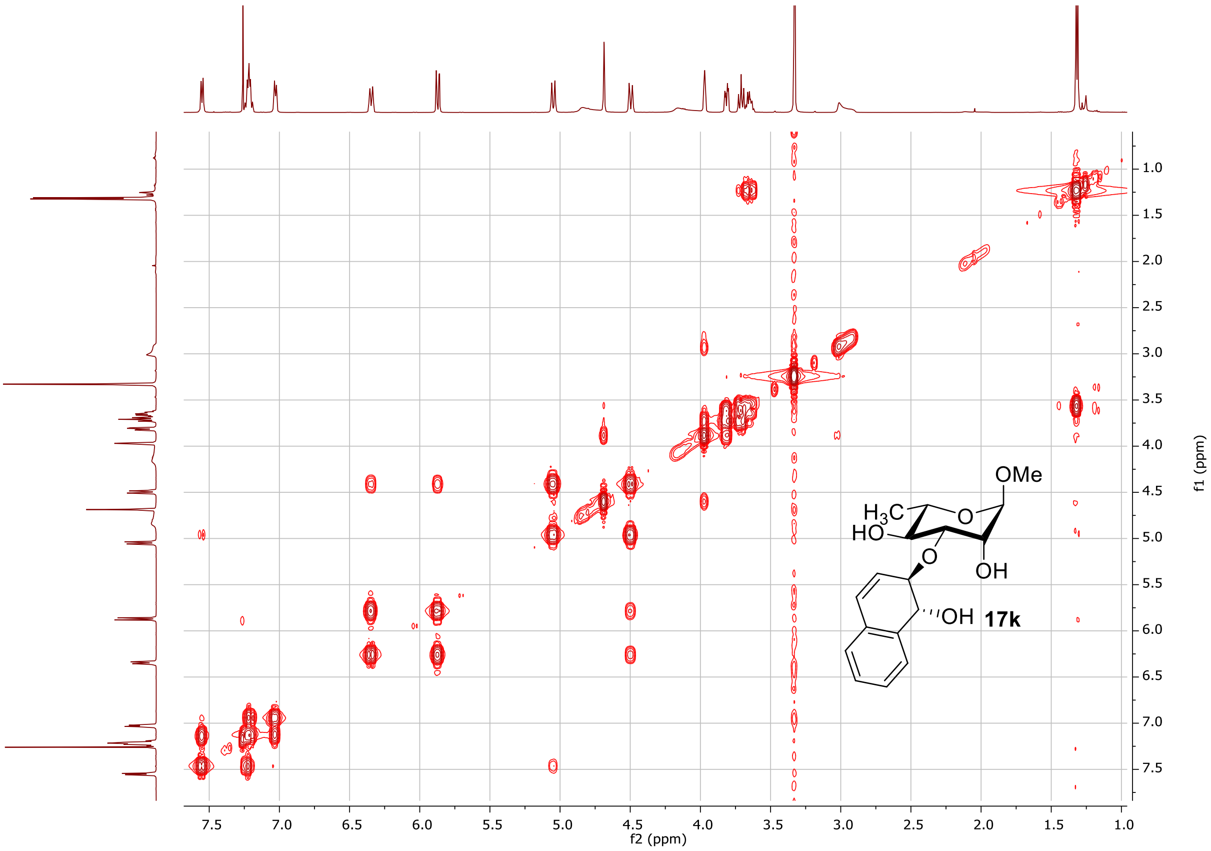

Supplementary Figure 163. COSY spectra for **17k**

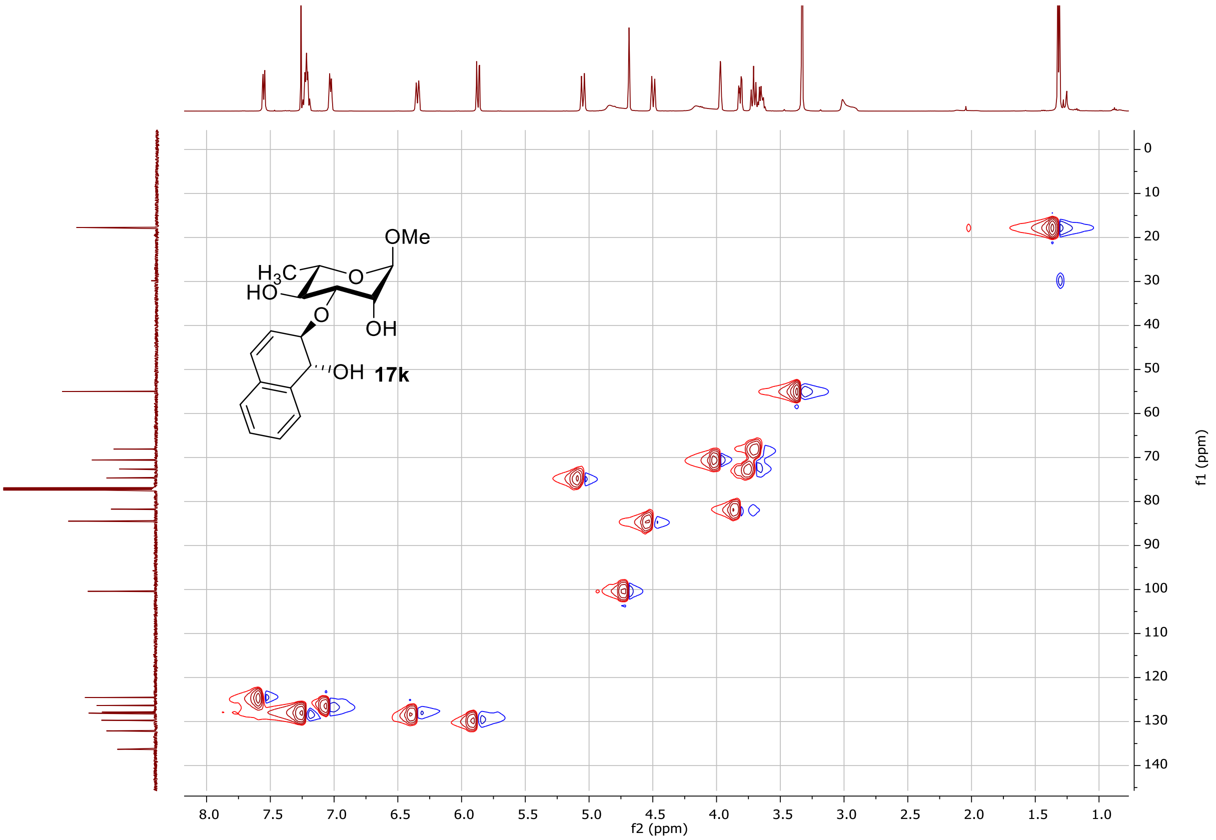

Supplementary Figure 164. HSQC spectra for **17k**

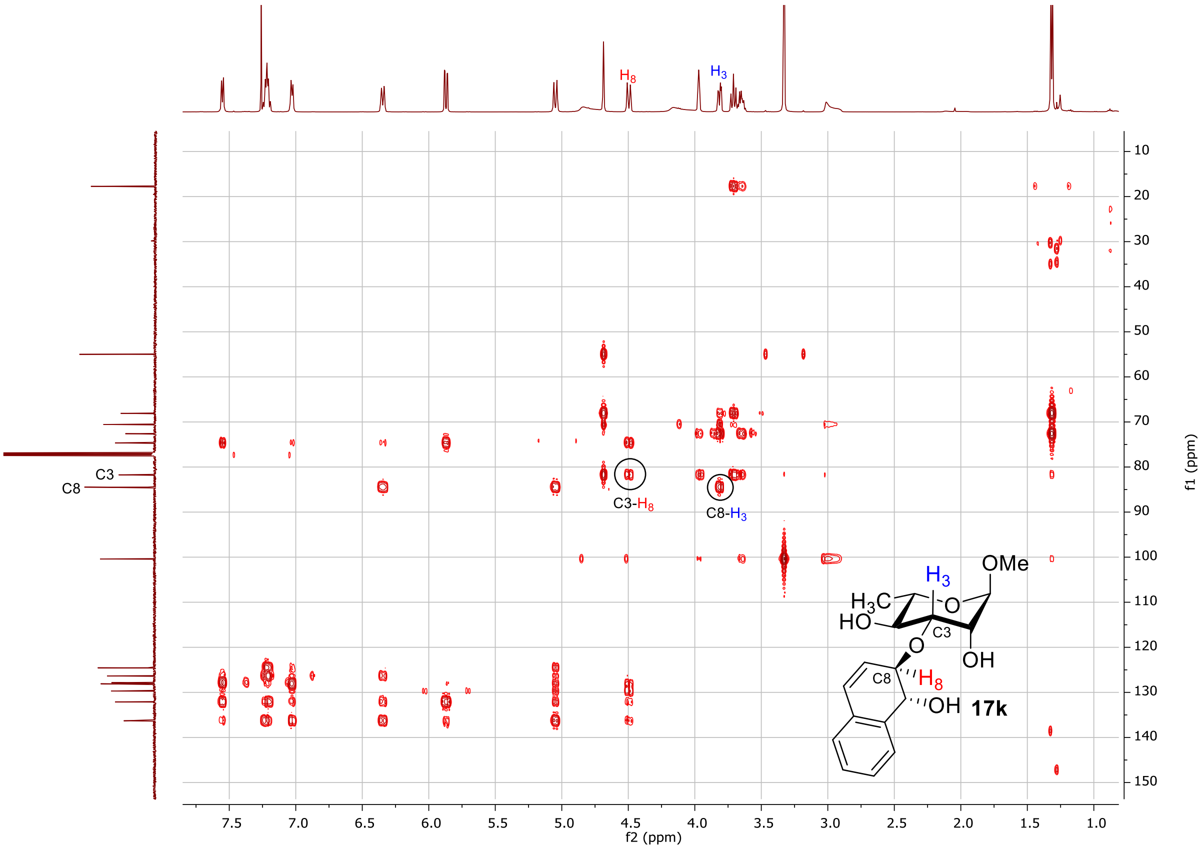

Supplementary Figure 165. HMBC spectra for **17k**

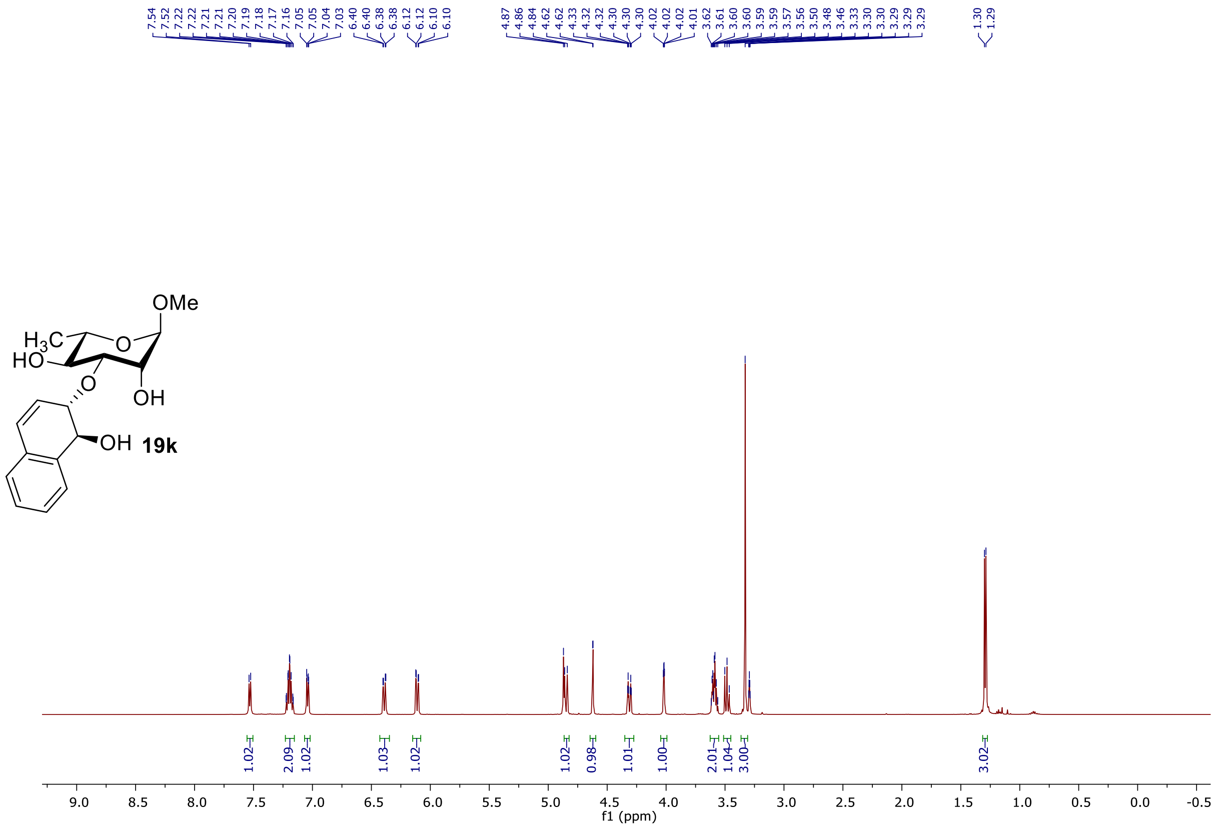

Supplementary Figure 166. ^{1}H spectra for **19k**

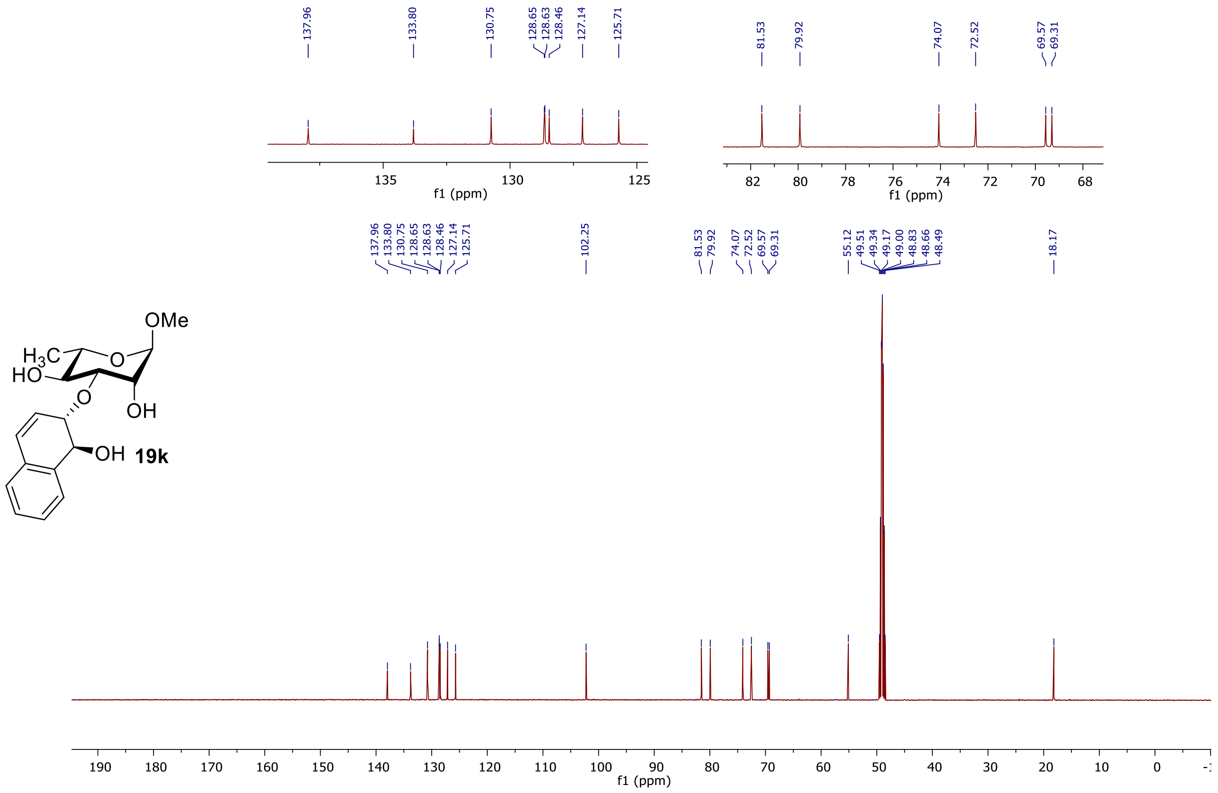

Supplementary Figure 167. ^{13}C spectra for **19k**

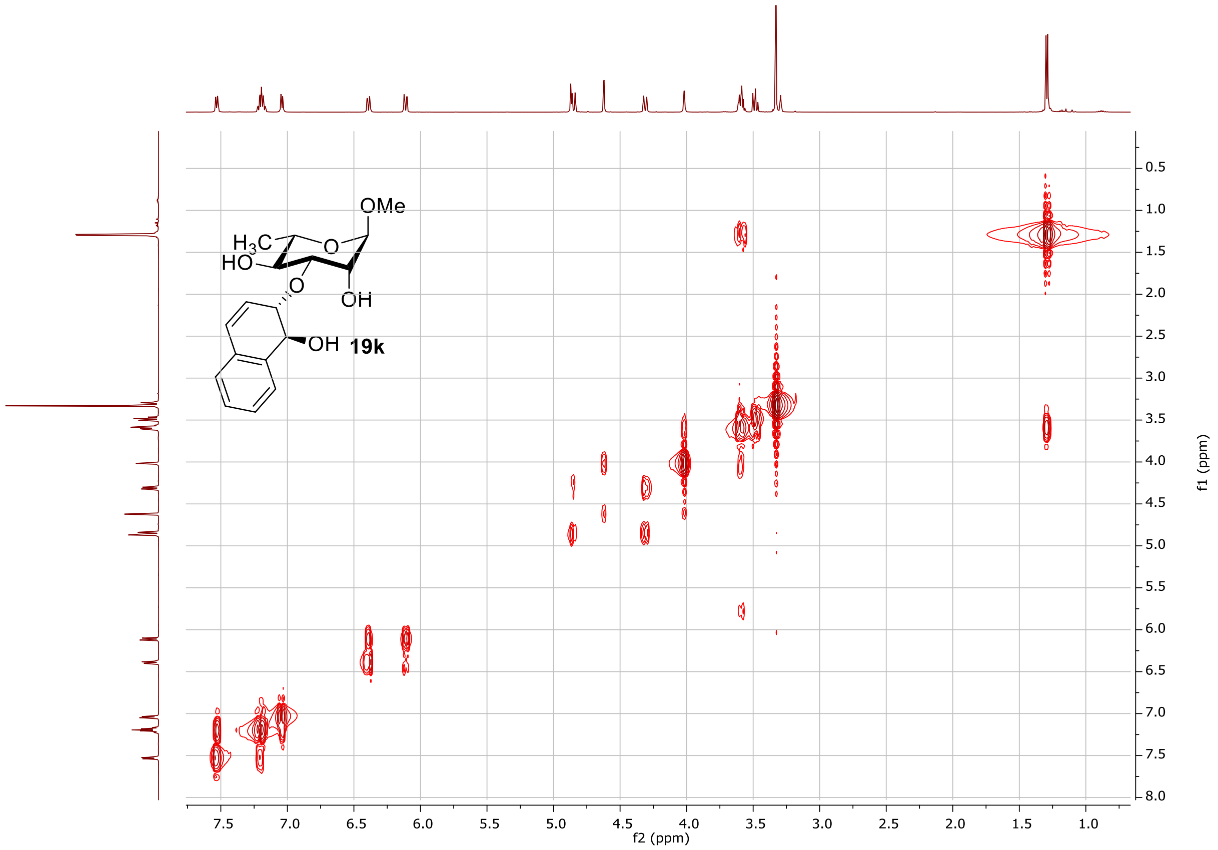

Supplementary Figure 168. COSY spectra for **19k**

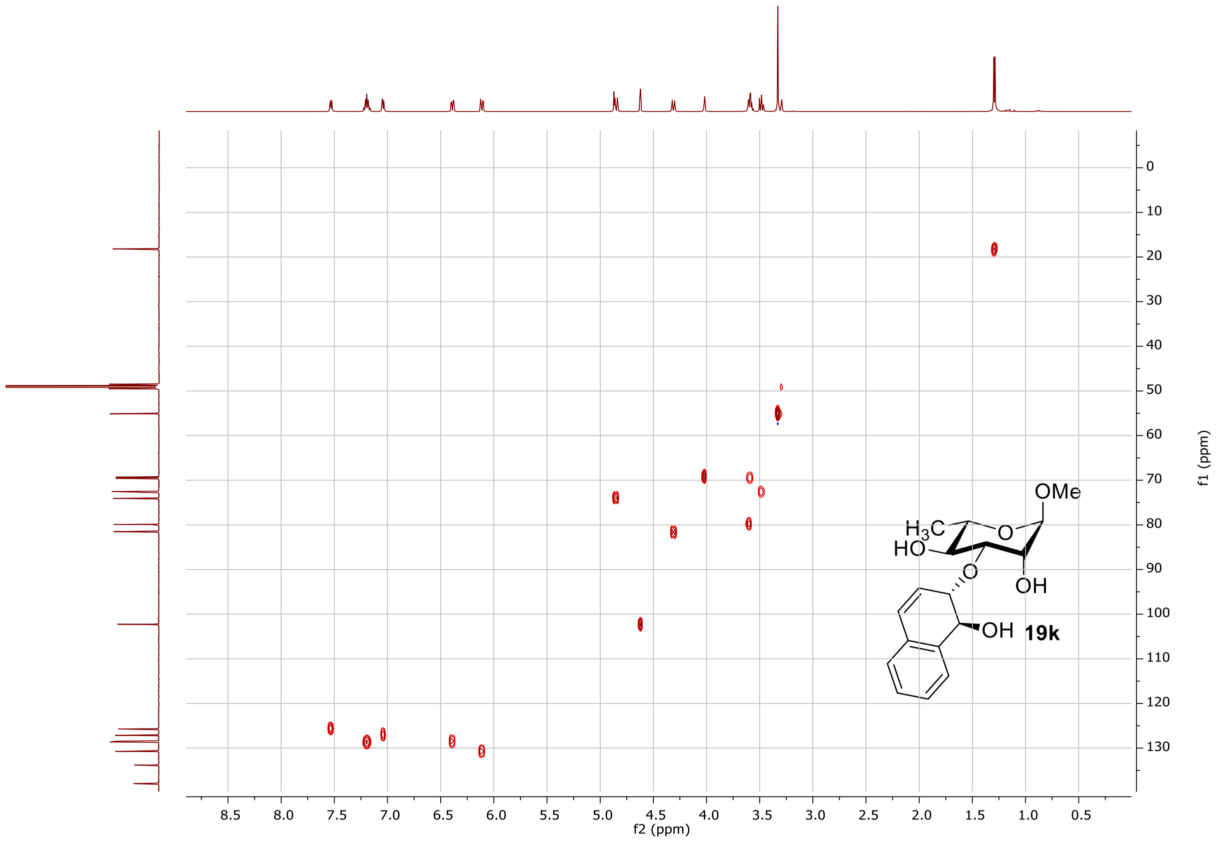

Supplementary Figure 169. HSQC spectra for **19k**

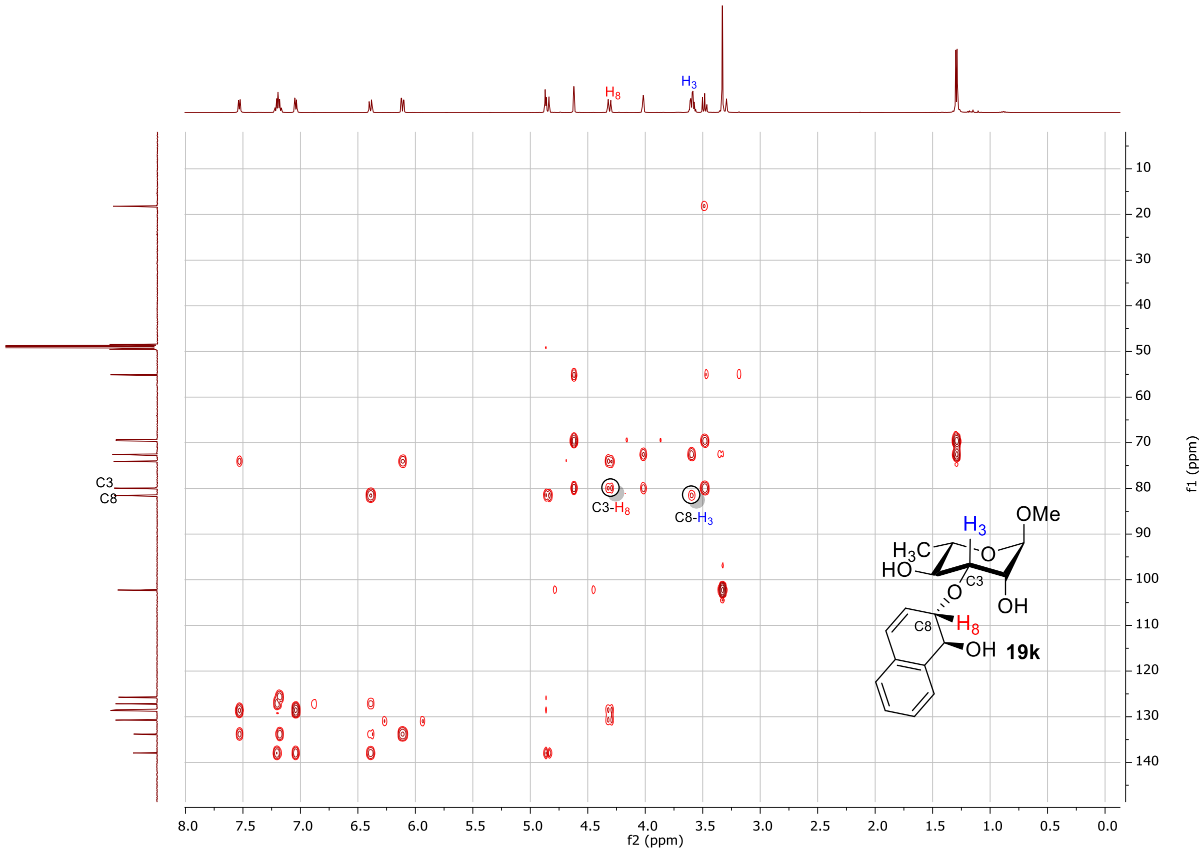

Supplementary Figure 170. HMBC spectra for **19k**

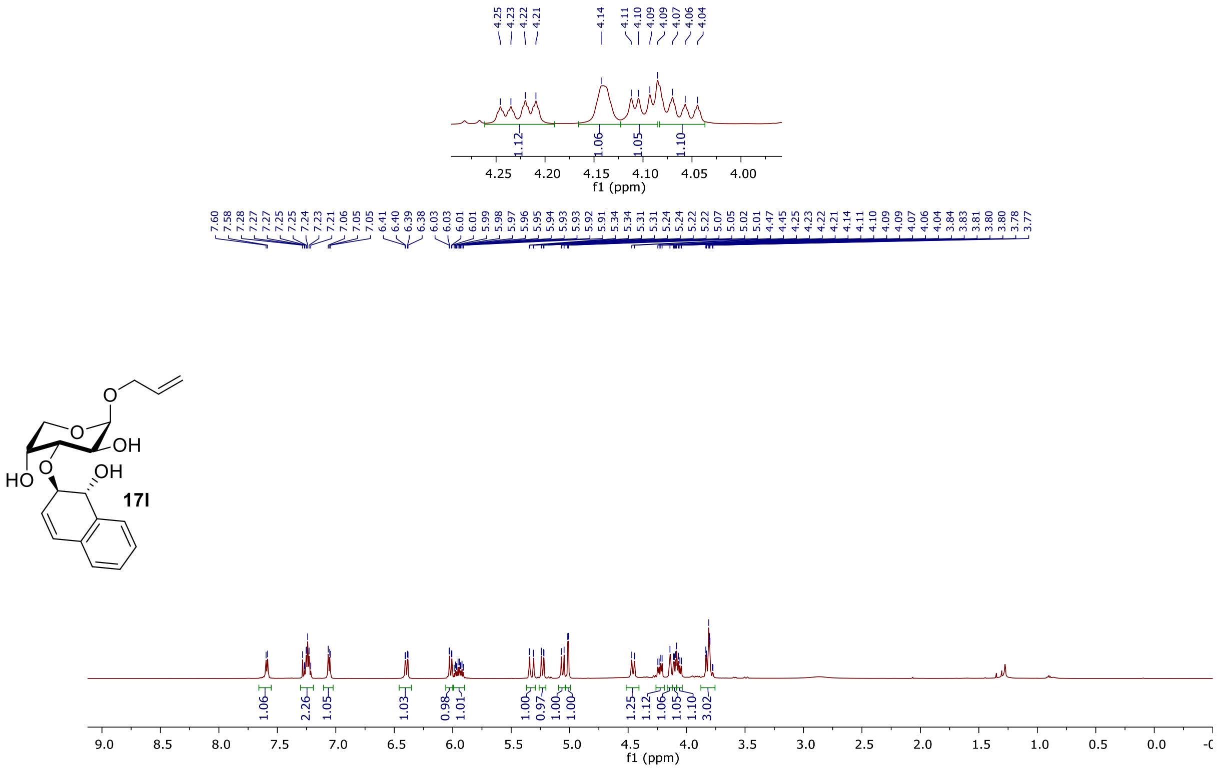

Supplementary Figure 171. ^{1}H spectra for **17l**

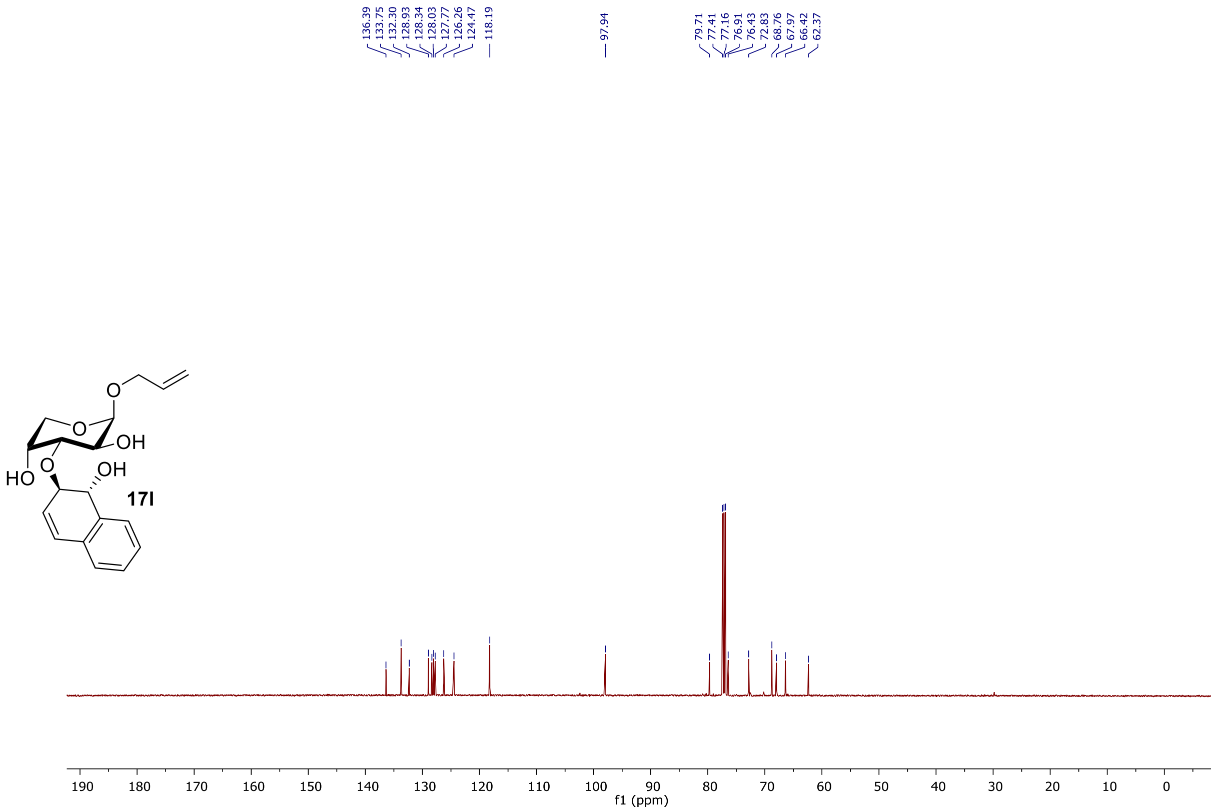

Supplementary Figure S172. ^{13}C spectra for **17l**

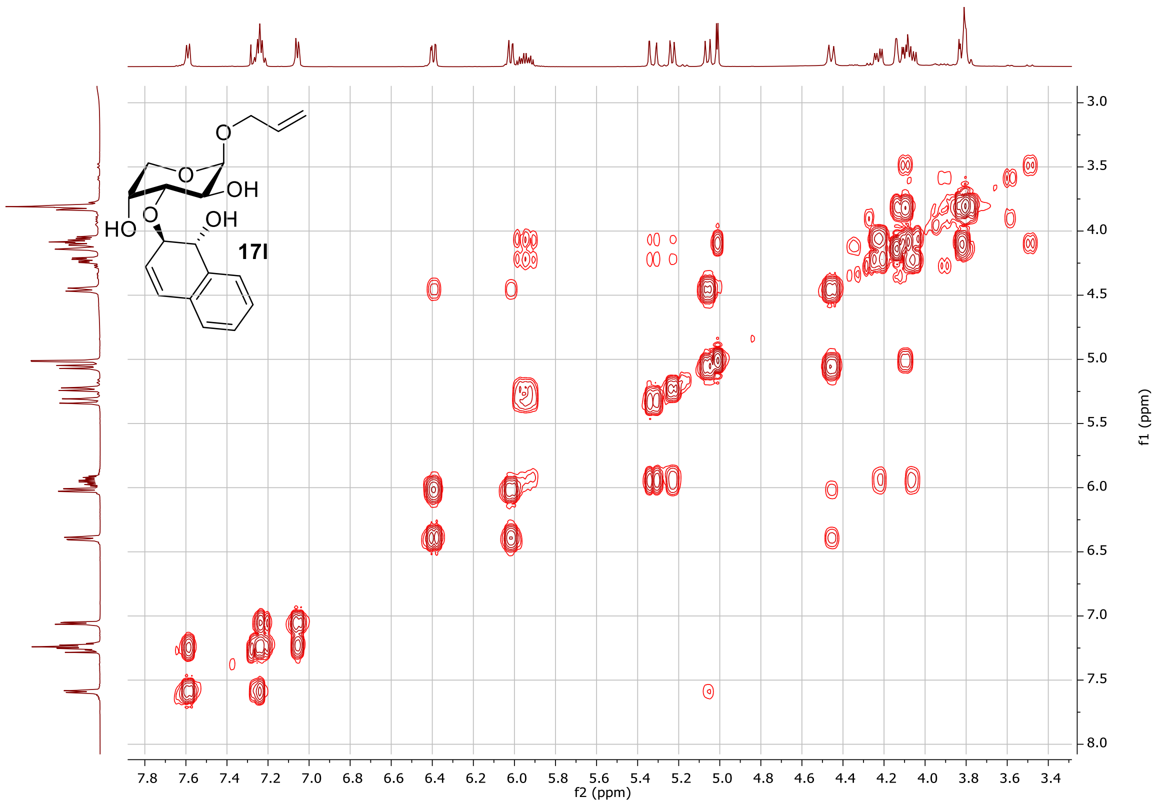

Supplementary Figure 173. COSY spectra for **17l**

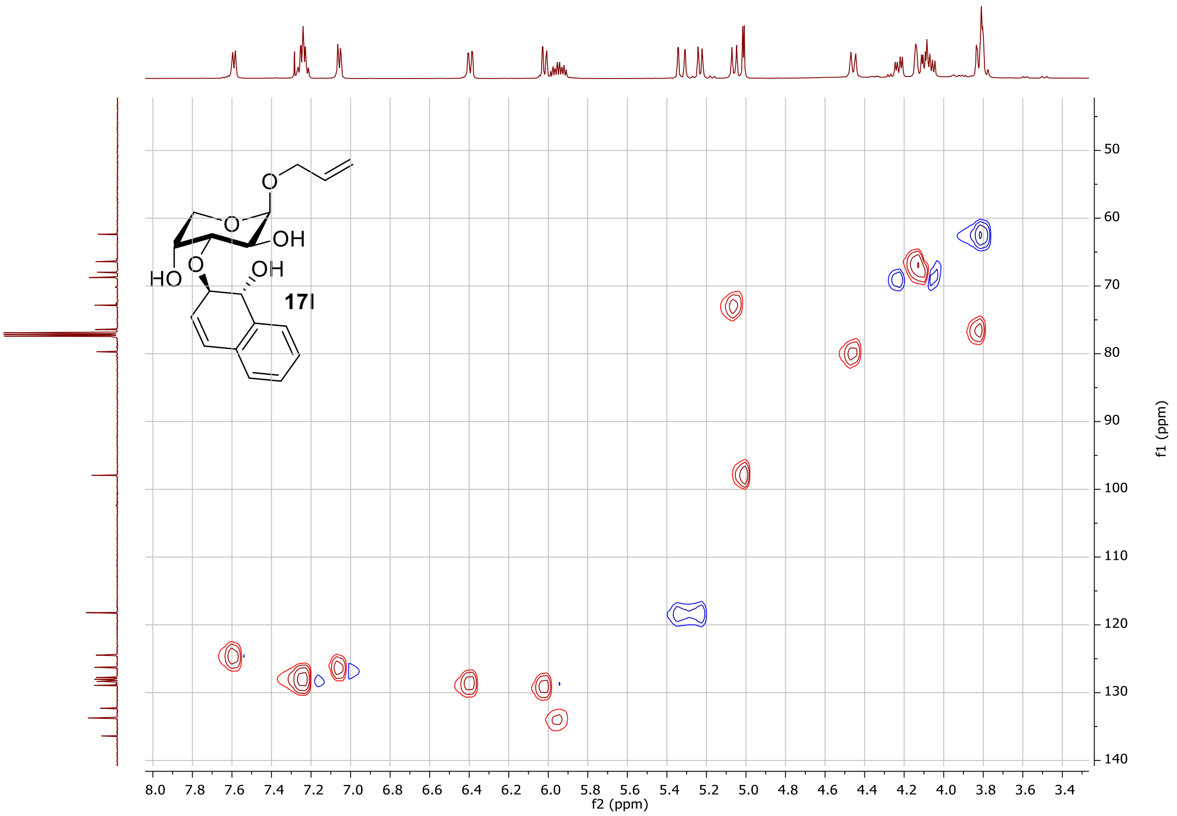

Supplementary Figure 174. HSQC spectra for **17l**

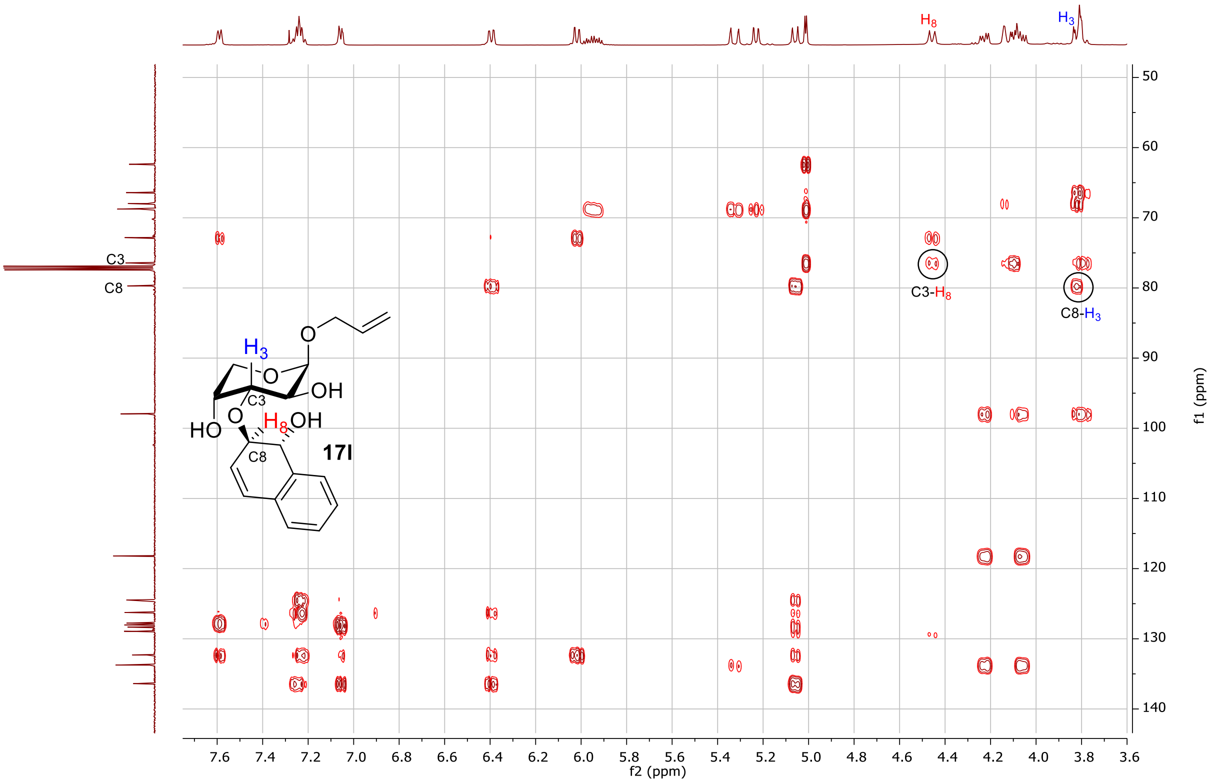

Supplementary Figure 175. HMBC spectra for **17l**

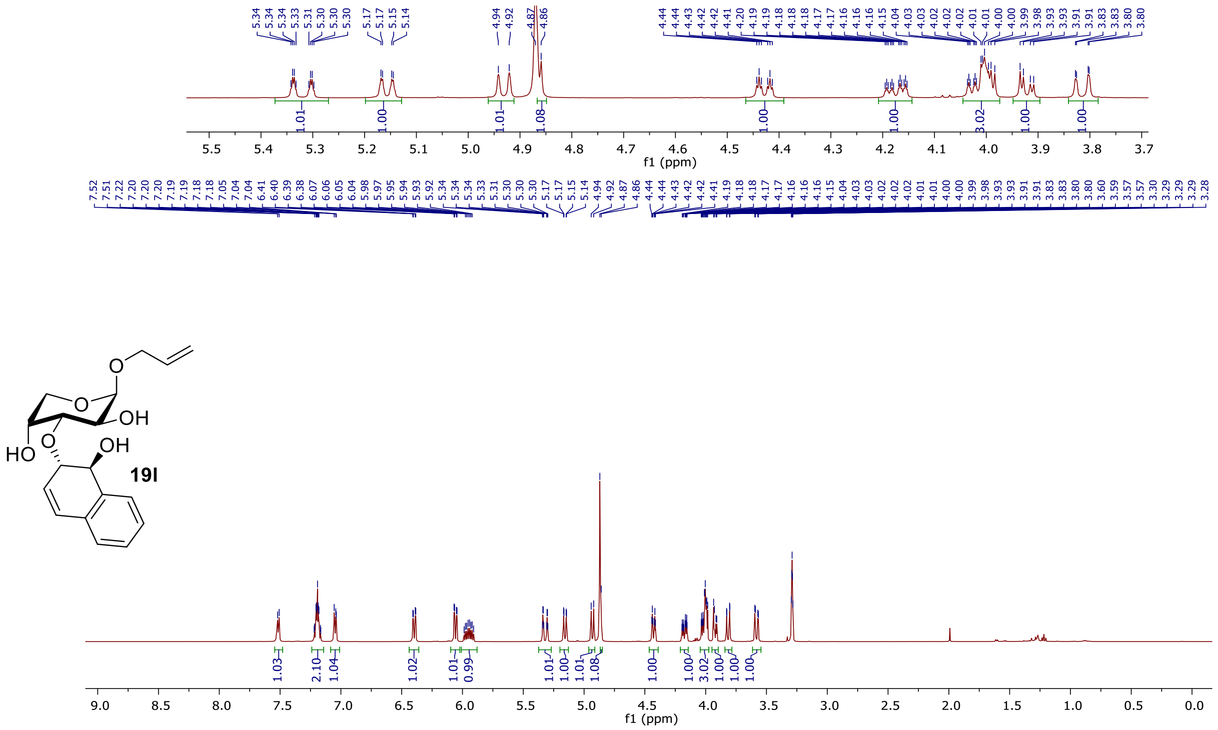

Supplementary Figure 176. ^{1}H spectra for **19l**

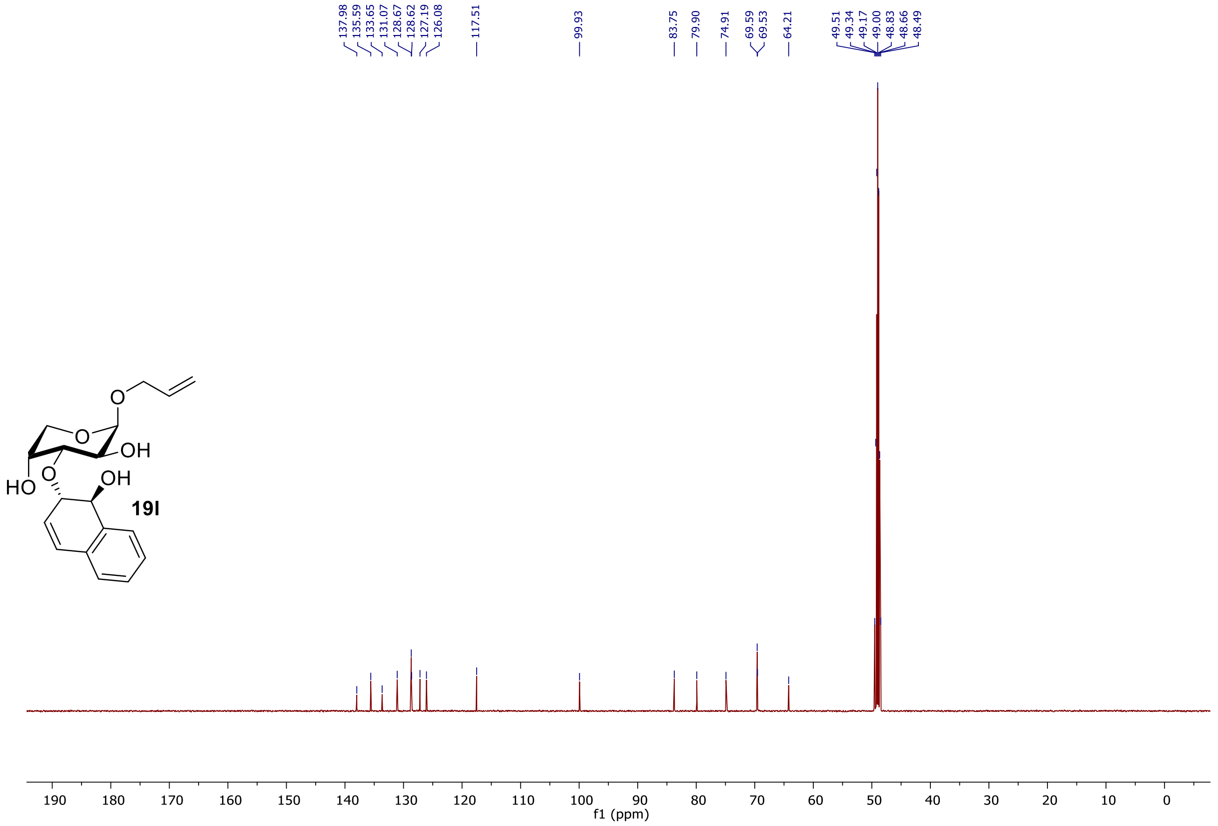

Supplementary Figure 177. ^{13}C spectra for **19l**

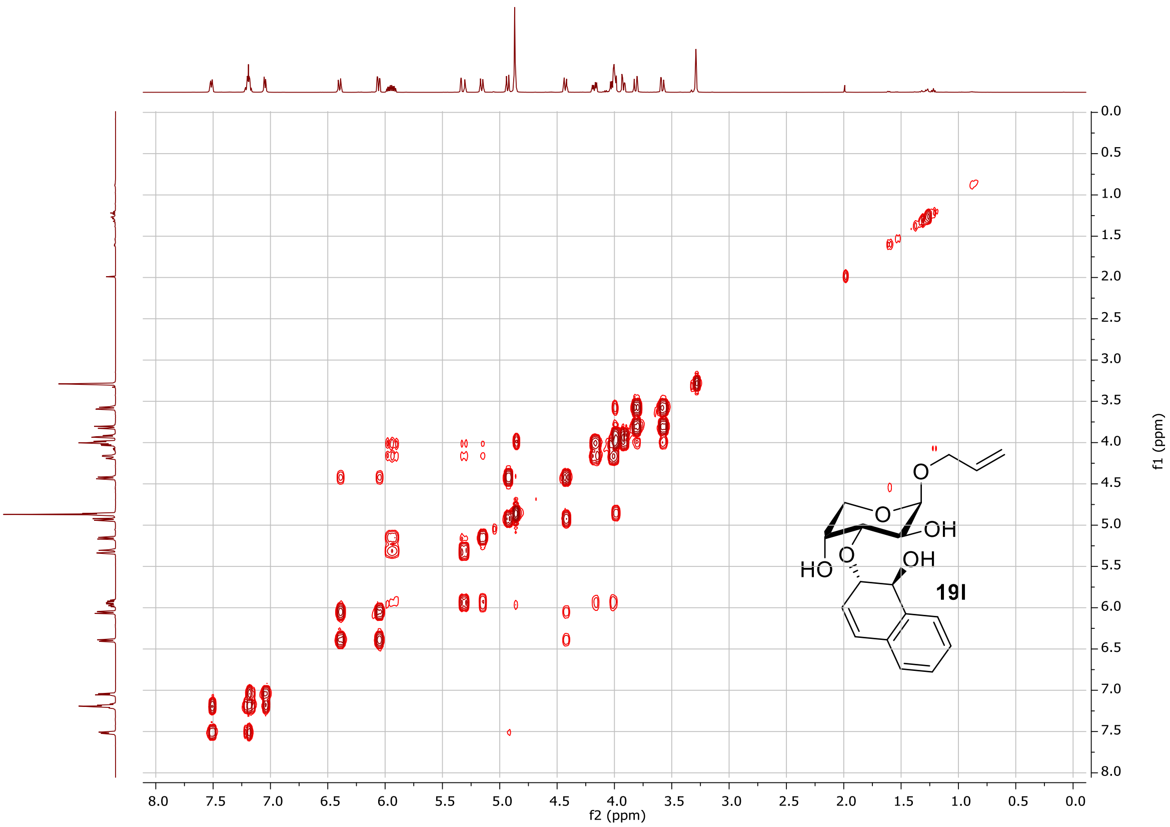

Supplementary Figure 178. COSY spectra for **19l**

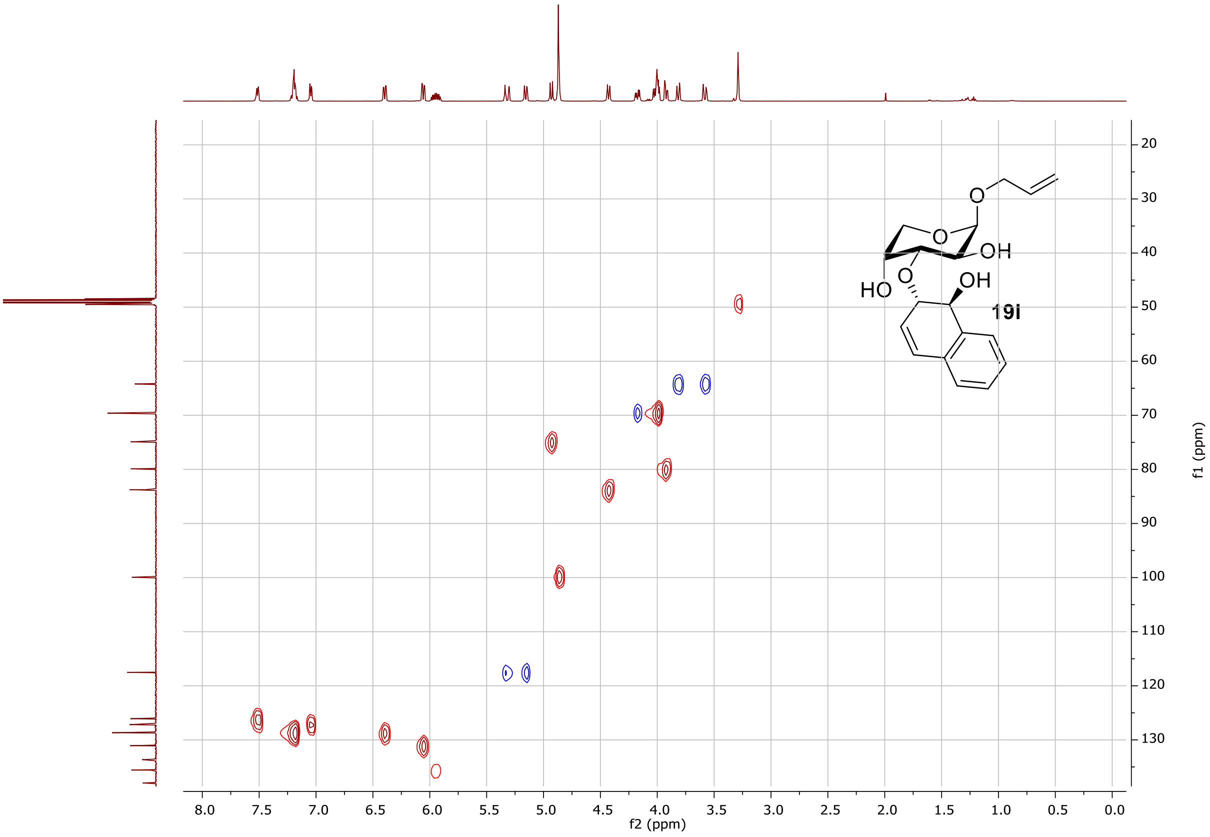

Supplementary Figure 179. HSQC spectra for **19l**

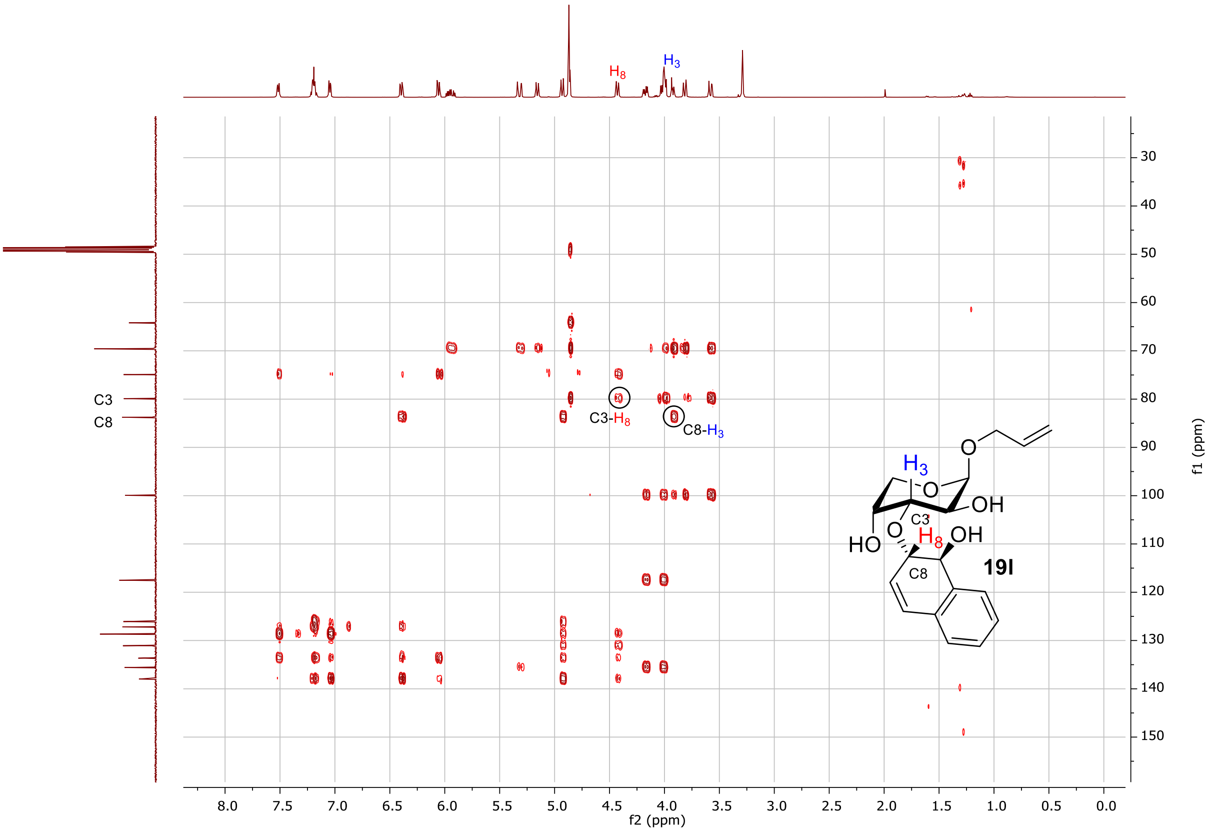

Supplementary Figure 180. HMBC spectra for **19l**

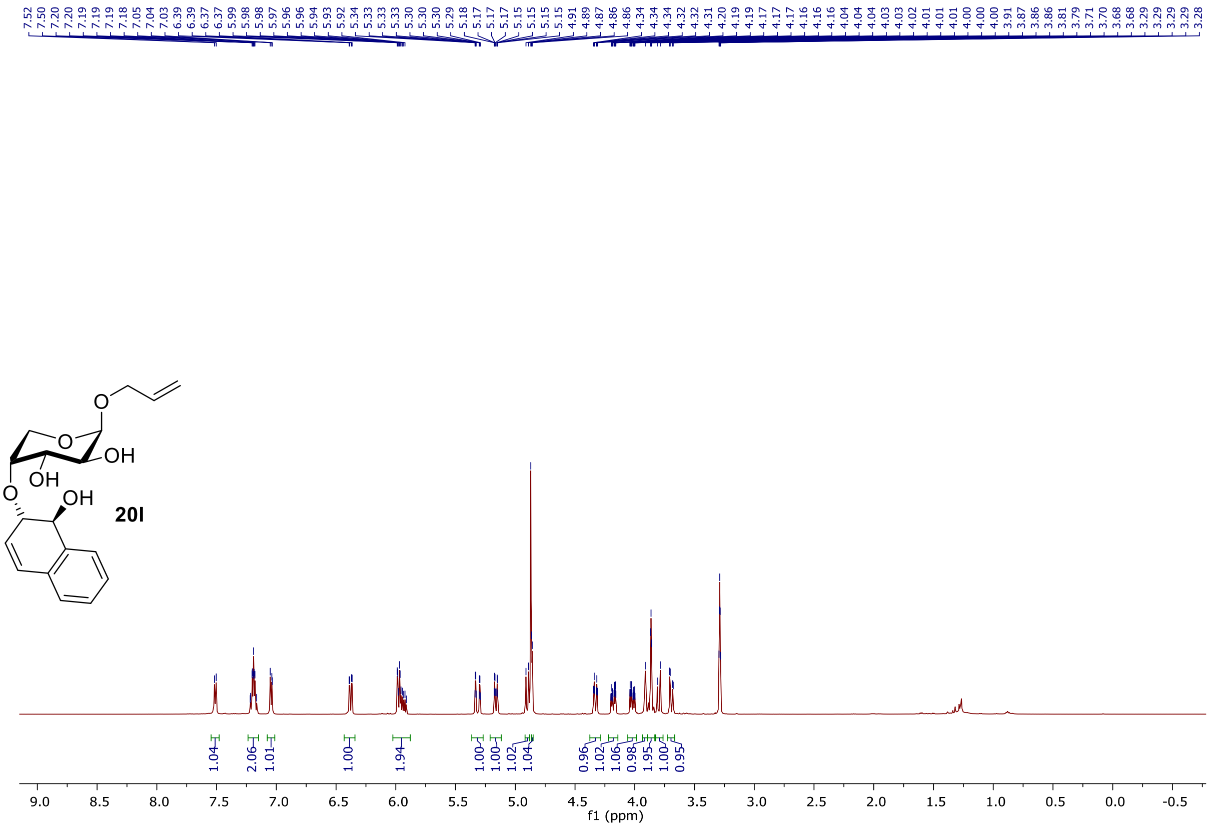

Supplementary Figure 181. ^{1}H spectra for **20l**

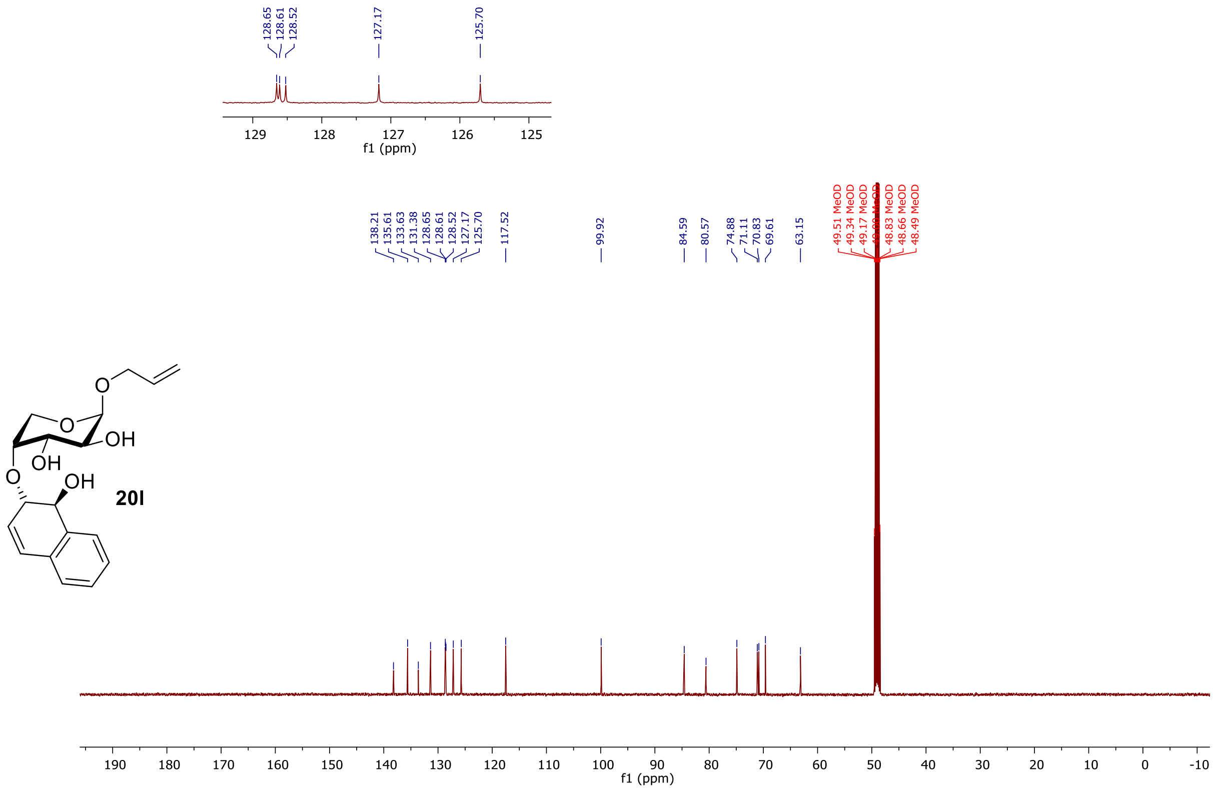

Supplementary Figure 182. ^{13}C spectra for **20l**

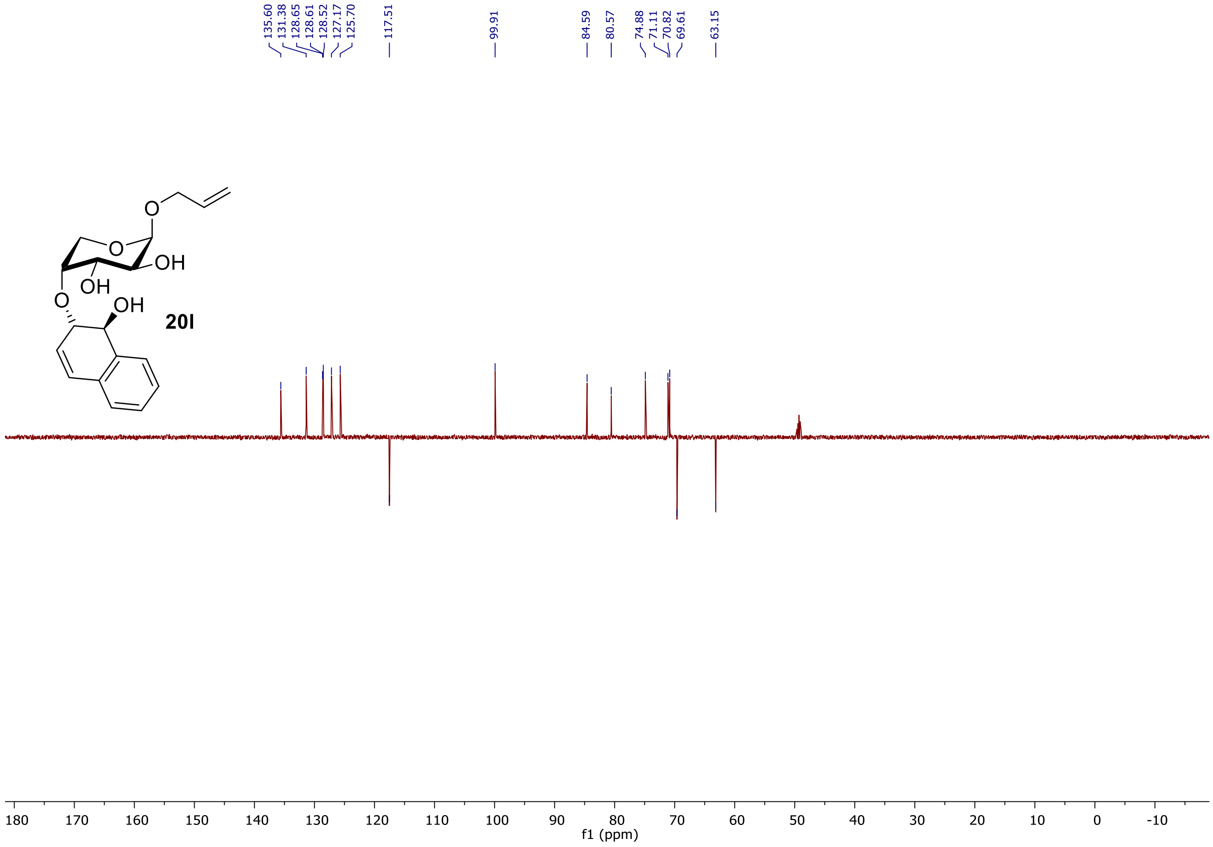

Supplementary Figure 183. DEPT spectra for **20l**

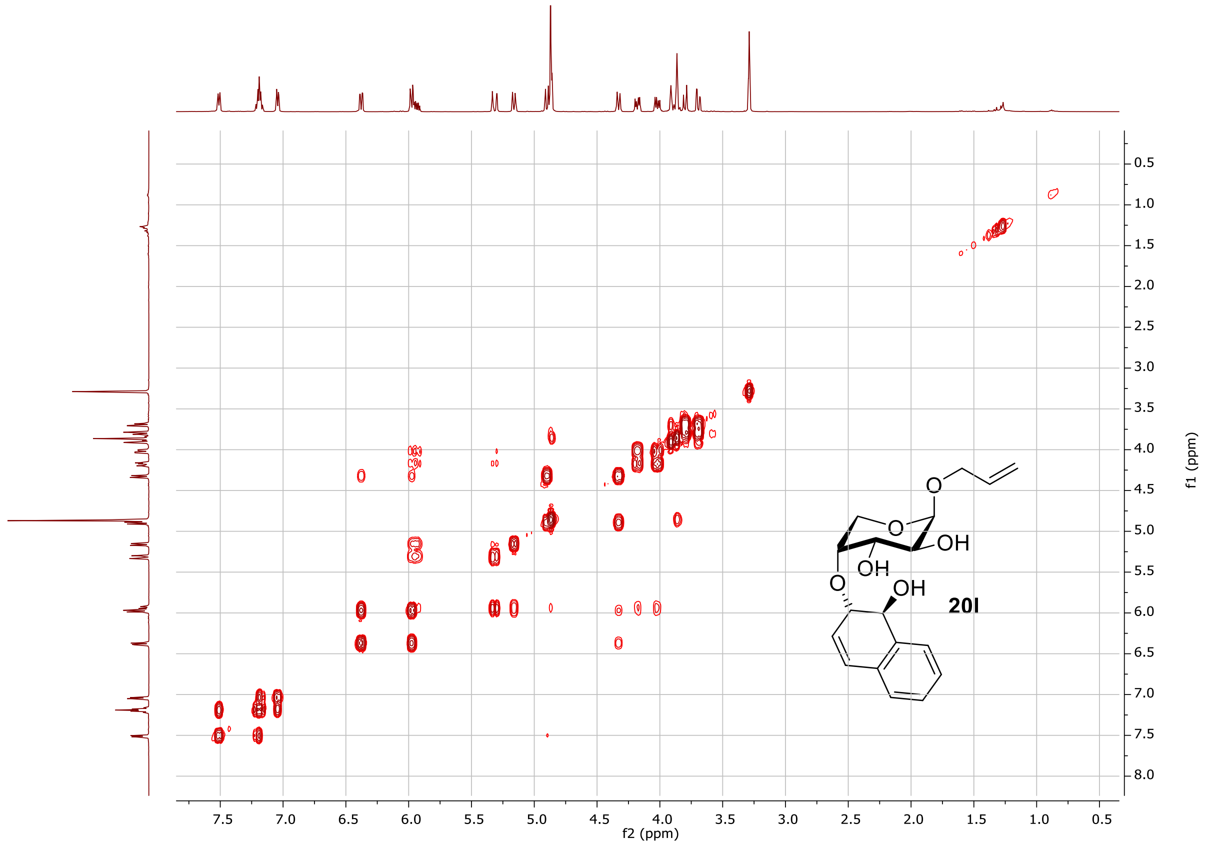

Supplementary Figure 184. COSY spectra for **20l**

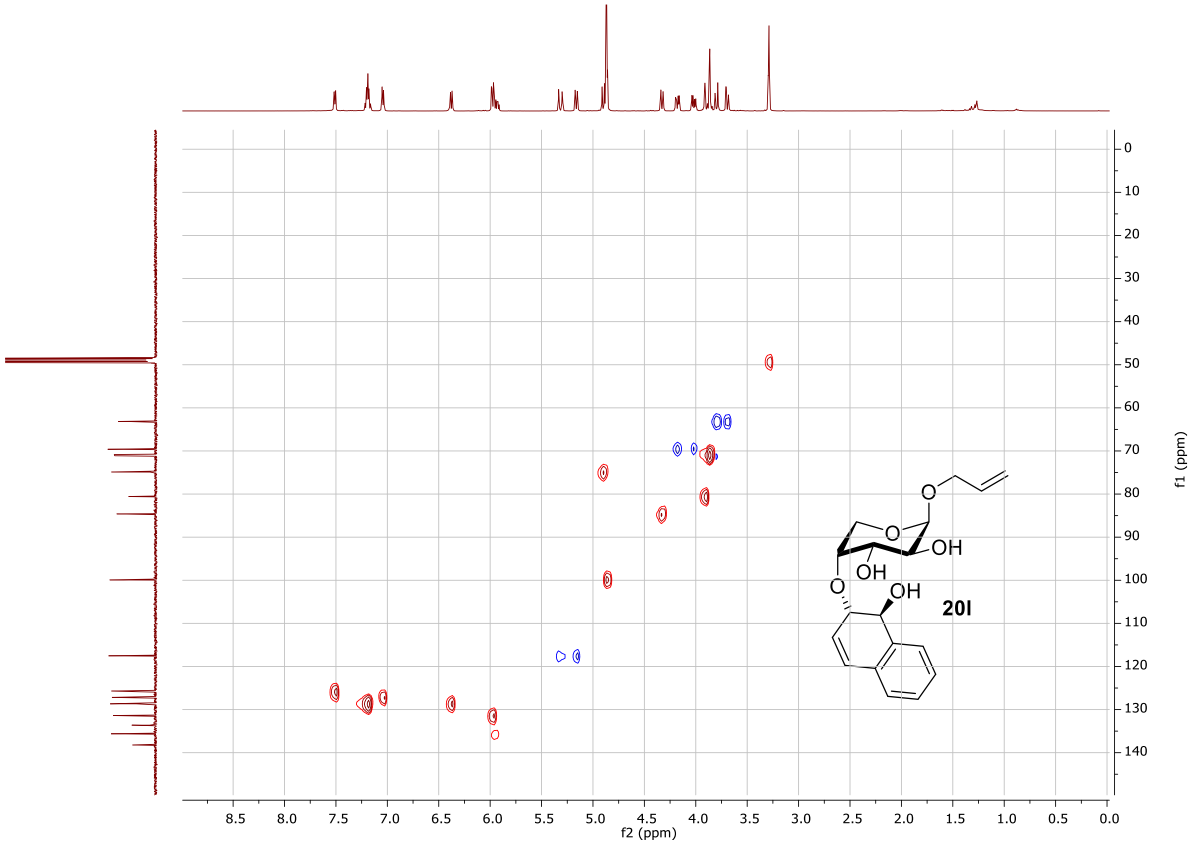

Supplementary Figure 185. HSQC spectra for **20l**

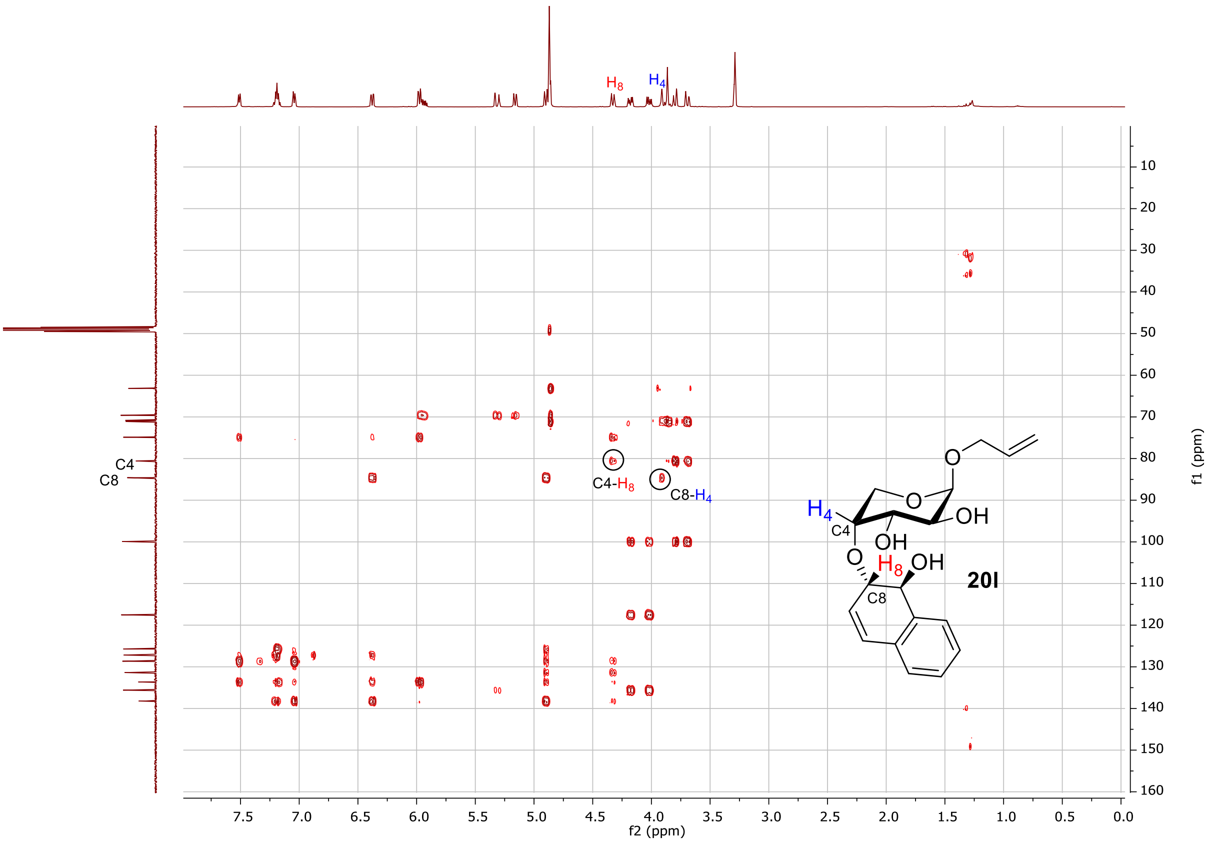

Supplementary Figure 186. HMBC spectra for **20l**

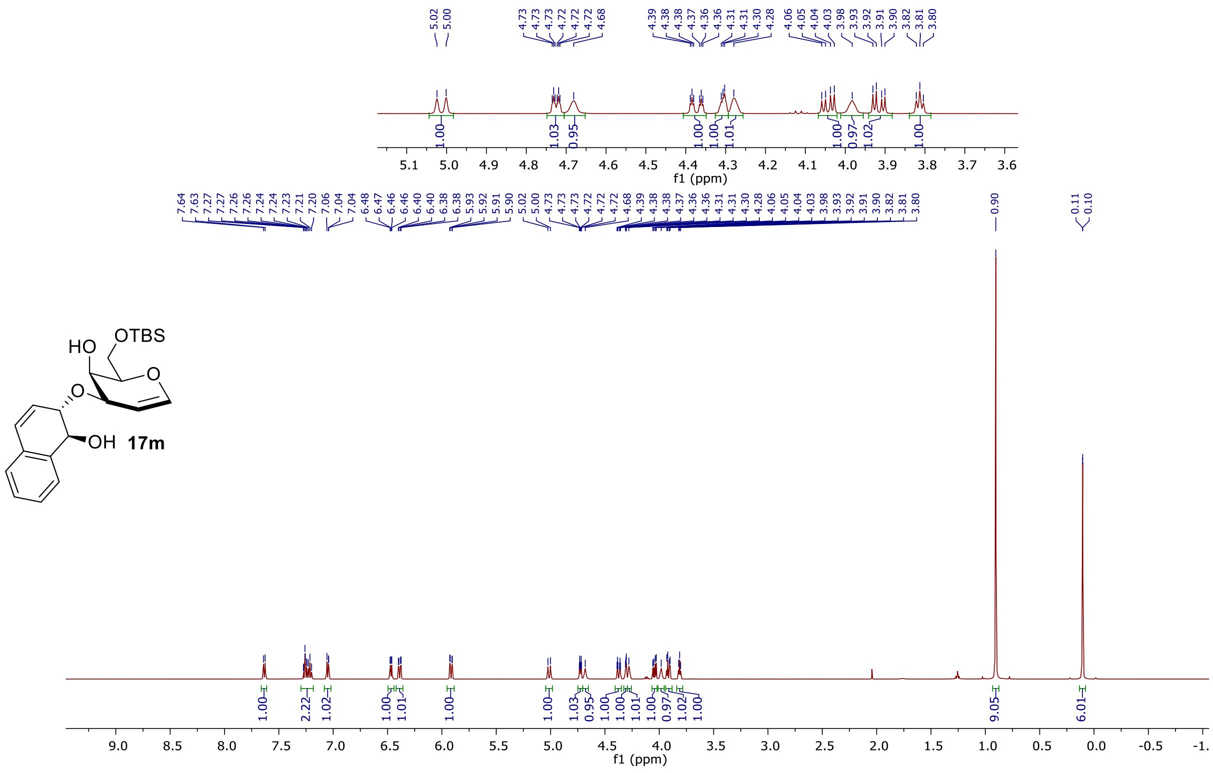

Supplementary Figure 187. ^{1}H spectra for **17m**

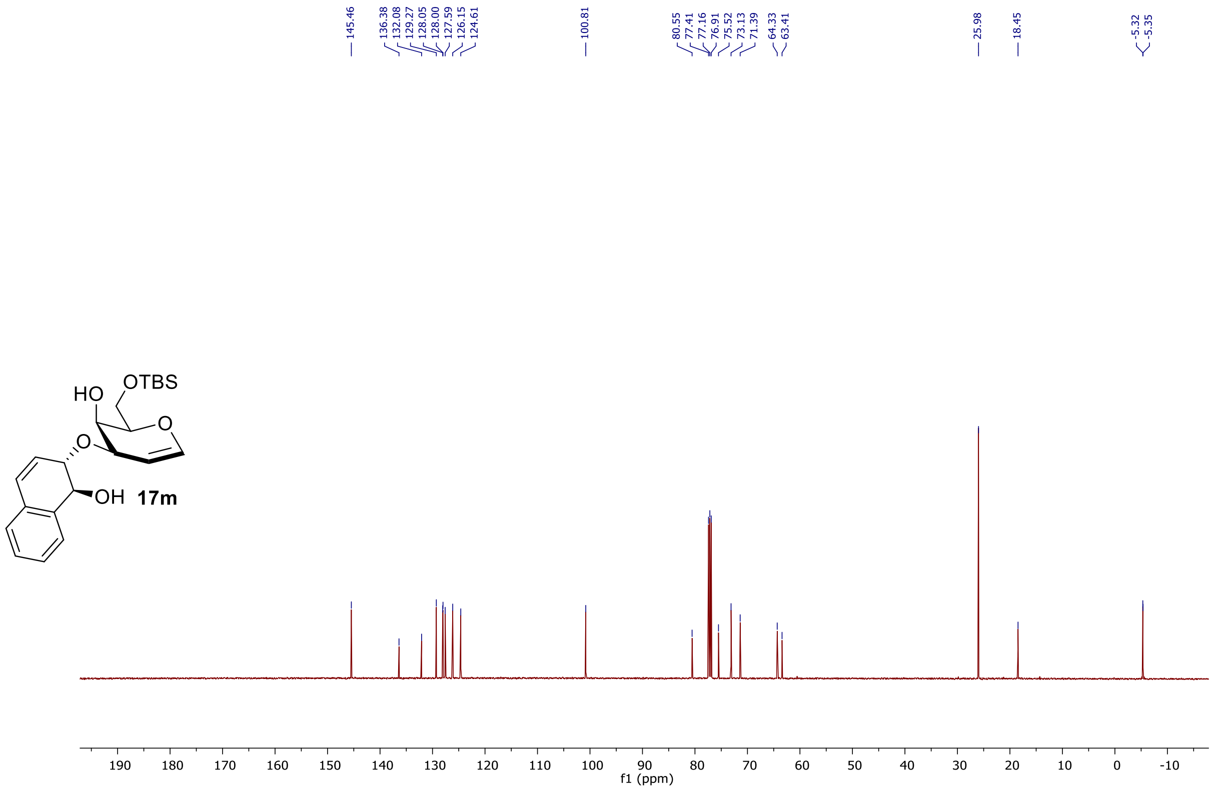

Supplementary Figure 188. ^{13}C spectra for **17m**

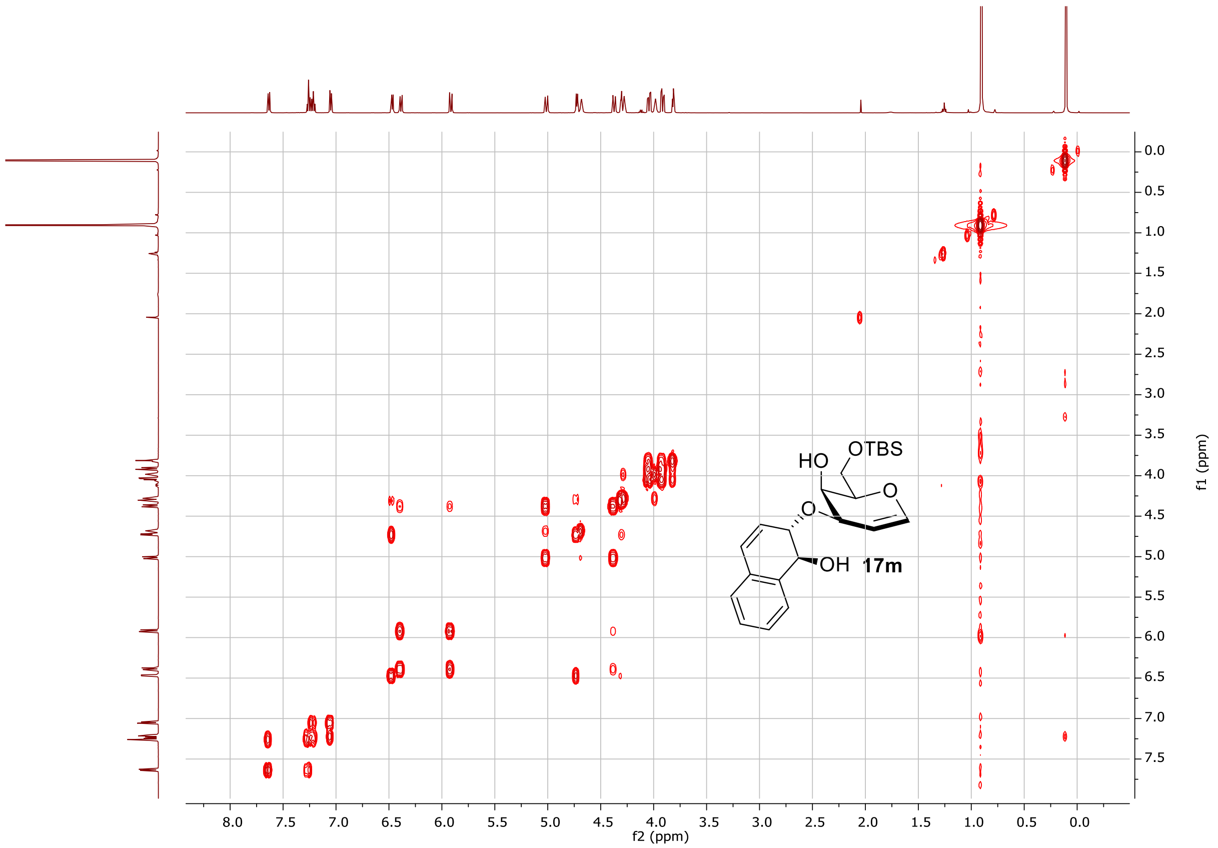

Supplementary Figure 189. COSY spectra for **17m**

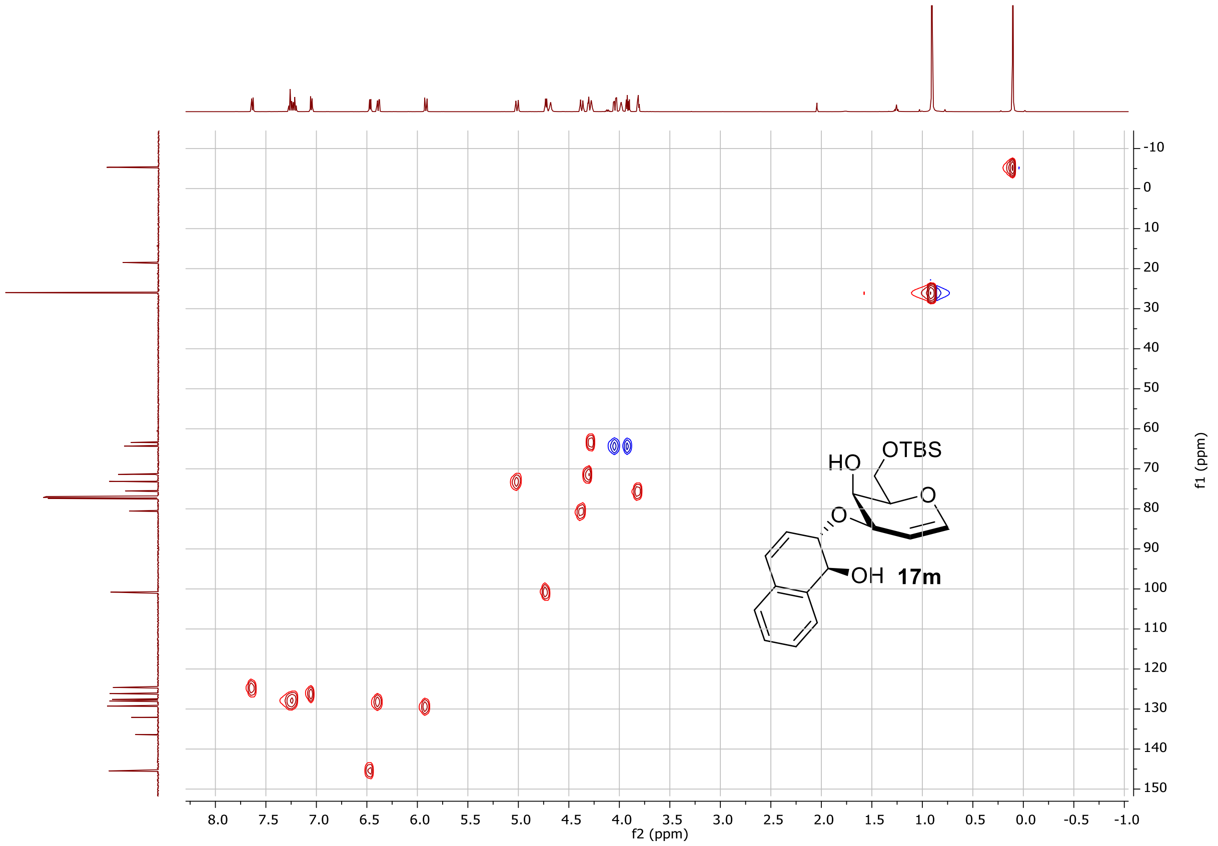

Supplementary Figure S190. HSQC spectra for **17m**

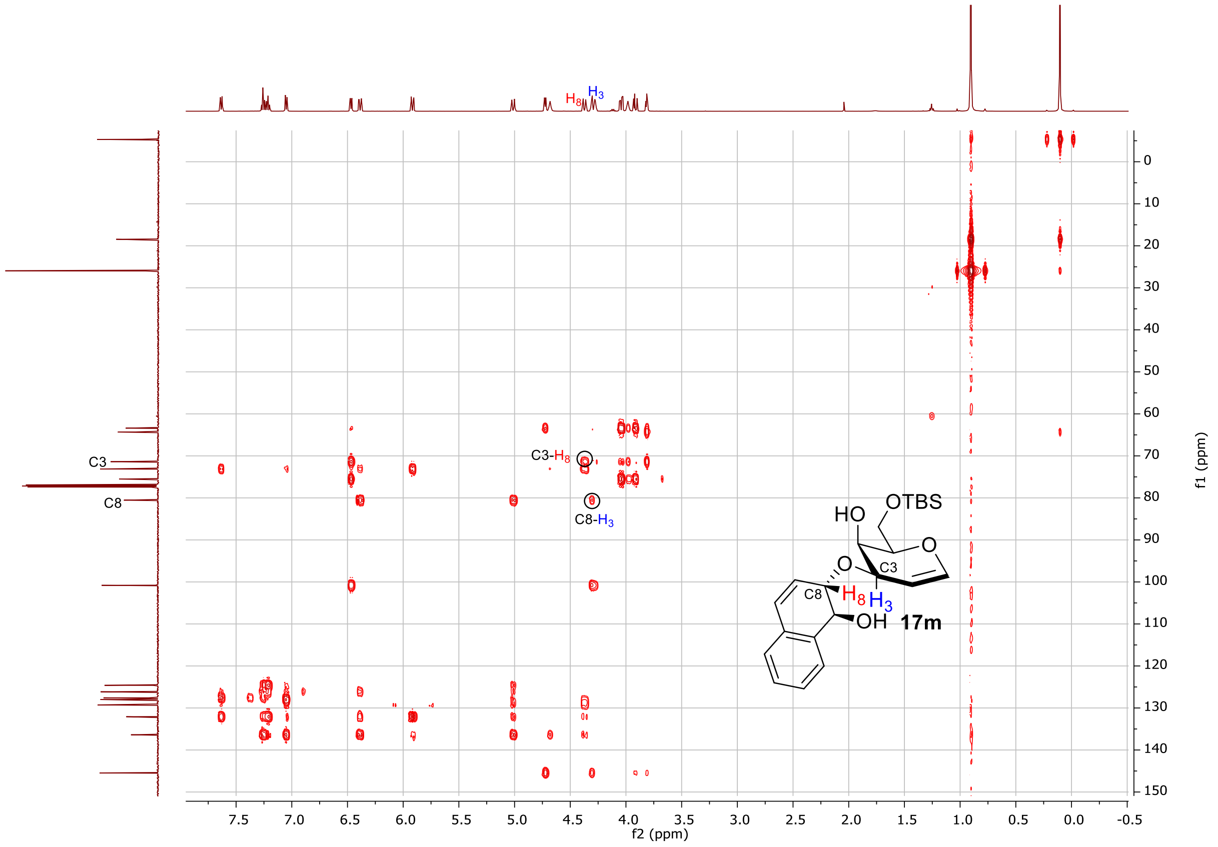

Supplementary Figure S191. HMBC spectra for **17m**

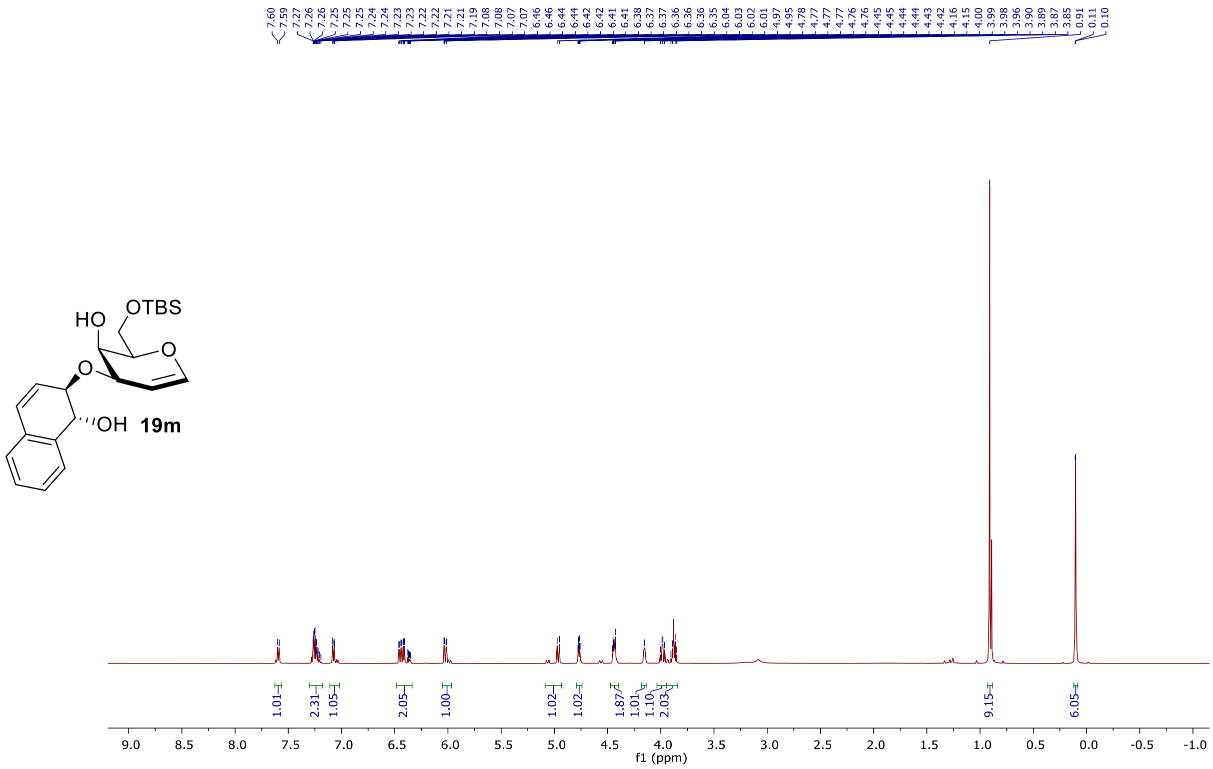

Supplementary Figure 192. ^{1}H spectra for **19m**

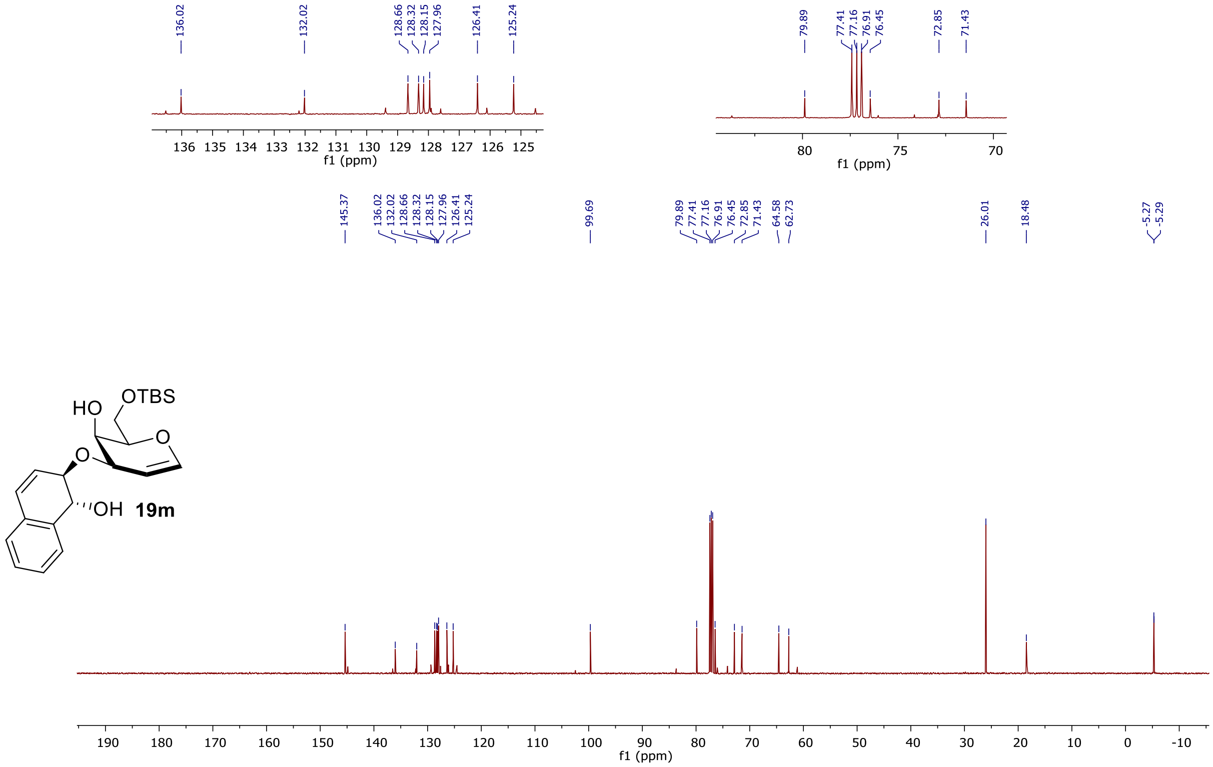

Supplementary Figure 193. ^{13}C spectra for **19m**

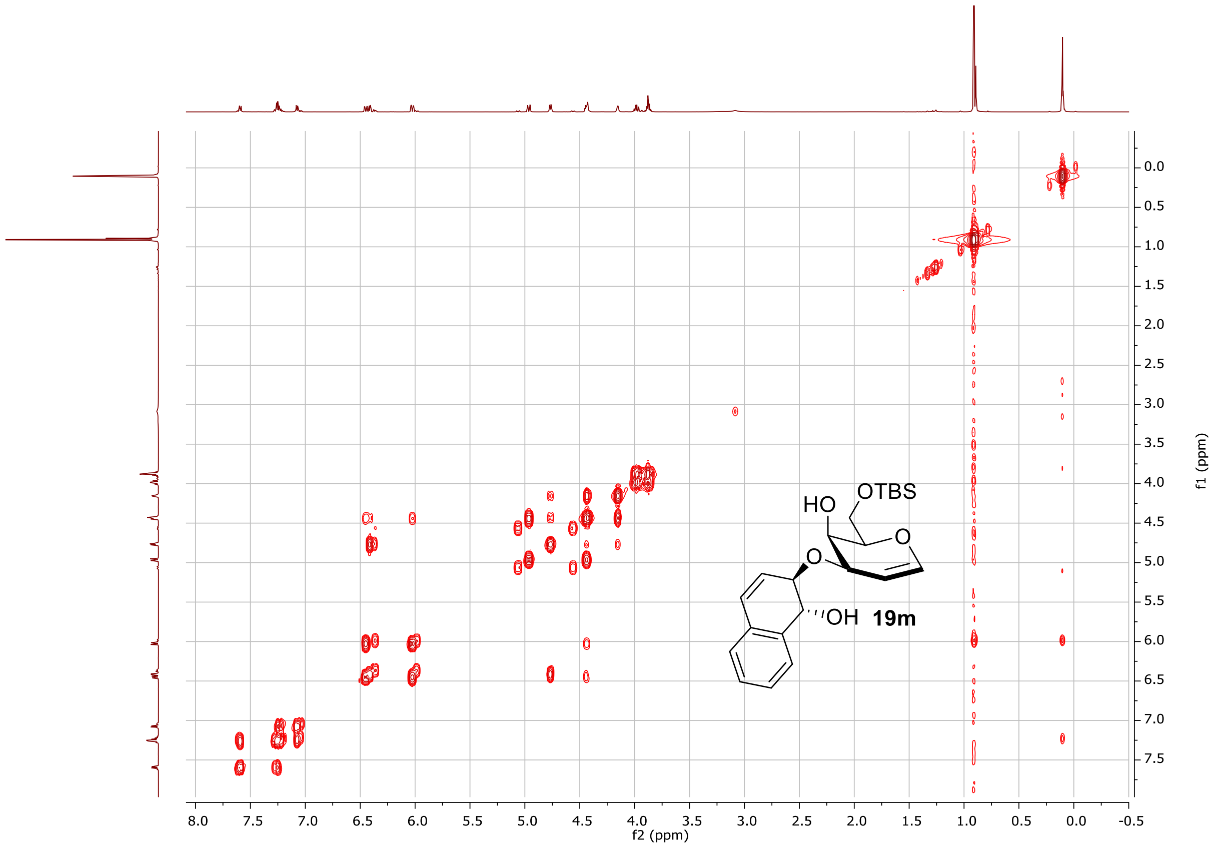

Supplementary Figure 194. COSY spectra for **19m**

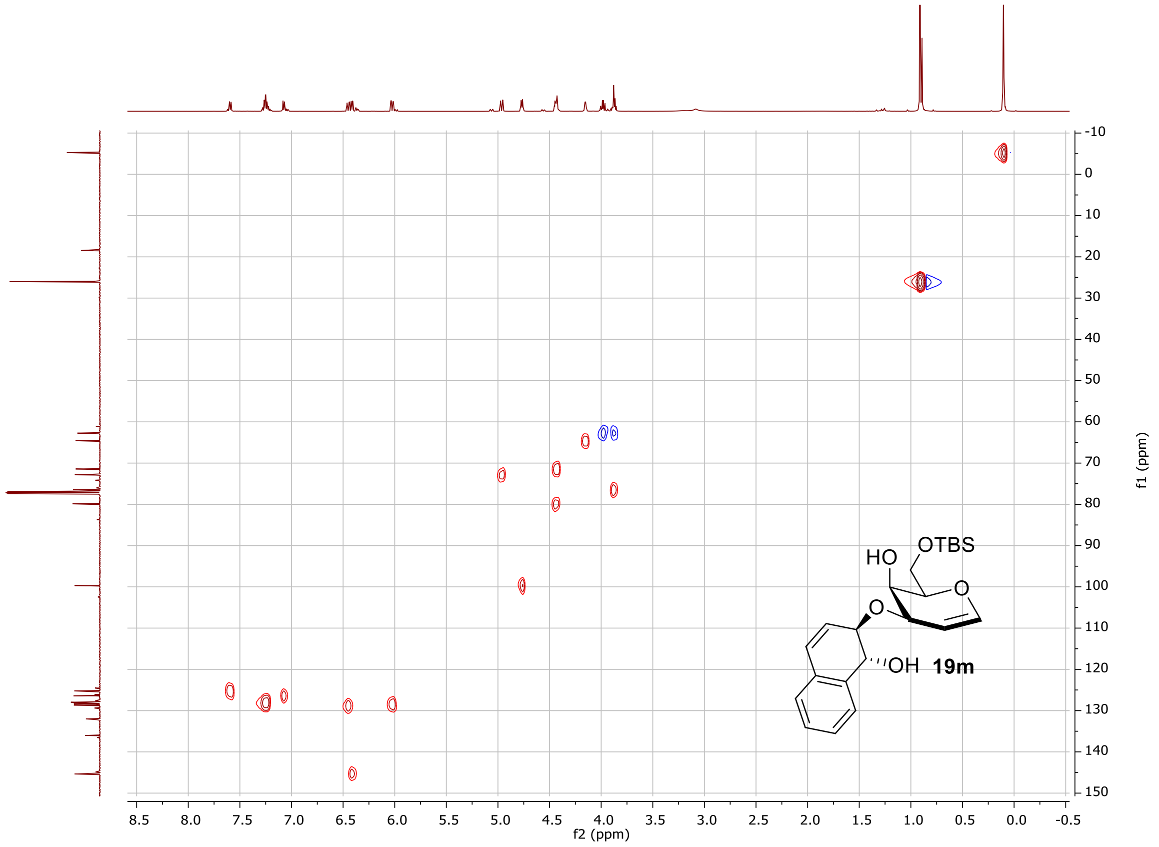

Supplementary Figure 195. HSQC spectra for **19m**

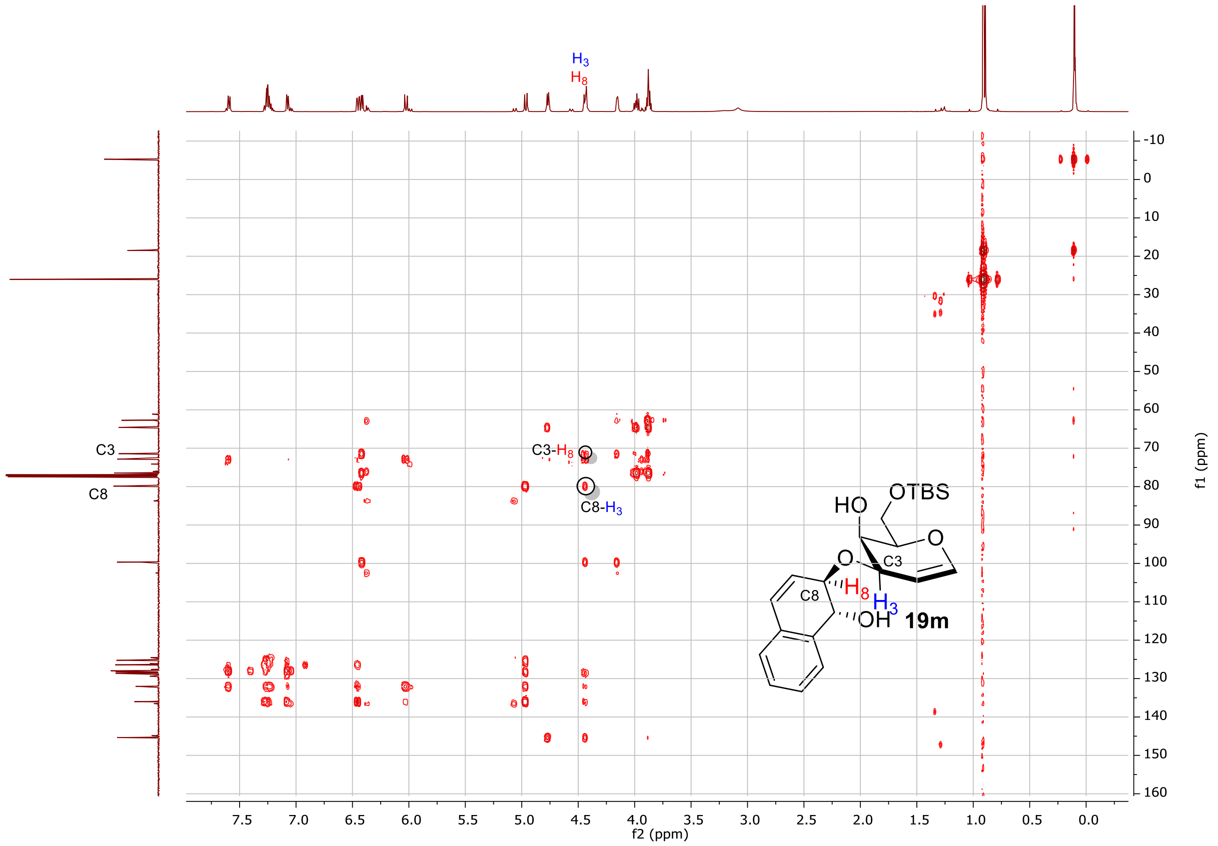

Supplementary Figure 196. HMBC spectra for **19m**

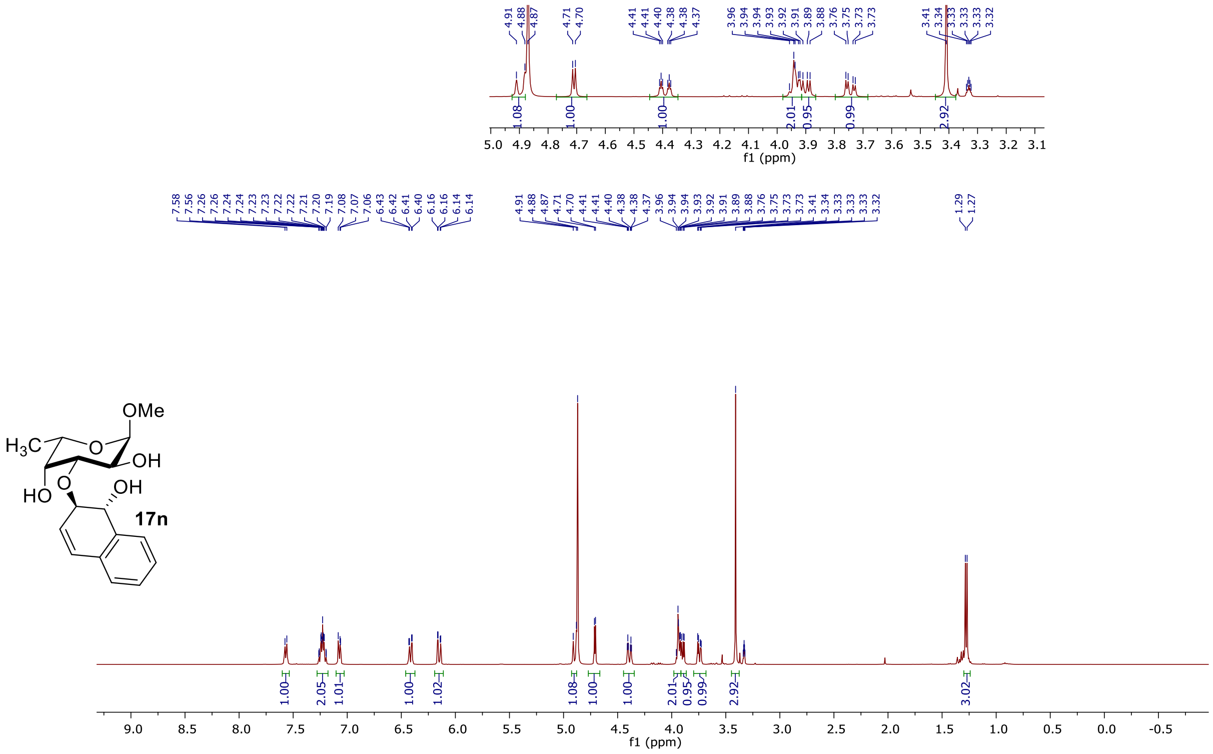

Supplementary Figure 197. ^{1}H spectra for **17n**

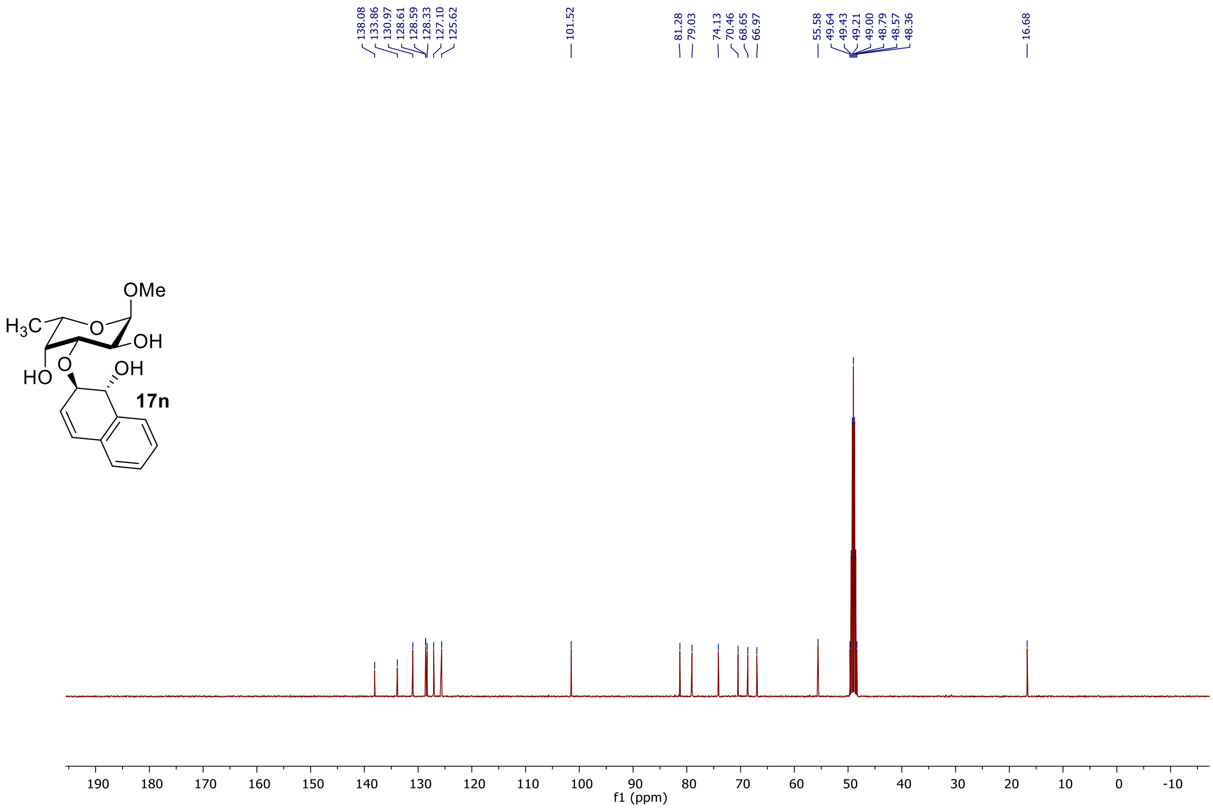

Supplementary Figure 198. ^{13}C spectra for **17n**

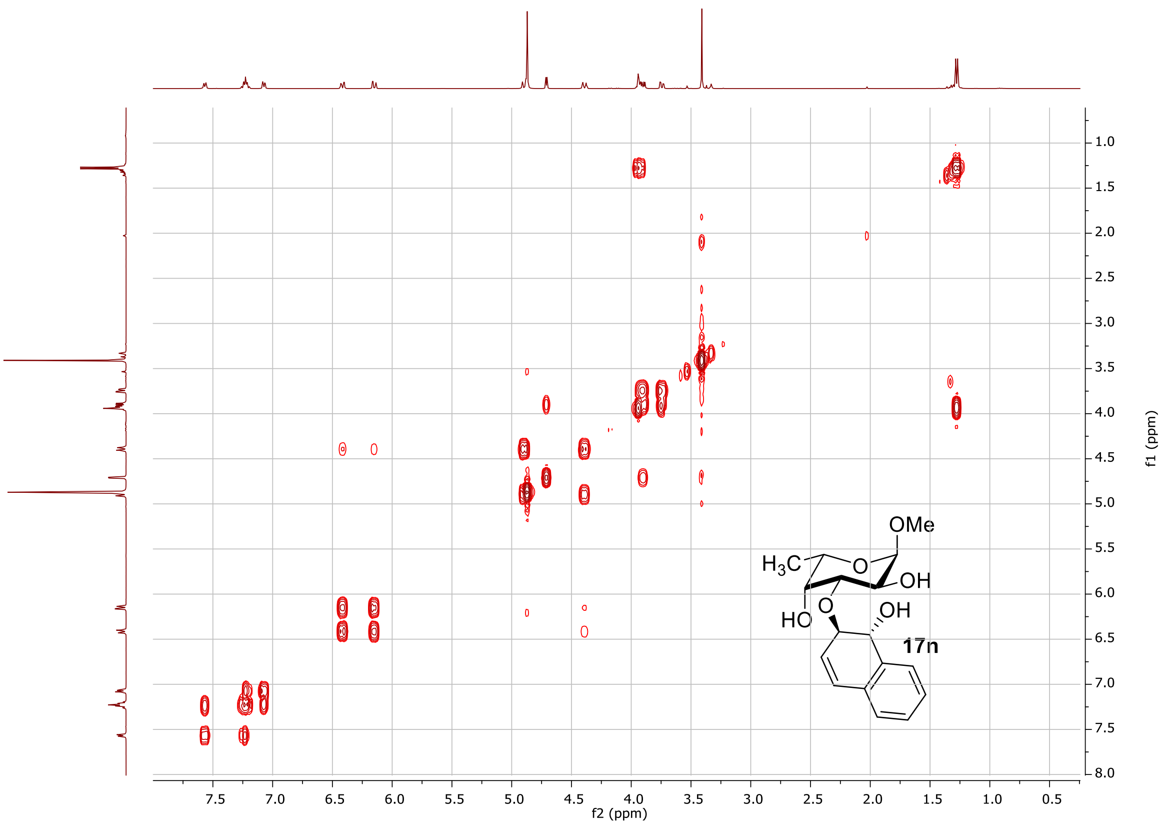

Supplementary Figure 199. COSY spectra for **17n**

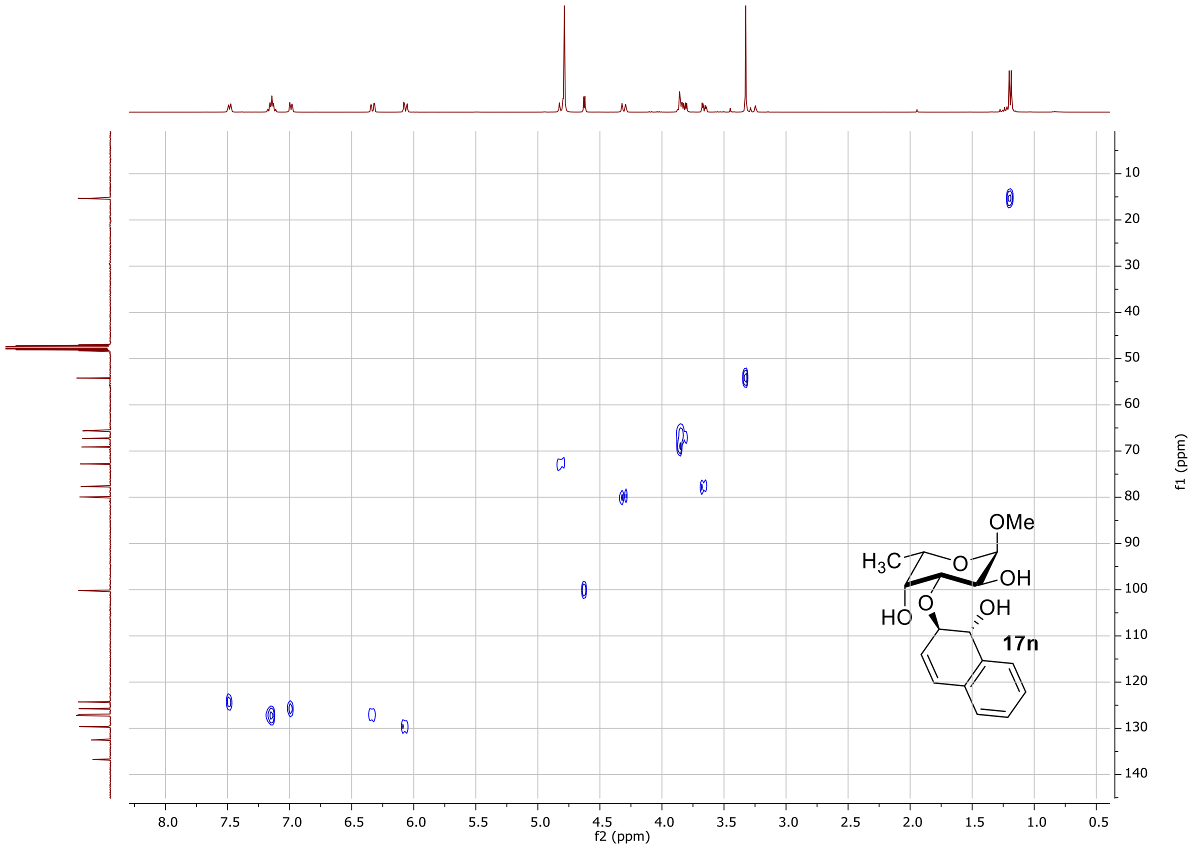

Supplementary Figure 200. HSQC spectra for **17n**

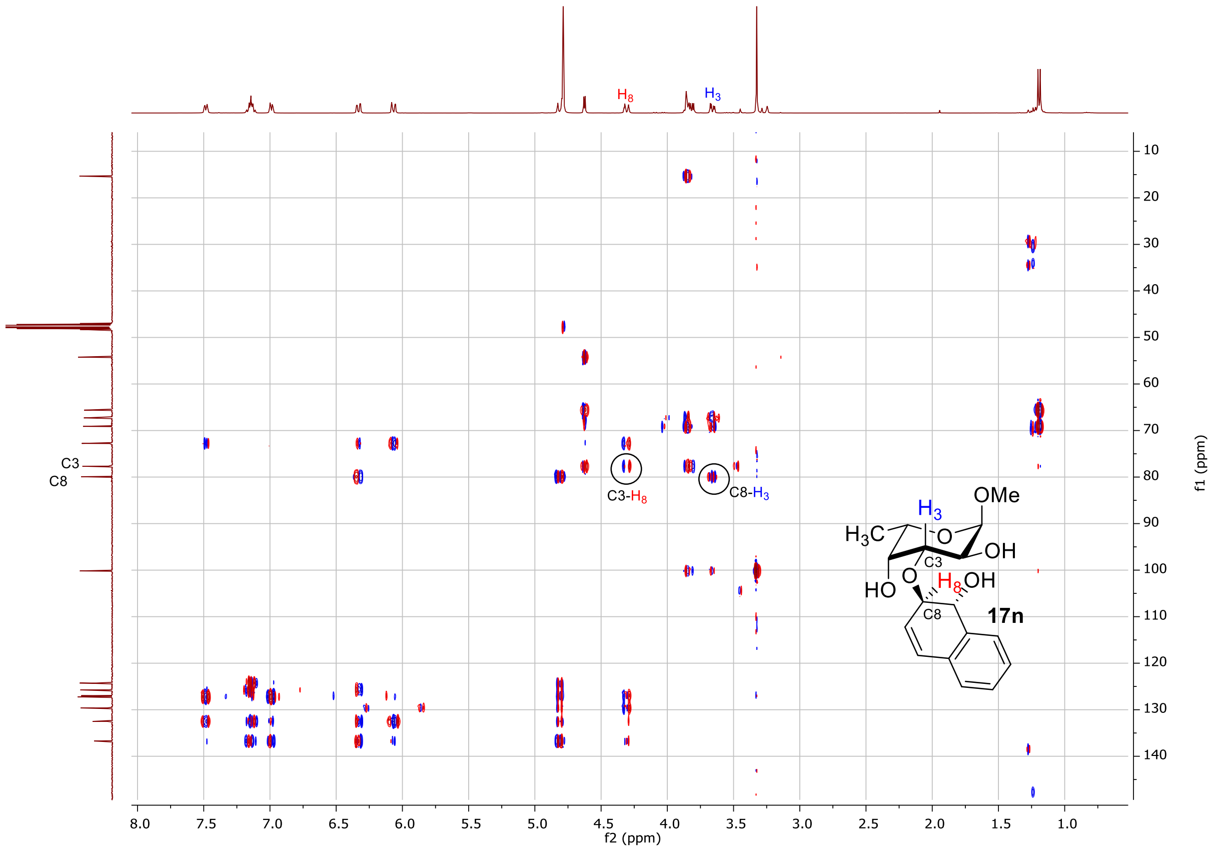

Supplementary Figure 201. HMBC spectra for **17n**

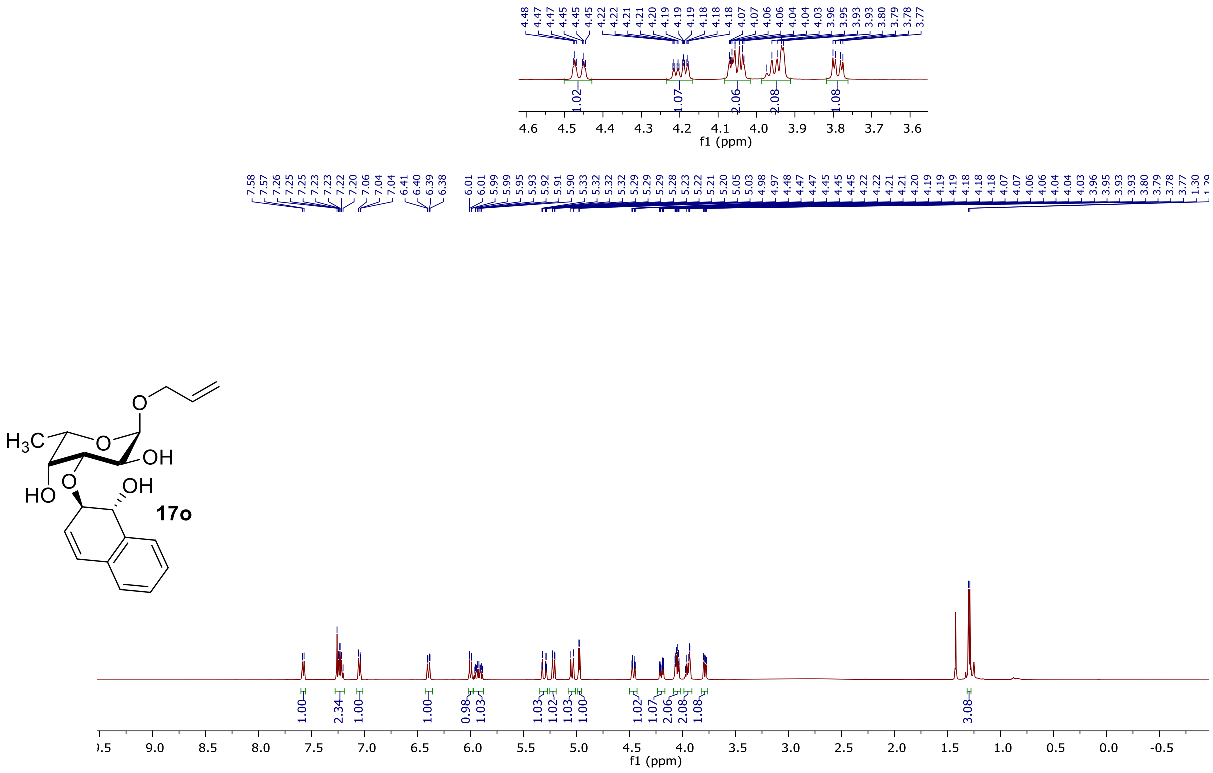

Supplementary Figure 202. ^{1}H spectra for **17o**

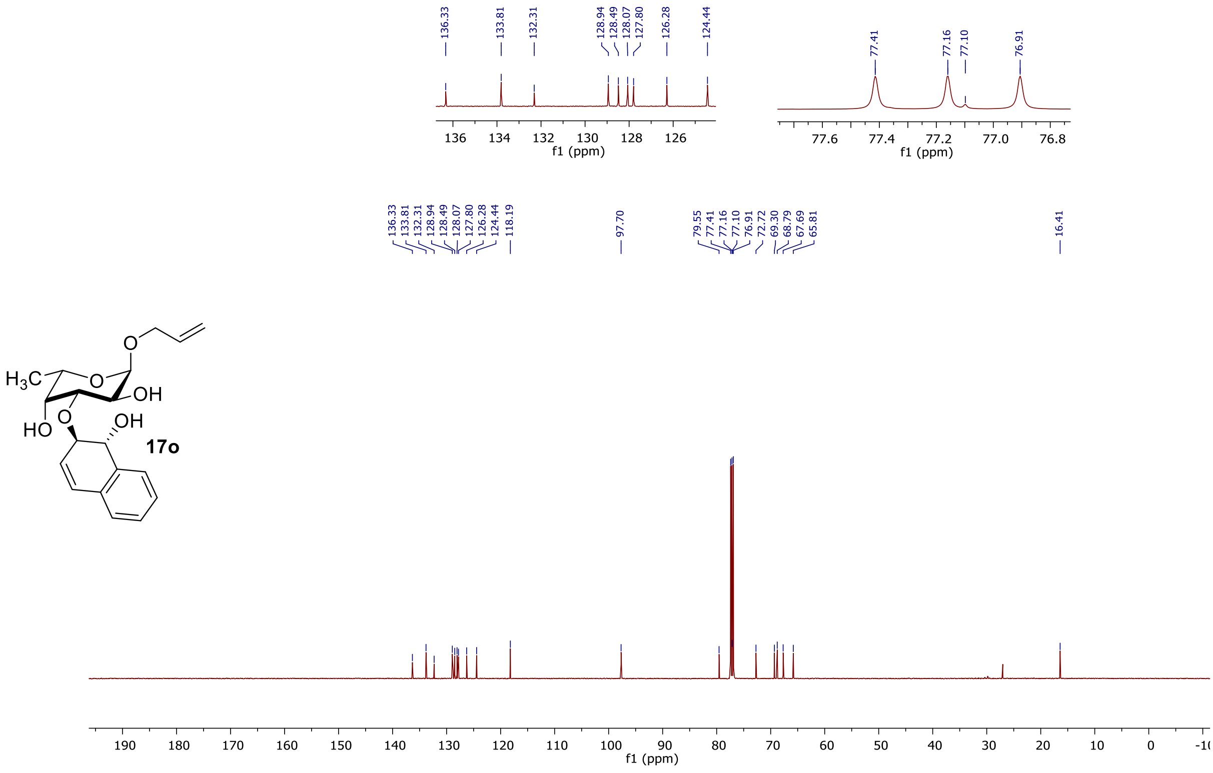

Supplementary Figure 203. ^{13}C spectra for **17o**

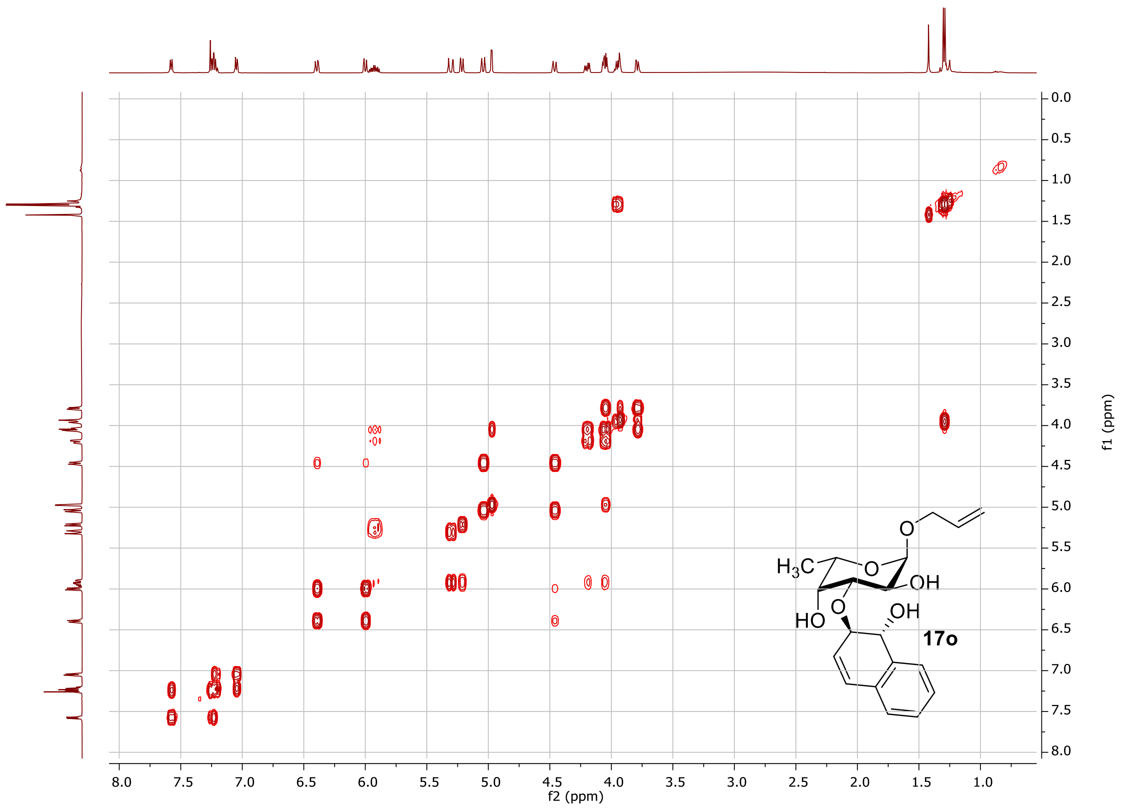

Supplementary Figure 204. COSY spectra for **17o**

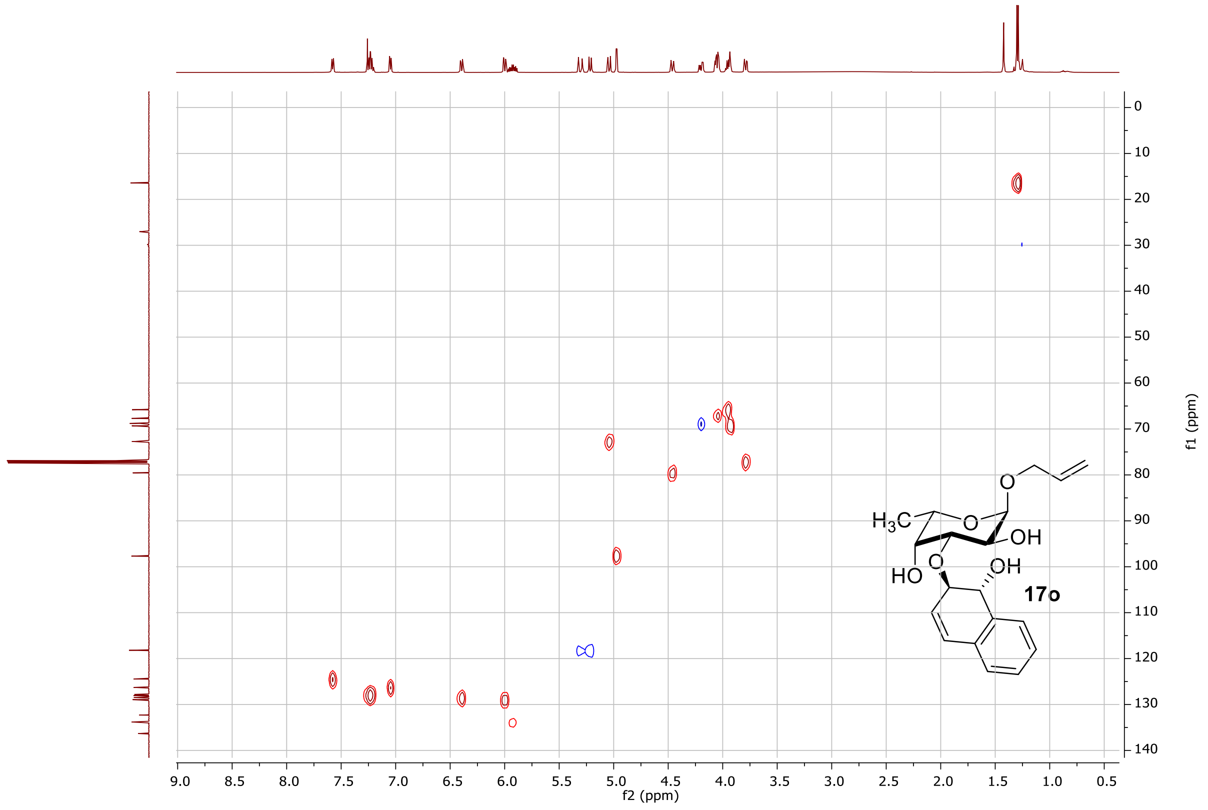

Supplementary Figure 205. HSQC spectra for **17o**

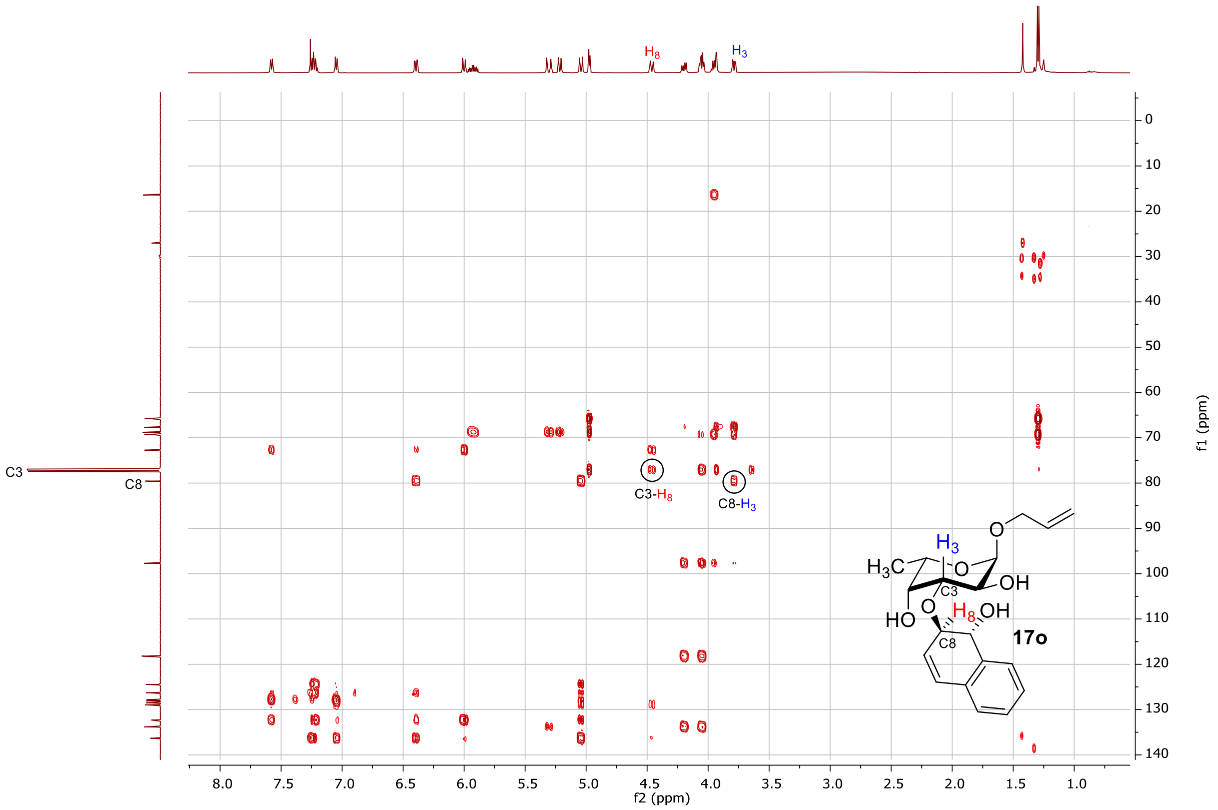

Supplementary Figure 206. HMBC spectra for **17o**

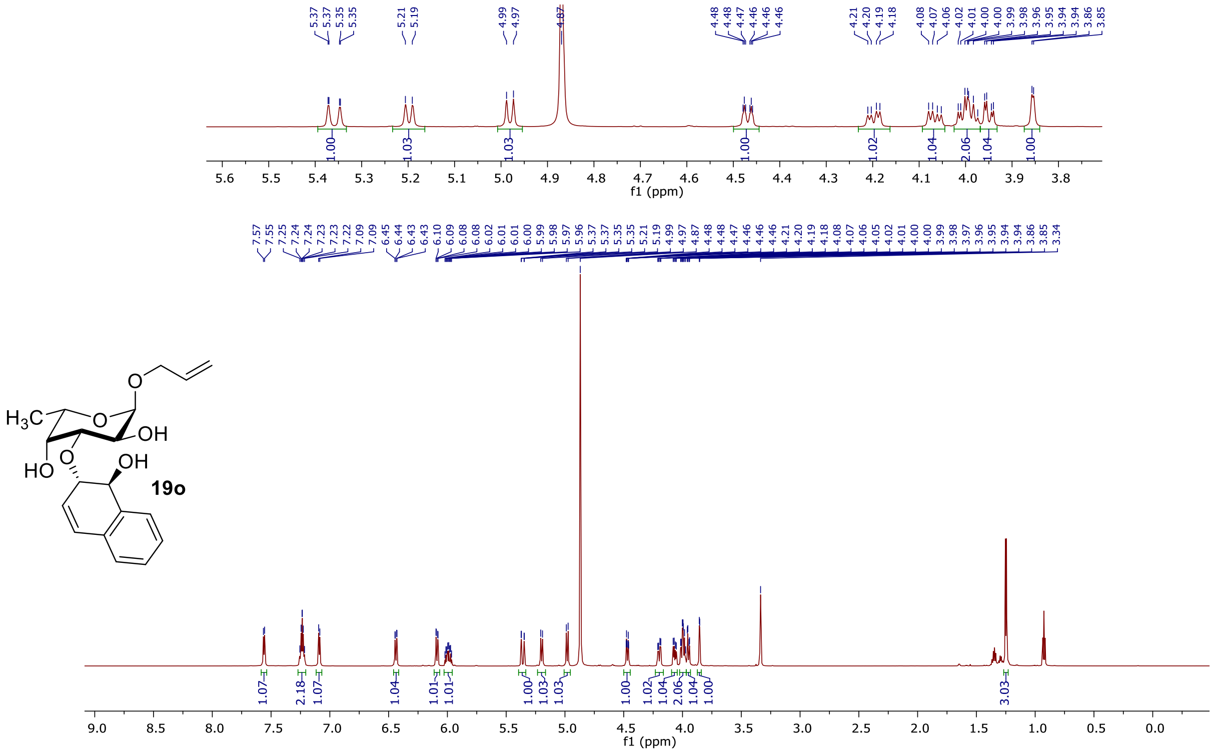

Supplementary Figure 207. ^{1}H spectra for **19o**

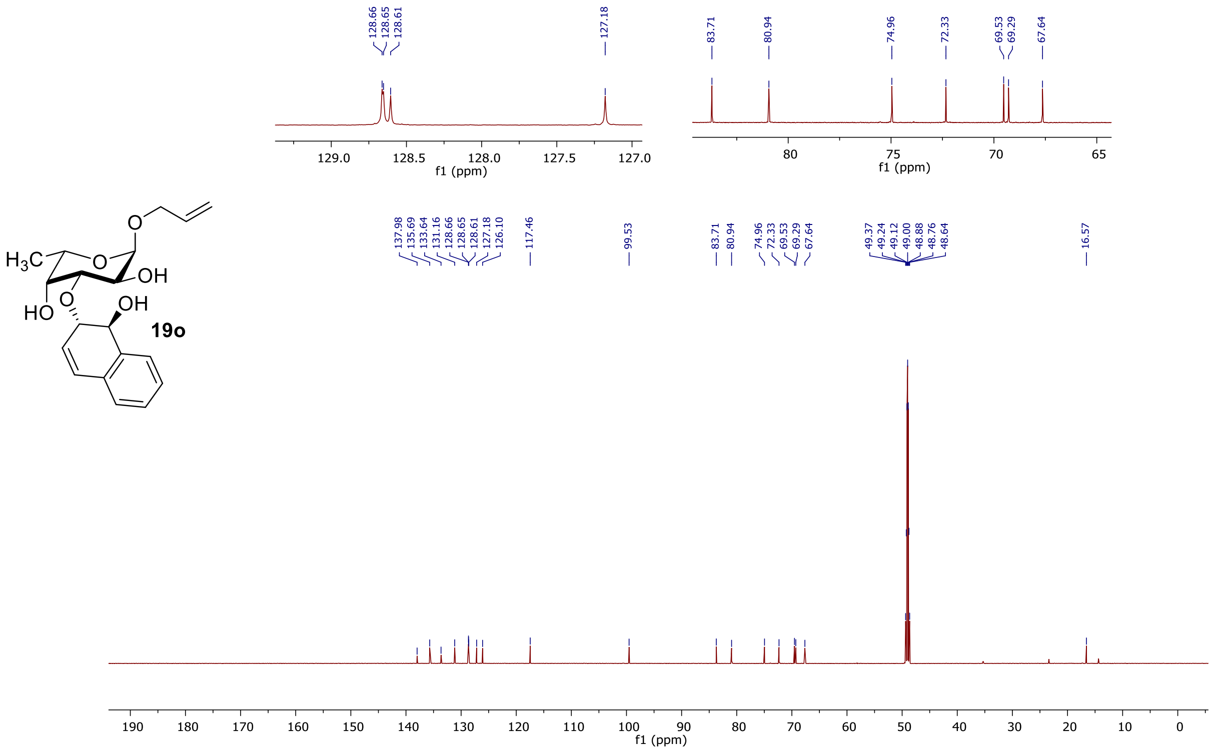

Supplementary Figure 208. ^{13}C spectra for **19o**

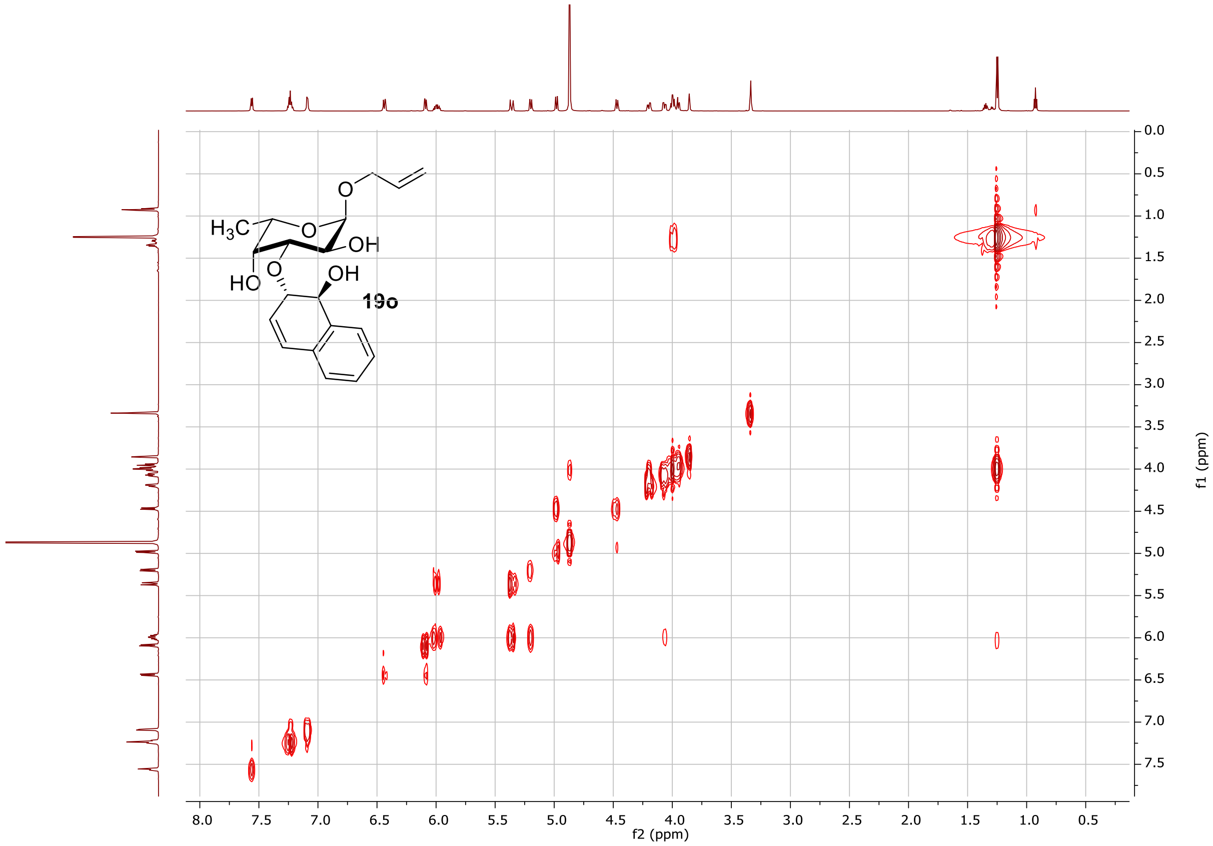

Supplementary Figure 209. COSY spectra for **19o**

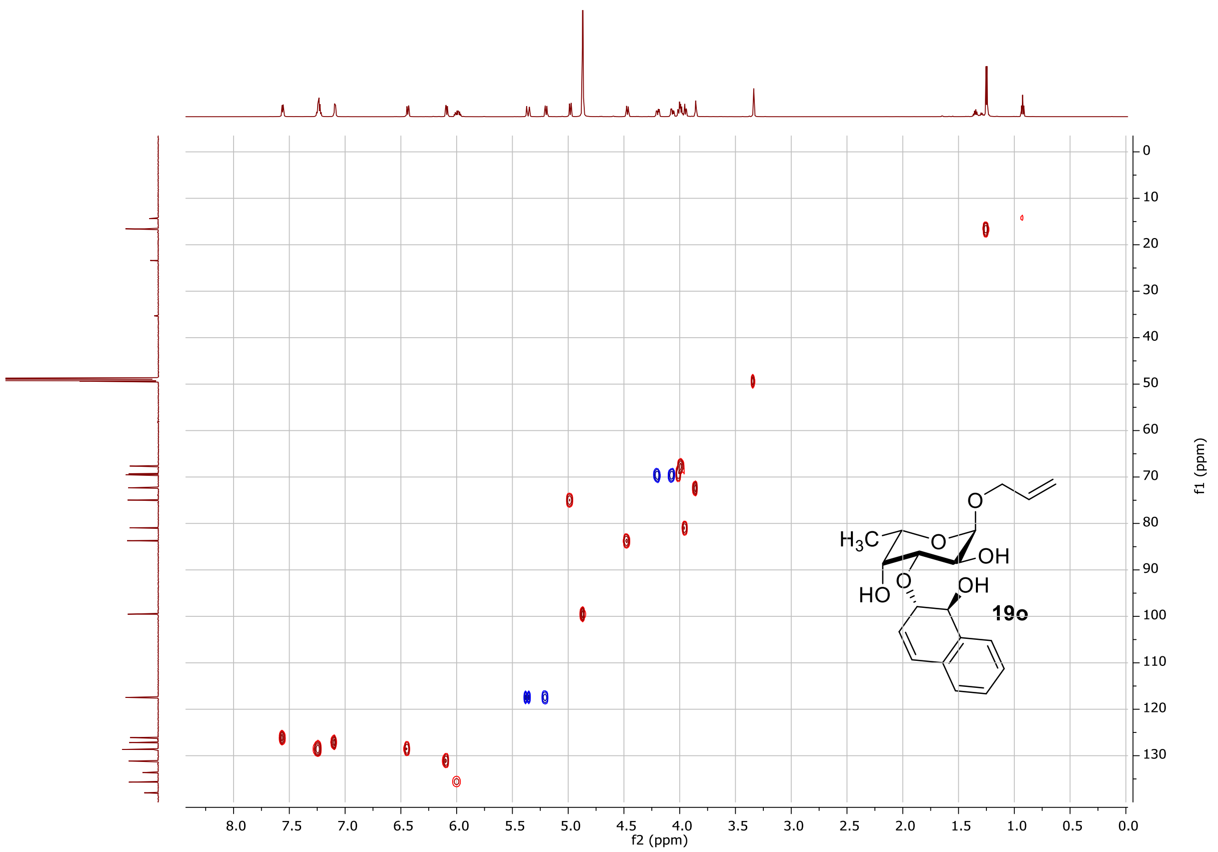

Supplementary Figure 210. HSQC spectra for **19o**

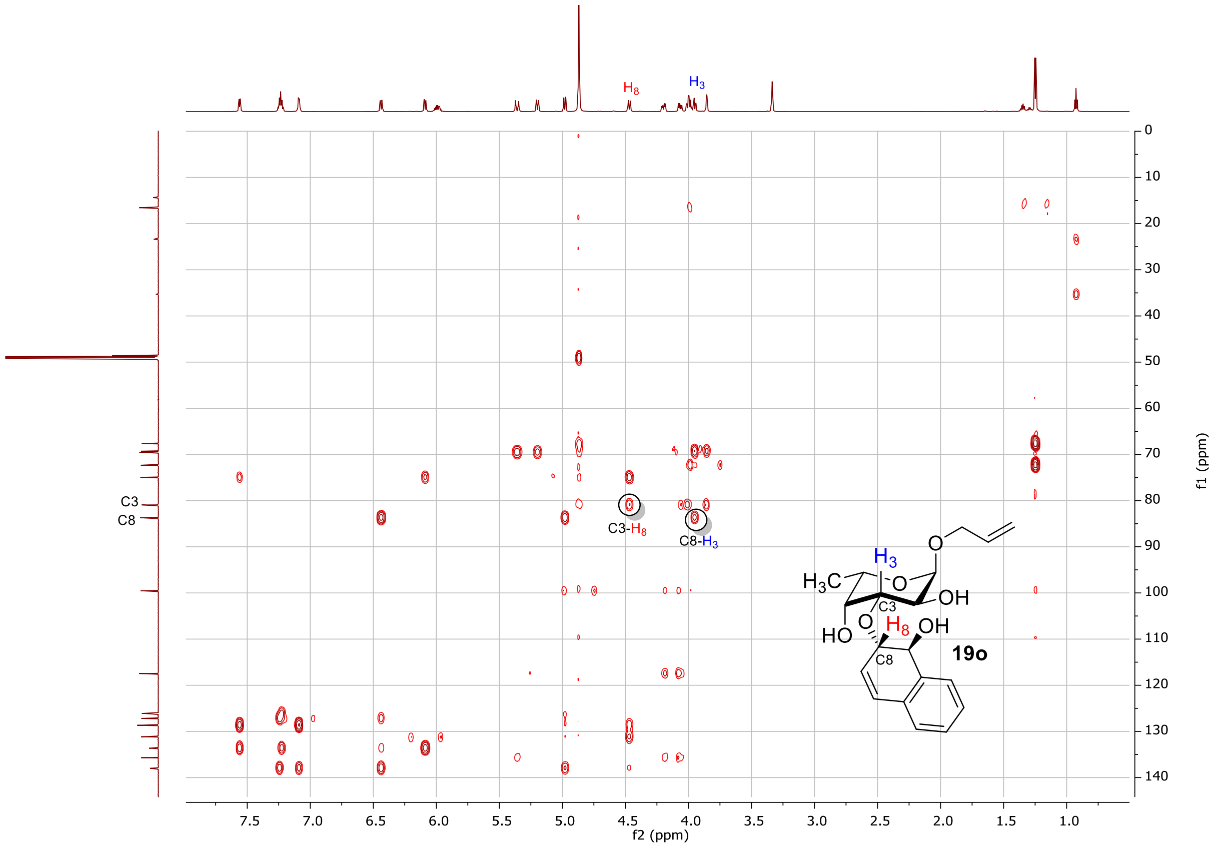

Supplementary Figure 211. HMBC spectra for **19o**

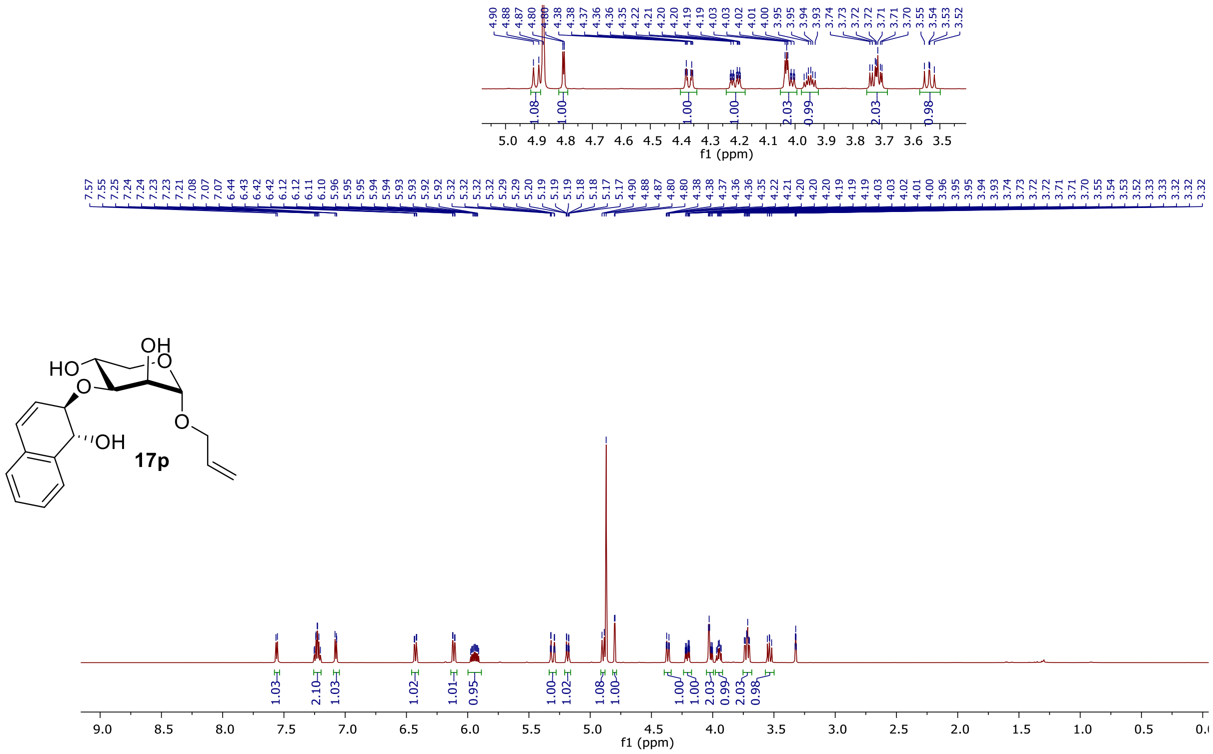

Supplementary Figure 212. ^{1}H spectra for **17p**

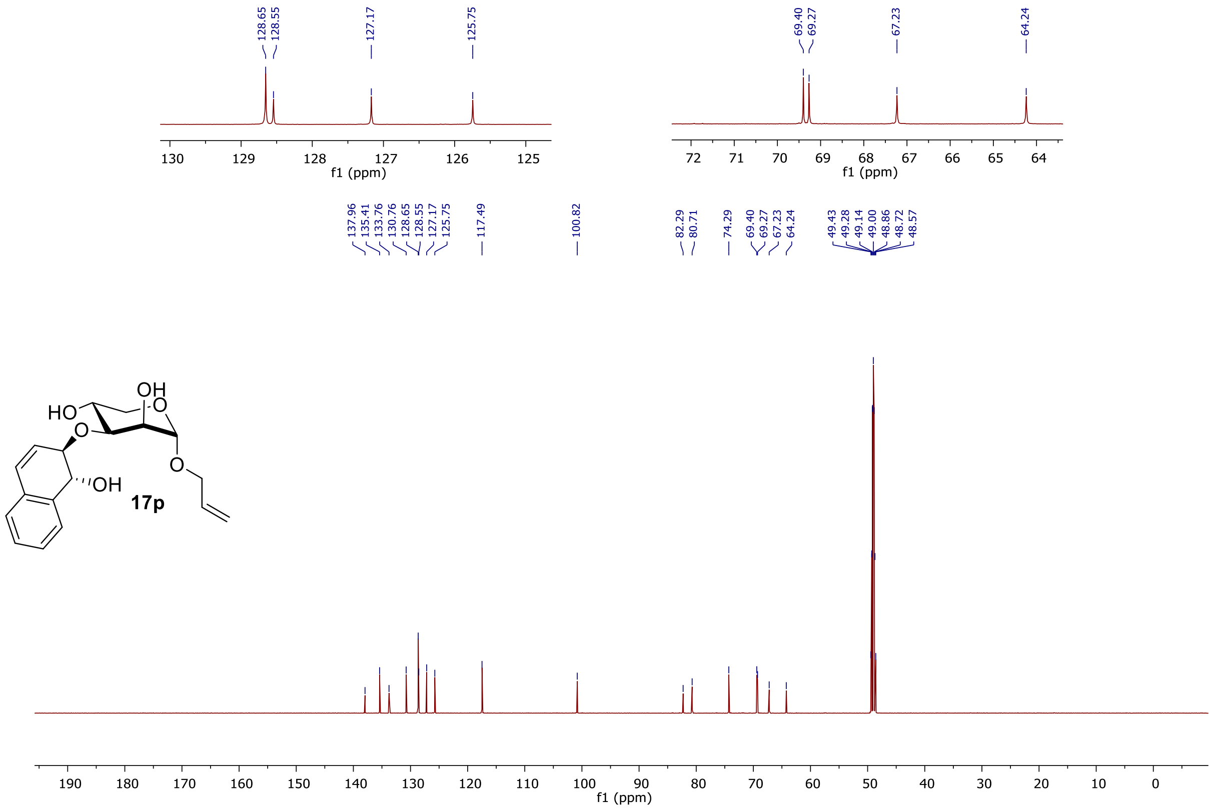

Supplementary Figure 213. ^{13}C spectra for **17p**

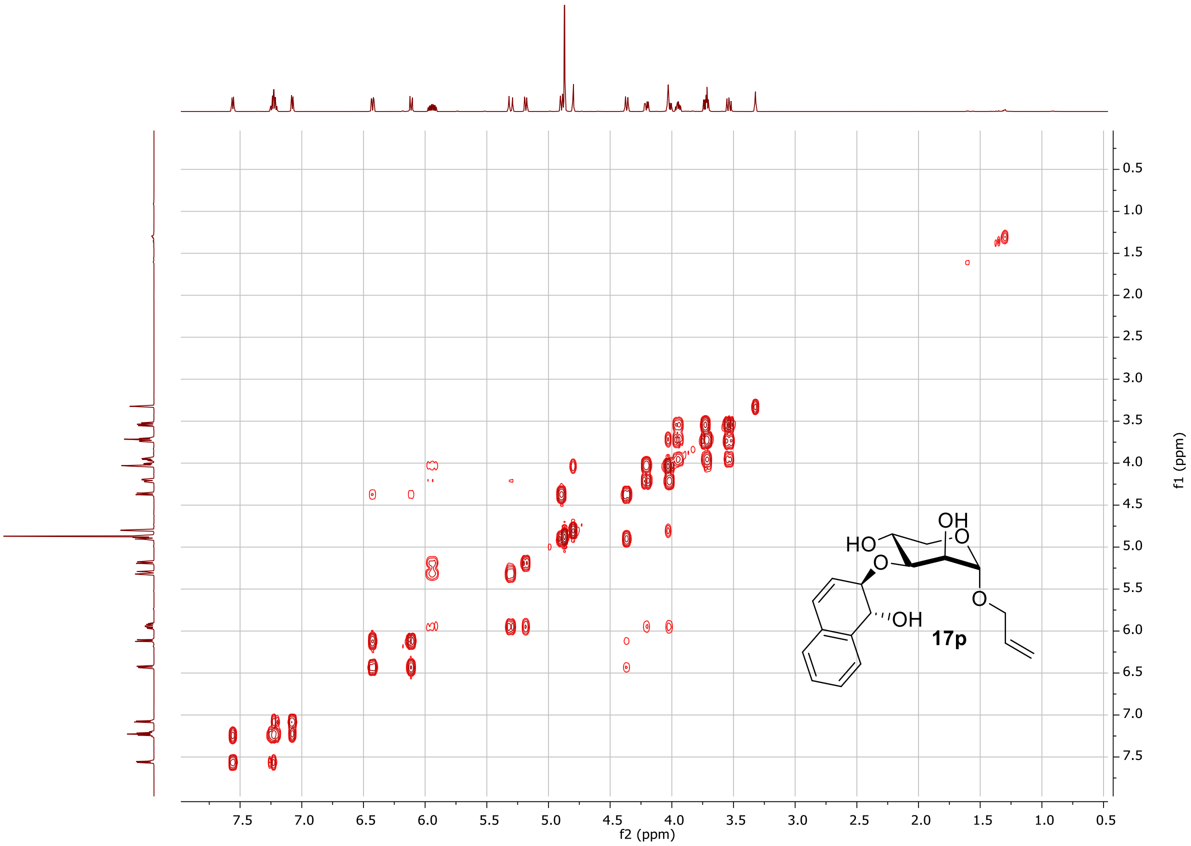

Supplementary Figure 214. COSY spectra for **17p**

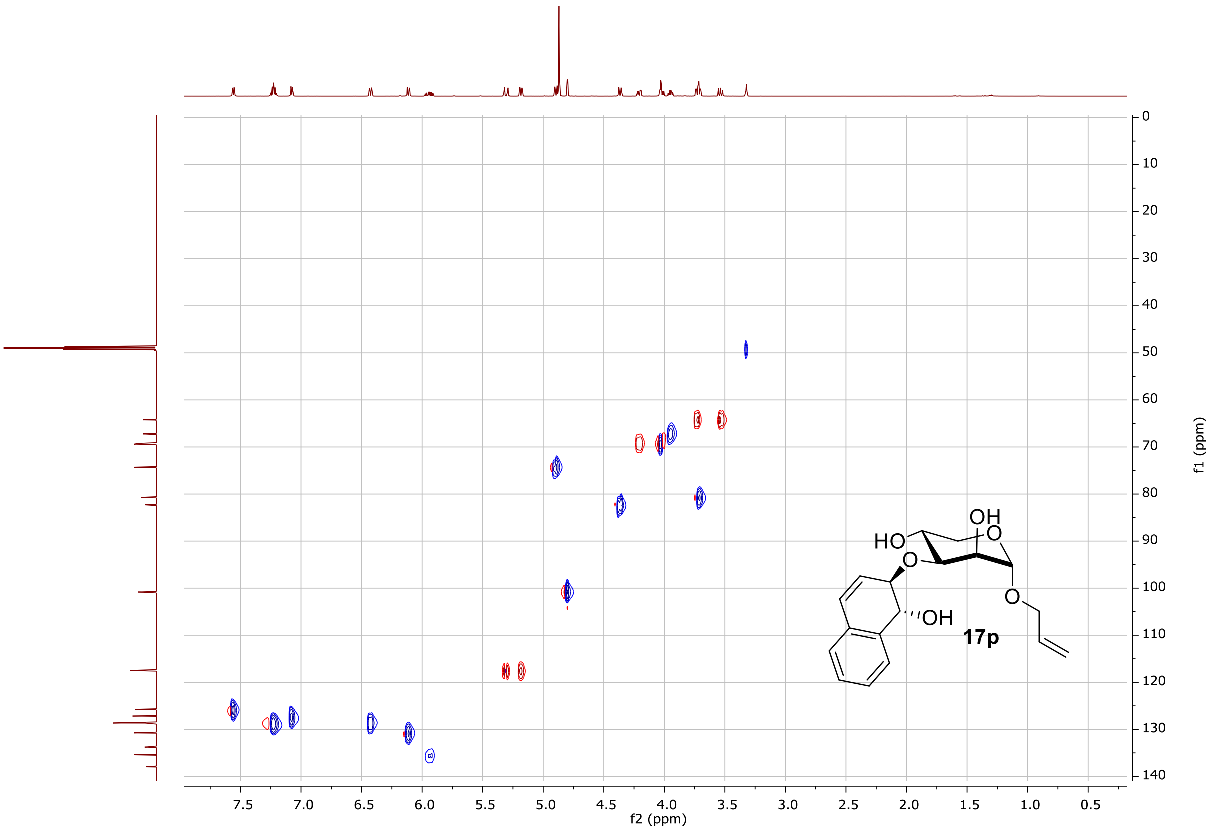

Supplementary Figure 215. HSQC spectra for **17p**

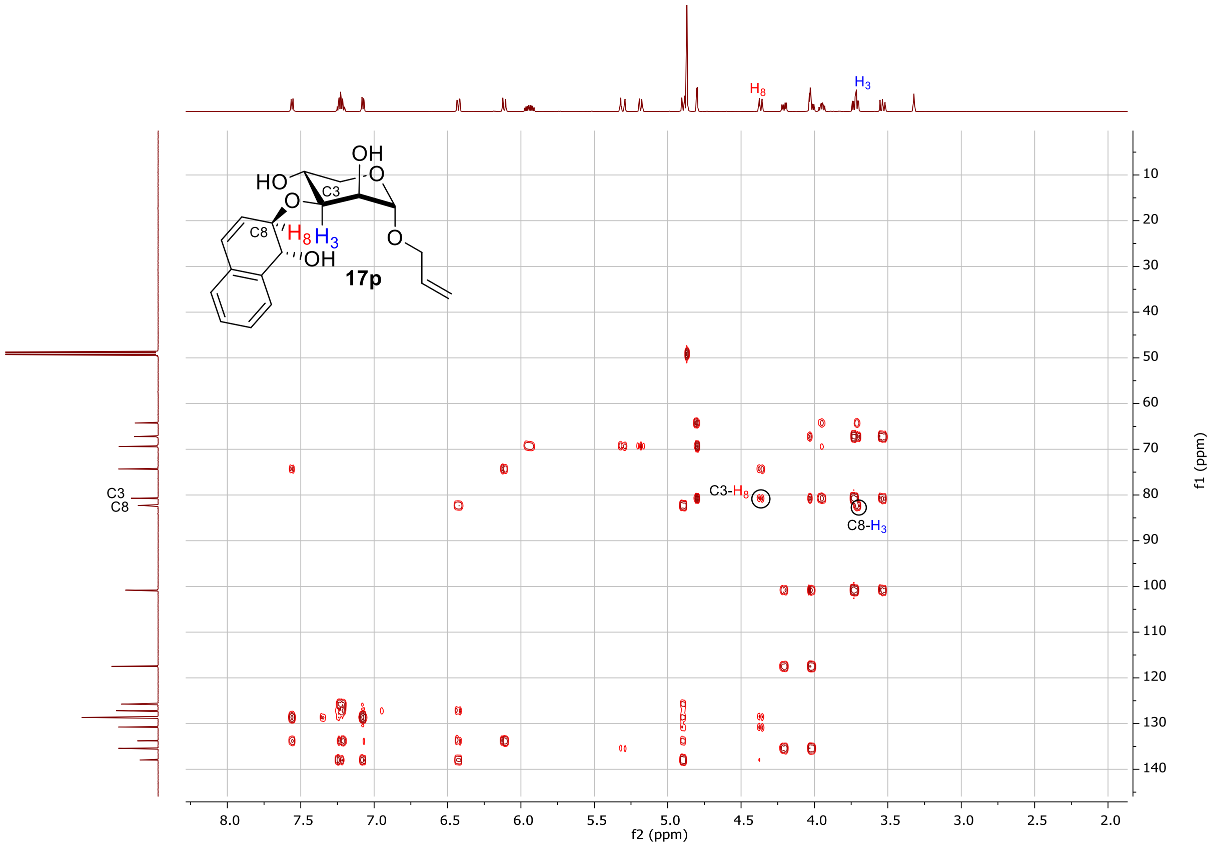

Supplementary Figure 216. HMBC spectra for **17p**

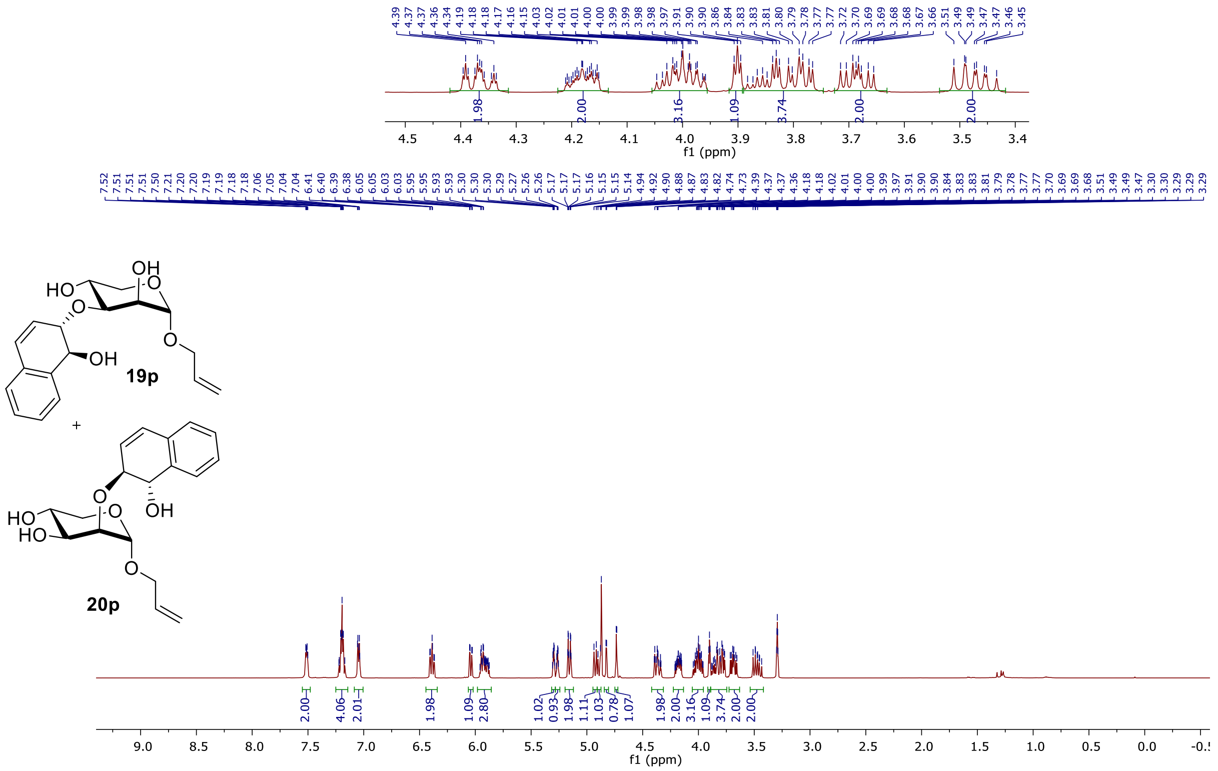

Supplementary Figure 217. ^{1}H spectra for **20p**

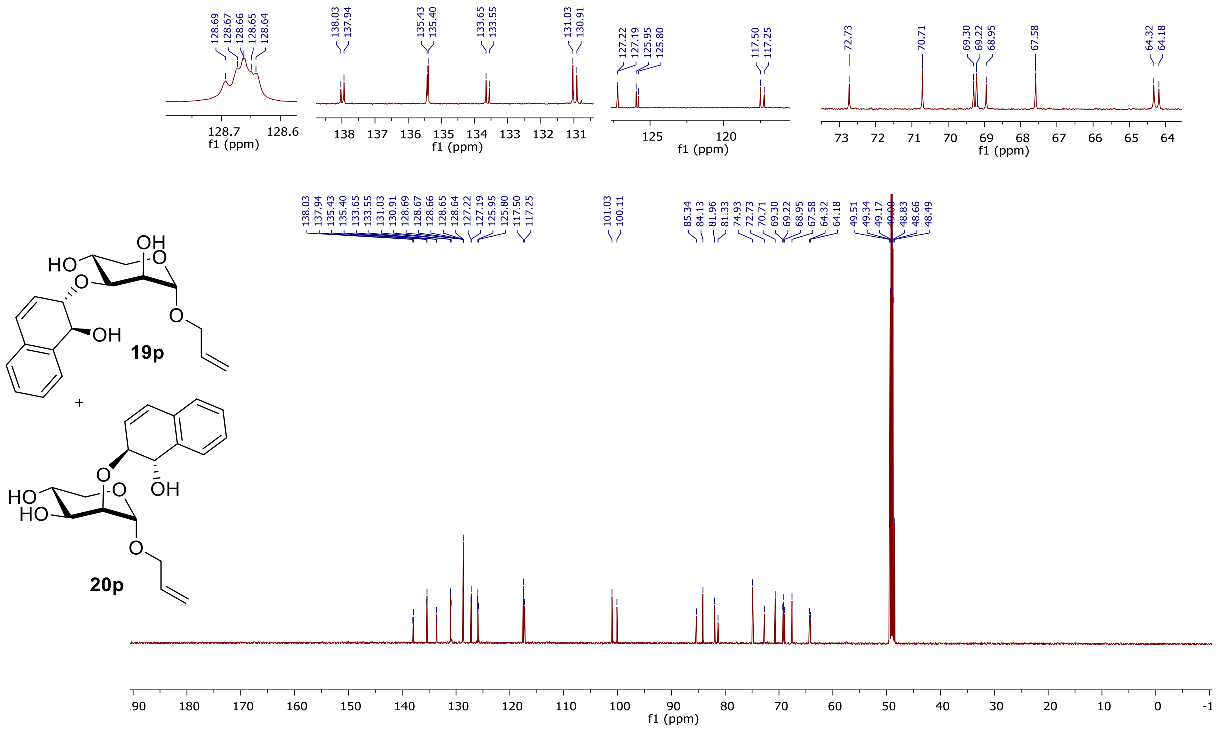

Supplementary Figure 218. ^{13}C spectra for **20p**

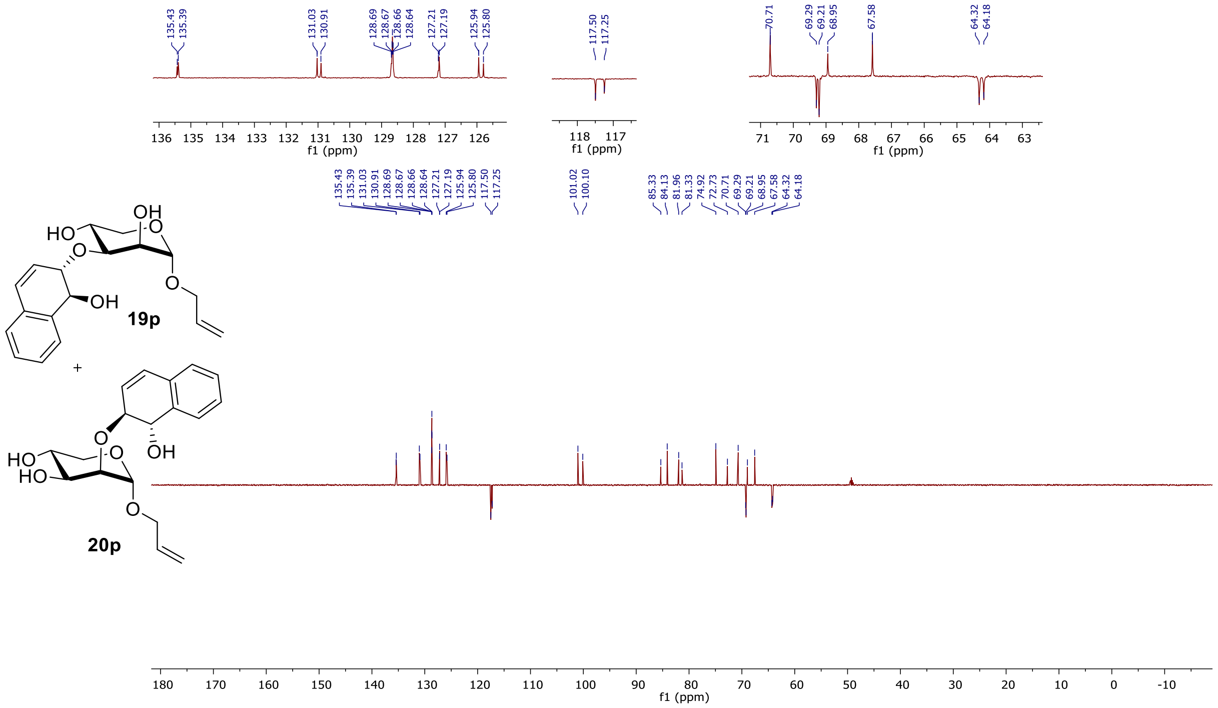

Supplementary Figure 219. DEPT spectra for **20p**

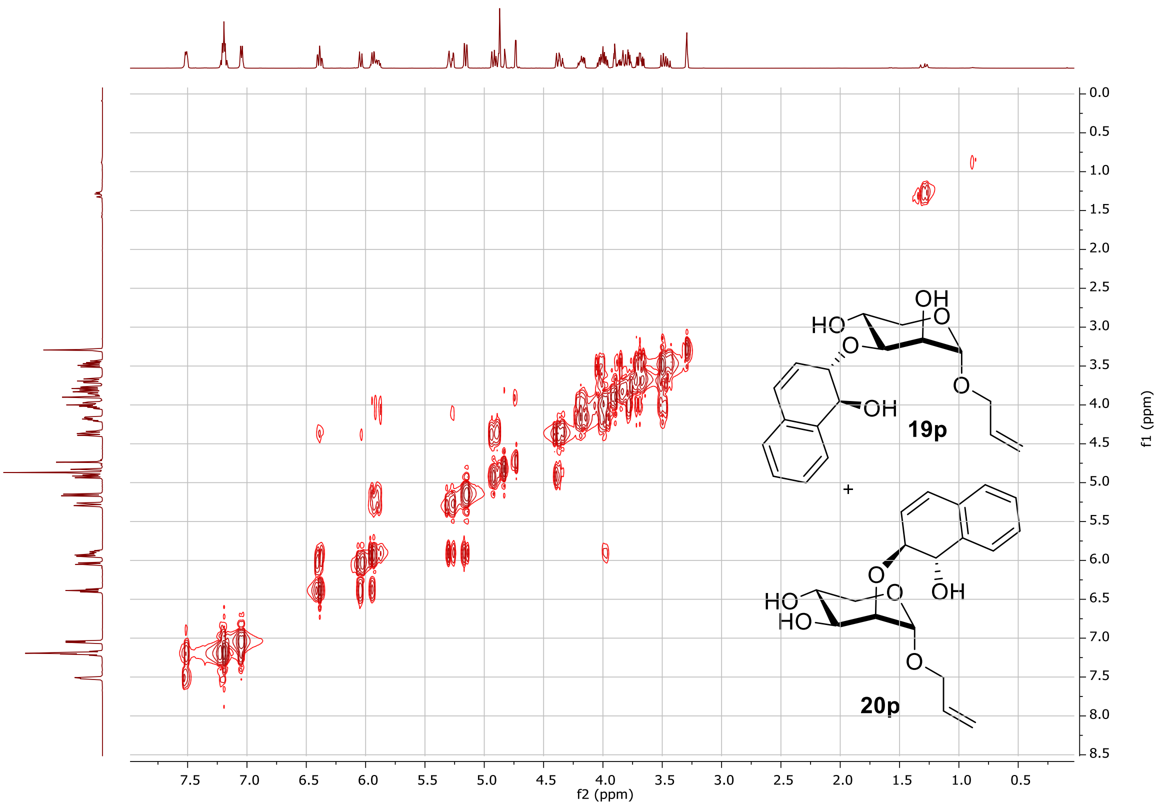

Supplementary Figure 220. COSY spectra for **20p**

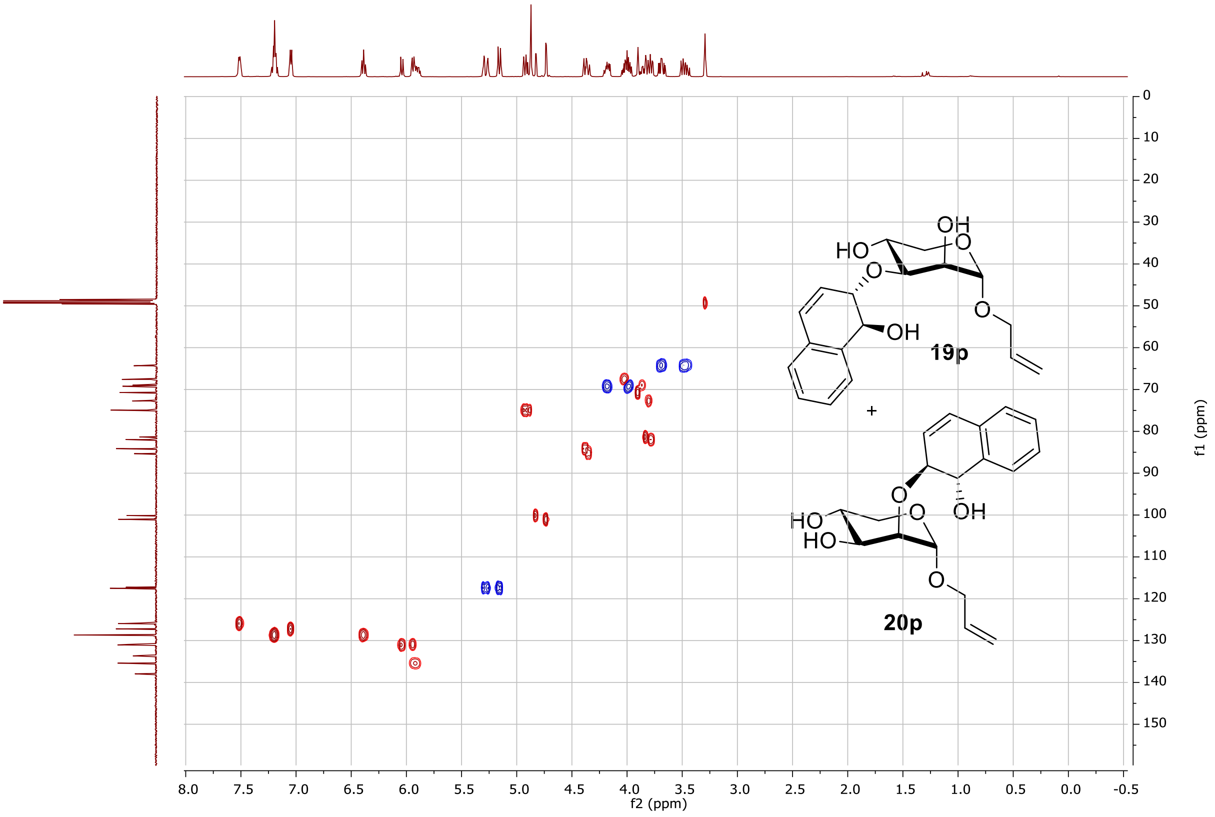

Supplementary Figure 221. HSQC spectra for **20p**

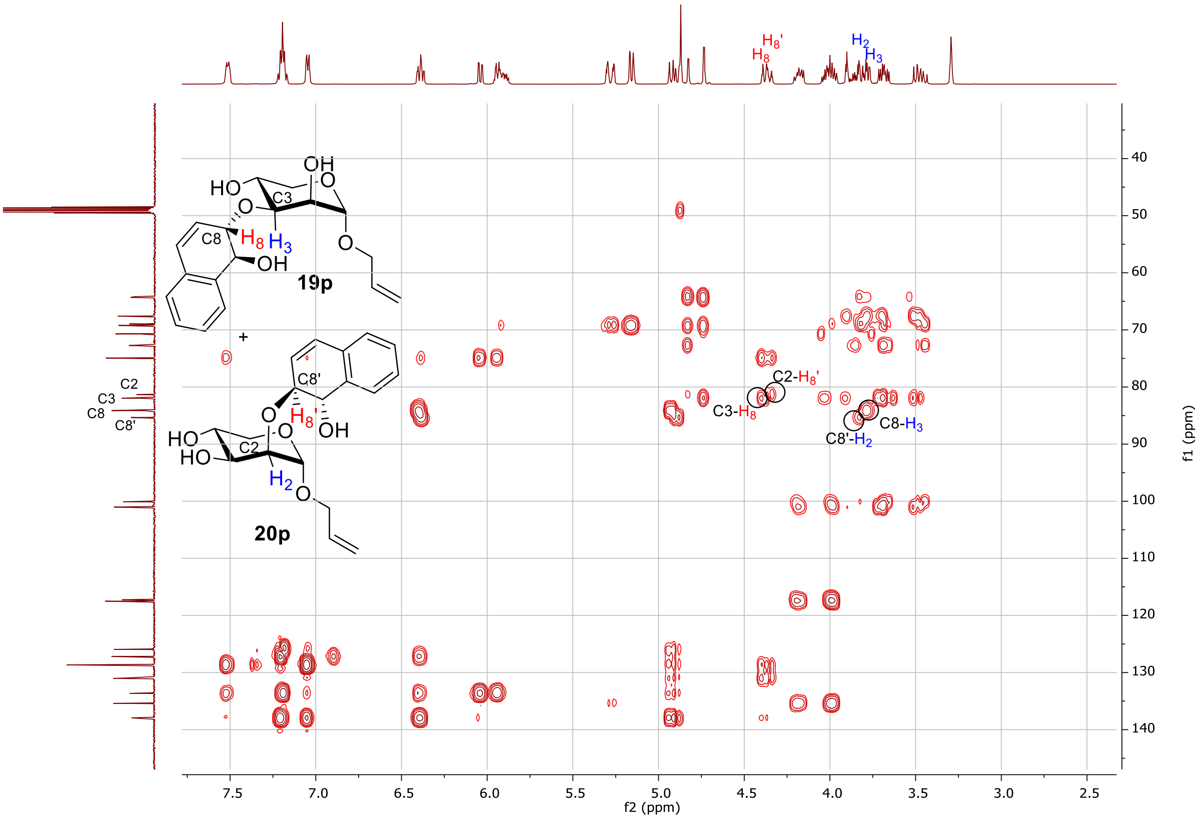

Supplementary Figure 222. HMBC spectra for **20p**

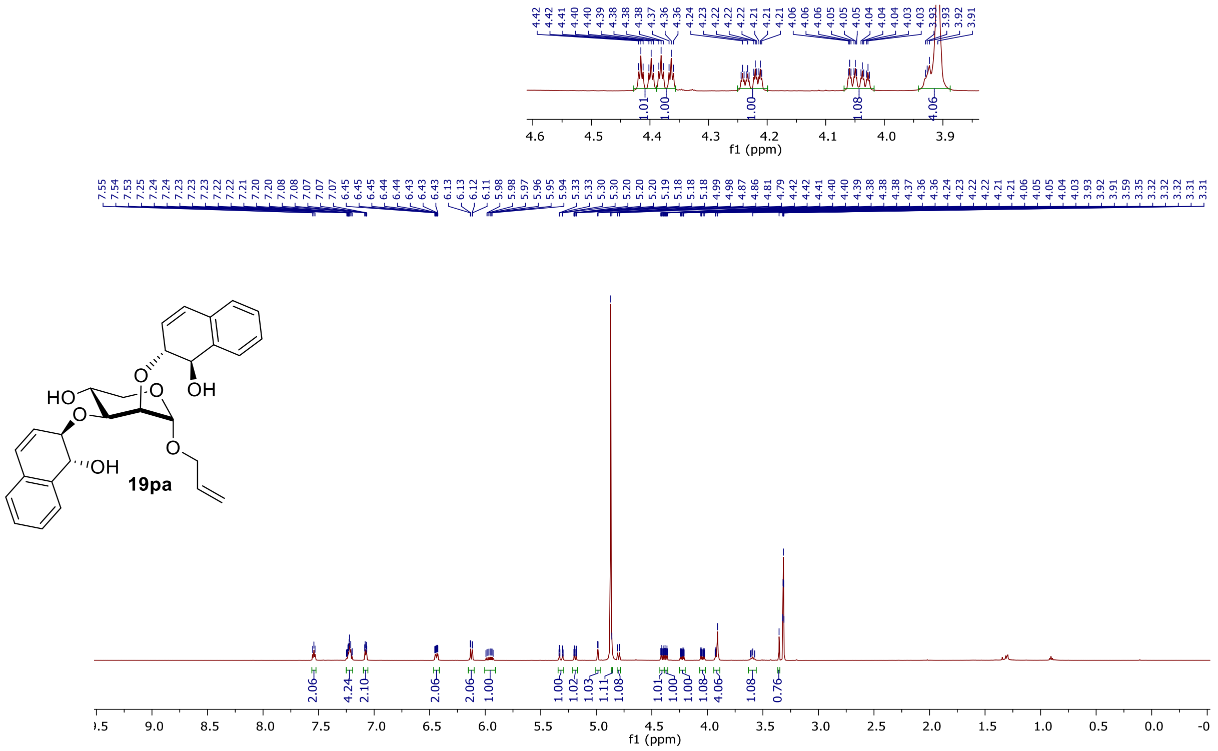

Supplementary Figure 223. ^{1}H spectra for **19pa**

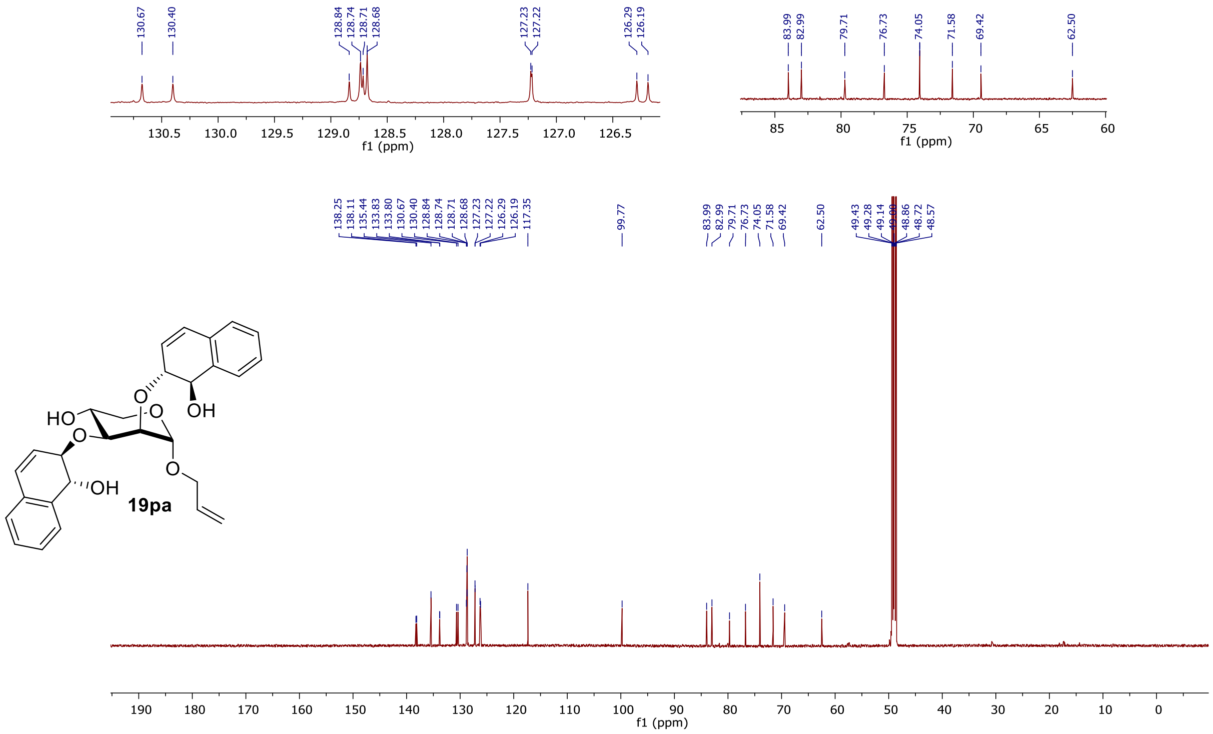

Supplementary Figure 224. ^{1}H spectra for **19pa**

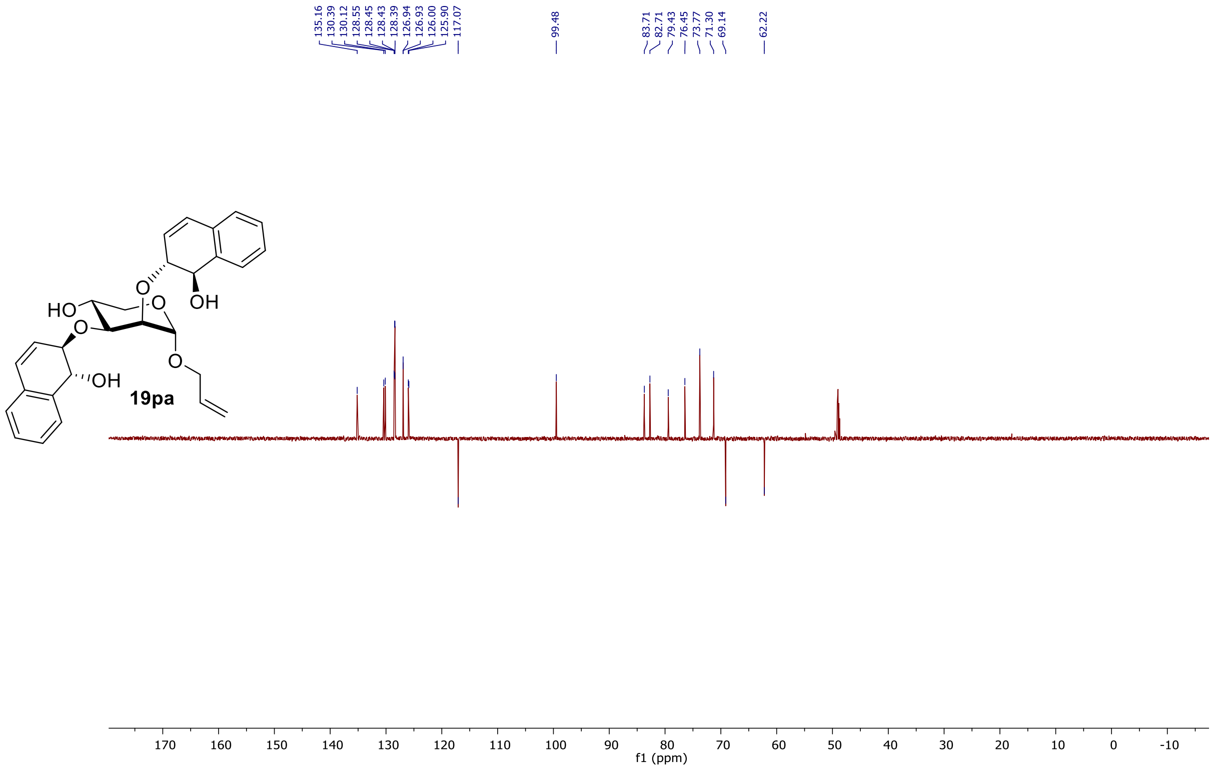

Supplementary Figure 225. ^{13}C spectra for **19pa**

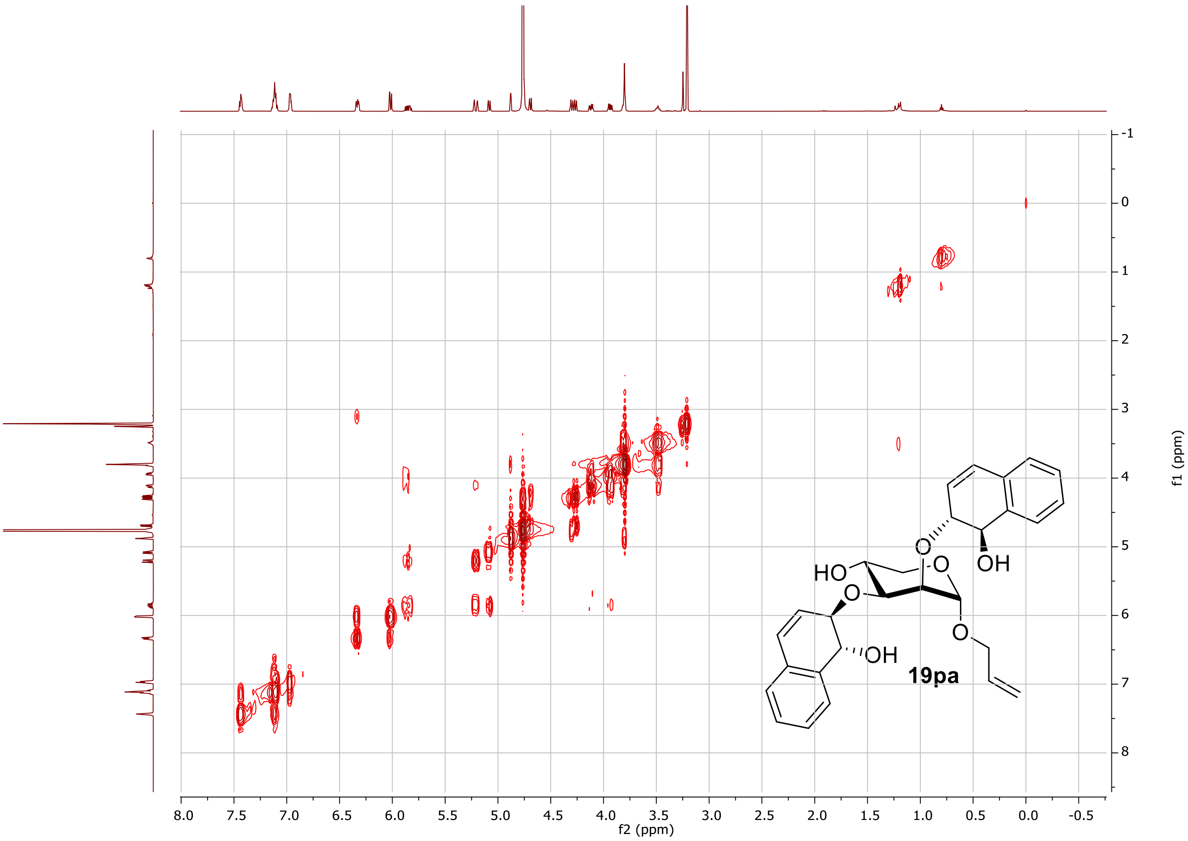

Supplementary Figure 226. COSY spectra for **19pa**

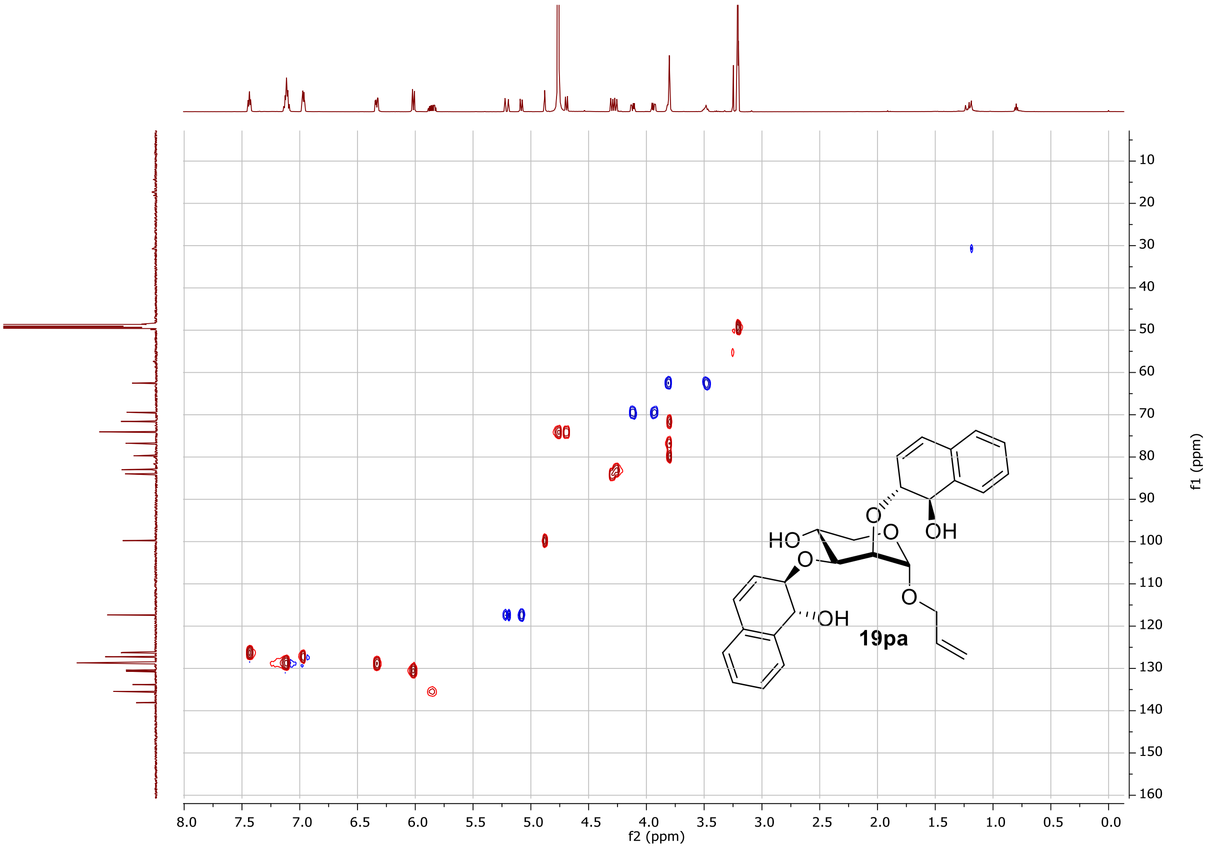

Supplementary Figure 227. HSQC spectra for **19pa**

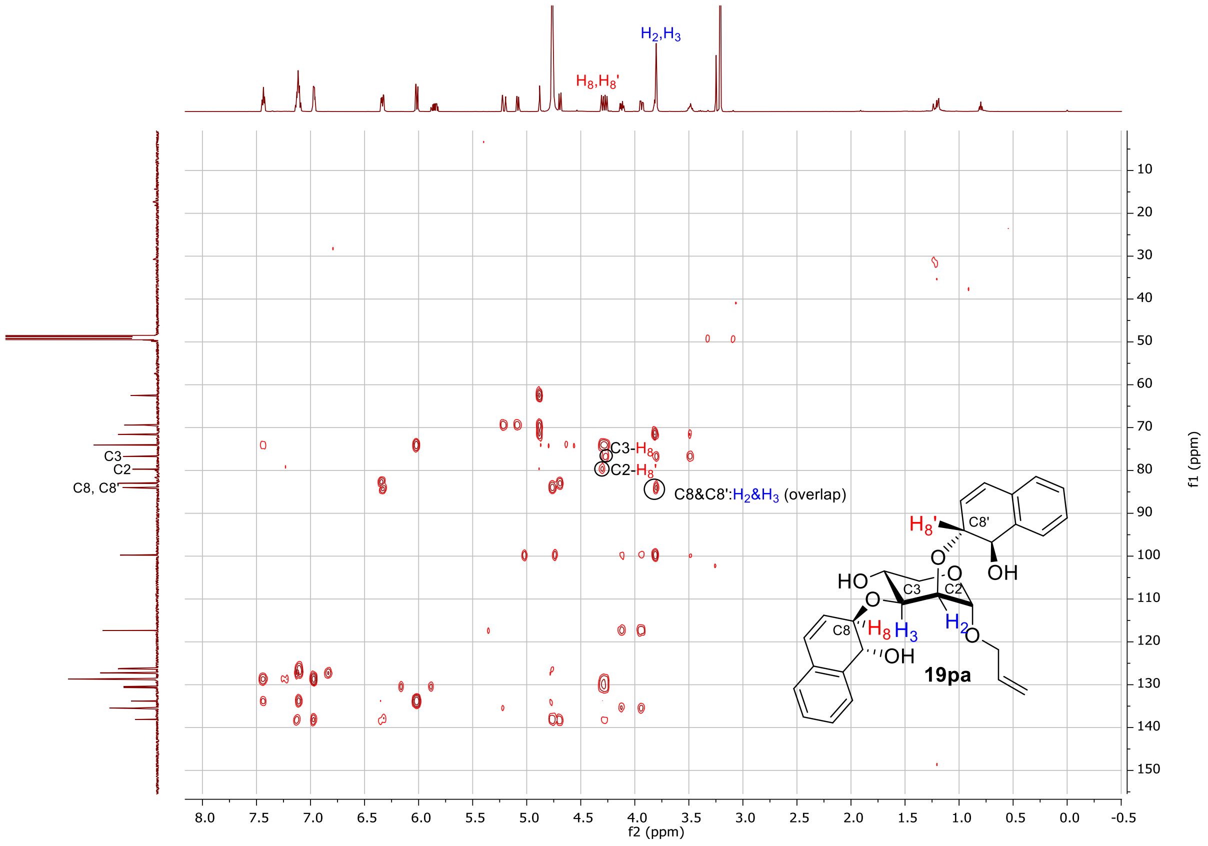

Supplementary Figure 228. HMBC spectra for **19pa**

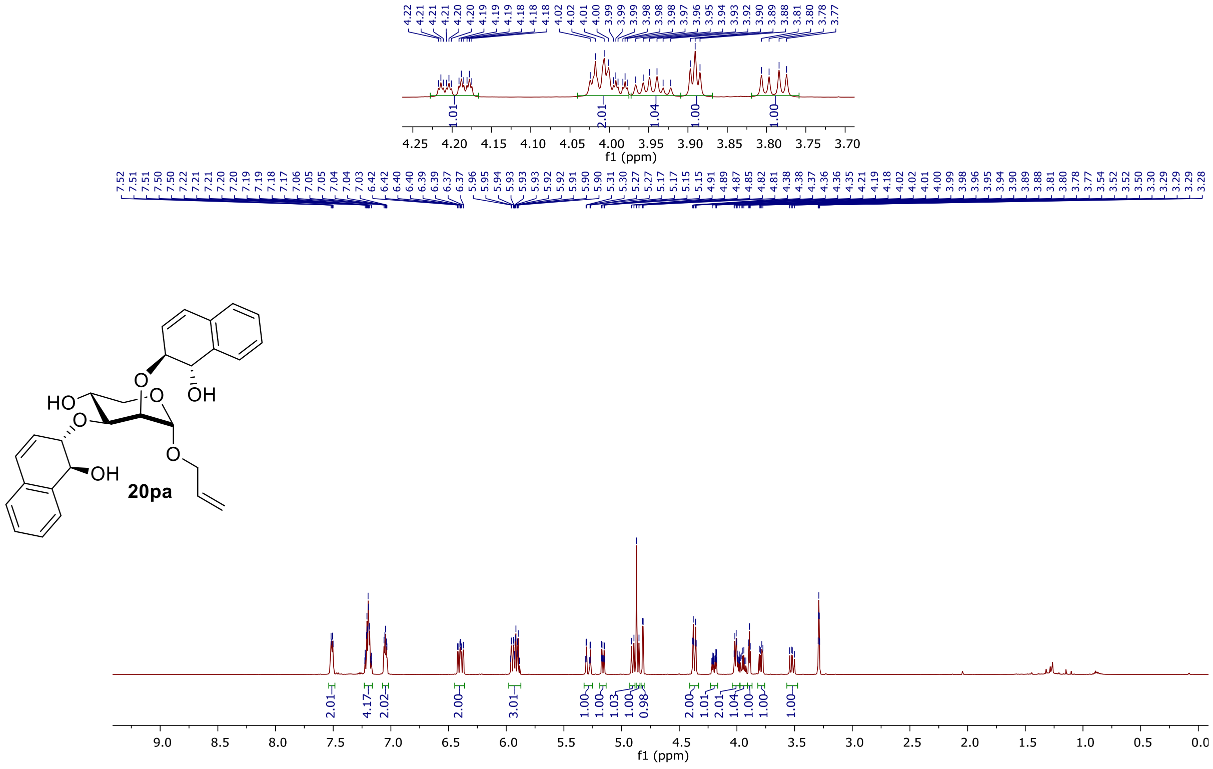

Supplementary Figure 229. ^{1}H spectra for **20pa**

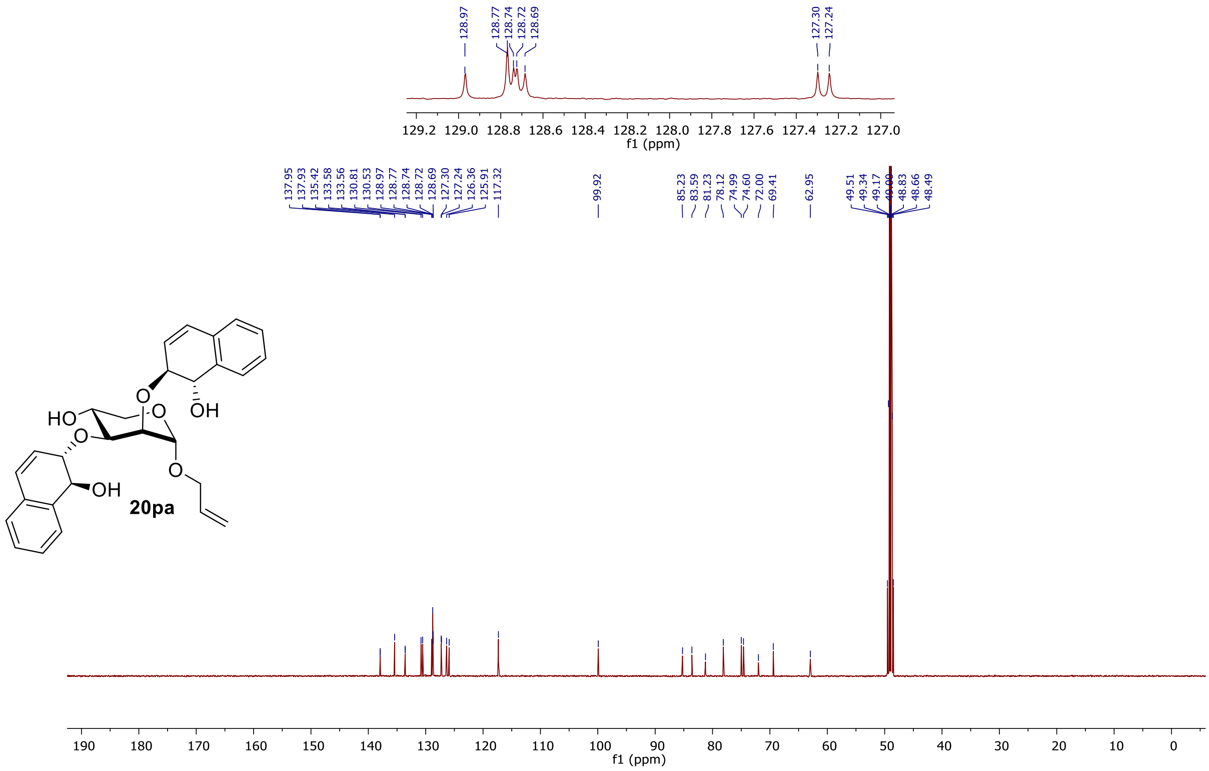

Supplementary Figure 230. ^{13}C spectra for **20pa**

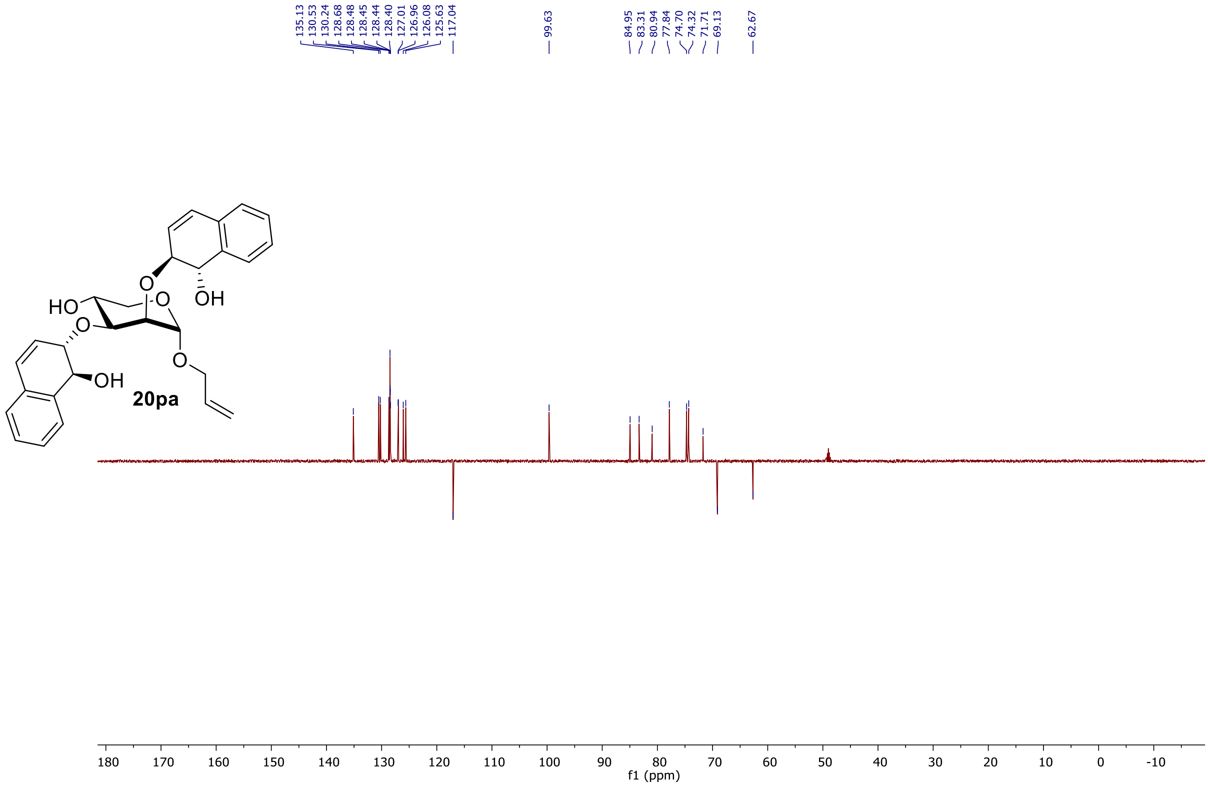

Supplementary Figure 231. DEPT spectra for **20pa**

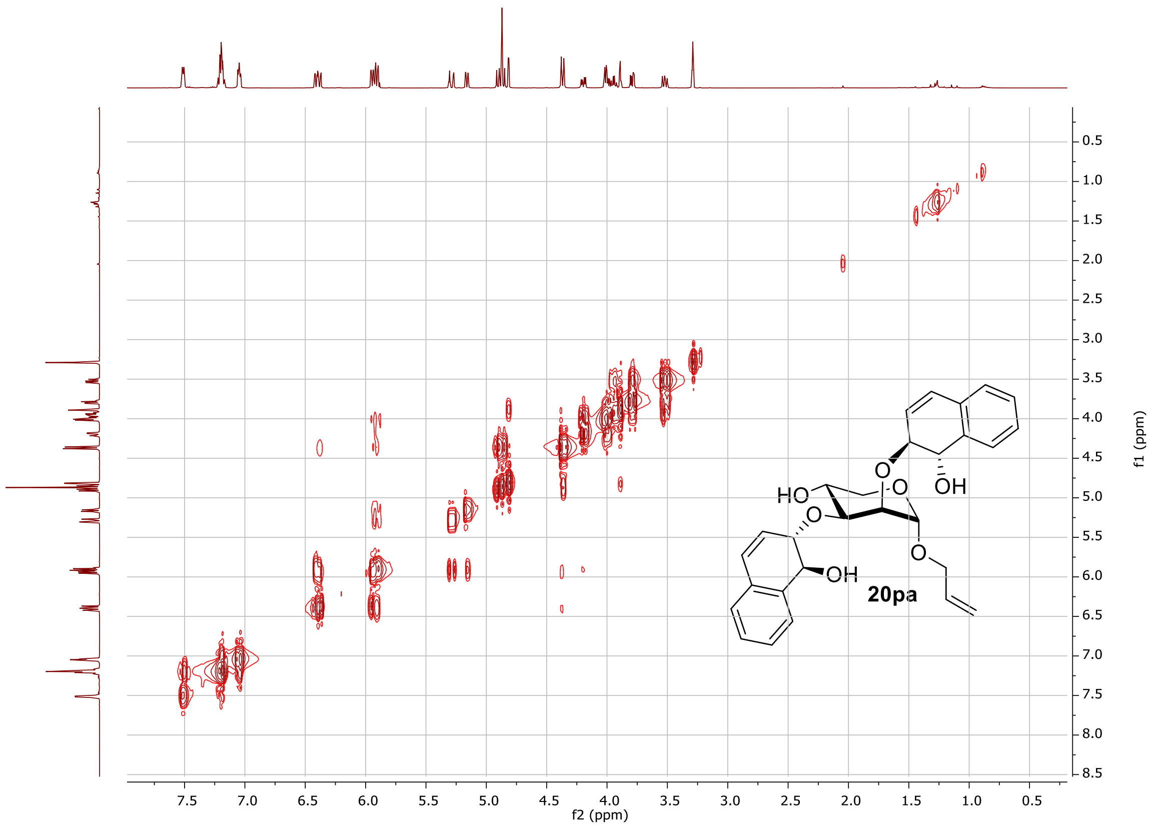

Supplementary Figure 232. COSY spectra for **20pa**

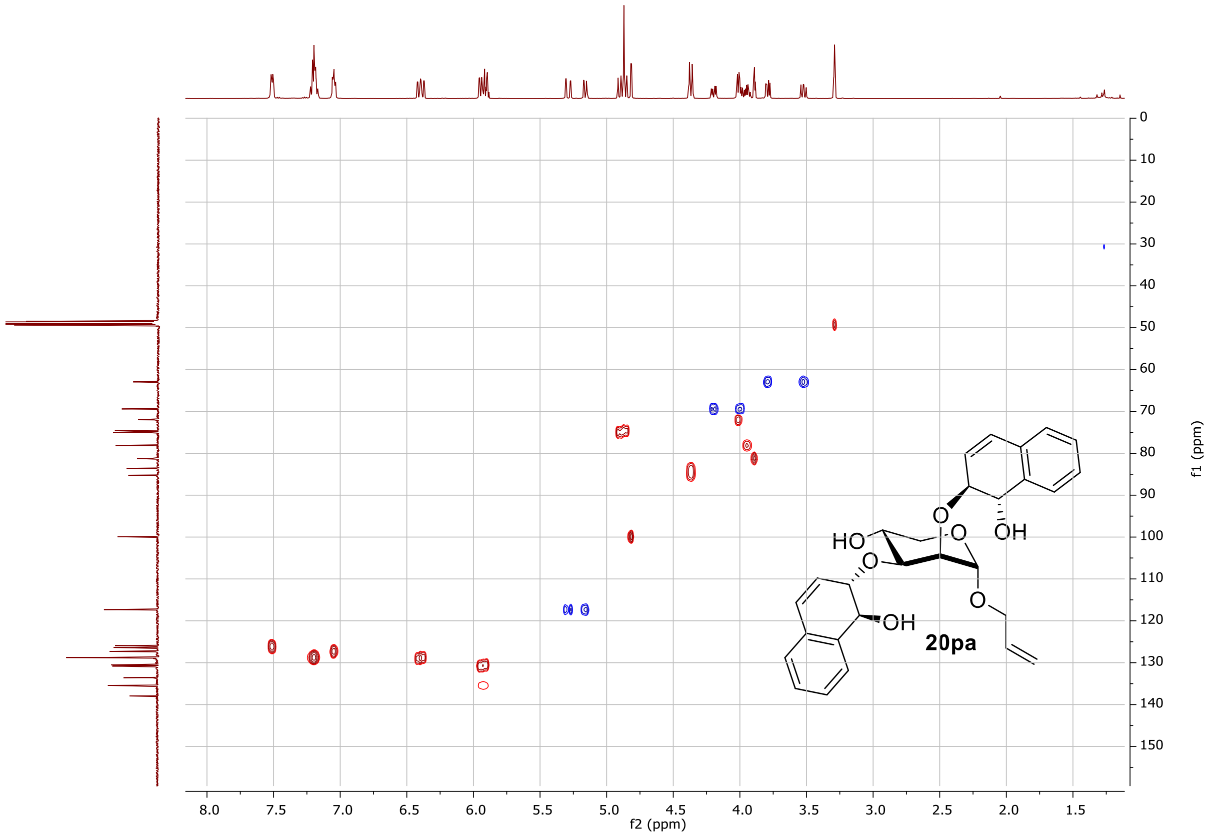

Supplementary Figure 233. HSQC spectra for **20pa**

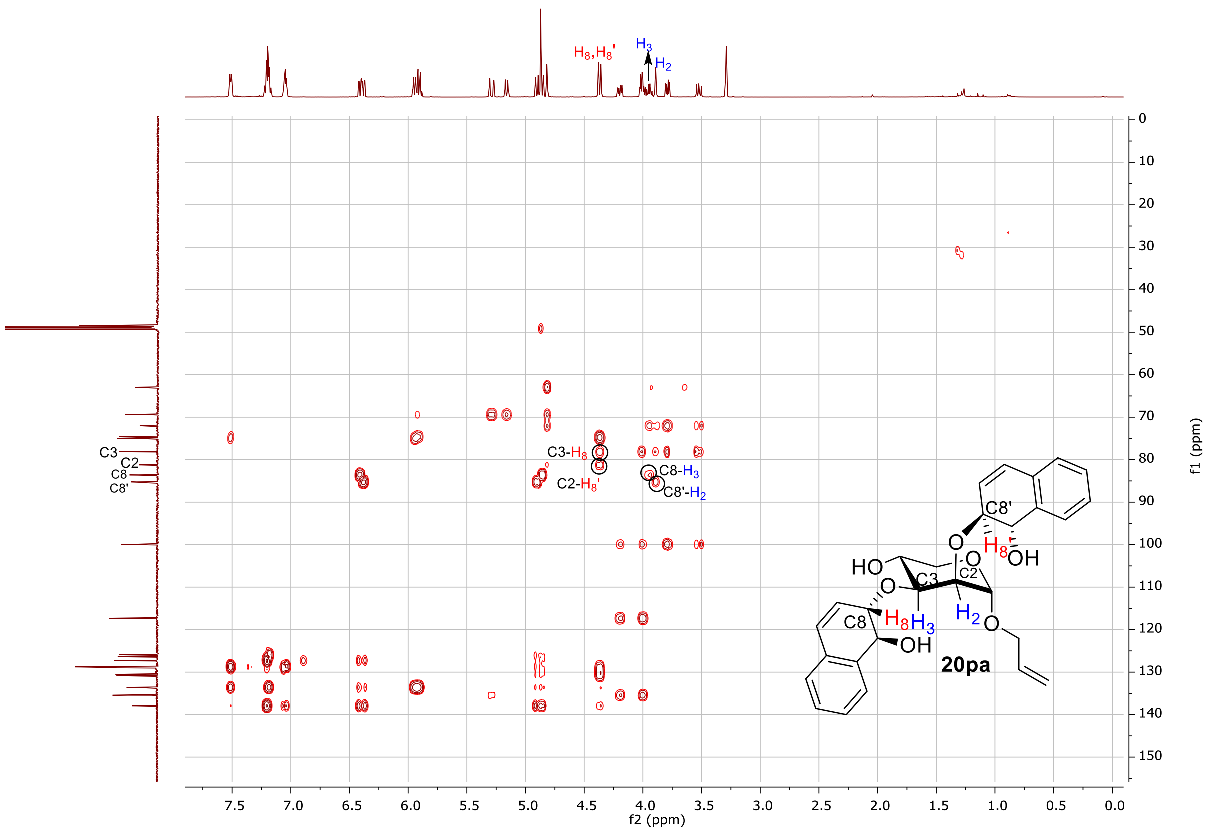

Supplementary Figure 234. HMBC spectra for **20pa**

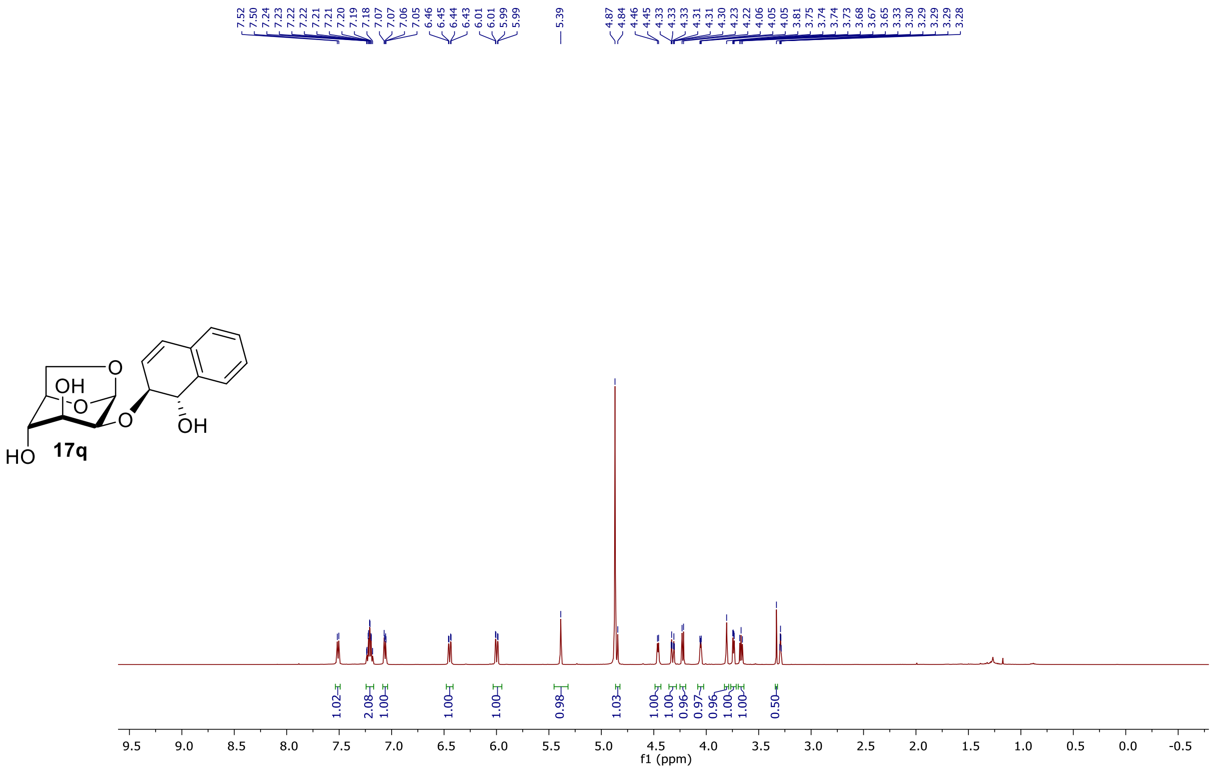

Supplementary Figure 235. ^{1}H spectra for **17q**

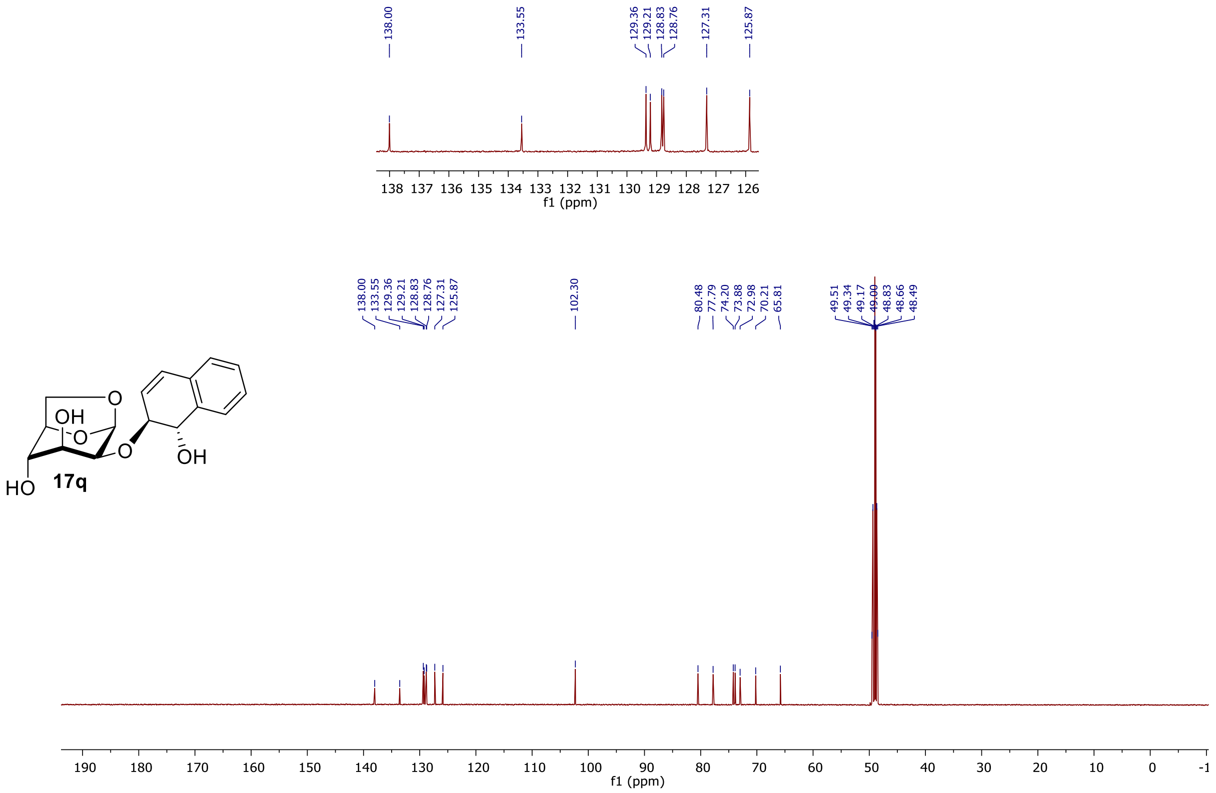

Supplementary Figure 236. ^{13}C spectra for **17q**

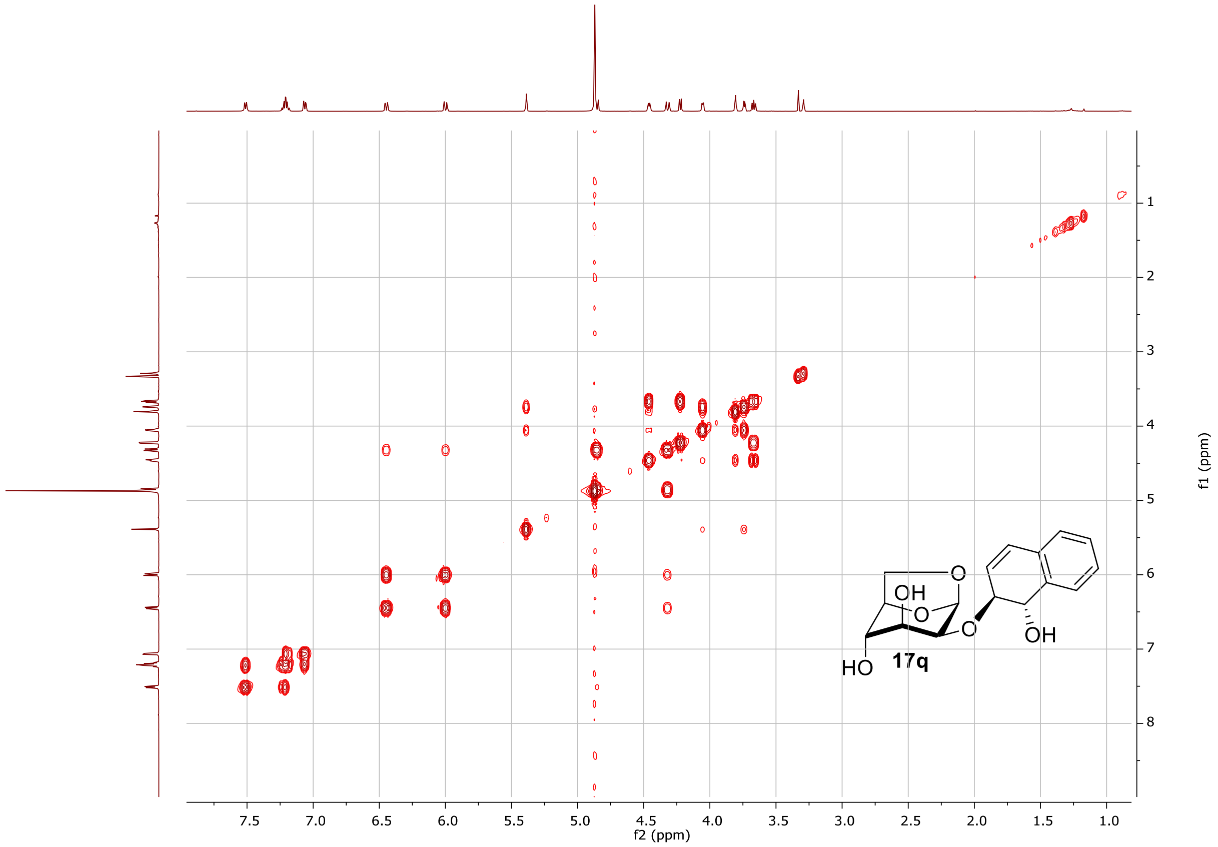

Supplementary Figure 237. COSY spectra for **17q**

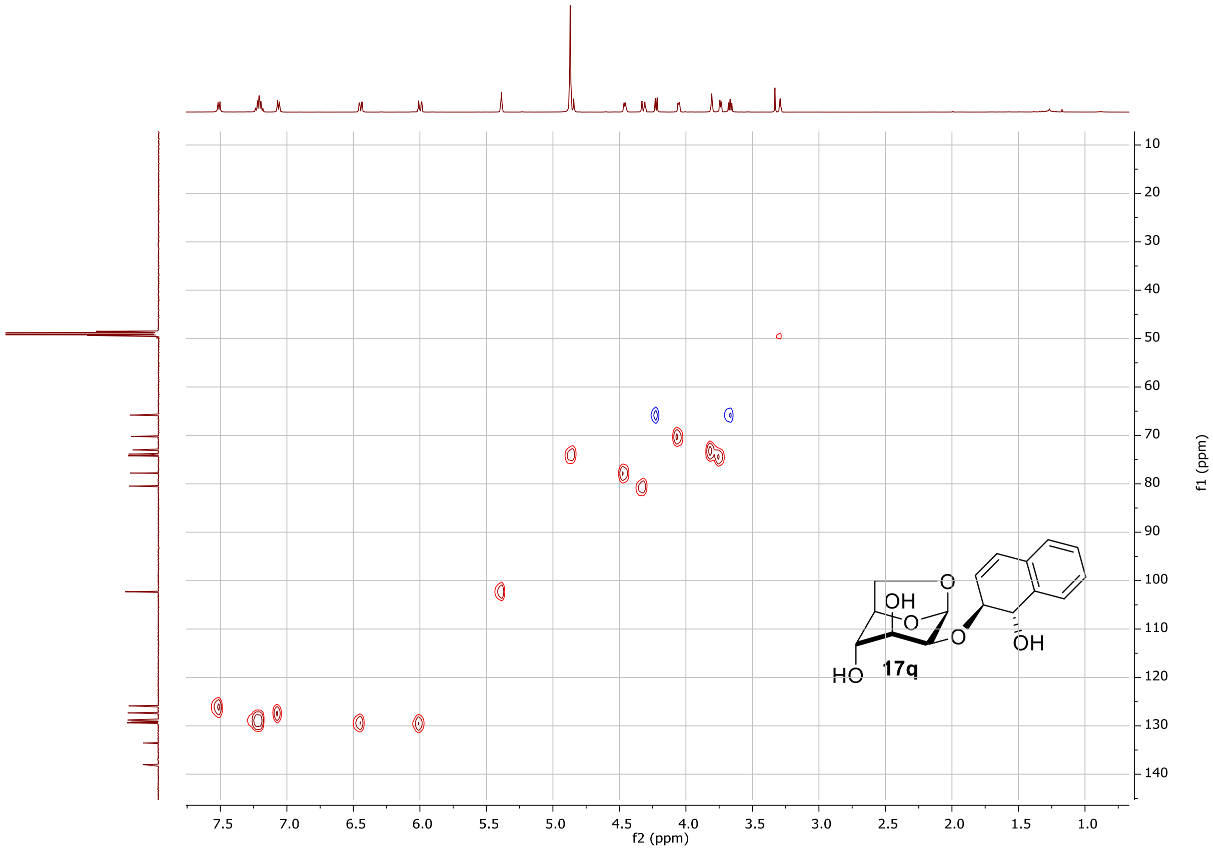

Supplementary Figure 238. HSQC spectra for **17q**

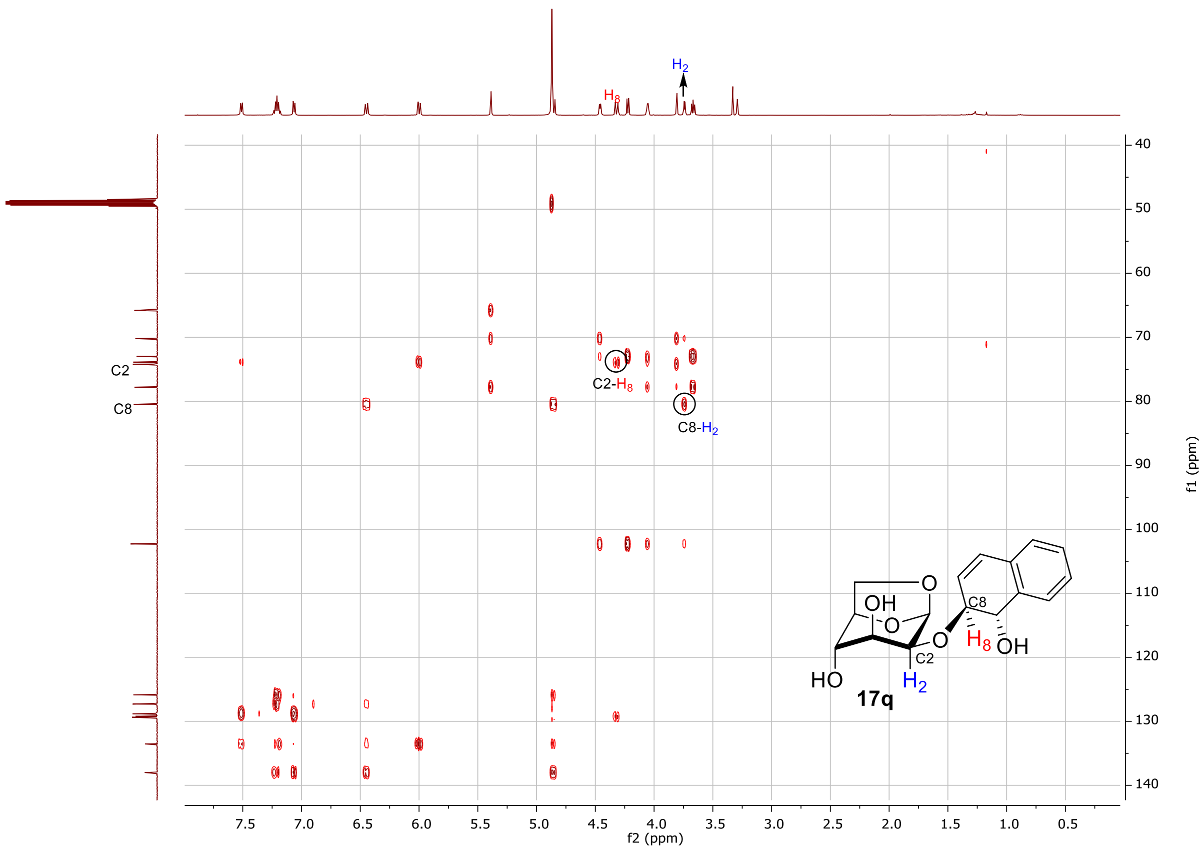

Supplementary Figure 239. HMBC spectra for **17q**

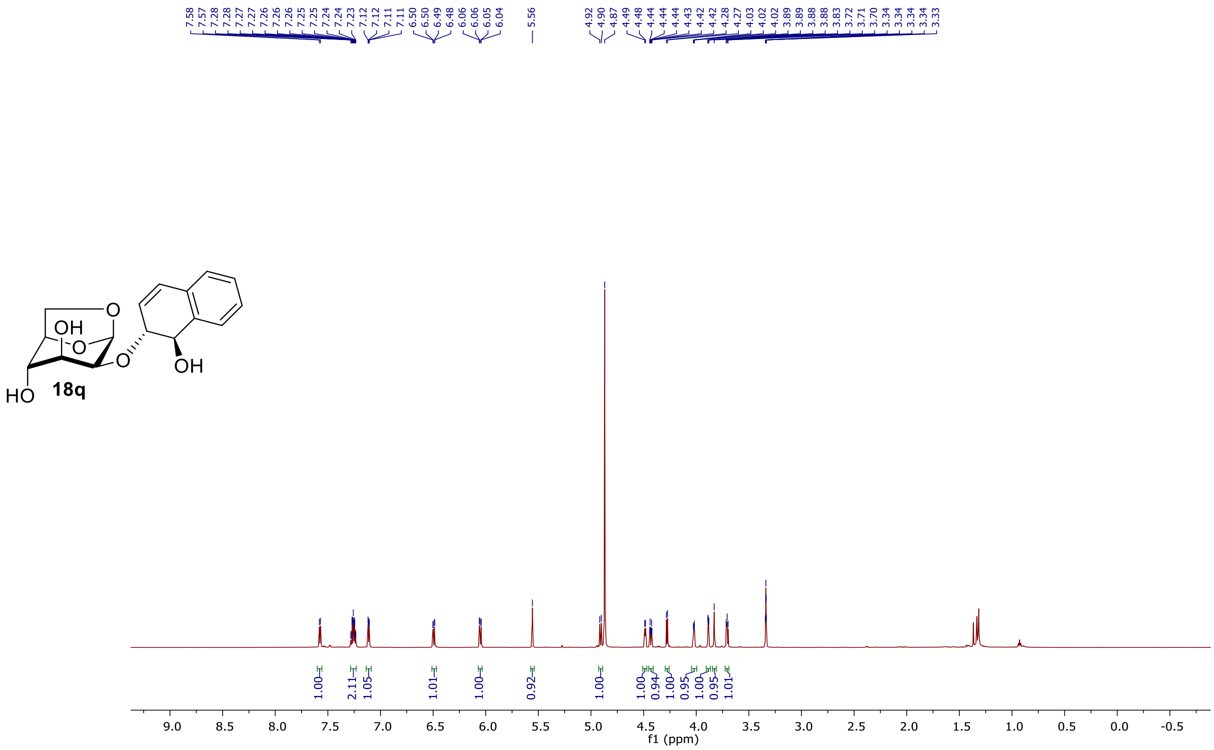

Supplementary Figure 240. ¹H spectra for **18q**

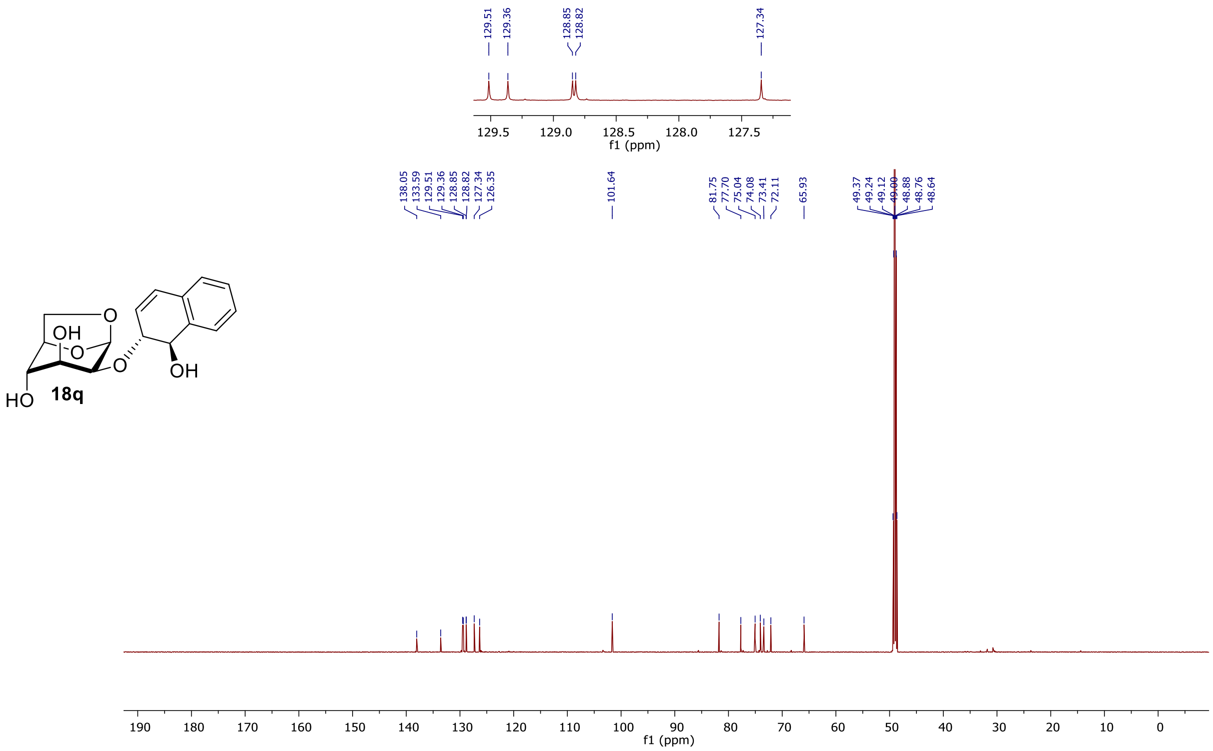

Supplementary Figure 241. ¹³C spectra for **18q**

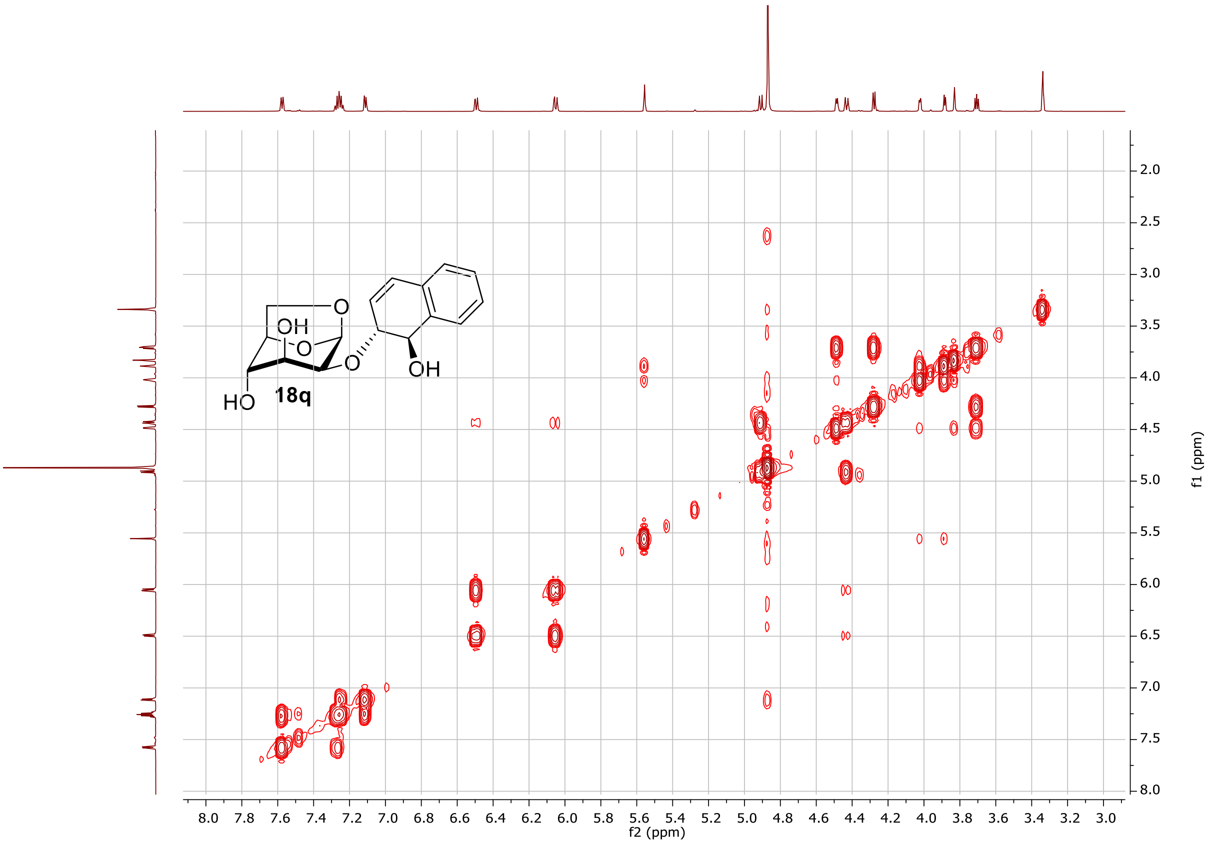

Supplementary Figure 242. COSY spectra for **18q**

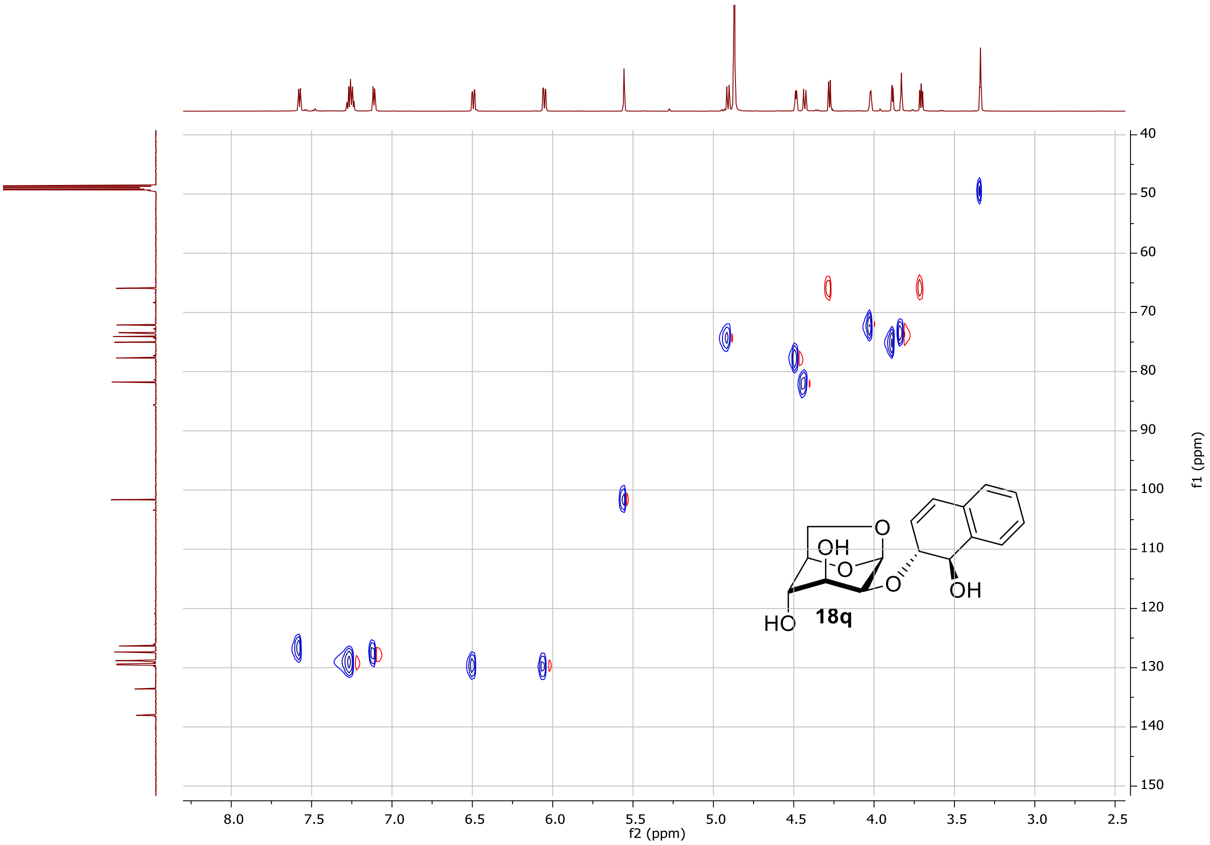

Supplementary Figure 243. HSQC spectra for **18q**

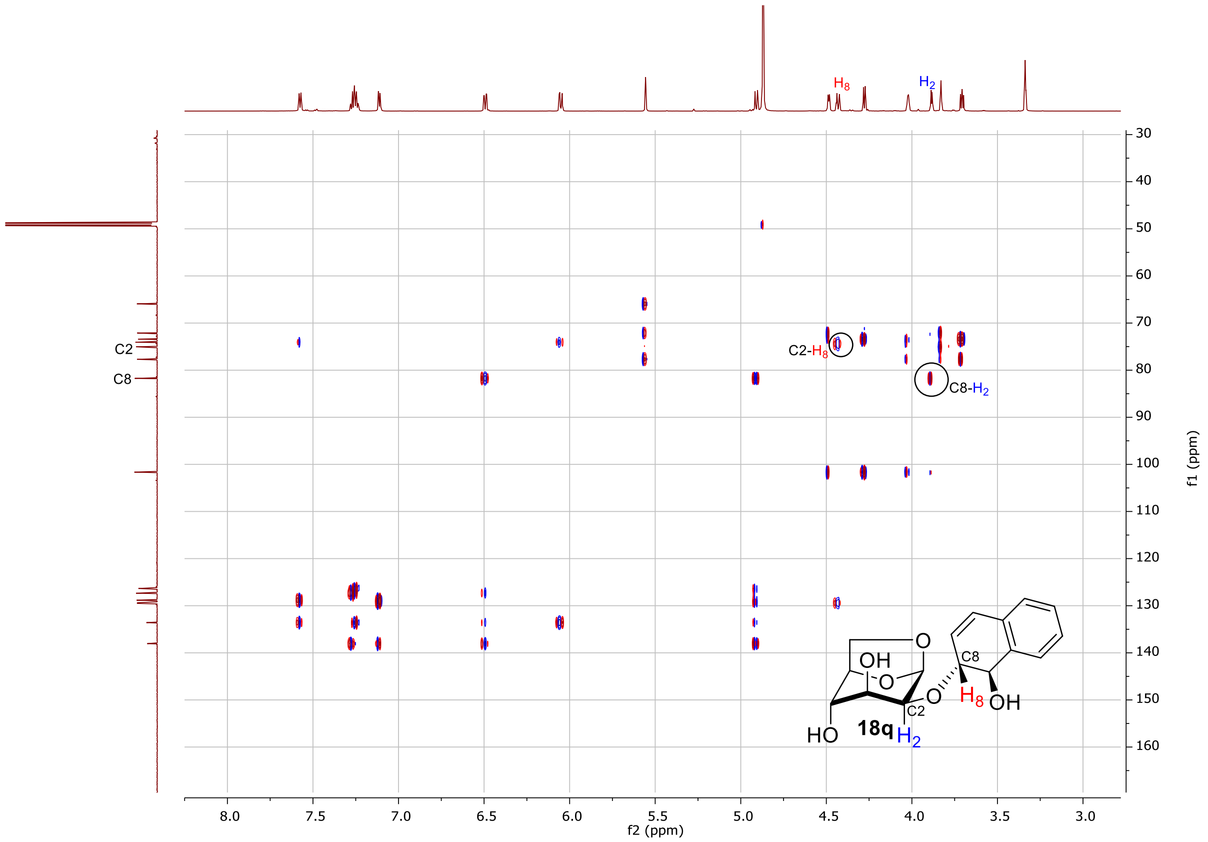

Supplementary Figure 244. HMBC spectra for **18q**

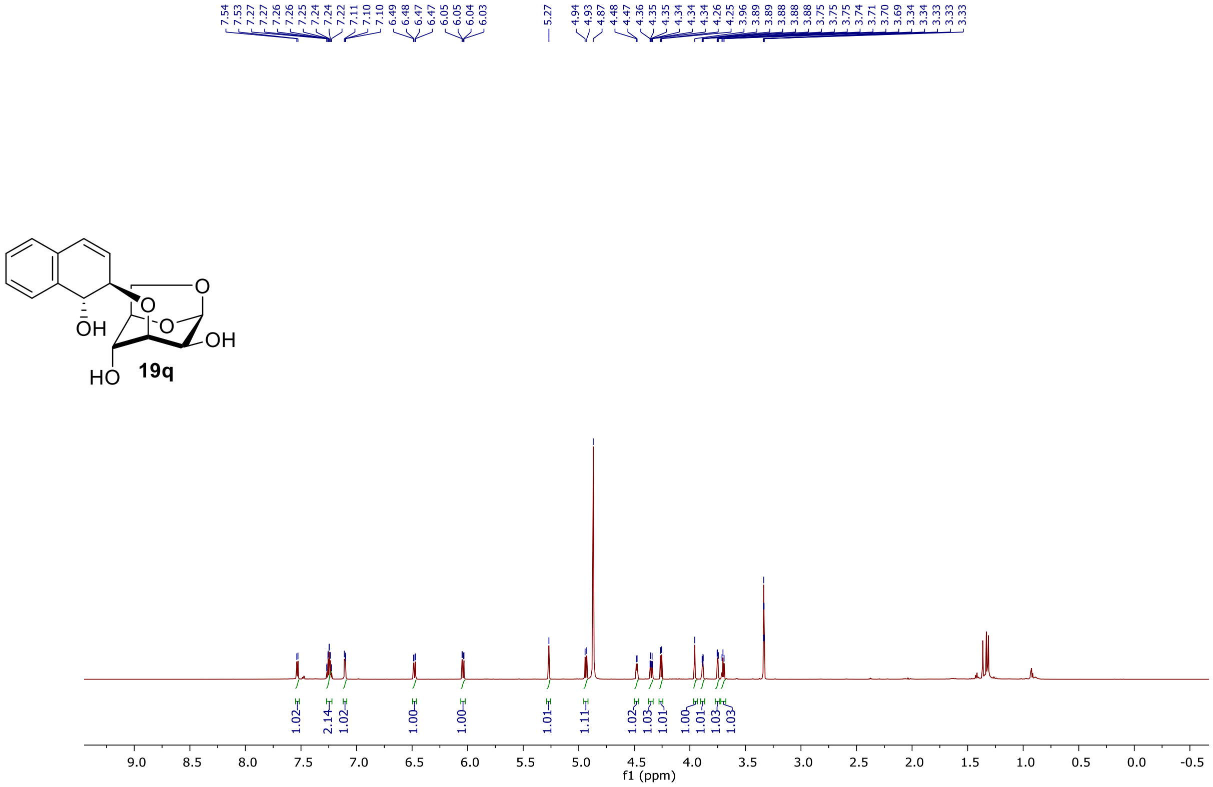

Supplementary Figure 245. ^{1}H spectra for **19q**

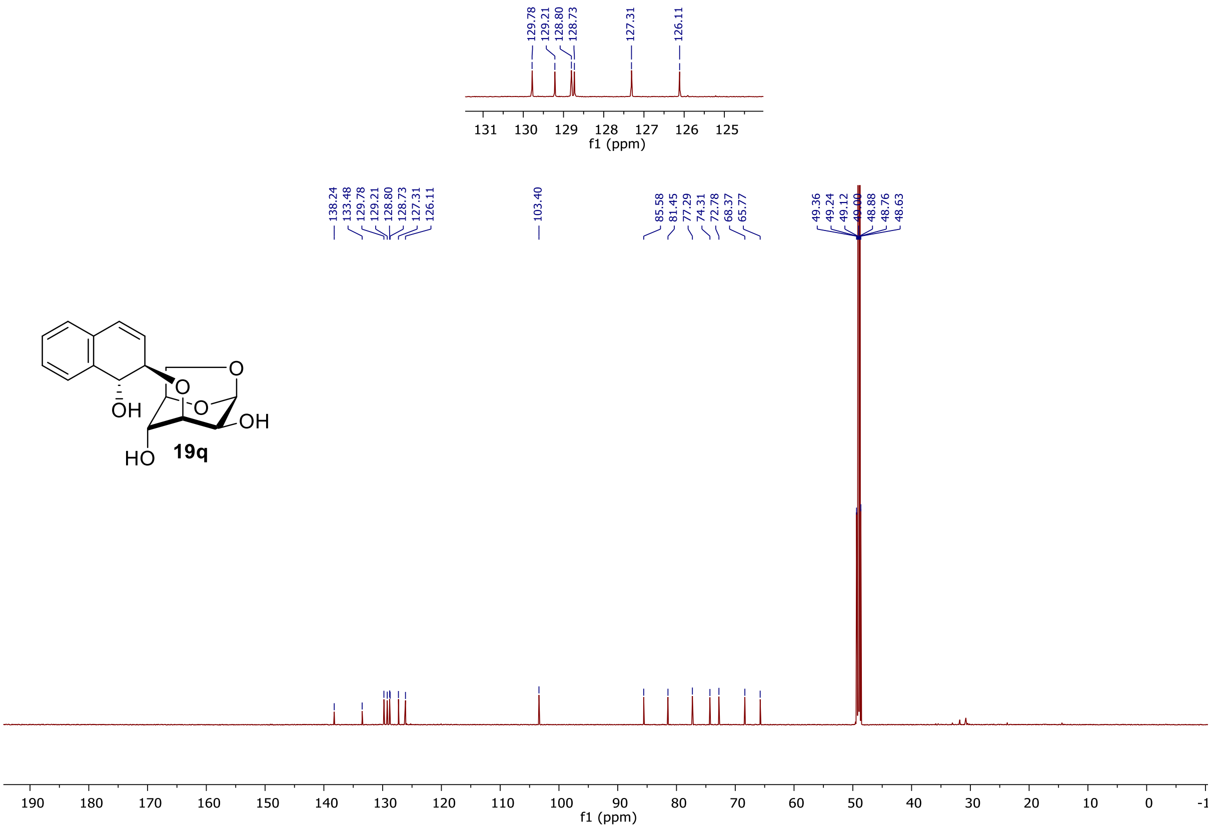

Supplementary Figure 246. ^{13}C spectra for **19q**

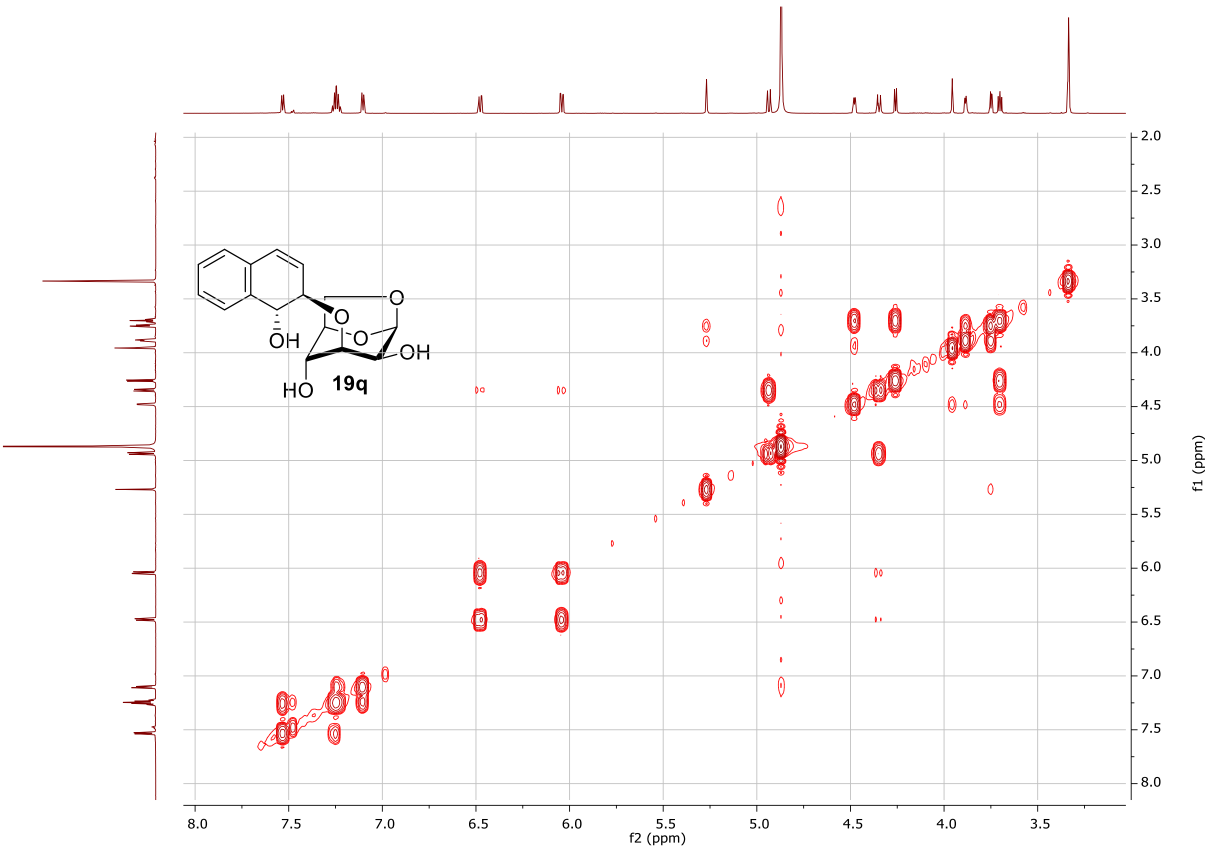

Supplementary Figure 247. COSY spectra for **19q**

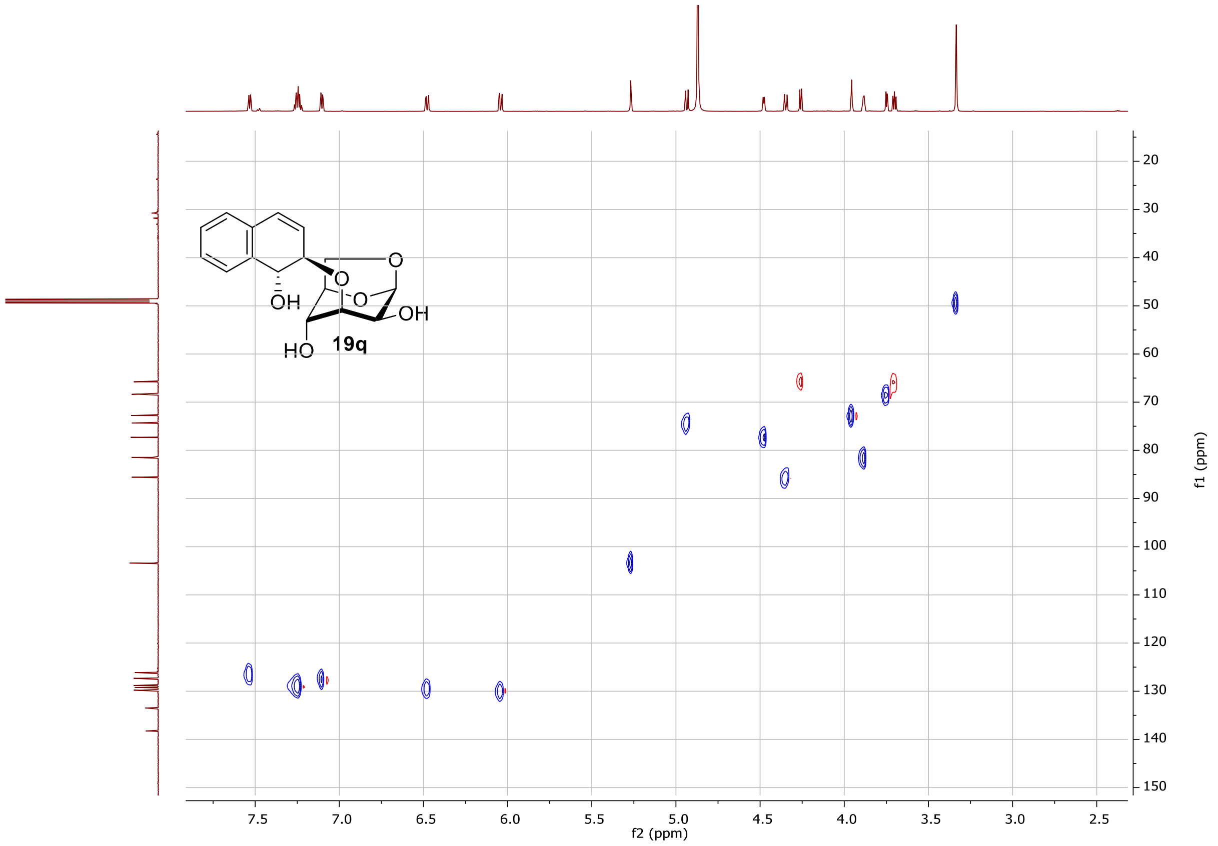

Supplementary Figure 248. HSQC spectra for **19q**

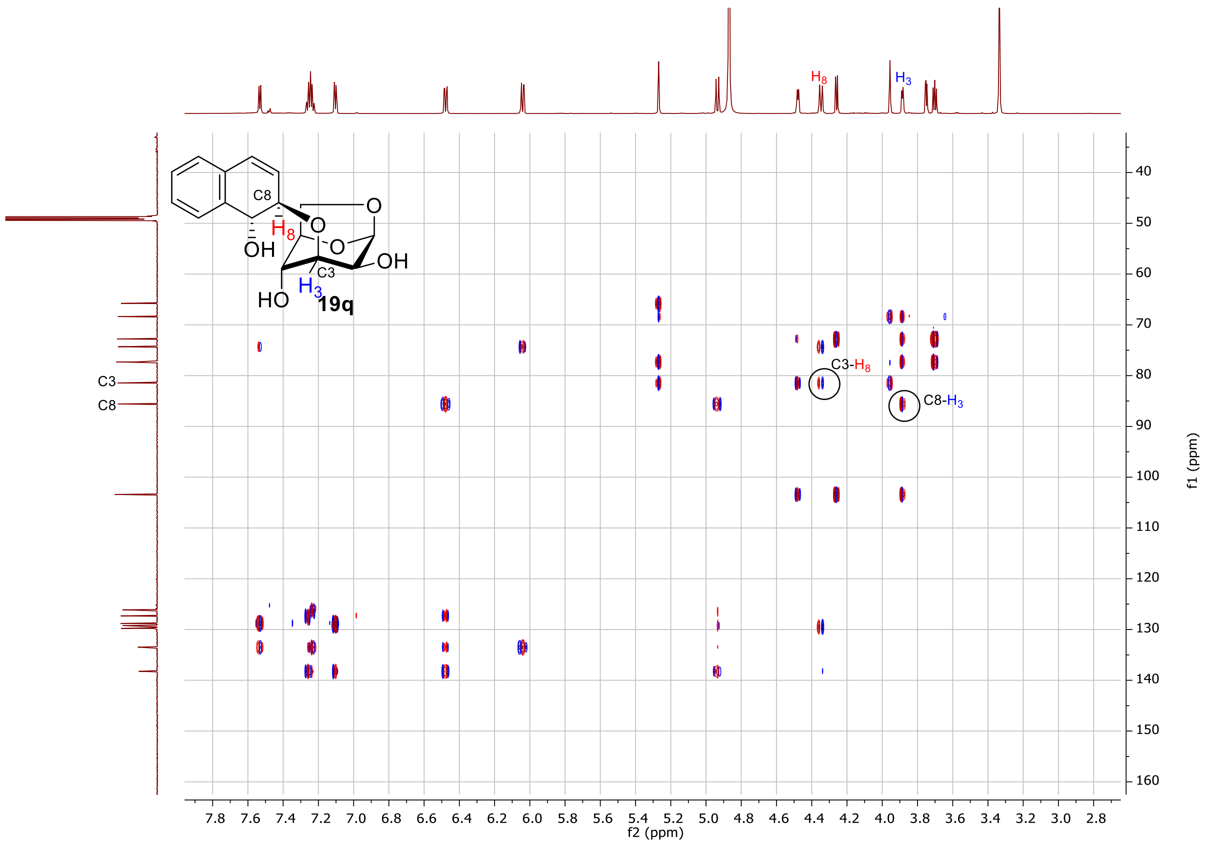

Supplementary Figure 249. HMBC spectra for **19q**

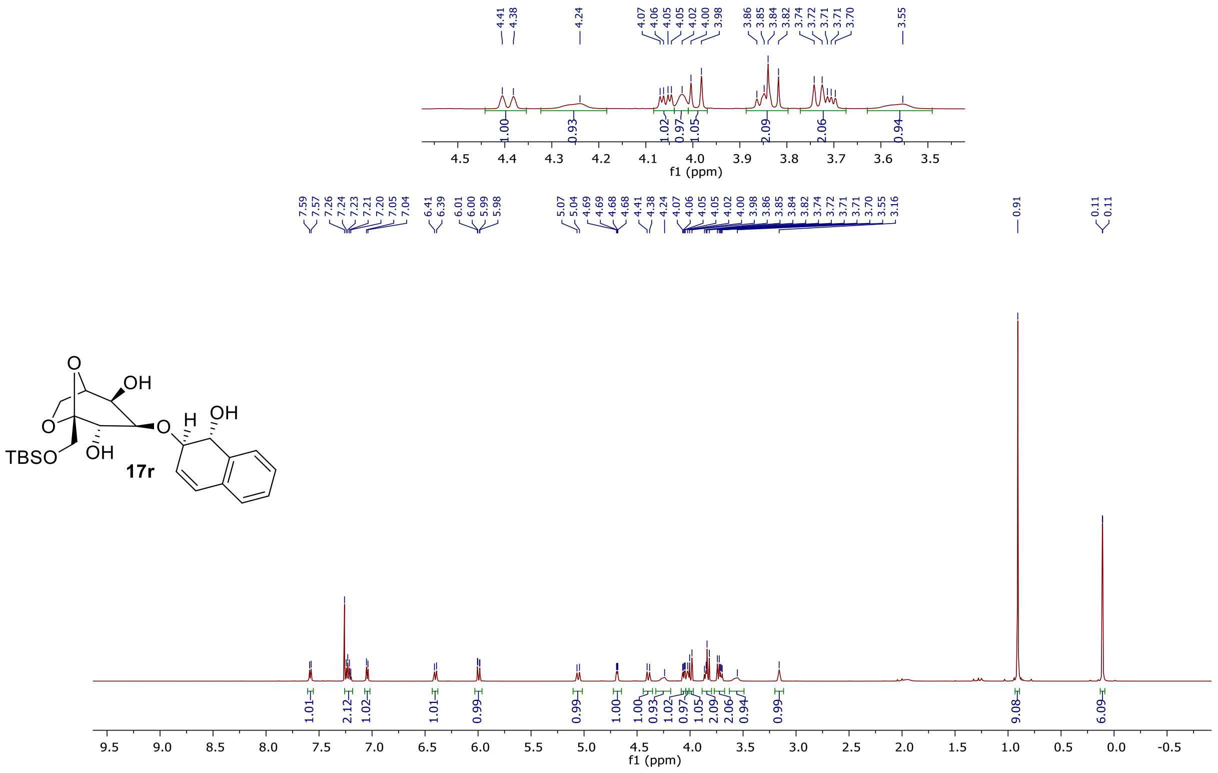

Supplementary Figure 250. ^{1}H spectra for **17r**

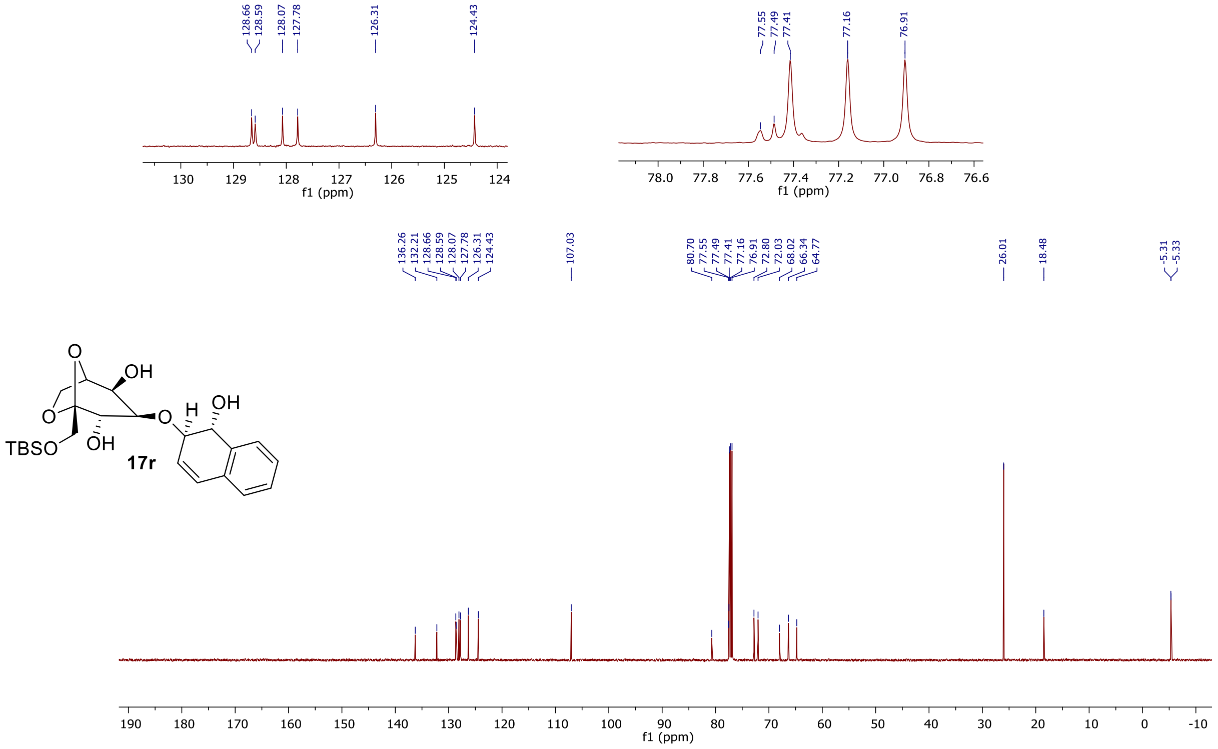

Supplementary Figure 251. ^{13}C spectra for **17r**

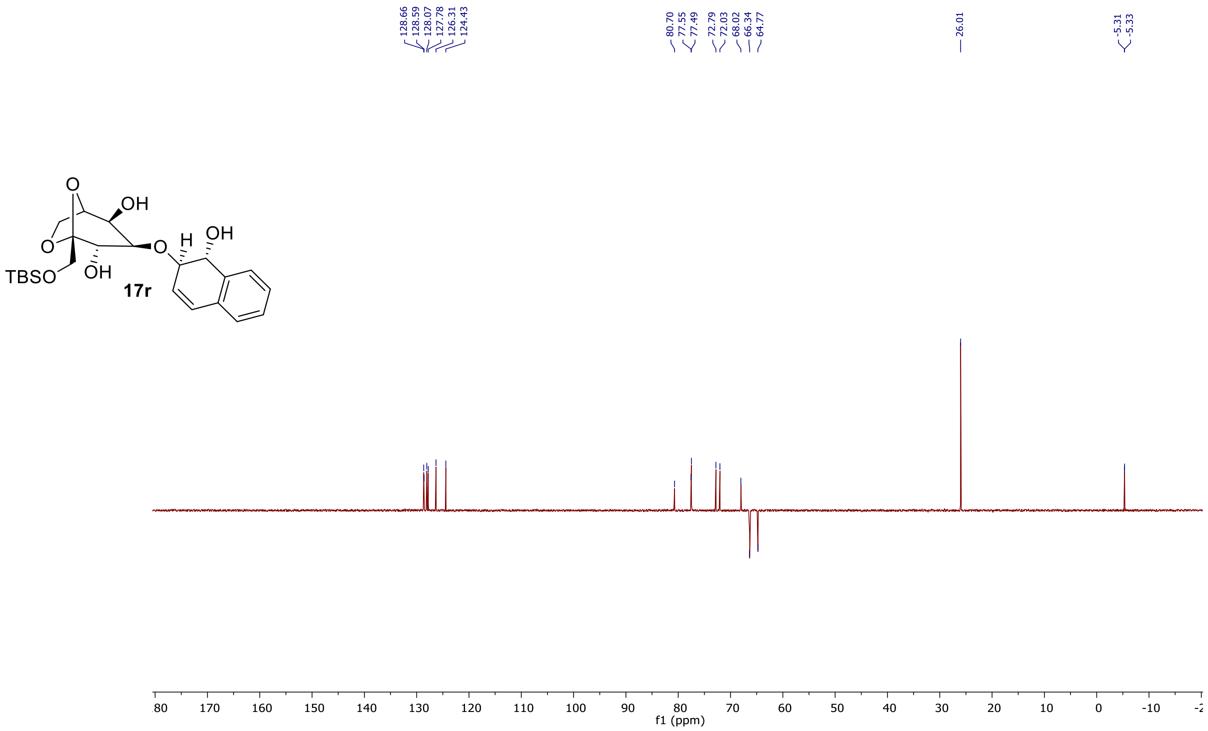

Supplementary Figure 252. DEPT spectra for **17r**

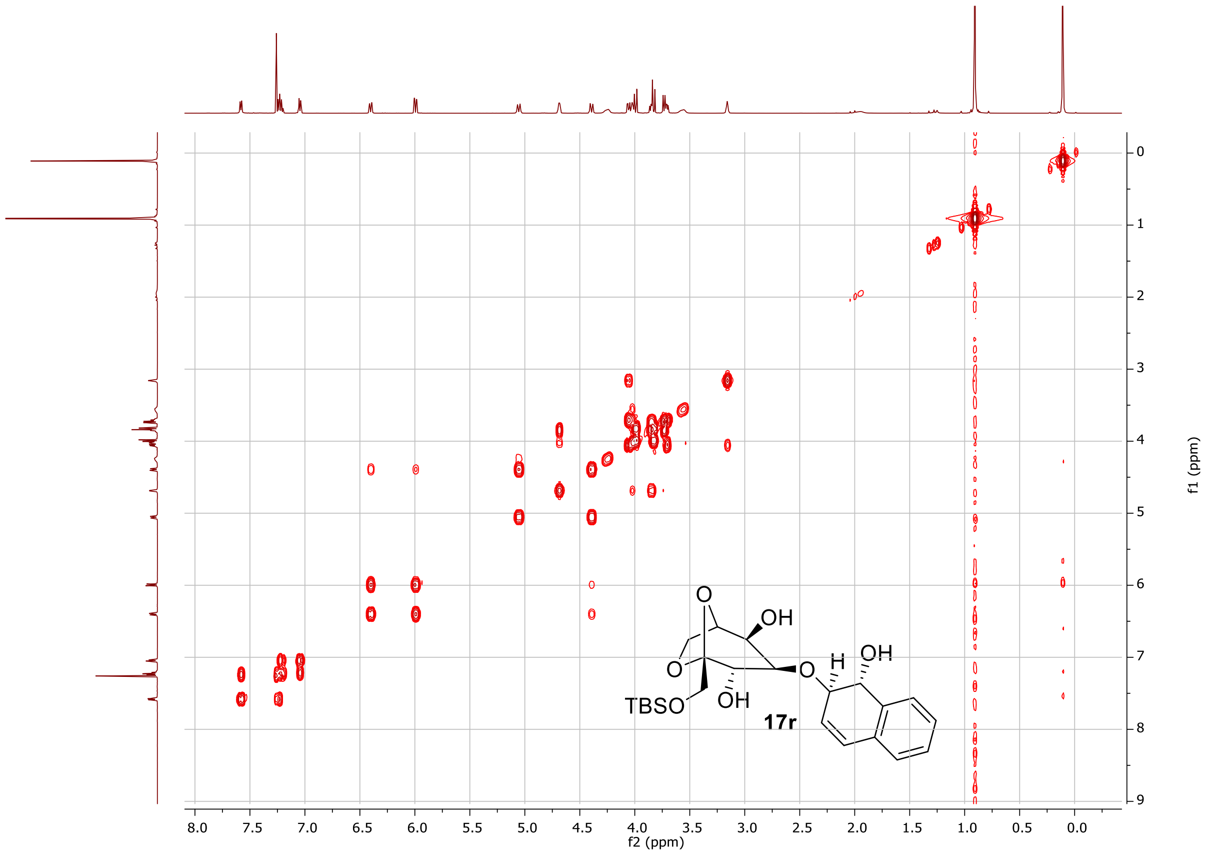

Supplementary Figure 253. COSY spectra for **17r**

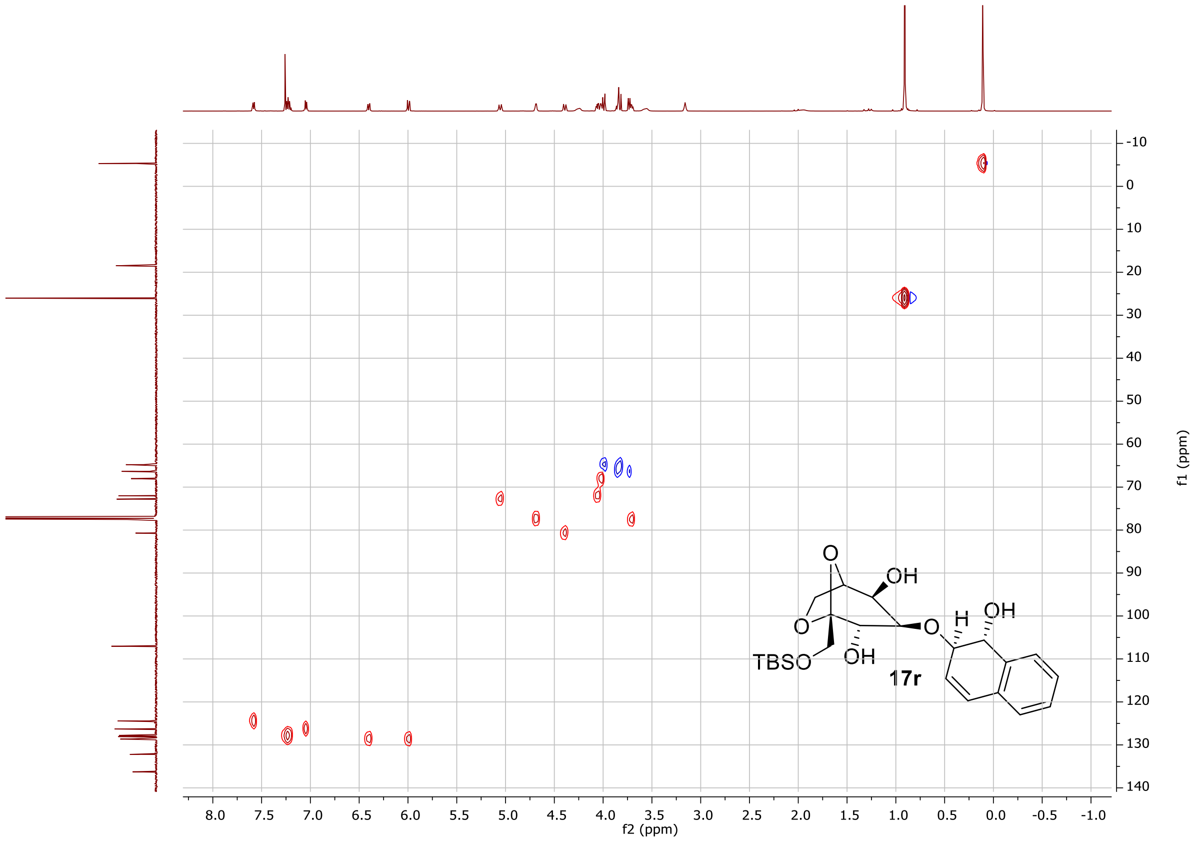

Supplementary Figure 254. HSQC spectra for **17r**

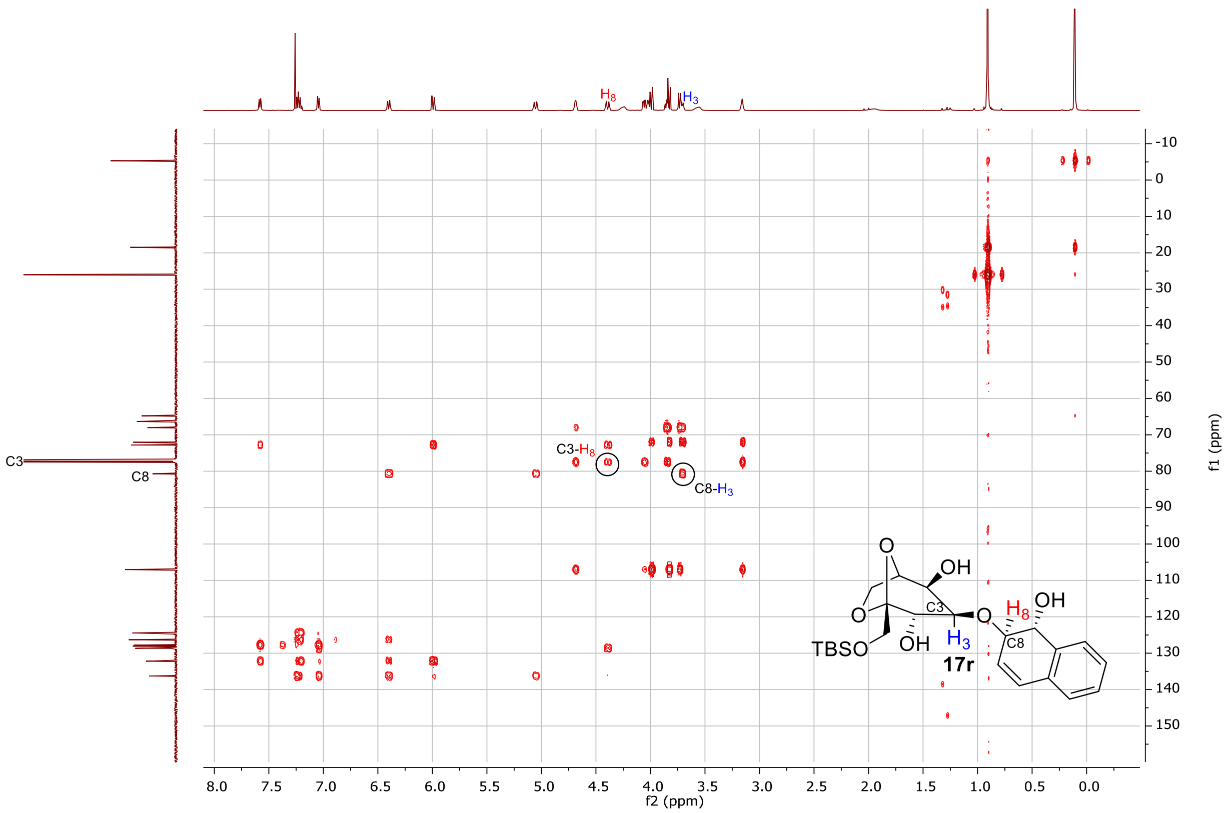

Supplementary Figure 255. HMBC spectra for **17r**

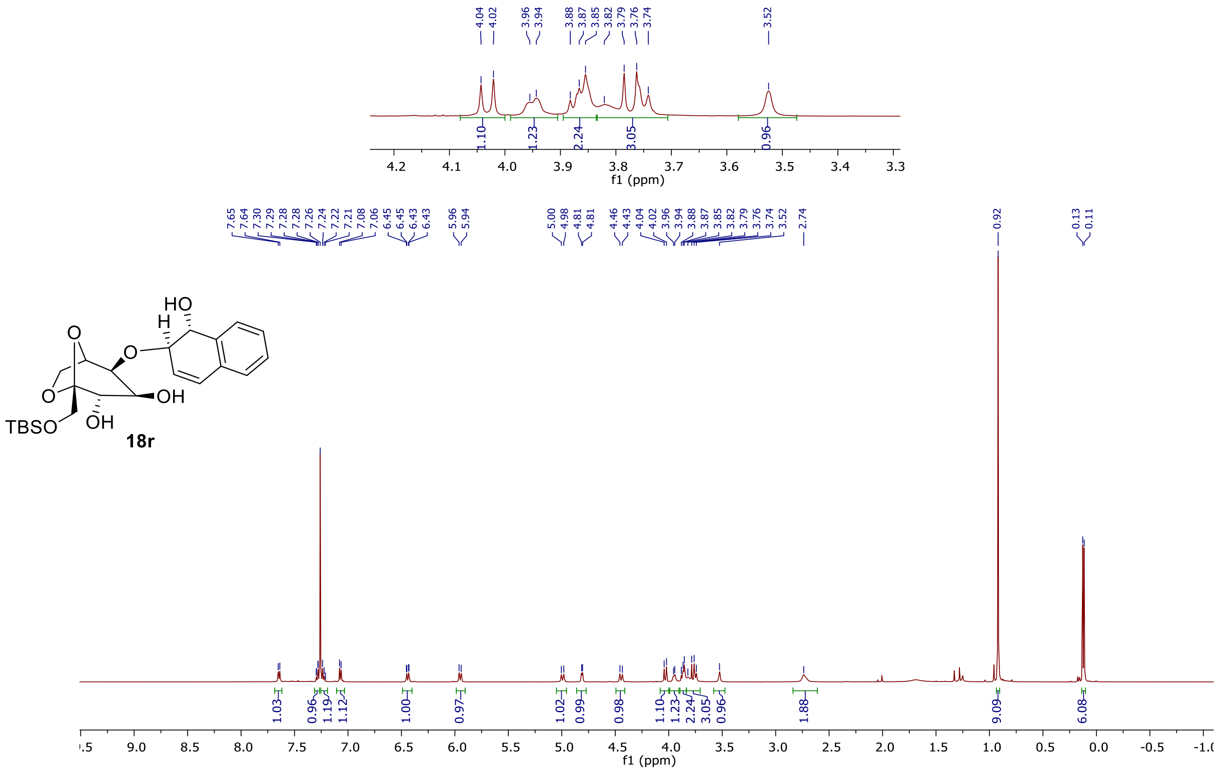

Supplementary Figure 256. ^{1}H spectra for **18r**

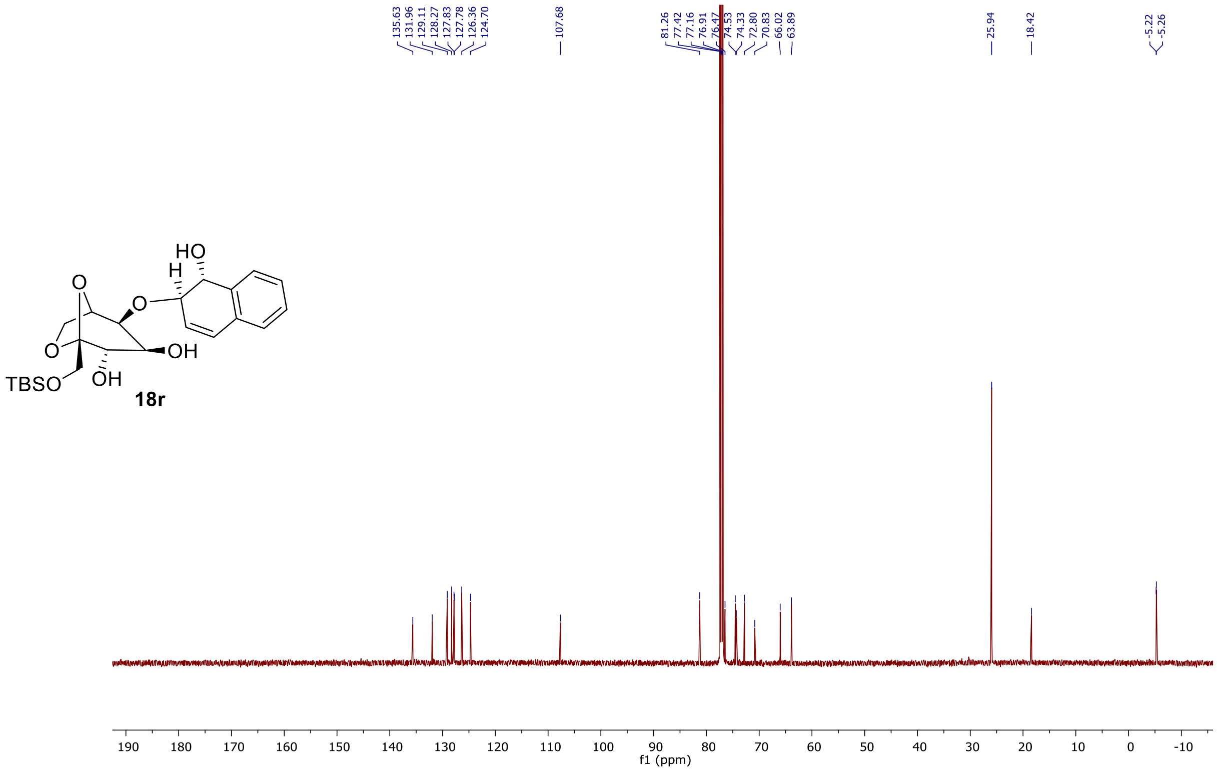

Supplementary Figure 257. ^{13}C spectra for **18r**

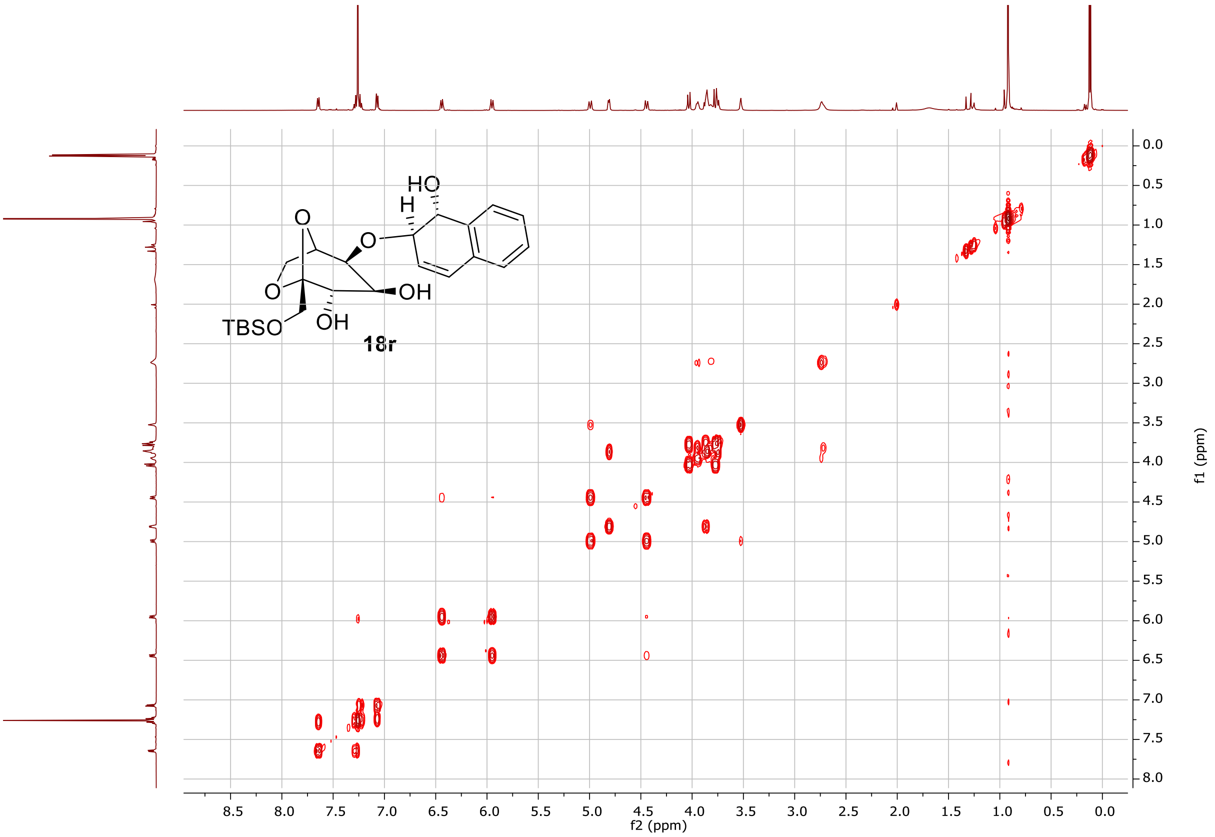

Supplementary Figure 258. COSY spectra for **18r**

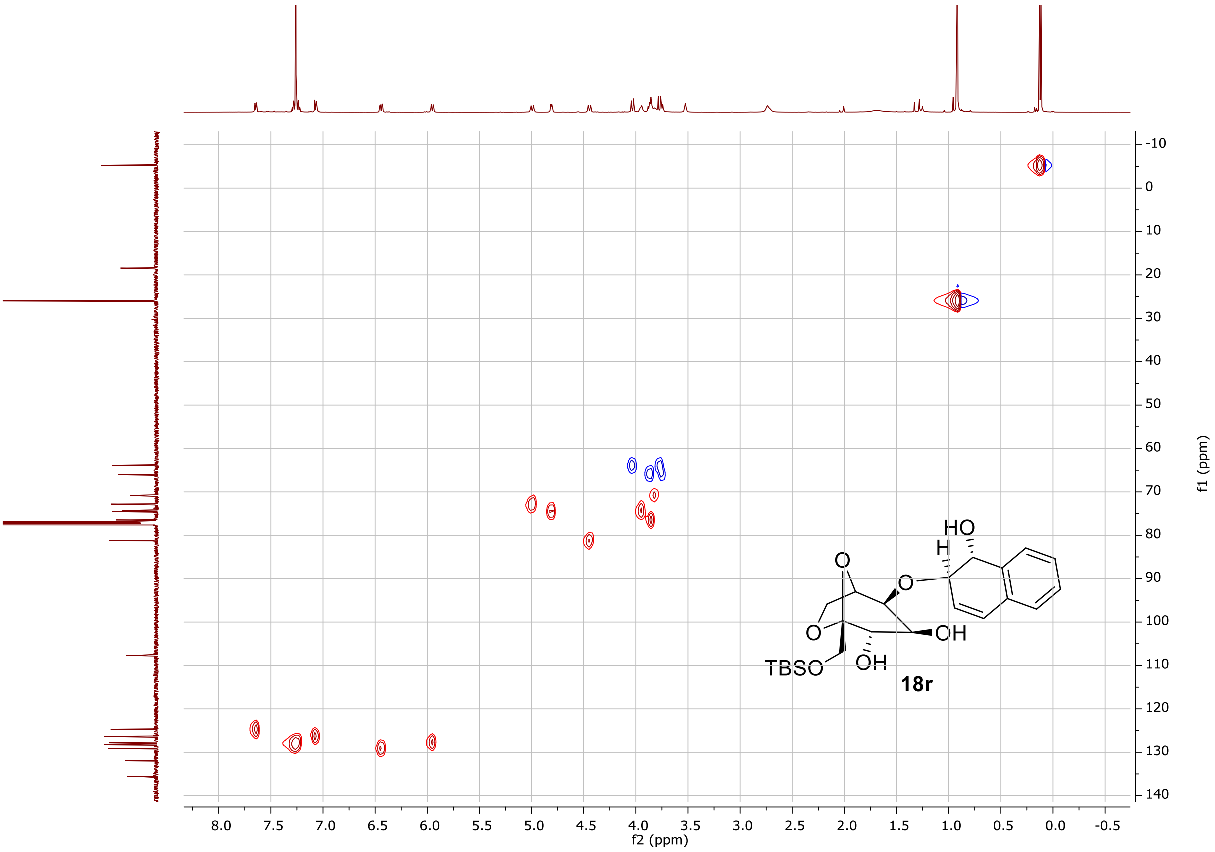

Supplementary Figure 259. HSQC spectra for **18r**

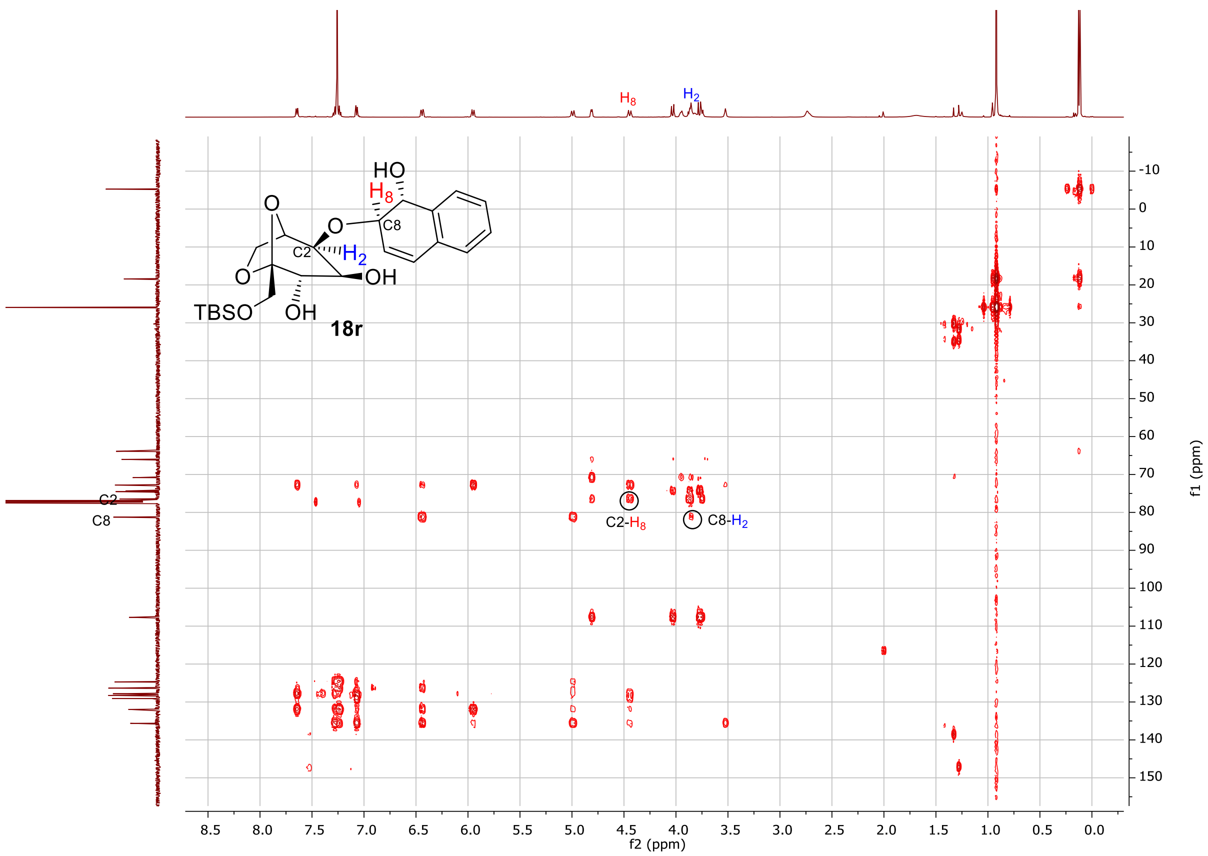

Supplementary Figure 260. HMBC spectra for **18r**

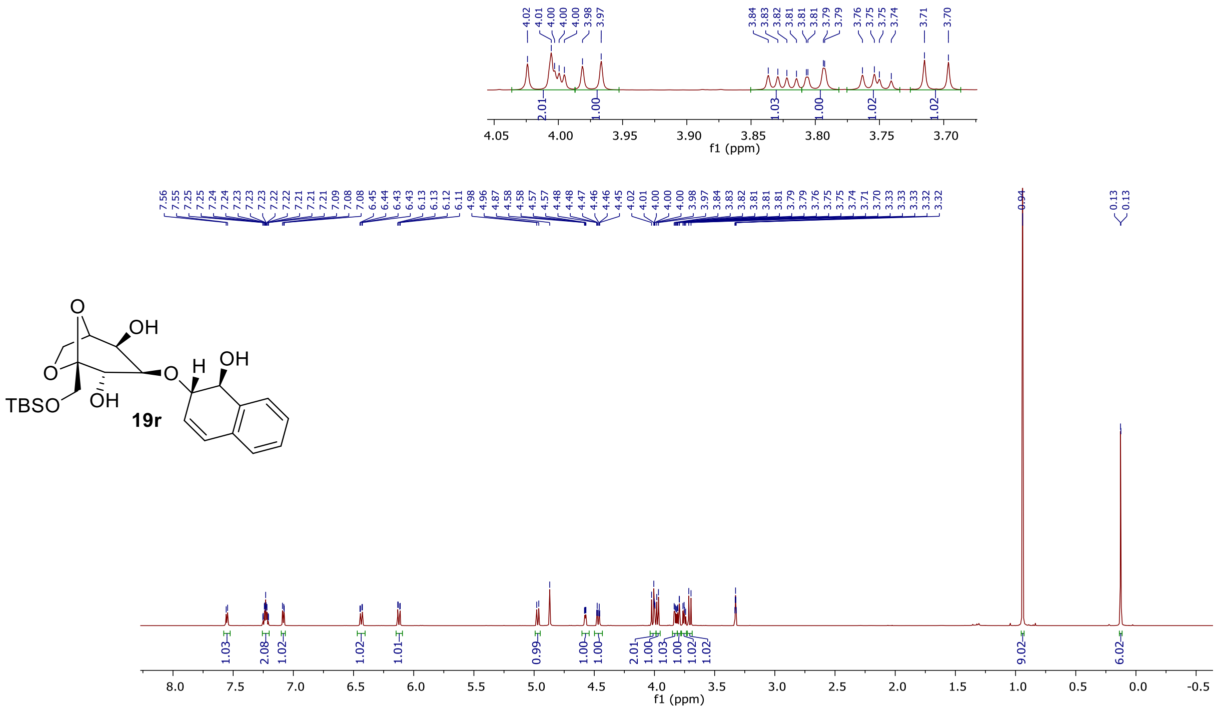

Supplementary Figure 261. ^{1}H spectra for **19r**

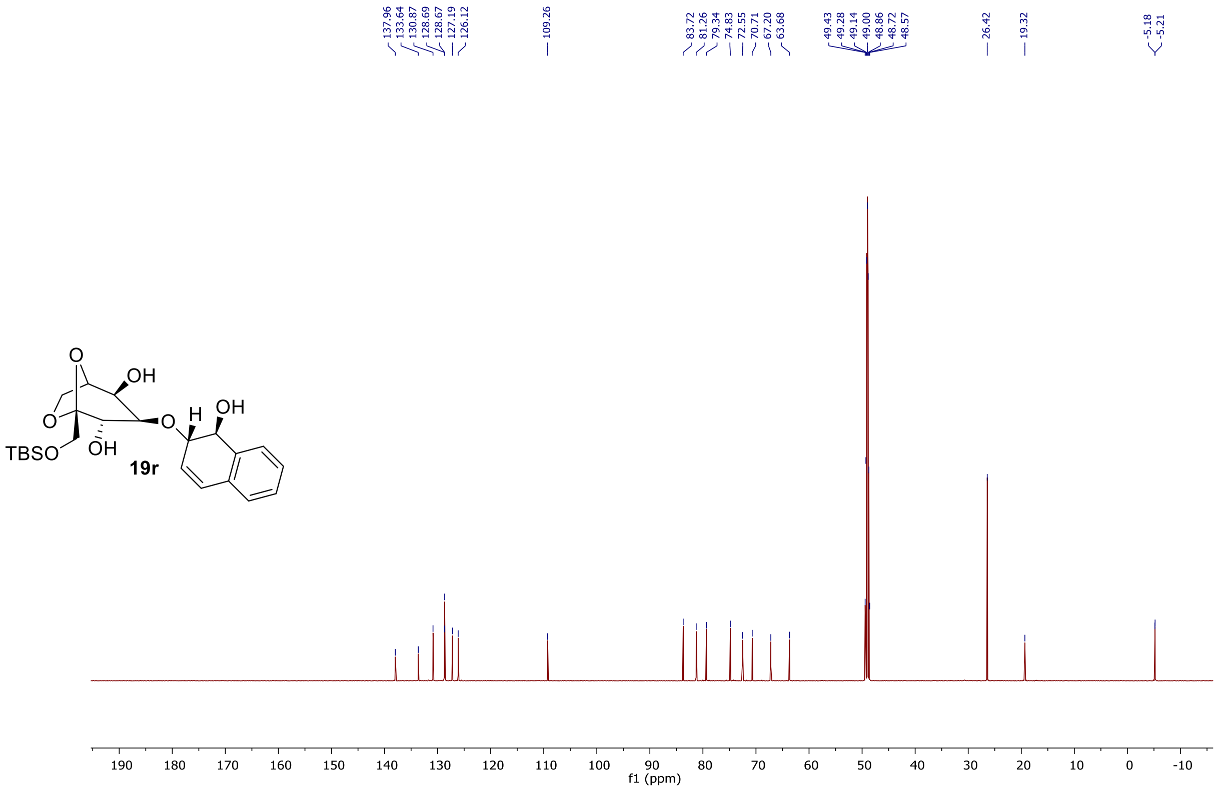

Supplementary Figure 262. ^{13}C spectra for **19r**

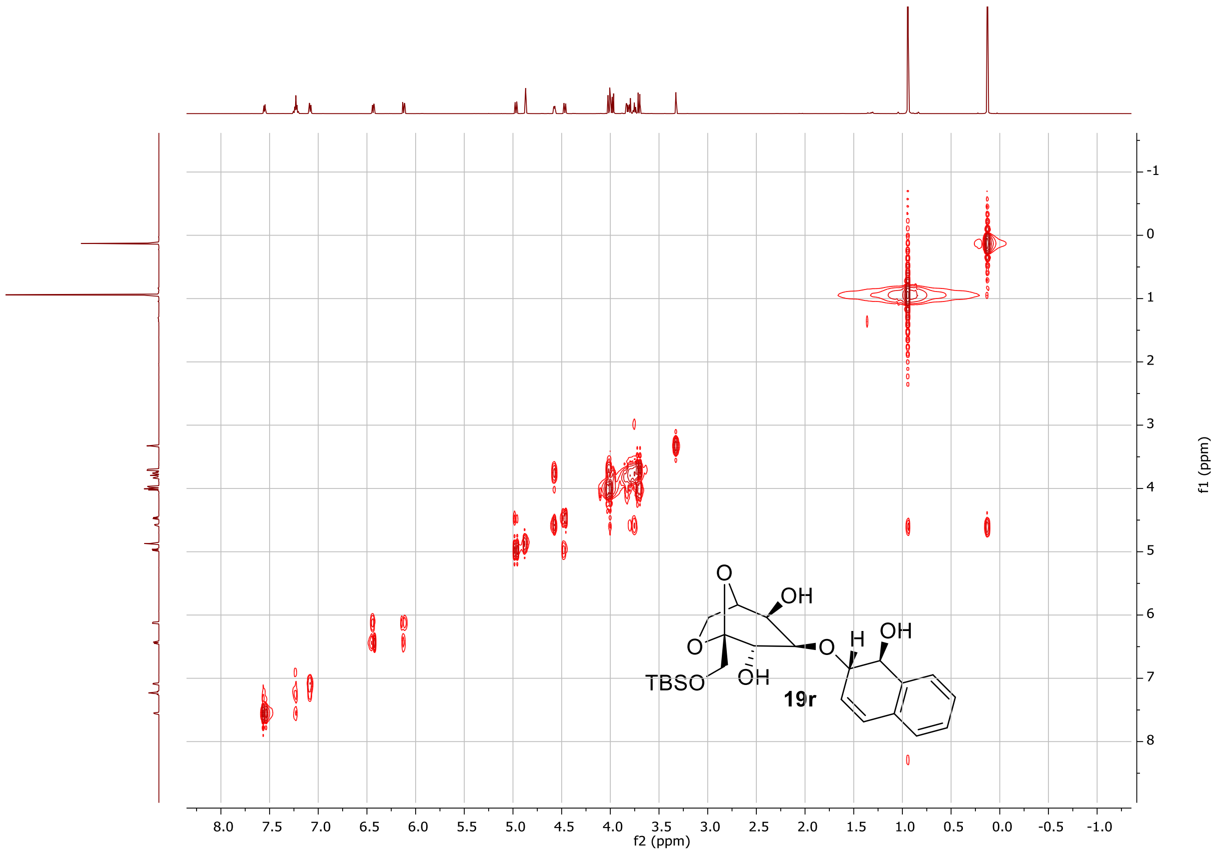

Supplementary Figure S263. COSY spectra for **19r**

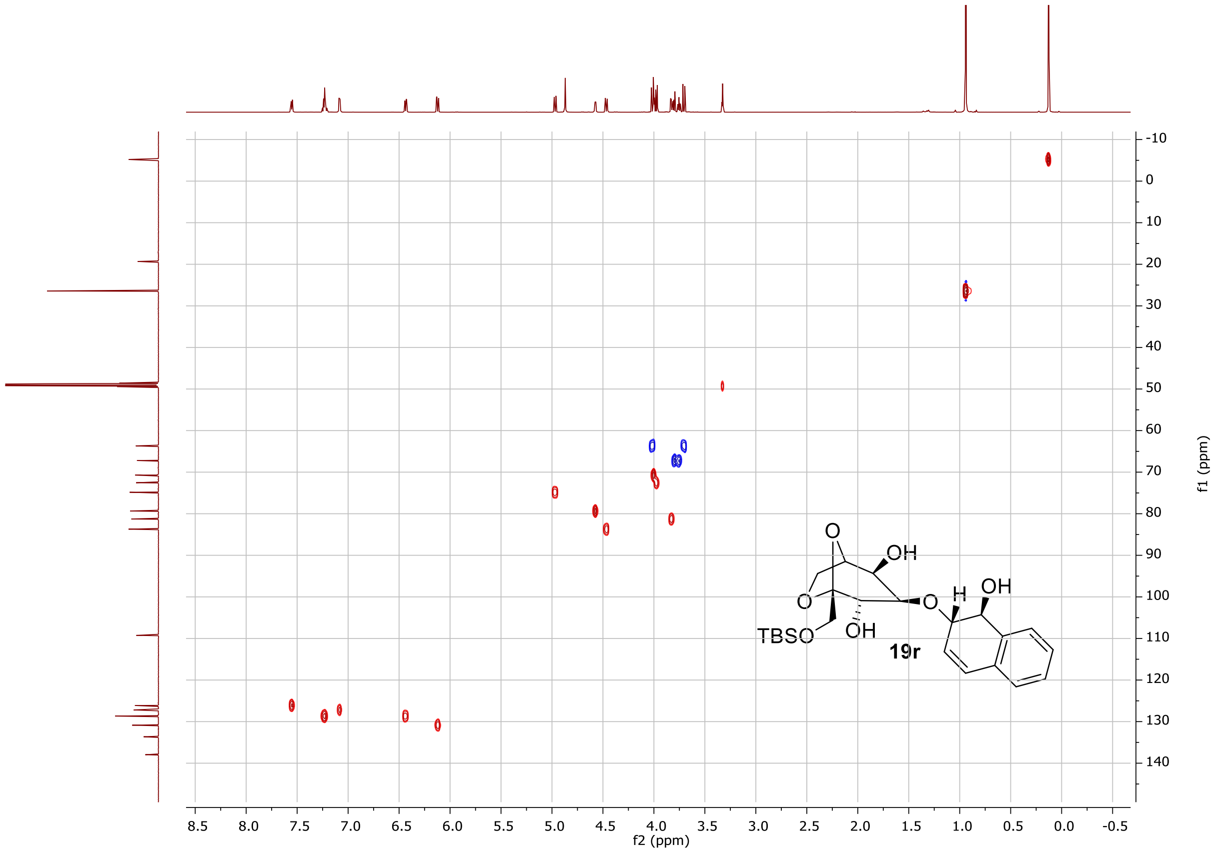

Supplementary Figure 264. HSQC spectra for **19r**

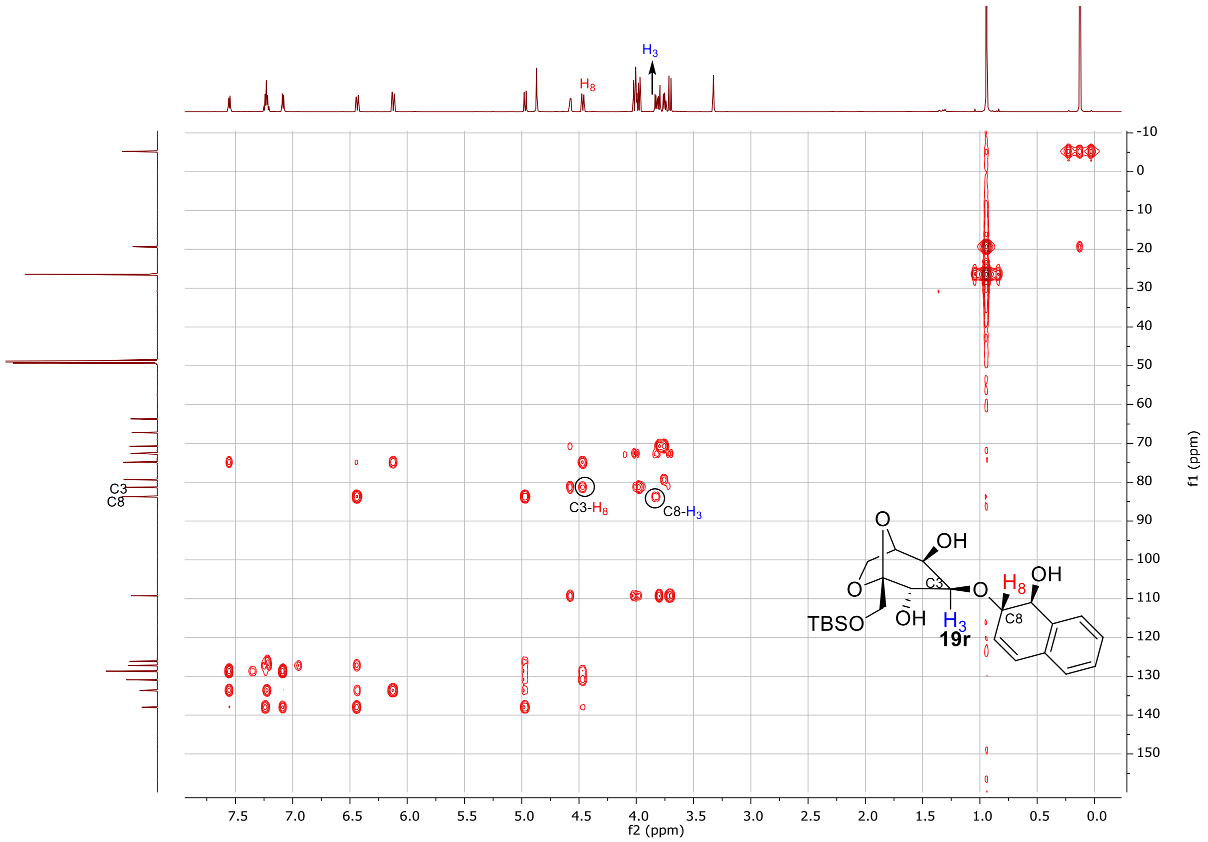

Supplementary Figure 265. HMBC spectra for **19r**

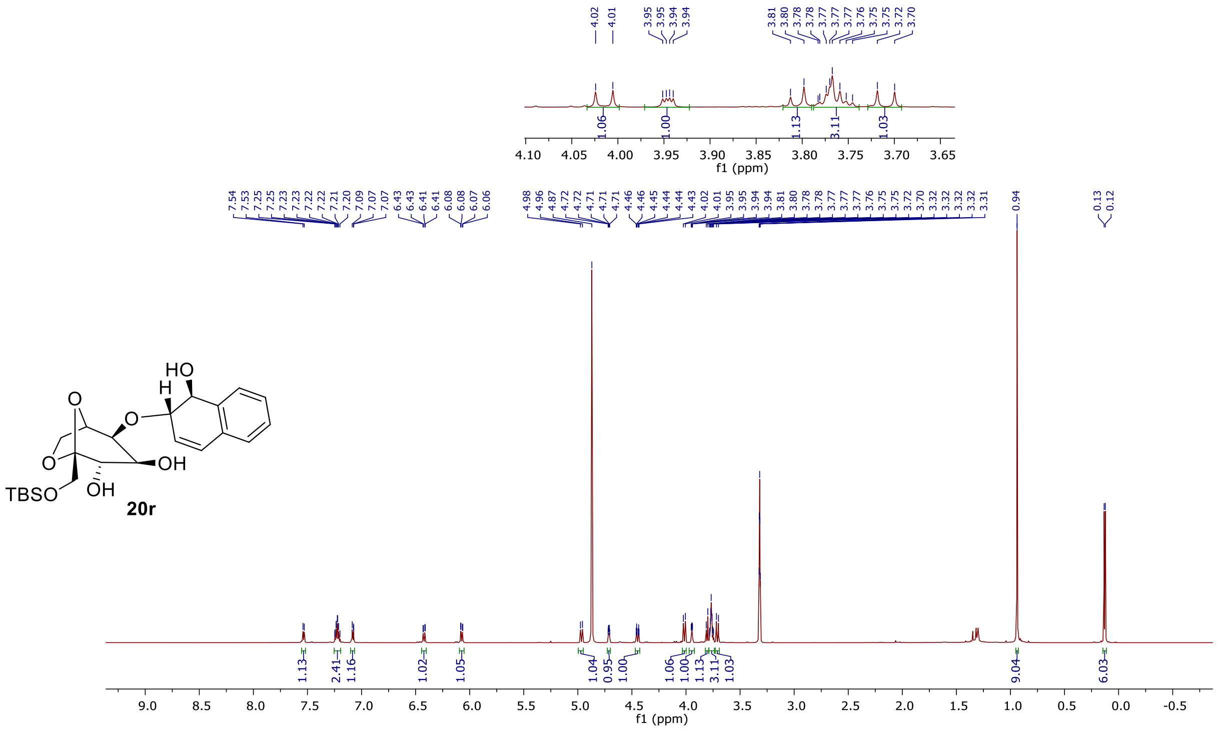

Supplementary Figure 266. ^{1}H spectra for **20r**

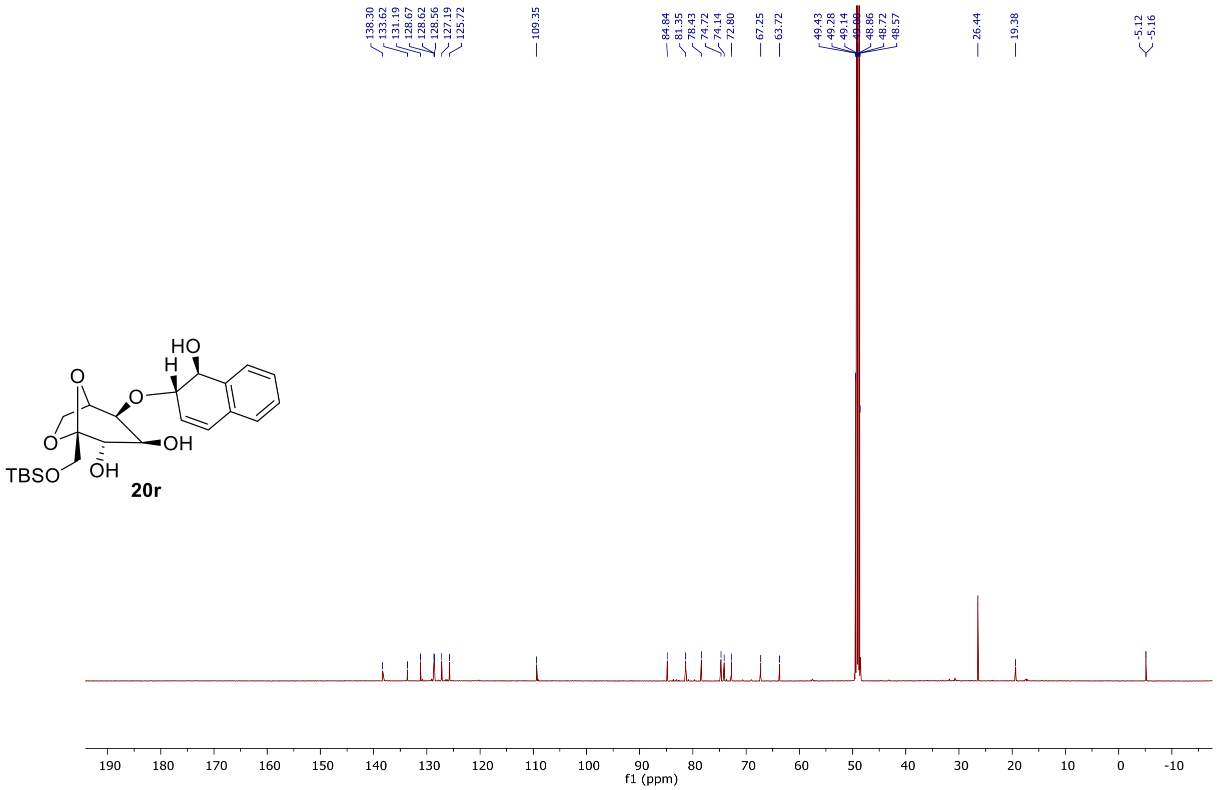

Supplementary Figure 267. ^{13}C spectra for **20r**

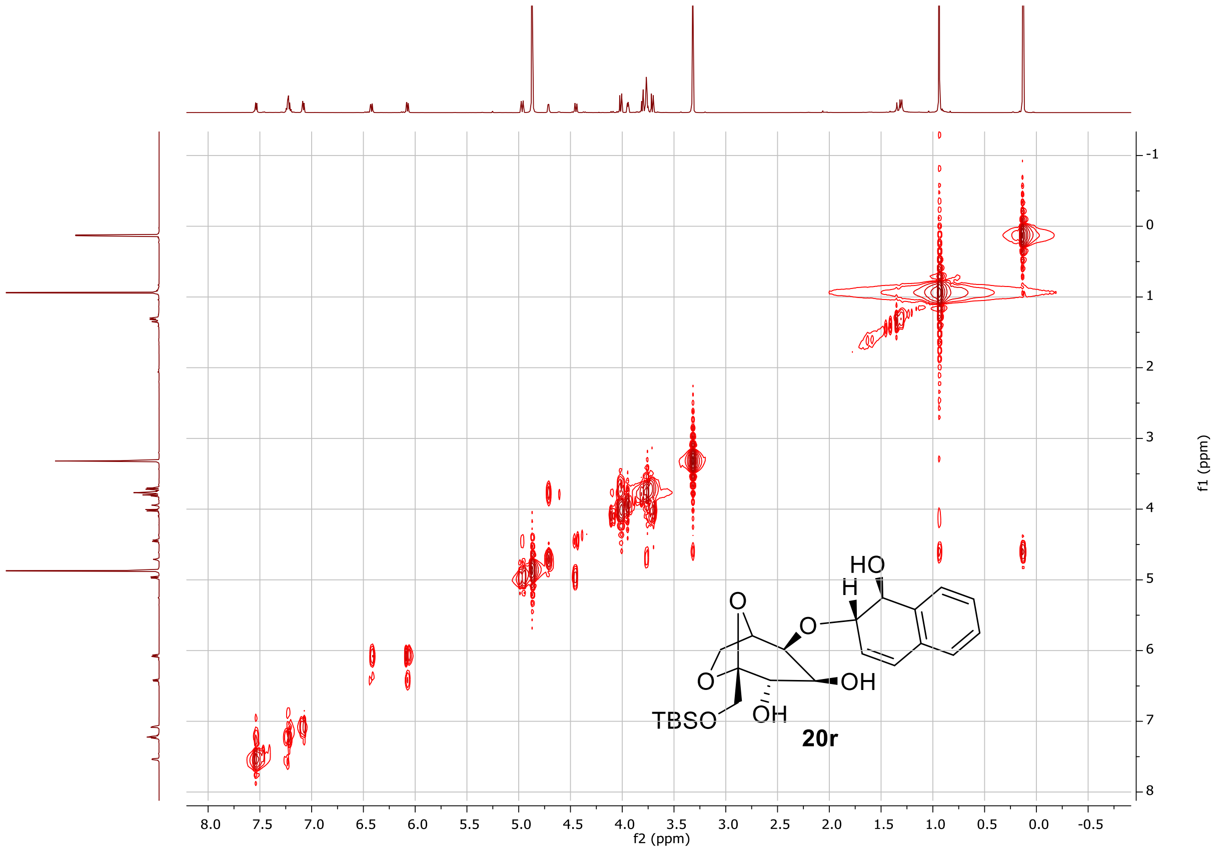

Supplementary Figure 268. COSY spectra for **20r**

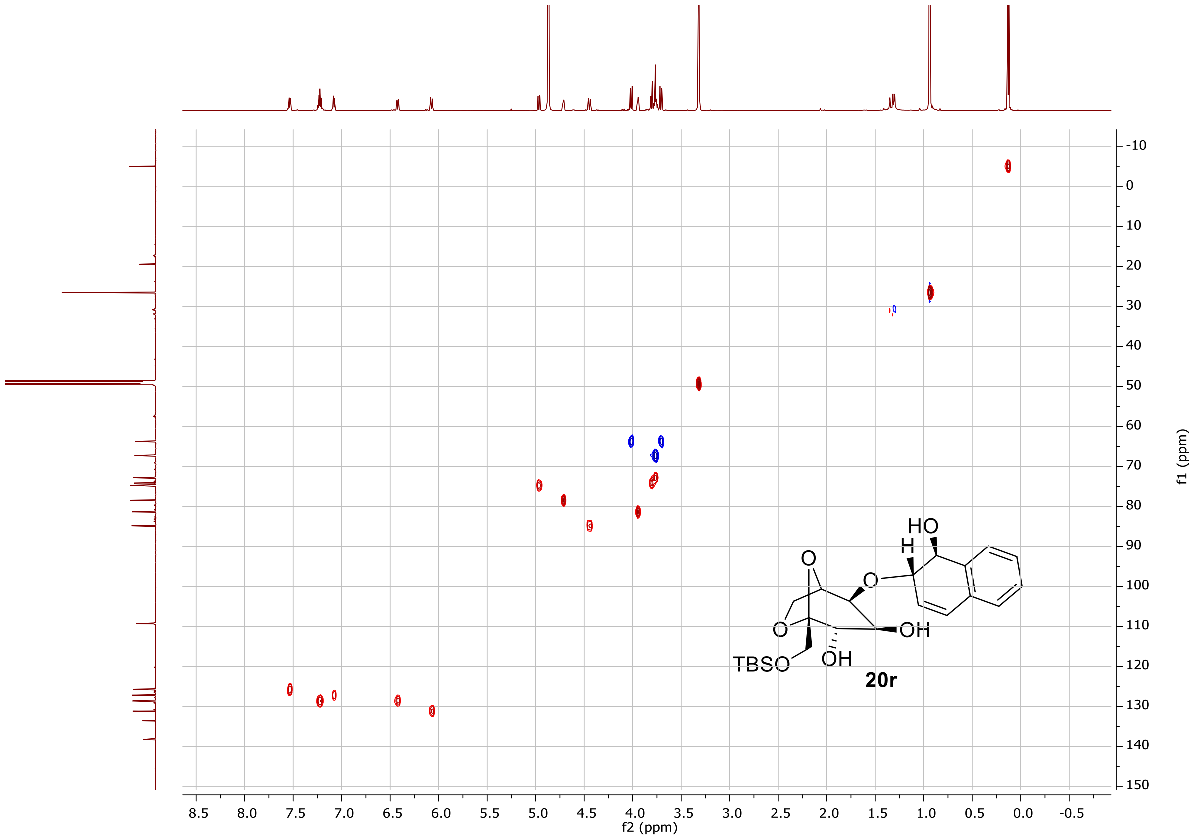

Supplementary Figure 269. HSQC spectra for **20r**

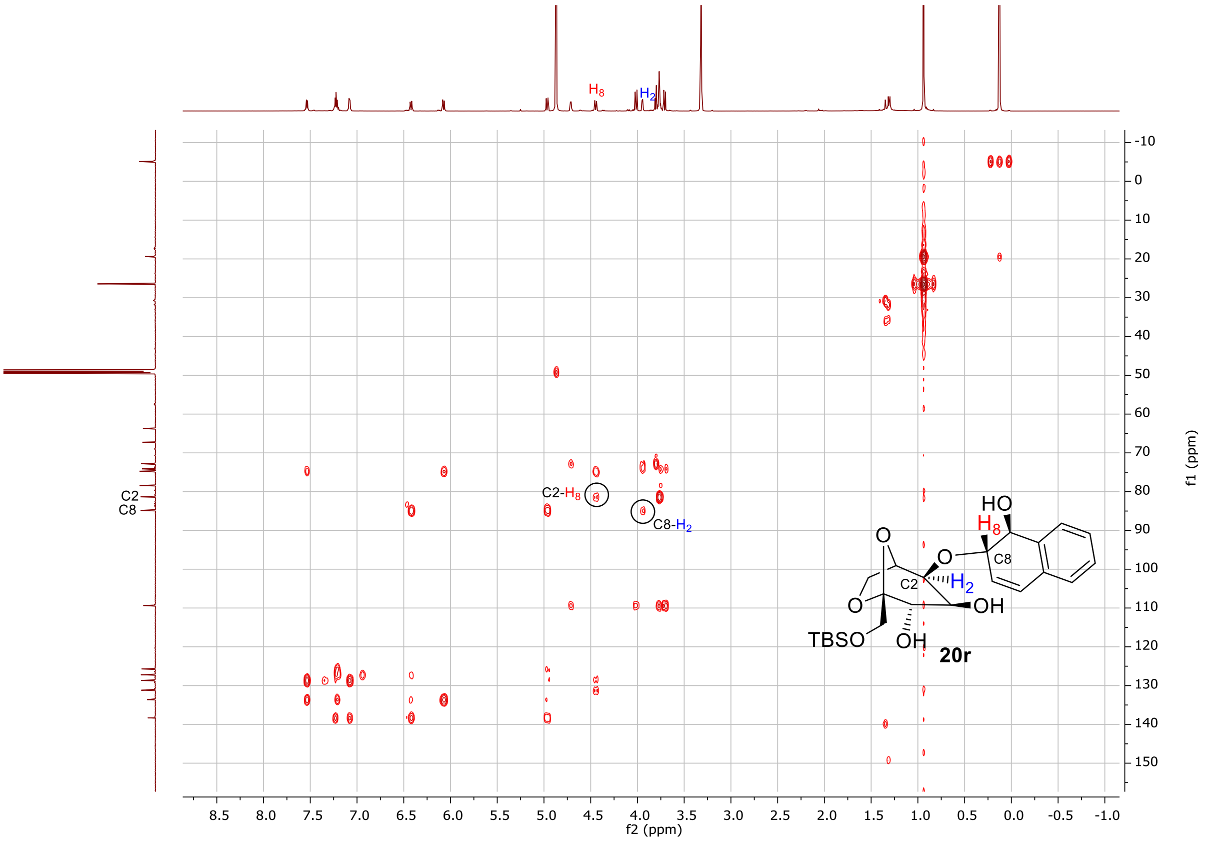

Supplementary Figure 270. HMBC spectra for **20r**

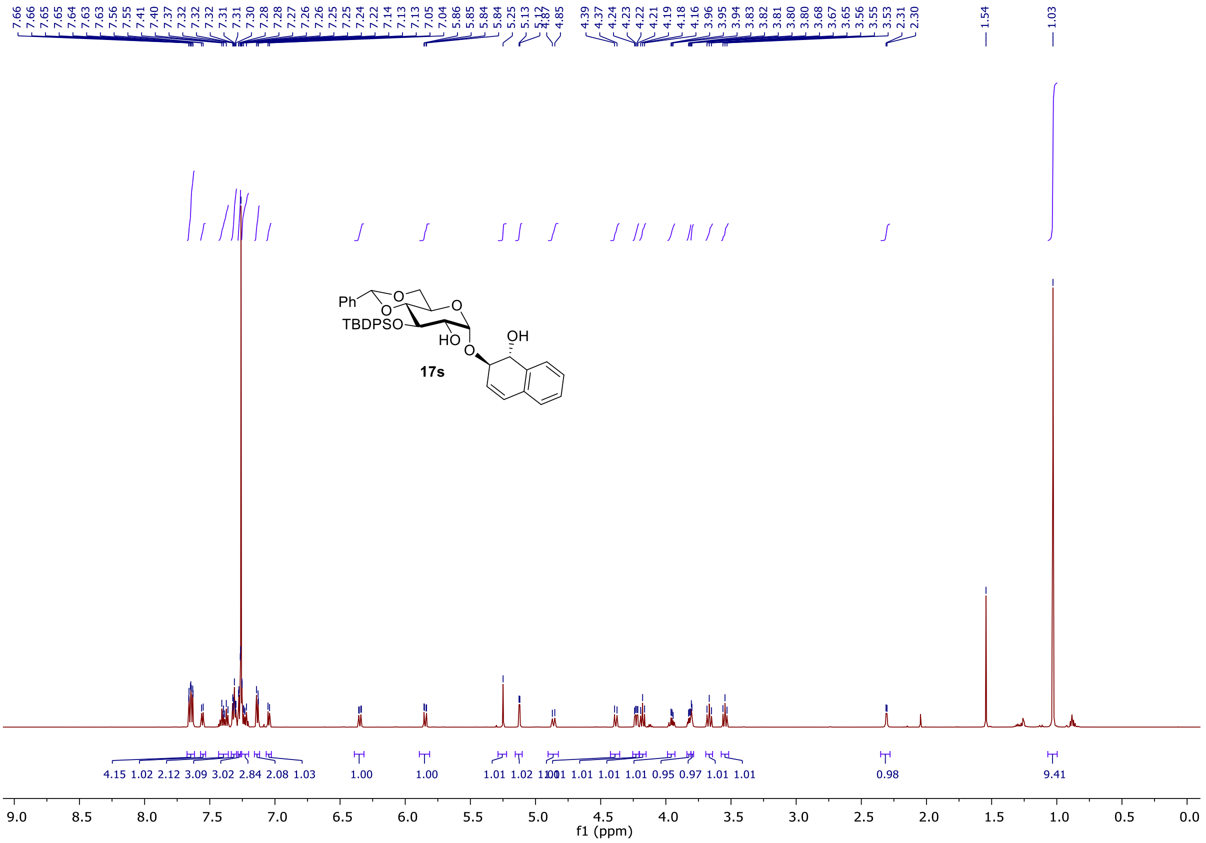

Supplementary Figure 271. ^{1}H spectra for **17s**

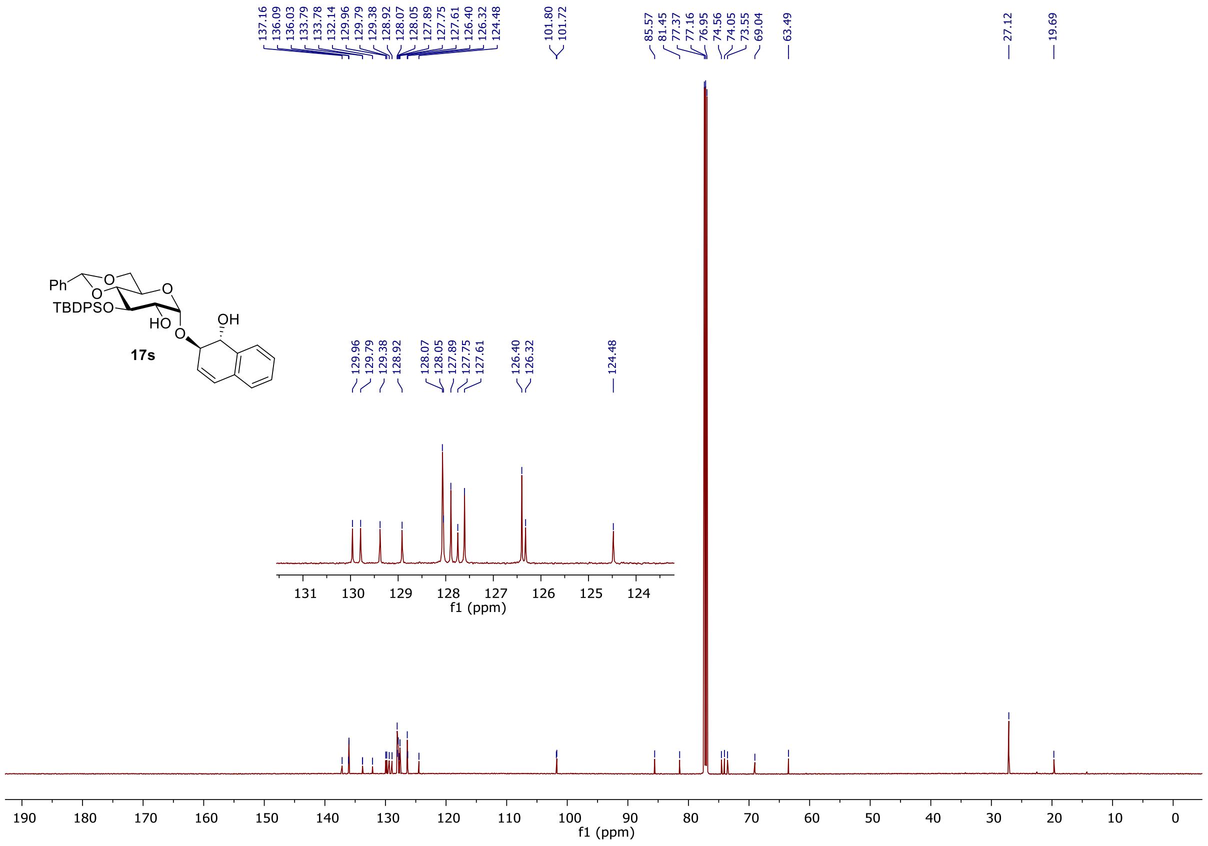

Supplementary Figure 272. ^{13}C spectra for **17s**

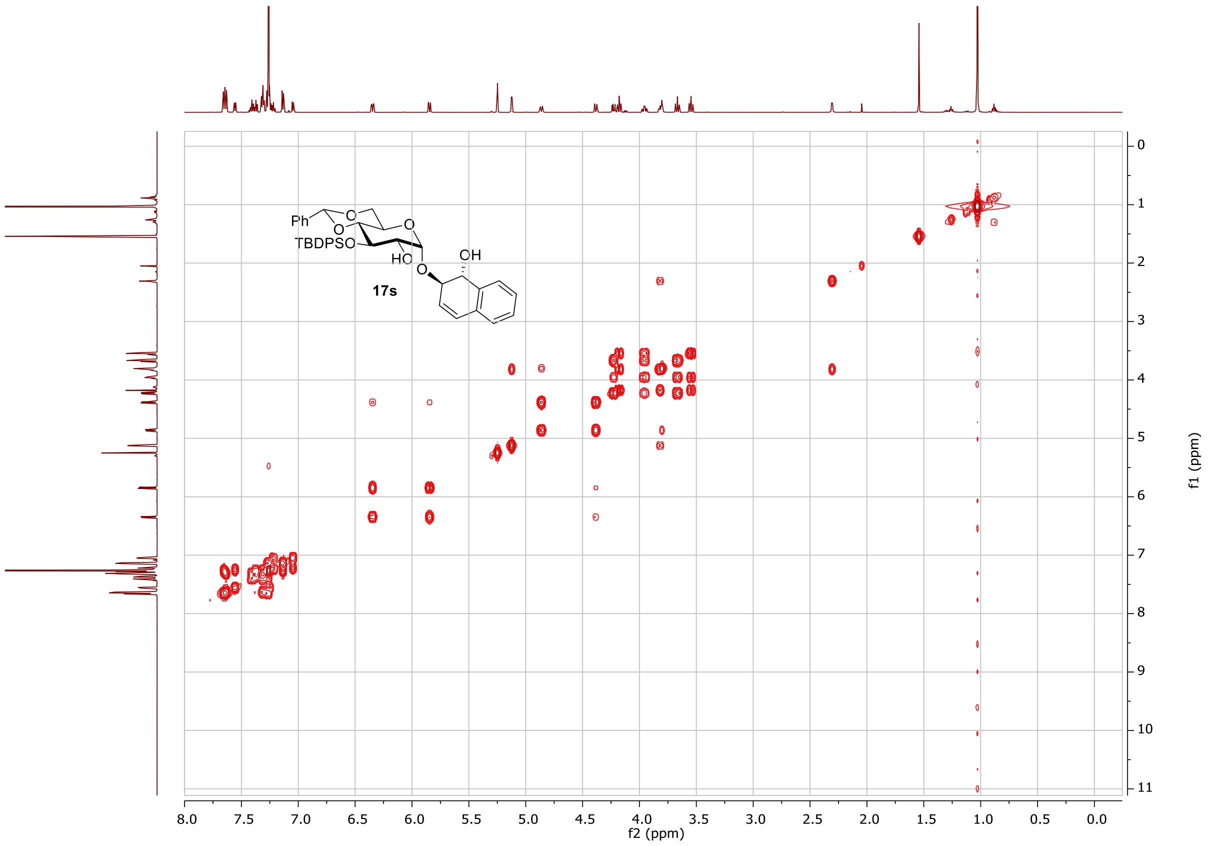

Supplementary Figure 273. COSY spectra for **17s**

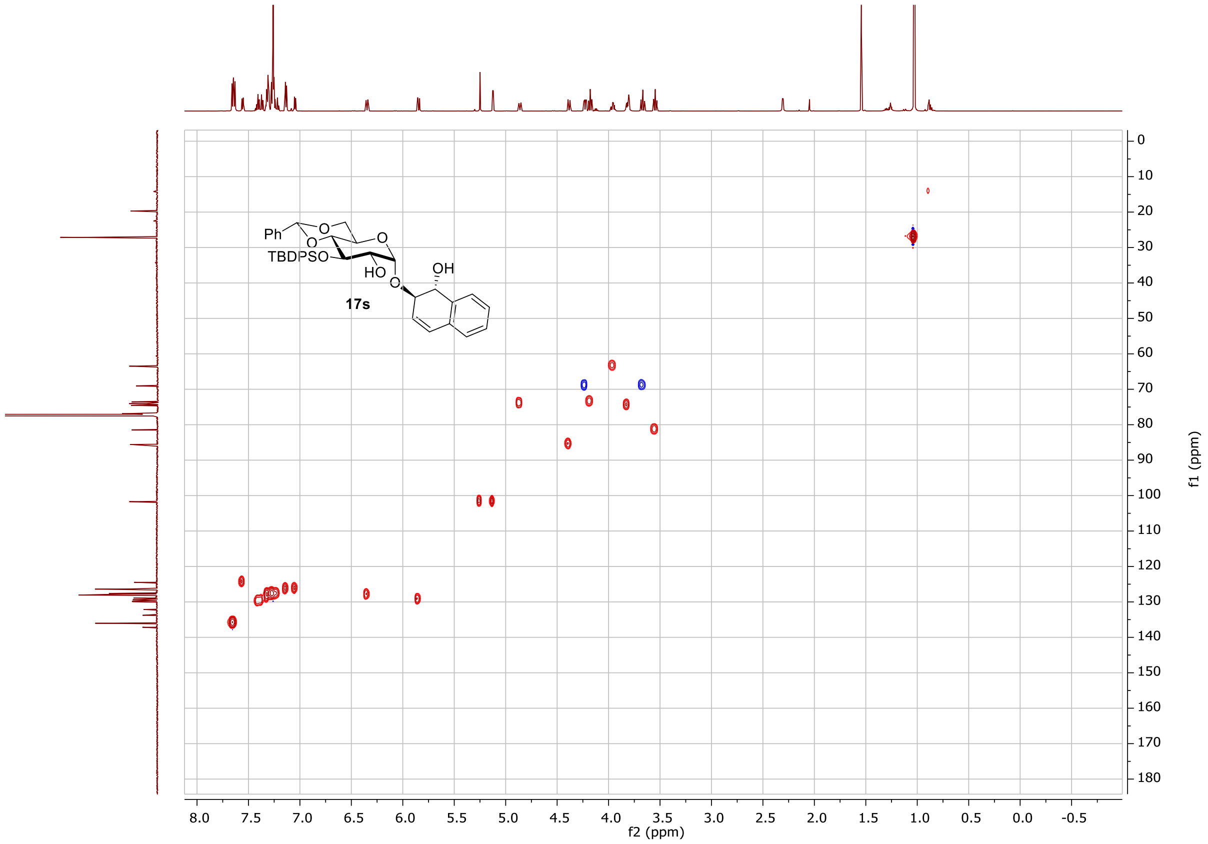

Supplementary Figure 274. HSQC spectra for **17s**

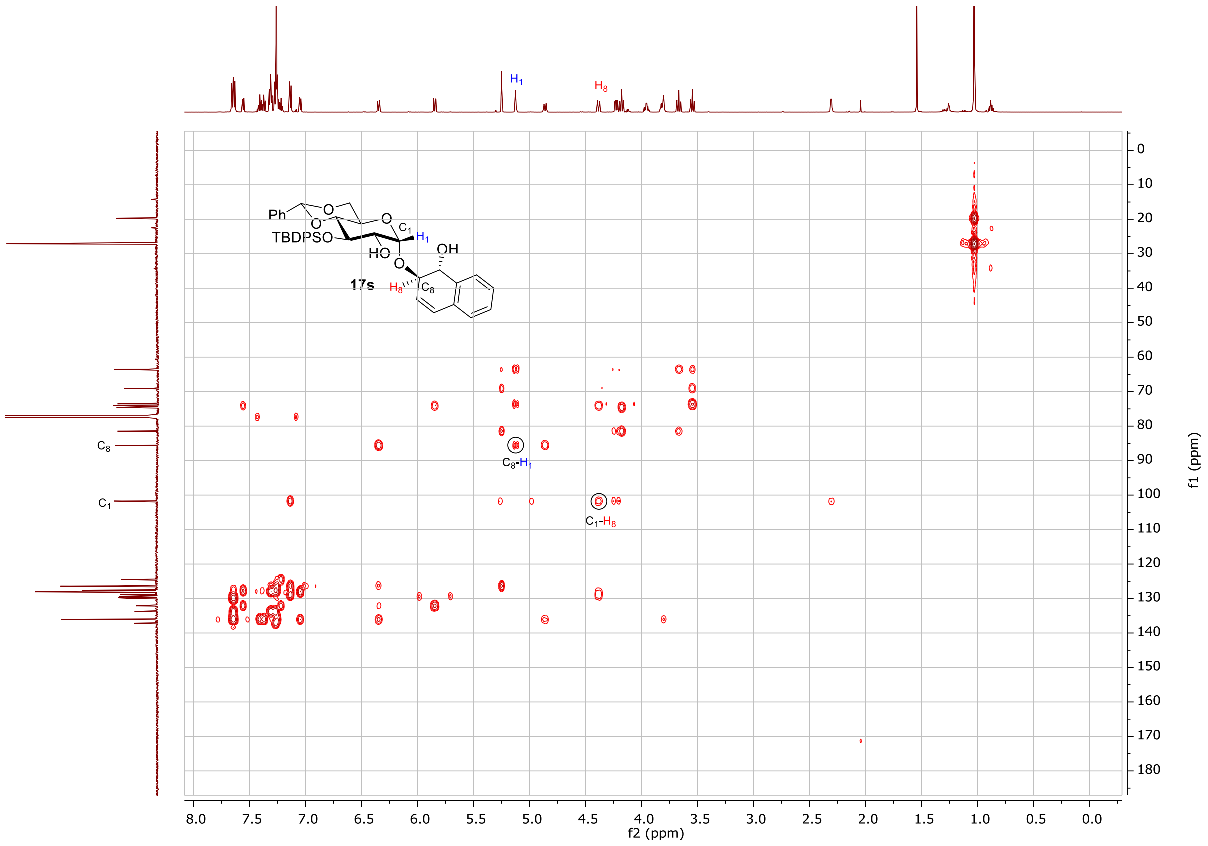

Supplementary Figure 275. HMBC spectra for **17s**

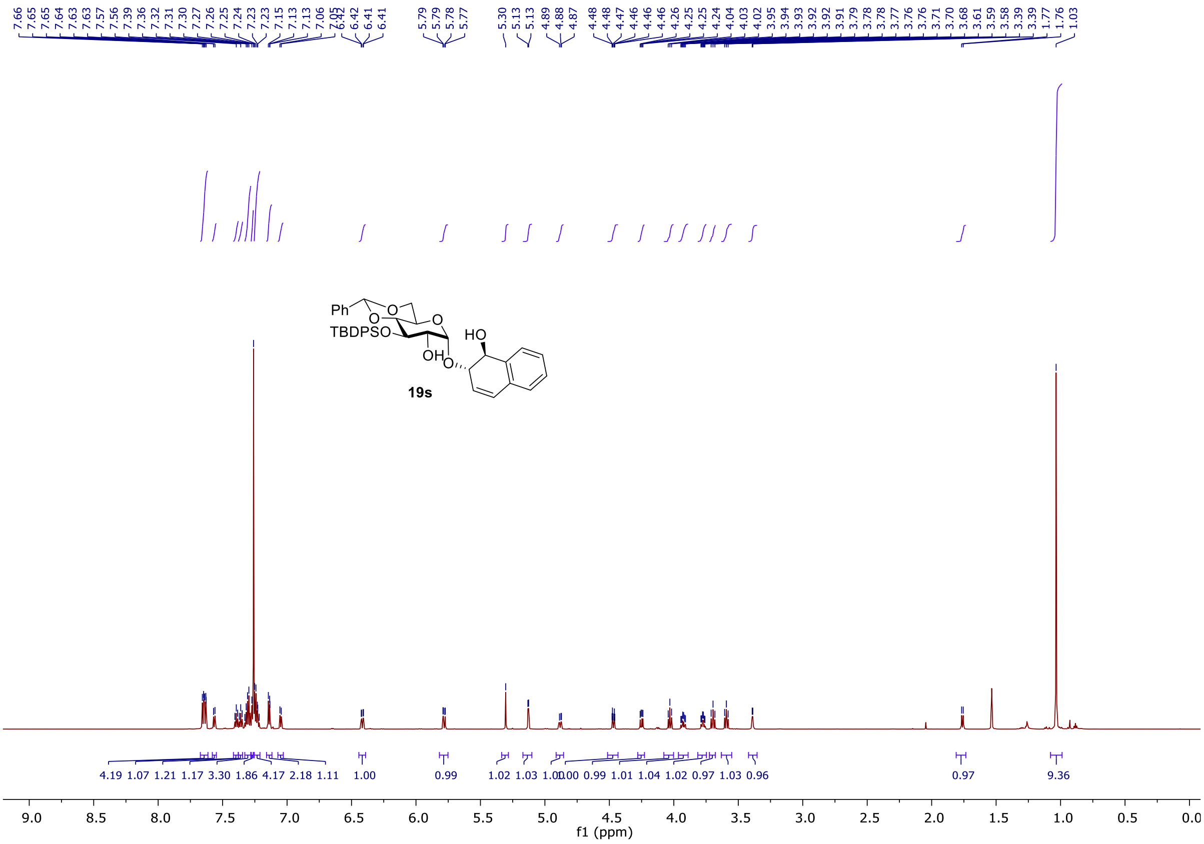

Supplementary Figure 276. ^{1}H spectra for **19s**

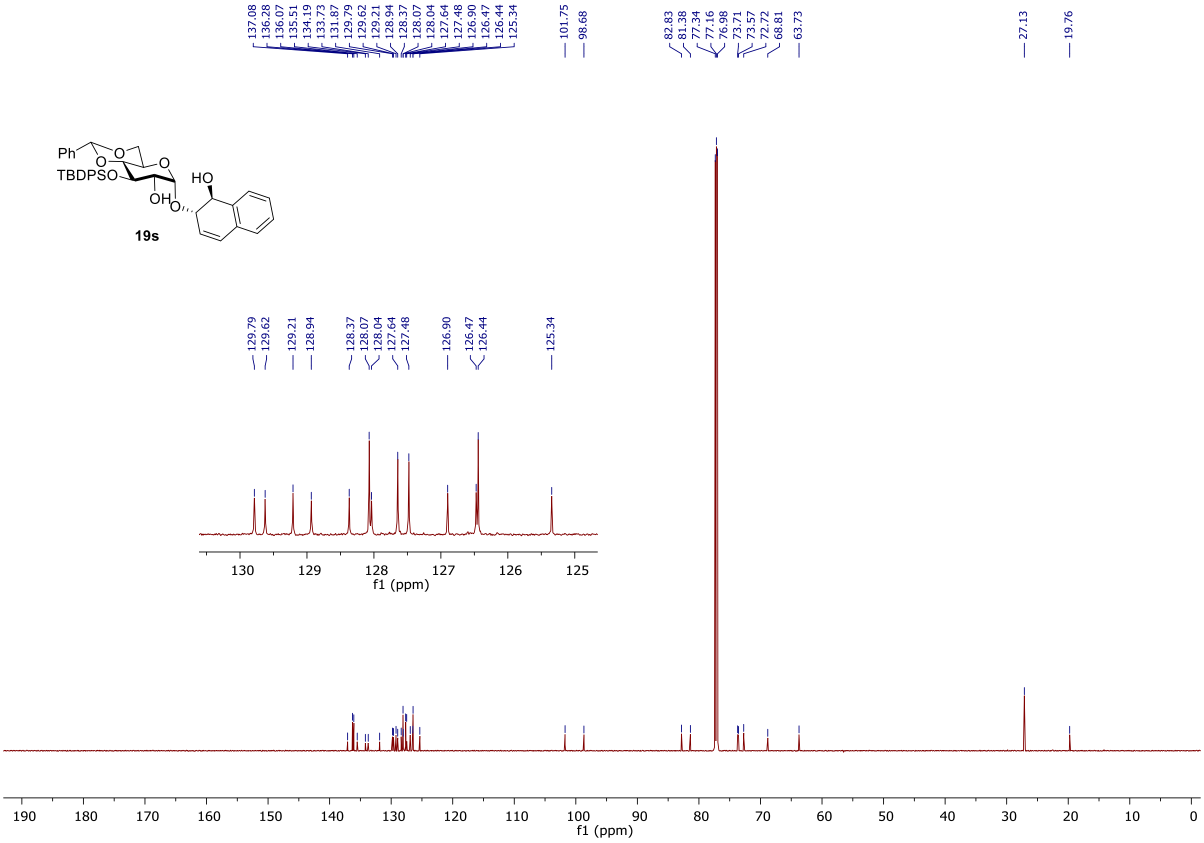

Supplementary Figure 277. ^{13}C spectra for **19s**

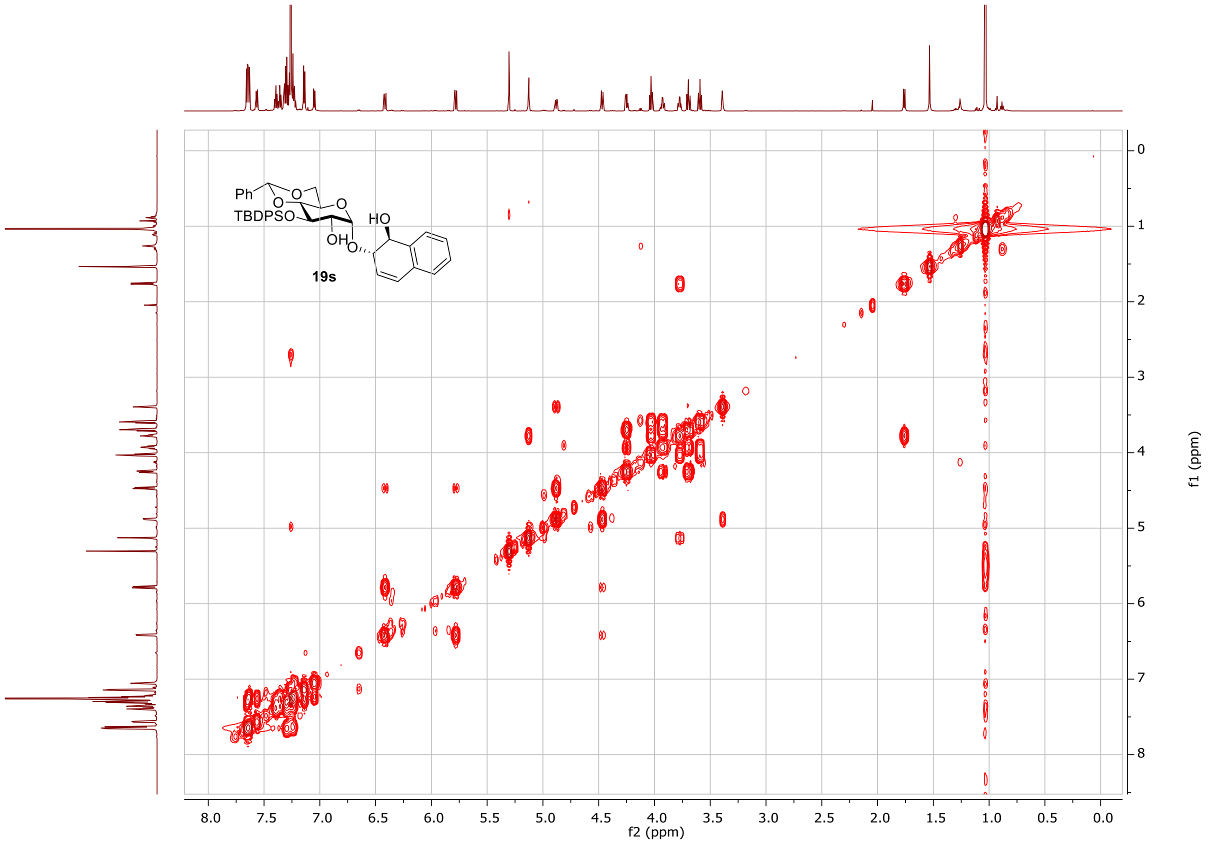

Supplementary Figure 278. COSY spectra for **19s**

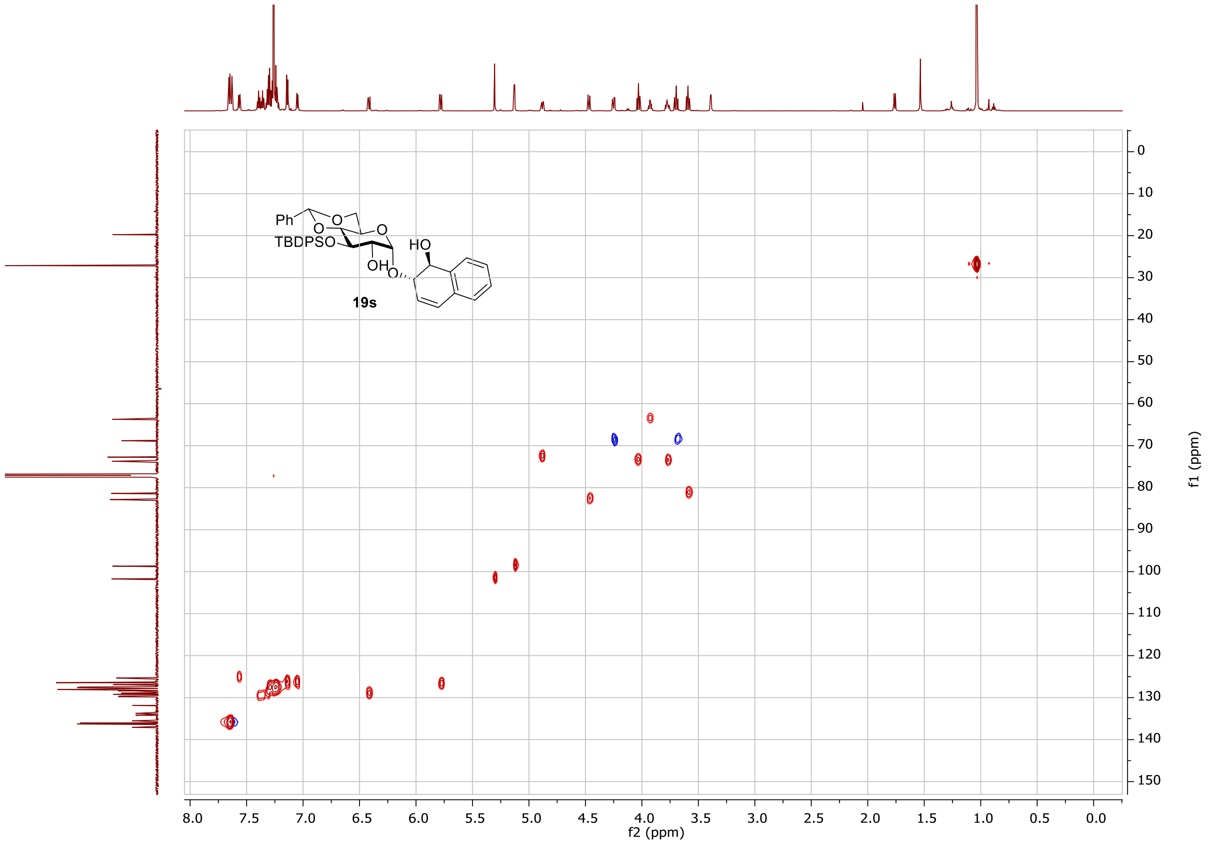

Supplementary Figure 279. HSQC spectra for **19s**

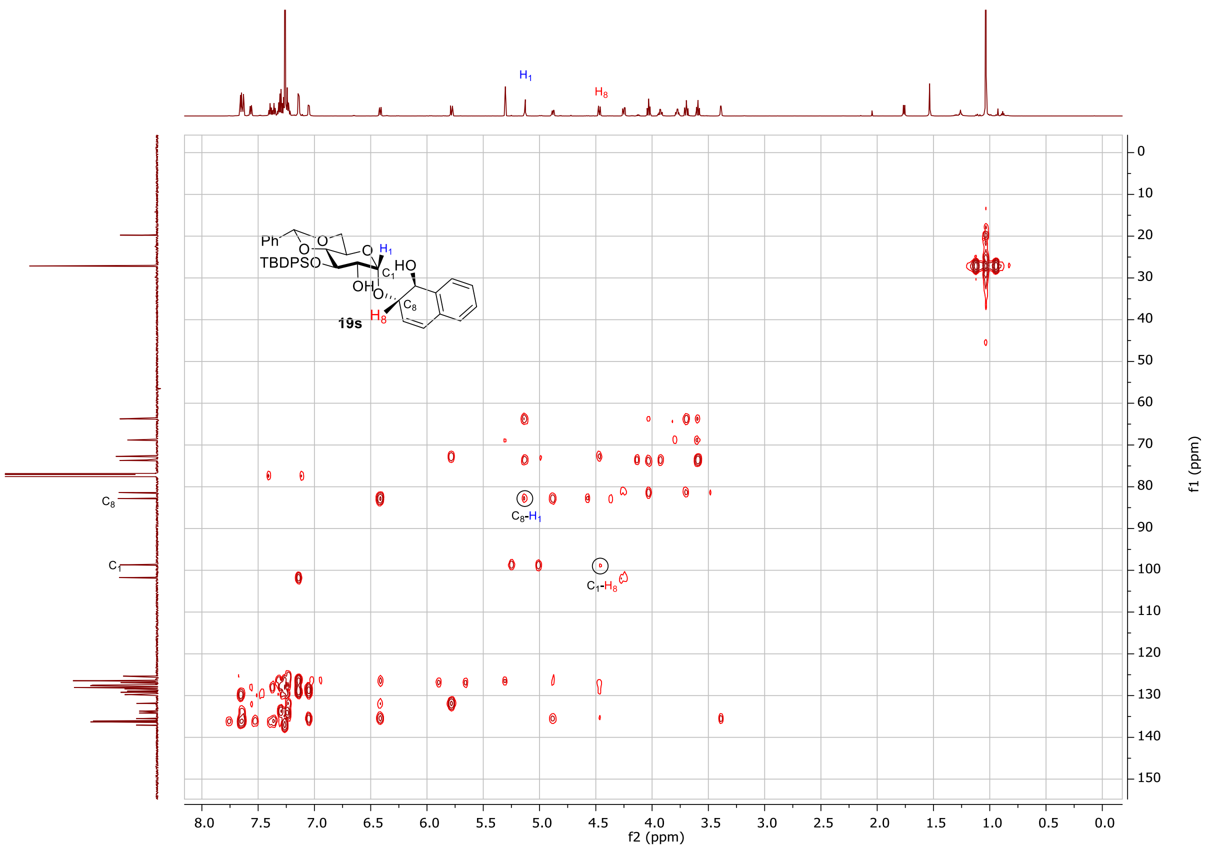

Supplementary Figure S280. HMBC spectra for **19s**

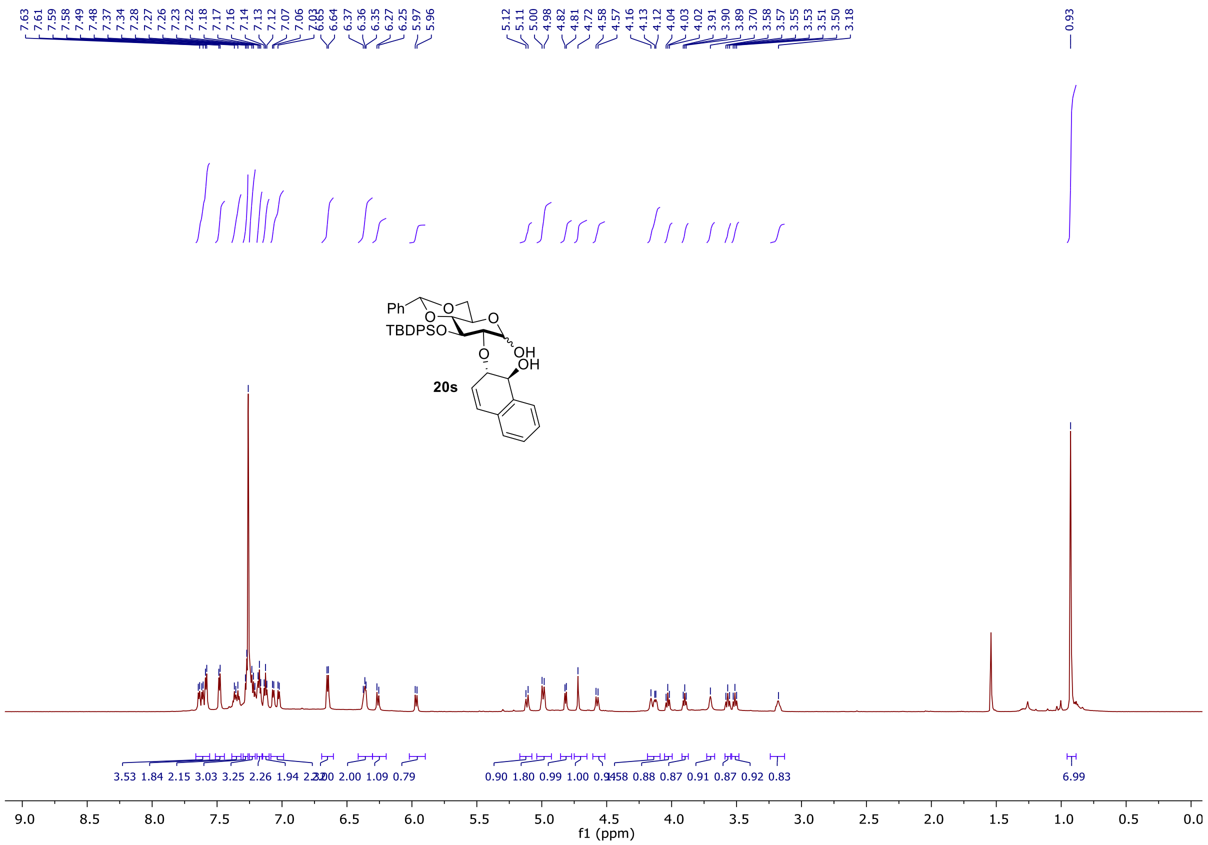

Supplementary Figure 281. ^{1}H spectra for **20s**

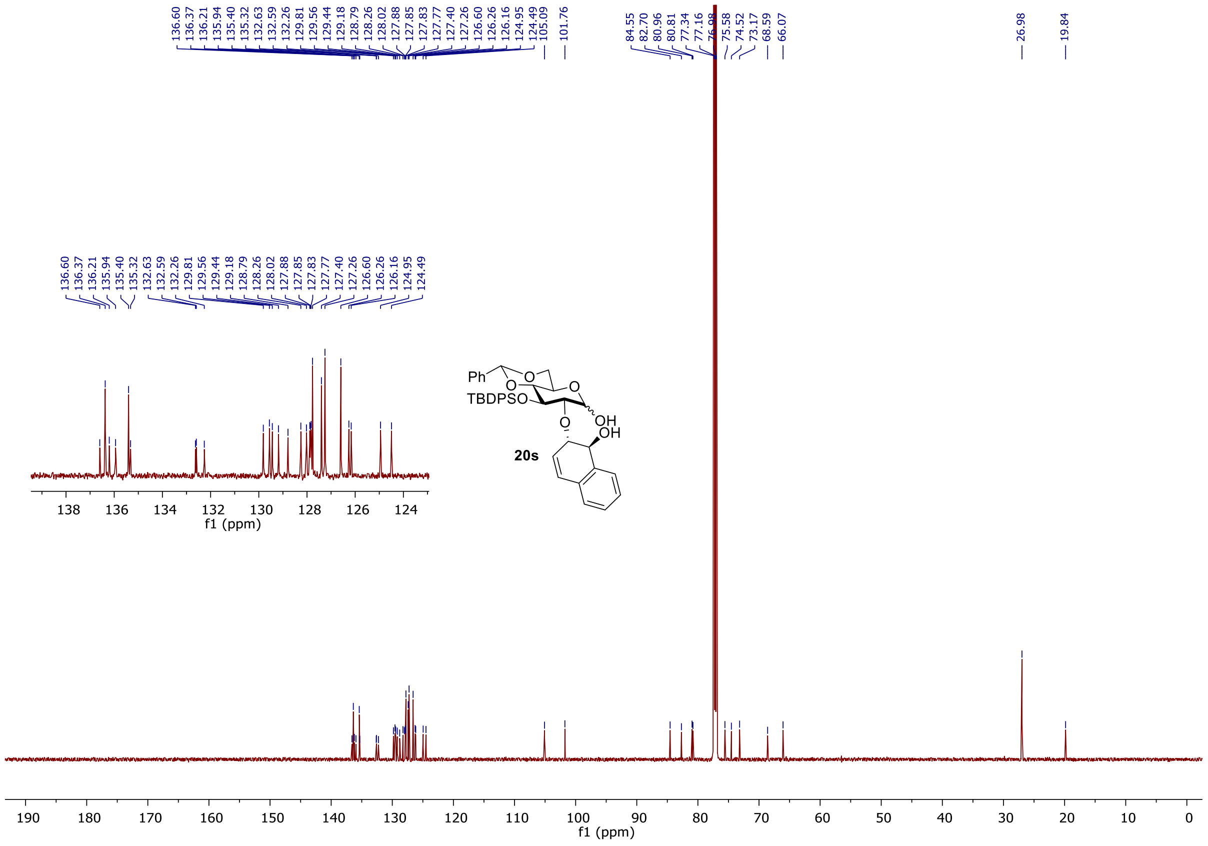

Supplementary Figure 282. ^{13}C spectra for **20s**

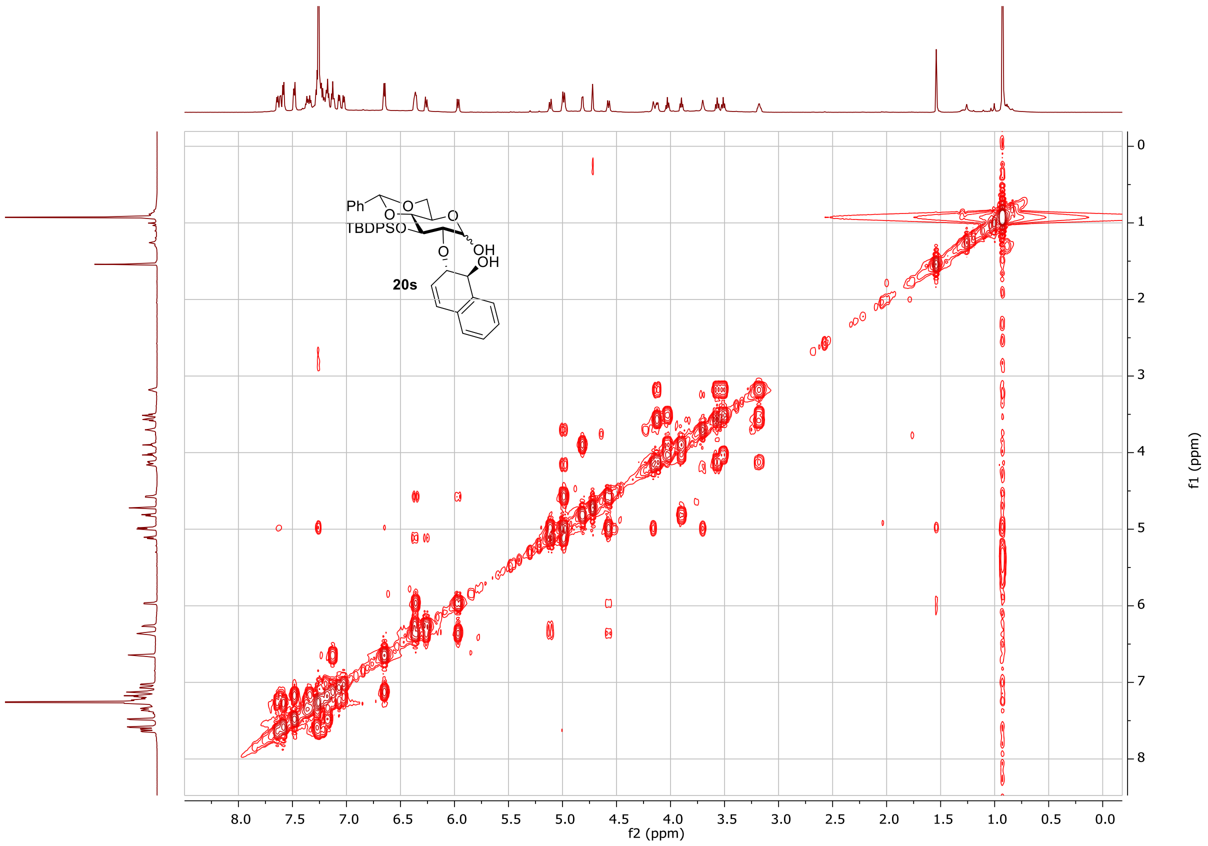

Supplementary Figure 283. COSY spectra for **20s**

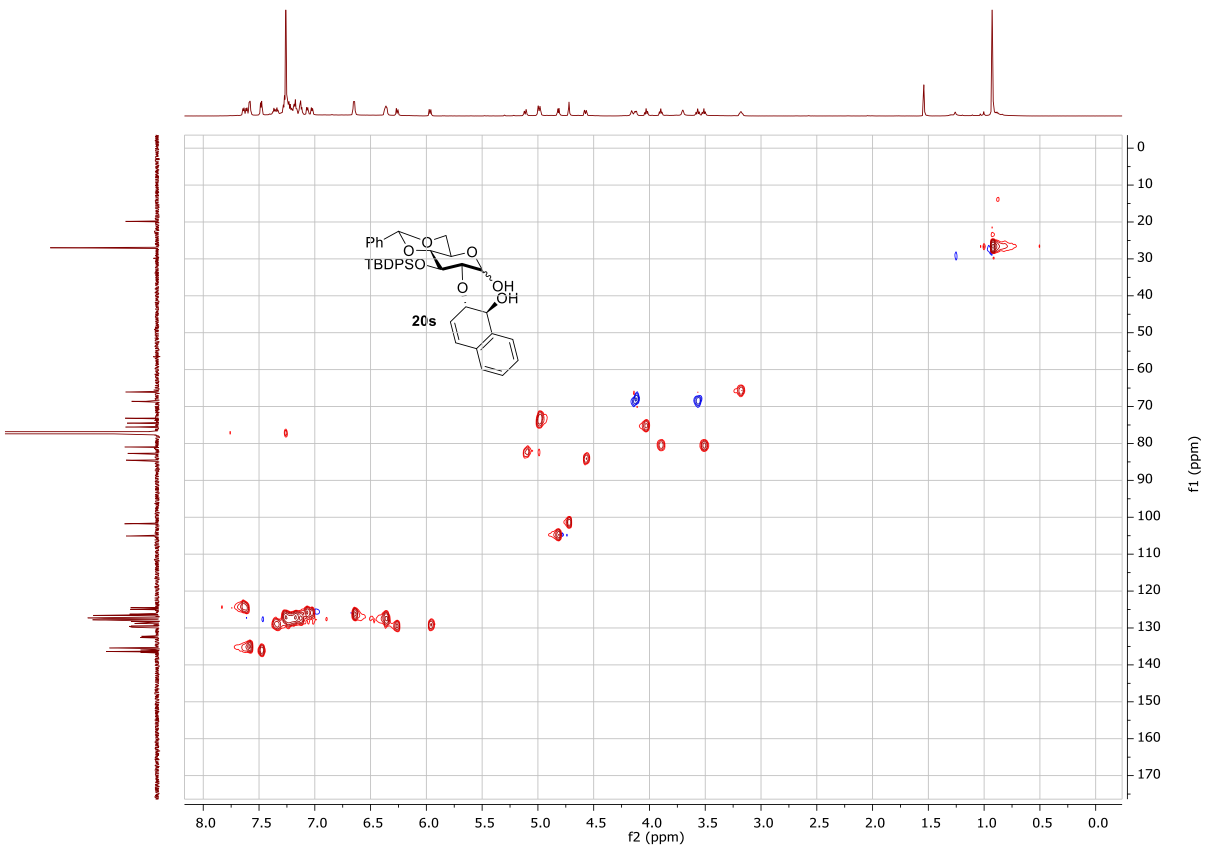

Supplementary Figure 284. HSQC spectra for **20s**

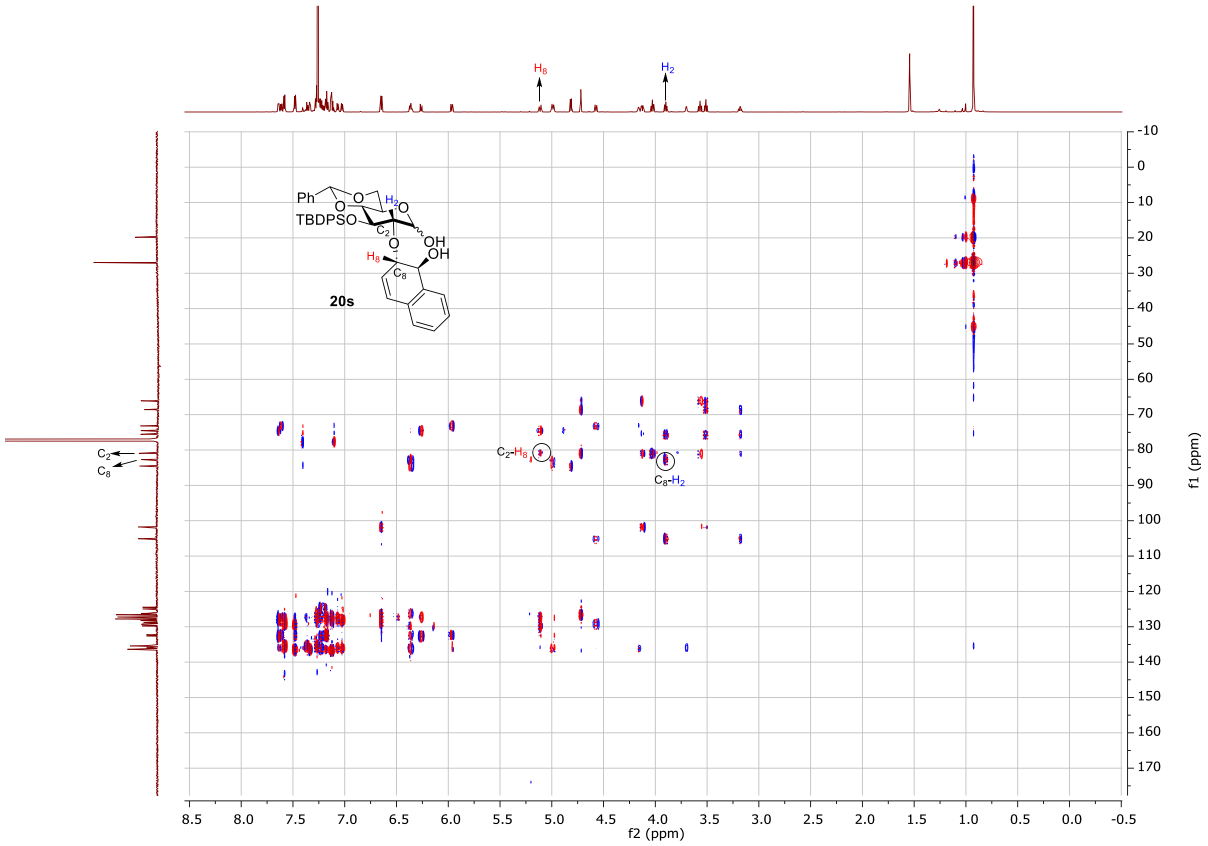

Supplementary Figure 285. HMBC spectra for **20s**

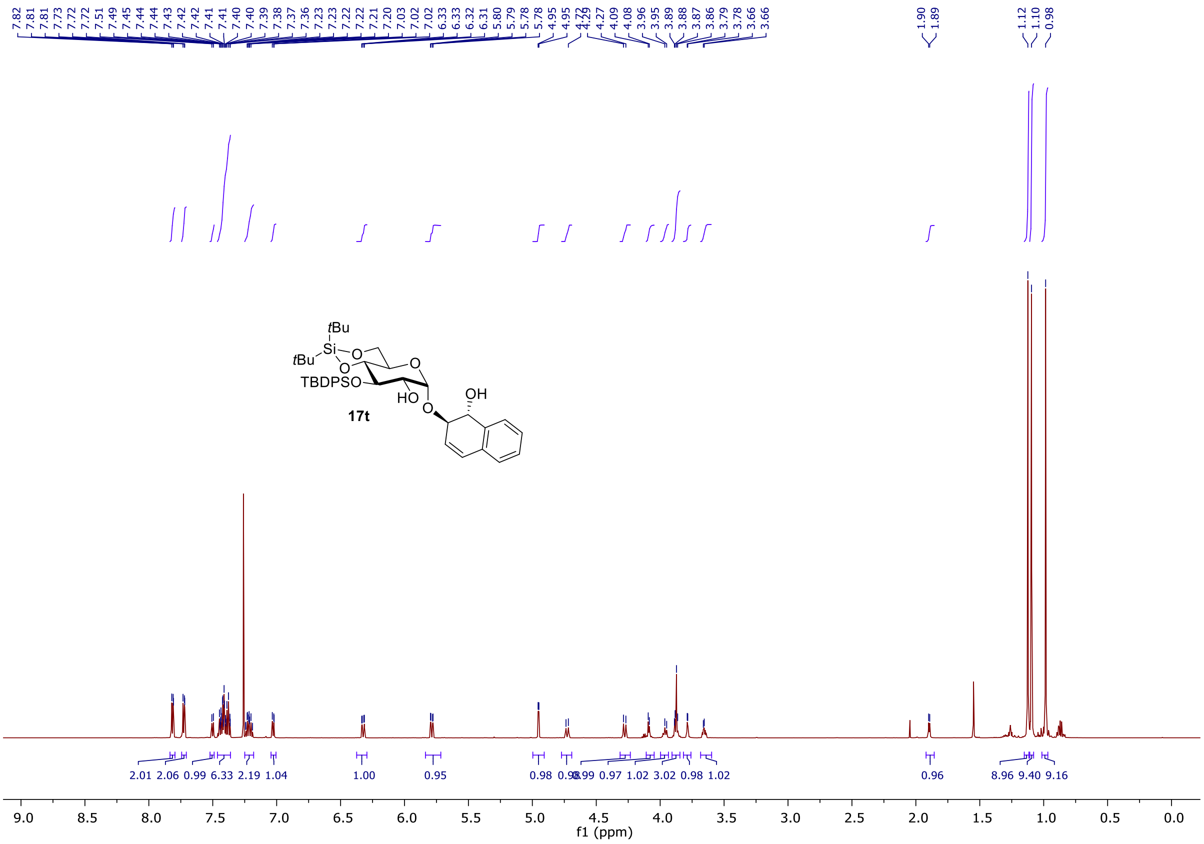

Supplementary Figure 286. ^{1}H spectra for **17t**

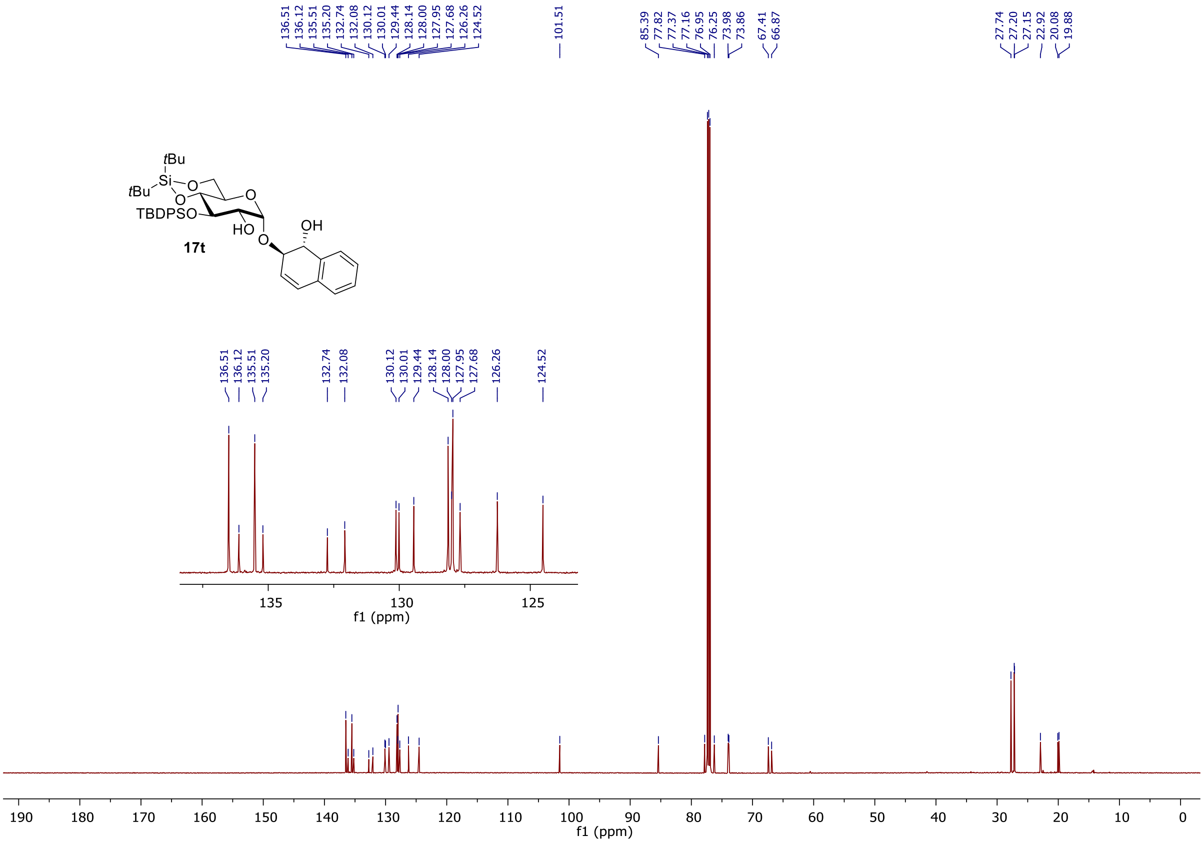

Supplementary Figure 287. ^{13}C spectra for **17t**

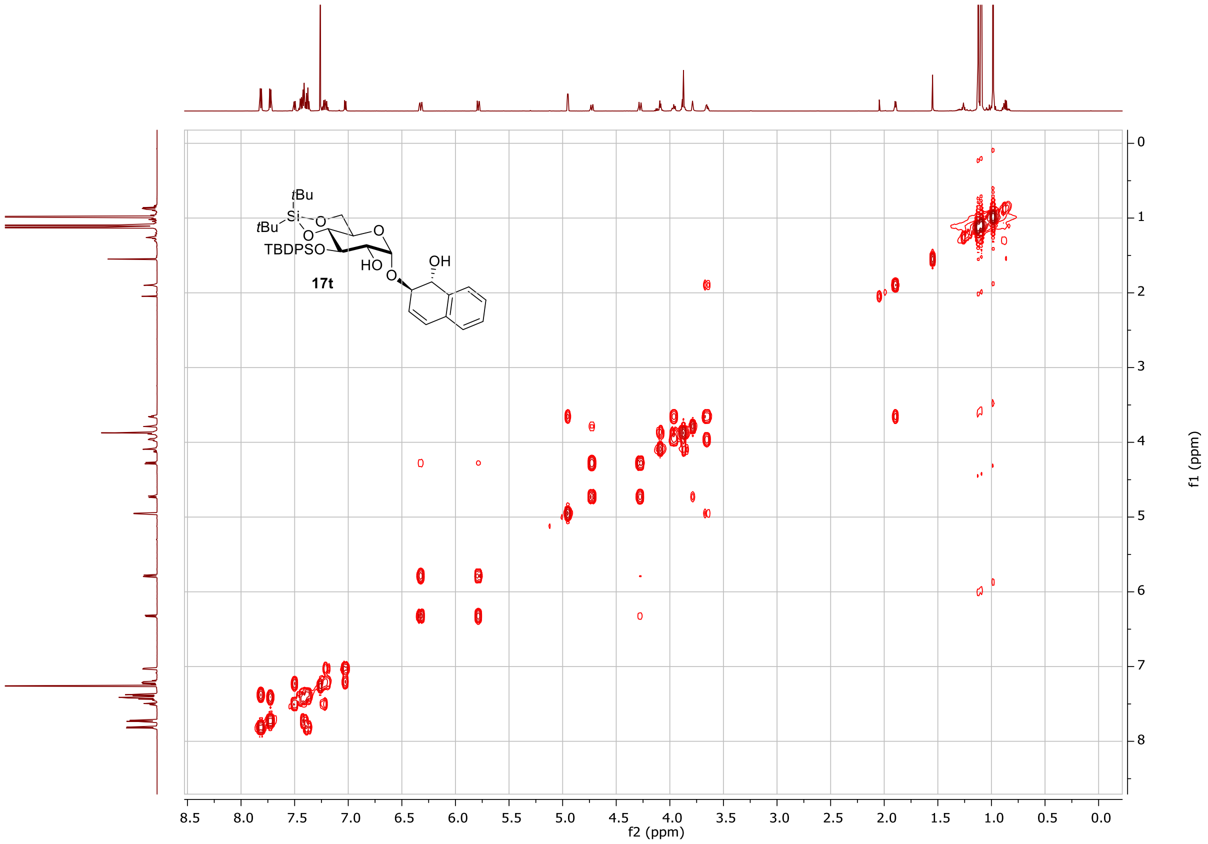

Supplementary Figure S288. COSY spectra for **17t**

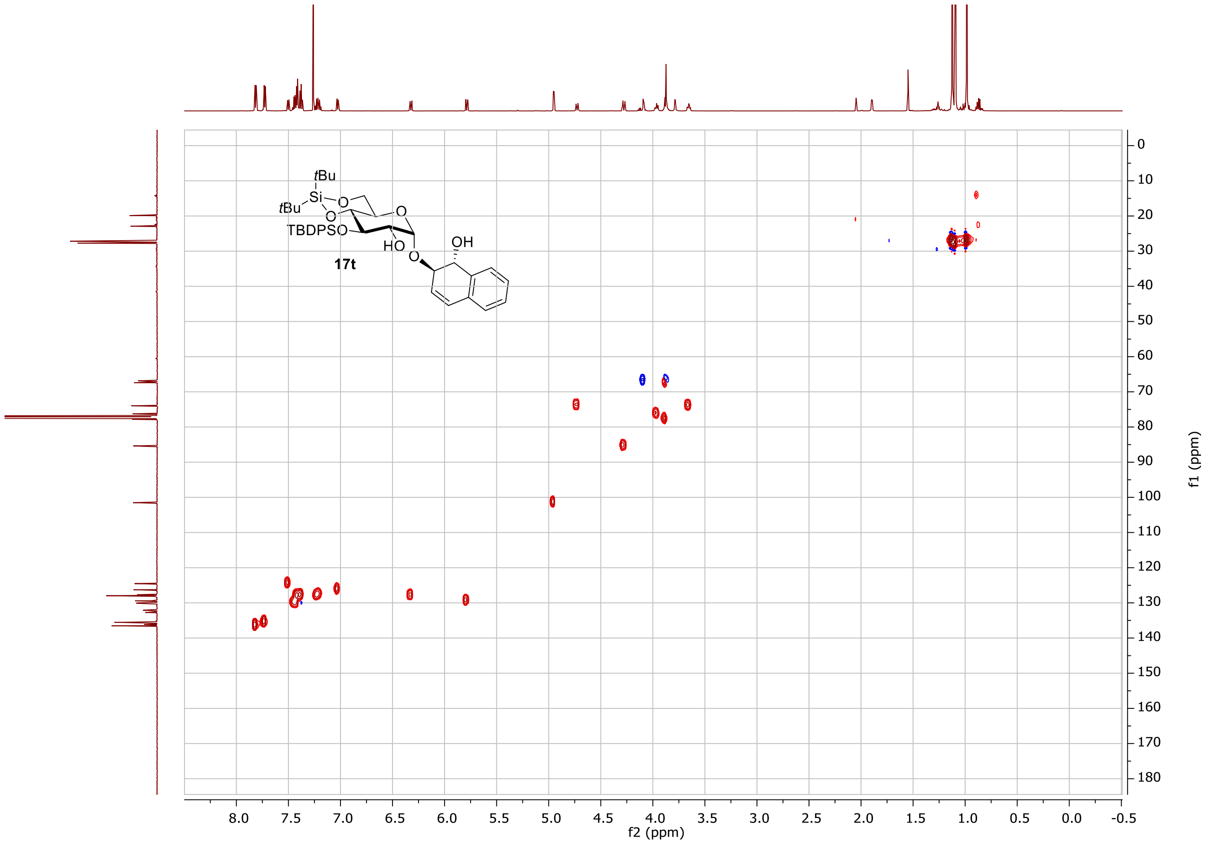

Supplementary Figure S289. HSQC spectra for **17t**

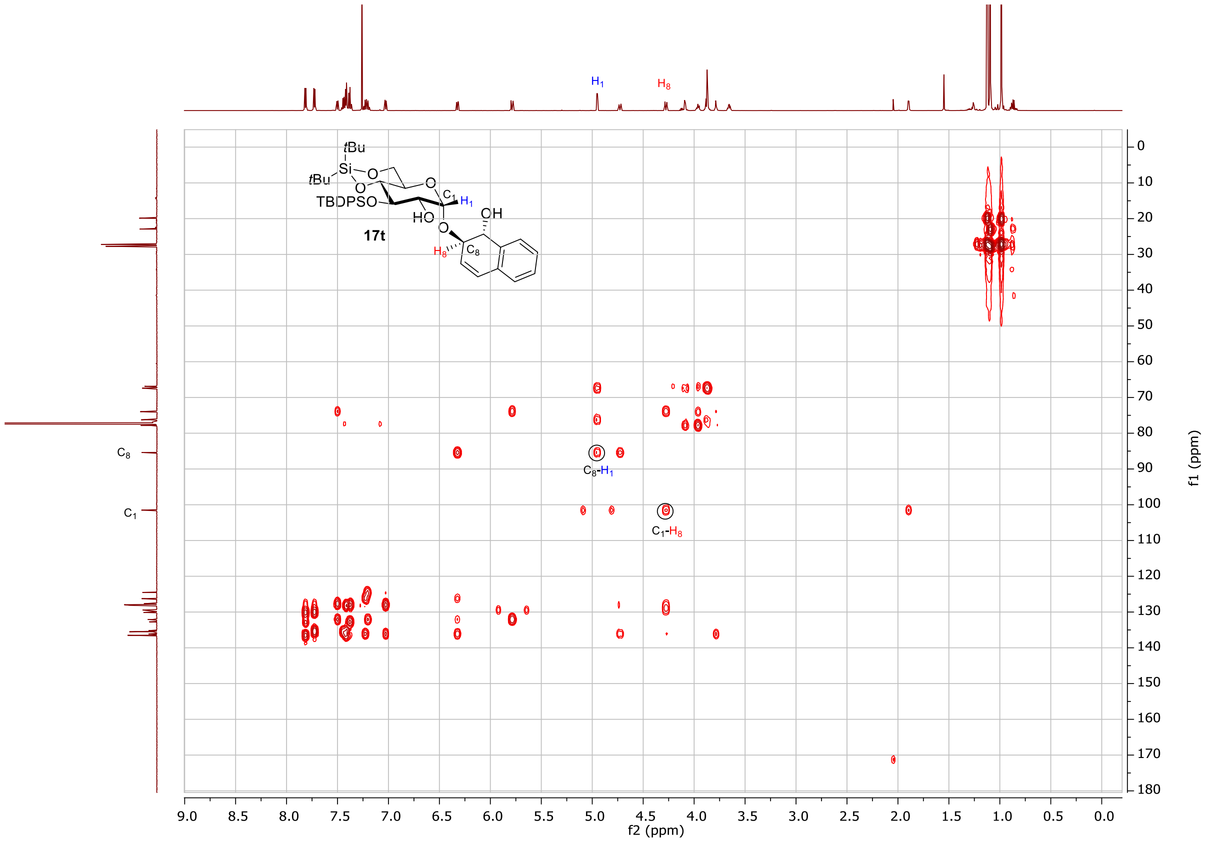

Supplementary Figure S290. HMBC spectra for **17t**

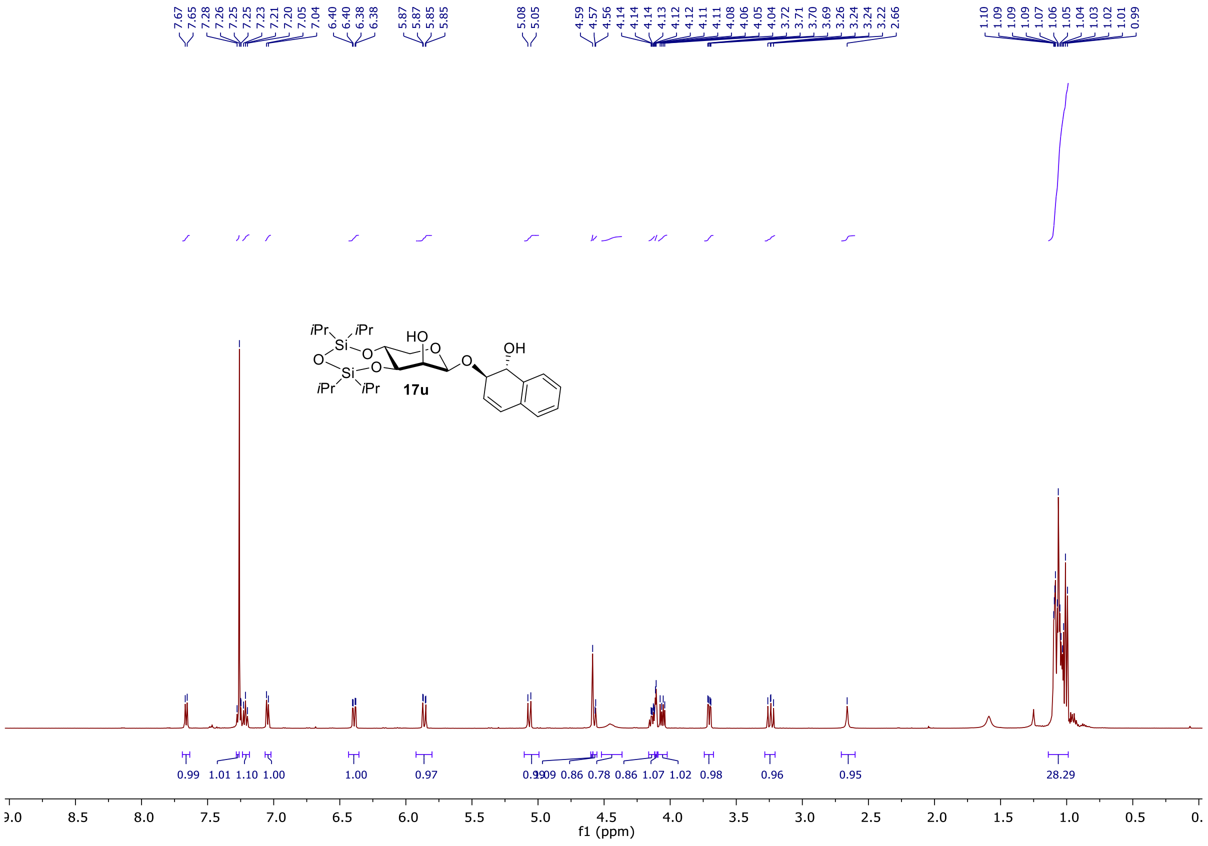

Supplementary Figure S291. ^{1}H spectra for **17u**

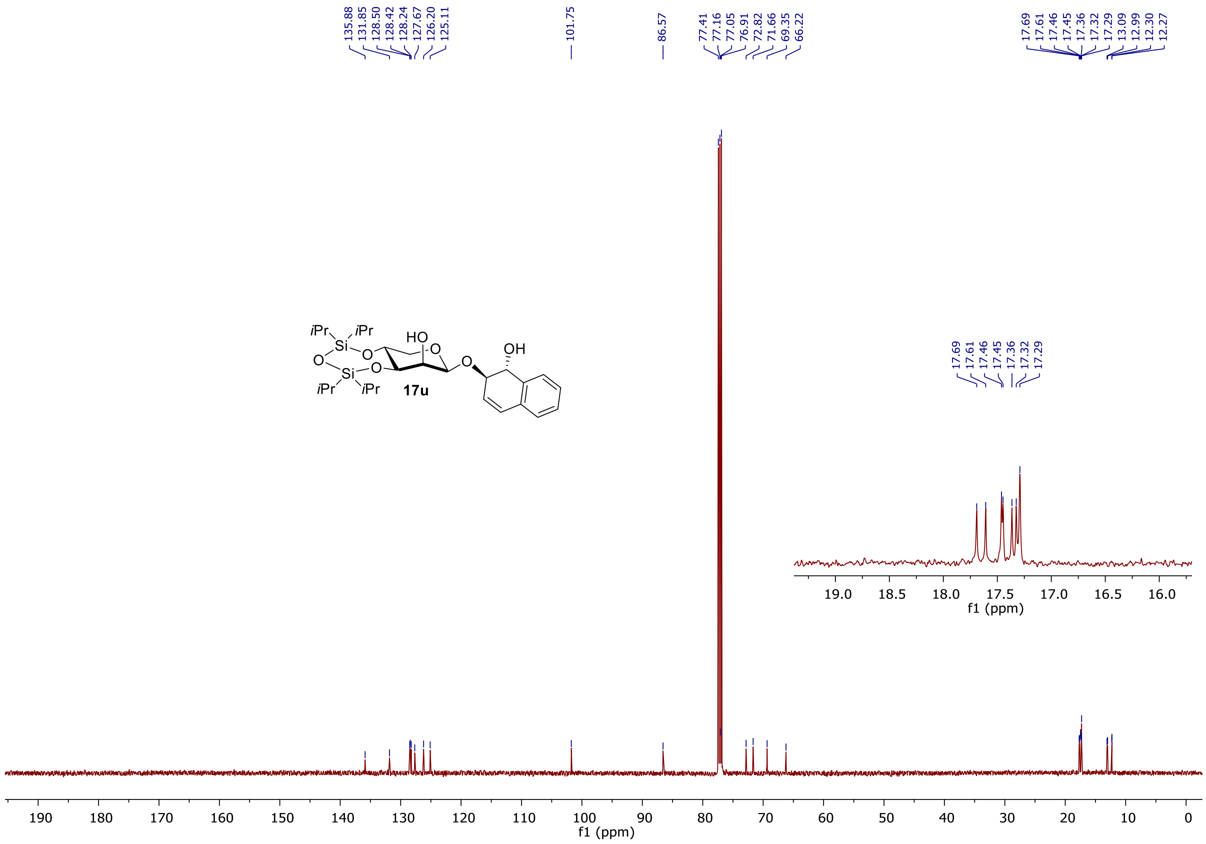

Supplementary Figure S292. ^{13}C spectra for **17u**

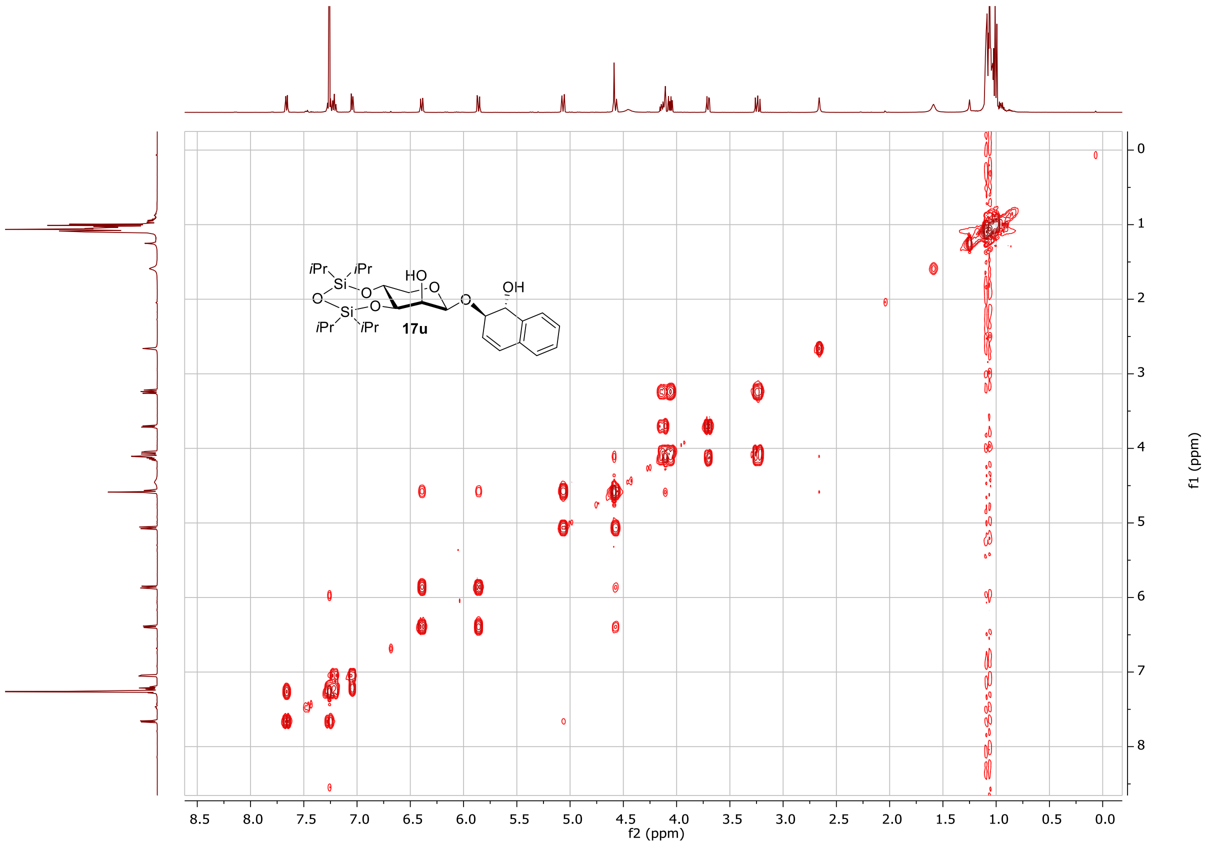

Supplementary Figure S293. COSY spectra for **17u**

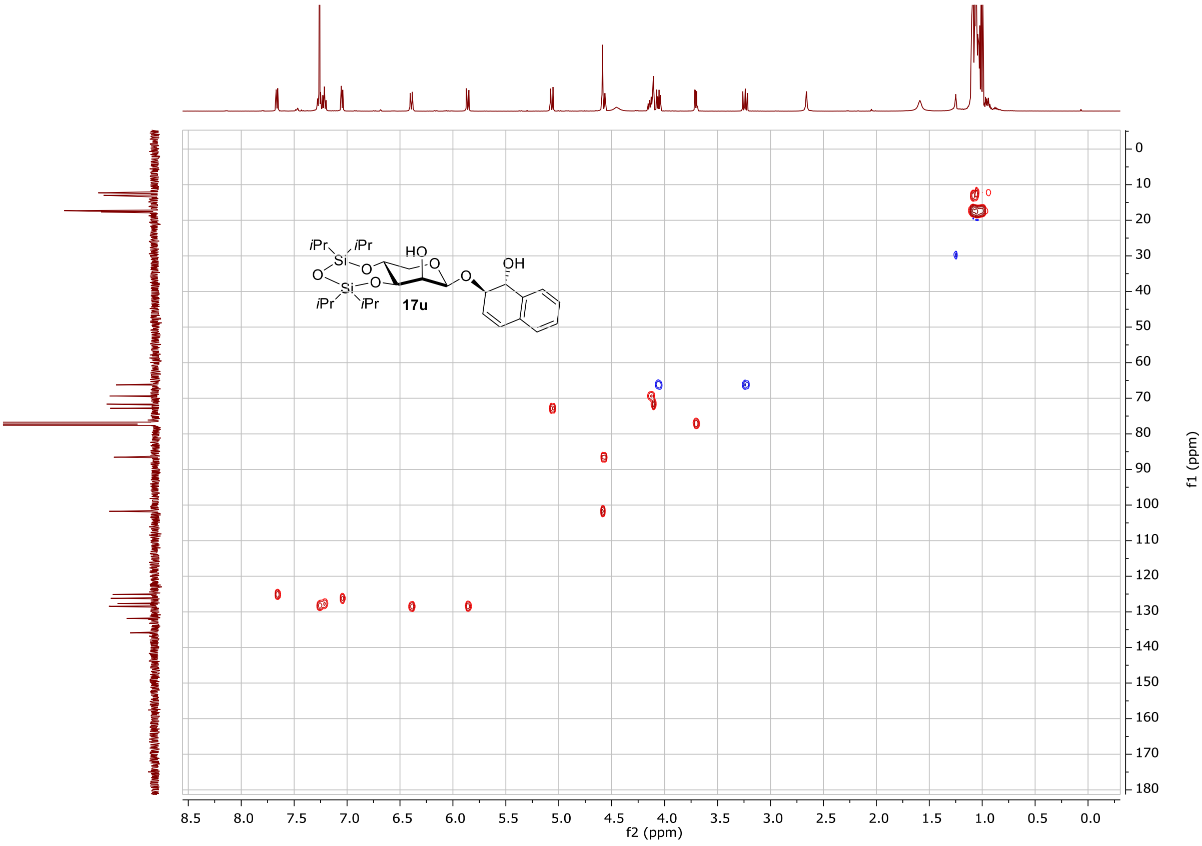

Supplementary Figure S294. HSQC spectra for **17u**

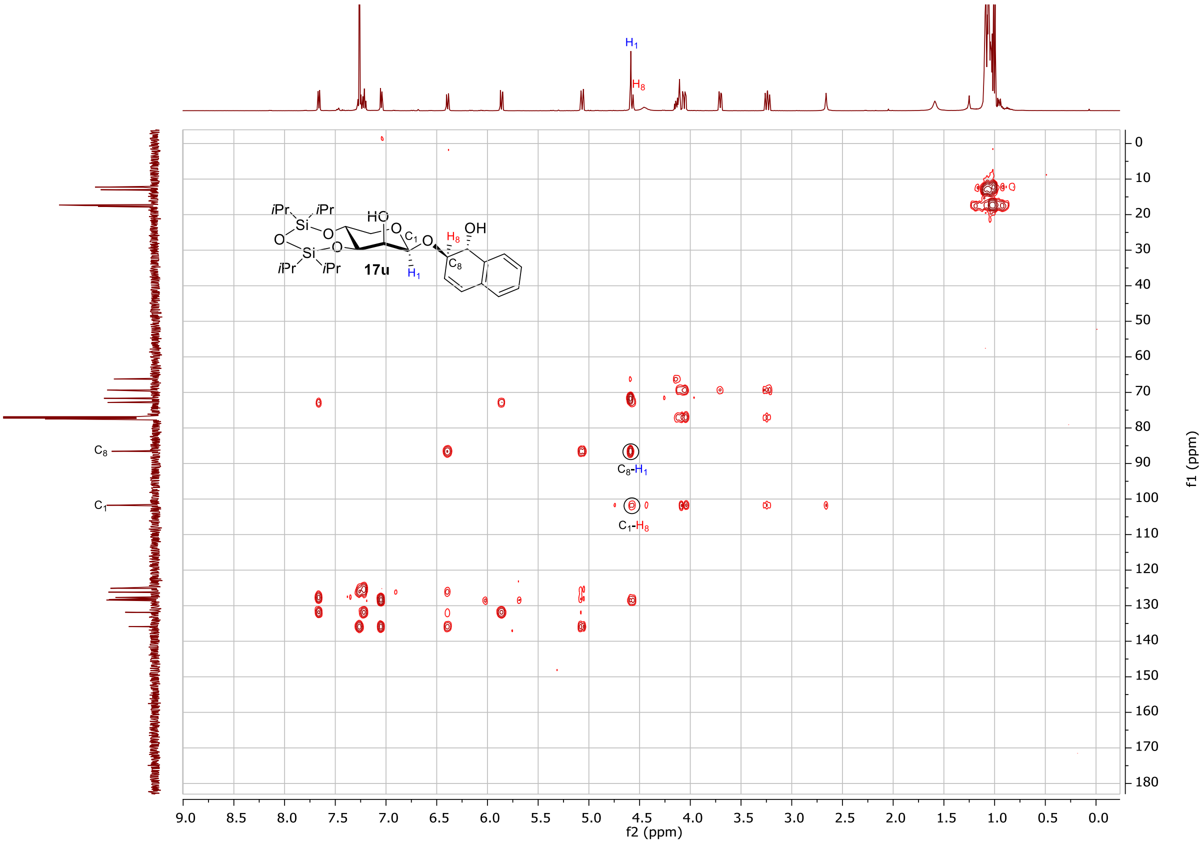

Supplementary Figure S295. HMBC spectra for **17u**

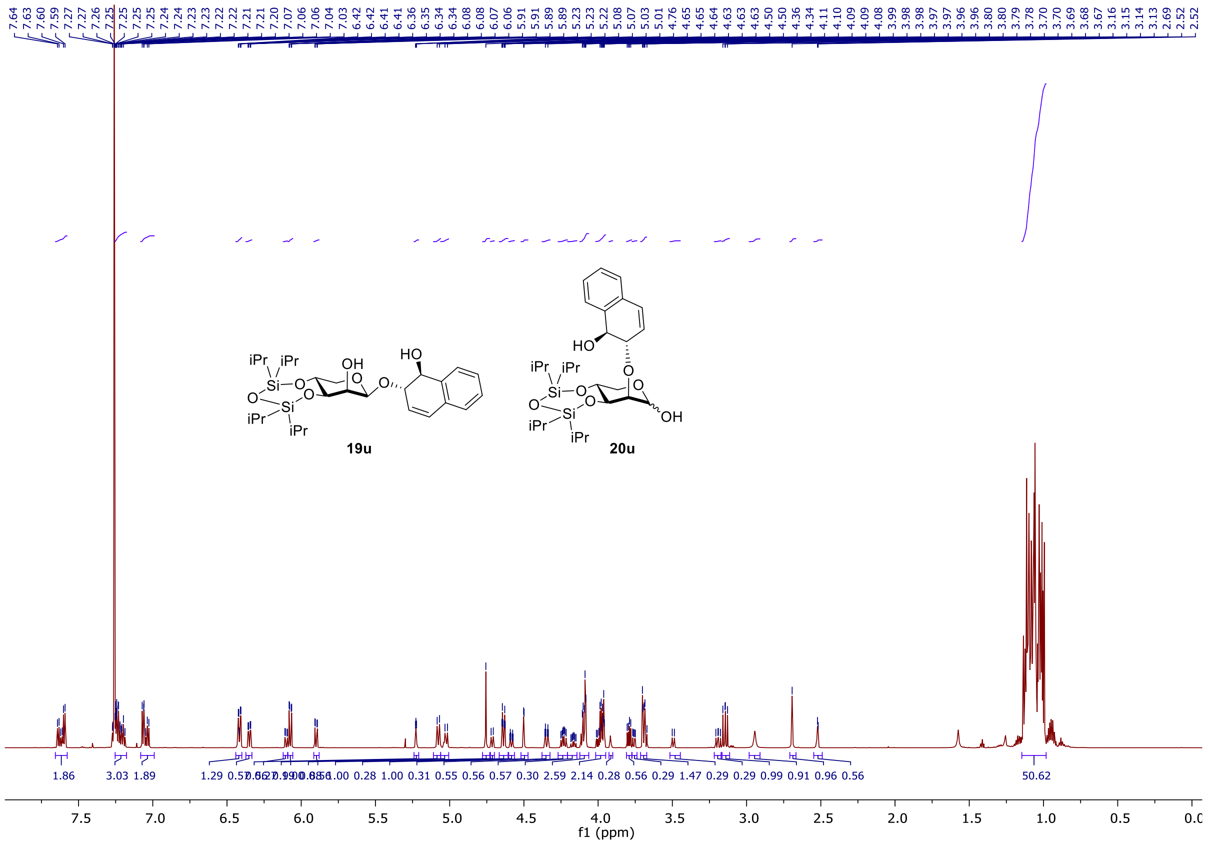

Supplementary Figure S296. ^{1}H spectra for **19u&20u**

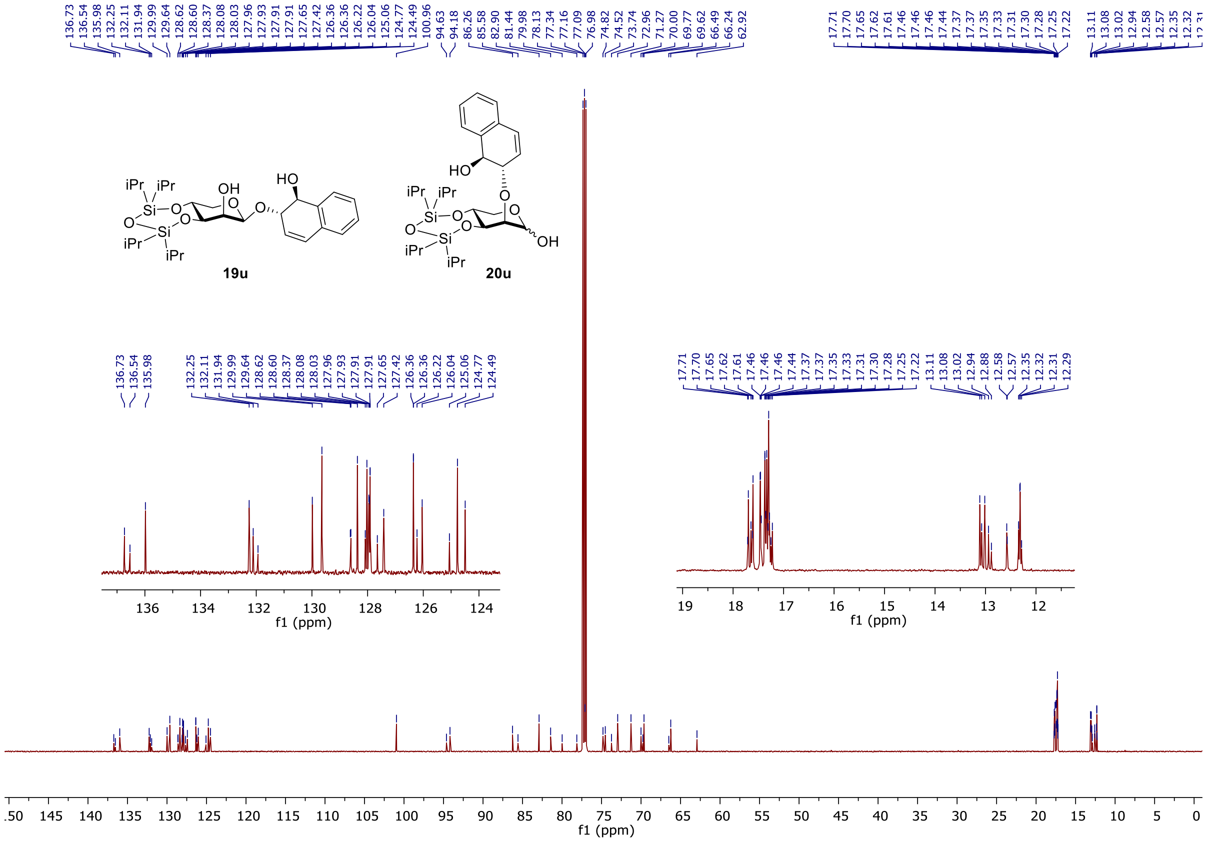

Supplementary Figure S297. ^{13}C spectra for **19u&20u**

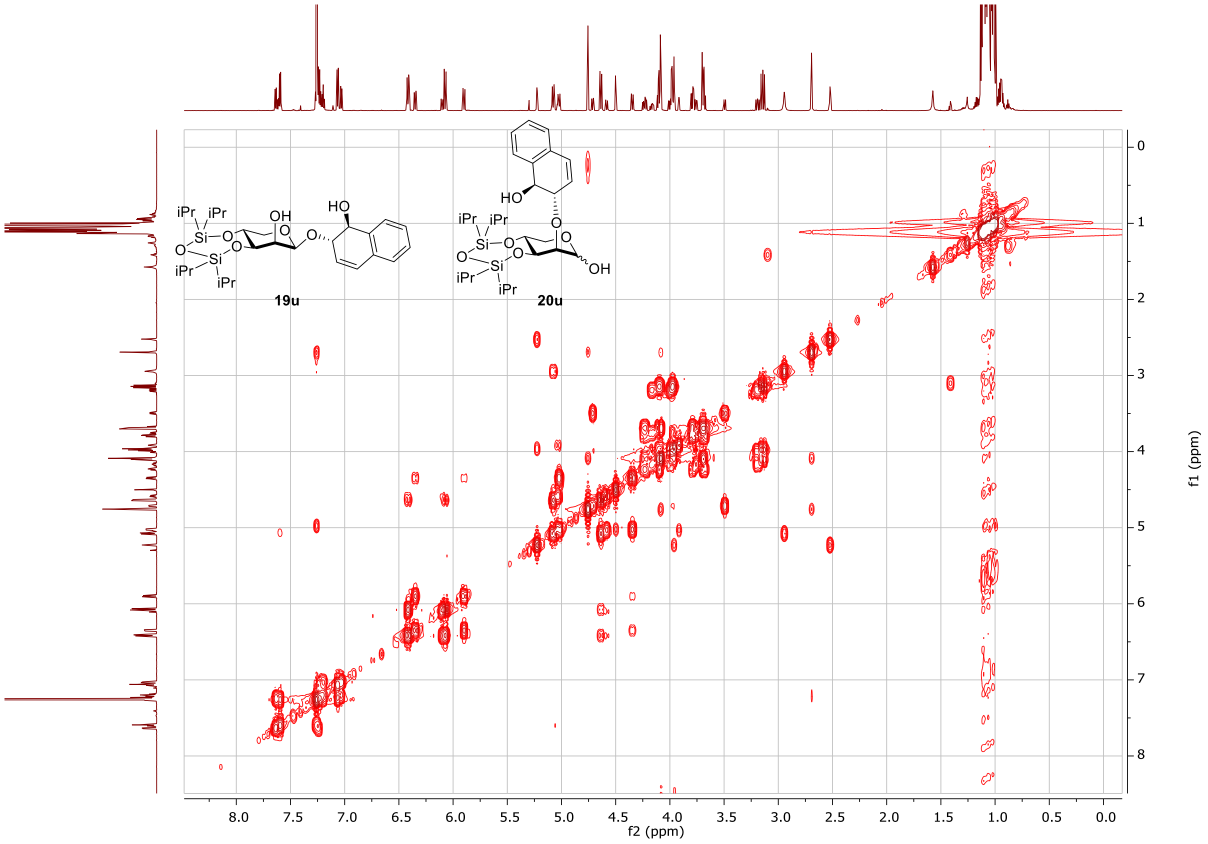

Supplementary Figure S298. COSY spectra for **19u&20u**

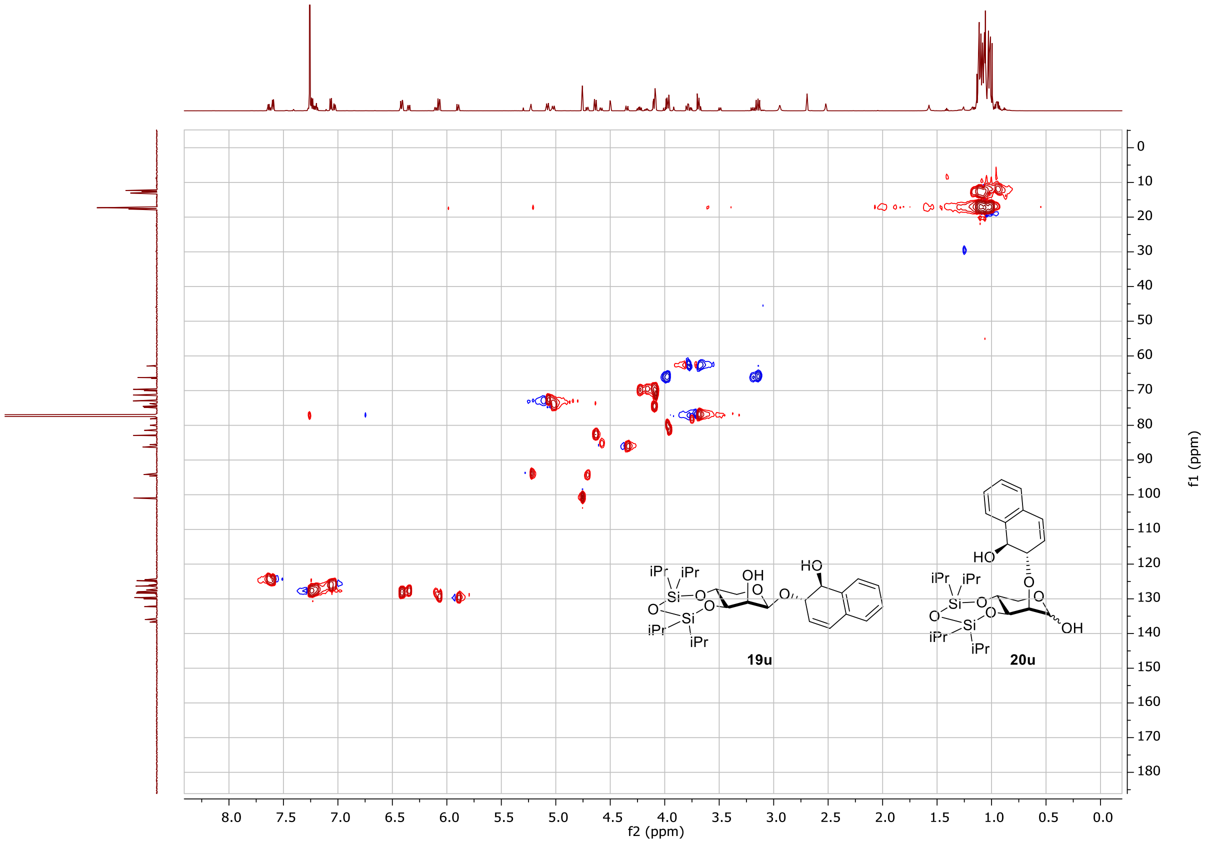

Supplementary Figure S299. HSQC spectra for **19u&20u**

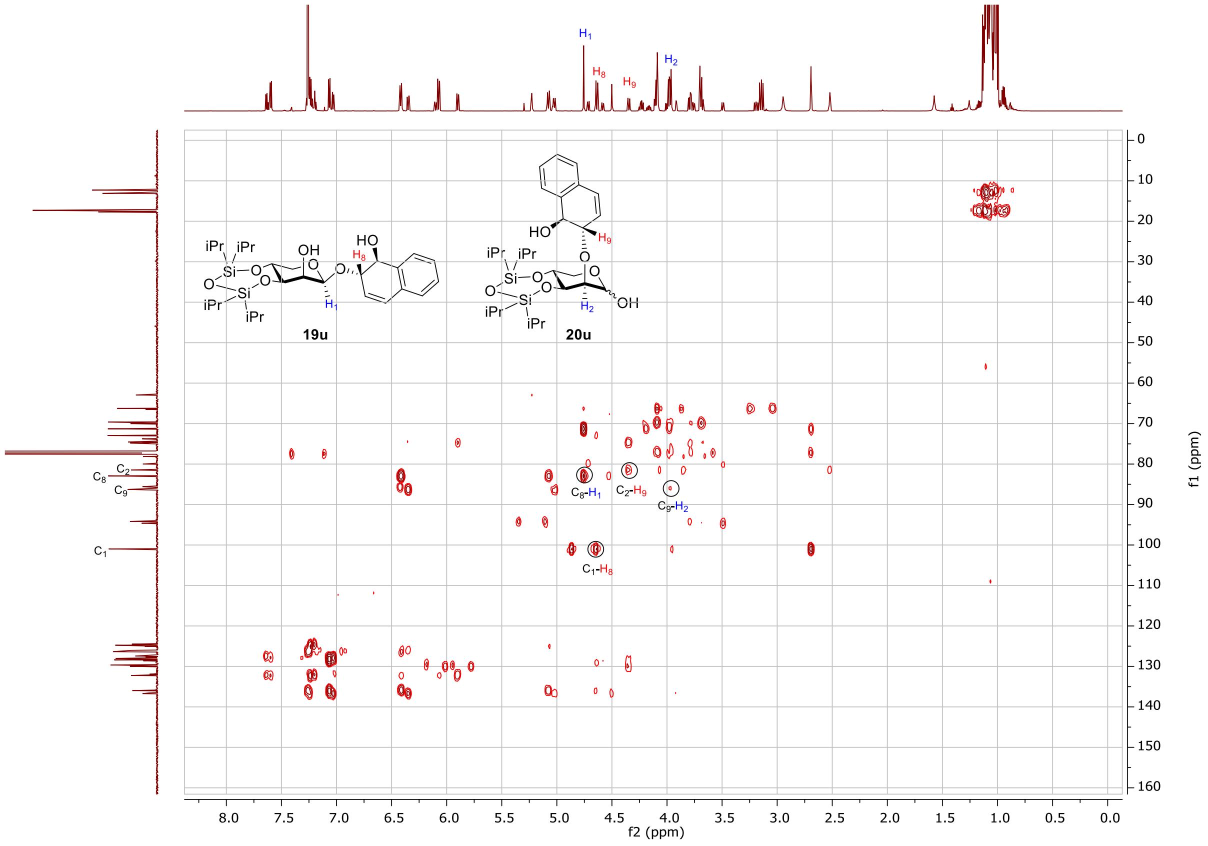

Supplementary Figure S300. HMBC spectra for **19u&20u**

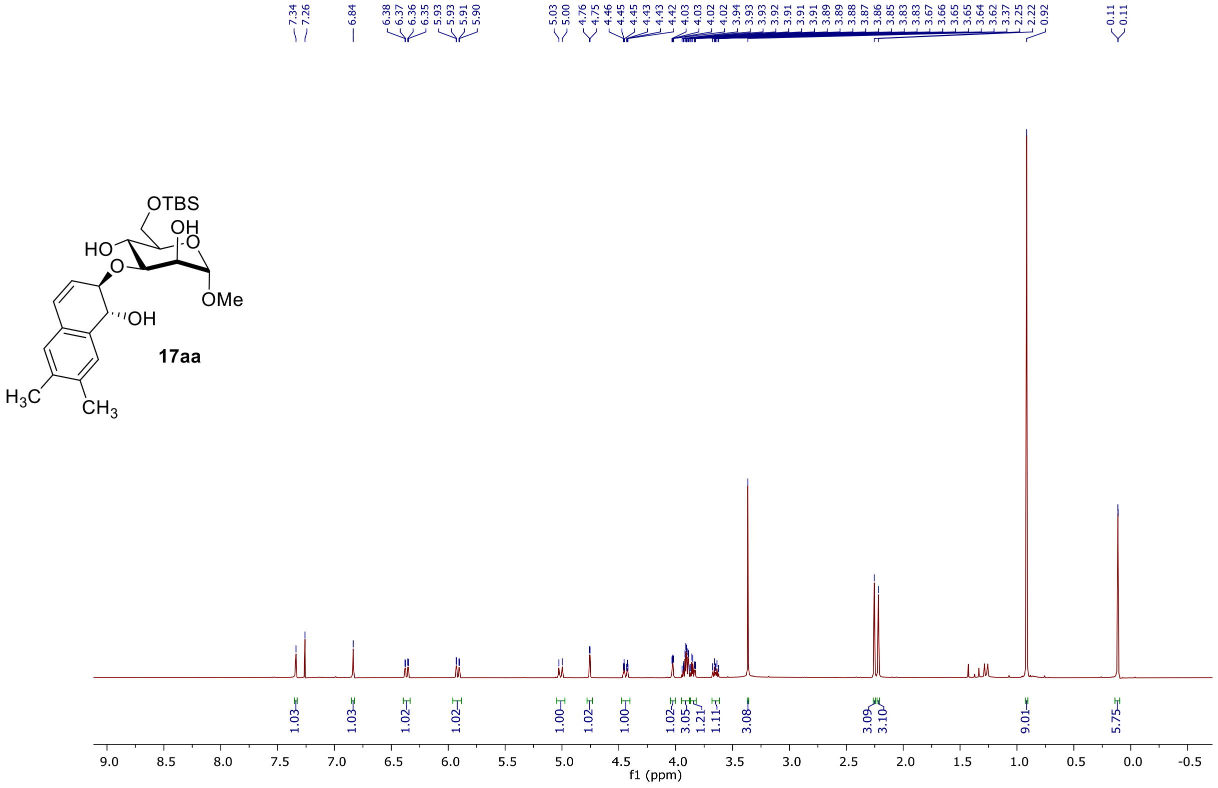

Supplementary Figure S301. ^{1}H spectra for **17aa**

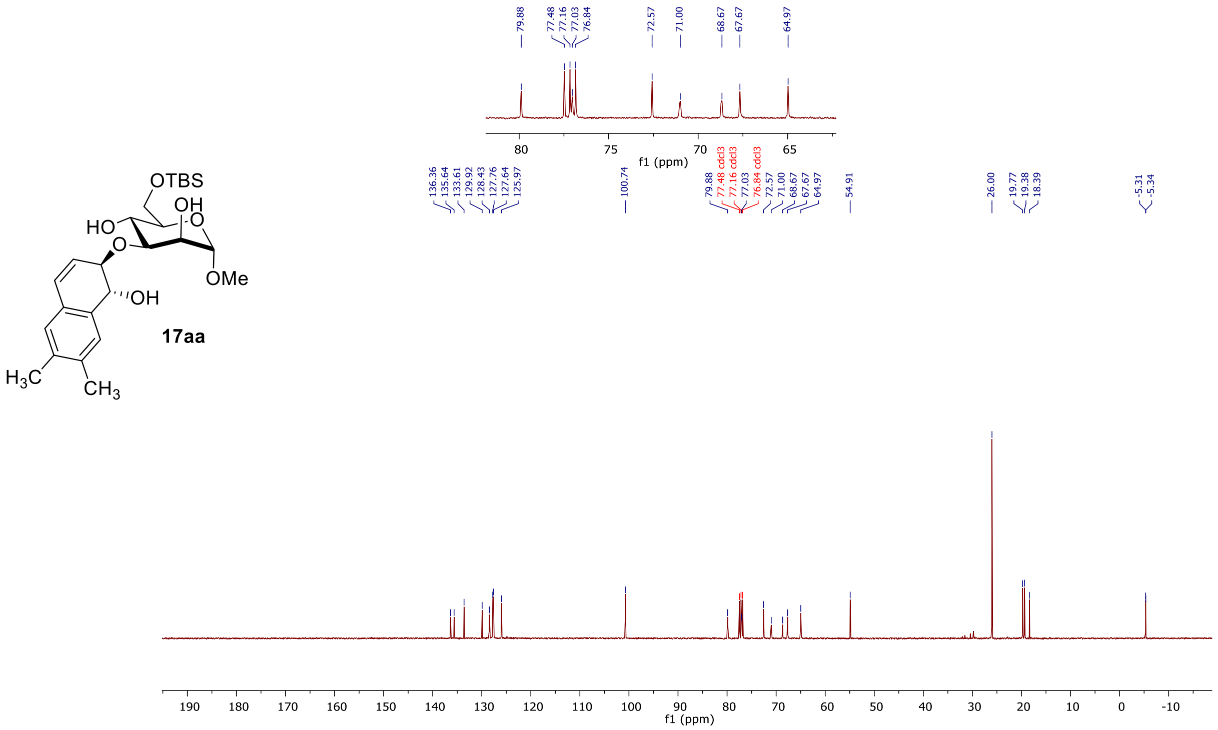

Supplementary Figure 302. ^{13}C spectra for **17aa**

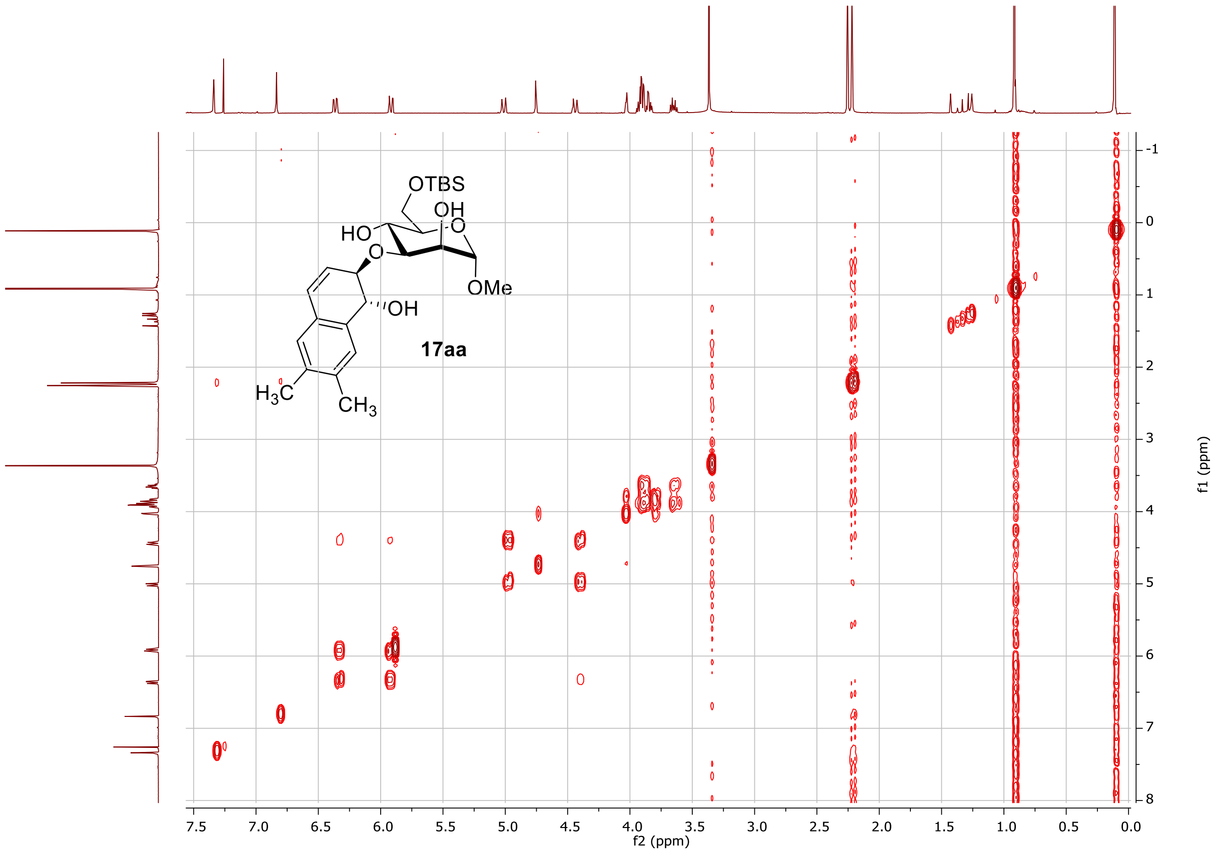

Supplementary Figure 303. COSY spectra for **17aa**

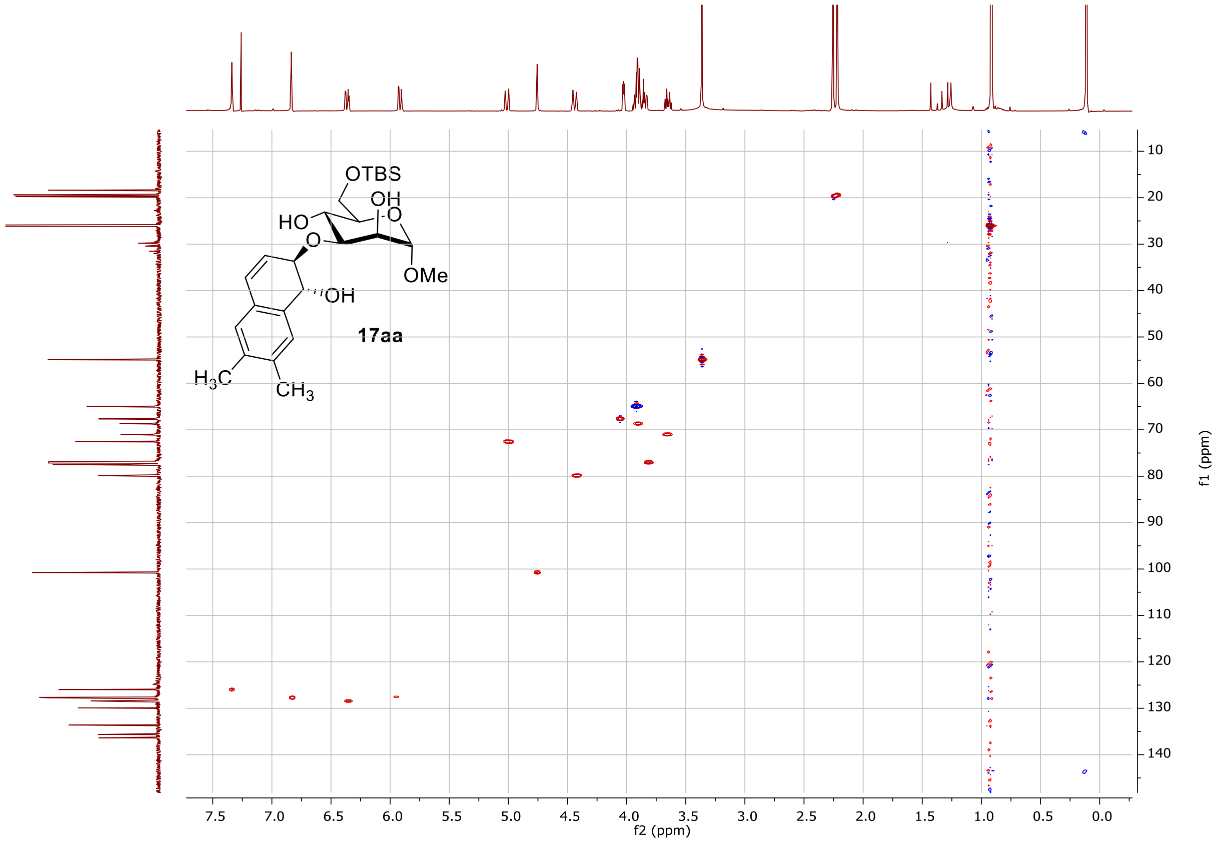

Supplementary Figure 304. HSQC spectra for **17aa**

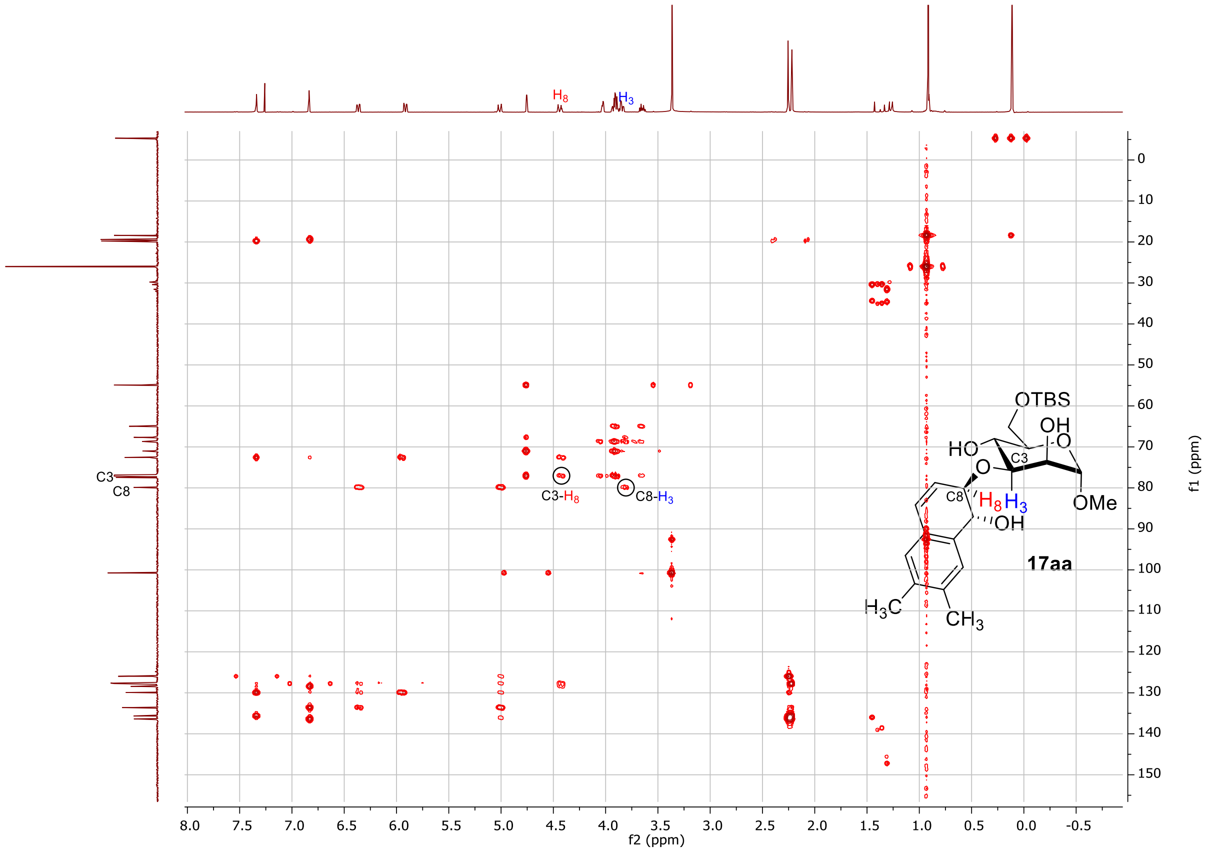

Supplementary Figure 305. HMBC spectra for **17aa**

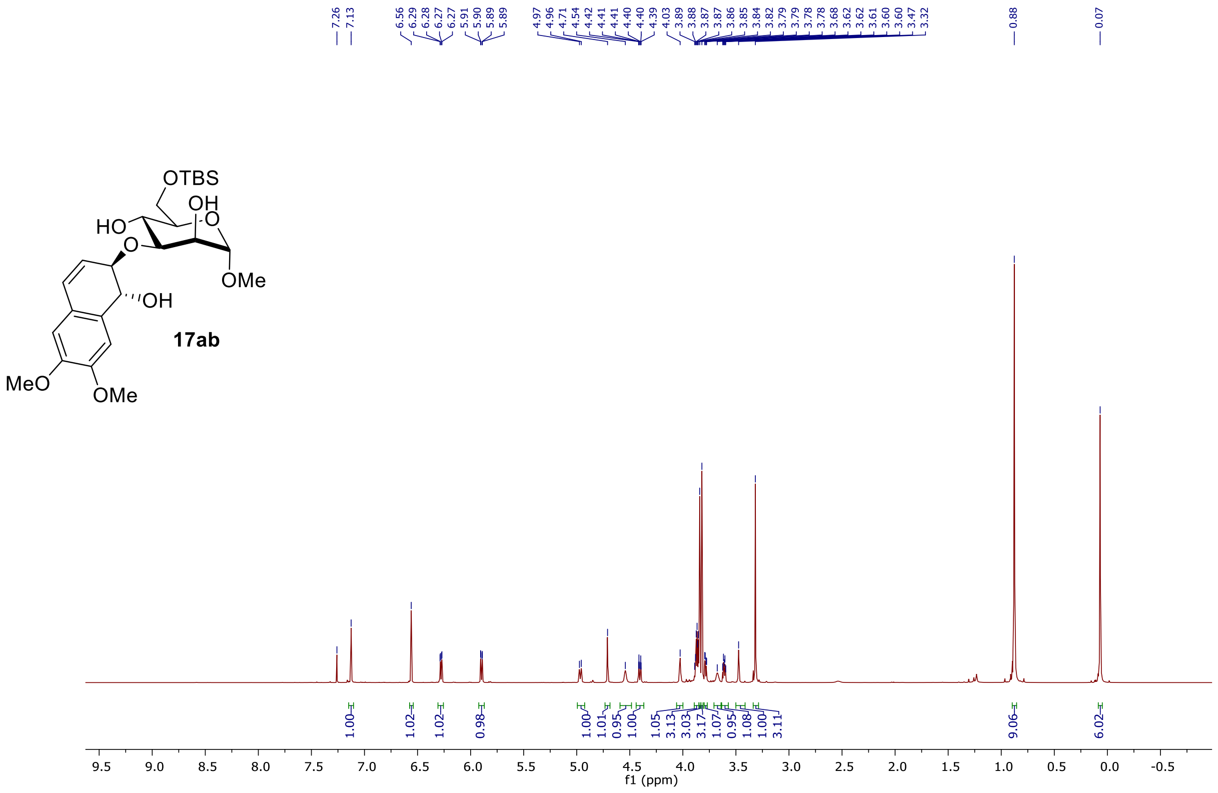

Supplementary Figure 306. ^{1}H spectra for **17ab**

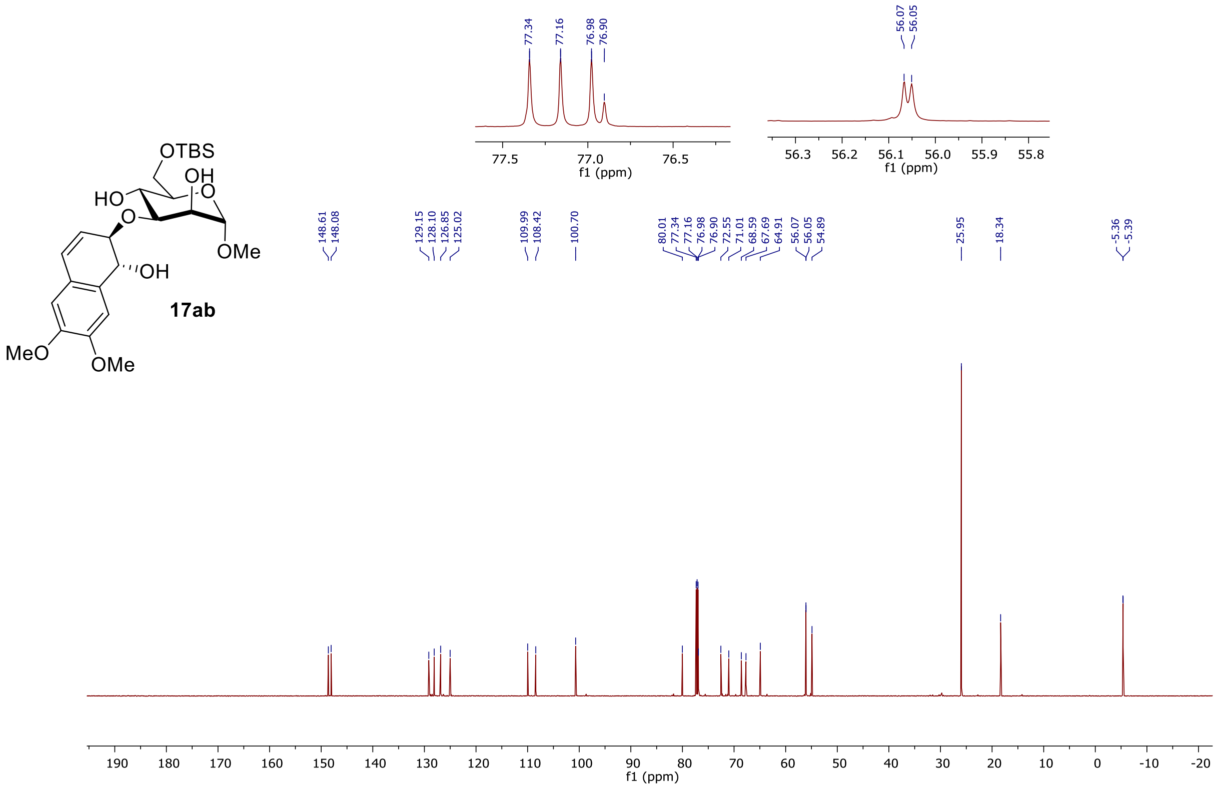

Supplementary Figure 307. ^{13}C spectra for **17ab**

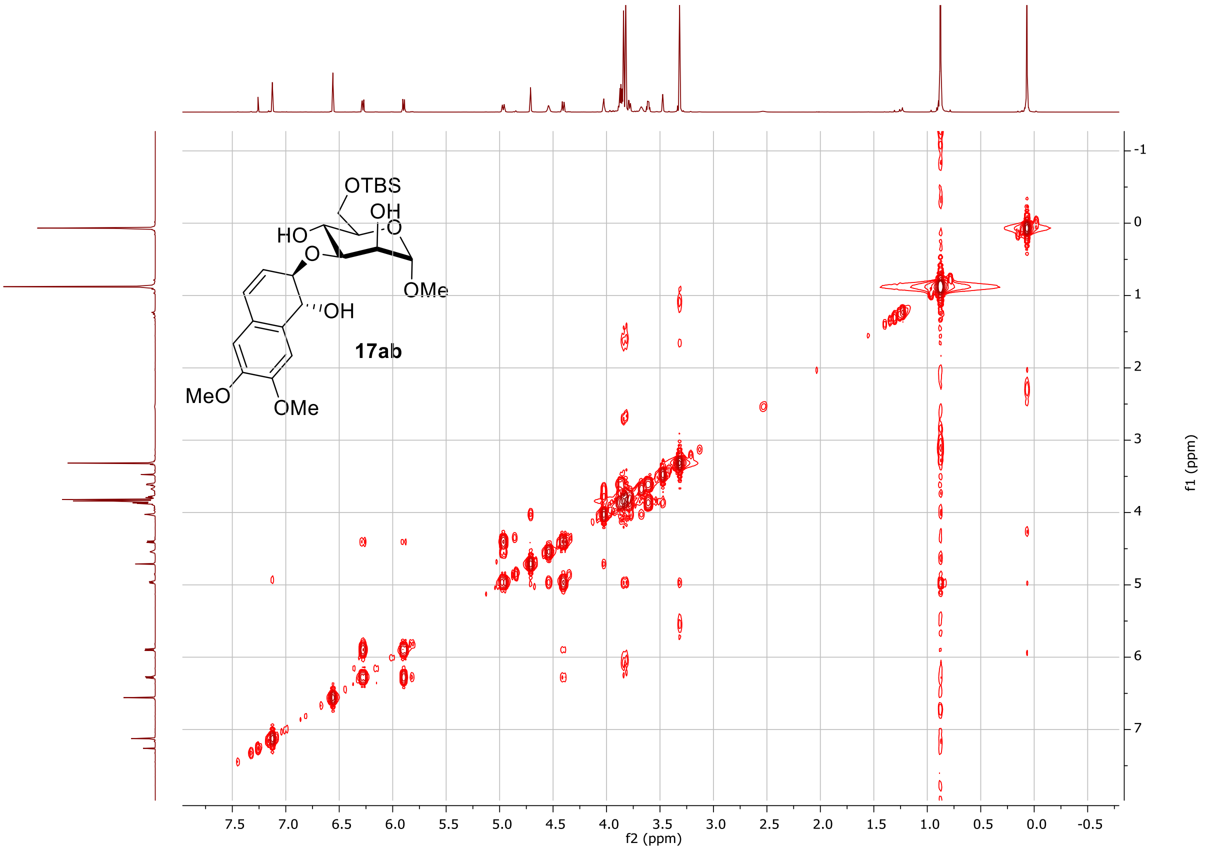

Supplementary Figure 308. COSY spectra for **17ab**

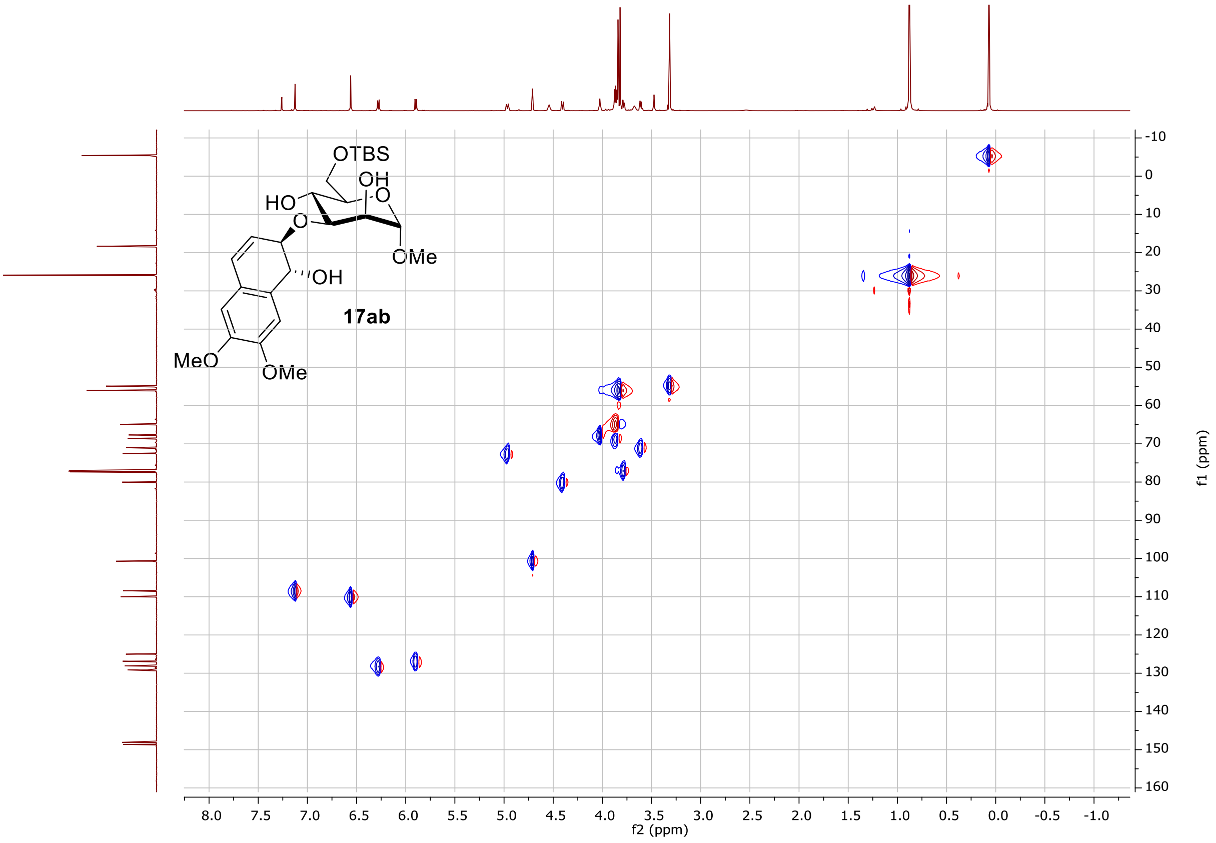

Supplementary Figure 309. HSQC spectra for **17a**

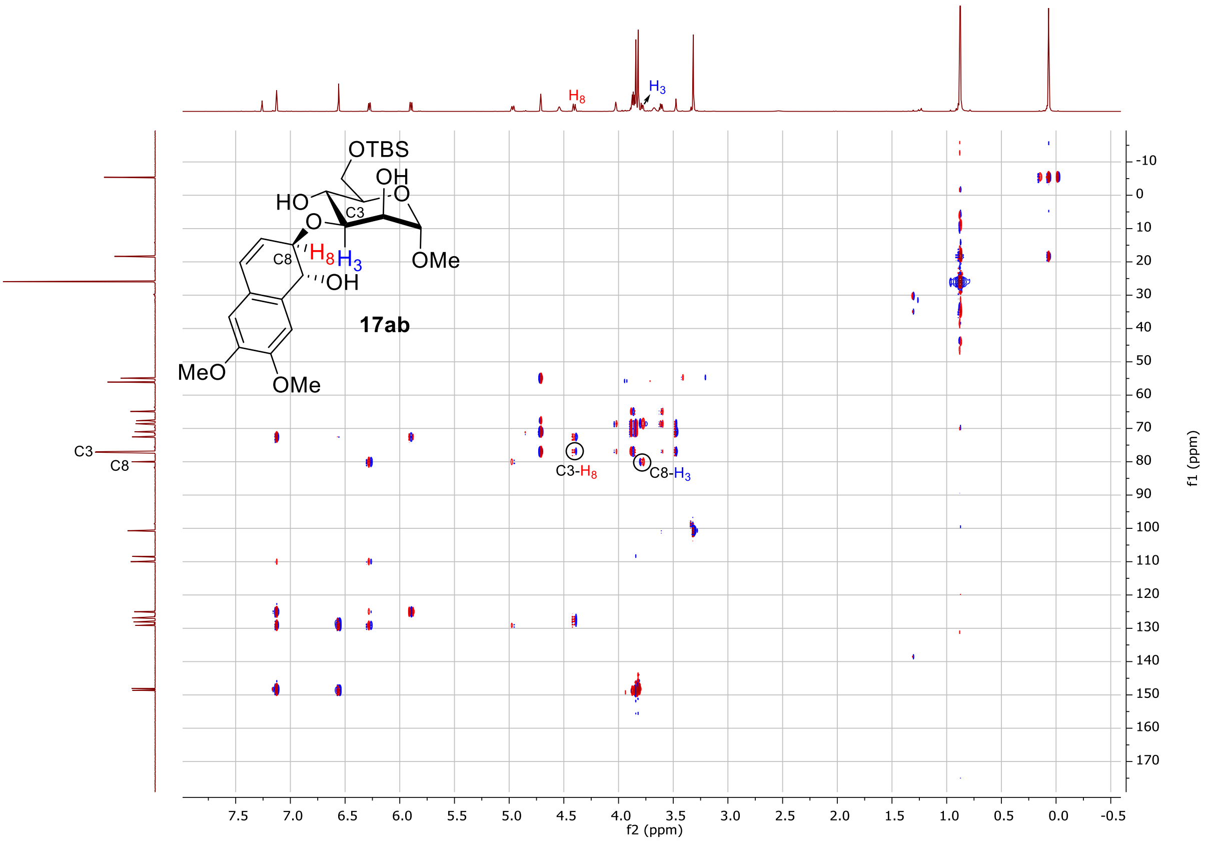

Supplementary Figure 310. HMBC spectra for **17ab**

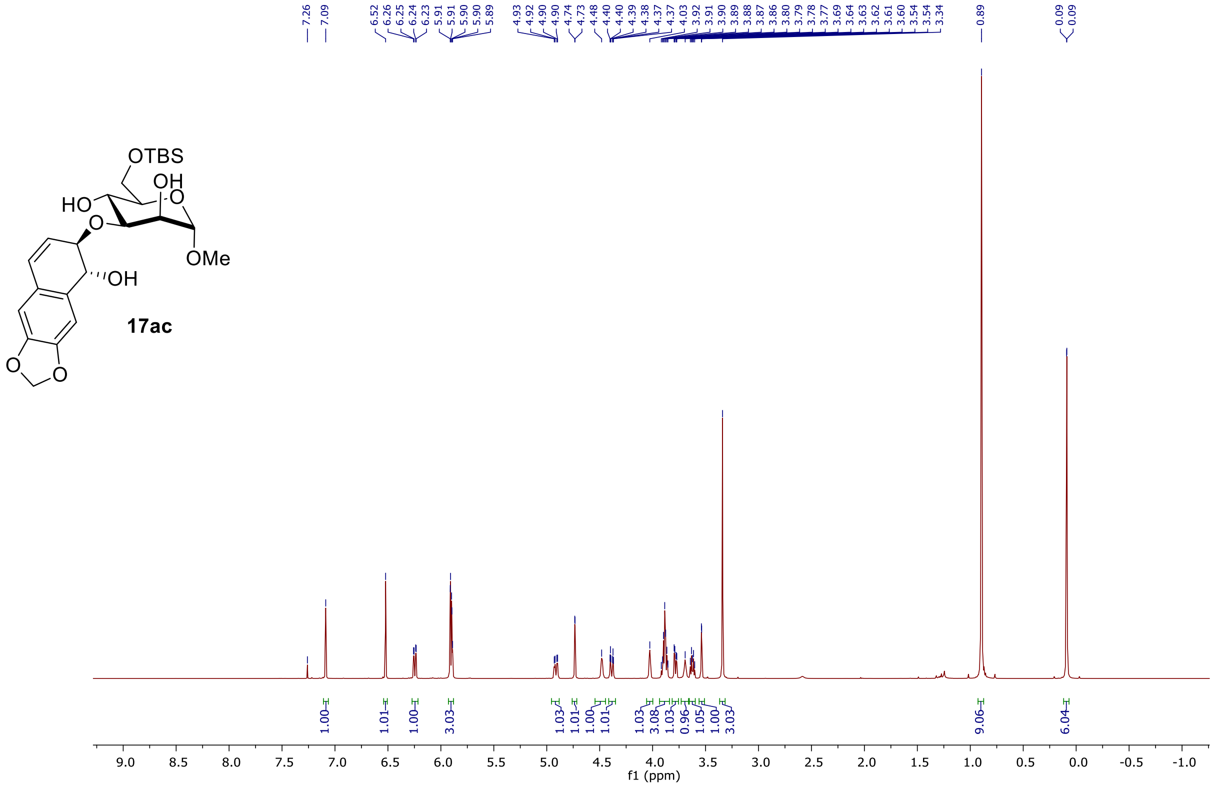

Supplementary Figure 311. ^{1}H spectra for **17ac**

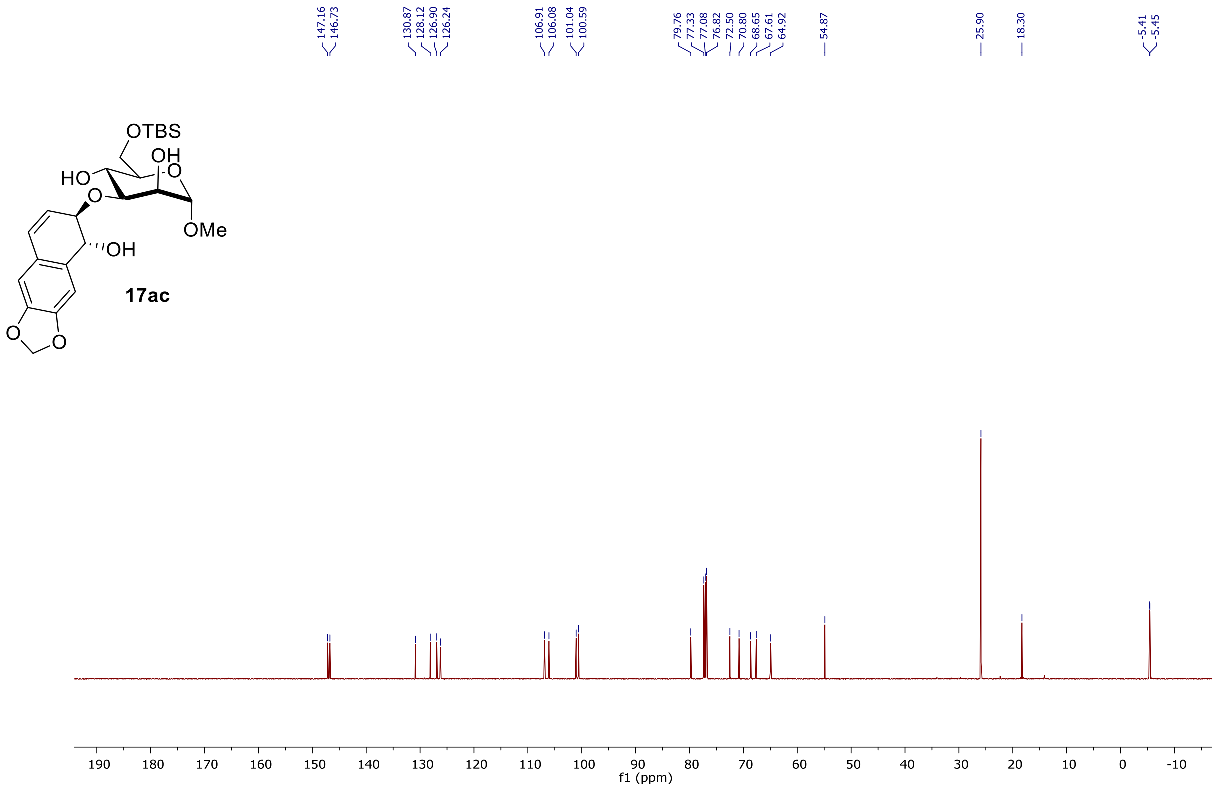

Supplementary Figure 312. ^{13}C spectra for **17ac**

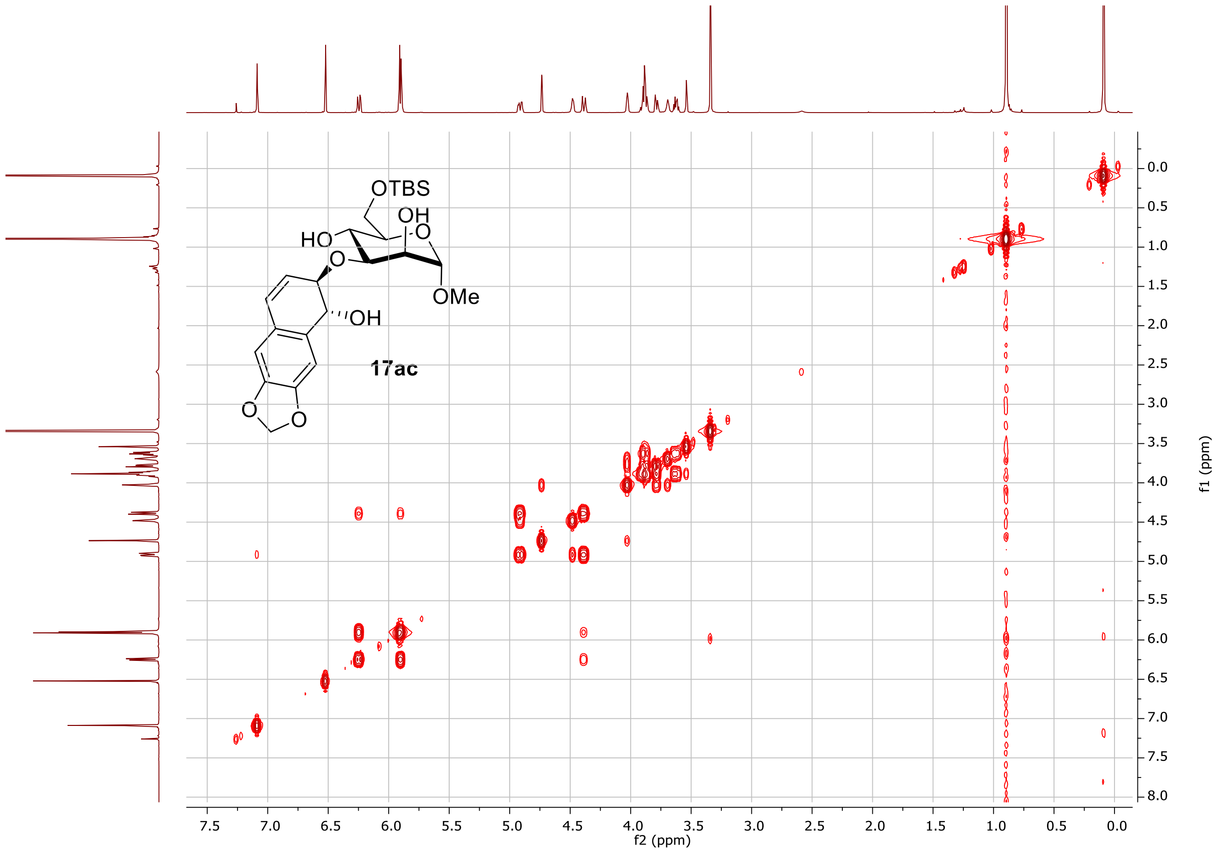

Supplementary Figure 313. COSY spectra for **17ac**

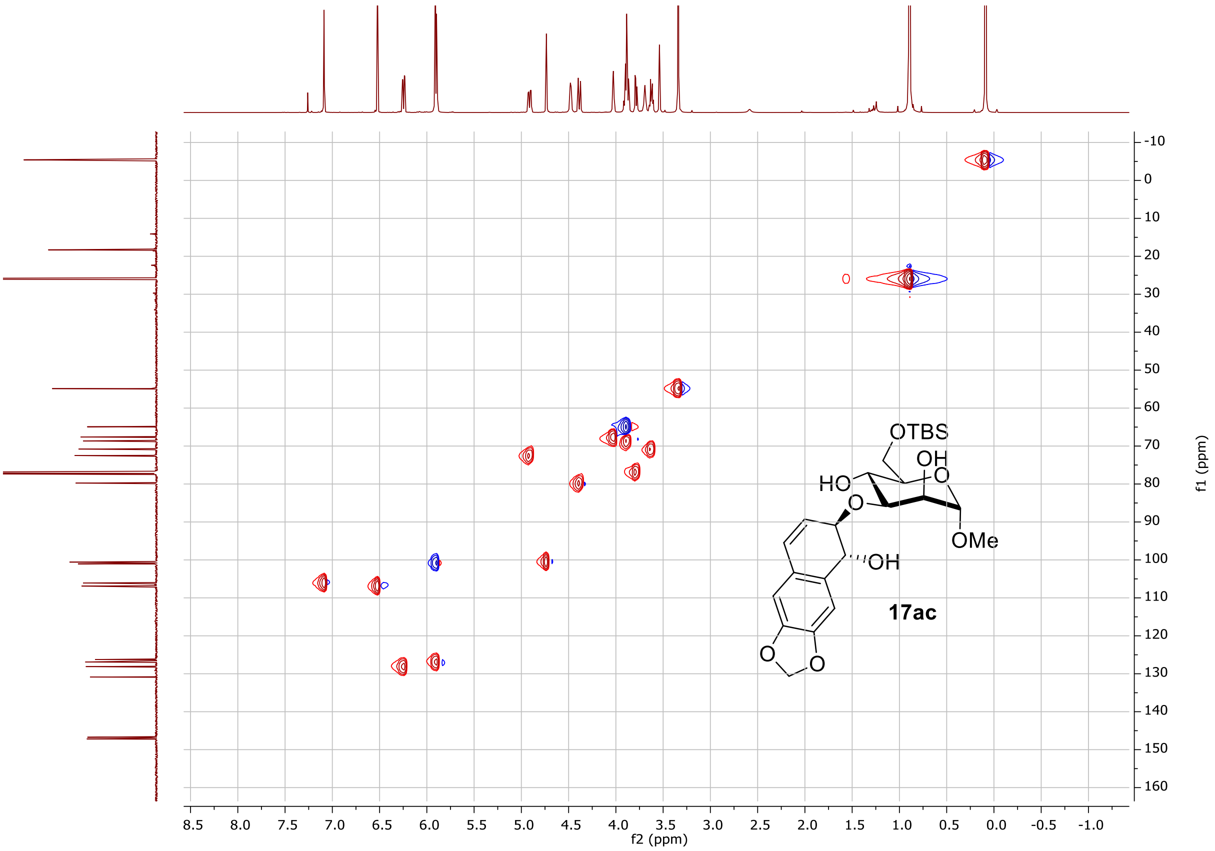

Supplementary Figure 314. HSQC spectra for **17ac**

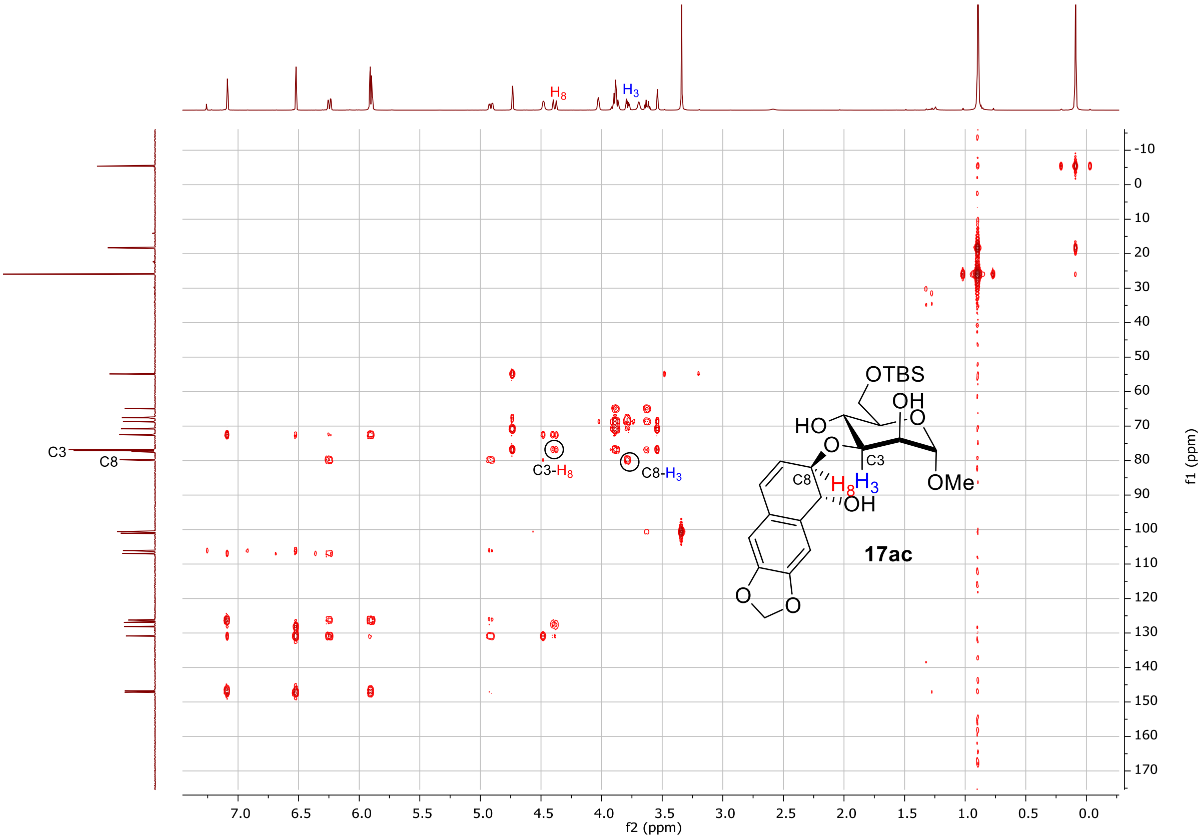

Supplementary Figure 315. HMBC spectra for **17ac**

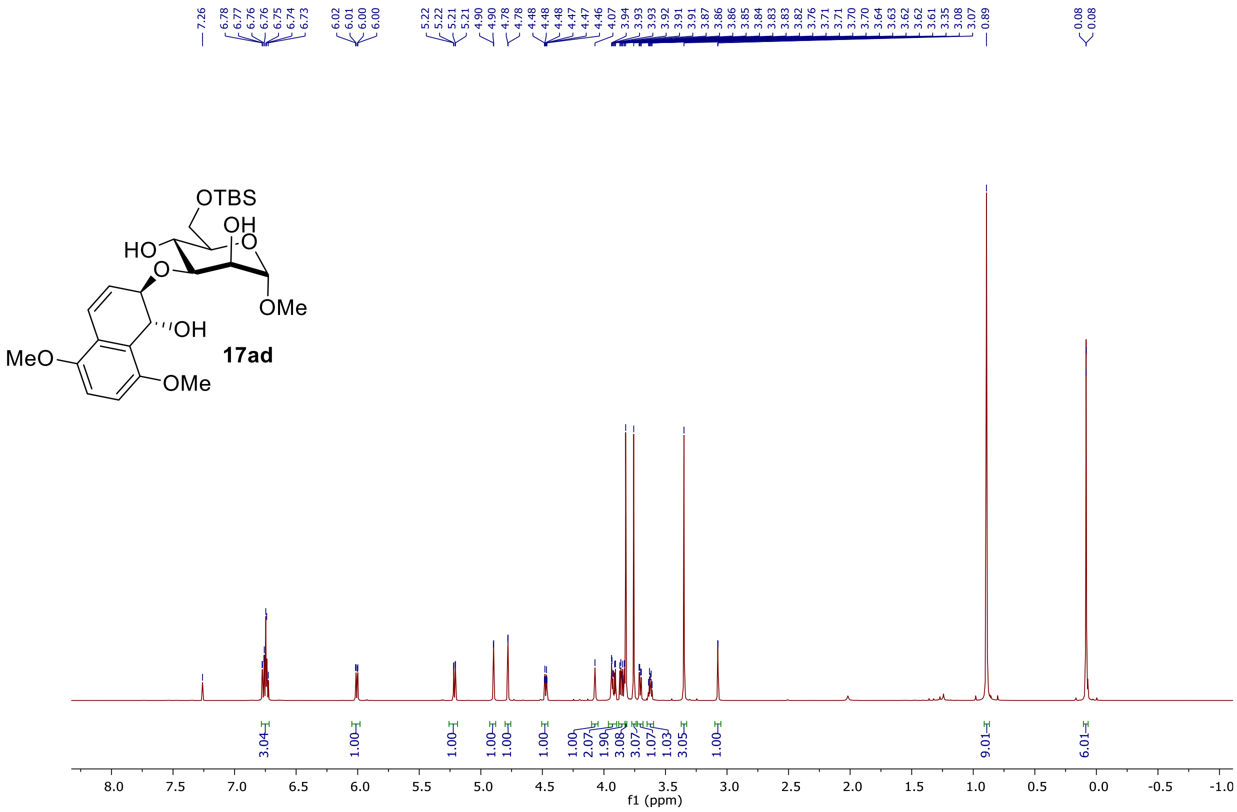

Supplementary Figure 316. ^{1}H spectra for **17ad**

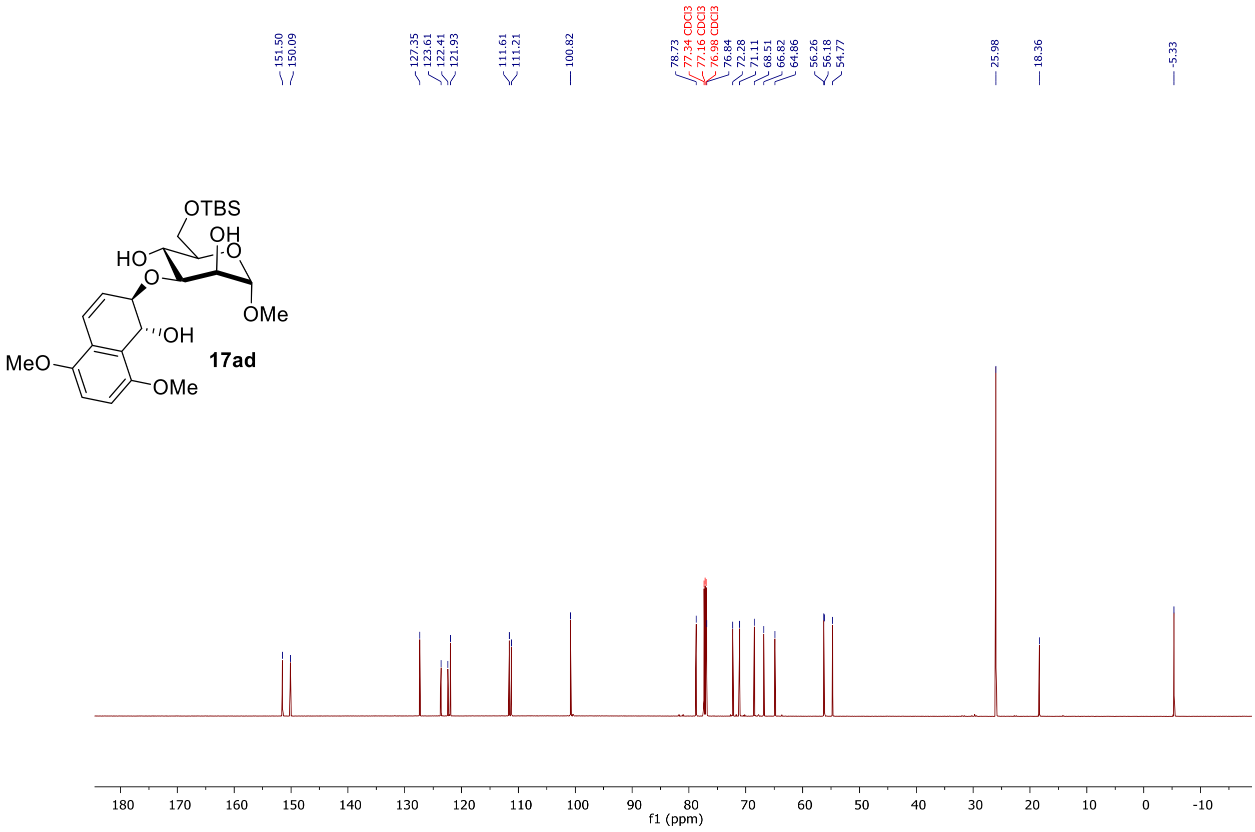

Supplementary Figure 317. ^{13}C spectra for **17ad**

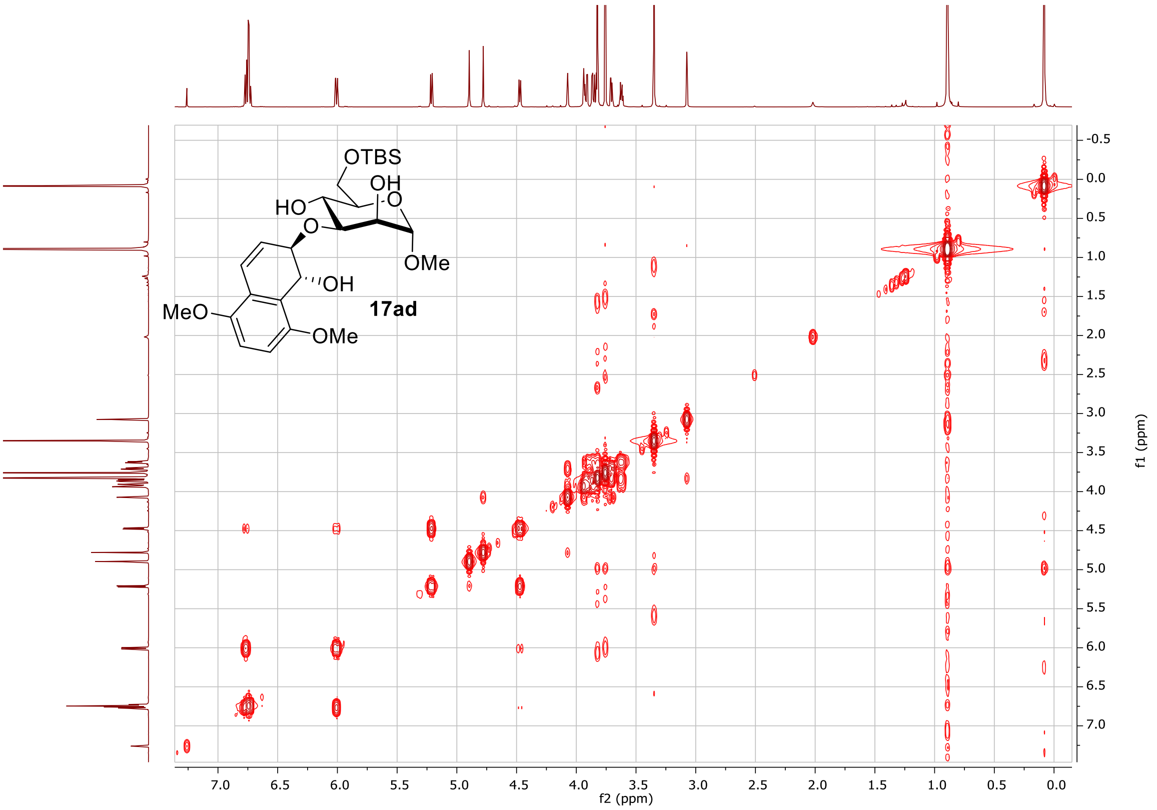

Supplementary Figure 318. COSY spectra for **17ad**

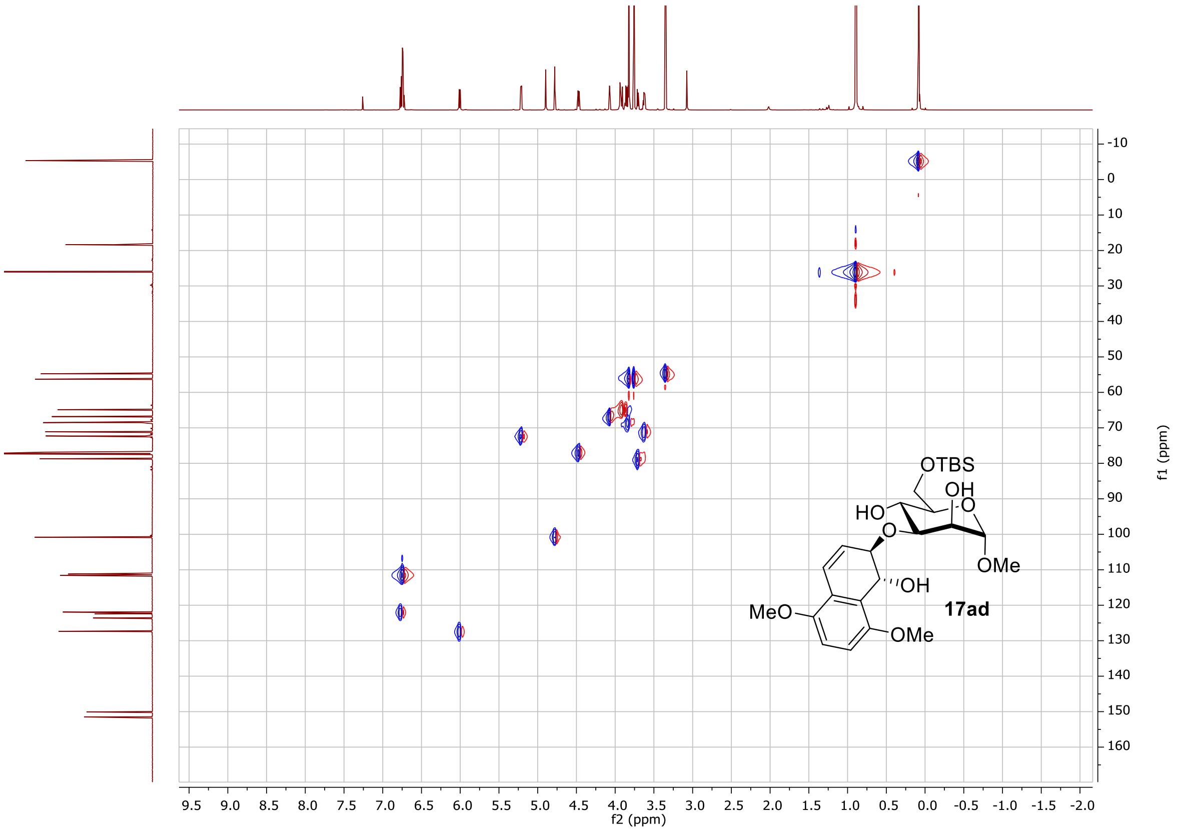

Supplementary Figure 319. HSQC spectra for **17ad**

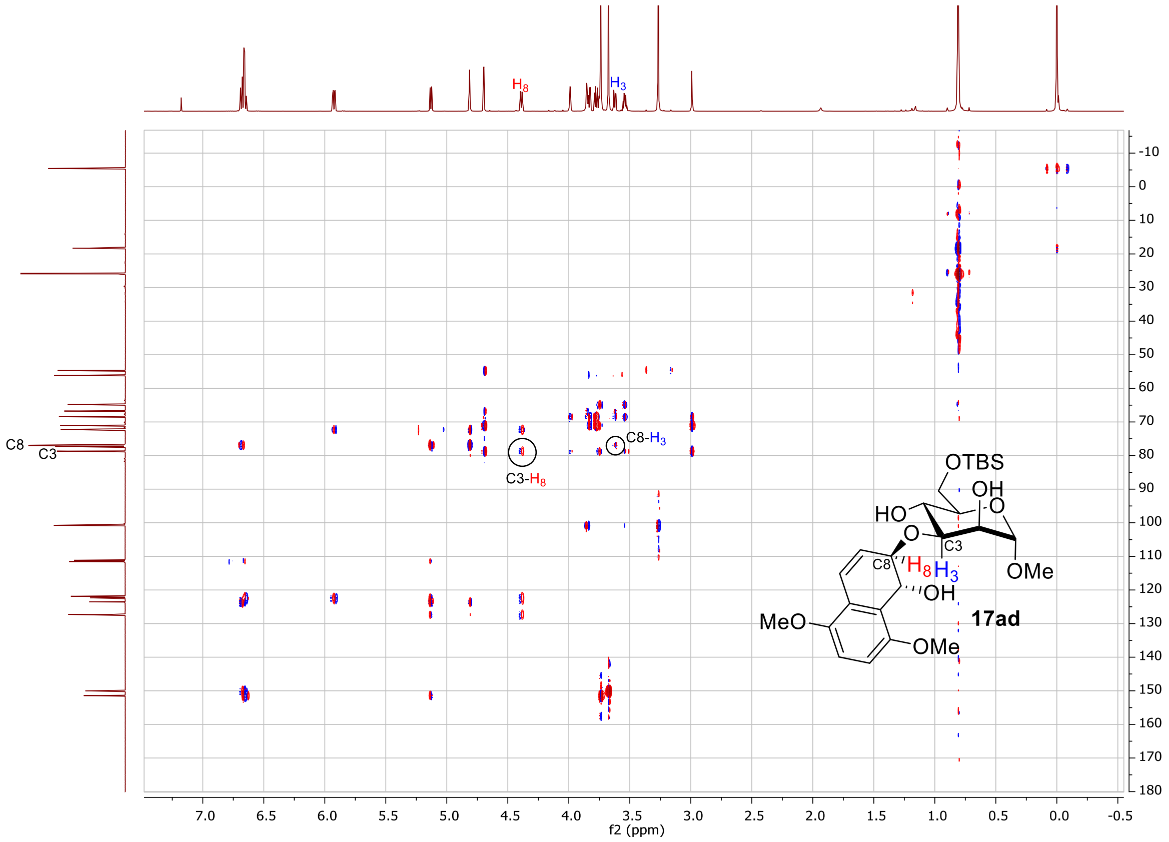

Supplementary Figure 320. HMBC spectra for **17ad**

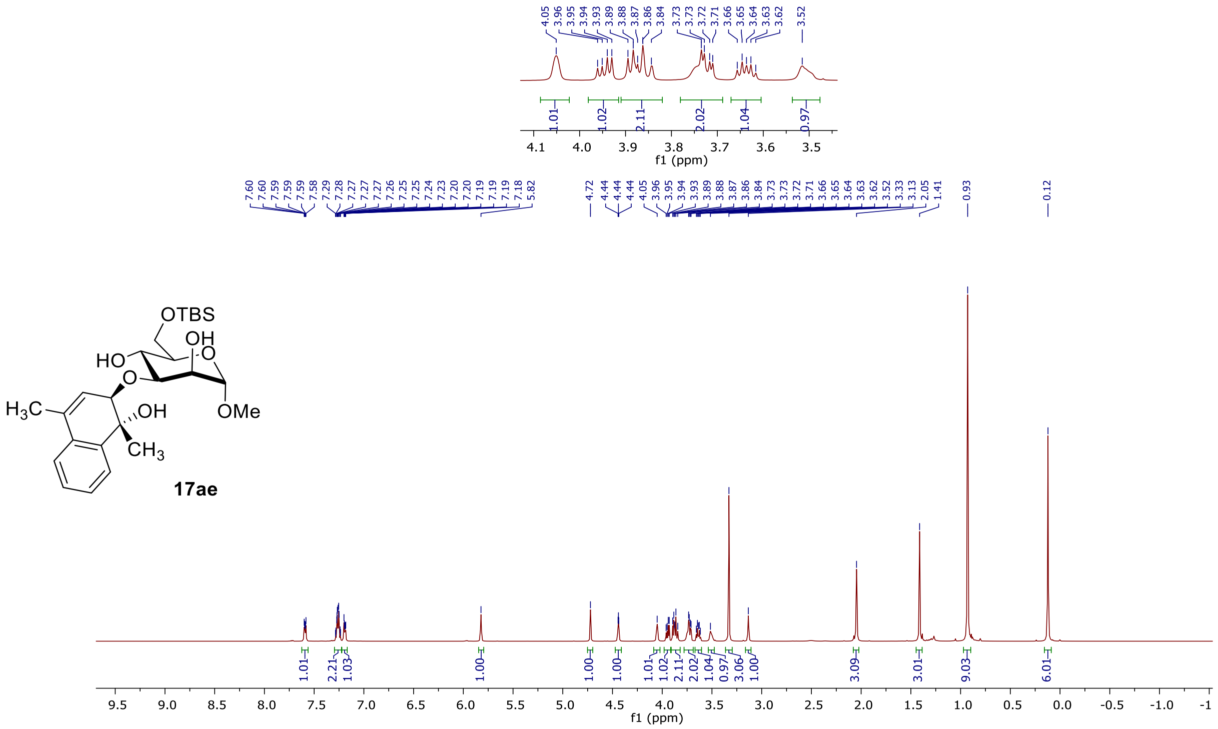

Supplementary Figure 321. ^{1}H spectra for **17ae**

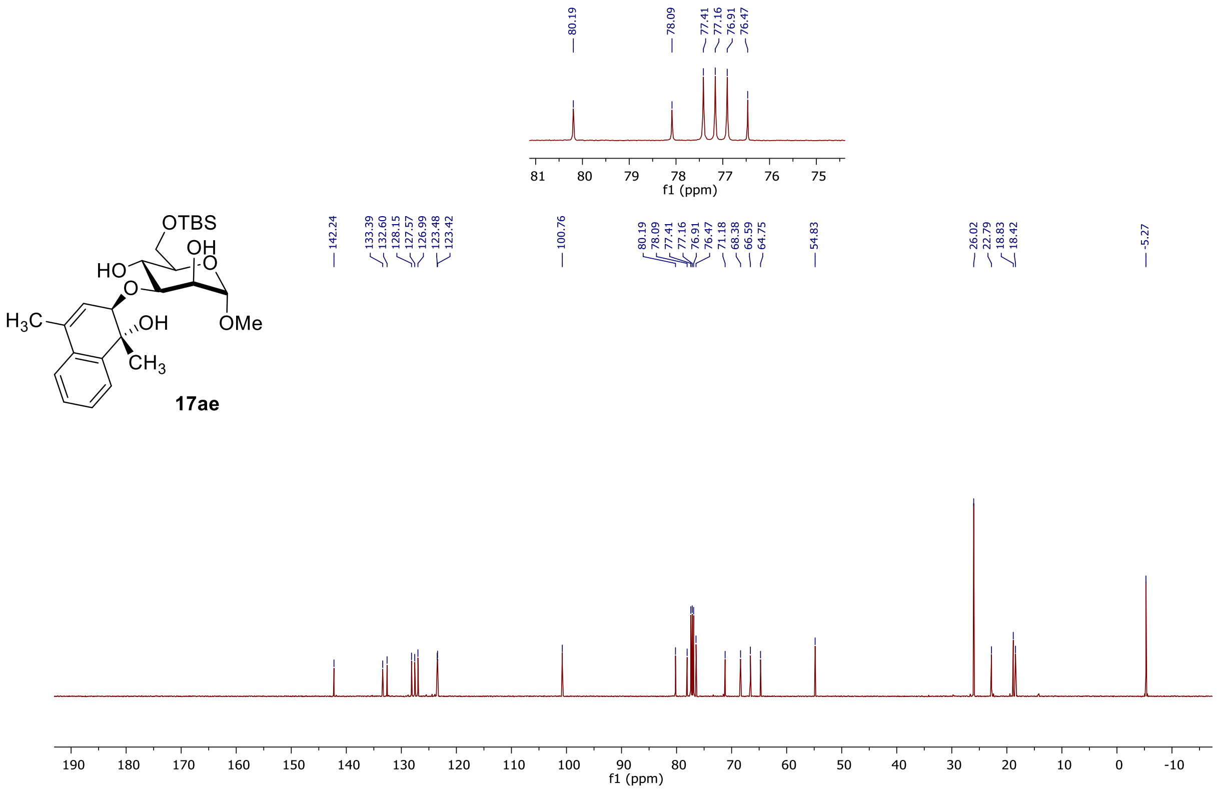

Supplementary Figure 322. ^{13}C spectra for **17ae**

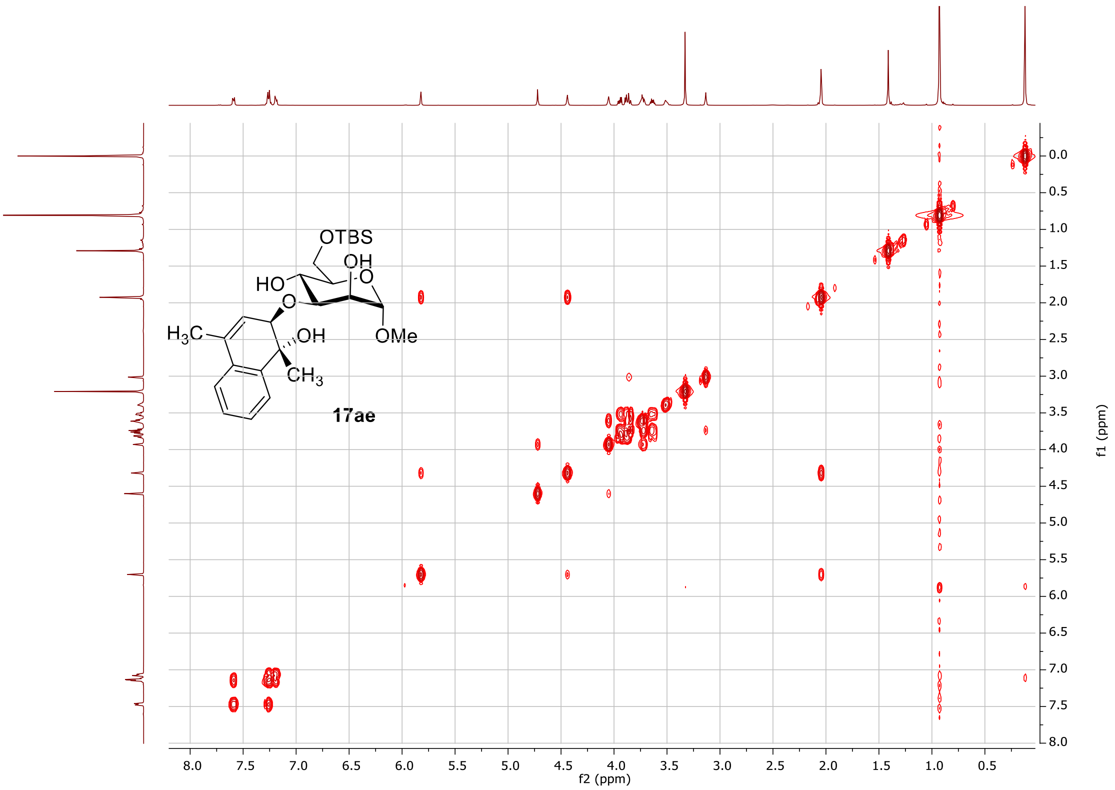

Supplementary Figure 323. COSY spectra for **17ae**

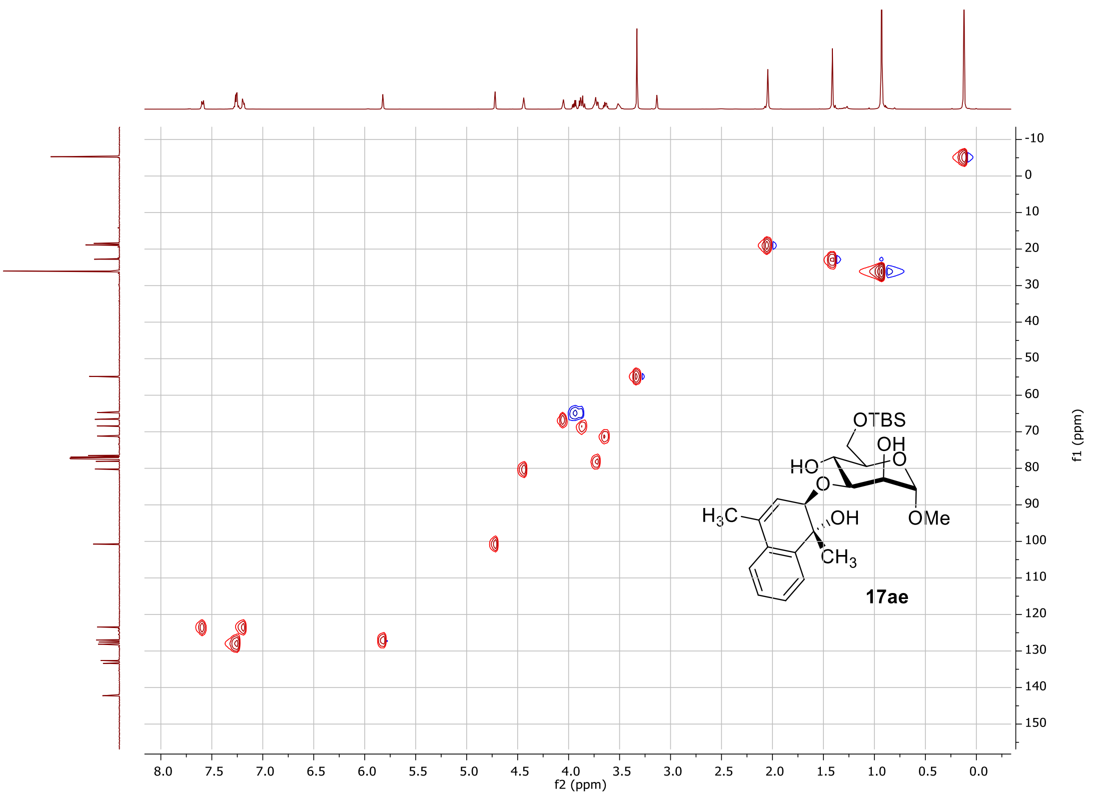

Supplementary Figure 324. HSQC spectra for **17ae**

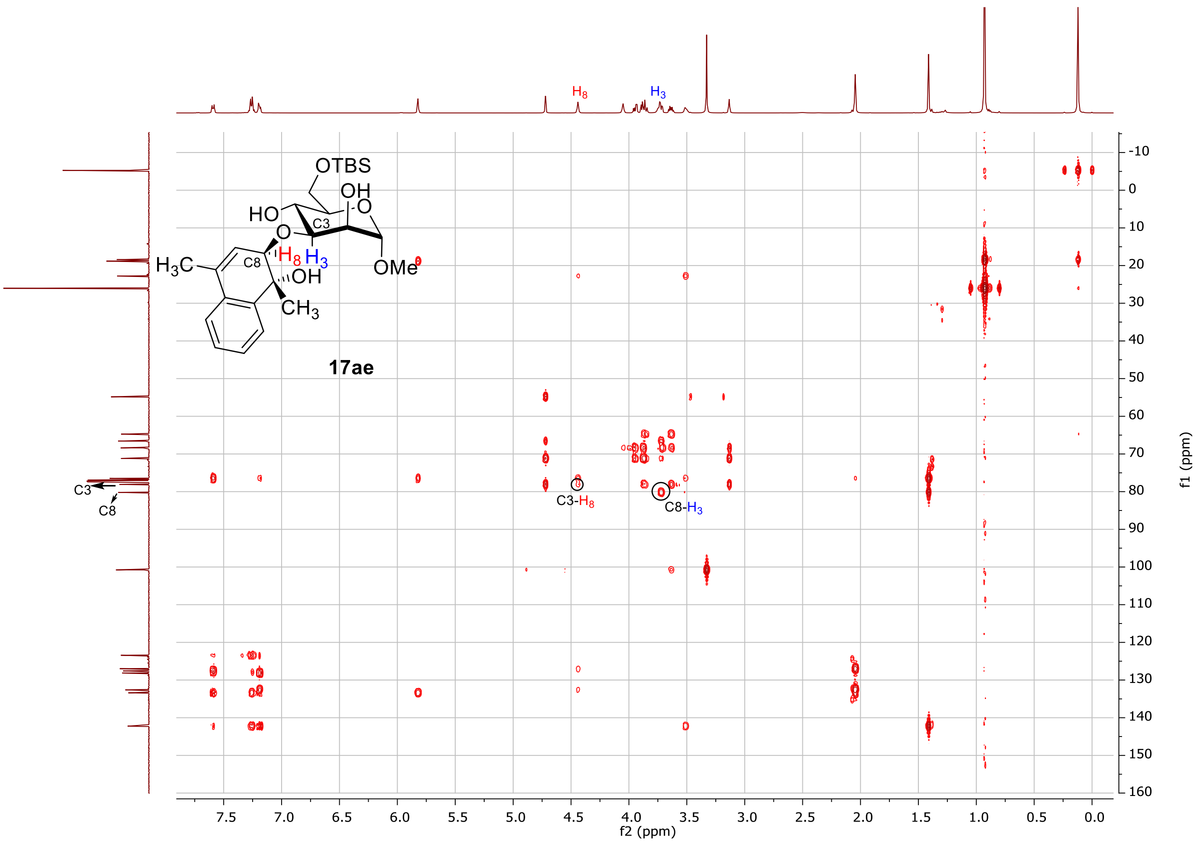

Supplementary Figure 325. HMBC spectra for **17ae**

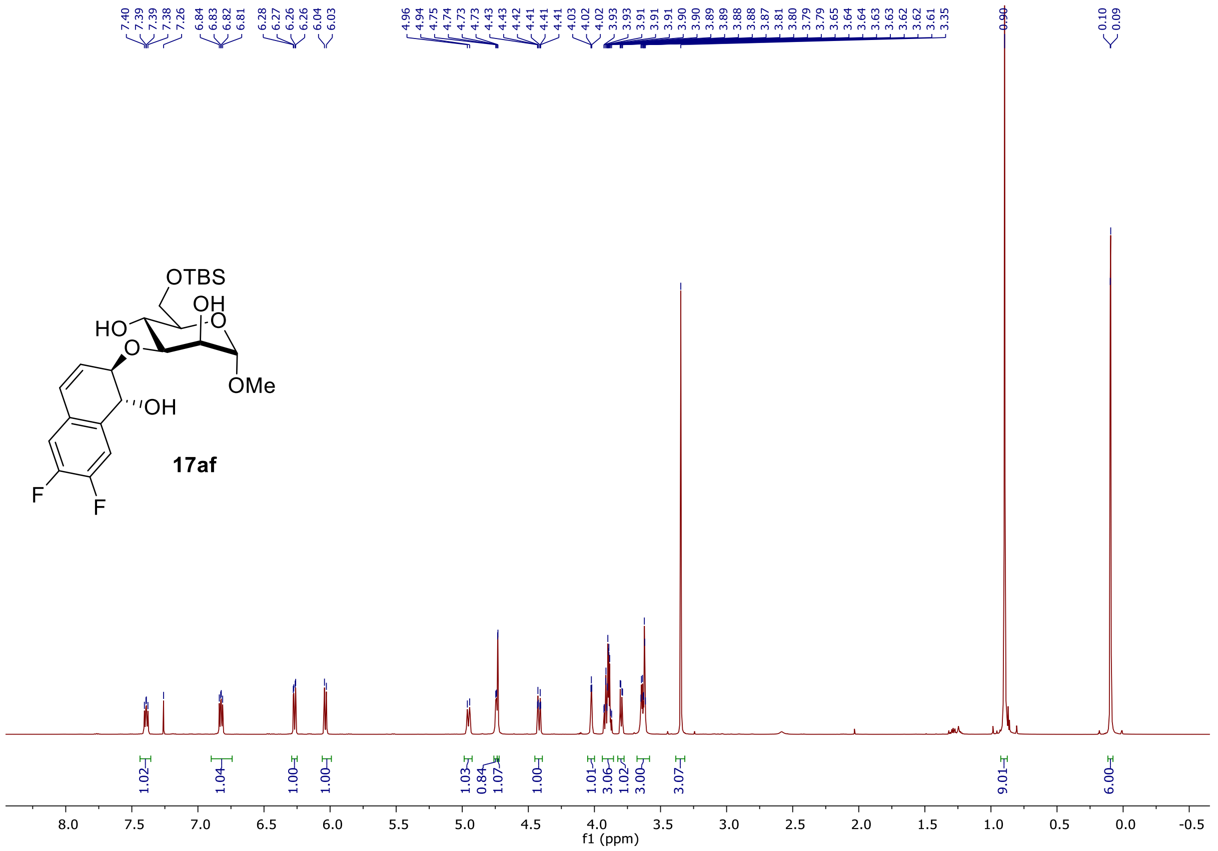

Supplementary Figure 326. ^{1}H spectra for **17af**

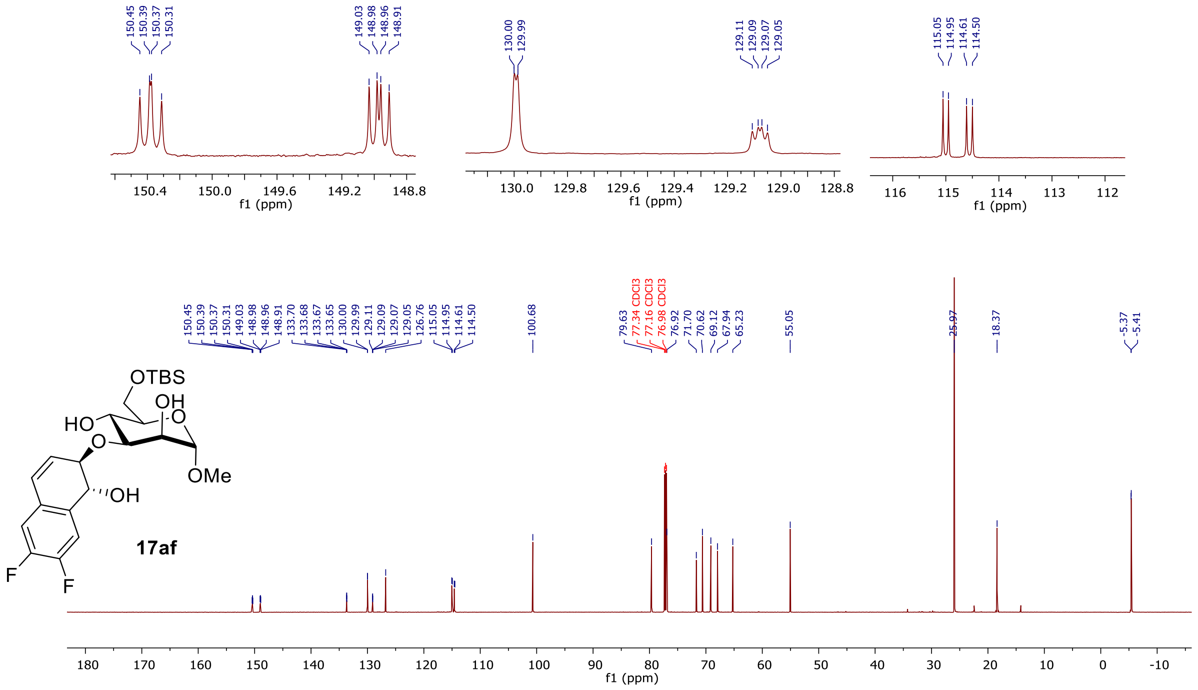

Supplementary Figure 327. ^{13}C spectra for **17af**

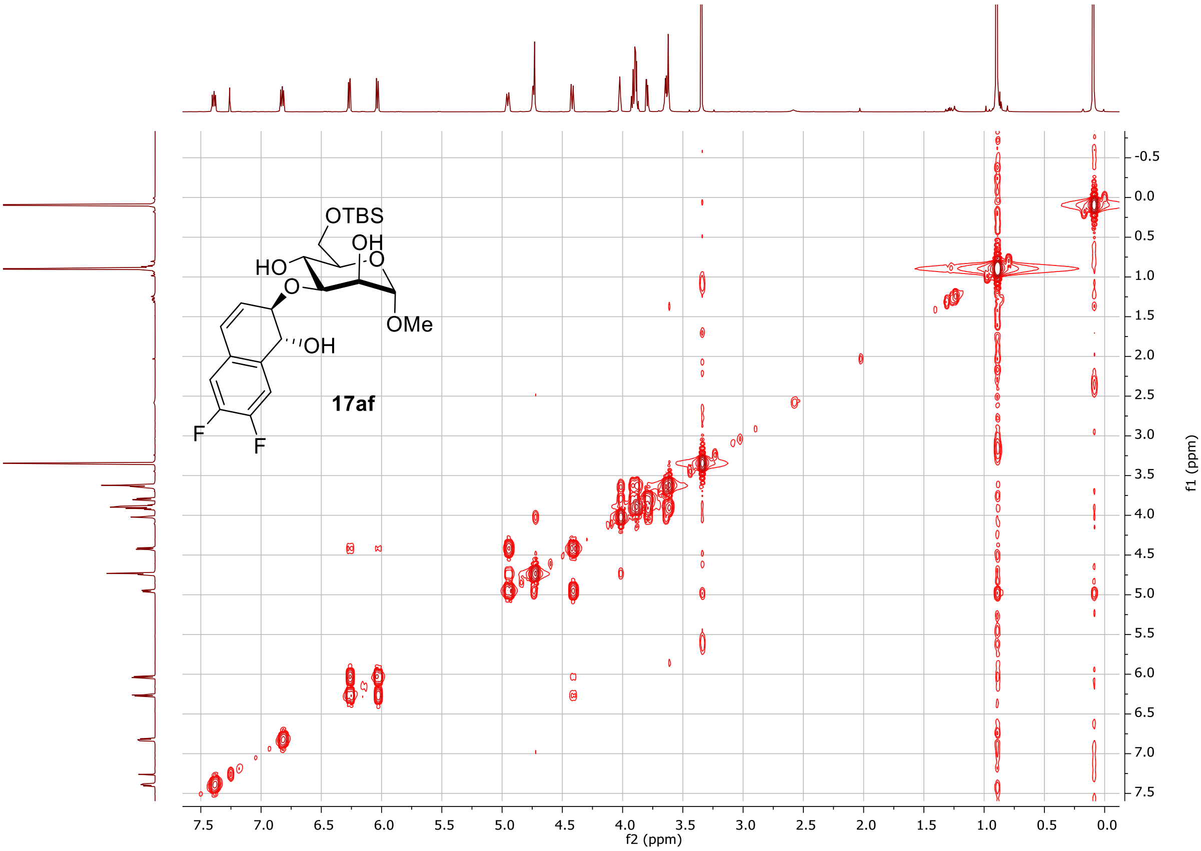

Supplementary Figure 328. COSY spectra for **17af**

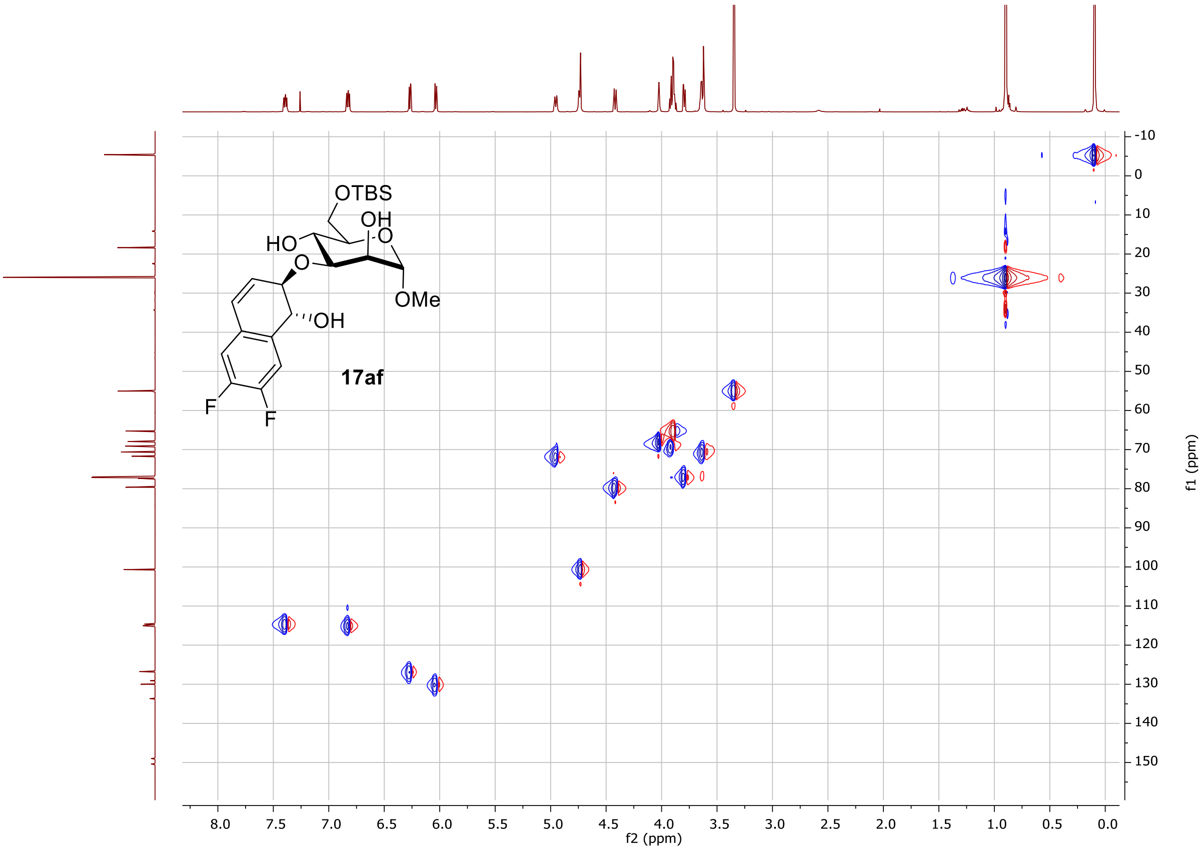

Supplementary Figure 329. HSQC spectra for **17af**

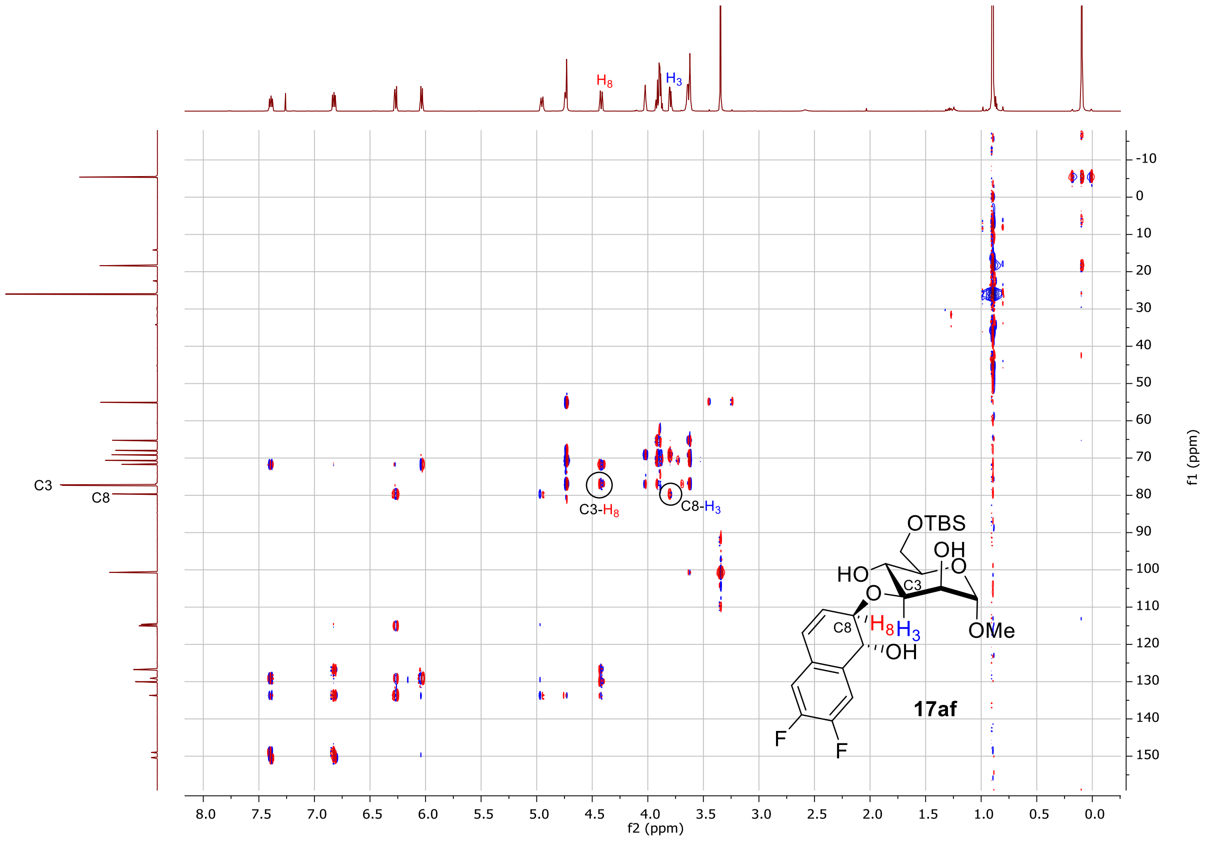

Supplementary Figure 330. HMBC spectra for **17af**

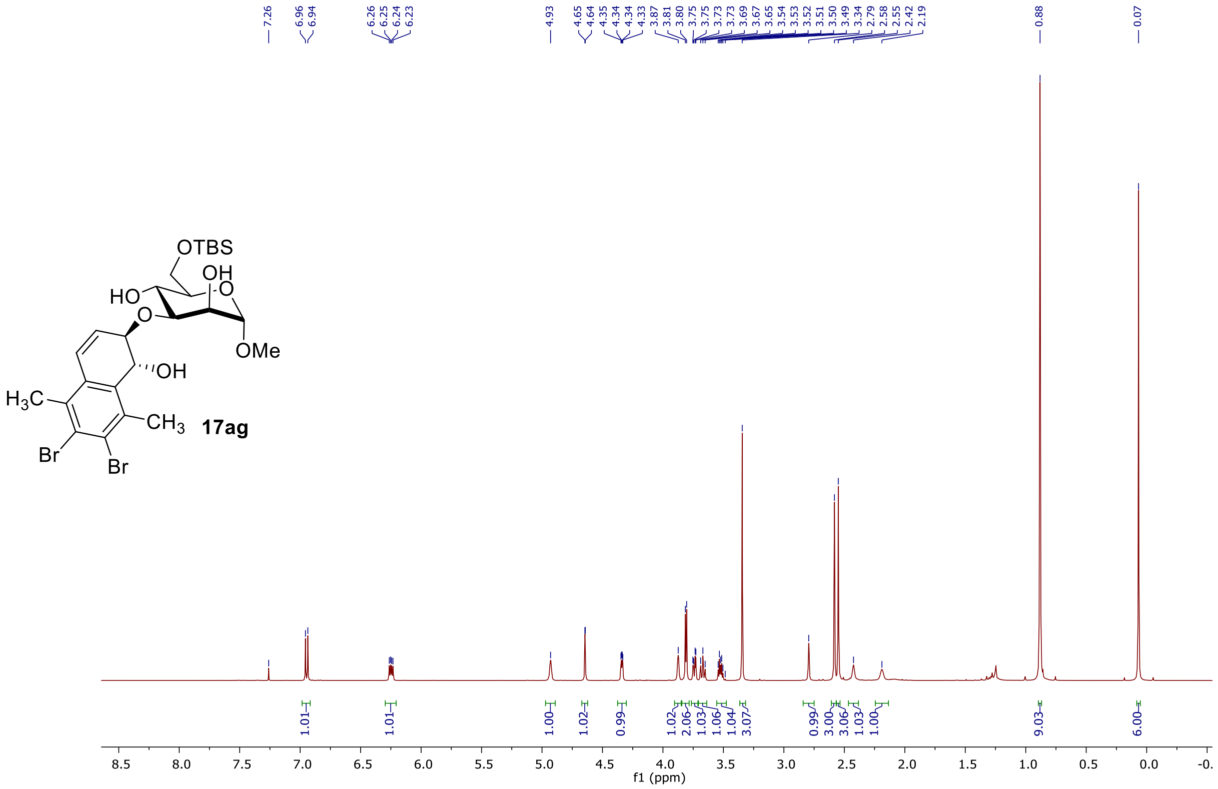

Supplementary Figure 331. ^{1}H spectra for **17ag**

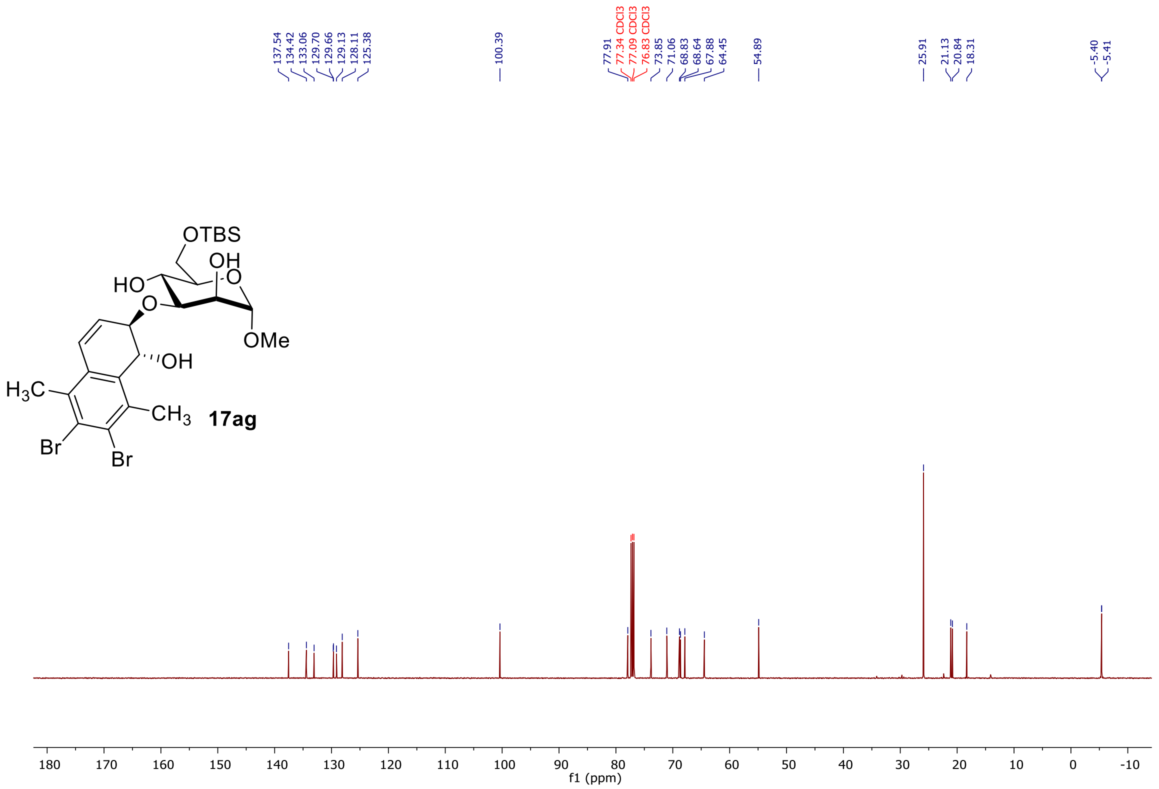

Supplementary Figure 332. ^{13}C spectra for **17ag**

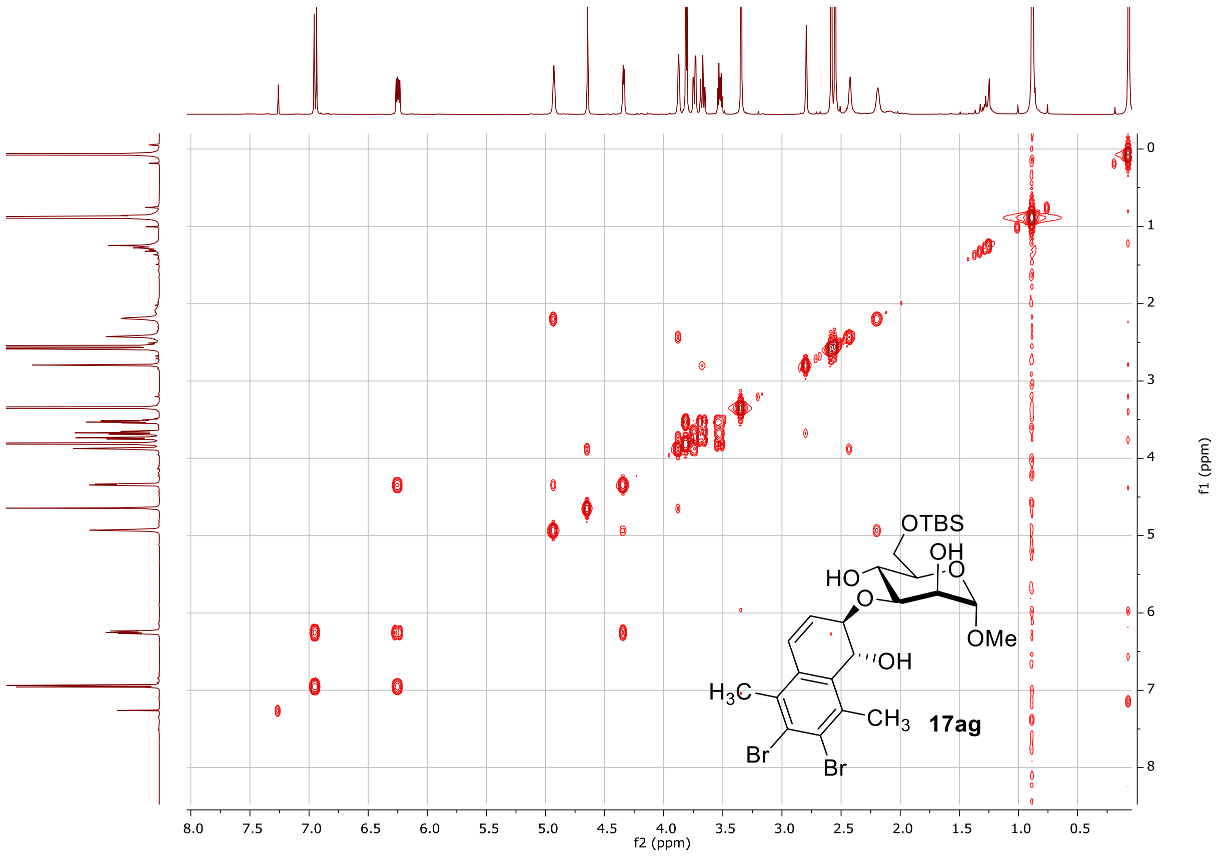

Supplementary Figure 333. COSY spectra for **17ag**

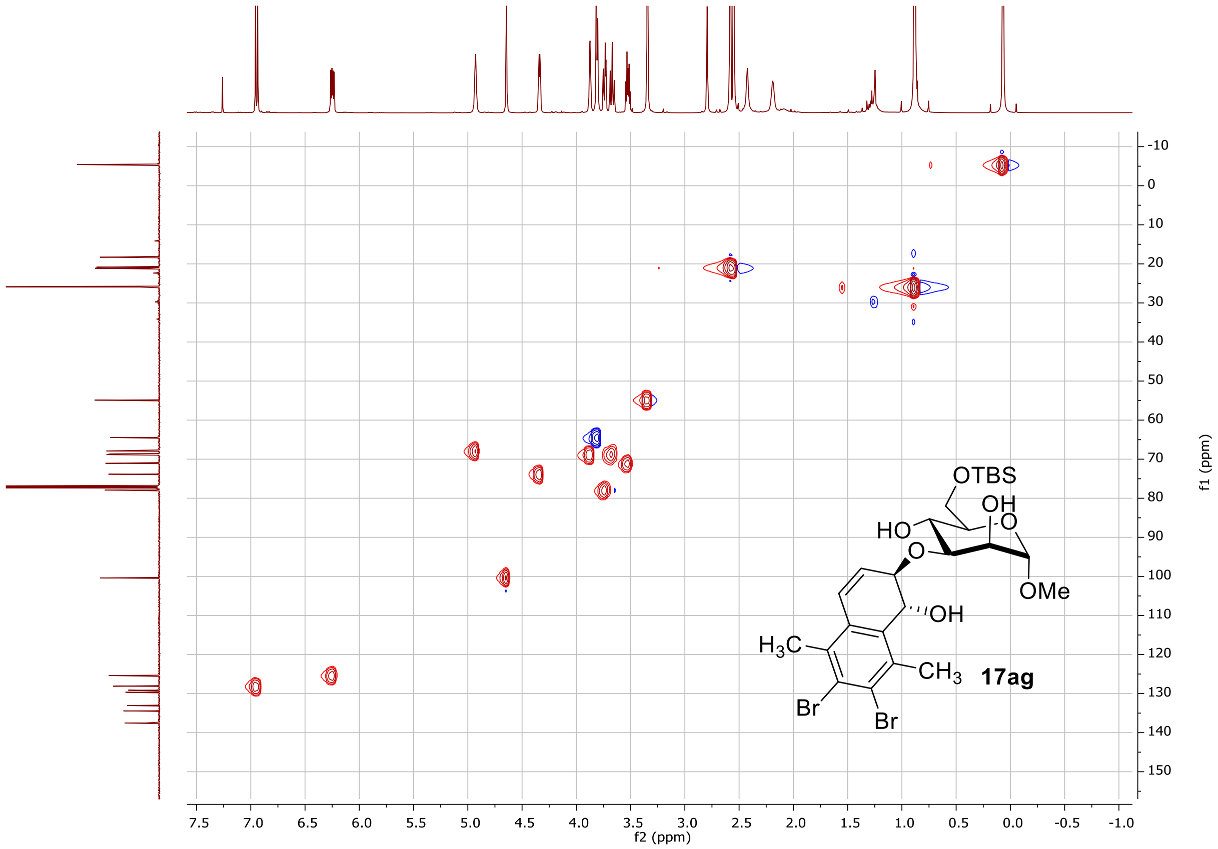

Supplementary Figure 334. HSQC spectra for **17ag**

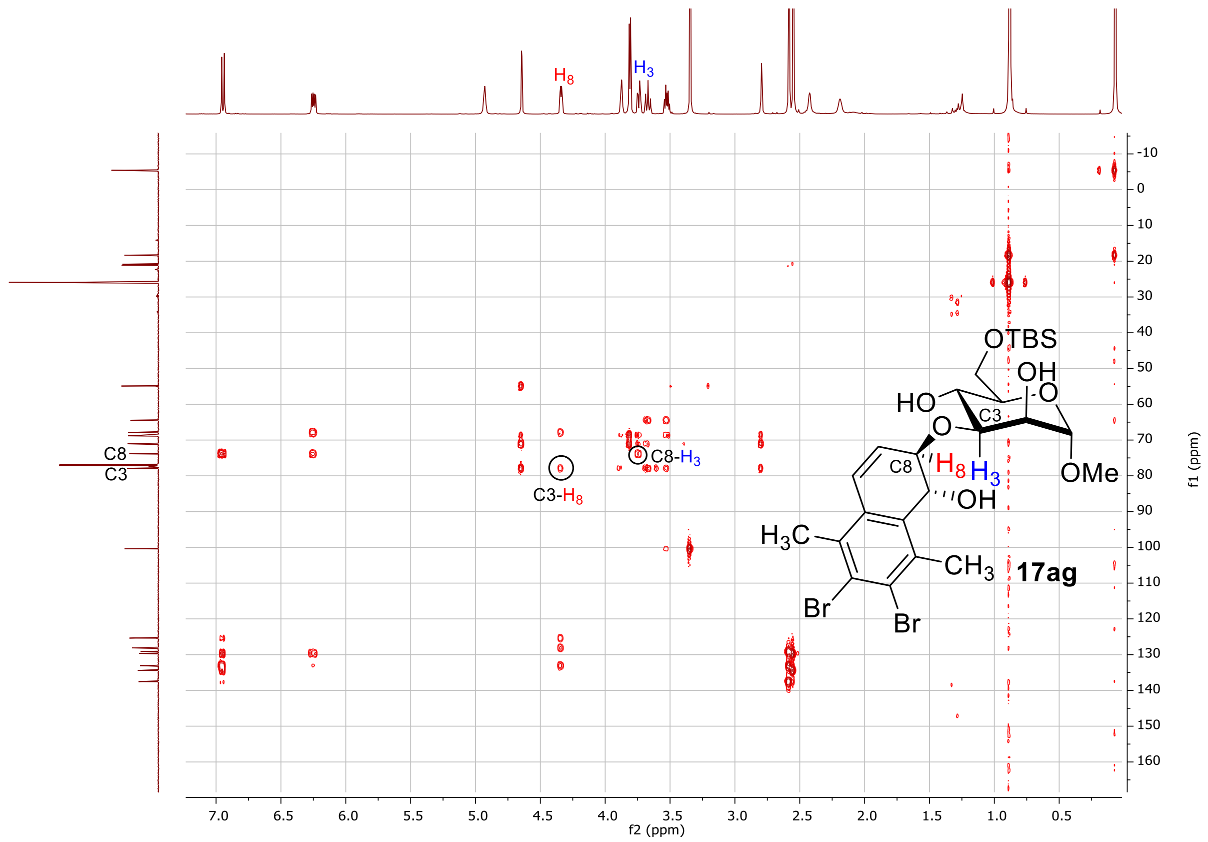

Supplementary Figure 335. HMBC spectra for **17ag**

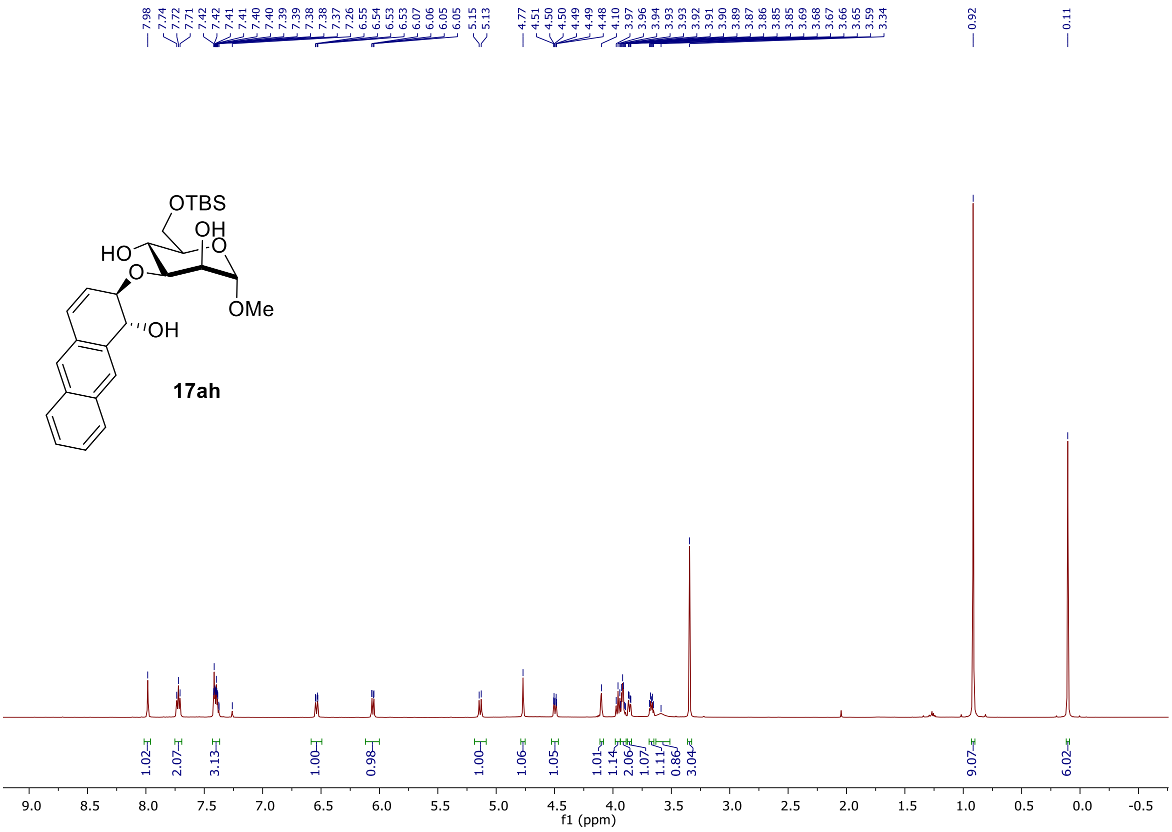

Supplementary Figure 336. ^{1}H spectra for **17ah**

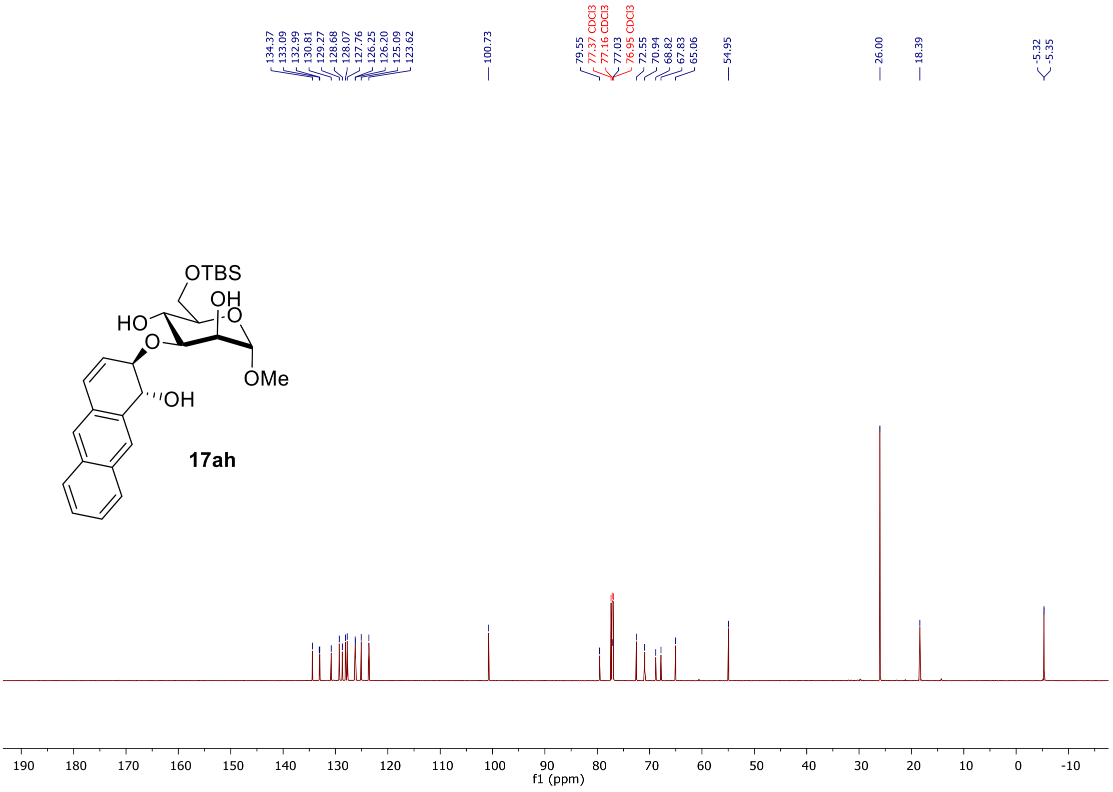

Supplementary Figure 337. ^{13}C spectra for **17ah**

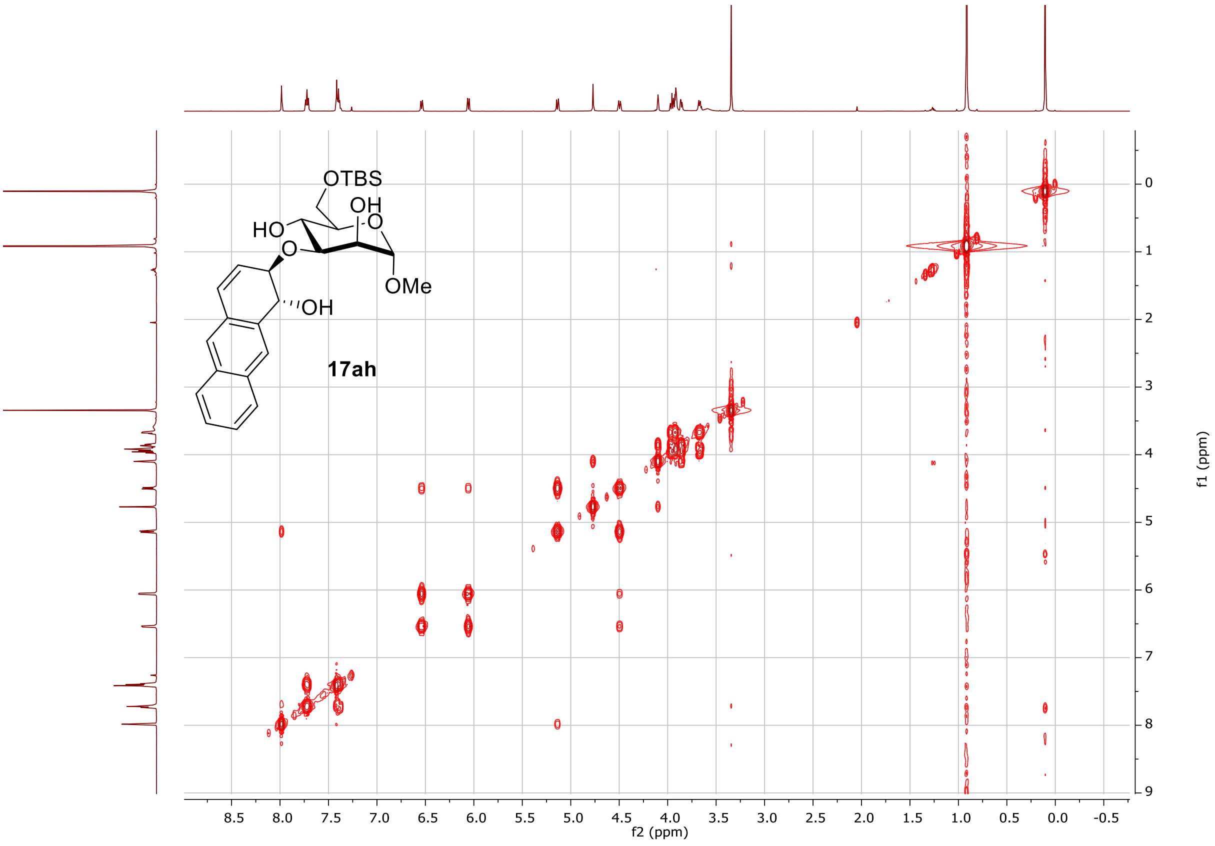

Supplementary Figure 338. COSY spectra for **17ah**

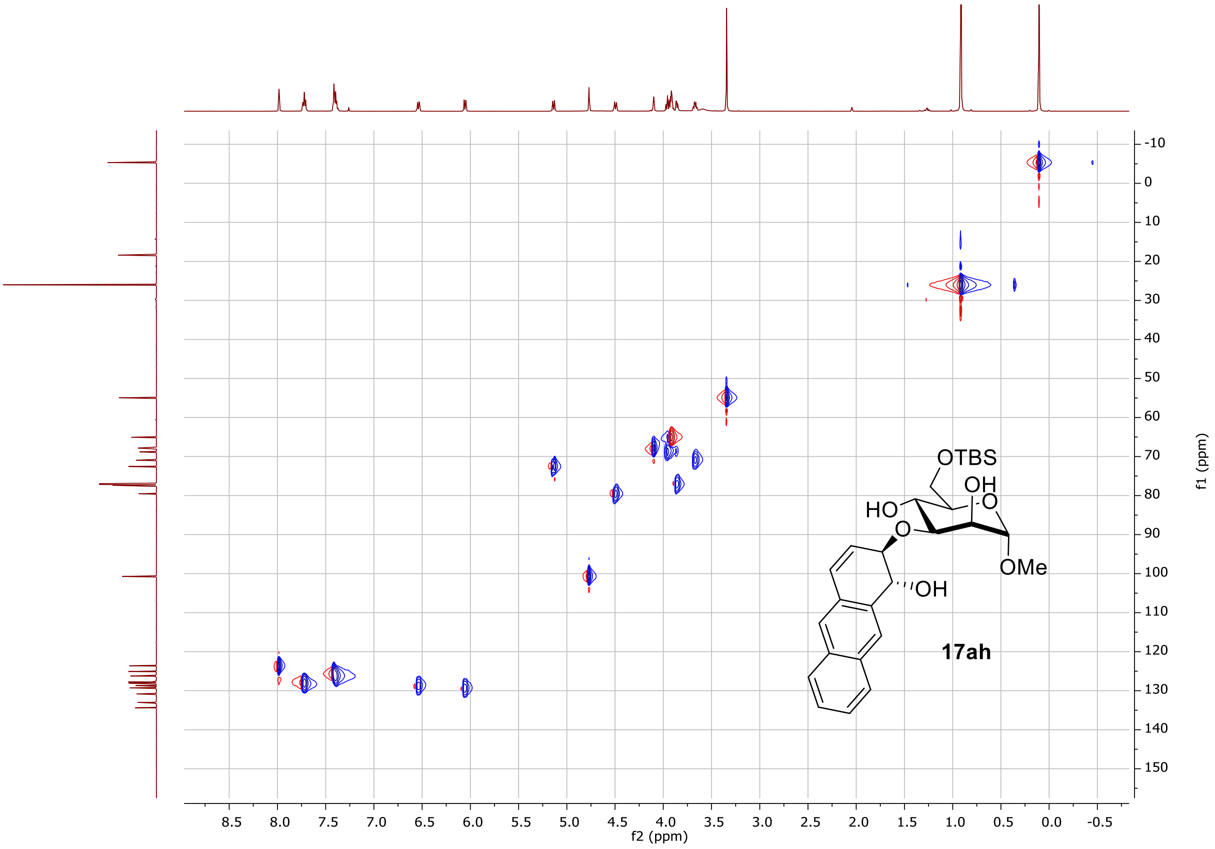

Supplementary Figure 339. HSQC spectra for **17ah**

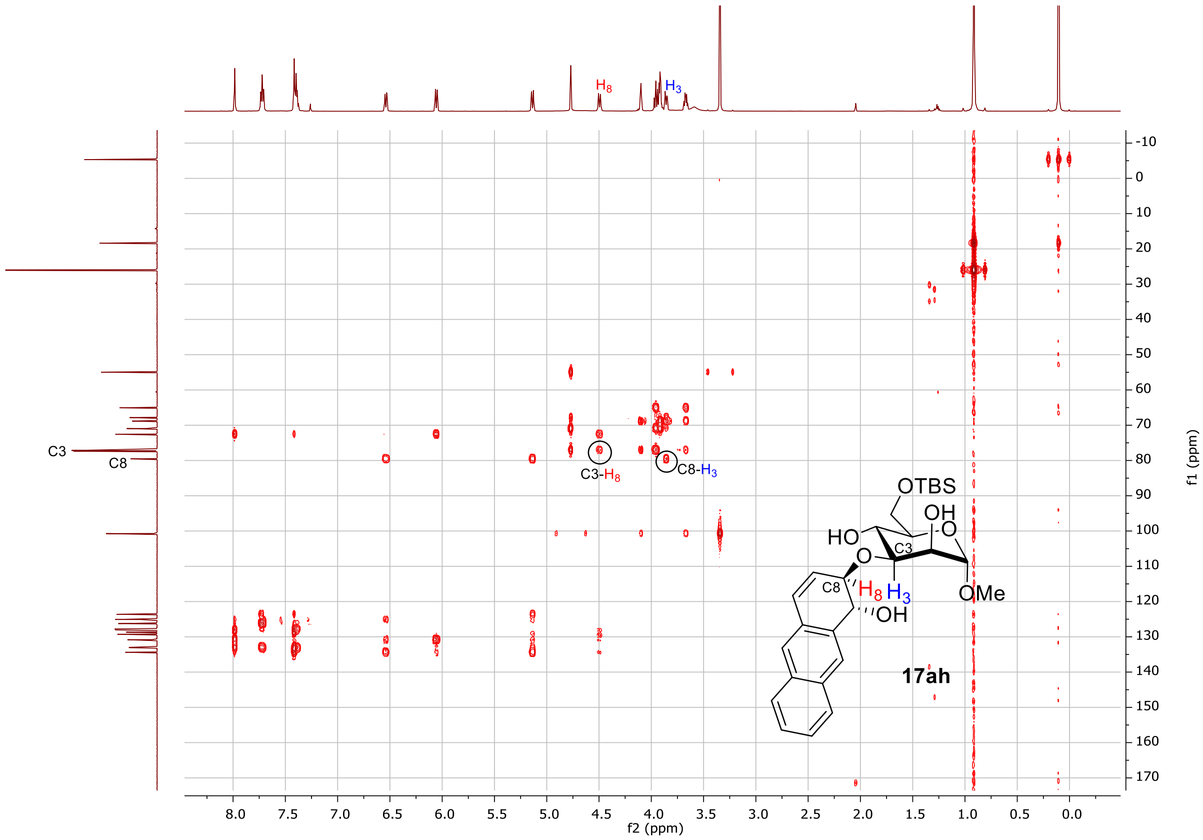

Supplementary Figure 340. HMBC spectra for **17ah**

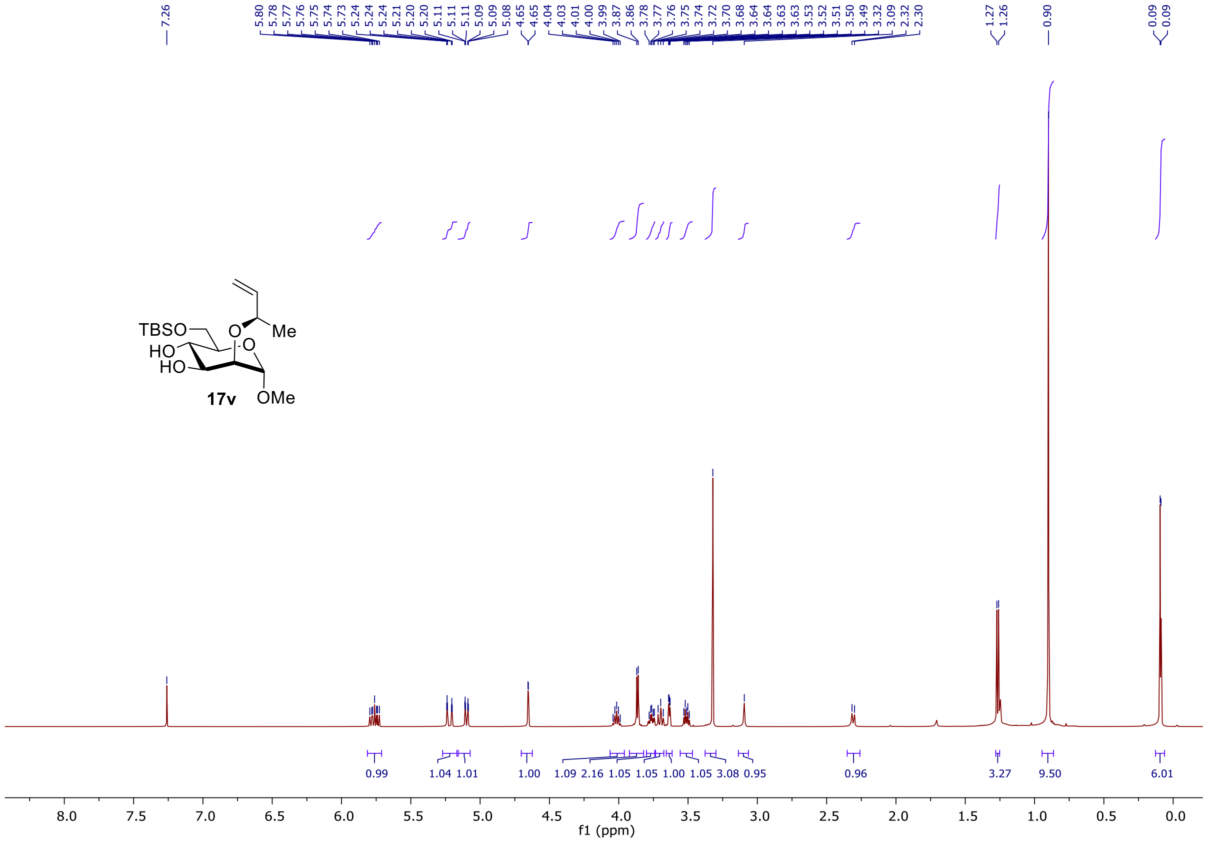

Supplementary Figure 341. ^{1}H spectra for **17v**

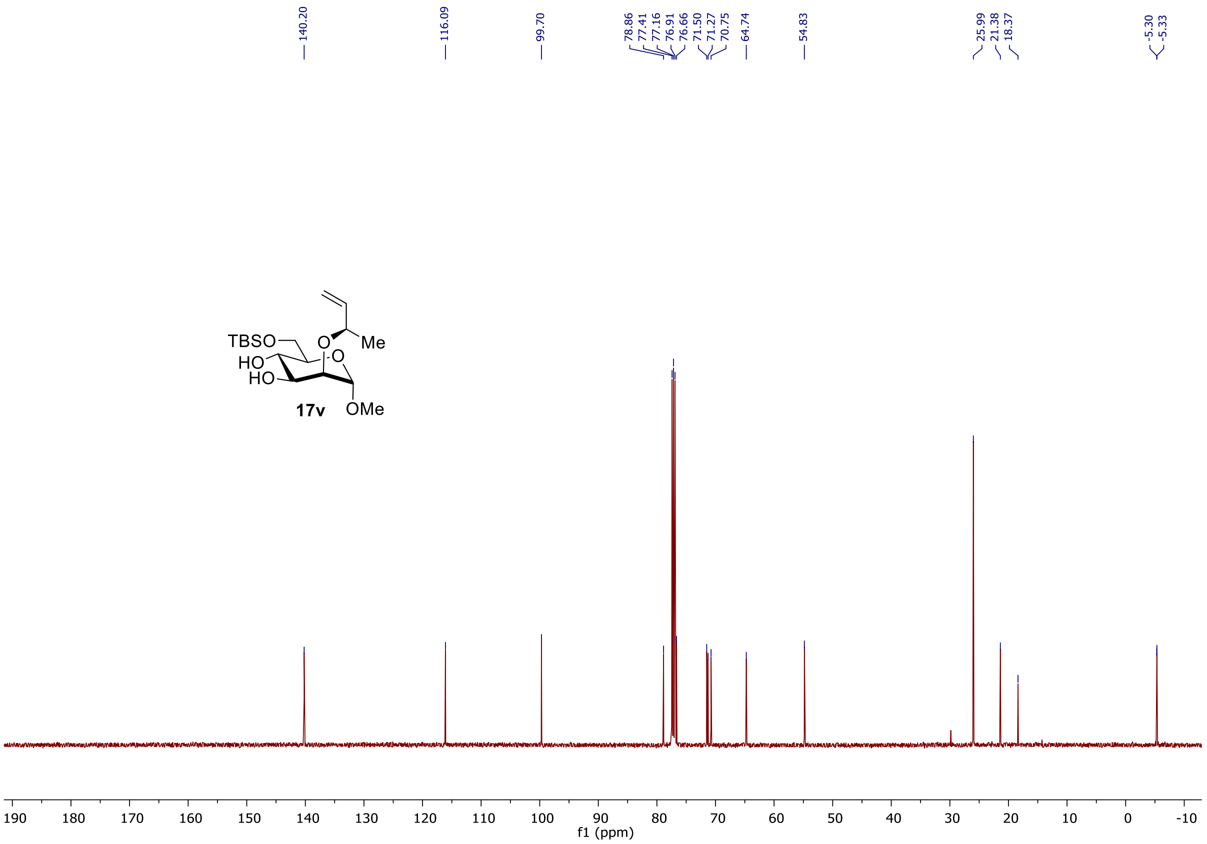

Supplementary Figure 342. ^{13}C spectra for **17v**

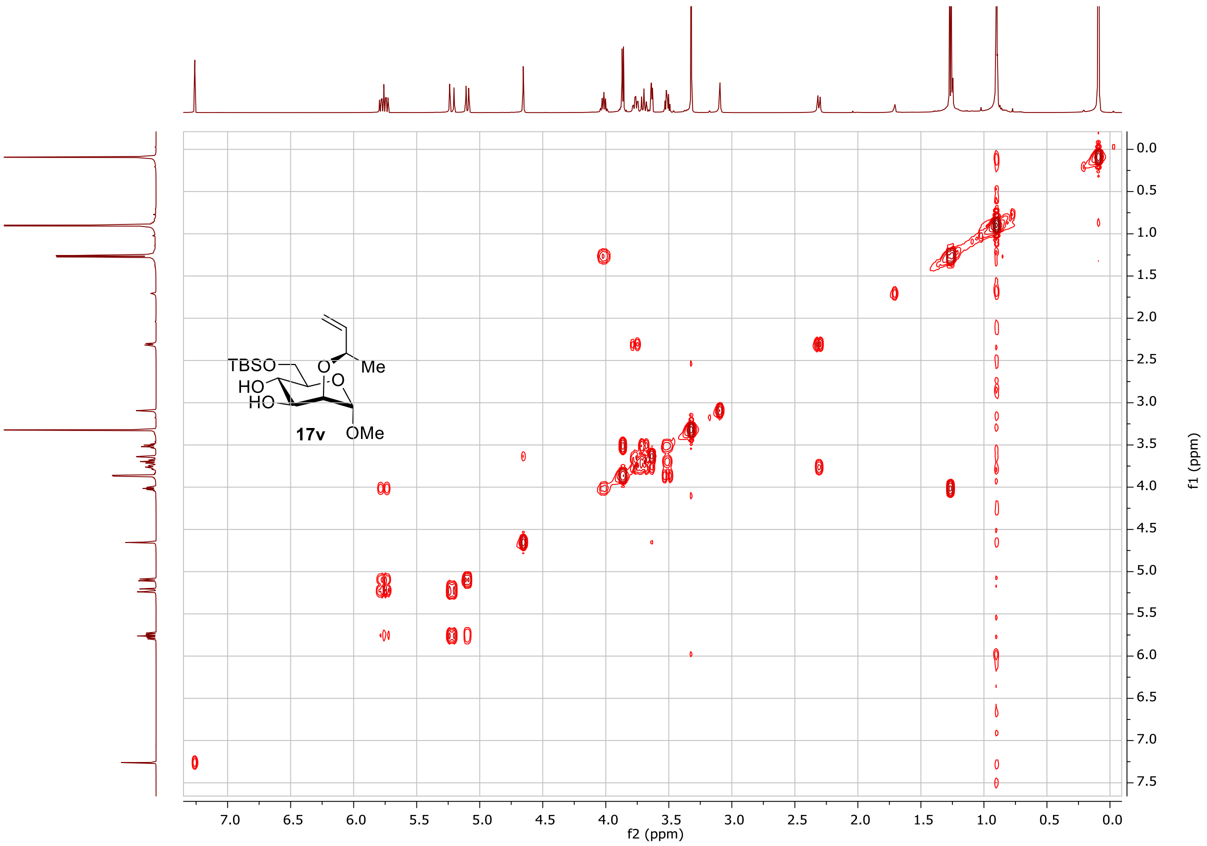

Supplementary Figure 343. COSY spectra for **17v**

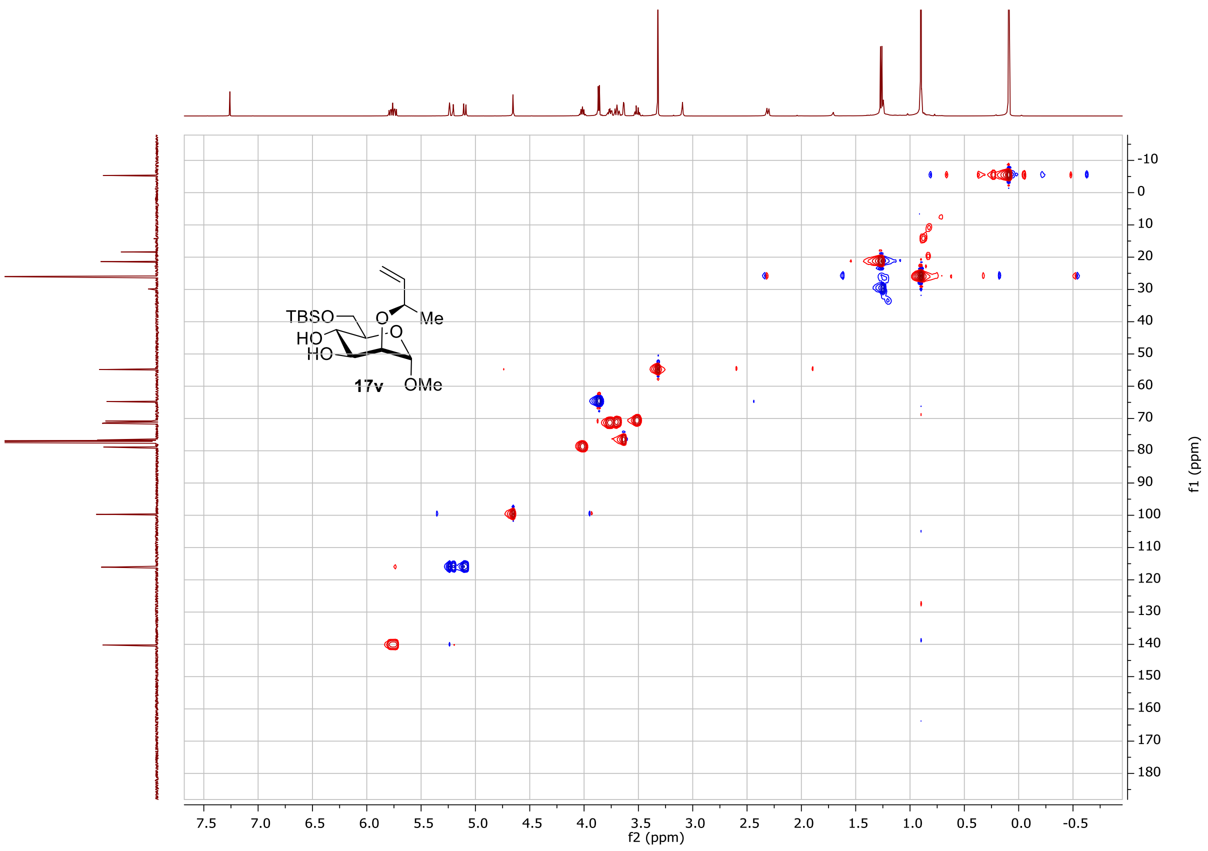

Supplementary Figure 344. HSQC spectra for **17v**

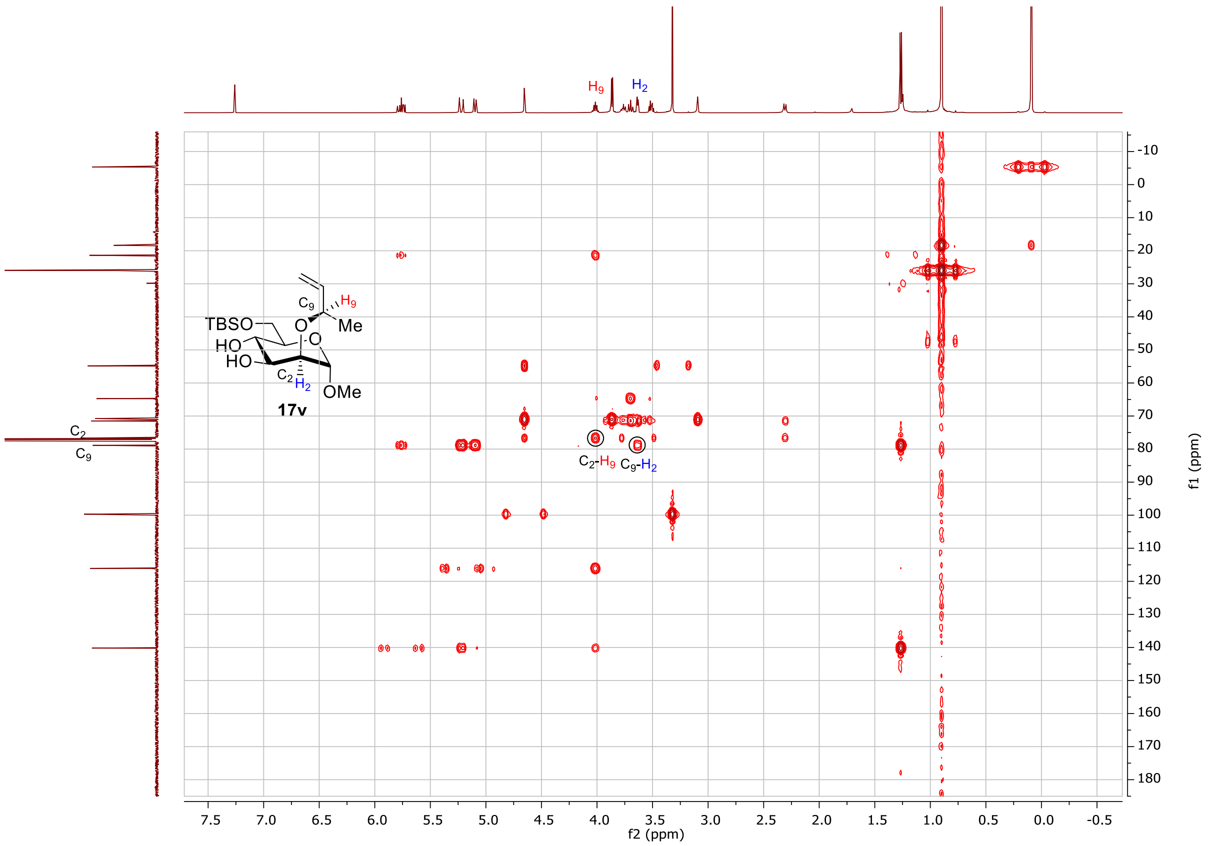

Supplementary Figure 345. HMBC spectra for **17v**

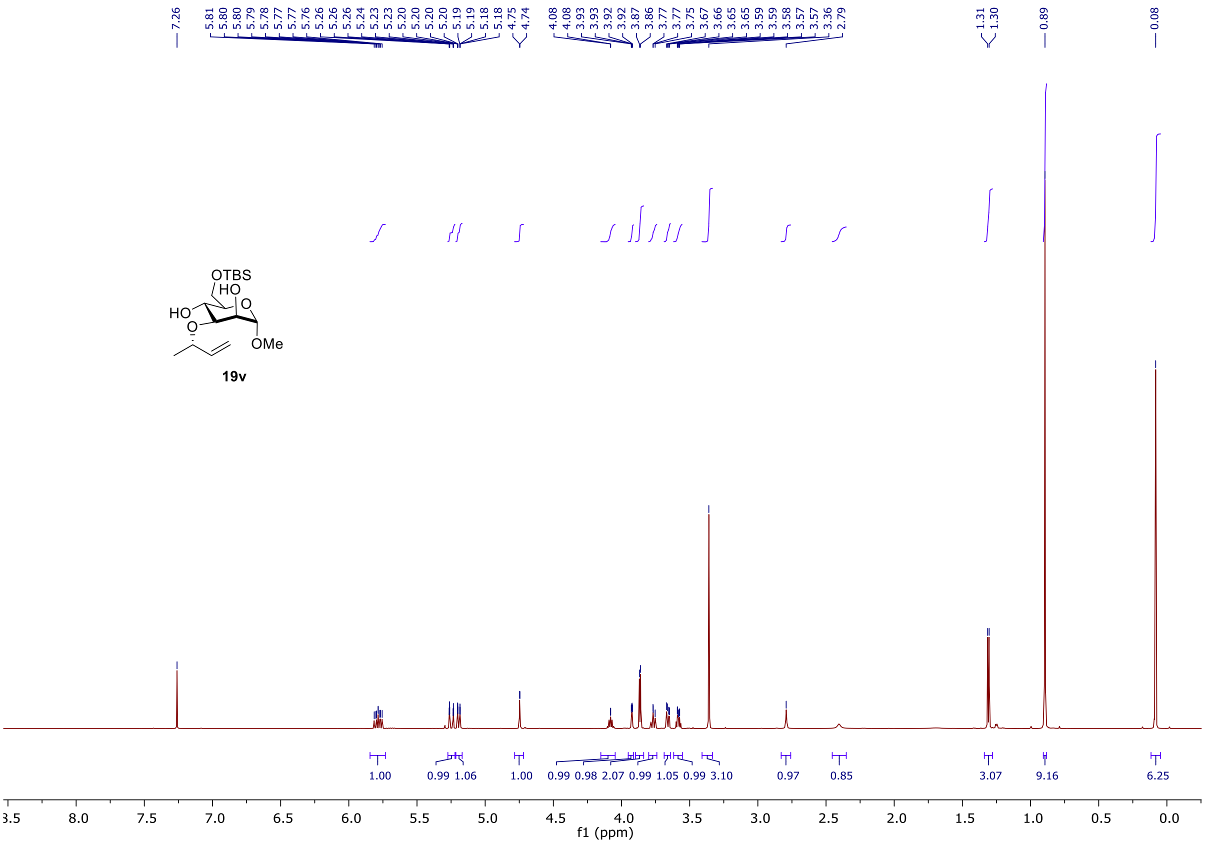

Supplementary Figure 346. ^{1}H spectra for **19v**

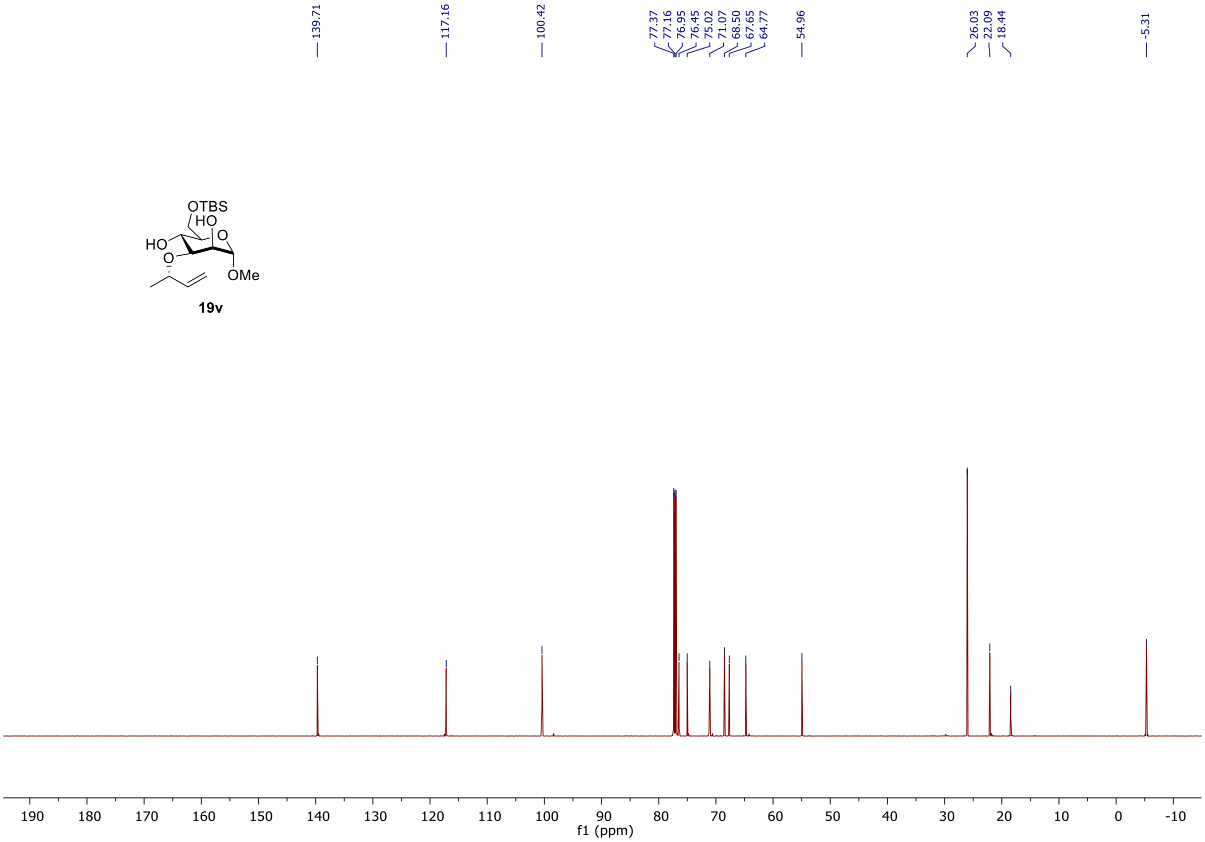

Supplementary Figure 347. ^{13}C spectra for **19v**

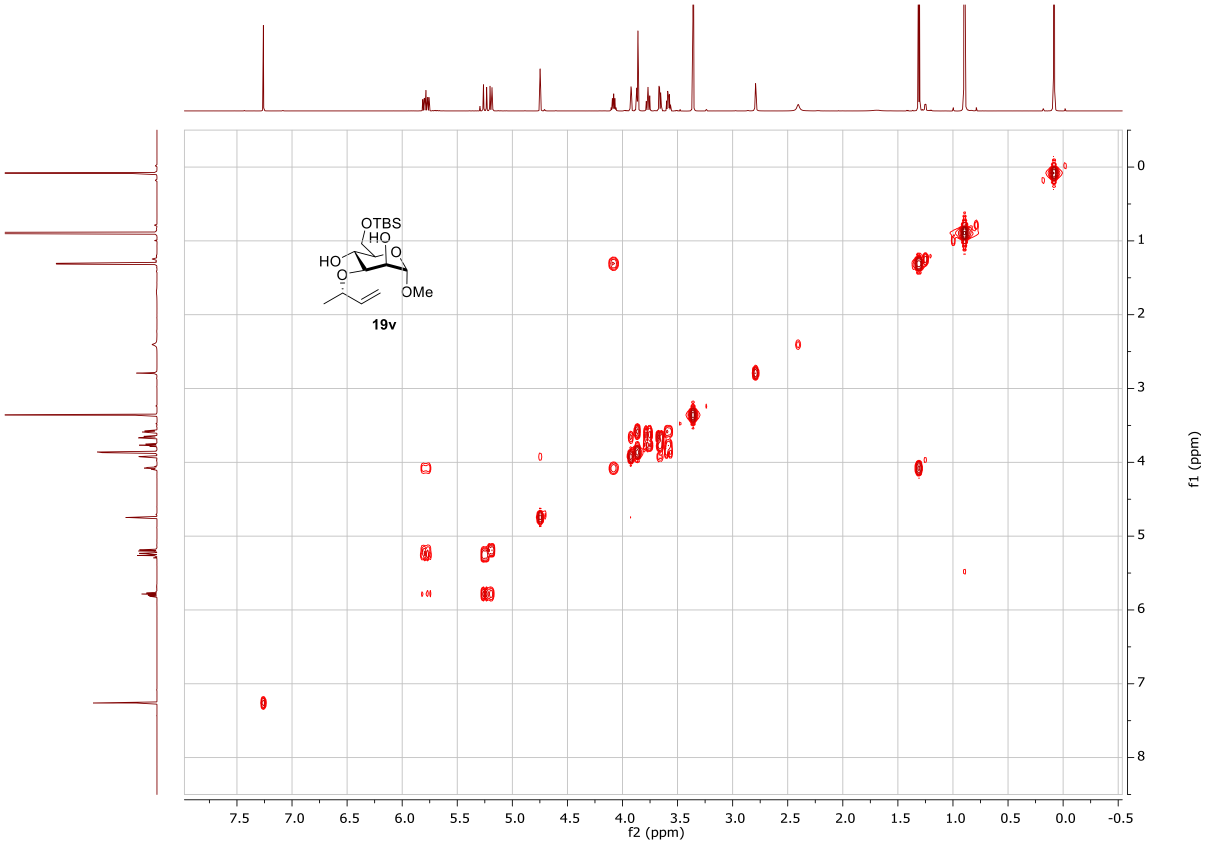

Supplementary Figure 348. COSY spectra for **19v**

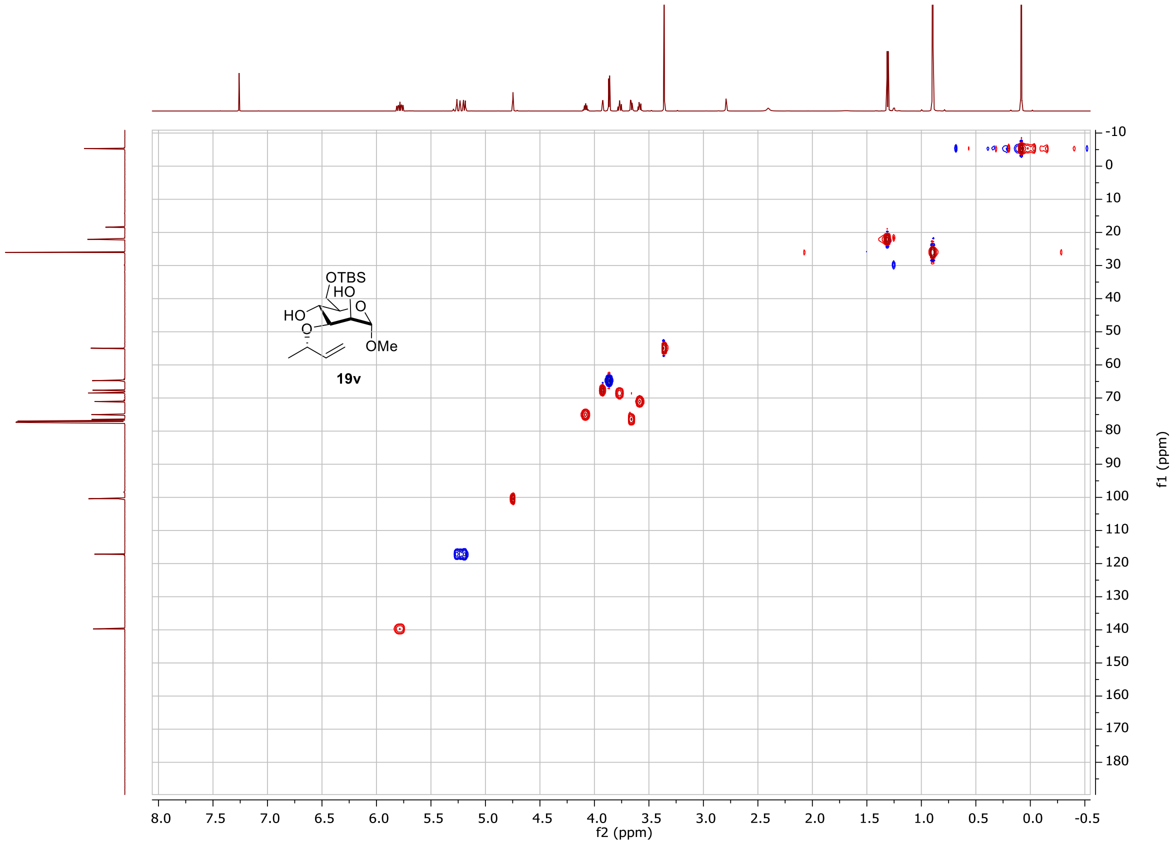

Supplementary Figure 349. HSQC spectra for **19v**

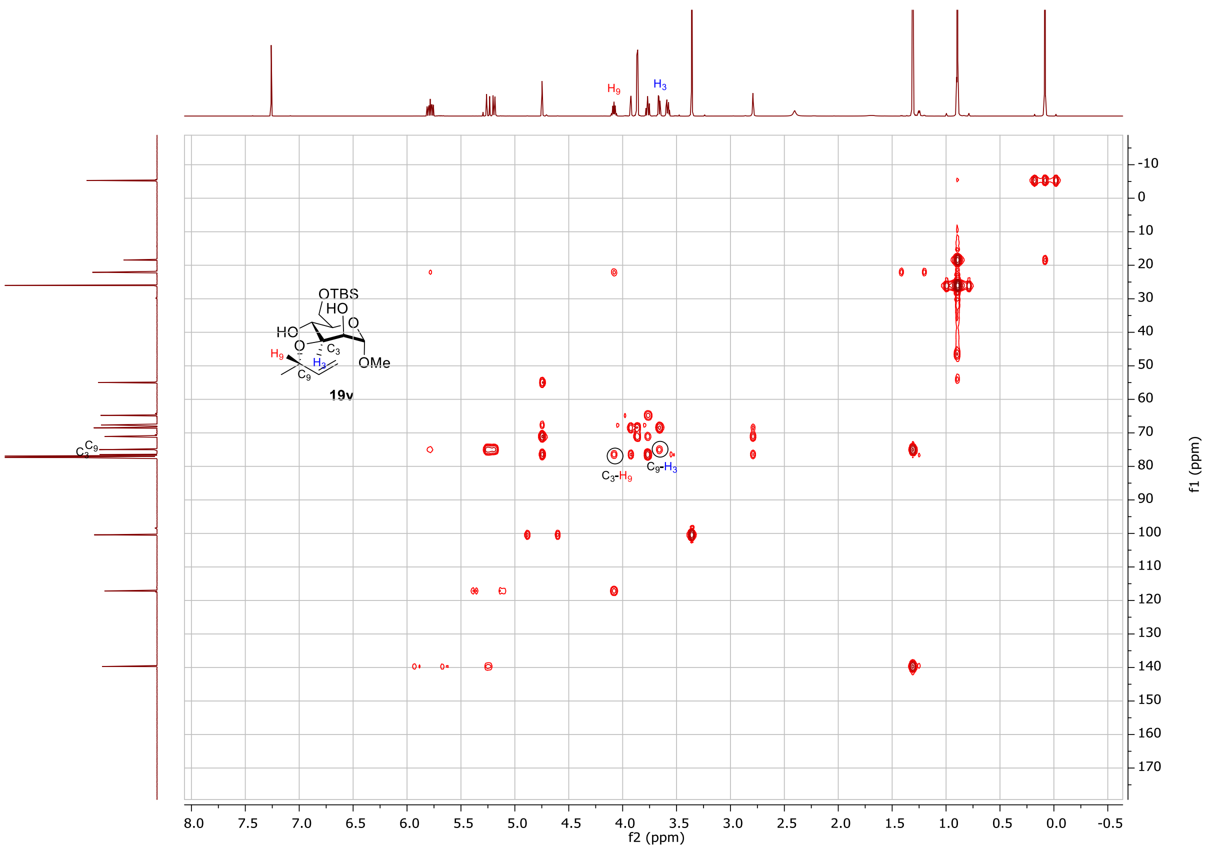

Supplementary Figure 350. HMBC spectra for **19v**

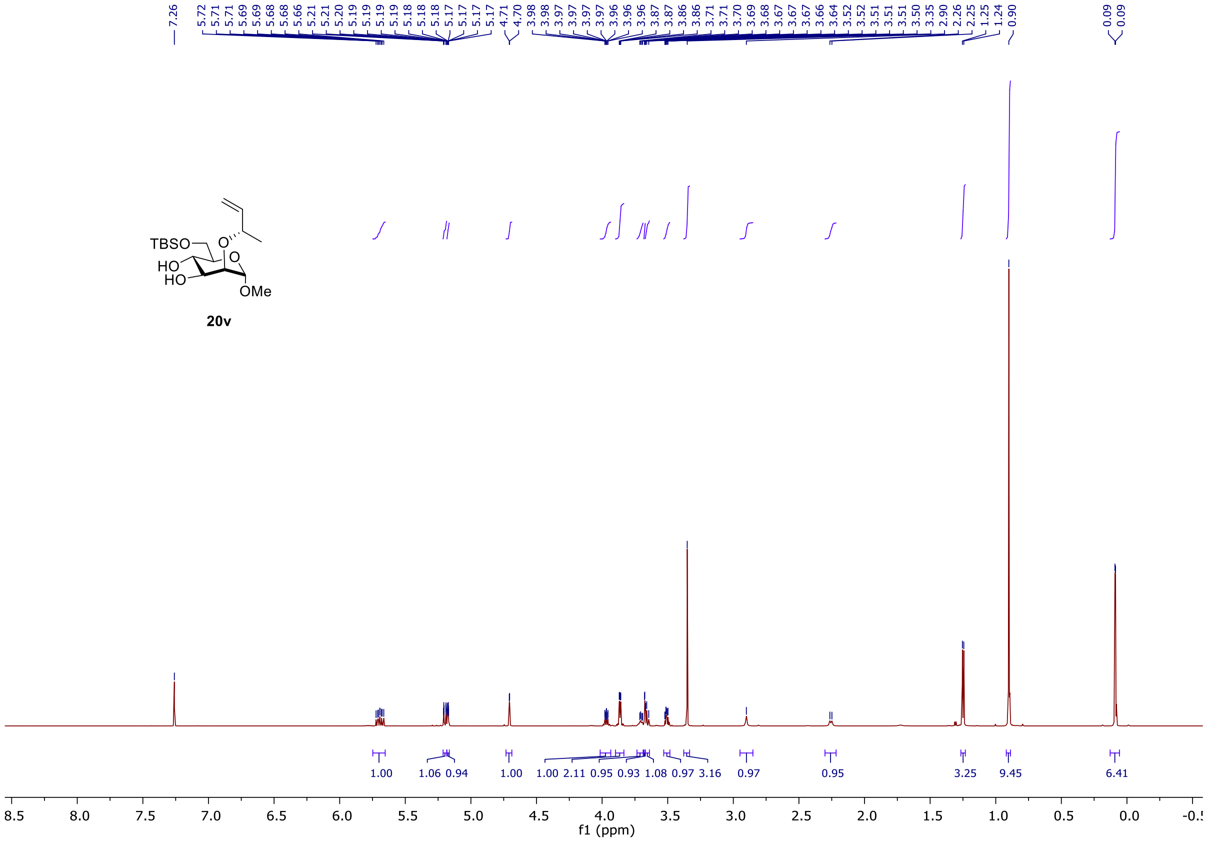

Supplementary Figure 351. ^{1}H spectra for **20v**

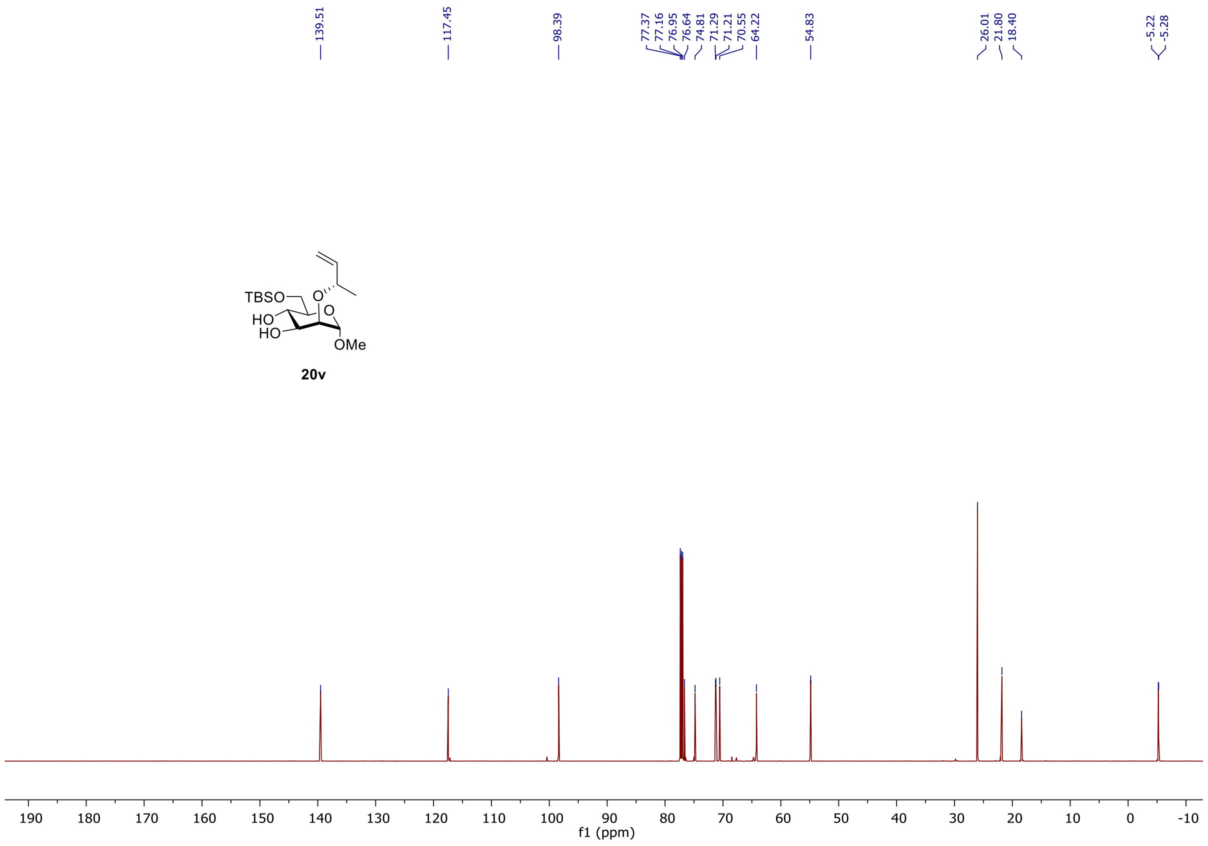

Supplementary Figure 352. ^{13}C spectra for **20v**

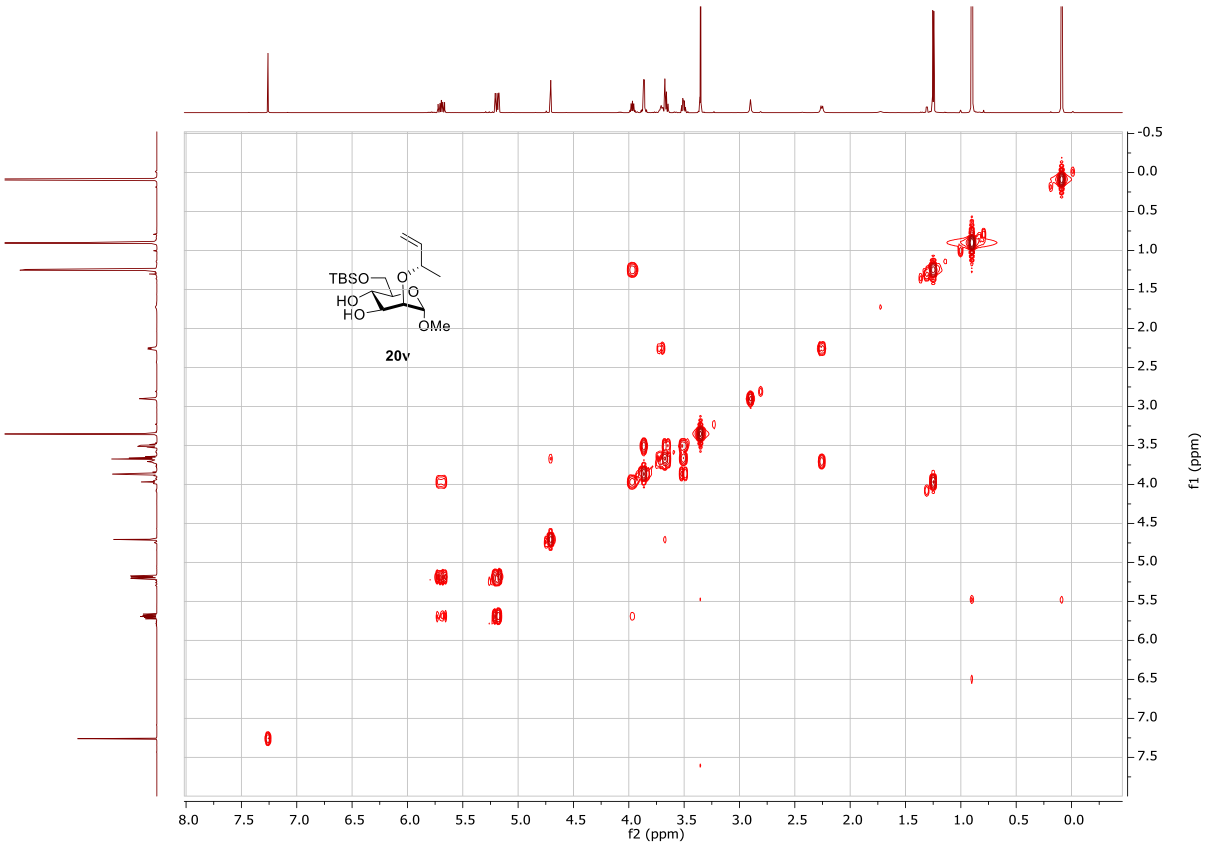

Supplementary Figure 353: COSY spectra for **20v**

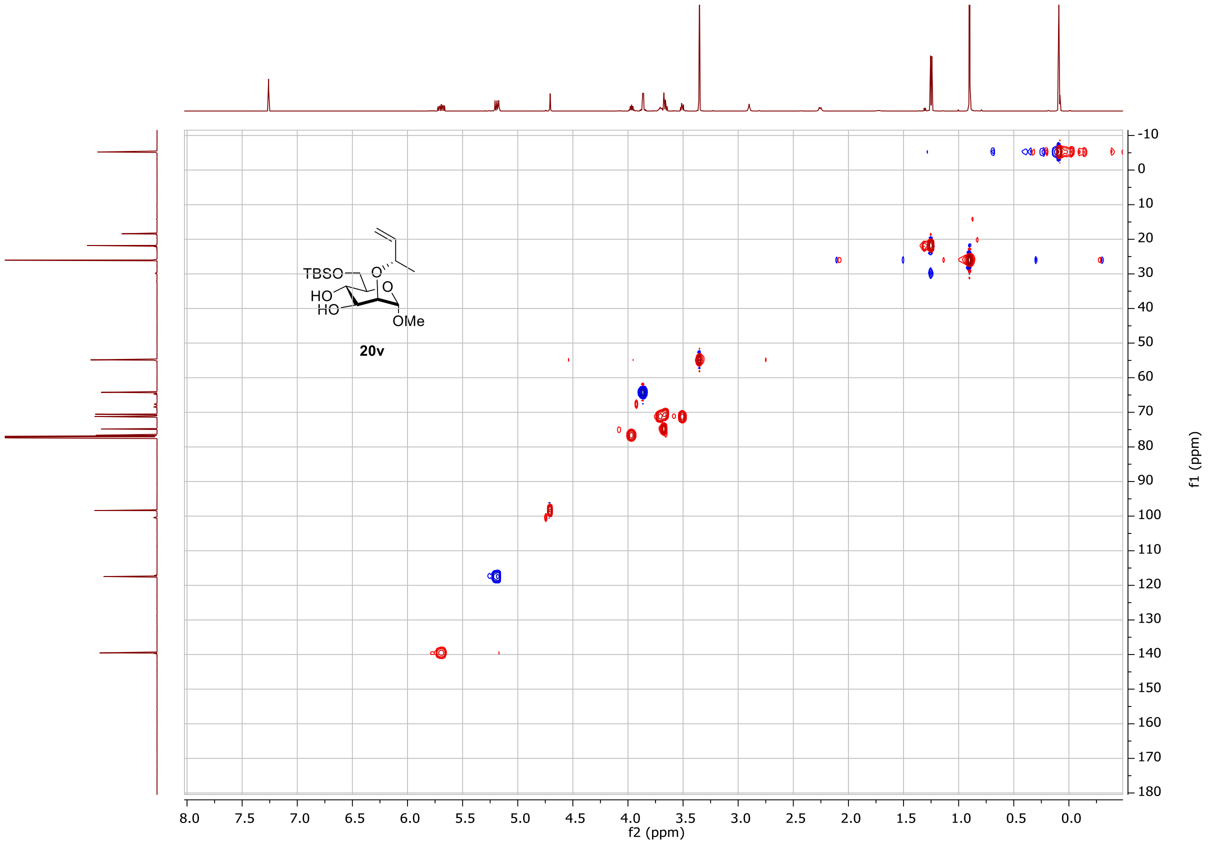

Supplementary Figure 354. HSQC spectra for **20v**

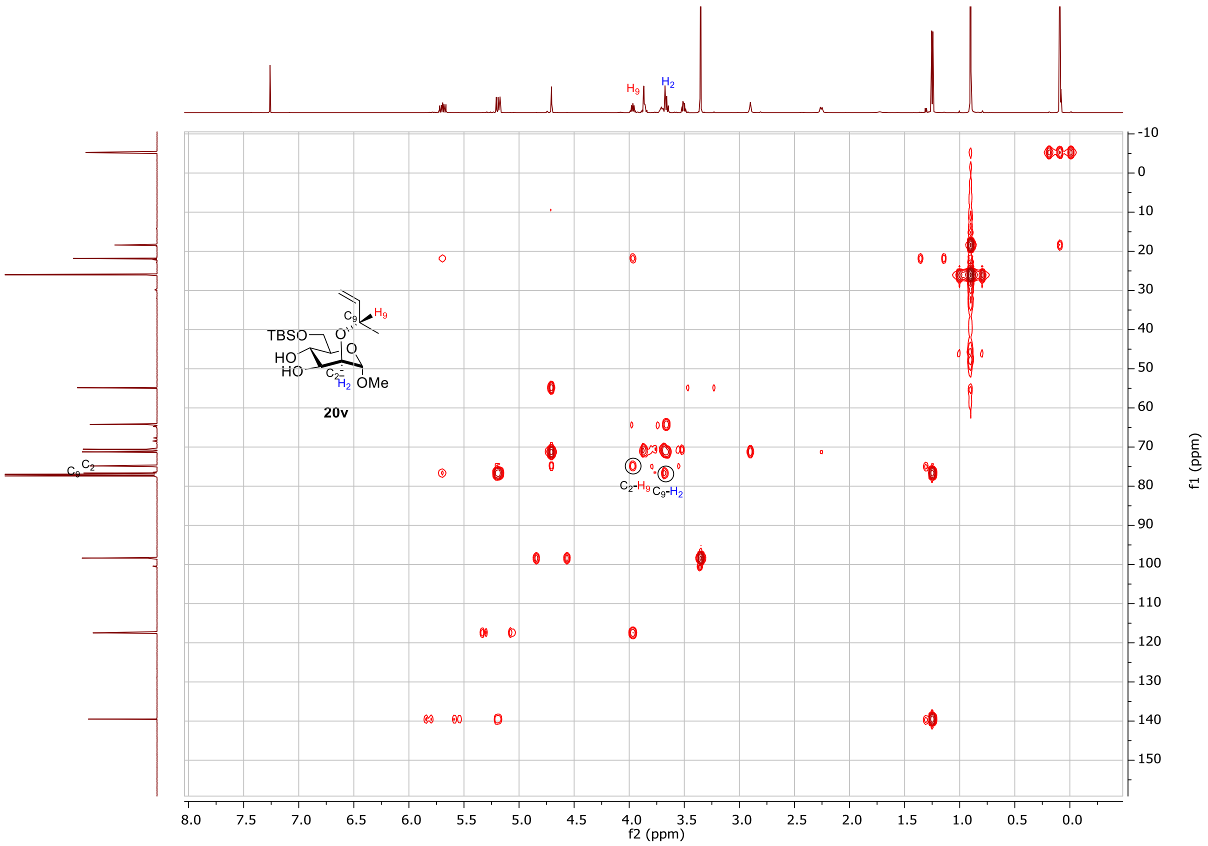

Supplementary Figure 355. HMBC spectra for **20v**

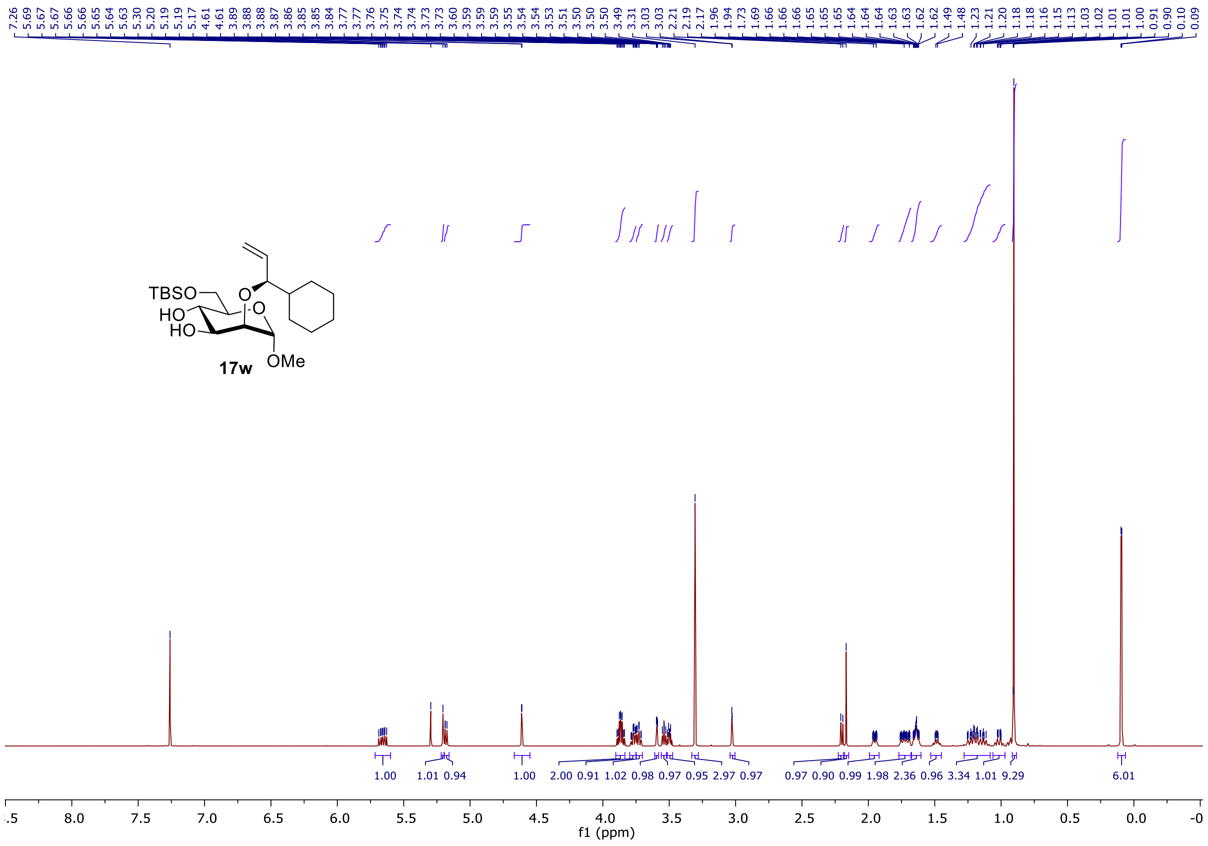

Supplementary Figure 356. ^{1}H spectra for **17w**

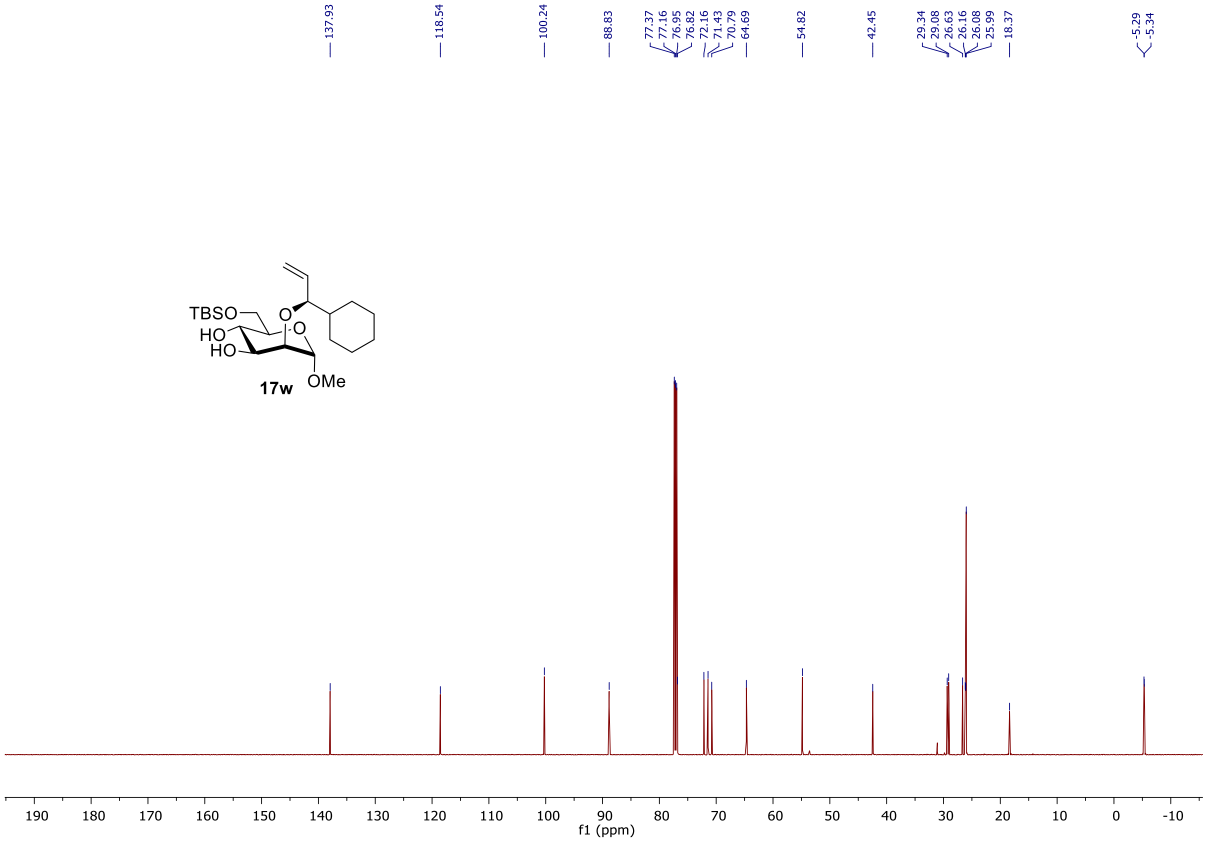

Supplementary Figure 357. ^{13}C spectra for **17w**

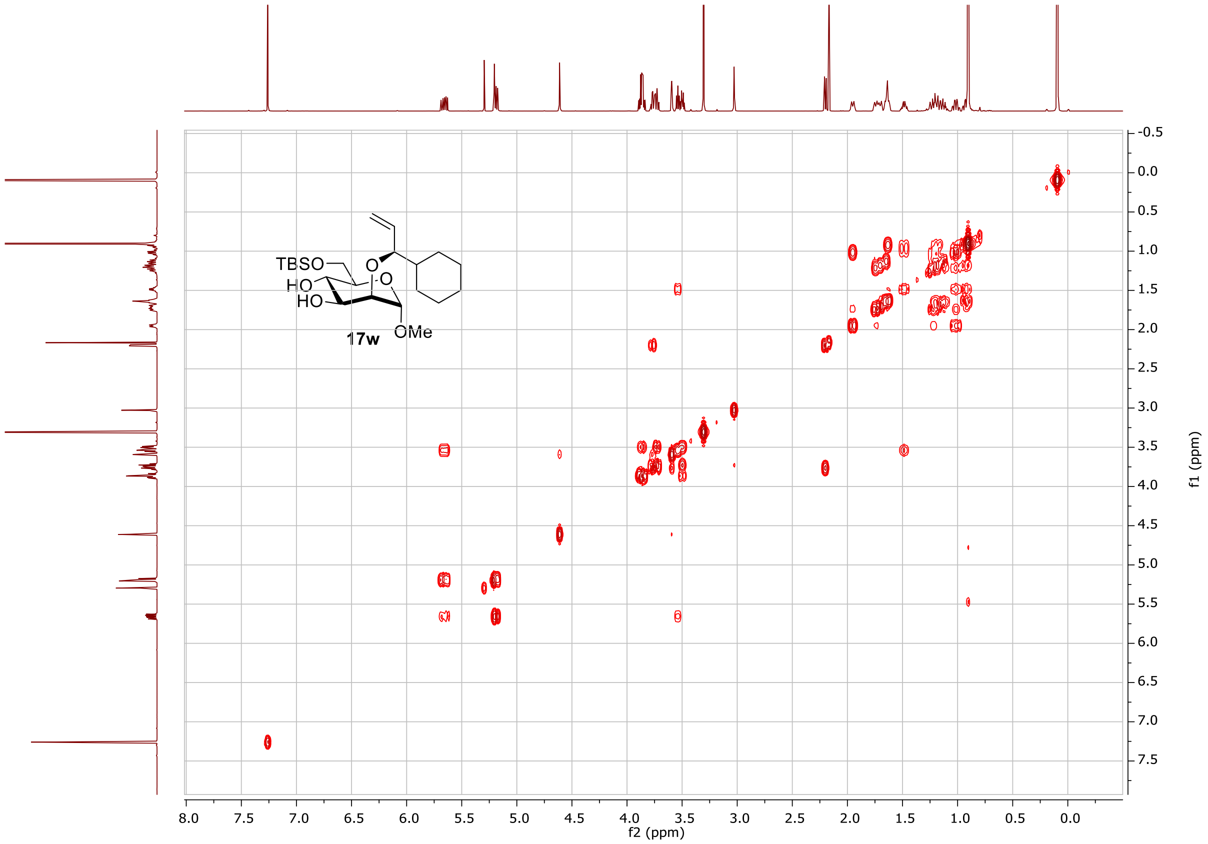

Supplementary Figure 358. COSY spectra for **17w**

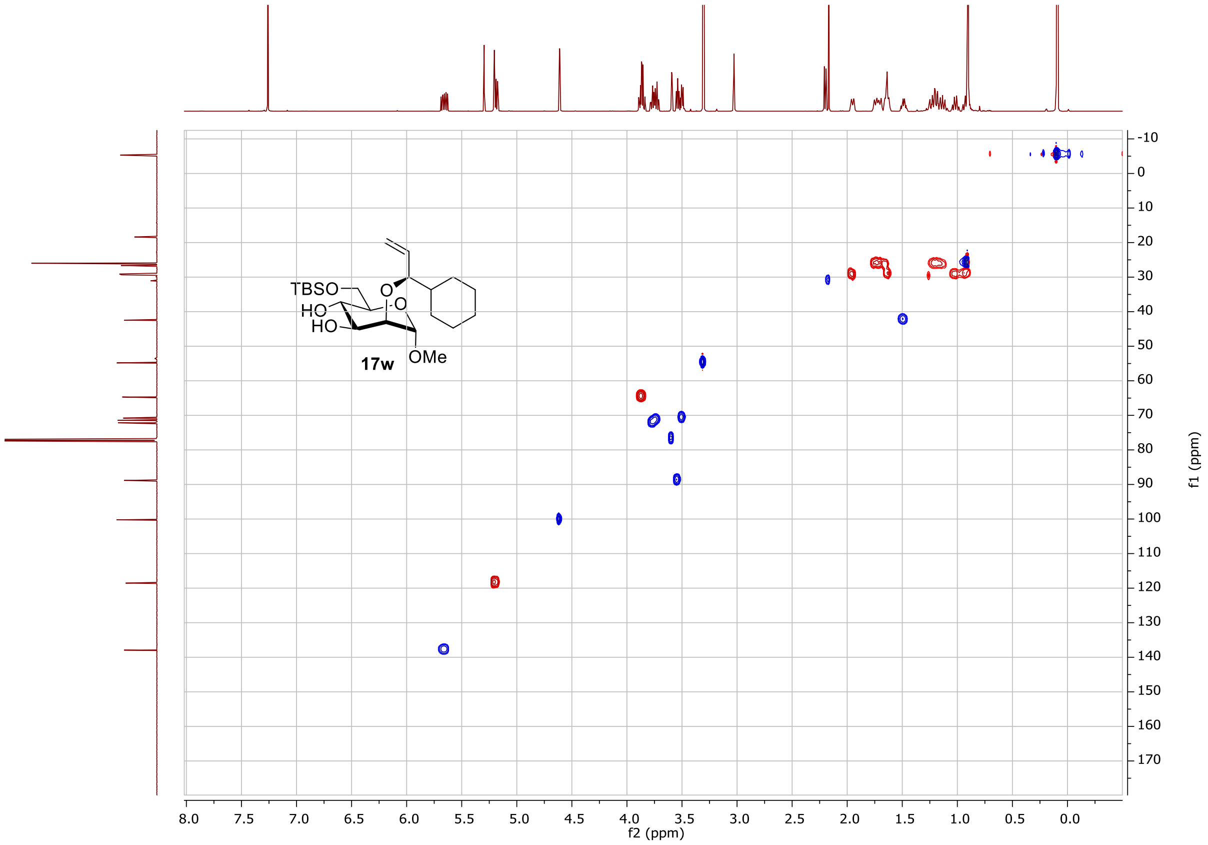

Supplementary Figure 359. HSQC spectra for **17w**

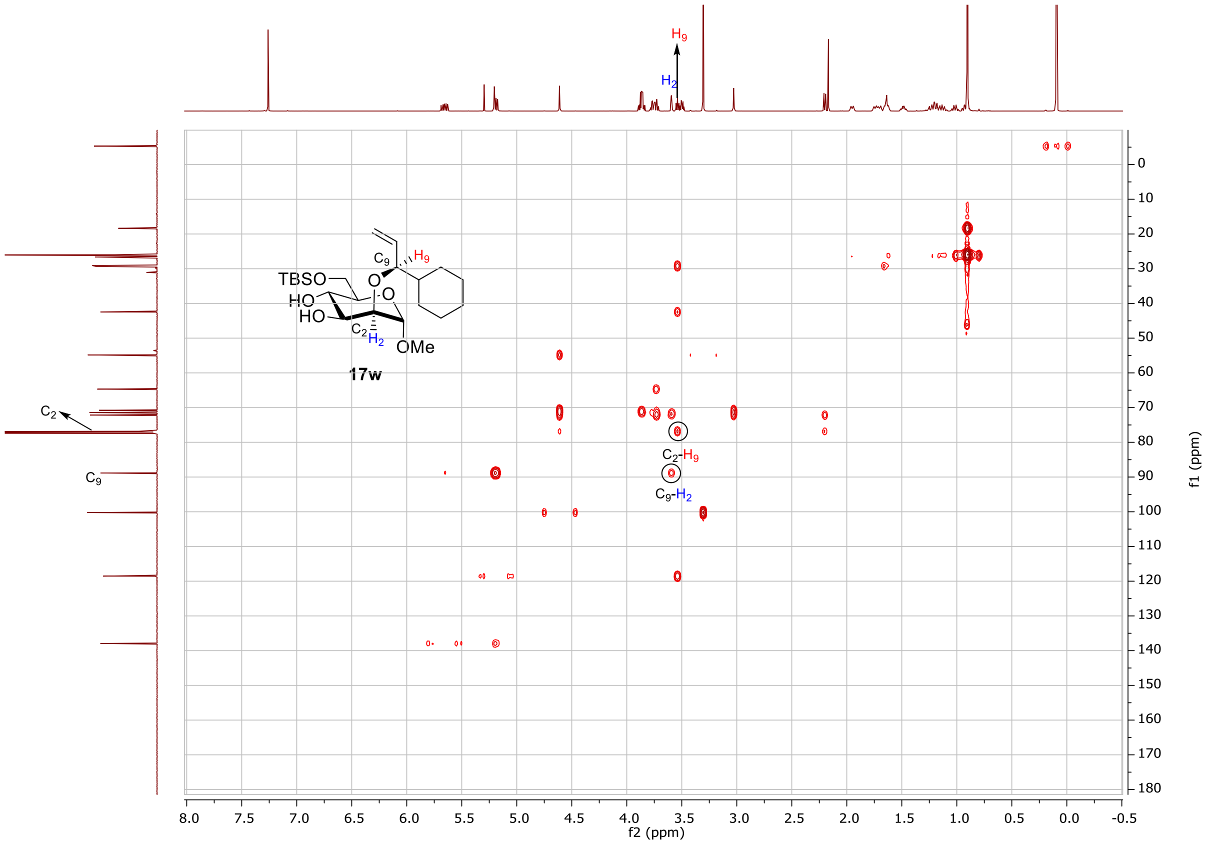

Supplementary Figure 360. HMBC spectra for **17w**

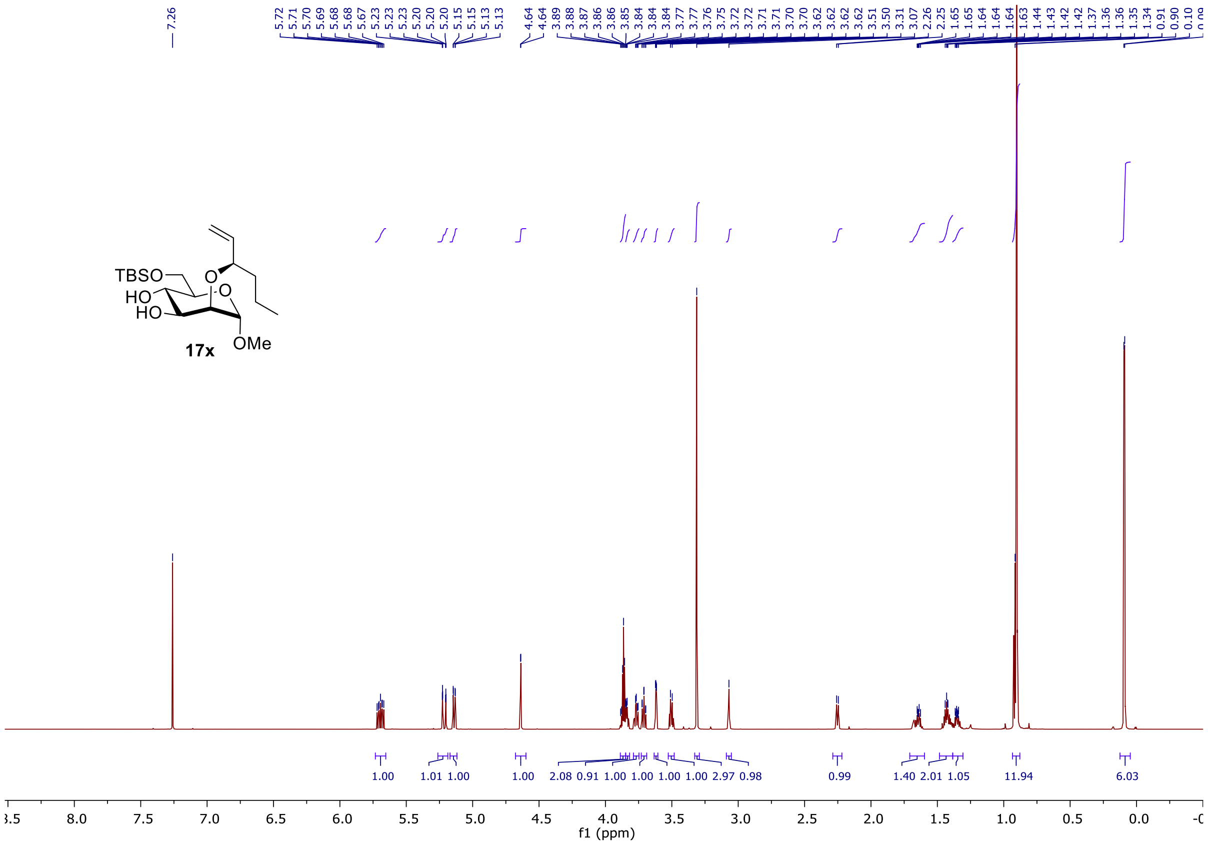

Supplementary Figure 361. ^{1}H spectra for **17x**

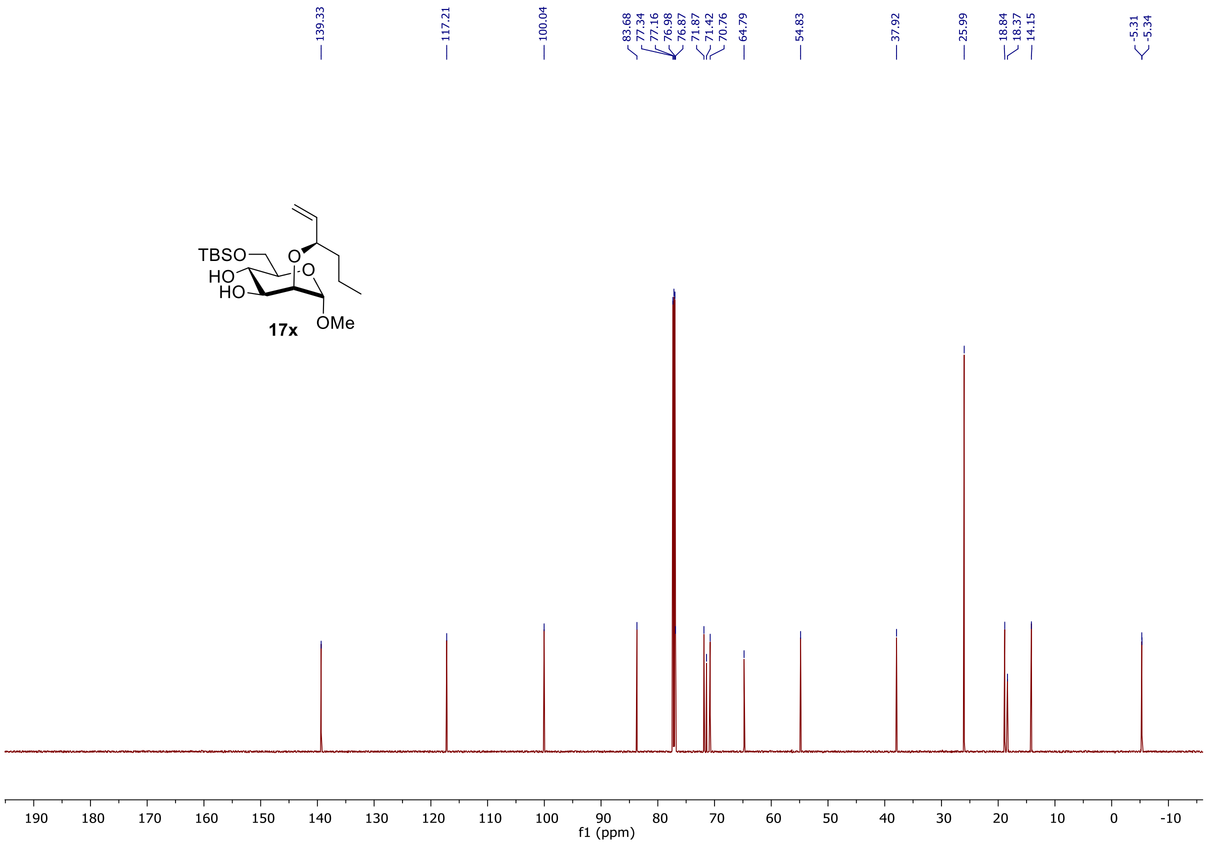

Supplementary Figure 362. ^{13}C spectra for **17x**

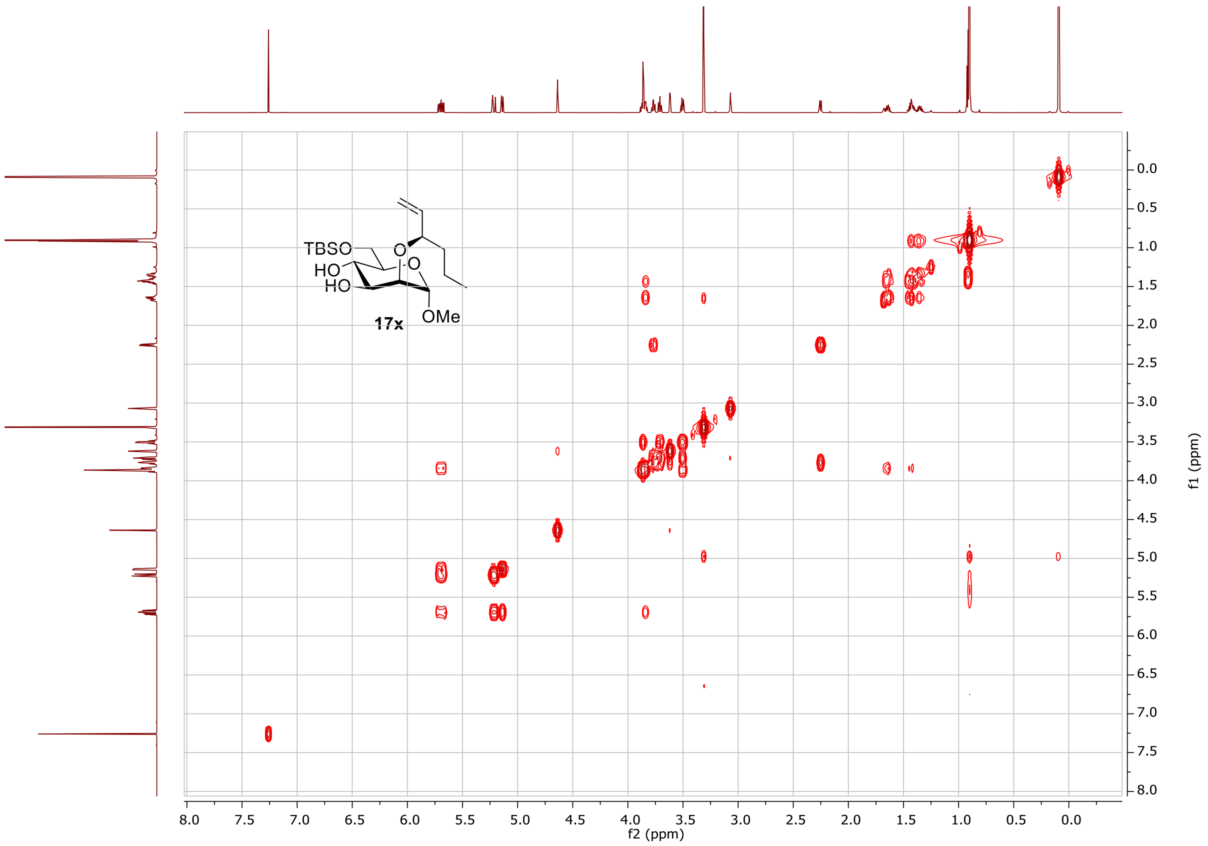

Supplementary Figure 363. COSY spectra for **17x**

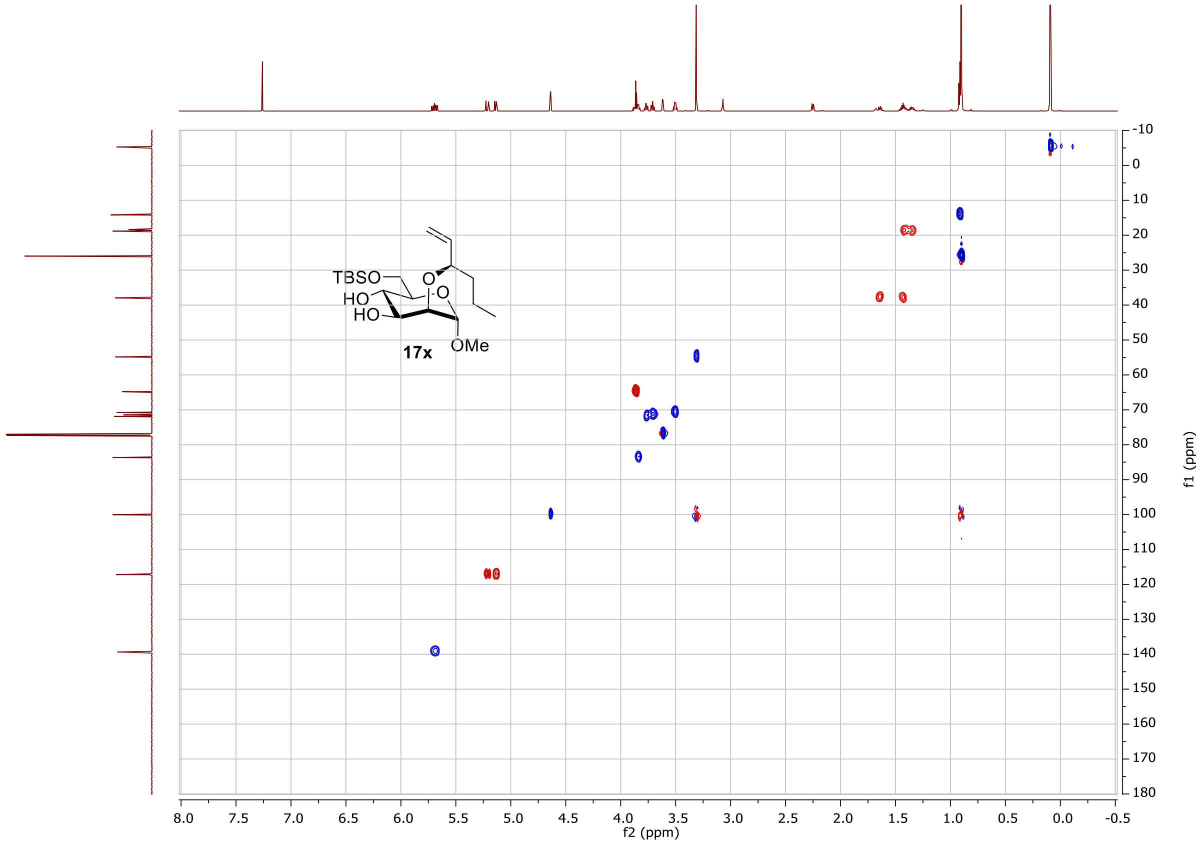

Supplementary Figure 364. HSQC spectra for **17x**

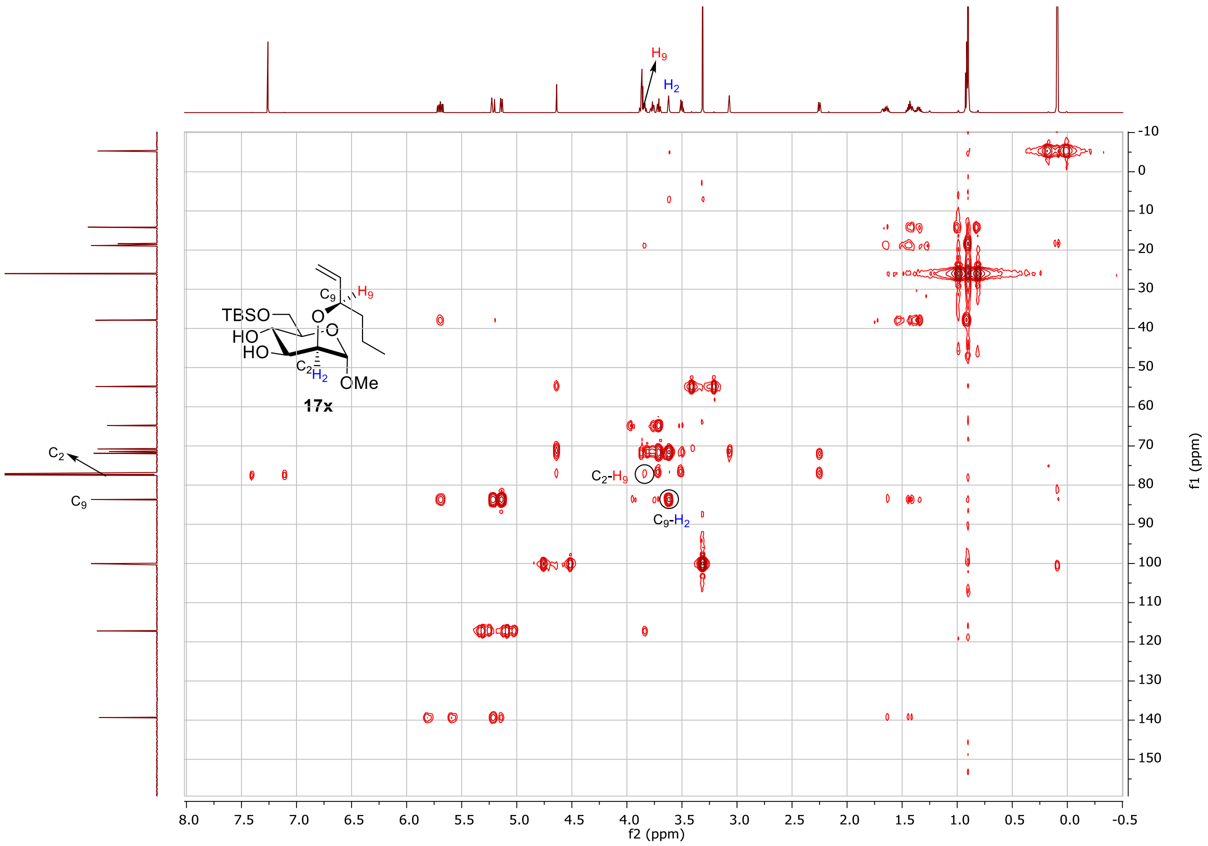

Supplementary Figure 365. HMBC spectra for **17x**

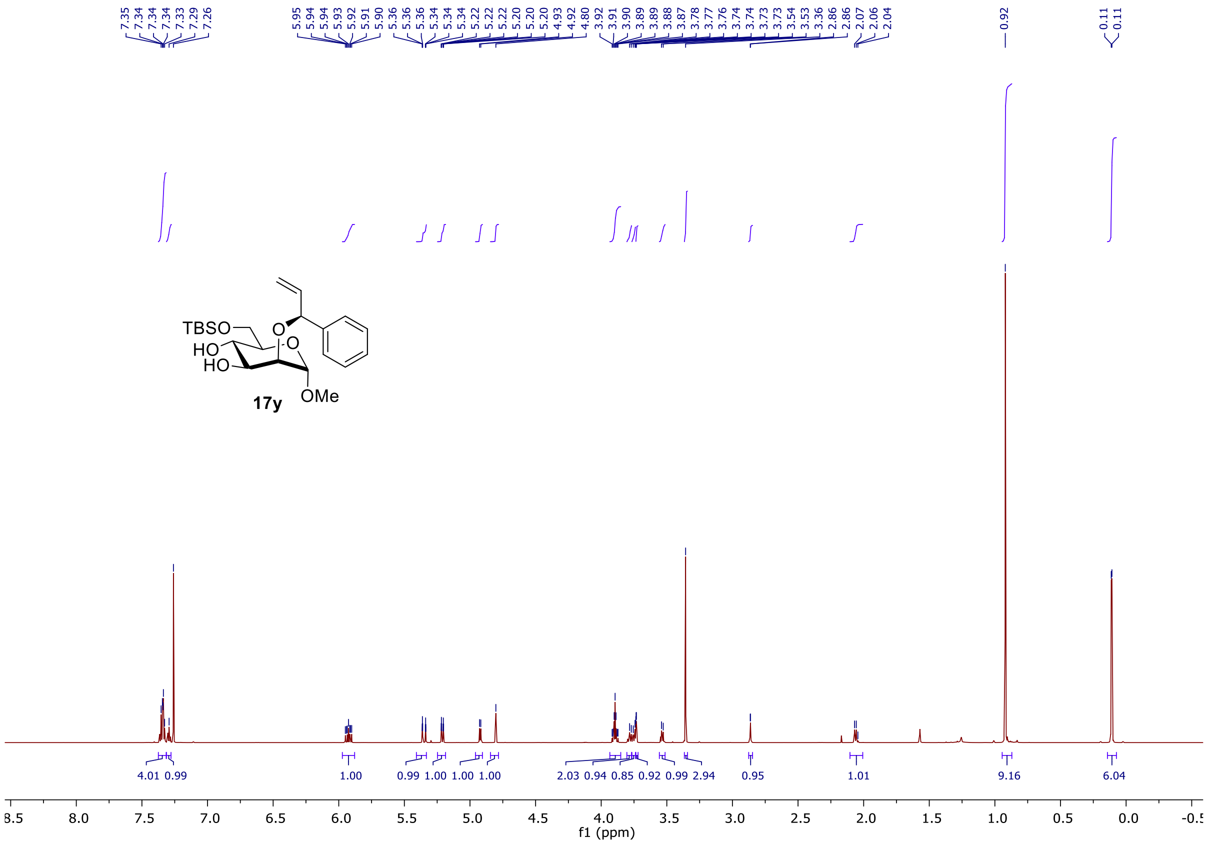

Supplementary Figure 366. ^{1}H spectra for **17y**

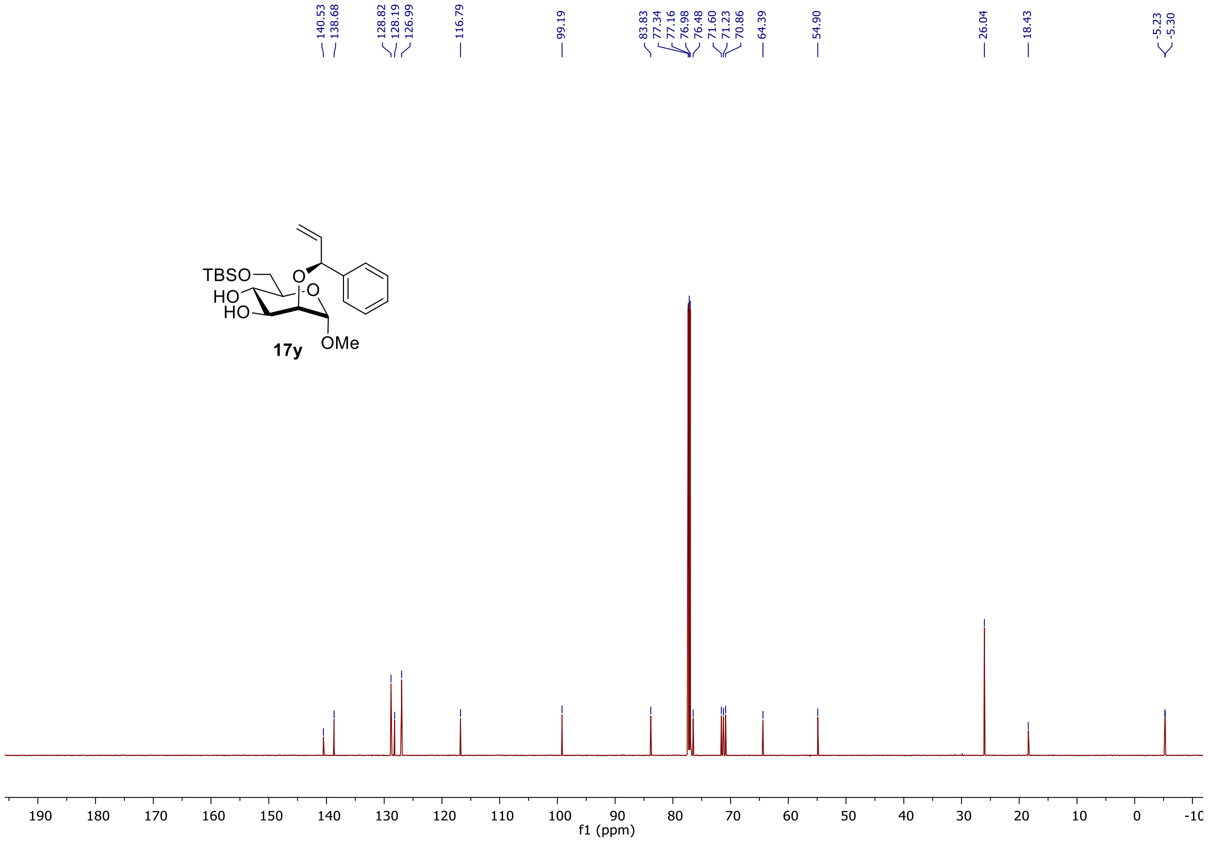

Supplementary Figure 367. ^{13}C spectra for **17y**

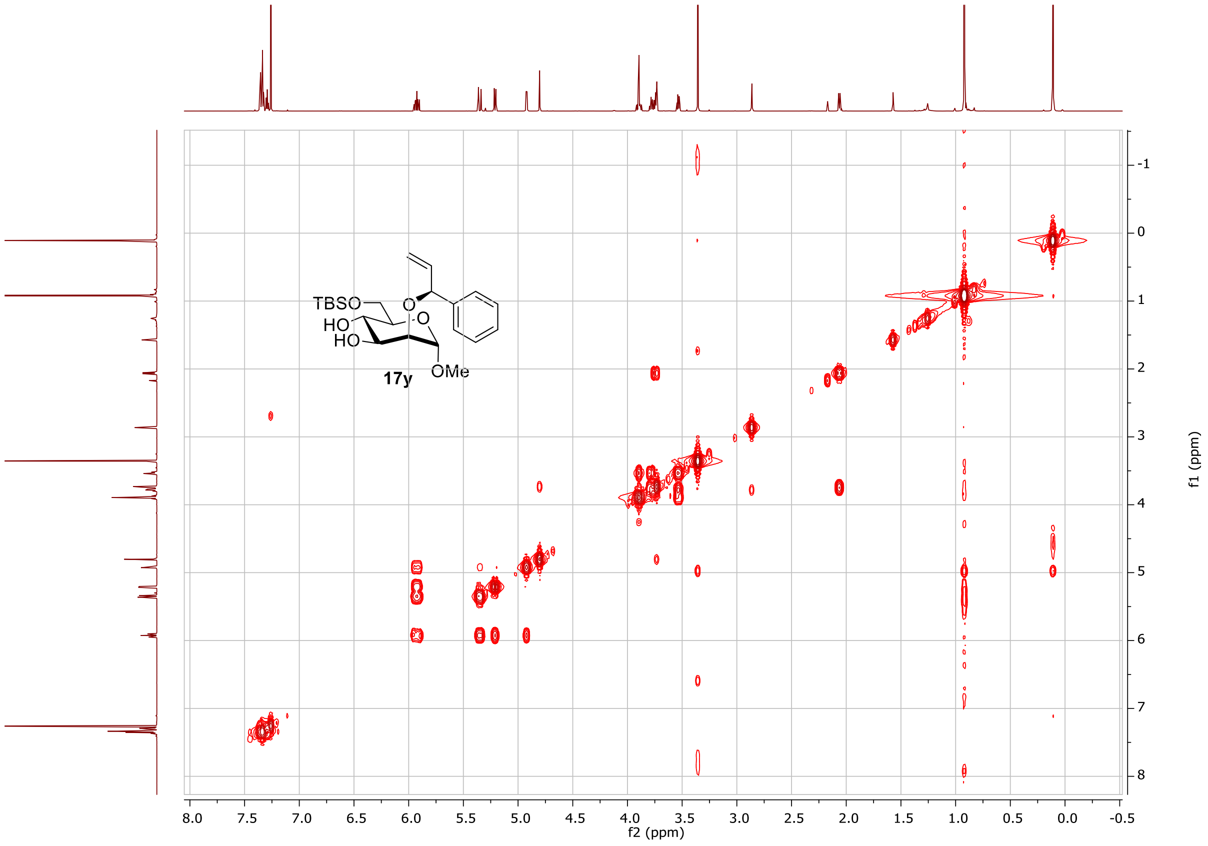

Supplementary Figure 368. COSY spectra for **17y**

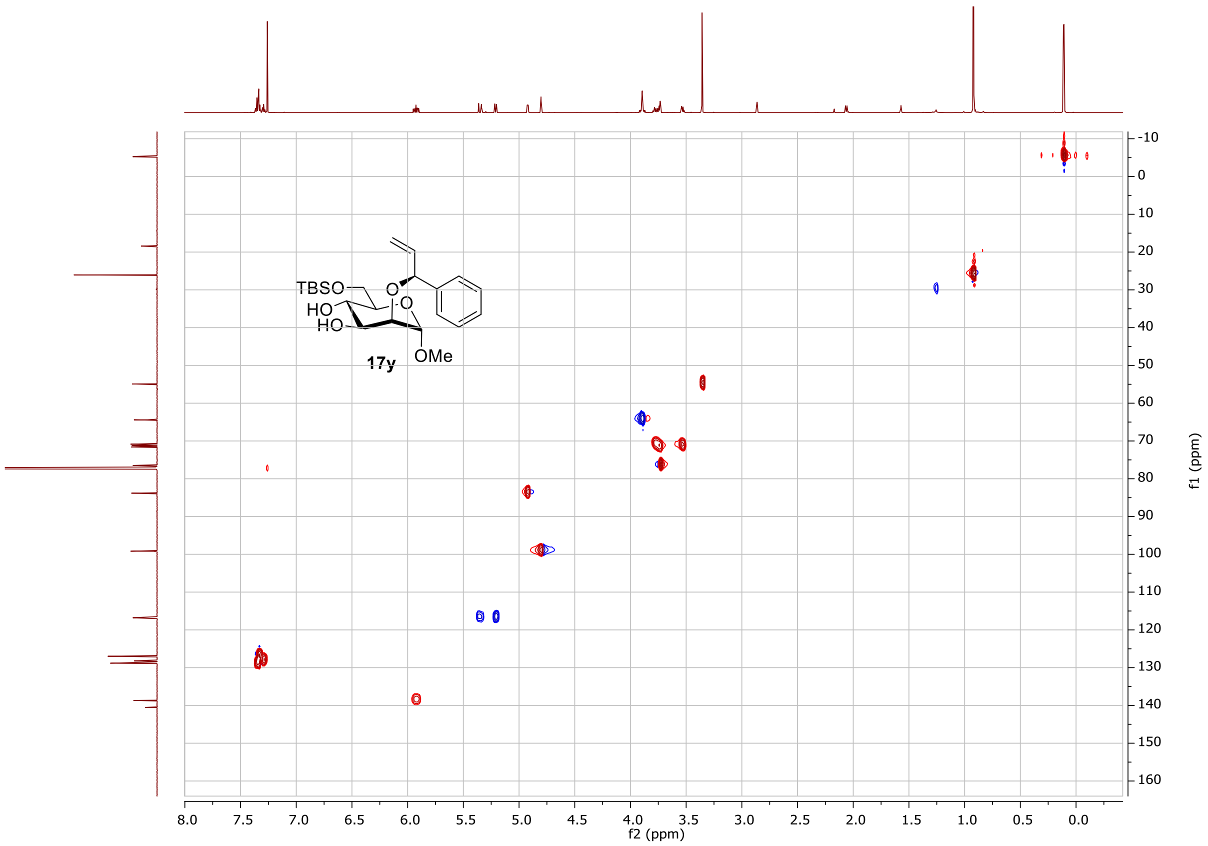

Supplementary Figure 369. HSQC spectra for **17y**

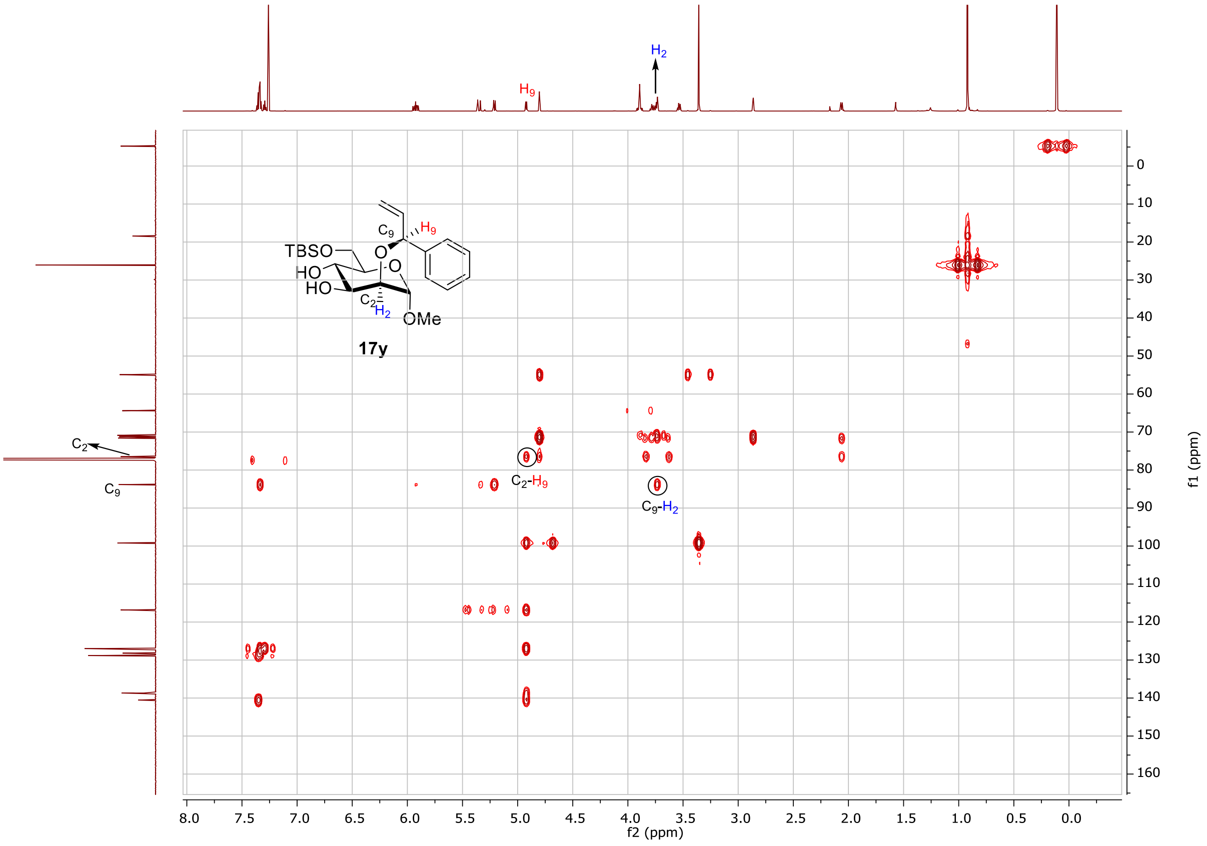

Supplementary Figure 370. HMBC spectra for **17y**

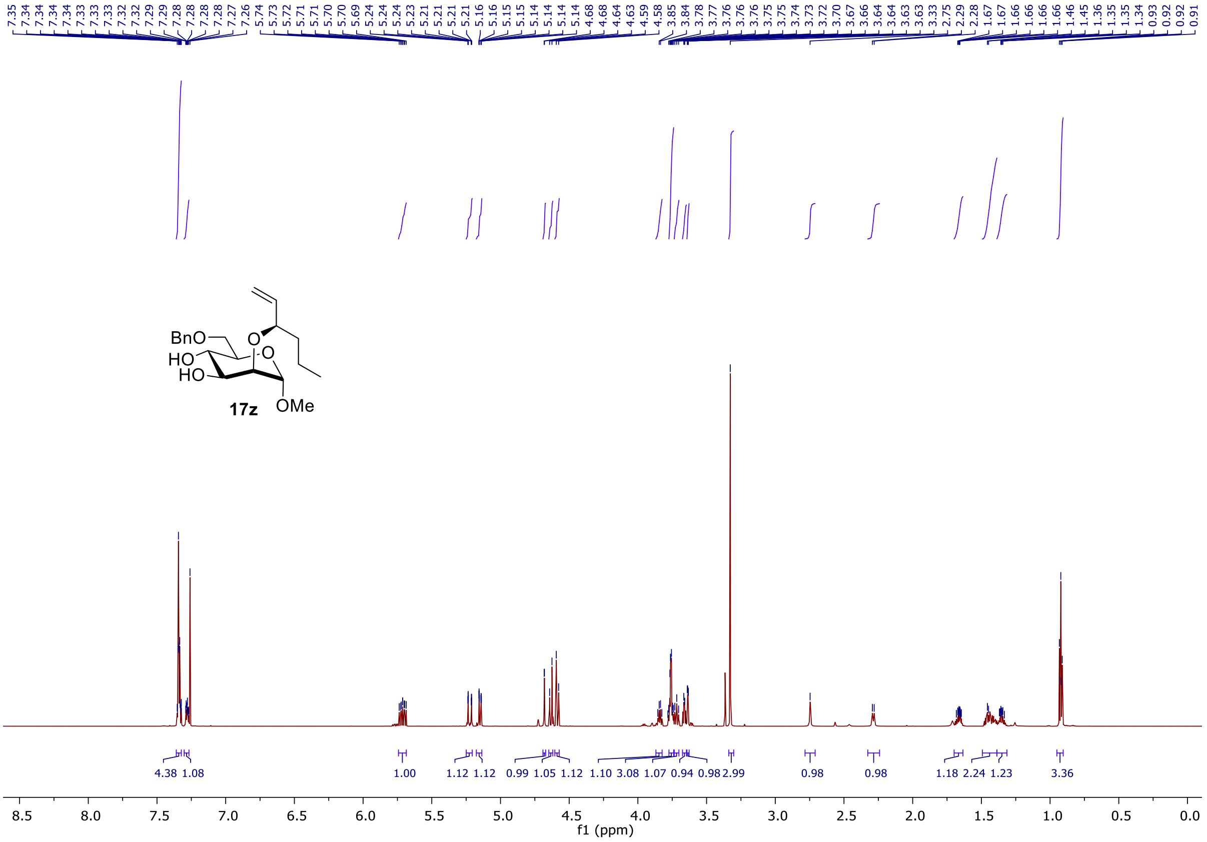

Supplementary Figure 371. ¹H spectra for **17z**

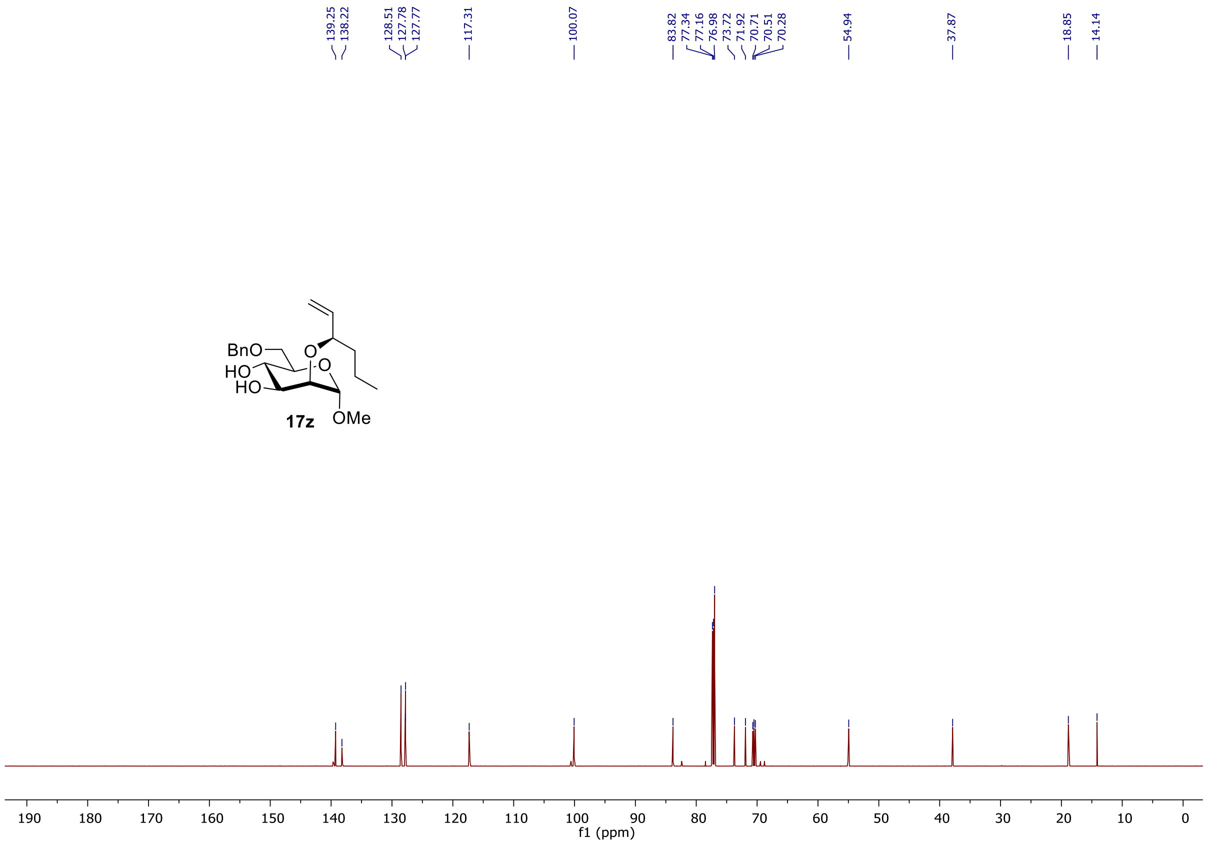

Supplementary Figure 372. ^{13}C spectra for **17z**

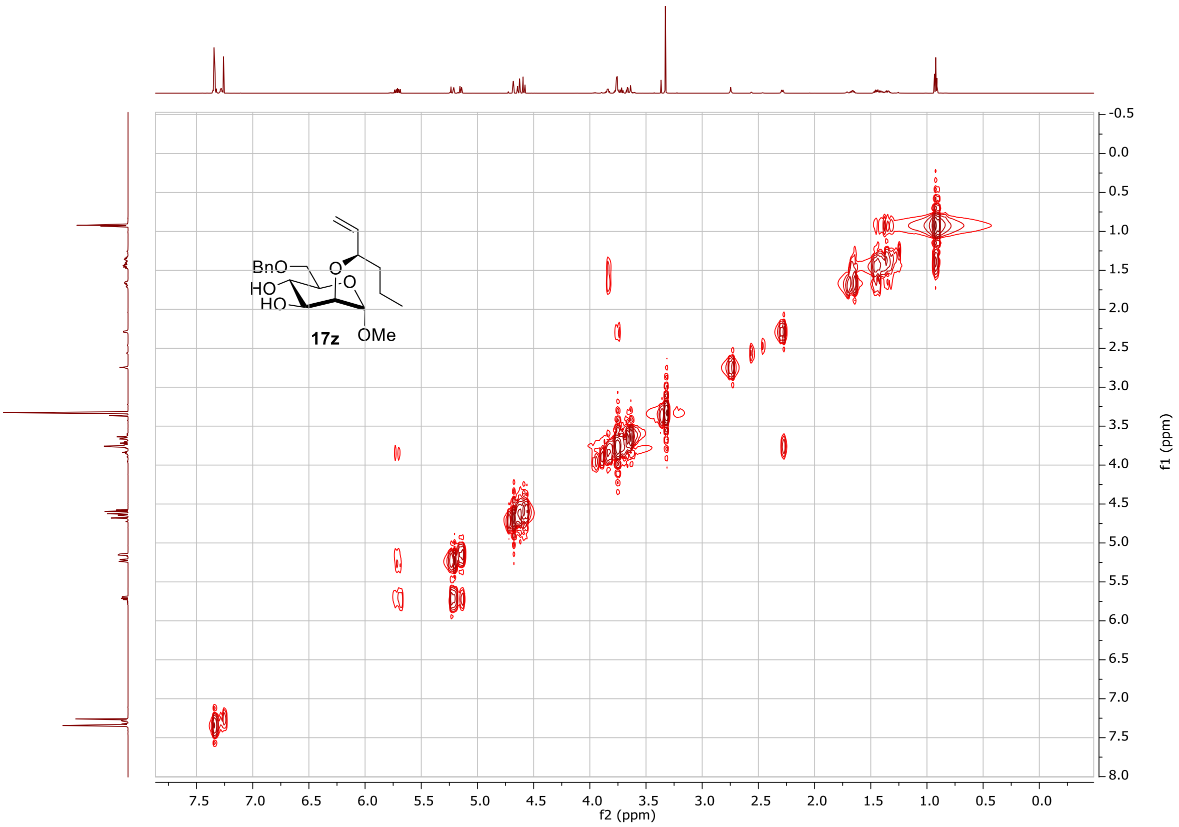

Supplementary Figure 373. COSY spectra for **17z**

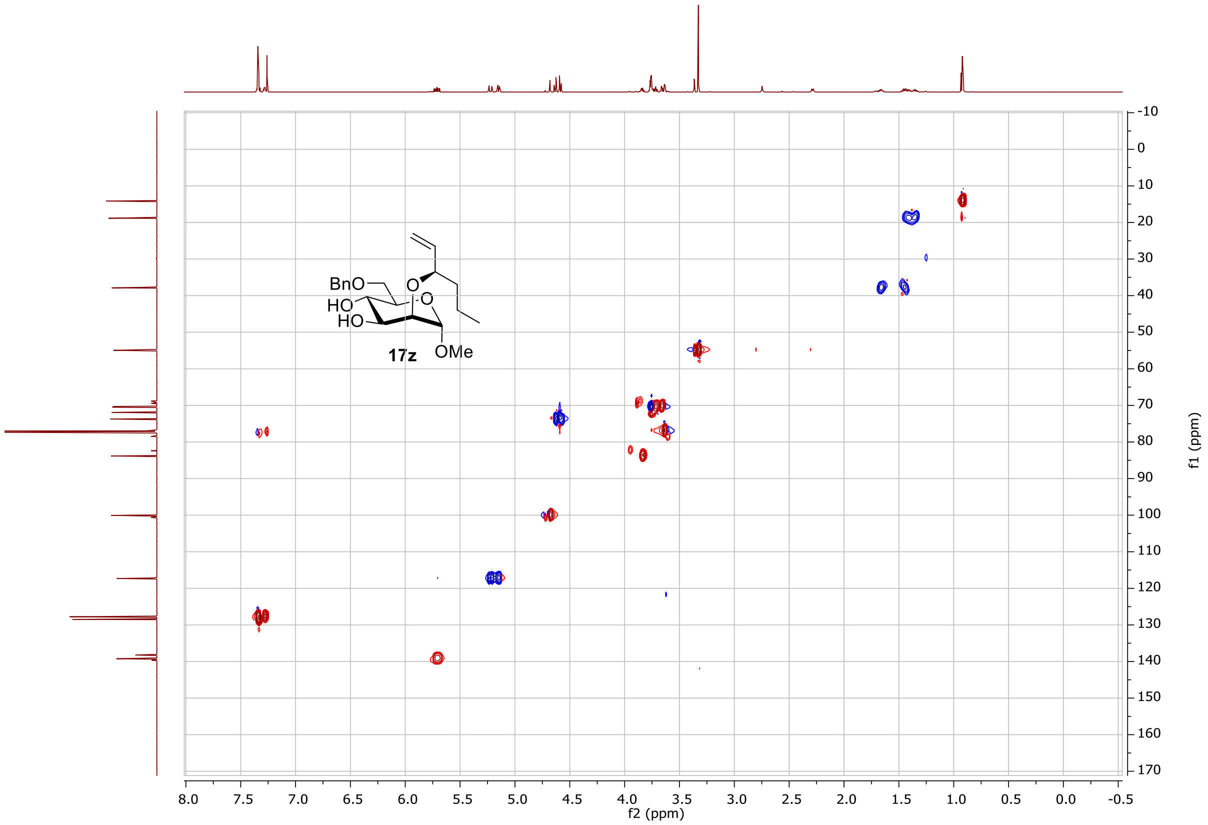

Supplementary Figure 374. HSQC spectra for **17z**

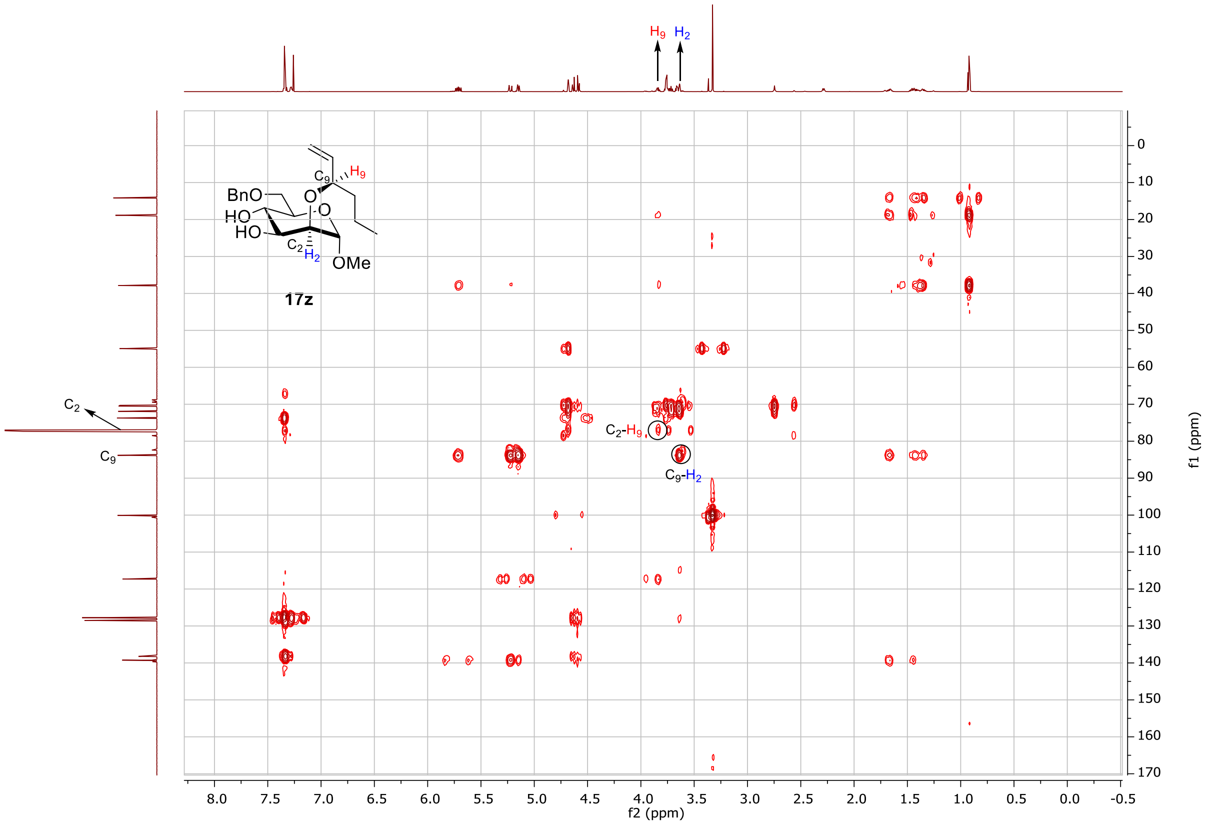

Supplementary Figure 375. HMBC spectra for **17z**

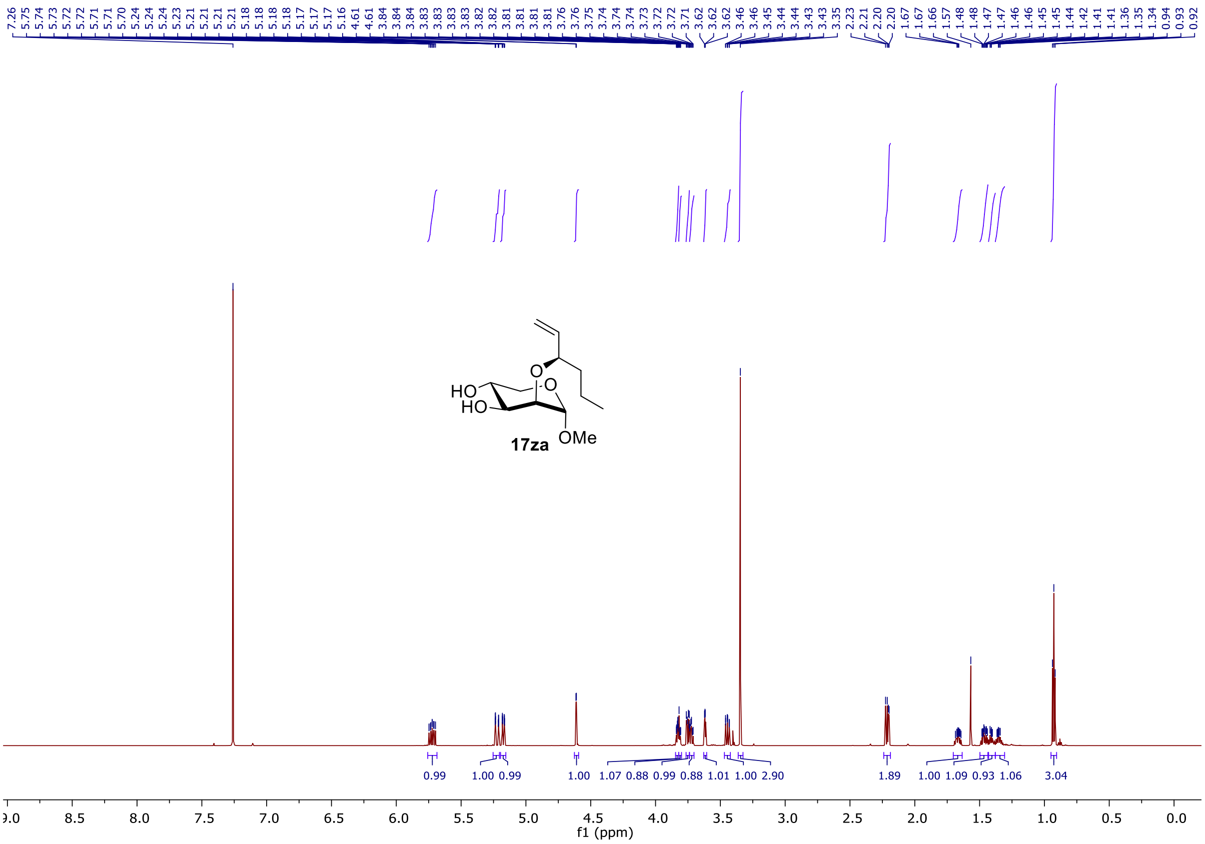

Supplementary Figure 376. ^{1}H spectra for **17za**

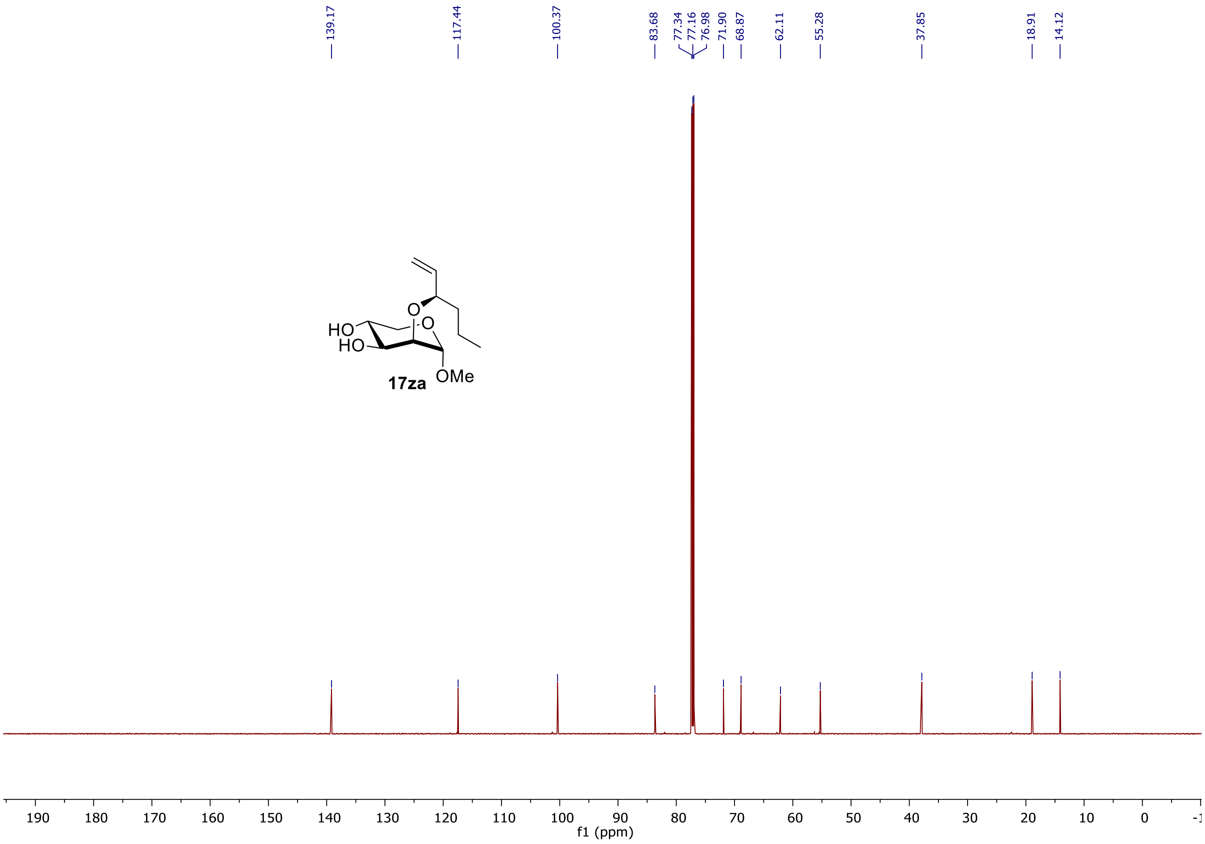

Supplementary Figure 377. ^{13}C spectra for **17za**

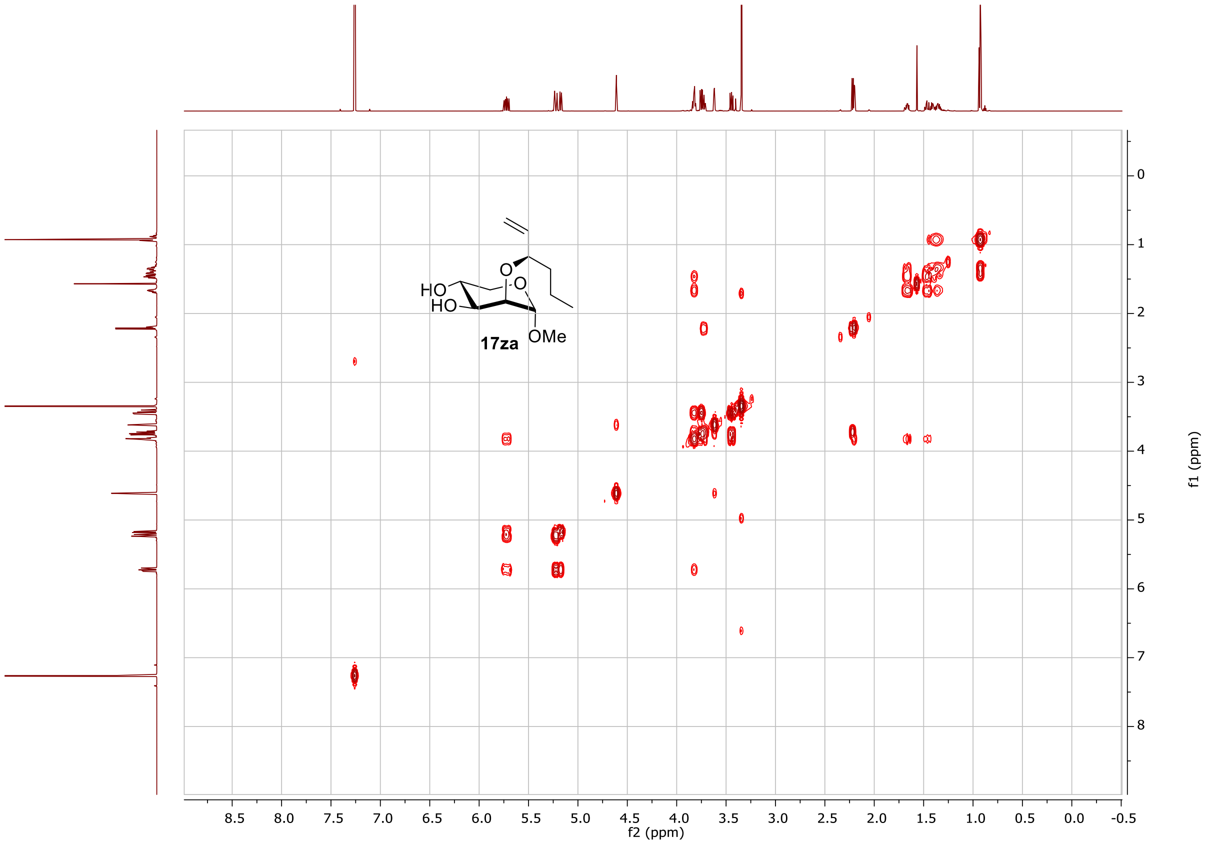

Supplementary Figure 378. COSY spectra for **17za**

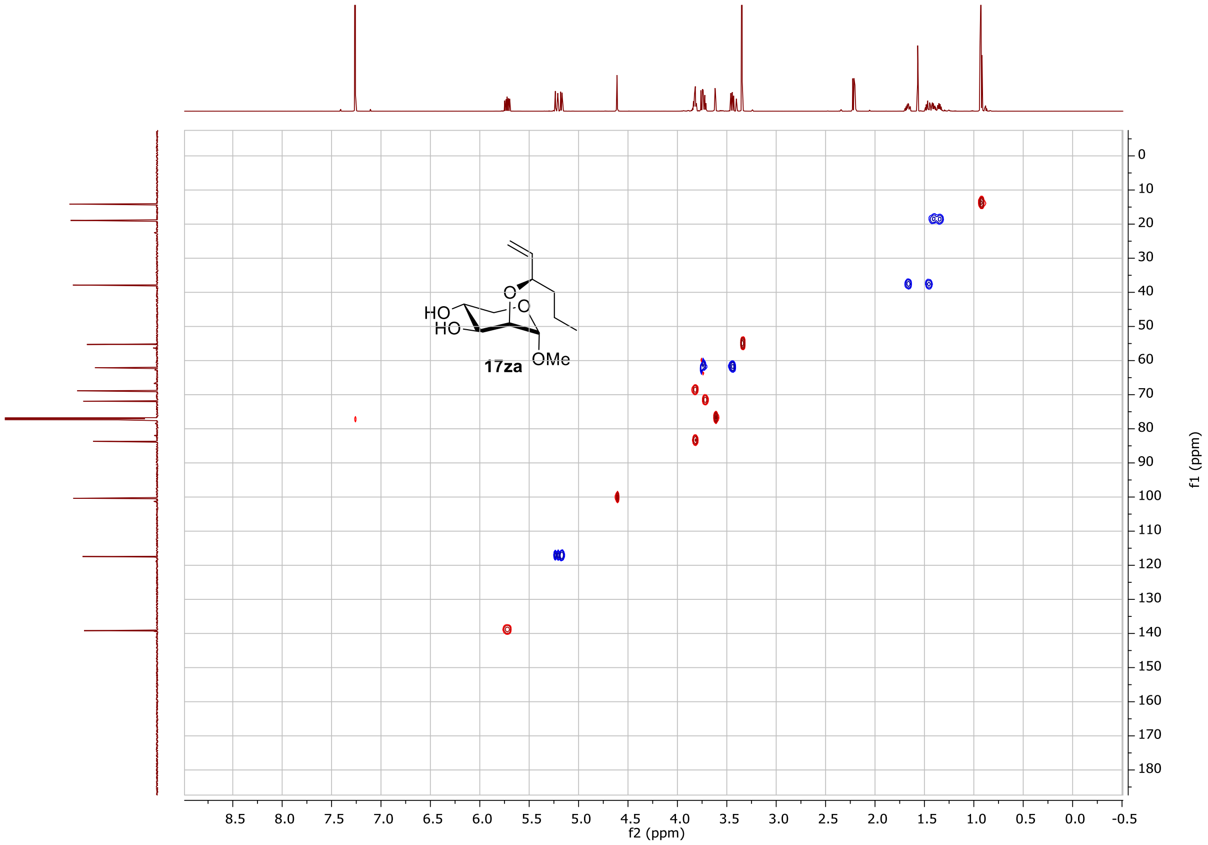

Supplementary Figure 379. HSQC spectra for **17za**

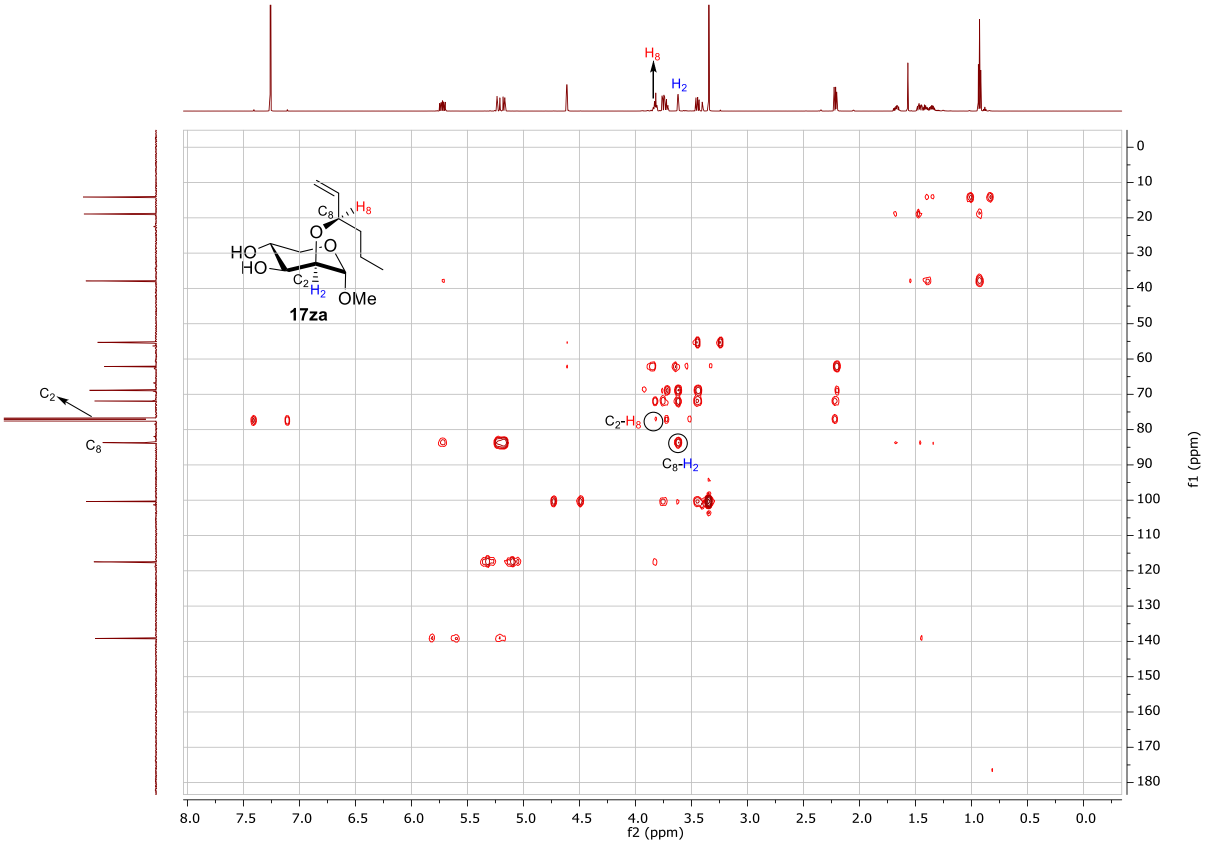

Supplementary Figure 380. HMBC spectra for **17za**

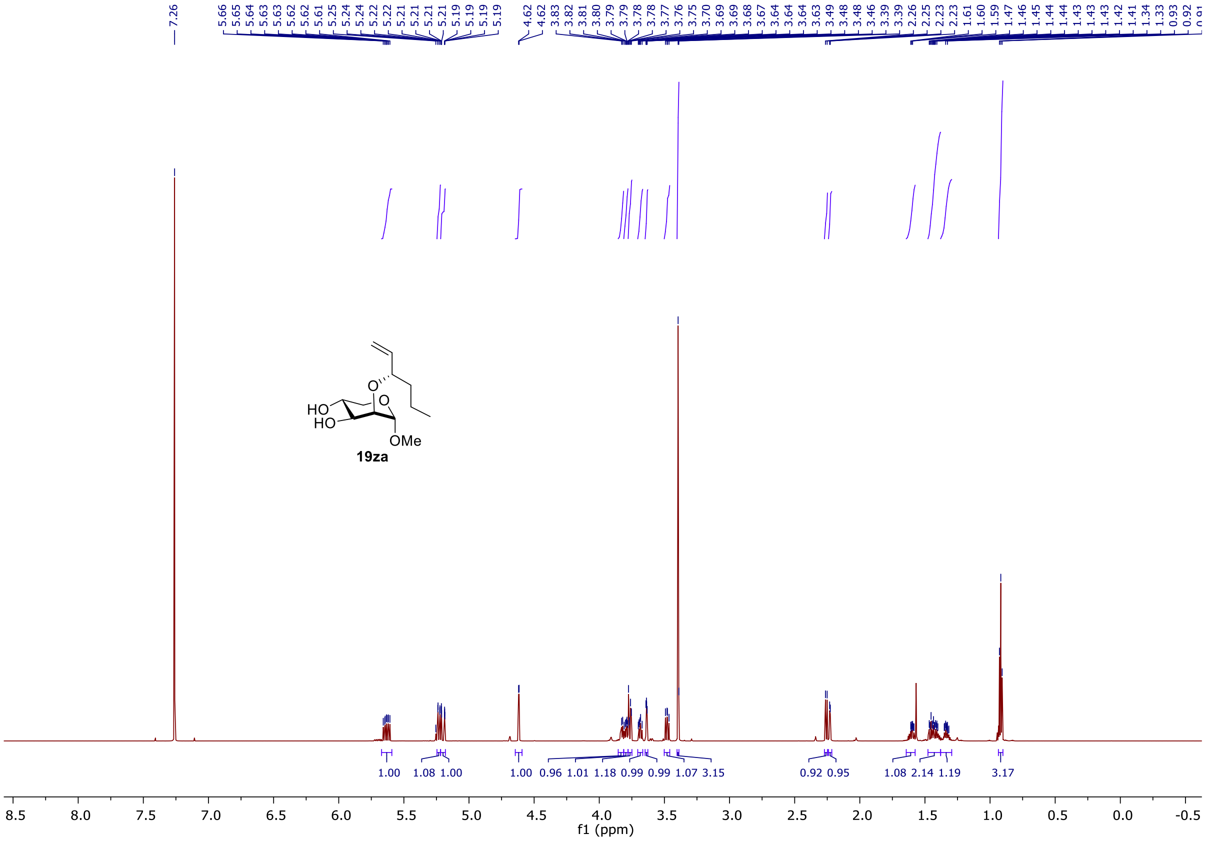

Supplementary Figure 381. ^{1}H spectra for **19za**

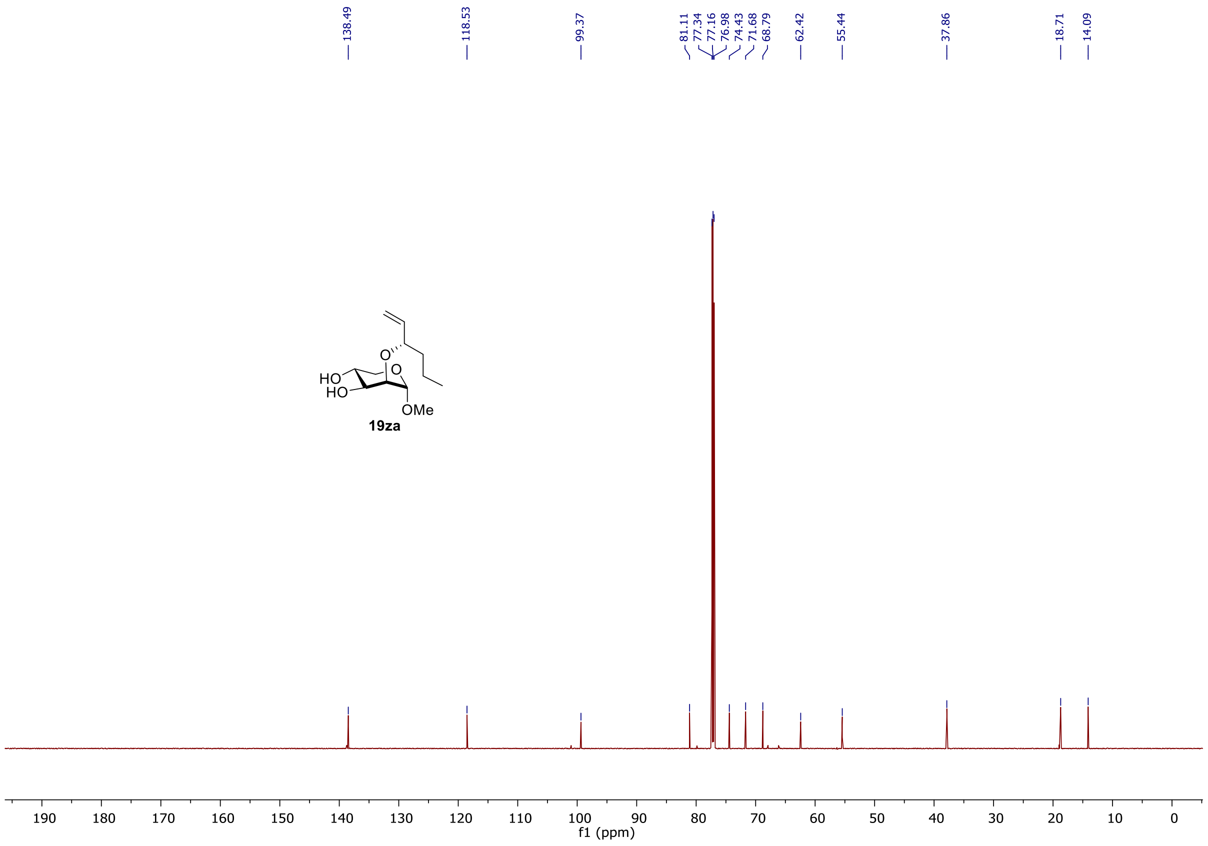

Supplementary Figure 382. ^{13}C spectra for **19za**

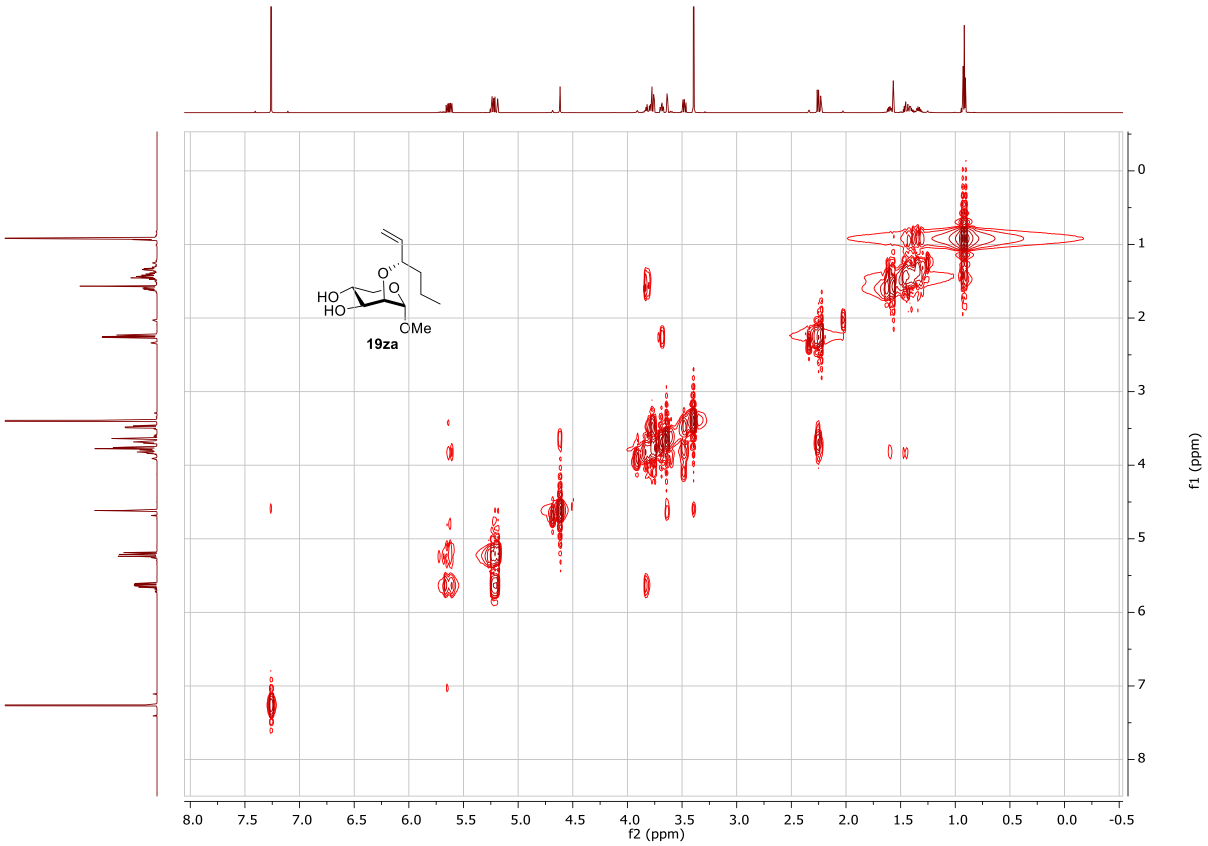

Supplementary Figure 383. COSY spectra for **19za**

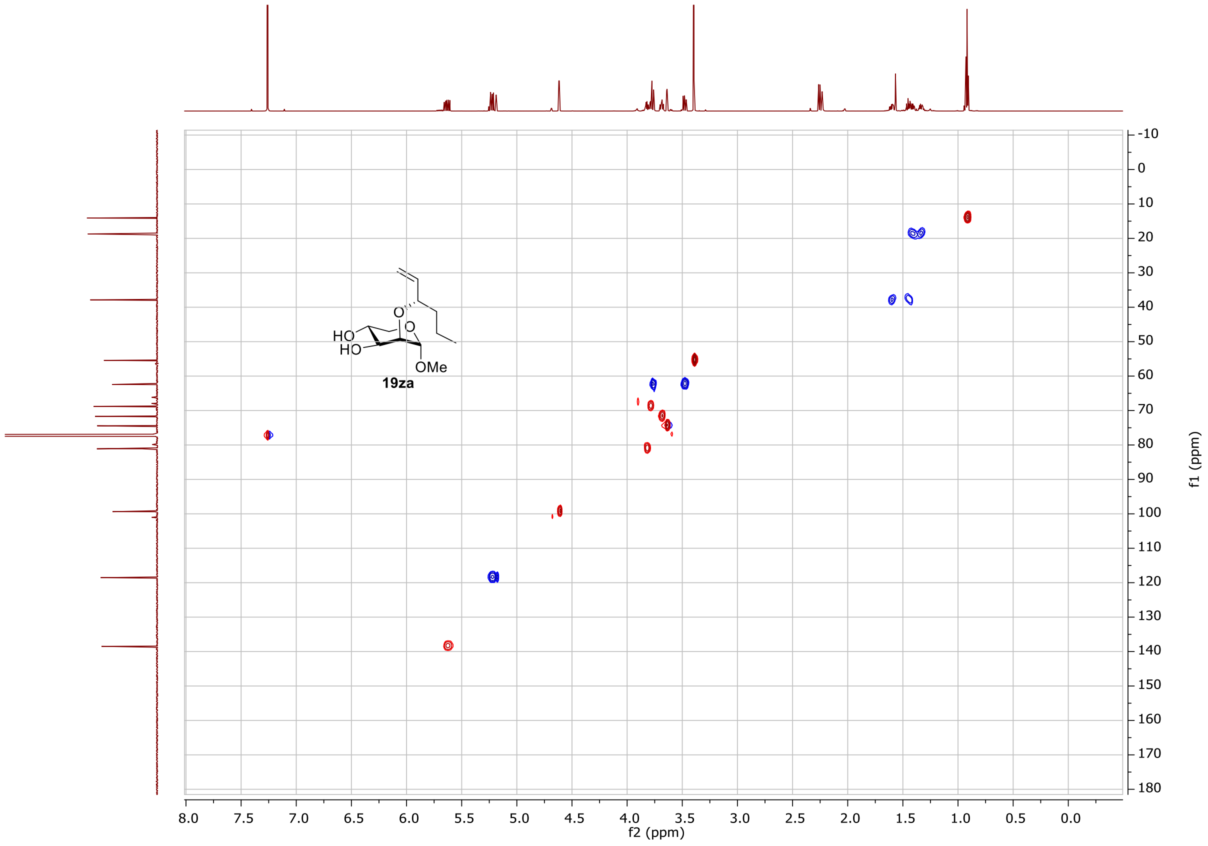

Supplementary Figure 384. HSQC spectra for **19za**

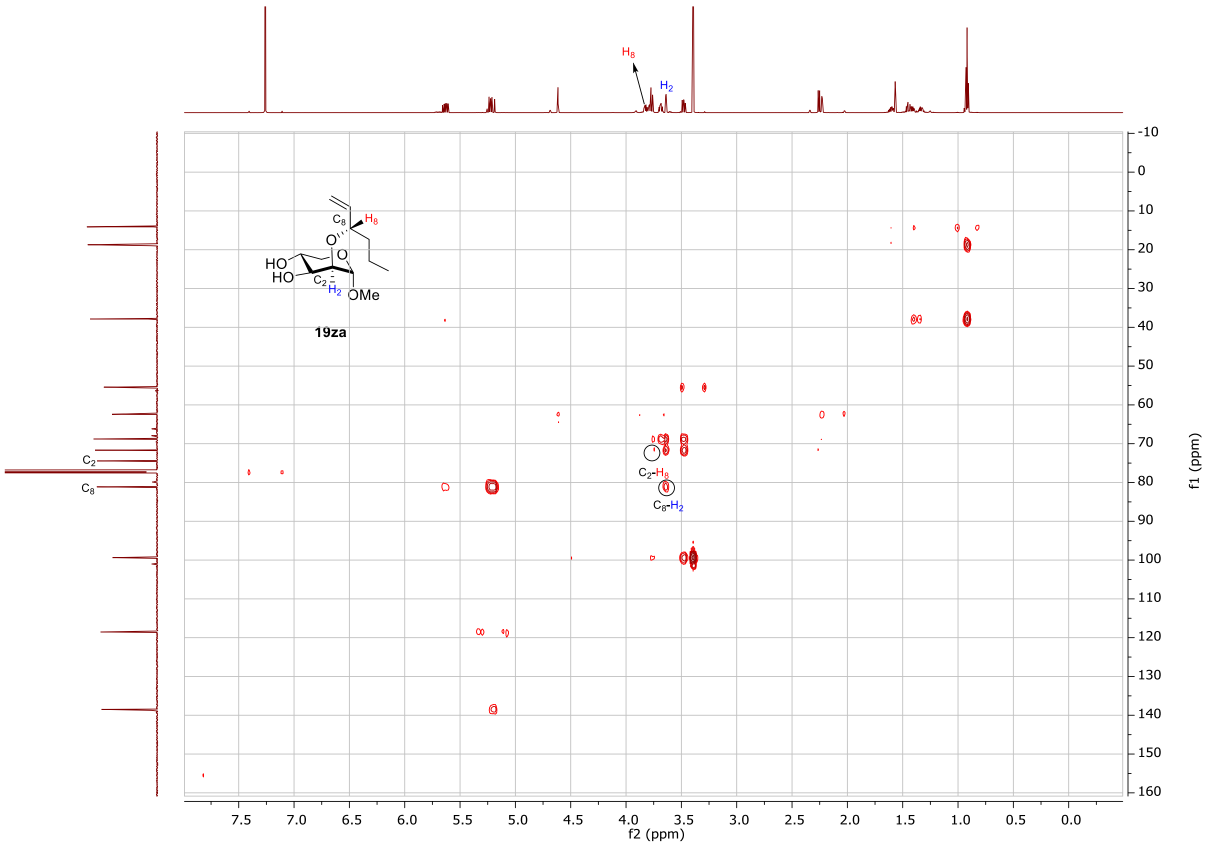

Supplementary Figure 385. HMBC spectra for **19za**

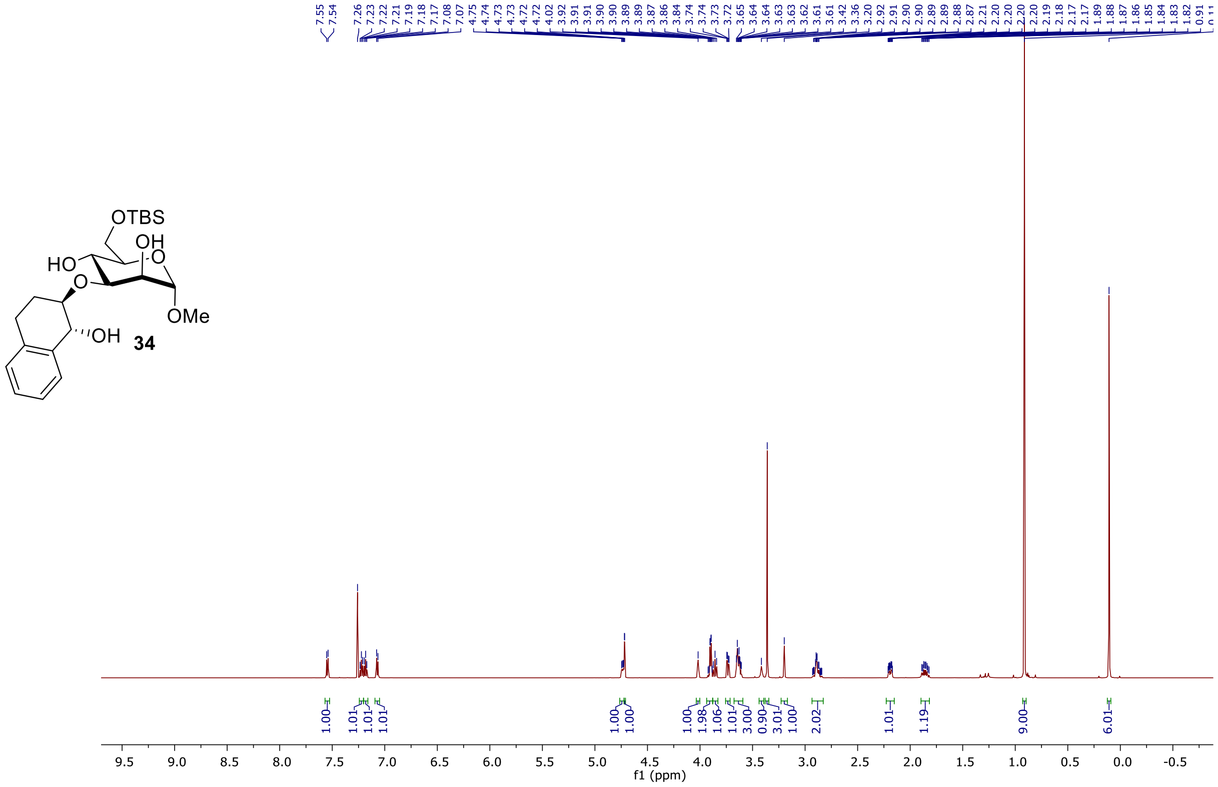

Supplementary Figure 386. ^{1}H spectra for **34**

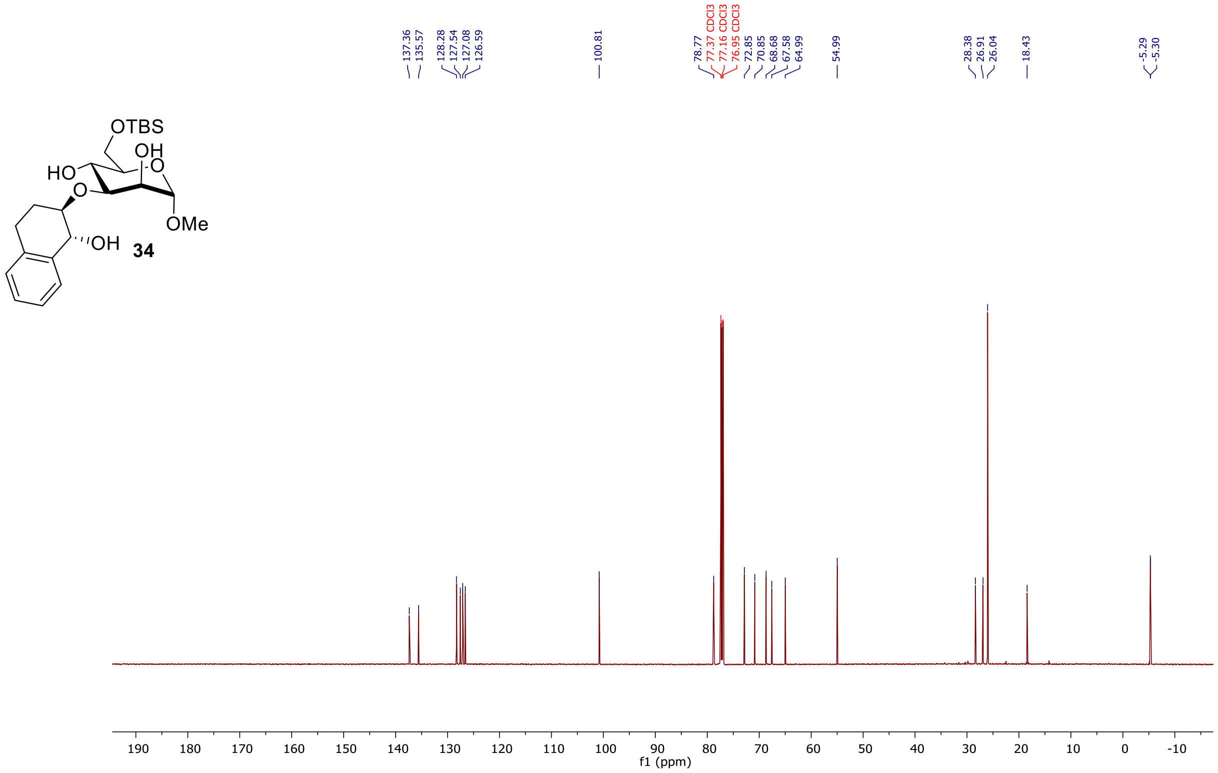

Supplementary Figure 387. ^{13}C spectra for **34**

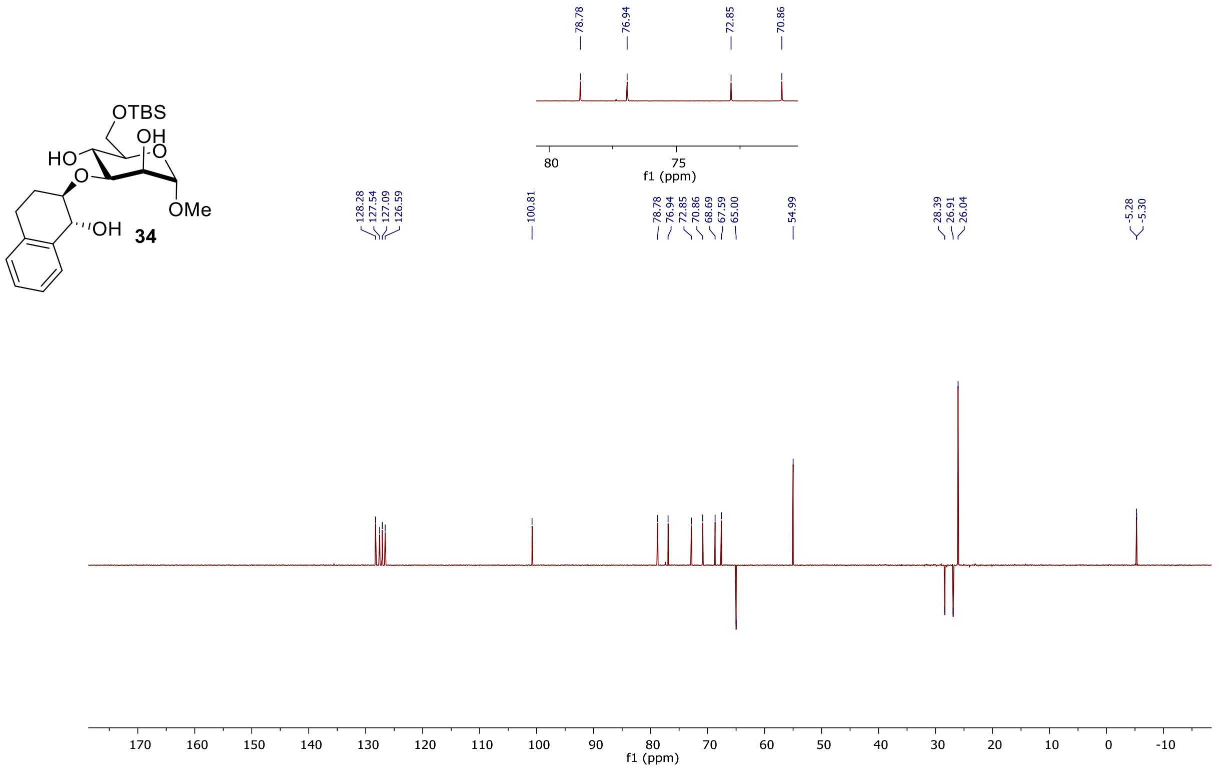

Supplementary Figure 388. DEPT spectra for **34**

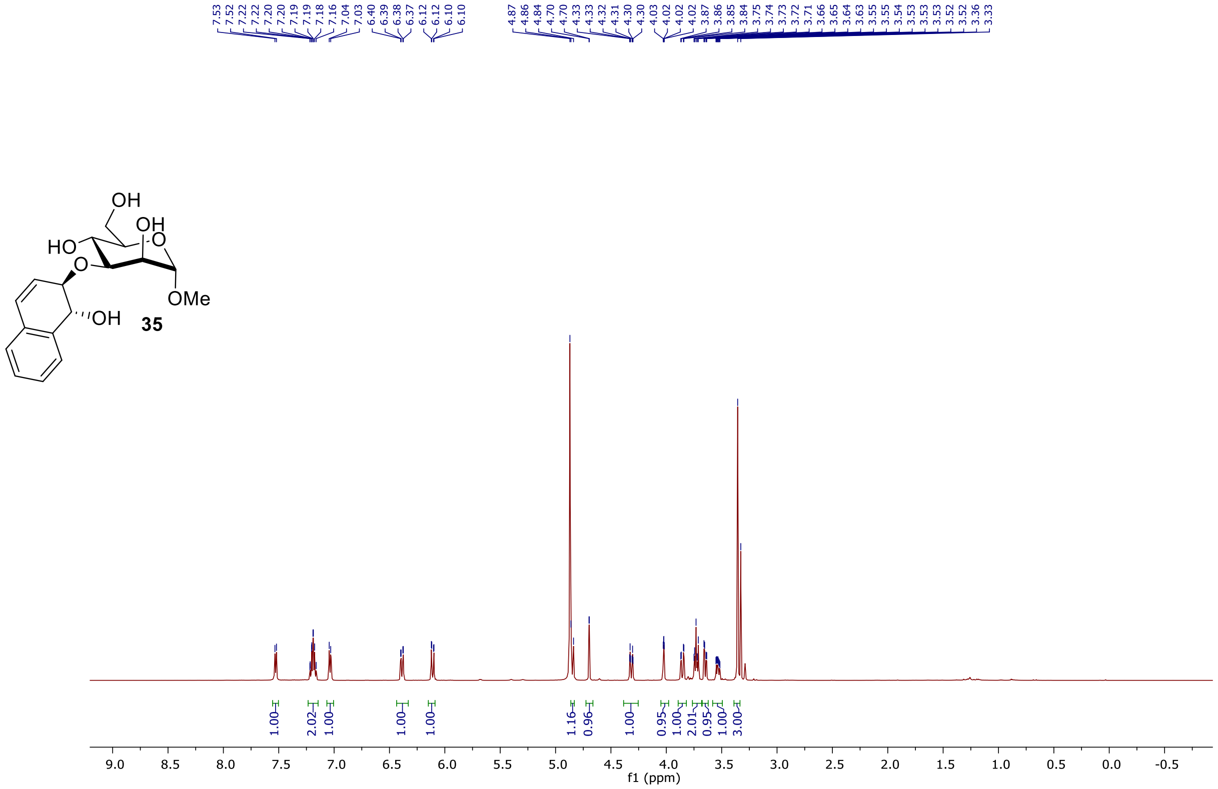

Supplementary Figure 389. ^{1}H spectra for **35**

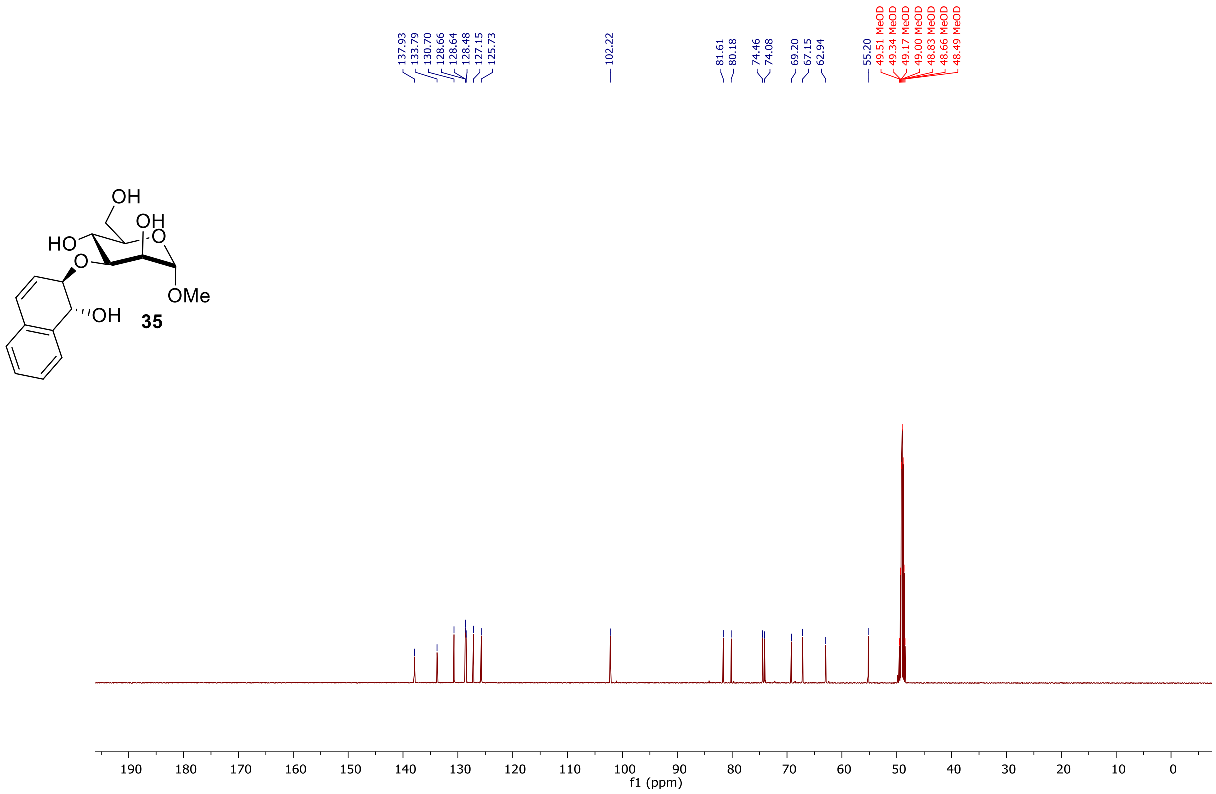

Supplementary Figure 390. ^{13}C spectra for **35**

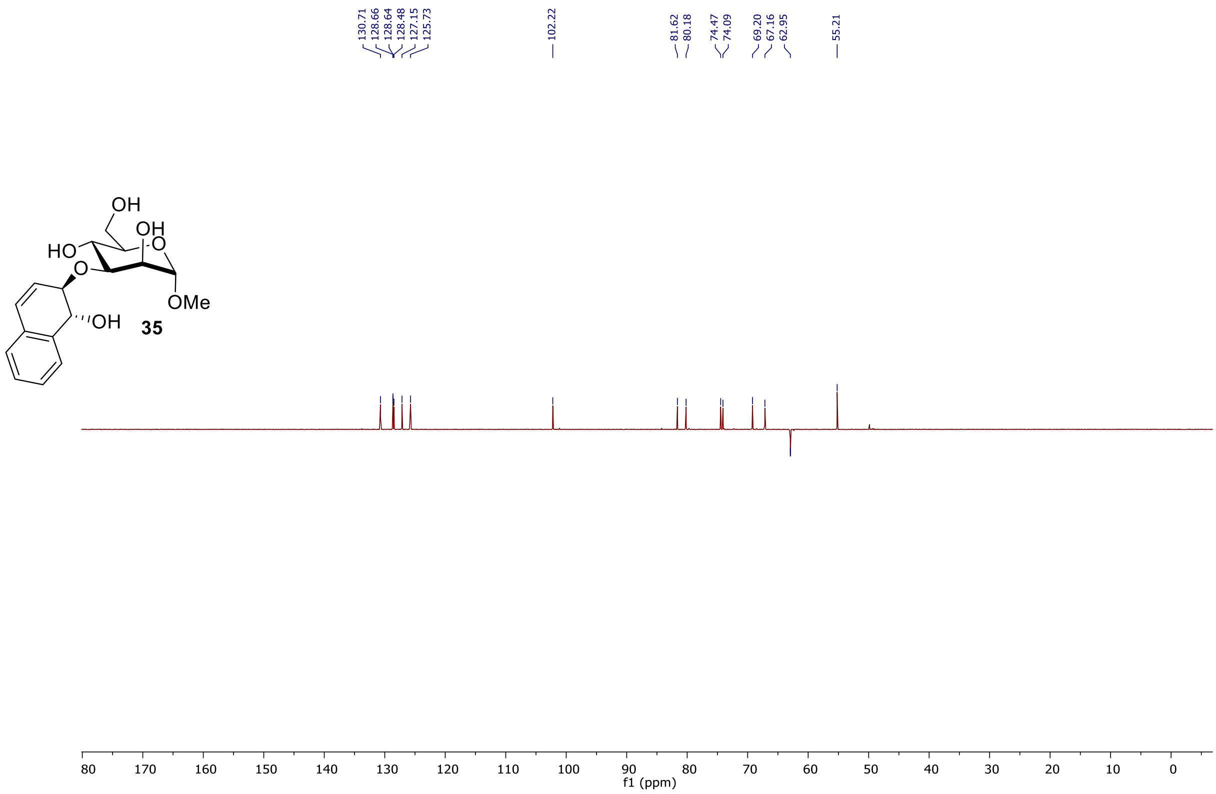

Supplementary Figure 391. ^{13}C spectra for **35**

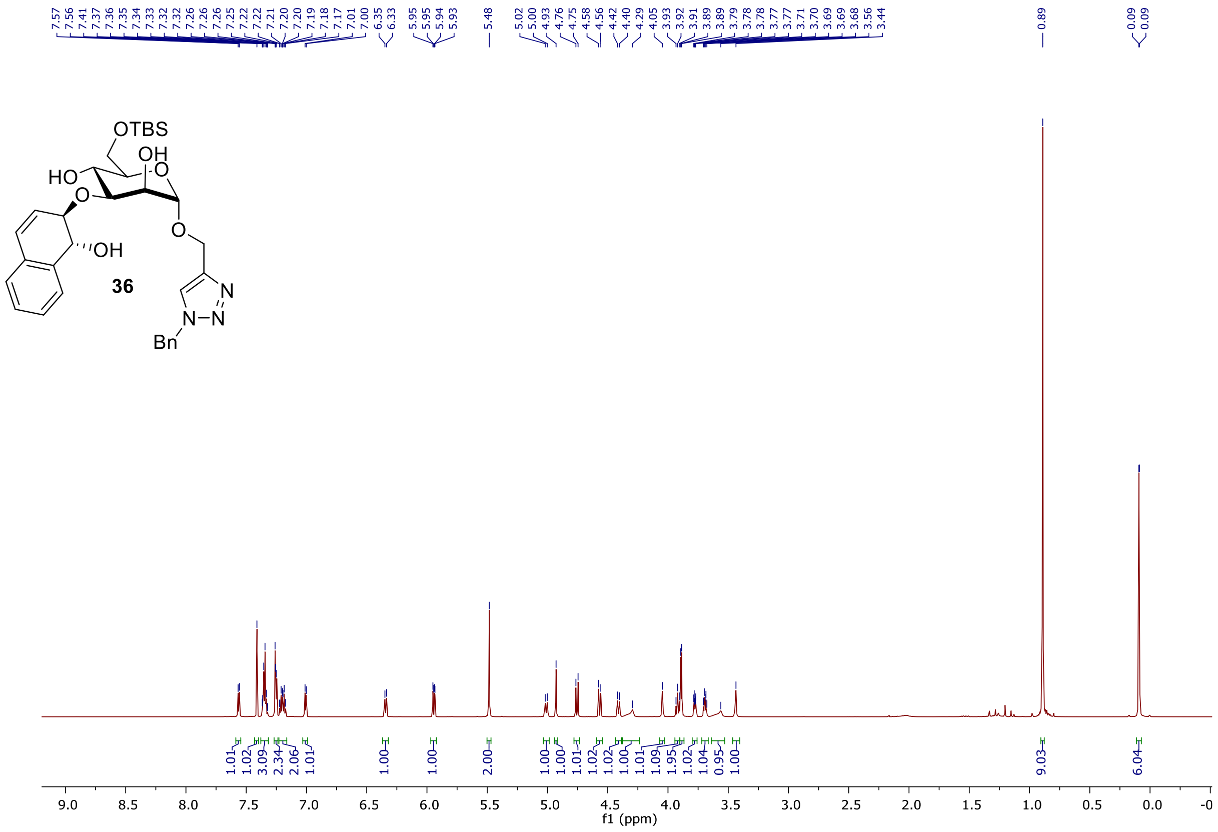

Supplementary Figure 392. ^{1}H spectra for **36**

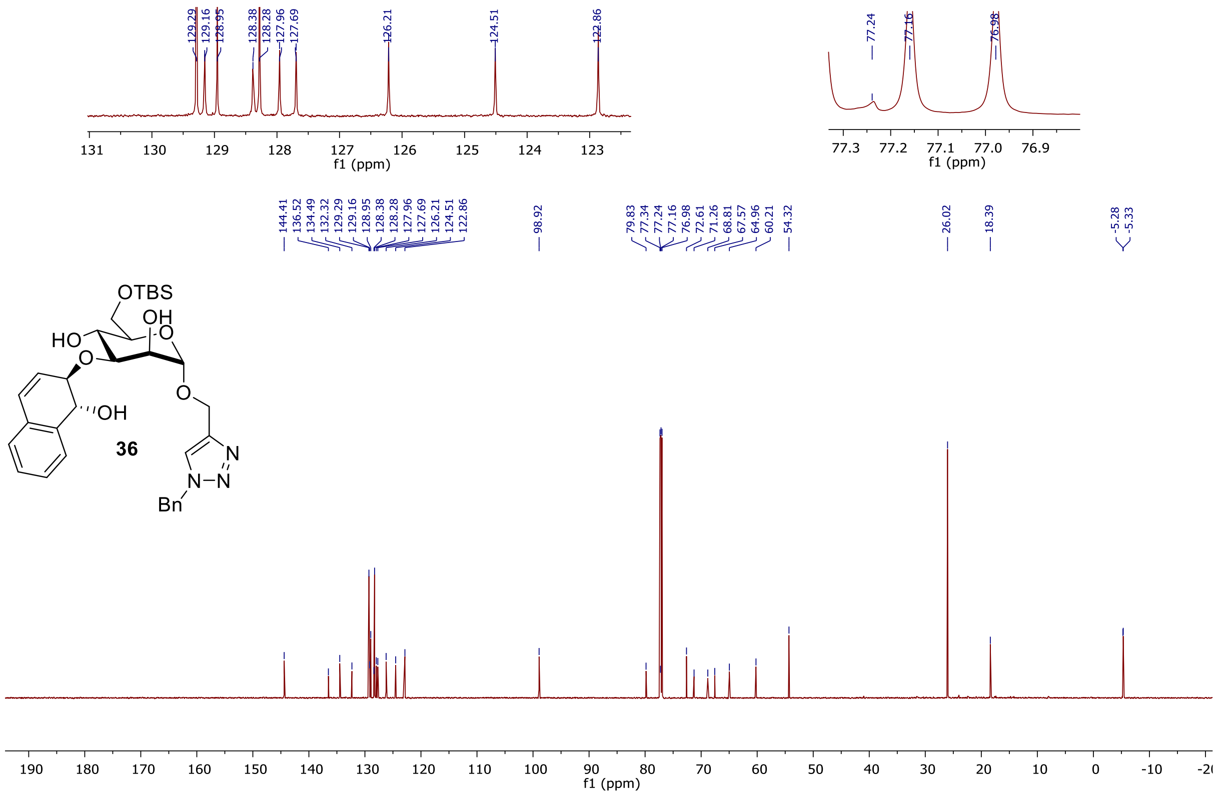

Supplementary Figure 393. ^{13}C spectra for **36**

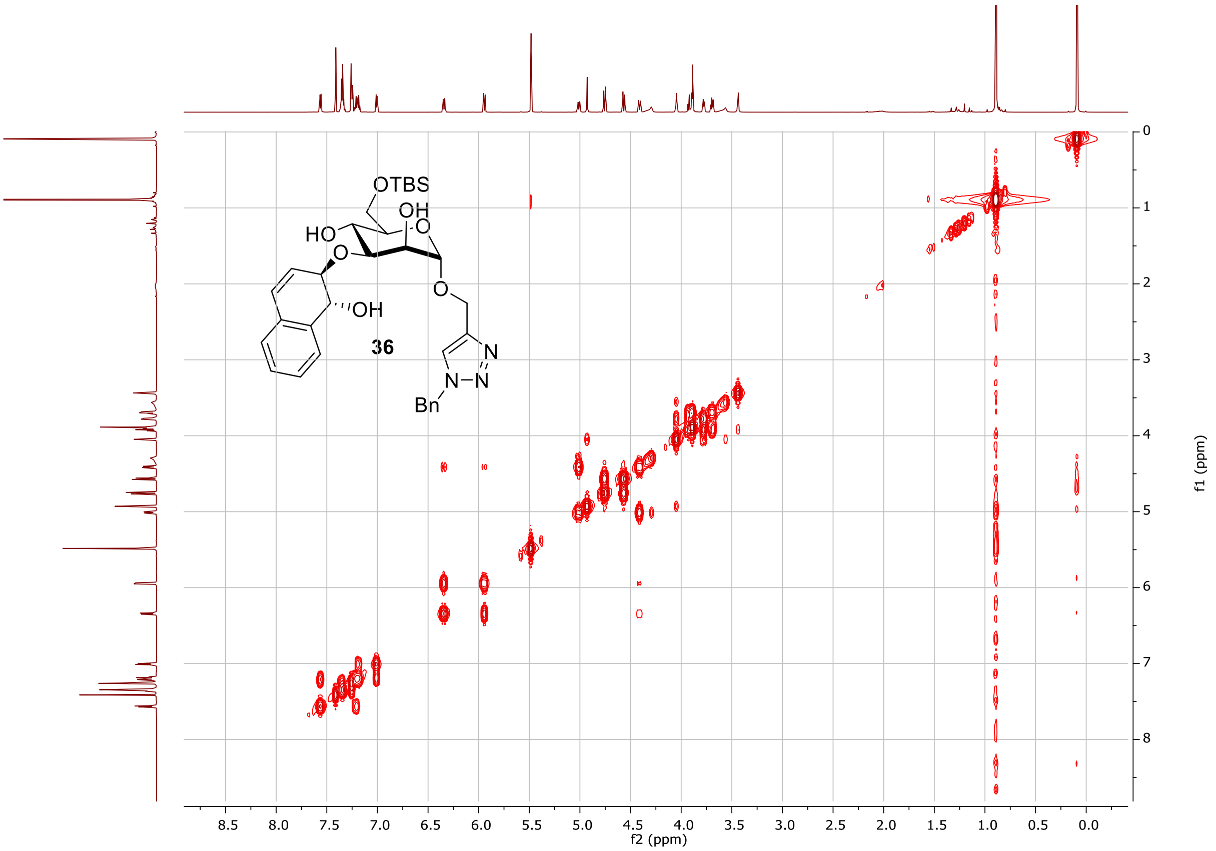

Supplementary Figure 394. COSY spectra for **36**

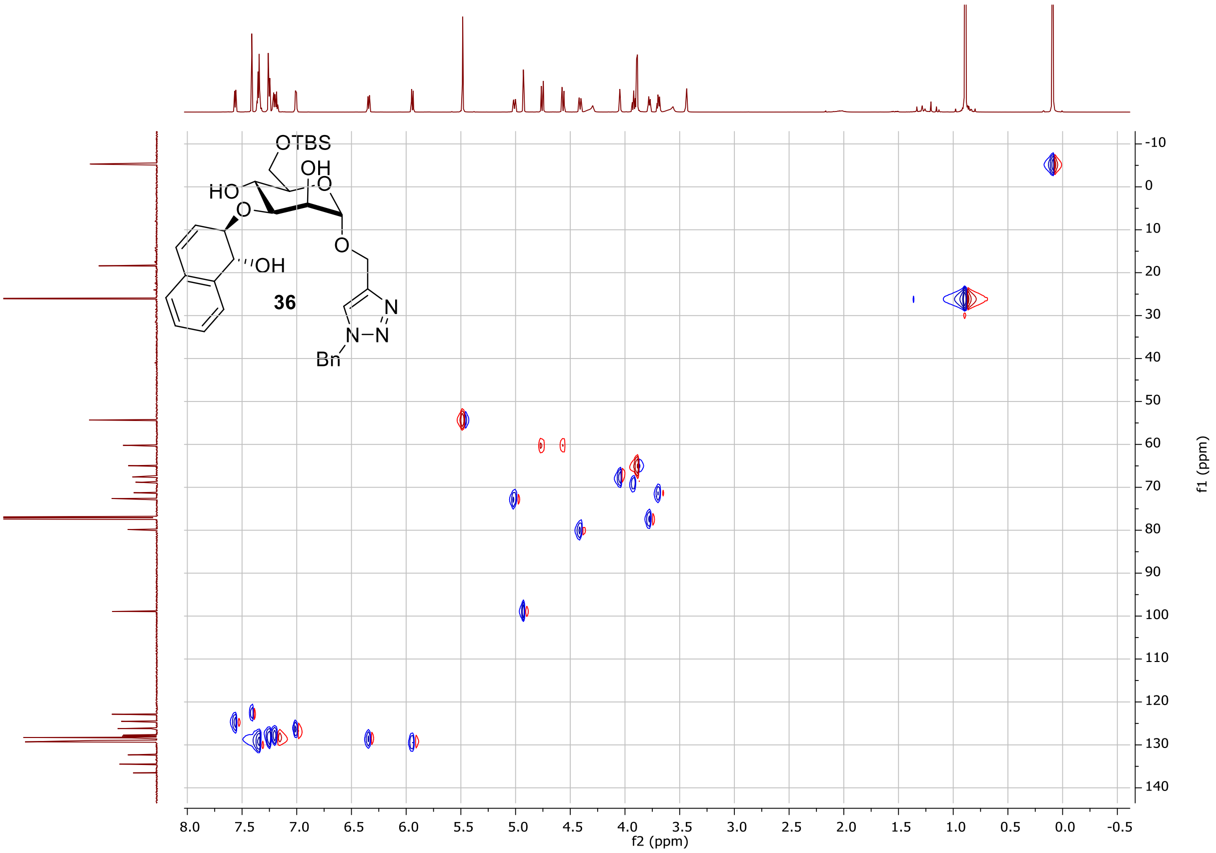

Supplementary Figure 395. HSQC spectra for **36**

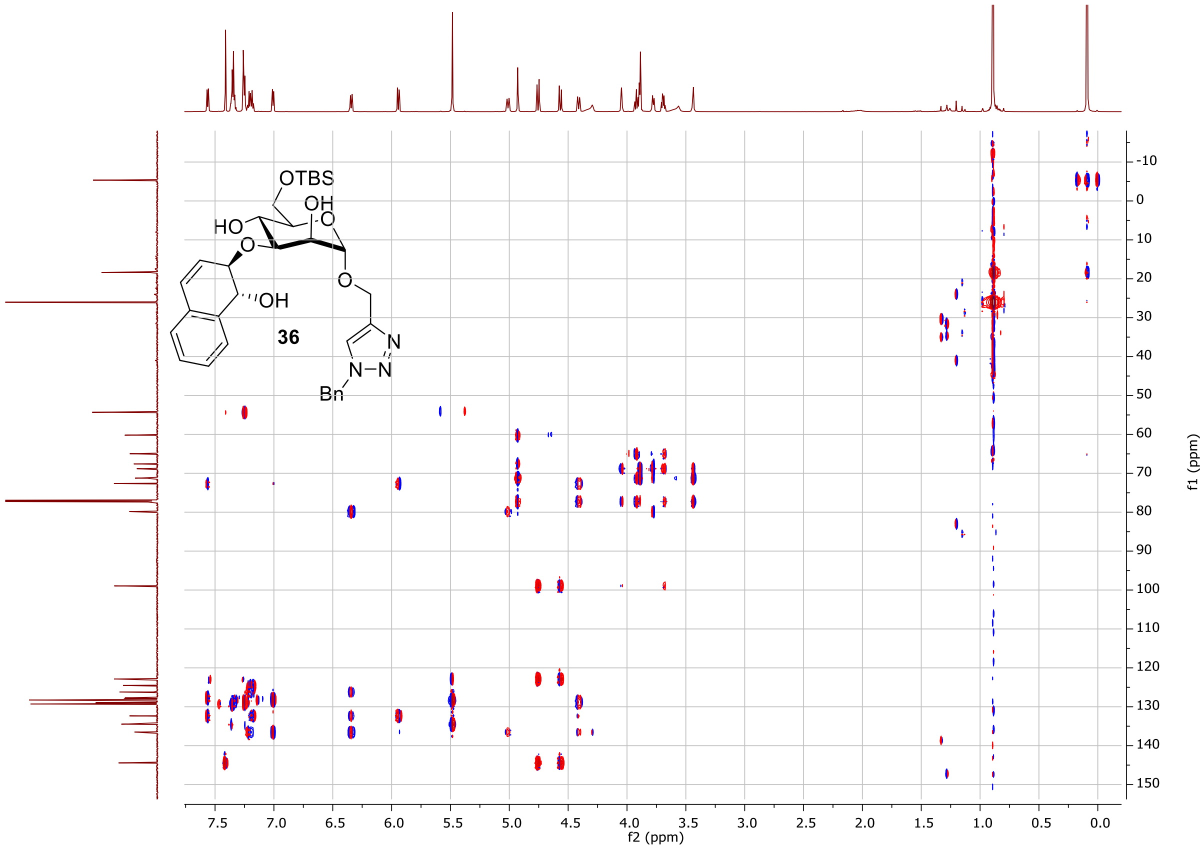

Supplementary Figure 396. HMBC spectra for **36**

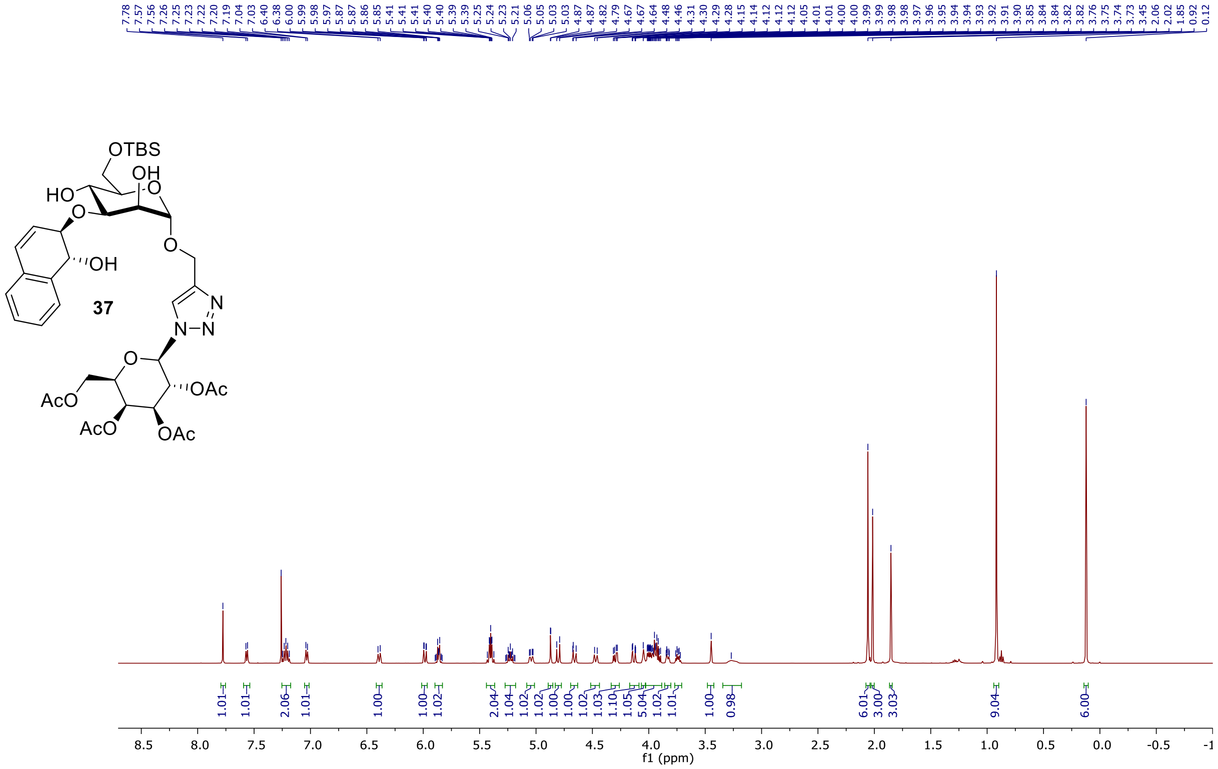

Supplementary Figure 397. ^{1}H spectra for **37**

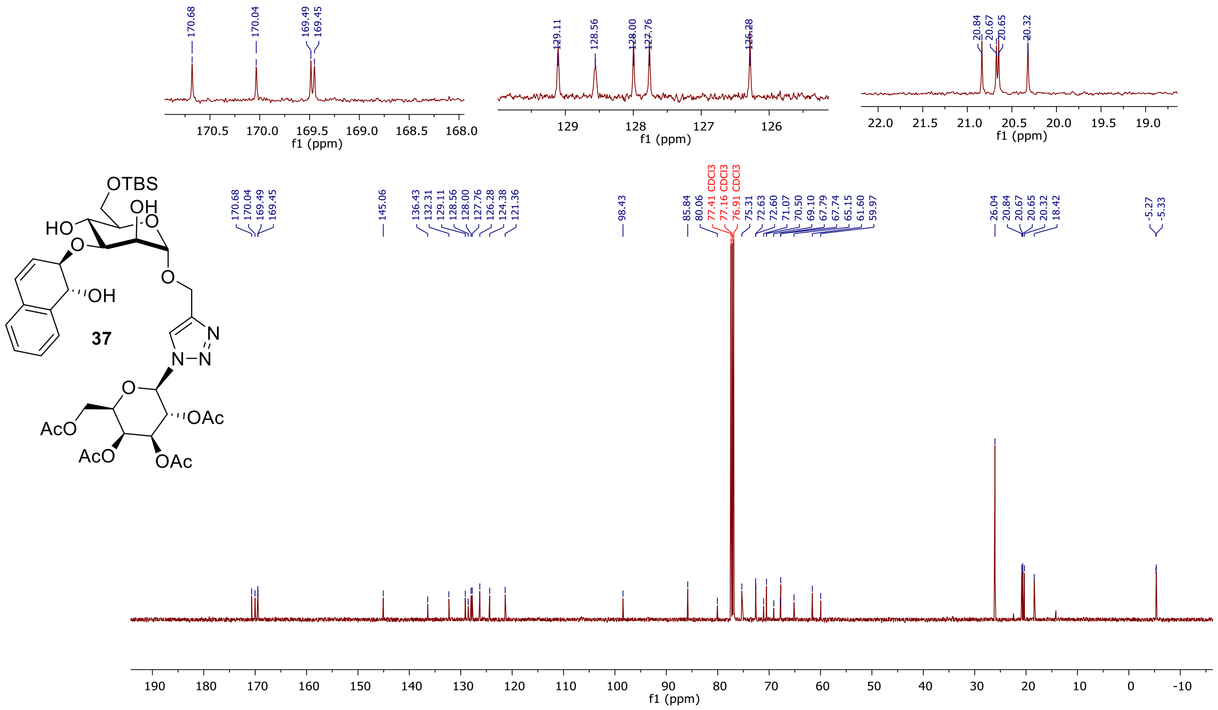

Supplementary Figure 398. ^{13}C spectra for **37**

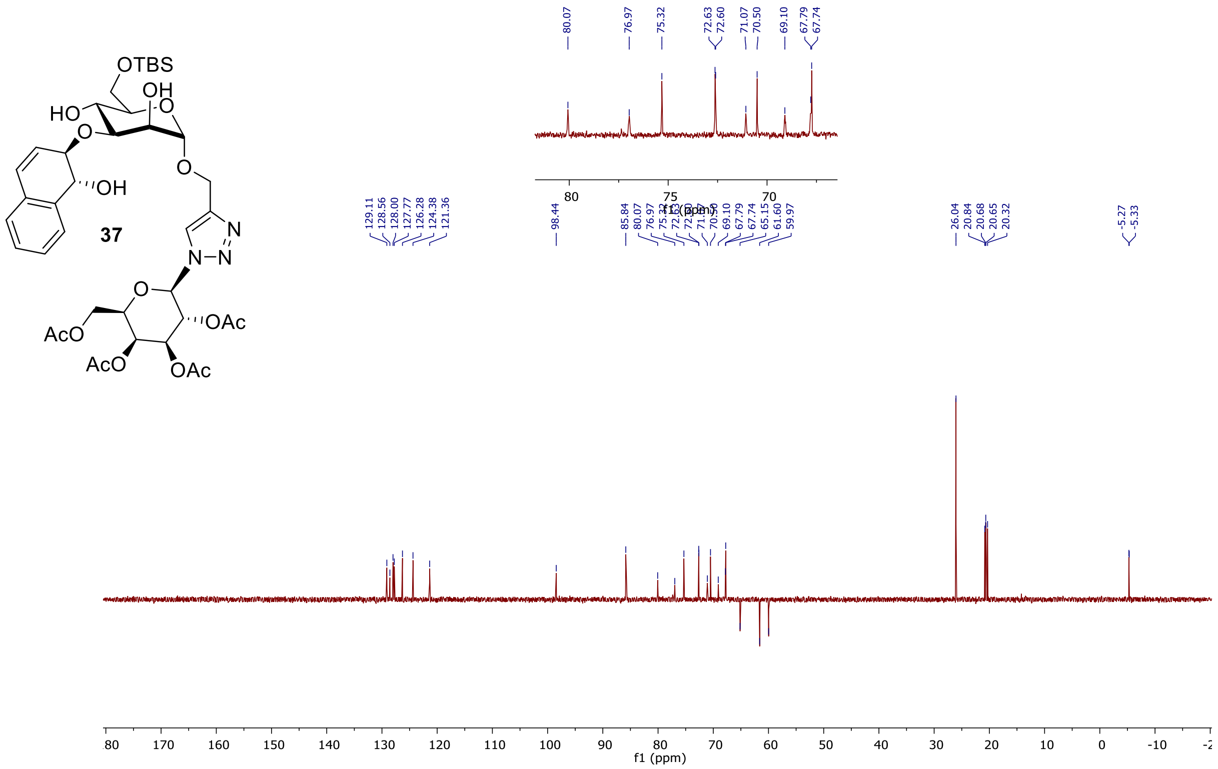

Supplementary Figure 399. DEPT spectra for **37**

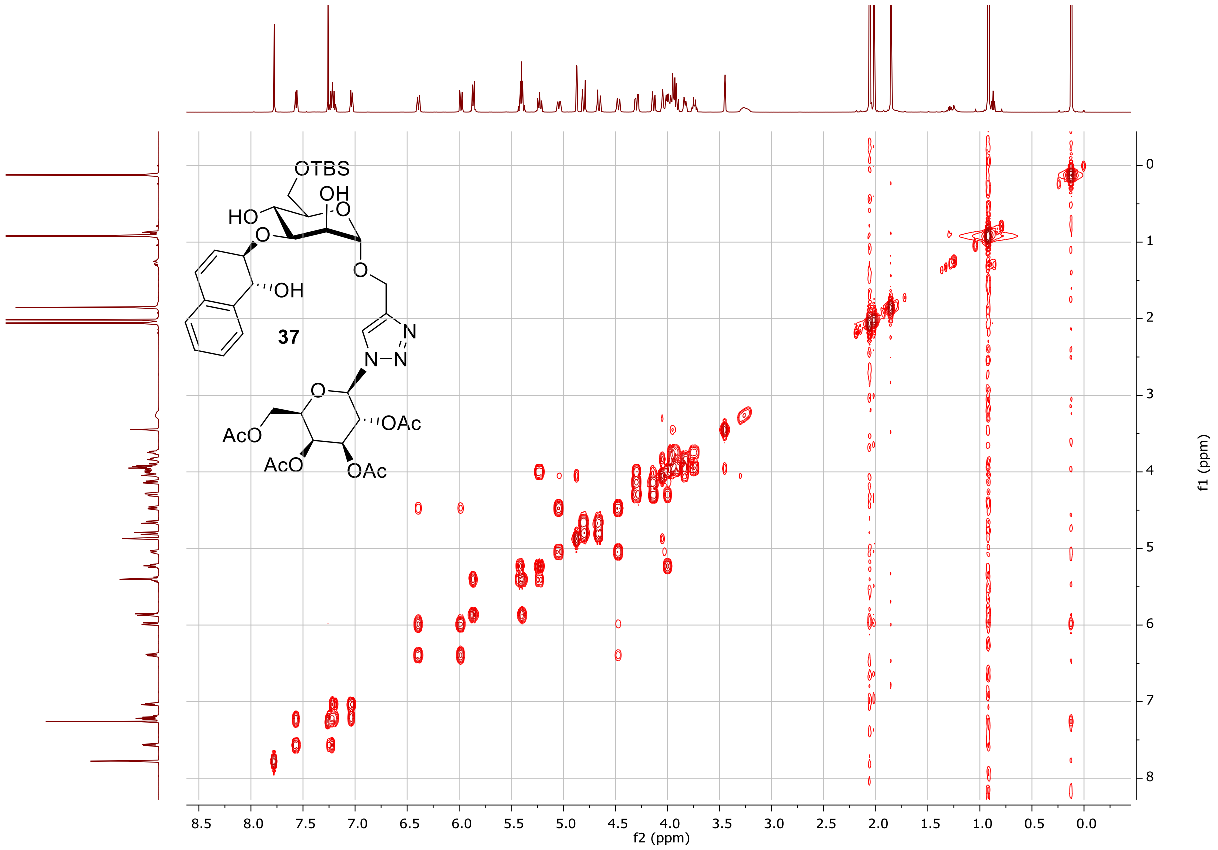

Supplementary Figure 400. COSY spectra for **37**

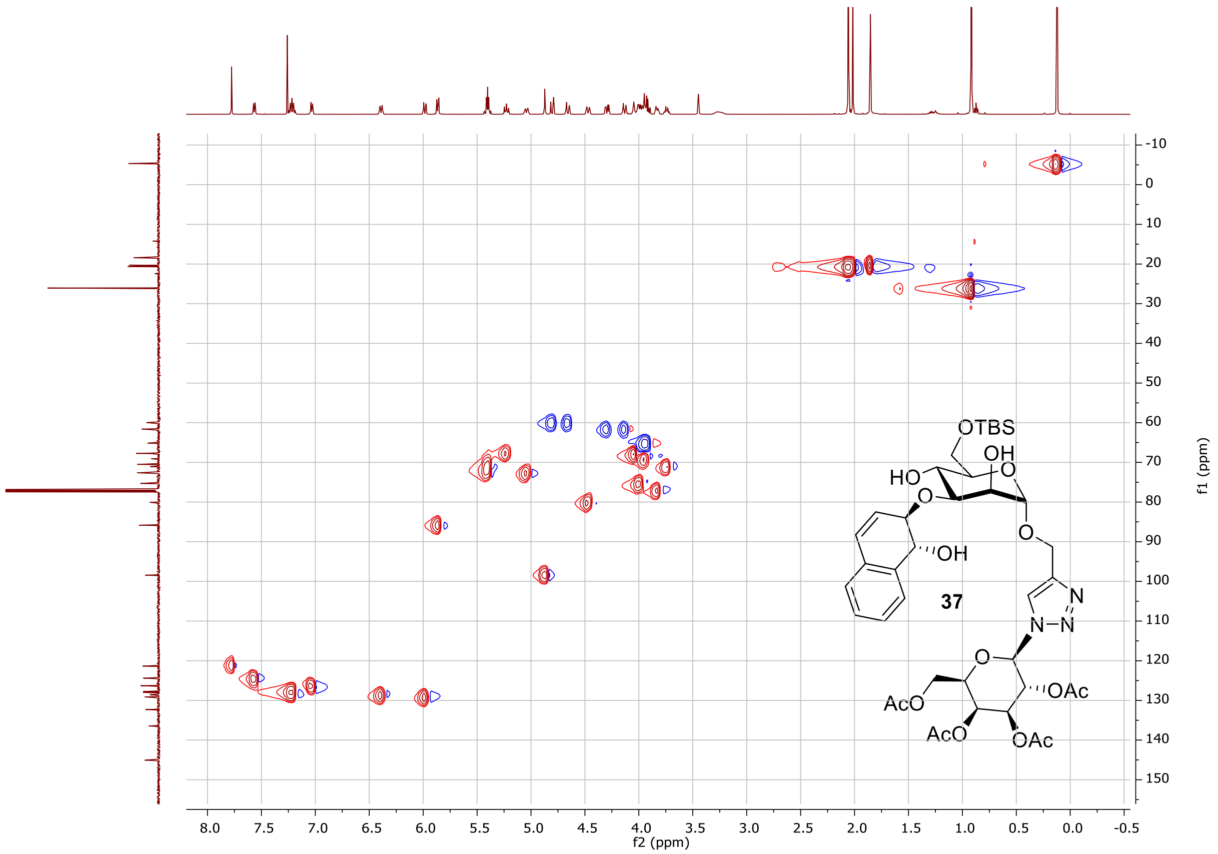

Supplementary Figure 401. HSQC spectra for **37**

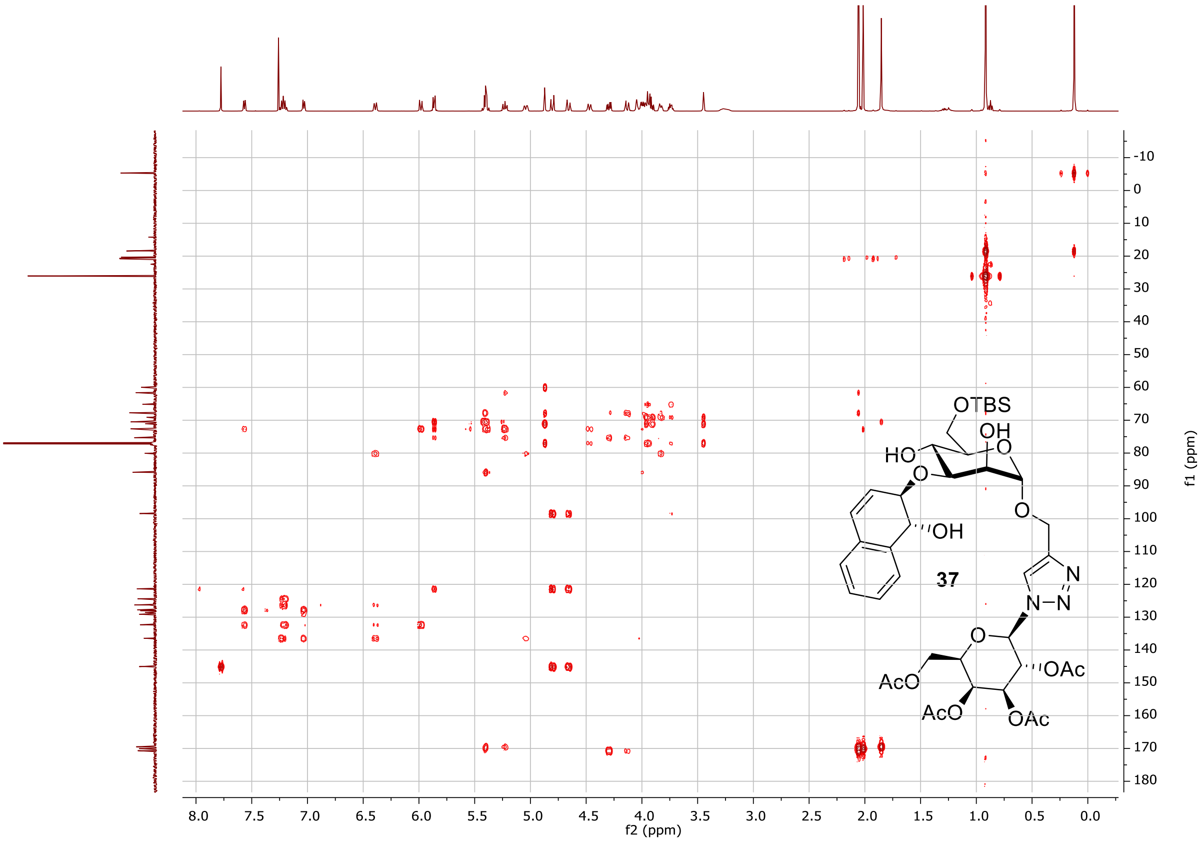

Supplementary Figure 402. HMBC spectra for **37**

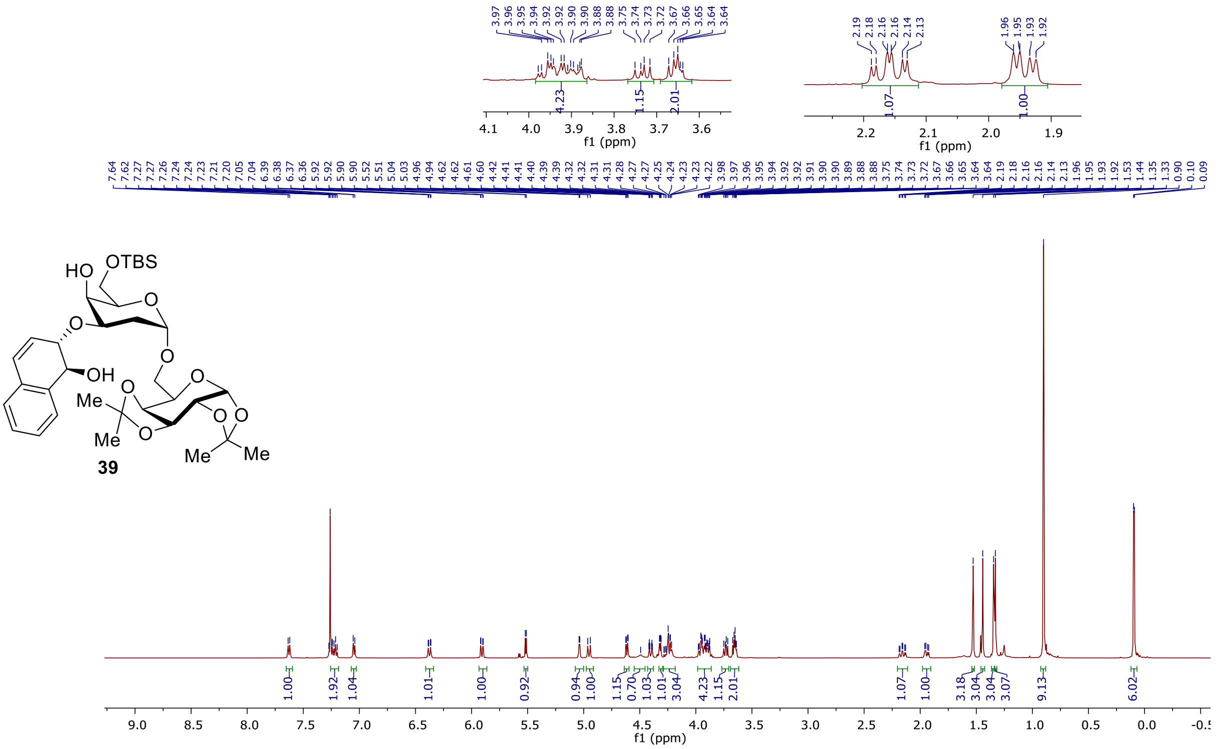

Supplementary Figure 403. ^{1}H spectra for **39**

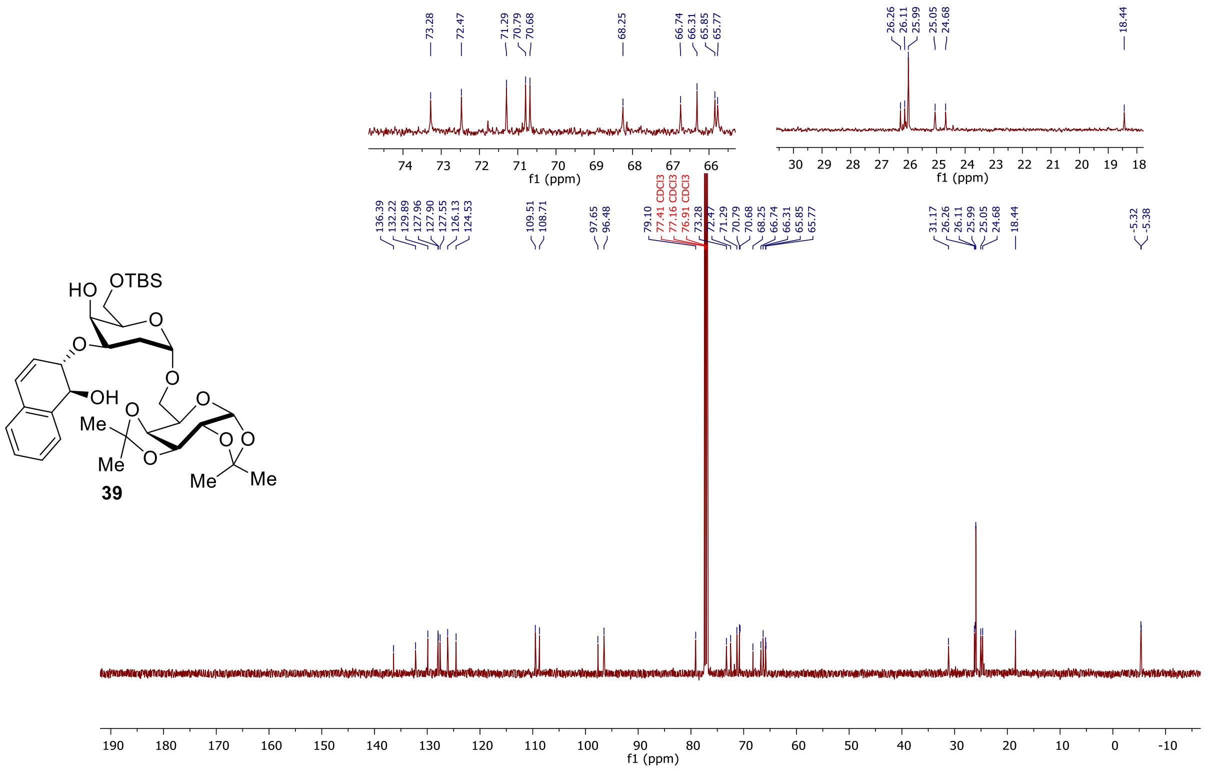

Supplementary Figure 404. ^{13}C spectra for **39**

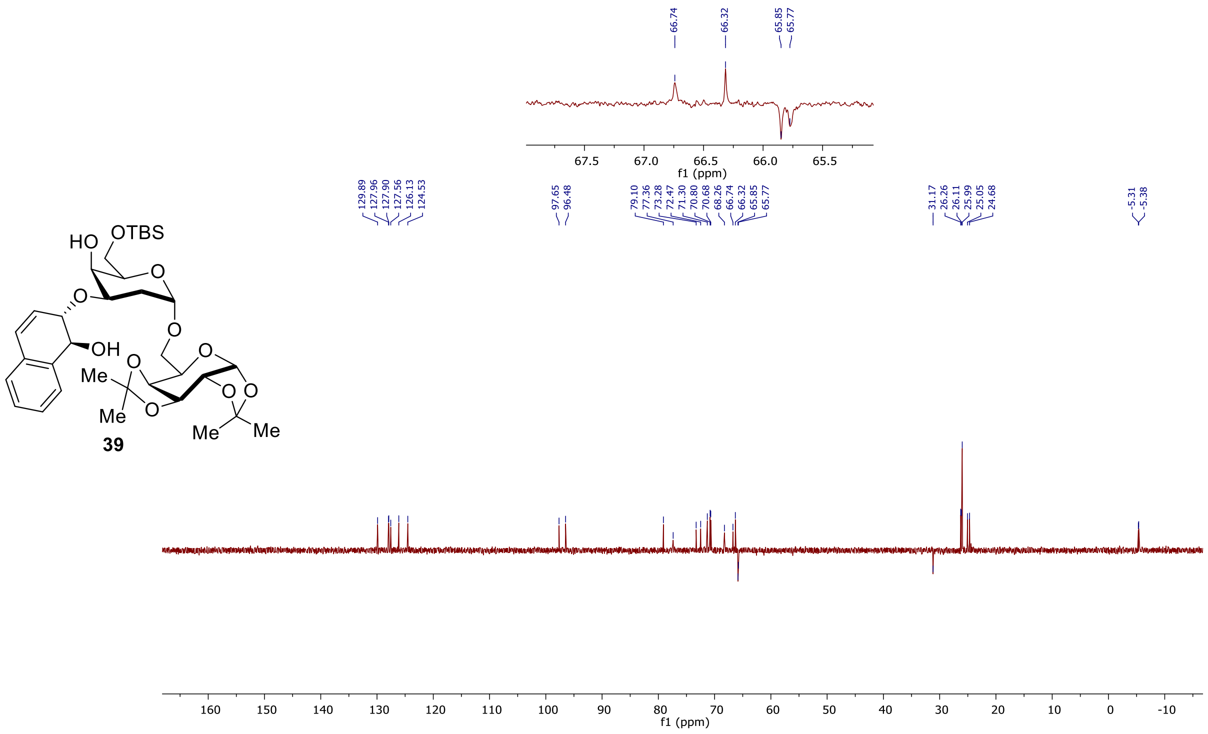

Supplementary Figure 405. DEPT spectra for **39**

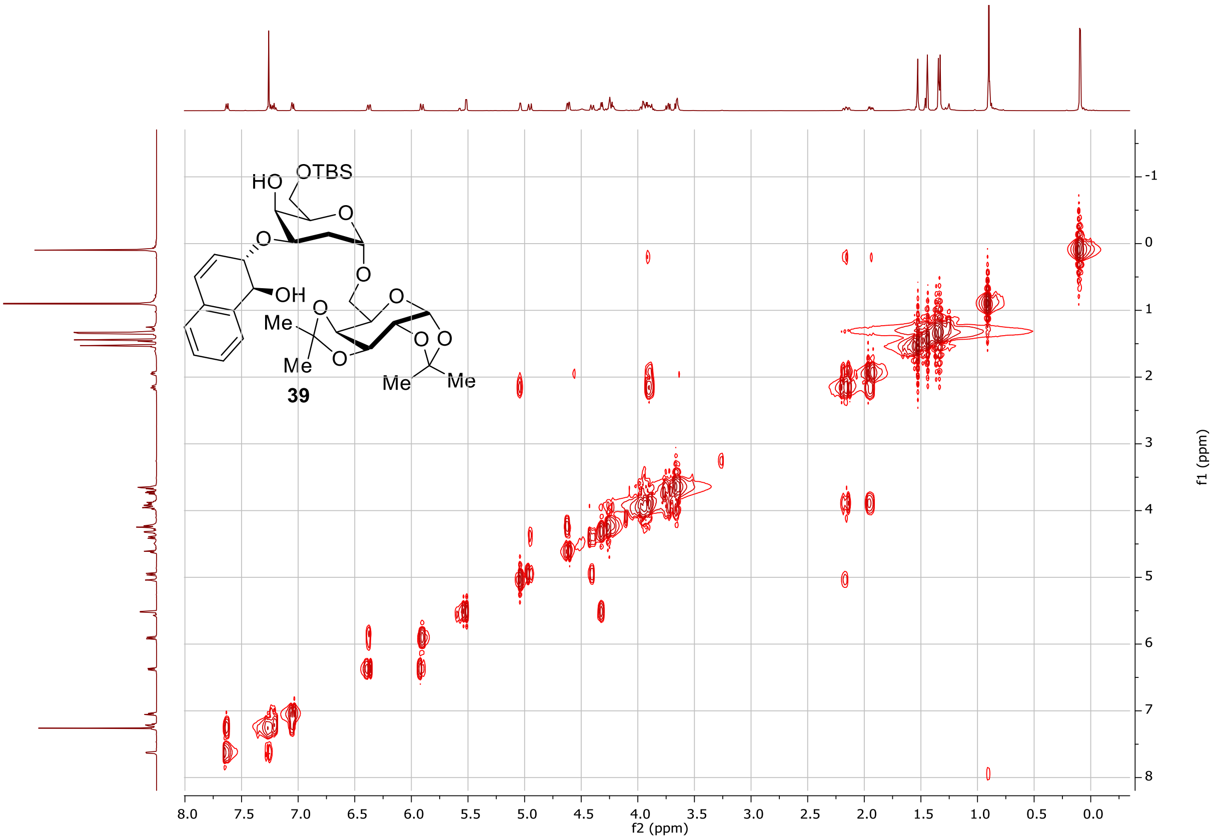

Supplementary Figure 406. COSY spectra for **39**

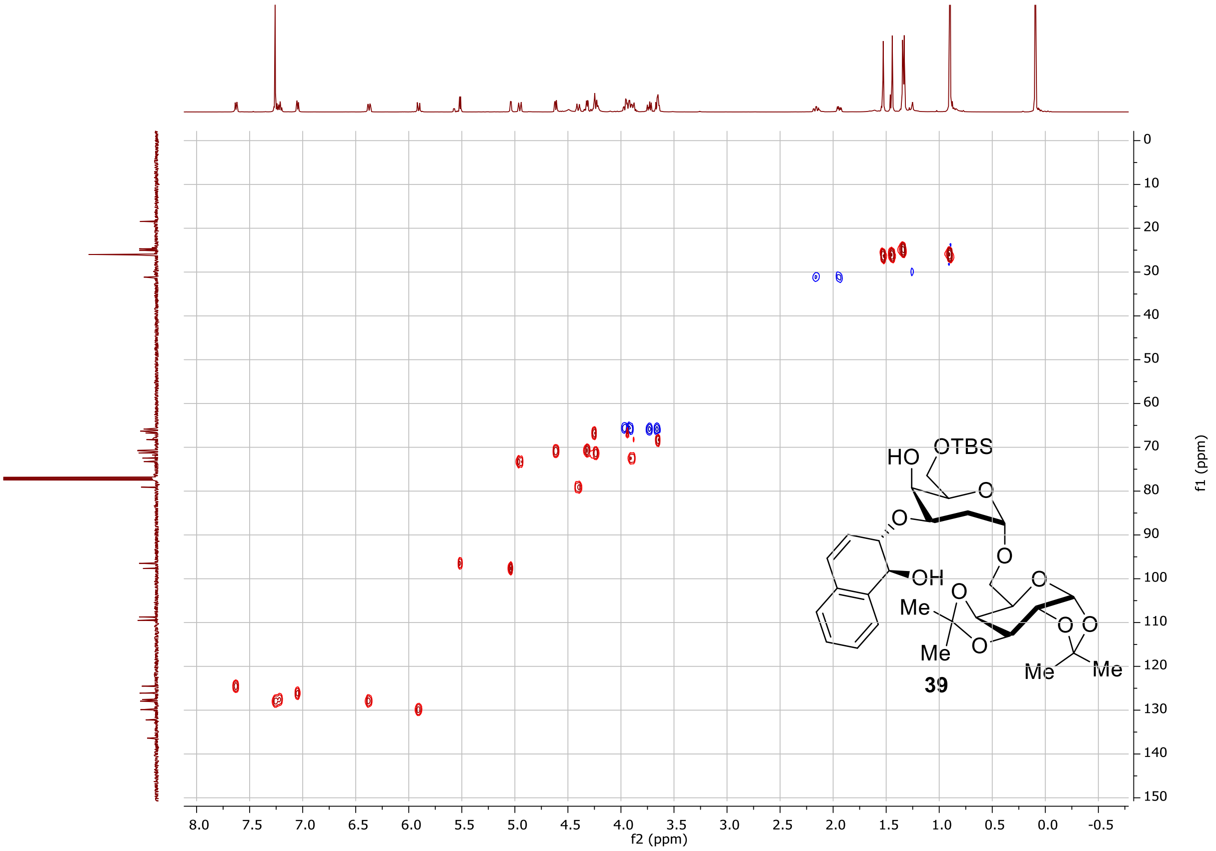

Supplementary Figure 407. HSQC spectra for **39**

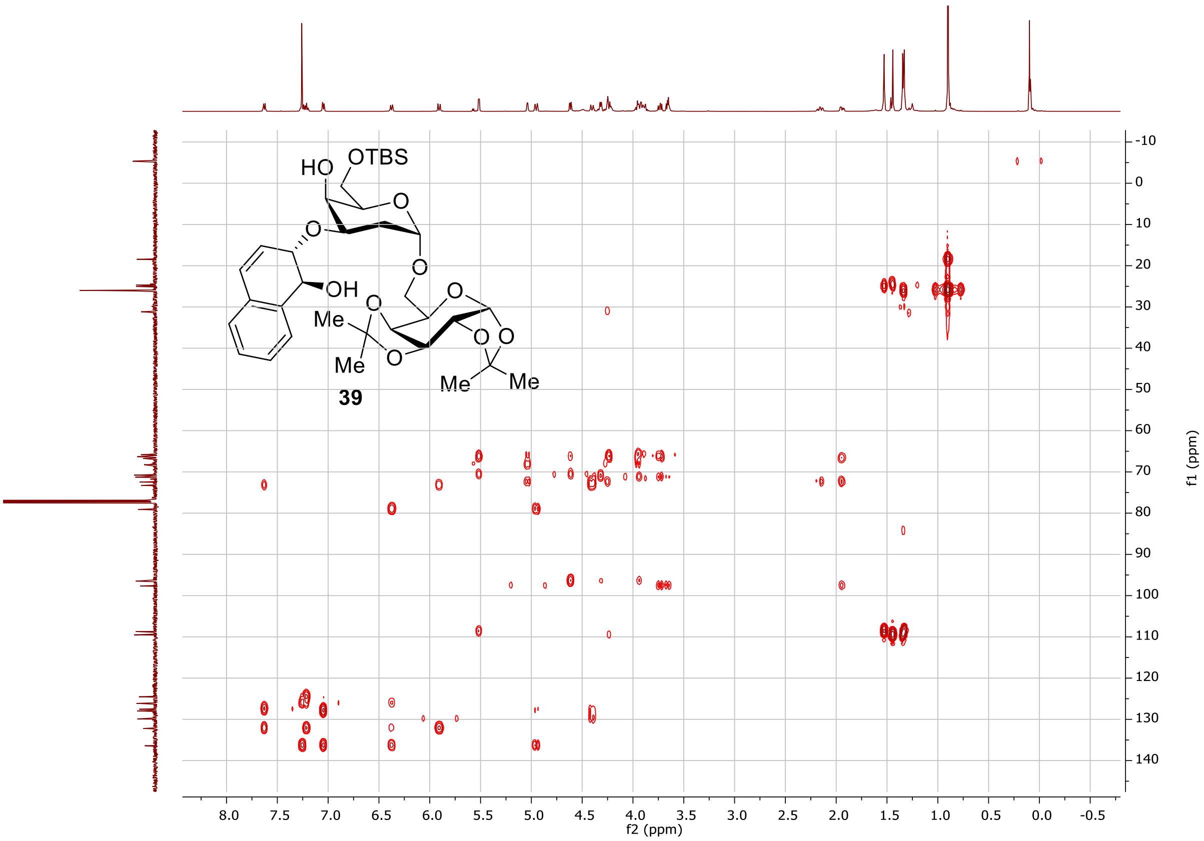

Supplementary Figure 408. HMBC spectra for **39**

Proton coupled ^{13}C spectra and NOESY for determining anomeric configuration of 17s, 17t and 17u (See Supplementary Note 15).

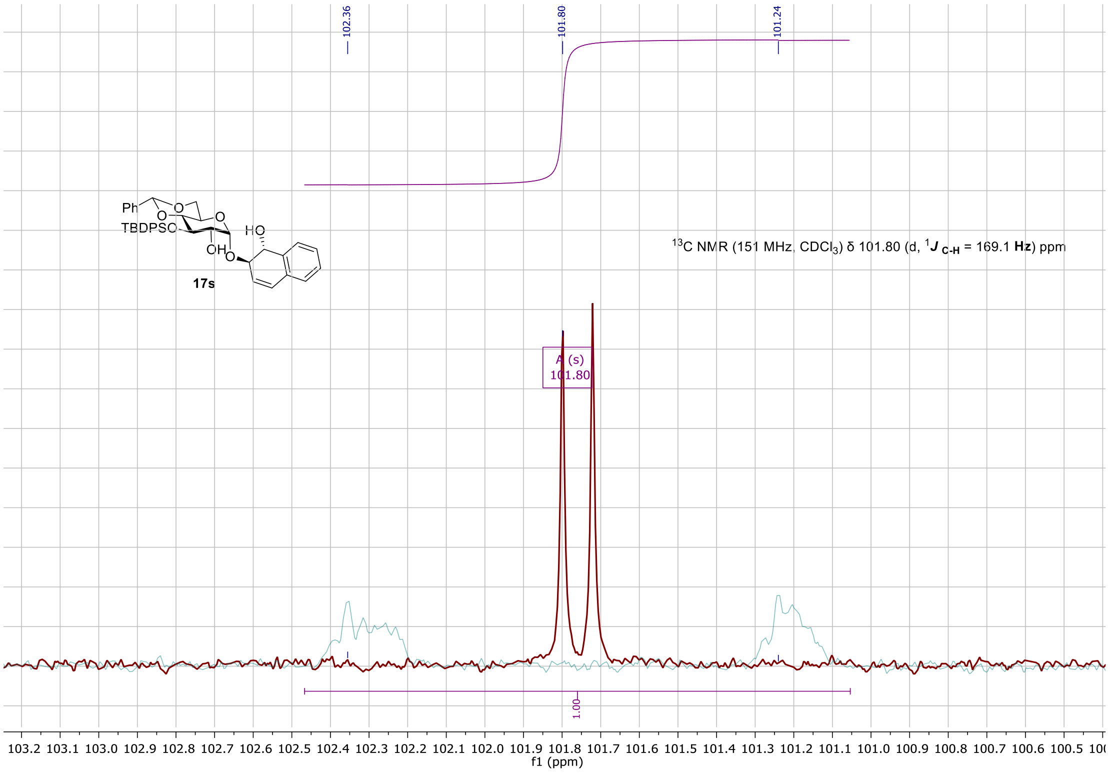

Supplementary Figure 409. Proton coupled ^{13}C NMR spectra and $^1J_{C\text{-}H}$ for **17s**

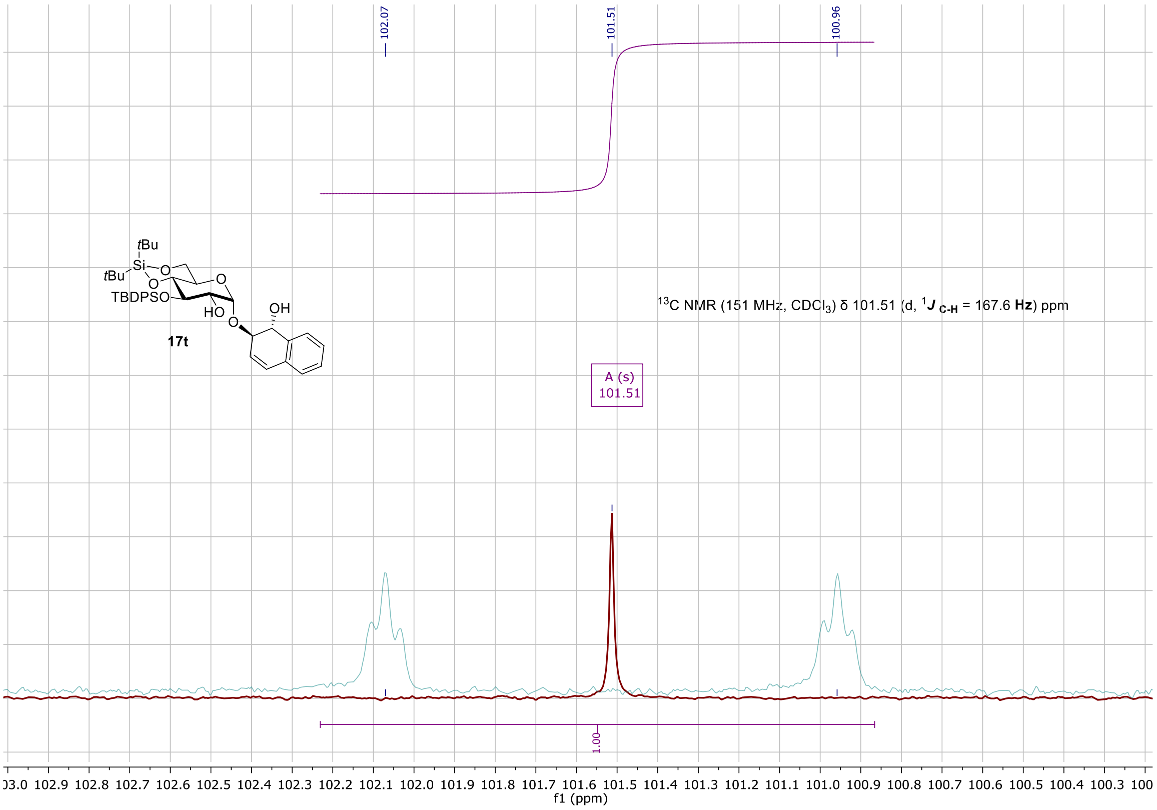

Supplementary Figure 410. Proton coupled ^{13}C NMR spectra and $^{1}J_{C\text{-}H}$ for **17t**

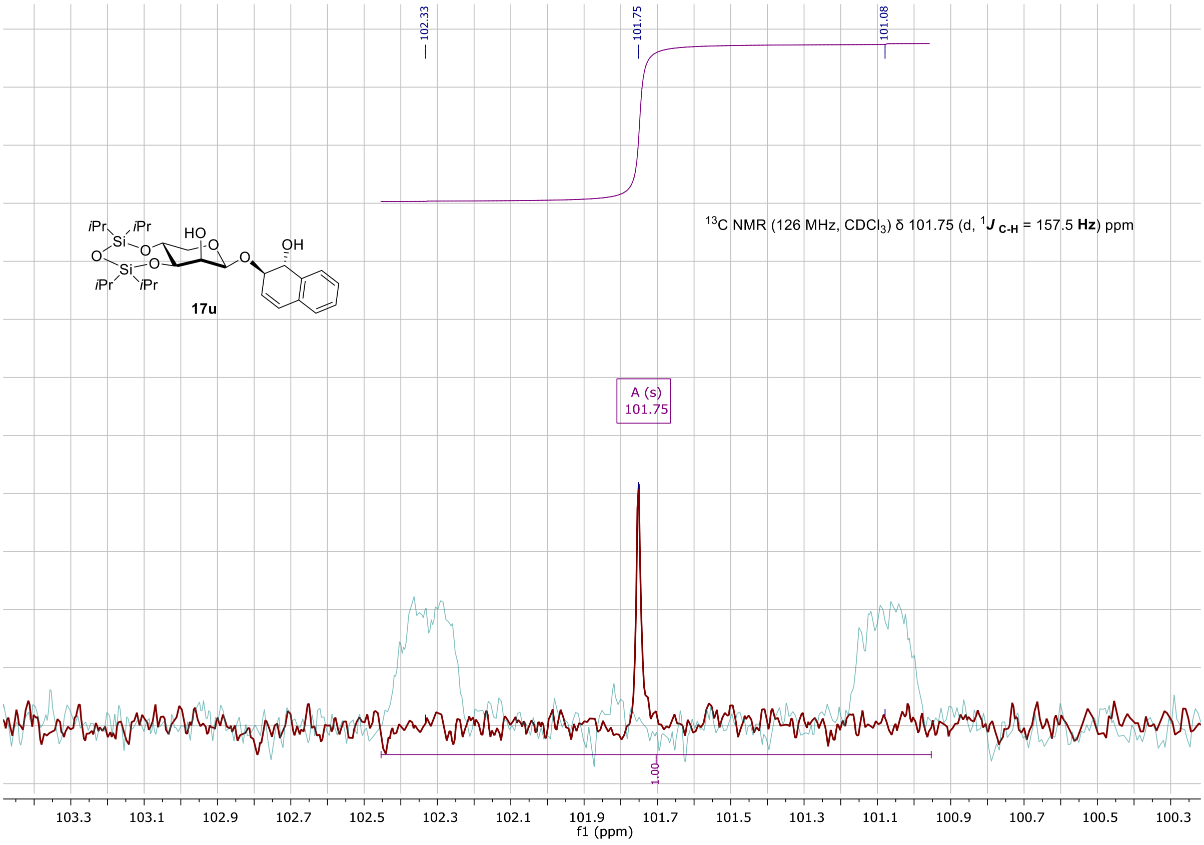

Supplementary Figure 411. Proton coupled ^{13}C NMR spectra and $^{1}J_{C\text{-}H}$ for **17u**

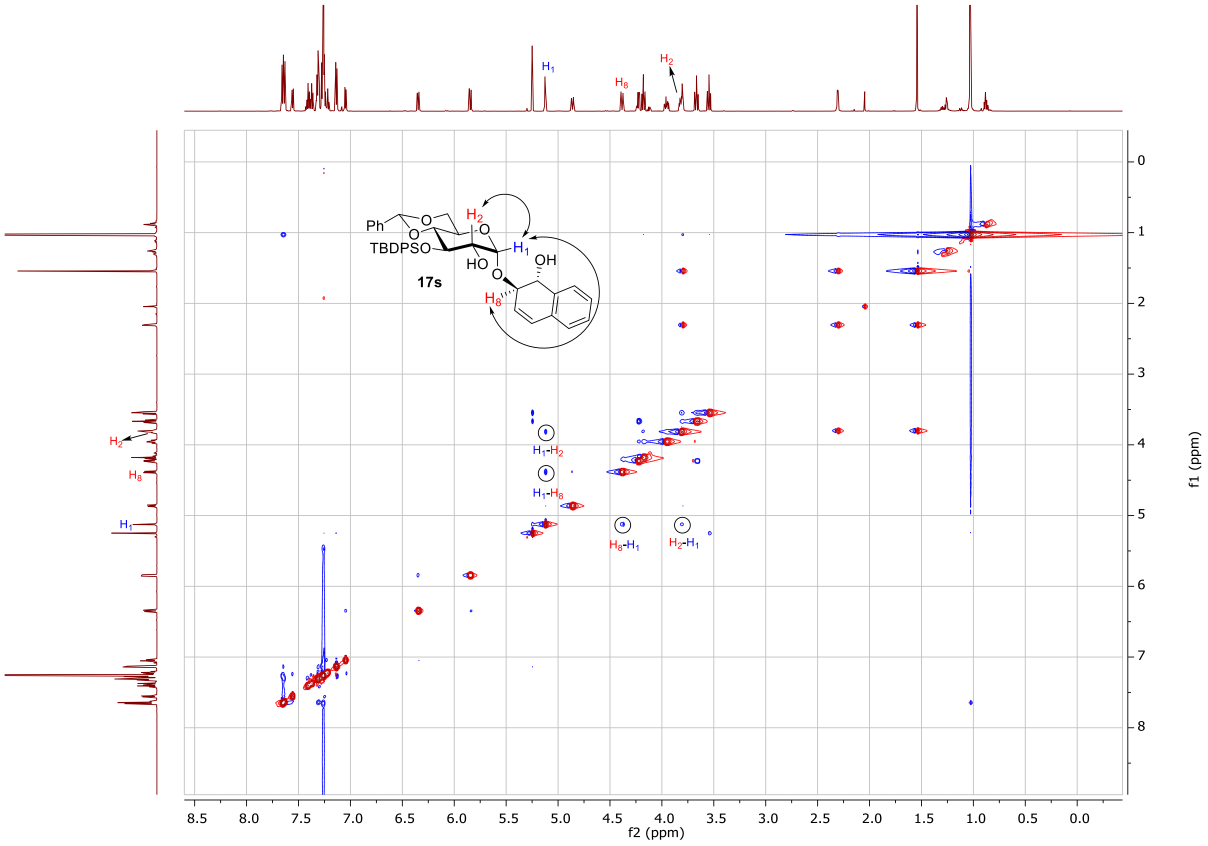

Supplementary Figure 412. NOESY spectra for **17s**

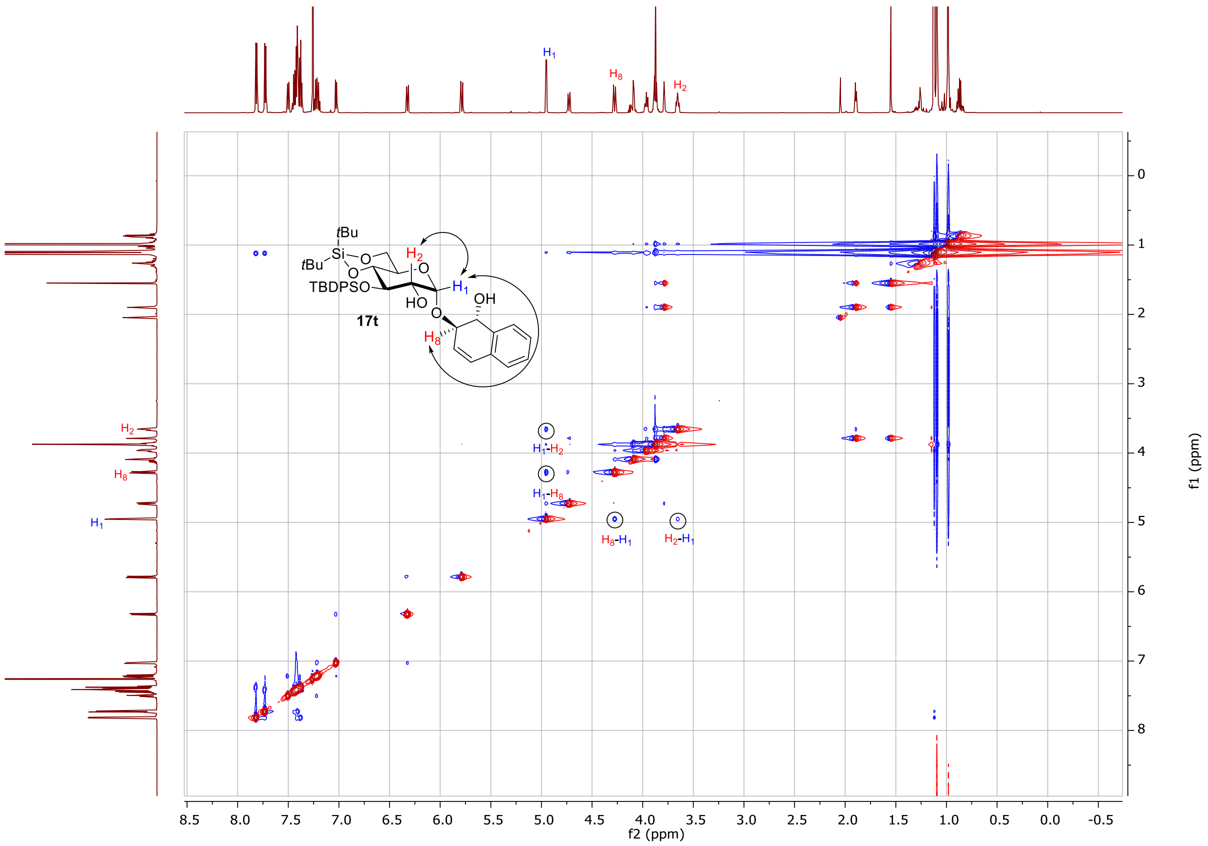

Supplementary Figure 413. NOESY spectra for **17t**

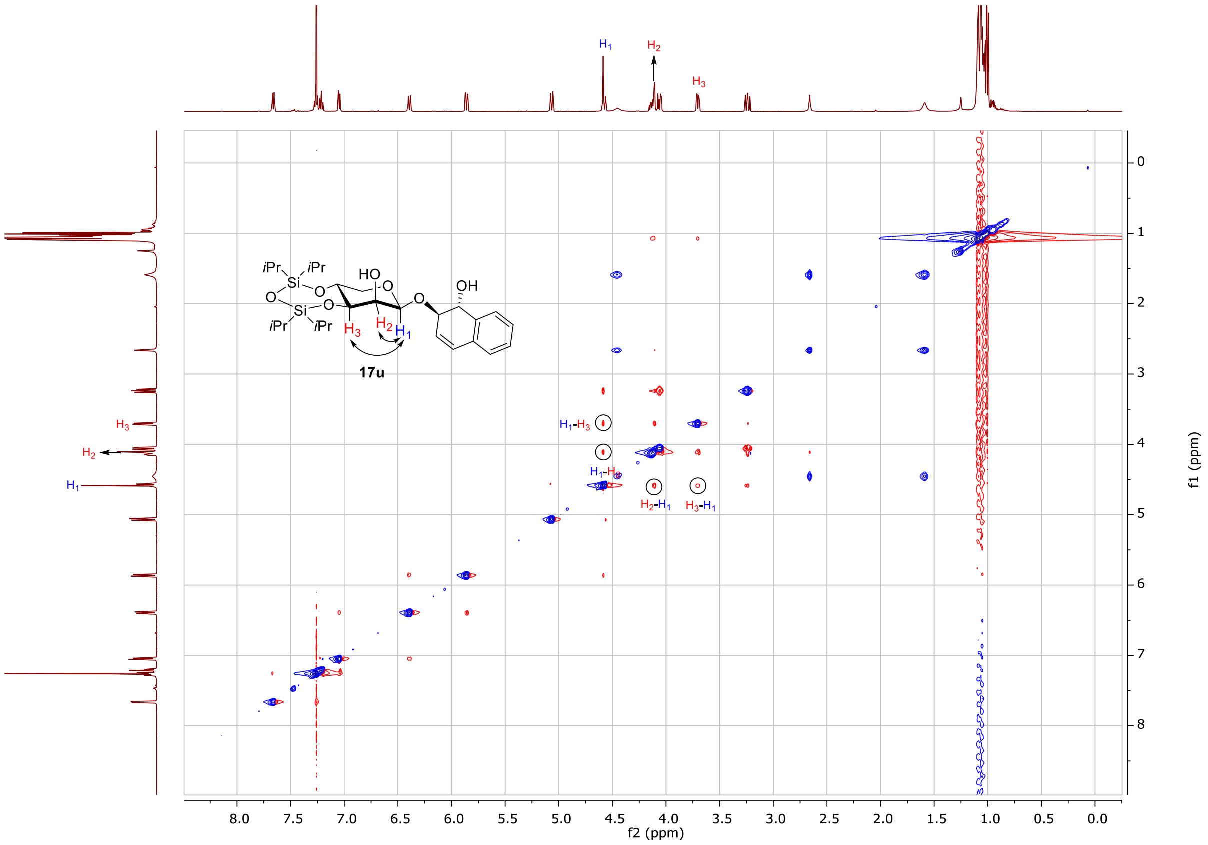

Supplementary Figure 414. NOESY spectra for **17u**
